# Contrasting Distribution of SARS-CoV-2 Lineages across Multiple Rounds of Pandemic Waves in West Bengal, the Gateway of East and North-East States of India

**DOI:** 10.1128/spectrum.00914-22

**Published:** 2022-07-19

**Authors:** Animesh K. Singh, Rezwanuzzaman Laskar, Anindita Banerjee, Rajiv Kumar Mondal, Bishal Gupta, Sonia Deb, Shreelekha Dutta, Subrata Patra, Trinath Ghosh, Sumanta Sarkar, Shekhar Ghosh, Sabyasachi Bhattacharya, Debojyoti Roy, Ankita Chakraborty, Meghna Chowdhury, Surajit Mahaptra, Antara Paul, Anup Mazumder, Aparna Chowdhury, Shiv Sekhar Chatterjee, Arunabha Sarkar, Raja Ray, Kuhu Pal, Angshuman Jana, Goutam Barik, Swagata Ganguly, Mitali Chatterjee, Dipankar Majhi, Bhaswati Bandopadhyay, Saumitra Das, Arindam Maitra, Nidhan K. Biswas

**Affiliations:** a National Institute of Biomedical Genomics, Kalyani, West Bengal, India; b School of Tropical Medicine, Kolkata, West Bengal, India; c Diamond Harbour Medical College, Diamond Harbour, West Bengal, India; d North Bengal Medical College, Siliguri, West Bengal, India; e Institute of Post-Graduate Medical Education and Research, Kolkata, West Bengal, India; f College of Medicine and JNM Hospital, Kalyani, West Bengal, India; g Bankura Sammilani Medical College, Bankura, West Bengal, India; h Medical College and Hospital, Kolkata, West Bengal, India; i Nil Ratan Sircar Medical College and Hospital, Kolkata, West Bengal, India; j R.G. Kar Medical College and Hospital, Kolkata, West Bengal, India; k Department of Health and Family Welfare, Government of West Bengal, Kolkata, West Bengal, India; Karolinska Institutet

**Keywords:** SARS-CoV-2, lineage, mutation, pandemic, West Bengal, epidemiology, coronavirus, evolution, miRNA

## Abstract

The evolution of viral variants and their impact on viral transmission have been an area of considerable importance in this pandemic of severe acute respiratory syndrome coronavirus 2 (SARS-CoV-2). We analyzed the viral variants in different phases of the pandemic in West Bengal, a state in India that is important geographically, and compared the variants with other states like Delhi, Maharashtra, and Karnataka, located in other regions of the country. We have identified 57 pango-lineages in 3,198 SARS-CoV-2 genomes, alteration in their distribution, as well as contrasting profiles of amino acid mutational dynamics across different waves in different states. The evolving characteristics of Delta (B.1.617.2) sublineages and alterations in hydrophobicity profiles of the viral proteins caused by these mutations were also studied. Additionally, implications of predictive host miRNA binding/unbinding to emerging spike or nucleocapsid mutations were highlighted. Our results throw considerable light on interesting aspects of the viral genomic variation and provide valuable information for improved understanding of wave-defining mutations in unfolding the pandemic.

**IMPORTANCE** Multiple waves of infection were observed in many states in India during the coronavirus disease 2019 (COVID19) pandemic. Fine-scale evolution of major SARS-CoV-2 lineages and sublineages during four wave-window categories: Pre-Wave 1, Wave 1, Pre-Wave 2, and Wave 2 in four major states of India: Delhi (North), Maharashtra (West), Karnataka (South), and West Bengal (East) was studied using large-scale virus genome sequencing data. Our comprehensive analysis reveals contrasting molecular profiles of the wave-defining mutations and their implications in host miRNA binding/unbinding of the lineages in the major states of India.

## INTRODUCTION

The coronavirus disease 2019 (COVID-19) pandemic has significantly impacted human health globally. Most countries have seen multiple rounds of waves. In India, most states have seen two waves: one in the summer of 2020 and the next one in the summer of 2021 (Table S1). Around 30,410,768 confirmed cases (https://www.covid19india.org; access on June 30, 2021) were reported from India in the first and second wave since the declaration of the pandemic on March 11, 2020 (1). It is of paramount importance to understand the evolution of the severe acute respiratory syndrome coronavirus 2 (SARS-CoV-2) lineages during the pandemic waves in India. The evolution of the viruses can be best studied using the tracking of new constellations of mutations that the viruses have acquired during the process of replication within the host system. The mutations provide a mechanism for viruses to adapt and/or evade host immune responses (2–6). Compared to other RNA viruses, SARS-CoV-2 has a slow evolutionary rate of ~0.8 × 10^−3^ substitutions per site per year (7). The rate of viral transmission was found to be variable during the ongoing pandemic (https://nextstrain.org/ncov/gisaid/global). With an increased transmission rate in specific periods of waves, the SARS-CoV-2 virus has infected a large number of individuals thereby increasing the possibility to acquire new mutations (8, 9). This has led to the emergence of new lineages that have impacted viral transmission, virulence, and severity of the disease (10). Sequencing of the complete SARS-CoV-2 genome was proved to be useful for high-throughput screening and detection of SARS-CoV-2 lineages to understand the virus transmission during multiple waves in a geographic region.

The complete SARS-CoV-2 RNA genome encodes 29 proteins, of which 4 are structural (spike [S], envelope [E], membrane [M], and nucleocapsid [N]), 16 are nonstructural (NSP1 to 16), and 9 are accessory proteins (open reading frame [ORF]) (ORF3a, ORF3b, ORF6, ORF7a, ORF7b, ORF8, ORF9b, ORF9c, and ORF10) (11, 12). In terms of amino acid substitution in protein-coding genes, S, ORF1a/b, and ORF8 are more prone to mutation, whereas E, M, ORF6, and ORF7a/b are comparatively more conserved genes (13). Monitoring the emerging mutations in these functional regions of SARS-CoV-2 RNA is immensely important for the management of public health.

In this study, we analyzed the SARS-CoV-2 sequences from West Bengal, a gateway state to the East and North-East regions of India. It shares international borders with neighboring countries (China, Nepal, Bhutan, Bangladesh, and Myanmar). Additionally, West Bengal is the fourth most populous (18 million) and second most densely populated (11,029/sq. km) state of India. It is well connected to the other parts of the world and has the largest air traffic hub and maritime traffic in East India as well as the busiest railway station in the world. This scenario creates a window of opportunity for the introduction and transmission of new variants in West Bengal from other countries, which can spread subsequently to other regions in India.

The regional population diversity and frequent travel histories in the urban population of India, along with the emergence of new mutations, have led us to divulge deeper into a comparative study of the viral genome sequence of the circulating virus in the first wave and second wave. Additionally, we have attempted to contrast the mutations observed in the circulating lineages in West Bengal (WB) with three other major states from the north (Delhi [DL]), west (Maharashtra [MH]), and south (Karnataka [KA]) regions of India that are also extensively connected to the other states and world through major international airports and are also densely populated.

Furthermore, to understand the functional significance of the emerging mutations in the background of various circulating sublineages during the first and second waves, we analyzed (i) prospective human-miRNA binding sites of the SARS-CoV-2 transcripts upon accumulation of specific emerging mutations, and (ii) hydrophobicity profiles of emerging mutations to understand the efficacy against the vaccine and natural infection.

Overall, the study highlights the differential distribution of SARS-CoV-2 sublineages in different gateway states of India and explored the possible contribution of host-virus interaction behind the evolution of the variants and their sustenance in the population.

## RESULTS

### Temporal spread of circulating lineages across pandemic waves in West Bengal.

**FIG 1:**
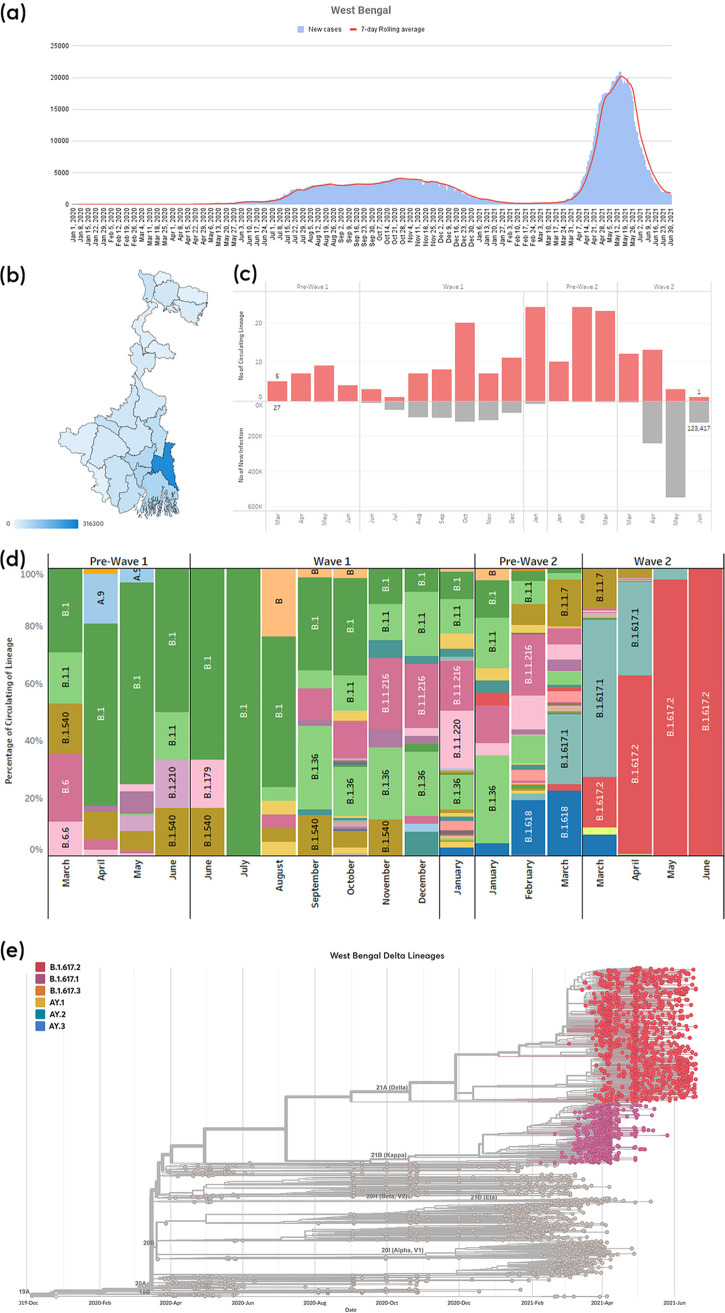
(a) Numbers of new daily SARS-CoV-2 infected cases (blue) and 7-day rolling average (red). (b) Map of the state of West Bengal with district-wise cumulative new infection distribution is highlighted. (c) Comparison between circulating lineages (top: red) and new infection cases (gray) in pandemic wave windows in the state of West Bengal. (d) Circulating lineage with pangolin nomenclature across four wave-windows. (e) Phylogenetic time tree of circulating SARS sequences sampled from West Bengal state during the study period.

We identified 57 lineages (pangolin nomenclature) in 3,198 genomes of SARS-CoV-2 sampled for the entire duration of the study period (Fig. 1d and Table S3). Out of these, the most prevalent lineages were Delta (B.1.617.2) (no of sequences: *n* = 1,301; 40.68%), Kappa (B.1.617.1) (*n* = 590; 18.45%), and B.1 (*n* = 253; 7.91%). Furthermore, it was observed that (i) Pre-Wave 1: PW1 (*n* = 192) had 12 circulating lineages spread across a 4-month period; (ii) Wave 1: W1 (*n* = 461) had 35 lineages spread across 7 months; (iii) Pre-Wave 2: PW2 (*n* = 597) had 32 lineages spread across 2 months; and (iv) Wave 2: W2 (*n* = 1,948) had 15 lineages spread across a 4-month period (Table 1). There are specific high-frequency (>5%) mutations on the spike and other genes that were observed in the background of the highly frequent lineages in each wave window time period: PW1: B.1.540 (ORF8:E110*); W1: B.1.36 (ORF3a:Q57H); B.1.1.216 (ORF3a:T223I, N:R203K, and N:G204R); PW2: B.1.618 (S:E484K); W2: B.1.617.1 (S:L452R, S:E484Q, and S:P681R); and B.1.617.2 (S:L452R, S:T478K, and S:D950N) emerged in the circulating virus in West Bengal.

**TABLE 1:** The top lineages for each state having more than or equal to 5% frequency in each wave category^*a*^

State	Pre-Wave 1	Wave 1	Pre-Wave 2	Wave 2
West Bengal	**B.1 (*n* = 123; 64.06%)**, B.1.540 (*n* = 17; 8.85%), A.9 (*n* = 15; 7.81%)	**B.1 (*n* = 112; 24.3%), B.1.36 (*n* = 81; 17.57%), B.1.1.216 (*n* = 67; 14.53%), B.1.1 (*n* = 52; 11.28%)**, B.1.1.220 (*n* = 31; 6.72%), B.1.540 (*n* = 26; 5.64%)	**B.1.618 (*n* = 121; 20.27%)**, B.1.617.1 (*n* = 80; 13.4%), B.1.1.216 (*n* = 77; 12.9%), **B.1.1.7 (*n* = 69; 11.56%)**, B.1.1.220 (*n* = 49; 8.21%), **B.1.36 (*n* = 47; 7.87%), B.1.1 (*n* = 33; 5.53%)**	**B.1.617.2 (*n* = 1294; 66.43%), B.1.617.1 (*n* = 510; 26.18%)**
Delhi	**B.6 (*n* = 149; 45.43%)**, B.1.36 (*n* = 69; 21.04%), B.6.6 (*n* = 56; 17.07%), **B.1.1 (*n* = 24; 7.32%)**	**B.1.36 (*n* = 440; 45.83%), B.1 (*n* = 172; 17.92%), B.1.1.216 (*n* = 135; 14.06%), B.1.1 (*n* = 55; 5.73%)**	**B.1.1.7 (*n* = 193; 32.38%), B.1 (*n* = 118; 19.8%), B.1.617.1 (*n* = 59; 9.9%), B.1.36 (*n* = 55; 9.23%)**, B.1.525 (*n* = 33; 5.54%)	**B.1.617.2 (*n* = 604; 48.67%), B.1.1.7 (*n* = 311; 25.06%), B.1.617.1 (*n* = 172; 13.86%)**
Karnataka	B.1.1.46 (*n* = 51; 23.61%), **B.6 (*n* = 50; 23.15%), B.1.210 (*n* = 27; 12.5%), B.1.1 (*n* = 23; 10.65%), B.1 (*n* = 21; 9.72%)**, B.1.36.8 (*n* = 11; 5.09%)	B.1.36.29 (*n* = 80; 29.74%), **B.1.1 (*n* = 44; 16.36%),** B.1.1.46 (*n* = 22; 8.18%), B.1.560 (*n* = 22; 8.18%), **B.1.36 (*n* = 18; 6.69%), B.1 (*n* = 18; 6.69%)**, B.1.1.7 (*n* = 15; 5.58%)	**B.1.36.29 (*n* = 50; 23.81%), B.1.1.7 (*n* = 31; 14.76%), B.1.617.1 (*n* = 23; 10.95%)**, B.1.560 (*n* = 17; 8.1%), B.1.617.2 (*n* = 17; 8.1%), **B.1 (*n* = 13; 6.19%), B.1.1 (*n* = 12; 5.71%)**	**B.1.617.2 (*n* = 733; 72.15%), B.1.617.1 (*n* = 145; 14.27%), B.1.1.7 (*n* = 75; 7.38%)**
Maharashtra	B.1.1.306 (*n* = 403; 58.75%), **B.1.210 (*n* = 117; 17.06%), B.1 (*n* = 54; 7.87%), B.6 (*n* = 38; 5.54%)**	B.1.1.306 (*n* = 484; 61.81%), B.1.210 (*n* = 103; 13.15%), **B.1 (*n* = 40; 5.11%)**	**B.1.617.1 (*n* = 193; 29.42%), B.1.36.29 (*n* = 109; 16.62%)**, B.1.1.306 (*n* = 71; 10.82%), **B.1.36 (*n* = 66; 10.06%), B.1.1 (*n* = 34; 5.18%), B.1.1.7 (*n* = 33; 5.03%)**	**B.1.617.1 (*n* = 1563; 44.18%), B.1.617.2 (*n* = 1206; 34.09%)**

a Lineages that are common across two states are highlighted in bold.

### Contrasting features of lineage distribution of West Bengal with other gateway states of India.

For genomes analyzed from the state of Maharashtra, Karnataka, and Delhi, Pangolin lineage classification identified 79, 55, and 77 lineages, respectively (Fig. S1d, Fig. S2d, and Fig. S3d). During the PW1, West Bengal had two unique lineages with high-frequency B.1.540 and A.9. In contrast, unique and most frequent lineages found in other states were as follows: (i) Maharashtra: B.1.1.306, (ii) Karnataka: B.1.1.46 and B.1.36.8, and (iii) Delhi: B.1.36 and B.6.6 (Table 1). With progress toward W1, an increase (2-fold) in the number of circulating lineages in all four states, West Bengal (12 to 35), Maharashtra (19 to 28), Karnataka (16 to 28), and Delhi (13 to 44), was observed. No new unique lineage emerged in Delhi; B.1.36 was the most prevalent lineage. Throughout this period, the lineage B.1.1.220 replaced B.1.540 as the most dominant lineage in West Bengal. A similar scenario was observed in Karnataka where B.1.36.29 replaced B.1.1.46. While in Maharashtra, B.1.1.306 continued to be the most frequent lineage in both PW1 and W1.

In PW2, a sudden emergence of B.1.618 was observed in West Bengal, which was unique to the state and had acquired an immune evasion mutation (E484K) in the spike protein. In West Bengal, B.1.618 was followed by B.1.617.1, which was the most prevalent lineage in Maharashtra during the same time period. In Delhi, B.1.1.7 (alpha) was the most frequent lineage, whereas in Karnataka B.1.36.29 continued to be the most frequent lineage. In W2, B.1.617.2, B.1.617.1, and B.1.1.7 were the only prominent lineages circulating in all the states. Of these, B.1.617.2 emerged to be the dominant lineage in all states except Maharashtra where B.1.617.1 was the most dominant (Fig. 1e, Fig. S1e, Fig. S2e, and Fig. S3e). The W2 time period of the pandemic witnessed a reduction in the number of circulating lineages in all the states i.e., West Bengal (32 to 15), Maharashtra (42 to 39), Karnataka (28 to 15), and Delhi (37 to 25). The complete list of lineages with high frequency (≥5%) observed for each wave category is provided in Table S3.

### Contrasting profiles of mutational dynamics in SARS-CoV-2 genes across wave windows.

**FIG 2:**
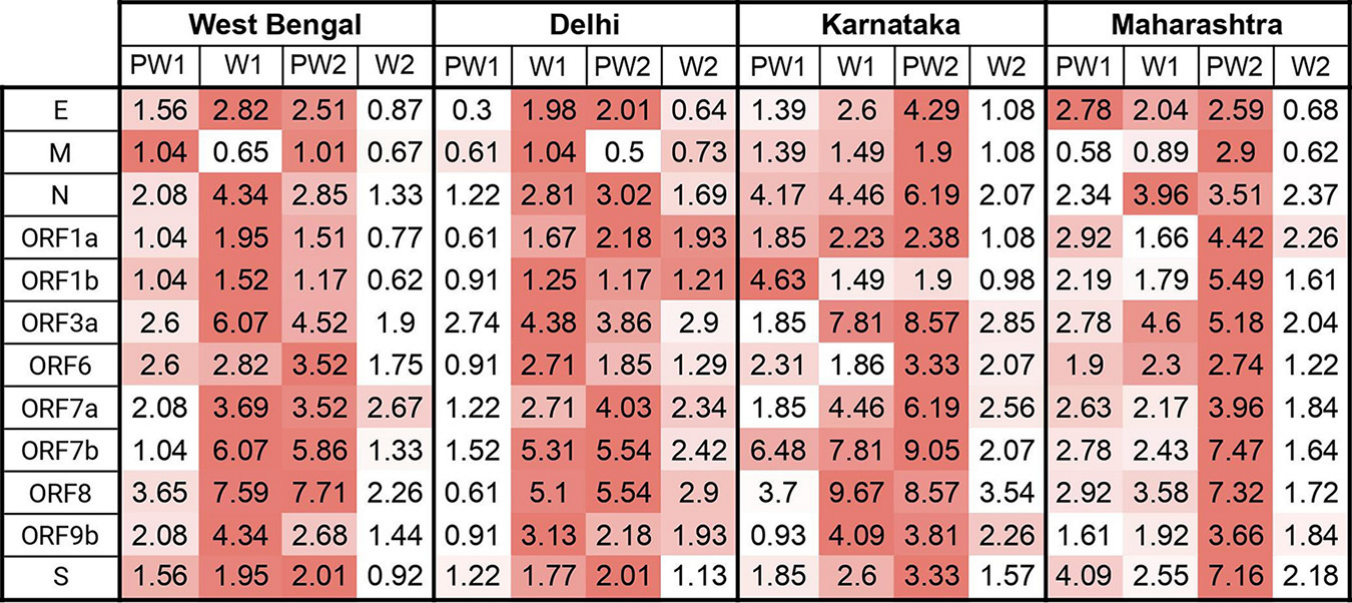
The normalized mutation rate in each of the SARS-CoV-2 proteins per wave window for each of the four states.

In order to understand the underlying dynamics of base substitution in the sequences, we calculated the number of sites (θ) mutated per gene per sampled sequence of SARS-CoV-2. For the SARS-CoV-2 sequences sampled from West Bengal, PW1, the most mutated gene was ORF8 followed by ORF3a and ORF6 in PW1. For W1, the most mutated gene was ORF8 followed by ORF7b and ORF3a. During PW2, most mutations were acquired in the ORF8 gene followed by ORF7b and ORF3a. For W2, the most mutated gene was ORF7a followed by ORF8 and ORF3a. While comparing W1 with W2, a decreasing trend in the value of θ was observed in most of the genes across all four states. The major exceptions for the M gene in West Bengal, the ORF1a gene in Delhi and Maharashtra, and the ORF6 gene in Karnataka where the decrease in θ was not detected. For the states of West Bengal, Karnataka, and Maharashtra the lowest value of θ for most of the genes was observed in W2 while in Delhi a similar trend was observed in PW1 (Fig. 2).

### Comparative features of mutational profile in different states across the waves.

In our data set, we found contrasting mutational profiles per wave in three major states of India compared to West Bengal (Table S4). In the spike protein, D614G was the most prevalent mutation across all waves in all the four states with frequencies greater than 31% for PW1 while the rest of the waves had frequencies greater than 88%. Apart from D614G, the following mutations on the spike protein S:G261S (WB-PW1: 8.13%), S:Q677H (DL-PW1: 2.44% and DL-W1: 14.58%), S:K77M (KA-PW1: 3.24%), S:T29I (MH-PW1:2.48%), S:P681H (WB-W1: 10.85%, MH-W1: 8.3%, DL-PW2: 31.71%), S:N440K (KA-W1: 27.14%), S:E484K (WB-PW2: 29.31%), S:L452R (KA-PW2: 18.57%, MH-PW2: 27.44%), and S:P681R (in W2, WB: 92.76%; DL: 65.83%; KA: 85.53%; and MH: 74.65%) were found to be dominant in a specific wave window in any one of the four states. The frequencies of S:E484K and S:V1230L mutation in the spike gene from the PW2 time window of sequences sampled from West Bengal were 29.31% and 10.89%, respectively, while it was lower (>6%) in other waves across all the states. In the envelop gene, E:S68F, E:P71L, E:L73F, E:V75F, E:L21F, and E:V62F were the most frequent mutations. Some unique prevalent substitutions of the envelope gene were identified from Delhi; E:I46M (W1: 2.6%) and E:V47F (W1: 2.6%). Furthermore, mutations E:A41V (PW2: 9.05%) and E:V58F (W2: 0.49%) found in KA was dominant. In the sequences analyzed, two sets of mutations in the nucleocapsid gene, N203K/N204R and R203M/D377Y, were dominant during multiple waves separately in all four states, except for N:P13L (50.61% in PW1 in Delhi), N:S194L (53.02% in W1 in Delhi), and N:S2P (28.1% in PW2 in Karnataka). A 50-times increase in the frequency of N:D63G was observed during PW2 to W2 in West Bengal, Maharashtra, and Delhi while the increase in Karnataka was 8 times. The membrane protein of SARS-CoV-2 is associated with immune response, and the amino acid position 82 across the transmembrane domain was found to have immune evasion potential (14). At this position, two dominant substitutions, M:I82S and M:I82T, were found in high frequencies in PW2 and W2, respectively in all states. Similar to N:D63G, we found wave wise increase in the frequency of M:I82T substitutions from PW2 to W2 in West Bengal (33-fold), Maharashtra (74-fold), Karnataka (8-fold), and Delhi (9-fold). In the ORF1a gene, unique mutations with low frequency were observed in each wave from sequences collected in West Bengal and Delhi, whereas in Maharashtra and Karnataka, some mutations dominated in more than one wave, such as ORF1a:T3646A was observed in both PW2 (27.74%) and W2 (52.77%) in Maharashtra (Table S4). During PW1, A88V was the most dominant mutation in ORF1b in sequences from Delhi (60.37%), whereas in other states it was ORF1b:P314L. It was observed that the most common amino acid deletions in W1 in Karnataka and PW2 in Karnataka and Delhi were S:H69-, S:V70-, and S:Y144-. Along with these deletions, ORF1a:S3675-, ORF1a:G3676-, and ORF1a:F3677- were also detected in significant frequency in Karnataka. The deletion at ORF8:D119-, ORF8:F120-, was observed frequently in W2 in Delhi, whereas in the sequences collected from Karnataka, an additional deletion at S:E156-, S:F157-, was observed.

### Evolving characteristics of Delta (B.1.617.2) sublineages in West Bengal and other states.

SARS-CoV-2 genomes showed closely related clusters within the B.1.617.2 lineage, which became noticeably more pronounced as the pandemic progressed. We identified characteristic mutations, synonymous substitutions or nonsynonymous or deletions, occurring in more than 75% of the samples labeled with the lineage. The characteristic substitutions of B.1.617.2 lineage observed in all the states had the following mutation constellations common to them: M:I82T, N:D63G, N:R203M, N:D377Y, ORF1b:G662S, ORF1b:P1000L, ORF1b:P314L, ORF3a:S26L, ORF7a:V82A, ORF7a:T120I, ORF8:D119-, ORF8:F120-, ORF9b:T60A, S:T19R, S:L452R, S:T478K, S:D614G, S:P681R, and S:D950N (28248 to 28253). Deletion at S:E156-, S:F157- (22029 to 22034), and substitution at S:R158G were observed in sequences from West Bengal, whereas sequences from Delhi presented a nucleotide deletion at 28271.

**FIG 3:**
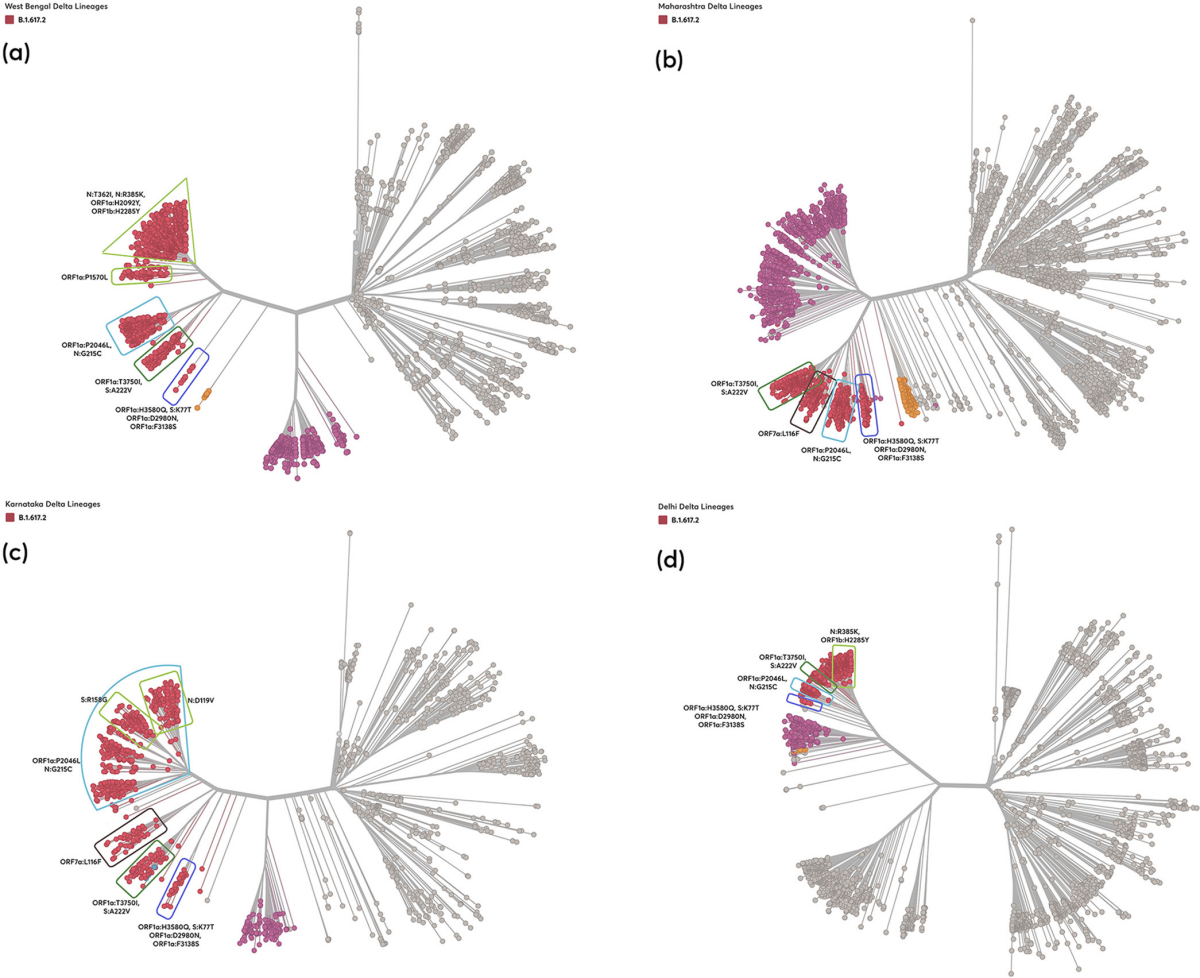
Unrooted phylogenetic tree of the Delta sublineages for sequences sampled from the state of West Bengal (a), Maharashtra (b), Karnataka (c), and (d) Delhi (d).

With the accumulation of new sets of substitutions, further branching of Delta lineage was identified through phylodynamic analysis. For West Bengal, 5 subclusters (C1 to C5) were observed in the background of Delta lineage (Table 2 and Fig. 3). Three of these clusters were common among the other states under study. The C1 subcluster with defining mutation (ORF1a:T3750I, S:A222V) has wide representation across all states: West Bengal (*n* = 123 sequences; earliest sequence collected on March 26, 2021), Maharashtra (*n* = 357; March 1, 2021), Karnataka (*n* = 89, March 4, 2021), and Delhi (*n* = 46, March 23, 2021). C2 subcluster with defining mutation (ORF1a:P2046L, N:G215C) was identified from West Bengal (*n* = 293, March 27, 2021), Maharashtra (*n* = 353, February 12, 2021), in Karnataka(*n* = 518, February 4, 2021), and Delhi (*n* = 94, March 4, 2021). C3 subcluster with defining mutation (ORF1a:D2980N, ORF1a:F3138S, ORF1a:H3580Q, S:K77T) was observed from West Bengal (*n* = 9, April 2, 2021), Maharashtra (*n* = 90, March 6, 2021), Karnataka (*n* = 41, March 30, 2021), and Delhi (*n* = 6, April 1, 2021). Two unique subclusters (C4 and C5) specific to West Bengal were detected. The defining mutation in these subclusters were (i) (ORF1b:P1570L) (*n* = 89, March 27, 2021) and (ii) (N:T362I, N:R385K, ORF1a:H2092Y, ORF1b:H2285Y) (*n* = 615, March 15, 2021). In contrast, Delhi had one unique subcluster (C6) with (N:R385K, ORF1b:H2285Y) mutation (*n* = 329), with the earliest sequence dating back to March 4, 2021. The other two states, Maharashtra (*n* = 257) and Karnataka (*n* = 57), had a Delta subcluster (C7) common to both states with defining mutation (ORF7a:L116F) with the earliest sequence dating back to February 18, 2021, and March 4, 2021. Further pathogenicity profiles of cluster contributing ns-substitutions were assessed as deleterious or neutral, which has been summarized in Table S5.

**TABLE 2:** The sequence cluster along with the respective states, defining mutation, number of sequences in each cluster, and earliest date sampled from each state is reported

Cluster	States	Defining mutation	No. of sequence	Earliest date
C1	West Bengal, Maharashtra, Karnataka, Delhi	ORF1a:T3750I (nsp6), S:A222V (NTD domain)^*a*^	West Bengal = 123, Maharashtra = 357, Karnataka = 89, Delhi = 46	West Bengal (March 26, 2021), Maharashtra (March 1, 2021), Karnataka (March 4, 2021), Delhi (March 23, 2021)
C2	West Bengal, Maharashtra, Karnataka, Delhi	ORF1a:P2046L (nsp3), N:G215C	West Bengal = 293, Maharashtra = 353, Karnataka = 518, Delhi = 94	West Bengal (March 27, 2021), Maharashtra (February 12, 2021), Karnataka (February 4, 2021), Delhi (March 4, 2021)
C3	West Bengal, Maharashtra, Karnataka, Delhi	ORF1a:D2980N (nsp4), ORF1a:F3138S (nsp4), ORF1a:H3580Q (nsp6), S:K77T (NTD domain)	West Bengal = 9, Maharashtra = 90, Karnataka = 41, Delhi = 6	West Bengal (April 2, 2021), Maharashtra (March 6, 2021), Karnataka (March 30, 2021), Delhi (April 1, 2021)
C4	West Bengal	ORF1b:P1570L	West Bengal = 89	West Bengal (March 27, 2021)
C5	West Bengal	N:T362I (dimerization domain), N:R385K (CTD domain), ORF1a:H2092Y (nsp3), ORF1b:H2285Y (nsp15)	West Bengal = 615	West Bengal (March 15, 2021)
C6	Delhi	N:R385K, ORF1b:H2285Y (nsp15)	Delhi = 329	Delhi (March 04, 2021)
C7	Maharashtra, Karnataka	ORF7a:L116F	Maharashtra = 257, Karnataka = 57	Maharashtra (February 18, 2021), Karnataka (March 4, 2021)

a NTD, N-terminal domain.

### Differential hydrophobicity profiles of epitope regions of emerging mutations.

**FIG 4:**
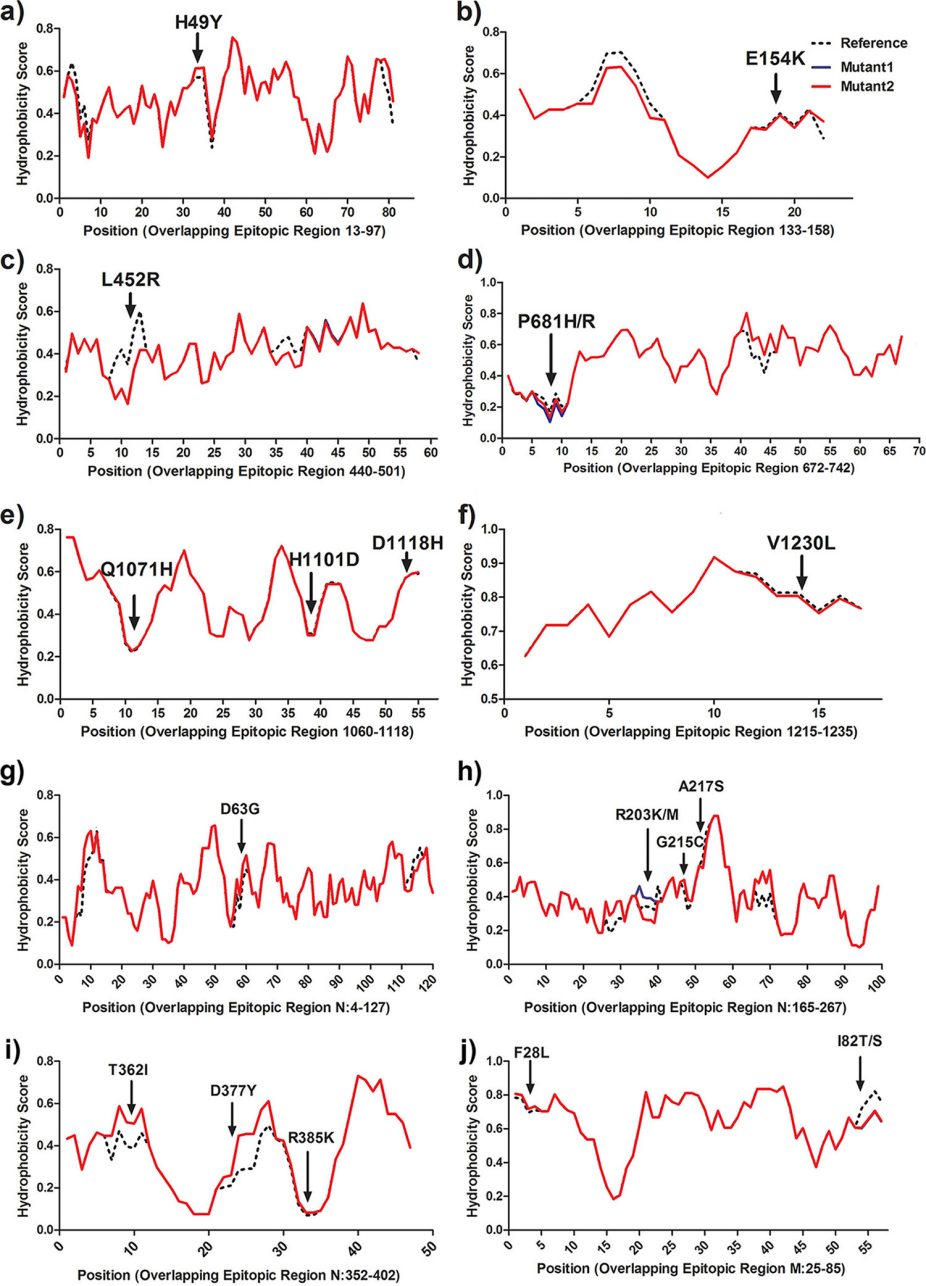
Hydrophobicity plots of the epitope regions show altered hydrophobicity due to the mentioned mutations: spike protein (a to f); nucleocapsid protein (g to i); and membrane protein (j).

To understand the potential implications of the dominant mutations of each wave, the hydrophobicity profiles of each mutation inside the overlapping epitopic regions of B cell, major histocompatibility complex (MHC) class I and class II were analyzed (Fig. 4). This analysis revealed that most of the mutations were found in either the B-cell epitope or MHC class I epitope regions (cytotoxic T-cell epitope), particularly in the membrane, nucleocapsid, and spike proteins. In the nucleocapsid protein, out of the 19 amino acid mutations, 16 mutations overlap with the epitopic regions inside the B cell. Most of these mutations had affected the hydrophobicity score except N:G215C, N:A217S, and N:R385K. Three mutations of M protein, M:F28L, M:I82S, and M:I82T, were located in both MHC class I and class II epitopes. 23 out of the 25 mutations in the spike protein overlap with B-cell, MHC class I, and MHC class II epitopic regions except mutations S:A222V and S:D950N (Table S6). Mutations such as S:H49Y, S:E154K, S:Q1071H, S:H1101D, S:D1118H, and S:V1230L had little or no effect on the hydrophobicity score while others had prominent changes. We observed the presence of several independent mutations at the same position (N:203, S:681, and M:82), where one amino acid changed to another and eventually dominated in wave 2. A hydropathy shift was observed in these sites N:R203K (0.067) to N:R203M (0.711), S:P681H (0.144) to S:P681R (0), and M:I82S (0.411) to M:I82T (0.422).

### Implications of emerging spike, nucleocapsid, and membrane gene mutations on predictive host-miRNA targets.

Predictive binding of 3500 human miRNAs to the wave defining mutation sites of the spike (S), nucleocapsid (N), and membrane (M) protein-coding genes of the viral genome and to the corresponding reference-type (Wuhan Hu-1) sequences was analyzed *in silico*. 191 host miRNAs were predicted to target the reference-type sequences of S, N, and M genes through their seed region, while the binding of all these miRNAs was completely disrupted due to any of those wave-defining mutations. Alternatively, a new set of 131 miRNAs were predicted to target the mutated sequences, resulting in a 31.4% reduction in overall miRNA binding to those genes (Fig. S4). Signature mutations of Delta VoC (variants of concern) like S:P681R, S:L452R, and M:I82T, which revealed an upsurge across wave 2, resulted in complete loss of the predictive host-miRNA binding, compared to its respective reference sequence. In addition to this, spike mutations: G142D, A222V, and nucleocapsid mutations: G215C, A217S, T362I, and D377Y (which depicted higher frequency across W2) revealed >50% reduction in host miRNA binding. These results portray that a compromised host miRNA binding is probably giving a selective advantage to these mutations across W2 in the four states of India.

## DISCUSSION

India, the seventh largest country in the world, is a subcontinent of 28 states, and 8 union territories with a total of 727 districts (http://districts.nic.in/sitemap.php). In this study, we included genomic epidemiology data for four major states of India, namely, West Bengal, Maharashtra, Karnataka, and Delhi, which are from the Eastern, Western, Southern, and Northern gateway regions of the country, respectively. These four states of India contributed to 36.65% of the total cases in India as of June 30, 2021 (https://www.covid19india.org). We analyzed SARS-CoV-2 genomes sampled from these states for the following reasons: (i) the presence of a major international airport in each of these states, that serves as an entry route to human movement through the international corridor; (ii) good connectivity of the three transport systems (railway, flights, and buses) throughout the country; (iii) these states have at least one big metro-city and multiple suburban cities with a large number of residents; and (iv) represent one of the north, east, south and west geographic regions of India. All the aforementioned considerations play an active role in enhancing viral transmission during a pandemic. The COVID-19 pandemic has spread fast throughout India in multiple waves. Various factors (lockdown measures, demographic factors, viral fitness, availability of vaccines, host genetics, etc.) have impacted viral transmission during these waves. To understand the SARS-CoV-2 transmission trajectories in the Eastern corridor of India (West Bengal), as well as the other three corridors of India, Maharashtra, Karnataka, and Delhi, major wave window time periods were identified from 2020 to 2021. The genome sequences were sampled and analyzed for these time periods appropriately. SARS-CoV-2 accumulates substitutions at an average rate of one mutation per 2 to 3 transmissions (15). Our study found a downward trend in mutation rate in specific genes across the state of West Bengal from W1 to W2. We found decrease (1.20 to 2.35 fold) in mutation rate on the SARS-CoV-2 genome from PW2 to W2 and simultaneous increase (8.04 to 33.68 fold) in infection count across four states of India (Table S7). We did not observe any quantitative relationship between the number of circulating lineages with the surge in infection in a specific time period (Fig. 1c, Fig. S1c, Fig. S2c, and Fig. S3c). Toward the end of W2, lineage diversity reduced and only one lineage, B.1.617.2 (Delta lineage), existed in all four states. This indicates that viral transmissibility and fitness were considerably high during W2.

We explored the spatial and temporal variations in the sequences of the B.1.617.2 parent lineage to identify the emerging mutations which may have the potential to give rise to a new microlineage during the progression of the pandemic (Table 2). Phylogenetic analysis revealed seven microlineage subclusters (C1 to C7) of the parent lineage B.1.617.2 from the four states. Multiple microlineages of the parent Delta variant indicate aggregation of mutations over a period of time, reaffirming the importance and need for genomic surveillance of transmission in these states. Within the ORF1 polyprotein gene (ORF1a and ORF1b), mutations in nsp3, nsp4, nsp6, and nsp15 contributed mostly to the formation of microlineages. Mutation in nsp4 and nsp6 proteins may affect replication kinetics as these are associated with the formation of replication compartments (16). Previous research on SARS-CoV and Middle East respiratory syndrome (MERS) has shown that ORF1a has a role in survival and adaptability to the host and that its positive selection may lead to intrahost transmission or immune evasion (17–19). In the nucleocapsid gene, mutation at position 362 (RNA binding dimerization domain) and 385 (intrinsically disordered regions [IDRs] of the C-terminal domain [CTD] domain) contributed to the formation of microlineages. The IDRs are crucial for several biological mechanisms that contribute to viral pathogenicity (20). The mutation T362I has been shown to play a crucial role in T-cell evasion in experimental systems (21). On the spike protein, two mutations, K77T and A222V, were major contributors to the formation of subclusters. Our micro lineage analysis found ORF1a and N genes have the most cluster contributing defining mutations suggesting a higher potential for the continuous evolution of these proteins.

We measured the hydrophobicity profiles of the wave-associated mutations to understand structural motif changes in the epitope regions of the viral proteins. Antibodies generated by the host immune system and/or vaccine-induced immunity, interact and clear the viruses by recognizing the epitope regions of the viral proteins. The hydropathic shift was observed in the dominant mutations of spike, envelope, and membrane genes of the most dominant mutations in wave 2 across all the states. In the nucleocapsid gene, hydrophilic (lysine [R203K]) amino acid was replaced by hydrophobic amino acid (methionine [R203M]) and in the spike gene the hydrophobic (histidine [P681H]) was replaced by hydrophilic (arginine [P681R]) where in membrane gene, hydrophobic (aerine [I82S]) was substituted by slightly polar (threonine [I82T]) residue. We found most of the mutations that evolved during the second wave changed the hydrophobicity score of the corresponding epitope regions particularly mutations, N:D63G, N:R203M, and N:D377Y, inside nucleocapsid protein, S:L452R and S:P681R inside the spike proteins of the SARS-CoV-2. These mutations were found to have various roles in the spread of infection, N:D63G might increase immune escape abilities (22), N:D377Y might affect the antibody binding (23–25), S:L452R might be involved in decreased neutralization by MAbs (26), and S:P681R might be involved in furin cleavage (27).

To further understand the contribution of host molecular arsenals toward the emergence of important mutations during pandemic waves, we analyzed the predictive host miRNA-viral RNA interaction through bioinformatic approaches. As replication of positive-strand RNA viruses simulates host mRNAs, thereby permitting host cellular-miRNAs to target viral RNA, mimicking host mRNA regulation. Upon binding, specific host miRNAs can inhibit viral protein translation, subsequently compromising virus replication (28, 29). In a previous study from our group, we investigated the alteration in miRNA targets in the nucleocapsid coding region due to the mutation RG203-204KR (28881-3 GGG/AAC) in the first 9 SARS-CoV-2 sequences reported from eastern India (30). In this study, we have extended our analyses to various mutations in the viral structural genes and the rise in frequency of a few mutants across W2 can be assigned to one such probable cause where the absence of any miRNA binding can augment virus propagation. As evident from our analysis, mutations like P681R, L452R in spike, and I82T in membrane protein resulted in complete loss of host-miRNA binding. P681R mutation is unique in context to its emergence solely in Delta variant to date, and thereby, Delta might have gained a selection advantage across W2 in the absence of any host-miRNA roadblock.

In our study, hsa-let-7a-3p, which has been known to decrease in SARS-CoV-2 infected lung cells, was one of the miRNAs whose binding was disrupted upon spike gene mutation L452R (31). In this way, the mutant virus could have gained a selection benefit through the abolition of the hsa-let-7a-3p effect on its propagation in the lungs. To support this effect, we analyzed another contrasting case of the omnipotent D614G spike mutant sequence, revealing binding to hsa-miR-122b-3p. hsa-miR-122 family has been shown to facilitate hepatitis C virus replication in the liver; hence, its binding can equally promote SARS-CoV-2 associated liver pathogenesis (32). SARS-CoV-2 replication within human cells is restricted by host miRNA defense machinery, and the viral genomic sites targeted by several cellular miRNAs are lost due to mutations within the SARS-CoV-2 genome. Several other *in silico* studies have been conducted where many human encoded miRNAs, which are potentially targeting the SARS-CoV-2 genome, have been predicted and some of their pro- and antiviral roles have been underscored (33–36). In this way, host determinants like miRNAs might modulate the adaptive selection of the emerging SARS-CoV-2 variants. However, further experimental validation is the key to a complete understanding of the consequences of our predictive miRNA binding.

Overall, our analyses shed light on the evolution of major viral lineages and sublineages that have driven two major pandemic waves in four major states of India. We were able to find contrasting molecular profiles of wave-defining mutations that have shaped the spatiotemporal circulation of viral lineages in major states of India. This knowledge will be useful in the management of future pandemic waves in India.

## MATERIALS AND METHODS

### Clinical sample collection from the state of West Bengal, India.

West Bengal samples sequenced were undertaken by the National Institute of Biomedical Genomics (NIBMG), Kalyani as part of this study were undertaken by the initiative of Indian SARS-CoV-2 Genomics Consortium (INSACOG) (https://dbtindia.gov.in/insacog) and PAN India 1000 SARS-CoV-2 Genomics Consortium (37). Viral RNA isolated from nasopharyngeal and oropharyngeal swabs that tested positive for SARS-CoV-2 by qRT-PCR were sequenced as per the established protocol (30). About 3,393 good-quality SARS-CoV-2 genomes were sequenced from 22 districts of West Bengal (April 2, 2020 to June 19, 2021) during our study period for 2 years encompassing the first and second waves.

### Viral genome sequencing of West Bengal samples.

Viral whole-genome sequences included in this analysis were generated using any one of the following approaches, i.e., paired-end sequencing of total RNA libraries after rRNA depletion using Truseq stranded total RNA library preparation kit (Illumina), amplification of the SARS-CoV-2 genome using standard Arctic primers primarily using COVID-Seq kits (Illumina) or Qiaseq (Qiagen) using Novaseq or Miseq platforms (Illumina).

### Sequence data aggregation for the other three states of India.

To compare and contrast the temporal dynamics of SARS-CoV-2 lineages in the state of West Bengal with other geographic regions of India, three states were selected having at least one major international airport and at least one densely populated metro city. SARS-CoV-2 genome sequences from North India (Delhi [*n* = 3,905]), West India (Maharashtra [*n* = 6,025]), and South India (Karnataka [*n* = 1,950]) states, were obtained from GISAID (https://www.gisaid.org/) for an overlapping period of first and second waves with the constraint that the collection date was provided for comparison with data from West Bengal. The SARS-CoV-2 sequences with >10% N content and genome length <29,000 bases were filtered out.

### Epidemiological data collection and defining wave-window periods for each state.

**FIG 5:**
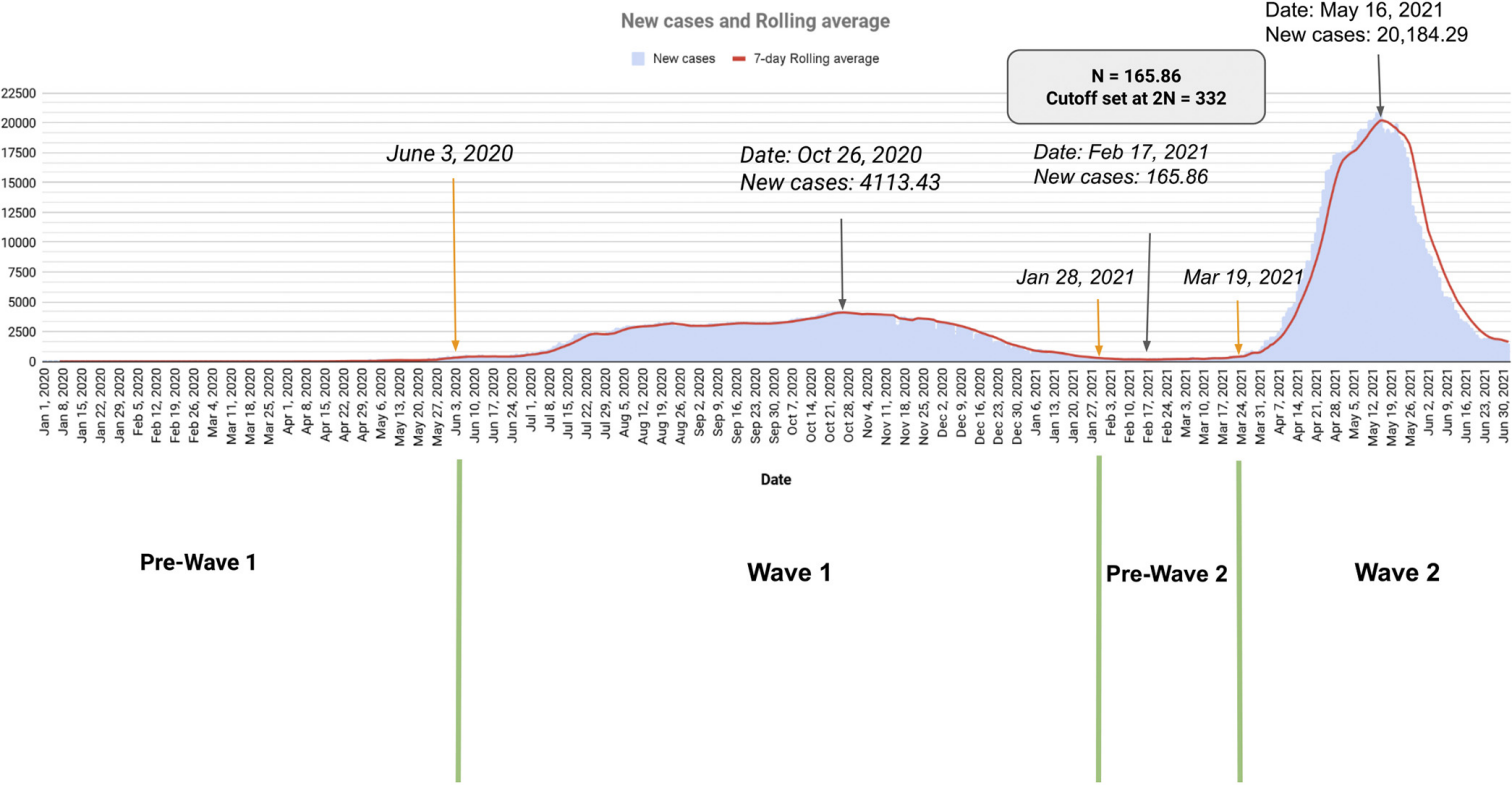
Selection of wave-window time periods based on the daily infection counts from the state of West Bengal.

The data for the new infections in the West Bengal state were collected from the WB corona bulletin (https://www.wbhealth.gov.in/pages/corona/bulletin) provided by the West Bengal government health and family welfare department. We utilized publicly supported time series infection information accessible on the web by covid19india (https://www.covid19india.org) for other states. The information in this database was directly sourced from individual states’ daily government corona bulletin. It contains state-wise data on total cases, total deceased, and total recovered. The data were obtained from the API provided by them (https://data.covid19india.org/csv/latest/states.csv) using Python scripts on June 30, 2021 for the current analysis (Table S2). Since the data for new infections are highly variable across each state, a 7-day rolling average was taken for further analysis (Fig. 1a and b, Fig. S1a and b, Fig. S2a and b, and Fig. S3a and b). To calculate wave window periods of each state, the lowest value of the 7-day rolling average between the highest infection peaks of the first and second wave was selected and labeled as N. Specific time points on the infection graph were selected when the number of new infections increased to 2N or decreased to 2N to define four-wave time-windows: Pre-Wave 1 (PW1), Wave 1 (W1), Pre-Wave (PW2), and Wave 2 (W2) were defined for each state (details in Fig. 5). The dates for each wave window category in the selected state are provided in Table 3.

**TABLE 3:** Date windows and number of sequenced genomes corresponding to the start and end of the various wave category for each of the four selected states of India

State	Pre-Wave 1 (PW1)		Wave 1 (W1)		Pre-Wave 2 (PW2)		Wave 2 (W2)	
	Time window (start to end)	Genome Sequenced	Time window (start to end)	Genome sequenced	Time window (start to end)	Genome Sequenced	Time window (start to end)	Genome sequenced
West Bengal	17-Mar-20 to 03-Jun-20	192	04-Jun-20 to 28-Jan-21	461	29-Jan-21 to 19-Mar-21	597	20-Mar-21 to 30-Jun-21	1,948
Delhi	02-Mar-20 to 16-JuL-20	328	17-JuL-20 to 27-Dec-20	960	28-Dec-20 to 13-Mar-21	596	14-Mar-21 to 30-Jun-21	1,241
Maharashtra	09-Mar-20 to 28-Jun-20	684	29-Jun-20 to 05-Dec-20	783	06-Dec-20 to 20-Feb-21	656	21-Feb-21 to 30-Jun-21	3,538
Karnataka	08-Mar-20 to 29-Jun-20	216	30-Jun-20 to 12-Jan-21	269	13-Jan-21 to 14-Mar-21	210	15-Mar-21 to 30-Jun-21	1,016

### Analysis of sequencing data for viral lineage assignment.

Reference mapping-based alignment of the genomes was performed using MAFFT version 7.453 (38) using MN908947 as the reference. All quality control (QC) passed genomes were evaluated through the command-line version of Nextstrain’s NextClade (v1.2) software (https://github.com/nextstrain/nextclade) to obtain a list of all nucleotide and amino-acid substitutions. To generate the community standard pangolin lineage nomenclature, we used the pangolin software package comprising of Pangolin v3.1.5 (39) (https://github.com/cov-lineages/pangolin), PangoLEARN v2021-06-15 (https://github.com/cov-lineages/pangoLEARN), pango-designation v1.2.32 (https://github.com/cov-lineages/pango-designation), scorpio v0.3.4 (https://github.com/cov-lineages/scorpio), and constellations v0.0.9 (https://github.com/cov-lineages/constellations). Standard Unix command line scripts and python data plotting packages (https://github.com/animesh-workplace/Manuscript-MS-2022) were utilized to analyze temporal data for each state and to identify viral lineages in the circulation of a specific window. The collection date of the sample was used for all temporal analysis.

### Phylodynamic analysis of SARS-CoV-2 sequences.

After initial QC, the number of sequences available for downstream phylodynamic analysis from each state reduced to West Bengal (*n* = 3,198), Delhi (*n* = 3,125), Maharashtra (*n* = 5,661), and Karnataka (*n* = 1,711), respectively. All QC passed genomes were used for phylogenetic inference. Nextstrain’s Augur (40) version 12.0.0 pipeline utilized by Nextstrain filtered out genomes that have a minimum length of 27,000 bp before aligning them using the MAFFT package. As a reference, the Nextstrain nCoV toolkit (41) (https://github.com/nextstrain/ncov) aligns SARS-CoV-2 sequences to the SARS-CoV-2 isolate Wuhan-Hu-1 complete genome (MN908947). The pipeline implements a maximum likelihood phylogenetic analysis using the general time-reversible model allowing for invariant sites and a gamma distribution (GTR+I+G) in IQ-TREE (42) version 2.1.2. Augur further refines and annotates the tree in various ways. The resulting files are combined and used by Auspice version 2.23.0 (https://github.com/nextstrain/auspice) to visualize phylogenetic relationships and geographic distributions of SARS-CoV-2 across time. To predict the pathogenicity of nonsynonymous substitutions (ns-substitutions) responsible for a specific cluster on the phylogenetic tree, we utilized predictSNP (https://loschmidt.chemi.muni.cz/predictsnp/) that integrates machine learning-based consensus classifier of six algorithms, including SIFT, PhD-SNP, PolyPhen-1, PolyPhen-2, MAPP, and SNAP. In addition, to understand the underlying dynamics of substitution, we calculated the number of sites mutated per gene (mutation rate) in each wave category normalized by the number of genomes used for analysis for a specific state in that time window.

### Prediction and hydrophobicity profiles of epitope regions.

The prediction of host B-cell, MHC class I, and MHC class II epitopic region of the nucleocapsid (N), spike (S), membrane (M), and envelope (E) proteins of the virus were carried out using the Immune Epitope Database (IEDB) portal (https://www.iedb.org/). The hydrophobicity profile of the epitopic regions was estimated through Kyte-Doolittle plot using the PROTSCALE program in EXPASY online tools. A five amino acid slide window was used to normalize the values between 0 and 1. The plot for the hydrophobicity score has been generated by GraphPad Prism 5.0 package.

### Analyses of structural and functional implications of the preponderant SARS-CoV-2 mutations for host-miRNA binding.

To understand the plausible host-miRNA binding sites across the protein-coding regions of SARS-CoV-2 transcripts, the STarMir (http://sfold.wadsworth.org/cgi-bin/starmirtest2.pl) software was used to identify host-miRNA targets of the mutated sequences (those mutations were taken into considerations which showed a rise in frequency above 10% during PW1, W1, PW2, and W2 time windows across all the 4 states). Mainly, spike, nucleocapsid, and membrane protein-coding genes of the virus were analyzed. The database on 3,500 human miRNA library was taken from the miRbase repository (http://microrna.sanger.ac.uk/), and subsequently, only those miRNAs were taken for downstream analysis for which binding to the SARS-CoV-2 coding transcripts was seen via their seed sequence, both with the reference type as well as mutated regions. A flanking 50 nucleotides upstream and downstream sequence of the mutated site was used for the above analysis.

### Code availability.

The code used for analyzing the temporal data for each state is available on the GitHub Link https://github.com/animesh-workplace/Manuscript-MS-2022.

### Ethical aspects.

The study was approved by the Institutional Ethics Committee of the National Institute of Biomedical Genomics.

## References

[1] General WD. 2020. WHO Director General’s opening remarks at the media briefing on COVID-19. World Health Organization, Geneva, Switzerland.

[2] Callaway E. 2020. The coronavirus is mutating–does it matter? Nature 585:174–177. doi:10.1038/d41586-020-02544-6. 32901123

[3] Grubaugh ND, Petrone ME, Holmes EC. 2020. We shouldn’t worry when a virus mutates during disease outbreaks. Nat Microbiol 5:529–530. doi:10.1038/s41564-020-0690-4. 32071422 PMC7095397

[4] V’Kovski P, Kratzel A, Steiner S, Stalder H, Thiel V. 2021. Coronavirus biology and replication: implications for SARS-CoV-2. Nat Rev Microbiol 19:155–170. doi:10.1038/s41579-020-00468-6. 33116300 PMC7592455

[5] Lucas M, Karrer U, Lucas A, Klenerman P. 2001. Viral escape mechanisms–escapology taught by viruses. Int J Exp Pathol 82:269–286. doi:10.1046/j.1365-2613.2001.00204.x. 11703537 PMC2517780

[6] Shah VK, Firmal P, Alam A, Ganguly D, Chattopadhyay S. 2020. Overview of immune response during SARS-CoV-2 infection: lessons from the past. Front Immunol 11:1949. doi:10.3389/fimmu.2020.01949. 32849654 PMC7426442

[7] Day T, Gandon S, Lion S, Otto SP. 2020. On the evolutionary epidemiology of SARS-CoV-2. Curr Biol 30:R849–R857. doi:10.1016/j.cub.2020.06.031. 32750338 PMC7287426

[8] van Dorp L, Richard D, Tan CCS, Shaw LP, Acman M, Balloux F. 2020. No evidence for increased transmissibility from recurrent mutations in SARS-CoV-2. Nat Commun 11:5986. doi:10.1038/s41467-020-19818-2. 33239633 PMC7688939

[9] Braun KM, Moreno GK, Halfmann PJ, Hodcroft EB, Baker DA, Boehm EC, Weiler AM, Haj AK, Hatta M, Chiba S, Maemura T, Kawaoka Y, Koelle K, O’Connor DH, Friedrich TC. 2021. Transmission of SARS-CoV-2 in domestic cats imposes a narrow bottleneck. PLoS Pathog 17:e1009373. doi:10.1371/journal.ppat.1009373. 33635912 PMC7946358

[10] Bhattacharyya C, Das C, Ghosh A, Singh AK, Mukherjee S, Majumder PP, Basu A, Biswas NK. 2021. SARS-CoV-2 mutation 614G creates an elastase cleavage site enhancing its spread in high AAT-deficient regions. Infect Genet Evol 90:104760. doi:10.1016/j.meegid.2021.104760. 33556558 PMC7863758

[11] Mackenzie JS, Smith DW. 2020. COVID-19: a novel zoonotic disease caused by a coronavirus from China: what we know and what we don’t. Microbiol Austral 41:45–50. doi:10.1071/MA20013. PMC7086482 32226946

[12] Gordon DE, Jang GM, Bouhaddou M, Xu J, Obernier K, White KM, O’Meara MJ, Rezelj VV, Guo JZ, Swaney DL, Tummino TA, Hüttenhain R, Kaake RM, Richards AL, Tutuncuoglu B, Foussard H, Batra J, Haas K, Modak M, Kim M, Haas P, Polacco BJ, Braberg H, Fabius JM, Eckhardt M, Soucheray M, Bennett MJ, Cakir M, McGregor MJ, Li Q, Meyer B, Roesch F, Vallet T, Mac Kain A, Miorin L, Moreno E, Naing ZZC, Zhou Y, Peng S, Shi Y, Zhang Z, Shen W, Kirby IT, Melnyk JE, Chorba JS, Lou K, Dai SA, Barrio-Hernandez I, Memon D, Hernandez-Armenta C, et al. 2020. A SARS-CoV-2 protein interaction map reveals targets for drug repurposing. Nature 583:459–468. doi:10.1038/s41586-020-2286-9. 32353859 PMC7431030

[13] Laha S, Chakraborty J, Das S, Manna SK, Biswas S, Chatterjee R. 2020. Characterizations of SARS-CoV-2 mutational profile, spike protein stability and viral transmission. Infect Genet Evol 85:104445. doi:10.1016/j.meegid.2020.104445. 32615316 PMC7324922

[14] Shen L, Bard JD, Triche TJ, Judkins AR, Biegel JA, Gai X. 2021. Emerging variants of concern in SARS-CoV-2 membrane protein: a highly conserved target with potential pathological and therapeutic implications. Emerg Microbes Infect 10:885–893. doi:10.1080/22221751.2021.1922097. 33896413 PMC8118436

[15] Komissarov AB, Safina KR, Garushyants SK, Fadeev AV, Sergeeva MV, Ivanova AA, Danilenko DM, Lioznov D, Shneider OV, Shvyrev N, Spirin V, Glyzin D, Shchur V, Bazykin GA. 2021. Genomic epidemiology of the early stages of the SARS-CoV-2 outbreak in Russia. Nat Commun 12:649. doi:10.1038/s41467-020-20880-z. 33510171 PMC7844267

[16] Obermeyer F, Schaffner SF, Jankowiak M, Barkas N, Pyle JD, Park DJ, MacInnis BL, Luban J, Sabeti PC, Lemieux JE. 2021. Analysis of 2.1 million SARS-CoV-2 genomes identifies mutations associated with transmissibility. medRxiv doi:10.1101/2021.09.07.21263228. PMC9161372 35608456

[17] Forni D, Cagliani R, Mozzi A, Pozzoli U, Al-Daghri N, Clerici M, Sironi M. 2016. Extensive positive selection drives the evolution of nonstructural proteins in lineage C betacoronaviruses. J Virol 90:3627–3639. doi:10.1128/JVI.02988-15. 26792741 PMC4794664

[18] Graham RL, Sparks JS, Eckerle LD, Sims AC, Denison MR. 2008. SARS coronavirus replicase proteins in pathogenesis. Virus Res 133:88–100. doi:10.1016/j.virusres.2007.02.017. 17397959 PMC2637536

[19] Sant’Anna FH, Muterle Varela AP, Prichula J, Comerlato J, Comerlato CB, Roglio VS, Mendes Pereira GF, Moreno F, Seixas A, Wendland EM. 2021. Emergence of the novel SARS-CoV-2 lineage VUI-NP13L and massive spread of P.2 in South Brazil. Emerg Microbes Infect 10:1431–1440. doi:10.1080/22221751.2021.1949948. 34184973 PMC8284128

[20] Yesudhas D, Srivastava A, Sekijima M, Gromiha MM. 2021. Tackling Covid-19 using disordered-to-order transition of residues in the spike protein upon angiotensin-converting enzyme 2 binding. Proteins 89:1158–1166. doi:10.1002/prot.26088. 33893649 PMC8251098

[21] de Silva TI, Liu G, Lindsey BB, Dong D, Moore SC, Hsu NS, Shah D, Wellington D, Mentzer AJ, Angyal A, Brown R, Parker MD, Ying Z, Yao X, Turtle L, Dunachie S; COVID-19 Genomics UK (COG-UK) Consortium, Maini MK, Ogg G, Knight JC; ISARIC4C Investigators, Peng Y, Rowland-Jones SL, Dong T. 2021. The impact of viral mutations on recognition by SARS-CoV-2 specific T-cells. iScience 24:103353. doi:10.1016/j.isci.2021.103353. 34729465 PMC8552693

[22] Liu W, Li H. 2021. COVID-19: attacks immune cells and interferences with antigen presentation through MHC-like decoy system. ChemRxiv doi:10.26434/chemrxiv-2021-m7456. PMC9987643 36799912

[23] Moody R, Wilson KL, Boer JC, Holien JK, Flanagan KL, Jaworowski A, Plebanski M. 2021. Predicted B cell epitopes highlight the potential for COVID-19 to drive self-reactive immunity. Front Bioinform 1:709533. doi:10.3389/fbinf.2021.709533. 36303764 PMC9581003

[24] Osterman A, Badell I, Basara E, Stern M, Kriesel F, Eletreby M, Öztan GN, Huber M, Autenrieth H, Knabe R, Späth PM, Muenchhoff M, Graf A, Krebs S, Blum H, Durner J, Czibere L, Dächert C, Kaderali L, Baldauf H-M, Keppler OT. 2022. Impaired detection of omicron by SARS-CoV-2 rapid antigen tests. Med Microbiol Immunol 211:105–117. doi:10.1007/s00430-022-00730-z. 35187580 PMC8858605

[25] Jungnick S, Hobmaier B, Mautner L, Hoyos M, Haase M, Baiker A, Lahne H, Eberle U, Wimmer C, Hepner S, Sprenger A, Berger C, Dangel A, Ippisch S, Hahner S, Wildner M, Liebl B, Ackermann N, Sing A, Fingerle V. 2021. In vitro rapid antigen test performance with the SARS-CoV-2 variants of concern B.1.1.7 (alpha), B.1.351 (beta), P.1 (gamma), and B.1.617.2 (delta). Microorganisms 9:1967. doi:10.3390/microorganisms9091967. 34576862 PMC8465346

[26] Harvey WT, Carabelli AM, Jackson B, Gupta RK, Thomson EC, Harrison EM, Ludden C, Reeve R, Rambaut A, Peacock SJ, Robertson DL, COVID-19 Genomics UK (COG-UK) Consortium. 2021. SARS-CoV-2 variants, spike mutations and immune escape. Nat Rev Microbiol 19:409–424. doi:10.1038/s41579-021-00573-0. 34075212 PMC8167834

[27] Cherian S, Potdar V, Jadhav S, Yadav P, Gupta N, Das M, Rakshit P, Singh S, Abraham P, Panda S, Team NIC. 2021. SARS-CoV-2 spike mutations, L452R, T478K, E484Q and P681R, in the second wave of COVID-19 in Maharashtra, India. Microorganisms 9:1542. doi:10.3390/microorganisms9071542. 34361977 PMC8307577

[28] Trobaugh DW, Klimstra WB. 2017. MicroRNA regulation of RNA virus replication and pathogenesis. Trends Mol Med 23:80–93. doi:10.1016/j.molmed.2016.11.003. 27989642 PMC5836316

[29] Trobaugh DW, Gardner CL, Sun C, Haddow AD, Wang E, Chapnik E, Mildner A, Weaver SC, Ryman KD, Klimstra WB. 2014. RNA viruses can hijack vertebrate microRNAs to suppress innate immunity. Nature 506:245–248. doi:10.1038/nature12869. 24352241 PMC4349380

[30] Maitra A, Sarkar MC, Raheja H, Biswas NK, Chakraborti S, Singh AK, Ghosh S, Sarkar S, Patra S, Mondal RK, Ghosh T, Chatterjee A, Banu H, Majumdar A, Chinnaswamy S, Srinivasan N, Dutta S, Das S. 2020. Mutations in SARS-CoV-2 viral RNA identified in Eastern India: possible implications for the ongoing outbreak in India and impact on viral structure and host susceptibility. J Biosci 45:76. doi:10.1007/s12038-020-00046-1. 32515358 PMC7269891

[31] Chow JT, Salmena L. 2020. Prediction and analysis of SARS-CoV-2-targeting microRNA in human lung epithelium. Genes (Basel) 11:1002. doi:10.3390/genes11091002. 32858958 PMC7565861

[32] Jopling CL, Yi M, Lancaster AM, Lemon SM, Sarnow P. 2005. Modulation of hepatitis C virus RNA abundance by a liver-specific microRNA. Science 309:1577–1581. doi:10.1126/science.1113329. 16141076

[33] Jafarinejad-Farsangi S, Jazi MM, Rostamzadeh F, Hadizadeh M. 2020. High affinity of host human microRNAs to SARS-CoV-2 genome: an in silico analysis. Noncoding RNA Res 5:222–231. doi:10.1016/j.ncrna.2020.11.005. 33251388 PMC7680021

[34] Siniscalchi C, Di Palo A, Russo A, Potenza N. 2021. Human microRNAs interacting with SARS-CoV-2 RNA sequences: computational analysis and experimental target validation. Front Genet 12:678994. doi:10.3389/fgene.2021.678994. 34163530 PMC8215607

[35] Hosseini Rad Sm A, McLellan AD. 2020. Implications of SARS-CoV-2 mutations for genomic RNA structure and host microRNA targeting. Int J Mol Sci 21:4807. doi:10.3390/ijms21134807. 32645951 PMC7370282

[36] Sardar R, Satish D, Birla S, Gupta D. 2020. Integrative analyses of SARS-CoV-2 genomes from different geographical locations reveal unique features potentially consequential to host-virus interaction, pathogenesis and clues for novel therapies. Heliyon 6:e04658. doi:10.1016/j.heliyon.2020.e04658. 32844125 PMC7439967

[37] Maitra A, Raghav S, Dalal A, Ali F, Paynter VM, Paul D, Biswas NK, Ghosh A, Jani K, Chinnaswamy S, Pati S, Sahu A, Mitra D, Bhat MK, Mayor S, Sarin A, The PAN-INDIA 1000 SARS-CoV-2 RNA Genome Sequencing Consortium, Sauche YS, Seshasayee ASN, Palakodeti D, Bashyam MD, Parida A, Das S. 2020. PAN-INDIA 1000 SARS-CoV-2 RNA genome sequencing reveals important insights into the outbreak. bioRxiv. doi:10.1101/2020.08.03.233718.

[38] Katoh K, Misawa K, Kuma K-i, Miyata T. 2002. MAFFT: a novel method for rapid multiple sequence alignment based on fast Fourier transform. Nucleic Acids Res 30:3059–3066. doi:10.1093/nar/gkf436. 12136088 PMC135756

[39] Rambaut A, Holmes EC, O’Toole Á, Hill V, McCrone JT, Ruis C, Du Plessis L, Pybus OG. 2020. A dynamic nomenclature proposal for SARS-CoV-2 lineages to assist genomic epidemiology. Nat Microbiol 5:1403–1407. doi:10.1038/s41564-020-0770-5. 32669681 PMC7610519

[40] Huddleston J, Hadfield J, Sibley TR, Lee J, Fay K, Ilcisin M, Harkins E, Bedford T, Neher RA, Hodcroft EB. 2021. Augur: a bioinformatics toolkit for phylogenetic analyses of human pathogens. J Open Source Softw 6:2906. doi:10.21105/joss.02906. 34189396 PMC8237802

[41] Hadfield J, Megill C, Bell SM, Huddleston J, Potter B, Callender C, Sagulenko P, Bedford T, Neher RA. 2018. Nextstrain: real-time tracking of pathogen evolution. Bioinformatics 34:4121–4123. doi:10.1093/bioinformatics/bty407. 29790939 PMC6247931

[42] Minh BQ, Schmidt HA, Chernomor O, Schrempf D, Woodhams MD, von Haeseler A, Lanfear R. 2020. IQ-TREE 2: new models and efficient methods for phylogenetic inference in the genomic era. Mol Biol Evol 37:1530–1534. doi:10.1093/molbev/msaa015. 32011700 PMC7182206

