# Supplementary material for: Contrasting Distribution of SARS-CoV-2 Lineages across Multiple Rounds of Pandemic Waves in West Bengal, the Gateway of East and North-East States of India
Source: Microbiol Spectr. 2022 Jul 19;10(4):e00914-22. doi: 10.1128/spectrum.00914-22 (PMC9430150; doi:10.1128/spectrum.00914-22)
Supplement: Supplemental file 1 — Supplemental material. Download spectrum.00914-22-s0001.pdf, PDF file, 7.3 MB [file spectrum.00914-22-s0001.pdf]

Figure S1. (a) Numbers of new daily SARS-CoV-2 infected cases (blue) and 7-day rolling average (red), (b) Map of the state of Delhi with district-wise cumulative new infection distribution is highlighted, (c) Comparison between circulating lineages (upper panel - Red) and new infection cases (grey) in pandemic wave windows in the state of Delhi, (d) Circulating lineage with pangolin nomenclature across four wave-windows, (e) Phylogenetic time tree of circulating SARS sequences sampled from Delhi state during the study period.

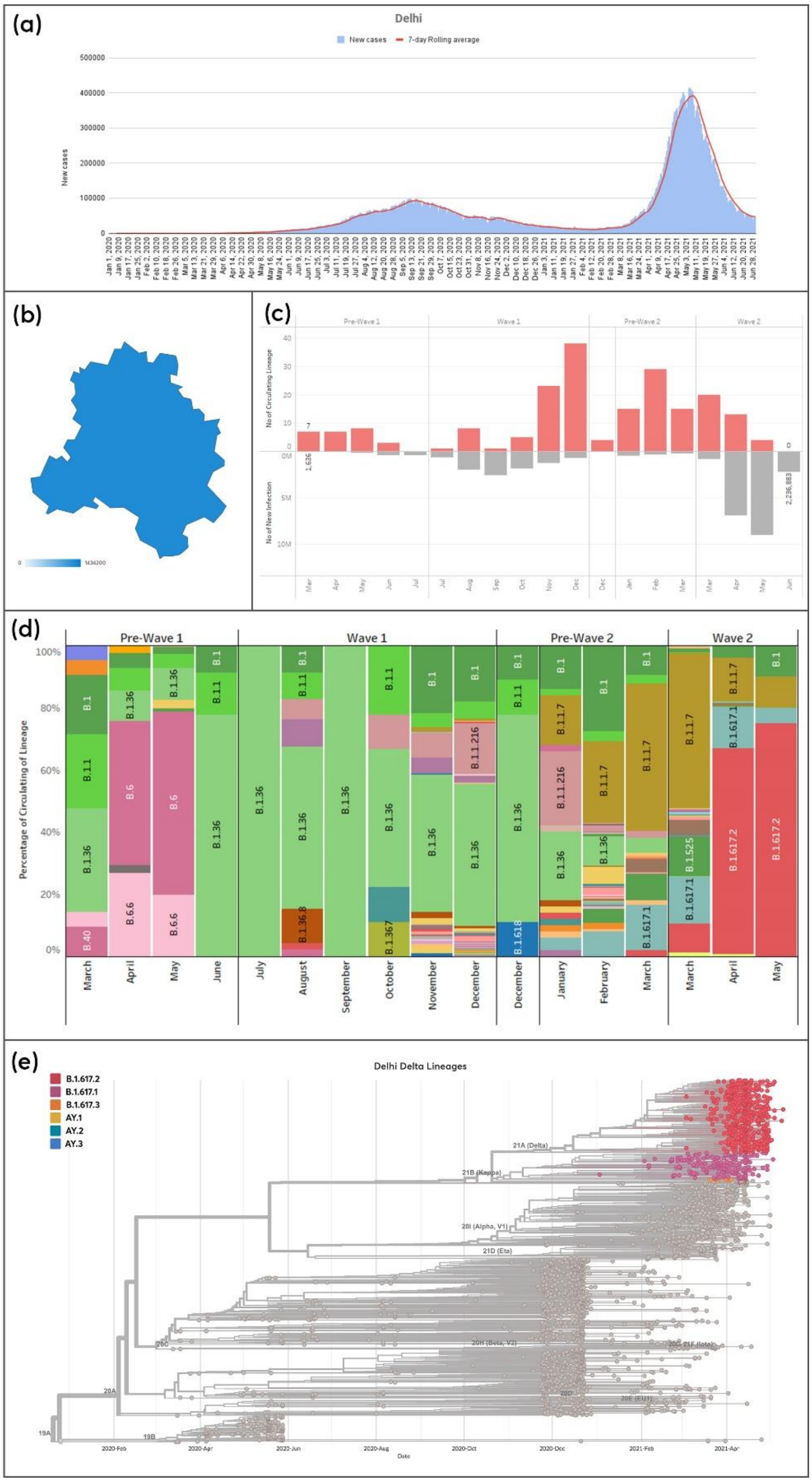

Figure S2. (a) Numbers of new daily SARS-CoV-2 infected cases (blue) and 7-day rolling average (red), (b) Map of the state of Karnataka with district-wise cumulative new infection distribution is highlighted, (c) Comparison between circulating lineages (upper panel - Red) and new infection cases (grey) in pandemic wave windows in the state of Karnataka, (d) Circulating lineage with pangolin nomenclature across four wave-windows, (e) Phylogenetic time tree of circulating SARS sequences sampled from Karnataka state during the study period.

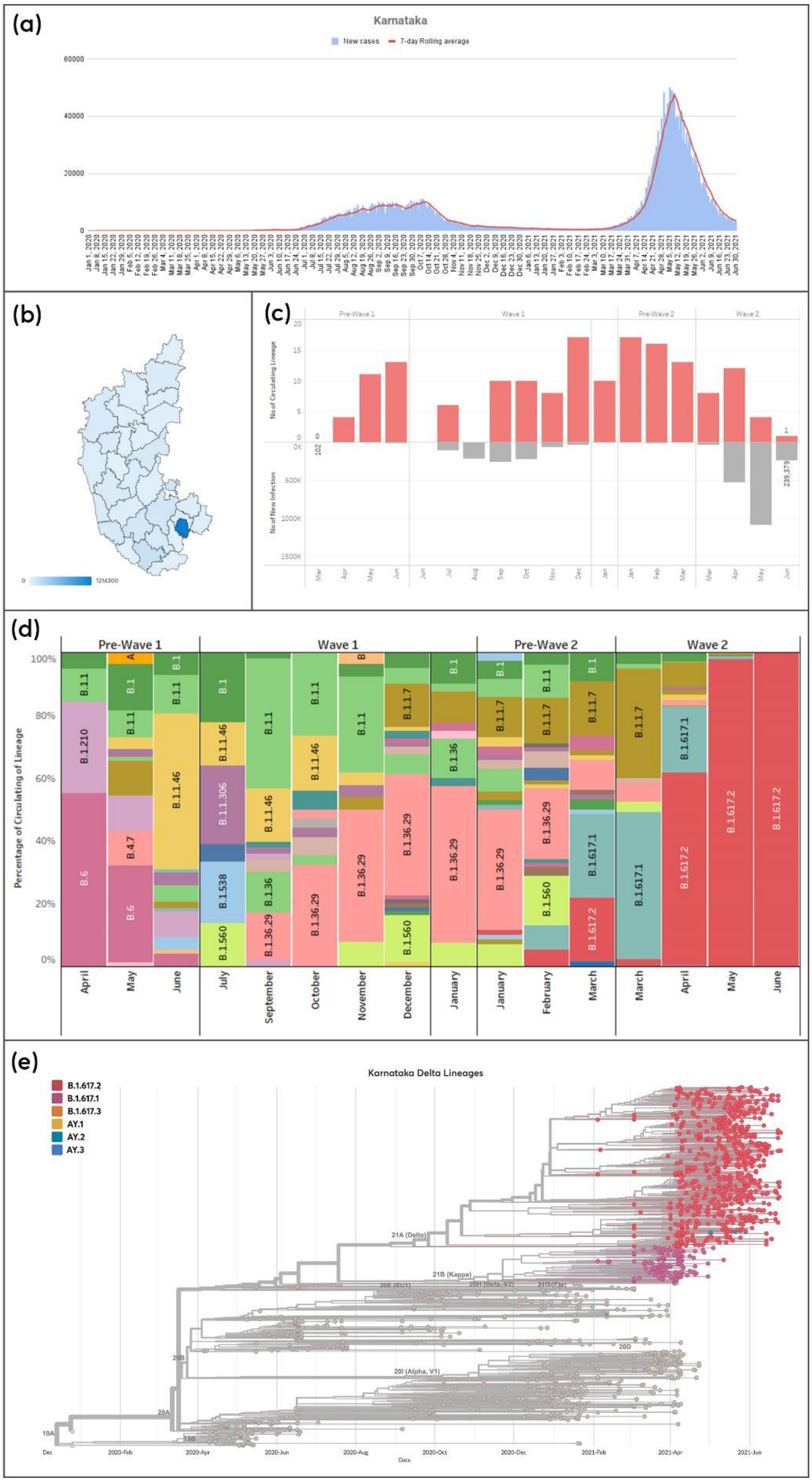

Figure S3. Numbers of new daily SARS-CoV-2 infected cases (blue) and 7-day rolling average (red), (b) Map of the state of Maharashtra with district-wise cumulative new infection distribution is highlighted, (c) Comparison between circulating lineages (upper panel - Red) and new infection cases (grey) in pandemic wave windows in the state of Maharashtra, (d) Circulating lineage with pangolin nomenclature across four wave-windows, (e) Phylogenetic time tree of circulating SARS sequences sampled from Maharashtra state during the study period.

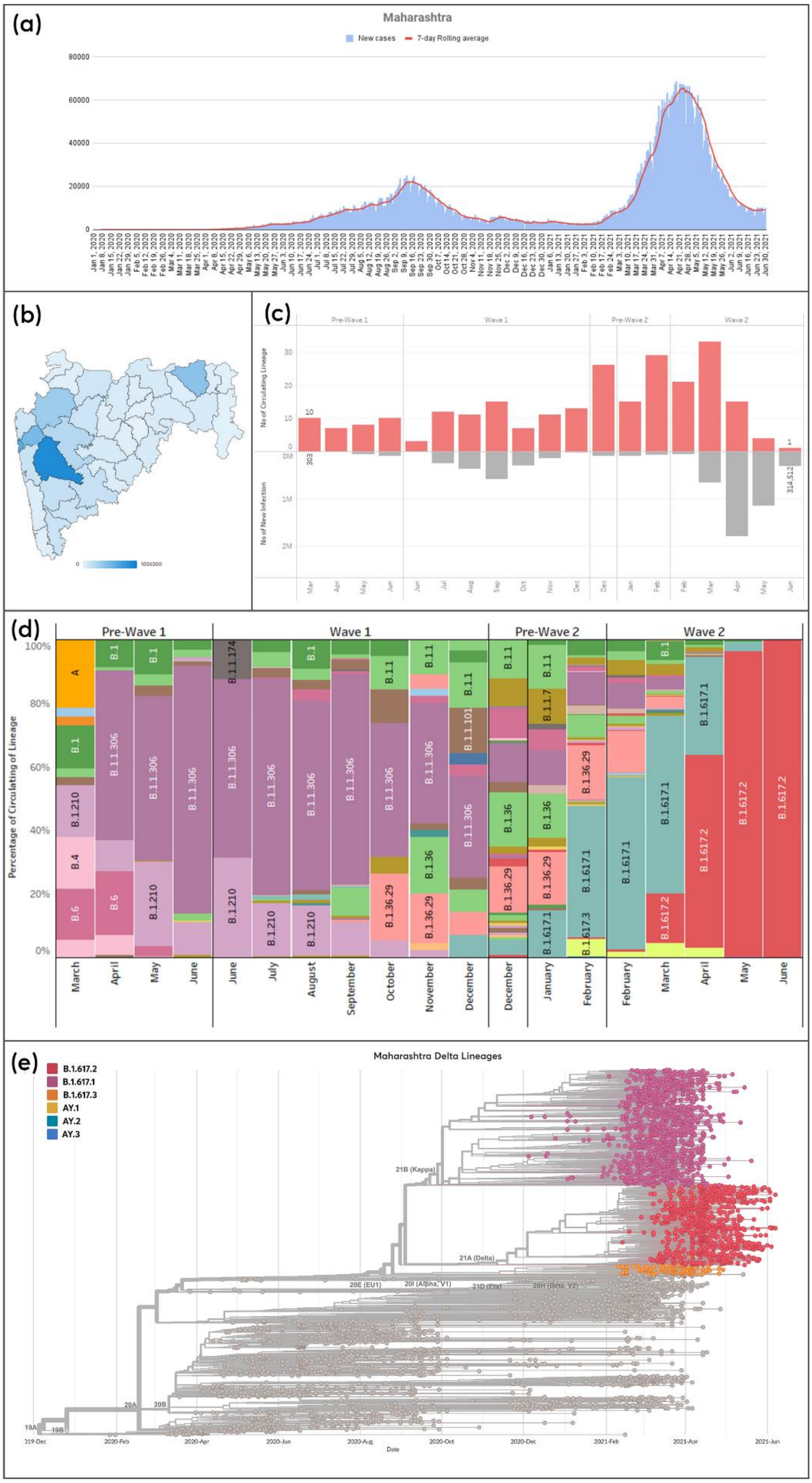

Figure S4. Putative human miRNA binding atlas to reference-type and mutated sites of – (a) spike, (b) nucleocapsid, and (c) membrane encoding gene of SARS-CoV-2.

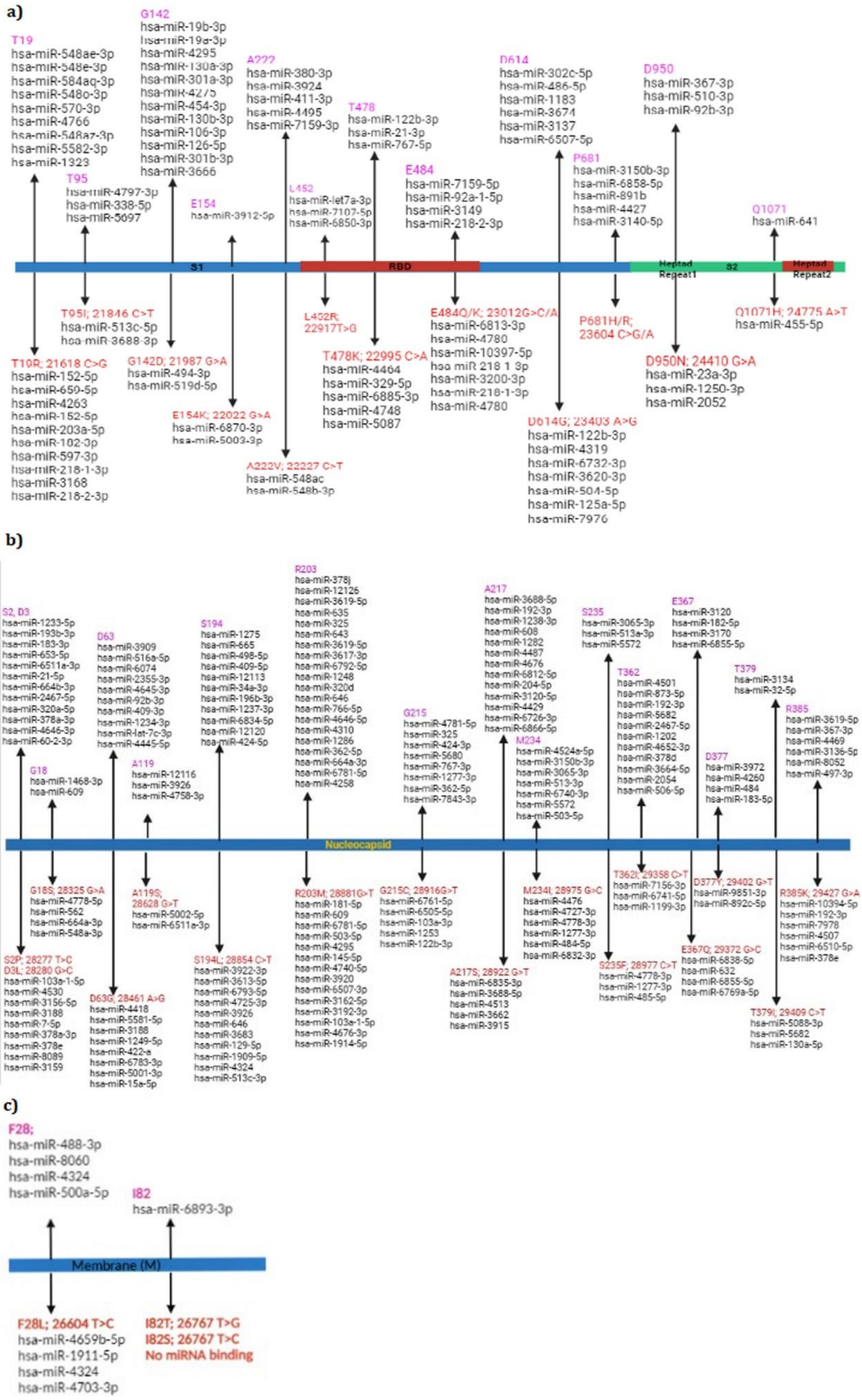

Table S1: Important events regarding SARS-CoV-2 infection worldwide with special reference to India.

S No	Date	Events	References
1	**December 24, 2019**	First case in the World- Wuhan, China	DOI: 10.1056/NEJMoa2001017
2	**January 10, 2020**	First genome sequence of SARS-CoV-2 publicly available	DOI: 10.1128/MRA.00169-20
3	**January 27, 2020**	First case in India - Kerala: International traveller link to Wuhan, China	DOI: 10.4103/ijmr.IJMR_2131_20
4	**March 3, 2020**	First case in Maharashtra, India : International traveller link to Dubai.	https://www.covid19maharashtragov.in/mh-covid/dashboard, https://www.livemint.com/news/india/coronavirus-update-first-2-patients-confirmed-in-mumbai-68-cases-in-india-now-11583977689230.html
5	**March 2, 2020**	First case in Delhi, India : International traveller link to Italy.	https://timesofindia.indiatimes.com/life-style/health-fitness/health-news/first-case-of-coronavirus-confirmed-in-delhi-here-are-the-details/articleshow/74439313.cms
6	**March 6, 2020**	First complete genome sequence from India (GISAID accession id: EPI_ISL_413522 and EPI_ISL_413523)	DOI: 10.4103/ijmr.IJMR_663_20
7	**March 8, 2020**	First case in Karnataka, India : International traveller link to USA.	https://www.thehindu.com/news/national/karnataka/coronavirus-karnataka-confirms-first-positive-covid-19-case/article31025099.ece
8	**March 11, 2020**	WHO declare Covid-19 pandemic	https://www.who.int/director-general/speeches/detail/who-director-general-s-opening-remarks-at-the-media-briefing-on-covid-19---11-march-2020
9	**March 17, 2020**	First case in West Bengal, India : International traveller link to UK.	https://www.thehindu.com/news/national/other-states/coronavirus-west-bengal-reports-first-case/article31093265.ece
10	**March 25, 2020**	Wave 1 Lock Down start in India	https://covid19.india.gov.in/document/mha-order-covid-19/ , https://cdnbbsr.s3waas.gov.in/s3850af92f8d9903e7a4e0559a98ecc857/uploads/2020/05/2020051363.pdf
11	**April 27, 2020**	First Complete Genome Sequence from West Bengal submitted in GISAID	Accession id: EPI_ISL_430468
12	**June 1, 2020**	Wave 1 Lock down relaxations from India.	https://www.ncbi.nlm.nih.gov/pmc/articles/PMC7785398/
13	**January 16 2021**	First vaccination start in India	https://www.thehindu.com/news/national/india-to-start-covid-19-vaccination-drive-on-jan-16/article33536670.ece, https://www.unicef.org/india/stories/covid-19-vaccine-launch-india, https://www.nature.com/articles/s41541-021-00327-2
14	**May 16, 2021**	Second wave lockdown in West Bengal	https://indianexpress.com/article/india/west-bengal-lockdown-guidelines-may-30-7316056/
15	**June 16, 2021**	Lockdown relaxation in West Bengal	https://epaper.telegraphindia.com/index.php?pagedate=2021-6-15&edcode=71&subcode=79&mod=1&pgnum=1&type=a

Table S2: Daily new infections (cases) and 7-Day rolling average of West Bengal, Karnataka, Delhi and Maharashtra

West Bengal			Karnataka			Delhi			Maharashtra		
**Date**	**New cases**	**7-day Rolling average**	**Date**	**New cases**	**7-day Rolling average**	**Date**	**New cases**	**7-day Rolling average**	**Date**	**New cases**	**7-day Rolling average**
01-01-2020	0		01-01-2020	0		01-01-2020	0		01-01-2020	0	
02-01-2020	0		02-01-2020	0		02-01-2020	0		02-01-2020	0	
03-01-2020	0		03-01-2020	0		03-01-2020	0		03-01-2020	0	
04-01-2020	0		04-01-2020	0		04-01-2020	0		04-01-2020	0	
05-01-2020	0		05-01-2020	0		05-01-2020	0		05-01-2020	0	
06-01-2020	0		06-01-2020	0		06-01-2020	0		06-01-2020	0	
07-01-2020	0	0	07-01-2020	0	0	07-01-2020	0	0	07-01-2020	0	0
08-01-2020	0	0	08-01-2020	0	0	08-01-2020	0	0	08-01-2020	0	0
09-01-2020	0	0	09-01-2020	0	0	09-01-2020	0	0	09-01-2020	0	0
10-01-2020	0	0	10-01-2020	0	0	10-01-2020	0	0	10-01-2020	0	0
11-01-2020	0	0	11-01-2020	0	0	11-01-2020	0	0	11-01-2020	0	0
12-01-2020	0	0	12-01-2020	0	0	12-01-2020	0	0	12-01-2020	0	0
13-01-2020	0	0	13-01-2020	0	0	13-01-2020	0	0	13-01-2020	0	0
14-01-2020	0	0	14-01-2020	0	0	14-01-2020	0	0	14-01-2020	0	0
15-01-2020	0	0	15-01-2020	0	0	15-01-2020	0	0	15-01-2020	0	0
16-01-2020	0	0	16-01-2020	0	0	16-01-2020	0	0	16-01-2020	0	0
17-01-2020	0	0	17-01-2020	0	0	17-01-2020	0	0	17-01-2020	0	0
18-01-2020	0	0	18-01-2020	0	0	18-01-2020	0	0	18-01-2020	0	0
19-01-2020	0	0	19-01-2020	0	0	19-01-2020	0	0	19-01-2020	0	0
20-01-2020	0	0	20-01-2020	0	0	20-01-2020	0	0	20-01-2020	0	0
21-01-2020	0	0	21-01-2020	0	0	21-01-2020	0	0	21-01-2020	0	0
22-01-2020	0	0	22-01-2020	0	0	22-01-2020	0	0	22-01-2020	0	0
23-01-2020	0	0	23-01-2020	0	0	23-01-2020	0	0	23-01-2020	0	0
24-01-2020	0	0	24-01-2020	0	0	24-01-2020	0	0	24-01-2020	0	0
25-01-2020	0	0	25-01-2020	0	0	25-01-2020	0	0	25-01-2020	0	0
26-01-2020	0	0	26-01-2020	0	0	26-01-2020	0	0	26-01-2020	0	0
27-01-2020	0	0	27-01-2020	0	0	27-01-2020	0	0	27-01-2020	0	0
28-01-2020	0	0	28-01-2020	0	0	28-01-2020	0	0	28-01-2020	0	0
29-01-2020	0	0	29-01-2020	0	0	29-01-2020	0	0	29-01-2020	0	0
30-01-2020	0	0	30-01-2020	0	0	30-01-2020	0	0	30-01-2020	0	0
31-01-2020	0	0	31-01-2020	0	0	31-01-2020	0	0	31-01-2020	0	0
01-02-2020	0	0	01-02-2020	0	0	01-02-2020	0	0	01-02-2020	0	0
02-02-2020	0	0	02-02-2020	0	0	02-02-2020	0	0	02-02-2020	0	0
03-02-2020	0	0	03-02-2020	0	0	03-02-2020	0	0	03-02-2020	0	0
04-02-2020	0	0	04-02-2020	0	0	04-02-2020	0	0	04-02-2020	0	0
05-02-2020	0	0	05-02-2020	0	0	05-02-2020	0	0	05-02-2020	0	0
06-02-2020	0	0	06-02-2020	0	0	06-02-2020	0	0	06-02-2020	0	0
07-02-2020	0	0	07-02-2020	0	0	07-02-2020	0	0	07-02-2020	0	0
08-02-2020	0	0	08-02-2020	0	0	08-02-2020	0	0	08-02-2020	0	0
09-02-2020	0	0	09-02-2020	0	0	09-02-2020	0	0	09-02-2020	0	0
10-02-2020	0	0	10-02-2020	0	0	10-02-2020	0	0	10-02-2020	0	0
11-02-2020	0	0	11-02-2020	0	0	11-02-2020	0	0	11-02-2020	0	0
12-02-2020	0	0	12-02-2020	0	0	12-02-2020	0	0	12-02-2020	0	0
13-02-2020	0	0	13-02-2020	0	0	13-02-2020	0	0	13-02-2020	0	0
14-02-2020	0	0	14-02-2020	0	0	14-02-2020	0	0	14-02-2020	0	0
15-02-2020	0	0	15-02-2020	0	0	15-02-2020	0	0	15-02-2020	0	0
16-02-2020	0	0	16-02-2020	0	0	16-02-2020	0	0	16-02-2020	0	0
17-02-2020	0	0	17-02-2020	0	0	17-02-2020	0	0	17-02-2020	0	0
18-02-2020	0	0	18-02-2020	0	0	18-02-2020	0	0	18-02-2020	0	0
19-02-2020	0	0	19-02-2020	0	0	19-02-2020	0	0	19-02-2020	0	0
20-02-2020	0	0	20-02-2020	0	0	20-02-2020	0	0	20-02-2020	0	0
21-02-2020	0	0	21-02-2020	0	0	21-02-2020	0	0	21-02-2020	0	0
22-02-2020	0	0	22-02-2020	0	0	22-02-2020	0	0	22-02-2020	0	0
23-02-2020	0	0	23-02-2020	0	0	23-02-2020	0	0	23-02-2020	0	0
24-02-2020	0	0	24-02-2020	0	0	24-02-2020	0	0	24-02-2020	0	0

West Bengal			Karnataka			Delhi			Maharashtra		
Date	New cases	7-day Rolling average	Date	New cases	7-day Rolling average	Date	New cases	7-day Rolling average	Date	New cases	7-day Rolling average
25-02-2020	0	0	25-02-2020	0	0	25-02-2020	0	0	25-02-2020	0	0
26-02-2020	0	0	26-02-2020	0	0	26-02-2020	0	0	26-02-2020	0	0
27-02-2020	0	0	27-02-2020	0	0	27-02-2020	0	0	27-02-2020	0	0
28-02-2020	0	0	28-02-2020	0	0	28-02-2020	0	0	28-02-2020	0	0
29-02-2020	0	0	29-02-2020	0	0	29-02-2020	0	0	29-02-2020	0	0
01-03-2020	0	0	01-03-2020	0	0	01-03-2020	0	0	01-03-2020	0	0
02-03-2020	0	0	02-03-2020	0	0	02-03-2020	1	0.14	02-03-2020	0	0
03-03-2020	0	0	03-03-2020	0	0	03-03-2020	0	0.14	03-03-2020	0	0
04-03-2020	0	0	04-03-2020	0	0	04-03-2020	0	0.14	04-03-2020	0	0
05-03-2020	0	0	05-03-2020	0	0	05-03-2020	0	0.14	05-03-2020	0	0
06-03-2020	0	0	06-03-2020	0	0	06-03-2020	0	0.14	06-03-2020	0	0
07-03-2020	0	0	07-03-2020	0	0	07-03-2020	0	0.14	07-03-2020	0	0
08-03-2020	0	0	08-03-2020	1	0.14	08-03-2020	0	0.14	08-03-2020	0	0
09-03-2020	0	0	09-03-2020	0	0.14	09-03-2020	0	0	09-03-2020	1	0.14
10-03-2020	0	0	10-03-2020	0	0.14	10-03-2020	0	0	10-03-2020	0	0.14
11-03-2020	0	0	11-03-2020	0	0.14	11-03-2020	0	0	11-03-2020	0	0.14
12-03-2020	0	0	12-03-2020	0	0.14	12-03-2020	0	0	12-03-2020	0	0.14
13-03-2020	0	0	13-03-2020	0	0.14	13-03-2020	0	0	13-03-2020	0	0.14
14-03-2020	0	0	14-03-2020	6	1	14-03-2020	81	11.57	14-03-2020	14	2.14
15-03-2020	0	0	15-03-2020	0	0.86	15-03-2020	27	15.43	15-03-2020	18	4.71
16-03-2020	0	0	16-03-2020	1	1	16-03-2020	15	17.57	16-03-2020	6	5.43
17-03-2020	1	0.14	17-03-2020	2	1.29	17-03-2020	11	19.14	17-03-2020	3	5.86
18-03-2020	0	0.14	18-03-2020	5	2	18-03-2020	37	24.43	18-03-2020	3	6.29
19-03-2020	0	0.14	19-03-2020	1	2.14	19-03-2020	27	28.29	19-03-2020	4	6.86
20-03-2020	1	0.29	20-03-2020	0	2.14	20-03-2020	58	36.57	20-03-2020	4	7.43
21-03-2020	1	0.43	21-03-2020	5	2	21-03-2020	78	36.14	21-03-2020	12	7.14
22-03-2020	1	0.57	22-03-2020	6	2.86	22-03-2020	67	41.86	22-03-2020	10	6
23-03-2020	3	1	23-03-2020	7	3.71	23-03-2020	102	54.29	23-03-2020	23	8.43
24-03-2020	2	1.14	24-03-2020	8	4.57	24-03-2020	64	61.86	24-03-2020	10	9.43
25-03-2020	0	1.14	25-03-2020	10	5.29	25-03-2020	90	69.43	25-03-2020	15	11.14
26-03-2020	1	1.29	26-03-2020	4	5.71	26-03-2020	65	74.86	26-03-2020	3	11
27-03-2020	5	1.86	27-03-2020	9	7	27-03-2020	164	90	27-03-2020	31	14.86
28-03-2020	2	2	28-03-2020	17	8.71	28-03-2020	143	99.29	28-03-2020	30	17.43
29-03-2020	3	2.29	29-03-2020	2	8.14	29-03-2020	110	105.43	29-03-2020	17	18.43
30-03-2020	2	2.14	30-03-2020	5	7.86	30-03-2020	187	117.57	30-03-2020	17	17.57
31-03-2020	5	2.57	31-03-2020	13	8.57	31-03-2020	309	152.57	31-03-2020	82	27.86
01-04-2020	10	4	01-04-2020	9	8.43	01-04-2020	424	200.29	01-04-2020	33	30.43
02-04-2020	0	3.86	02-04-2020	14	9.86	02-04-2020	486	260.43	02-04-2020	88	42.57
03-04-2020	0	3.14	03-04-2020	4	9.14	03-04-2020	560	317	03-04-2020	64	47.29
04-04-2020	12	4.57	04-04-2020	16	9	04-04-2020	579	379.29	04-04-2020	148	64.14
05-04-2020	0	4.14	05-04-2020	7	9.71	05-04-2020	609	450.57	05-04-2020	112	77.71
06-04-2020	12	5.57	06-04-2020	12	10.71	06-04-2020	484	493	06-04-2020	121	92.57
07-04-2020	8	6	07-04-2020	12	10.57	07-04-2020	572	530.57	07-04-2020	150	102.29
08-04-2020	2	4.86	08-04-2020	6	10.14	08-04-2020	565	550.71	08-04-2020	117	114.29
09-04-2020	9	6.14	09-04-2020	16	10.43	09-04-2020	813	597.43	09-04-2020	229	134.43
10-04-2020	9	7.43	10-04-2020	10	11.29	10-04-2020	871	641.86	10-04-2020	210	155.29
11-04-2020	6	6.57	11-04-2020	8	10.14	11-04-2020	854	681.14	11-04-2020	187	160.86
12-04-2020	0	6.57	12-04-2020	17	11.57	12-04-2020	758	702.43	12-04-2020	221	176.43
13-04-2020	15	7	13-04-2020	15	12	13-04-2020	1243	810.86	13-04-2020	352	209.43
14-04-2020	10	7.29	14-04-2020	13	12.14	14-04-2020	1031	876.43	14-04-2020	346	237.43
15-04-2020	12	8.71	15-04-2020	19	14	15-04-2020	886	922.29	15-04-2020	236	254.43
16-04-2020	12	9.14	16-04-2020	36	16.86	16-04-2020	1061	957.71	16-04-2020	285	262.43
17-04-2020	18	10.43	17-04-2020	44	21.71	17-04-2020	922	965	17-04-2020	120	249.57
18-04-2020	18	12.14	18-04-2020	25	24.14	18-04-2020	1371	1038.86	18-04-2020	327	269.57
19-04-2020	20	15	19-04-2020	6	22.57	19-04-2020	1580	1156.29	19-04-2020	552	316.86
20-04-2020	45	19.29	20-04-2020	18	23	20-04-2020	1239	1155.71	20-04-2020	466	333.14

West Bengal			Karnataka			Delhi			Maharashtra		
Date	New cases	7-day Rolling average	Date	New cases	7-day Rolling average	Date	New cases	7-day Rolling average	Date	New cases	7-day Rolling average
21-04-2020	29	22	21-04-2020	10	22.57	21-04-2020	1537	1228	21-04-2020	552	362.57
22-04-2020	26	24	22-04-2020	9	21.14	22-04-2020	1292	1286	22-04-2020	431	390.43
23-04-2020	34	27.14	23-04-2020	18	18.57	23-04-2020	1667	1372.57	23-04-2020	778	460.86
24-04-2020	51	31.86	24-04-2020	29	16.43	24-04-2020	1408	1442	24-04-2020	390	499.43
25-04-2020	38	34.71	25-04-2020	26	16.57	25-04-2020	1835	1508.29	25-04-2020	811	568.57
26-04-2020	38	37.29	26-04-2020	3	16.14	26-04-2020	1607	1512.14	26-04-2020	440	552.57
27-04-2020	43	37	27-04-2020	9	14.86	27-04-2020	1568	1559.14	27-04-2020	522	560.57
28-04-2020	18	35.43	28-04-2020	11	15	28-04-2020	1902	1611.29	28-04-2020	728	585.71
29-04-2020	28	35.71	29-04-2020	12	15.43	29-04-2020	1705	1670.29	29-04-2020	597	609.43
30-04-2020	22	34	30-04-2020	30	17.14	30-04-2020	1802	1689.57	30-04-2020	583	581.57
01-05-2020	57	34.86	01-05-2020	24	16.43	01-05-2020	2396	1830.71	01-05-2020	1008	669.86
02-05-2020	70	39.43	02-05-2020	12	14.43	02-05-2020	2564	1934.86	02-05-2020	790	666.86
03-05-2020	41	39.86	03-05-2020	13	15.86	03-05-2020	2952	2127	03-05-2020	678	700.86
04-05-2020	61	42.43	04-05-2020	37	19.86	04-05-2020	3656	2425.29	04-05-2020	1567	850.14
05-05-2020	84	51.86	05-05-2020	22	21.43	05-05-2020	2971	2578	05-05-2020	984	886.71
06-05-2020	112	63.86	06-05-2020	20	22.57	06-05-2020	3602	2849	06-05-2020	1233	977.57
07-05-2020	89	73.43	07-05-2020	12	20	07-05-2020	3344	3069.29	07-05-2020	1216	1068
08-05-2020	130	83.86	08-05-2020	48	23.43	08-05-2020	3339	3204	08-05-2020	1089	1079.57
09-05-2020	107	89.14	09-05-2020	41	27.57	09-05-2020	3175	3291.29	09-05-2020	1165	1133.14
10-05-2020	153	105.14	10-05-2020	54	33.43	10-05-2020	4311	3485.43	10-05-2020	1943	1313.86
11-05-2020	122	113.86	11-05-2020	14	30.14	11-05-2020	3592	3476.29	11-05-2020	1230	1265.71
12-05-2020	111	117.71	12-05-2020	63	36	12-05-2020	3562	3560.71	12-05-2020	1026	1271.71
13-05-2020	117	118.43	13-05-2020	34	38	13-05-2020	3726	3578.43	13-05-2020	1495	1309.14
14-05-2020	86	118	14-05-2020	28	40.29	14-05-2020	3991	3670.86	14-05-2020	1602	1364.29
15-05-2020	84	111.43	15-05-2020	69	43.29	15-05-2020	3808	3737.86	15-05-2020	1576	1433.86
16-05-2020	113	112.29	16-05-2020	36	42.57	16-05-2020	4794	3969.14	16-05-2020	1606	1496.86
17-05-2020	100	104.71	17-05-2020	55	42.71	17-05-2020	5049	4074.57	17-05-2020	2347	1554.57
18-05-2020	148	108.43	18-05-2020	99	54.86	18-05-2020	4628	4222.57	18-05-2020	2005	1665.29
19-05-2020	134	111.71	19-05-2020	149	67.14	19-05-2020	6154	4592.86	19-05-2020	2078	1815.57
20-05-2020	139	114.86	20-05-2020	67	71.86	20-05-2020	5720	4877.71	20-05-2020	2161	1910.71
21-05-2020	94	116	21-05-2020	143	88.29	21-05-2020	6023	5168	21-05-2020	2345	2016.86
22-05-2020	135	123.29	22-05-2020	138	98.14	22-05-2020	6536	5557.71	22-05-2020	2940	2211.71
23-05-2020	125	125	23-05-2020	216	123.86	23-05-2020	6663	5824.71	23-05-2020	2608	2354.86
24-05-2020	204	139.86	24-05-2020	130	134.57	24-05-2020	7111	6119.29	24-05-2020	3041	2454
25-05-2020	144	139.29	25-05-2020	93	133.71	25-05-2020	6414	6374.43	25-05-2020	2436	2515.57
26-05-2020	186	146.71	26-05-2020	101	126.86	26-05-2020	5907	6339.14	26-05-2020	2091	2517.43
27-05-2020	181	152.71	27-05-2020	135	136.57	27-05-2020	7246	6557.14	27-05-2020	2190	2521.57
28-05-2020	342	188.14	28-05-2020	115	132.57	28-05-2020	7254	6733	28-05-2020	2598	2557.71
29-05-2020	271	207.57	29-05-2020	248	148.29	29-05-2020	8138	6961.86	29-05-2020	2682	2520.86
30-05-2020	314	234.57	30-05-2020	141	137.57	30-05-2020	8364	7204.86	30-05-2020	2940	2568.29
31-05-2020	370	258.29	31-05-2020	299	161.71	31-05-2020	8789	7444.57	31-05-2020	2487	2489.14
01-06-2020	270	276.29	01-06-2020	187	175.14	01-06-2020	7723	7631.57	01-06-2020	2358	2478
02-06-2020	396	306.29	02-06-2020	388	216.14	02-06-2020	8812	8046.57	02-06-2020	2287	2506
03-06-2020	339	328.86	03-06-2020	267	235	03-06-2020	9689	8395.57	03-06-2020	2560	2558.86
04-06-2020	368	332.57	04-06-2020	257	255.29	04-06-2020	9847	8766	04-06-2020	2933	2606.71
05-06-2020	426	354.71	05-06-2020	515	293.43	05-06-2020	9472	8956.57	05-06-2020	2436	2571.57
06-06-2020	432	371.57	06-06-2020	378	327.29	06-06-2020	10408	9248.57	06-06-2020	2739	2542.86
07-06-2020	449	382.86	07-06-2020	239	318.71	07-06-2020	10882	9547.57	07-06-2020	3007	2617.14
08-06-2020	426	405.14	08-06-2020	308	336	08-06-2020	8536	9663.71	08-06-2020	2554	2645.14
09-06-2020	372	401.71	09-06-2020	161	303.57	09-06-2020	9981	9830.71	09-06-2020	2258	2641
10-06-2020	343	402.29	10-06-2020	120	282.57	10-06-2020	11156	10040.29	10-06-2020	3254	2740.14
11-06-2020	440	412.57	11-06-2020	204	275	11-06-2020	11135	10224.29	11-06-2020	3607	2836.43
12-06-2020	476	419.71	12-06-2020	271	240.14	12-06-2020	11306	10486.29	12-06-2020	3493	2987.43
13-06-2020	454	422.86	13-06-2020	308	230.14	13-06-2020	12039	10719.29	13-06-2020	3427	3085.71
14-06-2020	389	414.29	14-06-2020	176	221.14	14-06-2020	11405	10794	14-06-2020	3390	3140.43
15-06-2020	407	411.57	15-06-2020	213	207.57	15-06-2020	10032	11007.71	15-06-2020	2786	3173.57

West Bengal			Karnataka			Delhi			Maharashtra		
Date	New cases	7-day Rolling average	Date	New cases	7-day Rolling average	Date	New cases	7-day Rolling average	Date	New cases	7-day Rolling average
16-06-2020	415	417.71	16-06-2020	317	229.86	16-06-2020	11086	11165.57	16-06-2020	2701	3236.86
17-06-2020	391	424.57	17-06-2020	204	241.86	17-06-2020	13108	11444.43	17-06-2020	3307	3244.43
18-06-2020	435	423.86	18-06-2020	210	242.71	18-06-2020	13829	11829.29	18-06-2020	3752	3265.14
19-06-2020	355	406.57	19-06-2020	337	252.14	19-06-2020	14638	12305.29	19-06-2020	3827	3312.86
20-06-2020	441	404.71	20-06-2020	416	267.57	20-06-2020	15918	12859.43	20-06-2020	3874	3376.71
21-06-2020	414	408.29	21-06-2020	453	307.14	21-06-2020	15151	13394.57	21-06-2020	3870	3445.29
22-06-2020	413	409.14	22-06-2020	249	312.29	22-06-2020	13560	13898.57	22-06-2020	3721	3578.86
23-06-2020	370	402.71	23-06-2020	322	313	23-06-2020	15656	14551.43	23-06-2020	3214	3652.14
24-06-2020	445	410.43	24-06-2020	397	340.57	24-06-2020	16868	15088.57	24-06-2020	3889	3735.29
25-06-2020	475	416.14	25-06-2020	442	373.71	25-06-2020	18205	15713.71	25-06-2020	4842	3891
26-06-2020	542	442.86	26-06-2020	445	389.14	26-06-2020	18255	16230.43	26-06-2020	5024	4062
27-06-2020	521	454.29	27-06-2020	918	460.86	27-06-2020	20142	16833.86	27-06-2020	6368	4418.29
28-06-2020	572	476.86	28-06-2020	1267	577.14	28-06-2020	19610	17470.86	28-06-2020	5493	4650.14
29-06-2020	624	507	29-06-2020	1105	699.43	29-06-2020	18339	18153.57	29-06-2020	5257	4869.57
30-06-2020	652	547.29	30-06-2020	947	788.71	30-06-2020	18256	18525	30-06-2020	4878	5107.29
01-07-2020	611	571	01-07-2020	1272	913.71	01-07-2020	19429	18890.86	01-07-2020	5537	5342.71
02-07-2020	649	595.86	02-07-2020	1502	1065.14	02-07-2020	21947	19425.43	02-07-2020	6328	5555
03-07-2020	669	614	03-07-2020	1694	1243.57	03-07-2020	22718	20063	03-07-2020	6364	5746.43
04-07-2020	743	645.71	04-07-2020	1839	1375.14	04-07-2020	24018	20616.71	04-07-2020	7074	5847.29
05-07-2020	895	691.86	05-07-2020	1925	1469.14	05-07-2020	23942	21235.57	05-07-2020	6555	5999
06-07-2020	861	725.71	06-07-2020	1843	1574.57	06-07-2020	22500	21830	06-07-2020	5368	6014.86
07-07-2020	850	754	07-07-2020	1498	1653.29	07-07-2020	23147	22528.71	07-07-2020	5134	6051.43
08-07-2020	986	807.57	08-07-2020	2062	1766.14	08-07-2020	25561	23404.71	08-07-2020	6603	6203.71
09-07-2020	1088	870.29	09-07-2020	2228	1869.86	09-07-2020	25790	23953.71	09-07-2020	6875	6281.86
10-07-2020	1198	945.86	10-07-2020	2313	1958.29	10-07-2020	27762	24674.29	10-07-2020	7862	6495.86
11-07-2020	1344	1031.71	11-07-2020	2798	2095.29	11-07-2020	27757	25208.43	11-07-2020	8139	6648
12-07-2020	1560	1126.71	12-07-2020	2627	2195.57	12-07-2020	29106	25946.14	12-07-2020	7827	6829.71
13-07-2020	1435	1208.71	13-07-2020	2738	2323.43	13-07-2020	28178	26757.29	13-07-2020	6497	6991
14-07-2020	1390	1285.86	14-07-2020	2496	2466	14-07-2020	29917	27724.43	14-07-2020	6741	7220.57
15-07-2020	1589	1372	15-07-2020	3176	2625.14	15-07-2020	32607	28731	15-07-2020	7975	7416.57
16-07-2020	1690	1458	16-07-2020	4169	2902.43	16-07-2020	35468	30113.57	16-07-2020	8641	7668.86
17-07-2020	1894	1557.43	17-07-2020	3693	3099.57	17-07-2020	34820	31121.86	17-07-2020	8308	7732.57
18-07-2020	2198	1679.43	18-07-2020	4537	3348	18-07-2020	37411	32501	18-07-2020	8348	7762.43
19-07-2020	2278	1782	19-07-2020	4120	3561.29	19-07-2020	40235	34090.86	19-07-2020	9518	8004
20-07-2020	2282	1903	20-07-2020	3648	3691.29	20-07-2020	36806	35323.43	20-07-2020	8240	8253
21-07-2020	2261	2027.43	21-07-2020	3649	3856	21-07-2020	39170	36645.29	21-07-2020	8336	8480.86
22-07-2020	2291	2127.71	22-07-2020	4764	4082.86	22-07-2020	45601	38501.57	22-07-2020	10576	8852.43
23-07-2020	2436	2234.29	23-07-2020	5030	4205.86	23-07-2020	48443	40355.14	23-07-2020	9895	9031.57
24-07-2020	2216	2280.29	24-07-2020	5007	4393.57	24-07-2020	48888	42364.86	24-07-2020	9615	9218.29
25-07-2020	2404	2309.71	25-07-2020	5072	4470	25-07-2020	50072	44173.57	25-07-2020	9251	9347.29
26-07-2020	2341	2318.71	26-07-2020	5199	4624.14	26-07-2020	48932	45416	26-07-2020	9431	9334.86
27-07-2020	2112	2294.43	27-07-2020	5324	4863.57	27-07-2020	46484	46798.57	27-07-2020	7924	9289.71
28-07-2020	2134	2276.29	28-07-2020	5536	5133.14	28-07-2020	49631	48293	28-07-2020	7717	9201.29
29-07-2020	2294	2276.71	29-07-2020	5503	5238.71	29-07-2020	52479	49275.57	29-07-2020	9211	9006.29
30-07-2020	2434	2276.43	30-07-2020	6128	5395.57	30-07-2020	54968	50207.71	30-07-2020	11147	9185.14
31-07-2020	2496	2316.43	31-07-2020	5483	5463.57	31-07-2020	57486	51436	31-07-2020	10320	9285.86
01-08-2020	2589	2342.86	01-08-2020	5172	5477.86	01-08-2020	55117	52156.71	01-08-2020	9601	9335.86
02-08-2020	2739	2399.71	02-08-2020	5532	5525.43	02-08-2020	52672	52691	02-08-2020	9509	9347
03-08-2020	2716	2486	03-08-2020	4752	5443.71	03-08-2020	50488	53263	03-08-2020	8968	9496.14
04-08-2020	2752	2574.29	04-08-2020	6259	5547	04-08-2020	51282	53498.86	04-08-2020	7760	9502.29
05-08-2020	2816	2648.86	05-08-2020	5619	5563.57	05-08-2020	56626	54091.29	05-08-2020	10309	9659.14
06-08-2020	2954	2723.14	06-08-2020	6805	5660.29	06-08-2020	62170	55120.14	06-08-2020	11514	9711.57
07-08-2020	2912	2782.57	07-08-2020	6670	5829.86	07-08-2020	61455	55687.14	07-08-2020	10483	9734.86
08-08-2020	2949	2834	08-08-2020	7178	6116.43	08-08-2020	65156	57121.29	08-08-2020	12822	10195
09-08-2020	2939	2862.57	09-08-2020	5985	6181.14	09-08-2020	62117	58470.57	09-08-2020	12248	10586.29
10-08-2020	2905	2889.57	10-08-2020	4267	6111.86	10-08-2020	53016	58831.71	10-08-2020	9181	10616.71

West Bengal			Karnataka			Delhi			Maharashtra		
Date	New cases	7-day Rolling average	Date	New cases	7-day Rolling average	Date	New cases	7-day Rolling average	Date	New cases	7-day Rolling average
11-08-2020	2931	2915.14	11-08-2020	6257	6111.57	11-08-2020	61252	60256	11-08-2020	11088	11092.14
12-08-2020	2936	2932.29	12-08-2020	7883	6435	12-08-2020	67066	61747.43	12-08-2020	12712	11435.43
13-08-2020	2997	2938.43	13-08-2020	6706	6420.86	13-08-2020	64141	62029	13-08-2020	11813	11478.14
14-08-2020	3035	2956	14-08-2020	7908	6597.71	14-08-2020	65610	62622.57	14-08-2020	12608	11781.71
15-08-2020	3074	2973.86	15-08-2020	8818	6832	15-08-2020	63986	62455.43	15-08-2020	12020	11667.14
16-08-2020	3066	2992	16-08-2020	7040	6982.71	16-08-2020	58096	61881	16-08-2020	11111	11504.71
17-08-2020	3080	3017	17-08-2020	6317	7275.57	17-08-2020	54298	62064.14	17-08-2020	8493	11406.43
18-08-2020	3175	3051.86	18-08-2020	7665	7476.71	18-08-2020	64999	62599.43	18-08-2020	11119	11410.86
19-08-2020	3169	3085.14	19-08-2020	8642	7585.14	19-08-2020	69196	62903.71	19-08-2020	13165	11475.57
20-08-2020	3197	3113.71	20-08-2020	7385	7682.14	20-08-2020	68518	63529	20-08-2020	14647	11880.43
21-08-2020	3245	3143.71	21-08-2020	7571	7634	21-08-2020	68519	63944.57	21-08-2020	14161	12102.29
22-08-2020	3232	3166.29	22-08-2020	7330	7421.43	22-08-2020	70067	64813.29	22-08-2020	14492	12455.43
23-08-2020	3274	3196	23-08-2020	5938	7264	23-08-2020	61749	65335.14	23-08-2020	10441	12359.71
24-08-2020	2967	3179.86	24-08-2020	5851	7197.43	24-08-2020	59696	66106.29	24-08-2020	11015	12720
25-08-2020	2964	3149.71	25-08-2020	8161	7268.29	25-08-2020	66873	66374	25-08-2020	10425	12620.86
26-08-2020	2974	3121.86	26-08-2020	8580	7259.43	26-08-2020	75995	67345.29	26-08-2020	14888	12867
27-08-2020	2997	3093.29	27-08-2020	9386	7545.29	27-08-2020	76827	68532.29	27-08-2020	14857	12897
28-08-2020	2982	3055.71	28-08-2020	8960	7743.71	28-08-2020	76657	69694.86	28-08-2020	14427	12935
29-08-2020	3012	3024.29	29-08-2020	8324	7885.71	29-08-2020	78479	70896.57	29-08-2020	16286	13191.29
30-08-2020	3019	2987.86	30-08-2020	8852	8302	30-08-2020	79461	73426.86	30-08-2020	16408	14043.71
31-08-2020	2993	2991.57	31-08-2020	6495	8394	31-08-2020	68766	74722.57	31-08-2020	11852	14163.29
01-09-2020	2943	2988.57	01-09-2020	9058	8522.14	01-09-2020	78168	76336.14	01-09-2020	15765	14926.14
02-09-2020	2976	2988.86	02-09-2020	9860	8705	02-09-2020	82860	77316.86	02-09-2020	17433	15289.71
03-09-2020	2984	2987	03-09-2020	8865	8630.57	03-09-2020	84156	78363.86	03-09-2020	18105	15753.71
04-09-2020	2978	2986.43	04-09-2020	9280	8676.29	04-09-2020	87115	79857.86	04-09-2020	19218	16438.14
05-09-2020	3042	2990.71	05-09-2020	9746	8879.43	05-09-2020	90600	81589.43	05-09-2020	20800	17083
06-09-2020	3087	3000.43	06-09-2020	9319	8946.14	06-09-2020	91725	83341.43	06-09-2020	23350	18074.71
07-09-2020	3077	3012.43	07-09-2020	5773	8843	07-09-2020	75015	84234.14	07-09-2020	16429	18728.57
08-09-2020	3091	3033.57	08-09-2020	7866	8672.71	08-09-2020	89855	85903.71	08-09-2020	20131	19352.29
09-09-2020	3107	3052.29	09-09-2020	9540	8627	09-09-2020	95536	87714.57	09-09-2020	23577	20230
10-09-2020	3112	3070.57	10-09-2020	9217	8677.29	10-09-2020	96762	89515.43	10-09-2020	23446	20993
11-09-2020	3157	3096.14	11-09-2020	9464	8703.57	11-09-2020	97655	91021.14	11-09-2020	24886	21802.71
12-09-2020	3161	3113.14	12-09-2020	9140	8617	12-09-2020	94414	91566	12-09-2020	22084	21986.14
13-09-2020	3215	3131.43	13-09-2020	9894	8699.14	13-09-2020	93220	91779.57	13-09-2020	22543	21870.86
14-09-2020	3211	3150.57	14-09-2020	8244	9052.14	14-09-2020	81913	92765	14-09-2020	17066	21961.86
15-09-2020	3227	3170	15-09-2020	7576	9010.71	15-09-2020	91098	92942.57	15-09-2020	20482	22012
16-09-2020	3237	3188.57	16-09-2020	9725	9037.14	16-09-2020	97860	93274.57	16-09-2020	23365	21981.71
17-09-2020	3197	3200.71	17-09-2020	9366	9058.43	17-09-2020	96787	93278.14	17-09-2020	24619	22149.29
18-09-2020	3192	3205.71	18-09-2020	8626	8938.71	18-09-2020	92973	92609.29	18-09-2020	21656	21687.86
19-09-2020	3188	3209.57	19-09-2020	8364	8827.86	19-09-2020	92574	92346.43	19-09-2020	20519	21464.29
20-09-2020	3177	3204.14	20-09-2020	8191	8584.57	20-09-2020	87395	91514.29	20-09-2020	20627	21190.57
21-09-2020	3165	3197.57	21-09-2020	7339	8455.29	21-09-2020	74493	90454.29	21-09-2020	15738	21000.86
22-09-2020	3182	3191.14	22-09-2020	6974	8369.29	22-09-2020	83362	89349.14	22-09-2020	18390	20702
23-09-2020	3189	3184.29	23-09-2020	6997	7979.57	23-09-2020	86703	87755.29	23-09-2020	21029	20368.29
24-09-2020	3196	3184.14	24-09-2020	7710	7743	24-09-2020	85921	86203	24-09-2020	19164	19589
25-09-2020	3190	3183.86	25-09-2020	8655	7747.14	25-09-2020	85717	85166.43	25-09-2020	17794	19037.29
26-09-2020	3181	3182.86	26-09-2020	8811	7811	26-09-2020	88759	84621.43	26-09-2020	20419	19023
27-09-2020	3185	3184	27-09-2020	9543	8004.14	27-09-2020	82770	83960.71	27-09-2020	18056	18655.71
28-09-2020	3155	3182.57	28-09-2020	6892	7940.29	28-09-2020	69669	83271.57	28-09-2020	11921	18110.43
29-09-2020	3188	3183.43	29-09-2020	10453	8437.29	29-09-2020	80500	82862.71	29-09-2020	14976	17622.71
30-09-2020	3281	3196.57	30-09-2020	8856	8702.86	30-09-2020	86748	82869.14	30-09-2020	18317	17235.29
01-10-2020	3275	3207.86	01-10-2020	10070	9040	01-10-2020	81784	82278.14	01-10-2020	16476	16851.29
02-10-2020	3310	3225	02-10-2020	8793	9059.71	02-10-2020	79883	81444.71	02-10-2020	15591	16536.57
03-10-2020	3340	3247.71	03-10-2020	9886	9213.29	03-10-2020	75479	79547.57	03-10-2020	14348	15669.29
04-10-2020	3357	3272.29	04-10-2020	10145	9299.29	04-10-2020	74770	78404.71	04-10-2020	12548	14882.43
05-10-2020	3348	3299.86	05-10-2020	7051	9322	05-10-2020	60130	77042	05-10-2020	10244	14642.86

West Bengal			Karnataka			Delhi			Maharashtra		
Date	New cases	7-day Rolling average	Date	New cases	7-day Rolling average	Date	New cases	7-day Rolling average	Date	New cases	7-day Rolling average
06-10-2020	3370	3325.86	06-10-2020	9993	9256.29	06-10-2020	71869	75809	06-10-2020	12258	14254.57
07-10-2020	3455	3350.71	07-10-2020	10947	9555	07-10-2020	78809	74674.86	07-10-2020	14578	13720.43
08-10-2020	3526	3386.57	08-10-2020	10704	9645.57	08-10-2020	70797	73105.29	08-10-2020	13395	13280.29
09-10-2020	3573	3424.14	09-10-2020	10913	9948.43	09-10-2020	73305	72165.57	09-10-2020	12134	12786.43
10-10-2020	3591	3460	10-10-2020	10517	10038.57	10-10-2020	74418	72014	10-10-2020	11416	12367.57
11-10-2020	3612	3496.43	11-10-2020	9523	9949.71	11-10-2020	67789	71016.71	11-10-2020	10792	12116.71
12-10-2020	3583	3530	12-10-2020	7606	10029	12-10-2020	54262	70178.43	12-10-2020	7089	11666
13-10-2020	3631	3567.29	13-10-2020	8191	9771.57	13-10-2020	63717	69013.86	13-10-2020	8522	11132.29
14-10-2020	3677	3599	14-10-2020	9265	9531.29	14-10-2020	67811	67442.71	14-10-2020	10552	10557.14
15-10-2020	3720	3626.71	15-10-2020	8477	9213.14	15-10-2020	63441	66391.86	15-10-2020	10226	10104.43
16-10-2020	3771	3655	16-10-2020	7542	8731.57	16-10-2020	62304	64820.29	16-10-2020	11447	10006.29
17-10-2020	3864	3694	17-10-2020	7184	8255.43	17-10-2020	61893	63031	17-10-2020	10259	9841
18-10-2020	3983	3747	18-10-2020	7012	7896.71	18-10-2020	56519	61421	18-10-2020	9060	9593.57
19-10-2020	3992	3805.43	19-10-2020	5018	7527	19-10-2020	45506	60170.14	19-10-2020	5984	9435.71
20-10-2020	4029	3862.29	20-10-2020	6297	7256.43	20-10-2020	54348	58831.71	20-10-2020	8151	9382.71
21-10-2020	4069	3918.29	21-10-2020	5872	6771.71	21-10-2020	56264	57182.14	21-10-2020	8142	9038.43
22-10-2020	4157	3980.71	22-10-2020	5778	6386.14	22-10-2020	54371	55886.43	22-10-2020	7539	8654.57
23-10-2020	4143	4033.86	23-10-2020	5356	6073.86	23-10-2020	53931	54690.29	23-10-2020	7347	8068.86
24-10-2020	4148	4074.43	24-10-2020	4471	5686.29	24-10-2020	50366	53043.57	24-10-2020	6417	7520
25-10-2020	4127	4095	25-10-2020	4439	5318.71	25-10-2020	45922	51529.71	25-10-2020	6059	7091.29
26-10-2020	4121	4113.43	26-10-2020	3130	5049	26-10-2020	36019	50174.43	26-10-2020	3645	6757.14
27-10-2020	3957	4103.14	27-10-2020	3691	4676.71	27-10-2020	43036	48558.43	27-10-2020	5363	6358.86
28-10-2020	3924	4082.43	28-10-2020	3146	4287.29	28-10-2020	50188	47690.43	28-10-2020	6738	6158.29
29-10-2020	3989	4058.43	29-10-2020	4025	4036.86	29-10-2020	48765	46889.57	29-10-2020	5902	5924.43
30-10-2020	3979	4035	30-10-2020	3589	3784.43	30-10-2020	48117	46059	30-10-2020	6190	5759.14
31-10-2020	3993	4012.86	31-10-2020	3014	3576.29	31-10-2020	47228	45610.71	31-10-2020	5548	5635
01-11-2020	3987	3992.86	01-11-2020	3652	3463.86	01-11-2020	45928	45611.57	01-11-2020	5369	5536.43
02-11-2020	3957	3969.43	02-11-2020	2576	3384.71	02-11-2020	37592	45836.29	02-11-2020	4009	5588.43
03-11-2020	3981	3972.86	03-11-2020	2756	3251.14	03-11-2020	46027	46263.57	03-11-2020	4909	5523.57
04-11-2020	3987	3981.86	04-11-2020	3377	3284.14	04-11-2020	50465	46303.14	04-11-2020	5505	5347.43
05-11-2020	3948	3976	05-11-2020	3156	3160	05-11-2020	47628	46140.71	05-11-2020	5246	5253.71
06-11-2020	3942	3970.71	06-11-2020	2960	3070.14	06-11-2020	50017	46412.14	06-11-2020	6870	5350.86
07-11-2020	3928	3961.43	07-11-2020	2258	2962.14	07-11-2020	46153	46258.57	07-11-2020	3959	5123.86
08-11-2020	3920	3951.86	08-11-2020	2740	2831.86	08-11-2020	46707	46369.86	08-11-2020	5585	5154.71
09-11-2020	3907	3944.71	09-11-2020	1963	2744.29	09-11-2020	37119	46302.29	09-11-2020	3277	5050.14
10-11-2020	3872	3929.14	10-11-2020	2362	2688	10-11-2020	44724	46116.14	10-11-2020	3791	4890.43
11-11-2020	3872	3912.71	11-11-2020	2584	2574.71	11-11-2020	48285	45804.71	11-11-2020	4907	4805
12-11-2020	3856	3899.57	12-11-2020	2116	2426.14	12-11-2020	44585	45370	12-11-2020	4496	4697.86
13-11-2020	3835	3884.29	13-11-2020	2016	2291.29	13-11-2020	44620	44599	13-11-2020	4132	4306.71
14-11-2020	3823	3869.29	14-11-2020	2154	2276.43	14-11-2020	41692	43961.71	14-11-2020	4237	4346.43
15-11-2020	3053	3745.43	15-11-2020	1565	2108.57	15-11-2020	30681	41672.29	15-11-2020	2544	3912
16-11-2020	3012	3617.57	16-11-2020	1157	1993.43	16-11-2020	28609	40456.57	16-11-2020	2535	3806
17-11-2020	3654	3586.43	17-11-2020	1336	1846.86	17-11-2020	38548	39574.29	17-11-2020	2732	3654.71
18-11-2020	3668	3557.29	18-11-2020	1791	1733.57	18-11-2020	45366	39157.29	18-11-2020	5011	3669.57
19-11-2020	3620	3523.57	19-11-2020	1849	1695.43	19-11-2020	46185	39385.86	19-11-2020	5535	3818
20-11-2020	3626	3493.71	20-11-2020	1781	1661.86	20-11-2020	46283	39623.43	20-11-2020	5640	4033.43
21-11-2020	3639	3467.43	21-11-2020	1781	1608.57	21-11-2020	45301	40139	21-11-2020	5760	4251
22-11-2020	3591	3544.29	22-11-2020	1704	1628.43	22-11-2020	44404	42099.43	22-11-2020	5753	4709.43
23-11-2020	3557	3622.14	23-11-2020	1509	1678.71	23-11-2020	37441	43361.14	23-11-2020	4153	4940.57
24-11-2020	3545	3606.57	24-11-2020	1870	1755	24-11-2020	44245	44175	24-11-2020	5439	5327.29
25-11-2020	3528	3586.57	25-11-2020	1630	1732	25-11-2020	44699	44079.71	25-11-2020	6159	5491.29
26-11-2020	3507	3570.43	26-11-2020	1505	1682.86	26-11-2020	43174	43649.57	26-11-2020	6406	5615.71
27-11-2020	3489	3550.86	27-11-2020	1526	1646.43	27-11-2020	41353	42945.29	27-11-2020	6185	5693.57
28-11-2020	3459	3525.14	28-11-2020	1522	1609.43	28-11-2020	41815	42447.29	28-11-2020	5965	5722.86
29-11-2020	3367	3493.14	29-11-2020	1291	1550.43	29-11-2020	39036	41680.43	29-11-2020	5544	5693
30-11-2020	2671	3366.57	30-11-2020	998	1477.43	30-11-2020	31179	40785.86	30-11-2020	3837	5647.86

West Bengal			Karnataka			Delhi			Maharashtra		
Date	New cases	7-day Rolling average	Date	New cases	7-day Rolling average	Date	New cases	7-day Rolling average	Date	New cases	7-day Rolling average
01-12-2020	3315	3333.71	01-12-2020	1330	1400.29	01-12-2020	36474	39675.71	01-12-2020	4930	5575.14
02-12-2020	3271	3297	02-12-2020	1440	1373.14	02-12-2020	35506	38362.43	02-12-2020	3350	5173.86
03-12-2020	3246	3259.71	03-12-2020	1446	1364.71	03-12-2020	36574	37419.57	03-12-2020	5182	4999
04-12-2020	3206	3219.29	04-12-2020	1247	1324.86	04-12-2020	36711	36756.43	04-12-2020	5229	4862.43
05-12-2020	3175	3178.71	05-12-2020	1325	1296.71	05-12-2020	36010	35927.14	05-12-2020	4922	4713.43
06-12-2020	3143	3146.71	06-12-2020	1321	1301	06-12-2020	33178	35090.29	06-12-2020	4757	4601
07-12-2020	2214	3081.43	07-12-2020	998	1301	07-12-2020	26227	34382.86	07-12-2020	3075	4492.14
08-12-2020	2941	3028	08-12-2020	1280	1293.86	08-12-2020	32083	33755.57	08-12-2020	4026	4363
09-12-2020	2956	2983	09-12-2020	1279	1270.86	09-12-2020	31637	33202.86	09-12-2020	4981	4596
10-12-2020	2811	2920.86	10-12-2020	1238	1241.14	10-12-2020	29411	32179.57	10-12-2020	3824	4402
11-12-2020	2753	2856.14	11-12-2020	1210	1235.86	11-12-2020	29961	31215.29	11-12-2020	4268	4264.71
12-12-2020	2710	2789.71	12-12-2020	1203	1218.43	12-12-2020	30354	30407.29	12-12-2020	4259	4170
13-12-2020	2580	2709.29	13-12-2020	1196	1200.57	13-12-2020	27336	29572.71	13-12-2020	3717	4021.43
14-12-2020	1834	2655	14-12-2020	830	1176.57	14-12-2020	21941	28960.43	14-12-2020	2949	4003.43
15-12-2020	2289	2561.86	15-12-2020	1185	1163	15-12-2020	26251	28127.29	15-12-2020	3442	3920
16-12-2020	2293	2467.14	16-12-2020	1240	1157.43	16-12-2020	18172	26203.71	16-12-2020	0	3208.43
17-12-2020	2245	2386.29	17-12-2020	1236	1157.14	17-12-2020	26754	25824.14	17-12-2020	3880	3216.43
18-12-2020	2239	2312.86	18-12-2020	1222	1158.86	18-12-2020	26991	25399.86	18-12-2020	3994	3177.29
19-12-2020	2155	2233.57	19-12-2020	1152	1151.57	19-12-2020	26834	24897	19-12-2020	3940	3131.71
20-12-2020	1978	2147.57	20-12-2020	1194	1151.29	20-12-2020	24622	24509.29	20-12-2020	3811	3145.14
21-12-2020	1515	2102	21-12-2020	772	1143	21-12-2020	19147	24110.14	21-12-2020	2834	3128.71
22-12-2020	1653	2011.14	22-12-2020	1141	1136.71	22-12-2020	23880	23771.43	22-12-2020	3106	3080.71
23-12-2020	1628	1916.14	23-12-2020	958	1096.43	23-12-2020	24716	24706.29	23-12-2020	3913	3639.71
24-12-2020	1590	1822.57	24-12-2020	1143	1083.14	24-12-2020	23444	24233.43	24-12-2020	3580	3596.86
25-12-2020	1541	1722.86	25-12-2020	1005	1052.14	25-12-2020	22349	23570.29	25-12-2020	3431	3516.43
26-12-2020	1253	1594	26-12-2020	857	1010	26-12-2020	18575	22390.43	26-12-2020	2854	3361.29
27-12-2020	1433	1516.14	27-12-2020	911	969.57	27-12-2020	20333	21777.71	27-12-2020	3314	3290.29
28-12-2020	1028	1446.57	28-12-2020	653	952.57	28-12-2020	16072	21338.43	28-12-2020	2498	3242.29
29-12-2020	1244	1388.14	29-12-2020	662	884.14	29-12-2020	20542	20861.57	29-12-2020	3018	3229.71
30-12-2020	1178	1323.86	30-12-2020	973	886.29	30-12-2020	21945	20465.71	30-12-2020	3537	3176
31-12-2020	1170	1263.86	31-12-2020	952	859	31-12-2020	19026	19834.57	31-12-2020	3509	3165.86
01-01-2021	1153	1208.43	01-01-2021	877	840.71	01-01-2021	20159	19521.71	01-01-2021	3524	3179.14
02-01-2021	863	1152.71	02-01-2021	755	826.14	02-01-2021	18144	19460.14	02-01-2021	3218	3231.14
03-01-2021	896	1076	03-01-2021	810	811.71	03-01-2021	16678	18938	03-01-2021	3282	3226.57
04-01-2021	597	1014.43	04-01-2021	600	804.14	04-01-2021	16278	18967.43	04-01-2021	4875	3566.14
05-01-2021	812	952.71	05-01-2021	815	826	05-01-2021	17909	18591.29	05-01-2021	3160	3586.43
06-01-2021	868	908.43	06-01-2021	784	799	06-01-2021	20472	18380.86	06-01-2021	4382	3707.14
07-01-2021	921	872.86	07-01-2021	761	771.71	07-01-2021	18123	18251.86	07-01-2021	3729	3738.57
08-01-2021	926	840.43	08-01-2021	970	785	08-01-2021	18453	18008.14	08-01-2021	3693	3762.71
09-01-2021	787	829.57	09-01-2021	899	805.57	09-01-2021	18820	18104.71	09-01-2021	3581	3814.57
10-01-2021	823	819.14	10-01-2021	792	803	10-01-2021	16086	18020.14	10-01-2021	3558	3854
11-01-2021	612	821.29	11-01-2021	496	788.14	11-01-2021	12481	17477.71	11-01-2021	2438	3505.86
12-01-2021	751	812.57	12-01-2021	751	779	12-01-2021	15903	17191.14	12-01-2021	2936	3473.86
13-01-2021	723	791.86	13-01-2021	746	773.57	13-01-2021	17015	16697.29	13-01-2021	3556	3355.86
14-01-2021	680	757.43	14-01-2021	408	723.14	14-01-2021	15677	16347.86	14-01-2021	3579	3334.43
15-01-2021	623	714.14	15-01-2021	708	685.71	15-01-2021	15155	15876.71	15-01-2021	3145	3256.14
16-01-2021	609	688.71	16-01-2021	584	640.71	16-01-2021	15050	15338.14	16-01-2021	2910	3160.29
17-01-2021	565	651.86	17-01-2021	745	634	17-01-2021	13962	15034.71	17-01-2021	3081	3092.14
18-01-2021	389	620	18-01-2021	435	625.29	18-01-2021	9987	14678.43	18-01-2021	1924	3018.71
19-01-2021	412	571.57	19-01-2021	645	610.14	19-01-2021	13787	14376.14	19-01-2021	2294	2927
20-01-2021	409	526.71	20-01-2021	501	575.14	20-01-2021	15279	14128.14	20-01-2021	3015	2849.71
21-01-2021	416	489	21-01-2021	674	613.14	21-01-2021	14495	13959.29	21-01-2021	2886	2750.71
22-01-2021	406	458	22-01-2021	324	558.29	22-01-2021	14323	13840.43	22-01-2021	2779	2698.43
23-01-2021	410	429.57	23-01-2021	902	603.71	23-01-2021	14896	13818.43	23-01-2021	2697	2668
24-01-2021	389	404.43	24-01-2021	573	579.14	24-01-2021	13232	13714.14	24-01-2021	2752	2621
25-01-2021	252	384.86	25-01-2021	375	570.57	25-01-2021	9098	13587.14	25-01-2021	1842	2609.29

West Bengal			Karnataka			Delhi			Maharashtra		
**Date**	**New cases**	**7-day Rolling average**	**Date**	**New cases**	**7-day Rolling average**	**Date**	**New cases**	**7-day Rolling average**	**Date**	**New cases**	**7-day Rolling average**
26-01-2021	295	368.14	26-01-2021	529	554	26-01-2021	12733	13436.57	26-01-2021	2405	2625.14
27-01-2021	234	343.14	27-01-2021	428	543.57	27-01-2021	11556	12904.71	27-01-2021	2171	2504.57
28-01-2021	289	325	28-01-2021	550	525.86	28-01-2021	18912	13535.71	28-01-2021	2889	2505
29-01-2021	286	307.86	29-01-2021	468	546.43	29-01-2021	13054	13354.43	29-01-2021	2771	2503.86
30-01-2021	310	293.57	30-01-2021	464	483.86	30-01-2021	13073	13094	30-01-2021	2630	2494.29
31-01-2021	229	270.71	31-01-2021	522	476.57	31-01-2021	11527	12850.43	31-01-2021	2585	2470.43
01-02-2021	179	247.14	01-02-2021	388	478.43	01-02-2021	8579	12776.29	01-02-2021	1948	2485.57
02-02-2021	203	242.43	02-02-2021	395	459.29	02-02-2021	11001	12528.86	02-02-2021	1927	2417.29
03-02-2021	201	230.57	03-02-2021	426	459	03-02-2021	12925	12724.43	03-02-2021	2992	2534.57
04-02-2021	206	217.43	04-02-2021	474	448.14	04-02-2021	12401	11794.29	04-02-2021	2736	2512.71
05-02-2021	194	201.29	05-02-2021	430	442.71	05-02-2021	11711	11602.43	05-02-2021	2628	2492.29
06-02-2021	197	199	06-02-2021	531	452.29	06-02-2021	12069	11459	06-02-2021	2768	2512
07-02-2021	193	187.57	07-02-2021	487	447.29	07-02-2021	11786	11496	07-02-2021	2673	2524.57
08-02-2021	119	179.43	08-02-2021	328	438.71	08-02-2021	8715	11515.43	08-02-2021	2216	2562.86
09-02-2021	146	176.29	09-02-2021	366	434.57	09-02-2021	10731	11476.86	09-02-2021	2515	2646.86
10-02-2021	179	177.86	10-02-2021	415	433	10-02-2021	12539	11421.71	10-02-2021	3451	2712.43
11-02-2021	217	177	11-02-2021	430	426.71	11-02-2021	9353	10986.29	11-02-2021	652	2414.71
12-02-2021	188	175.29	12-02-2021	380	419.57	12-02-2021	12137	11047.14	12-02-2021	3670	2563.57
13-02-2021	185	174.86	13-02-2021	419	403.57	13-02-2021	12198	11065.57	13-02-2021	3611	2684
14-02-2021	190	176.86	14-02-2021	414	393.14	14-02-2021	11706	11054.14	14-02-2021	4092	2886.71
15-02-2021	133	177.57	15-02-2021	368	398.86	15-02-2021	9086	11107.14	15-02-2021	3365	3050.86
16-02-2021	151	171	16-02-2021	438	409.14	16-02-2021	11592	11230.14	16-02-2021	3663	3214.86
17-02-2021	133	165.86	17-02-2021	378	403.86	17-02-2021	12862	11276.29	17-02-2021	4787	3405.71
18-02-2021	181	166.71	18-02-2021	406	400.43	18-02-2021	13237	11831.14	18-02-2021	5427	4087.86
19-02-2021	194	167.86	19-02-2021	386	401.29	19-02-2021	13916	12085.29	19-02-2021	6112	4436.71
20-02-2021	193	166.71	20-02-2021	490	411.43	20-02-2021	13919	12331.14	20-02-2021	6281	4818.14
21-02-2021	182	168.86	21-02-2021	413	411.29	21-02-2021	14278	12698.57	21-02-2021	6971	5229.43
22-02-2021	148	174.29	22-02-2021	317	404	22-02-2021	10494	12899.71	22-02-2021	5210	5493
23-02-2021	189	184.14	23-02-2021	383	396.14	23-02-2021	13682	13198.29	23-02-2021	6218	5858
24-02-2021	202	186.71	24-02-2021	334	389.86	24-02-2021	16930	13779.43	24-02-2021	8807	6432.29
25-02-2021	199	189.86	25-02-2021	453	396.57	25-02-2021	16599	14259.71	25-02-2021	8702	6900.14
26-02-2021	216	192.29	26-02-2021	571	423	26-02-2021	16562	14637.71	26-02-2021	8333	7217.43
27-02-2021	210	193.71	27-02-2021	523	427.71	27-02-2021	16805	15050	27-02-2021	8623	7552
28-02-2021	192	200.86	28-02-2021	521	443.14	28-02-2021	15614	15240.86	28-02-2021	8293	7740.86
01-03-2021	198	202.83	01-03-2021	349	447.71	01-03-2021	12270	15494.57	01-03-2021	6397	7910.43
02-03-2021	171	197.67	02-03-2021	437	455.43	02-03-2021	14998	15682.57	02-03-2021	7863	8145.43
03-03-2021	225	202	03-03-2021	528	483.14	03-03-2021	17425	15753.29	03-03-2021	9855	8295.14
04-03-2021	209	199	04-03-2021	571	500	04-03-2021	16824	15785.43	04-03-2021	8998	8337.43
05-03-2021	255	215	05-03-2021	677	515.14	05-03-2021	18324	16037.14	05-03-2021	10216	8606.43
06-03-2021	259	237	06-03-2021	580	523.29	06-03-2021	18724	16311.29	06-03-2021	10187	8829.86
07-03-2021	188	227.75	07-03-2021	622	537.71	07-03-2021	18650	16745	07-03-2021	11141	9236.71
08-03-2021	215	229.25	08-03-2021	436	550.14	08-03-2021	15353	17185.43	08-03-2021	8744	9572
09-03-2021	188	212.5	09-03-2021	590	572	09-03-2021	17873	17596.14	09-03-2021	9927	9866.86
10-03-2021	241	208	10-03-2021	760	605.14	10-03-2021	22851	18371.29	10-03-2021	13659	10410.29
11-03-2021	244	222	11-03-2021	783	635.43	11-03-2021	23298	19296.14	11-03-2021	14317	11170.14
12-03-2021	277	237.5	12-03-2021	833	657.71	12-03-2021	24845	20227.71	12-03-2021	15817	11970.29
13-03-2021	276	259.5	13-03-2021	921	706.43	13-03-2021	25154	21146.29	13-03-2021	15602	12743.86
14-03-2021	283	270	14-03-2021	934	751	14-03-2021	26513	22269.57	14-03-2021	16620	13526.57
15-03-2021	251	271.75	15-03-2021	932	821.86	15-03-2021	24437	23567.29	15-03-2021	15051	14427.57
16-03-2021	255	266.25	16-03-2021	1135	899.71	16-03-2021	28869	25138.14	16-03-2021	17864	15561.43
17-03-2021	303	273	17-03-2021	1275	973.29	17-03-2021	35838	26993.43	17-03-2021	23179	16921.43
18-03-2021	323	283	18-03-2021	1488	1074	18-03-2021	39687	29334.71	18-03-2021	25833	18566.57
19-03-2021	347	307	19-03-2021	1587	1181.71	19-03-2021	40906	31629.14	19-03-2021	25681	19975.71
20-03-2021	383	339	20-03-2021	1798	1307	20-03-2021	43815	34295	20-03-2021	27126	21622
21-03-2021	422	368.75	21-03-2021	1715	1418.57	21-03-2021	47009	37223	21-03-2021	30535	23609.86
22-03-2021	368	380	22-03-2021	1445	1491.86	22-03-2021	40636	39537.14	22-03-2021	24645	24980.43

West Bengal			Karnataka			Delhi			Maharashtra		
Date	New cases	7-day Rolling average	Date	New cases	7-day Rolling average	Date	New cases	7-day Rolling average	Date	New cases	7-day Rolling average
23-03-2021	404	394.25	23-03-2021	2010	1616.86	23-03-2021	47239	42161.43	23-03-2021	28699	26528.29
24-03-2021	462	414	24-03-2021	2298	1763	24-03-2021	53419	44673	24-03-2021	31855	27767.71
25-03-2021	516	437.5	25-03-2021	2523	1910.86	25-03-2021	59083	47443.86	25-03-2021	35952	29213.29
26-03-2021	646	507	26-03-2021	2566	2050.71	26-03-2021	62276	50496.71	26-03-2021	36902	30816.29
27-03-2021	812	609	27-03-2021	2886	2206.14	27-03-2021	62632	53184.86	27-03-2021	35726	32044.86
28-03-2021	827	700.25	28-03-2021	3082	2401.43	28-03-2021	68206	56213	28-03-2021	40414	33456.14
29-03-2021	639	731	29-03-2021	2792	2593.86	29-03-2021	56152	58429.57	29-03-2021	31643	34455.86
30-03-2021	628	726.5	30-03-2021	2975	2731.71	30-03-2021	53237	59286.43	30-03-2021	27918	34344.29
31-03-2021	982	769	31-03-2021	4225	3007	31-03-2021	72113	61957	31-03-2021	39544	35442.71
01-04-2021	1274	870	01-04-2021	4234	3251.43	01-04-2021	81398	65144.86	01-04-2021	43183	36475.71
02-04-2021	1733	1051.2	02-04-2021	4991	3597.86	02-04-2021	89023	68965.86	02-04-2021	47913	38048.71
03-04-2021	1736	1165.33	03-04-2021	4373	3810.29	03-04-2021	92994	73303.29	03-04-2021	49447	40008.86
04-04-2021	1957	1385	04-04-2021	4553	4020.43	04-04-2021	103794	78387.29	04-04-2021	57074	42388.86
05-04-2021	1961	1467.29	05-04-2021	5279	4375.71	05-04-2021	96563	84160.29	05-04-2021	47288	44623.86
06-04-2021	2058	1671.57	06-04-2021	6150	4829.29	06-04-2021	115312	93028.14	06-04-2021	55469	48559.71
07-04-2021	2390	1872.71	07-04-2021	6976	5222.29	07-04-2021	126276	100765.71	07-04-2021	59907	51468.71
08-04-2021	2783	2088.29	08-04-2021	6570	5556	08-04-2021	131878	107977.14	08-04-2021	56286	53340.57
09-04-2021	3648	2361.86	09-04-2021	7955	5979.43	09-04-2021	144945	115966	09-04-2021	58993	54923.43
10-04-2021	4043	2691.43	10-04-2021	6955	6348.29	10-04-2021	152565	124476.14	10-04-2021	55411	55775.43
11-04-2021	4398	3040.14	11-04-2021	10250	7162.14	11-04-2021	169914	133921.86	11-04-2021	63294	56664
12-04-2021	4511	3404.43	12-04-2021	9579	7776.43	12-04-2021	160838	143104	12-04-2021	51751	57301.57
13-04-2021	4817	3798.57	13-04-2021	8778	8151.86	13-04-2021	185297	153101.86	13-04-2021	60212	57979.14
14-04-2021	5892	4298.86	14-04-2021	11265	8764.57	14-04-2021	199584	163574.43	14-04-2021	58952	57842.71
15-04-2021	6769	4868.29	15-04-2021	14738	9931.43	15-04-2021	216828	175710.14	15-04-2021	61695	58615.43
16-04-2021	6910	5334.29	16-04-2021	14859	10917.71	16-04-2021	234002	188432.57	16-04-2021	63729	59292
17-04-2021	7713	5858.57	17-04-2021	17489	12422.57	17-04-2021	260895	203908.29	17-04-2021	67123	60965.14
18-04-2021	8419	6433	18-04-2021	19067	13682.14	18-04-2021	275063	218929.57	18-04-2021	68631	61727.57
19-04-2021	8426	6992.29	19-04-2021	15785	14568.71	19-04-2021	257003	232667.43	19-04-2021	58924	62752.29
20-04-2021	9819	7706.86	20-04-2021	21794	16428.14	20-04-2021	294365	248248.57	20-04-2021	62097	63021.57
21-04-2021	10784	8405.71	21-04-2021	23558	18184.29	21-04-2021	315752	264844	21-04-2021	67468	64238.14
22-04-2021	11948	9145.57	22-04-2021	25795	19763.86	22-04-2021	332531	281373	22-04-2021	67013	64997.86
23-04-2021	12876	9997.86	23-04-2021	26962	21492.86	23-04-2021	345296	297272.14	23-04-2021	66836	65441.71
24-04-2021	14281	10936.14	24-04-2021	29438	23199.86	24-04-2021	348996	309858	24-04-2021	67160	65447
25-04-2021	15889	12003.29	25-04-2021	34804	25448	25-04-2021	354658	321228.71	25-04-2021	66191	65098.43
26-04-2021	15992	13084.14	26-04-2021	29744	27442.14	26-04-2021	319471	330152.71	26-04-2021	48700	63637.86
27-04-2021	16403	14024.71	27-04-2021	31830	28875.86	27-04-2021	362913	339945.29	27-04-2021	66358	64246.57
28-04-2021	17207	14942.29	28-04-2021	39047	31088.57	28-04-2021	379404	349038.43	28-04-2021	63309	63652.43
29-04-2021	17403	15721.57	29-04-2021	35024	32407	29-04-2021	386773	356787.29	29-04-2021	66159	63530.43
30-04-2021	17411	16369.43	30-04-2021	48296	35454.71	30-04-2021	402014	364889.86	30-04-2021	62919	62970.86
01-05-2021	17512	16831	01-05-2021	40990	37105	01-05-2021	392576	371115.57	01-05-2021	63282	62416.86
02-05-2021	17515	17063.29	02-05-2021	37733	37523.43	02-05-2021	370090	373320.14	02-05-2021	56647	61053.43
03-05-2021	17501	17278.86	03-05-2021	44438	39622.57	03-05-2021	355769	378505.57	03-05-2021	48621	61042.14
04-05-2021	17639	17455.43	04-05-2021	44631	41451.29	04-05-2021	382847	381353.29	04-05-2021	51880	58973.86
05-05-2021	18102	17583.29	05-05-2021	50112	43032	05-05-2021	412624	386099	05-05-2021	57640	58164
06-05-2021	18431	17730.14	06-05-2021	49058	45036.86	06-05-2021	414280	390028.57	06-05-2021	62194	57597.57
07-05-2021	19216	17988	07-05-2021	48781	45106.14	07-05-2021	406901	390726.71	07-05-2021	54022	56326.57
08-05-2021	19436	18262.86	08-05-2021	47563	46045.14	08-05-2021	403808	392331.29	08-05-2021	56578	55368.86
09-05-2021	19441	18538	09-05-2021	47930	47501.86	09-05-2021	366455	391812	09-05-2021	48401	54190.86
10-05-2021	19445	18815.71	10-05-2021	39305	46768.57	10-05-2021	329491	388058	10-05-2021	37236	52564.43
11-05-2021	20136	19172.43	11-05-2021	39510	46037	11-05-2021	348555	383159.14	11-05-2021	40956	51003.86
12-05-2021	20136	19463	12-05-2021	39998	44592.14	12-05-2021	362632	376017.43	12-05-2021	46781	49452.57
13-05-2021	20377	19741	13-05-2021	35297	42626.29	13-05-2021	343005	365835.29	13-05-2021	42582	46650.86
14-05-2021	20839	19972.86	14-05-2021	41779	41626	14-05-2021	326256	354314.57	14-05-2021	39923	44636.71
15-05-2021	20846	20174.29	15-05-2021	41664	40783.29	15-05-2021	310757	341021.57	15-05-2021	34848	41532.43
16-05-2021	19511	20184.29	16-05-2021	31531	38440.57	16-05-2021	281837	328933.29	16-05-2021	34389	39530.71
17-05-2021	19003	20121.14	17-05-2021	38603	38340.29	17-05-2021	263021	319437.57	17-05-2021	26616	38013.57

West Bengal			Karnataka			Delhi			Maharashtra		
Date	New cases	7-day Rolling average	Date	New cases	7-day Rolling average	Date	New cases	7-day Rolling average	Date	New cases	7-day Rolling average
18-05-2021	19428	20020	18-05-2021	30309	37025.86	18-05-2021	267246	307822	18-05-2021	28438	36225.29
19-05-2021	19006	19858.57	19-05-2021	34281	36209.14	19-05-2021	276187	295472.71	19-05-2021	34031	34403.86
20-05-2021	19091	19674.86	20-05-2021	28869	35290.86	20-05-2021	259242	283506.57	20-05-2021	29911	32593.71
21-05-2021	19847	19533.14	21-05-2021	32218	33925	21-05-2021	257299	273655.57	21-05-2021	29644	31125.29
22-05-2021	18863	19249.86	22-05-2021	31183	32427.71	22-05-2021	240897	263675.57	22-05-2021	26133	29880.29
23-05-2021	18422	19094.29	23-05-2021	25979	31634.57	23-05-2021	222834	255246.57	23-05-2021	26672	28777.86
24-05-2021	17883	18934.29	24-05-2021	25311	29735.71	24-05-2021	195857	245651.71	24-05-2021	22122	28135.86
25-05-2021	17005	18588.14	25-05-2021	22758	28657	25-05-2021	208992	237329.71	25-05-2021	24136	27521.29
26-05-2021	16225	18190.86	26-05-2021	26811	27589.86	26-05-2021	211510	228090.14	26-05-2021	24752	26195.71
27-05-2021	13046	17327.29	27-05-2021	24214	26924.86	27-05-2021	186075	217637.71	27-05-2021	21273	24961.71
28-05-2021	12193	16233.86	28-05-2021	22823	25582.71	28-05-2021	174083	205749.71	28-05-2021	20740	23689.71
29-05-2021	11514	15184	29-05-2021	20628	24074.86	29-05-2021	165282	194947.57	29-05-2021	20295	22855.71
30-05-2021	11284	14164.29	30-05-2021	20378	23274.71	30-05-2021	153396	185027.86	30-05-2021	18600	21702.57
31-05-2021	10136	13057.57	31-05-2021	16604	22030.86	31-05-2021	126883	175174.43	31-05-2021	15077	20696.14
01-06-2021	9424	11974.57	01-06-2021	14304	20823.14	01-06-2021	133152	164340.14	01-06-2021	14123	19265.71
02-06-2021	8923	10931.43	02-06-2021	16387	19334	02-06-2021	134044	153273.57	02-06-2021	15169	17896.71
03-06-2021	8811	10326.43	03-06-2021	18324	18492.57	03-06-2021	132424	145609.14	03-06-2021	15229	17033.29
04-06-2021	7913	9715	04-06-2021	16068	17527.57	04-06-2021	120454	137947.86	04-06-2021	14152	16092.14
05-06-2021	7682	9167.57	05-06-2021	13800	16552.14	05-06-2021	114488	130691.57	05-06-2021	13659	15144.14
06-06-2021	7002	8555.86	06-06-2021	12209	15385.14	06-06-2021	101209	123236.29	06-06-2021	12557	14280.86
07-06-2021	5887	7948.86	07-06-2021	11958	14721.43	07-06-2021	85804	117367.86	07-06-2021	10219	13586.86
08-06-2021	5427	7377.86	08-06-2021	9808	14079.14	08-06-2021	92787	111601.43	08-06-2021	10891	13125.14
09-06-2021	5384	6872.29	09-06-2021	10959	13303.71	09-06-2021	93883	105864.14	09-06-2021	10989	12528
10-06-2021	5274	6367	10-06-2021	11042	12263.43	10-06-2021	91849	100067.71	10-06-2021	12207	12096.29
11-06-2021	4883	5934.14	11-06-2021	8249	11146.43	11-06-2021	84574	94942	11-06-2021	11766	11755.43
12-06-2021	4296	5450.43	12-06-2021	9785	10572.86	12-06-2021	80525	90090.14	12-06-2021	10697	11332.29
13-06-2021	3984	5019.29	13-06-2021	7810	9944.43	13-06-2021	71001	85774.71	13-06-2021	10442	11030.14
14-06-2021	3520	4681.14	14-06-2021	6835	9212.57	14-06-2021	60008	82089.57	14-06-2021	8129	10731.57
15-06-2021	3268	4372.71	15-06-2021	5041	8531.57	15-06-2021	62215	77722.14	15-06-2021	7652	10268.86
16-06-2021	3187	4058.86	16-06-2021	7345	8015.29	16-06-2021	67289	73923	16-06-2021	10107	10142.86
17-06-2021	3018	3736.57	17-06-2021	5983	7292.57	17-06-2021	62436	69721.14	17-06-2021	9830	9803.29
18-06-2021	2788	3437.29	18-06-2021	5783	6940.29	18-06-2021	60765	66319.86	18-06-2021	9798	9522.14
19-06-2021	2486	3178.71	19-06-2021	5815	6373.14	19-06-2021	58615	63189.86	19-06-2021	8912	9267.14
20-06-2021	2184	2921.57	20-06-2021	4517	5902.71	20-06-2021	52978	60615.14	20-06-2021	9361	9112.71
21-06-2021	1879	2687.14	21-06-2021	4867	5621.57	21-06-2021	42683	58140.14	21-06-2021	6270	8847.14
22-06-2021	1852	2484.86	22-06-2021	3709	5431.29	22-06-2021	50817	56511.86	22-06-2021	8470	8964
23-06-2021	1925	2304.57	23-06-2021	4436	5015.71	23-06-2021	54309	54657.57	23-06-2021	10066	8958.14
24-06-2021	1923	2148.14	24-06-2021	3979	4729.43	24-06-2021	51659	53118	24-06-2021	9844	8960.14
25-06-2021	1933	2026	25-06-2021	3310	4376.14	25-06-2021	48768	51404.14	25-06-2021	9604	8932.43
26-06-2021	1894	1941.43	26-06-2021	4272	4155.71	26-06-2021	49844	50151.14	26-06-2021	9812	9061
27-06-2021	1836	1891.71	27-06-2021	3604	4025.29	27-06-2021	46523	49229	27-06-2021	9974	9148.57
28-06-2021	1761	1874.86	28-06-2021	2576	3698	28-06-2021	37070	48427.14	28-06-2021	6727	9213.86
29-06-2021	1595	1838.14	29-06-2021	3222	3628.43	29-06-2021	46104	47753.86	29-06-2021	8085	9158.86
30-06-2021	1478	1774.29	30-06-2021	3382	3477.86	30-06-2021	48606	46939.14	30-06-2021	9771	9116.71

Table S3 : Tempo-spatial distribution of circulating lineages in four states.

State	Lineage	Mar-2020	Apr-2020	May-2020	Jun-2020	Jul-2020	Aug-2020	Sep-2020	Oct-2020	Nov-2020	Dec-2020	Jan-2021	Feb-2021	Mar-2021	Apr-2021	May-2021	Jun-2021	Total	Percent
West Bengal	**B.1.1**	3 (17.65%)			1 (8.33%)		1 (4.76%)	6 (6.45%)	17 (12.23%)	2 (12.5%)	8 (22.22%)	22 (12.79%)	22 (8.18%)	7 (1.1%)				89	2.78
West Bengal	**B.1**	5 (29.41%)	33 (63.46%)	82 (70.09%)	7 (58.33%)	1 (100.0%)	11 (52.38%)	30 (32.26%)	47 (33.81%)	2 (12.5%)	3 (8.33%)	17 (9.88%)	9 (3.35%)	4 (0.63%)	2 (0.21%)			253	7.91
West Bengal	**B.1.540**	3 (17.65%)	5 (9.62%)	8 (6.84%)	2 (16.67%)		1 (4.76%)	13 (13.98%)	8 (5.76%)	2 (12.5%)		1 (0.58%)	2 (0.74%)	1 (0.16%)				46	1.44
West Bengal	**B.6**	4 (23.53%)	2 (3.85%)	1 (0.85%)														7	0.22
West Bengal	**B.6.6**	2 (11.76%)	1 (1.92%)	1 (0.85%)														4	0.13
West Bengal	**A.9**		9 (17.31%)	6 (5.13%)														15	0.47
West Bengal	**B.1.206**		1 (1.92%)															1	0.03
West Bengal	**A**		1 (1.92%)											1 (0.16%)				2	0.06
West Bengal	**B.1.1.306**			9 (7.69%)				2 (2.15%)		1 (6.25%)	1 (2.78%)		5 (1.86%)	13 (2.03%)				31	0.97
West Bengal	**B.1.36**			1 (0.85%)				27 (29.03%)	24 (17.27%)	4 (25.0%)	8 (22.22%)	25 (14.53%)	26 (9.67%)	14 (2.19%)				129	4.03
West Bengal	**B.1.210**			6 (5.13%)	1 (8.33%)													7	0.22
West Bengal	**B.1.1.220**			3 (2.56%)							1 (2.78%)	31 (18.02%)	32 (11.9%)	18 (2.82%)	1 (0.1%)			86	2.69
West Bengal	**B.1.179**				1 (8.33%)													1	0.03
West Bengal	**B**						5 (23.81%)	3 (3.23%)	5 (3.6%)			3 (1.74%)						16	0.5
West Bengal	**B.1.560**						1 (4.76%)		3 (2.16%)			3 (1.74%)	3 (1.12%)					10	0.31
West Bengal	**B.1.1.216**						1 (4.76%)	10 (10.75%)	18 (12.95%)	4 (25.0%)	8 (22.22%)	29 (16.86%)	57 (21.19%)	19 (2.97%)	2 (0.21%)			148	4.63
West Bengal	**B.1.1.25**						1 (4.76%)		5 (3.6%)			9 (5.23%)	8 (2.97%)	1 (0.16%)				24	0.75
West Bengal	**B.1.36.18**							2 (2.15%)	1 (0.72%)			2 (1.16%)	4 (1.49%)	2 (0.31%)				11	0.34
West Bengal	**B.1.1.310**								1 (0.72%)									1	0.03
West Bengal	**B.1.380**								1 (0.72%)									1	0.03
West Bengal	**B.1.36.10**								1 (0.72%)			2 (1.16%)	1 (0.37%)					4	0.13
West Bengal	**B.1.111**								1 (0.72%)									1	0.03
West Bengal	**B.1.36.19**								1 (0.72%)									1	0.03
West Bengal	**B.1.1.326**								1 (0.72%)									1	0.03
West Bengal	**B.3**								1 (0.72%)									1	0.03
West Bengal	**B.1.177**								1 (0.72%)		1 (2.78%)	1 (0.58%)	2 (0.74%)	1 (0.16%)				6	0.19
West Bengal	**B.1.36.29**								1 (0.72%)			5 (2.91%)	10 (3.72%)	12 (1.88%)	1 (0.1%)			29	0.91
West Bengal	**B.1.2**								1 (0.72%)		1 (2.78%)	1 (0.58%)						3	0.09
West Bengal	**B.1.1.409**								1 (0.72%)									1	0.03
West Bengal	**B.1.1.44**									1 (6.25%)	1 (2.78%)	5 (2.91%)						7	0.22
West Bengal	**B.1.582**										3 (8.33%)			1 (0.16%)				4	0.13
West Bengal	**B.1.382**										1 (2.78%)							1	0.03
West Bengal	**B.1.94**											2 (1.16%)						2	0.06
West Bengal	**B.1.1.416**											1 (0.58%)						1	0.03
West Bengal	**B.1.1.200**											1 (0.58%)						1	0.03
West Bengal	**B.1.36.8**											2 (1.16%)						2	0.06
West Bengal	**B.1.526**											1 (0.58%)						1	0.03
West Bengal	**B.1.618**											5 (2.91%)	51 (18.96%)	93 (14.55%)	3 (0.31%)			152	4.75
West Bengal	**B.1.1.97**											1 (0.58%)						1	0.03
West Bengal	**B.1.1.452**											1 (0.58%)						1	0.03
West Bengal	**B.1.476**											1 (0.58%)						1	0.03
West Bengal	**B.1.1.141**											1 (0.58%)						1	0.03
West Bengal	**P.2**												1 (0.37%)					1	0.03
West Bengal	**B.1.1.7**												19 (7.06%)	96 (15.02%)	28 (2.92%)			143	4.47
West Bengal	**B.1.1.174**												1 (0.37%)					1	0.03
West Bengal	**B.1.617.1**												6 (2.23%)	257 (40.22%)	312 (32.53%)	15 (3.75%)		590	18.45
West Bengal	**B.1.460**												1 (0.37%)					1	0.03
West Bengal	**B.1.36.22**												1 (0.37%)	5 (0.78%)				6	0.19
West Bengal	**B.1.1.354**												1 (0.37%)					1	0.03
West Bengal	**A.29**												3 (1.12%)	1 (0.16%)	1 (0.1%)			5	0.16
West Bengal	**B.1.525**												3 (1.12%)	3 (0.47%)	2 (0.21%)	1 (0.25%)		9	0.28
West Bengal	**B.1.351.3**												1 (0.37%)	11 (1.72%)	7 (0.73%)			19	0.59
West Bengal	**B.1.351**													3 (0.47%)	1 (0.1%)			4	0.13
West Bengal	**B.1.1.500**													1 (0.16%)				1	0.03
West Bengal	**B.1.617.2**													65 (10.17%)	597 (62.25%)	384 (96.0%)	255 (100.0%)	1301	40.68
West Bengal	**B.1.1.164**													1 (0.16%)				1	0.03
West Bengal	**B.1.617.3**													9 (1.41%)	2 (0.21%)			11	0.34

Delhi

**Delhi**	**Lineage**	**Mar-2020**	**Apr-2020**	**May-2020**	**Jun-2020**	**Jul-2020**	**Aug-2020**	**Sep-2020**	**Oct-2020**	**Nov-2020**	**Dec-2020**	**Jan-2021**	**Feb-2021**	**Mar-2021**	**Apr-2021**	**May-2021**	**Jun-2021**	**Total**	**Percent**
**Delhi**	**B.1.1.7**									3 (1.49%)	5 (0.7%)	8 (16.0%)	86 (26.3%)	290 (48.9%)	118 (14.08%)	2 (10.0%)		512	16.38
**Delhi**	**B.1.617.2**													39 (6.58%)	554 (66.11%)	15 (75.0%)		608	19.46
**Delhi**	**B.1.617.1**											2 (4.0%)	26 (7.95%)	89 (15.01%)	113 (13.48%)	1 (5.0%)		231	7.39

**Delhi**	**B.1.575**														2 (0.24%)			2	0.06
**Delhi**	**B.1.469**										1 (0.14%)				1 (0.12%)			2	0.06
**Delhi**	**B.1**	4 (19.05%)	2 (4.88%)	5 (2.26%)	4 (8.89%)		4 (8.7%)			43 (21.29%)	126 (17.75%)	7 (14.0%)	90 (27.52%)	25 (4.22%)	34 (4.06%)	2 (10.0%)		346	11.07
**Delhi**	**B.1.617.3**													5 (0.84%)	5 (0.6%)			10	0.32
**Delhi**	**B.1.36.19**												1 (0.31%)		1 (0.12%)			2	0.06
**Delhi**	**B.1.36.8**						5 (10.87%)			4 (1.98%)	5 (0.7%)	1 (2.0%)	1 (0.31%)					16	0.51
**Delhi**	**B.1.1.216**						3 (6.52%)		1 (11.11%)	16 (7.92%)	115 (16.2%)	12 (24.0%)	7 (2.14%)	6 (1.01%)				160	5.12
**Delhi**	**B.1.36**	7 (33.33%)	4 (9.76%)	23 (10.41%)	35 (77.78%)	1 (100.0%)	24 (52.17%)	1 (100.0%)	4 (44.44%)	89 (44.06%)	327 (46.06%)	11 (22.0%)	31 (9.48%)	8 (1.35%)	1 (0.12%)			566	18.11
**Delhi**	**B.1.1.214**											1 (2.0%)						1	0.03
**Delhi**	**B.1.1**	5 (23.81%)	3 (7.32%)	10 (4.52%)	6 (13.33%)		4 (8.7%)		2 (22.22%)	9 (4.46%)	41 (5.77%)	1 (2.0%)	11 (3.36%)	7 (1.18%)				99	3.17
**Delhi**	**B.1.36.18**								1 (11.11%)	1 (0.5%)	4 (0.56%)	1 (2.0%)	2 (0.61%)					9	0.29
**Delhi**	**B.1.36.29**									2 (0.99%)	12 (1.69%)		7 (2.14%)	5 (0.84%)				26	0.83
**Delhi**	**B.1.1.306**						4 (8.7%)			10 (4.95%)	13 (1.83%)		1 (0.31%)	2 (0.34%)				30	0.96
**Delhi**	**B.1.1.201**									1 (0.5%)								1	0.03
**Delhi**	**B.1.210**										1 (0.14%)							1	0.03
**Delhi**	**B.1.36.22**						1 (2.17%)			2 (0.99%)	1 (0.14%)		1 (0.31%)					5	0.16
**Delhi**	**B.1.1.25**										2 (0.28%)			1 (0.17%)				3	0.1
**Delhi**	**B.1.36.10**			6 (2.71%)						4 (1.98%)	7 (0.99%)	1 (2.0%)	18 (5.5%)	3 (0.51%)				39	1.25
**Delhi**	**B.1.533**										4 (0.56%)	1 (2.0%)	6 (1.83%)					11	0.35
**Delhi**	**B.1.459**									2 (0.99%)	1 (0.14%)		1 (0.31%)					4	0.13
**Delhi**	**B.1.538**									2 (0.99%)	2 (0.28%)	1 (2.0%)	3 (0.92%)	3 (0.51%)				11	0.35
**Delhi**	**B.1.526**										1 (0.14%)			1 (0.17%)				2	0.06
**Delhi**	**B.1.170**										1 (0.14%)							1	0.03
**Delhi**	**B.1.398**												3 (0.92%)					3	0.1
**Delhi**	**AM.4**									1 (0.5%)								1	0.03
**Delhi**	**B.1.465**										3 (0.42%)							3	0.1
**Delhi**	**B.1.1.57**												1 (0.31%)					1	0.03
**Delhi**	**B.1.525**												15 (4.59%)	67 (11.3%)	4 (0.48%)			86	2.75
**Delhi**	**B.1.36.31**												2 (0.61%)					2	0.06
**Delhi**	**B.1.466.1**									1 (0.5%)			2 (0.61%)					3	0.1
**Delhi**	**B.1.1.174**												1 (0.31%)					1	0.03
**Delhi**	**B.1.560**									4 (1.98%)	5 (0.7%)							9	0.29
**Delhi**	**B.1.1.220**										4 (0.56%)							4	0.13
**Delhi**	**B.1.36.17**									1 (0.5%)	2 (0.28%)	1 (2.0%)						4	0.13
**Delhi**	**B.1.36.38**									1 (0.5%)								1	0.03
**Delhi**	**B.1.371**												1 (0.31%)					1	0.03
**Delhi**	**B.1.177**						1 (2.17%)				3 (0.42%)		1 (0.31%)					5	0.16
**Delhi**	**B.1.441**												1 (0.31%)					1	0.03
**Delhi**	**B.1.1.419**												3 (0.92%)	1 (0.17%)				4	0.13
**Delhi**	**B.1.351**												3 (0.92%)	27 (4.55%)	3 (0.36%)			33	1.06
**Delhi**	**B.1.36.35**										1 (0.14%)							1	0.03
**Delhi**	**B.1.1.318**													3 (0.51%)				3	0.1
**Delhi**	**B.6**		19 (46.34%)	130 (58.82%)											1 (0.12%)			150	4.8
**Delhi**	**B.6.6**	1 (4.76%)	11 (26.83%)	44 (19.91%)														56	1.79
**Delhi**	**B.1.2**													3 (0.51%)				3	0.1
**Delhi**	**B.1.468**									2 (0.99%)	3 (0.42%)							5	0.16
**Delhi**	**B.1.36.16**									1 (0.5%)	3 (0.42%)							4	0.13
**Delhi**	**B.1.1.354**										1 (0.14%)	1 (2.0%)	1 (0.31%)					3	0.1
**Delhi**	**B.1.557**										1 (0.14%)							1	0.03
**Delhi**	**B.1.1.291**										2 (0.28%)							2	0.06
**Delhi**	**B.1.1.398**										1 (0.14%)							1	0.03
**Delhi**	**B.1.618**									2 (0.99%)	2 (0.28%)							4	0.13
**Delhi**	**C.36**										2 (0.28%)							2	0.06
**Delhi**	**A**		1 (2.44%)								1 (0.14%)							2	0.06
**Delhi**	**B.1.1.315**									1 (0.5%)								1	0.03
**Delhi**	**B.1.1.46**										2 (0.28%)							2	0.06
**Delhi**	**B.1.1.274**										1 (0.14%)							1	0.03
**Delhi**	**B.1.523**										1 (0.14%)							1	0.03
**Delhi**	**A.23.1**													4 (0.67%)				4	0.13
**Delhi**	**B.1.466.2**													1 (0.17%)				1	0.03
**Delhi**	**B.1.36.33**										3 (0.42%)							3	0.1
**Delhi**	**B.1.221**													1 (0.17%)				1	0.03
**Delhi**	**B.1.429**													1 (0.17%)				1	0.03
**Delhi**	**B.1.367**								1 (11.11%)									1	0.03
**Delhi**	**B.1.260**			2 (0.9%)														2	0.06
**Delhi**	**B**			1 (0.45%)														1	0.03
**Delhi**	**B.1.232**												1 (0.31%)					1	0.03

**Delhi**	**L.3**											1 (2.0%)						1	0.03
**Delhi**	**B.1.411**														1 (0.12%)			1	0.03
**Delhi**	**B.1.397**													1 (0.17%)				1	0.03
**Delhi**	**B.40**	2 (9.52%)																2	0.06
**Delhi**	**A.1**	1 (4.76%)																1	0.03
**Delhi**	**A.2**	1 (4.76%)																1	0.03
**Delhi**	**B.6.1**		1 (2.44%)															1	0.03

Karnataka

**Karnataka**	**Lineage**	**Apr-2020**	**May-2020**	**Jun-2020**	**Jul-2020**	**Aug-2020**	**Sep-2020**	**Oct-2020**	**Nov-2020**	**Dec-2020**	**Jan-2021**	**Feb-2021**	**Mar-2021**	**Apr-2021**	**May-2021**	**Jun-2021**		**Total**	**Percent**
**Karnataka**	**B.1.36.29**						8 (15.09%)	11 (32.35%)	11 (42.31%)	31 (38.75%)	46 (41.82%)	17 (22.37%)	14 (7.22%)	7 (1.84%)				145	8.47
**Karnataka**	**B.1**	2 (5.26%)	12 (14.81%)	7 (7.22%)	8 (22.22%)		1 (1.89%)		1 (3.85%)	4 (5.0%)	8 (7.27%)	3 (3.95%)	11 (5.67%)	11 (2.89%)	1 (0.29%)			69	4.03
**Karnataka**	**B.1.1.7**									11 (13.75%)	13 (11.82%)	11 (14.47%)	56 (28.87%)	30 (7.87%)				121	7.07
**Karnataka**	**B.1.1**	4 (10.53%)	7 (8.64%)	12 (12.37%)			22 (41.51%)	9 (26.47%)	8 (30.77%)	4 (5.0%)	5 (4.55%)	8 (10.53%)	2 (1.03%)	1 (0.26%)				82	4.79
**Karnataka**	**B.1.1.46**		3 (3.7%)	48 (49.48%)	5 (13.89%)		9 (16.98%)	6 (17.65%)	1 (3.85%)	1 (1.25%)	2 (1.82%)							75	4.38
**Karnataka**	**B.1.1.306**		2 (2.47%)	4 (4.12%)	9 (25.0%)		1 (1.89%)	1 (2.94%)	1 (3.85%)	1 (1.25%)	1 (0.91%)	1 (1.32%)		2 (0.52%)				23	1.34
**Karnataka**	**B.1.210**	11 (28.95%)	9 (11.11%)	7 (7.22%)			1 (1.89%)											28	1.64
**Karnataka**	**B.1.36**		1 (1.23%)	5 (5.15%)			7 (13.21%)	1 (2.94%)		5 (6.25%)	10 (9.09%)							29	1.69
**Karnataka**	**B.1.1.354**						2 (3.77%)	1 (2.94%)		2 (2.5%)	2 (1.82%)	4 (5.26%)	2 (1.03%)					13	0.76
**Karnataka**	**B.1.1.326**						1 (1.89%)											1	0.06
**Karnataka**	**B.1.1.194**						1 (1.89%)	2 (5.88%)		2 (2.5%)								5	0.29
**Karnataka**	**B.1.1.231**							1 (2.94%)										1	0.06
**Karnataka**	**B.1.1.291**							1 (2.94%)										1	0.06
**Karnataka**	**B.1.1.374**							1 (2.94%)										1	0.06
**Karnataka**	**B.1.404**									1 (1.25%)								1	0.06
**Karnataka**	**B.1.617.1**											6 (7.89%)	78 (40.21%)	80 (21.0%)	4 (1.15%)			168	9.82
**Karnataka**	**B.1.560**				5 (13.89%)				2 (7.69%)	12 (15.0%)	8 (7.27%)	12 (15.79%)	4 (2.06%)					43	2.51
**Karnataka**	**B.1.617.2**											4 (5.26%)	16 (8.25%)	233 (61.15%)	340 (97.7%)	157 (100.0%)		750	43.83
**Karnataka**	**B.1.36.10**											1 (1.32%)	1 (0.52%)	6 (1.57%)				8	0.47
**Karnataka**	**AY.2**														3 (0.86%)			3	0.18
**Karnataka**	**B.1.540**										1 (0.91%)							1	0.06
**Karnataka**	**B.6**	21 (55.26%)	25 (30.86%)	4 (4.12%)														50	2.92
**Karnataka**	**B.1.36.8**		9 (11.11%)	2 (2.06%)					1 (3.85%)		2 (1.82%)	1 (1.32%)	1 (0.52%)	6 (1.57%)				22	1.29
**Karnataka**	**B.4.7**		9 (11.11%)															9	0.53
**Karnataka**	**A**		3 (3.7%)															3	0.18
**Karnataka**	**B.6.6**		1 (1.23%)															1	0.06
**Karnataka**	**B.1.537**				2 (5.56%)					1 (1.25%)								3	0.18
**Karnataka**	**B.1.177**									1 (1.25%)								1	0.06
**Karnataka**	**B.1.538**			4 (4.12%)	7 (19.44%)						1 (0.91%)		1 (0.52%)					13	0.76
**Karnataka**	**B.1.369**			1 (1.03%)														1	0.06
**Karnataka**	**B.1.548**									1 (1.25%)								1	0.06
**Karnataka**	**B.1.36.17**										1 (0.91%)							1	0.06
**Karnataka**	**A.23.1**										2 (1.82%)							2	0.12
**Karnataka**	**B.1.1.216**									1 (1.25%)	3 (2.73%)		3 (1.55%)					7	0.41
**Karnataka**	**B.1.177.4**										1 (0.91%)							1	0.06
**Karnataka**	**B.1.243**											1 (1.32%)						1	0.06
**Karnataka**	**B.1.1.274**											1 (1.32%)						1	0.06
**Karnataka**	**B.1.351**											2 (2.63%)	1 (0.52%)					3	0.18
**Karnataka**	**B.1.525**												2 (1.03%)					2	0.12
**Karnataka**	**B.1.618**												1 (0.52%)					1	0.06
**Karnataka**	**B.1.351.2**												1 (0.52%)					1	0.06
**Karnataka**	**B.1.149**											1 (1.32%)						1	0.06
**Karnataka**	**B.1.9.5**											3 (3.95%)						3	0.18
**Karnataka**	**C.36**													2 (0.52%)				2	0.12
**Karnataka**	**B.1.575**													1 (0.26%)				1	0.06
**Karnataka**	**B.1.1.318**													2 (0.52%)				2	0.12
**Karnataka**	**B.1.36.19**			1 (1.03%)							1 (0.91%)							2	0.12
**Karnataka**	**B.1.1.201**			1 (1.03%)														1	0.06
**Karnataka**	**B.1.544**			1 (1.03%)														1	0.06
**Karnataka**	**B.1.177.19**									1 (1.25%)								1	0.06
**Karnataka**	**B.1.596**									1 (1.25%)								1	0.06
**Karnataka**	**B.1.177.7**										1 (0.91%)							1	0.06
**Karnataka**	**B**								1 (3.85%)									1	0.06
**Karnataka**	**B.1.1.317**										1 (0.91%)							1	0.06
**Karnataka**	**B.1.36.18**										1 (0.91%)							1	0.06

Maharashtra

Maharashtra	Lineage	Jan-2020	Mar-2020	Apr-2020	May-2020	Jun-2020	Jul-2020	Aug-2020	Sep-2020	Oct-2020	Nov-2020	Dec-2020	Jan-2021	Feb-2021	Mar-2021	Apr-2021	May-2021	Jun-2021	Total	Percent
**Maharashtra**	**B.1.1**		1 (2.7%)	1 (0.88%)	10 (3.57%)	6 (2.23%)	13 (4.73%)	6 (3.51%)	3 (1.31%)	2 (10.53%)	5 (11.11%)	20 (12.74%)	15 (13.76%)	12 (1.62%)	22 (1.17%)	8 (0.77%)			124	2.19
**Maharashtra**	**B.1.210**		6 (16.22%)	11 (9.65%)	74 (26.43%)	31 (11.52%)	46 (16.73%)	27 (15.79%)	24 (10.48%)	1 (5.26%)		1 (0.64%)							221	3.9
**Maharashtra**	**B.1.1.306**			61 (53.51%)	145 (51.79%)	206 (76.58%)	187 (68.0%)	102 (59.65%)	152 (66.38%)	8 (42.11%)	17 (37.78%)	25 (15.92%)	12 (11.01%)	69 (9.29%)	72 (3.82%)	5 (0.48%)			1061	18.74
**Maharashtra**	**B.1.36**					6 (2.23%)	1 (0.36%)		20 (8.73%)		8 (17.78%)	24 (15.29%)	15 (13.76%)	37 (4.98%)	22 (1.17%)	1 (0.1%)			134	2.37
**Maharashtra**	**B.1.540**													3 (0.4%)	1 (0.05%)				4	0.07
**Maharashtra**	**B.1.36.29**								2 (0.87%)	4 (21.05%)	7 (15.56%)	20 (12.74%)	18 (16.51%)	114 (15.34%)	67 (3.55%)	1 (0.1%)			233	4.11
**Maharashtra**	**B.1.1.33**								1 (0.44%)										1	0.02
**Maharashtra**	**B.1.1.101**		1 (2.7%)		9 (3.21%)	3 (1.12%)	8 (2.91%)	5 (2.92%)	7 (3.06%)	2 (10.53%)		4 (2.55%)							39	0.69
**Maharashtra**	**B.1.617.1**											6 (3.82%)	16 (14.68%)	348 (46.84%)	1056 (55.99%)	323 (31.03%)	8 (2.94%)		1757	31.03
**Maharashtra**	**B.1.36.17**													6 (0.81%)	2 (0.11%)				8	0.14
**Maharashtra**	**B.1.1.7**											11 (7.01%)	12 (11.01%)	26 (3.5%)	67 (3.55%)	16 (1.54%)			132	2.33
**Maharashtra**	**B.1.1.216**						1 (0.36%)	6 (3.51%)	2 (0.87%)		1 (2.22%)	13 (8.28%)	7 (6.42%)	12 (1.62%)	12 (0.64%)				54	0.95
**Maharashtra**	**B.1.258.20**													1 (0.13%)					1	0.02
**Maharashtra**	**B.1.36.8**				1 (0.36%)		2 (0.73%)	2 (1.17%)	2 (0.87%)	1 (5.26%)		3 (1.91%)	3 (2.75%)	6 (0.81%)	1 (0.05%)				21	0.37
**Maharashtra**	**B.1**		5 (13.51%)	10 (8.77%)	31 (11.07%)	8 (2.97%)	11 (4.0%)	16 (9.36%)	11 (4.8%)	1 (5.26%)		1 (0.64%)	2 (1.83%)	32 (4.31%)	121 (6.42%)	17 (1.63%)	2 (0.74%)		268	4.73
**Maharashtra**	**B.1.468**													3 (0.4%)	1 (0.05%)	2 (0.19%)			6	0.11
**Maharashtra**	**B.1.351**											2 (1.27%)			4 (0.21%)				6	0.11
**Maharashtra**	**AE.3**											1 (0.64%)							1	0.02
**Maharashtra**	**B.1.617.2**											1 (0.64%)		4 (0.54%)	297 (15.75%)	632 (60.71%)	261 (95.96%)	14 (100.0%)	1209	21.35
**Maharashtra**	**B.1.1.326**							2 (1.17%)			1 (2.22%)	5 (3.18%)		1 (0.13%)					9	0.16
**Maharashtra**	**B.1.1.220**											1 (0.64%)		1 (0.13%)					2	0.04
**Maharashtra**	**B.1.432**										1 (2.22%)	1 (0.64%)							2	0.04
**Maharashtra**	**B.1.1.411**										1 (2.22%)								1	0.02
**Maharashtra**	**B.1.1.5**										2 (4.44%)								2	0.04
**Maharashtra**	**B.1.153**										1 (2.22%)								1	0.02
**Maharashtra**	**B.1.617.3**													29 (3.9%)	83 (4.4%)	31 (2.98%)			143	2.53
**Maharashtra**	**B.1.36.33**														2 (0.11%)				2	0.04
**Maharashtra**	**B.1.538**					2 (0.74%)	1 (0.36%)	1 (0.58%)	1 (0.44%)						8 (0.42%)	1 (0.1%)			14	0.25
**Maharashtra**	**B.1.533**											1 (0.64%)		1 (0.13%)					2	0.04
**Maharashtra**	**B.1.525**														13 (0.69%)	1 (0.1%)			14	0.25
**Maharashtra**	**AE.2**														2 (0.11%)				2	0.04
**Maharashtra**	**B.1.1.354**						1 (0.36%)		1 (0.44%)				3 (2.75%)	16 (2.15%)	8 (0.42%)				29	0.51
**Maharashtra**	**B**	2 (100.0%)													1 (0.05%)				3	0.05
**Maharashtra**	**B.6**		6 (16.22%)	23 (20.18%)	9 (3.21%)												1 (0.37%)		39	0.69
**Maharashtra**	**B.1.1.414**						3 (1.09%)	3 (1.75%)	1 (0.44%)										7	0.12
**Maharashtra**	**B.1.36.16**								1 (0.44%)			1 (0.64%)							2	0.04
**Maharashtra**	**B.6.6**		2 (5.41%)	7 (6.14%)	1 (0.36%)														10	0.18
**Maharashtra**	**B.1.36.10**					1 (0.37%)							1 (0.92%)	2 (0.27%)	4 (0.21%)	1 (0.1%)			9	0.16
**Maharashtra**	**B.1.1.28**					1 (0.37%)								1 (0.13%)					2	0.04
**Maharashtra**	**B.1.1.294**											1 (0.64%)							1	0.02
**Maharashtra**	**B.1.36.22**											3 (1.91%)	1 (0.92%)	4 (0.54%)	7 (0.37%)				15	0.26
**Maharashtra**	**B.1.1.192**											1 (0.64%)							1	0.02
**Maharashtra**	**B.1.221**											1 (0.64%)							1	0.02
**Maharashtra**	**B.1.1.174**					2 (0.74%)			1 (0.44%)				2 (1.83%)	3 (0.4%)					8	0.14
**Maharashtra**	**B.1.453**												1 (0.92%)						1	0.02
**Maharashtra**	**B.1.569**											2 (1.27%)		1 (0.13%)					3	0.05
**Maharashtra**	**B.1.560**													3 (0.4%)					3	0.05
**Maharashtra**	**A**		8 (21.62%)																8	0.14
**Maharashtra**	**B.1.1.50**											1 (0.64%)							1	0.02
**Maharashtra**	**B.1.2**													2 (0.27%)					2	0.04
**Maharashtra**	**B.1.177.86**											1 (0.64%)							1	0.02
**Maharashtra**	**B.1.36.9**											1 (0.64%)							1	0.02
**Maharashtra**	**AE.4**														1 (0.05%)				1	0.02
**Maharashtra**	**A.1**		1 (2.7%)																1	0.02
**Maharashtra**	**B.4**		6 (16.22%)																6	0.11
**Maharashtra**	**A.9**		1 (2.7%)																1	0.02
**Maharashtra**	**B.61**			1 (0.88%)															1	0.02
**Maharashtra**	**B.1.160**											1 (0.64%)	1 (0.92%)						2	0.04
**Maharashtra**	**B.1.243**													1 (0.13%)					1	0.02
**Maharashtra**	**B.1.36.35**														3 (0.16%)				3	0.05
**Maharashtra**	**B.1.222**														1 (0.05%)				1	0.02
**Maharashtra**	**B.1.413**														1 (0.05%)				1	0.02

**Maharashtra**	**A.21**													1 (0.13%)					1	0.02
**Maharashtra**	**B.1.459**														1 (0.05%)				1	0.02
**Maharashtra**	**B.1.469**													1 (0.13%)	1 (0.05%)				2	0.04
**Maharashtra**	**B.1.1.57**					3 (1.12%)													3	0.05
**Maharashtra**	**B.1.1.194**										1 (2.22%)								1	0.02
**Maharashtra**	**B.1.184**														1 (0.05%)				1	0.02
**Maharashtra**	**B.1.195**														2 (0.11%)				2	0.04
**Maharashtra**	**B.1.177.81**											1 (0.64%)							1	0.02
**Maharashtra**	**B.1.524**											1 (0.64%)							1	0.02
**Maharashtra**	**B.1.575**														1 (0.05%)	1 (0.1%)			2	0.04
**Maharashtra**	**B.1.465**														1 (0.05%)				1	0.02
**Maharashtra**	**AY.1**															1 (0.1%)			1	0.02
**Maharashtra**	**B.1.1.462**													2 (0.27%)					2	0.04
**Maharashtra**	**B.1.1.46**						1 (0.36%)												1	0.02
**Maharashtra**	**B.1.618**													1 (0.13%)					1	0.02
**Maharashtra**	**B.1.177.16**											2 (1.27%)							2	0.04
**Maharashtra**	**B.1.36.18**							1 (0.58%)											1	0.02

Table S4 : Distribution of mutations for all proteins across all states and waves

State/Gene	ORF1a											
West Bengal	Pre-Wave 1			Wave 1			Pre-Wave 2			Wave 2		
West Bengal	Mutation	Count	percent	Mutation	Count	percent	Mutation	Count	percent	Mutation	Count	percent
West Bengal	ORF1a:L3606F	13	6.77	ORF1a:D3042N	112	24.3	ORF1a:S1515F	122	20.44	ORF1a:P1640L	1005	51.59
West Bengal	ORF1a:T2016K	11	5.73	ORF1a:V3968F	52	11.28	ORF1a:S1189G	119	19.93	ORF1a:A3209V	986	50.62
West Bengal	ORF1a:S212L	9	4.69	ORF1a:L3116F	47	10.2	ORF1a:D3042N	100	16.75	ORF1a:V3718A	983	50.46
West Bengal	ORF1a:A1812D	9	4.69	ORF1a:V561A	47	10.2	ORF1a:T1001I	91	15.24	ORF1a:P309L	869	44.61
West Bengal	ORF1a:Q362L	7	3.65	ORF1a:P309L	22	4.77	ORF1a:T1567I	81	13.57	ORF1a:T3646A	810	41.58
West Bengal	ORF1a:S1029I	6	3.12	ORF1a:I404T	22	4.77	ORF1a:T3646A	79	13.23	ORF1a:H2092Y	768	39.43
West Bengal	ORF1a:G519S	5	2.6	ORF1a:K3353R	21	4.56	ORF1a:A1708D	70	11.73	ORF1a:T1567I	510	26.18
West Bengal	ORF1a:S2015R	5	2.6	ORF1a:L3606F	20	4.34	ORF1a:I2230T	69	11.56	ORF1a:P2046L	312	16.02
West Bengal	ORF1a:T2016I	5	2.6	ORF1a:K1247N	18	3.9	ORF1a:L3116F	65	10.89	ORF1a:A1306S	300	15.4
West Bengal	ORF1a:K2200N	3	1.56	ORF1a:T3032I	17	3.69	ORF1a:V3968F	65	10.89	ORF1a:V2930L	300	15.4
West Bengal	ORF1a:M3087I	3	1.56	ORF1a:T609I	15	3.25	ORF1a:P309L	46	7.71	ORF1a:T3255I	299	15.35
West Bengal	ORF1a:T1822I	3	1.56	ORF1a:M3087I	15	3.25	ORF1a:V561A	43	7.2	ORF1a:P2287S	288	14.78
West Bengal	ORF1a:Q2702H	3	1.56	ORF1a:S1029I	12	2.6	ORF1a:I404T	39	6.53	ORF1a:T3750I	135	6.93
West Bengal	ORF1a:T436I	2	1.04	ORF1a:Q3777H	12	2.6	ORF1a:K1247N	32	5.36	ORF1a:H3580Y	86	4.41
West Bengal	ORF1a:T708I	2	1.04	ORF1a:V4372I	10	2.17	ORF1a:K3353R	20	3.35	ORF1a:T1001I	76	3.9
West Bengal	ORF1a:T568I	2	1.04	ORF1a:P4211S	10	2.17	ORF1a:L3606F	20	3.35	ORF1a:A1708D	74	3.8
West Bengal	ORF1a:T592I	2	1.04	ORF1a:A1432S	8	1.74	ORF1a:M3655I	20	3.35	ORF1a:I2230T	73	3.75
West Bengal	ORF1a:P309L	2	1.04	ORF1a:S2500F	8	1.74	ORF1a:H3580Y	20	3.35	ORF1a:K261N	48	2.46
West Bengal	ORF1a:G442S	2	1.04	ORF1a:A4136V	7	1.52	ORF1a:A1812D	17	2.85	ORF1a:S1189G	27	1.39
West Bengal	ORF1a:K701N	2	1.04	ORF1a:K680N	7	1.52	ORF1a:T265I	17	2.85	ORF1a:S1515F	27	1.39
West Bengal	ORF1a:T1543I	2	1.04	ORF1a:V2238F	7	1.52	ORF1a:M3752I	15	2.51	ORF1a:K3353R	27	1.39
West Bengal	ORF1a:T1055P	2	1.04	ORF1a:T4087I	6	1.3	ORF1a:V1056L	14	2.35	ORF1a:A2587V	26	1.33
West Bengal	ORF1a:V503I	2	1.04	ORF1a:W630C	6	1.3	ORF1a:V3718F	13	2.18	ORF1a:L3606F	26	1.33
West Bengal	ORF1a:A2621V	2	1.04	ORF1a:A4094T	6	1.3	ORF1a:V1765L	12	2.01	ORF1a:K1655N	18	0.92
West Bengal	ORF1a:T3959N	2	1.04	ORF1a:A3143V	6	1.3	ORF1a:A3209V	12	2.01	ORF1a:T2836I	17	0.87
West Bengal	ORF1a:I476T	2	1.04	ORF1a:T1001I	6	1.3	ORF1a:M315V	11	1.84	ORF1a:T3284I	17	0.87
West Bengal	ORF1a:A307V	2	1.04	ORF1a:K1202N	6	1.3	ORF1a:I4049V	11	1.84	ORF1a:A2248T	16	0.82
West Bengal	ORF1a:K2849E	2	1.04	ORF1a:T2153I	6	1.3	ORF1a:P2221S	10	1.68	ORF1a:T265I	13	0.67
West Bengal	ORF1a:S1218G	2	1.04	ORF1a:T3255I	5	1.08	ORF1a:M3087I	10	1.68	ORF1a:A2344V	12	0.62
West Bengal	ORF1a:A540V	2	1.04	ORF1a:Y925N	5	1.08	ORF1a:A1043V	10	1.68	ORF1a:N2596S	12	0.62
West Bengal	ORF1a:T760S	1	0.52	ORF1a:V2588F	5	1.08	ORF1a:T2166I	10	1.68	ORF1a:M2259I	12	0.62
West Bengal	ORF1a:T2280I	1	0.52	ORF1a:N1026S	5	1.08	ORF1a:K1655N	10	1.68	ORF1a:A2249V	12	0.62
West Bengal	ORF1a:S481F	1	0.52	ORF1a:K2589R	5	1.08	ORF1a:T3032I	9	1.51	ORF1a:T2648I	11	0.56
West Bengal	ORF1a:V2324A	1	0.52	ORF1a:E102K	5	1.08	ORF1a:V3718A	9	1.51	ORF1a:A3457S	11	0.56
West Bengal	ORF1a:I3835T	1	0.52	ORF1a:M3752I	5	1.08	ORF1a:H2092Y	9	1.51	ORF1a:A3686V	11	0.56
West Bengal	ORF1a:T1589I	1	0.52	ORF1a:V1056L	5	1.08	ORF1a:A3143V	8	1.34	ORF1a:H110Y	11	0.56
West Bengal	ORF1a:V1880F	1	0.52	ORF1a:M2606L	5	1.08	ORF1a:P1640L	8	1.34	ORF1a:D2980N	11	0.56
West Bengal	ORF1a:M1083I	1	0.52	ORF1a:S1189G	5	1.08	ORF1a:P2046L	8	1.34	ORF1a:Y1814C	11	0.56
West Bengal	ORF1a:V4242I	1	0.52	ORF1a:S1515F	5	1.08	ORF1a:P1640S	8	1.34	ORF1a:S610L	11	0.56
West Bengal	ORF1a:D1547Y	1	0.52	ORF1a:V3718F	5	1.08	ORF1a:V4372I	7	1.17	ORF1a:F3138S	10	0.51
West Bengal	ORF1a:A2701T	1	0.52	ORF1a:K3678N	5	1.08	ORF1a:P4211S	7	1.17	ORF1a:H3580Q	9	0.46
West Bengal	ORF1a:A2759T	1	0.52	ORF1a:I475S	4	0.87	ORF1a:M2259I	7	1.17	ORF1a:E2089D	8	0.41
West Bengal	ORF1a:A3529V	1	0.52	ORF1a:A1061T	4	0.87	ORF1a:A2587V	7	1.17	ORF1a:P4211S	8	0.41
West Bengal	ORF1a:I2663V	1	0.52	ORF1a:T2007I	4	0.87	ORF1a:S2535L	7	1.17	ORF1a:L730F	8	0.41
West Bengal	ORF1a:A1432S	1	0.52	ORF1a:V4057I	4	0.87	ORF1a:T3459M	7	1.17	ORF1a:L3330F	8	0.41
West Bengal	ORF1a:V2613F	1	0.52	ORF1a:T4364I	4	0.87	ORF1a:N2596S	7	1.17	ORF1a:K2029R	8	0.41
West Bengal	ORF1a:S2661F	1	0.52	ORF1a:A2123V	4	0.87	ORF1a:I453V	6	1.01	ORF1a:N1662S	7	0.36
West Bengal	ORF1a:P1213L	1	0.52	ORF1a:S383G	4	0.87	ORF1a:V86F	6	1.01	ORF1a:T2906I	7	0.36
West Bengal	ORF1a:L4037F	1	0.52	ORF1a:F509S	4	0.87	ORF1a:L730F	6	1.01	ORF1a:A498V	7	0.36
West Bengal	ORF1a:A482V	1	0.52	ORF1a:S2535L	4	0.87	ORF1a:V2588F	6	1.01	ORF1a:T403I	7	0.36
West Bengal	ORF1a:T882I	1	0.52	ORF1a:A1812D	4	0.87	ORF1a:T2007I	6	1.01	ORF1a:R175C	7	0.36
West Bengal	ORF1a:N3937S	1	0.52	ORF1a:L3338F	4	0.87	ORF1a:S302F	6	1.01	ORF1a:S376L	7	0.36
West Bengal	ORF1a:Y621S	1	0.52	ORF1a:V3653F	4	0.87	ORF1a:P2018S	5	0.84	ORF1a:L3588F	7	0.36
West Bengal	ORF1a:M1312I	1	0.52	ORF1a:V2580F	4	0.87	ORF1a:A4092V	5	0.84	ORF1a:V3559I	7	0.36
West Bengal	ORF1a:V511I	1	0.52	ORF1a:F2787V	4	0.87	ORF1a:G728C	5	0.84	ORF1a:D1603E	6	0.31
West Bengal	ORF1a:S2114F	1	0.52	ORF1a:L3935M	4	0.87	ORF1a:L3935M	5	0.84	ORF1a:S2578Y	6	0.31
West Bengal	ORF1a:S531N	1	0.52	ORF1a:T1754I	4	0.87	ORF1a:T3904I	5	0.84	ORF1a:T2007I	6	0.31
West Bengal	ORF1a:R24C	1	0.52	ORF1a:V1765L	4	0.87	ORF1a:K3832N	5	0.84	ORF1a:K2029N	6	0.31
West Bengal	ORF1a:P3613L	1	0.52	ORF1a:T654I	4	0.87	ORF1a:A1679S	5	0.84	ORF1a:H2794Y	6	0.31
West Bengal	ORF1a:K399N	1	0.52	ORF1a:D996N	3	0.65	ORF1a:A876T	5	0.84	ORF1a:L3116F	5	0.26
West Bengal	ORF1a:T1637I	1	0.52	ORF1a:T2166I	3	0.65	ORF1a:A1049V	5	0.84	ORF1a:M3655I	5	0.26
West Bengal	ORF1a:G112S	1	0.52	ORF1a:S3517F	3	0.65	ORF1a:A2637V	5	0.84	ORF1a:K1247N	5	0.26
West Bengal	ORF1a:G2987S	1	0.52	ORF1a:I431M	3	0.65	ORF1a:G192S	5	0.84	ORF1a:K680N	5	0.26
West Bengal	ORF1a:S4398L	1	0.52	ORF1a:D992Y	3	0.65	ORF1a:A4016V	4	0.67	ORF1a:T2823I	5	0.26
West Bengal	ORF1a:A2614V	1	0.52	ORF1a:A3730S	3	0.65	ORF1a:M4241I	4	0.67	ORF1a:A3454V	5	0.26
West Bengal	ORF1a:S2797F	1	0.52	ORF1a:V2943I	3	0.65	ORF1a:Q314K	4	0.67	ORF1a:K634Q	5	0.26
West Bengal	ORF1a:H1160Y	1	0.52	ORF1a:V3367A	3	0.65	ORF1a:T3058I	4	0.67	ORF1a:K3979N	5	0.26
West Bengal	ORF1a:A385V	1	0.52	ORF1a:Q362L	3	0.65	ORF1a:E940D	4	0.67	ORF1a:M3752I	5	0.26

West Bengal	ORF1a:T2532A	1	0.52	ORF1a:D1228Y	3	0.65	ORF1a:E57K	4	0.67	ORF1a:E441D	5	0.26
West Bengal	ORF1a:V1299L	1	0.52	ORF1a:T4083M	3	0.65	ORF1a:V54I	4	0.67	ORF1a:P3952S	5	0.26
West Bengal	ORF1a:N1691D	1	0.52	ORF1a:P959S	3	0.65	ORF1a:E913D	4	0.67	ORF1a:M3087I	5	0.26
West Bengal	ORF1a:H3076Y	1	0.52	ORF1a:S302F	3	0.65	ORF1a:V274L	4	0.67	ORF1a:V2613F	5	0.26
West Bengal	ORF1a:D996N	1	0.52	ORF1a:A117S	3	0.65	ORF1a:S2625F	4	0.67	ORF1a:T1022I	5	0.26
West Bengal	ORF1a:V3510F	1	0.52	ORF1a:T592A	3	0.65	ORF1a:V2061I	4	0.67	ORF1a:A1151V	5	0.26
West Bengal	ORF1a:H110Y	1	0.52	ORF1a:K2511N	3	0.65	ORF1a:T4031I	4	0.67	ORF1a:H45Y	5	0.26
West Bengal	ORF1a:A2994F	1	0.52	ORF1a:I453V	3	0.65	ORF1a:S2578Y	4	0.67	ORF1a:D3042N	4	0.21
West Bengal	ORF1a:I1577V	1	0.52	ORF1a:P1158S	3	0.65	ORF1a:G519S	3	0.5	ORF1a:V3968F	4	0.21
West Bengal	ORF1a:R2515K	1	0.52	ORF1a:P1921S	3	0.65	ORF1a:K680N	3	0.5	ORF1a:A819T	4	0.21
West Bengal	ORF1a:T2846I	1	0.52	ORF1a:S2104I	3	0.65	ORF1a:M3934I	3	0.5	ORF1a:I404T	4	0.21
West Bengal	ORF1a:I431T	1	0.52	ORF1a:E1007D	3	0.65	ORF1a:V3653F	3	0.5	ORF1a:T1000I	4	0.21
West Bengal	ORF1a:L454F	1	0.52	ORF1a:T2197I	3	0.65	ORF1a:T1665I	3	0.5	ORF1a:E755K	4	0.21
West Bengal	ORF1a:K4039R	1	0.52	ORF1a:S3687L	3	0.65	ORF1a:R24C	3	0.5	ORF1a:L2062F	4	0.21
West Bengal	ORF1a:A1314T	1	0.52	ORF1a:E754D	3	0.65	ORF1a:T781A	3	0.5	ORF1a:T2166I	4	0.21
West Bengal	ORF1a:S1520F	1	0.52	ORF1a:P3613S	3	0.65	ORF1a:M1193I	3	0.5	ORF1a:S2797F	4	0.21
West Bengal	ORF1a:A498V	1	0.52	ORF1a:P1640S	3	0.65	ORF1a:T1754I	3	0.5	ORF1a:R398C	4	0.21
West Bengal	ORF1a:L3694F	1	0.52	ORF1a:P2221S	3	0.65	ORF1a:D4165A	3	0.5	ORF1a:T2967I	4	0.21
West Bengal	ORF1a:H2799Y	1	0.52	ORF1a:S2981F	2	0.43	ORF1a:P3613S	3	0.5	ORF1a:P1497L	4	0.21
West Bengal	ORF1a:D335G	1	0.52	ORF1a:Q607H	2	0.43	ORF1a:A3220V	3	0.5	ORF1a:A656V	4	0.21
West Bengal	ORF1a:K2511N	1	0.52	ORF1a:W646L	2	0.43	ORF1a:Q3777R	3	0.5	ORF1a:Y422C	4	0.21
West Bengal	ORF1a:N1094S	1	0.52	ORF1a:L3951F	2	0.43	ORF1a:S1733N	3	0.5	ORF1a:M3129I	4	0.21
West Bengal	ORF1a:G3334D	1	0.52	ORF1a:S1188L	2	0.43	ORF1a:K2029N	3	0.5	ORF1a:S485I	4	0.21
West Bengal	ORF1a:G638S	1	0.52	ORF1a:A656V	2	0.43	ORF1a:S1361F	3	0.5	ORF1a:T4090I	4	0.21
West Bengal	ORF1a:G894C	1	0.52	ORF1a:N1137S	2	0.43	ORF1a:S279F	3	0.5	ORF1a:S2048F	4	0.21
West Bengal	ORF1a:E3909G	1	0.52	ORF1a:P804L	2	0.43	ORF1a:G445V	3	0.5	ORF1a:I1045V	4	0.21
West Bengal	ORF1a:V306F	1	0.52	ORF1a:K1280N	2	0.43	ORF1a:L552I	3	0.5	ORF1a:E1340D	4	0.21
West Bengal	ORF1a:D1228Y	1	0.52	ORF1a:V2061F	2	0.43	ORF1a:T1638I	3	0.5	ORF1a:V3349I	4	0.21
West Bengal	ORF1a:K3353R	1	0.52	ORF1a:A2584V	2	0.43	ORF1a:A2033T	3	0.5	ORF1a:M85I	4	0.21
West Bengal	ORF1a:V1211F	1	0.52	ORF1a:P2568S	2	0.43	ORF1a:P927L	3	0.5	ORF1a:C2210F	4	0.21
West Bengal	ORF1a:V2061F	1	0.52	ORF1a:P1497S	2	0.43	ORF1a:A498S	3	0.5	ORF1a:A1204T	4	0.21
West Bengal	ORF1a:R4053K	1	0.52	ORF1a:G519S	2	0.43	ORF1a:L1955F	3	0.5	ORF1a:E4097D	4	0.21
West Bengal				ORF1a:L631F	2	0.43	ORF1a:P3371S	3	0.5	ORF1a:K798N	4	0.21
West Bengal				ORF1a:E1013D	2	0.43	ORF1a:T760I	3	0.5	ORF1a:Y2968N	3	0.15
West Bengal				ORF1a:D928Y	2	0.43	ORF1a:L4182F	3	0.5	ORF1a:G192S	3	0.15
West Bengal				ORF1a:S944L	2	0.43	ORF1a:L3667F	3	0.5	ORF1a:S2900L	3	0.15
West Bengal				ORF1a:A3620V	2	0.43	ORF1a:P1497S	3	0.5	ORF1a:V1765L	3	0.15
West Bengal				ORF1a:S3884L	2	0.43	ORF1a:A3730S	3	0.5	ORF1a:T760I	3	0.15
West Bengal				ORF1a:R1170H	2	0.43	ORF1a:A1352V	3	0.5	ORF1a:L4182F	3	0.15
West Bengal				ORF1a:S3973A	2	0.43	ORF1a:P748L	3	0.5	ORF1a:A516V	3	0.15
West Bengal				ORF1a:A498V	2	0.43	ORF1a:I3306V	3	0.5	ORF1a:T1597I	3	0.15
West Bengal				ORF1a:T2124I	2	0.43	ORF1a:L2105F	2	0.34	ORF1a:E2112A	3	0.15
West Bengal				ORF1a:D930Y	2	0.43	ORF1a:A1997V	2	0.34	ORF1a:E37D	3	0.15
West Bengal				ORF1a:T1682I	2	0.43	ORF1a:D2801E	2	0.34	ORF1a:Q1224H	3	0.15
West Bengal				ORF1a:A1420V	2	0.43	ORF1a:T592I	2	0.34	ORF1a:D2136Y	3	0.15
West Bengal				ORF1a:V2793I	2	0.43	ORF1a:A3518V	2	0.34	ORF1a:L3027F	3	0.15
West Bengal				ORF1a:G1100C	2	0.43	ORF1a:P1497L	2	0.34	ORF1a:V2061I	3	0.15
West Bengal				ORF1a:T1474I	2	0.43	ORF1a:V3367A	2	0.34	ORF1a:A3497V	3	0.15
West Bengal				ORF1a:I2490V	2	0.43	ORF1a:L3930F	2	0.34	ORF1a:D139N	3	0.15
West Bengal				ORF1a:S2151N	2	0.43	ORF1a:S376L	2	0.34	ORF1a:S2103F	3	0.15
West Bengal				ORF1a:P3504S	2	0.43	ORF1a:A3657D	2	0.34	ORF1a:G728C	3	0.15
West Bengal				ORF1a:S376L	2	0.43	ORF1a:T2153I	2	0.34	ORF1a:P1692S	3	0.15
West Bengal				ORF1a:P2110S	2	0.43	ORF1a:K3678N	2	0.34	ORF1a:I2138V	3	0.15
West Bengal				ORF1a:M4152I	2	0.43	ORF1a:E102K	2	0.34	ORF1a:L815F	3	0.15
West Bengal				ORF1a:S2103F	2	0.43	ORF1a:K2589R	2	0.34	ORF1a:P1054S	3	0.15
West Bengal				ORF1a:S428G	2	0.43	ORF1a:L3338F	2	0.34	ORF1a:T568I	3	0.15
West Bengal				ORF1a:L1853F	2	0.43	ORF1a:T1682I	2	0.34	ORF1a:Q1813H	3	0.15
West Bengal				ORF1a:G894D	2	0.43	ORF1a:P4075S	2	0.34	ORF1a:M2036I	3	0.15
West Bengal				ORF1a:D571N	2	0.43	ORF1a:I300F	2	0.34	ORF1a:L2869F	3	0.15
West Bengal				ORF1a:M3221I	2	0.43	ORF1a:M3661V	2	0.34	ORF1a:V1143F	3	0.15
West Bengal				ORF1a:D1547N	2	0.43	ORF1a:C655F	2	0.34	ORF1a:V1550F	3	0.15
West Bengal				ORF1a:E457K	2	0.43	ORF1a:A2593V	2	0.34	ORF1a:M3761V	3	0.15
West Bengal				ORF1a:L3027F	2	0.43	ORF1a:T4087I	2	0.34	ORF1a:A486V	3	0.15
West Bengal				ORF1a:T4164I	2	0.43	ORF1a:Q990R	2	0.34	ORF1a:V1287L	3	0.15
West Bengal				ORF1a:P1213T	2	0.43	ORF1a:E472D	2	0.34	ORF1a:I3758V	3	0.15
West Bengal				ORF1a:G2509D	2	0.43	ORF1a:S1029I	2	0.34	ORF1a:S443F	3	0.15
West Bengal				ORF1a:K1343R	2	0.43	ORF1a:T1820A	2	0.34	ORF1a:E743D	3	0.15
West Bengal				ORF1a:Q3536K	2	0.43	ORF1a:A1432V	2	0.34	ORF1a:S2389L	3	0.15
West Bengal				ORF1a:S443F	2	0.43	ORF1a:Q563R	2	0.34	ORF1a:T1881I	3	0.15
West Bengal				ORF1a:D1926G	2	0.43	ORF1a:E1293D	2	0.34	ORF1a:Q3777H	3	0.15
West Bengal				ORF1a:D953Y	2	0.43	ORF1a:A2344T	2	0.34	ORF1a:M1647I	3	0.15
West Bengal				ORF1a:L2077F	2	0.43	ORF1a:L3754F	2	0.34	ORF1a:A1234V	3	0.15
West Bengal				ORF1a:S3384L	2	0.43	ORF1a:A4136V	2	0.34	ORF1a:K2511N	3	0.15

**West Bengal**				**ORF1a:T1998I**	2	0.43	**ORF1a:R402C**	2	0.34	**ORF1a:V649F**	3	0.15
**West Bengal**				**ORF1a:T3459M**	2	0.43	**ORF1a:P309S**	2	0.34	**ORF1a:I849V**	3	0.15
**West Bengal**				**ORF1a:A3497V**	2	0.43	**ORF1a:P959S**	2	0.34	**ORF1a:S944L**	3	0.15
**West Bengal**				**ORF1a:S610A**	2	0.43	**ORF1a:A1234V**	2	0.34	**ORF1a:G1155S**	3	0.15
**West Bengal**				**ORF1a:A968V**	2	0.43	**ORF1a:M1014V**	2	0.34	**ORF1a:M3862I**	3	0.15
**West Bengal**				**ORF1a:T333M**	2	0.43	**ORF1a:T1474I**	2	0.34	**ORF1a:T891I**	2	0.1
**West Bengal**				**ORF1a:L2084I**	2	0.43	**ORF1a:Y3722C**	2	0.34	**ORF1a:L681R**	2	0.1
**West Bengal**				**ORF1a:A1938S**	1	0.22	**ORF1a:T3750I**	2	0.34	**ORF1a:R287G**	2	0.1
**West Bengal**				**ORF1a:V3336I**	1	0.22	**ORF1a:V2061F**	2	0.34	**ORF1a:R2875H**	2	0.1
**West Bengal**				**ORF1a:K3979N**	1	0.22	**ORF1a:S2378L**	2	0.34	**ORF1a:M2796I**	2	0.1
**West Bengal**				**ORF1a:A372V**	1	0.22	**ORF1a:V1169A**	2	0.34	**ORF1a:L1105V**	2	0.1
**West Bengal**				**ORF1a:T1504I**	1	0.22	**ORF1a:V3751I**	2	0.34	**ORF1a:R550H**	2	0.1
**West Bengal**				**ORF1a:P1622H**	1	0.22	**ORF1a:T1881I**	2	0.34	**ORF1a:P971L**	2	0.1
**West Bengal**				**ORF1a:N2147K**	1	0.22	**ORF1a:Y1536C**	2	0.34	**ORF1a:V1583F**	2	0.1
**West Bengal**				**ORF1a:F3397L**	1	0.22	**ORF1a:M1014I**	2	0.34	**ORF1a:T4065I**	2	0.1
**West Bengal**				**ORF1a:K634N**	1	0.22	**ORF1a:Q501K**	2	0.34	**ORF1a:Q4397R**	2	0.1
**West Bengal**				**ORF1a:G696S**	1	0.22	**ORF1a:A339V**	2	0.34	**ORF1a:S2015I**	2	0.1
**West Bengal**				**ORF1a:V1550F**	1	0.22	**ORF1a:T2087I**	2	0.34	**ORF1a:P1640S**	2	0.1
**West Bengal**				**ORF1a:V3524I**	1	0.22	**ORF1a:Q2702H**	2	0.34	**ORF1a:L3313F**	2	0.1
**West Bengal**				**ORF1a:T753I**	1	0.22	**ORF1a:A967V**	2	0.34	**ORF1a:V86F**	2	0.1
**West Bengal**				**ORF1a:T1678I**	1	0.22	**ORF1a:E870A**	2	0.34	**ORF1a:A2891V**	2	0.1
**West Bengal**				**ORF1a:Q2563H**	1	0.22	**ORF1a:S944L**	2	0.34	**ORF1a:S245F**	2	0.1
**West Bengal**				**ORF1a:T2876I**	1	0.22	**ORF1a:V3759F**	2	0.34	**ORF1a:V337I**	2	0.1
**West Bengal**				**ORF1a:V288A**	1	0.22	**ORF1a:P271S**	2	0.34	**ORF1a:P1622S**	2	0.1
**West Bengal**				**ORF1a:L628F**	1	0.22	**ORF1a:H1818Y**	2	0.34	**ORF1a:V424I**	2	0.1
**West Bengal**				**ORF1a:P2144S**	1	0.22	**ORF1a:S692F**	2	0.34	**ORF1a:T4031I**	2	0.1
**West Bengal**				**ORF1a:T4090I**	1	0.22	**ORF1a:E910D**	2	0.34	**ORF1a:G2509S**	2	0.1
**West Bengal**				**ORF1a:S4398L**	1	0.22	**ORF1a:M3221I**	2	0.34	**ORF1a:L215F**	2	0.1
**West Bengal**				**ORF1a:A1049V**	1	0.22	**ORF1a:P305L**	2	0.34	**ORF1a:T882I**	2	0.1
**West Bengal**				**ORF1a:R3802C**	1	0.22	**ORF1a:S376A**	2	0.34	**ORF1a:T340I**	2	0.1
**West Bengal**				**ORF1a:T3940I**	1	0.22	**ORF1a:D4335N**	2	0.34	**ORF1a:E1413D**	2	0.1
**West Bengal**				**ORF1a:T882I**	1	0.22	**ORF1a:T2069I**	2	0.34	**ORF1a:P1921Q**	2	0.1
**West Bengal**				**ORF1a:H2125Y**	1	0.22	**ORF1a:P1207H**	2	0.34	**ORF1a:V2340I**	2	0.1
**West Bengal**				**ORF1a:K399N**	1	0.22	**ORF1a:Q3729K**	2	0.34	**ORF1a:K2078E**	2	0.1
**West Bengal**				**ORF1a:L2122F**	1	0.22	**ORF1a:T4129I**	2	0.34	**ORF1a:V3718F**	2	0.1
**West Bengal**				**ORF1a:V1888I**	1	0.22	**ORF1a:T4204A**	2	0.34	**ORF1a:V1798I**	2	0.1
**West Bengal**				**ORF1a:K1305N**	1	0.22	**ORF1a:R207H**	2	0.34	**ORF1a:P748L**	2	0.1
**West Bengal**				**ORF1a:E1843G**	1	0.22	**ORF1a:A3454V**	2	0.34	**ORF1a:I4049V**	2	0.1
**West Bengal**				**ORF1a:E1209D**	1	0.22	**ORF1a:D828Y**	2	0.34	**ORF1a:P1220H**	2	0.1
**West Bengal**				**ORF1a:H110Y**	1	0.22	**ORF1a:L1130F**	2	0.34	**ORF1a:P305L**	2	0.1
**West Bengal**				**ORF1a:S2132G**	1	0.22	**ORF1a:V2047F**	2	0.34	**ORF1a:I3835L**	2	0.1
**West Bengal**				**ORF1a:K1860R**	1	0.22	**ORF1a:S1478Y**	2	0.34	**ORF1a:D48N**	2	0.1
**West Bengal**				**ORF1a:P2018S**	1	0.22	**ORF1a:L2179F**	2	0.34	**ORF1a:I3052V**	2	0.1
**West Bengal**				**ORF1a:L893M**	1	0.22	**ORF1a:M3911V**	2	0.34	**ORF1a:D928A**	2	0.1
**West Bengal**				**ORF1a:E311K**	1	0.22	**ORF1a:H388Y**	2	0.34	**ORF1a:T614I**	2	0.1
**West Bengal**				**ORF1a:K669N**	1	0.22	**ORF1a:I1215V**	2	0.34	**ORF1a:P1596L**	2	0.1
**West Bengal**				**ORF1a:Q1403H**	1	0.22	**ORF1a:G1095R**	2	0.34	**ORF1a:I1935T**	2	0.1
**West Bengal**				**ORF1a:R3368H**	1	0.22	**ORF1a:T3117I**	2	0.34	**ORF1a:P1786L**	2	0.1
**West Bengal**				**ORF1a:R2267G**	1	0.22	**ORF1a:L3086F**	2	0.34	**ORF1a:T1055A**	2	0.1
**West Bengal**				**ORF1a:T3904I**	1	0.22	**ORF1a:P971L**	2	0.34	**ORF1a:W630C**	2	0.1
**West Bengal**				**ORF1a:S4027N**	1	0.22	**ORF1a:L1546I**	2	0.34	**ORF1a:V3017F**	2	0.1
**West Bengal**				**ORF1a:A1352V**	1	0.22	**ORF1a:D982Y**	2	0.34	**ORF1a:A537V**	2	0.1
**West Bengal**				**ORF1a:P1786L**	1	0.22	**ORF1a:V2766F**	2	0.34	**ORF1a:G4380S**	2	0.1
**West Bengal**				**ORF1a:I1568T**	1	0.22	**ORF1a:D4200N**	2	0.34	**ORF1a:F2641L**	2	0.1
**West Bengal**				**ORF1a:A3705V**	1	0.22	**ORF1a:A690V**	2	0.34	**ORF1a:E87D**	2	0.1
**West Bengal**				**ORF1a:G4177E**	1	0.22	**ORF1a:T2836I**	2	0.34	**ORF1a:T708I**	2	0.1
**West Bengal**				**ORF1a:S3309P**	1	0.22	**ORF1a:A555V**	1	0.17	**ORF1a:T677I**	2	0.1
**West Bengal**				**ORF1a:E973G**	1	0.22	**ORF1a:T1241I**	1	0.17	**ORF1a:D3936N**	2	0.1
**West Bengal**				**ORF1a:L2062F**	1	0.22	**ORF1a:V2238F**	1	0.17	**ORF1a:G1155C**	2	0.1
**West Bengal**				**ORF1a:P6L**	1	0.22	**ORF1a:K1051N**	1	0.17	**ORF1a:L533F**	2	0.1
**West Bengal**				**ORF1a:G989D**	1	0.22	**ORF1a:T2093I**	1	0.17	**ORF1a:P3613S**	2	0.1
**West Bengal**				**ORF1a:N1109K**	1	0.22	**ORF1a:I453T**	1	0.17	**ORF1a:L580F**	2	0.1
**West Bengal**				**ORF1a:E102A**	1	0.22	**ORF1a:T1017I**	1	0.17	**ORF1a:T3487I**	2	0.1
**West Bengal**				**ORF1a:M2194V**	1	0.22	**ORF1a:K1569R**	1	0.17	**ORF1a:Q1224R**	2	0.1
**West Bengal**				**ORF1a:D2592G**	1	0.22	**ORF1a:S3309F**	1	0.17	**ORF1a:P1010S**	2	0.1
**West Bengal**				**ORF1a:A2855T**	1	0.22	**ORF1a:V4057I**	1	0.17	**ORF1a:V2324F**	2	0.1
**West Bengal**				**ORF1a:A3045T**	1	0.22	**ORF1a:T4364I**	1	0.17	**ORF1a:G934S**	2	0.1
**West Bengal**				**ORF1a:K2533R**	1	0.22	**ORF1a:M2606L**	1	0.17	**ORF1a:K1132N**	2	0.1
**West Bengal**				**ORF1a:T4159K**	1	0.22	**ORF1a:N2603S**	1	0.17	**ORF1a:A4357V**	2	0.1
**West Bengal**				**ORF1a:M3627I**	1	0.22	**ORF1a:V3107I**	1	0.17	**ORF1a:Q1021H**	2	0.1
**West Bengal**				**ORF1a:L3930F**	1	0.22	**ORF1a:L3468V**	1	0.17	**ORF1a:M1378I**	2	0.1
**West Bengal**				**ORF1a:N1934S**	1	0.22	**ORF1a:S3983F**	1	0.17	**ORF1a:K2589R**	2	0.1
**West Bengal**				**ORF1a:I1551V**	1	0.22	**ORF1a:P943S**	1	0.17	**ORF1a:P2685T**	2	0.1

**West Bengal**				**ORF1a:K2029N**	1	0.22	**ORF1a:S2797F**	1	0.17	**ORF1a:Y2513C**	2	0.1
**West Bengal**				**ORF1a:P1862S**	1	0.22	**ORF1a:R1464Q**	1	0.17	**ORF1a:K1280N**	2	0.1
**West Bengal**				**ORF1a:A3333T**	1	0.22	**ORF1a:T333A**	1	0.17	**ORF1a:M3269I**	2	0.1
**West Bengal**				**ORF1a:I3512T**	1	0.22	**ORF1a:P2605S**	1	0.17	**ORF1a:E199D**	2	0.1
**West Bengal**				**ORF1a:M1014I**	1	0.22	**ORF1a:G1155S**	1	0.17	**ORF1a:I3618V**	2	0.1
**West Bengal**				**ORF1a:P4220S**	1	0.22	**ORF1a:T2283I**	1	0.17	**ORF1a:A1420V**	2	0.1
**West Bengal**				**ORF1a:H374Y**	1	0.22	**ORF1a:Q3777H**	1	0.17	**ORF1a:V3660L**	2	0.1
**West Bengal**				**ORF1a:E973K**	1	0.22	**ORF1a:V1295F**	1	0.17	**ORF1a:S2024L**	2	0.1
**West Bengal**				**ORF1a:S2273F**	1	0.22	**ORF1a:N267K**	1	0.17	**ORF1a:G295V**	2	0.1
**West Bengal**				**ORF1a:N2140S**	1	0.22	**ORF1a:A4133V**	1	0.17	**ORF1a:E93K**	2	0.1
**West Bengal**				**ORF1a:T1874I**	1	0.22	**ORF1a:I1935S**	1	0.17	**ORF1a:M3087V**	2	0.1
**West Bengal**				**ORF1a:C3682Y**	1	0.22	**ORF1a:H2794Y**	1	0.17	**ORF1a:T1543I**	2	0.1
**West Bengal**				**ORF1a:E390G**	1	0.22	**ORF1a:G3846S**	1	0.17	**ORF1a:H2659Y**	2	0.1
**West Bengal**				**ORF1a:G2269C**	1	0.22	**ORF1a:R848M**	1	0.17	**ORF1a:A903V**	2	0.1
**West Bengal**				**ORF1a:V3420L**	1	0.22	**ORF1a:R3993L**	1	0.17	**ORF1a:G493C**	2	0.1
**West Bengal**				**ORF1a:D1167N**	1	0.22	**ORF1a:T4083M**	1	0.17	**ORF1a:V2061F**	2	0.1
**West Bengal**				**ORF1a:T2283I**	1	0.22	**ORF1a:L3829F**	1	0.17	**ORF1a:Q4385L**	2	0.1
**West Bengal**				**ORF1a:I529M**	1	0.22	**ORF1a:S212L**	1	0.17	**ORF1a:T2277I**	2	0.1
**West Bengal**				**ORF1a:I2075S**	1	0.22	**ORF1a:Q607H**	1	0.17	**ORF1a:K2067E**	2	0.1
**West Bengal**				**ORF1a:S443P**	1	0.22	**ORF1a:G445C**	1	0.17	**ORF1a:L590F**	2	0.1
**West Bengal**				**ORF1a:L2609F**	1	0.22	**ORF1a:I3842T**	1	0.17	**ORF1a:P1158S**	2	0.1
**West Bengal**				**ORF1a:S318L**	1	0.22	**ORF1a:T4090I**	1	0.17	**ORF1a:I2765L**	2	0.1
**West Bengal**				**ORF1a:A4094V**	1	0.22	**ORF1a:L3602F**	1	0.17	**ORF1a:P2950S**	2	0.1
**West Bengal**				**ORF1a:S2488F**	1	0.22	**ORF1a:I567T**	1	0.17	**ORF1a:E754K**	2	0.1
**West Bengal**				**ORF1a:P3359L**	1	0.22	**ORF1a:Y1846C**	1	0.17	**ORF1a:I1402L**	2	0.1
**West Bengal**				**ORF1a:D1625Y**	1	0.22	**ORF1a:A385V**	1	0.17	**ORF1a:D2026G**	2	0.1
**West Bengal**				**ORF1a:A482V**	1	0.22	**ORF1a:G1294D**	1	0.17	**ORF1a:T1761N**	2	0.1
**West Bengal**				**ORF1a:S1978F**	1	0.22	**ORF1a:M1647V**	1	0.17	**ORF1a:P4389L**	2	0.1
**West Bengal**				**ORF1a:V1866I**	1	0.22	**ORF1a:T2274I**	1	0.17	**ORF1a:I652T**	2	0.1
**West Bengal**				**ORF1a:G415S**	1	0.22	**ORF1a:P193S**	1	0.17	**ORF1a:G49C**	2	0.1
**West Bengal**				**ORF1a:E1024K**	1	0.22	**ORF1a:C720R**	1	0.17	**ORF1a:S2396C**	2	0.1
**West Bengal**				**ORF1a:A876T**	1	0.22	**ORF1a:L2620F**	1	0.17	**ORF1a:N1181K**	2	0.1
**West Bengal**				**ORF1a:D2094E**	1	0.22	**ORF1a:K4198N**	1	0.17	**ORF1a:K1763N**	2	0.1
**West Bengal**				**ORF1a:S984I**	1	0.22	**ORF1a:V2973I**	1	0.17	**ORF1a:Y2217C**	2	0.1
**West Bengal**				**ORF1a:P2950S**	1	0.22	**ORF1a:A2388V**	1	0.17	**ORF1a:V3017I**	2	0.1
**West Bengal**				**ORF1a:I3758V**	1	0.22	**ORF1a:M315T**	1	0.17	**ORF1a:T3665I**	1	0.05
**West Bengal**				**ORF1a:V3968A**	1	0.22	**ORF1a:T2183I**	1	0.17	**ORF1a:D1157G**	1	0.05
**West Bengal**				**ORF1a:G415D**	1	0.22	**ORF1a:A1151V**	1	0.17	**ORF1a:S2535L**	1	0.05
**West Bengal**				**ORF1a:P732S**	1	0.22	**ORF1a:K3577N**	1	0.17	**ORF1a:A690V**	1	0.05
**West Bengal**				**ORF1a:M902I**	1	0.22	**ORF1a:N3725D**	1	0.17	**ORF1a:S391F**	1	0.05
**West Bengal**				**ORF1a:S1612L**	1	0.22	**ORF1a:S2517F**	1	0.17	**ORF1a:S944P**	1	0.05
**West Bengal**				**ORF1a:N418S**	1	0.22	**ORF1a:F3064S**	1	0.17	**ORF1a:Q3777R**	1	0.05
**West Bengal**				**ORF1a:V852I**	1	0.22	**ORF1a:T4035I**	1	0.17	**ORF1a:I222V**	1	0.05
**West Bengal**				**ORF1a:V1629A**	1	0.22	**ORF1a:I280L**	1	0.17	**ORF1a:V779I**	1	0.05
**West Bengal**				**ORF1a:P305L**	1	0.22	**ORF1a:V3559I**	1	0.17	**ORF1a:M2347V**	1	0.05
**West Bengal**				**ORF1a:I431T**	1	0.22	**ORF1a:T4164I**	1	0.17	**ORF1a:L3350I**	1	0.05
**West Bengal**				**ORF1a:T554N**	1	0.22	**ORF1a:F3604L**	1	0.17	**ORF1a:W612C**	1	0.05
**West Bengal**				**ORF1a:L1130F**	1	0.22	**ORF1a:V60I**	1	0.17	**ORF1a:A776S**	1	0.05
**West Bengal**				**ORF1a:T2684I**	1	0.22	**ORF1a:T4161I**	1	0.17	**ORF1a:G514R**	1	0.05
**West Bengal**				**ORF1a:G1155C**	1	0.22	**ORF1a:A1473S**	1	0.17	**ORF1a:T1474I**	1	0.05
**West Bengal**				**ORF1a:T1890I**	1	0.22	**ORF1a:P4220L**	1	0.17	**ORF1a:D795N**	1	0.05
**West Bengal**				**ORF1a:V2203I**	1	0.22	**ORF1a:P1213L**	1	0.17	**ORF1a:M4241I**	1	0.05
**West Bengal**				**ORF1a:A903V**	1	0.22	**ORF1a:K3351R**	1	0.17	**ORF1a:A3392V**	1	0.05
**West Bengal**				**ORF1a:V2019F**	1	0.22	**ORF1a:I695V**	1	0.17	**ORF1a:K290N**	1	0.05
**West Bengal**				**ORF1a:P197S**	1	0.22	**ORF1a:D953Y**	1	0.17	**ORF1a:A2320V**	1	0.05
**West Bengal**				**ORF1a:A4162T**	1	0.22	**ORF1a:M2606T**	1	0.17	**ORF1a:A3939V**	1	0.05
**West Bengal**				**ORF1a:M2096R**	1	0.22	**ORF1a:K225R**	1	0.17	**ORF1a:G7C**	1	0.05
**West Bengal**				**ORF1a:N1999T**	1	0.22	**ORF1a:K564N**	1	0.17	**ORF1a:L3951F**	1	0.05
**West Bengal**				**ORF1a:E775V**	1	0.22	**ORF1a:A599V**	1	0.17	**ORF1a:T2106I**	1	0.05
**West Bengal**				**ORF1a:T4065I**	1	0.22	**ORF1a:G2118D**	1	0.17	**ORF1a:P4212S**	1	0.05
**West Bengal**				**ORF1a:I4127V**	1	0.22	**ORF1a:G2251C**	1	0.17	**ORF1a:T999I**	1	0.05
**West Bengal**				**ORF1a:S3309A**	1	0.22	**ORF1a:V1866I**	1	0.17	**ORF1a:K672N**	1	0.05
**West Bengal**				**ORF1a:C873Y**	1	0.22	**ORF1a:K1202N**	1	0.17	**ORF1a:P3371L**	1	0.05
**West Bengal**				**ORF1a:T1017A**	1	0.22	**ORF1a:R3368H**	1	0.17	**ORF1a:A859V**	1	0.05
**West Bengal**				**ORF1a:P1054L**	1	0.22	**ORF1a:Y925N**	1	0.17	**ORF1a:A206V**	1	0.05
**West Bengal**				**ORF1a:H2659Y**	1	0.22	**ORF1a:T708I**	1	0.17	**ORF1a:D928Y**	1	0.05
**West Bengal**				**ORF1a:S538L**	1	0.22	**ORF1a:T882I**	1	0.17	**ORF1a:I453T**	1	0.05
**West Bengal**				**ORF1a:L1243P**	1	0.22	**ORF1a:T1055I**	1	0.17	**ORF1a:T1589I**	1	0.05
**West Bengal**				**ORF1a:G1591R**	1	0.22	**ORF1a:G2091C**	1	0.17	**ORF1a:G227D**	1	0.05
**West Bengal**				**ORF1a:P782L**	1	0.22	**ORF1a:L4191F**	1	0.17	**ORF1a:D1849N**	1	0.05
**West Bengal**				**ORF1a:P3952L**	1	0.22	**ORF1a:K719Q**	1	0.17	**ORF1a:P1207S**	1	0.05
**West Bengal**				**ORF1a:D2074N**	1	0.22	**ORF1a:G1069E**	1	0.17	**ORF1a:S2695R**	1	0.05
**West Bengal**				**ORF1a:L384F**	1	0.22	**ORF1a:P1692S**	1	0.17	**ORF1a:V3986M**	1	0.05

West Bengal				ORF1a:C3059Y	1	0.22	ORF1a:T3749I	1	0.17	ORF1a:F3624S	1	0.05
West Bengal				ORF1a:S1478Y	1	0.22	ORF1a:V660A	1	0.17	ORF1a:K2200R	1	0.05
West Bengal				ORF1a:I569V	1	0.22	ORF1a:V879I	1	0.17	ORF1a:I238V	1	0.05
West Bengal				ORF1a:V3593F	1	0.22	ORF1a:P3447L	1	0.17	ORF1a:A3061D	1	0.05
West Bengal				ORF1a:A211V	1	0.22	ORF1a:P926S	1	0.17	ORF1a:V54I	1	0.05
West Bengal				ORF1a:F487L	1	0.22	ORF1a:S3732F	1	0.17	ORF1a:D981G	1	0.05
West Bengal				ORF1a:Q985K	1	0.22	ORF1a:Y196H	1	0.17	ORF1a:A1812D	1	0.05
West Bengal				ORF1a:A1306V	1	0.22	ORF1a:T1945I	1	0.17	ORF1a:V3653F	1	0.05
West Bengal				ORF1a:T3202M	1	0.22	ORF1a:T4207I	1	0.17	ORF1a:M2365V	1	0.05
West Bengal				ORF1a:A599V	1	0.22	ORF1a:E102A	1	0.17	ORF1a:P309S	1	0.05
West Bengal				ORF1a:V1085I	1	0.22	ORF1a:T554N	1	0.17	ORF1a:I300T	1	0.05
West Bengal				ORF1a:H1160Y	1	0.22	ORF1a:T2152I	1	0.17	ORF1a:T2093I	1	0.05
West Bengal				ORF1a:R3938G	1	0.22	ORF1a:A1397V	1	0.17	ORF1a:L397F	1	0.05
West Bengal				ORF1a:L802F	1	0.22	ORF1a:A1268T	1	0.17	ORF1a:S558F	1	0.05
West Bengal				ORF1a:H3125Y	1	0.22	ORF1a:K3499R	1	0.17	ORF1a:V378I	1	0.05
West Bengal				ORF1a:E57K	1	0.22	ORF1a:A1204V	1	0.17	ORF1a:M3312I	1	0.05
West Bengal				ORF1a:G137V	1	0.22	ORF1a:A4394T	1	0.17	ORF1a:L1797I	1	0.05
West Bengal				ORF1a:T1000I	1	0.22	ORF1a:G963C	1	0.17	ORF1a:E347K	1	0.05
West Bengal				ORF1a:P2046S	1	0.22	ORF1a:P3359S	1	0.17	ORF1a:P2929L	1	0.05
West Bengal				ORF1a:P4220L	1	0.22	ORF1a:M3627I	1	0.17	ORF1a:P1213L	1	0.05
West Bengal				ORF1a:T1637I	1	0.22	ORF1a:L469F	1	0.17	ORF1a:A354V	1	0.05
West Bengal				ORF1a:T2800I	1	0.22	ORF1a:S1102G	1	0.17	ORF1a:T1822I	1	0.05
West Bengal				ORF1a:A429G	1	0.22	ORF1a:I3108S	1	0.17	ORF1a:N840S	1	0.05
West Bengal				ORF1a:F3156L	1	0.22	ORF1a:A903V	1	0.17	ORF1a:T3308I	1	0.05
West Bengal				ORF1a:A3657D	1	0.22	ORF1a:T2846I	1	0.17	ORF1a:K399R	1	0.05
West Bengal				ORF1a:L446F	1	0.22	ORF1a:G514R	1	0.17	ORF1a:Y621H	1	0.05
West Bengal				ORF1a:T4035I	1	0.22	ORF1a:N840S	1	0.17	ORF1a:S3195G	1	0.05
West Bengal				ORF1a:M85V	1	0.22	ORF1a:S1218I	1	0.17	ORF1a:G728S	1	0.05
West Bengal				ORF1a:A1283V	1	0.22	ORF1a:A1679V	1	0.17	ORF1a:V2400F	1	0.05
West Bengal				ORF1a:A2909T	1	0.22	ORF1a:G728D	1	0.17	ORF1a:R560C	1	0.05
West Bengal				ORF1a:D3009G	1	0.22	ORF1a:I1970V	1	0.17	ORF1a:A872T	1	0.05
West Bengal				ORF1a:S3099L	1	0.22	ORF1a:P3359L	1	0.17	ORF1a:A2554V	1	0.05
West Bengal				ORF1a:V439I	1	0.22	ORF1a:R119H	1	0.17	ORF1a:D1933G	1	0.05
West Bengal				ORF1a:L3201I	1	0.22	ORF1a:T1035I	1	0.17	ORF1a:S100N	1	0.05
West Bengal				ORF1a:E113G	1	0.22	ORF1a:T1822I	1	0.17	ORF1a:S485C	1	0.05
West Bengal				ORF1a:V3166A	1	0.22	ORF1a:T4364K	1	0.17	ORF1a:T3749I	1	0.05
West Bengal				ORF1a:L3776F	1	0.22	ORF1a:S858F	1	0.17	ORF1a:S2151G	1	0.05
West Bengal				ORF1a:A1677S	1	0.22	ORF1a:L2760F	1	0.17	ORF1a:F2780L	1	0.05
West Bengal				ORF1a:P271S	1	0.22	ORF1a:D928Y	1	0.17	ORF1a:T2197I	1	0.05
West Bengal				ORF1a:Y4227C	1	0.22	ORF1a:M3312I	1	0.17	ORF1a:P1622T	1	0.05
West Bengal				ORF1a:D2074Y	1	0.22	ORF1a:D1624G	1	0.17	ORF1a:S3309F	1	0.05
West Bengal				ORF1a:Q575R	1	0.22	ORF1a:T4265I	1	0.17	ORF1a:G761C	1	0.05
West Bengal				ORF1a:V410M	1	0.22	ORF1a:A1306S	1	0.17	ORF1a:G94C	1	0.05
West Bengal				ORF1a:G82C	1	0.22	ORF1a:T2121I	1	0.17	ORF1a:L624P	1	0.05
West Bengal				ORF1a:I300F	1	0.22	ORF1a:S4398L	1	0.17	ORF1a:Q956H	1	0.05
West Bengal				ORF1a:K3360R	1	0.22	ORF1a:R3267K	1	0.17	ORF1a:L747F	1	0.05
West Bengal				ORF1a:S3547G	1	0.22	ORF1a:L1249F	1	0.17	ORF1a:T2087A	1	0.05
West Bengal				ORF1a:W630R	1	0.22	ORF1a:Y1064C	1	0.17	ORF1a:T224I	1	0.05
West Bengal				ORF1a:A1234V	1	0.22	ORF1a:T1496I	1	0.17	ORF1a:G3617S	1	0.05
West Bengal				ORF1a:A181T	1	0.22	ORF1a:M2254T	1	0.17	ORF1a:A1060T	1	0.05
West Bengal				ORF1a:V1211F	1	0.22	ORF1a:L204F	1	0.17	ORF1a:K2059Q	1	0.05
West Bengal				ORF1a:S1872G	1	0.22	ORF1a:V823I	1	0.17	ORF1a:T3032I	1	0.05
West Bengal				ORF1a:G3541E	1	0.22	ORF1a:N1436I	1	0.17	ORF1a:S3658N	1	0.05
West Bengal				ORF1a:T3308I	1	0.22	ORF1a:L3201F	1	0.17	ORF1a:V4372I	1	0.05
West Bengal				ORF1a:A4044V	1	0.22	ORF1a:C1114F	1	0.17	ORF1a:S1905F	1	0.05
West Bengal				ORF1a:P1220S	1	0.22	ORF1a:A1092V	1	0.17	ORF1a:T1429I	1	0.05
West Bengal				ORF1a:H1500R	1	0.22	ORF1a:E1341D	1	0.17	ORF1a:N1749K	1	0.05
West Bengal				ORF1a:H325Y	1	0.22	ORF1a:A690S	1	0.17	ORF1a:V1056L	1	0.05
West Bengal				ORF1a:A1151V	1	0.22	ORF1a:Y4077H	1	0.17	ORF1a:I2107T	1	0.05
West Bengal				ORF1a:N2966S	1	0.22	ORF1a:L2146F	1	0.17	ORF1a:D2043N	1	0.05
West Bengal				ORF1a:A1306T	1	0.22	ORF1a:A3392V	1	0.17	ORF1a:Q1758R	1	0.05
West Bengal				ORF1a:A1679V	1	0.22	ORF1a:T727I	1	0.17	ORF1a:L494F	1	0.05
West Bengal				ORF1a:V3056I	1	0.22	ORF1a:T3665I	1	0.17	ORF1a:R73C	1	0.05
West Bengal				ORF1a:T1840I	1	0.22	ORF1a:A776V	1	0.17	ORF1a:F2172V	1	0.05
West Bengal				ORF1a:Q22R	1	0.22	ORF1a:P2110S	1	0.17	ORF1a:G3757S	1	0.05
West Bengal				ORF1a:A3061T	1	0.22	ORF1a:A2696V	1	0.17	ORF1a:A1298V	1	0.05
West Bengal				ORF1a:P1158L	1	0.22	ORF1a:C3790F	1	0.17	ORF1a:S1856F	1	0.05
West Bengal				ORF1a:R232S	1	0.22	ORF1a:I473L	1	0.17	ORF1a:I4114L	1	0.05
West Bengal				ORF1a:T3284I	1	0.22	ORF1a:V1583F	1	0.17	ORF1a:Y2489H	1	0.05
West Bengal				ORF1a:E940G	1	0.22	ORF1a:S2500F	1	0.17	ORF1a:M4009V	1	0.05
West Bengal				ORF1a:M2796I	1	0.22	ORF1a:A3497V	1	0.17	ORF1a:V2652I	1	0.05
West Bengal				ORF1a:M584V	1	0.22	ORF1a:S1510F	1	0.17	ORF1a:S17I	1	0.05
West Bengal				ORF1a:V274L	1	0.22	ORF1a:S3687L	1	0.17	ORF1a:L27F	1	0.05

**West Bengal**				**ORF1a:T2002M**	1	0.22	**ORF1a:Q1021H**	1	0.17	**ORF1a:L700F**	1	0.05
**West Bengal**				**ORF1a:C2210F**	1	0.22	**ORF1a:E3764G**	1	0.17	**ORF1a:M2259L**	1	0.05
**West Bengal**				**ORF1a:Q970R**	1	0.22	**ORF1a:T814I**	1	0.17	**ORF1a:H1738Y**	1	0.05
**West Bengal**				**ORF1a:H3509Y**	1	0.22	**ORF1a:A3070V**	1	0.17	**ORF1a:I3306V**	1	0.05
**West Bengal**				**ORF1a:A2784V**	1	0.22	**ORF1a:T4174I**	1	0.17	**ORF1a:N3335S**	1	0.05
**West Bengal**				**ORF1a:V774F**	1	0.22	**ORF1a:Y136N**	1	0.17	**ORF1a:L1896W**	1	0.05
**West Bengal**				**ORF1a:L3321F**	1	0.22	**ORF1a:K734R**	1	0.17	**ORF1a:P1497S**	1	0.05
**West Bengal**				**ORF1a:A591V**	1	0.22	**ORF1a:A1268S**	1	0.17	**ORF1a:F2387L**	1	0.05
**West Bengal**				**ORF1a:S1427I**	1	0.22	**ORF1a:S1587L**	1	0.17	**ORF1a:L349F**	1	0.05
**West Bengal**				**ORF1a:T1572I**	1	0.22	**ORF1a:T2800I**	1	0.17	**ORF1a:P2221L**	1	0.05
**West Bengal**				**ORF1a:T2280I**	1	0.22	**ORF1a:H3233Y**	1	0.17	**ORF1a:R1628L**	1	0.05
**West Bengal**				**ORF1a:V3718A**	1	0.22	**ORF1a:A4396V**	1	0.17	**ORF1a:D1167E**	1	0.05
**West Bengal**				**ORF1a:A3392V**	1	0.22	**ORF1a:V1058I**	1	0.17	**ORF1a:G137S**	1	0.05
**West Bengal**				**ORF1a:D335N**	1	0.22	**ORF1a:A2529V**	1	0.17	**ORF1a:A964V**	1	0.05
**West Bengal**				**ORF1a:I880V**	1	0.22	**ORF1a:S34A**	1	0.17	**ORF1a:V1056A**	1	0.05
**West Bengal**				**ORF1a:V3653A**	1	0.22	**ORF1a:T1000I**	1	0.17	**ORF1a:T4161I**	1	0.05
**West Bengal**				**ORF1a:R398C**	1	0.22	**ORF1a:L3330F**	1	0.17	**ORF1a:P276S**	1	0.05
**West Bengal**				**ORF1a:K1281R**	1	0.22	**ORF1a:P1786S**	1	0.17	**ORF1a:K3499R**	1	0.05
**West Bengal**				**ORF1a:E1815A**	1	0.22	**ORF1a:A1793S**	1	0.17	**ORF1a:V366F**	1	0.05
**West Bengal**				**ORF1a:F2633L**	1	0.22	**ORF1a:T891I**	1	0.17	**ORF1a:P1018L**	1	0.05
**West Bengal**				**ORF1a:D2032Y**	1	0.22	**ORF1a:S4393A**	1	0.17	**ORF1a:A2759T**	1	0.05
**West Bengal**				**ORF1a:T1638I**	1	0.22	**ORF1a:A2249V**	1	0.17	**ORF1a:A2856V**	1	0.05
**West Bengal**				**ORF1a:G950D**	1	0.22	**ORF1a:S1612L**	1	0.17	**ORF1a:G209C**	1	0.05
**West Bengal**				**ORF1a:S3675F**	1	0.22	**ORF1a:I1877V**	1	0.17	**ORF1a:R2638Q**	1	0.05
**West Bengal**				**ORF1a:A1314V**	1	0.22	**ORF1a:S3099L**	1	0.17	**ORF1a:N3833S**	1	0.05
**West Bengal**				**ORF1a:A1298V**	1	0.22	**ORF1a:V3593A**	1	0.17	**ORF1a:L1680F**	1	0.05
**West Bengal**				**ORF1a:P2055S**	1	0.22	**ORF1a:P1862S**	1	0.17	**ORF1a:L3829F**	1	0.05
**West Bengal**				**ORF1a:V3367F**	1	0.22	**ORF1a:A354S**	1	0.17	**ORF1a:A1283V**	1	0.05
**West Bengal**				**ORF1a:P3447S**	1	0.22	**ORF1a:T866I**	1	0.17	**ORF1a:V2027I**	1	0.05
**West Bengal**				**ORF1a:T3868I**	1	0.22	**ORF1a:R3163G**	1	0.17	**ORF1a:T3361S**	1	0.05
**West Bengal**				**ORF1a:I2873V**	1	0.22	**ORF1a:T3361A**	1	0.17	**ORF1a:K4278R**	1	0.05
**West Bengal**				**ORF1a:L2084F**	1	0.22	**ORF1a:Y3649C**	1	0.17	**ORF1a:G98S**	1	0.05
**West Bengal**				**ORF1a:L590F**	1	0.22	**ORF1a:H1580Y**	1	0.17	**ORF1a:P305S**	1	0.05
**West Bengal**				**ORF1a:A3357T**	1	0.22	**ORF1a:Q1592R**	1	0.17	**ORF1a:K2219N**	1	0.05
**West Bengal**				**ORF1a:T4249I**	1	0.22	**ORF1a:T1773I**	1	0.17	**ORF1a:M4071K**	1	0.05
**West Bengal**				**ORF1a:E775D**	1	0.22	**ORF1a:Q4253H**	1	0.17	**ORF1a:Y185H**	1	0.05
**West Bengal**				**ORF1a:Q1292E**	1	0.22	**ORF1a:L3027F**	1	0.17	**ORF1a:T2186I**	1	0.05
**West Bengal**				**ORF1a:T2408I**	1	0.22	**ORF1a:L3808F**	1	0.17	**ORF1a:T2283I**	1	0.05
**West Bengal**				**ORF1a:P2483S**	1	0.22	**ORF1a:L3313F**	1	0.17	**ORF1a:P1950L**	1	0.05
**West Bengal**				**ORF1a:G2035R**	1	0.22	**ORF1a:V3986M**	1	0.17	**ORF1a:P1220L**	1	0.05
**West Bengal**				**ORF1a:V2340A**	1	0.22	**ORF1a:S2389T**	1	0.17	**ORF1a:K2764R**	1	0.05
**West Bengal**				**ORF1a:T1429I**	1	0.22	**ORF1a:G3334V**	1	0.17	**ORF1a:I601V**	1	0.05
**West Bengal**				**ORF1a:M2194I**	1	0.22	**ORF1a:A917V**	1	0.17	**ORF1a:M3652I**	1	0.05
**West Bengal**				**ORF1a:R207C**	1	0.22	**ORF1a:A3040V**	1	0.17	**ORF1a:L2969V**	1	0.05
**West Bengal**				**ORF1a:L16F**	1	0.22	**ORF1a:L2062F**	1	0.17	**ORF1a:I1276T**	1	0.05
**West Bengal**				**ORF1a:P1693L**	1	0.22	**ORF1a:K3979N**	1	0.17	**ORF1a:V2019F**	1	0.05
**West Bengal**				**ORF1a:I1607T**	1	0.22	**ORF1a:G2987S**	1	0.17	**ORF1a:V2865I**	1	0.05
**West Bengal**				**ORF1a:A2128V**	1	0.22	**ORF1a:A1473V**	1	0.17	**ORF1a:S1361F**	1	0.05
**West Bengal**				**ORF1a:A2098V**	1	0.22	**ORF1a:L3736F**	1	0.17	**ORF1a:P3359L**	1	0.05
**West Bengal**				**ORF1a:K734R**	1	0.22	**ORF1a:S2285F**	1	0.17	**ORF1a:V561A**	1	0.05
**West Bengal**				**ORF1a:A2033T**	1	0.22	**ORF1a:T4175I**	1	0.17	**ORF1a:Y2968H**	1	0.05
**West Bengal**				**ORF1a:N1871S**	1	0.22	**ORF1a:Q4397R**	1	0.17	**ORF1a:D992N**	1	0.05
**West Bengal**				**ORF1a:L2612I**	1	0.22	**ORF1a:D1849N**	1	0.17	**ORF1a:D981Y**	1	0.05
**West Bengal**				**ORF1a:G3846S**	1	0.22	**ORF1a:L3951F**	1	0.17	**ORF1a:I3853V**	1	0.05
**West Bengal**				**ORF1a:L1579F**	1	0.22	**ORF1a:M4009V**	1	0.17	**ORF1a:Y196H**	1	0.05
**West Bengal**				**ORF1a:S2706G**	1	0.22	**ORF1a:P380H**	1	0.17	**ORF1a:L716F**	1	0.05
**West Bengal**				**ORF1a:G327D**	1	0.22				**ORF1a:T1437K**	1	0.05
**West Bengal**				**ORF1a:G519D**	1	0.22				**ORF1a:M1807L**	1	0.05
**West Bengal**				**ORF1a:H2357Y**	1	0.22				**ORF1a:S3913L**	1	0.05
**West Bengal**				**ORF1a:T2859I**	1	0.22				**ORF1a:T4355I**	1	0.05
**West Bengal**				**ORF1a:V660D**	1	0.22				**ORF1a:M2606I**	1	0.05
**West Bengal**				**ORF1a:T1542I**	1	0.22				**ORF1a:V488L**	1	0.05
**West Bengal**				**ORF1a:V28I**	1	0.22				**ORF1a:E217G**	1	0.05
**West Bengal**				**ORF1a:T1567I**	1	0.22				**ORF1a:Q2772R**	1	0.05
**West Bengal**				**ORF1a:V3759F**	1	0.22				**ORF1a:A76T**	1	0.05
**West Bengal**				**ORF1a:P3952S**	1	0.22				**ORF1a:A3143V**	1	0.05
**West Bengal**				**ORF1a:I453T**	1	0.22				**ORF1a:T436I**	1	0.05
**West Bengal**				**ORF1a:E725K**	1	0.22				**ORF1a:V1750L**	1	0.05
**West Bengal**				**ORF1a:T3586I**	1	0.22				**ORF1a:D3526G**	1	0.05
**West Bengal**				**ORF1a:Y2788H**	1	0.22				**ORF1a:I849L**	1	0.05
**West Bengal**				**ORF1a:A3955S**	1	0.22				**ORF1a:N313Y**	1	0.05
**West Bengal**				**ORF1a:E504K**	1	0.22				**ORF1a:E250D**	1	0.05
**West Bengal**				**ORF1a:M3280I**	1	0.22				**ORF1a:Q1003H**	1	0.05

**West Bengal**				**ORF1a:A1649T**	1	0.22				**ORF1a:S2030L**	1	0.05
**West Bengal**				**ORF1a:I2010M**	1	0.22				**ORF1a:Q556K**	1	0.05
**West Bengal**				**ORF1a:T2154I**	1	0.22				**ORF1a:L4228I**	1	0.05
**West Bengal**				**ORF1a:T3258I**	1	0.22				**ORF1a:V3336F**	1	0.05
**West Bengal**				**ORF1a:Y3722C**	1	0.22				**ORF1a:A3436S**	1	0.05
**West Bengal**				**ORF1a:V4260M**	1	0.22				**ORF1a:M3626T**	1	0.05
**West Bengal**										**ORF1a:T746I**	1	0.05
**West Bengal**										**ORF1a:V2604A**	1	0.05
**West Bengal**										**ORF1a:H3233Y**	1	0.05
**West Bengal**										**ORF1a:E1549G**	1	0.05
**West Bengal**										**ORF1a:N389Y**	1	0.05
**West Bengal**										**ORF1a:A1679V**	1	0.05
**West Bengal**										**ORF1a:L1130I**	1	0.05
**West Bengal**										**ORF1a:M3780I**	1	0.05
**West Bengal**										**ORF1a:E3188K**	1	0.05
**West Bengal**										**ORF1a:T2910I**	1	0.05
**West Bengal**										**ORF1a:G7A**	1	0.05
**West Bengal**										**ORF1a:H1108Y**	1	0.05
**West Bengal**										**ORF1a:K2849R**	1	0.05
**West Bengal**										**ORF1a:I280T**	1	0.05
**West Bengal**										**ORF1a:Q455K**	1	0.05
**West Bengal**										**ORF1a:I431M**	1	0.05
**West Bengal**										**ORF1a:L628F**	1	0.05
**West Bengal**										**ORF1a:V2588F**	1	0.05
**West Bengal**										**ORF1a:L3796F**	1	0.05
**West Bengal**										**ORF1a:R542C**	1	0.05
**West Bengal**										**ORF1a:T3716I**	1	0.05
**West Bengal**										**ORF1a:A1397V**	1	0.05
**West Bengal**										**ORF1a:T2186A**	1	0.05
**West Bengal**										**ORF1a:E940D**	1	0.05
**West Bengal**										**ORF1a:E55K**	1	0.05
**West Bengal**										**ORF1a:A967T**	1	0.05
**West Bengal**										**ORF1a:P4211L**	1	0.05
**West Bengal**										**ORF1a:K1212N**	1	0.05
**West Bengal**										**ORF1a:C655R**	1	0.05
**West Bengal**										**ORF1a:K669N**	1	0.05
**West Bengal**										**ORF1a:G82D**	1	0.05
**West Bengal**										**ORF1a:I2385V**	1	0.05
**West Bengal**										**ORF1a:K2901R**	1	0.05
**West Bengal**										**ORF1a:A1649S**	1	0.05
**West Bengal**										**ORF1a:V503A**	1	0.05
**West Bengal**										**ORF1a:L204F**	1	0.05
**West Bengal**										**ORF1a:A2279T**	1	0.05
**West Bengal**										**ORF1a:L1507F**	1	0.05
**West Bengal**										**ORF1a:T1444N**	1	0.05
**West Bengal**										**ORF1a:V2203I**	1	0.05
**West Bengal**										**ORF1a:A2097V**	1	0.05
**West Bengal**										**ORF1a:Y1465C**	1	0.05
**West Bengal**										**ORF1a:F3085S**	1	0.05
**West Bengal**										**ORF1a:D2980G**	1	0.05
**West Bengal**										**ORF1a:H417Y**	1	0.05
**West Bengal**										**ORF1a:V3968I**	1	0.05
**West Bengal**										**ORF1a:M585T**	1	0.05
**West Bengal**										**ORF1a:V1071I**	1	0.05
**West Bengal**										**ORF1a:D996Y**	1	0.05
**West Bengal**										**ORF1a:C689F**	1	0.05
**West Bengal**										**ORF1a:T2906A**	1	0.05
**West Bengal**										**ORF1a:M1312I**	1	0.05
**West Bengal**										**ORF1a:P271L**	1	0.05
**West Bengal**										**ORF1a:Q1140H**	1	0.05
**West Bengal**										**ORF1a:A540V**	1	0.05
**West Bengal**										**ORF1a:T1840I**	1	0.05
**West Bengal**										**ORF1a:K2200N**	1	0.05
**West Bengal**										**ORF1a:T727I**	1	0.05
**West Bengal**										**ORF1a:A1631V**	1	0.05
**West Bengal**										**ORF1a:C1235R**	1	0.05
**West Bengal**										**ORF1a:A1462S**	1	0.05
**West Bengal**										**ORF1a:F3085L**	1	0.05
**West Bengal**										**ORF1a:Q563H**	1	0.05
**West Bengal**										**ORF1a:T4207I**	1	0.05
**West Bengal**										**ORF1a:V2715F**	1	0.05
**West Bengal**										**ORF1a:P732S**	1	0.05
**West Bengal**										**ORF1a:A3705V**	1	0.05

**West Bengal**										**ORF1a:L1145I**	1	0.05
**West Bengal**										**ORF1a:A1508V**	1	0.05
**West Bengal**										**ORF1a:M2169T**	1	0.05
**West Bengal**										**ORF1a:S531I**	1	0.05
**West Bengal**										**ORF1a:G957C**	1	0.05
**West Bengal**										**ORF1a:M2565I**	1	0.05
**West Bengal**										**ORF1a:T4174I**	1	0.05
**West Bengal**										**ORF1a:T4204I**	1	0.05
**West Bengal**										**ORF1a:P4075S**	1	0.05
**West Bengal**										**ORF1a:A405V**	1	0.05
**West Bengal**										**ORF1a:A2621S**	1	0.05
**West Bengal**										**ORF1a:R1628H**	1	0.05
**West Bengal**										**ORF1a:A1380V**	1	0.05
**West Bengal**										**ORF1a:D762G**	1	0.05
**West Bengal**										**ORF1a:T3459M**	1	0.05
**West Bengal**										**ORF1a:T965I**	1	0.05
**West Bengal**										**ORF1a:Y3010H**	1	0.05
**West Bengal**										**ORF1a:D935G**	1	0.05
**West Bengal**										**ORF1a:S428G**	1	0.05
**West Bengal**										**ORF1a:V274L**	1	0.05
**West Bengal**										**ORF1a:S838I**	1	0.05
**West Bengal**										**ORF1a:I3522T**	1	0.05
**West Bengal**										**ORF1a:M1769I**	1	0.05
**West Bengal**										**ORF1a:M584V**	1	0.05
**West Bengal**										**ORF1a:E775A**	1	0.05
**West Bengal**										**ORF1a:K2143R**	1	0.05
**West Bengal**										**ORF1a:M920I**	1	0.05
**West Bengal**										**ORF1a:P2018L**	1	0.05
**West Bengal**										**ORF1a:T2274I**	1	0.05
**West Bengal**										**ORF1a:K734R**	1	0.05
**West Bengal**										**ORF1a:R124C**	1	0.05
**West Bengal**										**ORF1a:T2495I**	1	0.05
**West Bengal**										**ORF1a:K4014R**	1	0.05
**West Bengal**										**ORF1a:V1339I**	1	0.05
**West Bengal**										**ORF1a:E2049D**	1	0.05
**West Bengal**										**ORF1a:G1068R**	1	0.05
**West Bengal**										**ORF1a:T3543I**	1	0.05
**West Bengal**										**ORF1a:S308L**	1	0.05
**West Bengal**										**ORF1a:V3281I**	1	0.05
**West Bengal**										**ORF1a:M1771T**	1	0.05
**West Bengal**										**ORF1a:V3078L**	1	0.05
**West Bengal**										**ORF1a:G52C**	1	0.05
**West Bengal**										**ORF1a:E390K**	1	0.05
**West Bengal**										**ORF1a:M789I**	1	0.05
**West Bengal**										**ORF1a:S3099L**	1	0.05
**West Bengal**										**ORF1a:N447D**	1	0.05
**West Bengal**										**ORF1a:V627A**	1	0.05
**West Bengal**										**ORF1a:N3414D**	1	0.05
**West Bengal**										**ORF1a:N1109D**	1	0.05
**West Bengal**										**ORF1a:T1496I**	1	0.05
**West Bengal**										**ORF1a:D4166N**	1	0.05
**West Bengal**										**ORF1a:A555V**	1	0.05
**West Bengal**										**ORF1a:M4116I**	1	0.05
**West Bengal**										**ORF1a:K3351R**	1	0.05
**West Bengal**										**ORF1a:T2153I**	1	0.05
**West Bengal**										**ORF1a:K1219R**	1	0.05
**West Bengal**										**ORF1a:P2195S**	1	0.05
**West Bengal**										**ORF1a:C231S**	1	0.05
**West Bengal**										**ORF1a:Q1943K**	1	0.05
**West Bengal**										**ORF1a:T554A**	1	0.05
**West Bengal**										**ORF1a:R724S**	1	0.05
**West Bengal**										**ORF1a:E553Q**	1	0.05
**West Bengal**										**ORF1a:K120N**	1	0.05
**West Bengal**										**ORF1a:T2183I**	1	0.05
**West Bengal**										**ORF1a:E913D**	1	0.05
**West Bengal**										**ORF1a:H81D**	1	0.05
**West Bengal**										**ORF1a:I1505V**	1	0.05
**West Bengal**										**ORF1a:L2609F**	1	0.05
**West Bengal**										**ORF1a:D1282Y**	1	0.05
**West Bengal**										**ORF1a:A2460V**	1	0.05
**West Bengal**										**ORF1a:Q2348***	1	0.05
**West Bengal**										**ORF1a:K634N**	1	0.05
**West Bengal**										**ORF1a:A3889V**	1	0.05

West Bengal										ORF1a:H110R	1	0.05
West Bengal										ORF1a:G1155D	1	0.05
West Bengal										ORF1a:A3333T	1	0.05
West Bengal										ORF1a:V1431I	1	0.05
West Bengal										ORF1a:Q3332R	1	0.05
West Bengal										ORF1a:P2079S	1	0.05
West Bengal										ORF1a:H1545Y	1	0.05
West Bengal										ORF1a:K650E	1	0.05
West Bengal										ORF1a:M2374I	1	0.05
West Bengal										ORF1a:S1510F	1	0.05
West Bengal										ORF1a:D1903Y	1	0.05
West Bengal										ORF1a:S2599L	1	0.05
West Bengal										ORF1a:I2634V	1	0.05
West Bengal										ORF1a:E899D	1	0.05
West Bengal										ORF1a:Y2437H	1	0.05
West Bengal										ORF1a:G445V	1	0.05
West Bengal										ORF1a:D2060Y	1	0.05
West Bengal										ORF1a:V2892F	1	0.05
West Bengal										ORF1a:A3645V	1	0.05
West Bengal										ORF1a:A599V	1	0.05
West Bengal										ORF1a:M3921I	1	0.05
West Bengal										ORF1a:T1437I	1	0.05
West Bengal										ORF1a:T4302S	1	0.05
West Bengal										ORF1a:T4368I	1	0.05
West Bengal										ORF1a:Y3723H	1	0.05
West Bengal										ORF1a:V3975F	1	0.05
West Bengal										ORF1a:S4153F	1	0.05
West Bengal										ORF1a:L3210F	1	0.05
West Bengal										ORF1a:K4176N	1	0.05
West Bengal										ORF1a:T3224A	1	0.05
West Bengal										ORF1a:A2379V	1	0.05
West Bengal										ORF1a:G442S	1	0.05
West Bengal										ORF1a:L3977I	1	0.05
West Bengal										ORF1a:P1054L	1	0.05
West Bengal										ORF1a:P3767L	1	0.05
West Bengal										ORF1a:V3976F	1	0.05
West Bengal										ORF1a:D1547Y	1	0.05
West Bengal										ORF1a:L788F	1	0.05
West Bengal										ORF1a:Q1129R	1	0.05
West Bengal										ORF1a:P197S	1	0.05
West Bengal										ORF1a:A3625P	1	0.05
West Bengal										ORF1a:V774I	1	0.05
West Bengal										ORF1a:A3220V	1	0.05
West Bengal										ORF1a:L2084I	1	0.05
West Bengal										ORF1a:K1230N	1	0.05
West Bengal										ORF1a:A482V	1	0.05
West Bengal										ORF1a:M4390T	1	0.05
West Bengal										ORF1a:E4313D	1	0.05
West Bengal										ORF1a:M85T	1	0.05
West Bengal										ORF1a:V2783I	1	0.05
West Bengal										ORF1a:P361H	1	0.05
West Bengal										ORF1a:F2338L	1	0.05
West Bengal										ORF1a:D1600G	1	0.05
West Bengal										ORF1a:S1133I	1	0.05
West Bengal										ORF1a:D1184E	1	0.05
West Bengal										ORF1a:P193A	1	0.05
West Bengal										ORF1a:R287S	1	0.05
West Bengal										ORF1a:R3574K	1	0.05
West Bengal										ORF1a:A702V	1	0.05
West Bengal										ORF1a:D571Y	1	0.05
West Bengal										ORF1a:N2054S	1	0.05
West Bengal										ORF1a:Q2702H	1	0.05
West Bengal										ORF1a:T2791P	1	0.05
West Bengal										ORF1a:E738K	1	0.05
West Bengal										ORF1a:T4088I	1	0.05
West Bengal										ORF1a:A2584V	1	0.05
West Bengal										ORF1a:G519S	1	0.05
West Bengal										ORF1a:P892S	1	0.05
West Bengal										ORF1a:R3480S	1	0.05
West Bengal										ORF1a:E386D	1	0.05
West Bengal										ORF1a:M2365I	1	0.05
West Bengal										ORF1a:A3523V	1	0.05
West Bengal										ORF1a:L16F	1	0.05

West Bengal										ORF1a:V2051L	1	0.05
West Bengal										ORF1a:K1720N	1	0.05
West Bengal										ORF1a:R550C	1	0.05
West Bengal										ORF1a:L530P	1	0.05
West Bengal										ORF1a:K564N	1	0.05
West Bengal										ORF1a:S1027G	1	0.05
West Bengal										ORF1a:A1151S	1	0.05
West Bengal										ORF1a:A372V	1	0.05
West Bengal										ORF1a:V1295I	1	0.05
West Bengal										ORF1a:A2614V	1	0.05
West Bengal										ORF1a:A591V	1	0.05
West Bengal										ORF1a:T12I	1	0.05
West Bengal										ORF1a:E289G	1	0.05
West Bengal										ORF1a:T866I	1	0.05
West Bengal										ORF1a:S302F	1	0.05
West Bengal										ORF1a:R119C	1	0.05
West Bengal										ORF1a:S538L	1	0.05
West Bengal										ORF1a:V1888I	1	0.05
West Bengal										ORF1a:A3963V	1	0.05
West Bengal										ORF1a:G133D	1	0.05
West Bengal										ORF1a:T2016I	1	0.05
West Bengal										ORF1a:R2163C	1	0.05
West Bengal										ORF1a:S2488F	1	0.05
West Bengal										ORF1a:A776D	1	0.05
West Bengal										ORF1a:N2847S	1	0.05
West Bengal										ORF1a:T1168I	1	0.05
West Bengal										ORF1a:P3371Q	1	0.05
West Bengal										ORF1a:T3308N	1	0.05
West Bengal										ORF1a:A735V	1	0.05
West Bengal										ORF1a:T4164N	1	0.05
West Bengal										ORF1a:V3759F	1	0.05
West Bengal										ORF1a:I280V	1	0.05
West Bengal										ORF1a:A4394V	1	0.05
West Bengal										ORF1a:R643I	1	0.05
West Bengal										ORF1a:V28I	1	0.05
West Bengal										ORF1a:T4304I	1	0.05
West Bengal										ORF1a:T4364I	1	0.05
West Bengal										ORF1a:S528L	1	0.05
West Bengal										ORF1a:G1073E	1	0.05
West Bengal										ORF1a:D1624G	1	0.05
West Bengal										ORF1a:A429T	1	0.05
West Bengal										ORF1a:P1265S	1	0.05
West Bengal										ORF1a:P1921L	1	0.05
West Bengal										ORF1a:T3095I	1	0.05
West Bengal										ORF1a:V2047F	1	0.05
West Bengal										ORF1a:V3136L	1	0.05
West Bengal										ORF1a:K3532R	1	0.05
West Bengal										ORF1a:P1810L	1	0.05
West Bengal										ORF1a:V3021A	1	0.05
West Bengal										ORF1a:Q526H	1	0.05
West Bengal										ORF1a:E352D	1	0.05
West Bengal										ORF1a:A2618V	1	0.05
West Bengal										ORF1a:V3510F	1	0.05
West Bengal										ORF1a:V1239F	1	0.05
West Bengal										ORF1a:L3694F	1	0.05
West Bengal										ORF1a:T4066I	1	0.05
West Bengal										ORF1a:D4384G	1	0.05
	ORF1b											
West Bengal	Pre-Wave 1			Wave 1			Pre-Wave 2			Wave 2		
West Bengal	Mutation	Count	percent	Mutation	Count	percent	Mutation	Count	percent	Mutation	Count	percent
West Bengal	ORF1b:P314L	165	85.94	ORF1b:P314L	445	96.53	ORF1b:P314L	589	98.66	ORF1b:P314L	1941	99.64
West Bengal	ORF1b:A88V	26	13.54	ORF1b:I235T	31	6.72	ORF1b:Y1200C	122	20.44	ORF1b:P1000L	1286	66.02
West Bengal	ORF1b:G662S	19	9.9	ORF1b:G662S	18	3.9	ORF1b:D260N	121	20.27	ORF1b:G662S	1284	65.91
West Bengal	ORF1b:T11A	9	4.69	ORF1b:A88V	17	3.69	ORF1b:M1352I	80	13.4	ORF1b:H2285Y	765	39.27
West Bengal	ORF1b:I1539T	3	1.56	ORF1b:R2308C	12	2.6	ORF1b:K2310R	79	13.23	ORF1b:K2310R	522	26.8
West Bengal	ORF1b:L2508F	2	1.04	ORF1b:I2320V	7	1.52	ORF1b:S2312A	71	11.89	ORF1b:M1352I	509	26.13
West Bengal	ORF1b:R1226C	2	1.04	ORF1b:K2557R	6	1.3	ORF1b:G1129C	68	11.39	ORF1b:G1129C	471	24.18
West Bengal	ORF1b:P2256L	2	1.04	ORF1b:V2102I	6	1.3	ORF1b:P2116S	29	4.86	ORF1b:S2312A	470	24.13
West Bengal	ORF1b:A1432S	2	1.04	ORF1b:L2523F	6	1.3	ORF1b:D1306E	27	4.52	ORF1b:P2116S	299	15.35
West Bengal	ORF1b:A2132V	2	1.04	ORF1b:T2163I	5	1.08	ORF1b:L2523F	22	3.69	ORF1b:A1918V	299	15.35

West Bengal	ORF1b:V222I	1	0.52	ORF1b:R2257S	5	1.08	ORF1b:V321A	15	2.51	ORF1b:D1306E	279	14.32
West Bengal	ORF1b:K2557R	1	0.52	ORF1b:D260N	5	1.08	ORF1b:K2557R	14	2.35	ORF1b:P1570L	89	4.57
West Bengal	ORF1b:E1124A	1	0.52	ORF1b:Y1200C	5	1.08	ORF1b:I235T	12	2.01	ORF1b:V321A	82	4.21
West Bengal	ORF1b:H286Y	1	0.52	ORF1b:T1774I	4	0.87	ORF1b:R1315C	11	1.84	ORF1b:T310I	68	3.49
West Bengal	ORF1b:P287T	1	0.52	ORF1b:T2432I	4	0.87	ORF1b:T1050N	11	1.84	ORF1b:D260N	27	1.39
West Bengal	ORF1b:V2152D	1	0.52	ORF1b:D2541Y	4	0.87	ORF1b:K469N	10	1.68	ORF1b:Y1200C	27	1.39
West Bengal	ORF1b:C2153Y	1	0.52	ORF1b:S1779R	4	0.87	ORF1b:P218L	9	1.51	ORF1b:M1839I	16	0.82
West Bengal	ORF1b:S2154F	1	0.52	ORF1b:V2223L	4	0.87	ORF1b:N2452S	8	1.34	ORF1b:V2237F	15	0.77
West Bengal	ORF1b:M2155V	1	0.52	ORF1b:R1315C	4	0.87	ORF1b:G662S	8	1.34	ORF1b:K469N	14	0.72
West Bengal	ORF1b:T2156P	1	0.52	ORF1b:A1884S	4	0.87	ORF1b:G1129V	7	1.17	ORF1b:T1545A	13	0.67
West Bengal	ORF1b:I2465V	1	0.52	ORF1b:P2648S	3	0.65	ORF1b:I1074V	7	1.17	ORF1b:I1074V	12	0.62
West Bengal	ORF1b:Y140H	1	0.52	ORF1b:P1727L	3	0.65	ORF1b:P1000L	7	1.17	ORF1b:D1381Y	12	0.62
West Bengal	ORF1b:G1638V	1	0.52	ORF1b:Q813H	3	0.65	ORF1b:H2285Y	7	1.17	ORF1b:P218L	12	0.62
West Bengal	ORF1b:Q813H	1	0.52	ORF1b:N725T	3	0.65	ORF1b:R2257S	6	1.01	ORF1b:T2490M	11	0.56
West Bengal	ORF1b:S1003G	1	0.52	ORF1b:A1160V	3	0.65	ORF1b:K1873N	6	1.01	ORF1b:K2557R	10	0.51
West Bengal	ORF1b:L1551I	1	0.52	ORF1b:V1092F	3	0.65	ORF1b:V2371L	5	0.84	ORF1b:D884Y	10	0.51
West Bengal	ORF1b:V248F	1	0.52	ORF1b:T1637I	3	0.65	ORF1b:K82R	5	0.84	ORF1b:T2488M	9	0.46
West Bengal	ORF1b:V321L	1	0.52	ORF1b:A1521V	3	0.65	ORF1b:A1643V	5	0.84	ORF1b:Y449H	8	0.41
West Bengal	ORF1b:L966F	1	0.52	ORF1b:V628I	3	0.65	ORF1b:V2073L	5	0.84	ORF1b:R1600S	8	0.41
West Bengal	ORF1b:D2090Y	1	0.52	ORF1b:K82R	3	0.65	ORF1b:W2383C	5	0.84	ORF1b:K2063N	8	0.41
West Bengal	ORF1b:L1681F	1	0.52	ORF1b:L1676I	3	0.65	ORF1b:E1264D	4	0.67	ORF1b:N141S	8	0.41
West Bengal	ORF1b:L1701F	1	0.52	ORF1b:T284I	3	0.65	ORF1b:D2090Y	4	0.67	ORF1b:P1975L	8	0.41
West Bengal	ORF1b:D2179Y	1	0.52	ORF1b:L1220H	3	0.65	ORF1b:Q813H	4	0.67	ORF1b:Y1176H	7	0.36
West Bengal	ORF1b:Y2018H	1	0.52	ORF1b:P1567L	2	0.43	ORF1b:N734Y	4	0.67	ORF1b:V1092F	7	0.36
West Bengal	ORF1b:T2325K	1	0.52	ORF1b:P2256S	2	0.43	ORF1b:T1424I	4	0.67	ORF1b:T2537I	6	0.31
West Bengal	ORF1b:P2648S	1	0.52	ORF1b:L1966S	2	0.43	ORF1b:M1499I	4	0.67	ORF1b:M2025T	6	0.31
West Bengal	ORF1b:D152E	1	0.52	ORF1b:T76I	2	0.43	ORF1b:D1735G	4	0.67	ORF1b:P314F	5	0.26
West Bengal	ORF1b:W1037R	1	0.52	ORF1b:A302S	2	0.43	ORF1b:I1987V	4	0.67	ORF1b:M1499I	5	0.26
West Bengal	ORF1b:G2005C	1	0.52	ORF1b:L2017V	2	0.43	ORF1b:P314F	4	0.67	ORF1b:T2048I	5	0.26
West Bengal	ORF1b:Q2425K	1	0.52	ORF1b:K2340N	2	0.43	ORF1b:D884Y	4	0.67	ORF1b:T1274I	5	0.26
West Bengal	ORF1b:V2686F	1	0.52	ORF1b:H1979Y	2	0.43	ORF1b:T385M	4	0.67	ORF1b:V248F	5	0.26
West Bengal	ORF1b:I1044T	1	0.52	ORF1b:D1028Y	2	0.43	ORF1b:M809I	4	0.67	ORF1b:N2452S	4	0.21
West Bengal	ORF1b:A2585T	1	0.52	ORF1b:P1727S	2	0.43	ORF1b:I958L	4	0.67	ORF1b:V2371L	4	0.21
West Bengal	ORF1b:S1273L	1	0.52	ORF1b:V1983L	2	0.43	ORF1b:A1521S	4	0.67	ORF1b:K1383R	4	0.21
West Bengal	ORF1b:V2061A	1	0.52	ORF1b:D2429Y	2	0.43	ORF1b:P1975S	4	0.67	ORF1b:A1219S	4	0.21
West Bengal	ORF1b:V1708I	1	0.52	ORF1b:S1159I	2	0.43	ORF1b:A88V	3	0.5	ORF1b:L2213F	4	0.21
West Bengal	ORF1b:S1779R	1	0.52	ORF1b:T310I	2	0.43	ORF1b:T1511I	3	0.5	ORF1b:P2313S	4	0.21
West Bengal	ORF1b:V2536I	1	0.52	ORF1b:D146G	2	0.43	ORF1b:G2337S	3	0.5	ORF1b:M454I	4	0.21
West Bengal	ORF1b:K82R	1	0.52	ORF1b:A1521S	2	0.43	ORF1b:P2256S	3	0.5	ORF1b:S1408A	4	0.21
West Bengal	ORF1b:E2107D	1	0.52	ORF1b:T1621I	2	0.43	ORF1b:A690S	3	0.5	ORF1b:A2132V	4	0.21
West Bengal	ORF1b:T1162I	1	0.52	ORF1b:V2345A	2	0.43	ORF1b:A1432V	3	0.5	ORF1b:Q813H	4	0.21
West Bengal	ORF1b:V389I	1	0.52	ORF1b:T2488M	2	0.43	ORF1b:A1744S	3	0.5	ORF1b:H1979Y	4	0.21
West Bengal	ORF1b:D1358N	1	0.52	ORF1b:E735G	2	0.43	ORF1b:M1677I	3	0.5	ORF1b:S1898F	4	0.21
West Bengal	ORF1b:I214V	1	0.52	ORF1b:R1260S	2	0.43	ORF1b:D870Y	3	0.5	ORF1b:G2510C	4	0.21
West Bengal	ORF1b:T1540I	1	0.52	ORF1b:S1552I	2	0.43	ORF1b:T1774I	3	0.5	ORF1b:Y337H	3	0.15
West Bengal				ORF1b:M371I	2	0.43	ORF1b:V119I	3	0.5	ORF1b:S1893F	3	0.15
West Bengal				ORF1b:A1219S	2	0.43	ORF1b:A2110S	3	0.5	ORF1b:L2523F	3	0.15
West Bengal				ORF1b:S1893F	2	0.43	ORF1b:L2017F	3	0.5	ORF1b:R1315C	3	0.15
West Bengal				ORF1b:N1946S	2	0.43	ORF1b:A1895T	3	0.5	ORF1b:A1744V	3	0.15
West Bengal				ORF1b:M115I	2	0.43	ORF1b:D1381Y	3	0.5	ORF1b:E910D	3	0.15
West Bengal				ORF1b:A1798S	2	0.43	ORF1b:A1547S	3	0.5	ORF1b:E913D	3	0.15
West Bengal				ORF1b:A2168V	2	0.43	ORF1b:V2178F	2	0.34	ORF1b:P1821S	3	0.15
West Bengal				ORF1b:S2212I	2	0.43	ORF1b:H2388Y	2	0.34	ORF1b:A1291V	3	0.15
West Bengal				ORF1b:T1522I	2	0.43	ORF1b:D2142Y	2	0.34	ORF1b:V1538L	3	0.15
West Bengal				ORF1b:T2537I	2	0.43	ORF1b:A1918V	2	0.34	ORF1b:K50N	3	0.15
West Bengal				ORF1b:P1001S	2	0.43	ORF1b:R2613N	2	0.34	ORF1b:D1848Y	3	0.15
West Bengal				ORF1b:S1408L	2	0.43	ORF1b:I257T	2	0.34	ORF1b:A941V	3	0.15
West Bengal				ORF1b:S2339Y	2	0.43	ORF1b:A2222V	2	0.34	ORF1b:V2178F	3	0.15
West Bengal				ORF1b:A2132V	2	0.43	ORF1b:S1089L	2	0.34	ORF1b:H1087Y	3	0.15
West Bengal				ORF1b:A1428V	2	0.43	ORF1b:V1691L	2	0.34	ORF1b:P1427L	3	0.15
West Bengal				ORF1b:R1737L	2	0.43	ORF1b:P1821L	2	0.34	ORF1b:G2662D	3	0.15
West Bengal				ORF1b:D1572Y	1	0.22	ORF1b:R240M	2	0.34	ORF1b:V1149L	3	0.15
West Bengal				ORF1b:S1391L	1	0.22	ORF1b:P2633S	2	0.34	ORF1b:S2212I	3	0.15
West Bengal				ORF1b:W153C	1	0.22	ORF1b:G1568C	2	0.34	ORF1b:F2353C	3	0.15
West Bengal				ORF1b:A1662V	1	0.22	ORF1b:S2312L	2	0.34	ORF1b:I1690V	3	0.15
West Bengal				ORF1b:T2376I	1	0.22	ORF1b:P1548T	2	0.34	ORF1b:N2214T	3	0.15
West Bengal				ORF1b:D815A	1	0.22	ORF1b:F1891C	2	0.34	ORF1b:Q2335P	3	0.15
West Bengal				ORF1b:D1746N	1	0.22	ORF1b:A167V	2	0.34	ORF1b:D2179G	3	0.15
West Bengal				ORF1b:P2633S	1	0.22	ORF1b:N2535S	2	0.34	ORF1b:A440S	3	0.15
West Bengal				ORF1b:M1397I	1	0.22	ORF1b:H604Y	2	0.34	ORF1b:E1264D	3	0.15
West Bengal				ORF1b:I1403V	1	0.22	ORF1b:G1824C	2	0.34	ORF1b:A373V	3	0.15
West Bengal				ORF1b:D2576Y	1	0.22	ORF1b:K360N	2	0.34	ORF1b:L1701F	3	0.15
West Bengal				ORF1b:V2371L	1	0.22	ORF1b:D1869Y	2	0.34	ORF1b:S2399I	3	0.15

West Bengal				ORF1b:H604Y	1	0.22	ORF1b:S2338L	2	0.34	ORF1b:D1183A	3	0.15
West Bengal				ORF1b:P218S	1	0.22	ORF1b:I1227M	2	0.34	ORF1b:T861A	3	0.15
West Bengal				ORF1b:G2304R	1	0.22	ORF1b:P2313L	2	0.34	ORF1b:D729G	2	0.1
West Bengal				ORF1b:P2313S	1	0.22	ORF1b:A1798S	2	0.34	ORF1b:T17I	2	0.1
West Bengal				ORF1b:F1469L	1	0.22	ORF1b:T861I	2	0.34	ORF1b:S1182L	2	0.1
West Bengal				ORF1b:V2406I	1	0.22	ORF1b:E1728D	2	0.34	ORF1b:K1246N	2	0.1
West Bengal				ORF1b:Y1278C	1	0.22	ORF1b:I1611V	2	0.34	ORF1b:A1326S	2	0.1
West Bengal				ORF1b:K50R	1	0.22	ORF1b:Q1867L	2	0.34	ORF1b:A1884S	2	0.1
West Bengal				ORF1b:S1273L	1	0.22	ORF1b:S2312P	2	0.34	ORF1b:P1427S	2	0.1
West Bengal				ORF1b:V2475G	1	0.22	ORF1b:R2308C	2	0.34	ORF1b:E1184D	2	0.1
West Bengal				ORF1b:A1954V	1	0.22	ORF1b:V666I	2	0.34	ORF1b:D1735G	2	0.1
West Bengal				ORF1b:G2474E	1	0.22	ORF1b:F2173L	2	0.34	ORF1b:T2336I	2	0.1
West Bengal				ORF1b:R2141G	1	0.22	ORF1b:M1839I	2	0.34	ORF1b:D2067E	2	0.1
West Bengal				ORF1b:A914S	1	0.22	ORF1b:I1355M	2	0.34	ORF1b:R2613N	2	0.1
West Bengal				ORF1b:A176V	1	0.22	ORF1b:V463L	2	0.34	ORF1b:D161G	2	0.1
West Bengal				ORF1b:A1190V	1	0.22	ORF1b:A1160V	2	0.34	ORF1b:G977V	2	0.1
West Bengal				ORF1b:L1504F	1	0.22	ORF1b:G2289E	2	0.34	ORF1b:L2017F	2	0.1
West Bengal				ORF1b:D51Y	1	0.22	ORF1b:V1092F	2	0.34	ORF1b:L2382F	2	0.1
West Bengal				ORF1b:V1922A	1	0.22	ORF1b:A1744V	2	0.34	ORF1b:S961L	2	0.1
West Bengal				ORF1b:V345L	1	0.22	ORF1b:D599G	1	0.17	ORF1b:A86V	2	0.1
West Bengal				ORF1b:K2063N	1	0.22	ORF1b:I2320V	1	0.17	ORF1b:A1847S	2	0.1
West Bengal				ORF1b:Y2329C	1	0.22	ORF1b:P2612L	1	0.17	ORF1b:T1404M	2	0.1
West Bengal				ORF1b:V2061L	1	0.22	ORF1b:E2196A	1	0.17	ORF1b:V2120L	2	0.1
West Bengal				ORF1b:C2341F	1	0.22	ORF1b:Q435H	1	0.17	ORF1b:D2429N	2	0.1
West Bengal				ORF1b:G1541W	1	0.22	ORF1b:T1060M	1	0.17	ORF1b:P2633L	2	0.1
West Bengal				ORF1b:L2671I	1	0.22	ORF1b:T2163I	1	0.17	ORF1b:T216I	2	0.1
West Bengal				ORF1b:V1279I	1	0.22	ORF1b:M1397I	1	0.17	ORF1b:T2163I	2	0.1
West Bengal				ORF1b:M2260I	1	0.22	ORF1b:R164L	1	0.17	ORF1b:S638I	2	0.1
West Bengal				ORF1b:V396F	1	0.22	ORF1b:V1649F	1	0.17	ORF1b:V2342M	2	0.1
West Bengal				ORF1b:V2178F	1	0.22	ORF1b:V1706I	1	0.17	ORF1b:T1774I	2	0.1
West Bengal				ORF1b:A1895V	1	0.22	ORF1b:R1577K	1	0.17	ORF1b:A697T	2	0.1
West Bengal				ORF1b:V1685L	1	0.22	ORF1b:T887I	1	0.17	ORF1b:D2270Y	2	0.1
West Bengal				ORF1b:V1493L	1	0.22	ORF1b:T1303I	1	0.17	ORF1b:V1852I	2	0.1
West Bengal				ORF1b:D1815Y	1	0.22	ORF1b:D2270Y	1	0.17	ORF1b:V1905L	2	0.1
West Bengal				ORF1b:K2111N	1	0.22	ORF1b:M1197I	1	0.17	ORF1b:L621I	2	0.1
West Bengal				ORF1b:M620I	1	0.22	ORF1b:A1805S	1	0.17	ORF1b:K2340N	2	0.1
West Bengal				ORF1b:T1511I	1	0.22	ORF1b:Y1176H	1	0.17	ORF1b:Q2400L	2	0.1
West Bengal				ORF1b:G1568S	1	0.22	ORF1b:K1571N	1	0.17	ORF1b:Q2070H	2	0.1
West Bengal				ORF1b:G1772S	1	0.22	ORF1b:P85L	1	0.17	ORF1b:K1199N	2	0.1
West Bengal				ORF1b:M2025I	1	0.22	ORF1b:T2225K	1	0.17	ORF1b:V2216I	2	0.1
West Bengal				ORF1b:C1740F	1	0.22	ORF1b:D2423N	1	0.17	ORF1b:N480K	2	0.1
West Bengal				ORF1b:P319S	1	0.22	ORF1b:L2558I	1	0.17	ORF1b:V1685I	2	0.1
West Bengal				ORF1b:A373V	1	0.22	ORF1b:H1213Y	1	0.17	ORF1b:A2565V	2	0.1
West Bengal				ORF1b:T1424I	1	0.22	ORF1b:G2662V	1	0.17	ORF1b:I2554V	2	0.1
West Bengal				ORF1b:V119A	1	0.22	ORF1b:I1822V	1	0.17	ORF1b:P1452Q	2	0.1
West Bengal				ORF1b:V87I	1	0.22	ORF1b:T1655I	1	0.17	ORF1b:V1110L	1	0.05
West Bengal				ORF1b:A975D	1	0.22	ORF1b:R1576K	1	0.17	ORF1b:V1840F	1	0.05
West Bengal				ORF1b:A1392V	1	0.22	ORF1b:D1130G	1	0.17	ORF1b:A1895T	1	0.05
West Bengal				ORF1b:A2110S	1	0.22	ORF1b:D2020Y	1	0.17	ORF1b:G1041C	1	0.05
West Bengal				ORF1b:V2365F	1	0.22	ORF1b:L1681F	1	0.17	ORF1b:T797I	1	0.05
West Bengal				ORF1b:D1183G	1	0.22	ORF1b:S904L	1	0.17	ORF1b:V839L	1	0.05
West Bengal				ORF1b:M897I	1	0.22	ORF1b:T2099I	1	0.17	ORF1b:T1354I	1	0.05
West Bengal				ORF1b:E1264D	1	0.22	ORF1b:A1428V	1	0.17	ORF1b:M1197I	1	0.05
West Bengal				ORF1b:P1095S	1	0.22	ORF1b:T2592I	1	0.17	ORF1b:A1033S	1	0.05
West Bengal				ORF1b:I192V	1	0.22	ORF1b:T1064I	1	0.17	ORF1b:I1674T	1	0.05
West Bengal				ORF1b:K2367N	1	0.22	ORF1b:L1423F	1	0.17	ORF1b:V364A	1	0.05
West Bengal				ORF1b:F274S	1	0.22	ORF1b:G2387S	1	0.17	ORF1b:M1693I	1	0.05
West Bengal				ORF1b:L2017F	1	0.22	ORF1b:A1386T	1	0.17	ORF1b:L1681F	1	0.05
West Bengal				ORF1b:R741H	1	0.22	ORF1b:N168S	1	0.17	ORF1b:V666I	1	0.05
West Bengal				ORF1b:A390T	1	0.22	ORF1b:M2269I	1	0.17	ORF1b:V2I	1	0.05
West Bengal				ORF1b:M1499I	1	0.22	ORF1b:T1050I	1	0.17	ORF1b:D144Y	1	0.05
West Bengal				ORF1b:N2695K	1	0.22	ORF1b:V248F	1	0.17	ORF1b:T1540I	1	0.05
West Bengal				ORF1b:P2169S	1	0.22	ORF1b:P1645S	1	0.17	ORF1b:T2548I	1	0.05
West Bengal				ORF1b:M1693I	1	0.22	ORF1b:A1225S	1	0.17	ORF1b:A625V	1	0.05
West Bengal				ORF1b:L1890F	1	0.22	ORF1b:V426I	1	0.17	ORF1b:L820F	1	0.05
West Bengal				ORF1b:F1891S	1	0.22	ORF1b:S2339F	1	0.17	ORF1b:P1342S	1	0.05
West Bengal				ORF1b:A1031S	1	0.22	ORF1b:T267A	1	0.17	ORF1b:S2673I	1	0.05
West Bengal				ORF1b:V1840F	1	0.22	ORF1b:T284I	1	0.17	ORF1b:Y987H	1	0.05
West Bengal				ORF1b:V415M	1	0.22	ORF1b:A1832V	1	0.17	ORF1b:M2414I	1	0.05
West Bengal				ORF1b:L1701F	1	0.22	ORF1b:S1974I	1	0.17	ORF1b:A1443V	1	0.05
West Bengal				ORF1b:P976L	1	0.22	ORF1b:L2382F	1	0.17	ORF1b:A1643V	1	0.05
West Bengal				ORF1b:T899I	1	0.22	ORF1b:T2432I	1	0.17	ORF1b:K1141R	1	0.05
West Bengal				ORF1b:P1325S	1	0.22	ORF1b:T2537I	1	0.17	ORF1b:N902S	1	0.05

**West Bengal**				**ORF1b:A2188T**	1	0.22	**ORF1b:K151N**	1	0.17	**ORF1b:I235T**	1	0.05
**West Bengal**				**ORF1b:A1525S**	1	0.22	**ORF1b:S1023G**	1	0.17	**ORF1b:P909L**	1	0.05
**West Bengal**				**ORF1b:L1681F**	1	0.22	**ORF1b:H1087Y**	1	0.17	**ORF1b:D1127Y**	1	0.05
**West Bengal**				**ORF1b:A2222S**	1	0.22	**ORF1b:L1701F**	1	0.17	**ORF1b:A302S**	1	0.05
**West Bengal**				**ORF1b:A520T**	1	0.22	**ORF1b:M1156I**	1	0.17	**ORF1b:D2576Y**	1	0.05
**West Bengal**				**ORF1b:P1000L**	1	0.22	**ORF1b:Q1878H**	1	0.17	**ORF1b:G99V**	1	0.05
**West Bengal**				**ORF1b:H1550Y**	1	0.22	**ORF1b:S2212I**	1	0.17	**ORF1b:I1987V**	1	0.05
**West Bengal**				**ORF1b:E1560K**	1	0.22	**ORF1b:H2466Y**	1	0.17	**ORF1b:P1223L**	1	0.05
**West Bengal**				**ORF1b:Q1669L**	1	0.22	**ORF1b:G2337V**	1	0.17	**ORF1b:T1173I**	1	0.05
**West Bengal**				**ORF1b:M1839I**	1	0.22	**ORF1b:S1273L**	1	0.17	**ORF1b:L1423F**	1	0.05
**West Bengal**				**ORF1b:L40I**	1	0.22	**ORF1b:P1851S**	1	0.17	**ORF1b:G170C**	1	0.05
**West Bengal**				**ORF1b:A2431V**	1	0.22	**ORF1b:I687L**	1	0.17	**ORF1b:G1583S**	1	0.05
**West Bengal**				**ORF1b:T1354I**	1	0.22	**ORF1b:P1682H**	1	0.17	**ORF1b:E2284G**	1	0.05
**West Bengal**				**ORF1b:A1369S**	1	0.22	**ORF1b:I257V**	1	0.17	**ORF1b:V1984L**	1	0.05
**West Bengal**				**ORF1b:D2351N**	1	0.22	**ORF1b:M659I**	1	0.17	**ORF1b:D2183Y**	1	0.05
**West Bengal**				**ORF1b:T2548I**	1	0.22	**ORF1b:G1638S**	1	0.17	**ORF1b:V225L**	1	0.05
**West Bengal**				**ORF1b:I2320T**	1	0.22	**ORF1b:K1383R**	1	0.17	**ORF1b:G328C**	1	0.05
**West Bengal**				**ORF1b:P1917S**	1	0.22	**ORF1b:A941V**	1	0.17	**ORF1b:P12S**	1	0.05
**West Bengal**				**ORF1b:H2583Y**	1	0.22	**ORF1b:T1540I**	1	0.17	**ORF1b:A1291S**	1	0.05
**West Bengal**				**ORF1b:V1811L**	1	0.22	**ORF1b:T1637I**	1	0.17	**ORF1b:I1257V**	1	0.05
**West Bengal**				**ORF1b:V2117L**	1	0.22	**ORF1b:T1545A**	1	0.17	**ORF1b:T1067I**	1	0.05
**West Bengal**				**ORF1b:M2381T**	1	0.22	**ORF1b:T17I**	1	0.17	**ORF1b:V1375L**	1	0.05
**West Bengal**				**ORF1b:K963R**	1	0.22	**ORF1b:D1848E**	1	0.17	**ORF1b:T903N**	1	0.05
**West Bengal**				**ORF1b:P1223L**	1	0.22	**ORF1b:A414V**	1	0.17	**ORF1b:D1042H**	1	0.05
**West Bengal**				**ORF1b:K1900R**	1	0.22	**ORF1b:P909L**	1	0.17	**ORF1b:V1806I**	1	0.05
**West Bengal**				**ORF1b:A927V**	1	0.22	**ORF1b:P1342S**	1	0.17	**ORF1b:N206D**	1	0.05
**West Bengal**				**ORF1b:E1167D**	1	0.22	**ORF1b:T2490M**	1	0.17	**ORF1b:A397V**	1	0.05
**West Bengal**				**ORF1b:L505F**	1	0.22	**ORF1b:T1555I**	1	0.17	**ORF1b:L2123I**	1	0.05
**West Bengal**				**ORF1b:I441T**	1	0.22	**ORF1b:K2579N**	1	0.17	**ORF1b:T76I**	1	0.05
**West Bengal**				**ORF1b:L2302M**	1	0.22	**ORF1b:A1884V**	1	0.17	**ORF1b:I1688V**	1	0.05
**West Bengal**				**ORF1b:M2462I**	1	0.22	**ORF1b:S2673I**	1	0.17	**ORF1b:A1624S**	1	0.05
**West Bengal**				**ORF1b:R1577K**	1	0.22	**ORF1b:R726G**	1	0.17	**ORF1b:T1064I**	1	0.05
**West Bengal**				**ORF1b:M1156I**	1	0.22	**ORF1b:V2206A**	1	0.17	**ORF1b:S316I**	1	0.05
**West Bengal**				**ORF1b:K709N**	1	0.22	**ORF1b:F1422Y**	1	0.17	**ORF1b:A2132T**	1	0.05
**West Bengal**				**ORF1b:V2368F**	1	0.22	**ORF1b:V2117L**	1	0.17	**ORF1b:I1604T**	1	0.05
**West Bengal**							**ORF1b:A517V**	1	0.17	**ORF1b:M101L**	1	0.05
**West Bengal**							**ORF1b:K2063N**	1	0.17	**ORF1b:A88V**	1	0.05
**West Bengal**							**ORF1b:K2160N**	1	0.17	**ORF1b:T1290I**	1	0.05
**West Bengal**							**ORF1b:D729G**	1	0.17	**ORF1b:D1389Y**	1	0.05
**West Bengal**										**ORF1b:D1758G**	1	0.05
**West Bengal**										**ORF1b:V2073L**	1	0.05
**West Bengal**										**ORF1b:A1843S**	1	0.05
**West Bengal**										**ORF1b:M371I**	1	0.05
**West Bengal**										**ORF1b:P1567S**	1	0.05
**West Bengal**										**ORF1b:V396F**	1	0.05
**West Bengal**										**ORF1b:F1635L**	1	0.05
**West Bengal**										**ORF1b:D729Y**	1	0.05
**West Bengal**										**ORF1b:V1271L**	1	0.05
**West Bengal**										**ORF1b:K1863N**	1	0.05
**West Bengal**										**ORF1b:S69P**	1	0.05
**West Bengal**										**ORF1b:Y819H**	1	0.05
**West Bengal**										**ORF1b:D708N**	1	0.05
**West Bengal**										**ORF1b:P2648L**	1	0.05
**West Bengal**										**ORF1b:E601D**	1	0.05
**West Bengal**										**ORF1b:P1967L**	1	0.05
**West Bengal**										**ORF1b:R1078C**	1	0.05
**West Bengal**										**ORF1b:W2383C**	1	0.05
**West Bengal**										**ORF1b:N482S**	1	0.05
**West Bengal**										**ORF1b:M1765I**	1	0.05
**West Bengal**										**ORF1b:V1708F**	1	0.05
**West Bengal**										**ORF1b:M1156V**	1	0.05
**West Bengal**										**ORF1b:P1851S**	1	0.05
**West Bengal**										**ORF1b:P1967S**	1	0.05
**West Bengal**										**ORF1b:Y1229C**	1	0.05
**West Bengal**										**ORF1b:D131Y**	1	0.05
**West Bengal**										**ORF1b:A1521V**	1	0.05
**West Bengal**										**ORF1b:Q813R**	1	0.05
**West Bengal**										**ORF1b:V1961F**	1	0.05
**West Bengal**										**ORF1b:K151N**	1	0.05
**West Bengal**										**ORF1b:S220I**	1	0.05
**West Bengal**										**ORF1b:I2147V**	1	0.05
**West Bengal**										**ORF1b:P2404S**	1	0.05
**West Bengal**										**ORF1b:P1936H**	1	0.05

**West Bengal**										**ORF1b:R726G**	1	0.05
**West Bengal**										**ORF1b:K1835N**	1	0.05
**West Bengal**										**ORF1b:T634I**	1	0.05
**West Bengal**										**ORF1b:M620I**	1	0.05
**West Bengal**										**ORF1b:S598I**	1	0.05
**West Bengal**										**ORF1b:E1242D**	1	0.05
**West Bengal**										**ORF1b:T2332I**	1	0.05
**West Bengal**										**ORF1b:A186T**	1	0.05
**West Bengal**										**ORF1b:F1955L**	1	0.05
**West Bengal**										**ORF1b:R188Q**	1	0.05
**West Bengal**										**ORF1b:G2297C**	1	0.05
**West Bengal**										**ORF1b:M2260T**	1	0.05
**West Bengal**										**ORF1b:T701I**	1	0.05
**West Bengal**										**ORF1b:H247Y**	1	0.05
**West Bengal**										**ORF1b:A414V**	1	0.05
**West Bengal**										**ORF1b:T917A**	1	0.05
**West Bengal**										**ORF1b:L966F**	1	0.05
**West Bengal**										**ORF1b:R1425H**	1	0.05
**West Bengal**										**ORF1b:M1800T**	1	0.05
**West Bengal**										**ORF1b:R1687C**	1	0.05
**West Bengal**										**ORF1b:T573N**	1	0.05
**West Bengal**										**ORF1b:A434V**	1	0.05
**West Bengal**										**ORF1b:P1727H**	1	0.05
**West Bengal**										**ORF1b:I2158V**	1	0.05
**West Bengal**										**ORF1b:P85S**	1	0.05
**West Bengal**										**ORF1b:P2531S**	1	0.05
**West Bengal**										**ORF1b:V464I**	1	0.05
**West Bengal**										**ORF1b:G2201D**	1	0.05
**West Bengal**										**ORF1b:C473S**	1	0.05
**West Bengal**										**ORF1b:E2014D**	1	0.05
**West Bengal**										**ORF1b:I2262V**	1	0.05
**West Bengal**										**ORF1b:I2465V**	1	0.05
**West Bengal**										**ORF1b:Q1878H**	1	0.05
**West Bengal**										**ORF1b:E1728G**	1	0.05
**West Bengal**										**ORF1b:V1705I**	1	0.05
**West Bengal**										**ORF1b:V119I**	1	0.05
**West Bengal**										**ORF1b:P1452L**	1	0.05
**West Bengal**										**ORF1b:V2233F**	1	0.05
**West Bengal**										**ORF1b:M1765T**	1	0.05
**West Bengal**										**ORF1b:A1525S**	1	0.05
**West Bengal**										**ORF1b:D2386Y**	1	0.05
**West Bengal**										**ORF1b:Y280C**	1	0.05
**West Bengal**										**ORF1b:A176V**	1	0.05
**West Bengal**										**ORF1b:C1880Y**	1	0.05
**West Bengal**										**ORF1b:V2082F**	1	0.05
**West Bengal**										**ORF1b:P1879S**	1	0.05
**West Bengal**										**ORF1b:S1273L**	1	0.05
**West Bengal**										**ORF1b:G2201V**	1	0.05
**West Bengal**										**ORF1b:V1649I**	1	0.05
**West Bengal**										**ORF1b:I1448V**	1	0.05
**West Bengal**										**ORF1b:S997L**	1	0.05
**West Bengal**										**ORF1b:A190V**	1	0.05
**West Bengal**										**ORF1b:H2388Y**	1	0.05
**West Bengal**										**ORF1b:D1899G**	1	0.05
**West Bengal**										**ORF1b:T42P**	1	0.05
**West Bengal**										**ORF1b:T335I**	1	0.05
**West Bengal**										**ORF1b:T2099I**	1	0.05
**West Bengal**										**ORF1b:V2287I**	1	0.05
**West Bengal**										**ORF1b:I2303V**	1	0.05
**West Bengal**										**ORF1b:M2417I**	1	0.05
**West Bengal**										**ORF1b:D2541A**	1	0.05
**West Bengal**										**ORF1b:A1392V**	1	0.05
**West Bengal**										**ORF1b:Q1546H**	1	0.05
**West Bengal**										**ORF1b:R2684S**	1	0.05
**West Bengal**										**ORF1b:V1913I**	1	0.05
**West Bengal**										**ORF1b:A1805S**	1	0.05
**West Bengal**										**ORF1b:L722F**	1	0.05
**West Bengal**										**ORF1b:E1288D**	1	0.05
**West Bengal**										**ORF1b:A176S**	1	0.05
**West Bengal**										**ORF1b:K1571N**	1	0.05
**West Bengal**										**ORF1b:M592I**	1	0.05
**West Bengal**										**ORF1b:A1521S**	1	0.05
**West Bengal**										**ORF1b:Q2484H**	1	0.05

West Bengal										ORF1b:V1649F	1	0.05
West Bengal										ORF1b:P2434S	1	0.05
West Bengal										ORF1b:H1807R	1	0.05
West Bengal										ORF1b:A1476S	1	0.05
West Bengal										ORF1b:P2256S	1	0.05
West Bengal										ORF1b:S442R	1	0.05
West Bengal										ORF1b:R2613S	1	0.05
West Bengal										ORF1b:A1225S	1	0.05
West Bengal										ORF1b:D152Y	1	0.05
West Bengal										ORF1b:A572T	1	0.05
West Bengal										ORF1b:R164C	1	0.05
West Bengal										ORF1b:M101V	1	0.05
West Bengal										ORF1b:L2350I	1	0.05
West Bengal										ORF1b:N1047I	1	0.05
West Bengal										ORF1b:P528S	1	0.05
West Bengal										ORF1b:D100G	1	0.05
West Bengal										ORF1b:A2585T	1	0.05
West Bengal										ORF1b:G2337S	1	0.05
West Bengal										ORF1b:T217A	1	0.05
West Bengal										ORF1b:A1190V	1	0.05
West Bengal										ORF1b:A1895V	1	0.05
West Bengal										ORF1b:M657V	1	0.05
West Bengal										ORF1b:I2320V	1	0.05
West Bengal										ORF1b:M1581I	1	0.05
West Bengal										ORF1b:V2591A	1	0.05
West Bengal										ORF1b:G2201C	1	0.05
West Bengal										ORF1b:A7V	1	0.05
West Bengal										ORF1b:K1447N	1	0.05
West Bengal										ORF1b:M187I	1	0.05
West Bengal										ORF1b:G814C	1	0.05
West Bengal										ORF1b:Q348H	1	0.05
West Bengal										ORF1b:M1156I	1	0.05
West Bengal										ORF1b:A2513S	1	0.05
West Bengal										ORF1b:D1746Y	1	0.05
West Bengal										ORF1b:S1795N	1	0.05
West Bengal										ORF1b:H1838Y	1	0.05
West Bengal										ORF1b:K82E	1	0.05
West Bengal										ORF1b:I570M	1	0.05
West Bengal										ORF1b:E1728D	1	0.05
West Bengal										ORF1b:K1828N	1	0.05
	Spike (S)											
West Bengal	Pre-Wave 1			Wave 1			Pre-Wave 2			Wave 2		
West Bengal	Mutation	Count	percent	Mutation	Count	percent	Mutation	Count	percent	Mutation	Count	percent
West Bengal	S:D614G	164	85.42	S:D614G	457	99.13	S:D614G	593	99.33	S:D614G	1939	99.54
West Bengal	S:G261S	16	8.33	S:P681H	50	10.85	S:E484K	175	29.31	S:P681R	1807	92.76
West Bengal	S:H49Y	10	5.21	S:V1230L	47	10.2	S:P681H	143	23.95	S:L452R	1791	91.94
West Bengal	S:V213L	3	1.56	S:Q677H	24	5.21	S:P681R	93	15.58	S:D950N	1295	66.48
West Bengal	S:G1124V	2	1.04	S:N440K	19	4.12	S:L452R	87	14.57	S:T19R	1281	65.76
West Bengal	S:G75A	2	1.04	S:H49Y	14	3.04	S:N501Y	84	14.07	S:T478K	1268	65.09
West Bengal	S:T323I	2	1.04	S:L5F	11	2.39	S:E484Q	80	13.4	S:R158G	1223	62.78
West Bengal	S:L54F	2	1.04	S:Q675H	10	2.17	S:Q1071H	80	13.4	S:G142D	663	34.03
West Bengal	S:P330S	2	1.04	S:G261S	9	1.95	S:E154K	79	13.23	S:T95I	553	28.39
West Bengal	S:T20A	2	1.04	S:H66R	9	1.95	S:G142D	77	12.9	S:E484Q	521	26.75
West Bengal	S:K558N	2	1.04	S:E484K	8	1.74	S:D1118H	70	11.73	S:Q1071H	509	26.13
West Bengal	S:T723I	1	0.52	S:S943P	7	1.52	S:T95I	70	11.73	S:E154K	483	24.79
West Bengal	S:T941K	1	0.52	S:D138H	7	1.52	S:A570D	69	11.56	S:A222V	130	6.67
West Bengal	S:E309Q	1	0.52	S:V1228L	7	1.52	S:T716I	69	11.56	S:N501Y	88	4.52
West Bengal	S:E1207*	1	0.52	S:D80Y	6	1.3	S:S982A	69	11.56	S:V3G	86	4.41
West Bengal	S:A871S	1	0.52	S:D178H	6	1.3	S:H49Y	66	11.06	S:P681H	78	4
West Bengal	S:A1078S	1	0.52	S:T19I	6	1.3	S:V1230L	65	10.89	S:A570D	74	3.8
West Bengal	S:M153I	1	0.52	S:A845S	5	1.08	S:N440K	51	8.54	S:T716I	74	3.8
West Bengal	S:G769V	1	0.52	S:V213L	5	1.08	S:Q677H	28	4.69	S:S982A	74	3.8
West Bengal	S:D796H	1	0.52	S:Q613H	5	1.08	S:L18F	23	3.85	S:D1118H	74	3.8
West Bengal	S:L5F	1	0.52	S:E702Q	5	1.08	S:V3G	20	3.35	S:E484K	47	2.41
West Bengal	S:T1231A	1	0.52	S:A701V	5	1.08	S:Q675H	16	2.68	S:H1101D	39	2
West Bengal	S:A1070S	1	0.52	S:G769V	4	0.87	S:L5F	14	2.35	S:L5F	19	0.98
West Bengal	S:G142S	1	0.52	S:A688V	4	0.87	S:A27S	12	2.01	S:V1104L	19	0.98
West Bengal	S:P26H	1	0.52	S:M153I	4	0.87	S:A701V	12	2.01	S:H49Y	16	0.82
West Bengal	S:A845S	1	0.52	S:Q23R	4	0.87	S:D80A	10	1.68	S:A701V	14	0.72
West Bengal	S:D1163Y	1	0.52	S:A1070S	4	0.87	S:D215G	10	1.68	S:Q677H	13	0.67

**West Bengal**	**S:E1262***	1	0.52	**S:S477N**	4	0.87	**S:K417N**	10	1.68	**S:D80A**	13	0.67
**West Bengal**	**S:D574Y**	1	0.52	**S:L18F**	3	0.65	**S:D138H**	8	1.34	**S:D215G**	13	0.67
**West Bengal**	**S:Q787H**	1	0.52	**S:T95I**	3	0.65	**S:A67V**	8	1.34	**S:K417N**	13	0.67
**West Bengal**	**S:S967G**	1	0.52	**S:K854N**	3	0.65	**S:D950N**	8	1.34	**S:Q613H**	13	0.67
**West Bengal**	**S:D215Y**	1	0.52	**S:A845V**	3	0.65	**S:H1101D**	8	1.34	**S:L18F**	12	0.62
**West Bengal**				**S:V213G**	3	0.65	**S:S94F**	7	1.17	**S:Q218H**	11	0.56
**West Bengal**				**S:F490S**	3	0.65	**S:T19R**	7	1.17	**S:L1141W**	11	0.56
**West Bengal**				**S:D936H**	3	0.65	**S:T478K**	7	1.17	**S:A879S**	9	0.46
**West Bengal**				**S:T1117I**	3	0.65	**S:E180K**	6	1.01	**S:K77T**	9	0.46
**West Bengal**				**S:L176F**	3	0.65	**S:D138Y**	6	1.01	**S:M177V**	8	0.41
**West Bengal**				**S:A262S**	3	0.65	**S:V382L**	6	1.01	**S:A67V**	8	0.41
**West Bengal**				**S:W152L**	3	0.65	**S:P26S**	6	1.01	**S:Q52R**	7	0.36
**West Bengal**				**S:W64L**	3	0.65	**S:Q52R**	6	1.01	**S:D178N**	7	0.36
**West Bengal**				**S:L452Q**	3	0.65	**S:R158G**	6	1.01	**S:S255F**	7	0.36
**West Bengal**				**S:D138Y**	3	0.65	**S:A688V**	5	0.84	**S:N440K**	6	0.31
**West Bengal**				**S:G1167V**	2	0.43	**S:Q23R**	5	0.84	**S:M153I**	6	0.31
**West Bengal**				**S:W258L**	2	0.43	**S:L176F**	5	0.84	**S:S98F**	6	0.31
**West Bengal**				**S:A771S**	2	0.43	**S:S494P**	4	0.67	**S:W64R**	6	0.31
**West Bengal**				**S:D796H**	2	0.43	**S:V1176F**	4	0.67	**S:F888L**	5	0.26
**West Bengal**				**S:S943T**	2	0.43	**S:H1101Y**	4	0.67	**S:H655Y**	5	0.26
**West Bengal**				**S:T547I**	2	0.43	**S:D1153Y**	4	0.67	**S:I712V**	5	0.26
**West Bengal**				**S:G1219C**	2	0.43	**S:G142L**	4	0.67	**S:A845S**	5	0.26
**West Bengal**				**S:L54F**	2	0.43	**S:Y144F**	4	0.67	**S:G566A**	5	0.26
**West Bengal**				**S:V1104L**	2	0.43	**S:Y449H**	4	0.67	**S:V1230L**	4	0.21
**West Bengal**				**S:H1058Y**	2	0.43	**S:F565L**	4	0.67	**S:V1264L**	4	0.21
**West Bengal**				**S:E180K**	2	0.43	**S:H655Y**	4	0.67	**S:S12F**	4	0.21
**West Bengal**				**S:R102I**	2	0.43	**S:F888L**	4	0.67	**S:A688V**	4	0.21
**West Bengal**				**S:K558N**	2	0.43	**S:N439K**	4	0.67	**S:N211S**	4	0.21
**West Bengal**				**S:T859I**	2	0.43	**S:T1231A**	4	0.67	**S:G1219V**	4	0.21
**West Bengal**				**S:A222V**	2	0.43	**S:T1117I**	3	0.5	**S:G181V**	4	0.21
**West Bengal**				**S:G184V**	2	0.43	**S:A520S**	3	0.5	**S:K1191N**	4	0.21
**West Bengal**				**S:V1104I**	2	0.43	**S:N679K**	3	0.5	**S:A1078S**	4	0.21
**West Bengal**				**S:V1264L**	2	0.43	**S:A846V**	3	0.5	**S:V1228L**	4	0.21
**West Bengal**				**S:A879S**	2	0.43	**S:T859I**	3	0.5	**S:D574Y**	4	0.21
**West Bengal**				**S:P26L**	2	0.43	**S:T29I**	3	0.5	**S:G75V**	4	0.21
**West Bengal**				**S:T20I**	1	0.22	**S:Q613H**	3	0.5	**S:V1176F**	4	0.21
**West Bengal**				**S:V367F**	1	0.22	**S:R102S**	3	0.5	**S:G446V**	4	0.21
**West Bengal**				**S:G446V**	1	0.22	**S:D574Y**	3	0.5	**S:I818V**	4	0.21
**West Bengal**				**S:L517F**	1	0.22	**S:A852S**	2	0.34	**S:E1072K**	3	0.15
**West Bengal**				**S:V1040F**	1	0.22	**S:I1169L**	2	0.34	**S:A27S**	3	0.15
**West Bengal**				**S:C1243F**	1	0.22	**S:F490S**	2	0.34	**S:A520S**	3	0.15
**West Bengal**				**S:E180D**	1	0.22	**S:P812L**	2	0.34	**S:S256L**	3	0.15
**West Bengal**				**S:L922F**	1	0.22	**S:N481D**	2	0.34	**S:T678I**	3	0.15
**West Bengal**				**S:G842S**	1	0.22	**S:L1200F**	2	0.34	**S:T250A**	3	0.15
**West Bengal**				**S:S939F**	1	0.22	**S:V1228L**	2	0.34	**S:V445A**	3	0.15
**West Bengal**				**S:S813I**	1	0.22	**S:Q173H**	2	0.34	**S:D1153Y**	3	0.15
**West Bengal**				**S:I210V**	1	0.22	**S:W258S**	2	0.34	**S:V367L**	3	0.15
**West Bengal**				**S:A871S**	1	0.22	**S:A262S**	2	0.34	**S:W152L**	3	0.15
**West Bengal**				**S:E1111G**	1	0.22	**S:V367L**	2	0.34	**S:P1263L**	3	0.15
**West Bengal**				**S:D1163Y**	1	0.22	**S:W152L**	2	0.34	**S:T573I**	3	0.15
**West Bengal**				**S:A1020S**	1	0.22	**S:M1229I**	2	0.34	**S:T572I**	3	0.15
**West Bengal**				**S:L1265F**	1	0.22	**S:S256L**	2	0.34	**S:C1235F**	3	0.15
**West Bengal**				**S:S13I**	1	0.22	**S:E780Q**	2	0.34	**S:S929T**	3	0.15
**West Bengal**				**S:S256P**	1	0.22	**S:K1181N**	2	0.34	**S:S680F**	3	0.15
**West Bengal**				**S:P1112L**	1	0.22	**S:D1146E**	2	0.34	**S:E583D**	3	0.15
**West Bengal**				**S:V595I**	1	0.22	**S:M153I**	2	0.34	**S:L455F**	3	0.15
**West Bengal**				**S:H1101Y**	1	0.22	**S:S810L**	2	0.34	**S:C1247F**	3	0.15
**West Bengal**				**S:G261R**	1	0.22	**S:A879S**	2	0.34	**S:C1243S**	2	0.1
**West Bengal**				**S:L335F**	1	0.22	**S:K811I**	2	0.34	**S:T299I**	2	0.1
**West Bengal**				**S:N679K**	1	0.22	**S:A222V**	2	0.34	**S:T307I**	2	0.1
**West Bengal**				**S:N148T**	1	0.22	**S:G261V**	2	0.34	**S:S155I**	2	0.1
**West Bengal**				**S:A520S**	1	0.22	**S:M153V**	2	0.34	**S:G142L**	2	0.1
**West Bengal**				**S:P809S**	1	0.22	**S:E1202Q**	2	0.34	**S:Y144F**	2	0.1
**West Bengal**				**S:M1237I**	1	0.22	**S:D80Y**	2	0.34	**S:Y449H**	2	0.1
**West Bengal**				**S:D1259Y**	1	0.22	**S:N137K**	2	0.34	**S:F565L**	2	0.1
**West Bengal**				**S:E309Q**	1	0.22	**S:H69Y**	2	0.34	**S:K147N**	2	0.1
**West Bengal**				**S:A263G**	1	0.22	**S:N679Y**	2	0.34	**S:Y145D**	2	0.1
**West Bengal**				**S:G1251V**	1	0.22	**S:L54F**	2	0.34	**S:T572S**	2	0.1
**West Bengal**				**S:I197V**	1	0.22	**S:F157S**	2	0.34	**S:R21T**	2	0.1
**West Bengal**				**S:V1228I**	1	0.22	**S:V1040F**	2	0.34	**S:P812S**	2	0.1
**West Bengal**				**S:A352S**	1	0.22	**S:T22I**	2	0.34	**S:T827K**	2	0.1
**West Bengal**				**S:I850V**	1	0.22	**S:V622F**	2	0.34	**S:T747N**	2	0.1
**West Bengal**				**S:L1063F**	1	0.22	**S:V130F**	2	0.34	**S:F186L**	2	0.1

West Bengal				S:S155I	1	0.22	S:G75V	1	0.17	S:V785I	2	0.1
West Bengal				S:G257S	1	0.22	S:H66R	1	0.17	S:A243S	2	0.1
West Bengal				S:V382L	1	0.22	S:F157L	1	0.17	S:W258L	2	0.1
West Bengal				S:P337T	1	0.22	S:D839Y	1	0.17	S:W258R	2	0.1
West Bengal				S:S13G	1	0.22	S:A575S	1	0.17	S:V705F	2	0.1
West Bengal				S:G181A	1	0.22	S:A829V	1	0.17	S:T20N	2	0.1
West Bengal				S:A243V	1	0.22	S:S477N	1	0.17	S:A684S	2	0.1
West Bengal				S:D1118Y	1	0.22	S:D936H	1	0.17	S:G476S	2	0.1
West Bengal				S:A846V	1	0.22	S:K814R	1	0.17	S:R1091L	2	0.1
West Bengal				S:V952F	1	0.22	S:G252D	1	0.17	S:V16F	2	0.1
West Bengal				S:I1130M	1	0.22	S:A783V	1	0.17	S:E1150D	2	0.1
West Bengal				S:T478I	1	0.22	S:Q675R	1	0.17	S:D215Y	2	0.1
West Bengal				S:L1244F	1	0.22	S:G769V	1	0.17	S:R765C	2	0.1
West Bengal				S:V6F	1	0.22	S:E1144Q	1	0.17	S:A1087S	2	0.1
West Bengal				S:T618A	1	0.22	S:E702Q	1	0.17	S:V705I	2	0.1
West Bengal				S:F157L	1	0.22	S:R214L	1	0.17	S:M1229I	2	0.1
West Bengal				S:A27V	1	0.22	S:S939F	1	0.17	S:D830G	2	0.1
West Bengal				S:R78M	1	0.22	S:N1023S	1	0.17	S:V622F	2	0.1
West Bengal				S:T1231A	1	0.22	S:G1267V	1	0.17	S:Y508H	2	0.1
West Bengal				S:G252D	1	0.22	S:M153T	1	0.17	S:L229F	2	0.1
West Bengal				S:N148S	1	0.22	S:G257S	1	0.17	S:P1162L	2	0.1
West Bengal				S:A684T	1	0.22	S:E583D	1	0.17	S:T732I	2	0.1
West Bengal				S:R408I	1	0.22	S:T20I	1	0.17	S:I834V	2	0.1
West Bengal				S:I834S	1	0.22	S:G181E	1	0.17	S:N148T	2	0.1
West Bengal				S:S98F	1	0.22	S:T76I	1	0.17	S:S1242I	2	0.1
West Bengal				S:P174S	1	0.22	S:V159I	1	0.17	S:A713S	2	0.1
West Bengal				S:T259I	1	0.22	S:T299I	1	0.17	S:C1243F	2	0.1
West Bengal				S:G261C	1	0.22	S:S255F	1	0.17	S:P793L	2	0.1
West Bengal				S:G1219A	1	0.22	S:S205A	1	0.17	S:L18R	2	0.1
West Bengal				S:E154G	1	0.22	S:H1083Q	1	0.17	S:T76I	1	0.05
West Bengal				S:D253G	1	0.22	S:L212I	1	0.17	S:Q1208H	1	0.05
West Bengal							S:D796E	1	0.17	S:I909V	1	0.05
West Bengal							S:P812A	1	0.17	S:D215H	1	0.05
West Bengal							S:Q1113K	1	0.17	S:G142S	1	0.05
West Bengal							S:G181A	1	0.17	S:F543L	1	0.05
West Bengal							S:N641S	1	0.17	S:N164I	1	0.05
West Bengal							S:N658D	1	0.17	S:D253Y	1	0.05
West Bengal							S:P272S	1	0.17	S:L822F	1	0.05
West Bengal							S:A623V	1	0.17	S:V382L	1	0.05
West Bengal							S:W64C	1	0.17	S:T333R	1	0.05
West Bengal							S:S98F	1	0.17	S:C1247Y	1	0.05
West Bengal							S:M1237I	1	0.17	S:P384S	1	0.05
West Bengal							S:S13I	1	0.17	S:V615F	1	0.05
West Bengal							S:Y1007H	1	0.17	S:G413V	1	0.05
West Bengal							S:P499L	1	0.17	S:A892V	1	0.05
West Bengal							S:A845V	1	0.17	S:P1162S	1	0.05
West Bengal							S:S698L	1	0.17	S:P793S	1	0.05
West Bengal							S:Q779E	1	0.17	S:Q474H	1	0.05
West Bengal							S:A522S	1	0.17	S:V143D	1	0.05
West Bengal							S:V6A	1	0.17	S:Y144D	1	0.05
West Bengal							S:Q913H	1	0.17	S:A222S	1	0.05
West Bengal							S:I651V	1	0.17	S:L922F	1	0.05
West Bengal							S:D936Y	1	0.17	S:D253N	1	0.05
West Bengal							S:T678I	1	0.17	S:R683L	1	0.05
West Bengal							S:A352S	1	0.17	S:S1252F	1	0.05
West Bengal							S:A67S	1	0.17	S:L24S	1	0.05
West Bengal							S:V1264L	1	0.17	S:R1107S	1	0.05
West Bengal							S:K1191N	1	0.17	S:A262S	1	0.05
West Bengal							S:G181V	1	0.17	S:R847T	1	0.05
West Bengal							S:P330S	1	0.17	S:G594V	1	0.05
West Bengal							S:Q218H	1	0.17	S:V16A	1	0.05
West Bengal							S:V213L	1	0.17	S:Q675H	1	0.05
West Bengal							S:A243T	1	0.17	S:T20I	1	0.05
West Bengal							S:P9L	1	0.17	S:D253G	1	0.05
West Bengal							S:A845S	1	0.17	S:R21I	1	0.05
West Bengal							S:V1122L	1	0.17	S:A958S	1	0.05
West Bengal							S:D215A	1	0.17	S:A522S	1	0.05
West Bengal							S:M1229T	1	0.17	S:V826L	1	0.05
West Bengal							S:C1243S	1	0.17	S:F186S	1	0.05
West Bengal										S:S1242T	1	0.05
West Bengal										S:G1167V	1	0.05
West Bengal										S:S1147L	1	0.05
West Bengal										S:G798S	1	0.05

**West Bengal**										**S:L54F**	1	0.05
**West Bengal**										**S:V687L**	1	0.05
**West Bengal**										**S:D253A**	1	0.05
**West Bengal**										**S:G75S**	1	0.05
**West Bengal**										**S:A1070S**	1	0.05
**West Bengal**										**S:V367I**	1	0.05
**West Bengal**										**S:D1184Y**	1	0.05
**West Bengal**										**S:P174S**	1	0.05
**West Bengal**										**S:K1149R**	1	0.05
**West Bengal**										**S:I870V**	1	0.05
**West Bengal**										**S:Q173H**	1	0.05
**West Bengal**										**S:V1122M**	1	0.05
**West Bengal**										**S:R765L**	1	0.05
**West Bengal**										**S:R683W**	1	0.05
**West Bengal**										**S:S221L**	1	0.05
**West Bengal**										**S:A67S**	1	0.05
**West Bengal**										**S:A831V**	1	0.05
**West Bengal**										**S:V1177L**	1	0.05
**West Bengal**										**S:I1169F**	1	0.05
**West Bengal**										**S:G446S**	1	0.05
**West Bengal**										**S:R683Q**	1	0.05
**West Bengal**										**S:E1202Q**	1	0.05
**West Bengal**										**S:T547A**	1	0.05
**West Bengal**										**S:W104L**	1	0.05
**West Bengal**										**S:S151I**	1	0.05
**West Bengal**										**S:D1084H**	1	0.05
**West Bengal**										**S:A372V**	1	0.05
**West Bengal**										**S:L1203F**	1	0.05
**West Bengal**										**S:W64C**	1	0.05
**West Bengal**										**S:V36F**	1	0.05
**West Bengal**										**S:A522V**	1	0.05
**West Bengal**										**S:T859N**	1	0.05
**West Bengal**										**S:H245R**	1	0.05
**West Bengal**										**S:R246I**	1	0.05
**West Bengal**										**S:R567I**	1	0.05
**West Bengal**										**S:G1124V**	1	0.05
**West Bengal**										**S:K1157E**	1	0.05
**West Bengal**										**S:V785G**	1	0.05
**West Bengal**										**S:D111H**	1	0.05
**West Bengal**										**S:P463S**	1	0.05
**West Bengal**										**S:R646H**	1	0.05
**West Bengal**										**S:A852V**	1	0.05
**West Bengal**										**S:A570S**	1	0.05
**West Bengal**										**S:M1229L**	1	0.05
**West Bengal**										**S:Q14H**	1	0.05
**West Bengal**										**S:S514F**	1	0.05
**West Bengal**										**S:G413R**	1	0.05
**West Bengal**										**S:K1073N**	1	0.05
**West Bengal**										**S:S940F**	1	0.05
**West Bengal**										**S:D1163G**	1	0.05
**West Bengal**										**S:P1112L**	1	0.05
**West Bengal**										**S:D198Y**	1	0.05
**West Bengal**										**S:S698L**	1	0.05
**West Bengal**										**S:Q1201R**	1	0.05
**West Bengal**										**S:P681C**	1	0.05
**West Bengal**										**S:T1120I**	1	0.05
**West Bengal**										**S:R214H**	1	0.05
**West Bengal**										**S:K529E**	1	0.05
**West Bengal**										**S:A575G**	1	0.05
**West Bengal**										**S:S673T**	1	0.05
**West Bengal**										**S:N717S**	1	0.05
**West Bengal**										**S:A1020S**	1	0.05
**West Bengal**										**S:T167I**	1	0.05
**West Bengal**										**S:W258C**	1	0.05
**West Bengal**										**S:V6I**	1	0.05
**West Bengal**										**S:C1248G**	1	0.05
**West Bengal**										**S:S349A**	1	0.05
**West Bengal**										**S:E654V**	1	0.05
**West Bengal**										**S:N658Y**	1	0.05
**West Bengal**										**S:L179F**	1	0.05
**West Bengal**										**S:I1114L**	1	0.05
**West Bengal**										**S:G842D**	1	0.05
**West Bengal**										**S:T1066N**	1	0.05

West Bengal										S:T747I	1	0.05
West Bengal										S:I1130L	1	0.05
West Bengal										S:I1130M	1	0.05
West Bengal										S:S172Y	1	0.05
West Bengal										S:S477G	1	0.05
West Bengal										S:D1259Y	1	0.05
West Bengal										S:V362F	1	0.05
West Bengal										S:V445I	1	0.05
West Bengal										S:K558N	1	0.05
West Bengal										S:D80Y	1	0.05
West Bengal										S:M1237I	1	0.05
West Bengal										S:P82S	1	0.05
West Bengal										S:D1163Y	1	0.05
West Bengal										S:V213A	1	0.05
West Bengal										S:S494P	1	0.05
West Bengal										S:S477I	1	0.05
West Bengal										S:T19I	1	0.05
West Bengal										S:D936Y	1	0.05
West Bengal										S:S162R	1	0.05
West Bengal										S:N185K	1	0.05
West Bengal										S:K458N	1	0.05
West Bengal										S:A344S	1	0.05
West Bengal										S:S494L	1	0.05
West Bengal										S:I472V	1	0.05
West Bengal										S:E516Q	1	0.05
West Bengal										S:T95N	1	0.05
West Bengal										S:A899S	1	0.05
West Bengal										S:H146Q	1	0.05
West Bengal										S:R214L	1	0.05
West Bengal										S:T547I	1	0.05
West Bengal										S:G485R	1	0.05
West Bengal										S:H1101Y	1	0.05
West Bengal										S:P322S	1	0.05
West Bengal										S:A1070V	1	0.05
West Bengal												
West Bengal												
	Envelope (E)											
West Bengal	Pre-Wave 1			Wave 1			Pre-Wave 2			Wave 2		
West Bengal	Mutation	Count	percent	Mutation	Count	percent	Mutation	Count	percent	Mutation	Count	percent
West Bengal	E:S68F	3	1.56	E:L73F	2	0.43	E:P71L	10	1.68	E:V62F	16	0.82
West Bengal	E:P71L	2	1.04	E:C43F	1	0.22	E:L73F	6	1.01	E:P71L	13	0.67
West Bengal	E:P71S	1	0.52	E:P71S	1	0.22	E:L21F	4	0.67	E:T9I	7	0.36
West Bengal				E:V62F	1	0.22	E:S68F	3	0.5	E:L21F	6	0.31
West Bengal				E:D72A	1	0.22	E:V49L	3	0.5	E:L37F	2	0.1
West Bengal				E:K53R	1	0.22	E:V62F	2	0.34	E:V49L	2	0.1
West Bengal				E:S68F	1	0.22	E:S60T	2	0.34	E:S55F	2	0.1
West Bengal				E:T9I	1	0.22	E:A22V	1	0.17	E:V14I	1	0.05
West Bengal				E:V24M	1	0.22	E:L27F	1	0.17	E:R61L	1	0.05
West Bengal				E:S60T	1	0.22	E:L31Q	1	0.17	E:S68P	1	0.05
West Bengal							E:V14I	1	0.17	E:V24L	1	0.05
West Bengal										E:G10C	1	0.05
West Bengal										E:S68F	1	0.05
West Bengal										E:L73F	1	0.05
	Membrane (M)											
West Bengal	Pre-Wave 1			Wave 1			Pre-Wave 2			Wave 2		
West Bengal	Mutation	Count	percent	Mutation	Count	percent	Mutation	Count	percent	Mutation	Count	percent
West Bengal	M:V70F	4	2.08	M:H125Y	4	0.87	M:I82S	72	12.06	M:I82T	1299	66.68
West Bengal	M:K162N	1	0.52	M:D209Y	1	0.22	M:I82T	12	2.01	M:I82S	471	24.18
West Bengal	M:Q185H	1	0.52	M:A104V	1	0.22	M:F28L	3	0.5	M:L124F	6	0.31
West Bengal	M:I76V	1	0.52	M:I128T	1	0.22	M:I80V	3	0.5	M:A85V	6	0.31
West Bengal				M:H155Y	1	0.22	M:I49V	2	0.34	M:A2V	6	0.31
West Bengal				M:S4F	1	0.22	M:T208I	2	0.34	M:S214I	5	0.26
West Bengal							M:S214I	2	0.34	M:F28L	3	0.15
West Bengal							M:H155Y	2	0.34	M:V70F	3	0.15
West Bengal							M:I144V	2	0.34	M:D209N	3	0.15
West Bengal							M:A85S	1	0.17	M:H125Y	2	0.1

**West Bengal**							**M:A98S**	1	0.17	**M:R146H**	2	0.1
**West Bengal**							**M:H125Y**	1	0.17	**M:L29F**	2	0.1
**West Bengal**							**M:G78C**	1	0.17	**M:Q19R**	2	0.1
**West Bengal**							**M:L124F**	1	0.17	**M:T127S**	2	0.1
**West Bengal**										**M:L17F**	2	0.1
**West Bengal**										**M:G78C**	1	0.05
**West Bengal**										**M:I82F**	1	0.05
**West Bengal**										**M:L57S**	1	0.05
**West Bengal**										**M:A2S**	1	0.05
**West Bengal**										**M:E12D**	1	0.05
**West Bengal**										**M:G89S**	1	0.05
**West Bengal**										**M:L124I**	1	0.05
**West Bengal**										**M:N207S**	1	0.05
**West Bengal**										**M:M109I**	1	0.05
**West Bengal**										**M:Q185H**	1	0.05
**West Bengal**										**M:D209Y**	1	0.05
**West Bengal**										**M:V10A**	1	0.05
**West Bengal**										**M:A85S**	1	0.05
**West Bengal**										**M:A142V**	1	0.05
**West Bengal**										**M:D3N**	1	0.05
**West Bengal**										**M:E167Q**	1	0.05
**West Bengal**										**M:K162N**	1	0.05
**West Bengal**										**M:I49V**	1	0.05
**West Bengal**										**M:T30I**	1	0.05
**West Bengal**												
	**Nucleocapsid (N)**											
**West Bengal**	**Pre-Wave 1**			**Wave 1**			**Pre-Wave 2**			**Wave 2**		
**West Bengal**	**Mutation**	**Count**	**percent**	**Mutation**	**Count**	**percent**	**Mutation**	**Count**	**percent**	**Mutation**	**Count**	**percent**
**West Bengal**	**N:R203K**	16	8.33	**N:R203K**	178	38.61	**N:G204R**	264	44.22	**N:R203M**	1812	93.02
**West Bengal**	**N:G204R**	16	8.33	**N:G204R**	178	38.61	**N:R203K**	262	43.89	**N:D377Y**	1746	89.63
**West Bengal**	**N:S202N**	16	8.33	**N:S194L**	107	23.21	**N:M234I**	124	20.77	**N:D63G**	1292	66.32
**West Bengal**	**N:P13L**	11	5.73	**N:T205I**	33	7.16	**N:A119S**	123	20.6	**N:R385K**	742	38.09
**West Bengal**	**N:D401Y**	4	2.08	**N:R209I**	21	4.56	**N:A217S**	123	20.6	**N:T362I**	623	31.98
**West Bengal**	**N:S194L**	3	1.56	**N:D3Y**	20	4.34	**N:G18S**	120	20.1	**N:G215C**	297	15.25
**West Bengal**	**N:S37L**	1	0.52	**N:S235F**	15	3.25	**N:E367Q**	118	19.77	**N:R203K**	79	4.06
**West Bengal**	**N:P279S**	1	0.52	**N:A217S**	11	2.39	**N:R203M**	86	14.41	**N:G204R**	79	4.06
**West Bengal**	**N:A359S**	1	0.52	**N:T379I**	9	1.95	**N:D377Y**	86	14.41	**N:S235F**	74	3.8
**West Bengal**	**N:T379I**	1	0.52	**N:M234I**	9	1.95	**N:S194L**	83	13.9	**N:D3L**	70	3.59
**West Bengal**	**N:D144Y**	1	0.52	**N:T135I**	8	1.74	**N:S235F**	72	12.06	**N:S413I**	38	1.95
**West Bengal**	**N:L221F**	1	0.52	**N:A182S**	8	1.74	**N:D3L**	59	9.88	**N:D3Y**	28	1.44
**West Bengal**	**N:V392L**	1	0.52	**N:D128Y**	7	1.52	**N:D3Y**	32	5.36	**N:G18S**	28	1.44
**West Bengal**	**N:D22Y**	1	0.52	**N:D377Y**	6	1.3	**N:S2P**	22	3.69	**N:M234I**	28	1.44
**West Bengal**	**N:R185C**	1	0.52	**N:S2P**	6	1.3	**N:T205I**	19	3.18	**N:A119S**	27	1.39
**West Bengal**	**N:R195I**	1	0.52	**N:A251S**	5	1.08	**N:T362I**	8	1.34	**N:A217S**	27	1.39
**West Bengal**				**N:G18S**	5	1.08	**N:T334I**	7	1.17	**N:E367Q**	26	1.33
**West Bengal**				**N:A119S**	5	1.08	**N:D63G**	7	1.17	**N:T205I**	19	0.98
**West Bengal**				**N:E367Q**	5	1.08	**N:R385K**	7	1.17	**N:T135I**	13	0.67
**West Bengal**				**N:R203M**	4	0.87	**N:D22Y**	5	0.84	**N:P67S**	12	0.62
**West Bengal**				**N:V392L**	3	0.65	**N:S202N**	5	0.84	**N:E136D**	8	0.41
**West Bengal**				**N:P67L**	3	0.65	**N:P13L**	4	0.67	**N:D144H**	7	0.36
**West Bengal**				**N:P383S**	3	0.65	**N:S2M**	4	0.67	**N:P13L**	6	0.31
**West Bengal**				**N:V270L**	3	0.65	**N:A12G**	4	0.67	**N:S2M**	5	0.26
**West Bengal**				**N:T271I**	3	0.65	**N:V270L**	4	0.67	**N:A12G**	5	0.26
**West Bengal**				**N:S327L**	3	0.65	**N:A211V**	4	0.67	**N:L139F**	5	0.26
**West Bengal**				**N:P199S**	2	0.43	**N:T379I**	3	0.5	**N:S193T**	5	0.26
**West Bengal**				**N:T366I**	2	0.43	**N:T135I**	3	0.5	**N:P13S**	5	0.26
**West Bengal**				**N:Q418H**	2	0.43	**N:R209I**	3	0.5	**N:T366I**	4	0.21
**West Bengal**				**N:A152S**	2	0.43	**N:N11S**	3	0.5	**N:D401Y**	4	0.21
**West Bengal**				**N:G34W**	2	0.43	**N:S413I**	3	0.5	**N:K373N**	4	0.21
**West Bengal**				**N:D399Y**	2	0.43	**N:E367A**	2	0.34	**N:G204V**	4	0.21
**West Bengal**				**N:S412I**	2	0.43	**N:K169R**	2	0.34	**N:T296I**	4	0.21
**West Bengal**				**N:T391I**	2	0.43	**N:D144Y**	2	0.34	**N:D144Y**	3	0.15
**West Bengal**				**N:G30R**	2	0.43	**N:K373N**	2	0.34	**N:T271I**	3	0.15
**West Bengal**				**N:Q418L**	2	0.43	**N:A35S**	2	0.34	**N:S194L**	3	0.15
**West Bengal**				**N:S2Y**	2	0.43	**N:P142S**	2	0.34	**N:P46S**	3	0.15
**West Bengal**				**N:A211V**	2	0.43	**N:T265I**	2	0.34	**N:Q39L**	3	0.15
**West Bengal**				**N:D144Y**	2	0.43	**N:D128Y**	2	0.34	**N:S187L**	3	0.15
**West Bengal**				**N:D144H**	1	0.22	**N:M210I**	2	0.34	**N:T391I**	3	0.15
**West Bengal**				**N:S193I**	1	0.22	**N:E367G**	2	0.34	**N:Q229H**	3	0.15
**West Bengal**				**N:R209K**	1	0.22	**N:A182S**	2	0.34	**N:K374N**	3	0.15

West Bengal				N:P365S	1	0.22	N:H300Y	2	0.34	N:S193I	3	0.15
West Bengal				N:G212S	1	0.22	N:S193I	2	0.34	N:S33I	3	0.15
West Bengal				N:A398S	1	0.22	N:D343Y	2	0.34	N:Q9H	3	0.15
West Bengal				N:D402Y	1	0.22	N:G243C	2	0.34	N:D144A	3	0.15
West Bengal				N:H300Y	1	0.22	N:D371V	2	0.34	N:D297H	3	0.15
West Bengal				N:R40C	1	0.22	N:G34W	2	0.34	N:T325I	2	0.1
West Bengal				N:E378Q	1	0.22	N:T271I	2	0.34	N:T334I	2	0.1
West Bengal				N:I292L	1	0.22	N:D3E	2	0.34	N:S2P	2	0.1
West Bengal				N:S413I	1	0.22	N:P383S	2	0.34	N:S26I	2	0.1
West Bengal				N:P67S	1	0.22	N:R385I	2	0.34	N:D402Y	2	0.1
West Bengal				N:G5E	1	0.22	N:K369N	1	0.17	N:D22Y	2	0.1
West Bengal				N:D340Y	1	0.22	N:P279L	1	0.17	N:A381S	2	0.1
West Bengal				N:Q7H	1	0.22	N:E280V	1	0.17	N:T379I	2	0.1
West Bengal				N:L339F	1	0.22	N:G238C	1	0.17	N:D399H	2	0.1
West Bengal				N:T296I	1	0.22	N:I351F	1	0.17	N:A211S	2	0.1
West Bengal				N:K373N	1	0.22	N:A12V	1	0.17	N:L331F	2	0.1
West Bengal				N:G120V	1	0.22	N:D348Y	1	0.17	N:G120V	2	0.1
West Bengal				N:D63E	1	0.22	N:R226K	1	0.17	N:P199S	2	0.1
West Bengal				N:P20L	1	0.22	N:R191L	1	0.17	N:V270L	2	0.1
West Bengal				N:A218V	1	0.22	N:V392F	1	0.17	N:T198I	2	0.1
West Bengal				N:R293K	1	0.22	N:Q380R	1	0.17	N:A414S	2	0.1
West Bengal				N:T16M	1	0.22	N:A211T	1	0.17	N:Q160R	2	0.1
West Bengal				N:R40L	1	0.22	N:P365L	1	0.17	N:R209I	2	0.1
West Bengal				N:P151S	1	0.22	N:G124D	1	0.17	N:A55S	2	0.1
West Bengal				N:P6L	1	0.22	N:P6T	1	0.17	N:K387N	1	0.05
West Bengal				N:L167F	1	0.22	N:K102R	1	0.17	N:A35V	1	0.05
West Bengal				N:Q70R	1	0.22	N:P13T	1	0.17	N:P168S	1	0.05
West Bengal				N:Q409L	1	0.22	N:T393I	1	0.17	N:T379S	1	0.05
West Bengal				N:M210I	1	0.22	N:Q386L	1	0.17	N:D297Y	1	0.05
West Bengal				N:P344S	1	0.22	N:T16M	1	0.17	N:S310N	1	0.05
West Bengal				N:P122L	1	0.22	N:S183F	1	0.17	N:K102R	1	0.05
West Bengal				N:A308S	1	0.22	N:A35V	1	0.17	N:P383A	1	0.05
West Bengal				N:A173V	1	0.22	N:D343H	1	0.17	N:E136Q	1	0.05
West Bengal				N:D371N	1	0.22	N:A208S	1	0.17	N:S202N	1	0.05
West Bengal				N:T334I	1	0.22	N:P13S	1	0.17	N:P142S	1	0.05
West Bengal				N:R191L	1	0.22	N:T417I	1	0.17	N:N8D	1	0.05
West Bengal				N:Q289H	1	0.22	N:A398V	1	0.17	N:Q389H	1	0.05
West Bengal				N:D144G	1	0.22	N:V392L	1	0.17	N:A155V	1	0.05
West Bengal				N:Q380R	1	0.22	N:A12T	1	0.17	N:R195I	1	0.05
West Bengal				N:G238S	1	0.22	N:G25C	1	0.17	N:T165I	1	0.05
West Bengal				N:P13L	1	0.22	N:D3Q	1	0.17	N:T379A	1	0.05
West Bengal				N:A359S	1	0.22	N:T366I	1	0.17	N:R36Q	1	0.05
West Bengal				N:R185C	1	0.22	N:D401Y	1	0.17	N:A90T	1	0.05
West Bengal				N:G215C	1	0.22	N:D402H	1	0.17	N:G212V	1	0.05
West Bengal				N:D348Y	1	0.22	N:A90S	1	0.17	N:A156S	1	0.05
West Bengal				N:D22H	1	0.22	N:D128H	1	0.17	N:M322I	1	0.05
West Bengal				N:P279L	1	0.22				N:G25C	1	0.05
West Bengal				N:P383L	1	0.22				N:A90S	1	0.05
West Bengal				N:P142S	1	0.22				N:G236V	1	0.05
West Bengal				N:T265I	1	0.22				N:G34E	1	0.05
West Bengal				N:Q229H	1	0.22				N:R203I	1	0.05
West Bengal				N:S193T	1	0.22				N:P383L	1	0.05
West Bengal										N:T263N	1	0.05
West Bengal										N:A152S	1	0.05
West Bengal										N:S21P	1	0.05
West Bengal										N:G178C	1	0.05
West Bengal										N:A311V	1	0.05
West Bengal										N:P207L	1	0.05
West Bengal										N:L230F	1	0.05
West Bengal										N:Q418H	1	0.05
West Bengal										N:G243C	1	0.05
West Bengal										N:R32H	1	0.05
West Bengal										N:I157L	1	0.05
West Bengal										N:K372N	1	0.05
West Bengal										N:A217T	1	0.05
West Bengal										N:R89T	1	0.05
West Bengal										N:D343H	1	0.05
West Bengal										N:R191H	1	0.05
West Bengal										N:G30R	1	0.05
West Bengal										N:G321V	1	0.05
West Bengal										N:S327L	1	0.05
West Bengal										N:G179V	1	0.05
West Bengal										N:R385I	1	0.05

West Bengal										N:Q181R	1	0.05
West Bengal										N:N8Y	1	0.05
West Bengal										N:K361R	1	0.05
West Bengal										N:A414V	1	0.05
West Bengal										N:Q7L	1	0.05
West Bengal										N:N8H	1	0.05
West Bengal										N:P168Q	1	0.05
West Bengal										N:D348H	1	0.05
West Bengal										N:T417I	1	0.05
West Bengal										N:N8S	1	0.05
West Bengal										N:P80S	1	0.05
West Bengal										N:K248N	1	0.05
West Bengal										N:D103H	1	0.05
West Bengal										N:D371N	1	0.05
West Bengal										N:N140S	1	0.05
West Bengal										N:R40C	1	0.05
West Bengal										N:P326S	1	0.05
State/Gene	ORF3a											
West Bengal	Pre-Wave 1			Wave 1			Pre-Wave 2			Wave 2		
West Bengal	Mutation	Count	percent	Mutation	Count	percent	Mutation	Count	percent	Mutation	Count	percent
West Bengal	ORF3a:V13L	1	6.25	ORF3a:Q57H	99	22.86	ORF3a:T151I	137	20.3	ORF3a:S26L	1804	77.03
West Bengal	ORF3a:G100C	1	6.25	ORF3a:T223I	77	17.78	ORF3a:T223I	95	14.07	ORF3a:D238Y	40	1.71
West Bengal	ORF3a:W131C	2	12.5	ORF3a:G172C	51	11.78	ORF3a:Q57H	89	13.19	ORF3a:T151I	32	1.37
West Bengal	ORF3a:E239D	1	6.25	ORF3a:E239D	12	2.77	ORF3a:S26L	88	13.04	ORF3a:Q57H	16	0.68
West Bengal	ORF3a:K198R	1	6.25	ORF3a:T151I	11	2.54	ORF3a:G172C	64	9.48	ORF3a:S171L	15	0.64
West Bengal	ORF3a:A103S	1	6.25	ORF3a:V55G	10	2.31	ORF3a:E261*	22	3.26	ORF3a:A23V	12	0.51
West Bengal	ORF3a:S60F	1	6.25	ORF3a:L73F	9	2.08	ORF3a:S171L	12	1.78	ORF3a:G172C	11	0.47
West Bengal	ORF3a:M125T	2	12.5	ORF3a:G224C	8	1.85	ORF3a:E239D	9	1.33	ORF3a:R6D	11	0.47
West Bengal	ORF3a:D155Y	1	6.25	ORF3a:T175I	7	1.62	ORF3a:T175I	9	1.33	ORF3a:I7L	11	0.47
West Bengal	ORF3a:A99V	1	6.25	ORF3a:S171L	7	1.62	ORF3a:V77I	8	1.19	ORF3a:T9R	11	0.47
West Bengal	ORF3a:D155H	1	6.25	ORF3a:W128C	6	1.39	ORF3a:P240H	7	1.04	ORF3a:G11F	11	0.47
West Bengal	ORF3a:G18V	1	6.25	ORF3a:D155Y	6	1.39	ORF3a:G174V	6	0.89	ORF3a:K21G	11	0.47
West Bengal	ORF3a:Q57H	1	6.25	ORF3a:E261*	6	1.39	ORF3a:V273L	5	0.74	ORF3a:D22T	11	0.47
West Bengal	ORF3a:Q245R	1	6.25	ORF3a:V77I	5	1.15	ORF3a:D155Y	5	0.74	ORF3a:P25G	11	0.47
West Bengal				ORF3a:S165F	5	1.15	ORF3a:N257D	5	0.74	ORF3a:D27R	11	0.47
West Bengal				ORF3a:V97I	4	0.92	ORF3a:G224C	5	0.74	ORF3a:F28Y	11	0.47
West Bengal				ORF3a:L275F	4	0.92	ORF3a:D173G	4	0.59	ORF3a:V29C	11	0.47
West Bengal				ORF3a:W45L	4	0.92	ORF3a:K21N	4	0.59	ORF3a:R30N	11	0.47
West Bengal				ORF3a:L94F	4	0.92	ORF3a:G18V	4	0.59	ORF3a:A31D	11	0.47
West Bengal				ORF3a:A99S	4	0.92	ORF3a:T14I	4	0.59	ORF3a:A33D	11	0.47
West Bengal				ORF3a:A103S	3	0.69	ORF3a:G187S	4	0.59	ORF3a:A110S	10	0.43
West Bengal				ORF3a:K75N	3	0.69	ORF3a:T12I	4	0.59	ORF3a:R134H	9	0.38
West Bengal				ORF3a:S166L	3	0.69	ORF3a:T9K	3	0.44	ORF3a:C81F	9	0.38
West Bengal				ORF3a:G172V	3	0.69	ORF3a:L108F	3	0.44	ORF3a:W69C	9	0.38
West Bengal				ORF3a:D173V	3	0.69	ORF3a:A98T	3	0.44	ORF3a:L106F	9	0.38
West Bengal				ORF3a:L108F	2	0.46	ORF3a:S40P	2	0.3	ORF3a:W131C	8	0.34
West Bengal				ORF3a:A99V	2	0.46	ORF3a:G49V	2	0.3	ORF3a:D155Y	6	0.26
West Bengal				ORF3a:M125I	2	0.46	ORF3a:K61T	2	0.3	ORF3a:L85F	6	0.26
West Bengal				ORF3a:L140F	2	0.46	ORF3a:G100C	2	0.3	ORF3a:S252L	6	0.26
West Bengal				ORF3a:G174C	2	0.46	ORF3a:R126M	2	0.3	ORF3a:G49V	6	0.26
West Bengal				ORF3a:A23V	2	0.46	ORF3a:L94F	2	0.3	ORF3a:T221K	6	0.26
West Bengal				ORF3a:K21N	2	0.46	ORF3a:H78Y	2	0.3	ORF3a:T89I	6	0.26
West Bengal				ORF3a:V256F	2	0.46	ORF3a:V13L	2	0.3	ORF3a:W128L	6	0.26
West Bengal				ORF3a:T9K	2	0.46	ORF3a:G254*	2	0.3	ORF3a:T223I	5	0.21
West Bengal				ORF3a:G49V	2	0.46	ORF3a:K136E	2	0.3	ORF3a:Q57R	5	0.21
West Bengal				ORF3a:H78Y	2	0.46	ORF3a:A110V	2	0.3	ORF3a:K16N	5	0.21
West Bengal				ORF3a:A59T	2	0.46	ORF3a:A23S	2	0.3	ORF3a:V77F	5	0.21
West Bengal				ORF3a:W131C	2	0.46	ORF3a:D173N	2	0.3	ORF3a:P42S	5	0.21
West Bengal				ORF3a:G11R	2	0.46	ORF3a:P42L	2	0.3	ORF3a:L95F	4	0.17
West Bengal				ORF3a:G174V	2	0.46	ORF3a:S166L	2	0.3	ORF3a:T32I	4	0.17
West Bengal				ORF3a:I20V	2	0.46	ORF3a:F43L	1	0.15	ORF3a:L147F	4	0.17
West Bengal				ORF3a:S40L	1	0.23	ORF3a:V48F	1	0.15	ORF3a:E239D	4	0.17
West Bengal				ORF3a:L106I	1	0.23	ORF3a:D222Y	1	0.15	ORF3a:I10T	4	0.17
West Bengal				ORF3a:P42S	1	0.23	ORF3a:V55G	1	0.15	ORF3a:P240L	4	0.17
West Bengal				ORF3a:Y156H	1	0.23	ORF3a:D155A	1	0.15	ORF3a:V90F	4	0.17
West Bengal				ORF3a:P240L	1	0.23	ORF3a:Q185H	1	0.15	ORF3a:V112F	4	0.17
West Bengal				ORF3a:V55F	1	0.23	ORF3a:L101F	1	0.15	ORF3a:S92L	3	0.13
West Bengal				ORF3a:L95V	1	0.23	ORF3a:A99T	1	0.15	ORF3a:K21N	3	0.13
West Bengal				ORF3a:T24I	1	0.23	ORF3a:L53F	1	0.15	ORF3a:D222E	3	0.13
West Bengal				ORF3a:T32I	1	0.23	ORF3a:C133F	1	0.15	ORF3a:G18V	3	0.13
West Bengal				ORF3a:G100C	1	0.23	ORF3a:D27A	1	0.15	ORF3a:S216L	3	0.13
West Bengal				ORF3a:P104R	1	0.23	ORF3a:L95F	1	0.15	ORF3a:L108F	3	0.13

**West Bengal**				**ORF3a:V90F**	1	0.23	**ORF3a:D173Y**	1	0.15	**ORF3a:T34A**	3	0.13
**West Bengal**				**ORF3a:L46F**	1	0.23	**ORF3a:D22Y**	1	0.15	**ORF3a:S166L**	2	0.09
**West Bengal**				**ORF3a:L101F**	1	0.23	**ORF3a:V29L**	1	0.15	**ORF3a:E261***	2	0.09
**West Bengal**				**ORF3a:D155H**	1	0.23	**ORF3a:L140F**	1	0.15	**ORF3a:G224C**	2	0.09
**West Bengal**				**ORF3a:K16N**	1	0.23	**ORF3a:S177N**	1	0.15	**ORF3a:T175I**	2	0.09
**West Bengal**				**ORF3a:G18C**	1	0.23	**ORF3a:K198E**	1	0.15	**ORF3a:R68I**	2	0.09
**West Bengal**				**ORF3a:V48F**	1	0.23	**ORF3a:S74F**	1	0.15	**ORF3a:Y113H**	2	0.09
**West Bengal**				**ORF3a:W131L**	1	0.23	**ORF3a:P240S**	1	0.15	**ORF3a:V202L**	2	0.09
**West Bengal**				**ORF3a:V29A**	1	0.23	**ORF3a:K67N**	1	0.15	**ORF3a:T64I**	2	0.09
**West Bengal**				**ORF3a:H243Y**	1	0.23	**ORF3a:L15F**	1	0.15	**ORF3a:A54S**	2	0.09
**West Bengal**				**ORF3a:G224V**	1	0.23	**ORF3a:K16N**	1	0.15	**ORF3a:W69R**	2	0.09
**West Bengal**				**ORF3a:C81F**	1	0.23	**ORF3a:G172V**	1	0.15	**ORF3a:Q245H**	2	0.09
**West Bengal**				**ORF3a:H93Y**	1	0.23	**ORF3a:L65F**	1	0.15	**ORF3a:A110V**	2	0.09
**West Bengal**				**ORF3a:P104L**	1	0.23	**ORF3a:V112F**	1	0.15	**ORF3a:A23S**	2	0.09
**West Bengal**				**ORF3a:D199Y**	1	0.23	**ORF3a:Q245L**	1	0.15	**ORF3a:H78Y**	2	0.09
**West Bengal**				**ORF3a:G251C**	1	0.23	**ORF3a:V163L**	1	0.15	**ORF3a:G44V**	2	0.09
**West Bengal**				**ORF3a:D210Y**	1	0.23	**ORF3a:S92L**	1	0.15	**ORF3a:L108P**	2	0.09
**West Bengal**				**ORF3a:P104S**	1	0.23	**ORF3a:N144Y**	1	0.15	**ORF3a:P240H**	1	0.04
**West Bengal**				**ORF3a:A23P**	1	0.23	**ORF3a:V97F**	1	0.15	**ORF3a:D142G**	1	0.04
**West Bengal**				**ORF3a:V112I**	1	0.23	**ORF3a:R68I**	1	0.15	**ORF3a:P42L**	1	0.04
**West Bengal**				**ORF3a:T89I**	1	0.23	**ORF3a:G49C**	1	0.15	**ORF3a:V97F**	1	0.04
**West Bengal**				**ORF3a:D265Y**	1	0.23	**ORF3a:A98V**	1	0.15	**ORF3a:Q38L**	1	0.04
**West Bengal**				**ORF3a:K67N**	1	0.23	**ORF3a:L106R**	1	0.15	**ORF3a:T269M**	1	0.04
**West Bengal**				**ORF3a:Q185H**	1	0.23	**ORF3a:V202A**	1	0.15	**ORF3a:P262S**	1	0.04
**West Bengal**				**ORF3a:G187S**	1	0.23	**ORF3a:P240L**	1	0.15	**ORF3a:V29F**	1	0.04
**West Bengal**				**ORF3a:Y107H**	1	0.23	**ORF3a:D27V**	1	0.15	**ORF3a:L15F**	1	0.04
**West Bengal**				**ORF3a:L53F**	1	0.23	**ORF3a:T89I**	1	0.15	**ORF3a:Q213R**	1	0.04
**West Bengal**				**ORF3a:D27Y**	1	0.23	**ORF3a:D27H**	1	0.15	**ORF3a:S165F**	1	0.04
**West Bengal**				**ORF3a:E19D**	1	0.23	**ORF3a:D222A**	1	0.15	**ORF3a:L140F**	1	0.04
**West Bengal**				**ORF3a:D27A**	1	0.23	**ORF3a:G254R**	1	0.15	**ORF3a:V273L**	1	0.04
**West Bengal**				**ORF3a:M260I**	1	0.23	**ORF3a:A110S**	1	0.15	**ORF3a:Y91C**	1	0.04
**West Bengal**				**ORF3a:V202L**	1	0.23	**ORF3a:S60F**	1	0.15	**ORF3a:S60N**	1	0.04
**West Bengal**				**ORF3a:I35T**	1	0.23	**ORF3a:V88L**	1	0.15	**ORF3a:L108H**	1	0.04
**West Bengal**				**ORF3a:S26L**	1	0.23	**ORF3a:I37T**	1	0.15	**ORF3a:V256A**	1	0.04
**West Bengal**				**ORF3a:V273L**	1	0.23				**ORF3a:D22Y**	1	0.04
**West Bengal**				**ORF3a:V13L**	1	0.23				**ORF3a:F114L**	1	0.04
**West Bengal**										**ORF3a:A99V**	1	0.04
**West Bengal**										**ORF3a:V13L**	1	0.04
**West Bengal**										**ORF3a:L106R**	1	0.04
**West Bengal**										**ORF3a:F207L**	1	0.04
**West Bengal**										**ORF3a:W131R**	1	0.04
**West Bengal**										**ORF3a:A31S**	1	0.04
**West Bengal**										**ORF3a:R126M**	1	0.04
**West Bengal**										**ORF3a:I186L**	1	0.04
**West Bengal**										**ORF3a:F28L**	1	0.04
**West Bengal**										**ORF3a:A72V**	1	0.04
**West Bengal**										**ORF3a:E194D**	1	0.04
**West Bengal**										**ORF3a:D27Y**	1	0.04
**West Bengal**										**ORF3a:A99S**	1	0.04
**West Bengal**										**ORF3a:D27N**	1	0.04
**West Bengal**										**ORF3a:D155N**	1	0.04
**West Bengal**										**ORF3a:W45L**	1	0.04
**West Bengal**										**ORF3a:L15S**	1	0.04
**West Bengal**										**ORF3a:S74F**	1	0.04
**West Bengal**										**ORF3a:G100C**	1	0.04
**West Bengal**										**ORF3a:A33S**	1	0.04
**West Bengal**										**ORF3a:D199N**	1	0.04
**West Bengal**										**ORF3a:G100V**	1	0.04
**West Bengal**										**ORF3a:Q218P**	1	0.04
**West Bengal**										**ORF3a:A54V**	1	0.04
**West Bengal**										**ORF3a:D142H**	1	0.04
**West Bengal**										**ORF3a:S177I**	1	0.04
**West Bengal**										**ORF3a:T175A**	1	0.04
**West Bengal**										**ORF3a:L275F**	1	0.04
**West Bengal**										**ORF3a:D222Y**	1	0.04
**West Bengal**										**ORF3a:A103S**	1	0.04
**West Bengal**										**ORF3a:L53I**	1	0.04
**West Bengal**										**ORF3a:Q185H**	1	0.04
**West Bengal**										**ORF3a:V55F**	1	0.04
**West Bengal**										**ORF3a:Q116H**	1	0.04
**West Bengal**										**ORF3a:T229I**	1	0.04
**West Bengal**										**ORF3a:G44R**	1	0.04
**West Bengal**										**ORF3a:P25S**	1	0.04

**West Bengal**										**ORF3a:A99G**	1	0.04
**West Bengal**										**ORF3a:Y113C**	1	0.04
**West Bengal**										**ORF3a:I35T**	1	0.04
**West Bengal**										**ORF3a:G254R**	1	0.04
**West Bengal**										**ORF3a:L86F**	1	0.04
**West Bengal**										**ORF3a:V88L**	1	0.04
**West Bengal**										**ORF3a:G172V**	1	0.04
**State/Gene**	**ORF6**											
**West Bengal**	**Pre-Wave 1**			**Wave 1**			**Pre-Wave 2**			**Wave 2**		
**West Bengal**	**Mutation**	**Count**	**percent**	**Mutation**	**Count**	**percent**	**Mutation**	**Count**	**percent**	**Mutation**	**Count**	**percent**
**West Bengal**	**ORF6:D30Y**	1	33.33	**ORF6:K42N**	2	22.22	**ORF6:I33T**	12	30.77	**ORF6:I33T**	40	43.01
**West Bengal**	**ORF6:F2S**	1	33.33	**ORF6:L4F**	1	11.11	**ORF6:L15V**	5	12.82	**ORF6:R20K**	11	11.83
**West Bengal**	**ORF6:K48R**	1	33.33	**ORF6:D53G**	1	11.11	**ORF6:F2M**	4	10.26	**ORF6:F2M**	5	5.38
**West Bengal**				**ORF6:H3Y**	1	11.11	**ORF6:V24F**	3	7.69	**ORF6:V24F**	5	5.38
**West Bengal**				**ORF6:F7L**	1	11.11	**ORF6:R20K**	3	7.69	**ORF6:I36L**	3	3.23
**West Bengal**				**ORF6:I32T**	1	11.11	**ORF6:K42T**	2	5.13	**ORF6:E13D**	3	3.23
**West Bengal**				**ORF6:W27L**	1	11.11	**ORF6:E55Q**	2	5.13	**ORF6:W27L**	3	3.23
**West Bengal**				**ORF6:V24F**	1	11.11	**ORF6:D61G**	2	5.13	**ORF6:Q8***	2	2.15
**West Bengal**							**ORF6:I32T**	2	5.13	**ORF6:E54***	2	2.15
**West Bengal**							**ORF6:L29F**	1	2.56	**ORF6:M19I**	2	2.15
**West Bengal**							**ORF6:T21I**	1	2.56	**ORF6:I33L**	2	2.15
**West Bengal**							**ORF6:N39H**	1	2.56	**ORF6:I37L**	2	2.15
**West Bengal**							**ORF6:I36L**	1	2.56	**ORF6:E59***	1	1.08
**West Bengal**										**ORF6:D61L**	1	1.08
**West Bengal**										**ORF6:P57R**	1	1.08
**West Bengal**										**ORF6:Y49H**	1	1.08
**West Bengal**										**ORF6:E59D**	1	1.08
**West Bengal**										**ORF6:R20S**	1	1.08
**West Bengal**										**ORF6:D6G**	1	1.08
**West Bengal**										**ORF6:I26V**	1	1.08
**West Bengal**										**ORF6:M58T**	1	1.08
**West Bengal**										**ORF6:D53Y**	1	1.08
**West Bengal**										**ORF6:E55***	1	1.08
**West Bengal**										**ORF6:S43P**	1	1.08
**West Bengal**										**ORF6:D53V**	1	1.08
**State/Gene**	**ORF7a**											
**West Bengal**	**Pre-Wave 1**			**Wave 1**			**Pre-Wave 2**			**Wave 2**		
**West Bengal**	**Mutation**	**Count**	**percent**	**Mutation**	**Count**	**percent**	**Mutation**	**Count**	**percent**	**Mutation**	**Count**	**percent**
**West Bengal**	**ORF7a:P99L**	1	16.67	**ORF7a:P34S**	5	15.15	**ORF7a:P34S**	119	47.04	**ORF7a:V82A**	1478	43.77
**West Bengal**	**ORF7a:A79S**	2	33.33	**ORF7a:A8T**	2	6.06	**ORF7a:V82A**	73	28.85	**ORF7a:T120I**	1070	31.68
**West Bengal**	**ORF7a:P34S**	1	16.67	**ORF7a:T120I**	2	6.06	**ORF7a:Q62***	9	3.56	**ORF7a:L116F**	681	20.17
**West Bengal**	**ORF7a:E121***	1	16.67	**ORF7a:T14I**	2	6.06	**ORF7a:L116F**	6	2.37	**ORF7a:P34S**	27	0.8
**West Bengal**	**ORF7a:V104F**	1	16.67	**ORF7a:R89T**	2	6.06	**ORF7a:P99L**	5	1.98	**ORF7a:N43Y**	13	0.38
**West Bengal**				**ORF7a:A64S**	2	6.06	**ORF7a:T39I**	5	1.98	**ORF7a:Q62***	5	0.15
**West Bengal**				**ORF7a:S83L**	2	6.06	**ORF7a:V71L**	5	1.98	**ORF7a:C23F**	5	0.15
**West Bengal**				**ORF7a:H47Y**	1	3.03	**ORF7a:P99S**	4	1.58	**ORF7a:R25I**	4	0.12
**West Bengal**				**ORF7a:A106T**	1	3.03	**ORF7a:E22D**	3	1.19	**ORF7a:P84S**	4	0.12
**West Bengal**				**ORF7a:F63H**	1	3.03	**ORF7a:E95K**	2	0.79	**ORF7a:V29L**	4	0.12
**West Bengal**				**ORF7a:L116V**	1	3.03	**ORF7a:E95Q**	2	0.79	**ORF7a:R89I**	4	0.12
**West Bengal**				**ORF7a:L5F**	1	3.03	**ORF7a:T28I**	2	0.79	**ORF7a:T14I**	3	0.09
**West Bengal**				**ORF7a:P45L**	1	3.03	**ORF7a:K119N**	2	0.79	**ORF7a:E41***	3	0.09
**West Bengal**				**ORF7a:G38V**	1	3.03	**ORF7a:*122S**	2	0.79	**ORF7a:E91D**	3	0.09
**West Bengal**				**ORF7a:P34L**	1	3.03	**ORF7a:V93I**	1	0.4	**ORF7a:R78C**	2	0.06
**West Bengal**				**ORF7a:S37Y**	1	3.03	**ORF7a:P84L**	1	0.4	**ORF7a:R80I**	2	0.06
**West Bengal**				**ORF7a:R89I**	1	3.03	**ORF7a:R78P**	1	0.4	**ORF7a:G38V**	2	0.06
**West Bengal**				**ORF7a:E121***	1	3.03	**ORF7a:Q90R**	1	0.4	**ORF7a:V24F**	2	0.06
**West Bengal**				**ORF7a:P84S**	1	3.03	**ORF7a:G70D**	1	0.4	**ORF7a:T61I**	2	0.06
**West Bengal**				**ORF7a:I10V**	1	3.03	**ORF7a:E92***	1	0.4	**ORF7a:C113F**	2	0.06
**West Bengal**				**ORF7a:V71I**	1	3.03	**ORF7a:A105V**	1	0.4	**ORF7a:E95***	2	0.06
**West Bengal**				**ORF7a:V29L**	1	3.03	**ORF7a:R78C**	1	0.4	**ORF7a:R89T**	2	0.06
**West Bengal**				**ORF7a:E95Q**	1	3.03	**ORF7a:R25I**	1	0.4	**ORF7a:S36P**	2	0.06
**West Bengal**							**ORF7a:Y75H**	1	0.4	**ORF7a:R89S**	1	0.03
**West Bengal**							**ORF7a:G38V**	1	0.4	**ORF7a:P99S**	1	0.03
**West Bengal**							**ORF7a:T120I**	1	0.4	**ORF7a:L102F**	1	0.03
**West Bengal**							**ORF7a:N43Y**	1	0.4	**ORF7a:I10T**	1	0.03
**West Bengal**							**ORF7a:P68L**	1	0.4	**ORF7a:V24I**	1	0.03
**West Bengal**										**ORF7a:T39I**	1	0.03
**West Bengal**										**ORF7a:H73Y**	1	0.03

**West Bengal**										**ORF7a:L96P**	1	0.03
**West Bengal**										**ORF7a:F101I**	1	0.03
**West Bengal**										**ORF7a:Y20H**	1	0.03
**West Bengal**										**ORF7a:E95Q**	1	0.03
**West Bengal**										**ORF7a:V104F**	1	0.03
**West Bengal**										**ORF7a:A8V**	1	0.03
**West Bengal**										**ORF7a:A8T**	1	0.03
**West Bengal**										**ORF7a:L31I**	1	0.03
**West Bengal**										**ORF7a:K2E**	1	0.03
**West Bengal**										**ORF7a:T111I**	1	0.03
**West Bengal**										**ORF7a:V71L**	1	0.03
**West Bengal**										**ORF7a:T28I**	1	0.03
**West Bengal**										**ORF7a:F65I**	1	0.03
**West Bengal**										**ORF7a:A66R**	1	0.03
**West Bengal**										**ORF7a:C67Q**	1	0.03
**West Bengal**										**ORF7a:P68E**	1	0.03
**West Bengal**										**ORF7a:S44C**	1	0.03
**West Bengal**										**ORF7a:T61P**	1	0.03
**West Bengal**										**ORF7a:Q62K**	1	0.03
**West Bengal**										**ORF7a:F63L**	1	0.03
**West Bengal**										**ORF7a:A64F**	1	0.03
**West Bengal**										**ORF7a:S81L**	1	0.03
**West Bengal**										**ORF7a:I88T**	1	0.03
**West Bengal**										**ORF7a:C58F**	1	0.03
**West Bengal**										**ORF7a:I107M**	1	0.03
**West Bengal**										**ORF7a:G42D**	1	0.03
**West Bengal**										**ORF7a:S98F**	1	0.03
**West Bengal**										**ORF7a:A66S**	1	0.03
**West Bengal**										**ORF7a:I100L**	1	0.03
**West Bengal**										**ORF7a:S83L**	1	0.03
**West Bengal**										**ORF7a:I10V**	1	0.03
**West Bengal**										**ORF7a:E41D**	1	0.03
**West Bengal**										**ORF7a:V82S**	1	0.03
**West Bengal**										**ORF7a:P99L**	1	0.03
**West Bengal**										**ORF7a:K119N**	1	0.03
**West Bengal**										**ORF7a:E121Q**	1	0.03
**West Bengal**										**ORF7a:A50S**	1	0.03
**West Bengal**										**ORF7a:L30F**	1	0.03
**West Bengal**										**ORF7a:E22D**	1	0.03
**West Bengal**										**ORF7a:V104K**	1	0.03
**West Bengal**										**ORF7a:A105R**	1	0.03
**West Bengal**										**ORF7a:A106K**	1	0.03
**West Bengal**										**ORF7a:V108E**	1	0.03
**West Bengal**										**ORF7a:F109***	1	0.03
**West Bengal**										**ORF7a:K32N**	1	0.03
**West Bengal**										**ORF7a:V82G**	1	0.03
**West Bengal**										**ORF7a:V93F**	1	0.03
**West Bengal**										**ORF7a:L96F**	1	0.03
**State/Gene**	**ORF7b**											
**West Bengal**	**Pre-Wave 1**			**Wave 1**			**Pre-Wave 2**			**Wave 2**		
**West Bengal**	**Mutation**	**Count**	**percent**	**Mutation**	**Count**	**percent**	**Mutation**	**Count**	**percent**	**Mutation**	**Count**	**percent**
**West Bengal**	**ORF7b:T40I**	1	100	**ORF7b:E33***	6	23.08	**ORF7b:E33***	123	76.4	**ORF7b:T40I**	300	82.19
**West Bengal**				**ORF7b:A43S**	4	15.38	**ORF7b:S31L**	10	6.21	**ORF7b:E33***	27	7.4
**West Bengal**				**ORF7b:A15S**	3	11.54	**ORF7b:L34Q**	5	3.11	**ORF7b:A43V**	18	4.93
**West Bengal**				**ORF7b:A43T**	2	7.69	**ORF7b:L14S**	4	2.48	**ORF7b:E39***	4	1.1
**West Bengal**				**ORF7b:E39***	2	7.69	**ORF7b:L32F**	3	1.86	**ORF7b:A43T**	3	0.82
**West Bengal**				**ORF7b:S31L**	2	7.69	**ORF7b:E3***	3	1.86	**ORF7b:L20P**	3	0.82
**West Bengal**				**ORF7b:L11F**	1	3.85	**ORF7b:E39***	3	1.86	**ORF7b:E33A**	2	0.55
**West Bengal**				**ORF7b:C41S**	1	3.85	**ORF7b:T40I**	2	1.24	**ORF7b:D36N**	2	0.55
**West Bengal**				**ORF7b:I7V**	1	3.85	**ORF7b:M1I**	2	1.24	**ORF7b:W29C**	1	0.27
**West Bengal**				**ORF7b:D8A**	1	3.85	**ORF7b:C12M**	1	0.62	**ORF7b:S5L**	1	0.27
**West Bengal**				**ORF7b:Y10C**	1	3.85	**ORF7b:F13I**	1	0.62	**ORF7b:L32F**	1	0.27
**West Bengal**				**ORF7b:F28V**	1	3.85	**ORF7b:A43T**	1	0.62	**ORF7b:E39G**	1	0.27
**West Bengal**				**ORF7b:T40I**	1	3.85	**ORF7b:A15S**	1	0.62	**ORF7b:F28L**	1	0.27
**West Bengal**							**ORF7b:I27T**	1	0.62	**ORF7b:L4F**	1	0.27
**West Bengal**							**ORF7b:W29R**	1	0.62			
**State/Gene**	**ORF8**											
**West Bengal**	**Pre-Wave 1**			**Wave 1**			**Pre-Wave 2**			**Wave 2**		
**West Bengal**	**Mutation**	**Count**	**percent**	**Mutation**	**Count**	**percent**	**Mutation**	**Count**	**percent**	**Mutation**	**Count**	**percent**

**West Bengal**	**ORF8:E110***	19	42.22	**ORF8:V62L**	60	23.08	**ORF8:Q29L**	122	18.15	**ORF8:R52I**	78	16.81
**West Bengal**	**ORF8:L84S**	16	35.56	**ORF8:L95F**	44	16.92	**ORF8:R52I**	81	12.05	**ORF8:Q27***	75	16.16
**West Bengal**	**ORF8:V32L**	1	2.22	**ORF8:E110***	29	11.15	**ORF8:V62L**	71	10.57	**ORF8:Y73C**	74	15.95
**West Bengal**	**ORF8:K94I**	1	2.22	**ORF8:C20F**	21	8.08	**ORF8:Q27***	69	10.27	**ORF8:K68***	42	9.05
**West Bengal**	**ORF8:V62L**	1	2.22	**ORF8:R52I**	12	4.62	**ORF8:Y73C**	69	10.27	**ORF8:Q29L**	28	6.03
**West Bengal**	**ORF8:V99L**	1	2.22	**ORF8:F86C**	8	3.08	**ORF8:L95F**	51	7.59	**ORF8:S69L**	27	5.82
**West Bengal**	**ORF8:D119N**	1	2.22	**ORF8:G77C**	6	2.31	**ORF8:K68***	47	6.99	**ORF8:V62L**	14	3.02
**West Bengal**	**ORF8:Q72H**	3	6.67	**ORF8:S97I**	6	2.31	**ORF8:S97I**	22	3.27	**ORF8:T26I**	11	2.37
**West Bengal**	**ORF8:V62A**	2	4.44	**ORF8:Q23H**	5	1.92	**ORF8:Q18***	19	2.83	**ORF8:W45C**	11	2.37
**West Bengal**				**ORF8:Q29L**	5	1.92	**ORF8:E106***	9	1.34	**ORF8:Q18***	10	2.16
**West Bengal**				**ORF8:I9T**	4	1.54	**ORF8:T11I**	8	1.19	**ORF8:A65V**	6	1.29
**West Bengal**				**ORF8:W45L**	4	1.54	**ORF8:S69L**	8	1.19	**ORF8:L95F**	4	0.86
**West Bengal**				**ORF8:Q29***	4	1.54	**ORF8:T26I**	5	0.74	**ORF8:Q72H**	4	0.86
**West Bengal**				**ORF8:Q18***	4	1.54	**ORF8:C20F**	4	0.6	**ORF8:S67F**	3	0.65
**West Bengal**				**ORF8:A15V**	3	1.15	**ORF8:A15V**	4	0.6	**ORF8:S54L**	3	0.65
**West Bengal**				**ORF8:E106***	3	1.15	**ORF8:L84S**	4	0.6	**ORF8:F16S**	3	0.65
**West Bengal**				**ORF8:W45C**	2	0.77	**ORF8:E92K**	4	0.6	**ORF8:D35Y**	3	0.65
**West Bengal**				**ORF8:A51S**	2	0.77	**ORF8:I10S**	4	0.6	**ORF8:L84S**	3	0.65
**West Bengal**				**ORF8:A65V**	2	0.77	**ORF8:K68E**	3	0.45	**ORF8:H40N**	3	0.65
**West Bengal**				**ORF8:K68E**	2	0.77	**ORF8:E110***	3	0.45	**ORF8:G8V**	3	0.65
**West Bengal**				**ORF8:C102F**	2	0.77	**ORF8:W45***	3	0.45	**ORF8:E106***	2	0.43
**West Bengal**				**ORF8:V81F**	2	0.77	**ORF8:I121N**	3	0.45	**ORF8:S97I**	2	0.43
**West Bengal**				**ORF8:D35E**	2	0.77	**ORF8:D34E**	2	0.3	**ORF8:W45L**	2	0.43
**West Bengal**				**ORF8:T11I**	2	0.77	**ORF8:S67F**	2	0.3	**ORF8:S82F**	2	0.43
**West Bengal**				**ORF8:I39T**	2	0.77	**ORF8:V81F**	2	0.3	**ORF8:E92K**	2	0.43
**West Bengal**				**ORF8:I71M**	1	0.38	**ORF8:Q29***	2	0.3	**ORF8:C37W**	2	0.43
**West Bengal**				**ORF8:T11K**	1	0.38	**ORF8:V114I**	2	0.3	**ORF8:F120V**	2	0.43
**West Bengal**				**ORF8:V117F**	1	0.38	**ORF8:G77C**	2	0.3	**ORF8:H17Y**	2	0.43
**West Bengal**				**ORF8:I9N**	1	0.38	**ORF8:H17Y**	2	0.3	**ORF8:I121L**	2	0.43
**West Bengal**				**ORF8:P93H**	1	0.38	**ORF8:D119Y**	2	0.3	**ORF8:D63G**	2	0.43
**West Bengal**				**ORF8:P36L**	1	0.38	**ORF8:I121L**	2	0.3	**ORF8:C102F**	2	0.43
**West Bengal**				**ORF8:A14T**	1	0.38	**ORF8:F16S**	2	0.3	**ORF8:C83F**	2	0.43
**West Bengal**				**ORF8:P93L**	1	0.38	**ORF8:S103A**	2	0.3	**ORF8:V100L**	2	0.43
**West Bengal**				**ORF8:L60F**	1	0.38	**ORF8:W45L**	2	0.3	**ORF8:T11I**	1	0.22
**West Bengal**				**ORF8:H112Y**	1	0.38	**ORF8:F86C**	1	0.15	**ORF8:D119N**	1	0.22
**West Bengal**				**ORF8:F3L**	1	0.38	**ORF8:T87I**	1	0.15	**ORF8:C102S**	1	0.22
**West Bengal**				**ORF8:E19***	1	0.38	**ORF8:H40Y**	1	0.15	**ORF8:E64A**	1	0.22
**West Bengal**				**ORF8:W45S**	1	0.38	**ORF8:D63R**	1	0.15	**ORF8:K94***	1	0.22
**West Bengal**				**ORF8:S69L**	1	0.38	**ORF8:E64G**	1	0.15	**ORF8:H28R**	1	0.22
**West Bengal**				**ORF8:G8E**	1	0.38	**ORF8:A65***	1	0.15	**ORF8:I10V**	1	0.22
**West Bengal**				**ORF8:F16S**	1	0.38	**ORF8:K68F**	1	0.15	**ORF8:T12I**	1	0.22
**West Bengal**				**ORF8:P30L**	1	0.38	**ORF8:P70F**	1	0.15	**ORF8:S69P**	1	0.22
**West Bengal**				**ORF8:E64***	1	0.38	**ORF8:I71T**	1	0.15	**ORF8:V33F**	1	0.22
**West Bengal**				**ORF8:A65T**	1	0.38	**ORF8:Q72F**	1	0.15	**ORF8:Y42***	1	0.22
**West Bengal**				**ORF8:D75Y**	1	0.38	**ORF8:S82N**	1	0.15	**ORF8:R101H**	1	0.22
**West Bengal**				**ORF8:V32L**	1	0.38	**ORF8:C83***	1	0.15	**ORF8:I39T**	1	0.22
**West Bengal**				**ORF8:S67F**	1	0.38	**ORF8:F86G**	1	0.15	**ORF8:I9F**	1	0.22
**West Bengal**				**ORF8:I121L**	1	0.38	**ORF8:N89G**	1	0.15	**ORF8:Q18R**	1	0.22
**West Bengal**				**ORF8:C37F**	1	0.38	**ORF8:C90***	1	0.15	**ORF8:E19***	1	0.22
**West Bengal**							**ORF8:Q91S**	1	0.15	**ORF8:A65S**	1	0.22
**West Bengal**							**ORF8:E92C**	1	0.15	**ORF8:E110***	1	0.22
**West Bengal**							**ORF8:P93S**	1	0.15	**ORF8:F3S**	1	0.22
**West Bengal**							**ORF8:K94A**	1	0.15	**ORF8:P30T**	1	0.22
**West Bengal**							**ORF8:G96F**	1	0.15	**ORF8:L4F**	1	0.22
**West Bengal**							**ORF8:S97V**	1	0.15	**ORF8:E110Q**	1	0.22
**West Bengal**							**ORF8:V99***	1	0.15	**ORF8:E64D**	1	0.22
**West Bengal**							**ORF8:V100R**	1	0.15	**ORF8:I10T**	1	0.22
**West Bengal**							**ORF8:R101S**	1	0.15	**ORF8:P38L**	1	0.22
**West Bengal**							**ORF8:S103C**	1	0.15	**ORF8:K68E**	1	0.22
**West Bengal**							**ORF8:D107***	1	0.15	**ORF8:S67Y**	1	0.22
**West Bengal**							**ORF8:E19***	1	0.15	**ORF8:G77S**	1	0.22
**West Bengal**							**ORF8:W45C**	1	0.15	**ORF8:C25Y**	1	0.22
**West Bengal**							**ORF8:I71L**	1	0.15	**ORF8:A65T**	1	0.22
**West Bengal**							**ORF8:Q23H**	1	0.15	**ORF8:C25R**	1	0.22
**West Bengal**							**ORF8:F120L**	1	0.15	**ORF8:P36L**	1	0.22
**West Bengal**							**ORF8:P36S**	1	0.15	**ORF8:E64***	1	0.22
**West Bengal**							**ORF8:A65V**	1	0.15			
**West Bengal**							**ORF8:I10T**	1	0.15			
**West Bengal**							**ORF8:V33F**	1	0.15			
**State/Gene**	**ORF9b**											
**West Bengal**	**Pre-Wave 1**			**Wave 1**			**Pre-Wave 2**			**Wave 2**		

West Bengal	Mutation	Count	percent	Mutation	Count	percent	Mutation	Count	percent	Mutation	Count	percent
West Bengal	ORF9b:P10S	11	78.57	ORF9b:L64F	3	10	ORF9b:R32P	9	16.98	ORF9b:T60A	1293	94.66
West Bengal	ORF9b:Q34*	1	7.14	ORF9b:E65G	3	10	ORF9b:T60A	7	13.21	ORF9b:R25K	13	0.95
West Bengal	ORF9b:Q18H	1	7.14	ORF9b:T72I	2	6.67	ORF9b:R32L	6	11.32	ORF9b:R32L	7	0.51
West Bengal	ORF9b:A82V	1	7.14	ORF9b:V37A	2	6.67	ORF9b:Q18H	5	9.43	ORF9b:P10S	6	0.44
West Bengal				ORF9b:R32P	2	6.67	ORF9b:P10S	4	7.55	ORF9b:H9D	5	0.37
West Bengal				ORF9b:M26I	2	6.67	ORF9b:H9D	4	7.55	ORF9b:N36Y	3	0.22
West Bengal				ORF9b:N28S	1	3.33	ORF9b:M8V	3	5.66	ORF9b:R32H	3	0.22
West Bengal				ORF9b:S6N	1	3.33	ORF9b:A57V	2	3.77	ORF9b:P10L	3	0.22
West Bengal				ORF9b:D2N	1	3.33	ORF9b:V37A	1	1.89	ORF9b:V30L	3	0.22
West Bengal				ORF9b:K4T	1	3.33	ORF9b:M78T	1	1.89	ORF9b:S6I	3	0.22
West Bengal				ORF9b:P43L	1	3.33	ORF9b:H9Y	1	1.89	ORF9b:V23L	2	0.15
West Bengal				ORF9b:T60N	1	3.33	ORF9b:E85D	1	1.89	ORF9b:Q18H	2	0.15
West Bengal				ORF9b:P17S	1	3.33	ORF9b:D2E	1	1.89	ORF9b:P3L	2	0.15
West Bengal				ORF9b:R13C	1	3.33	ORF9b:H9Q	1	1.89	ORF9b:A82V	2	0.15
West Bengal				ORF9b:V37F	1	3.33	ORF9b:R13C	1	1.89	ORF9b:R32P	1	0.07
West Bengal				ORF9b:P3S	1	3.33	ORF9b:R32C	1	1.89	ORF9b:R32C	1	0.07
West Bengal				ORF9b:K67E	1	3.33	ORF9b:D16G	1	1.89	ORF9b:P43L	1	0.07
West Bengal				ORF9b:P39L	1	3.33	ORF9b:R47L	1	1.89	ORF9b:N62S	1	0.07
West Bengal				ORF9b:R32L	1	3.33	ORF9b:M8I	1	1.89	ORF9b:D33N	1	0.07
West Bengal				ORF9b:P10S	1	3.33	ORF9b:E86D	1	1.89	ORF9b:G38D	1	0.07
West Bengal				ORF9b:Q18H	1	3.33	ORF9b:T72I	1	1.89	ORF9b:E86D	1	0.07
West Bengal				ORF9b:L64P	1	3.33				ORF9b:G31R	1	0.07
West Bengal										ORF9b:R13L	1	0.07
West Bengal										ORF9b:A29T	1	0.07
West Bengal										ORF9b:P39L	1	0.07
West Bengal										ORF9b:E86Q	1	0.07
West Bengal										ORF9b:L61S	1	0.07
West Bengal										ORF9b:M26I	1	0.07
West Bengal										ORF9b:K4N	1	0.07
West Bengal										ORF9b:K4Y	1	0.07
West Bengal										ORF9b:I5V	1	0.07
West Bengal										ORF9b:P51L	1	0.07
West Bengal										ORF9b:R47H	1	0.07
West Bengal	Amino acid deletions											
West Bengal	Pre-Wave 1			Wave 1			Pre-Wave 2			Wave 2		
West Bengal	Mutation	Count	percent	Mutation	Count	percent	Mutation	Count	percent	Mutation	Count	percent
West Bengal	ORF3a:V255-	2	40	S:Y145-	5	7.69	S:Y145-	121	13.6	ORF1a:S3675-	89	1.42
West Bengal	ORF1a:K141-	1	20	S:H146-	5	7.69	S:H146-	121	13.6	ORF1a:G3676-	89	1.42
West Bengal	ORF1a:S142-	1	20	ORF3a:V255-	4	6.15	ORF1a:S3675-	84	9.44	ORF1a:F3677-	89	1.42
West Bengal	ORF1a:F143-	1	20	S:Y144-	2	3.08	ORF1a:G3676-	84	9.44	S:H69-	81	1.29
West Bengal				ORF1a:G82-	2	3.08	ORF1a:F3677-	84	9.44	S:V70-	81	1.29
West Bengal				ORF1a:H83-	2	3.08	S:Y144-	80	8.99	S:Y144-	81	1.29
West Bengal				ORF1a:V84-	2	3.08	S:H69-	73	8.2	S:Y145-	27	0.43
West Bengal				ORF8:G66-	2	3.08	S:V70-	73	8.2	S:H146-	27	0.43
West Bengal				ORF8:S67-	2	3.08	S:L241-	10	1.12	ORF3a:M1-	11	0.18
West Bengal				ORF1a:I1023-	2	3.08	S:L242-	10	1.12	ORF3a:D2-	11	0.18
West Bengal				ORF1a:S2083-	2	3.08	S:A243-	10	1.12	ORF3a:L3-	11	0.18
West Bengal				S:H69-	1	1.54	ORF8:D119-	8	0.9	ORF3a:F4-	11	0.18
West Bengal				S:V70-	1	1.54	ORF8:F120-	8	0.9	ORF3a:V13-	11	0.18
West Bengal				ORF1a:K141-	1	1.54	S:E156-	6	0.67	ORF3a:T14-	11	0.18
West Bengal				ORF1a:S142-	1	1.54	S:F157-	6	0.67	ORF3a:L15-	11	0.18
West Bengal				ORF1a:F143-	1	1.54	ORF3a:V255-	5	0.56	ORF3a:K16-	11	0.18
West Bengal				ORF1a:E55-	1	1.54	ORF1a:M85-	4	0.45	ORF3a:Q17-	11	0.18
West Bengal				ORF1a:V56-	1	1.54	S:F140-	4	0.45	ORF3a:G18-	11	0.18
West Bengal				ORF1a:E57-	1	1.54	S:L141-	4	0.45	ORF3a:E19-	11	0.18
West Bengal				ORF1a:K58-	1	1.54	N:M1-	4	0.45	ORF3a:I35-	11	0.18
West Bengal				ORF1a:G59-	1	1.54	ORF6:M1-	4	0.45	ORF3a:P36-	11	0.18
West Bengal				ORF1a:V60-	1	1.54	ORF8:G66-	3	0.34	ORF3a:I37-	11	0.18
West Bengal				ORF1a:L61-	1	1.54	ORF8:S67-	3	0.34	ORF3a:Q38-	11	0.18
West Bengal				ORF1a:P62-	1	1.54	ORF7b:F13-	2	0.22	ORF3a:A39-	11	0.18
West Bengal				ORF1a:Q63-	1	1.54	ORF1a:V84-	2	0.22	S:E156-	1224	19.47
West Bengal				ORF1a:L64-	1	1.54	ORF1a:S4199-	2	0.22	S:F157-	1224	19.47
West Bengal				ORF7a:*122-	1	1.54	ORF8:E19-	1	0.11	ORF8:D119-	1271	20.22
West Bengal				ORF7a:Q62-	1	1.54	ORF7b:M1-	1	0.11	ORF8:F120-	1270	20.21
West Bengal				ORF1a:M85-	1	1.54	ORF7b:I2-	1	0.11	S:L241-	13	0.21
West Bengal				ORF1a:V86-	1	1.54	ORF7b:E3-	1	0.11	S:L242-	13	0.21
West Bengal				S:F140-	1	1.54	ORF7b:L4-	1	0.11	S:A243-	13	0.21
West Bengal				ORF1a:E1024-	1	1.54	ORF7b:S5-	1	0.11	ORF1a:D1089-	2	0.03
West Bengal				ORF1a:V1025-	1	1.54	ORF7b:L6-	1	0.11	ORF1a:Y1090-	2	0.03

West Bengal				ORF1a:N1026-	1	1.54	ORF7b:I7-	1	0.11	ORF1a:I1091-	2	0.03
West Bengal				ORF3a:V256-	1	1.54	ORF7b:D8-	1	0.11	ORF1a:A1092-	2	0.03
West Bengal				ORF3a:N257-	1	1.54	ORF7b:F9-	1	0.11	ORF1a:T1093-	2	0.03
West Bengal				ORF3a:P258-	1	1.54	ORF7b:Y10-	1	0.11	ORF1a:N1094-	2	0.03
West Bengal				ORF3a:V259-	1	1.54	ORF7b:L11-	1	0.11	ORF1a:G1095-	2	0.03
West Bengal				ORF3a:M260-	1	1.54	ORF8:V33-	1	0.11	ORF1a:P1096-	2	0.03
West Bengal				ORF3a:E261-	1	1.54	ORF8:D34-	1	0.11	ORF1a:L1097-	2	0.03
West Bengal				ORF3a:P262-	1	1.54	ORF8:D35-	1	0.11	ORF1a:K1098-	2	0.03
West Bengal				ORF3a:I263-	1	1.54	ORF8:P36-	1	0.11	ORF1a:V1099-	2	0.03
West Bengal				S:L242-	1	1.54	ORF8:C37-	1	0.11	ORF1a:G1100-	2	0.03
West Bengal				S:A243-	1	1.54	ORF8:P38-	1	0.11	ORF1a:G1101-	2	0.03
West Bengal				ORF7a:L102-	1	1.54	ORF8:I39-	1	0.11	ORF1a:S1102-	2	0.03
West Bengal				ORF7a:I103-	1	1.54	ORF8:H40-	1	0.11	ORF1a:C1103-	2	0.03
West Bengal							ORF8:F41-	1	0.11	ORF1a:V1104-	2	0.03
West Bengal							ORF8:Y42-	1	0.11	S:V143-	6	0.1
West Bengal							ORF8:S43-	1	0.11	N:M1-	5	0.08
West Bengal							ORF8:K44-	1	0.11	ORF6:M1-	5	0.08
West Bengal							ORF8:W45-	1	0.11	S:V126-	1	0.02
West Bengal							ORF8:R48-	1	0.11	ORF1a:K141-	7	0.11
West Bengal							ORF8:V49-	1	0.11	ORF1a:S142-	7	0.11
West Bengal							ORF8:G50-	1	0.11	ORF1a:F143-	7	0.11
West Bengal							ORF8:A51-	1	0.11	S:N196-	1	0.02
West Bengal							ORF8:R52-	1	0.11	S:F140-	2	0.03
West Bengal							ORF8:K53-	1	0.11	S:L141-	2	0.03
West Bengal							ORF8:S54-	1	0.11	S:G142-	4	0.06
West Bengal							ORF8:A55-	1	0.11	S:S151-	13	0.21
West Bengal							ORF8:P56-	1	0.11	S:W152-	10	0.16
West Bengal							ORF8:L57-	1	0.11	ORF1a:V84-	7	0.11
West Bengal							ORF8:I58-	1	0.11	ORF1a:M85-	10	0.16
West Bengal							ORF8:E59-	1	0.11	S:M153-	3	0.05
West Bengal							ORF8:L60-	1	0.11	ORF1a:N2131-	1	0.02
West Bengal							ORF8:C61-	1	0.11	ORF1a:S2132-	1	0.02
West Bengal							ORF8:I74-	1	0.11	ORF1a:V2133-	1	0.02
West Bengal							ORF8:D75-	1	0.11	ORF1a:P2134-	1	0.02
West Bengal							ORF8:I76-	1	0.11	ORF1a:W2135-	1	0.02
West Bengal							ORF8:G77-	1	0.11	ORF1a:D2136-	1	0.02
West Bengal							ORF8:N78-	1	0.11	ORF1a:T2137-	1	0.02
West Bengal							ORF8:Y79-	1	0.11	ORF1a:I2138-	1	0.02
West Bengal							ORF8:T80-	1	0.11	ORF1a:A2139-	1	0.02
West Bengal							ORF8:V81-	1	0.11	S:N149-	3	0.05
West Bengal							ORF8:F108-	1	0.11	S:K150-	7	0.11
West Bengal							ORF8:L109-	1	0.11	ORF7a:I88-	2	0.03
West Bengal							ORF8:E110-	1	0.11	ORF7a:R89-	2	0.03
West Bengal							ORF8:Y111-	1	0.11	ORF7a:Q90-	2	0.03
West Bengal							ORF8:H112-	1	0.11	ORF7a:E91-	2	0.03
West Bengal							ORF8:D113-	1	0.11	ORF7a:E92-	2	0.03
West Bengal							ORF8:V114-	1	0.11	ORF7a:V93-	2	0.03
West Bengal							ORF8:R115-	1	0.11	ORF7a:Q94-	2	0.03
West Bengal							ORF8:V116-	1	0.11	ORF7a:E95-	2	0.03
West Bengal							ORF8:V117-	1	0.11	ORF7a:L96-	2	0.03
West Bengal							ORF8:L118-	1	0.11	ORF7a:Y97-	2	0.03
West Bengal							ORF8:I121-	1	0.11	ORF7a:S98-	2	0.03
West Bengal							ORF8:*122-	1	0.11	ORF7a:P99-	2	0.03
West Bengal							S:N211-	1	0.11	S:N148-	1	0.02
West Bengal							ORF1a:K141-	1	0.11	S:T51-	2	0.03
West Bengal							ORF1a:S142-	1	0.11	S:Q52-	3	0.05
West Bengal							ORF1a:F143-	1	0.11	ORF7b:*44-	1	0.02
West Bengal							ORF1a:G82-	1	0.11	ORF8:M1-	1	0.02
West Bengal							ORF1a:H83-	1	0.11	ORF8:K2-	1	0.02
West Bengal							ORF1a:V86-	1	0.11	ORF8:F3-	1	0.02
West Bengal							ORF1a:S2114-	1	0.11	ORF8:L4-	1	0.02
West Bengal							ORF1a:R2115-	1	0.11	ORF8:V5-	1	0.02
West Bengal							ORF1a:V2116-	1	0.11	ORF8:L118-	1	0.02
West Bengal							ORF1a:S135-	1	0.11	S:Y91-	1	0.02
West Bengal							ORF1a:N1094-	1	0.11	S:F92-	1	0.02
West Bengal							S:S50-	1	0.11	ORF7a:I110-	6	0.1
West Bengal							S:T51-	1	0.11	ORF7a:I100-	3	0.05
West Bengal							ORF1a:N2081-	1	0.11	ORF7a:F101-	3	0.05
West Bengal										ORF7a:L102-	3	0.05
West Bengal										ORF7a:I103-	4	0.06
West Bengal										ORF7a:V104-	3	0.05
West Bengal										ORF7a:A105-	3	0.05
West Bengal										ORF7a:A106-	3	0.05

West Bengal										ORF7a:I107-	3	0.05
West Bengal										ORF7a:V108-	3	0.05
West Bengal										ORF7a:F109-	3	0.05
West Bengal										ORF7a:T111-	4	0.06
West Bengal										ORF7a:L112-	4	0.06
West Bengal										ORF7a:C113-	4	0.06
West Bengal										ORF7a:F114-	4	0.06
West Bengal										ORF7a:T115-	4	0.06
West Bengal										ORF7a:L116-	4	0.06
West Bengal										ORF7a:K117-	5	0.08
West Bengal										ORF7a:R118-	5	0.08
West Bengal										ORF7a:K119-	5	0.08
West Bengal										ORF7a:T120-	5	0.08
West Bengal										ORF7a:E121-	5	0.08
West Bengal										ORF7a:*122-	6	0.1
West Bengal										ORF7a:F54-	6	0.1
West Bengal										ORF7a:A55-	6	0.1
West Bengal										ORF7a:L56-	6	0.1
West Bengal										ORF7a:T57-	6	0.1
West Bengal										ORF7a:C58-	5	0.08
West Bengal										ORF7a:F59-	7	0.11
West Bengal										ORF7a:S60-	7	0.11
West Bengal										ORF7a:T61-	7	0.11
West Bengal										ORF7a:Q62-	7	0.11
West Bengal										ORF7a:F63-	7	0.11
West Bengal										ORF7a:A64-	7	0.11
West Bengal										ORF7a:F65-	5	0.08
West Bengal										ORF7a:A66-	5	0.08
West Bengal										ORF7a:C67-	5	0.08
West Bengal										ORF7a:P68-	5	0.08
West Bengal										ORF7a:D69-	5	0.08
West Bengal										ORF7a:G70-	5	0.08
West Bengal										ORF7a:V71-	5	0.08
West Bengal										ORF7a:K72-	5	0.08
West Bengal										ORF7a:H73-	5	0.08
West Bengal										ORF7a:V74-	5	0.08
West Bengal										ORF7a:Y75-	5	0.08
West Bengal										ORF7a:Q76-	5	0.08
West Bengal										ORF1a:G82-	5	0.08
West Bengal										ORF1a:H83-	6	0.1
West Bengal										ORF1a:V86-	6	0.1
West Bengal										S:E154-	7	0.11
West Bengal										S:S155-	6	0.1
West Bengal										ORF7a:C35-	1	0.02
West Bengal										ORF7a:S36-	1	0.02
West Bengal										ORF7a:S37-	1	0.02
West Bengal										ORF7a:G38-	1	0.02
West Bengal										ORF7a:T39-	1	0.02
West Bengal										ORF7a:Y40-	2	0.03
West Bengal										ORF7a:E41-	2	0.03
West Bengal										ORF7a:G42-	2	0.03
West Bengal										ORF7a:N43-	2	0.03
West Bengal										ORF7a:S44-	2	0.03
West Bengal										ORF7a:P45-	2	0.03
West Bengal										ORF7a:F46-	2	0.03
West Bengal										ORF7a:H47-	2	0.03
West Bengal										ORF7a:P48-	2	0.03
West Bengal										ORF7a:L49-	2	0.03
West Bengal										ORF7a:A50-	2	0.03
West Bengal										ORF7a:D51-	2	0.03
West Bengal										ORF7a:N52-	2	0.03
West Bengal										ORF7a:K53-	2	0.03
West Bengal										ORF7b:F13-	1	0.02
West Bengal										ORF7b:L14-	1	0.02
West Bengal										ORF7b:A15-	1	0.02
West Bengal										S:D53-	1	0.02
West Bengal										ORF7b:M1-	1	0.02
West Bengal										ORF7b:I2-	1	0.02
West Bengal										ORF7b:E3-	1	0.02
West Bengal										ORF7b:L4-	1	0.02
West Bengal										ORF7b:S5-	1	0.02
West Bengal										ORF7b:L6-	1	0.02
West Bengal										ORF7b:I7-	1	0.02

West Bengal										ORF7b:D8-	1	0.02
West Bengal										ORF8:I121-	1	0.02
West Bengal										ORF8:*122-	1	0.02
West Bengal										ORF6:K23-	1	0.02
West Bengal										ORF6:V24-	1	0.02
West Bengal										ORF6:S25-	1	0.02
West Bengal										ORF6:I26-	1	0.02
West Bengal										ORF6:W27-	1	0.02
West Bengal										ORF6:N28-	1	0.02
West Bengal										ORF6:L29-	1	0.02
West Bengal										ORF3a:V29-	1	0.02
West Bengal										ORF1a:N389-	1	0.02
West Bengal										ORF1a:E444-	1	0.02
West Bengal										ORF1a:G445-	1	0.02
West Bengal										ORF1a:L446-	1	0.02
West Bengal										S:I210-	1	0.02
West Bengal										S:S680-	1	0.02
West Bengal										ORF3a:V256-	1	0.02
West Bengal										ORF3a:N257-	1	0.02
West Bengal										ORF3a:P258-	1	0.02
West Bengal										ORF3a:V259-	1	0.02
West Bengal										ORF3a:M260-	1	0.02
West Bengal										ORF3a:E261-	1	0.02
West Bengal										ORF3a:P262-	1	0.02
West Bengal										ORF3a:I263-	1	0.02
West Bengal										ORF8:G66-	1	0.02
West Bengal										ORF8:S67-	1	0.02
West Bengal										N:G214-	2	0.03
West Bengal										N:G215-	2	0.03
West Bengal										ORF1a:S2083-	1	0.02
West Bengal										ORF1a:D1088-	1	0.02
West Bengal										S:L244-	1	0.02
West Bengal										ORF1a:E1024-	1	0.02
West Bengal										ORF1a:V1025-	1	0.02
West Bengal										ORF1a:N1026-	1	0.02
West Bengal										S:R44-	1	0.02
West Bengal										S:S45-	1	0.02
West Bengal										ORF7a:L77-	1	0.02
**West Bengal**	**Nucleotide deletions**											
**West Bengal**	**Pre-Wave 1**			**Wave 1**			**Pre-Wave 2**			**Wave 2**		
West Bengal	4	7	38.89	21993-21998	5	10.2	28271	162	26.47	28271	1885	38.55
West Bengal	26155-26157	2	11.11	26155-26157	4	8.16	21993-21998	121	19.77	28248-28253	1274	26.05
West Bengal	29	1	5.56	29725-29775	3	6.12	11288-11296	84	13.73	22029-22034	1229	25.13
West Bengal	2249	1	5.56	27792-27793	2	4.08	21992-21994	80	13.07	11288-11296	89	1.82
West Bengal	686-694	1	5.56	21992-21994	2	4.08	21765-21770	73	11.93	21992-21994	84	1.72
West Bengal	29869	1	5.56	28090-28095	2	4.08	22283-22291	10	1.63	21765-21770	81	1.66
West Bengal	29680	1	5.56	29729-29730	2	4.08	28248-28253	7	1.14	21993-21998	27	0.55
West Bengal	15-16	1	5.56	29726-29727	2	4.08	26158-26161	6	0.98	22283-22291	13	0.27
West Bengal	29752-29754	1	5.56	6513-6515	2	4.08	22029-22034	6	0.98	25448-25476	11	0.22
West Bengal	29759-29768	1	5.56	21765-21770	1	2.04	29581	5	0.82	25519	11	0.22
West Bengal	29738-29761	1	5.56	686-694	1	2.04	26159-26161	5	0.82	22013-22018	11	0.22
West Bengal				428-457	1	2.04	21982-21985	4	0.65	27578-27581	9	0.18
West Bengal				27529-27531	1	2.04	21992-21993	4	0.65	29574-29580	8	0.16
West Bengal				27579-27581	1	2.04	27205-27207	4	0.65	686-694	7	0.14
West Bengal				29738-29748	1	2.04	28278-28280	4	0.65	27205-27207	5	0.1
West Bengal				29749-29755	1	2.04	28090-28095	3	0.49	28278-28280	5	0.1
West Bengal				29747-29758	1	2.04	27792-27794	2	0.33	27553-27621	5	0.1
West Bengal				29726	1	2.04	27721-27722	2	0.33	509-523	5	0.1
West Bengal				509-523	1	2.04	29728-29776	2	0.33	241	4	0.08
West Bengal				21980-21982	1	2.04	12861-12863	2	0.33	29728-29776	4	0.08
West Bengal				3333-3335	1	2.04	29660	1	0.16	22025-22030	4	0.08
West Bengal				27694-27700	1	2.04	29726-29727	1	0.16	22016-22021	3	0.06
West Bengal				26159-26160	1	2.04	27948-27950	1	0.16	22010-22015	3	0.06
West Bengal				29728-29776	1	2.04	27762-27794	1	0.16	28011-28015	3	0.06
West Bengal				510-518	1	2.04	27792-27793	1	0.16	516-518	3	0.06
West Bengal				3332-3343	1	2.04	516-518	1	0.16	29756-29762	3	0.06
West Bengal				29	1	2.04	28254	1	0.16	21982-21985	2	0.04
West Bengal				26158-26181	1	2.04	27982-28004	1	0.16	21992-21993	2	0.04
West Bengal				29819-29822	1	2.04	28251-28254	1	0.16	21987-21995	2	0.04
West Bengal				26161-26177	1	2.04	22194-22196	1	0.16	28248	2	0.04
West Bengal				27721-27722	1	2.04	686-694	1	0.16	21716-21721	2	0.04

West Bengal				28254	1	2.04	29750-29751	1	0.16	27553-27623	2	0.04
West Bengal				22289-22294	1	2.04	518-520	1	0.16	27721-27723	2	0.04
West Bengal				27697-27702	1	2.04	509-523	1	0.16	27511-27579	2	0.04
West Bengal							29727-29747	1	0.16	27511-27581	2	0.04
West Bengal							6605-6613	1	0.16	27568-27585	2	0.04
West Bengal							515-520	1	0.16	22028-22033	2	0.04
West Bengal							669-671	1	0.16	28913-28918	2	0.04
West Bengal							27578-27581	1	0.16	29738-29748	1	0.02
West Bengal							27694-27697	1	0.16	3531-3578	1	0.02
West Bengal							3546-3548	1	0.16	21983-21985	1	0.02
West Bengal							21713-21718	1	0.16	22148-22150	1	0.02
West Bengal							6506-6508	1	0.16	28137-28150	1	0.02
West Bengal							27795-27801	1	0.16	515-520	1	0.02
West Bengal							28252-28256	1	0.16	6656-6682	1	0.02
West Bengal							241	1	0.16	22010	1	0.02
West Bengal										27659-27694	1	0.02
West Bengal										27796-27797	1	0.02
West Bengal										22009-22014	1	0.02
West Bengal										21990-21992	1	0.02
West Bengal										27883-27910	1	0.02
West Bengal										21833-21838	1	0.02
West Bengal										26479-26485	1	0.02
West Bengal										27496-27564	1	0.02
West Bengal										28248-28251	1	0.02
West Bengal										27556-27586	1	0.02
West Bengal										29743	1	0.02
West Bengal										29780-29787	1	0.02
West Bengal										29792-29800	1	0.02
West Bengal										27792-27793	1	0.02
West Bengal										27792-27800	1	0.02
West Bengal										240-241	1	0.02
West Bengal										21719-21724	1	0.02
West Bengal										27655-27781	1	0.02
West Bengal										27409	1	0.02
West Bengal										28248-28259	1	0.02
West Bengal										27553-27563	1	0.02
West Bengal										27586-27639	1	0.02
West Bengal										29730	1	0.02
West Bengal										27700-27702	1	0.02
West Bengal										27512-27515	1	0.02
West Bengal										27268-27288	1	0.02
West Bengal										27526-27543	1	0.02
West Bengal										22008-22009	1	0.02
West Bengal										22007	1	0.02
West Bengal										29746-29748	1	0.02
West Bengal										27514-27544	1	0.02
West Bengal										22021-22026	1	0.02
West Bengal										25477-25479	1	0.02
West Bengal										21743-21781	1	0.02
West Bengal										21887-21891	1	0.02
West Bengal										27517-27519	1	0.02
West Bengal										29730-29759	1	0.02
West Bengal										26158-26161	1	0.02
West Bengal										22022-22027	1	0.02
West Bengal										1431-1433	1	0.02
West Bengal										1596-1604	1	0.02
West Bengal										21556-21561	1	0.02
West Bengal										22190-22192	1	0.02
West Bengal										23135	1	0.02
West Bengal										23603-23605	1	0.02
West Bengal										29378-29379	1	0.02
West Bengal										26158-26181	1	0.02
West Bengal										28090-28095	1	0.02
West Bengal										27565-27587	1	0.02
West Bengal										27390-27395	1	0.02
West Bengal										512-523	1	0.02
West Bengal										27556-27617	1	0.02
West Bengal										6513-6515	1	0.02
West Bengal										29727-29747	1	0.02
West Bengal										3528-3578	1	0.02
West Bengal										27555-27578	1	0.02
West Bengal										27609-27628	1	0.02
West Bengal										27511-27512	1	0.02

West Bengal										22292-22294	1	0.02
West Bengal										27526-27548	1	0.02
West Bengal										3336-3344	1	0.02
West Bengal										27701-27740	1	0.02
West Bengal										21692-21697	1	0.02
West Bengal										22016-22019	1	0.02
West Bengal										27512	1	0.02
West Bengal										3839-3840	1	0.02
West Bengal										27511-27554	1	0.02
West Bengal										27703-27741	1	0.02
West Bengal										27792	1	0.02
West Bengal										29680-29681	1	0.02
West Bengal										27250-27253	1	0.02
West Bengal										22014	1	0.02
West Bengal												
**Delhi**	**ORF1a**											
**Delhi**	**Pre-Wave 1**			**Wave 1**			**Pre-Wave 2**			**Wave 2**		
Delhi	**Mutation**	**Count**	**percent**	**Mutation**	**Count**	**percent**	**Mutation**	**Count**	**percent**	**Mutation**	**Count**	**percent**
Delhi	ORF1a:T2016K	188	57.32	ORF1a:V561A	442	46.04	ORF1a:A1708D	151	25.34	ORF1a:P1640L	426	34.33
Delhi	ORF1a:L3606F	92	28.05	ORF1a:V1056L	110	11.46	ORF1a:T1001I	149	25	ORF1a:A3209V	407	32.8
Delhi	ORF1a:V561A	39	11.89	ORF1a:L3935M	82	8.54	ORF1a:I2230T	131	21.98	ORF1a:V3718A	406	32.72
Delhi	ORF1a:S2015R	10	3.05	ORF1a:D3042N	64	6.67	ORF1a:T1567I	48	8.05	ORF1a:P309L	350	28.2
Delhi	ORF1a:A339V	8	2.44	ORF1a:L3606F	59	6.15	ORF1a:T3646A	47	7.89	ORF1a:H2092Y	274	22.08
Delhi	ORF1a:V2586G	4	1.22	ORF1a:A4016V	56	5.83	ORF1a:V561A	46	7.72	ORF1a:T1001I	214	17.24
Delhi	ORF1a:S481F	4	1.22	ORF1a:P309S	52	5.42	ORF1a:L730F	31	5.2	ORF1a:A1708D	213	17.16
Delhi	ORF1a:G519S	4	1.22	ORF1a:V3759F	50	5.21	ORF1a:T2007I	29	4.87	ORF1a:T3646A	207	16.68
Delhi	ORF1a:Q4140H	3	0.91	ORF1a:M3752I	49	5.1	ORF1a:T760I	25	4.19	ORF1a:I2230T	203	16.36
Delhi	ORF1a:T265I	2	0.61	ORF1a:V3718F	38	3.96	ORF1a:L4182F	25	4.19	ORF1a:T1567I	126	10.15
Delhi	ORF1a:T436I	2	0.61	ORF1a:Q3777R	37	3.85	ORF1a:K3353R	15	2.52	ORF1a:L4182F	98	7.9
Delhi	ORF1a:A2139V	2	0.61	ORF1a:A1812D	33	3.44	ORF1a:K1655N	14	2.35	ORF1a:T760I	97	7.82
Delhi	ORF1a:T1731A	2	0.61	ORF1a:S302F	30	3.12	ORF1a:P3359L	13	2.18	ORF1a:T3255I	96	7.74
Delhi	ORF1a:V2061F	2	0.61	ORF1a:T1001I	29	3.02	ORF1a:Q3729K	13	2.18	ORF1a:P2046L	92	7.41
Delhi	ORF1a:A903V	2	0.61	ORF1a:T1794P	18	1.88	ORF1a:A3958V	13	2.18	ORF1a:A1306S	91	7.33
Delhi	ORF1a:T4065I	2	0.61	ORF1a:K3353R	17	1.77	ORF1a:T265I	13	2.18	ORF1a:P2287S	91	7.33
Delhi	ORF1a:I739V	2	0.61	ORF1a:V3653F	17	1.77	ORF1a:A3209V	12	2.01	ORF1a:V2930L	90	7.25
Delhi	ORF1a:P765S	2	0.61	ORF1a:A1078T	16	1.67	ORF1a:M3752I	9	1.51	ORF1a:T3750I	59	4.75
Delhi	ORF1a:Y3160H	1	0.3	ORF1a:V2061F	15	1.56	ORF1a:V1056L	8	1.34	ORF1a:L730F	40	3.22
Delhi	ORF1a:G1125S	1	0.3	ORF1a:G519S	12	1.25	ORF1a:L3606F	8	1.34	ORF1a:T2007I	31	2.5
Delhi	ORF1a:T1168I	1	0.3	ORF1a:L3116F	12	1.25	ORF1a:V54I	7	1.17	ORF1a:K3353R	22	1.77
Delhi	ORF1a:N2222Y	1	0.3	ORF1a:A405V	11	1.15	ORF1a:S302F	7	1.17	ORF1a:K261N	14	1.13
Delhi	ORF1a:K58R	1	0.3	ORF1a:P1640S	11	1.15	ORF1a:P309S	6	1.01	ORF1a:T265I	13	1.05
Delhi	ORF1a:A2994V	1	0.3	ORF1a:T1788M	11	1.15	ORF1a:A1812D	6	1.01	ORF1a:K1655N	13	1.05
Delhi	ORF1a:I4334V	1	0.3	ORF1a:T3032I	11	1.15	ORF1a:P309L	6	1.01	ORF1a:T609I	10	0.81
Delhi	ORF1a:S135N	1	0.3	ORF1a:A3730S	10	1.04	ORF1a:L3935M	5	0.84	ORF1a:L3606F	9	0.73
Delhi	ORF1a:S2500F	1	0.3	ORF1a:T3665I	10	1.04	ORF1a:D3042N	5	0.84	ORF1a:A2587V	9	0.73
Delhi	ORF1a:M3921I	1	0.3	ORF1a:V3968F	10	1.04	ORF1a:A2587V	5	0.84	ORF1a:P193L	9	0.73
Delhi	ORF1a:R398G	1	0.3	ORF1a:M2259I	10	1.04	ORF1a:A4016V	5	0.84	ORF1a:L3829F	8	0.64
Delhi	ORF1a:R24C	1	0.3	ORF1a:K141N	10	1.04	ORF1a:V3718A	5	0.84	ORF1a:E1564A	8	0.64
Delhi	ORF1a:E441K	1	0.3	ORF1a:Y576C	10	1.04	ORF1a:S1612L	5	0.84	ORF1a:L3116F	7	0.56
Delhi	ORF1a:A3697V	1	0.3	ORF1a:V2862L	10	1.04	ORF1a:V3718F	5	0.84	ORF1a:A1983V	7	0.56
Delhi	ORF1a:P1054L	1	0.3	ORF1a:S2500F	9	0.94	ORF1a:F2780L	4	0.67	ORF1a:D2980N	7	0.56
Delhi	ORF1a:D1323Y	1	0.3	ORF1a:L3754F	9	0.94	ORF1a:K2511N	4	0.67	ORF1a:F3138S	7	0.56
Delhi	ORF1a:I3618V	1	0.3	ORF1a:K1655N	9	0.94	ORF1a:S2488F	4	0.67	ORF1a:H3580Q	7	0.56
Delhi	ORF1a:S318L	1	0.3	ORF1a:I573T	9	0.94	ORF1a:V3759F	4	0.67	ORF1a:K2589R	7	0.56
Delhi	ORF1a:I2076V	1	0.3	ORF1a:M3221I	9	0.94	ORF1a:N1662S	4	0.67	ORF1a:D48N	7	0.56
Delhi	ORF1a:L642F	1	0.3	ORF1a:D928Y	8	0.83	ORF1a:A3730S	4	0.67	ORF1a:S944L	6	0.48
Delhi	ORF1a:S1272G	1	0.3	ORF1a:G989V	8	0.83	ORF1a:V2061F	4	0.67	ORF1a:K2771N	6	0.48
Delhi	ORF1a:H2357Y	1	0.3	ORF1a:V2613F	8	0.83	ORF1a:P1220L	4	0.67	ORF1a:I3142V	6	0.48
Delhi	ORF1a:S3244L	1	0.3	ORF1a:N1094I	7	0.73	ORF1a:A967V	4	0.67	ORF1a:M3752I	6	0.48
Delhi	ORF1a:L3736F	1	0.3	ORF1a:S142P	7	0.73	ORF1a:E995D	4	0.67	ORF1a:T2181I	6	0.48
Delhi	ORF1a:A2344T	1	0.3	ORF1a:E2112D	7	0.73	ORF1a:T4090I	4	0.67	ORF1a:L4391I	6	0.48
Delhi	ORF1a:Q556K	1	0.3	ORF1a:H388Y	7	0.73	ORF1a:K2589R	4	0.67	ORF1a:T3202M	5	0.4
Delhi	ORF1a:S40A	1	0.3	ORF1a:K2589R	7	0.73	ORF1a:G445V	3	0.5	ORF1a:K680N	5	0.4
Delhi	ORF1a:M3087I	1	0.3	ORF1a:P309L	7	0.73	ORF1a:V1291I	3	0.5	ORF1a:T1881I	5	0.4
Delhi	ORF1a:A2123V	1	0.3	ORF1a:T3255I	7	0.73	ORF1a:N3537D	3	0.5	ORF1a:A2344V	5	0.4
Delhi	ORF1a:P309L	1	0.3	ORF1a:P971S	7	0.73	ORF1a:A2320V	3	0.5	ORF1a:T2648I	5	0.4
Delhi	ORF1a:D144Y	1	0.3	ORF1a:H81Y	7	0.73	ORF1a:Q3966R	3	0.5	ORF1a:M3087I	5	0.4
Delhi	ORF1a:Q998H	1	0.3	ORF1a:I2230T	7	0.73	ORF1a:A486V	3	0.5	ORF1a:F2780L	5	0.4
Delhi	ORF1a:P1640S	1	0.3	ORF1a:P2221S	7	0.73	ORF1a:T1429I	3	0.5	ORF1a:T1638I	5	0.4
Delhi	ORF1a:G1531C	1	0.3	ORF1a:V2588F	6	0.62	ORF1a:E347D	3	0.5	ORF1a:T350I	5	0.4

Delhi	**ORF1a:G82W**	1	0.3	**ORF1a:Q1021H**	6	0.62	**ORF1a:T3255I**	3	0.5	**ORF1a:T2274I**	4	0.32
Delhi	**ORF1a:M1014I**	1	0.3	**ORF1a:A1352V**	6	0.62	**ORF1a:M1378I**	3	0.5	**ORF1a:D3936N**	4	0.32
Delhi	**ORF1a:V2453G**	1	0.3	**ORF1a:I453V**	6	0.62	**ORF1a:V2588F**	3	0.5	**ORF1a:T3488I**	4	0.32
Delhi	**ORF1a:N2454S**	1	0.3	**ORF1a:T4090I**	6	0.62	**ORF1a:A2249V**	3	0.5	**ORF1a:K2511N**	4	0.32
Delhi	**ORF1a:V3759F**	1	0.3	**ORF1a:M3616I**	6	0.62	**ORF1a:P971L**	3	0.5	**ORF1a:G445V**	4	0.32
Delhi	**ORF1a:S248G**	1	0.3	**ORF1a:T224I**	6	0.62	**ORF1a:K2497N**	3	0.5	**ORF1a:P2018L**	4	0.32
Delhi	**ORF1a:H1113Y**	1	0.3	**ORF1a:T4065I**	6	0.62	**ORF1a:Q2563H**	3	0.5	**ORF1a:V3349I**	4	0.32
Delhi	**ORF1a:P1921S**	1	0.3	**ORF1a:A4136V**	6	0.62	**ORF1a:D48N**	3	0.5	**ORF1a:E2112A**	4	0.32
Delhi	**ORF1a:S2972P**	1	0.3	**ORF1a:A903V**	6	0.62	**ORF1a:T350I**	3	0.5	**ORF1a:L580F**	4	0.32
Delhi	**ORF1a:L3919F**	1	0.3	**ORF1a:P4075S**	6	0.62	**ORF1a:I404T**	3	0.5	**ORF1a:A819T**	4	0.32
Delhi	**ORF1a:T1035I**	1	0.3	**ORF1a:P1158S**	6	0.62	**ORF1a:T708I**	3	0.5	**ORF1a:A3457S**	4	0.32
Delhi	**ORF1a:A591V**	1	0.3	**ORF1a:P1786S**	6	0.62	**ORF1a:V1768D**	3	0.5	**ORF1a:A3686V**	4	0.32
Delhi	**ORF1a:D928Y**	1	0.3	**ORF1a:A1708D**	6	0.62	**ORF1a:M1769N**	3	0.5	**ORF1a:S1612L**	4	0.32
Delhi	**ORF1a:T2906I**	1	0.3	**ORF1a:T4364I**	6	0.62	**ORF1a:Y1770P**	3	0.5	**ORF1a:V3718F**	4	0.32
Delhi	**ORF1a:L4111F**	1	0.3	**ORF1a:T1168I**	5	0.52	**ORF1a:M1771***	3	0.5	**ORF1a:A1283V**	4	0.32
Delhi	**ORF1a:G112D**	1	0.3	**ORF1a:P959S**	5	0.52	**ORF1a:L1797D**	3	0.5	**ORF1a:V54I**	4	0.32
Delhi	**ORF1a:S2188F**	1	0.3	**ORF1a:P971L**	5	0.52	**ORF1a:T1820S**	3	0.5	**ORF1a:V561A**	4	0.32
Delhi	**ORF1a:A2696V**	1	0.3	**ORF1a:S2242F**	5	0.52	**ORF1a:F1821T**	3	0.5	**ORF1a:N1662S**	4	0.32
Delhi	**ORF1a:V1117I**	1	0.3	**ORF1a:K1202N**	5	0.52	**ORF1a:A1824W**	3	0.5	**ORF1a:T4031I**	4	0.32
Delhi	**ORF1a:P1321S**	1	0.3	**ORF1a:S3256T**	5	0.52	**ORF1a:E1826L**	3	0.5	**ORF1a:G400S**	3	0.24
Delhi	**ORF1a:T2648I**	1	0.3	**ORF1a:D981Y**	5	0.52	**ORF1a:Y1827***	3	0.5	**ORF1a:P971L**	3	0.24
Delhi	**ORF1a:V3975F**	1	0.3	**ORF1a:S318L**	5	0.52	**ORF1a:G1829Y**	3	0.5	**ORF1a:L3338F**	3	0.24
Delhi	**ORF1a:V830A**	1	0.3	**ORF1a:S1515F**	5	0.52	**ORF1a:Y1831N**	3	0.5	**ORF1a:A1812D**	3	0.24
Delhi	**ORF1a:K2741E**	1	0.3	**ORF1a:D2060N**	5	0.52	**ORF1a:Q1832F**	3	0.5	**ORF1a:A964V**	3	0.24
Delhi	**ORF1a:I831L**	1	0.3	**ORF1a:H110Y**	5	0.52	**ORF1a:C1833V**	3	0.5	**ORF1a:A1670T**	3	0.24
Delhi	**ORF1a:K2204Q**	1	0.3	**ORF1a:G7V**	5	0.52	**ORF1a:G1834L**	3	0.5	**ORF1a:T1474I**	3	0.24
Delhi	**ORF1a:E832K**	1	0.3	**ORF1a:G493C**	5	0.52	**ORF1a:Y1836R**	3	0.5	**ORF1a:S428G**	3	0.24
Delhi	**ORF1a:N2405D**	1	0.3	**ORF1a:G524V**	4	0.42	**ORF1a:K1837R**	3	0.5	**ORF1a:S2396I**	3	0.24
Delhi	**ORF1a:E1687D**	1	0.3	**ORF1a:T2154I**	4	0.42	**ORF1a:H1838C**	3	0.5	**ORF1a:Y3722C**	3	0.24
Delhi	**ORF1a:D846N**	1	0.3	**ORF1a:A3615V**	4	0.42	**ORF1a:I1839F**	3	0.5	**ORF1a:L747F**	3	0.24
Delhi	**ORF1a:S2625F**	1	0.3	**ORF1a:S2104N**	4	0.42	**ORF1a:S1841Y**	3	0.5	**ORF1a:P3767S**	3	0.24
Delhi	**ORF1a:I3341V**	1	0.3	**ORF1a:P2079S**	4	0.42	**ORF1a:F1867L**	3	0.5	**ORF1a:L3027F**	3	0.24
Delhi	**ORF1a:I3052V**	1	0.3	**ORF1a:P2685T**	4	0.42	**ORF1a:Y1868R**	3	0.5	**ORF1a:T4083M**	3	0.24
Delhi	**ORF1a:L3845F**	1	0.3	**ORF1a:L3711F**	4	0.42	**ORF1a:K1869I**	3	0.5	**ORF1a:A1397V**	3	0.24
Delhi	**ORF1a:R3069T**	1	0.3	**ORF1a:T1542I**	4	0.42	**ORF1a:E1870Q**	3	0.5	**ORF1a:K856R**	3	0.24
Delhi	**ORF1a:F3071Y**	1	0.3	**ORF1a:I201V**	4	0.42	**ORF1a:N1871R**	3	0.5	**ORF1a:M3655I**	3	0.24
Delhi	**ORF1a:C1114F**	1	0.3	**ORF1a:P2144L**	4	0.42	**ORF1a:T1881S**	3	0.5	**ORF1a:L3667F**	3	0.24
Delhi	**ORF1a:V1798L**	1	0.3	**ORF1a:T1881I**	4	0.42	**ORF1a:K1883L**	3	0.5	**ORF1a:T3284I**	3	0.24
Delhi	**ORF1a:T2777I**	1	0.3	**ORF1a:A2128D**	4	0.42	**ORF1a:L1884***	3	0.5	**ORF1a:G728C**	3	0.24
Delhi	**ORF1a:P971S**	1	0.3	**ORF1a:A591V**	4	0.42	**ORF1a:D1885I**	3	0.5	**ORF1a:E1244K**	3	0.24
Delhi	**ORF1a:T1794I**	1	0.3	**ORF1a:I2076T**	4	0.42	**ORF1a:V1887W**	3	0.5	**ORF1a:K2078E**	3	0.24
Delhi	**ORF1a:S2114P**	1	0.3	**ORF1a:G1155C**	4	0.42	**ORF1a:V1888C**	3	0.5	**ORF1a:A2529V**	2	0.16
Delhi				**ORF1a:K3832N**	4	0.42	**ORF1a:E1954W**	3	0.5	**ORF1a:S3099L**	2	0.16
Delhi				**ORF1a:S2981F**	4	0.42	**ORF1a:K1956***	3	0.5	**ORF1a:S2193F**	2	0.16
Delhi				**ORF1a:S2625F**	4	0.42	**ORF1a:V1957E**	3	0.5	**ORF1a:P2079L**	2	0.16
Delhi				**ORF1a:A3143V**	4	0.42	**ORF1a:F1959C**	3	0.5	**ORF1a:I581L**	2	0.16
Delhi				**ORF1a:M1378I**	4	0.42	**ORF1a:K1988S**	3	0.5	**ORF1a:M85V**	2	0.16
Delhi				**ORF1a:Q3777H**	4	0.42	**ORF1a:P1989***	3	0.5	**ORF1a:K120N**	2	0.16
Delhi				**ORF1a:M3934I**	4	0.42	**ORF1a:W1992S**	3	0.5	**ORF1a:T4355I**	2	0.16
Delhi				**ORF1a:I1002S**	4	0.42	**ORF1a:S2015***	3	0.5	**ORF1a:N2256H**	2	0.16
Delhi				**ORF1a:M3669I**	4	0.42	**ORF1a:P2018L**	3	0.5	**ORF1a:S376P**	2	0.16
Delhi				**ORF1a:T4031I**	4	0.42	**ORF1a:E2020Y**	3	0.5	**ORF1a:M1378I**	2	0.16
Delhi				**ORF1a:P1803L**	4	0.42	**ORF1a:S2022G**	3	0.5	**ORF1a:A555V**	2	0.16
Delhi				**ORF1a:A2325T**	4	0.42	**ORF1a:E2049***	3	0.5	**ORF1a:S2048F**	2	0.16
Delhi				**ORF1a:V2943F**	4	0.42	**ORF1a:E2050V**	3	0.5	**ORF1a:F3677L**	2	0.16
Delhi				**ORF1a:R550C**	4	0.42	**ORF1a:V2051***	3	0.5	**ORF1a:R184C**	2	0.16
Delhi				**ORF1a:A1306T**	4	0.42	**ORF1a:V2052C**	3	0.5	**ORF1a:S2030L**	2	0.16
Delhi				**ORF1a:V3056I**	4	0.42	**ORF1a:A2080S**	3	0.5	**ORF1a:T283I**	2	0.16
Delhi				**ORF1a:T708I**	4	0.42	**ORF1a:N2082G**	3	0.5	**ORF1a:E347D**	2	0.16
Delhi				**ORF1a:I3052V**	4	0.42	**ORF1a:S2083C**	3	0.5	**ORF1a:P4223L**	2	0.16
Delhi				**ORF1a:T2202S**	4	0.42	**ORF1a:N2111S**	3	0.5	**ORF1a:S4398L**	2	0.16
Delhi				**ORF1a:M3087I**	4	0.42	**ORF1a:E2112W**	3	0.5	**ORF1a:S610L**	2	0.16
Delhi				**ORF1a:P2134S**	3	0.31	**ORF1a:L2113F**	3	0.5	**ORF1a:H2794Y**	2	0.16
Delhi				**ORF1a:L1130F**	3	0.31	**ORF1a:T2166Y**	3	0.5	**ORF1a:E256D**	2	0.16
Delhi				**ORF1a:H1113R**	3	0.31	**ORF1a:N2167F**	3	0.5	**ORF1a:P3371S**	2	0.16
Delhi				**ORF1a:R3993L**	3	0.31	**ORF1a:M2169***	3	0.5	**ORF1a:S1433L**	2	0.16
Delhi				**ORF1a:H2125Y**	3	0.31	**ORF1a:P2170K**	3	0.5	**ORF1a:G514V**	2	0.16
Delhi				**ORF1a:A427V**	3	0.31	**ORF1a:F2172K**	3	0.5	**ORF1a:T3959N**	2	0.16
Delhi				**ORF1a:I404T**	3	0.31	**ORF1a:T2186***	3	0.5	**ORF1a:D75N**	2	0.16
Delhi				**ORF1a:K2029N**	3	0.31	**ORF1a:S2188***	3	0.5	**ORF1a:E913D**	2	0.16
Delhi				**ORF1a:G295C**	3	0.31	**ORF1a:R2189S**	3	0.5	**ORF1a:S383N**	2	0.16
Delhi				**ORF1a:P1497L**	3	0.31	**ORF1a:E2268C**	3	0.5	**ORF1a:V86I**	2	0.16
Delhi				**ORF1a:G445V**	3	0.31	**ORF1a:G2269H**	3	0.5	**ORF1a:S2900L**	2	0.16

Delhi				**ORF1a:T1474I**	3	0.31	**ORF1a:L2271C**	3	0.5	**ORF1a:K1202N**	2	0.16
Delhi				**ORF1a:S4398L**	3	0.31	**ORF1a:S2273L**	3	0.5	**ORF1a:Y1064C**	2	0.16
Delhi				**ORF1a:T2952I**	3	0.31	**ORF1a:T2274L**	3	0.5	**ORF1a:P1207S**	2	0.16
Delhi				**ORF1a:V2580F**	3	0.31	**ORF1a:N2275Y**	3	0.5	**ORF1a:M1647I**	2	0.16
Delhi				**ORF1a:S3675F**	3	0.31	**ORF1a:V2276W**	3	0.5	**ORF1a:M2365I**	2	0.16
Delhi				**ORF1a:L3338F**	3	0.31	**ORF1a:L2349T**	3	0.5	**ORF1a:P3359S**	2	0.16
Delhi				**ORF1a:M3655I**	3	0.31	**ORF1a:F2351Y**	3	0.5	**ORF1a:P309S**	2	0.16
Delhi				**ORF1a:K292N**	3	0.31	**ORF1a:S2352***	3	0.5	**ORF1a:P80H**	2	0.16
Delhi				**ORF1a:K1781R**	3	0.31	**ORF1a:Y2353***	3	0.5	**ORF1a:K292N**	2	0.16
Delhi				**ORF1a:D1167G**	3	0.31	**ORF1a:Q2373H**	3	0.5	**ORF1a:K669E**	2	0.16
Delhi				**ORF1a:I4049V**	3	0.31	**ORF1a:M2374L**	3	0.5	**ORF1a:E1007V**	2	0.16
Delhi				**ORF1a:A1049V**	3	0.31	**ORF1a:A2375L**	3	0.5	**ORF1a:K2622N**	2	0.16
Delhi				**ORF1a:G392D**	3	0.31	**ORF1a:P2376C**	3	0.5	**ORF1a:L4386F**	2	0.16
Delhi				**ORF1a:L3027F**	3	0.31	**ORF1a:V2401I**	3	0.5	**ORF1a:E2049D**	2	0.16
Delhi				**ORF1a:A3657D**	3	0.31	**ORF1a:D2402L**	3	0.5	**ORF1a:I2812V**	2	0.16
Delhi				**ORF1a:A1473V**	3	0.31	**ORF1a:G2403L**	3	0.5	**ORF1a:T1246I**	2	0.16
Delhi				**ORF1a:T4175I**	3	0.31	**ORF1a:S2406K**	3	0.5	**ORF1a:A3610V**	2	0.16
Delhi				**ORF1a:S1189G**	3	0.31	**ORF1a:S2433R**	3	0.5	**ORF1a:M2259I**	2	0.16
Delhi				**ORF1a:P3952S**	3	0.31	**ORF1a:F2434M**	3	0.5	**ORF1a:T1168I**	2	0.16
Delhi				**ORF1a:T1035I**	3	0.31	**ORF1a:V2436N**	3	0.5	**ORF1a:E1196V**	2	0.16
Delhi				**ORF1a:L681F**	3	0.31	**ORF1a:A2460L**	3	0.5	**ORF1a:A2098T**	2	0.16
Delhi				**ORF1a:A2508T**	3	0.31	**ORF1a:G2461V**	3	0.5	**ORF1a:T2936I**	2	0.16
Delhi				**ORF1a:Y3722C**	3	0.31	**ORF1a:S2462T**	3	0.5	**ORF1a:V1483A**	2	0.16
Delhi				**ORF1a:A3523V**	3	0.31	**ORF1a:I2481V**	3	0.5	**ORF1a:L2122F**	2	0.16
Delhi				**ORF1a:K141R**	3	0.31	**ORF1a:N2482***	3	0.5	**ORF1a:P1786L**	2	0.16
Delhi				**ORF1a:P3371S**	3	0.31	**ORF1a:P2483K**	3	0.5	**ORF1a:H374Y**	2	0.16
Delhi				**ORF1a:L730F**	3	0.31	**ORF1a:D2485N**	3	0.5	**ORF1a:L3588F**	2	0.16
Delhi				**ORF1a:N1749S**	3	0.31	**ORF1a:Q2486K**	3	0.5	**ORF1a:P1096L**	2	0.16
Delhi				**ORF1a:P2018S**	3	0.31	**ORF1a:Y2489L**	3	0.5	**ORF1a:P2929L**	2	0.16
Delhi				**ORF1a:D144V**	3	0.31	**ORF1a:I2490L**	3	0.5	**ORF1a:A599V**	2	0.16
Delhi				**ORF1a:E347D**	3	0.31	**ORF1a:V2491***	3	0.5	**ORF1a:S1587L**	2	0.16
Delhi				**ORF1a:D795N**	3	0.31	**ORF1a:D2492***	3	0.5	**ORF1a:A2320V**	2	0.16
Delhi				**ORF1a:T3202M**	3	0.31	**ORF1a:F2505W**	3	0.5	**ORF1a:H110Y**	2	0.16
Delhi				**ORF1a:L1546I**	3	0.31	**ORF1a:D2506S**	3	0.5	**ORF1a:I71V**	2	0.16
Delhi				**ORF1a:G295D**	3	0.31	**ORF1a:A2508D**	3	0.5	**ORF1a:G519S**	2	0.16
Delhi				**ORF1a:K3979N**	3	0.31	**ORF1a:G2509L**	3	0.5	**ORF1a:M2796I**	2	0.16
Delhi				**ORF1a:R3802C**	3	0.31	**ORF1a:Q2510***	3	0.5	**ORF1a:D1184G**	2	0.16
Delhi				**ORF1a:A2637V**	3	0.31	**ORF1a:H2516F**	3	0.5	**ORF1a:A2994V**	2	0.16
Delhi				**ORF1a:R398C**	3	0.31	**ORF1a:V2522C**	3	0.5	**ORF1a:M2259L**	2	0.16
Delhi				**ORF1a:S2797F**	3	0.31	**ORF1a:N2523***	3	0.5	**ORF1a:T1309Y**	2	0.16
Delhi				**ORF1a:K458E**	3	0.31	**ORF1a:S2535R**	3	0.5	**ORF1a:E1311R**	2	0.16
Delhi				**ORF1a:A1670T**	3	0.31	**ORF1a:L2536Q**	3	0.5	**ORF1a:M1312Q**	2	0.16
Delhi				**ORF1a:G401S**	3	0.31	**ORF1a:N2539C**	3	0.5	**ORF1a:A1314Y**	2	0.16
Delhi				**ORF1a:I473T**	2	0.21	**ORF1a:V2540K**	3	0.5	**ORF1a:K1315N**	2	0.16
Delhi				**ORF1a:S1520P**	2	0.21	**ORF1a:L2570C**	3	0.5	**ORF1a:A1316H**	2	0.16
Delhi				**ORF1a:T1637I**	2	0.21	**ORF1a:D2573Q**	3	0.5	**ORF1a:Q1332S**	2	0.16
Delhi				**ORF1a:K1230N**	2	0.21	**ORF1a:Q2574S**	3	0.5	**ORF1a:L1346F**	2	0.16
Delhi				**ORF1a:D828Y**	2	0.21	**ORF1a:L2576Y**	3	0.5	**ORF1a:K1350H**	2	0.16
Delhi				**ORF1a:P3613S**	2	0.21	**ORF1a:D2579***	3	0.5	**ORF1a:A1352T**	2	0.16
Delhi				**ORF1a:T4083M**	2	0.21	**ORF1a:V2580***	3	0.5	**ORF1a:F1353I**	2	0.16
Delhi				**ORF1a:A90V**	2	0.21	**ORF1a:G2581C**	3	0.5	**ORF1a:I1355Y**	2	0.16
Delhi				**ORF1a:S538L**	2	0.21	**ORF1a:D2582G**	3	0.5	**ORF1a:P1357***	2	0.16
Delhi				**ORF1a:E1801D**	2	0.21	**ORF1a:A2584C**	3	0.5	**ORF1a:S1358***	2	0.16
Delhi				**ORF1a:P1803S**	2	0.21	**ORF1a:E2585S**	3	0.5	**ORF1a:I1359E**	2	0.16
Delhi				**ORF1a:V28I**	2	0.21	**ORF1a:V2586***	3	0.5	**ORF1a:I1360A**	2	0.16
Delhi				**ORF1a:S2289G**	2	0.21	**ORF1a:A2587N**	3	0.5	**ORF1a:S1361R**	2	0.16
Delhi				**ORF1a:N2065S**	2	0.21	**ORF1a:K2589***	3	0.5	**ORF1a:L1375C**	2	0.16
Delhi				**ORF1a:A2994V**	2	0.21	**ORF1a:M2590C**	3	0.5	**ORF1a:R1376F**	2	0.16
Delhi				**ORF1a:D1440H**	2	0.21	**ORF1a:F2591L**	3	0.5	**ORF1a:E1377L**	2	0.16
Delhi				**ORF1a:G2241C**	2	0.21	**ORF1a:D2592R**	3	0.5	**ORF1a:M1378E**	2	0.16
Delhi				**ORF1a:G3676C**	2	0.21	**ORF1a:A2593***	3	0.5	**ORF1a:L1379F**	2	0.16
Delhi				**ORF1a:Q4015H**	2	0.21	**ORF1a:N2623E**	3	0.5	**ORF1a:H1381R**	2	0.16
Delhi				**ORF1a:V86F**	2	0.21	**ORF1a:V2624C**	3	0.5	**ORF1a:A1382N**	2	0.16
Delhi				**ORF1a:R2159W**	2	0.21	**ORF1a:S2625V**	3	0.5	**ORF1a:E1383A**	2	0.16
Delhi				**ORF1a:P1692S**	2	0.21	**ORF1a:D2627R**	3	0.5	**ORF1a:E1384C**	2	0.16
Delhi				**ORF1a:L1144I**	2	0.21	**ORF1a:N2628Q**	3	0.5	**ORF1a:V1393N**	2	0.16
Delhi				**ORF1a:S2535L**	2	0.21	**ORF1a:V2629C**	3	0.5	**ORF1a:E1394Y**	2	0.16
Delhi				**ORF1a:T3459M**	2	0.21	**ORF1a:S2631F**	3	0.5	**ORF1a:K1396A**	2	0.16
Delhi				**ORF1a:T403I**	2	0.21	**ORF1a:T2632Y**	3	0.5	**ORF1a:A1397***	2	0.16
Delhi				**ORF1a:T4207I**	2	0.21	**ORF1a:I2634S**	3	0.5	**ORF1a:R1421***	2	0.16
Delhi				**ORF1a:V2540I**	2	0.21	**ORF1a:A2636S**	3	0.5	**ORF1a:F1422G**	2	0.16
Delhi				**ORF1a:A2529V**	2	0.21	**ORF1a:E2653R**	3	0.5	**ORF1a:F1424***	2	0.16
Delhi				**ORF1a:Q2563H**	2	0.21	**ORF1a:L2655C**	3	0.5	**ORF1a:Y1425N**	2	0.16

Delhi				**ORF1a:M1014I**	2	0.21	**ORF1a:K2656***	3	0.5	**ORF1a:K1428V**	2	0.16
Delhi				**ORF1a:L1182F**	2	0.21	**ORF1a:L2657M**	3	0.5	**ORF1a:V1431***	2	0.16
Delhi				**ORF1a:A4094V**	2	0.21	**ORF1a:Q2660R**	3	0.5	**ORF1a:A1432R**	2	0.16
Delhi				**ORF1a:G150C**	2	0.21	**ORF1a:N2678***	3	0.5	**ORF1a:L1434K**	2	0.16
Delhi				**ORF1a:T2615I**	2	0.21	**ORF1a:V2680H**	3	0.5	**ORF1a:I1435***	2	0.16
Delhi				**ORF1a:D762N**	2	0.21	**ORF1a:E2681D**	3	0.5	**ORF1a:T1437N**	2	0.16
Delhi				**ORF1a:E1340D**	2	0.21	**ORF1a:N2682T**	3	0.5	**ORF1a:L1438A**	2	0.16
Delhi				**ORF1a:E1413D**	2	0.21	**ORF1a:M2683P**	3	0.5	**ORF1a:N1439T**	2	0.16
Delhi				**ORF1a:E37D**	2	0.21	**ORF1a:T2684***	3	0.5	**ORF1a:D1440W**	2	0.16
Delhi				**ORF1a:H2092Y**	2	0.21	**ORF1a:R2686W**	3	0.5	**ORF1a:N1442C**	2	0.16
Delhi				**ORF1a:L580F**	2	0.21	**ORF1a:D2687C**	3	0.5	**ORF1a:E1443N**	2	0.16
Delhi				**ORF1a:E2089D**	2	0.21	**ORF1a:G2689C**	3	0.5	**ORF1a:S1468W**	2	0.16
Delhi				**ORF1a:A3454V**	2	0.21	**ORF1a:C2691S**	3	0.5	**ORF1a:V1471F**	2	0.16
Delhi				**ORF1a:S2151I**	2	0.21	**ORF1a:I2692Y**	3	0.5	**ORF1a:P1472G**	2	0.16
Delhi				**ORF1a:N3537D**	2	0.21	**ORF1a:D2693***	3	0.5	**ORF1a:A1473R**	2	0.16
Delhi				**ORF1a:A429T**	2	0.21	**ORF1a:Q2702G**	3	0.5	**ORF1a:T1474S**	2	0.16
Delhi				**ORF1a:A698S**	2	0.21	**ORF1a:V2703S**	3	0.5	**ORF1a:V1475C**	2	0.16
Delhi				**ORF1a:T2087I**	2	0.21	**ORF1a:A2704K**	3	0.5	**ORF1a:S1478Y**	2	0.16
Delhi				**ORF1a:I1259V**	2	0.21	**ORF1a:N2708V**	3	0.5	**ORF1a:S1479E**	2	0.16
Delhi				**ORF1a:L3930F**	2	0.21	**ORF1a:Q2724***	3	0.5	**ORF1a:P1480I**	2	0.16
Delhi				**ORF1a:R24C**	2	0.21	**ORF1a:R2748F**	3	0.5	**ORF1a:D1481S**	2	0.16
Delhi				**ORF1a:E995D**	2	0.21	**ORF1a:Q2749***	3	0.5	**ORF1a:A1482Q**	2	0.16
Delhi				**ORF1a:Y1039C**	2	0.21	**ORF1a:K2775C**	3	0.5	**ORF1a:V1483S**	2	0.16
Delhi				**ORF1a:K225R**	2	0.21	**ORF1a:L2781P**	3	0.5	**ORF1a:T1484A**	2	0.16
Delhi				**ORF1a:Q526H**	2	0.21	**ORF1a:V2783C**	3	0.5	**ORF1a:A1485S**	2	0.16
Delhi				**ORF1a:L204F**	2	0.21	**ORF1a:A2784C**	3	0.5	**ORF1a:G1488T**	2	0.16
Delhi				**ORF1a:K2511N**	2	0.21	**ORF1a:A2785C**	3	0.5	**ORF1a:Y1489***	2	0.16
Delhi				**ORF1a:I1215T**	2	0.21	**ORF1a:M315V**	3	0.5	**ORF1a:L1490R**	2	0.16
Delhi				**ORF1a:V1299L**	2	0.21	**ORF1a:H110Y**	3	0.5	**ORF1a:S1515F**	2	0.16
Delhi				**ORF1a:S391P**	2	0.21	**ORF1a:Q3777R**	3	0.5	**ORF1a:G1518***	2	0.16
Delhi				**ORF1a:A2097V**	2	0.21	**ORF1a:S2900L**	2	0.34	**ORF1a:Q1519E**	2	0.16
Delhi				**ORF1a:G4157S**	2	0.21	**ORF1a:T1246A**	2	0.34	**ORF1a:S1520R**	2	0.16
Delhi				**ORF1a:T1429I**	2	0.21	**ORF1a:V3748I**	2	0.34	**ORF1a:T1521***	2	0.16
Delhi				**ORF1a:L969F**	2	0.21	**ORF1a:T592I**	2	0.34	**ORF1a:Q1522***	2	0.16
Delhi				**ORF1a:R1566K**	2	0.21	**ORF1a:T1567A**	2	0.34	**ORF1a:L1523K**	2	0.16
Delhi				**ORF1a:K47R**	2	0.21	**ORF1a:V3653F**	2	0.34	**ORF1a:G1524C**	2	0.16
Delhi				**ORF1a:F1248S**	2	0.21	**ORF1a:S944L**	2	0.34	**ORF1a:D1575Y**	2	0.16
Delhi				**ORF1a:L4182F**	2	0.21	**ORF1a:K1053R**	2	0.34	**ORF1a:N1662E**	2	0.16
Delhi				**ORF1a:G2091D**	2	0.21	**ORF1a:P2018Q**	2	0.34	**ORF1a:G1663I**	2	0.16
Delhi				**ORF1a:T3090I**	2	0.21	**ORF1a:D1547Y**	2	0.34	**ORF1a:L1664P**	2	0.16
Delhi				**ORF1a:K680N**	2	0.21	**ORF1a:Y3722C**	2	0.34	**ORF1a:A1707C**	2	0.16
Delhi				**ORF1a:A1074V**	2	0.21	**ORF1a:T2495I**	2	0.34	**ORF1a:A1708S**	2	0.16
Delhi				**ORF1a:T4304A**	2	0.21	**ORF1a:C3762Y**	2	0.34	**ORF1a:N1709T**	2	0.16
Delhi				**ORF1a:G392C**	2	0.21	**ORF1a:G524V**	2	0.34	**ORF1a:F1710R**	2	0.16
Delhi				**ORF1a:K669N**	2	0.21	**ORF1a:T2648I**	2	0.34	**ORF1a:A1712L**	2	0.16
Delhi				**ORF1a:P1158L**	2	0.21	**ORF1a:G1119S**	2	0.34	**ORF1a:I1714L**	2	0.16
Delhi				**ORF1a:K2798E**	2	0.21	**ORF1a:P2046L**	2	0.34	**ORF1a:A1716***	2	0.16
Delhi				**ORF1a:V620I**	2	0.21	**ORF1a:K3832N**	2	0.34	**ORF1a:Y1717***	2	0.16
Delhi				**ORF1a:T2152I**	2	0.21	**ORF1a:P3371S**	2	0.34	**ORF1a:C1718D**	2	0.16
Delhi				**ORF1a:P1921R**	2	0.21	**ORF1a:G1125C**	2	0.34	**ORF1a:N1719S**	2	0.16
Delhi				**ORF1a:T4087I**	2	0.21	**ORF1a:T4265I**	2	0.34	**ORF1a:K1720R**	2	0.16
Delhi				**ORF1a:T568I**	2	0.21	**ORF1a:D3296Y**	2	0.34	**ORF1a:T1721***	2	0.16
Delhi				**ORF1a:G2091C**	2	0.21	**ORF1a:T4355I**	2	0.34	**ORF1a:G1723N**	2	0.16
Delhi				**ORF1a:T999I**	2	0.21	**ORF1a:L1853F**	2	0.34	**ORF1a:N1749V**	2	0.16
Delhi				**ORF1a:A516V**	2	0.21	**ORF1a:A540V**	2	0.34	**ORF1a:V1750S**	2	0.16
Delhi				**ORF1a:E948D**	2	0.21	**ORF1a:I3126M**	2	0.34	**ORF1a:V1751T**	2	0.16
Delhi				**ORF1a:T1246I**	2	0.21	**ORF1a:P1321S**	2	0.34	**ORF1a:K1753Q**	2	0.16
Delhi				**ORF1a:G3278S**	2	0.21	**ORF1a:L642F**	2	0.34	**ORF1a:T1754F**	2	0.16
Delhi				**ORF1a:S2103F**	2	0.21	**ORF1a:A3620V**	2	0.34	**ORF1a:C1755R**	2	0.16
Delhi				**ORF1a:Q1140H**	2	0.21	**ORF1a:P1921L**	2	0.34	**ORF1a:G1756F**	2	0.16
Delhi				**ORF1a:R207C**	2	0.21	**ORF1a:T4035I**	2	0.34	**ORF1a:Q1757L**	2	0.16
Delhi				**ORF1a:L2688F**	2	0.21	**ORF1a:T4249I**	2	0.34	**ORF1a:Q1759K**	2	0.16
Delhi				**ORF1a:V1783I**	2	0.21	**ORF1a:T1822I**	2	0.34	**ORF1a:T1760S**	2	0.16
Delhi				**ORF1a:P361T**	2	0.21	**ORF1a:T1444A**	2	0.34	**ORF1a:T1761L**	2	0.16
Delhi				**ORF1a:L393F**	2	0.21	**ORF1a:S302A**	2	0.34	**ORF1a:L1762E**	2	0.16
Delhi				**ORF1a:K714N**	2	0.21	**ORF1a:I1002S**	2	0.34	**ORF1a:K1763R**	2	0.16
Delhi				**ORF1a:R1566S**	2	0.21	**ORF1a:K2622R**	2	0.34	**ORF1a:E1766T**	2	0.16
Delhi				**ORF1a:S3195N**	2	0.21	**ORF1a:K3577E**	2	0.34	**ORF1a:V1768D**	2	0.16
Delhi				**ORF1a:T3940I**	2	0.21	**ORF1a:P4220L**	2	0.34	**ORF1a:M1769N**	2	0.16
Delhi				**ORF1a:P943S**	2	0.21	**ORF1a:L446F**	2	0.34	**ORF1a:Y1770P**	2	0.16
Delhi				**ORF1a:Q575R**	2	0.21	**ORF1a:P2739L**	2	0.34	**ORF1a:M1771***	2	0.16
Delhi				**ORF1a:D953Y**	2	0.21	**ORF1a:Y3063S**	2	0.34	**ORF1a:T1773C**	2	0.16
Delhi				**ORF1a:D1228Y**	2	0.21	**ORF1a:P3767L**	2	0.34	**ORF1a:L1774R**	2	0.16

Delhi				ORF1a:S2246L	2	0.21	ORF1a:G1726R	2	0.34	ORF1a:L1797D	2	0.16
Delhi				ORF1a:V2130I	2	0.21	ORF1a:D1727*	2	0.34	ORF1a:T1820S	2	0.16
Delhi				ORF1a:V2116I	2	0.21	ORF1a:E1730L	2	0.34	ORF1a:F1821T	2	0.16
Delhi				ORF1a:C3682W	2	0.21	ORF1a:M1732K	2	0.34	ORF1a:A1824W	2	0.16
Delhi				ORF1a:V774I	2	0.21	ORF1a:Y1734L	2	0.34	ORF1a:E1826L	2	0.16
Delhi				ORF1a:L2146F	2	0.21	ORF1a:L1735E	2	0.34	ORF1a:Y1827*	2	0.16
Delhi				ORF1a:G3002S	2	0.21	ORF1a:F1736R	2	0.34	ORF1a:G1829Y	2	0.16
Delhi				ORF1a:T1496I	2	0.21	ORF1a:Q1737G	2	0.34	ORF1a:Y1831F	2	0.16
Delhi				ORF1a:P2144S	2	0.21	ORF1a:H1738V	2	0.34	ORF1a:Q1832*	2	0.16
Delhi				ORF1a:L3919F	2	0.21	ORF1a:A1739*	2	0.34	ORF1a:C1833R	2	0.16
Delhi				ORF1a:H165Y	2	0.21	ORF1a:Q1759W	2	0.34	ORF1a:G1834N	2	0.16
Delhi				ORF1a:K261N	2	0.21	ORF1a:Y1899L	2	0.34	ORF1a:H1835F	2	0.16
Delhi				ORF1a:R1170H	2	0.21	ORF1a:K1901T	2	0.34	ORF1a:Y1836V	2	0.16
Delhi				ORF1a:S2224F	2	0.21	ORF1a:N1922T	2	0.34	ORF1a:K1837L	2	0.16
Delhi				ORF1a:T2967I	2	0.21	ORF1a:A1923I	2	0.34	ORF1a:I1863R	2	0.16
Delhi				ORF1a:S2890N	2	0.21	ORF1a:F1925C	2	0.34	ORF1a:T1864R	2	0.16
Delhi				ORF1a:T2283I	2	0.21	ORF1a:D1926*	2	0.34	ORF1a:D1865C	2	0.16
Delhi				ORF1a:A1420V	2	0.21	ORF1a:N1927*	2	0.34	ORF1a:V1866F	2	0.16
Delhi				ORF1a:T609I	2	0.21	ORF1a:F1930P	2	0.34	ORF1a:F1867T	2	0.16
Delhi				ORF1a:A3686V	2	0.21	ORF1a:R1953N	2	0.34	ORF1a:E1870K	2	0.16
Delhi				ORF1a:S1857L	2	0.21	ORF1a:K2191Y	2	0.34	ORF1a:N1871T	2	0.16
Delhi				ORF1a:K634N	2	0.21	ORF1a:S2242F	2	0.34	ORF1a:D1897F	2	0.16
Delhi				ORF1a:S944L	2	0.21	ORF1a:C2263F	2	0.34	ORF1a:N1898L	2	0.16
Delhi				ORF1a:T1626I	2	0.21	ORF1a:T2264E	2	0.34	ORF1a:Y1899F	2	0.16
Delhi				ORF1a:T1822I	2	0.21	ORF1a:G2265L	2	0.34	ORF1a:K1901R	2	0.16
Delhi				ORF1a:F3138S	2	0.21	ORF1a:R2267*	2	0.34	ORF1a:K1902A	2	0.16
Delhi				ORF1a:N160Y	2	0.21	ORF1a:V2751*	2	0.34	ORF1a:D1903T	2	0.16
Delhi				ORF1a:T436I	2	0.21	ORF1a:V2776*	2	0.34	ORF1a:S1905*	2	0.16
Delhi				ORF1a:A2033T	2	0.21	ORF1a:T2777*	2	0.34	ORF1a:Y1906S	2	0.16
Delhi				ORF1a:T2181I	2	0.21	ORF1a:F2780V	2	0.34	ORF1a:F1907C	2	0.16
Delhi				ORF1a:P1054L	2	0.21	ORF1a:L1681G	2	0.34	ORF1a:V1931K	2	0.16
Delhi				ORF1a:S212L	2	0.21	ORF1a:E1687Q	2	0.34	ORF1a:C1932P	2	0.16
Delhi				ORF1a:I300V	2	0.21	ORF1a:L1688S	2	0.34	ORF1a:D1933V	2	0.16
Delhi				ORF1a:Q998H	2	0.21	ORF1a:A1707G	2	0.34	ORF1a:E1954W	2	0.16
Delhi				ORF1a:T945N	2	0.21	ORF1a:A1708W	2	0.34	ORF1a:K1956*	2	0.16
Delhi				ORF1a:T3258I	2	0.21	ORF1a:N1709*	2	0.34	ORF1a:V1957E	2	0.16
Delhi				ORF1a:S2803L	2	0.21	ORF1a:F1710S	2	0.34	ORF1a:F1959C	2	0.16
Delhi				ORF1a:L3776F	2	0.21	ORF1a:A1712*	2	0.34	ORF1a:P1961Y	2	0.16
Delhi				ORF1a:V4369A	2	0.21	ORF1a:I1714L	2	0.34	ORF1a:D1962*	2	0.16
Delhi				ORF1a:I1785T	2	0.21	ORF1a:L1715C	2	0.34	ORF1a:K1984*	2	0.16
Delhi				ORF1a:G298S	2	0.21	ORF1a:A1716T	2	0.34	ORF1a:L1985T	2	0.16
Delhi				ORF1a:M3425I	2	0.21	ORF1a:A3454V	2	0.34	ORF1a:K1988S	2	0.16
Delhi				ORF1a:A964T	1	0.1	ORF1a:W630C	2	0.34	ORF1a:P1989*	2	0.16
Delhi				ORF1a:S1517F	1	0.1	ORF1a:R3802H	2	0.34	ORF1a:W1992S	2	0.16
Delhi				ORF1a:E976G	1	0.1	ORF1a:E2089D	2	0.34	ORF1a:S2015*	2	0.16
Delhi				ORF1a:G2342E	1	0.1	ORF1a:D935G	2	0.34	ORF1a:P2018Y	2	0.16
Delhi				ORF1a:I1286V	1	0.1	ORF1a:T1168I	2	0.34	ORF1a:V2019L	2	0.16
Delhi				ORF1a:T1874I	1	0.1	ORF1a:L4391I	2	0.34	ORF1a:E2020V	2	0.16
Delhi				ORF1a:V1295F	1	0.1	ORF1a:P1096L	2	0.34	ORF1a:T2021Y	2	0.16
Delhi				ORF1a:T2836I	1	0.1	ORF1a:L552I	2	0.34	ORF1a:S2022T	2	0.16
Delhi				ORF1a:T3461I	1	0.1	ORF1a:P959S	2	0.34	ORF1a:N2023L	2	0.16
Delhi				ORF1a:I3944V	1	0.1	ORF1a:T4031I	2	0.34	ORF1a:F2025L	2	0.16
Delhi				ORF1a:S858Y	1	0.1	ORF1a:P1640L	2	0.34	ORF1a:D2026E	2	0.16
Delhi				ORF1a:T4204A	1	0.1	ORF1a:H2092Y	2	0.34	ORF1a:V2027H	2	0.16
Delhi				ORF1a:N1042K	1	0.1	ORF1a:L293F	2	0.34	ORF1a:L2028K	2	0.16
Delhi				ORF1a:P2046S	1	0.1	ORF1a:L3338F	2	0.34	ORF1a:K2029T	2	0.16
Delhi				ORF1a:S911F	1	0.1	ORF1a:S318L	1	0.17	ORF1a:I2057R	2	0.16
Delhi				ORF1a:S279C	1	0.1	ORF1a:T2846I	1	0.17	ORF1a:Q2058S	2	0.16
Delhi				ORF1a:T1344A	1	0.1	ORF1a:M3539V	1	0.17	ORF1a:D2060T	2	0.16
Delhi				ORF1a:A1923V	1	0.1	ORF1a:S4398L	1	0.17	ORF1a:V2061S	2	0.16
Delhi				ORF1a:S2255F	1	0.1	ORF1a:S166G	1	0.17	ORF1a:N2140*	2	0.16
Delhi				ORF1a:Q946H	1	0.1	ORF1a:K2029N	1	0.17	ORF1a:M2169F	2	0.16
Delhi				ORF1a:A2832V	1	0.1	ORF1a:S887F	1	0.17	ORF1a:P2170L	2	0.16
Delhi				ORF1a:L320F	1	0.1	ORF1a:D1966A	1	0.17	ORF1a:K2208E	2	0.16
Delhi				ORF1a:S3949G	1	0.1	ORF1a:A1812V	1	0.17	ORF1a:F2209Y	2	0.16
Delhi				ORF1a:N2361K	1	0.1	ORF1a:N4042S	1	0.17	ORF1a:L2211*	2	0.16
Delhi				ORF1a:D2801G	1	0.1	ORF1a:R24C	1	0.17	ORF1a:A2213*	2	0.16
Delhi				ORF1a:A4130S	1	0.1	ORF1a:A3967V	1	0.17	ORF1a:S2214F	2	0.16
Delhi				ORF1a:G3478R	1	0.1	ORF1a:A1283V	1	0.17	ORF1a:N2216*	2	0.16
Delhi				ORF1a:N363T	1	0.1	ORF1a:A2040T	1	0.17	ORF1a:Y2217T	2	0.16
Delhi				ORF1a:G1119C	1	0.1	ORF1a:A2909T	1	0.17	ORF1a:L2218D	2	0.16
Delhi				ORF1a:R2875H	1	0.1	ORF1a:M3087I	1	0.17	ORF1a:S2220Y	2	0.16
Delhi				ORF1a:M3733V	1	0.1	ORF1a:P3395S	1	0.17	ORF1a:P2221Y	2	0.16

Delhi				**ORF1a:T2124I**	1	0.1	**ORF1a:T951I**	1	0.17	**ORF1a:F2223L**	2	0.16
Delhi				**ORF1a:K1247N**	1	0.1	**ORF1a:S1361P**	1	0.17	**ORF1a:S2224V**	2	0.16
Delhi				**ORF1a:V1765L**	1	0.1	**ORF1a:A474T**	1	0.17	**ORF1a:K2225F**	2	0.16
Delhi				**ORF1a:A2636T**	1	0.1	**ORF1a:E2268A**	1	0.17	**ORF1a:L2226T**	2	0.16
Delhi				**ORF1a:D1228G**	1	0.1	**ORF1a:C1718Y**	1	0.17	**ORF1a:N2228K**	2	0.16
Delhi				**ORF1a:T2495I**	1	0.1	**ORF1a:P2221S**	1	0.17	**ORF1a:I2229C**	2	0.16
Delhi				**ORF1a:T2419I**	1	0.1	**ORF1a:R4132K**	1	0.17	**ORF1a:I2230L**	2	0.16
Delhi				**ORF1a:V1008F**	1	0.1	**ORF1a:A1043V**	1	0.17	**ORF1a:I2231P**	2	0.16
Delhi				**ORF1a:V543F**	1	0.1	**ORF1a:T2087I**	1	0.17	**ORF1a:W2232R**	2	0.16
Delhi				**ORF1a:T4174I**	1	0.1	**ORF1a:T2021I**	1	0.17	**ORF1a:L2341F**	2	0.16
Delhi				**ORF1a:I4291V**	1	0.1	**ORF1a:T1754I**	1	0.17	**ORF1a:L2343H**	2	0.16
Delhi				**ORF1a:M2606I**	1	0.1	**ORF1a:T1682I**	1	0.17	**ORF1a:A2345I**	2	0.16
Delhi				**ORF1a:Y3765H**	1	0.1	**ORF1a:T727I**	1	0.17	**ORF1a:I2346V**	2	0.16
Delhi				**ORF1a:T340I**	1	0.1	**ORF1a:A903V**	1	0.17	**ORF1a:M2347F**	2	0.16
Delhi				**ORF1a:G2241V**	1	0.1	**ORF1a:S1027I**	1	0.17	**ORF1a:F2351C**	2	0.16
Delhi				**ORF1a:L4391F**	1	0.1	**ORF1a:A1342S**	1	0.17	**ORF1a:Y2353T**	2	0.16
Delhi				**ORF1a:K3351N**	1	0.1	**ORF1a:V2496L**	1	0.17	**ORF1a:A2355Y**	2	0.16
Delhi				**ORF1a:A3061V**	1	0.1	**ORF1a:E386D**	1	0.17	**ORF1a:V2356***	2	0.16
Delhi				**ORF1a:M315V**	1	0.1	**ORF1a:D2032N**	1	0.17	**ORF1a:H2357***	2	0.16
Delhi				**ORF1a:M3862I**	1	0.1	**ORF1a:A1234V**	1	0.17	**ORF1a:V2401L**	2	0.16
Delhi				**ORF1a:I431M**	1	0.1	**ORF1a:L2778F**	1	0.17	**ORF1a:D2402Q**	2	0.16
Delhi				**ORF1a:G728C**	1	0.1	**ORF1a:E2681G**	1	0.17	**ORF1a:G2403T**	2	0.16
Delhi				**ORF1a:A4005T**	1	0.1	**ORF1a:A3548V**	1	0.17	**ORF1a:C2404***	2	0.16
Delhi				**ORF1a:E3906A**	1	0.1	**ORF1a:T3032I**	1	0.17	**ORF1a:N2405***	2	0.16
Delhi				**ORF1a:P3613L**	1	0.1	**ORF1a:A566S**	1	0.17	**ORF1a:S2407N**	2	0.16
Delhi				**ORF1a:I2569T**	1	0.1	**ORF1a:P2018S**	1	0.17	**ORF1a:T2408K**	2	0.16
Delhi				**ORF1a:E910D**	1	0.1	**ORF1a:T3284I**	1	0.17	**ORF1a:C2409S**	2	0.16
Delhi				**ORF1a:R1170C**	1	0.1	**ORF1a:K1202N**	1	0.17	**ORF1a:M2410R**	2	0.16
Delhi				**ORF1a:K2507N**	1	0.1	**ORF1a:I1259N**	1	0.17	**ORF1a:V2436N**	2	0.16
Delhi				**ORF1a:M2096I**	1	0.1	**ORF1a:T1637I**	1	0.17	**ORF1a:A2438Q**	2	0.16
Delhi				**ORF1a:K3839R**	1	0.1	**ORF1a:A427V**	1	0.17	**ORF1a:N2439T**	2	0.16
Delhi				**ORF1a:V2157F**	1	0.1	**ORF1a:V3912I**	1	0.17	**ORF1a:G2440T**	2	0.16
Delhi				**ORF1a:E1209D**	1	0.1	**ORF1a:L3829F**	1	0.17	**ORF1a:G2441Q**	2	0.16
Delhi				**ORF1a:K1132N**	1	0.1	**ORF1a:R232C**	1	0.17	**ORF1a:K2442L**	2	0.16
Delhi				**ORF1a:S2261F**	1	0.1	**ORF1a:P1207H**	1	0.17	**ORF1a:G2443E**	2	0.16
Delhi				**ORF1a:A2249V**	1	0.1	**ORF1a:A2139V**	1	0.17	**ORF1a:F2444L**	2	0.16
Delhi				**ORF1a:S1133I**	1	0.1	**ORF1a:M2606I**	1	0.17	**ORF1a:K2446***	2	0.16
Delhi				**ORF1a:T2153I**	1	0.1	**ORF1a:L3352F**	1	0.17	**ORF1a:A2470***	2	0.16
Delhi				**ORF1a:N4078S**	1	0.1	**ORF1a:G728C**	1	0.17	**ORF1a:R2471Y**	2	0.16
Delhi				**ORF1a:A3705V**	1	0.1	**ORF1a:T3904I**	1	0.17	**ORF1a:D2472I**	2	0.16
Delhi				**ORF1a:K458R**	1	0.1	**ORF1a:N2065S**	1	0.17	**ORF1a:S2474***	2	0.16
Delhi				**ORF1a:P309Q**	1	0.1	**ORF1a:R2748K**	1	0.17	**ORF1a:L2475P**	2	0.16
Delhi				**ORF1a:G3286D**	1	0.1	**ORF1a:H1545Y**	1	0.17	**ORF1a:Q2476V**	2	0.16
Delhi				**ORF1a:T4164I**	1	0.1	**ORF1a:I1551V**	1	0.17	**ORF1a:Y2504S**	2	0.16
Delhi				**ORF1a:V111M**	1	0.1	**ORF1a:G3617S**	1	0.17	**ORF1a:F2505W**	2	0.16
Delhi				**ORF1a:K1396N**	1	0.1	**ORF1a:L4040F**	1	0.17	**ORF1a:D2506S**	2	0.16
Delhi				**ORF1a:H2125N**	1	0.1	**ORF1a:A3497V**	1	0.17	**ORF1a:A2508D**	2	0.16
Delhi				**ORF1a:A2128S**	1	0.1	**ORF1a:T2283I**	1	0.17	**ORF1a:G2509L**	2	0.16
Delhi				**ORF1a:A1234V**	1	0.1	**ORF1a:S2822A**	1	0.17	**ORF1a:Q2510***	2	0.16
Delhi				**ORF1a:A2344V**	1	0.1	**ORF1a:R550C**	1	0.17	**ORF1a:H2516F**	2	0.16
Delhi				**ORF1a:G3704S**	1	0.1	**ORF1a:S1221L**	1	0.17	**ORF1a:K2548V**	2	0.16
Delhi				**ORF1a:P4220L**	1	0.1	**ORF1a:M2606L**	1	0.17	**ORF1a:D2573Q**	2	0.16
Delhi				**ORF1a:D629G**	1	0.1	**ORF1a:G519S**	1	0.17	**ORF1a:Q2574S**	2	0.16
Delhi				**ORF1a:A354V**	1	0.1	**ORF1a:V1008F**	1	0.17	**ORF1a:L2576Y**	2	0.16
Delhi				**ORF1a:L590F**	1	0.1	**ORF1a:R2159W**	1	0.17	**ORF1a:D2579***	2	0.16
Delhi				**ORF1a:F941L**	1	0.1	**ORF1a:A1397T**	1	0.17	**ORF1a:V2580C**	2	0.16
Delhi				**ORF1a:A1397V**	1	0.1	**ORF1a:K1247R**	1	0.17	**ORF1a:S2600C**	2	0.16
Delhi				**ORF1a:S3099L**	1	0.1	**ORF1a:L2062F**	1	0.17	**ORF1a:T2601S**	2	0.16
Delhi				**ORF1a:E347G**	1	0.1	**ORF1a:S2224F**	1	0.17	**ORF1a:F2602***	2	0.16
Delhi				**ORF1a:T1794I**	1	0.1	**ORF1a:I523V**	1	0.17	**ORF1a:V2629C**	2	0.16
Delhi				**ORF1a:V2588I**	1	0.1	**ORF1a:A1314V**	1	0.17	**ORF1a:L2630R**	2	0.16
Delhi				**ORF1a:L4037F**	1	0.1	**ORF1a:W2232L**	1	0.17	**ORF1a:T2632Y**	2	0.16
Delhi				**ORF1a:P371L**	1	0.1	**ORF1a:P4120L**	1	0.17	**ORF1a:I2634S**	2	0.16
Delhi				**ORF1a:K2200N**	1	0.1	**ORF1a:A4136V**	1	0.17	**ORF1a:A2636S**	2	0.16
Delhi				**ORF1a:A208V**	1	0.1	**ORF1a:T1543I**	1	0.17	**ORF1a:E2653R**	2	0.16
Delhi				**ORF1a:P1693L**	1	0.1	**ORF1a:E2070V**	1	0.17	**ORF1a:L2655C**	2	0.16
Delhi				**ORF1a:S1520F**	1	0.1	**ORF1a:S2193F**	1	0.17	**ORF1a:K2656***	2	0.16
Delhi				**ORF1a:S2844I**	1	0.1	**ORF1a:I2156V**	1	0.17	**ORF1a:L2657M**	2	0.16
Delhi				**ORF1a:V337F**	1	0.1	**ORF1a:D2582E**	1	0.17	**ORF1a:Q2660R**	2	0.16
Delhi				**ORF1a:R184S**	1	0.1	**ORF1a:T403I**	1	0.17	**ORF1a:N2678***	2	0.16
Delhi				**ORF1a:I3145T**	1	0.1	**ORF1a:M2674I**	1	0.17	**ORF1a:V2680H**	2	0.16
Delhi				**ORF1a:T4129I**	1	0.1	**ORF1a:T3868I**	1	0.17	**ORF1a:E2681D**	2	0.16
Delhi				**ORF1a:A307V**	1	0.1	**ORF1a:V627I**	1	0.17	**ORF1a:N2682T**	2	0.16

Delhi				**ORF1a:Q22R**	1	0.1	**ORF1a:A1631V**	1	0.17	**ORF1a:M2683P**	2	0.16
Delhi				**ORF1a:A3061T**	1	0.1	**ORF1a:R184C**	1	0.17	**ORF1a:T2684***	2	0.16
Delhi				**ORF1a:T1395A**	1	0.1	**ORF1a:A1306T**	1	0.17	**ORF1a:R2686W**	2	0.16
Delhi				**ORF1a:F263L**	1	0.1	**ORF1a:V3056I**	1	0.17	**ORF1a:D2687C**	2	0.16
Delhi				**ORF1a:V2491I**	1	0.1	**ORF1a:E3947D**	1	0.17	**ORF1a:G2689C**	2	0.16
Delhi				**ORF1a:P1497S**	1	0.1	**ORF1a:S1189G**	1	0.17	**ORF1a:C2691S**	2	0.16
Delhi				**ORF1a:I2663V**	1	0.1	**ORF1a:S1515F**	1	0.17	**ORF1a:I2692Y**	2	0.16
Delhi				**ORF1a:D2848N**	1	0.1	**ORF1a:S2030L**	1	0.17	**ORF1a:D2693***	2	0.16
Delhi				**ORF1a:D4187N**	1	0.1	**ORF1a:A3023V**	1	0.17	**ORF1a:Q2702G**	2	0.16
Delhi				**ORF1a:Q1582R**	1	0.1	**ORF1a:K1668A**	1	0.17	**ORF1a:V2703S**	2	0.16
Delhi				**ORF1a:N313K**	1	0.1	**ORF1a:W1669Q**	1	0.17	**ORF1a:A2704K**	2	0.16
Delhi				**ORF1a:A803S**	1	0.1	**ORF1a:S1666C**	1	0.17	**ORF1a:N2708V**	2	0.16
Delhi				**ORF1a:L469F**	1	0.1	**ORF1a:F1690V**	1	0.17	**ORF1a:N2736V**	2	0.16
Delhi				**ORF1a:E1013D**	1	0.1	**ORF1a:P1692T**	1	0.17	**ORF1a:N2737***	2	0.16
Delhi				**ORF1a:T951I**	1	0.1	**ORF1a:A1694***	1	0.17	**ORF1a:L2738T**	2	0.16
Delhi				**ORF1a:N1324D**	1	0.1	**ORF1a:Q1696L**	1	0.17	**ORF1a:P2739T**	2	0.16
Delhi				**ORF1a:E342A**	1	0.1	**ORF1a:D1697C**	1	0.17	**ORF1a:F2740T**	2	0.16
Delhi				**ORF1a:F3605V**	1	0.1	**ORF1a:A1698T**	1	0.17	**ORF1a:W2769***	2	0.16
Delhi				**ORF1a:R1464W**	1	0.1	**ORF1a:T1731Q**	1	0.17	**ORF1a:A2784E**	2	0.16
Delhi				**ORF1a:V543I**	1	0.1	**ORF1a:D1962R**	1	0.17	**ORF1a:M4116R**	1	0.08
Delhi				**ORF1a:D992Y**	1	0.1	**ORF1a:C2210E**	1	0.17	**ORF1a:D4165G**	1	0.08
Delhi				**ORF1a:A498V**	1	0.1	**ORF1a:L2211V**	1	0.17	**ORF1a:A3615V**	1	0.08
Delhi				**ORF1a:A1074T**	1	0.1	**ORF1a:E2212T**	1	0.17	**ORF1a:V3298L**	1	0.08
Delhi				**ORF1a:L4040F**	1	0.1	**ORF1a:A2213***	1	0.17	**ORF1a:I3944V**	1	0.08
Delhi				**ORF1a:S1150A**	1	0.1	**ORF1a:S2214F**	1	0.17	**ORF1a:P4075S**	1	0.08
Delhi				**ORF1a:T2121I**	1	0.1	**ORF1a:N2216***	1	0.17	**ORF1a:L2084I**	1	0.08
Delhi				**ORF1a:E870A**	1	0.1	**ORF1a:Y2217T**	1	0.17	**ORF1a:T2952I**	1	0.08
Delhi				**ORF1a:E1007K**	1	0.1	**ORF1a:L2218D**	1	0.17	**ORF1a:G661S**	1	0.08
Delhi				**ORF1a:E1909D**	1	0.1	**ORF1a:S2220Y**	1	0.17	**ORF1a:C3682Y**	1	0.08
Delhi				**ORF1a:E3073V**	1	0.1	**ORF1a:P2221Y**	1	0.17	**ORF1a:V2061F**	1	0.08
Delhi				**ORF1a:A2123V**	1	0.1	**ORF1a:G2241V**	1	0.17	**ORF1a:V1143F**	1	0.08
Delhi				**ORF1a:L2062F**	1	0.1	**ORF1a:L2243T**	1	0.17	**ORF1a:P2260S**	1	0.08
Delhi				**ORF1a:T3287A**	1	0.1	**ORF1a:G2251Y**	1	0.17	**ORF1a:L890F**	1	0.08
Delhi				**ORF1a:G4178S**	1	0.1	**ORF1a:C1718L**	1	0.17	**ORF1a:K714N**	1	0.08
Delhi				**ORF1a:E2112K**	1	0.1	**ORF1a:N1719S**	1	0.17	**ORF1a:L27F**	1	0.08
Delhi				**ORF1a:P4220S**	1	0.1	**ORF1a:K1720L**	1	0.17	**ORF1a:A2891V**	1	0.08
Delhi				**ORF1a:D1554Y**	1	0.1	**ORF1a:T1721L**	1	0.17	**ORF1a:A3235V**	1	0.08
Delhi				**ORF1a:G1069E**	1	0.1	**ORF1a:V1722***	1	0.17	**ORF1a:K634Q**	1	0.08
Delhi				**ORF1a:A1298V**	1	0.1	**ORF1a:G1723***	1	0.17	**ORF1a:E1136G**	1	0.08
Delhi				**ORF1a:A2139V**	1	0.1	**ORF1a:E1724D**	1	0.17	**ORF1a:L1115P**	1	0.08
Delhi				**ORF1a:V2116L**	1	0.1	**ORF1a:L1725S**	1	0.17	**ORF1a:V2052L**	1	0.08
Delhi				**ORF1a:V3170A**	1	0.1	**ORF1a:T1890***	1	0.17	**ORF1a:G2763S**	1	0.08
Delhi				**ORF1a:I2229V**	1	0.1	**ORF1a:D1933V**	1	0.17	**ORF1a:L1144P**	1	0.08
Delhi				**ORF1a:H3580Y**	1	0.1	**ORF1a:K2191C**	1	0.17	**ORF1a:C1755S**	1	0.08
Delhi				**ORF1a:A702T**	1	0.1	**ORF1a:F2223T**	1	0.17	**ORF1a:K1077R**	1	0.08
Delhi				**ORF1a:P782L**	1	0.1	**ORF1a:S2224I**	1	0.17	**ORF1a:N2111S**	1	0.08
Delhi				**ORF1a:V1583F**	1	0.1	**ORF1a:L2226C**	1	0.17	**ORF1a:A1631V**	1	0.08
Delhi				**ORF1a:M1769V**	1	0.1	**ORF1a:Y2245F**	1	0.17	**ORF1a:I1892V**	1	0.08
Delhi				**ORF1a:V3166F**	1	0.1	**ORF1a:S2246R**	1	0.17	**ORF1a:V639I**	1	0.08
Delhi				**ORF1a:T283I**	1	0.1	**ORF1a:T2247H**	1	0.17	**ORF1a:E347V**	1	0.08
Delhi				**ORF1a:V337I**	1	0.1	**ORF1a:A2249F**	1	0.17	**ORF1a:T4065I**	1	0.08
Delhi				**ORF1a:L624F**	1	0.1	**ORF1a:S2255L**	1	0.17	**ORF1a:H81N**	1	0.08
Delhi				**ORF1a:M3280I**	1	0.1	**ORF1a:N2256Y**	1	0.17	**ORF1a:Q1140H**	1	0.08
Delhi				**ORF1a:G227S**	1	0.1	**ORF1a:V2751N**	1	0.17	**ORF1a:S279T**	1	0.08
Delhi				**ORF1a:G379E**	1	0.1	**ORF1a:V2753K**	1	0.17	**ORF1a:K1981Q**	1	0.08
Delhi				**ORF1a:D589G**	1	0.1	**ORF1a:V2754D**	1	0.17	**ORF1a:S3884L**	1	0.08
Delhi				**ORF1a:E670K**	1	0.1	**ORF1a:T2755S**	1	0.17	**ORF1a:A1352V**	1	0.08
Delhi				**ORF1a:Y3062F**	1	0.1	**ORF1a:K2757***	1	0.17	**ORF1a:L1762F**	1	0.08
Delhi				**ORF1a:T1567I**	1	0.1	**ORF1a:I2758G**	1	0.17	**ORF1a:R3163G**	1	0.08
Delhi				**ORF1a:G7S**	1	0.1	**ORF1a:A2759W**	1	0.17	**ORF1a:T3361A**	1	0.08
Delhi				**ORF1a:S610L**	1	0.1	**ORF1a:L2760***	1	0.17	**ORF1a:Y3649C**	1	0.08
Delhi				**ORF1a:T2106I**	1	0.1	**ORF1a:K2761N**	1	0.17	**ORF1a:S2312P**	1	0.08
Delhi				**ORF1a:K1197T**	1	0.1	**ORF1a:G2762C**	1	0.17	**ORF1a:E87A**	1	0.08
Delhi				**ORF1a:T4265I**	1	0.1	**ORF1a:G2763***	1	0.17	**ORF1a:T1242I**	1	0.08
Delhi				**ORF1a:E952A**	1	0.1	**ORF1a:K2764***	1	0.17	**ORF1a:V1477I**	1	0.08
Delhi				**ORF1a:A2345V**	1	0.1	**ORF1a:I2765L**	1	0.17	**ORF1a:M3669T**	1	0.08
Delhi				**ORF1a:T1242I**	1	0.1	**ORF1a:N2767E**	1	0.17	**ORF1a:T4207I**	1	0.08
Delhi				**ORF1a:V2052L**	1	0.1	**ORF1a:I2774T**	1	0.17	**ORF1a:E755K**	1	0.08
Delhi				**ORF1a:Y3250H**	1	0.1	**ORF1a:S1666G**	1	0.17	**ORF1a:E1205D**	1	0.08
Delhi				**ORF1a:P3252E**	1	0.1	**ORF1a:I1667T**	1	0.17	**ORF1a:R426C**	1	0.08
Delhi				**ORF1a:P3253S**	1	0.1	**ORF1a:W1669G**	1	0.17	**ORF1a:H3580Y**	1	0.08
Delhi				**ORF1a:V627F**	1	0.1	**ORF1a:A1670T**	1	0.17	**ORF1a:E233K**	1	0.08
Delhi				**ORF1a:E743D**	1	0.1	**ORF1a:D1671G**	1	0.17	**ORF1a:M2606I**	1	0.08

Delhi				ORF1a:T554I	1	0.1	ORF1a:S1587L	1	0.17	ORF1a:A486V	1	0.08
Delhi				ORF1a:T614I	1	0.1	ORF1a:E1196V	1	0.17	ORF1a:P2685T	1	0.08
Delhi				ORF1a:M2259V	1	0.1	ORF1a:V5I	1	0.17	ORF1a:P3504S	1	0.08
Delhi				ORF1a:T2877I	1	0.1	ORF1a:H45Y	1	0.17	ORF1a:F190Y	1	0.08
Delhi				ORF1a:H1987Y	1	0.1	ORF1a:Y906H	1	0.17	ORF1a:P3359L	1	0.08
Delhi				ORF1a:T2280I	1	0.1	ORF1a:V3367A	1	0.17	ORF1a:Q3729K	1	0.08
Delhi				ORF1a:S3530L	1	0.1	ORF1a:P3613L	1	0.17	ORF1a:A3958V	1	0.08
Delhi				ORF1a:E472K	1	0.1	ORF1a:L1507F	1	0.17	ORF1a:M731I	1	0.08
Delhi				ORF1a:V3057L	1	0.1	ORF1a:L3644F	1	0.17	ORF1a:P1596S	1	0.08
Delhi				ORF1a:R124C	1	0.1	ORF1a:M2259I	1	0.17	ORF1a:R2011C	1	0.08
Delhi				ORF1a:K684R	1	0.1	ORF1a:M3712I	1	0.17	ORF1a:D795Y	1	0.08
Delhi				ORF1a:L2226P	1	0.1	ORF1a:S1361F	1	0.17	ORF1a:F3614V	1	0.08
Delhi				ORF1a:C3766F	1	0.1	ORF1a:K680N	1	0.17	ORF1a:P2685S	1	0.08
Delhi				ORF1a:V1584M	1	0.1	ORF1a:I3731V	1	0.17	ORF1a:P371S	1	0.08
Delhi				ORF1a:Y2168H	1	0.1	ORF1a:T866I	1	0.17	ORF1a:V2795A	1	0.08
Delhi				ORF1a:F1214I	1	0.1	ORF1a:T2274I	1	0.17	ORF1a:M3934I	1	0.08
Delhi				ORF1a:V1025L	1	0.1	ORF1a:L3116F	1	0.17	ORF1a:E726K	1	0.08
Delhi				ORF1a:L2176F	1	0.1	ORF1a:M902V	1	0.17	ORF1a:L1249F	1	0.08
Delhi				ORF1a:M789I	1	0.1	ORF1a:G7S	1	0.17	ORF1a:R550H	1	0.08
Delhi				ORF1a:T1543I	1	0.1	ORF1a:D2579G	1	0.17	ORF1a:I4074V	1	0.08
Delhi				ORF1a:L3313F	1	0.1	ORF1a:M3934I	1	0.17	ORF1a:R1628S	1	0.08
Delhi				ORF1a:S1587L	1	0.1	ORF1a:A1078T	1	0.17	ORF1a:G3617S	1	0.08
Delhi				ORF1a:M1732I	1	0.1	ORF1a:A2325T	1	0.17	ORF1a:T2632N	1	0.08
Delhi				ORF1a:S1952L	1	0.1	ORF1a:V2943F	1	0.17	ORF1a:S1027G	1	0.08
Delhi				ORF1a:Y185H	1	0.1	ORF1a:L3754F	1	0.17	ORF1a:T1504A	1	0.08
Delhi				ORF1a:D139G	1	0.1	ORF1a:Y2266C	1	0.17	ORF1a:G1068R	1	0.08
Delhi				ORF1a:Y1846C	1	0.1	ORF1a:S383I	1	0.17	ORF1a:N267K	1	0.08
Delhi				ORF1a:A2128V	1	0.1	ORF1a:K2771N	1	0.17	ORF1a:P777L	1	0.08
Delhi				ORF1a:S279F	1	0.1	ORF1a:I3142V	1	0.17	ORF1a:K1655R	1	0.08
Delhi				ORF1a:A3648V	1	0.1	ORF1a:T3665I	1	0.17	ORF1a:R2748K	1	0.08
Delhi				ORF1a:N4367S	1	0.1	ORF1a:M3661L	1	0.17	ORF1a:E93G	1	0.08
Delhi				ORF1a:L2778F	1	0.1	ORF1a:K4226R	1	0.17	ORF1a:T592I	1	0.08
Delhi				ORF1a:H1067Y	1	0.1	ORF1a:P380L	1	0.17	ORF1a:D981Y	1	0.08
Delhi				ORF1a:T3287I	1	0.1	ORF1a:L450F	1	0.17	ORF1a:G137S	1	0.08
Delhi				ORF1a:L3495F	1	0.1	ORF1a:A2994V	1	0.17	ORF1a:S2242F	1	0.08
Delhi				ORF1a:V1887I	1	0.1	ORF1a:A3657D	1	0.17	ORF1a:A3856S	1	0.08
Delhi				ORF1a:R3574I	1	0.1	ORF1a:A1352V	1	0.17	ORF1a:T4249I	1	0.08
Delhi				ORF1a:V1085I	1	0.1	ORF1a:T3459M	1	0.17	ORF1a:C1235Y	1	0.08
Delhi				ORF1a:H1160Y	1	0.1	ORF1a:I4205V	1	0.17	ORF1a:K2029N	1	0.08
Delhi				ORF1a:G192S	1	0.1	ORF1a:A1151V	1	0.17	ORF1a:Y387C	1	0.08
Delhi				ORF1a:G1595C	1	0.1	ORF1a:E942D	1	0.17	ORF1a:I785V	1	0.08
Delhi				ORF1a:K2497N	1	0.1	ORF1a:N2081T	1	0.17	ORF1a:V1991F	1	0.08
Delhi				ORF1a:T2746I	1	0.1	ORF1a:M2365T	1	0.17	ORF1a:I1232T	1	0.08
Delhi				ORF1a:K2059E	1	0.1	ORF1a:I547V	1	0.17	ORF1a:V3865I	1	0.08
Delhi				ORF1a:L61M	1	0.1	ORF1a:K1949R	1	0.17	ORF1a:I1045V	1	0.08
Delhi				ORF1a:I3731T	1	0.1	ORF1a:F3957L	1	0.17	ORF1a:T403I	1	0.08
Delhi				ORF1a:I280T	1	0.1	ORF1a:K1956R	1	0.17	ORF1a:P2110L	1	0.08
Delhi				ORF1a:P361S	1	0.1	ORF1a:K2656R	1	0.17	ORF1a:I659V	1	0.08
Delhi				ORF1a:V1211F	1	0.1	ORF1a:I1301V	1	0.17	ORF1a:A405V	1	0.08
Delhi				ORF1a:T1597I	1	0.1	ORF1a:L320I	1	0.17	ORF1a:A2621S	1	0.08
Delhi				ORF1a:V424A	1	0.1	ORF1a:L893M	1	0.17	ORF1a:A3620S	1	0.08
Delhi				ORF1a:D1273E	1	0.1	ORF1a:E2K	1	0.17	ORF1a:P959S	1	0.08
Delhi				ORF1a:S1221P	1	0.1	ORF1a:R3993H	1	0.17	ORF1a:G442D	1	0.08
Delhi				ORF1a:K2771N	1	0.1	ORF1a:S528L	1	0.17	ORF1a:T1543I	1	0.08
Delhi				ORF1a:V3107I	1	0.1	ORF1a:Q3878R	1	0.17	ORF1a:T2153I	1	0.08
Delhi				ORF1a:L122F	1	0.1	ORF1a:P4223L	1	0.17	ORF1a:T781I	1	0.08
Delhi				ORF1a:L450V	1	0.1	ORF1a:A1306S	1	0.17	ORF1a:P1692S	1	0.08
Delhi				ORF1a:V4372I	1	0.1	ORF1a:P2287S	1	0.17	ORF1a:T3032I	1	0.08
Delhi				ORF1a:F709L	1	0.1	ORF1a:V2930L	1	0.17	ORF1a:T1000I	1	0.08
Delhi				ORF1a:T1840I	1	0.1	ORF1a:G298S	1	0.17	ORF1a:Q3729R	1	0.08
Delhi				ORF1a:S2193F	1	0.1	ORF1a:T3308I	1	0.17	ORF1a:A2732G	1	0.08
Delhi				ORF1a:A3143T	1	0.1	ORF1a:L3667F	1	0.17	ORF1a:V28I	1	0.08
Delhi				ORF1a:E1205A	1	0.1	ORF1a:K1428R	1	0.17	ORF1a:P1213S	1	0.08
Delhi				ORF1a:D1547Y	1	0.1	ORF1a:M2365I	1	0.17	ORF1a:G4374S	1	0.08
Delhi				ORF1a:V3130A	1	0.1	ORF1a:P3359S	1	0.17	ORF1a:P1921L	1	0.08
Delhi				ORF1a:A3220V	1	0.1	ORF1a:T2124I	1	0.17	ORF1a:S2015R	1	0.08
Delhi				ORF1a:T3487I	1	0.1	ORF1a:S1188P	1	0.17	ORF1a:T2016K	1	0.08
Delhi				ORF1a:T265I	1	0.1	ORF1a:T4087I	1	0.17	ORF1a:V3810D	1	0.08
Delhi				ORF1a:M3129I	1	0.1	ORF1a:L2503F	1	0.17	ORF1a:T2484I	1	0.08
Delhi				ORF1a:W612C	1	0.1	ORF1a:M3221I	1	0.17	ORF1a:C667R	1	0.08
Delhi				ORF1a:P3447L	1	0.1	ORF1a:I2634V	1	0.17	ORF1a:D1585N	1	0.08
Delhi				ORF1a:E797A	1	0.1	ORF1a:G3546D	1	0.17	ORF1a:P3767L	1	0.08
Delhi				ORF1a:P271L	1	0.1	ORF1a:E102K	1	0.17	ORF1a:T554I	1	0.08

Delhi				ORF1a:M1647I	1	0.1	ORF1a:P361L	1	0.17	ORF1a:V3559I	1	0.08
Delhi				ORF1a:A2587V	1	0.1	ORF1a:T1246I	1	0.17	ORF1a:M1586I	1	0.08
Delhi				ORF1a:A4357V	1	0.1	ORF1a:D1639N	1	0.17	ORF1a:R24C	1	0.08
Delhi				ORF1a:Y600S	1	0.1	ORF1a:V2892F	1	0.17	ORF1a:A211V	1	0.08
Delhi				ORF1a:T4088K	1	0.1	ORF1a:D3222N	1	0.17	ORF1a:A3625V	1	0.08
Delhi				ORF1a:L552P	1	0.1	ORF1a:G3278S	1	0.17	ORF1a:M3087V	1	0.08
Delhi				ORF1a:T4088I	1	0.1	ORF1a:L3691S	1	0.17	ORF1a:T1682I	1	0.08
Delhi				ORF1a:S4398A	1	0.1	ORF1a:D582G	1	0.17	ORF1a:V3759F	1	0.08
Delhi				ORF1a:S2396G	1	0.1	ORF1a:V3968F	1	0.17	ORF1a:A4016V	1	0.08
Delhi				ORF1a:A3987V	1	0.1	ORF1a:A2891V	1	0.17	ORF1a:S302F	1	0.08
Delhi				ORF1a:F3085C	1	0.1	ORF1a:G3676C	1	0.17	ORF1a:I2332V	1	0.08
Delhi				ORF1a:H2520Y	1	0.1	ORF1a:D3836N	1	0.17	ORF1a:Y1183H	1	0.08
Delhi				ORF1a:L3440I	1	0.1	ORF1a:P1158L	1	0.17	ORF1a:F296L	1	0.08
Delhi				ORF1a:P3359S	1	0.1	ORF1a:R4387C	1	0.17	ORF1a:I3146F	1	0.08
Delhi				ORF1a:P1147S	1	0.1	ORF1a:L3027F	1	0.17	ORF1a:G604C	1	0.08
Delhi				ORF1a:Q1813H	1	0.1	ORF1a:R3999H	1	0.17	ORF1a:A2828S	1	0.08
Delhi				ORF1a:T2021I	1	0.1	ORF1a:T1035I	1	0.17	ORF1a:P1497L	1	0.08
Delhi				ORF1a:Q1201L	1	0.1	ORF1a:T3058I	1	0.17	ORF1a:N310K	1	0.08
Delhi				ORF1a:V306F	1	0.1	ORF1a:T3150I	1	0.17	ORF1a:S2352R	1	0.08
Delhi				ORF1a:P1810L	1	0.1	ORF1a:S2981F	1	0.17	ORF1a:H2659Y	1	0.08
Delhi				ORF1a:I476T	1	0.1	ORF1a:S2695C	1	0.17	ORF1a:G2499S	1	0.08
Delhi				ORF1a:D1229G	1	0.1	ORF1a:M315I	1	0.17	ORF1a:T3356I	1	0.08
Delhi				ORF1a:N1094D	1	0.1	ORF1a:L2882F	1	0.17	ORF1a:L2969R	1	0.08
Delhi				ORF1a:K2497R	1	0.1	ORF1a:T3150A	1	0.17	ORF1a:M4390V	1	0.08
Delhi				ORF1a:C357S	1	0.1	ORF1a:T224I	1	0.17	ORF1a:P748L	1	0.08
Delhi				ORF1a:S3001T	1	0.1	ORF1a:T1168A	1	0.17	ORF1a:K3499R	1	0.08
Delhi				ORF1a:M4375I	1	0.1	ORF1a:I1259V	1	0.17	ORF1a:P926H	1	0.08
Delhi				ORF1a:T770I	1	0.1	ORF1a:G2035R	1	0.17	ORF1a:T1721I	1	0.08
Delhi				ORF1a:V1765I	1	0.1	ORF1a:A859V	1	0.17	ORF1a:I453T	1	0.08
Delhi				ORF1a:T551I	1	0.1	ORF1a:S2822P	1	0.17	ORF1a:A1380V	1	0.08
Delhi				ORF1a:D629H	1	0.1	ORF1a:G2294S	1	0.17	ORF1a:Q2724H	1	0.08
Delhi				ORF1a:S838R	1	0.1	ORF1a:M3655I	1	0.17	ORF1a:I1359T	1	0.08
Delhi				ORF1a:S1468F	1	0.1	ORF1a:C1718N	1	0.17	ORF1a:V121F	1	0.08
Delhi				ORF1a:A668V	1	0.1	ORF1a:D1742V	1	0.17	ORF1a:S3547G	1	0.08
Delhi				ORF1a:L1676I	1	0.1	ORF1a:T2202D	1	0.17	ORF1a:P1472S	1	0.08
Delhi				ORF1a:M731I	1	0.1	ORF1a:V2203Y	1	0.17	ORF1a:M1769I	1	0.08
Delhi				ORF1a:T3356A	1	0.1	ORF1a:K2204Y	1	0.17	ORF1a:N3537D	1	0.08
Delhi				ORF1a:V665F	1	0.1	ORF1a:G2207T	1	0.17	ORF1a:K2017T	1	0.08
Delhi				ORF1a:V1751L	1	0.1	ORF1a:K2208*	1	0.17	ORF1a:E102A	1	0.08
Delhi				ORF1a:V2665F	1	0.1	ORF1a:L2234N	1	0.17	ORF1a:P309T	1	0.08
Delhi				ORF1a:I1505V	1	0.1	ORF1a:S2237N	1	0.17	ORF1a:T554N	1	0.08
Delhi				ORF1a:A3209V	1	0.1	ORF1a:V2238R	1	0.17	ORF1a:T2152I	1	0.08
Delhi				ORF1a:T350I	1	0.1	ORF1a:S2261F	1	0.17	ORF1a:M3823L	1	0.08
Delhi				ORF1a:S1510F	1	0.1	ORF1a:Y2262R	1	0.17	ORF1a:E633D	1	0.08
Delhi				ORF1a:D2043A	1	0.1	ORF1a:D928Y	1	0.17	ORF1a:T749I	1	0.08
Delhi				ORF1a:V2866M	1	0.1	ORF1a:K1319T	1	0.17	ORF1a:I300F	1	0.08
Delhi				ORF1a:T1552I	1	0.1	ORF1a:I114T	1	0.17	ORF1a:V1880F	1	0.08
Delhi				ORF1a:E2982K	1	0.1	ORF1a:R287K	1	0.17	ORF1a:A3379V	1	0.08
Delhi				ORF1a:S1272G	1	0.1	ORF1a:T1395I	1	0.17	ORF1a:A3497V	1	0.08
Delhi				ORF1a:S2193P	1	0.1	ORF1a:S2972P	1	0.17	ORF1a:E1413D	1	0.08
Delhi				ORF1a:Q1943H	1	0.1				ORF1a:S2500F	1	0.08
Delhi				ORF1a:T2152A	1	0.1				ORF1a:G835C	1	0.08
Delhi				ORF1a:S142L	1	0.1				ORF1a:M1312I	1	0.08
Delhi				ORF1a:L628F	1	0.1				ORF1a:A4396V	1	0.08
Delhi				ORF1a:V1038A	1	0.1				ORF1a:G3676C	1	0.08
Delhi				ORF1a:T2300I	1	0.1				ORF1a:Q3777H	1	0.08
Delhi				ORF1a:A2701V	1	0.1				ORF1a:C2549F	1	0.08
Delhi				ORF1a:T2632I	1	0.1				ORF1a:V3748I	1	0.08
Delhi				ORF1a:V2150A	1	0.1				ORF1a:P804T	1	0.08
Delhi				ORF1a:T2532A	1	0.1				ORF1a:G94D	1	0.08
Delhi				ORF1a:S308L	1	0.1				ORF1a:L3915F	1	0.08
Delhi				ORF1a:F536L	1	0.1				ORF1a:T183N	1	0.08
Delhi				ORF1a:D930Y	1	0.1				ORF1a:V2276A	1	0.08
Delhi				ORF1a:S558F	1	0.1				ORF1a:V2340I	1	0.08
Delhi				ORF1a:Q991K	1	0.1				ORF1a:A1812V	1	0.08
Delhi				ORF1a:D2043H	1	0.1				ORF1a:A2033V	1	0.08
Delhi				ORF1a:S3739A	1	0.1				ORF1a:G150V	1	0.08
Delhi				ORF1a:G1723C	1	0.1				ORF1a:E3310N	1	0.08
Delhi				ORF1a:G2258C	1	0.1				ORF1a:A3529V	1	0.08
Delhi				ORF1a:T2906I	1	0.1				ORF1a:E1377D	1	0.08
Delhi				ORF1a:G894S	1	0.1				ORF1a:C3766Y	1	0.08
Delhi				ORF1a:L3694F	1	0.1				ORF1a:T224I	1	0.08
Delhi				ORF1a:P804L	1	0.1				ORF1a:E1223A	1	0.08

Delhi				**ORF1a:P927S**	1	0.1				**ORF1a:R1464W**	1	0.08
Delhi				**ORF1a:T1521I**	1	0.1				**ORF1a:D2060Y**	1	0.08
Delhi				**ORF1a:V1169A**	1	0.1				**ORF1a:T2069I**	1	0.08
Delhi				**ORF1a:L27I**	1	0.1				**ORF1a:A2759T**	1	0.08
Delhi				**ORF1a:G963D**	1	0.1				**ORF1a:G105C**	1	0.08
Delhi				**ORF1a:A4162V**	1	0.1				**ORF1a:T727A**	1	0.08
Delhi				**ORF1a:M1312I**	1	0.1				**ORF1a:T2800A**	1	0.08
Delhi				**ORF1a:L3674F**	1	0.1				**ORF1a:R2991S**	1	0.08
Delhi				**ORF1a:K3678R**	1	0.1				**ORF1a:E390K**	1	0.08
Delhi				**ORF1a:K2622N**	1	0.1				**ORF1a:S887A**	1	0.08
Delhi				**ORF1a:P1950S**	1	0.1				**ORF1a:V1187I**	1	0.08
Delhi				**ORF1a:S2822A**	1	0.1				**ORF1a:E328A**	1	0.08
Delhi				**ORF1a:N3928D**	1	0.1				**ORF1a:N2966S**	1	0.08
Delhi				**ORF1a:V1477I**	1	0.1				**ORF1a:L3644F**	1	0.08
Delhi				**ORF1a:T3665A**	1	0.1				**ORF1a:H3076Y**	1	0.08
Delhi				**ORF1a:M2606L**	1	0.1				**ORF1a:C3423F**	1	0.08
Delhi				**ORF1a:E10A**	1	0.1				**ORF1a:A4262V**	1	0.08
Delhi				**ORF1a:P1213L**	1	0.1				**ORF1a:A3697V**	1	0.08
Delhi				**ORF1a:P2739S**	1	0.1				**ORF1a:I3522T**	1	0.08
Delhi				**ORF1a:L27F**	1	0.1				**ORF1a:D827A**	1	0.08
Delhi				**ORF1a:T677I**	1	0.1				**ORF1a:I1128V**	1	0.08
Delhi				**ORF1a:G1101V**	1	0.1				**ORF1a:R560C**	1	0.08
Delhi				**ORF1a:L4126F**	1	0.1				**ORF1a:D1440Y**	1	0.08
Delhi				**ORF1a:M2194I**	1	0.1				**ORF1a:S2552P**	1	0.08
Delhi				**ORF1a:V2975L**	1	0.1				**ORF1a:V779F**	1	0.08
Delhi				**ORF1a:F143L**	1	0.1				**ORF1a:S1905F**	1	0.08
Delhi				**ORF1a:A1432V**	1	0.1				**ORF1a:T2183I**	1	0.08
Delhi				**ORF1a:E311N**	1	0.1				**ORF1a:A3755S**	1	0.08
Delhi				**ORF1a:S1029I**	1	0.1				**ORF1a:I4205T**	1	0.08
Delhi				**ORF1a:G3846S**	1	0.1				**ORF1a:Q3893R**	1	0.08
Delhi				**ORF1a:L3899I**	1	0.1				**ORF1a:V306F**	1	0.08
Delhi				**ORF1a:I2873V**	1	0.1				**ORF1a:E4097G**	1	0.08
Delhi				**ORF1a:A486V**	1	0.1				**ORF1a:A3705V**	1	0.08
Delhi				**ORF1a:T2166I**	1	0.1				**ORF1a:R3267K**	1	0.08
Delhi				**ORF1a:R2726Q**	1	0.1				**ORF1a:P3504L**	1	0.08
Delhi				**ORF1a:L3591I**	1	0.1				**ORF1a:I2538T**	1	0.08
Delhi				**ORF1a:N4051S**	1	0.1				**ORF1a:A668V**	1	0.08
Delhi				**ORF1a:H3076Y**	1	0.1				**ORF1a:L716F**	1	0.08
Delhi				**ORF1a:S443P**	1	0.1				**ORF1a:Q4392H**	1	0.08
Delhi				**ORF1a:P892S**	1	0.1				**ORF1a:V660A**	1	0.08
Delhi				**ORF1a:I1215V**	1	0.1				**ORF1a:P1213L**	1	0.08
Delhi				**ORF1a:P1207L**	1	0.1				**ORF1a:Q2702H**	1	0.08
Delhi				**ORF1a:P3504H**	1	0.1				**ORF1a:D2924Y**	1	0.08
Delhi				**ORF1a:T3905I**	1	0.1				**ORF1a:A2554V**	1	0.08
Delhi				**ORF1a:A4005S**	1	0.1				**ORF1a:S887F**	1	0.08
Delhi				**ORF1a:A429V**	1	0.1				**ORF1a:T1538I**	1	0.08
Delhi				**ORF1a:V3560I**	1	0.1				**ORF1a:T3461I**	1	0.08
Delhi				**ORF1a:V459A**	1	0.1				**ORF1a:T1945N**	1	0.08
Delhi				**ORF1a:E633D**	1	0.1				**ORF1a:I695V**	1	0.08
Delhi				**ORF1a:E2982D**	1	0.1				**ORF1a:V1888I**	1	0.08
Delhi				**ORF1a:D953G**	1	0.1				**ORF1a:T346I**	1	0.08
Delhi				**ORF1a:S1102G**	1	0.1				**ORF1a:A3969T**	1	0.08
Delhi				**ORF1a:A2850V**	1	0.1				**ORF1a:S546L**	1	0.08
Delhi				**ORF1a:R4132K**	1	0.1				**ORF1a:T2615I**	1	0.08
Delhi				**ORF1a:K1529R**	1	0.1				**ORF1a:A4155T**	1	0.08
Delhi				**ORF1a:A3497V**	1	0.1				**ORF1a:A372T**	1	0.08
Delhi				**ORF1a:G4201E**	1	0.1				**ORF1a:Q2563H**	1	0.08
Delhi				**ORF1a:T891I**	1	0.1				**ORF1a:T2300I**	1	0.08
Delhi				**ORF1a:T2846I**	1	0.1				**ORF1a:S3517Y**	1	0.08
Delhi				**ORF1a:I523V**	1	0.1				**ORF1a:S34T**	1	0.08
Delhi				**ORF1a:G1531C**	1	0.1				**ORF1a:G514R**	1	0.08
Delhi				**ORF1a:T4161N**	1	0.1				**ORF1a:N840S**	1	0.08
Delhi				**ORF1a:V2542A**	1	0.1				**ORF1a:L864P**	1	0.08
Delhi				**ORF1a:G465V**	1	0.1				**ORF1a:S3344F**	1	0.08
Delhi				**ORF1a:P1207S**	1	0.1				**ORF1a:A1204V**	1	0.08
Delhi				**ORF1a:G2118V**	1	0.1				**ORF1a:K1343R**	1	0.08
Delhi				**ORF1a:S2214L**	1	0.1				**ORF1a:T3058I**	1	0.08
Delhi				**ORF1a:A3657V**	1	0.1				**ORF1a:V1295A**	1	0.08
Delhi				**ORF1a:E235D**	1	0.1				**ORF1a:E940G**	1	0.08
Delhi				**ORF1a:F679L**	1	0.1				**ORF1a:K1132R**	1	0.08
Delhi				**ORF1a:K1470R**	1	0.1				**ORF1a:A3523V**	1	0.08
Delhi				**ORF1a:E390K**	1	0.1				**ORF1a:K1051N**	1	0.08
Delhi				**ORF1a:E942G**	1	0.1				**ORF1a:T1876I**	1	0.08

Delhi				**ORF1a:L3667F**	1	0.1				**ORF1a:S3675F**	1	0.08
Delhi				**ORF1a:V757I**	1	0.1				**ORF1a:T1760I**	1	0.08
Delhi				**ORF1a:A1342S**	1	0.1				**ORF1a:G1068E**	1	0.08
Delhi				**ORF1a:T2859I**	1	0.1				**ORF1a:A1809T**	1	0.08
Delhi				**ORF1a:T727I**	1	0.1				**ORF1a:P1010S**	1	0.08
Delhi				**ORF1a:M1769I**	1	0.1				**ORF1a:T1241I**	1	0.08
Delhi				**ORF1a:D1440N**	1	0.1				**ORF1a:V2051I**	1	0.08
Delhi				**ORF1a:V2164F**	1	0.1				**ORF1a:T4090I**	1	0.08
Delhi				**ORF1a:T2186A**	1	0.1				**ORF1a:I2286T**	1	0.08
Delhi				**ORF1a:A3040S**	1	0.1				**ORF1a:A3040V**	1	0.08
Delhi				**ORF1a:L2122F**	1	0.1				**ORF1a:A520V**	1	0.08
Delhi				**ORF1a:M315I**	1	0.1				**ORF1a:M584V**	1	0.08
Delhi				**ORF1a:T1000I**	1	0.1				**ORF1a:H1381Y**	1	0.08
Delhi				**ORF1a:R4387C**	1	0.1				**ORF1a:A3995V**	1	0.08
Delhi				**ORF1a:T602I**	1	0.1				**ORF1a:G150C**	1	0.08
Delhi				**ORF1a:L349V**	1	0.1				**ORF1a:E1196A**	1	0.08
Delhi				**ORF1a:D795Y**	1	0.1				**ORF1a:P971S**	1	0.08
Delhi				**ORF1a:G334S**	1	0.1				**ORF1a:M1588S**	1	0.08
Delhi				**ORF1a:K3353N**	1	0.1				**ORF1a:T1589C**	1	0.08
Delhi				**ORF1a:T602A**	1	0.1				**ORF1a:I1667***	1	0.08
Delhi				**ORF1a:E37K**	1	0.1				**ORF1a:K1668W**	1	0.08
Delhi				**ORF1a:I222V**	1	0.1				**ORF1a:W1669F**	1	0.08
Delhi				**ORF1a:H45D**	1	0.1				**ORF1a:A1670N**	1	0.08
Delhi				**ORF1a:A206V**	1	0.1				**ORF1a:L1681S**	1	0.08
Delhi				**ORF1a:T821I**	1	0.1				**ORF1a:P2110N**	1	0.08
Delhi				**ORF1a:V649F**	1	0.1				**ORF1a:N2111G**	1	0.08
Delhi				**ORF1a:V1295G**	1	0.1				**ORF1a:E2112C**	1	0.08
Delhi				**ORF1a:A540V**	1	0.1				**ORF1a:L2234F**	1	0.08
Delhi				**ORF1a:V1143I**	1	0.1				**ORF1a:L2235N**	1	0.08
Delhi				**ORF1a:I280V**	1	0.1				**ORF1a:G2258N**	1	0.08
Delhi				**ORF1a:N997D**	1	0.1				**ORF1a:P2260C**	1	0.08
Delhi				**ORF1a:C2041F**	1	0.1				**ORF1a:S2261F**	1	0.08
Delhi				**ORF1a:I3693T**	1	0.1				**ORF1a:Y2262R**	1	0.08
Delhi				**ORF1a:H1738Y**	1	0.1				**ORF1a:T2264E**	1	0.08
Delhi				**ORF1a:A1061T**	1	0.1				**ORF1a:G2265L**	1	0.08
Delhi				**ORF1a:I3341V**	1	0.1				**ORF1a:R2267***	1	0.08
Delhi				**ORF1a:S3983F**	1	0.1				**ORF1a:E2268C**	1	0.08
Delhi				**ORF1a:T781A**	1	0.1				**ORF1a:G2269H**	1	0.08
Delhi				**ORF1a:L890F**	1	0.1				**ORF1a:L2271C**	1	0.08
Delhi				**ORF1a:E974G**	1	0.1				**ORF1a:G2294L**	1	0.08
Delhi				**ORF1a:D4085G**	1	0.1				**ORF1a:D2296Y**	1	0.08
Delhi				**ORF1a:P3767S**	1	0.1				**ORF1a:S2297W**	1	0.08
Delhi				**ORF1a:S538P**	1	0.1				**ORF1a:L2298F**	1	0.08
Delhi				**ORF1a:S723F**	1	0.1				**ORF1a:Y2301L**	1	0.08
Delhi				**ORF1a:Y906H**	1	0.1				**ORF1a:P2302***	1	0.08
Delhi				**ORF1a:V3367A**	1	0.1				**ORF1a:S2303C**	1	0.08
Delhi				**ORF1a:E471K**	1	0.1				**ORF1a:E2305S**	1	0.08
Delhi				**ORF1a:I1398M**	1	0.1				**ORF1a:T2306***	1	0.08
Delhi				**ORF1a:S549F**	1	0.1				**ORF1a:I2307W**	1	0.08
Delhi				**ORF1a:L2105F**	1	0.1				**ORF1a:Q2308F**	1	0.08
Delhi				**ORF1a:G2959D**	1	0.1				**ORF1a:I2309R**	1	0.08
Delhi				**ORF1a:V274A**	1	0.1				**ORF1a:I2311F**	1	0.08
Delhi				**ORF1a:V2779A**	1	0.1				**ORF1a:F2337C**	1	0.08
Delhi				**ORF1a:V3847I**	1	0.1				**ORF1a:F2338R**	1	0.08
Delhi				**ORF1a:V1143F**	1	0.1				**ORF1a:Y2339V**	1	0.08
Delhi				**ORF1a:E41K**	1	0.1				**ORF1a:Q455H**	1	0.08
Delhi				**ORF1a:P193L**	1	0.1				**ORF1a:P943L**	1	0.08
Delhi				**ORF1a:D935G**	1	0.1				**ORF1a:I1577S**	1	0.08
Delhi				**ORF1a:D928N**	1	0.1				**ORF1a:N1578R**	1	0.08
Delhi				**ORF1a:F70L**	1	0.1				**ORF1a:L1579Q**	1	0.08
Delhi				**ORF1a:G3541E**	1	0.1				**ORF1a:Q1582T**	1	0.08
Delhi				**ORF1a:T1241I**	1	0.1				**ORF1a:P1609W**	1	0.08
Delhi				**ORF1a:S1361P**	1	0.1				**ORF1a:H1610S**	1	0.08
Delhi				**ORF1a:S3244L**	1	0.1				**ORF1a:Y1634***	1	0.08
Delhi				**ORF1a:P4211S**	1	0.1				**ORF1a:Y1635***	1	0.08
Delhi				**ORF1a:R232H**	1	0.1				**ORF1a:T1637S**	1	0.08
Delhi				**ORF1a:E976K**	1	0.1				**ORF1a:D1639C**	1	0.08
Delhi				**ORF1a:S1027G**	1	0.1				**ORF1a:P1640***	1	0.08
Delhi				**ORF1a:V1931I**	1	0.1				**ORF1a:S1641G**	1	0.08
Delhi				**ORF1a:L2609F**	1	0.1				**ORF1a:L1643G**	1	0.08
Delhi				**ORF1a:Q2660R**	1	0.1				**ORF1a:G1644***	1	0.08
Delhi				**ORF1a:T3750I**	1	0.1				**ORF1a:R1645V**	1	0.08
Delhi				**ORF1a:A1283V**	1	0.1				**ORF1a:Y1646H**	1	0.08

Delhi				**ORF1a:T2069I**	1	0.1				**ORF1a:M1647V**	1	0.08
Delhi				**ORF1a:V627I**	1	0.1				**ORF1a:A1649I**	1	0.08
Delhi				**ORF1a:R848K**	1	0.1				**ORF1a:L1650K**	1	0.08
Delhi				**ORF1a:T2183I**	1	0.1				**ORF1a:N1651S**	1	0.08
Delhi				**ORF1a:L1853F**	1	0.1				**ORF1a:T1653***	1	0.08
Delhi				**ORF1a:V2071F**	1	0.1				**ORF1a:S2103G**	1	0.08
Delhi				**ORF1a:P2537S**	1	0.1				**ORF1a:S2104C**	1	0.08
Delhi				**ORF1a:S3246A**	1	0.1				**ORF1a:T2106S**	1	0.08
Delhi				**ORF1a:A702V**	1	0.1				**ORF1a:K2108R**	1	0.08
Delhi										**ORF1a:K2109F**	1	0.08
Delhi										**ORF1a:P2110E**	1	0.08
Delhi										**ORF1a:W2135S**	1	0.08
Delhi										**ORF1a:D2136***	1	0.08
Delhi										**ORF1a:T2137Q**	1	0.08
Delhi										**ORF1a:I2138S**	1	0.08
Delhi										**ORF1a:A2139C**	1	0.08
Delhi										**ORF1a:S2255F**	1	0.08
Delhi										**ORF1a:G2258L**	1	0.08
Delhi										**ORF1a:M2259F**	1	0.08
Delhi										**ORF1a:P2260E**	1	0.08
Delhi										**ORF1a:S2261L**	1	0.08
Delhi										**ORF1a:C2263***	1	0.08
Delhi										**ORF1a:T2264C**	1	0.08
Delhi										**ORF1a:G2265H**	1	0.08
Delhi										**ORF1a:S2289L**	1	0.08
Delhi										**ORF1a:V2290***	1	0.08
Delhi										**ORF1a:G2294H**	1	0.08
Delhi										**ORF1a:K2315F**	1	0.08
Delhi										**ORF1a:W2316R**	1	0.08
Delhi										**ORF1a:D2317N**	1	0.08
Delhi										**ORF1a:L2318Y**	1	0.08
Delhi										**ORF1a:A2320C**	1	0.08
Delhi										**ORF1a:G2322W**	1	0.08
Delhi										**ORF1a:I222V**	1	0.08
Delhi										**ORF1a:M3862I**	1	0.08
Delhi										**ORF1a:S3950F**	1	0.08
Delhi										**ORF1a:S376L**	1	0.08
Delhi										**ORF1a:N1651K**	1	0.08
Delhi										**ORF1a:A1462T**	1	0.08
Delhi										**ORF1a:V2793I**	1	0.08
Delhi										**ORF1a:V1056L**	1	0.08
Delhi										**ORF1a:T2859I**	1	0.08
Delhi										**ORF1a:S2981F**	1	0.08
Delhi										**ORF1a:V3091I**	1	0.08
Delhi										**ORF1a:Q3777R**	1	0.08
Delhi										**ORF1a:L3935M**	1	0.08
Delhi										**ORF1a:S443P**	1	0.08
Delhi										**ORF1a:G2258C**	1	0.08
Delhi										**ORF1a:Y2788F**	1	0.08
Delhi										**ORF1a:L2789S**	1	0.08
Delhi										**ORF1a:T2791***	1	0.08
Delhi										**ORF1a:P2792***	1	0.08
Delhi										**ORF1a:V2793H**	1	0.08
Delhi										**ORF1a:H2794L**	1	0.08
Delhi										**ORF1a:V2795F**	1	0.08
Delhi										**ORF1a:H2799S**	1	0.08
Delhi										**ORF1a:T2800***	1	0.08
Delhi										**ORF1a:D2819Q**	1	0.08
Delhi										**ORF1a:A2821L**	1	0.08
Delhi										**ORF1a:S2822V**	1	0.08
Delhi										**ORF1a:T2823L**	1	0.08
Delhi										**ORF1a:D2824L**	1	0.08
Delhi										**ORF1a:R2841N**	1	0.08
Delhi										**ORF1a:G2842M**	1	0.08
Delhi										**ORF1a:G2843L**	1	0.08
Delhi										**ORF1a:S2844I**	1	0.08
Delhi										**ORF1a:Y2845L**	1	0.08
Delhi										**ORF1a:N2847H**	1	0.08
Delhi										**ORF1a:D2848G**	1	0.08
Delhi										**ORF1a:K2849L**	1	0.08
Delhi										**ORF1a:C2851S**	1	0.08
Delhi										**ORF1a:P2852***	1	0.08
Delhi										**ORF1a:L2853Q**	1	0.08

Delhi										**ORF1a:I2854E**	1	0.08
Delhi										**ORF1a:A2855K**	1	0.08
Delhi										**ORF1a:A2856W**	1	0.08
Delhi										**ORF1a:R2875V**	1	0.08
Delhi										**ORF1a:T2877F**	1	0.08
Delhi										**ORF1a:N2878C**	1	0.08
Delhi										**ORF1a:G2879I**	1	0.08
Delhi										**ORF1a:D2880S**	1	0.08
Delhi										**ORF1a:F2881Y**	1	0.08
Delhi										**ORF1a:H2883E**	1	0.08
Delhi										**ORF1a:P2886V**	1	0.08
Delhi										**ORF1a:R2887Q**	1	0.08
Delhi										**ORF1a:V2888L**	1	0.08
Delhi										**ORF1a:F2889V**	1	0.08
Delhi										**ORF1a:S2890T**	1	0.08
Delhi										**ORF1a:A2891S**	1	0.08
Delhi										**ORF1a:G2893T**	1	0.08
Delhi										**ORF1a:N2894H**	1	0.08
Delhi										**ORF1a:I2895H**	1	0.08
Delhi										**ORF1a:C2896Q**	1	0.08
Delhi										**ORF1a:Y2897N**	1	0.08
Delhi										**ORF1a:T2898L**	1	0.08
Delhi										**ORF1a:P2899***	1	0.08
Delhi										**ORF1a:K2901T**	1	0.08
Delhi										**ORF1a:S2911L**	1	0.08
Delhi										**ORF1a:A2912Q**	1	0.08
Delhi										**ORF1a:C2913H**	1	0.08
Delhi										**ORF1a:V2914Q**	1	0.08
Delhi										**ORF1a:T2936L**	1	0.08
Delhi										**ORF1a:N2937L**	1	0.08
Delhi										**ORF1a:E2940L**	1	0.08
Delhi										**ORF1a:G2941M**	1	0.08
Delhi										**ORF1a:S2942K**	1	0.08
Delhi										**ORF1a:V2955A**	1	0.08
Delhi										**ORF1a:E2970T**	1	0.08
Delhi										**ORF1a:G2971P**	1	0.08
Delhi										**ORF1a:S2972T**	1	0.08
Delhi										**ORF1a:V2973L**	1	0.08
Delhi										**ORF1a:R2974K**	1	0.08
Delhi										**ORF1a:T2977G**	1	0.08
Delhi										**ORF1a:G2995L**	1	0.08
Delhi										**ORF1a:C2997V**	1	0.08
Delhi										**ORF1a:Y3010G**	1	0.08
Delhi										**ORF1a:R3012***	1	0.08
Delhi										**ORF1a:S3013M**	1	0.08
Delhi										**ORF1a:M3030***	1	0.08
Delhi										**ORF1a:F3031I**	1	0.08
Delhi										**ORF1a:T3032Y**	1	0.08
Delhi										**ORF1a:P3033L**	1	0.08
Delhi										**ORF1a:P3037H**	1	0.08
Delhi										**ORF1a:I3038L**	1	0.08
Delhi										**ORF1a:G3039***	1	0.08
Delhi										**ORF1a:A3040***	1	0.08
Delhi										**ORF1a:C3059I**	1	0.08
Delhi										**ORF1a:F3071***	1	0.08
Delhi										**ORF1a:E3073L**	1	0.08
Delhi										**ORF1a:Y3074E**	1	0.08
Delhi										**ORF1a:S3075E**	1	0.08
Delhi										**ORF1a:H3076L**	1	0.08
Delhi										**ORF1a:V3077L**	1	0.08
Delhi										**ORF1a:A3079N**	1	0.08
Delhi										**ORF1a:F3080T**	1	0.08
Delhi										**ORF1a:N3081V**	1	0.08
Delhi										**ORF1a:T3082M**	1	0.08
Delhi										**ORF1a:L3083***	1	0.08
Delhi										**ORF1a:F3085P**	1	0.08
Delhi										**ORF1a:S3088L**	1	0.08
Delhi										**ORF1a:F3089Y**	1	0.08
Delhi										**ORF1a:T3090Y**	1	0.08
Delhi										**ORF1a:V3091S**	1	0.08
Delhi										**ORF1a:T3095Y**	1	0.08
Delhi										**ORF1a:P3096S**	1	0.08
Delhi										**ORF1a:Y3098***	1	0.08

Delhi										**ORF1a:S3099H**	1	0.08
Delhi										**ORF1a:F3100Q**	1	0.08
Delhi										**ORF1a:L3101F**	1	0.08
Delhi										**ORF1a:P3102T**	1	0.08
Delhi										**ORF1a:G3103H**	1	0.08
Delhi										**ORF1a:V3104S**	1	0.08
Delhi										**ORF1a:S3106L**	1	0.08
Delhi										**ORF1a:I3108F**	1	0.08
Delhi										**ORF1a:Y3109I**	1	0.08
Delhi										**ORF1a:Y3111L**	1	0.08
Delhi										**ORF1a:L3112F**	1	0.08
Delhi										**ORF1a:Y3115I**	1	0.08
Delhi										**ORF1a:Q3127L**	1	0.08
Delhi										**ORF1a:W3128M**	1	0.08
Delhi										**ORF1a:V3130F**	1	0.08
Delhi										**ORF1a:M3131L**	1	0.08
Delhi										**ORF1a:T3133Q**	1	0.08
Delhi										**ORF1a:P3134L**	1	0.08
Delhi										**ORF1a:Y3154V**	1	0.08
Delhi										**ORF1a:W3155F**	1	0.08
Delhi										**ORF1a:F3156P**	1	0.08
Delhi										**ORF1a:F3157Q**	1	0.08
Delhi										**ORF1a:N3159I**	1	0.08
Delhi										**ORF1a:Y3160S**	1	0.08
Delhi										**ORF1a:L3161I**	1	0.08
Delhi										**ORF1a:K3162G**	1	0.08
Delhi										**ORF1a:R3163S**	1	0.08
Delhi										**ORF1a:R3164L**	1	0.08
Delhi										**ORF1a:N3168P**	1	0.08
Delhi										**ORF1a:G3169L**	1	0.08
Delhi										**ORF1a:N3186K**	1	0.08
Delhi										**ORF1a:E3188C**	1	0.08
Delhi										**ORF1a:M3189Y**	1	0.08
Delhi										**ORF1a:Y3211R**	1	0.08
Delhi										**ORF1a:Y3226I**	1	0.08
Delhi										**ORF1a:R3227L**	1	0.08
Delhi										**ORF1a:E3228V**	1	0.08
Delhi										**ORF1a:A3229E**	1	0.08
Delhi										**ORF1a:A3230Q**	1	0.08
Delhi										**ORF1a:C3231W**	1	0.08
Delhi										**ORF1a:C3232I**	1	0.08
Delhi										**ORF1a:H3233Q**	1	0.08
Delhi										**ORF1a:A3237L**	1	0.08
Delhi										**ORF1a:N3239V**	1	0.08
Delhi										**ORF1a:D3240V**	1	0.08
Delhi										**ORF1a:F3241I**	1	0.08
Delhi										**ORF1a:N3243T**	1	0.08
Delhi										**ORF1a:G3245V**	1	0.08
Delhi										**ORF1a:S3246T**	1	0.08
Delhi										**ORF1a:D3247Q**	1	0.08
Delhi										**ORF1a:Y3250P**	1	0.08
Delhi										**ORF1a:Q3251L**	1	0.08
Delhi										**ORF1a:P3252S**	1	0.08
Delhi										**ORF1a:A3270L**	1	0.08
Delhi										**ORF1a:P3272H**	1	0.08
Delhi										**ORF1a:G3274H**	1	0.08
Delhi										**ORF1a:K3275L**	1	0.08
Delhi										**ORF1a:E3277K**	1	0.08
Delhi										**ORF1a:G3278L**	1	0.08
Delhi										**ORF1a:C3279R**	1	0.08
Delhi										**ORF1a:M3280V**	1	0.08
Delhi										**ORF1a:Q3282L**	1	0.08
Delhi										**ORF1a:T3284V**	1	0.08
Delhi										**ORF1a:C3285Q**	1	0.08
Delhi										**ORF1a:G3286L**	1	0.08
Delhi										**ORF1a:T3287H**	1	0.08
Delhi										**ORF1a:T3288L**	1	0.08
Delhi										**ORF1a:L3290V**	1	0.08
Delhi										**ORF1a:N3291F**	1	0.08
Delhi										**ORF1a:H3304M**	1	0.08
Delhi										**ORF1a:V3305T**	1	0.08
Delhi										**ORF1a:I3306***	1	0.08
Delhi										**ORF1a:C3307F**	1	0.08

Delhi										**ORF1a:S3309V**	1	0.08
Delhi										**ORF1a:E3310Q**	1	0.08
Delhi										**ORF1a:L3320T**	1	0.08
Delhi										**ORF1a:R3323M**	1	0.08
Delhi										**ORF1a:S3325I**	1	0.08
Delhi										**ORF1a:N3326S**	1	0.08
Delhi										**ORF1a:H3327W**	1	0.08
Delhi										**ORF1a:N3328Y**	1	0.08
Delhi										**ORF1a:F3329R**	1	0.08
Delhi										**ORF1a:Q3346F**	1	0.08
Delhi										**ORF1a:C3348S**	1	0.08
Delhi										**ORF1a:V3349G**	1	0.08
Delhi										**ORF1a:K3351I**	1	0.08
Delhi										**ORF1a:I3369H**	1	0.08
Delhi										**ORF1a:Q3370L**	1	0.08
Delhi										**ORF1a:G3372I**	1	0.08
Delhi										**ORF1a:Q3373S**	1	0.08
Delhi										**ORF1a:T3374L**	1	0.08
Delhi										**ORF1a:S3376H**	1	0.08
Delhi										**ORF1a:V3377H**	1	0.08
Delhi										**ORF1a:N3396V**	1	0.08
Delhi										**ORF1a:C3408L**	1	0.08
Delhi										**ORF1a:G3409L**	1	0.08
Delhi										**ORF1a:S3410R**	1	0.08
Delhi										**ORF1a:G3412L**	1	0.08
Delhi										**ORF1a:H3435L**	1	0.08
Delhi										**ORF1a:G3437Q**	1	0.08
Delhi										**ORF1a:D3439L**	1	0.08
Delhi										**ORF1a:E3441T**	1	0.08
Delhi										**ORF1a:N3443K**	1	0.08
Delhi										**ORF1a:F3444Q**	1	0.08
Delhi										**ORF1a:Y3445H**	1	0.08
Delhi										**ORF1a:G3446K**	1	0.08
Delhi										**ORF1a:P3447Q**	1	0.08
Delhi										**ORF1a:F3448L**	1	0.08
Delhi										**ORF1a:D3450R**	1	0.08
Delhi										**ORF1a:R3451T**	1	0.08
Delhi										**ORF1a:W3470L**	1	0.08
Delhi										**ORF1a:Y3472Q**	1	0.08
Delhi										**ORF1a:A3473L**	1	0.08
Delhi										**ORF1a:A3474M**	1	0.08
Delhi										**ORF1a:V3475F**	1	0.08
Delhi										**ORF1a:I3476***	1	0.08
Delhi										**ORF1a:N3477L**	1	0.08
Delhi										**ORF1a:D3479C**	1	0.08
Delhi										**ORF1a:R3480T**	1	0.08
Delhi										**ORF1a:W3481L**	1	0.08
Delhi										**ORF1a:F3482L**	1	0.08
Delhi										**ORF1a:N3484L**	1	0.08
Delhi										**ORF1a:R3485L**	1	0.08
Delhi										**ORF1a:F3486M**	1	0.08
Delhi										**ORF1a:T3488L**	1	0.08
Delhi										**ORF1a:Q3507W**	1	0.08
Delhi										**ORF1a:D3508L**	1	0.08
Delhi										**ORF1a:H3509***	1	0.08
Delhi										**ORF1a:V3510S**	1	0.08
Delhi										**ORF1a:D3511T**	1	0.08
Delhi										**ORF1a:G3514L**	1	0.08
Delhi										**ORF1a:P3515K**	1	0.08
Delhi										**ORF1a:S3517E**	1	0.08
Delhi										**ORF1a:A3518L**	1	0.08
Delhi										**ORF1a:Q3519P**	1	0.08
Delhi										**ORF1a:T3520F**	1	0.08
Delhi										**ORF1a:G3521***	1	0.08
Delhi										**ORF1a:A3523C**	1	0.08
Delhi										**ORF1a:D3526H**	1	0.08
Delhi										**ORF1a:M3527***	1	0.08
Delhi										**ORF1a:C3528K**	1	0.08
Delhi										**ORF1a:A3529N**	1	0.08
Delhi										**ORF1a:S3530Y**	1	0.08
Delhi										**ORF1a:L3531C**	1	0.08
Delhi										**ORF1a:A3548H**	1	0.08
Delhi										**ORF1a:A3571R**	1	0.08

Delhi										**ORF1a:L3584H**	1	0.08
Delhi										**ORF1a:L3585T**	1	0.08
Delhi										**ORF1a:I3587G**	1	0.08
Delhi										**ORF1a:L3588C**	1	0.08
Delhi										**ORF1a:T3589Y**	1	0.08
Delhi										**ORF1a:L3591Q**	1	0.08
Delhi										**ORF1a:L3592F**	1	0.08
Delhi										**ORF1a:V3593***	1	0.08
Delhi										**ORF1a:Q3596L**	1	0.08
Delhi										**ORF1a:S3597N**	1	0.08
Delhi										**ORF1a:T3598G**	1	0.08
Delhi										**ORF1a:Q3599L**	1	0.08
Delhi										**ORF1a:W3600C**	1	0.08
Delhi										**ORF1a:L3602F**	1	0.08
Delhi										**ORF1a:F3605Y**	1	0.08
Delhi										**ORF1a:Y3607L**	1	0.08
Delhi										**ORF1a:E3608L**	1	0.08
Delhi										**ORF1a:N3609W**	1	0.08
Delhi										**ORF1a:F3611L**	1	0.08
Delhi										**ORF1a:P3613L**	1	0.08
Delhi										**ORF1a:F3614C**	1	0.08
Delhi										**ORF1a:A3615L**	1	0.08
Delhi										**ORF1a:M3616L**	1	0.08
Delhi										**ORF1a:G3617L**	1	0.08
Delhi										**ORF1a:I3618Q**	1	0.08
Delhi										**ORF1a:I3619***	1	0.08
Delhi										**ORF1a:A3620C**	1	0.08
Delhi										**ORF1a:M3621L**	1	0.08
Delhi										**ORF1a:A3623N**	1	0.08
Delhi										**ORF1a:F3624I**	1	0.08
Delhi										**ORF1a:A3625S**	1	0.08
Delhi										**ORF1a:M3627H**	1	0.08
Delhi										**ORF1a:K3630C**	1	0.08
Delhi										**ORF1a:H3631F**	1	0.08
Delhi										**ORF1a:K3632C**	1	0.08
Delhi										**ORF1a:H3633Y**	1	0.08
Delhi										**ORF1a:A3634L**	1	0.08
Delhi										**ORF1a:F3635L**	1	0.08
Delhi										**ORF1a:C3637P**	1	0.08
Delhi										**ORF1a:F3639***	1	0.08
Delhi										**ORF1a:S3658I**	1	0.08
Delhi										**ORF1a:W3659L**	1	0.08
Delhi										**ORF1a:V3660I**	1	0.08
Delhi										**ORF1a:M3661W**	1	0.08
Delhi										**ORF1a:R3662S**	1	0.08
Delhi										**ORF1a:M3664W**	1	0.08
Delhi										**ORF1a:T3665I**	1	0.08
Delhi										**ORF1a:D3668I**	1	0.08
Delhi										**ORF1a:M3669L**	1	0.08
Delhi										**ORF1a:D3671C**	1	0.08
Delhi										**ORF1a:T3672L**	1	0.08
Delhi										**ORF1a:S3673V**	1	0.08
Delhi										**ORF1a:S3687***	1	0.08
Delhi										**ORF1a:A3688K**	1	0.08
Delhi										**ORF1a:V3689T**	1	0.08
Delhi										**ORF1a:I3693C**	1	0.08
Delhi										**ORF1a:L3694M**	1	0.08
Delhi										**ORF1a:W3709M**	1	0.08
Delhi										**ORF1a:T3710V**	1	0.08
Delhi										**ORF1a:P1158L**	1	0.08
Delhi										**ORF1a:T1542I**	1	0.08
	**ORF1b**											
**Delhi**	**Pre-Wave 1**			**Wave 1**			**Pre-Wave 2**			**Wave 2**		
Delhi	**Mutation**	**Count**	**percent**	**Mutation**	**Count**	**percent**	**Mutation**	**Count**	**percent**	**Mutation**	**Count**	**percent**
Delhi	**ORF1b:A88V**	198	60.37	**ORF1b:P314L**	952	99.17	**ORF1b:P314L**	538	90.27	**ORF1b:P314L**	1126	90.73
Delhi	**ORF1b:P314L**	87	26.52	**ORF1b:I235T**	38	3.96	**ORF1b:M1352I**	58	9.73	**ORF1b:P1000L**	562	45.29
Delhi	**ORF1b:P2612Q**	15	4.57	**ORF1b:K1873N**	23	2.4	**ORF1b:K2310R**	54	9.06	**ORF1b:G662S**	554	44.64
Delhi	**ORF1b:R2613S**	12	3.66	**ORF1b:A1190V**	19	1.98	**ORF1b:P218L**	50	8.39	**ORF1b:H2285Y**	310	24.98
Delhi	**ORF1b:E1288D**	9	2.74	**ORF1b:L2523F**	18	1.88	**ORF1b:S2312A**	43	7.21	**ORF1b:K2310R**	162	13.05
Delhi	**ORF1b:Y1944***	6	1.83	**ORF1b:K2557R**	17	1.77	**ORF1b:G1129C**	37	6.21	**ORF1b:M1352I**	159	12.81

Delhi	ORF1b:A1620V	3	0.91	ORF1b:Q813H	11	1.15	ORF1b:P314F	29	4.87	ORF1b:P218L	133	10.72
Delhi	ORF1b:F2174Y	3	0.91	ORF1b:T797I	11	1.15	ORF1b:K1383R	17	2.85	ORF1b:G1129C	95	7.66
Delhi	ORF1b:Q435H	2	0.61	ORF1b:A391S	10	1.04	ORF1b:L2523F	12	2.01	ORF1b:A1918V	92	7.41
Delhi	ORF1b:D1869Y	2	0.61	ORF1b:V1493L	10	1.04	ORF1b:I2158L	10	1.68	ORF1b:S2312A	88	7.09
Delhi	ORF1b:P2321L	2	0.61	ORF1b:D1899E	10	1.04	ORF1b:T1545A	8	1.34	ORF1b:P314F	52	4.19
Delhi	ORF1b:A1643V	1	0.3	ORF1b:K2579N	10	1.04	ORF1b:G16D	7	1.17	ORF1b:P1570L	48	3.87
Delhi	ORF1b:L2669F	1	0.3	ORF1b:A1798S	9	0.94	ORF1b:V666I	7	1.17	ORF1b:K2557R	33	2.66
Delhi	ORF1b:G1980*	1	0.3	ORF1b:K1900R	9	0.94	ORF1b:P1095S	7	1.17	ORF1b:P2116S	26	2.1
Delhi	ORF1b:F1950L	1	0.3	ORF1b:S904L	8	0.83	ORF1b:I235T	7	1.17	ORF1b:G219S	19	1.53
Delhi	ORF1b:S1972Y	1	0.3	ORF1b:D1389Y	8	0.83	ORF1b:S638I	6	1.01	ORF1b:K469N	18	1.45
Delhi	ORF1b:C2001Y	1	0.3	ORF1b:T1336I	8	0.83	ORF1b:P821S	6	1.01	ORF1b:M1839I	17	1.37
Delhi	ORF1b:K2063N	1	0.3	ORF1b:S1182L	7	0.73	ORF1b:A1312S	6	1.01	ORF1b:K1383R	13	1.05
Delhi	ORF1b:Q813H	1	0.3	ORF1b:M1677I	7	0.73	ORF1b:P1249L	5	0.84	ORF1b:T2490M	12	0.97
Delhi	ORF1b:P2074S	1	0.3	ORF1b:S2198I	7	0.73	ORF1b:L1701F	5	0.84	ORF1b:L2558I	10	0.81
Delhi	ORF1b:A1895V	1	0.3	ORF1b:P2256S	7	0.73	ORF1b:P2116S	5	0.84	ORF1b:T1511I	9	0.73
Delhi	ORF1b:S2379L	1	0.3	ORF1b:G1129V	7	0.73	ORF1b:T243I	5	0.84	ORF1b:A414V	9	0.73
Delhi	ORF1b:P1594S	1	0.3	ORF1b:L535I	7	0.73	ORF1b:V2685F	5	0.84	ORF1b:T1545A	9	0.73
Delhi	ORF1b:T1817I	1	0.3	ORF1b:M659I	6	0.62	ORF1b:K2557R	4	0.67	ORF1b:A1291S	9	0.73
Delhi	ORF1b:P2612L	1	0.3	ORF1b:K151N	6	0.62	ORF1b:H1087Y	4	0.67	ORF1b:D2138N	9	0.73
Delhi	ORF1b:R2613N	1	0.3	ORF1b:A2306V	6	0.62	ORF1b:D1183Y	4	0.67	ORF1b:T17I	7	0.56
Delhi	ORF1b:E2221Q	1	0.3	ORF1b:A2585S	6	0.62	ORF1b:Q348H	4	0.67	ORF1b:S638I	7	0.56
Delhi	ORF1b:A520V	1	0.3	ORF1b:H1087Y	6	0.62	ORF1b:M657I	4	0.67	ORF1b:L820F	7	0.56
Delhi	ORF1b:V224I	1	0.3	ORF1b:G1824C	6	0.62	ORF1b:M890I	4	0.67	ORF1b:I1299T	6	0.48
Delhi	ORF1b:V767L	1	0.3	ORF1b:V2061L	6	0.62	ORF1b:K469N	4	0.67	ORF1b:H1087Y	6	0.48
Delhi	ORF1b:S1512C	1	0.3	ORF1b:R2257S	6	0.62	ORF1b:T1050N	4	0.67	ORF1b:V666I	6	0.48
Delhi	ORF1b:V1077L	1	0.3	ORF1b:T2537I	5	0.52	ORF1b:P1001S	3	0.5	ORF1b:E1977D	5	0.4
Delhi	ORF1b:A2623S	1	0.3	ORF1b:R905K	5	0.52	ORF1b:T1774I	3	0.5	ORF1b:S2344T	5	0.4
Delhi	ORF1b:A1959V	1	0.3	ORF1b:S1089L	4	0.42	ORF1b:V1644L	3	0.5	ORF1b:S961L	5	0.4
Delhi	ORF1b:G2662C	1	0.3	ORF1b:A1219S	4	0.42	ORF1b:A1525S	3	0.5	ORF1b:A1895T	5	0.4
Delhi	ORF1b:A1805S	1	0.3	ORF1b:H1550Y	4	0.42	ORF1b:V1706L	3	0.5	ORF1b:Y1944F	5	0.4
Delhi	ORF1b:A1190V	1	0.3	ORF1b:V2073L	4	0.42	ORF1b:T1511I	3	0.5	ORF1b:A2028C	5	0.4
Delhi	ORF1b:E2544D	1	0.3	ORF1b:A1525S	4	0.42	ORF1b:A1369S	3	0.5	ORF1b:F2030G	5	0.4
Delhi	ORF1b:H247P	1	0.3	ORF1b:K82R	4	0.42	ORF1b:A1219S	3	0.5	ORF1b:V2034L	5	0.4
Delhi	ORF1b:S2292G	1	0.3	ORF1b:V1170F	4	0.42	ORF1b:E2196D	3	0.5	ORF1b:Y2035C	5	0.4
Delhi	ORF1b:D2290Y	1	0.3	ORF1b:E1728D	4	0.42	ORF1b:S1003G	3	0.5	ORF1b:L2523F	5	0.4
Delhi	ORF1b:Q2635*	1	0.3	ORF1b:D260N	4	0.42	ORF1b:A1040T	3	0.5	ORF1b:T2432I	5	0.4
Delhi	ORF1b:K565Q	1	0.3	ORF1b:D1848E	4	0.42	ORF1b:A1521V	3	0.5	ORF1b:K50N	4	0.32
Delhi	ORF1b:K1054R	1	0.3	ORF1b:K469N	4	0.42	ORF1b:G662S	3	0.5	ORF1b:E1288D	4	0.32
Delhi	ORF1b:D1083Y	1	0.3	ORF1b:T132I	4	0.42	ORF1b:P1000L	3	0.5	ORF1b:V1945L	4	0.32
Delhi	ORF1b:D1988G	1	0.3	ORF1b:K1317R	4	0.42	ORF1b:H2285Y	3	0.5	ORF1b:Y1944C	4	0.32
Delhi	ORF1b:V1945A	1	0.3	ORF1b:A2431V	4	0.42	ORF1b:P1567L	2	0.34	ORF1b:T1621I	3	0.24
Delhi	ORF1b:D1572N	1	0.3	ORF1b:N932Y	4	0.42	ORF1b:V2365F	2	0.34	ORF1b:P1427S	3	0.24
Delhi	ORF1b:N2023D	1	0.3	ORF1b:N974S	4	0.42	ORF1b:T1336I	2	0.34	ORF1b:T1139N	3	0.24
Delhi	ORF1b:P2648S	1	0.3	ORF1b:P1223L	3	0.31	ORF1b:P1287H	2	0.34	ORF1b:T1354I	3	0.24
Delhi	ORF1b:L922*	1	0.3	ORF1b:A2110S	3	0.31	ORF1b:T1896I	2	0.34	ORF1b:E1242D	3	0.24
Delhi	ORF1b:A2596V	1	0.3	ORF1b:L1099F	3	0.31	ORF1b:M1693I	2	0.34	ORF1b:Q813H	3	0.24
Delhi	ORF1b:S2344T	1	0.3	ORF1b:T2432I	3	0.31	ORF1b:A520V	2	0.34	ORF1b:T2548I	3	0.24
Delhi	ORF1b:R1101*	1	0.3	ORF1b:D2090Y	3	0.31	ORF1b:T2165A	2	0.34	ORF1b:V467A	3	0.24
Delhi	ORF1b:T1999A	1	0.3	ORF1b:S1881I	3	0.31	ORF1b:T2432I	2	0.34	ORF1b:V1493L	3	0.24
Delhi	ORF1b:S2599P	1	0.3	ORF1b:P976L	3	0.31	ORF1b:D1869Y	2	0.34	ORF1b:V2371M	3	0.24
Delhi	ORF1b:S2673C	1	0.3	ORF1b:P1727L	3	0.31	ORF1b:T310I	2	0.34	ORF1b:G2337S	3	0.24
Delhi	ORF1b:K2209R	1	0.3	ORF1b:V464F	3	0.31	ORF1b:N1653D	2	0.34	ORF1b:V1984L	3	0.24
Delhi	ORF1b:T2569P	1	0.3	ORF1b:T1274I	3	0.31	ORF1b:L1504F	2	0.34	ORF1b:Y1944W	3	0.24
Delhi	ORF1b:T1050I	1	0.3	ORF1b:A397S	3	0.31	ORF1b:Q813H	2	0.34	ORF1b:A1884S	3	0.24
Delhi	ORF1b:S2597L	1	0.3	ORF1b:V290F	3	0.31	ORF1b:C1880Y	2	0.34	ORF1b:D1306E	3	0.24
Delhi	ORF1b:A2021T	1	0.3	ORF1b:T730I	3	0.31	ORF1b:N932Y	2	0.34	ORF1b:S1159I	2	0.16
Delhi	ORF1b:P2612S	1	0.3	ORF1b:D1869Y	3	0.31	ORF1b:N974S	2	0.34	ORF1b:L1935F	2	0.16
Delhi	ORF1b:G2610C	1	0.3	ORF1b:V2368F	3	0.31	ORF1b:Y1944C	2	0.34	ORF1b:E913D	2	0.16
Delhi	ORF1b:S759N	1	0.3	ORF1b:L2017F	3	0.31	ORF1b:K1873N	2	0.34	ORF1b:F2173L	2	0.16
Delhi	ORF1b:D2576Y	1	0.3	ORF1b:A1291V	3	0.31	ORF1b:L1351F	2	0.34	ORF1b:D2183Y	2	0.16
Delhi	ORF1b:M1397V	1	0.3	ORF1b:Y1200C	3	0.31	ORF1b:P1682S	2	0.34	ORF1b:P1727S	2	0.16
Delhi	ORF1b:Y1464C	1	0.3	ORF1b:R1687C	3	0.31	ORF1b:R2000C	2	0.34	ORF1b:G2220E	2	0.16
Delhi	ORF1b:P1165S	1	0.3	ORF1b:E1977D	3	0.31	ORF1b:L2558I	2	0.34	ORF1b:T310I	2	0.16
Delhi	ORF1b:K1537N	1	0.3	ORF1b:L1220F	3	0.31	ORF1b:E1264D	2	0.34	ORF1b:A517V	2	0.16
Delhi	ORF1b:K963R	1	0.3	ORF1b:T2488M	3	0.31	ORF1b:R2613S	2	0.34	ORF1b:A2222V	2	0.16
Delhi	ORF1b:V2073L	1	0.3	ORF1b:S769C	3	0.31	ORF1b:D870Y	2	0.34	ORF1b:M620I	2	0.16
Delhi	ORF1b:S1779I	1	0.3	ORF1b:A1884S	3	0.31	ORF1b:A1291S	2	0.34	ORF1b:V2371L	2	0.16
Delhi	ORF1b:S2212I	1	0.3	ORF1b:G2180V	3	0.31	ORF1b:K258N	1	0.17	ORF1b:A520V	2	0.16
Delhi				ORF1b:P218L	3	0.31	ORF1b:R2684I	1	0.17	ORF1b:H2388Y	2	0.16
Delhi				ORF1b:L273I	3	0.31	ORF1b:A576V	1	0.17	ORF1b:R1315C	2	0.16
Delhi				ORF1b:I570V	3	0.31	ORF1b:P2256L	1	0.17	ORF1b:A1291V	2	0.16
Delhi				ORF1b:M1197K	3	0.31	ORF1b:V1840F	1	0.17	ORF1b:V1644I	2	0.16
Delhi				ORF1b:T1637I	3	0.31	ORF1b:V2192L	1	0.17	ORF1b:A88V	2	0.16

Delhi				**ORF1b:I2687V**	3	0.31	**ORF1b:M454I**	1	0.17	**ORF1b:V1407F**	2	0.16
Delhi				**ORF1b:A2132V**	2	0.21	**ORF1b:A1231S**	1	0.17	**ORF1b:P1223S**	2	0.16
Delhi				**ORF1b:P1095S**	2	0.21	**ORF1b:A1798S**	1	0.17	**ORF1b:S1089L**	2	0.16
Delhi				**ORF1b:L380I**	2	0.21	**ORF1b:L1179F**	1	0.17	**ORF1b:V1092F**	2	0.16
Delhi				**ORF1b:S2312P**	2	0.21	**ORF1b:D2333G**	1	0.17	**ORF1b:I1799T**	2	0.16
Delhi				**ORF1b:M747I**	2	0.21	**ORF1b:A1291V**	1	0.17	**ORF1b:K771R**	2	0.16
Delhi				**ORF1b:A2268V**	2	0.21	**ORF1b:E1977D**	1	0.17	**ORF1b:A176S**	2	0.16
Delhi				**ORF1b:V2117L**	2	0.21	**ORF1b:S961L**	1	0.17	**ORF1b:D2142Y**	2	0.16
Delhi				**ORF1b:V1691L**	2	0.21	**ORF1b:D53Y**	1	0.17	**ORF1b:T1050I**	2	0.16
Delhi				**ORF1b:T1774I**	2	0.21	**ORF1b:T797I**	1	0.17	**ORF1b:P1727L**	2	0.16
Delhi				**ORF1b:P970L**	2	0.21	**ORF1b:L1449F**	1	0.17	**ORF1b:D100Y**	2	0.16
Delhi				**ORF1b:I1598T**	2	0.21	**ORF1b:G1638C**	1	0.17	**ORF1b:M1156I**	2	0.16
Delhi				**ORF1b:M1352I**	2	0.21	**ORF1b:P1727L**	1	0.17	**ORF1b:K1873N**	2	0.16
Delhi				**ORF1b:G1129C**	2	0.21	**ORF1b:E2164A**	1	0.17	**ORF1b:D727N**	2	0.16
Delhi				**ORF1b:R240K**	2	0.21	**ORF1b:M1397I**	1	0.17	**ORF1b:G2297C**	2	0.16
Delhi				**ORF1b:P2313S**	2	0.21	**ORF1b:E1242A**	1	0.17	**ORF1b:L2382F**	2	0.16
Delhi				**ORF1b:H2065Q**	2	0.21	**ORF1b:R2613C**	1	0.17	**ORF1b:P85L**	2	0.16
Delhi				**ORF1b:P1567L**	2	0.21	**ORF1b:P314S**	1	0.17	**ORF1b:P1567S**	2	0.16
Delhi				**ORF1b:M1397I**	2	0.21	**ORF1b:W2383C**	1	0.17	**ORF1b:N1587T**	1	0.08
Delhi				**ORF1b:A2401S**	2	0.21	**ORF1b:T1173I**	1	0.17	**ORF1b:P1427H**	1	0.08
Delhi				**ORF1b:E1601D**	2	0.21	**ORF1b:D144Y**	1	0.17	**ORF1b:N1311S**	1	0.08
Delhi				**ORF1b:F2254L**	2	0.21	**ORF1b:P1095H**	1	0.17	**ORF1b:R109C**	1	0.08
Delhi				**ORF1b:P218T**	2	0.21	**ORF1b:V464F**	1	0.17	**ORF1b:V326A**	1	0.08
Delhi				**ORF1b:Q1878H**	2	0.21	**ORF1b:R1704S**	1	0.17	**ORF1b:I162M**	1	0.08
Delhi				**ORF1b:D870Y**	2	0.21	**ORF1b:S1779I**	1	0.17	**ORF1b:F2174Y**	1	0.08
Delhi				**ORF1b:M2269I**	2	0.21	**ORF1b:Y2619C**	1	0.17	**ORF1b:Q108P**	1	0.08
Delhi				**ORF1b:S1273L**	2	0.21	**ORF1b:A2306V**	1	0.17	**ORF1b:D54Y**	1	0.08
Delhi				**ORF1b:E1977G**	2	0.21	**ORF1b:A440V**	1	0.17	**ORF1b:V350I**	1	0.08
Delhi				**ORF1b:E2544D**	2	0.21	**ORF1b:T2488M**	1	0.17	**ORF1b:T1274I**	1	0.08
Delhi				**ORF1b:R2613C**	2	0.21	**ORF1b:E913D**	1	0.17	**ORF1b:D1381Y**	1	0.08
Delhi				**ORF1b:L2170I**	2	0.21	**ORF1b:K1835N**	1	0.17	**ORF1b:D2234N**	1	0.08
Delhi				**ORF1b:K50N**	2	0.21	**ORF1b:G2068E**	1	0.17	**ORF1b:L2641S**	1	0.08
Delhi				**ORF1b:M592I**	2	0.21	**ORF1b:T2084I**	1	0.17	**ORF1b:V1706I**	1	0.08
Delhi				**ORF1b:V2371L**	2	0.21	**ORF1b:P2633L**	1	0.17	**ORF1b:F2314L**	1	0.08
Delhi				**ORF1b:P1727S**	2	0.21	**ORF1b:D260N**	1	0.17	**ORF1b:N1018K**	1	0.08
Delhi				**ORF1b:M2025I**	2	0.21	**ORF1b:Y1200C**	1	0.17	**ORF1b:L2362F**	1	0.08
Delhi				**ORF1b:V2287I**	2	0.21	**ORF1b:V1149L**	1	0.17	**ORF1b:S2645N**	1	0.08
Delhi				**ORF1b:L1351F**	2	0.21	**ORF1b:D2323N**	1	0.17	**ORF1b:I847V**	1	0.08
Delhi				**ORF1b:E2092K**	2	0.21	**ORF1b:L1361I**	1	0.17	**ORF1b:G1772V**	1	0.08
Delhi				**ORF1b:T1019I**	2	0.21	**ORF1b:D2576V**	1	0.17	**ORF1b:A1547S**	1	0.08
Delhi				**ORF1b:T634I**	2	0.21	**ORF1b:L2577M**	1	0.17	**ORF1b:A241V**	1	0.08
Delhi				**ORF1b:V1538L**	2	0.21	**ORF1b:Y2578S**	1	0.17	**ORF1b:T1404M**	1	0.08
Delhi				**ORF1b:A2513S**	2	0.21	**ORF1b:K2579L**	1	0.17	**ORF1b:M454I**	1	0.08
Delhi				**ORF1b:I2679L**	2	0.21	**ORF1b:L2580K**	1	0.17	**ORF1b:T730I**	1	0.08
Delhi				**ORF1b:H1606Y**	2	0.21	**ORF1b:M2581E**	1	0.17	**ORF1b:T2165M**	1	0.08
Delhi				**ORF1b:T2099I**	2	0.21	**ORF1b:P1975S**	1	0.17	**ORF1b:A1609S**	1	0.08
Delhi				**ORF1b:M1573I**	2	0.21	**ORF1b:T1202I**	1	0.17	**ORF1b:V1644L**	1	0.08
Delhi				**ORF1b:S1974I**	2	0.21	**ORF1b:T2537I**	1	0.17	**ORF1b:A2480T**	1	0.08
Delhi				**ORF1b:S2212I**	2	0.21	**ORF1b:I2668H**	1	0.17	**ORF1b:G662C**	1	0.08
Delhi				**ORF1b:T2163I**	2	0.21	**ORF1b:L2669S**	1	0.17	**ORF1b:D51Y**	1	0.08
Delhi				**ORF1b:R188L**	2	0.21	**ORF1b:S2670R**	1	0.17	**ORF1b:R1622K**	1	0.08
Delhi				**ORF1b:G1107C**	2	0.21	**ORF1b:L2671S**	1	0.17	**ORF1b:P1851S**	1	0.08
Delhi				**ORF1b:A88V**	2	0.21	**ORF1b:L2672P**	1	0.17	**ORF1b:E135D**	1	0.08
Delhi				**ORF1b:T1896I**	2	0.21	**ORF1b:A1662V**	1	0.17	**ORF1b:V980F**	1	0.08
Delhi				**ORF1b:I2373T**	2	0.21	**ORF1b:T2490M**	1	0.17	**ORF1b:D2423Y**	1	0.08
Delhi				**ORF1b:V1092F**	2	0.21	**ORF1b:F2264V**	1	0.17	**ORF1b:T111A**	1	0.08
Delhi				**ORF1b:A2565S**	2	0.21	**ORF1b:L2265D**	1	0.17	**ORF1b:T2084I**	1	0.08
Delhi				**ORF1b:K2063N**	2	0.21	**ORF1b:L2267V**	1	0.17	**ORF1b:Q1546H**	1	0.08
Delhi				**ORF1b:E910D**	2	0.21	**ORF1b:H2065Q**	1	0.17	**ORF1b:S1273L**	1	0.08
Delhi				**ORF1b:S1187N**	2	0.21	**ORF1b:T2548I**	1	0.17	**ORF1b:N602D**	1	0.08
Delhi				**ORF1b:S1661I**	2	0.21	**ORF1b:E1288D**	1	0.17	**ORF1b:A788S**	1	0.08
Delhi				**ORF1b:E1288D**	2	0.21	**ORF1b:Q1393L**	1	0.17	**ORF1b:D2020G**	1	0.08
Delhi				**ORF1b:M1693I**	2	0.21	**ORF1b:A1428V**	1	0.17	**ORF1b:A1868V**	1	0.08
Delhi				**ORF1b:V1164L**	2	0.21	**ORF1b:I1799T**	1	0.17	**ORF1b:N1591D**	1	0.08
Delhi				**ORF1b:A1443V**	2	0.21	**ORF1b:A1805V**	1	0.17	**ORF1b:D83N**	1	0.08
Delhi				**ORF1b:S1779I**	2	0.21	**ORF1b:A1643V**	1	0.17	**ORF1b:A2132V**	1	0.08
Delhi				**ORF1b:K771R**	2	0.21	**ORF1b:I1448L**	1	0.17	**ORF1b:D1389E**	1	0.08
Delhi				**ORF1b:L1551V**	1	0.1	**ORF1b:Y1944W**	1	0.17	**ORF1b:D815Y**	1	0.08
Delhi				**ORF1b:P1570S**	1	0.1	**ORF1b:V1945L**	1	0.17	**ORF1b:Q2335K**	1	0.08
Delhi				**ORF1b:Y1761C**	1	0.1	**ORF1b:M2417I**	1	0.17	**ORF1b:V1961F**	1	0.08
Delhi				**ORF1b:D2429G**	1	0.1	**ORF1b:I441T**	1	0.17	**ORF1b:I2616M**	1	0.08
Delhi				**ORF1b:A2334V**	1	0.1	**ORF1b:M2644I**	1	0.17	**ORF1b:T2514I**	1	0.08
Delhi				**ORF1b:V2I**	1	0.1	**ORF1b:V2223L**	1	0.17	**ORF1b:M2414L**	1	0.08

Delhi				**ORF1b:V1520M**	1	0.1	**ORF1b:P314H**	1	0.17	**ORF1b:D1899G**	1	0.08
Delhi				**ORF1b:D146N**	1	0.1	**ORF1b:T239I**	1	0.17	**ORF1b:I235T**	1	0.08
Delhi				**ORF1b:I1539T**	1	0.1	**ORF1b:K1947***	1	0.17	**ORF1b:P1936L**	1	0.08
Delhi				**ORF1b:T2165K**	1	0.1	**ORF1b:K2063N**	1	0.17	**ORF1b:T76I**	1	0.08
Delhi				**ORF1b:A1063S**	1	0.1	**ORF1b:V248F**	1	0.17	**ORF1b:V1625G**	1	0.08
Delhi				**ORF1b:D185G**	1	0.1	**ORF1b:M1197I**	1	0.17	**ORF1b:A2159V**	1	0.08
Delhi				**ORF1b:E1602D**	1	0.1	**ORF1b:T2171N**	1	0.17	**ORF1b:K667Q**	1	0.08
Delhi				**ORF1b:K2310N**	1	0.1	**ORF1b:F2173L**	1	0.17	**ORF1b:Y1016C**	1	0.08
Delhi				**ORF1b:M2260I**	1	0.1	**ORF1b:A370V**	1	0.17	**ORF1b:K1017E**	1	0.08
Delhi				**ORF1b:V1402A**	1	0.1	**ORF1b:Q1546H**	1	0.17	**ORF1b:D1042N**	1	0.08
Delhi				**ORF1b:R1315C**	1	0.1	**ORF1b:P1223S**	1	0.17	**ORF1b:A2623V**	1	0.08
Delhi				**ORF1b:A390S**	1	0.1	**ORF1b:V33L**	1	0.17	**ORF1b:V2135M**	1	0.08
Delhi				**ORF1b:D2020Y**	1	0.1	**ORF1b:D1251N**	1	0.17	**ORF1b:L2017F**	1	0.08
Delhi				**ORF1b:E1242D**	1	0.1	**ORF1b:V1945G**	1	0.17	**ORF1b:I2194V**	1	0.08
Delhi				**ORF1b:A1428V**	1	0.1	**ORF1b:N1946G**	1	0.17	**ORF1b:A1033S**	1	0.08
Delhi				**ORF1b:H604Y**	1	0.1	**ORF1b:P85L**	1	0.17	**ORF1b:T2537I**	1	0.08
Delhi				**ORF1b:Y2139H**	1	0.1	**ORF1b:T67N**	1	0.17	**ORF1b:G2127S**	1	0.08
Delhi				**ORF1b:V1021F**	1	0.1	**ORF1b:F68V**	1	0.17	**ORF1b:A440S**	1	0.08
Delhi				**ORF1b:M1581I**	1	0.1	**ORF1b:C3R**	1	0.17	**ORF1b:T2048I**	1	0.08
Delhi				**ORF1b:T1621I**	1	0.1	**ORF1b:M1677I**	1	0.17	**ORF1b:F736C**	1	0.08
Delhi				**ORF1b:V1644I**	1	0.1	**ORF1b:Y512F**	1	0.17	**ORF1b:R2613S**	1	0.08
Delhi				**ORF1b:E2092Q**	1	0.1	**ORF1b:T267M**	1	0.17	**ORF1b:G2151C**	1	0.08
Delhi				**ORF1b:L2213F**	1	0.1	**ORF1b:A414V**	1	0.17	**ORF1b:K2340N**	1	0.08
Delhi				**ORF1b:E601D**	1	0.1	**ORF1b:D54G**	1	0.17	**ORF1b:A2431V**	1	0.08
Delhi				**ORF1b:R1109C**	1	0.1	**ORF1b:N1962K**	1	0.17	**ORF1b:A2407V**	1	0.08
Delhi				**ORF1b:L1681V**	1	0.1	**ORF1b:A2131S**	1	0.17	**ORF1b:G1129V**	1	0.08
Delhi				**ORF1b:R1915K**	1	0.1	**ORF1b:M1596I**	1	0.17	**ORF1b:D2090Y**	1	0.08
Delhi				**ORF1b:D349Y**	1	0.1	**ORF1b:A2431V**	1	0.17	**ORF1b:A2306V**	1	0.08
Delhi				**ORF1b:D1899Y**	1	0.1	**ORF1b:T1274I**	1	0.17	**ORF1b:V311A**	1	0.08
Delhi				**ORF1b:T1540I**	1	0.1	**ORF1b:T1739A**	1	0.17	**ORF1b:P314H**	1	0.08
Delhi				**ORF1b:N2695Y**	1	0.1	**ORF1b:N2535S**	1	0.17	**ORF1b:A1432V**	1	0.08
Delhi				**ORF1b:T1354N**	1	0.1	**ORF1b:A176S**	1	0.17	**ORF1b:A1831V**	1	0.08
Delhi				**ORF1b:D2361Y**	1	0.1	**ORF1b:K1900R**	1	0.17	**ORF1b:P2633S**	1	0.08
Delhi				**ORF1b:I485V**	1	0.1	**ORF1b:M1839I**	1	0.17	**ORF1b:T2163I**	1	0.08
Delhi				**ORF1b:E1184D**	1	0.1	**ORF1b:A1884S**	1	0.17	**ORF1b:Q1011H**	1	0.08
Delhi				**ORF1b:V2406F**	1	0.1	**ORF1b:V684I**	1	0.17	**ORF1b:M2414I**	1	0.08
Delhi				**ORF1b:P1427S**	1	0.1	**ORF1b:V783I**	1	0.17	**ORF1b:V2345A**	1	0.08
Delhi				**ORF1b:K2160N**	1	0.1				**ORF1b:D100N**	1	0.08
Delhi				**ORF1b:Q2635H**	1	0.1				**ORF1b:H1838Y**	1	0.08
Delhi				**ORF1b:R524C**	1	0.1				**ORF1b:A86V**	1	0.08
Delhi				**ORF1b:S997L**	1	0.1				**ORF1b:R276H**	1	0.08
Delhi				**ORF1b:V2493F**	1	0.1				**ORF1b:F2467L**	1	0.08
Delhi				**ORF1b:L505F**	1	0.1				**ORF1b:P1821L**	1	0.08
Delhi				**ORF1b:V2075L**	1	0.1				**ORF1b:L2213F**	1	0.08
Delhi				**ORF1b:A520T**	1	0.1				**ORF1b:M115L**	1	0.08
Delhi				**ORF1b:P1000L**	1	0.1				**ORF1b:T384I**	1	0.08
Delhi				**ORF1b:I2166L**	1	0.1				**ORF1b:I2077V**	1	0.08
Delhi				**ORF1b:G2297V**	1	0.1				**ORF1b:L2508F**	1	0.08
Delhi				**ORF1b:V1852F**	1	0.1				**ORF1b:A616T**	1	0.08
Delhi				**ORF1b:A7V**	1	0.1				**ORF1b:K1447N**	1	0.08
Delhi				**ORF1b:A1643P**	1	0.1				**ORF1b:I2166L**	1	0.08
Delhi				**ORF1b:V1840F**	1	0.1				**ORF1b:S358R**	1	0.08
Delhi				**ORF1b:C2001S**	1	0.1				**ORF1b:V2223L**	1	0.08
Delhi				**ORF1b:T239I**	1	0.1				**ORF1b:S598I**	1	0.08
Delhi				**ORF1b:E1514G**	1	0.1				**ORF1b:Y1784C**	1	0.08
Delhi				**ORF1b:A1662V**	1	0.1				**ORF1b:V1905L**	1	0.08
Delhi				**ORF1b:P314F**	1	0.1				**ORF1b:P1821H**	1	0.08
Delhi				**ORF1b:T2217A**	1	0.1				**ORF1b:D884Y**	1	0.08
Delhi				**ORF1b:T310I**	1	0.1				**ORF1b:V321L**	1	0.08
Delhi				**ORF1b:L1179F**	1	0.1				**ORF1b:K2209R**	1	0.08
Delhi				**ORF1b:E2311K**	1	0.1				**ORF1b:S2027L**	1	0.08
Delhi				**ORF1b:R524H**	1	0.1				**ORF1b:V33L**	1	0.08
Delhi				**ORF1b:H2065Y**	1	0.1				**ORF1b:D1572Y**	1	0.08
Delhi				**ORF1b:T2048I**	1	0.1				**ORF1b:K2579R**	1	0.08
Delhi				**ORF1b:A2105S**	1	0.1				**ORF1b:M2025I**	1	0.08
Delhi				**ORF1b:A1525T**	1	0.1				**ORF1b:E1264D**	1	0.08
Delhi				**ORF1b:T17I**	1	0.1				**ORF1b:L629F**	1	0.08
Delhi				**ORF1b:V1984M**	1	0.1				**ORF1b:D870Y**	1	0.08
Delhi				**ORF1b:S2338L**	1	0.1				**ORF1b:Y1134C**	1	0.08
Delhi				**ORF1b:V1787F**	1	0.1				**ORF1b:T284A**	1	0.08
Delhi				**ORF1b:L1681F**	1	0.1				**ORF1b:M371I**	1	0.08
Delhi				**ORF1b:V1649F**	1	0.1				**ORF1b:A2513S**	1	0.08
Delhi				**ORF1b:A176V**	1	0.1				**ORF1b:A914V**	1	0.08

Delhi				**ORF1b:T1290A**	1	0.1				**ORF1b:D152Y**	1	0.08
Delhi				**ORF1b:D275Y**	1	0.1				**ORF1b:K1199N**	1	0.08
Delhi				**ORF1b:M1197I**	1	0.1				**ORF1b:P2256S**	1	0.08
Delhi				**ORF1b:T1522I**	1	0.1				**ORF1b:L1701F**	1	0.08
Delhi				**ORF1b:T1404M**	1	0.1				**ORF1b:S2339F**	1	0.08
Delhi				**ORF1b:T899I**	1	0.1				**ORF1b:A1219S**	1	0.08
Delhi				**ORF1b:A2589V**	1	0.1				**ORF1b:S2399I**	1	0.08
Delhi				**ORF1b:Q2247K**	1	0.1				**ORF1b:D1042H**	1	0.08
Delhi				**ORF1b:L629F**	1	0.1				**ORF1b:T1453I**	1	0.08
Delhi				**ORF1b:V2206I**	1	0.1				**ORF1b:S1182L**	1	0.08
Delhi				**ORF1b:G814C**	1	0.1				**ORF1b:I1921V**	1	0.08
Delhi				**ORF1b:D2142A**	1	0.1				**ORF1b:R2416S**	1	0.08
Delhi				**ORF1b:Y1147C**	1	0.1				**ORF1b:M1693I**	1	0.08
Delhi				**ORF1b:M657I**	1	0.1				**ORF1b:A7V**	1	0.08
Delhi				**ORF1b:P1001L**	1	0.1				**ORF1b:T1027A**	1	0.08
Delhi				**ORF1b:T2084I**	1	0.1				**ORF1b:Q1878H**	1	0.08
Delhi				**ORF1b:G1824D**	1	0.1				**ORF1b:M890I**	1	0.08
Delhi				**ORF1b:S769N**	1	0.1				**ORF1b:G2088C**	1	0.08
Delhi				**ORF1b:V2216F**	1	0.1				**ORF1b:T1739A**	1	0.08
Delhi				**ORF1b:M991V**	1	0.1				**ORF1b:N2593S**	1	0.08
Delhi				**ORF1b:I1498M**	1	0.1				**ORF1b:N781Q**	1	0.08
Delhi				**ORF1b:V345L**	1	0.1				**ORF1b:E793G**	1	0.08
Delhi				**ORF1b:G1756E**	1	0.1				**ORF1b:T794L**	1	0.08
Delhi				**ORF1b:K1835N**	1	0.1				**ORF1b:D795R**	1	0.08
Delhi				**ORF1b:C455F**	1	0.1				**ORF1b:V811L**	1	0.08
Delhi				**ORF1b:E1167D**	1	0.1				**ORF1b:V839***	1	0.08
Delhi				**ORF1b:M187I**	1	0.1				**ORF1b:T841Q**	1	0.08
Delhi				**ORF1b:K2115G**	1	0.1				**ORF1b:D842M**	1	0.08
Delhi				**ORF1b:T2453I**	1	0.1				**ORF1b:G843V**	1	0.08
Delhi				**ORF1b:A391T**	1	0.1				**ORF1b:T844H**	1	0.08
Delhi				**ORF1b:L1783M**	1	0.1				**ORF1b:Y894M**	1	0.08
Delhi				**ORF1b:G1217S**	1	0.1				**ORF1b:V896***	1	0.08
Delhi				**ORF1b:K1537R**	1	0.1				**ORF1b:M897Q**	1	0.08
Delhi				**ORF1b:G977C**	1	0.1				**ORF1b:L898D**	1	0.08
Delhi				**ORF1b:G1568S**	1	0.1				**ORF1b:N900I**	1	0.08
Delhi				**ORF1b:A2168S**	1	0.1				**ORF1b:T903L**	1	0.08
Delhi				**ORF1b:S1512N**	1	0.1				**ORF1b:L922F**	1	0.08
Delhi				**ORF1b:V1760L**	1	0.1				**ORF1b:Q923M**	1	0.08
Delhi				**ORF1b:R2308L**	1	0.1				**ORF1b:A924R**	1	0.08
Delhi				**ORF1b:L518F**	1	0.1				**ORF1b:S1003N**	1	0.08
Delhi				**ORF1b:G940D**	1	0.1				**ORF1b:F1004H**	1	0.08
Delhi				**ORF1b:A788V**	1	0.1				**ORF1b:T1019H**	1	0.08
Delhi				**ORF1b:P2162S**	1	0.1				**ORF1b:G1022L**	1	0.08
Delhi				**ORF1b:E2614A**	1	0.1				**ORF1b:S1023M**	1	0.08
Delhi				**ORF1b:I1074V**	1	0.1				**ORF1b:N1025H**	1	0.08
Delhi				**ORF1b:S2339F**	1	0.1				**ORF1b:E1051K**	1	0.08
Delhi				**ORF1b:C1976***	1	0.1				**ORF1b:L1053S**	1	0.08
Delhi				**ORF1b:A1521V**	1	0.1				**ORF1b:V1077L**	1	0.08
Delhi				**ORF1b:K254N**	1	0.1				**ORF1b:E1079R**	1	0.08
Delhi				**ORF1b:P976S**	1	0.1				**ORF1b:V1080H**	1	0.08
Delhi				**ORF1b:S1458L**	1	0.1				**ORF1b:S1082L**	1	0.08
Delhi				**ORF1b:F1484L**	1	0.1				**ORF1b:D1083V**	1	0.08
Delhi				**ORF1b:K2063R**	1	0.1				**ORF1b:R1084N**	1	0.08
Delhi				**ORF1b:G2510V**	1	0.1				**ORF1b:E1085L**	1	0.08
Delhi				**ORF1b:V1814F**	1	0.1				**ORF1b:L1086D**	1	0.08
Delhi				**ORF1b:I2158M**	1	0.1				**ORF1b:L1088H**	1	0.08
Delhi				**ORF1b:A1521S**	1	0.1				**ORF1b:W1090T**	1	0.08
Delhi				**ORF1b:K2385R**	1	0.1				**ORF1b:K1112I**	1	0.08
Delhi				**ORF1b:M1765I**	1	0.1				**ORF1b:N1113M**	1	0.08
Delhi				**ORF1b:F2353I**	1	0.1				**ORF1b:Y1140***	1	0.08
Delhi				**ORF1b:M2414I**	1	0.1				**ORF1b:K1141M**	1	0.08
Delhi				**ORF1b:K2367N**	1	0.1				**ORF1b:P1157I**	1	0.08
Delhi				**ORF1b:R240G**	1	0.1				**ORF1b:G1174M**	1	0.08
Delhi				**ORF1b:S1003G**	1	0.1				**ORF1b:P1177T**	1	0.08
Delhi				**ORF1b:M991T**	1	0.1				**ORF1b:T1178Q**	1	0.08
Delhi				**ORF1b:S1779N**	1	0.1				**ORF1b:L1179H**	1	0.08
Delhi				**ORF1b:V2685F**	1	0.1				**ORF1b:N1180S**	1	0.08
Delhi				**ORF1b:P1975S**	1	0.1				**ORF1b:G1196Q**	1	0.08
Delhi				**ORF1b:S2148F**	1	0.1				**ORF1b:Q1198S**	1	0.08
Delhi				**ORF1b:E1264D**	1	0.1				**ORF1b:K1199F**	1	0.08
Delhi				**ORF1b:V2199F**	1	0.1				**ORF1b:Y1200L**	1	0.08
Delhi				**ORF1b:E2164A**	1	0.1				**ORF1b:S1201A**	1	0.08
Delhi				**ORF1b:T1817I**	1	0.1				**ORF1b:T1202M**	1	0.08

Delhi				**ORF1b:M1596I**	1	0.1				**ORF1b:A1219L**	1	0.08
Delhi				**ORF1b:M2025V**	1	0.1				**ORF1b:Y1221A**	1	0.08
Delhi				**ORF1b:A86V**	1	0.1				**ORF1b:Y1222***	1	0.08
Delhi				**ORF1b:A1843S**	1	0.1				**ORF1b:P1223L**	1	0.08
Delhi				**ORF1b:C2551Y**	1	0.1				**ORF1b:A1231T**	1	0.08
Delhi				**ORF1b:L262S**	1	0.1				**ORF1b:C1232L**	1	0.08
Delhi				**ORF1b:T2488P**	1	0.1				**ORF1b:S1233L**	1	0.08
Delhi				**ORF1b:L2672I**	1	0.1				**ORF1b:H1234L**	1	0.08
Delhi				**ORF1b:A2575V**	1	0.1				**ORF1b:A1236***	1	0.08
Delhi				**ORF1b:A914V**	1	0.1				**ORF1b:V1237C**	1	0.08
Delhi				**ORF1b:T1137I**	1	0.1				**ORF1b:D1238I**	1	0.08
Delhi				**ORF1b:V2223L**	1	0.1				**ORF1b:A1239Q**	1	0.08
Delhi				**ORF1b:K50R**	1	0.1				**ORF1b:K1246L**	1	0.08
Delhi				**ORF1b:A241V**	1	0.1				**ORF1b:P1249H**	1	0.08
Delhi				**ORF1b:D2499G**	1	0.1				**ORF1b:I1250V**	1	0.08
Delhi				**ORF1b:N591Y**	1	0.1				**ORF1b:D1251L**	1	0.08
Delhi				**ORF1b:K254R**	1	0.1				**ORF1b:K1252V**	1	0.08
Delhi				**ORF1b:Q2070H**	1	0.1				**ORF1b:C1253***	1	0.08
Delhi				**ORF1b:I623V**	1	0.1				**ORF1b:R1255V**	1	0.08
Delhi				**ORF1b:E2221G**	1	0.1				**ORF1b:I1256L**	1	0.08
Delhi				**ORF1b:L238F**	1	0.1				**ORF1b:T1274K**	1	0.08
Delhi				**ORF1b:P1664S**	1	0.1				**ORF1b:L1275***	1	0.08
Delhi				**ORF1b:Q2295L**	1	0.1				**ORF1b:E1276I**	1	0.08
Delhi				**ORF1b:V2236A**	1	0.1				**ORF1b:Y1278M**	1	0.08
Delhi				**ORF1b:I2273L**	1	0.1				**ORF1b:V1279F**	1	0.08
Delhi				**ORF1b:G814D**	1	0.1				**ORF1b:F1296I**	1	0.08
Delhi				**ORF1b:A503S**	1	0.1				**ORF1b:D1297***	1	0.08
Delhi				**ORF1b:M454I**	1	0.1				**ORF1b:E1298V**	1	0.08
Delhi				**ORF1b:Q1546H**	1	0.1				**ORF1b:I1299L**	1	0.08
Delhi				**ORF1b:V426F**	1	0.1				**ORF1b:K1317P**	1	0.08
Delhi				**ORF1b:P1570L**	1	0.1				**ORF1b:H1318D**	1	0.08
Delhi				**ORF1b:A1959V**	1	0.1				**ORF1b:P1329S**	1	0.08
Delhi				**ORF1b:T385M**	1	0.1				**ORF1b:A1330T**	1	0.08
Delhi				**ORF1b:A647S**	1	0.1				**ORF1b:P1331M**	1	0.08
Delhi				**ORF1b:L1248F**	1	0.1				**ORF1b:R1332C**	1	0.08
Delhi				**ORF1b:V783I**	1	0.1				**ORF1b:M1352***	1	0.08
Delhi				**ORF1b:R240S**	1	0.1				**ORF1b:P1368S**	1	0.08
Delhi				**ORF1b:P2313L**	1	0.1				**ORF1b:A1369S**	1	0.08
Delhi				**ORF1b:K667Q**	1	0.1				**ORF1b:I1371L**	1	0.08
Delhi				**ORF1b:L1943V**	1	0.1				**ORF1b:D1373G**	1	0.08
Delhi				**ORF1b:L2184I**	1	0.1				**ORF1b:T1374V**	1	0.08
Delhi				**ORF1b:I1181V**	1	0.1				**ORF1b:S1376L**	1	0.08
Delhi				**ORF1b:V1092A**	1	0.1				**ORF1b:A1377K**	1	0.08
Delhi				**ORF1b:T1303I**	1	0.1				**ORF1b:L1378H**	1	0.08
Delhi				**ORF1b:V666I**	1	0.1				**ORF1b:V1379I**	1	0.08
Delhi				**ORF1b:V2152I**	1	0.1				**ORF1b:Y1380K**	1	0.08
Delhi				**ORF1b:M2322I**	1	0.1				**ORF1b:D1381T**	1	0.08
Delhi				**ORF1b:T792I**	1	0.1				**ORF1b:K1383Q**	1	0.08
Delhi				**ORF1b:S638I**	1	0.1				**ORF1b:K1385N**	1	0.08
Delhi				**ORF1b:Q866H**	1	0.1				**ORF1b:H1387L**	1	0.08
Delhi				**ORF1b:M897I**	1	0.1				**ORF1b:K1396M**	1	0.08
Delhi				**ORF1b:I1293V**	1	0.1				**ORF1b:F1422K**	1	0.08
Delhi				**ORF1b:S2324I**	1	0.1				**ORF1b:I1448L**	1	0.08
Delhi										**ORF1b:F1469L**	1	0.08
Delhi										**ORF1b:T1470K**	1	0.08
Delhi										**ORF1b:T1472L**	1	0.08
Delhi										**ORF1b:N1485L**	1	0.08
Delhi										**ORF1b:A1487M**	1	0.08
Delhi										**ORF1b:I1488***	1	0.08
Delhi										**ORF1b:R1490F**	1	0.08
Delhi										**ORF1b:K1492***	1	0.08
Delhi										**ORF1b:V1493C**	1	0.08
Delhi										**ORF1b:G1494L**	1	0.08
Delhi										**ORF1b:L1496E**	1	0.08
Delhi										**ORF1b:C1497T**	1	0.08
Delhi										**ORF1b:I1498F**	1	0.08
Delhi										**ORF1b:S1536Y**	1	0.08
Delhi										**ORF1b:V1538Y**	1	0.08
Delhi										**ORF1b:T1540L**	1	0.08
Delhi										**ORF1b:G1541H**	1	0.08
Delhi										**ORF1b:L1542R**	1	0.08
Delhi										**ORF1b:T1549L**	1	0.08
Delhi										**ORF1b:L1551T**	1	0.08

Delhi										**ORF1b:D1554L**	1	0.08
Delhi										**ORF1b:F1557L**	1	0.08
Delhi										**ORF1b:T1559V**	1	0.08
Delhi										**ORF1b:E1560Y**	1	0.08
Delhi										**ORF1b:G1561V**	1	0.08
Delhi										**ORF1b:R1576K**	1	0.08
Delhi										**ORF1b:R1577***	1	0.08
Delhi										**ORF1b:L1578I**	1	0.08
Delhi										**ORF1b:S1580K**	1	0.08
Delhi										**ORF1b:M1581L**	1	0.08
Delhi										**ORF1b:H1606M**	1	0.08
Delhi										**ORF1b:V1607S**	1	0.08
Delhi										**ORF1b:T1627V**	1	0.08
Delhi										**ORF1b:N1628M**	1	0.08
Delhi										**ORF1b:P1630F**	1	0.08
Delhi										**ORF1b:D1650L**	1	0.08
Delhi										**ORF1b:H1672E**	1	0.08
Delhi										**ORF1b:P1675N**	1	0.08
Delhi										**ORF1b:V1686T**	1	0.08
Delhi										**ORF1b:R1687H**	1	0.08
Delhi										**ORF1b:I1688L**	1	0.08
Delhi										**ORF1b:E1715S**	1	0.08
Delhi										**ORF1b:M1719A**	1	0.08
Delhi										**ORF1b:K1720P**	1	0.08
Delhi										**ORF1b:Y1721V**	1	0.08
Delhi										**ORF1b:F1722V**	1	0.08
Delhi										**ORF1b:V1723Y**	1	0.08
Delhi										**ORF1b:K1724V**	1	0.08
Delhi										**ORF1b:C1750D**	1	0.08
Delhi										**ORF1b:W1751V**	1	0.08
Delhi										**ORF1b:H1752P**	1	0.08
Delhi										**ORF1b:D1767R**	1	0.08
Delhi										**ORF1b:V1768L**	1	0.08
Delhi										**ORF1b:Q1769***	1	0.08
Delhi										**ORF1b:Q1770L**	1	0.08
Delhi										**ORF1b:W1771M**	1	0.08
Delhi										**ORF1b:G1772F**	1	0.08
Delhi										**ORF1b:F1773N**	1	0.08
Delhi										**ORF1b:T1774N**	1	0.08
Delhi										**ORF1b:N1776V**	1	0.08
Delhi										**ORF1b:A1798M**	1	0.08
Delhi										**ORF1b:I1799V**	1	0.08
Delhi										**ORF1b:T1801H**	1	0.08
Delhi										**ORF1b:R1802M**	1	0.08
Delhi										**ORF1b:C1803***	1	0.08
Delhi										**ORF1b:A1805V**	1	0.08
Delhi										**ORF1b:E1826L**	1	0.08
Delhi										**ORF1b:K1828N**	1	0.08
Delhi										**ORF1b:A1831T**	1	0.08
Delhi										**ORF1b:A1832W**	1	0.08
Delhi										**ORF1b:C1833L**	1	0.08
Delhi										**ORF1b:R1834L**	1	0.08
Delhi										**ORF1b:E1871Y**	1	0.08
Delhi										**ORF1b:W1872L**	1	0.08
Delhi										**ORF1b:D1882***	1	0.08
Delhi										**ORF1b:A1884N**	1	0.08
Delhi										**ORF1b:K1886S**	1	0.08
Delhi										**ORF1b:Y1916G**	1	0.08
Delhi										**ORF1b:P1917I**	1	0.08
Delhi										**ORF1b:N1919M**	1	0.08
Delhi										**ORF1b:V1922E**	1	0.08
Delhi										**ORF1b:N1946C**	1	0.08
Delhi										**ORF1b:E2072A**	1	0.08
Delhi										**ORF1b:C2551F**	1	0.08
Delhi										**ORF1b:D412N**	1	0.08
Delhi										**ORF1b:A517S**	1	0.08
Delhi										**ORF1b:H1807Y**	1	0.08
Delhi										**ORF1b:V1723L**	1	0.08
Delhi										**ORF1b:R2613H**	1	0.08
Delhi										**ORF1b:Q348H**	1	0.08
Delhi										**ORF1b:P1666L**	1	0.08

	Spike (S)											
Delhi	Pre-Wave 1			Wave 1			Pre-Wave 2			Wave 2		
Delhi	Mutation	Count	percent	Mutation	Count	percent	Mutation	Count	percent	Mutation	Count	percent
Delhi	S:D614G	102	31.1	S:D614G	959	99.9	S:D614G	587	98.49	S:D614G	1231	99.19
Delhi	S:Q677H	8	2.44	S:Q677H	140	14.58	S:P681H	189	31.71	S:P681R	817	65.83
Delhi	S:W152R	2	0.61	S:Q23R	84	8.75	S:T716I	187	31.38	S:L452R	764	61.56
Delhi	S:P25L	2	0.61	S:Q675H	35	3.65	S:D1118H	186	31.21	S:D950N	601	48.43
Delhi	S:N1108S	2	0.61	S:D1146E	35	3.65	S:S982A	183	30.7	S:T478K	596	48.03
Delhi	S:T1077S	1	0.3	S:P681H	35	3.65	S:A570D	172	28.86	S:T19R	594	47.86
Delhi	S:S255F	1	0.3	S:D138Y	26	2.71	S:N501Y	162	27.18	S:R158G	381	30.7
Delhi	S:A879S	1	0.3	S:L5F	19	1.98	S:P681R	66	11.07	S:P681H	318	25.62
Delhi	S:D389M	1	0.3	S:A1078S	17	1.77	S:L452R	62	10.4	S:S982A	314	25.3
Delhi	S:C391I	1	0.3	S:L18F	16	1.67	S:Q1071H	59	9.9	S:T716I	313	25.22
Delhi	S:S929I	1	0.3	S:N440K	15	1.56	S:E484Q	58	9.73	S:A570D	309	24.9
Delhi	S:Q613H	1	0.3	S:G1251V	13	1.35	S:Q677H	52	8.72	S:D1118H	309	24.9
Delhi	S:P1263L	1	0.3	S:S477N	12	1.25	S:E154K	40	6.71	S:N501Y	307	24.74
Delhi	S:T299I	1	0.3	S:D80Y	12	1.25	S:G142D	37	6.21	S:G142D	191	15.39
Delhi	S:T29I	1	0.3	S:T716I	12	1.25	S:T95I	35	5.87	S:E484Q	166	13.38
Delhi	S:G181A	1	0.3	S:W152R	12	1.25	S:F888L	32	5.37	S:Q1071H	159	12.81
Delhi	S:V483F	1	0.3	S:E484K	11	1.15	S:Q52R	31	5.2	S:E154K	116	9.35
Delhi	S:I569T	1	0.3	S:V1230L	10	1.04	S:E484K	30	5.03	S:T95I	109	8.78
Delhi	S:V826L	1	0.3	S:Q677R	10	1.04	S:A67V	27	4.53	S:E484K	72	5.8
Delhi	S:M1237I	1	0.3	S:K558R	10	1.04	S:F157S	26	4.36	S:H1101D	59	4.75
Delhi	S:A93G	1	0.3	S:D1084N	9	0.94	S:N501T	15	2.52	S:Q677H	59	4.75
Delhi	S:L242P	1	0.3	S:I834N	9	0.94	S:A701V	14	2.35	S:Q52R	53	4.27
Delhi	S:A771S	1	0.3	S:V1264L	8	0.83	S:H1101D	14	2.35	S:F888L	53	4.27
Delhi	S:A67V	1	0.3	S:S982A	8	0.83	S:H49Y	13	2.18	S:A67V	52	4.19
Delhi	S:S151I	1	0.3	S:G181R	7	0.73	S:I712V	13	2.18	S:A222V	46	3.71
Delhi	S:E96D	1	0.3	S:M153I	7	0.73	S:D80A	13	2.18	S:L5F	31	2.5
Delhi	S:P26L	1	0.3	S:A570D	7	0.73	S:L18F	12	2.01	S:A701V	23	1.85
Delhi	S:Q675H	1	0.3	S:D1118H	7	0.73	S:D215G	12	2.01	S:D80A	21	1.69
Delhi	S:A845V	1	0.3	S:D138H	7	0.73	S:L24S	12	2.01	S:K417N	20	1.61
Delhi	S:A522V	1	0.3	S:S12F	7	0.73	S:K417N	11	1.85	S:D215G	18	1.45
Delhi	S:P728S	1	0.3	S:N501Y	7	0.73	S:Q675H	9	1.51	S:Q218H	14	1.13
Delhi	S:T470N	1	0.3	S:T29I	6	0.62	S:A27S	8	1.34	S:Y144V	13	1.05
Delhi	S:V772I	1	0.3	S:S939F	6	0.62	S:V382L	7	1.17	S:D1153Y	12	0.97
Delhi	S:I1227V	1	0.3	S:P681R	6	0.62	S:D1153Y	7	1.17	S:A262V	10	0.81
Delhi	S:L7F	1	0.3	S:A879S	6	0.62	S:N440K	7	1.17	S:L1141W	9	0.73
Delhi	S:F318S	1	0.3	S:L54F	6	0.62	S:D138Y	6	1.01	S:V382L	8	0.64
Delhi	S:N30H	1	0.3	S:A222V	6	0.62	S:S884A	5	0.84	S:S939F	7	0.56
Delhi	S:A771V	1	0.3	S:S98F	6	0.62	S:A222V	4	0.67	S:E1072K	7	0.56
Delhi	S:D138Y	1	0.3	S:P25S	5	0.52	S:D138H	4	0.67	S:T76I	7	0.56
Delhi	S:S943P	1	0.3	S:A575S	5	0.52	S:G75R	4	0.67	S:D796H	7	0.56
Delhi	S:N450K	1	0.3	S:T95I	5	0.52	S:Y144V	4	0.67	S:I712V	7	0.56
Delhi	S:A672V	1	0.3	S:H49Y	5	0.52	S:Q23R	4	0.67	S:L18F	6	0.48
Delhi	S:L5F	1	0.3	S:N679K	5	0.52	S:T19R	4	0.67	S:Q613H	5	0.4
Delhi	S:L18F	1	0.3	S:W64L	5	0.52	S:D950N	4	0.67	S:S940F	5	0.4
Delhi	S:E516*	1	0.3	S:T859I	5	0.52	S:E654Q	3	0.5	S:H1101Y	5	0.4
Delhi	S:A243S	1	0.3	S:A688V	5	0.52	S:V1228L	3	0.5	S:D138Y	5	0.4
Delhi	S:E224G	1	0.3	S:D215H	5	0.52	S:M1229I	3	0.5	S:D253N	5	0.4
Delhi	S:K77M	1	0.3	S:G769V	5	0.52	S:L5F	3	0.5	S:N440K	5	0.4
Delhi	S:K558N	1	0.3	S:D88N	5	0.52	S:G1124V	3	0.5	S:F157L	5	0.4
Delhi				S:A348S	5	0.52	S:A262S	3	0.5	S:G1219V	5	0.4
Delhi				S:D1165E	4	0.42	S:A263S	3	0.5	S:V1264L	4	0.32
Delhi				S:A771S	4	0.42	S:A264T	3	0.5	S:H655Y	4	0.32
Delhi				S:A520S	4	0.42	S:R158G	3	0.5	S:H49Y	4	0.32
Delhi				S:T20I	4	0.42	S:T478K	3	0.5	S:E484A	4	0.32
Delhi				S:P26S	4	0.42	S:R102S	2	0.34	S:T572N	4	0.32
Delhi				S:T1117I	4	0.42	S:N439K	2	0.34	S:T859N	4	0.32
Delhi				S:E1202Q	4	0.42	S:K558N	2	0.34	S:T1066N	4	0.32
Delhi				S:G1219V	4	0.42	S:S98F	2	0.34	S:S94F	4	0.32
Delhi				S:R78K	4	0.42	S:T678I	2	0.34	S:V367F	4	0.32
Delhi				S:I1183V	4	0.42	S:S477N	2	0.34	S:K77T	3	0.24
Delhi				S:L176F	4	0.42	S:P809S	2	0.34	S:V1176F	3	0.24
Delhi				S:Y144V	4	0.42	S:Q218H	2	0.34	S:F157S	3	0.24
Delhi				S:N354K	4	0.42	S:V16F	2	0.34	S:S704T	3	0.24
Delhi				S:E1111A	4	0.42	S:M153T	2	0.34	S:L176F	3	0.24
Delhi				S:A27S	3	0.31	S:P812S	2	0.34	S:L24S	3	0.24
Delhi				S:D178N	3	0.31	S:T572I	2	0.34	S:M1229I	3	0.24
Delhi				S:A67V	3	0.31	S:G769A	2	0.34	S:A27S	3	0.24
Delhi				S:E583D	3	0.31	S:N354K	2	0.34	S:K1086R	3	0.24
Delhi				S:G257S	3	0.31	S:E1111A	2	0.34	S:A1026S	2	0.16

Delhi				**S:A262S**	3	0.31	**S:Y1155F**	2	0.34	**S:E583D**	2	0.16
Delhi				**S:Q1208H**	3	0.31	**S:S94F**	2	0.34	**S:K558N**	2	0.16
Delhi				**S:V622F**	3	0.31	**S:V1264L**	2	0.34	**S:N501T**	2	0.16
Delhi				**S:E780Q**	3	0.31	**S:T76I**	2	0.34	**S:D1084Y**	2	0.16
Delhi				**S:L452R**	3	0.31	**S:E780D**	2	0.34	**S:K1205N**	2	0.16
Delhi				**S:T547I**	3	0.31	**S:S155I**	2	0.34	**S:R21T**	2	0.16
Delhi				**S:S247N**	3	0.31	**S:S689I**	2	0.34	**S:G446V**	2	0.16
Delhi				**S:Y248S**	3	0.31	**S:Y145D**	2	0.34	**S:N440T**	2	0.16
Delhi				**S:R102I**	3	0.31	**S:D1146E**	2	0.34	**S:S680F**	2	0.16
Delhi				**S:S929I**	3	0.31	**S:W152L**	2	0.34	**S:D796Y**	2	0.16
Delhi				**S:N148S**	3	0.31	**S:P209T**	1	0.17	**S:V127F**	2	0.16
Delhi				**S:D808N**	3	0.31	**S:V772I**	1	0.17	**S:Q1113K**	2	0.16
Delhi				**S:E309Q**	3	0.31	**S:C1247F**	1	0.17	**S:A647S**	2	0.16
Delhi				**S:S373L**	2	0.21	**S:I434V**	1	0.17	**S:S943N**	2	0.16
Delhi				**S:Q14H**	2	0.21	**S:K1191N**	1	0.17	**S:L141F**	2	0.16
Delhi				**S:T724A**	2	0.21	**S:A522V**	1	0.17	**S:R1091C**	2	0.16
Delhi				**S:D1259Y**	2	0.21	**S:I834N**	1	0.17	**S:V1104L**	2	0.16
Delhi				**S:V1228L**	2	0.21	**S:A352S**	1	0.17	**S:D1146H**	2	0.16
Delhi				**S:Q218H**	2	0.21	**S:N30S**	1	0.17	**S:A475V**	2	0.16
Delhi				**S:V213L**	2	0.21	**S:Y144F**	1	0.17	**S:H245Y**	2	0.16
Delhi				**S:E654K**	2	0.21	**S:N764Y**	1	0.17	**S:E554Q**	2	0.16
Delhi				**S:R214L**	2	0.21	**S:V963A**	1	0.17	**S:T791I**	2	0.16
Delhi				**S:H245Y**	2	0.21	**S:I1115L**	1	0.17	**S:R190K**	2	0.16
Delhi				**S:D1163Y**	2	0.21	**S:L517F**	1	0.17	**S:A262D**	2	0.16
Delhi				**S:V70F**	2	0.21	**S:D111N**	1	0.17	**S:A263G**	2	0.16
Delhi				**S:A672V**	2	0.21	**S:Q677R**	1	0.17	**S:A264S**	2	0.16
Delhi				**S:K77N**	2	0.21	**S:S673T**	1	0.17	**S:Y265D**	2	0.16
Delhi				**S:V90F**	2	0.21	**S:T859I**	1	0.17	**S:R1091L**	2	0.16
Delhi				**S:K1073N**	2	0.21	**S:K77N**	1	0.17	**S:P1263L**	2	0.16
Delhi				**S:T719I**	2	0.21	**S:V127F**	1	0.17	**S:M1237I**	2	0.16
Delhi				**S:P681L**	2	0.21	**S:M902I**	1	0.17	**S:V16F**	2	0.16
Delhi				**S:L1024F**	2	0.21	**S:V622F**	1	0.17	**S:D138H**	1	0.08
Delhi				**S:T1120I**	2	0.21	**S:N679K**	1	0.17	**S:A1078V**	1	0.08
Delhi				**S:Q613H**	2	0.21	**S:E1207D**	1	0.17	**S:Y28H**	1	0.08
Delhi				**S:F157L**	2	0.21	**S:T859N**	1	0.17	**S:D228N**	1	0.08
Delhi				**S:S255F**	2	0.21	**S:A899S**	1	0.17	**S:A706V**	1	0.08
Delhi				**S:L1197I**	2	0.21	**S:N148K**	1	0.17	**S:L582F**	1	0.08
Delhi				**S:E132D**	2	0.21	**S:E1150K**	1	0.17	**S:D215H**	1	0.08
Delhi				**S:P1162L**	2	0.21	**S:Q677K**	1	0.17	**S:K77M**	1	0.08
Delhi				**S:A684V**	2	0.21	**S:F490L**	1	0.17	**S:Q414R**	1	0.08
Delhi				**S:L118F**	2	0.21	**S:A570S**	1	0.17	**S:I1232S**	1	0.08
Delhi				**S:M153T**	2	0.21	**S:D936H**	1	0.17	**S:Q271L**	1	0.08
Delhi				**S:D808H**	2	0.21	**S:K1073N**	1	0.17	**S:E324V**	1	0.08
Delhi				**S:S1252P**	2	0.21	**S:V308L**	1	0.17	**S:D1118Y**	1	0.08
Delhi				**S:I1169F**	2	0.21	**S:D1184G**	1	0.17	**S:C1243F**	1	0.08
Delhi				**S:P812L**	2	0.21	**S:V3G**	1	0.17	**S:P1079S**	1	0.08
Delhi				**S:K558N**	2	0.21	**S:A262V**	1	0.17	**S:L8S**	1	0.08
Delhi				**S:S477I**	2	0.21	**S:A263T**	1	0.17	**S:I1210L**	1	0.08
Delhi				**S:G142A**	2	0.21	**S:Q493R**	1	0.17	**S:Q52H**	1	0.08
Delhi				**S:A570S**	2	0.21	**S:S680F**	1	0.17	**S:V213L**	1	0.08
Delhi				**S:S640F**	2	0.21	**S:T1066N**	1	0.17	**S:K1149N**	1	0.08
Delhi				**S:E1195K**	1	0.1	**S:D796H**	1	0.17	**S:Q607H**	1	0.08
Delhi				**S:T323I**	1	0.1	**S:F490S**	1	0.17	**S:P812L**	1	0.08
Delhi				**S:A522S**	1	0.1	**S:N164S**	1	0.17	**S:A852V**	1	0.08
Delhi				**S:N751K**	1	0.1	**S:I844T**	1	0.17	**S:L1265F**	1	0.08
Delhi				**S:I1114V**	1	0.1	**S:M1237I**	1	0.17	**S:A1087S**	1	0.08
Delhi				**S:D936G**	1	0.1	**S:P25S**	1	0.17	**S:Q787K**	1	0.08
Delhi				**S:R1185L**	1	0.1	**S:S943N**	1	0.17	**S:Q1010L**	1	0.08
Delhi				**S:P809S**	1	0.1	**S:W152R**	1	0.17	**S:I1221V**	1	0.08
Delhi				**S:Q804K**	1	0.1	**S:Y28H**	1	0.17	**S:R246T**	1	0.08
Delhi				**S:V1176F**	1	0.1	**S:P463S**	1	0.17	**S:L922F**	1	0.08
Delhi				**S:S359N**	1	0.1	**S:Y265S**	1	0.17	**S:F1075V**	1	0.08
Delhi				**S:P25L**	1	0.1	**S:L179F**	1	0.17	**S:Q14H**	1	0.08
Delhi				**S:V6L**	1	0.1	**S:E309Q**	1	0.17	**S:F157E**	1	0.08
Delhi				**S:N394T**	1	0.1	**S:S13I**	1	0.17	**S:S813I**	1	0.08
Delhi				**S:I358L**	1	0.1	**S:W152C**	1	0.17	**S:S514F**	1	0.08
Delhi				**S:V615I**	1	0.1	**S:A1078S**	1	0.17	**S:H66Q**	1	0.08
Delhi				**S:V445I**	1	0.1	**S:L938I**	1	0.17	**S:G199C**	1	0.08
Delhi				**S:G476S**	1	0.1	**S:S939F**	1	0.17	**S:E96D**	1	0.08
Delhi				**S:K113R**	1	0.1	**S:D228H**	1	0.17	**S:A522V**	1	0.08
Delhi				**S:D111N**	1	0.1	**S:M900I**	1	0.17	**S:V90I**	1	0.08
Delhi				**S:V503I**	1	0.1	**S:G446V**	1	0.17	**S:S875A**	1	0.08
Delhi				**S:C1247F**	1	0.1	**S:P330L**	1	0.17	**S:T22I**	1	0.08

Delhi				**S:S254F**	1	0.1	**S:V1040F**	1	0.17	**S:D830Y**	1	0.08
Delhi				**S:E702D**	1	0.1	**S:I909V**	1	0.17	**S:E340G**	1	0.08
Delhi				**S:Q314K**	1	0.1	**S:A688V**	1	0.17	**S:E583V**	1	0.08
Delhi				**S:V382L**	1	0.1	**S:M153K**	1	0.17	**S:L244S**	1	0.08
Delhi				**S:L1200F**	1	0.1	**S:L176F**	1	0.17	**S:D1259Y**	1	0.08
Delhi				**S:T73N**	1	0.1	**S:C1235F**	1	0.17	**S:V433I**	1	0.08
Delhi				**S:T676A**	1	0.1	**S:A688S**	1	0.17	**S:S689N**	1	0.08
Delhi				**S:E1150K**	1	0.1	**S:A845S**	1	0.17	**S:A520S**	1	0.08
Delhi				**S:V308L**	1	0.1	**S:S12F**	1	0.17	**S:E654K**	1	0.08
Delhi				**S:E1092D**	1	0.1	**S:I805M**	1	0.17	**S:P174S**	1	0.08
Delhi				**S:G1124V**	1	0.1	**S:V3A**	1	0.17	**S:S750N**	1	0.08
Delhi				**S:D936N**	1	0.1	**S:V1122M**	1	0.17	**S:R214L**	1	0.08
Delhi				**S:L828I**	1	0.1	**S:G1251V**	1	0.17	**S:G184V**	1	0.08
Delhi				**S:D215N**	1	0.1	**S:R847T**	1	0.17	**S:Q675H**	1	0.08
Delhi				**S:A1087S**	1	0.1	**S:H655Y**	1	0.17	**S:N658D**	1	0.08
Delhi				**S:P1213Q**	1	0.1	**S:E156V**	1	0.17	**S:D253G**	1	0.08
Delhi				**S:I68T**	1	0.1	**S:L1203F**	1	0.17	**S:R102S**	1	0.08
Delhi				**S:A1020V**	1	0.1	**S:E583Q**	1	0.17	**S:A688V**	1	0.08
Delhi				**S:P384R**	1	0.1	**S:Y248F**	1	0.17	**S:P631S**	1	0.08
Delhi				**S:R21T**	1	0.1	**S:V367F**	1	0.17	**S:E661D**	1	0.08
Delhi				**S:E654V**	1	0.1	**S:F374L**	1	0.17	**S:V622F**	1	0.08
Delhi				**S:G1219C**	1	0.1	**S:L452M**	1	0.17	**S:N439K**	1	0.08
Delhi				**S:L189F**	1	0.1	**S:T470N**	1	0.17	**S:A1020V**	1	0.08
Delhi				**S:A67S**	1	0.1	**S:D737Y**	1	0.17	**S:T547I**	1	0.08
Delhi				**S:A1020T**	1	0.1				**S:M740I**	1	0.08
Delhi				**S:S494P**	1	0.1				**S:A845S**	1	0.08
Delhi				**S:Q14M**	1	0.1				**S:H207Q**	1	0.08
Delhi				**S:C15F**	1	0.1				**S:Q173H**	1	0.08
Delhi				**S:N17F**	1	0.1				**S:K1073N**	1	0.08
Delhi				**S:T19V**	1	0.1				**S:L54F**	1	0.08
Delhi				**S:T20L**	1	0.1				**S:T618I**	1	0.08
Delhi				**S:R21L**	1	0.1				**S:K814R**	1	0.08
Delhi				**S:T22P**	1	0.1				**S:T732I**	1	0.08
Delhi				**S:Q23L**	1	0.1				**S:D1153H**	1	0.08
Delhi				**S:L24V**	1	0.1				**S:S1175P**	1	0.08
Delhi				**S:A27Q**	1	0.1				**S:P26S**	1	0.08
Delhi				**S:Y28C**	1	0.1				**S:Y145D**	1	0.08
Delhi				**S:T29V**	1	0.1				**S:M1233V**	1	0.08
Delhi				**S:L821V**	1	0.1				**S:S71F**	1	0.08
Delhi				**S:A845S**	1	0.1				**S:P812R**	1	0.08
Delhi				**S:T547K**	1	0.1				**S:T747N**	1	0.08
Delhi				**S:H1101Y**	1	0.1				**S:G181R**	1	0.08
Delhi				**S:D1139Y**	1	0.1				**S:F338L**	1	0.08
Delhi				**S:I844V**	1	0.1				**S:P1069S**	1	0.08
Delhi				**S:T747I**	1	0.1				**S:N679T**	1	0.08
Delhi				**S:A1078V**	1	0.1				**S:T1117I**	1	0.08
Delhi				**S:E340D**	1	0.1				**S:V193L**	1	0.08
Delhi				**S:G75R**	1	0.1				**S:S477N**	1	0.08
Delhi				**S:D215E**	1	0.1				**S:A892V**	1	0.08
Delhi				**S:A243V**	1	0.1				**S:I1114L**	1	0.08
Delhi				**S:V16F**	1	0.1				**S:R246I**	1	0.08
Delhi				**S:G75V**	1	0.1				**S:T747I**	1	0.08
Delhi				**S:T29A**	1	0.1				**S:D215Y**	1	0.08
Delhi				**S:C1243F**	1	0.1				**S:P217S**	1	0.08
Delhi				**S:A67D**	1	0.1				**S:V3G**	1	0.08
Delhi				**S:Q675R**	1	0.1				**S:T572I**	1	0.08
Delhi				**S:E1150D**	1	0.1				**S:E96G**	1	0.08
Delhi				**S:Q607H**	1	0.1				**S:T333A**	1	0.08
Delhi				**S:K795R**	1	0.1				**S:P1162S**	1	0.08
Delhi				**S:A846S**	1	0.1				**S:V1122L**	1	0.08
Delhi				**S:A903S**	1	0.1				**S:S112P**	1	0.08
Delhi				**S:C1235F**	1	0.1				**S:D228H**	1	0.08
Delhi				**S:M1229I**	1	0.1				**S:A892S**	1	0.08
Delhi				**S:Q1201K**	1	0.1				**S:H1101R**	1	0.08
Delhi				**S:H69Y**	1	0.1				**S:S477I**	1	0.08
Delhi				**S:N679Y**	1	0.1				**S:K150N**	1	0.08
Delhi				**S:T124S**	1	0.1				**S:M153V**	1	0.08
Delhi				**S:D1165Y**	1	0.1				**S:E1262V**	1	0.08
Delhi				**S:L858F**	1	0.1				**S:Q23R**	1	0.08
Delhi				**S:G261V**	1	0.1				**S:D1146E**	1	0.08
Delhi				**S:S689I**	1	0.1				**S:G1251V**	1	0.08
Delhi				**S:E1151D**	1	0.1				**S:S98F**	1	0.08
Delhi				**S:E180K**	1	0.1				**S:T19I**	1	0.08

Delhi				S:G252D	1	0.1				S:M153I	1	0.08
Delhi				S:E484Q	1	0.1						
Delhi				S:K1149R	1	0.1						
Delhi				S:F490L	1	0.1						
Delhi				S:D936H	1	0.1						
Delhi				S:T299I	1	0.1						
Delhi				S:A1070S	1	0.1						
Delhi				S:S514F	1	0.1						
Delhi				S:R847K	1	0.1						
Delhi				S:N185S	1	0.1						
Delhi				S:A623S	1	0.1						
Delhi				S:A222P	1	0.1						
Delhi				S:S691A	1	0.1						
Delhi				S:S71P	1	0.1						
Delhi				S:Q957L	1	0.1						
Delhi				S:V595I	1	0.1						
Delhi				S:E281Q	1	0.1						
Delhi				S:A846V	1	0.1						
Delhi				S:A27V	1	0.1						
Delhi				S:G261S	1	0.1						
Delhi				S:T572I	1	0.1						
Delhi				S:V483F	1	0.1						
Delhi				S:I231T	1	0.1						
Delhi				S:V1065L	1	0.1						
Delhi				S:S813N	1	0.1						
Delhi				S:M1237I	1	0.1						
Delhi				S:E1072Q	1	0.1						
Delhi				S:L1197F	1	0.1						
Delhi				S:D1260Y	1	0.1						
Delhi				S:V1133L	1	0.1						
Delhi				S:F306L	1	0.1						
Delhi				S:E406Q	1	0.1						
Delhi				S:N164S	1	0.1						
Delhi				S:V1060I	1	0.1						
Delhi				S:T1273I	1	0.1						
Delhi				S:L841R	1	0.1						
Delhi				S:C1248S	1	0.1						
Delhi				S:D796Y	1	0.1						
Delhi				S:I100V	1	0.1						
Delhi				S:T1027I	1	0.1						
Delhi				S:W258L	1	0.1						
Delhi				S:R634H	1	0.1						
Delhi				S:R346K	1	0.1						
Delhi												
	**Envelope (E)**											
**Delhi**	**Pre-Wave 1**			**Wave 1**			**Pre-Wave 2**			**Wave 2**		
Delhi	**Mutation**	**Count**	**percent**	**Mutation**	**Count**	**percent**	**Mutation**	**Count**	**percent**	**Mutation**	**Count**	**percent**
Delhi	E:L21F	1	0.3	E:I46M	25	2.6	E:L21F	29	4.87	E:L21F	28	2.26
Delhi				E:V47F	25	2.6	E:P71L	12	2.01	E:P71L	20	1.61
Delhi				E:L21F	6	0.62	E:T9I	2	0.34	E:V62F	9	0.73
Delhi				E:S68F	4	0.42	E:T30I	1	0.17	E:L73F	1	0.08
Delhi				E:V49L	2	0.21	E:V25T	1	0.17	E:D72G	1	0.08
Delhi				E:P71L	2	0.21	E:S16N	1	0.17	E:F26L	1	0.08
Delhi				E:P71S	2	0.21	E:L27F	1	0.17			
Delhi				E:A41V	2	0.21	E:P71S	1	0.17			
Delhi				E:D72Y	1	0.1	E:S55F	1	0.17			
Delhi				E:V62F	1	0.1	E:S68F	1	0.17			
Delhi				E:V62I	1	0.1						
Delhi				E:T9I	1	0.1						
Delhi				E:R69I	1	0.1						
Delhi				E:T30I	1	0.1						
Delhi				E:A41S	1	0.1						
Delhi				E:A22S	1	0.1						
Delhi				E:N48*	1	0.1						
	**Membrane (M)**											
**Delhi**	**Pre-Wave 1**			**Wave 1**			**Pre-Wave 2**			**Wave 2**		
Delhi	**Mutation**	**Count**	**percent**	**Mutation**	**Count**	**percent**	**Mutation**	**Count**	**percent**	**Mutation**	**Count**	**percent**
Delhi	M:V70F	1	0.3	M:A2S	9	0.94	M:F28L	42	7.05	M:I82T	662	53.34
Delhi	M:V23L	1	0.3	M:K15R	4	0.42	M:I82S	38	6.38	M:F28L	131	10.56

Delhi	**M:L138I**	1	0.3	**M:M109I**	3	0.31	**M:I82T**	37	6.21	**M:I82S**	94	7.57
Delhi	**M:A85S**	1	0.3	**M:I76M**	2	0.21	**M:H125Y**	3	0.5	**M:D3G**	19	1.53
Delhi				**M:V23L**	2	0.21	**M:D209Y**	2	0.34	**M:H125Y**	4	0.32
Delhi				**M:I76V**	2	0.21	**M:A104V**	1	0.17	**M:L29F**	3	0.24
Delhi				**M:V66L**	2	0.21	**M:L17F**	1	0.17	**M:R107L**	3	0.24
Delhi				**M:D209Y**	2	0.21	**M:D3G**	1	0.17	**M:D209Y**	2	0.16
Delhi				**M:V70F**	2	0.21				**M:D160N**	1	0.08
Delhi				**M:T7I**	1	0.1				**M:A40S**	1	0.08
Delhi				**M:L46F**	1	0.1				**M:R174L**	1	0.08
Delhi				**M:S212G**	1	0.1				**M:A69S**	1	0.08
Delhi				**M:A63S**	1	0.1				**M:A81S**	1	0.08
Delhi				**M:I168V**	1	0.1				**M:T77I**	1	0.08
Delhi				**M:A69S**	1	0.1				**M:T175M**	1	0.08
Delhi				**M:W75L**	1	0.1				**M:V70L**	1	0.08
Delhi				**M:A188S**	1	0.1				**M:Y204H**	1	0.08
Delhi				**M:A63V**	1	0.1				**M:G6C**	1	0.08
Delhi				**M:K162R**	1	0.1				**M:G78C**	1	0.08
Delhi				**M:H125Y**	1	0.1				**M:A152S**	1	0.08
Delhi				**M:C64S**	1	0.1				**M:M109I**	1	0.08
Delhi				**M:S211Y**	1	0.1						
Delhi				**M:R158L**	1	0.1						
Delhi				**M:M84I**	1	0.1						
Delhi				**M:V70L**	1	0.1						
	**Nucleocapsid (N)**											
**Delhi**	**Pre-Wave 1**			**Wave 1**			**Pre-Wave 2**			**Wave 2**		
Delhi	**Mutation**	**Count**	**percent**	**Mutation**	**Count**	**percent**	**Mutation**	**Count**	**percent**	**Mutation**	**Count**	**percent**
Delhi	**N:P13L**	166	50.61	**N:S194L**	509	53.02	**N:R203K**	195	32.72	**N:D377Y**	739	59.55
Delhi	**N:S194L**	80	24.39	**N:G204R**	251	26.15	**N:G204R**	191	32.05	**N:R203M**	705	56.81
Delhi	**N:R203K**	24	7.32	**N:R203K**	250	26.04	**N:S235F**	151	25.34	**N:D63G**	591	47.62
Delhi	**N:G204R**	24	7.32	**N:D377Y**	32	3.33	**N:S194L**	124	20.81	**N:R385K**	312	25.14
Delhi	**N:S190I**	2	0.61	**N:S235F**	22	2.29	**N:D3L**	97	16.28	**N:S235F**	235	18.94
Delhi	**N:G238C**	1	0.3	**N:A211V**	20	2.08	**N:D377Y**	63	10.57	**N:D3L**	216	17.41
Delhi	**N:S184Y**	1	0.3	**N:S2P**	19	1.98	**N:R203M**	60	10.07	**N:G204R**	215	17.32
Delhi	**N:S327L**	1	0.3	**N:M234I**	13	1.35	**N:D3Y**	44	7.38	**N:R203K**	214	17.24
Delhi	**N:G5E**	1	0.3	**N:D3Y**	11	1.15	**N:S2M**	32	5.37	**N:G215C**	97	7.82
Delhi	**N:G147S**	1	0.3	**N:T379I**	10	1.04	**N:A12G**	32	5.37	**N:D3Y**	80	6.45
Delhi	**N:G179C**	1	0.3	**N:S193I**	8	0.83	**N:D3E**	23	3.86	**N:T205I**	57	4.59
Delhi	**N:S184P**	1	0.3	**N:D144Y**	8	0.83	**N:T205I**	19	3.19	**N:S2M**	52	4.19
Delhi	**N:Q7K**	1	0.3	**N:D144H**	8	0.83	**N:I84V**	16	2.68	**N:A12G**	51	4.11
Delhi	**N:A251S**	1	0.3	**N:M322I**	7	0.73	**N:S2P**	11	1.85	**N:D402Y**	22	1.77
Delhi	**N:A217S**	1	0.3	**N:Q9H**	7	0.73	**N:A211V**	7	1.17	**N:D3E**	17	1.37
Delhi	**N:S197L**	1	0.3	**N:A414P**	6	0.62	**N:T362I**	6	1.01	**N:T362I**	16	1.29
Delhi				**N:T205I**	6	0.62	**N:M210I**	5	0.84	**N:L139F**	16	1.29
Delhi				**N:M210I**	6	0.62	**N:P199L**	4	0.67	**N:S194L**	12	0.97
Delhi				**N:G18S**	6	0.62	**N:D144Y**	4	0.67	**N:P13S**	12	0.97
Delhi				**N:H145Y**	5	0.52	**N:D63G**	4	0.67	**N:P67S**	10	0.81
Delhi				**N:D3L**	5	0.52	**N:T271I**	3	0.5	**N:N29K**	8	0.64
Delhi				**N:M322V**	5	0.52	**N:L221F**	3	0.5	**N:D144H**	7	0.56
Delhi				**N:D415G**	4	0.42	**N:P67S**	3	0.5	**N:K169N**	7	0.56
Delhi				**N:Q70K**	4	0.42	**N:R32H**	3	0.5	**N:M234I**	6	0.48
Delhi				**N:Q409L**	4	0.42	**N:R385K**	3	0.5	**N:A252S**	6	0.48
Delhi				**N:N48S**	4	0.42	**N:D402Y**	3	0.5	**N:G170D**	5	0.4
Delhi				**N:E378Q**	4	0.42	**N:M234I**	2	0.34	**N:F171N**	5	0.4
Delhi				**N:Q384H**	4	0.42	**N:K249R**	2	0.34	**N:Y172N**	5	0.4
Delhi				**N:Q418R**	4	0.42	**N:Q289H**	2	0.34	**N:P383S**	4	0.32
Delhi				**N:E367G**	4	0.42	**N:G215C**	2	0.34	**N:D144Y**	4	0.32
Delhi				**N:R209I**	4	0.42	**N:P365L**	2	0.34	**N:R10P**	4	0.32
Delhi				**N:T362I**	4	0.42	**N:G34W**	2	0.34	**N:Q28R**	4	0.32
Delhi				**N:A414S**	4	0.42	**N:D128Y**	2	0.34	**N:D3Q**	4	0.32
Delhi				**N:R195I**	3	0.31	**N:A414S**	2	0.34	**N:S2P**	4	0.32
Delhi				**N:S327L**	3	0.31	**N:D144H**	2	0.34	**N:S2Y**	4	0.32
Delhi				**N:G215C**	3	0.31	**N:R209I**	2	0.34	**N:G18V**	4	0.32
Delhi				**N:L219I**	3	0.31	**N:A252S**	2	0.34	**N:E367D**	3	0.24
Delhi				**N:Q380R**	3	0.31	**N:Q380L**	2	0.34	**N:G200S**	2	0.16
Delhi				**N:Q289H**	3	0.31	**N:P151L**	2	0.34	**N:T135I**	2	0.16
Delhi				**N:V270L**	3	0.31	**N:G19A**	2	0.34	**N:I84V**	2	0.16
Delhi				**N:A119S**	3	0.31	**N:A35V**	2	0.34	**N:T271I**	2	0.16
Delhi				**N:T334I**	3	0.31	**N:T141I**	2	0.34	**N:A55S**	2	0.16
Delhi				**N:D22Y**	3	0.31	**N:G204L**	1	0.17	**N:R209G**	2	0.16
Delhi				**N:S413I**	3	0.31	**N:T366I**	1	0.17	**N:A359S**	2	0.16
Delhi				**N:Q70R**	3	0.31	**N:A220V**	1	0.17	**N:A211V**	2	0.16

Delhi				**N:Q28H**	2	0.21	**N:L139F**	1	0.17	**N:Q389H**	2	0.16
Delhi				**N:D358N**	2	0.21	**N:G124S**	1	0.17	**N:E378A**	2	0.16
Delhi				**N:P279L**	2	0.21	**N:P13T**	1	0.17	**N:P13T**	2	0.16
Delhi				**N:A359S**	2	0.21	**N:G60R**	1	0.17	**N:S202N**	2	0.16
Delhi				**N:T135I**	2	0.21	**N:S190I**	1	0.17	**N:A267S**	2	0.16
Delhi				**N:P13S**	2	0.21	**N:P364Q**	1	0.17	**N:S193N**	1	0.08
Delhi				**N:S190I**	2	0.21	**N:K375N**	1	0.17	**N:G328V**	1	0.08
Delhi				**N:P142S**	2	0.21	**N:P383R**	1	0.17	**N:T265I**	1	0.08
Delhi				**N:P20L**	2	0.21	**N:Q181K**	1	0.17	**N:L230F**	1	0.08
Delhi				**N:L139F**	2	0.21	**N:S188***	1	0.17	**N:E378***	1	0.08
Delhi				**N:P207S**	2	0.21	**N:L219I**	1	0.17	**N:G212C**	1	0.08
Delhi				**N:A217S**	2	0.21	**N:A220T**	1	0.17	**N:A251V**	1	0.08
Delhi				**N:G212V**	2	0.21	**N:S193I**	1	0.17	**N:G212S**	1	0.08
Delhi				**N:P383L**	2	0.21	**N:G236V**	1	0.17	**N:A182S**	1	0.08
Delhi				**N:A12V**	2	0.21	**N:Q418H**	1	0.17	**N:T165I**	1	0.08
Delhi				**N:K373M**	2	0.21	**N:G204P**	1	0.17	**N:Q281K**	1	0.08
Delhi				**N:G34W**	2	0.21	**N:S413I**	1	0.17	**N:M210I**	1	0.08
Delhi				**N:Q7K**	2	0.21	**N:R177I**	1	0.17	**N:S193I**	1	0.08
Delhi				**N:A376S**	2	0.21	**N:G18S**	1	0.17	**N:D288N**	1	0.08
Delhi				**N:T379N**	2	0.21	**N:A119S**	1	0.17	**N:D63H**	1	0.08
Delhi				**N:D402Y**	2	0.21	**N:A217S**	1	0.17	**N:T325I**	1	0.08
Delhi				**N:P199S**	2	0.21	**N:P13S**	1	0.17	**N:D3V**	1	0.08
Delhi				**N:T205S**	2	0.21	**N:L219F**	1	0.17	**N:Q389L**	1	0.08
Delhi				**N:A381S**	2	0.21	**N:D3Q**	1	0.17	**N:P365S**	1	0.08
Delhi				**N:S416L**	1	0.1	**N:G238C**	1	0.17	**N:G179S**	1	0.08
Delhi				**N:P13L**	1	0.1	**N:N8D**	1	0.17	**N:A208S**	1	0.08
Delhi				**N:A398S**	1	0.1	**N:S23P**	1	0.17	**N:K373N**	1	0.08
Delhi				**N:A311S**	1	0.1	**N:D371Y**	1	0.17	**N:A173N**	1	0.08
Delhi				**N:S416T**	1	0.1	**N:P13L**	1	0.17	**N:E174N**	1	0.08
Delhi				**N:T271I**	1	0.1	**N:S206Y**	1	0.17	**N:G175N**	1	0.08
Delhi				**N:D128Y**	1	0.1	**N:A12S**	1	0.17	**N:S176N**	1	0.08
Delhi				**N:P365L**	1	0.1	**N:Q389H**	1	0.17	**N:R177N**	1	0.08
Delhi				**N:T166I**	1	0.1	**N:A398V**	1	0.17	**N:G178N**	1	0.08
Delhi				**N:Q418H**	1	0.1	**N:T265I**	1	0.17	**N:G179N**	1	0.08
Delhi				**N:G238C**	1	0.1	**N:S21P**	1	0.17	**N:S180N**	1	0.08
Delhi				**N:A156S**	1	0.1	**N:T379I**	1	0.17	**N:D348Y**	1	0.08
Delhi				**N:A152S**	1	0.1	**N:G212V**	1	0.17	**N:Q9H**	1	0.08
Delhi				**N:L56F**	1	0.1	**N:P6L**	1	0.17	**N:P199L**	1	0.08
Delhi				**N:P67L**	1	0.1	**N:K169N**	1	0.17	**N:G60R**	1	0.08
Delhi				**N:K374N**	1	0.1	**N:G170D**	1	0.17	**N:R14C**	1	0.08
Delhi				**N:M234L**	1	0.1	**N:F171N**	1	0.17	**N:G214D**	1	0.08
Delhi				**N:T265I**	1	0.1	**N:Y172N**	1	0.17	**N:P80T**	1	0.08
Delhi				**N:I320F**	1	0.1	**N:A267V**	1	0.17	**N:R41L**	1	0.08
Delhi				**N:R385I**	1	0.1				**N:N8T**	1	0.08
Delhi				**N:R191S**	1	0.1				**N:T296I**	1	0.08
Delhi				**N:S194***	1	0.1				**N:N8H**	1	0.08
Delhi				**N:S197***	1	0.1				**N:T379I**	1	0.08
Delhi				**N:A220D**	1	0.1				**N:K127I**	1	0.08
Delhi				**N:T391I**	1	0.1				**N:Q229H**	1	0.08
Delhi				**N:S187P**	1	0.1				**N:Q289H**	1	0.08
Delhi				**N:D22H**	1	0.1				**N:S327L**	1	0.08
Delhi				**N:D371Y**	1	0.1				**N:V270L**	1	0.08
Delhi				**N:A125D**	1	0.1				**N:P383L**	1	0.08
Delhi				**N:G214S**	1	0.1				**N:R209I**	1	0.08
Delhi				**N:D288N**	1	0.1				**N:S2F**	1	0.08
Delhi				**N:T282I**	1	0.1				**N:D358N**	1	0.08
Delhi				**N:T141I**	1	0.1				**N:A90S**	1	0.08
Delhi				**N:S2F**	1	0.1				**N:D401Y**	1	0.08
Delhi				**N:T325I**	1	0.1				**N:G238C**	1	0.08
Delhi				**N:D22G**	1	0.1				**N:R319H**	1	0.08
Delhi				**N:P6L**	1	0.1				**N:Q163E**	1	0.08
Delhi				**N:N8Y**	1	0.1				**N:A220V**	1	0.08
Delhi				**N:A90S**	1	0.1				**N:A173T**	1	0.08
Delhi				**N:A397V**	1	0.1						
Delhi				**N:P383S**	1	0.1						
Delhi				**N:D144G**	1	0.1						
Delhi				**N:S201N**	1	0.1						
Delhi				**N:P67S**	1	0.1						
Delhi				**N:P199L**	1	0.1						
Delhi				**N:P207A**	1	0.1						
Delhi				**N:A208T**	1	0.1						
Delhi				**N:R209G**	1	0.1						
Delhi				**N:T366I**	1	0.1						

Delhi				N:P13T	1	0.1						
Delhi				N:D144N	1	0.1						
Delhi				N:P344S	1	0.1						
Delhi				N:I351V	1	0.1						
Delhi				N:R40C	1	0.1						
Delhi				N:T16M	1	0.1						
Delhi				N:D103G	1	0.1						
Delhi				N:A35V	1	0.1						
Delhi				N:G179S	1	0.1						
Delhi				N:P365S	1	0.1						
Delhi				N:E290Q	1	0.1						
Delhi				N:L167F	1	0.1						
Delhi				N:Q229K	1	0.1						
Delhi				N:G212C	1	0.1						
Delhi				N:T247I	1	0.1						
Delhi				N:E323K	1	0.1						
Delhi				N:G204L	1	0.1						
Delhi				N:N29S	1	0.1						
Delhi				N:A359T	1	0.1						
Delhi				N:L331F	1	0.1						
Delhi				N:A220V	1	0.1						
Delhi				N:A182S	1	0.1						
Delhi				N:N354H	1	0.1						
Delhi				N:G19R	1	0.1						
Delhi				N:R189L	1	0.1						
**State/Gene**	**ORF3a**											
**Delhi**	**Pre-Wave 1**			**Wave 1**			**Pre-Wave 2**			**Wave 2**		
**Delhi**	**Mutation**	**Count**	**percent**	**Mutation**	**Count**	**percent**	**Mutation**	**Count**	**percent**	**Mutation**	**Count**	**percent**
**Delhi**	**ORF3a:Q57H**	57	59.38	**ORF3a:Q57H**	607	41.07	**ORF3a:Q57H**	145	31.25	**ORF3a:S26L**	804	62.42
**Delhi**	**ORF3a:S92L**	4	4.17	**ORF3a:T223I**	329	22.26	**ORF3a:S26L**	63	13.58	**ORF3a:G18V**	44	3.42
**Delhi**	**ORF3a:L275F**	3	3.12	**ORF3a:V55G**	43	2.91	**ORF3a:T223I**	47	10.13	**ORF3a:Q57H**	31	2.41
**Delhi**	**ORF3a:V55G**	3	3.12	**ORF3a:L106F**	42	2.84	**ORF3a:G18V**	17	3.66	**ORF3a:Q57R**	28	2.17
**Delhi**	**ORF3a:P240L**	3	3.12	**ORF3a:V77I**	35	2.37	**ORF3a:S171L**	13	2.8	**ORF3a:S171L**	21	1.63
**Delhi**	**ORF3a:G224C**	3	3.12	**ORF3a:G174V**	28	1.89	**ORF3a:V77I**	12	2.59	**ORF3a:D27Y**	16	1.24
**Delhi**	**ORF3a:S171L**	2	2.08	**ORF3a:S171L**	24	1.62	**ORF3a:S92L**	12	2.59	**ORF3a:A54S**	14	1.09
**Delhi**	**ORF3a:M260K**	2	2.08	**ORF3a:D155Y**	22	1.49	**ORF3a:E261***	11	2.37	**ORF3a:S92L**	14	1.09
**Delhi**	**ORF3a:V13L**	2	2.08	**ORF3a:E261***	16	1.08	**ORF3a:W131C**	9	1.94	**ORF3a:D238Y**	13	1.01
**Delhi**	**ORF3a:G251V**	2	2.08	**ORF3a:G172C**	15	1.01	**ORF3a:A54S**	9	1.94	**ORF3a:Q185H**	10	0.78
**Delhi**	**ORF3a:C81F**	1	1.04	**ORF3a:G224C**	12	0.81	**ORF3a:T89I**	7	1.51	**ORF3a:A23V**	10	0.78
**Delhi**	**ORF3a:A110S**	1	1.04	**ORF3a:W131C**	11	0.74	**ORF3a:G174V**	7	1.51	**ORF3a:R6D**	9	0.7
**Delhi**	**ORF3a:D155H**	1	1.04	**ORF3a:H182Y**	11	0.74	**ORF3a:L15F**	6	1.29	**ORF3a:I7L**	9	0.7
**Delhi**	**ORF3a:G254***	1	1.04	**ORF3a:Q185H**	11	0.74	**ORF3a:T175I**	5	1.08	**ORF3a:T9R**	9	0.7
**Delhi**	**ORF3a:N152S**	1	1.04	**ORF3a:L275F**	10	0.68	**ORF3a:D155Y**	5	1.08	**ORF3a:G11F**	9	0.7
**Delhi**	**ORF3a:A33S**	1	1.04	**ORF3a:E194G**	8	0.54	**ORF3a:V55G**	5	1.08	**ORF3a:K21G**	9	0.7
**Delhi**	**ORF3a:D173G**	1	1.04	**ORF3a:G100C**	7	0.47	**ORF3a:W131S**	5	1.08	**ORF3a:D22T**	9	0.7
**Delhi**	**ORF3a:T12I**	1	1.04	**ORF3a:A99S**	6	0.41	**ORF3a:L106F**	4	0.86	**ORF3a:P25G**	9	0.7
**Delhi**	**ORF3a:T151I**	1	1.04	**ORF3a:V13L**	6	0.41	**ORF3a:Q185H**	4	0.86	**ORF3a:F28Y**	9	0.7
**Delhi**	**ORF3a:S195F**	1	1.04	**ORF3a:T151I**	6	0.41	**ORF3a:G172V**	3	0.65	**ORF3a:R30N**	9	0.7
**Delhi**	**ORF3a:Q185H**	1	1.04	**ORF3a:W45L**	5	0.34	**ORF3a:G172C**	3	0.65	**ORF3a:A31D**	9	0.7
**Delhi**	**ORF3a:I167V**	1	1.04	**ORF3a:K16N**	5	0.34	**ORF3a:G18C**	3	0.65	**ORF3a:A33D**	9	0.7
**Delhi**	**ORF3a:T170A**	1	1.04	**ORF3a:D173Y**	5	0.34	**ORF3a:T151I**	2	0.43	**ORF3a:V29C**	8	0.62
**Delhi**	**ORF3a:G196V**	1	1.04	**ORF3a:G254V**	5	0.34	**ORF3a:G224V**	2	0.43	**ORF3a:W131C**	7	0.54
**Delhi**	**ORF3a:T176I**	1	1.04	**ORF3a:L94F**	5	0.34	**ORF3a:T24I**	2	0.43	**ORF3a:G100D**	7	0.54
**Delhi**				**ORF3a:D27Y**	4	0.27	**ORF3a:W45L**	2	0.43	**ORF3a:L15F**	7	0.54
**Delhi**				**ORF3a:W131R**	4	0.27	**ORF3a:A54V**	2	0.43	**ORF3a:V202L**	5	0.39
**Delhi**				**ORF3a:V88L**	4	0.27	**ORF3a:P240L**	2	0.43	**ORF3a:D27R**	5	0.39
**Delhi**				**ORF3a:L108F**	4	0.27	**ORF3a:G224C**	2	0.43	**ORF3a:E239D**	4	0.31
**Delhi**				**ORF3a:P240L**	4	0.27	**ORF3a:H78Y**	2	0.43	**ORF3a:D155Y**	4	0.31
**Delhi**				**ORF3a:N234I**	4	0.27	**ORF3a:L73F**	2	0.43	**ORF3a:D27L**	4	0.31
**Delhi**				**ORF3a:L140F**	4	0.27	**ORF3a:K67N**	2	0.43	**ORF3a:V225F**	4	0.31
**Delhi**				**ORF3a:M125I**	4	0.27	**ORF3a:Y184H**	2	0.43	**ORF3a:E261***	4	0.31
**Delhi**				**ORF3a:T175I**	3	0.2	**ORF3a:S216T**	2	0.43	**ORF3a:A99V**	4	0.31
**Delhi**				**ORF3a:L15F**	3	0.2	**ORF3a:L53F**	2	0.43	**ORF3a:V259L**	3	0.23
**Delhi**				**ORF3a:S60F**	3	0.2	**ORF3a:D22Y**	2	0.43	**ORF3a:A72S**	3	0.23
**Delhi**				**ORF3a:D155H**	3	0.2	**ORF3a:D27Y**	2	0.43	**ORF3a:P42L**	3	0.23
**Delhi**				**ORF3a:T12I**	3	0.2	**ORF3a:Q218K**	2	0.43	**ORF3a:V13L**	3	0.23
**Delhi**				**ORF3a:L53F**	3	0.2	**ORF3a:P240S**	2	0.43	**ORF3a:L53F**	2	0.16
**Delhi**				**ORF3a:G18S**	3	0.2	**ORF3a:S74F**	1	0.22	**ORF3a:A110S**	2	0.16
**Delhi**				**ORF3a:P104S**	3	0.2	**ORF3a:L41F**	1	0.22	**ORF3a:G49V**	2	0.16
**Delhi**				**ORF3a:K16R**	3	0.2	**ORF3a:L275F**	1	0.22	**ORF3a:P240S**	2	0.16
**Delhi**				**ORF3a:P262S**	3	0.2	**ORF3a:W193Q**	1	0.22	**ORF3a:I37T**	2	0.16

Delhi				ORF3a:V55C	3	0.2	ORF3a:P104S	1	0.22	ORF3a:G100C	2	0.16
Delhi				ORF3a:L41I	2	0.14	ORF3a:L219S	1	0.22	ORF3a:I7F	2	0.16
Delhi				ORF3a:L219S	2	0.14	ORF3a:T229I	1	0.22	ORF3a:H93Y	2	0.16
Delhi				ORF3a:A59S	2	0.14	ORF3a:L94F	1	0.22	ORF3a:T223I	2	0.16
Delhi				ORF3a:G224V	2	0.14	ORF3a:V13L	1	0.22	ORF3a:G254*	2	0.16
Delhi				ORF3a:D173N	2	0.14	ORF3a:P267S	1	0.22	ORF3a:V77F	2	0.16
Delhi				ORF3a:T34M	2	0.14	ORF3a:G11A	1	0.22	ORF3a:V55A	2	0.16
Delhi				ORF3a:G174C	2	0.14	ORF3a:N144Y	1	0.22	ORF3a:L52F	2	0.16
Delhi				ORF3a:A110V	2	0.14	ORF3a:G100C	1	0.22	ORF3a:H78Y	2	0.16
Delhi				ORF3a:P240S	2	0.14	ORF3a:R30H	1	0.22	ORF3a:A98V	2	0.16
Delhi				ORF3a:V202L	2	0.14	ORF3a:L94P	1	0.22	ORF3a:W69C	2	0.16
Delhi				ORF3a:G224R	2	0.14	ORF3a:S60F	1	0.22	ORF3a:V112F	2	0.16
Delhi				ORF3a:K67N	2	0.14	ORF3a:G49C	1	0.22	ORF3a:T151I	2	0.16
Delhi				ORF3a:L86F	2	0.14	ORF3a:K21N	1	0.22	ORF3a:T175I	2	0.16
Delhi				ORF3a:R134L	2	0.14	ORF3a:D173N	1	0.22	ORF3a:A23S	2	0.16
Delhi				ORF3a:H93Y	2	0.14	ORF3a:L52F	1	0.22	ORF3a:W128L	1	0.08
Delhi				ORF3a:T229I	2	0.14	ORF3a:H182Y	1	0.22	ORF3a:V80I	1	0.08
Delhi				ORF3a:L41F	2	0.14	ORF3a:M260I	1	0.22	ORF3a:I62V	1	0.08
Delhi				ORF3a:P25L	2	0.14	ORF3a:R126S	1	0.22	ORF3a:L94I	1	0.08
Delhi				ORF3a:G172V	2	0.14	ORF3a:T12I	1	0.22	ORF3a:R30L	1	0.08
Delhi				ORF3a:V77F	2	0.14	ORF3a:S162T	1	0.22	ORF3a:M1I	1	0.08
Delhi				ORF3a:G49V	2	0.14	ORF3a:W131L	1	0.22	ORF3a:A31P	1	0.08
Delhi				ORF3a:D27H	2	0.14	ORF3a:G254*	1	0.22	ORF3a:C130Y	1	0.08
Delhi				ORF3a:V168I	2	0.14	ORF3a:D27H	1	0.22	ORF3a:T221K	1	0.08
Delhi				ORF3a:K75R	1	0.07	ORF3a:Q213L	1	0.22	ORF3a:G172C	1	0.08
Delhi				ORF3a:I20V	1	0.07	ORF3a:P42L	1	0.22	ORF3a:L95F	1	0.08
Delhi				ORF3a:E194D	1	0.07	ORF3a:S252P	1	0.22	ORF3a:P25L	1	0.08
Delhi				ORF3a:V163L	1	0.07	ORF3a:V90L	1	0.22	ORF3a:I35V	1	0.08
Delhi				ORF3a:L140I	1	0.07	ORF3a:P258S	1	0.22	ORF3a:F146Y	1	0.08
Delhi				ORF3a:N144Y	1	0.07	ORF3a:T176I	1	0.22	ORF3a:Y113H	1	0.08
Delhi				ORF3a:F230L	1	0.07	ORF3a:M260K	1	0.22	ORF3a:P240L	1	0.08
Delhi				ORF3a:E181K	1	0.07				ORF3a:L108I	1	0.08
Delhi				ORF3a:S195F	1	0.07				ORF3a:W149C	1	0.08
Delhi				ORF3a:V225F	1	0.07				ORF3a:G254V	1	0.08
Delhi				ORF3a:G11V	1	0.07				ORF3a:S216P	1	0.08
Delhi				ORF3a:E241*	1	0.07				ORF3a:L106F	1	0.08
Delhi				ORF3a:S166L	1	0.07				ORF3a:G224C	1	0.08
Delhi				ORF3a:Q218K	1	0.07				ORF3a:L275F	1	0.08
Delhi				ORF3a:L275S	1	0.07				ORF3a:G224S	1	0.08
Delhi				ORF3a:T32I	1	0.07				ORF3a:V29F	1	0.08
Delhi				ORF3a:I186V	1	0.07				ORF3a:L96P	1	0.08
Delhi				ORF3a:R68T	1	0.07				ORF3a:A51S	1	0.08
Delhi				ORF3a:F105L	1	0.07				ORF3a:G18C	1	0.08
Delhi				ORF3a:A33S	1	0.07				ORF3a:P159S	1	0.08
Delhi				ORF3a:E194K	1	0.07				ORF3a:L65F	1	0.08
Delhi				ORF3a:R126M	1	0.07				ORF3a:A103P	1	0.08
Delhi				ORF3a:P159S	1	0.07				ORF3a:T89I	1	0.08
Delhi				ORF3a:I236S	1	0.07				ORF3a:T248I	1	0.08
Delhi				ORF3a:I179T	1	0.07				ORF3a:L115S	1	0.08
Delhi				ORF3a:G11E	1	0.07				ORF3a:G172V	1	0.08
Delhi				ORF3a:P104R	1	0.07				ORF3a:T9I	1	0.08
Delhi				ORF3a:D210A	1	0.07				ORF3a:D22Y	1	0.08
Delhi				ORF3a:I37T	1	0.07				ORF3a:W131L	1	0.08
Delhi				ORF3a:Y113H	1	0.07				ORF3a:E241Q	1	0.08
Delhi				ORF3a:P25H	1	0.07				ORF3a:T12I	1	0.08
Delhi				ORF3a:V273L	1	0.07				ORF3a:L94F	1	0.08
Delhi				ORF3a:V259A	1	0.07				ORF3a:T9A	1	0.08
Delhi				ORF3a:G174D	1	0.07				ORF3a:L94V	1	0.08
Delhi				ORF3a:Q213K	1	0.07				ORF3a:V55F	1	0.08
Delhi				ORF3a:V259L	1	0.07				ORF3a:Q213K	1	0.08
Delhi				ORF3a:W128L	1	0.07				ORF3a:L41F	1	0.08
Delhi				ORF3a:I63T	1	0.07				ORF3a:L65H	1	0.08
Delhi				ORF3a:L85F	1	0.07				ORF3a:K67N	1	0.08
Delhi				ORF3a:I179F	1	0.07				ORF3a:T271I	1	0.08
Delhi				ORF3a:T176I	1	0.07				ORF3a:P104S	1	0.08
Delhi				ORF3a:A110S	1	0.07				ORF3a:L106P	1	0.08
Delhi				ORF3a:V80I	1	0.07				ORF3a:L127F	1	0.08
Delhi				ORF3a:L46F	1	0.07				ORF3a:V48F	1	0.08
Delhi				ORF3a:E239D	1	0.07				ORF3a:T268M	1	0.08
Delhi				ORF3a:K66N	1	0.07				ORF3a:S209L	1	0.08
Delhi				ORF3a:V55F	1	0.07				ORF3a:D210N	1	0.08
Delhi				ORF3a:A39T	1	0.07				ORF3a:N257D	1	0.08
Delhi				ORF3a:A99V	1	0.07				ORF3a:L3F	1	0.08

Delhi				ORF3a:G254*	1	0.07				ORF3a:A59T	1	0.08
Delhi				ORF3a:A54S	1	0.07				ORF3a:P262S	1	0.08
Delhi				ORF3a:T89I	1	0.07				ORF3a:T175K	1	0.08
Delhi				ORF3a:I47V	1	0.07				ORF3a:S74F	1	0.08
Delhi				ORF3a:D173G	1	0.07				ORF3a:H182N	1	0.08
Delhi				ORF3a:P36L	1	0.07				ORF3a:L129F	1	0.08
Delhi				ORF3a:K75N	1	0.07				ORF3a:D155H	1	0.08
Delhi				ORF3a:R134C	1	0.07				ORF3a:K198R	1	0.08
Delhi				ORF3a:R134H	1	0.07				ORF3a:L108F	1	0.08
Delhi				ORF3a:T9K	1	0.07				ORF3a:G49C	1	0.08
Delhi				ORF3a:P42L	1	0.07				ORF3a:T176I	1	0.08
Delhi				ORF3a:D210Y	1	0.07						
Delhi				ORF3a:R68I	1	0.07						
Delhi				ORF3a:I186F	1	0.07						
Delhi				ORF3a:R30C	1	0.07						
Delhi				ORF3a:T269M	1	0.07						
Delhi				ORF3a:S165P	1	0.07						
Delhi				ORF3a:V97I	1	0.07						
Delhi				ORF3a:R6D	1	0.07						
Delhi				ORF3a:I7L	1	0.07						
Delhi				ORF3a:T9R	1	0.07						
Delhi				ORF3a:G11F	1	0.07						
Delhi				ORF3a:K21G	1	0.07						
Delhi				ORF3a:D22T	1	0.07						
Delhi				ORF3a:A23V	1	0.07						
Delhi				ORF3a:S26K	1	0.07						
Delhi				ORF3a:D27Q	1	0.07						
Delhi				ORF3a:F28G	1	0.07						
Delhi				ORF3a:V29L	1	0.07						
Delhi				ORF3a:W45R	1	0.07						
Delhi				ORF3a:L52I	1	0.07						
Delhi				ORF3a:V55A	1	0.07						
Delhi				ORF3a:L129F	1	0.07						
Delhi				ORF3a:S180F	1	0.07						
Delhi				ORF3a:S165F	1	0.07						
Delhi				ORF3a:P262L	1	0.07						
Delhi				ORF3a:T170A	1	0.07						
Delhi				ORF3a:K21N	1	0.07						
Delhi				ORF3a:T12A	1	0.07						
Delhi				ORF3a:T268M	1	0.07						
Delhi				ORF3a:H78Y	1	0.07						
Delhi				ORF3a:D142H	1	0.07						
Delhi				ORF3a:D27A	1	0.07						
Delhi				ORF3a:F114V	1	0.07						
**State/Gene**	**ORF6**											
**Delhi**	**Pre-Wave 1**			**Wave 1**			**Pre-Wave 2**			**Wave 2**		
**Delhi**	**Mutation**	**Count**	**percent**	**Mutation**	**Count**	**percent**	**Mutation**	**Count**	**percent**	**Mutation**	**Count**	**percent**
Delhi	ORF6:E59*	1	50	ORF6:W27L	12	27.27	ORF6:F2M	31	38.75	ORF6:I33T	65	46.76
Delhi	ORF6:H3L	1	50	ORF6:E13D	6	13.64	ORF6:I33T	21	26.25	ORF6:F2M	53	38.13
Delhi				ORF6:T21I	4	9.09	ORF6:F2S	18	22.5	ORF6:R20K	5	3.6
Delhi				ORF6:K42N	4	9.09	ORF6:W27L	3	3.75	ORF6:E54*	5	3.6
Delhi				ORF6:Y49H	2	4.55	ORF6:Y49H	2	2.5	ORF6:H3Y	2	1.44
Delhi				ORF6:K42M	2	4.55	ORF6:Q8H	2	2.5	ORF6:E55*	2	1.44
Delhi				ORF6:F2S	2	4.55	ORF6:E13D	2	2.5	ORF6:F2S	2	1.44
Delhi				ORF6:V24F	2	4.55	ORF6:K42M	1	1.25	ORF6:T10N	2	1.44
Delhi				ORF6:I26V	1	2.27				ORF6:S41F	1	0.72
Delhi				ORF6:S50F	1	2.27				ORF6:V9F	1	0.72
Delhi				ORF6:D61E	1	2.27				ORF6:K42M	1	0.72
Delhi				ORF6:Q8*	1	2.27						
Delhi				ORF6:F2Y	1	2.27						
Delhi				ORF6:K38R	1	2.27						
Delhi				ORF6:I14T	1	2.27						
Delhi				ORF6:P57R	1	2.27						
Delhi				ORF6:K48N	1	2.27						
Delhi				ORF6:D6Y	1	2.27						
**State/Gene**	**ORF7a**											
**Delhi**	**Pre-Wave 1**			**Wave 1**			**Pre-Wave 2**			**Wave 2**		
**Delhi**	**Mutation**	**Count**	**percent**	**Mutation**	**Count**	**percent**	**Mutation**	**Count**	**percent**	**Mutation**	**Count**	**percent**
Delhi	ORF7a:R80S	1	16.67	ORF7a:E95Q	10	16.67	ORF7a:V82A	59	53.64	ORF7a:V82A	675	40.76
Delhi	ORF7a:P48L	1	16.67	ORF7a:E22D	4	6.67	ORF7a:K119N	7	6.36	ORF7a:T120I	537	32.43
Delhi	ORF7a:N43D	1	16.67	ORF7a:I100T	4	6.67	ORF7a:E22D	4	3.64	ORF7a:L116F	356	21.5

**Delhi**	**ORF7a:P48T**	1	16.67	**ORF7a:L5F**	3	5	**ORF7a:T120I**	4	3.64	**ORF7a:L9P**	14	0.85
**Delhi**	**ORF7a:L112F**	1	16.67	**ORF7a:T28R**	2	3.33	**ORF7a:G38E**	3	2.73	**ORF7a:N43Y**	11	0.66
**Delhi**	**ORF7a:H19Y**	1	16.67	**ORF7a:L116F**	2	3.33	**ORF7a:L116F**	3	2.73	**ORF7a:H73Y**	9	0.54
**Delhi**				**ORF7a:V93F**	2	3.33	**ORF7a:T39I**	2	1.82	**ORF7a:K119N**	6	0.36
**Delhi**				**ORF7a:P99L**	2	3.33	**ORF7a:F46L**	2	1.82	**ORF7a:E22D**	4	0.24
**Delhi**				**ORF7a:T14I**	2	3.33	**ORF7a:H73Y**	2	1.82	**ORF7a:T39I**	4	0.24
**Delhi**				**ORF7a:S81L**	2	3.33	**ORF7a:L9P**	2	1.82	**ORF7a:Q62***	4	0.24
**Delhi**				**ORF7a:L102F**	2	3.33	**ORF7a:E95Q**	2	1.82	**ORF7a:L5F**	3	0.18
**Delhi**				**ORF7a:I4T**	2	3.33	**ORF7a:Q62***	1	0.91	**ORF7a:P34S**	3	0.18
**Delhi**				**ORF7a:E95***	1	1.67	**ORF7a:T14I**	1	0.91	**ORF7a:L12P**	3	0.18
**Delhi**				**ORF7a:Q76H**	1	1.67	**ORF7a:L96F**	1	0.91	**ORF7a:A64V**	2	0.12
**Delhi**				**ORF7a:V29L**	1	1.67	**ORF7a:G38R**	1	0.91	**ORF7a:V93F**	2	0.12
**Delhi**				**ORF7a:P34L**	1	1.67	**ORF7a:G38***	1	0.91	**ORF7a:S81P**	2	0.12
**Delhi**				**ORF7a:Y75***	1	1.67	**ORF7a:P34L**	1	0.91	**ORF7a:S98F**	1	0.06
**Delhi**				**ORF7a:R80K**	1	1.67	**ORF7a:V24F**	1	0.91	**ORF7a:P68S**	1	0.06
**Delhi**				**ORF7a:H47N**	1	1.67	**ORF7a:A105V**	1	0.91	**ORF7a:T14I**	1	0.06
**Delhi**				**ORF7a:I10T**	1	1.67	**ORF7a:S44T**	1	0.91	**ORF7a:A8V**	1	0.06
**Delhi**				**ORF7a:A66S**	1	1.67	**ORF7a:F6L**	1	0.91	**ORF7a:G38V**	1	0.06
**Delhi**				**ORF7a:A105S**	1	1.67	**ORF7a:I4T**	1	0.91	**ORF7a:T61I**	1	0.06
**Delhi**				**ORF7a:F63H**	1	1.67	**ORF7a:S83L**	1	0.91	**ORF7a:A50V**	1	0.06
**Delhi**				**ORF7a:P84L**	1	1.67	**ORF7a:I100F**	1	0.91	**ORF7a:V24A**	1	0.06
**Delhi**				**ORF7a:A8T**	1	1.67	**ORF7a:F114L**	1	0.91	**ORF7a:P45L**	1	0.06
**Delhi**				**ORF7a:T39I**	1	1.67	**ORF7a:P45S**	1	0.91	**ORF7a:S37F**	1	0.06
**Delhi**				**ORF7a:A55S**	1	1.67	**ORF7a:F63L**	1	0.91	**ORF7a:T28I**	1	0.06
**Delhi**				**ORF7a:P99S**	1	1.67	**ORF7a:R78C**	1	0.91	**ORF7a:L56Q**	1	0.06
**Delhi**				**ORF7a:R118I**	1	1.67	**ORF7a:T28I**	1	0.91	**ORF7a:Q94***	1	0.06
**Delhi**				**ORF7a:S81P**	1	1.67	**ORF7a:Q94H**	1	0.91	**ORF7a:A66V**	1	0.06
**Delhi**				**ORF7a:T28I**	1	1.67	**ORF7a:N43Y**	1	0.91	**ORF7a:V24F**	1	0.06
**Delhi**				**ORF7a:A13T**	1	1.67				**ORF7a:R25T**	1	0.06
**Delhi**				**ORF7a:E121Q**	1	1.67				**ORF7a:A8T**	1	0.06
**Delhi**				**ORF7a:R25I**	1	1.67				**ORF7a:Q76H**	1	0.06
**Delhi**				**ORF7a:V24F**	1	1.67				**ORF7a:L77S**	1	0.06
**Delhi**										**ORF7a:R80I**	1	0.06
**Delhi**										**ORF7a:V71I**	1	0.06
**State/Gene**	**ORF7b**											
**Delhi**	**Pre-Wave 1**			**Wave 1**			**Pre-Wave 2**			**Wave 2**		
**Delhi**	**Mutation**	**Count**	**percent**	**Mutation**	**Count**	**percent**	**Mutation**	**Count**	**percent**	**Mutation**	**Count**	**percent**
**Delhi**	**ORF7b:T40I**	1	33.33	**ORF7b:M24I**	8	13.56	**ORF7b:C41F**	14	35	**ORF7b:T40I**	111	81.02
**Delhi**	**ORF7b:Y10F**	2	66.67	**ORF7b:E39***	6	10.17	**ORF7b:A15S**	5	12.5	**ORF7b:E39***	10	7.3
**Delhi**				**ORF7b:T40I**	6	10.17	**ORF7b:E39***	5	12.5	**ORF7b:A15S**	4	2.92
**Delhi**				**ORF7b:L4F**	5	8.47	**ORF7b:E33***	3	7.5	**ORF7b:A15V**	2	1.46
**Delhi**				**ORF7b:A15V**	3	5.08	**ORF7b:L4F**	2	5	**ORF7b:F28L**	1	0.73
**Delhi**				**ORF7b:E33***	3	5.08	**ORF7b:T40I**	2	5	**ORF7b:W29G**	1	0.73
**Delhi**				**ORF7b:A15S**	3	5.08	**ORF7b:L25F**	2	5	**ORF7b:A43T**	1	0.73
**Delhi**				**ORF7b:A43S**	2	3.39	**ORF7b:S31L**	1	2.5	**ORF7b:F19S**	1	0.73
**Delhi**				**ORF7b:E39Q**	2	3.39	**ORF7b:S5L**	1	2.5	**ORF7b:L11F**	1	0.73
**Delhi**				**ORF7b:H42Y**	2	3.39	**ORF7b:H42Y**	1	2.5	**ORF7b:I26T**	1	0.73
**Delhi**				**ORF7b:I2N**	2	3.39	**ORF7b:F13L**	1	2.5	**ORF7b:C41F**	1	0.73
**Delhi**				**ORF7b:H37Y**	1	1.69	**ORF7b:D8E**	1	2.5	**ORF7b:H37L**	1	0.73
**Delhi**				**ORF7b:F13L**	1	1.69	**ORF7b:I27V**	1	2.5	**ORF7b:I27V**	1	0.73
**Delhi**				**ORF7b:D8Y**	1	1.69	**ORF7b:A43S**	1	2.5	**ORF7b:F16L**	1	0.73
**Delhi**				**ORF7b:F9L**	1	1.69						
**Delhi**				**ORF7b:S31P**	1	1.69						
**Delhi**				**ORF7b:C41F**	1	1.69						
**Delhi**				**ORF7b:L34P**	1	1.69						
**Delhi**				**ORF7b:C12G**	1	1.69						
**Delhi**				**ORF7b:L25F**	1	1.69						
**Delhi**				**ORF7b:W29R**	1	1.69						
**Delhi**				**ORF7b:M1I**	1	1.69						
**Delhi**				**ORF7b:W29L**	1	1.69						
**Delhi**				**ORF7b:F9E**	1	1.69						
**Delhi**				**ORF7b:Y10C**	1	1.69						
**Delhi**				**ORF7b:E39G**	1	1.69						
**Delhi**				**ORF7b:S31L**	1	1.69						
**Delhi**				**ORF7b:E3***	1	1.69						
**State/Gene**	**ORF8**											
**Delhi**	**Pre-Wave 1**			**Wave 1**			**Pre-Wave 2**			**Wave 2**		
**Delhi**	**Mutation**	**Count**	**percent**	**Mutation**	**Count**	**percent**	**Mutation**	**Count**	**percent**	**Mutation**	**Count**	**percent**
**Delhi**	**ORF8:C25F**	1	25	**ORF8:I9T**	20	9.05	**ORF8:Q27***	197	24.91	**ORF8:Q27***	313	22.71
**Delhi**	**ORF8:V117F**	1	25	**ORF8:S97I**	15	6.79	**ORF8:R52I**	196	24.78	**ORF8:Y73C**	312	22.64
**Delhi**	**ORF8:L84S**	2	50	**ORF8:V62L**	13	5.88	**ORF8:Y73C**	190	24.02	**ORF8:R52I**	311	22.57

**Delhi**				**ORF8:Q18***	11	4.98	**ORF8:K68***	100	12.64	**ORF8:K68***	205	14.88
**Delhi**				**ORF8:Q27***	10	4.52	**ORF8:S69L**	13	1.64	**ORF8:F16S**	57	4.14
**Delhi**				**ORF8:R52I**	9	4.07	**ORF8:F16S**	13	1.64	**ORF8:S69L**	47	3.41
**Delhi**				**ORF8:S67F**	9	4.07	**ORF8:S97I**	11	1.39	**ORF8:I121L**	11	0.8
**Delhi**				**ORF8:Y73C**	7	3.17	**ORF8:A65V**	9	1.14	**ORF8:T26I**	10	0.73
**Delhi**				**ORF8:E106Q**	6	2.71	**ORF8:H112Y**	6	0.76	**ORF8:D75Y**	10	0.73
**Delhi**				**ORF8:K68***	6	2.71	**ORF8:D35Y**	5	0.63	**ORF8:G8V**	9	0.65
**Delhi**				**ORF8:L95F**	5	2.26	**ORF8:L95F**	4	0.51	**ORF8:E106***	5	0.36
**Delhi**				**ORF8:E19***	5	2.26	**ORF8:D75Y**	4	0.51	**ORF8:K68E**	4	0.29
**Delhi**				**ORF8:A65V**	5	2.26	**ORF8:V62L**	4	0.51	**ORF8:W45C**	4	0.29
**Delhi**				**ORF8:I39T**	4	1.81	**ORF8:P36S**	2	0.25	**ORF8:V62L**	4	0.29
**Delhi**				**ORF8:C20F**	4	1.81	**ORF8:Q18***	2	0.25	**ORF8:S97I**	4	0.29
**Delhi**				**ORF8:G77C**	4	1.81	**ORF8:V32L**	2	0.25	**ORF8:L84S**	4	0.29
**Delhi**				**ORF8:H17Y**	3	1.36	**ORF8:S24L**	2	0.25	**ORF8:E92K**	4	0.29
**Delhi**				**ORF8:P36L**	3	1.36	**ORF8:S67F**	2	0.25	**ORF8:V32L**	3	0.22
**Delhi**				**ORF8:V81F**	3	1.36	**ORF8:T26I**	2	0.25	**ORF8:F120L**	3	0.22
**Delhi**				**ORF8:Q29L**	3	1.36	**ORF8:K68E**	2	0.25	**ORF8:T87I**	3	0.22
**Delhi**				**ORF8:F3S**	3	1.36	**ORF8:I74V**	2	0.25	**ORF8:E19***	3	0.22
**Delhi**				**ORF8:K68R**	2	0.9	**ORF8:P36L**	2	0.25	**ORF8:A65S**	3	0.22
**Delhi**				**ORF8:E64***	2	0.9	**ORF8:I9T**	1	0.13	**ORF8:A65V**	3	0.22
**Delhi**				**ORF8:K53I**	2	0.9	**ORF8:G50***	1	0.13	**ORF8:C83F**	3	0.22
**Delhi**				**ORF8:I9V**	2	0.9	**ORF8:S103L**	1	0.13	**ORF8:T11I**	2	0.15
**Delhi**				**ORF8:V81I**	2	0.9	**ORF8:E19***	1	0.13	**ORF8:D35Y**	2	0.15
**Delhi**				**ORF8:G8V**	2	0.9	**ORF8:I88V**	1	0.13	**ORF8:W45L**	2	0.15
**Delhi**				**ORF8:W45L**	2	0.9	**ORF8:W45C**	1	0.13	**ORF8:V100A**	2	0.15
**Delhi**				**ORF8:W45C**	2	0.9	**ORF8:I71T**	1	0.13	**ORF8:F6S**	2	0.15
**Delhi**				**ORF8:I10T**	2	0.9	**ORF8:I71L**	1	0.13	**ORF8:C102F**	2	0.15
**Delhi**				**ORF8:V32L**	2	0.9	**ORF8:T12I**	1	0.13	**ORF8:P36S**	2	0.15
**Delhi**				**ORF8:S24L**	2	0.9	**ORF8:Q29L**	1	0.13	**ORF8:E64G**	2	0.15
**Delhi**				**ORF8:K68E**	2	0.9	**ORF8:E106***	1	0.13	**ORF8:I10T**	2	0.15
**Delhi**				**ORF8:F120L**	2	0.9	**ORF8:I121L**	1	0.13	**ORF8:I58F**	2	0.15
**Delhi**				**ORF8:H28Y**	2	0.9	**ORF8:V81F**	1	0.13	**ORF8:S67F**	1	0.07
**Delhi**				**ORF8:F108S**	2	0.9	**ORF8:V99L**	1	0.13	**ORF8:S67A**	1	0.07
**Delhi**				**ORF8:R101H**	2	0.9	**ORF8:C25F**	1	0.13	**ORF8:E106Q**	1	0.07
**Delhi**				**ORF8:F108L**	2	0.9	**ORF8:T87I**	1	0.13	**ORF8:P36L**	1	0.07
**Delhi**				**ORF8:T80I**	1	0.45	**ORF8:Q91R**	1	0.13	**ORF8:C37W**	1	0.07
**Delhi**				**ORF8:T11A**	1	0.45	**ORF8:I10T**	1	0.13	**ORF8:I71L**	1	0.07
**Delhi**				**ORF8:G66D**	1	0.45	**ORF8:T11I**	1	0.13	**ORF8:P38L**	1	0.07
**Delhi**				**ORF8:I76V**	1	0.45	**ORF8:G8V**	1	0.13	**ORF8:F120V**	1	0.07
**Delhi**				**ORF8:P30L**	1	0.45	**ORF8:L98R**	1	0.13	**ORF8:V99L**	1	0.07
**Delhi**				**ORF8:A51V**	1	0.45				**ORF8:I121N**	1	0.07
**Delhi**				**ORF8:T26I**	1	0.45				**ORF8:A14M**	1	0.07
**Delhi**				**ORF8:D34V**	1	0.45				**ORF8:A15K**	1	0.07
**Delhi**				**ORF8:G50***	1	0.45				**ORF8:H17L**	1	0.07
**Delhi**				**ORF8:E106***	1	0.45				**ORF8:Q18V**	1	0.07
**Delhi**				**ORF8:D35Y**	1	0.45				**ORF8:E19L**	1	0.07
**Delhi**				**ORF8:S103L**	1	0.45				**ORF8:C20Y**	1	0.07
**Delhi**				**ORF8:T11I**	1	0.45				**ORF8:V62F**	1	0.07
**Delhi**				**ORF8:L84S**	1	0.45				**ORF8:Y42C**	1	0.07
**Delhi**				**ORF8:E92K**	1	0.45				**ORF8:A51V**	1	0.07
**Delhi**				**ORF8:C102F**	1	0.45				**ORF8:G8R**	1	0.07
**Delhi**				**ORF8:E110Q**	1	0.45				**ORF8:K68I**	1	0.07
**Delhi**				**ORF8:D34Y**	1	0.45				**ORF8:S69P**	1	0.07
**Delhi**				**ORF8:A55V**	1	0.45				**ORF8:R115L**	1	0.07
**Delhi**				**ORF8:R115L**	1	0.45						
**Delhi**				**ORF8:E59***	1	0.45						
**Delhi**				**ORF8:P93S**	1	0.45						
**Delhi**				**ORF8:K2R**	1	0.45						
**Delhi**				**ORF8:E110***	1	0.45						
**Delhi**				**ORF8:S69L**	1	0.45						
**Delhi**				**ORF8:T11K**	1	0.45						
**Delhi**				**ORF8:A15V**	1	0.45						
**Delhi**				**ORF8:I39V**	1	0.45						
**Delhi**				**ORF8:I88V**	1	0.45						
**Delhi**				**ORF8:D34G**	1	0.45						
**Delhi**				**ORF8:C25F**	1	0.45						
**Delhi**				**ORF8:V114I**	1	0.45						
**Delhi**				**ORF8:G8D**	1	0.45						
**Delhi**				**ORF8:I9F**	1	0.45						
**Delhi**				**ORF8:T11***	1	0.45						
**Delhi**				**ORF8:G8K**	1	0.45						
**Delhi**				**ORF8:T87I**	1	0.45						
**Delhi**				**ORF8:D119G**	1	0.45						

Delhi				ORF8:T26S	1	0.45						
State/Gene	ORF9b											
Delhi	Pre-Wave 1			Wave 1			Pre-Wave 2			Wave 2		
Delhi	Mutation	Count	percent	Mutation	Count	percent	Mutation	Count	percent	Mutation	Count	percent
Delhi	ORF9b:P10S	166	98.81	ORF9b:R13H	9	10.34	ORF9b:H9D	32	44.44	ORF9b:T60A	592	82.22
Delhi	ORF9b:M1T	1	0.6	ORF9b:R13L	8	9.2	ORF9b:G38D	11	15.28	ORF9b:H9D	51	7.08
Delhi	ORF9b:D2N	1	0.6	ORF9b:A57V	7	8.05	ORF9b:R32L	6	8.33	ORF9b:G38D	22	3.06
Delhi				ORF9b:S6I	6	6.9	ORF9b:T60A	4	5.56	ORF9b:M26K	8	1.11
Delhi				ORF9b:R32P	5	5.75	ORF9b:A29T	3	4.17	ORF9b:P3L	7	0.97
Delhi				ORF9b:D66E	4	4.6	ORF9b:P3L	2	2.78	ORF9b:R32P	5	0.69
Delhi				ORF9b:I45V	4	4.6	ORF9b:D16H	2	2.78	ORF9b:E7Q	4	0.56
Delhi				ORF9b:Q18H	4	4.6	ORF9b:R32C	2	2.78	ORF9b:R25G	4	0.56
Delhi				ORF9b:R47H	3	3.45	ORF9b:H9Q	1	1.39	ORF9b:V15L	4	0.56
Delhi				ORF9b:K67E	3	3.45	ORF9b:M56I	1	1.39	ORF9b:R13L	3	0.42
Delhi				ORF9b:R25I	2	2.3	ORF9b:M26T	1	1.39	ORF9b:A75V	3	0.42
Delhi				ORF9b:V37A	2	2.3	ORF9b:A75V	1	1.39	ORF9b:H9Q	2	0.28
Delhi				ORF9b:A68V	2	2.3	ORF9b:P10L	1	1.39	ORF9b:L21Q	1	0.14
Delhi				ORF9b:P17S	2	2.3	ORF9b:P10S	1	1.39	ORF9b:S50L	1	0.14
Delhi				ORF9b:R32L	2	2.3	ORF9b:M8I	1	1.39	ORF9b:Q20R	1	0.14
Delhi				ORF9b:H9Y	2	2.3	ORF9b:A29V	1	1.39	ORF9b:K59N	1	0.14
Delhi				ORF9b:S6N	2	2.3	ORF9b:S6N	1	1.39	ORF9b:D33G	1	0.14
Delhi				ORF9b:T83I	1	1.15	ORF9b:P3S	1	1.39	ORF9b:R32L	1	0.14
Delhi				ORF9b:P10S	1	1.15				ORF9b:R32H	1	0.14
Delhi				ORF9b:K97R	1	1.15				ORF9b:S6I	1	0.14
Delhi				ORF9b:L64F	1	1.15				ORF9b:P17L	1	0.14
Delhi				ORF9b:N35S	1	1.15				ORF9b:M56I	1	0.14
Delhi				ORF9b:T72I	1	1.15				ORF9b:G38C	1	0.14
Delhi				ORF9b:I19V	1	1.15				ORF9b:I5L	1	0.14
Delhi				ORF9b:S63F	1	1.15				ORF9b:K4N	1	0.14
Delhi				ORF9b:P3S	1	1.15				ORF9b:G31E	1	0.14
Delhi				ORF9b:K4N	1	1.15				ORF9b:E86D	1	0.14
Delhi				ORF9b:E86D	1	1.15						
Delhi				ORF9b:M8T	1	1.15						
Delhi				ORF9b:H9Q	1	1.15						
Delhi				ORF9b:R13C	1	1.15						
Delhi				ORF9b:A75V	1	1.15						
Delhi				ORF9b:P3L	1	1.15						
Delhi				ORF9b:S6T	1	1.15						
Delhi				ORF9b:R32C	1	1.15						
Delhi				ORF9b:G38V	1	1.15						
Delhi				ORF9b:M26V	1	1.15						
Delhi	Amino acid deletions											
Delhi	Pre-Wave 1			Wave 1			Pre-Wave 2			Wave 2		
Delhi	ORF1a:M85-	2	2.17	S:Y144-	14	3.62	S:H69-	209	5.9	ORF1a:S3675-	254	3.24
Delhi	ORF1a:G82-	1	1.09	ORF1a:S3675-	10	2.58	S:V70-	209	5.9	ORF1a:G3676-	255	3.25
Delhi	ORF1a:H83-	1	1.09	ORF1a:G3676-	10	2.58	S:Y144-	208	5.87	ORF1a:F3677-	255	3.25
Delhi	ORF1a:V84-	1	1.09	ORF1a:F3677-	10	2.58	ORF1a:S3675-	168	4.74	S:H69-	416	5.31
Delhi	ORF1a:V86-	1	1.09	ORF8:E19-	8	2.07	ORF1a:G3676-	168	4.74	S:V70-	415	5.3
Delhi	S:V382-	1	1.09	S:G142-	8	2.07	ORF1a:F3677-	168	4.74	S:Y144-	401	5.12
Delhi	S:S383-	1	1.09	S:F140-	6	1.55	N:M1-	32	0.9	ORF8:D119-	525	6.7
Delhi	S:P384-	1	1.09	S:H69-	6	1.55	ORF6:M1-	31	0.87	ORF8:F120-	525	6.7
Delhi	S:T385-	1	1.09	S:V70-	6	1.55	S:L241-	12	0.34	ORF1a:K141-	19	0.24
Delhi	S:K386-	1	1.09	S:Y145-	5	1.29	S:L242-	12	0.34	ORF1a:S142-	19	0.24
Delhi	S:L387-	1	1.09	ORF1a:K141-	4	1.03	S:A243-	12	0.34	ORF1a:F143-	19	0.24
Delhi	S:N388-	1	1.09	ORF1a:S142-	4	1.03	S:G142-	6	0.17	S:E156-	381	4.86
Delhi	S:H69-	1	1.09	ORF1a:F143-	4	1.03	ORF1a:S1666-	5	0.14	S:F157-	381	4.86
Delhi	S:V70-	1	1.09	S:L141-	4	1.03	ORF1a:I1667-	5	0.14	S:Q52-	2	0.03
Delhi	ORF1a:A776-	1	1.09	S:V143-	4	1.03	ORF1a:K1668-	4	0.11	S:D53-	2	0.03
Delhi	ORF1a:P777-	1	1.09	ORF1a:L2028-	3	0.78	ORF1b:Y1944-	4	0.11	ORF1a:V84-	8	0.1
Delhi	ORF1a:L778-	1	1.09	S:H146-	3	0.78	ORF1b:V1945-	4	0.11	ORF1a:M85-	9	0.11
Delhi	ORF1a:V779-	1	1.09	ORF7b:F13-	3	0.78	ORF1b:N1946-	4	0.11	ORF1a:F1190-	4	0.05
Delhi	ORF1a:G780-	1	1.09	S:D138-	2	0.52	ORF8:D119-	4	0.11	ORF7a:A55-	4	0.05
Delhi	ORF1a:T781-	1	1.09	S:P139-	2	0.52	ORF8:F120-	4	0.11	ORF7a:L56-	4	0.05
Delhi	ORF1a:P782-	1	1.09	ORF1a:K2029-	2	0.52	ORF7a:*122-	3	0.08	ORF7a:T57-	4	0.05
Delhi	ORF1a:V783-	1	1.09	ORF1a:S2030-	2	0.52	S:V143-	3	0.08	ORF7a:C58-	4	0.05
Delhi	ORF1a:C784-	1	1.09	ORF1a:E2031-	2	0.52	ORF1a:W1669-	3	0.08	ORF7a:F59-	4	0.05
Delhi	ORF1a:I785-	1	1.09	ORF1a:D2032-	2	0.52	ORF1a:A1670-	3	0.08	ORF7a:S60-	4	0.05
Delhi	ORF1a:N786-	1	1.09	ORF1a:A2033-	2	0.52	ORF1a:D1671-	3	0.08	ORF7a:T61-	4	0.05
Delhi	ORF1a:G787-	1	1.09	ORF1a:Q2034-	2	0.52	ORF1a:N1672-	3	0.08	ORF7a:Q62-	4	0.05
Delhi	ORF1a:L788-	1	1.09	ORF1a:G2035-	2	0.52	ORF1a:N1673-	3	0.08	ORF7a:F63-	4	0.05

Delhi	ORF1a:M789-	1	1.09	ORF1a:M2036-	2	0.52	ORF1a:C1674-	3	0.08	ORF7a:A64-	4	0.05
Delhi	ORF1a:L790-	1	1.09	ORF1a:D2037-	2	0.52	ORF1a:Y1675-	3	0.08	ORF7a:F65-	4	0.05
Delhi	ORF1a:L791-	1	1.09	ORF1a:N2038-	2	0.52	ORF1a:L1676-	3	0.08	ORF7a:A66-	4	0.05
Delhi	ORF1a:E792-	1	1.09	ORF1a:L2039-	2	0.52	ORF1a:A1677-	3	0.08	ORF7a:C67-	4	0.05
Delhi	ORF1a:I793-	1	1.09	ORF1a:A2040-	2	0.52	ORF1a:T1678-	3	0.08	ORF7a:P68-	4	0.05
Delhi	ORF1a:K794-	1	1.09	ORF1a:C2041-	2	0.52	ORF1a:A1679-	3	0.08	ORF7a:D69-	4	0.05
Delhi	ORF1a:D795-	1	1.09	ORF1a:E2042-	2	0.52	ORF1a:L1680-	3	0.08	ORF7a:G70-	4	0.05
Delhi	ORF1a:T796-	1	1.09	ORF1a:D2043-	2	0.52	ORF1a:Y1700-	3	0.08	ORF7a:V71-	4	0.05
Delhi	ORF1a:E797-	1	1.09	ORF8:G66-	2	0.52	ORF1a:R1701-	3	0.08	ORF7a:K72-	4	0.05
Delhi	ORF1a:K798-	1	1.09	ORF8:S67-	2	0.52	ORF1a:A1702-	3	0.08	ORF7a:H73-	4	0.05
Delhi	ORF1a:Y799-	1	1.09	S:M1-	2	0.52	ORF1a:R1703-	3	0.08	ORF7a:V74-	4	0.05
Delhi	ORF1a:C800-	1	1.09	S:F2-	2	0.52	ORF1a:A1704-	3	0.08	ORF7a:Y75-	4	0.05
Delhi	ORF1a:A801-	1	1.09	S:V3-	2	0.52	ORF1a:G1705-	3	0.08	ORF7a:Q76-	4	0.05
Delhi	ORF1a:L802-	1	1.09	S:F4-	2	0.52	ORF1a:E1706-	3	0.08	ORF7a:L77-	4	0.05
Delhi	ORF1a:A803-	1	1.09	S:L5-	2	0.52	ORF1a:C1744-	3	0.08	ORF1a:S2083-	1	0.01
Delhi	ORF1a:P804-	1	1.09	S:V6-	2	0.52	ORF1a:K1745-	3	0.08	ORF8:G66-	4	0.05
Delhi	ORF1a:N805-	1	1.09	S:L7-	2	0.52	ORF1a:R1746-	3	0.08	ORF8:S67-	4	0.05
Delhi	ORF1a:M806-	1	1.09	S:L8-	2	0.52	ORF1a:V1747-	3	0.08	ORF1a:G82-	2	0.03
Delhi	ORF1a:M807-	1	1.09	S:P9-	2	0.52	ORF1a:L1748-	3	0.08	ORF1a:H83-	2	0.03
Delhi	ORF1a:V808-	1	1.09	S:L10-	2	0.52	ORF1a:N1749-	3	0.08	S:P57-	1	0.01
Delhi	ORF1a:T809-	1	1.09	S:V11-	2	0.52	ORF1a:V1750-	3	0.08	S:F58-	1	0.01
Delhi	ORF1a:N810-	1	1.09	S:S12-	2	0.52	ORF1a:V1751-	3	0.08	N:M1-	52	0.66
Delhi	ORF1a:N811-	1	1.09	S:S13-	2	0.52	ORF1a:C1752-	3	0.08	ORF6:M1-	53	0.68
Delhi	ORF1a:T812-	1	1.09	ORF1b:S1978-	2	0.52	ORF1a:K1753-	3	0.08	S:L241-	19	0.24
Delhi	ORF1a:F813-	1	1.09	ORF1b:H1979-	2	0.52	ORF1a:T1754-	3	0.08	S:L242-	19	0.24
Delhi	ORF1a:T814-	1	1.09	ORF1b:G1980-	2	0.52	ORF1a:C1755-	3	0.08	S:A243-	19	0.24
Delhi	ORF1a:L815-	1	1.09	ORF1b:K1981-	2	0.52	ORF1a:G1756-	3	0.08	ORF3a:V255-	4	0.05
Delhi	ORF1a:K816-	1	1.09	ORF1b:Q1982-	2	0.52	ORF1a:Q1757-	3	0.08	ORF1a:E1024-	1	0.01
Delhi	ORF1a:G817-	1	1.09	ORF1b:V1983-	2	0.52	ORF1a:Q1758-	3	0.08	ORF1a:V1025-	1	0.01
Delhi	ORF1a:G818-	1	1.09	ORF1b:V1984-	2	0.52	ORF1a:L1762-	3	0.08	ORF1a:N1026-	1	0.01
Delhi	ORF1a:A819-	1	1.09	ORF1b:S1985-	2	0.52	ORF1a:K1763-	3	0.08	S:I68-	1	0.01
Delhi	ORF1a:P820-	1	1.09	ORF1b:D1986-	2	0.52	ORF1a:G1764-	3	0.08	N:A208-	2	0.03
Delhi	ORF1a:T821-	1	1.09	ORF1b:I1987-	2	0.52	ORF1a:V1765-	3	0.08	ORF8:M1-	4	0.05
Delhi	ORF1a:K822-	1	1.09	ORF1b:D1988-	2	0.52	ORF1a:E1766-	3	0.08	ORF8:K2-	4	0.05
Delhi	ORF1a:V823-	1	1.09	ORF1b:Y1989-	2	0.52	ORF1a:S1775-	3	0.08	ORF3a:M1-	10	0.13
Delhi	ORF1a:T824-	1	1.09	ORF1a:K2085-	1	0.26	ORF1a:Y1776-	3	0.08	ORF3a:D2-	9	0.11
Delhi	ORF1a:F825-	1	1.09	ORF1a:I2086-	1	0.26	ORF1a:E1777-	3	0.08	ORF3a:L3-	9	0.11
Delhi	ORF1a:G826-	1	1.09	ORF3a:V255-	1	0.26	ORF1a:Q1778-	3	0.08	ORF3a:F4-	9	0.11
Delhi	ORF1a:D827-	1	1.09	ORF1a:S3195-	1	0.26	ORF1a:F1779-	3	0.08	ORF3a:V13-	9	0.11
Delhi	ORF1a:D828-	1	1.09	ORF1a:D3196-	1	0.26	ORF1a:K1780-	3	0.08	ORF3a:T14-	9	0.11
Delhi	ORF1a:T829-	1	1.09	ORF1a:V3197-	1	0.26	ORF1a:K1781-	3	0.08	ORF3a:L15-	9	0.11
Delhi	ORF1a:V830-	1	1.09	ORF1a:L3198-	1	0.26	ORF1a:G1782-	3	0.08	ORF3a:K16-	9	0.11
Delhi	ORF1a:I831-	1	1.09	ORF1a:L3199-	1	0.26	ORF1a:V1783-	3	0.08	ORF3a:Q17-	9	0.11
Delhi	ORF1a:E832-	1	1.09	ORF1a:P3200-	1	0.26	ORF1a:Q1784-	3	0.08	ORF3a:G18-	9	0.11
Delhi	ORF1a:V833-	1	1.09	ORF1a:L3201-	1	0.26	ORF1a:I1785-	3	0.08	ORF3a:E19-	9	0.11
Delhi	ORF1a:Q834-	1	1.09	ORF1a:T3202-	1	0.26	ORF1a:P1786-	3	0.08	ORF3a:I35-	9	0.11
Delhi	ORF1a:G835-	1	1.09	ORF1a:Q3203-	1	0.26	ORF1a:C1787-	3	0.08	ORF3a:P36-	9	0.11
Delhi	ORF1a:Y836-	1	1.09	ORF1a:Y3204-	1	0.26	ORF1a:T1788-	3	0.08	ORF3a:I37-	9	0.11
Delhi	ORF1a:K837-	1	1.09	ORF1a:N3205-	1	0.26	ORF1a:C1789-	3	0.08	ORF3a:Q38-	9	0.11
Delhi	ORF1a:S838-	1	1.09	ORF1a:R3206-	1	0.26	ORF1a:G1790-	3	0.08	ORF3a:A39-	9	0.11
Delhi	ORF1a:V839-	1	1.09	ORF1a:Y3207-	1	0.26	ORF1a:K1791-	3	0.08	ORF6:*62-	1	0.01
Delhi	ORF1a:N840-	1	1.09	ORF1a:L3208-	1	0.26	ORF1a:Q1792-	3	0.08	ORF1b:Y1944-	6	0.08
Delhi	ORF1a:I841-	1	1.09	ORF1a:A3209-	1	0.26	ORF1a:A1793-	3	0.08	ORF1b:V1945-	5	0.06
Delhi	ORF1a:T842-	1	1.09	ORF1a:L3210-	1	0.26	ORF1a:T1794-	3	0.08	ORF1b:N1946-	5	0.06
Delhi	ORF1a:F843-	1	1.09	ORF1a:Y3211-	1	0.26	ORF1a:K1795-	3	0.08	ORF1b:K1947-	5	0.06
Delhi	ORF1a:E844-	1	1.09	ORF1a:N3212-	1	0.26	ORF1a:Q1799-	3	0.08	ORF1b:H1948-	5	0.06
Delhi	ORF1a:L845-	1	1.09	ORF1a:K3213-	1	0.26	ORF1a:Q1800-	3	0.08	ORF1b:A1949-	5	0.06
Delhi	ORF1a:D846-	1	1.09	ORF1a:Y3214-	1	0.26	ORF1a:E1801-	3	0.08	ORF1b:F1950-	5	0.06
Delhi	ORF1a:E847-	1	1.09	ORF1a:K3215-	1	0.26	ORF1a:S1802-	3	0.08	ORF1b:H1951-	5	0.06
Delhi	ORF1a:R848-	1	1.09	ORF1a:Y3216-	1	0.26	ORF1a:P1803-	3	0.08	ORF1b:T1952-	5	0.06
Delhi	ORF1a:I849-	1	1.09	ORF1a:F3217-	1	0.26	ORF1a:F1804-	3	0.08	ORF1b:P1953-	5	0.06
Delhi	ORF1a:D850-	1	1.09	ORF1a:S3218-	1	0.26	ORF1a:V1805-	3	0.08	ORF1b:A1954-	5	0.06
Delhi	ORF1a:K851-	1	1.09	ORF1a:G3219-	1	0.26	ORF1a:M1806-	3	0.08	ORF1b:F1955-	5	0.06
Delhi	ORF1a:V852-	1	1.09	ORF1a:A3220-	1	0.26	ORF1a:M1807-	3	0.08	ORF1b:D1956-	5	0.06
Delhi				ORF1a:M3221-	1	0.26	ORF1a:S1808-	3	0.08	ORF1b:K1957-	5	0.06
Delhi				ORF1a:D3222-	1	0.26	ORF1a:A1809-	3	0.08	ORF1b:S1958-	5	0.06
Delhi				ORF1a:T3223-	1	0.26	ORF1a:P1810-	3	0.08	ORF1b:A1959-	5	0.06
Delhi				ORF1a:T3224-	1	0.26	ORF1a:P1811-	3	0.08	ORF1b:F1960-	5	0.06
Delhi				ORF1a:S3225-	1	0.26	ORF1a:A1812-	3	0.08	ORF1b:V1961-	5	0.06
Delhi				ORF1a:Y3226-	1	0.26	ORF1a:Q1813-	3	0.08	ORF1b:N1962-	5	0.06
Delhi				ORF1a:R3227-	1	0.26	ORF1a:Y1814-	3	0.08	ORF1b:L1963-	5	0.06
Delhi				ORF1a:E3228-	1	0.26	ORF1a:E1815-	3	0.08	ORF1b:K1964-	5	0.06
Delhi				ORF1a:A3229-	1	0.26	ORF1a:L1816-	3	0.08	ORF1b:Q1965-	5	0.06

Delhi				ORF1a:A3230-	1	0.26	ORF1a:K1817-	3	0.08	ORF1b:L1966-	5	0.06
Delhi				ORF1a:C3231-	1	0.26	ORF1a:H1818-	3	0.08	ORF1b:P1967-	5	0.06
Delhi				ORF1a:C3232-	1	0.26	ORF1a:G1819-	3	0.08	ORF1b:F1968-	5	0.06
Delhi				ORF1a:H3233-	1	0.26	ORF1a:E1843-	3	0.08	ORF1b:F1969-	5	0.06
Delhi				ORF1a:L3234-	1	0.26	ORF1a:T1844-	3	0.08	ORF1b:Y1970-	5	0.06
Delhi				ORF1a:A3235-	1	0.26	ORF1a:L1845-	3	0.08	ORF1b:Y1971-	5	0.06
Delhi				ORF1a:K3236-	1	0.26	ORF1a:Y1846-	3	0.08	ORF1b:S1972-	5	0.06
Delhi				ORF1a:A3237-	1	0.26	ORF1a:C1847-	3	0.08	ORF1b:D1973-	5	0.06
Delhi				ORF1a:L3238-	1	0.26	ORF1a:I1848-	3	0.08	ORF1b:S1974-	5	0.06
Delhi				ORF1a:N3239-	1	0.26	ORF1a:D1849-	3	0.08	ORF1b:P1975-	5	0.06
Delhi				ORF1a:D3240-	1	0.26	ORF1a:G1850-	3	0.08	ORF1b:C1976-	5	0.06
Delhi				ORF1a:F3241-	1	0.26	ORF1a:A1851-	3	0.08	ORF1b:E1977-	5	0.06
Delhi				ORF1a:S3242-	1	0.26	ORF1a:L1852-	3	0.08	ORF1b:S1978-	5	0.06
Delhi				ORF1a:N3243-	1	0.26	ORF1a:L1853-	3	0.08	ORF1b:H1979-	5	0.06
Delhi				ORF1a:S3244-	1	0.26	ORF1a:T1854-	3	0.08	ORF1b:G1980-	5	0.06
Delhi				ORF1a:G3245-	1	0.26	ORF1a:K1855-	3	0.08	ORF1b:K1981-	5	0.06
Delhi				ORF1a:V84-	1	0.26	ORF1a:S1856-	3	0.08	ORF1b:Q1982-	5	0.06
Delhi				ORF1a:M85-	1	0.26	ORF1a:S1857-	3	0.08	ORF1b:V1983-	5	0.06
Delhi				ORF1a:V86-	1	0.26	ORF1a:E1858-	3	0.08	ORF1b:V1984-	5	0.06
Delhi				ORF7a:Q62-	1	0.26	ORF1a:Y1859-	3	0.08	ORF1b:S1985-	5	0.06
Delhi				ORF3a:V256-	1	0.26	ORF1a:K1860-	3	0.08	ORF1b:D1986-	5	0.06
Delhi				ORF3a:N257-	1	0.26	ORF1a:G1861-	3	0.08	ORF1b:I1987-	5	0.06
Delhi				ORF3a:P258-	1	0.26	ORF1a:P1862-	3	0.08	ORF1b:D1988-	5	0.06
Delhi				ORF3a:V259-	1	0.26	ORF1a:I1863-	3	0.08	ORF1b:Y1989-	5	0.06
Delhi				ORF3a:M260-	1	0.26	ORF1a:T1864-	3	0.08	ORF1b:V1990-	5	0.06
Delhi				ORF3a:E261-	1	0.26	ORF1a:D1865-	3	0.08	ORF1b:P1991-	5	0.06
Delhi				ORF3a:P262-	1	0.26	ORF1a:T1875-	3	0.08	ORF1b:L1992-	5	0.06
Delhi				ORF8:M1-	1	0.26	ORF1a:T1876-	3	0.08	ORF1b:K1993-	5	0.06
Delhi				ORF8:K2-	1	0.26	ORF1a:I1877-	3	0.08	ORF1b:S1994-	5	0.06
Delhi				ORF1a:N2081-	1	0.26	ORF1a:K1878-	3	0.08	ORF1b:A1995-	5	0.06
Delhi				S:L24-	1	0.26	ORF1a:P1879-	3	0.08	ORF1b:T1996-	5	0.06
Delhi				ORF1a:K58-	1	0.26	ORF1a:V1880-	3	0.08	ORF1b:C1997-	5	0.06
Delhi				ORF1a:G59-	1	0.26	ORF1a:I1892-	3	0.08	ORF1b:I1998-	5	0.06
Delhi				ORF1a:V60-	1	0.26	ORF1a:D1893-	3	0.08	ORF1b:T1999-	5	0.06
Delhi				E:L39-	1	0.26	ORF1a:P1894-	3	0.08	ORF1b:R2000-	5	0.06
Delhi				E:C40-	1	0.26	ORF1a:K1895-	3	0.08	ORF1b:C2001-	5	0.06
Delhi				E:A41-	1	0.26	ORF1a:L1896-	3	0.08	ORF1b:N2002-	5	0.06
Delhi				E:Y42-	1	0.26	ORF1a:D1897-	3	0.08	ORF1b:L2003-	5	0.06
Delhi				E:C43-	1	0.26	ORF1a:N1898-	3	0.08	ORF1b:G2004-	5	0.06
Delhi				E:C44-	1	0.26	ORF1a:D1903-	3	0.08	ORF1b:G2005-	5	0.06
Delhi				E:N45-	1	0.26	ORF1a:N1904-	3	0.08	ORF1b:A2006-	5	0.06
Delhi				E:I46-	1	0.26	ORF1a:S1905-	3	0.08	ORF1b:V2007-	5	0.06
Delhi				ORF7b:L14-	1	0.26	ORF1a:Y1906-	3	0.08	ORF1b:C2008-	5	0.06
Delhi				ORF7b:A15-	1	0.26	ORF1a:F1907-	3	0.08	ORF1b:R2009-	5	0.06
Delhi				S:I210-	1	0.26	ORF1a:T1908-	3	0.08	ORF1b:H2010-	5	0.06
Delhi				ORF1a:K1895-	1	0.26	ORF1a:E1909-	3	0.08	ORF1b:H2011-	5	0.06
Delhi				ORF1a:L1896-	1	0.26	ORF1a:Q1910-	3	0.08	ORF1b:A2012-	5	0.06
Delhi				ORF1a:D1897-	1	0.26	ORF1a:P1911-	3	0.08	ORF1b:N2013-	5	0.06
Delhi				ORF1a:N1898-	1	0.26	ORF1a:I1912-	3	0.08	ORF1b:E2014-	5	0.06
Delhi				ORF1a:Y1899-	1	0.26	ORF1a:D1913-	3	0.08	ORF1b:Y2015-	5	0.06
Delhi				ORF1a:Y1900-	1	0.26	ORF1a:I1935-	3	0.08	ORF1b:R2016-	5	0.06
Delhi				ORF1a:K1901-	1	0.26	ORF1a:K1936-	3	0.08	ORF1b:L2017-	5	0.06
Delhi				ORF1a:K1902-	1	0.26	ORF1a:F1937-	3	0.08	ORF1b:Y2018-	5	0.06
Delhi				ORF1a:D1903-	1	0.26	ORF1a:A1938-	3	0.08	ORF1b:L2019-	5	0.06
Delhi				ORF1a:N1904-	1	0.26	ORF1a:D1939-	3	0.08	ORF1b:D2020-	5	0.06
Delhi				ORF1a:S1905-	1	0.26	ORF1a:D1940-	3	0.08	ORF1b:A2021-	5	0.06
Delhi				ORF1a:Y1906-	1	0.26	ORF1a:L1941-	3	0.08	ORF1b:Y2022-	5	0.06
Delhi				ORF1a:F1907-	1	0.26	ORF1a:N1942-	3	0.08	ORF1b:N2023-	5	0.06
Delhi				ORF1a:T1908-	1	0.26	ORF1a:Q1943-	3	0.08	ORF1b:M2024-	5	0.06
Delhi				ORF1a:E1909-	1	0.26	ORF1a:L1944-	3	0.08	ORF1b:M2025-	5	0.06
Delhi				ORF1a:Q1910-	1	0.26	ORF1a:T1945-	3	0.08	ORF1b:I2026-	5	0.06
Delhi				ORF1a:P1911-	1	0.26	ORF1a:G1946-	3	0.08	ORF1b:S2027-	5	0.06
Delhi				ORF1a:I1912-	1	0.26	ORF1a:Y1947-	3	0.08	ORF1a:S1666-	1	0.01
Delhi				ORF1a:D1913-	1	0.26	ORF1a:K1948-	3	0.08	ORF1a:I1667-	2	0.03
Delhi				ORF1a:L1914-	1	0.26	ORF1a:K1949-	3	0.08	ORF7a:M1-	1	0.01
Delhi				ORF1a:V1915-	1	0.26	ORF1a:P1950-	3	0.08	S:G142-	14	0.18
Delhi				ORF1a:P1916-	1	0.26	ORF1a:A1951-	3	0.08	ORF1a:N389-	1	0.01
Delhi				ORF1a:N1917-	1	0.26	ORF1a:S1952-	3	0.08	ORF8:F3-	1	0.01
Delhi				ORF1a:Q1918-	1	0.26	ORF1a:N1964-	3	0.08	ORF8:L4-	1	0.01
Delhi				ORF1a:P1919-	1	0.26	ORF1a:G1965-	3	0.08	ORF8:V5-	1	0.01
Delhi				ORF1a:Y1920-	1	0.26	ORF1a:D1966-	3	0.08	ORF8:F6-	1	0.01
Delhi				ORF1a:P1921-	1	0.26	ORF1a:V1967-	3	0.08	ORF8:L7-	1	0.01
Delhi				ORF1a:N1922-	1	0.26	ORF1a:V1968-	3	0.08	ORF8:G8-	1	0.01

Delhi				**ORF1a:A1923-**	1	0.26	**ORF1a:A1969-**	3	0.08	**ORF8:I9-**	1	0.01
Delhi				**ORF1a:S1924-**	1	0.26	**ORF1a:I1970-**	3	0.08	**ORF8:I10-**	1	0.01
Delhi				**ORF1a:F1925-**	1	0.26	**ORF1a:D1971-**	3	0.08	**ORF8:T11-**	1	0.01
Delhi				**ORF1a:D1926-**	1	0.26	**ORF1a:Y1972-**	3	0.08	**ORF8:T12-**	1	0.01
Delhi				**ORF3a:M1-**	1	0.26	**ORF1a:K1973-**	3	0.08	**ORF8:V13-**	1	0.01
Delhi				**ORF3a:D2-**	1	0.26	**ORF1a:H1974-**	3	0.08	**S:V143-**	1	0.01
Delhi				**ORF3a:L3-**	1	0.26	**ORF1a:Y1975-**	3	0.08	**S:F140-**	1	0.01
Delhi				**ORF3a:F4-**	1	0.26	**ORF1a:T1976-**	3	0.08	**ORF1a:T1269-**	2	0.03
Delhi				**ORF3a:V13-**	1	0.26	**ORF1a:P1977-**	3	0.08	**ORF1a:L1270-**	2	0.03
Delhi				**ORF3a:T14-**	1	0.26	**ORF1a:S1978-**	3	0.08	**ORF1a:V1271-**	2	0.03
Delhi				**ORF3a:L15-**	1	0.26	**ORF1a:F1979-**	3	0.08	**ORF1a:S1272-**	2	0.03
Delhi				**ORF3a:K16-**	1	0.26	**ORF1a:K1980-**	3	0.08	**ORF1a:D1273-**	2	0.03
Delhi				**ORF3a:Q17-**	1	0.26	**ORF1a:K1981-**	3	0.08	**ORF1a:I1274-**	2	0.03
Delhi				**ORF3a:G18-**	1	0.26	**ORF1a:G1982-**	3	0.08	**ORF1a:D1275-**	2	0.03
Delhi				**ORF3a:E19-**	1	0.26	**ORF1a:A1983-**	3	0.08	**ORF1a:I1276-**	2	0.03
Delhi				**ORF1a:N389-**	1	0.26	**ORF1a:K1984-**	3	0.08	**ORF1a:T1277-**	2	0.03
Delhi				**ORF1a:E2020-**	1	0.26	**ORF1a:L1985-**	3	0.08	**ORF1a:K1281-**	2	0.03
Delhi				**ORF1a:T2021-**	1	0.26	**ORF1a:N1995-**	3	0.08	**ORF1a:D1282-**	2	0.03
Delhi				**ORF1a:S2022-**	1	0.26	**ORF1a:N1996-**	3	0.08	**ORF1a:A1283-**	2	0.03
Delhi				**ORF1a:N2023-**	1	0.26	**ORF1a:A1997-**	3	0.08	**ORF1a:P1284-**	2	0.03
Delhi				**ORF1a:S2024-**	1	0.26	**ORF1a:T1998-**	3	0.08	**ORF1a:Y1285-**	2	0.03
Delhi				**ORF1a:F2025-**	1	0.26	**ORF1a:N1999-**	3	0.08	**ORF1a:I1286-**	2	0.03
Delhi				**ORF1a:D2026-**	1	0.26	**ORF1a:K2000-**	3	0.08	**ORF1a:V1287-**	2	0.03
Delhi				**ORF1a:V2027-**	1	0.26	**ORF1a:A2001-**	3	0.08	**ORF1a:G1288-**	2	0.03
Delhi				**ORF1b:A2655-**	1	0.26	**ORF1a:T2002-**	3	0.08	**ORF1a:D1289-**	2	0.03
Delhi				**ORF1b:V2656-**	1	0.26	**ORF1a:Y2003-**	3	0.08	**ORF1a:V1290-**	2	0.03
Delhi				**ORF1b:M2657-**	1	0.26	**ORF1a:K2004-**	3	0.08	**ORF1a:V1291-**	2	0.03
Delhi				**ORF1b:S2658-**	1	0.26	**ORF1a:P2005-**	3	0.08	**ORF1a:Q1292-**	2	0.03
Delhi				**ORF1b:L2659-**	1	0.26	**ORF1a:N2006-**	3	0.08	**ORF1a:G1294-**	2	0.03
Delhi				**ORF1b:K2660-**	1	0.26	**ORF1a:T2007-**	3	0.08	**ORF1a:V1295-**	2	0.03
Delhi				**ORF1b:E2661-**	1	0.26	**ORF1a:W2008-**	3	0.08	**ORF1a:L1296-**	2	0.03
Delhi				**ORF1b:G2662-**	1	0.26	**ORF1a:C2009-**	3	0.08	**ORF1a:T1297-**	2	0.03
Delhi				**ORF1b:Q2663-**	1	0.26	**ORF1a:I2010-**	3	0.08	**ORF1a:A1298-**	2	0.03
Delhi				**ORF1b:I2664-**	1	0.26	**ORF1a:R2011-**	3	0.08	**ORF1a:V1299-**	2	0.03
Delhi				**ORF1b:N2665-**	1	0.26	**ORF1a:C2012-**	3	0.08	**ORF1a:V1300-**	2	0.03
Delhi				**ORF1b:D2666-**	1	0.26	**ORF1a:L2013-**	3	0.08	**ORF1a:I1301-**	2	0.03
Delhi				**ORF1b:M2667-**	1	0.26	**ORF1a:W2014-**	3	0.08	**ORF1a:P1302-**	2	0.03
Delhi				**ORF1b:I2668-**	1	0.26	**ORF1a:S2024-**	3	0.08	**ORF1a:T1303-**	2	0.03
Delhi				**ORF1b:L2669-**	1	0.26	**ORF1a:F2025-**	3	0.08	**ORF1a:K1304-**	2	0.03
Delhi				**ORF1b:S2670-**	1	0.26	**ORF1a:D2026-**	3	0.08	**ORF1a:K1305-**	2	0.03
Delhi				**ORF1b:L2671-**	1	0.26	**ORF1a:V2027-**	3	0.08	**ORF1a:A1306-**	2	0.03
Delhi				**ORF1b:L2672-**	1	0.26	**ORF1a:L2028-**	3	0.08	**ORF1a:G1307-**	2	0.03
Delhi				**ORF1b:S2673-**	1	0.26	**ORF1a:K2029-**	3	0.08	**ORF1a:R1318-**	2	0.03
Delhi				**ORF1b:K2674-**	1	0.26	**ORF1a:S2030-**	3	0.08	**ORF1a:K1319-**	2	0.03
Delhi				**ORF1b:G2675-**	1	0.26	**ORF1a:E2031-**	3	0.08	**ORF1a:V1320-**	2	0.03
Delhi				**ORF1b:R2676-**	1	0.26	**ORF1a:D2032-**	3	0.08	**ORF1a:P1321-**	2	0.03
Delhi				**ORF1b:L2677-**	1	0.26	**ORF1a:A2033-**	3	0.08	**ORF1a:T1322-**	2	0.03
Delhi				**ORF1b:I2678-**	1	0.26	**ORF1a:Q2034-**	3	0.08	**ORF1a:D1323-**	2	0.03
Delhi				**ORF1b:I2679-**	1	0.26	**ORF1a:G2035-**	3	0.08	**ORF1a:N1324-**	2	0.03
Delhi				**ORF1b:R2680-**	1	0.26	**ORF1a:M2036-**	3	0.08	**ORF1a:Y1325-**	2	0.03
Delhi				**ORF1b:E2681-**	1	0.26	**ORF1a:D2037-**	3	0.08	**ORF1a:I1326-**	2	0.03
Delhi				**ORF1b:N2682-**	1	0.26	**ORF1a:N2038-**	3	0.08	**ORF1a:T1327-**	2	0.03
Delhi				**ORF1b:N2683-**	1	0.26	**ORF1a:L2039-**	3	0.08	**ORF1a:T1328-**	2	0.03
Delhi				**ORF1b:R2684-**	1	0.26	**ORF1a:A2040-**	3	0.08	**ORF1a:Y1329-**	2	0.03
Delhi				**ORF1b:V2685-**	1	0.26	**ORF1a:C2041-**	3	0.08	**ORF1a:L1334-**	2	0.03
Delhi				**ORF1b:V2686-**	1	0.26	**ORF1a:E2042-**	3	0.08	**ORF1a:N1335-**	2	0.03
Delhi				**ORF1b:I2687-**	1	0.26	**ORF1a:D2043-**	3	0.08	**ORF1a:G1336-**	2	0.03
Delhi				**ORF1b:S2688-**	1	0.26	**ORF1a:L2044-**	3	0.08	**ORF1a:Y1337-**	2	0.03
Delhi				**ORF1b:S2689-**	1	0.26	**ORF1a:K2045-**	3	0.08	**ORF1a:T1338-**	2	0.03
Delhi				**ORF1b:D2690-**	1	0.26	**ORF1a:P2046-**	3	0.08	**ORF1a:V1339-**	2	0.03
Delhi				**ORF1b:V2691-**	1	0.26	**ORF1a:V2047-**	3	0.08	**ORF1a:E1340-**	2	0.03
Delhi				**ORF1b:L2692-**	1	0.26	**ORF1a:P2055-**	3	0.08	**ORF1a:E1341-**	2	0.03
Delhi				**ORF1b:V2693-**	1	0.26	**ORF1a:T2056-**	3	0.08	**ORF1a:A1342-**	2	0.03
Delhi				**ORF1b:N2694-**	1	0.26	**ORF1a:I2057-**	3	0.08	**ORF1a:K1343-**	2	0.03
Delhi				**ORF1b:N2695-**	1	0.26	**ORF1a:Q2058-**	3	0.08	**ORF1a:T1344-**	2	0.03
Delhi				**ORF1b:*2696-**	1	0.26	**ORF1a:K2059-**	3	0.08	**ORF1a:V1345-**	2	0.03
Delhi				**S:Q14-**	1	0.26	**ORF1a:D2060-**	3	0.08	**ORF1a:E1363-**	2	0.03
Delhi				**S:C15-**	1	0.26	**ORF1a:V2061-**	3	0.08	**ORF1a:K1364-**	2	0.03
Delhi				**S:V16-**	1	0.26	**ORF1a:L2062-**	3	0.08	**ORF1a:Q1365-**	2	0.03
Delhi				**S:N17-**	1	0.26	**ORF1a:E2063-**	3	0.08	**ORF1a:E1366-**	2	0.03
Delhi				**S:L18-**	1	0.26	**ORF1a:C2064-**	3	0.08	**ORF1a:I1367-**	2	0.03
Delhi				**S:T19-**	1	0.26	**ORF1a:N2065-**	3	0.08	**ORF1a:L1368-**	2	0.03
Delhi				**S:T20-**	1	0.26	**ORF1a:V2066-**	3	0.08	**ORF1a:G1369-**	2	0.03

Delhi				S:R21-	1	0.26	ORF1a:K2067-	3	0.08	ORF1a:T1370-	2	0.03
Delhi				S:T22-	1	0.26	ORF1a:T2068-	3	0.08	ORF1a:V1371-	2	0.03
Delhi				S:Q23-	1	0.26	ORF1a:T2069-	3	0.08	ORF1a:R1386-	2	0.03
Delhi							ORF1a:E2070-	3	0.08	ORF1a:K1387-	2	0.03
Delhi							ORF1a:V2071-	3	0.08	ORF1a:L1388-	2	0.03
Delhi							ORF1a:V2072-	3	0.08	ORF1a:M1389-	2	0.03
Delhi							ORF1a:G2073-	3	0.08	ORF1a:P1390-	2	0.03
Delhi							ORF1a:D2074-	3	0.08	ORF1a:V1391-	2	0.03
Delhi							ORF1a:I2075-	3	0.08	ORF1a:V1399-	2	0.03
Delhi							ORF1a:I2076-	3	0.08	ORF1a:S1400-	2	0.03
Delhi							ORF1a:L2077-	3	0.08	ORF1a:T1401-	2	0.03
Delhi							ORF1a:K2078-	3	0.08	ORF1a:I1402-	2	0.03
Delhi							ORF1a:P2079-	3	0.08	ORF1a:Q1403-	2	0.03
Delhi							ORF1a:K2085-	3	0.08	ORF1a:R1404-	2	0.03
Delhi							ORF1a:I2086-	3	0.08	ORF1a:K1405-	2	0.03
Delhi							ORF1a:T2087-	3	0.08	ORF1a:Y1406-	2	0.03
Delhi							ORF1a:E2088-	3	0.08	ORF1a:K1407-	2	0.03
Delhi							ORF1a:E2089-	3	0.08	ORF1a:G1408-	2	0.03
Delhi							ORF1a:V2090-	3	0.08	ORF1a:I1409-	2	0.03
Delhi							ORF1a:G2091-	3	0.08	ORF1a:K1410-	2	0.03
Delhi							ORF1a:H2092-	3	0.08	ORF1a:I1411-	2	0.03
Delhi							ORF1a:T2093-	3	0.08	ORF1a:Q1412-	2	0.03
Delhi							ORF1a:D2094-	3	0.08	ORF1a:E1413-	2	0.03
Delhi							ORF1a:L2095-	3	0.08	ORF1a:G1414-	2	0.03
Delhi							ORF1a:M2096-	3	0.08	ORF1a:V1415-	2	0.03
Delhi							ORF1a:A2097-	3	0.08	ORF1a:V1416-	2	0.03
Delhi							ORF1a:A2098-	3	0.08	ORF1a:D1417-	2	0.03
Delhi							ORF1a:Y2099-	3	0.08	ORF1a:Y1418-	2	0.03
Delhi							ORF1a:V2100-	3	0.08	ORF1a:G1419-	2	0.03
Delhi							ORF1a:D2101-	3	0.08	ORF1a:A1420-	2	0.03
Delhi							ORF1a:N2102-	3	0.08	ORF1a:L1445-	2	0.03
Delhi							ORF1a:S2103-	3	0.08	ORF1a:V1446-	2	0.03
Delhi							ORF1a:S2104-	3	0.08	ORF1a:T1447-	2	0.03
Delhi							ORF1a:L2105-	3	0.08	ORF1a:M1448-	2	0.03
Delhi							ORF1a:T2106-	3	0.08	ORF1a:P1449-	2	0.03
Delhi							ORF1a:I2107-	3	0.08	ORF1a:L1450-	2	0.03
Delhi							ORF1a:K2108-	3	0.08	ORF1a:G1451-	2	0.03
Delhi							ORF1a:K2109-	3	0.08	ORF1a:Y1452-	2	0.03
Delhi							ORF1a:P2110-	3	0.08	ORF1a:V1453-	2	0.03
Delhi							ORF1a:R2115-	3	0.08	ORF1a:T1454-	2	0.03
Delhi							ORF1a:V2116-	3	0.08	ORF1a:H1455-	2	0.03
Delhi							ORF1a:L2117-	3	0.08	ORF1a:G1456-	2	0.03
Delhi							ORF1a:G2118-	3	0.08	ORF1a:L1457-	2	0.03
Delhi							ORF1a:L2119-	3	0.08	ORF1a:N1458-	2	0.03
Delhi							ORF1a:K2120-	3	0.08	ORF1a:L1459-	2	0.03
Delhi							ORF1a:T2121-	3	0.08	ORF1a:E1460-	2	0.03
Delhi							ORF1a:L2122-	3	0.08	ORF1a:E1461-	2	0.03
Delhi							ORF1a:A2123-	3	0.08	ORF1a:A1462-	2	0.03
Delhi							ORF1a:T2124-	3	0.08	ORF1a:A1463-	2	0.03
Delhi							ORF1a:H2125-	3	0.08	ORF1a:R1464-	2	0.03
Delhi							ORF1a:G2126-	3	0.08	ORF1a:Y1465-	2	0.03
Delhi							ORF1a:L2127-	3	0.08	ORF1a:M1466-	2	0.03
Delhi							ORF1a:A2128-	3	0.08	ORF1a:R1467-	2	0.03
Delhi							ORF1a:A2129-	3	0.08	ORF1a:S1492-	2	0.03
Delhi							ORF1a:V2130-	3	0.08	ORF1a:S1493-	2	0.03
Delhi							ORF1a:N2131-	3	0.08	ORF1a:S1494-	2	0.03
Delhi							ORF1a:S2132-	3	0.08	ORF1a:K1495-	2	0.03
Delhi							ORF1a:V2133-	3	0.08	ORF1a:T1496-	2	0.03
Delhi							ORF1a:P2134-	3	0.08	ORF1a:P1497-	2	0.03
Delhi							ORF1a:W2135-	3	0.08	ORF1a:E1498-	2	0.03
Delhi							ORF1a:D2136-	3	0.08	ORF1a:E1499-	2	0.03
Delhi							ORF1a:T2137-	3	0.08	ORF1a:H1500-	2	0.03
Delhi							ORF1a:I2138-	3	0.08	ORF1a:F1501-	2	0.03
Delhi							ORF1a:A2139-	3	0.08	ORF1a:I1502-	2	0.03
Delhi							ORF1a:A2142-	3	0.08	ORF1a:E1503-	2	0.03
Delhi							ORF1a:K2143-	3	0.08	ORF1a:T1504-	2	0.03
Delhi							ORF1a:P2144-	3	0.08	ORF1a:I1505-	2	0.03
Delhi							ORF1a:F2145-	3	0.08	ORF1a:S1506-	2	0.03
Delhi							ORF1a:L2146-	3	0.08	ORF1a:L1507-	2	0.03
Delhi							ORF1a:N2147-	3	0.08	ORF1a:A1508-	2	0.03
Delhi							ORF1a:K2148-	3	0.08	ORF1a:G1509-	2	0.03
Delhi							ORF1a:V2149-	3	0.08	ORF1a:S1510-	2	0.03
Delhi							ORF1a:V2150-	3	0.08	ORF1a:Y1511-	2	0.03

Delhi							ORF1a:S2151-	3	0.08	ORF1a:K1512-	2	0.03
Delhi							ORF1a:T2152-	3	0.08	ORF1a:D1513-	2	0.03
Delhi							ORF1a:T2153-	3	0.08	ORF1a:W1514-	2	0.03
Delhi							ORF1a:T2154-	3	0.08	ORF1a:E1526-	2	0.03
Delhi							ORF1a:N2155-	3	0.08	ORF1a:F1527-	2	0.03
Delhi							ORF1a:I2156-	3	0.08	ORF1a:L1528-	2	0.03
Delhi							ORF1a:V2157-	3	0.08	ORF1a:K1529-	2	0.03
Delhi							ORF1a:T2158-	3	0.08	ORF1a:R1530-	2	0.03
Delhi							ORF1a:R2159-	3	0.08	ORF1a:G1531-	2	0.03
Delhi							ORF1a:C2160-	3	0.08	ORF1a:D1532-	2	0.03
Delhi							ORF1a:L2161-	3	0.08	ORF1a:K1533-	2	0.03
Delhi							ORF1a:N2162-	3	0.08	ORF1a:S1534-	2	0.03
Delhi							ORF1a:R2163-	3	0.08	ORF1a:V1535-	2	0.03
Delhi							ORF1a:V2164-	3	0.08	ORF1a:Y1536-	2	0.03
Delhi							ORF1a:C2165-	3	0.08	ORF1a:Y1537-	2	0.03
Delhi							ORF1a:T2174-	3	0.08	ORF1a:T1538-	2	0.03
Delhi							ORF1a:L2175-	3	0.08	ORF1a:S1539-	2	0.03
Delhi							ORF1a:L2176-	3	0.08	ORF1a:N1540-	2	0.03
Delhi							ORF1a:L2177-	3	0.08	ORF1a:P1541-	2	0.03
Delhi							ORF1a:Q2178-	3	0.08	ORF1a:T1542-	2	0.03
Delhi							ORF1a:L2179-	3	0.08	ORF1a:T1543-	2	0.03
Delhi							ORF1a:C2180-	3	0.08	ORF1a:F1544-	2	0.03
Delhi							ORF1a:T2181-	3	0.08	ORF1a:H1545-	2	0.03
Delhi							ORF1a:F2182-	3	0.08	ORF1a:G1548-	2	0.03
Delhi							ORF1a:T2183-	3	0.08	ORF1a:E1549-	2	0.03
Delhi							ORF1a:R2184-	3	0.08	ORF1a:V1550-	2	0.03
Delhi							ORF1a:S2185-	3	0.08	ORF1a:I1551-	2	0.03
Delhi							ORF1a:A2355-	3	0.08	ORF1a:T1552-	2	0.03
Delhi							ORF1a:V2356-	3	0.08	ORF1a:F1553-	2	0.03
Delhi							ORF1a:H2357-	3	0.08	ORF1a:D1554-	2	0.03
Delhi							ORF1a:F2358-	3	0.08	ORF1a:N1555-	2	0.03
Delhi							ORF1a:I2359-	3	0.08	ORF1a:L1556-	2	0.03
Delhi							ORF1a:S2360-	3	0.08	ORF1a:K1557-	2	0.03
Delhi							ORF1a:N2361-	3	0.08	ORF1a:T1558-	2	0.03
Delhi							ORF1a:S2362-	3	0.08	ORF1a:L1559-	2	0.03
Delhi							ORF1a:W2363-	3	0.08	ORF1a:L1560-	2	0.03
Delhi							ORF1a:L2364-	3	0.08	ORF1a:S1561-	2	0.03
Delhi							ORF1a:M2365-	3	0.08	ORF1a:L1562-	2	0.03
Delhi							ORF1a:W2366-	3	0.08	ORF1a:R1563-	2	0.03
Delhi							ORF1a:L2367-	3	0.08	ORF1a:E1564-	2	0.03
Delhi							ORF1a:I2368-	3	0.08	ORF1a:V1565-	2	0.03
Delhi							ORF1a:I2369-	3	0.08	ORF1a:R1566-	2	0.03
Delhi							ORF1a:N2370-	3	0.08	ORF1a:T1567-	2	0.03
Delhi							ORF1a:S2378-	3	0.08	ORF1a:I1568-	2	0.03
Delhi							ORF1a:A2379-	3	0.08	ORF1a:K1569-	2	0.03
Delhi							ORF1a:M2380-	3	0.08	ORF1a:V1570-	2	0.03
Delhi							ORF1a:V2381-	3	0.08	ORF1a:F1571-	2	0.03
Delhi							ORF1a:R2382-	3	0.08	ORF1a:T1572-	2	0.03
Delhi							ORF1a:M2383-	3	0.08	ORF1a:I1577-	1	0.01
Delhi							ORF1a:Y2384-	3	0.08	ORF1a:N1578-	1	0.01
Delhi							ORF1a:I2385-	3	0.08	ORF1a:L1579-	1	0.01
Delhi							ORF1a:F2386-	3	0.08	ORF1a:H1580-	1	0.01
Delhi							ORF1a:F2387-	3	0.08	ORF1a:T1581-	1	0.01
Delhi							ORF1a:A2388-	3	0.08	ORF1a:Q1582-	1	0.01
Delhi							ORF1a:S2389-	3	0.08	ORF1a:V1583-	1	0.01
Delhi							ORF1a:F2390-	3	0.08	ORF1a:V1584-	2	0.03
Delhi							ORF1a:Y2391-	3	0.08	ORF1a:D1585-	2	0.03
Delhi							ORF1a:Y2392-	3	0.08	ORF1a:M1586-	2	0.03
Delhi							ORF1a:V2393-	3	0.08	ORF1a:L1683-	2	0.03
Delhi							ORF1a:W2394-	3	0.08	ORF1a:Q1684-	2	0.03
Delhi							ORF1a:K2395-	3	0.08	ORF1a:Q1685-	2	0.03
Delhi							ORF1a:S2396-	3	0.08	ORF1a:I1686-	2	0.03
Delhi							ORF1a:Y2397-	3	0.08	ORF1a:E1687-	2	0.03
Delhi							ORF1a:V2398-	3	0.08	ORF1a:L1688-	2	0.03
Delhi							ORF1a:H2399-	3	0.08	ORF1a:K1689-	2	0.03
Delhi							ORF1a:V2400-	3	0.08	ORF1a:F1690-	2	0.03
Delhi							ORF1a:T2408-	3	0.08	ORF1a:N1691-	2	0.03
Delhi							ORF1a:C2409-	3	0.08	ORF1a:P1692-	2	0.03
Delhi							ORF1a:M2410-	3	0.08	ORF1a:P1693-	2	0.03
Delhi							ORF1a:M2411-	3	0.08	ORF1a:A1694-	2	0.03
Delhi							ORF1a:C2412-	3	0.08	ORF1a:L1695-	2	0.03
Delhi							ORF1a:Y2413-	3	0.08	ORF1a:Q1696-	2	0.03
Delhi							ORF1a:K2414-	3	0.08	ORF1a:D1697-	2	0.03

Delhi							ORF1a:R2415-	3	0.08	ORF1a:A1698-	2	0.03
Delhi							ORF1a:N2416-	3	0.08	ORF1a:Y1699-	2	0.03
Delhi							ORF1a:R2417-	3	0.08	ORF1a:Y1700-	2	0.03
Delhi							ORF1a:A2418-	3	0.08	ORF1a:R1701-	2	0.03
Delhi							ORF1a:T2419-	3	0.08	ORF1a:A1702-	2	0.03
Delhi							ORF1a:R2420-	3	0.08	ORF1a:R1703-	2	0.03
Delhi							ORF1a:V2421-	3	0.08	ORF1a:A1704-	2	0.03
Delhi							ORF1a:E2422-	3	0.08	ORF1a:G1705-	2	0.03
Delhi							ORF1a:C2423-	3	0.08	ORF1a:E1706-	2	0.03
Delhi							ORF1a:T2424-	3	0.08	ORF1a:G1726-	2	0.03
Delhi							ORF1a:T2425-	3	0.08	ORF1a:D1727-	2	0.03
Delhi							ORF1a:I2426-	3	0.08	ORF1a:V1728-	2	0.03
Delhi							ORF1a:V2427-	3	0.08	ORF1a:R1729-	2	0.03
Delhi							ORF1a:N2428-	3	0.08	ORF1a:E1730-	2	0.03
Delhi							ORF1a:G2429-	3	0.08	ORF1a:T1731-	2	0.03
Delhi							ORF1a:V2430-	3	0.08	ORF1a:M1732-	2	0.03
Delhi							ORF1a:R2431-	3	0.08	ORF1a:S1733-	2	0.03
Delhi							ORF1a:R2432-	3	0.08	ORF1a:Y1734-	2	0.03
Delhi							ORF1a:A2438-	3	0.08	ORF1a:L1735-	2	0.03
Delhi							ORF1a:N2439-	3	0.08	ORF1a:F1736-	2	0.03
Delhi							ORF1a:G2440-	3	0.08	ORF1a:Q1737-	2	0.03
Delhi							ORF1a:G2441-	3	0.08	ORF1a:H1738-	2	0.03
Delhi							ORF1a:K2442-	3	0.08	ORF1a:A1739-	2	0.03
Delhi							ORF1a:G2443-	3	0.08	ORF1a:N1740-	2	0.03
Delhi							ORF1a:F2444-	3	0.08	ORF1a:L1741-	2	0.03
Delhi							ORF1a:C2445-	3	0.08	ORF1a:D1742-	2	0.03
Delhi							ORF1a:K2446-	3	0.08	ORF1a:S1743-	2	0.03
Delhi							ORF1a:L2447-	3	0.08	ORF1a:C1744-	2	0.03
Delhi							ORF1a:H2448-	3	0.08	ORF1a:K1745-	2	0.03
Delhi							ORF1a:N2449-	3	0.08	ORF1a:R1746-	2	0.03
Delhi							ORF1a:W2450-	3	0.08	ORF1a:V1747-	2	0.03
Delhi							ORF1a:N2451-	3	0.08	ORF1a:E1777-	2	0.03
Delhi							ORF1a:C2452-	3	0.08	ORF1a:Q1778-	2	0.03
Delhi							ORF1a:V2453-	3	0.08	ORF1a:F1779-	2	0.03
Delhi							ORF1a:N2454-	3	0.08	ORF1a:K1780-	2	0.03
Delhi							ORF1a:C2455-	3	0.08	ORF1a:K1781-	2	0.03
Delhi							ORF1a:D2456-	3	0.08	ORF1a:G1782-	2	0.03
Delhi							ORF1a:T2457-	3	0.08	ORF1a:V1783-	2	0.03
Delhi							ORF1a:F2458-	3	0.08	ORF1a:Q1784-	2	0.03
Delhi							ORF1a:F2464-	3	0.08	ORF1a:I1785-	2	0.03
Delhi							ORF1a:I2465-	3	0.08	ORF1a:P1786-	2	0.03
Delhi							ORF1a:S2466-	3	0.08	ORF1a:C1787-	2	0.03
Delhi							ORF1a:D2467-	3	0.08	ORF1a:T1788-	2	0.03
Delhi							ORF1a:E2468-	3	0.08	ORF1a:C1789-	2	0.03
Delhi							ORF1a:V2469-	3	0.08	ORF1a:G1790-	2	0.03
Delhi							ORF1a:A2470-	3	0.08	ORF1a:K1791-	2	0.03
Delhi							ORF1a:R2471-	3	0.08	ORF1a:Q1792-	2	0.03
Delhi							ORF1a:D2472-	3	0.08	ORF1a:A1793-	2	0.03
Delhi							ORF1a:L2473-	3	0.08	ORF1a:T1794-	2	0.03
Delhi							ORF1a:S2474-	3	0.08	ORF1a:K1795-	2	0.03
Delhi							ORF1a:L2475-	3	0.08	ORF1a:Q1799-	2	0.03
Delhi							ORF1a:Q2476-	3	0.08	ORF1a:Q1800-	2	0.03
Delhi							ORF1a:F2477-	3	0.08	ORF1a:E1801-	2	0.03
Delhi							ORF1a:K2478-	3	0.08	ORF1a:S1802-	2	0.03
Delhi							ORF1a:R2479-	3	0.08	ORF1a:P1803-	2	0.03
Delhi							ORF1a:P2480-	3	0.08	ORF1a:F1804-	2	0.03
Delhi							ORF1a:V2494-	3	0.08	ORF1a:V1805-	2	0.03
Delhi							ORF1a:T2495-	3	0.08	ORF1a:M1806-	2	0.03
Delhi							ORF1a:V2496-	3	0.08	ORF1a:M1807-	2	0.03
Delhi							ORF1a:K2497-	3	0.08	ORF1a:S1808-	2	0.03
Delhi							ORF1a:N2498-	3	0.08	ORF1a:A1809-	2	0.03
Delhi							ORF1a:G2499-	3	0.08	ORF1a:P1810-	2	0.03
Delhi							ORF1a:S2500-	3	0.08	ORF1a:P1811-	2	0.03
Delhi							ORF1a:I2501-	3	0.08	ORF1a:A1812-	2	0.03
Delhi							ORF1a:H2502-	3	0.08	ORF1a:Q1813-	2	0.03
Delhi							ORF1a:L2503-	3	0.08	ORF1a:Y1814-	2	0.03
Delhi							ORF1a:Y2504-	3	0.08	ORF1a:E1815-	2	0.03
Delhi							ORF1a:Y2513-	3	0.08	ORF1a:L1816-	2	0.03
Delhi							ORF1a:E2514-	3	0.08	ORF1a:K1817-	2	0.03
Delhi							ORF1a:R2515-	3	0.08	ORF1a:H1818-	2	0.03
Delhi							ORF1a:H2520-	3	0.08	ORF1a:G1819-	2	0.03
Delhi							ORF1a:D2525-	3	0.08	ORF1a:I1839-	2	0.03
Delhi							ORF1a:N2526-	3	0.08	ORF1a:T1840-	2	0.03

Delhi							ORF1a:L2527-	3	0.08	ORF1a:S1841-	2	0.03
Delhi							ORF1a:R2528-	3	0.08	ORF1a:K1842-	2	0.03
Delhi							ORF1a:A2529-	3	0.08	ORF1a:E1843-	2	0.03
Delhi							ORF1a:N2530-	3	0.08	ORF1a:T1844-	2	0.03
Delhi							ORF1a:N2531-	3	0.08	ORF1a:L1845-	2	0.03
Delhi							ORF1a:T2532-	3	0.08	ORF1a:Y1846-	2	0.03
Delhi							ORF1a:K2533-	3	0.08	ORF1a:C1847-	2	0.03
Delhi							ORF1a:G2534-	3	0.08	ORF1a:I1848-	2	0.03
Delhi							ORF1a:V2542-	3	0.08	ORF1a:D1849-	2	0.03
Delhi							ORF1a:F2543-	3	0.08	ORF1a:G1850-	2	0.03
Delhi							ORF1a:D2544-	3	0.08	ORF1a:A1851-	2	0.03
Delhi							ORF1a:G2545-	3	0.08	ORF1a:L1852-	2	0.03
Delhi							ORF1a:K2546-	3	0.08	ORF1a:L1853-	2	0.03
Delhi							ORF1a:S2547-	3	0.08	ORF1a:T1854-	2	0.03
Delhi							ORF1a:K2548-	3	0.08	ORF1a:K1855-	2	0.03
Delhi							ORF1a:C2549-	3	0.08	ORF1a:S1856-	2	0.03
Delhi							ORF1a:E2550-	3	0.08	ORF1a:S1857-	2	0.03
Delhi							ORF1a:E2551-	3	0.08	ORF1a:E1858-	2	0.03
Delhi							ORF1a:S2552-	3	0.08	ORF1a:Y1859-	2	0.03
Delhi							ORF1a:S2553-	3	0.08	ORF1a:K1860-	2	0.03
Delhi							ORF1a:A2554-	3	0.08	ORF1a:G1861-	2	0.03
Delhi							ORF1a:K2555-	3	0.08	ORF1a:P1862-	2	0.03
Delhi							ORF1a:S2556-	3	0.08	ORF1a:T1874-	2	0.03
Delhi							ORF1a:A2557-	3	0.08	ORF1a:T1875-	2	0.03
Delhi							ORF1a:Y2560-	3	0.08	ORF1a:T1876-	2	0.03
Delhi							ORF1a:Y2561-	3	0.08	ORF1a:I1877-	2	0.03
Delhi							ORF1a:S2562-	3	0.08	ORF1a:K1878-	2	0.03
Delhi							ORF1a:Q2563-	3	0.08	ORF1a:P1879-	2	0.03
Delhi							ORF1a:L2564-	3	0.08	ORF1a:V1880-	2	0.03
Delhi							ORF1a:M2565-	3	0.08	ORF1a:T1881-	2	0.03
Delhi							ORF1a:C2566-	3	0.08	ORF1a:Y1882-	2	0.03
Delhi							ORF1a:Q2567-	3	0.08	ORF1a:K1883-	2	0.03
Delhi							ORF1a:P2568-	3	0.08	ORF1a:L1884-	2	0.03
Delhi							ORF1a:I2569-	3	0.08	ORF1a:D1885-	2	0.03
Delhi							ORF1a:F2598-	3	0.08	ORF1a:G1886-	2	0.03
Delhi							ORF1a:S2599-	3	0.08	ORF1a:V1887-	2	0.03
Delhi							ORF1a:S2600-	3	0.08	ORF1a:V1888-	2	0.03
Delhi							ORF1a:T2601-	3	0.08	ORF1a:C1889-	2	0.03
Delhi							ORF1a:F2602-	3	0.08	ORF1a:T1890-	2	0.03
Delhi							ORF1a:N2603-	3	0.08	ORF1a:E1891-	2	0.03
Delhi							ORF1a:V2604-	3	0.08	ORF1a:I1892-	2	0.03
Delhi							ORF1a:P2605-	3	0.08	ORF1a:D1893-	2	0.03
Delhi							ORF1a:M2606-	3	0.08	ORF1a:P1894-	2	0.03
Delhi							ORF1a:E2607-	3	0.08	ORF1a:K1895-	2	0.03
Delhi							ORF1a:K2608-	3	0.08	ORF1a:E1909-	2	0.03
Delhi							ORF1a:L2609-	3	0.08	ORF1a:Q1910-	2	0.03
Delhi							ORF1a:K2610-	3	0.08	ORF1a:P1911-	2	0.03
Delhi							ORF1a:T2611-	3	0.08	ORF1a:I1912-	2	0.03
Delhi							ORF1a:L2612-	3	0.08	ORF1a:D1913-	2	0.03
Delhi							ORF1a:V2613-	3	0.08	ORF1a:L1914-	2	0.03
Delhi							ORF1a:A2614-	3	0.08	ORF1a:V1915-	2	0.03
Delhi							ORF1a:T2615-	3	0.08	ORF1a:P1916-	2	0.03
Delhi							ORF1a:A2616-	3	0.08	ORF1a:N1917-	2	0.03
Delhi							ORF1a:E2617-	3	0.08	ORF1a:Q1918-	2	0.03
Delhi							ORF1a:A2618-	3	0.08	ORF1a:P1919-	2	0.03
Delhi							ORF1a:E2619-	3	0.08	ORF1a:Y1920-	2	0.03
Delhi							ORF1a:L2620-	3	0.08	ORF1a:P1921-	2	0.03
Delhi							ORF1a:A2621-	3	0.08	ORF1a:N1922-	2	0.03
Delhi							ORF1a:Q2639-	3	0.08	ORF1a:A1923-	2	0.03
Delhi							ORF1a:G2640-	3	0.08	ORF1a:S1924-	2	0.03
Delhi							ORF1a:F2641-	3	0.08	ORF1a:F1925-	2	0.03
Delhi							ORF1a:V2642-	3	0.08	ORF1a:D1926-	2	0.03
Delhi							ORF1a:D2643-	3	0.08	ORF1a:N1927-	2	0.03
Delhi							ORF1a:S2644-	3	0.08	ORF1a:F1928-	2	0.03
Delhi							ORF1a:D2645-	3	0.08	ORF1a:I1935-	2	0.03
Delhi							ORF1a:V2646-	3	0.08	ORF1a:K1936-	2	0.03
Delhi							ORF1a:E2647-	3	0.08	ORF1a:F1937-	2	0.03
Delhi							ORF1a:T2648-	3	0.08	ORF1a:A1938-	2	0.03
Delhi							ORF1a:K2649-	3	0.08	ORF1a:D1939-	2	0.03
Delhi							ORF1a:D2650-	3	0.08	ORF1a:D1940-	2	0.03
Delhi							ORF1a:V2651-	3	0.08	ORF1a:L1941-	2	0.03
Delhi							ORF1a:D2662-	3	0.08	ORF1a:N1942-	2	0.03
Delhi							ORF1a:I2663-	3	0.08	ORF1a:Q1943-	2	0.03

Delhi							ORF1a:E2664-	3	0.08	ORF1a:L1944-	2	0.03
Delhi							ORF1a:V2665-	3	0.08	ORF1a:T1945-	2	0.03
Delhi							ORF1a:T2666-	3	0.08	ORF1a:G1946-	2	0.03
Delhi							ORF1a:G2667-	3	0.08	ORF1a:Y1947-	2	0.03
Delhi							ORF1a:D2668-	3	0.08	ORF1a:K1948-	2	0.03
Delhi							ORF1a:S2669-	3	0.08	ORF1a:K1949-	2	0.03
Delhi							ORF1a:C2670-	3	0.08	ORF1a:P1950-	2	0.03
Delhi							ORF1a:N2671-	3	0.08	ORF1a:A1951-	2	0.03
Delhi							ORF1a:N2672-	3	0.08	ORF1a:S1952-	2	0.03
Delhi							ORF1a:Y2673-	3	0.08	ORF1a:R1953-	2	0.03
Delhi							ORF1a:M2674-	3	0.08	ORF1a:N1964-	2	0.03
Delhi							ORF1a:L2675-	3	0.08	ORF1a:G1965-	2	0.03
Delhi							ORF1a:T2676-	3	0.08	ORF1a:D1966-	2	0.03
Delhi							ORF1a:S2695-	3	0.08	ORF1a:V1967-	2	0.03
Delhi							ORF1a:A2696-	3	0.08	ORF1a:V1968-	2	0.03
Delhi							ORF1a:R2697-	3	0.08	ORF1a:A1969-	2	0.03
Delhi							ORF1a:H2698-	3	0.08	ORF1a:I1970-	2	0.03
Delhi							ORF1a:I2699-	3	0.08	ORF1a:D1971-	2	0.03
Delhi							ORF1a:N2700-	3	0.08	ORF1a:Y1972-	2	0.03
Delhi							ORF1a:A2710-	3	0.08	ORF1a:K1973-	2	0.03
Delhi							ORF1a:L2711-	3	0.08	ORF1a:H1974-	2	0.03
Delhi							ORF1a:I2712-	3	0.08	ORF1a:Y1975-	2	0.03
Delhi							ORF1a:W2713-	3	0.08	ORF1a:T1976-	2	0.03
Delhi							ORF1a:N2714-	3	0.08	ORF1a:P1977-	2	0.03
Delhi							ORF1a:V2715-	3	0.08	ORF1a:S1978-	2	0.03
Delhi							ORF1a:K2716-	3	0.08	ORF1a:F1979-	2	0.03
Delhi							ORF1a:D2717-	3	0.08	ORF1a:K1980-	2	0.03
Delhi							ORF1a:F2718-	3	0.08	ORF1a:K1981-	2	0.03
Delhi							ORF1a:M2719-	3	0.08	ORF1a:G1982-	2	0.03
Delhi							ORF1a:S2720-	3	0.08	ORF1a:A1983-	2	0.03
Delhi							ORF1a:L2721-	3	0.08	ORF1a:N1995-	2	0.03
Delhi							ORF1a:S2722-	3	0.08	ORF1a:N1996-	2	0.03
Delhi							ORF1a:R2726-	3	0.08	ORF1a:A1997-	2	0.03
Delhi							ORF1a:K2727-	3	0.08	ORF1a:T1998-	2	0.03
Delhi							ORF1a:Q2728-	3	0.08	ORF1a:N1999-	2	0.03
Delhi							ORF1a:I2729-	3	0.08	ORF1a:K2000-	2	0.03
Delhi							ORF1a:R2730-	3	0.08	ORF1a:A2001-	2	0.03
Delhi							ORF1a:S2731-	3	0.08	ORF1a:T2002-	2	0.03
Delhi							ORF1a:A2732-	3	0.08	ORF1a:Y2003-	2	0.03
Delhi							ORF1a:A2733-	3	0.08	ORF1a:K2004-	2	0.03
Delhi							ORF1a:K2734-	3	0.08	ORF1a:P2005-	2	0.03
Delhi							ORF1a:K2735-	3	0.08	ORF1a:N2006-	2	0.03
Delhi							ORF1a:N2736-	3	0.08	ORF1a:T2007-	2	0.03
Delhi							ORF1a:N2737-	3	0.08	ORF1a:W2008-	2	0.03
Delhi							ORF1a:L2738-	3	0.08	ORF1a:C2009-	2	0.03
Delhi							ORF1a:P2739-	3	0.08	ORF1a:I2010-	2	0.03
Delhi							ORF1a:F2740-	3	0.08	ORF1a:R2011-	2	0.03
Delhi							ORF1a:K2741-	3	0.08	ORF1a:C2012-	2	0.03
Delhi							ORF1a:L2742-	3	0.08	ORF1a:L2013-	2	0.03
Delhi							ORF1a:T2743-	3	0.08	ORF1a:W2014-	2	0.03
Delhi							ORF1a:C2744-	3	0.08	ORF1a:E2031-	2	0.03
Delhi							ORF1a:A2745-	3	0.08	ORF1a:D2032-	2	0.03
Delhi							ORF1a:T2746-	3	0.08	ORF1a:A2033-	2	0.03
Delhi							S:F140-	3	0.08	ORF1a:Q2034-	2	0.03
Delhi							ORF1b:K1947-	3	0.08	ORF1a:G2035-	2	0.03
Delhi							ORF1b:H1948-	3	0.08	ORF1a:M2036-	2	0.03
Delhi							ORF1b:A1949-	3	0.08	ORF1a:D2037-	2	0.03
Delhi							ORF1b:F1950-	3	0.08	ORF1a:N2038-	2	0.03
Delhi							ORF1b:H1951-	3	0.08	ORF1a:L2039-	2	0.03
Delhi							ORF1b:T1952-	3	0.08	ORF1a:A2040-	2	0.03
Delhi							ORF1b:P1953-	3	0.08	ORF1a:C2041-	2	0.03
Delhi							ORF1b:A1954-	3	0.08	ORF1a:E2042-	2	0.03
Delhi							ORF1b:F1955-	3	0.08	ORF1a:D2043-	2	0.03
Delhi							ORF1b:D1956-	3	0.08	ORF1a:L2044-	2	0.03
Delhi							ORF1b:K1957-	3	0.08	ORF1a:K2045-	2	0.03
Delhi							ORF1b:S1958-	3	0.08	ORF1a:P2046-	2	0.03
Delhi							ORF1b:A1959-	3	0.08	ORF1a:V2047-	2	0.03
Delhi							ORF1b:F1960-	3	0.08	ORF1a:S2048-	2	0.03
Delhi							ORF1b:V1961-	3	0.08	ORF1a:E2049-	2	0.03
Delhi							ORF1b:N1962-	3	0.08	ORF1a:E2050-	2	0.03
Delhi							ORF1b:L1963-	3	0.08	ORF1a:V2051-	2	0.03
Delhi							ORF1b:K1964-	3	0.08	ORF1a:V2052-	2	0.03
Delhi							ORF1b:Q1965-	3	0.08	ORF1a:E2053-	2	0.03

Delhi							ORF1b:L1966-	3	0.08	ORF1a:N2054-	2	0.03
Delhi							ORF1b:P1967-	3	0.08	ORF1a:P2055-	2	0.03
Delhi							ORF1b:F1968-	3	0.08	ORF1a:T2056-	2	0.03
Delhi							ORF1b:F1969-	3	0.08	ORF1a:C2064-	2	0.03
Delhi							ORF1b:Y1970-	3	0.08	ORF1a:N2065-	2	0.03
Delhi							ORF1b:Y1971-	3	0.08	ORF1a:V2066-	2	0.03
Delhi							ORF1b:S1972-	3	0.08	ORF1a:K2067-	2	0.03
Delhi							ORF1b:D1973-	3	0.08	ORF1a:T2068-	2	0.03
Delhi							ORF1b:S1974-	3	0.08	ORF1a:T2069-	2	0.03
Delhi							ORF1b:P1975-	3	0.08	ORF1a:E2070-	2	0.03
Delhi							ORF1b:C1976-	3	0.08	ORF1a:V2071-	2	0.03
Delhi							ORF1b:E1977-	3	0.08	ORF1a:V2072-	2	0.03
Delhi							ORF1b:S1978-	3	0.08	ORF1a:G2073-	2	0.03
Delhi							ORF1b:H1979-	3	0.08	ORF1a:D2074-	2	0.03
Delhi							ORF1b:G1980-	3	0.08	ORF1a:I2075-	2	0.03
Delhi							ORF1b:K1981-	3	0.08	ORF1a:I2076-	2	0.03
Delhi							ORF1b:Q1982-	3	0.08	ORF1a:L2077-	2	0.03
Delhi							ORF1b:V1983-	3	0.08	ORF1a:K2078-	2	0.03
Delhi							ORF1b:V1984-	3	0.08	ORF1a:P2079-	2	0.03
Delhi							ORF1b:S1985-	3	0.08	ORF1a:A2080-	2	0.03
Delhi							ORF1b:D1986-	3	0.08	ORF1a:N2081-	2	0.03
Delhi							ORF1b:I1987-	3	0.08	ORF1a:L2084-	2	0.03
Delhi							ORF1b:D1988-	3	0.08	ORF1a:K2085-	2	0.03
Delhi							ORF1b:Y1989-	3	0.08	ORF1a:I2086-	2	0.03
Delhi							S:E156-	3	0.08	ORF1a:T2087-	2	0.03
Delhi							S:F157-	3	0.08	ORF1a:E2088-	2	0.03
Delhi							ORF1a:K141-	2	0.06	ORF1a:E2089-	2	0.03
Delhi							ORF1a:S142-	2	0.06	ORF1a:V2090-	2	0.03
Delhi							ORF1a:F143-	2	0.06	ORF1a:G2091-	2	0.03
Delhi							ORF8:G66-	2	0.06	ORF1a:H2092-	2	0.03
Delhi							ORF8:S67-	2	0.06	ORF1a:T2093-	2	0.03
Delhi							ORF1b:Q2663-	2	0.06	ORF1a:D2094-	2	0.03
Delhi							ORF1b:I2664-	2	0.06	ORF1a:L2095-	2	0.03
Delhi							ORF1b:N2665-	2	0.06	ORF1a:M2096-	2	0.03
Delhi							ORF1b:D2666-	2	0.06	ORF1a:A2097-	2	0.03
Delhi							ORF1b:M2667-	2	0.06	ORF1a:A2098-	2	0.03
Delhi							ORF1a:K1720-	2	0.06	ORF1a:Y2099-	2	0.03
Delhi							ORF1a:T1721-	2	0.06	ORF1a:V2100-	2	0.03
Delhi							ORF1a:V1722-	2	0.06	ORF1a:D2101-	2	0.03
Delhi							ORF1a:G1723-	2	0.06	ORF1a:N2102-	1	0.01
Delhi							ORF1a:D1742-	2	0.06	ORF1a:S2103-	1	0.01
Delhi							ORF1a:S1743-	2	0.06	ORF1a:S2104-	1	0.01
Delhi							ORF1a:T1890-	2	0.06	ORF1a:L2105-	1	0.01
Delhi							ORF1a:E1891-	2	0.06	ORF1a:T2106-	1	0.01
Delhi							ORF1a:L1914-	2	0.06	ORF1a:I2107-	1	0.01
Delhi							ORF1a:V1915-	2	0.06	ORF1a:K2108-	1	0.01
Delhi							ORF1a:P1916-	2	0.06	ORF1a:K2109-	1	0.01
Delhi							ORF1a:N1917-	2	0.06	ORF1a:R2115-	2	0.03
Delhi							ORF1a:Q1918-	2	0.06	ORF1a:V2116-	2	0.03
Delhi							ORF1a:P1919-	2	0.06	ORF1a:L2117-	2	0.03
Delhi							ORF1a:Y1920-	2	0.06	ORF1a:G2118-	2	0.03
Delhi							ORF1a:C1932-	2	0.06	ORF1a:L2119-	2	0.03
Delhi							ORF1a:D1933-	2	0.06	ORF1a:K2120-	2	0.03
Delhi							ORF1a:N1934-	2	0.06	ORF1a:T2121-	2	0.03
Delhi							ORF1a:M2194-	2	0.06	ORF1a:L2122-	2	0.03
Delhi							ORF1a:P2195-	2	0.06	ORF1a:A2123-	2	0.03
Delhi							ORF1a:T2196-	2	0.06	ORF1a:T2124-	2	0.03
Delhi							ORF1a:T2197-	2	0.06	ORF1a:H2125-	2	0.03
Delhi							ORF1a:I2198-	2	0.06	ORF1a:G2126-	2	0.03
Delhi							ORF1a:A2199-	2	0.06	ORF1a:L2127-	2	0.03
Delhi							ORF1a:K2200-	2	0.06	ORF1a:A2128-	2	0.03
Delhi							ORF1a:N2201-	2	0.06	ORF1a:A2129-	2	0.03
Delhi							ORF1a:F2223-	2	0.06	ORF1a:V2130-	2	0.03
Delhi							ORF1a:S2224-	2	0.06	ORF1a:N2131-	2	0.03
Delhi							ORF1a:K2225-	2	0.06	ORF1a:S2132-	2	0.03
Delhi							ORF1a:L2226-	2	0.06	ORF1a:V2133-	2	0.03
Delhi							ORF1a:I2227-	2	0.06	ORF1a:P2134-	1	0.01
Delhi							ORF1a:N2228-	2	0.06	ORF1a:W2135-	1	0.01
Delhi							ORF1a:I2229-	2	0.06	ORF1a:D2136-	1	0.01
Delhi							ORF1a:I2230-	2	0.06	ORF1a:T2137-	1	0.01
Delhi							ORF1a:I2231-	2	0.06	ORF1a:I2138-	1	0.01
Delhi							ORF1a:W2232-	2	0.06	ORF1a:A2139-	1	0.01
Delhi							ORF1a:G2258-	2	0.06	ORF1a:A2142-	2	0.03

Delhi							**ORF1a:M2259-**	2	0.06	**ORF1a:K2143-**	2	0.03
Delhi							**ORF1a:P2260-**	2	0.06	**ORF1a:P2144-**	2	0.03
Delhi							**ORF1a:V2753-**	2	0.06	**ORF1a:F2145-**	2	0.03
Delhi							**ORF1a:V2754-**	2	0.06	**ORF1a:L2146-**	2	0.03
Delhi							**ORF1a:T2755-**	2	0.06	**ORF1a:N2147-**	2	0.03
Delhi							**ORF1a:T2756-**	2	0.06	**ORF1a:K2148-**	2	0.03
Delhi							**ORF1a:K2757-**	2	0.06	**ORF1a:V2149-**	2	0.03
Delhi							**ORF1a:I2758-**	2	0.06	**ORF1a:V2150-**	2	0.03
Delhi							**ORF1a:A2759-**	2	0.06	**ORF1a:S2151-**	2	0.03
Delhi							**ORF1a:L2760-**	2	0.06	**ORF1a:T2152-**	2	0.03
Delhi							**ORF1a:K2761-**	2	0.06	**ORF1a:T2153-**	2	0.03
Delhi							**ORF1a:G2762-**	2	0.06	**ORF1a:T2154-**	2	0.03
Delhi							**ORF1a:G2763-**	2	0.06	**ORF1a:N2155-**	2	0.03
Delhi							**ORF1a:K2764-**	2	0.06	**ORF1a:I2156-**	2	0.03
Delhi							**ORF1a:I2765-**	2	0.06	**ORF1a:V2157-**	2	0.03
Delhi							**ORF1a:V2766-**	2	0.06	**ORF1a:T2158-**	2	0.03
Delhi							**ORF1a:N2767-**	2	0.06	**ORF1a:R2159-**	2	0.03
Delhi							**ORF1a:N2768-**	2	0.06	**ORF1a:C2160-**	2	0.03
Delhi							**ORF1a:W2769-**	2	0.06	**ORF1a:L2161-**	2	0.03
Delhi							**ORF1a:L2770-**	2	0.06	**ORF1a:N2162-**	2	0.03
Delhi							**ORF1a:K2771-**	2	0.06	**ORF1a:R2163-**	2	0.03
Delhi							**ORF1a:Q2772-**	2	0.06	**ORF1a:V2164-**	2	0.03
Delhi							**ORF1a:L2773-**	2	0.06	**ORF1a:C2165-**	2	0.03
Delhi							**ORF1a:I2774-**	2	0.06	**ORF1a:T2166-**	2	0.03
Delhi							**ORF1a:F1690-**	2	0.06	**ORF1a:F2173-**	2	0.03
Delhi							**ORF1a:N1691-**	2	0.06	**ORF1a:T2174-**	2	0.03
Delhi							**ORF1a:P1692-**	2	0.06	**ORF1a:L2175-**	2	0.03
Delhi							**ORF1a:P1693-**	2	0.06	**ORF1a:L2176-**	2	0.03
Delhi							**ORF1a:A1694-**	2	0.06	**ORF1a:L2177-**	2	0.03
Delhi							**ORF1a:L1695-**	2	0.06	**ORF1a:Q2178-**	2	0.03
Delhi							**ORF1a:Q1696-**	2	0.06	**ORF1a:L2179-**	2	0.03
Delhi							**ORF1a:D1697-**	2	0.06	**ORF1a:C2180-**	2	0.03
Delhi							**ORF1a:A1698-**	2	0.06	**ORF1a:T2181-**	2	0.03
Delhi							**ORF1a:Y1699-**	2	0.06	**ORF1a:F2182-**	2	0.03
Delhi							**ORF1a:P1961-**	2	0.06	**ORF1a:T2183-**	2	0.03
Delhi							**ORF1a:D1962-**	2	0.06	**ORF1a:R2184-**	2	0.03
Delhi							**ORF1a:L1963-**	2	0.06	**ORF1a:S2185-**	2	0.03
Delhi							**ORF1a:G2251-**	2	0.06	**ORF1a:T2186-**	2	0.03
Delhi							**ORF1a:V2252-**	2	0.06	**ORF1a:N2187-**	2	0.03
Delhi							**ORF1a:L2253-**	2	0.06	**ORF1a:S2188-**	2	0.03
Delhi							**ORF1a:M2254-**	2	0.06	**ORF1a:R2189-**	2	0.03
Delhi							**ORF1b:V1990-**	2	0.06	**ORF1a:A2192-**	2	0.03
Delhi							**ORF1b:P1991-**	2	0.06	**ORF1a:S2193-**	2	0.03
Delhi							**ORF1b:L1992-**	2	0.06	**ORF1a:M2194-**	2	0.03
Delhi							**ORF1b:K1993-**	2	0.06	**ORF1a:P2195-**	2	0.03
Delhi							**ORF1b:S1994-**	2	0.06	**ORF1a:T2196-**	2	0.03
Delhi							**ORF1b:A1995-**	2	0.06	**ORF1a:T2197-**	2	0.03
Delhi							**ORF1b:T1996-**	2	0.06	**ORF1a:I2198-**	2	0.03
Delhi							**ORF1b:C1997-**	2	0.06	**ORF1a:A2199-**	2	0.03
Delhi							**ORF1b:I1998-**	2	0.06	**ORF1a:K2200-**	2	0.03
Delhi							**ORF1b:T1999-**	2	0.06	**ORF1a:N2201-**	2	0.03
Delhi							**ORF7a:T120-**	1	0.03	**ORF1a:T2202-**	2	0.03
Delhi							**ORF7a:E121-**	1	0.03	**ORF1a:V2203-**	2	0.03
Delhi							**ORF7b:M1-**	1	0.03	**ORF1a:K2204-**	2	0.03
Delhi							**ORF7b:I2-**	1	0.03	**ORF1a:S2205-**	2	0.03
Delhi							**ORF1a:I1023-**	1	0.03	**ORF1a:V2206-**	2	0.03
Delhi							**ORF1a:E1024-**	1	0.03	**ORF1a:G2207-**	2	0.03
Delhi							**ORF1a:V1025-**	1	0.03	**ORF1a:S2237-**	2	0.03
Delhi							**ORF1a:N1026-**	1	0.03	**ORF1a:V2238-**	2	0.03
Delhi							**ORF1a:G4380-**	1	0.03	**ORF1a:C2239-**	2	0.03
Delhi							**ORF1a:C4381-**	1	0.03	**ORF1a:L2240-**	2	0.03
Delhi							**ORF1a:S4382-**	1	0.03	**ORF1a:G2241-**	2	0.03
Delhi							**ORF1a:C4383-**	1	0.03	**ORF1a:S2242-**	2	0.03
Delhi							**ORF1a:D4384-**	1	0.03	**ORF1a:L2243-**	2	0.03
Delhi							**ORF1a:Q4385-**	1	0.03	**ORF1a:I2244-**	2	0.03
Delhi							**ORF1a:L4386-**	1	0.03	**ORF1a:Y2245-**	2	0.03
Delhi							**ORF1a:R4387-**	1	0.03	**ORF1a:S2246-**	2	0.03
Delhi							**ORF1a:E4388-**	1	0.03	**ORF1a:T2247-**	2	0.03
Delhi							**ORF1a:P4389-**	1	0.03	**ORF1a:A2248-**	2	0.03
Delhi							**ORF1a:M4390-**	1	0.03	**ORF1a:A2249-**	2	0.03
Delhi							**ORF1a:L4391-**	1	0.03	**ORF1a:L2250-**	2	0.03
Delhi							**ORF1a:Q4392-**	1	0.03	**ORF1a:G2251-**	2	0.03
Delhi							**ORF1a:S4393-**	1	0.03	**ORF1a:V2252-**	2	0.03

Delhi							ORF1a:A4394-	1	0.03	ORF1a:L2253-	2	0.03
Delhi							ORF1a:D4395-	1	0.03	ORF1a:M2254-	2	0.03
Delhi							ORF1a:A4396-	1	0.03	ORF1a:S2255-	1	0.01
Delhi							ORF1a:Q4397-	1	0.03	ORF1a:N2256-	1	0.01
Delhi							ORF1a:S4398-	1	0.03	ORF1a:S2273-	2	0.03
Delhi							ORF1a:F4399-	1	0.03	ORF1a:T2274-	2	0.03
Delhi							ORF1a:L4400-	1	0.03	ORF1a:N2275-	2	0.03
Delhi							ORF1a:N4401-	1	0.03	ORF1a:V2276-	2	0.03
Delhi							ORF1b:R1-	1	0.03	ORF1a:T2277-	2	0.03
Delhi							S:L141-	1	0.03	ORF1a:I2278-	2	0.03
Delhi							S:Y145-	1	0.03	ORF1a:A2279-	2	0.03
Delhi							S:H146-	1	0.03	ORF1a:T2280-	2	0.03
Delhi							ORF1b:D2617-	1	0.03	ORF1a:Y2281-	2	0.03
Delhi							ORF1b:G2618-	1	0.03	ORF1a:C2282-	2	0.03
Delhi							ORF1b:Y2619-	1	0.03	ORF1a:T2283-	2	0.03
Delhi							ORF1b:V2620-	1	0.03	ORF1a:G2284-	2	0.03
Delhi							ORF1b:M2621-	1	0.03	ORF1a:S2285-	2	0.03
Delhi							ORF1b:H2622-	1	0.03	ORF1a:I2286-	2	0.03
Delhi							ORF1b:A2623-	1	0.03	ORF1a:P2287-	2	0.03
Delhi							ORF1b:N2624-	1	0.03	ORF1a:C2288-	1	0.01
Delhi							ORF1b:Y2625-	1	0.03	ORF1a:S2289-	1	0.01
Delhi							ORF1b:I2626-	1	0.03	ORF1a:V2290-	1	0.01
Delhi							ORF1b:F2627-	1	0.03	ORF1a:C2291-	1	0.01
Delhi							ORF1b:W2628-	1	0.03	ORF1a:L2292-	1	0.01
Delhi							ORF1b:R2629-	1	0.03	ORF1a:S2293-	1	0.01
Delhi							ORF1b:N2630-	1	0.03	ORF1a:F2314-	1	0.01
Delhi							ORF1b:T2631-	1	0.03	ORF1a:K2315-	1	0.01
Delhi							ORF1b:N2632-	1	0.03	ORF1a:W2316-	1	0.01
Delhi							ORF1b:P2633-	1	0.03	ORF1a:D2317-	1	0.01
Delhi							ORF1b:I2634-	1	0.03	ORF1a:L2318-	1	0.01
Delhi							ORF1b:Q2635-	1	0.03	ORF1a:T2319-	1	0.01
Delhi							ORF1b:L2636-	1	0.03	ORF1a:A2320-	1	0.01
Delhi							ORF1b:S2637-	1	0.03	ORF1a:F2321-	1	0.01
Delhi							ORF1b:S2638-	1	0.03	ORF1a:G2322-	1	0.01
Delhi							ORF1b:Y2639-	1	0.03	ORF1a:L2323-	1	0.01
Delhi							ORF1b:S2640-	1	0.03	ORF1a:V2324-	1	0.01
Delhi							ORF1b:L2641-	1	0.03	ORF1a:A2325-	2	0.03
Delhi							ORF1b:F2642-	1	0.03	ORF1a:E2326-	2	0.03
Delhi							ORF1b:D2643-	1	0.03	ORF1a:W2327-	2	0.03
Delhi							ORF1b:M2644-	1	0.03	ORF1a:F2328-	2	0.03
Delhi							ORF1b:S2645-	1	0.03	ORF1a:L2329-	2	0.03
Delhi							ORF1b:K2646-	1	0.03	ORF1a:A2330-	2	0.03
Delhi							ORF1b:F2647-	1	0.03	ORF1a:Y2331-	2	0.03
Delhi							ORF1b:P2648-	1	0.03	ORF1a:I2332-	2	0.03
Delhi							ORF1b:L2649-	1	0.03	ORF1a:L2333-	2	0.03
Delhi							ORF1b:K2650-	1	0.03	ORF1a:F2334-	2	0.03
Delhi							ORF1b:L2651-	1	0.03	ORF1a:T2335-	2	0.03
Delhi							ORF1b:R2652-	1	0.03	ORF1a:R2336-	2	0.03
Delhi							ORF1b:G2653-	1	0.03	ORF1a:I2359-	2	0.03
Delhi							ORF1b:T2654-	1	0.03	ORF1a:S2360-	2	0.03
Delhi							ORF1b:A2655-	1	0.03	ORF1a:N2361-	2	0.03
Delhi							ORF1b:V2656-	1	0.03	ORF1a:S2362-	2	0.03
Delhi							ORF1b:M2657-	1	0.03	ORF1a:W2363-	2	0.03
Delhi							ORF1b:S2658-	1	0.03	ORF1a:L2364-	2	0.03
Delhi							ORF1b:L2659-	1	0.03	ORF1a:M2365-	2	0.03
Delhi							ORF1b:K2660-	1	0.03	ORF1a:W2366-	2	0.03
Delhi							ORF1b:E2661-	1	0.03	ORF1a:L2367-	2	0.03
Delhi							ORF1b:G2662-	1	0.03	ORF1a:I2368-	2	0.03
Delhi							ORF1b:I2668-	1	0.03	ORF1a:I2369-	2	0.03
Delhi							ORF1b:L2669-	1	0.03	ORF1a:N2370-	2	0.03
Delhi							ORF1b:S2670-	1	0.03	ORF1a:V2372-	2	0.03
Delhi							ORF1b:L2671-	1	0.03	ORF1a:Q2373-	2	0.03
Delhi							ORF1b:L2672-	1	0.03	ORF1a:M2374-	2	0.03
Delhi							ORF1b:S2673-	1	0.03	ORF1a:A2375-	2	0.03
Delhi							ORF1b:K2674-	1	0.03	ORF1a:P2376-	2	0.03
Delhi							ORF1b:G2675-	1	0.03	ORF1a:I2377-	2	0.03
Delhi							ORF1b:R2676-	1	0.03	ORF1a:S2378-	2	0.03
Delhi							ORF1b:L2677-	1	0.03	ORF1a:A2379-	2	0.03
Delhi							ORF1b:I2678-	1	0.03	ORF1a:M2380-	2	0.03
Delhi							ORF1b:I2679-	1	0.03	ORF1a:V2381-	2	0.03
Delhi							ORF1b:R2680-	1	0.03	ORF1a:R2382-	2	0.03
Delhi							ORF1b:E2681-	1	0.03	ORF1a:M2383-	2	0.03
Delhi							ORF1b:N2682-	1	0.03	ORF1a:Y2384-	2	0.03

Delhi							ORF1b:N2683-	1	0.03	ORF1a:I2385-	2	0.03
Delhi							ORF1b:R2684-	1	0.03	ORF1a:F2386-	2	0.03
Delhi							ORF1b:V2685-	1	0.03	ORF1a:F2387-	2	0.03
Delhi							ORF1b:V2686-	1	0.03	ORF1a:A2388-	2	0.03
Delhi							ORF1b:I2687-	1	0.03	ORF1a:S2389-	2	0.03
Delhi							ORF1b:S2688-	1	0.03	ORF1a:F2390-	2	0.03
Delhi							ORF1b:S2689-	1	0.03	ORF1a:Y2391-	2	0.03
Delhi							ORF1b:D2690-	1	0.03	ORF1a:Y2392-	2	0.03
Delhi							ORF1b:V2691-	1	0.03	ORF1a:V2393-	2	0.03
Delhi							ORF1b:L2692-	1	0.03	ORF1a:W2394-	2	0.03
Delhi							ORF1b:V2693-	1	0.03	ORF1a:K2395-	2	0.03
Delhi							ORF1b:N2694-	1	0.03	ORF1a:S2396-	2	0.03
Delhi							ORF1b:N2695-	1	0.03	ORF1a:Y2397-	2	0.03
Delhi							ORF1b:*2696-	1	0.03	ORF1a:C2412-	2	0.03
Delhi							ORF1a:L1681-	1	0.03	ORF1a:Y2413-	2	0.03
Delhi							ORF1a:T1682-	1	0.03	ORF1a:K2414-	2	0.03
Delhi							ORF1a:L1683-	1	0.03	ORF1a:R2415-	2	0.03
Delhi							ORF1a:Q1684-	1	0.03	ORF1a:N2416-	2	0.03
Delhi							ORF1a:Q1685-	1	0.03	ORF1a:R2417-	2	0.03
Delhi							ORF1a:I1686-	1	0.03	ORF1a:A2418-	2	0.03
Delhi							ORF1a:E1687-	1	0.03	ORF1a:T2419-	2	0.03
Delhi							ORF1a:L1688-	1	0.03	ORF1a:R2420-	2	0.03
Delhi							ORF1a:K1689-	1	0.03	ORF1a:V2421-	2	0.03
Delhi							ORF1a:A1707-	1	0.03	ORF1a:E2422-	2	0.03
Delhi							ORF1a:A1708-	1	0.03	ORF1a:C2423-	2	0.03
Delhi							ORF1a:N1709-	1	0.03	ORF1a:T2424-	2	0.03
Delhi							ORF1a:F1710-	1	0.03	ORF1a:T2425-	2	0.03
Delhi							ORF1a:C1711-	1	0.03	ORF1a:I2426-	2	0.03
Delhi							ORF1a:A1712-	1	0.03	ORF1a:V2427-	2	0.03
Delhi							ORF1a:L1713-	1	0.03	ORF1a:N2428-	2	0.03
Delhi							ORF1a:I1714-	1	0.03	ORF1a:G2429-	2	0.03
Delhi							ORF1a:L1715-	1	0.03	ORF1a:V2430-	2	0.03
Delhi							ORF1a:A1716-	1	0.03	ORF1a:R2431-	2	0.03
Delhi							ORF1a:Y1717-	1	0.03	ORF1a:R2432-	2	0.03
Delhi							ORF1a:C1718-	1	0.03	ORF1a:S2433-	2	0.03
Delhi							ORF1a:N1719-	1	0.03	ORF1a:F2434-	2	0.03
Delhi							ORF1a:E1724-	1	0.03	ORF1a:H2448-	2	0.03
Delhi							ORF1a:T2202-	1	0.03	ORF1a:N2449-	2	0.03
Delhi							ORF1a:V2203-	1	0.03	ORF1a:W2450-	2	0.03
Delhi							ORF1a:K2204-	1	0.03	ORF1a:N2451-	2	0.03
Delhi							ORF1a:S2205-	1	0.03	ORF1a:C2452-	2	0.03
Delhi							ORF1a:V2206-	1	0.03	ORF1a:V2453-	2	0.03
Delhi							ORF1a:G2207-	1	0.03	ORF1a:N2454-	2	0.03
Delhi							ORF1a:F2233-	1	0.03	ORF1a:C2455-	2	0.03
Delhi							ORF1a:L2234-	1	0.03	ORF1a:D2456-	2	0.03
Delhi							ORF1a:L2235-	1	0.03	ORF1a:T2457-	2	0.03
Delhi							ORF1a:L2236-	1	0.03	ORF1a:F2458-	2	0.03
Delhi							ORF1a:S2237-	1	0.03	ORF1a:C2459-	2	0.03
Delhi							ORF1a:V2238-	1	0.03	ORF1a:A2460-	2	0.03
Delhi							ORF1a:C2239-	1	0.03	ORF1a:G2461-	2	0.03
Delhi							ORF1a:S2261-	1	0.03	ORF1a:S2462-	2	0.03
Delhi							ORF1a:Y2262-	1	0.03	ORF1a:T2463-	2	0.03
Delhi							ORF1a:Y1899-	1	0.03	ORF1a:F2464-	2	0.03
Delhi							ORF1a:Y1900-	1	0.03	ORF1a:I2465-	2	0.03
Delhi							ORF1a:K1901-	1	0.03	ORF1a:S2466-	2	0.03
Delhi							ORF1a:K1902-	1	0.03	ORF1a:D2467-	2	0.03
Delhi							ORF1a:R1953-	1	0.03	ORF1a:E2468-	2	0.03
Delhi							ORF1a:T2777-	1	0.03	ORF1a:V2469-	2	0.03
Delhi							ORF1a:L2778-	1	0.03	ORF1a:K2478-	2	0.03
Delhi							ORF1a:V2779-	1	0.03	ORF1a:R2479-	2	0.03
Delhi							ORF1a:F2780-	1	0.03	ORF1a:P2480-	2	0.03
Delhi							ORF1a:G445-	1	0.03	ORF1a:I2481-	2	0.03
Delhi							ORF1a:L446-	1	0.03	ORF1a:N2482-	2	0.03
Delhi							ORF7b:F13-	1	0.03	ORF1a:P2483-	2	0.03
Delhi							ORF1a:L3199-	1	0.03	ORF1a:T2484-	2	0.03
Delhi							ORF1a:P3200-	1	0.03	ORF1a:D2485-	2	0.03
Delhi							ORF1a:L3201-	1	0.03	ORF1a:Q2486-	2	0.03
Delhi							ORF1a:T3202-	1	0.03	ORF1a:S2487-	2	0.03
Delhi							ORF1a:Q3203-	1	0.03	ORF1a:S2488-	2	0.03
Delhi							ORF1a:Y3204-	1	0.03	ORF1a:Y2489-	2	0.03
Delhi							ORF1a:N3205-	1	0.03	ORF1a:I2490-	2	0.03
Delhi							ORF1a:R3206-	1	0.03	ORF1a:V2491-	2	0.03
Delhi							ORF1a:Y3207-	1	0.03	ORF1a:D2492-	2	0.03

Delhi							ORF1a:L3208-	1	0.03	ORF1a:S2493-	2	0.03
Delhi							ORF1a:A3209-	1	0.03	ORF1a:V2494-	2	0.03
Delhi							ORF1a:L3210-	1	0.03	ORF1a:T2495-	2	0.03
Delhi							ORF1a:Y3211-	1	0.03	ORF1a:V2496-	2	0.03
Delhi							ORF1a:N3212-	1	0.03	ORF1a:K2497-	2	0.03
Delhi							ORF1a:G1726-	1	0.03	ORF1a:N2498-	2	0.03
Delhi							ORF1a:D1727-	1	0.03	ORF1a:G2499-	2	0.03
Delhi							ORF1a:V1728-	1	0.03	ORF1a:S2500-	2	0.03
Delhi							ORF1a:R1729-	1	0.03	ORF1a:I2501-	2	0.03
Delhi							ORF1a:E1730-	1	0.03	ORF1a:H2502-	2	0.03
Delhi							ORF1a:T1731-	1	0.03	ORF1a:Y2513-	2	0.03
Delhi							ORF1a:M1732-	1	0.03	ORF1a:E2514-	2	0.03
Delhi							ORF1a:S1733-	1	0.03	ORF1a:R2515-	2	0.03
Delhi							ORF1a:Y1734-	1	0.03	ORF1a:F2521-	2	0.03
Delhi							ORF1a:L1735-	1	0.03	ORF1a:V2522-	2	0.03
Delhi							ORF1a:F1736-	1	0.03	ORF1a:N2523-	2	0.03
Delhi							ORF1a:Q1737-	1	0.03	ORF1a:L2524-	2	0.03
Delhi							ORF1a:H1738-	1	0.03	ORF1a:D2525-	2	0.03
Delhi							ORF1a:A1739-	1	0.03	ORF1a:N2526-	2	0.03
Delhi							ORF1a:N1740-	1	0.03	ORF1a:L2527-	2	0.03
Delhi							ORF1a:Q1759-	1	0.03	ORF1a:R2528-	2	0.03
Delhi							ORF1a:T1760-	1	0.03	ORF1a:A2529-	2	0.03
Delhi							ORF1a:T1761-	1	0.03	ORF1a:N2530-	2	0.03
Delhi							ORF1a:S2193-	1	0.03	ORF1a:N2531-	2	0.03
Delhi							ORF1a:C2210-	1	0.03	ORF1a:T2532-	2	0.03
Delhi							ORF1a:L2211-	1	0.03	ORF1a:K2533-	2	0.03
Delhi							ORF1a:E2212-	1	0.03	ORF1a:G2534-	2	0.03
Delhi							ORF1a:A2213-	1	0.03	ORF1a:S2535-	2	0.03
Delhi							ORF1a:S2214-	1	0.03	ORF1a:L2536-	2	0.03
Delhi							ORF1a:F2215-	1	0.03	ORF1a:P2537-	2	0.03
Delhi							ORF1a:N2216-	1	0.03	ORF1a:I2538-	2	0.03
Delhi							ORF1a:Y2217-	1	0.03	ORF1a:N2539-	2	0.03
Delhi							ORF1a:L2218-	1	0.03	ORF1a:V2540-	2	0.03
Delhi							ORF1a:K2219-	1	0.03	ORF1a:I2541-	2	0.03
Delhi							ORF1a:S2220-	1	0.03	ORF1a:V2542-	2	0.03
Delhi							ORF1a:P2221-	1	0.03	ORF1a:F2543-	2	0.03
Delhi							ORF1a:N2222-	1	0.03	ORF1a:D2544-	2	0.03
Delhi							ORF1a:L2240-	1	0.03	ORF1a:G2545-	2	0.03
Delhi							ORF1a:G2241-	1	0.03	ORF1a:K2546-	2	0.03
Delhi							ORF1a:S2242-	1	0.03	ORF1a:E2550-	2	0.03
Delhi							ORF1a:L2243-	1	0.03	ORF1a:E2551-	2	0.03
Delhi							ORF1a:I2244-	1	0.03	ORF1a:S2552-	2	0.03
Delhi							ORF1a:Y2245-	1	0.03	ORF1a:S2553-	2	0.03
Delhi							ORF1a:S2246-	1	0.03	ORF1a:A2554-	2	0.03
Delhi							ORF1a:T2247-	1	0.03	ORF1a:K2555-	2	0.03
Delhi							ORF1a:A2248-	1	0.03	ORF1a:S2556-	2	0.03
Delhi							ORF1a:A2249-	1	0.03	ORF1a:A2557-	2	0.03
Delhi							ORF1a:L2250-	1	0.03	ORF1a:S2558-	2	0.03
Delhi							ORF1a:S2255-	1	0.03	ORF1a:V2559-	2	0.03
Delhi							ORF1a:N2256-	1	0.03	ORF1a:Y2560-	2	0.03
Delhi							ORF1a:L2257-	1	0.03	ORF1a:Y2561-	2	0.03
Delhi							S:S112-	1	0.03	ORF1a:S2562-	2	0.03
Delhi							S:V120-	1	0.03	ORF1a:Q2563-	2	0.03
Delhi							S:E1262-	1	0.03	ORF1a:L2564-	2	0.03
Delhi							S:P1263-	1	0.03	ORF1a:M2565-	2	0.03
Delhi							S:L244-	1	0.03	ORF1a:C2566-	2	0.03
Delhi							S:H245-	1	0.03	ORF1a:Q2567-	2	0.03
Delhi							S:R246-	1	0.03	ORF1a:P2568-	2	0.03
Delhi							S:S247-	1	0.03	ORF1a:I2569-	2	0.03
Delhi										ORF1a:L2570-	2	0.03
Delhi										ORF1a:D2582-	2	0.03
Delhi										ORF1a:S2583-	2	0.03
Delhi										ORF1a:A2584-	2	0.03
Delhi										ORF1a:E2585-	2	0.03
Delhi										ORF1a:V2586-	2	0.03
Delhi										ORF1a:A2587-	2	0.03
Delhi										ORF1a:V2588-	2	0.03
Delhi										ORF1a:K2589-	2	0.03
Delhi										ORF1a:M2590-	2	0.03
Delhi										ORF1a:F2591-	2	0.03
Delhi										ORF1a:D2592-	2	0.03
Delhi										ORF1a:A2593-	2	0.03
Delhi										ORF1a:Y2594-	2	0.03

Delhi										**ORF1a:V2595-**	2	0.03
Delhi										**ORF1a:N2596-**	2	0.03
Delhi										**ORF1a:T2597-**	2	0.03
Delhi										**ORF1a:F2598-**	2	0.03
Delhi										**ORF1a:P2605-**	2	0.03
Delhi										**ORF1a:M2606-**	2	0.03
Delhi										**ORF1a:E2607-**	2	0.03
Delhi										**ORF1a:K2608-**	2	0.03
Delhi										**ORF1a:L2609-**	2	0.03
Delhi										**ORF1a:K2610-**	2	0.03
Delhi										**ORF1a:T2611-**	2	0.03
Delhi										**ORF1a:L2612-**	2	0.03
Delhi										**ORF1a:V2613-**	2	0.03
Delhi										**ORF1a:A2614-**	2	0.03
Delhi										**ORF1a:T2615-**	2	0.03
Delhi										**ORF1a:A2616-**	2	0.03
Delhi										**ORF1a:E2617-**	2	0.03
Delhi										**ORF1a:A2618-**	2	0.03
Delhi										**ORF1a:E2619-**	2	0.03
Delhi										**ORF1a:L2620-**	2	0.03
Delhi										**ORF1a:A2621-**	2	0.03
Delhi										**ORF1a:K2622-**	2	0.03
Delhi										**ORF1a:N2623-**	2	0.03
Delhi										**ORF1a:V2624-**	2	0.03
Delhi										**ORF1a:S2625-**	2	0.03
Delhi										**ORF1a:L2626-**	2	0.03
Delhi										**ORF1a:D2627-**	2	0.03
Delhi										**ORF1a:Q2639-**	2	0.03
Delhi										**ORF1a:G2640-**	2	0.03
Delhi										**ORF1a:F2641-**	2	0.03
Delhi										**ORF1a:V2642-**	2	0.03
Delhi										**ORF1a:D2643-**	2	0.03
Delhi										**ORF1a:S2644-**	2	0.03
Delhi										**ORF1a:D2645-**	2	0.03
Delhi										**ORF1a:V2646-**	2	0.03
Delhi										**ORF1a:E2647-**	2	0.03
Delhi										**ORF1a:T2648-**	2	0.03
Delhi										**ORF1a:K2649-**	2	0.03
Delhi										**ORF1a:D2650-**	2	0.03
Delhi										**ORF1a:V2651-**	2	0.03
Delhi										**ORF1a:D2662-**	2	0.03
Delhi										**ORF1a:I2663-**	2	0.03
Delhi										**ORF1a:E2664-**	2	0.03
Delhi										**ORF1a:V2665-**	2	0.03
Delhi										**ORF1a:T2666-**	2	0.03
Delhi										**ORF1a:G2667-**	2	0.03
Delhi										**ORF1a:D2668-**	2	0.03
Delhi										**ORF1a:S2669-**	2	0.03
Delhi										**ORF1a:C2670-**	2	0.03
Delhi										**ORF1a:N2671-**	2	0.03
Delhi										**ORF1a:N2672-**	2	0.03
Delhi										**ORF1a:Y2673-**	2	0.03
Delhi										**ORF1a:M2674-**	2	0.03
Delhi										**ORF1a:L2675-**	2	0.03
Delhi										**ORF1a:T2676-**	2	0.03
Delhi										**ORF1a:S2695-**	2	0.03
Delhi										**ORF1a:A2696-**	2	0.03
Delhi										**ORF1a:R2697-**	2	0.03
Delhi										**ORF1a:H2698-**	2	0.03
Delhi										**ORF1a:I2699-**	2	0.03
Delhi										**ORF1a:N2700-**	2	0.03
Delhi										**ORF1a:A2710-**	2	0.03
Delhi										**ORF1a:L2711-**	2	0.03
Delhi										**ORF1a:I2712-**	2	0.03
Delhi										**ORF1a:W2713-**	2	0.03
Delhi										**ORF1a:N2714-**	2	0.03
Delhi										**ORF1a:V2715-**	2	0.03
Delhi										**ORF1a:K2716-**	2	0.03
Delhi										**ORF1a:D2717-**	2	0.03
Delhi										**ORF1a:F2718-**	2	0.03
Delhi										**ORF1a:M2719-**	2	0.03
Delhi										**ORF1a:S2720-**	2	0.03
Delhi										**ORF1a:L2721-**	2	0.03

Delhi										ORF1a:S2722-	2	0.03
Delhi										ORF1a:E2723-	2	0.03
Delhi										ORF1a:Q2724-	2	0.03
Delhi										ORF1a:L2725-	2	0.03
Delhi										ORF1a:R2726-	2	0.03
Delhi										ORF1a:K2727-	2	0.03
Delhi										ORF1a:Q2728-	2	0.03
Delhi										ORF1a:I2729-	2	0.03
Delhi										ORF1a:R2730-	2	0.03
Delhi										ORF1a:S2731-	2	0.03
Delhi										ORF1a:A2732-	2	0.03
Delhi										ORF1a:A2733-	2	0.03
Delhi										ORF1a:K2734-	2	0.03
Delhi										ORF1a:K2735-	2	0.03
Delhi										ORF1a:A2745-	2	0.03
Delhi										ORF1a:T2746-	2	0.03
Delhi										ORF1a:T2747-	2	0.03
Delhi										ORF1a:R2748-	2	0.03
Delhi										ORF1a:Q2749-	2	0.03
Delhi										ORF1a:V2750-	2	0.03
Delhi										ORF1a:V2751-	2	0.03
Delhi										ORF1a:N2752-	2	0.03
Delhi										ORF1a:V2753-	2	0.03
Delhi										ORF1a:V2754-	2	0.03
Delhi										ORF1a:T2755-	2	0.03
Delhi										ORF1a:T2756-	2	0.03
Delhi										ORF1a:K2757-	2	0.03
Delhi										ORF1a:I2758-	2	0.03
Delhi										ORF1a:A2759-	2	0.03
Delhi										ORF1a:L2760-	2	0.03
Delhi										ORF1a:K2761-	2	0.03
Delhi										ORF1a:G2762-	2	0.03
Delhi										ORF1a:G2763-	2	0.03
Delhi										ORF1a:K2764-	2	0.03
Delhi										ORF1a:I2765-	2	0.03
Delhi										ORF1a:V2766-	2	0.03
Delhi										ORF1a:K2771-	2	0.03
Delhi										ORF1a:Q2772-	2	0.03
Delhi										ORF1a:L2773-	2	0.03
Delhi										ORF1a:I2774-	2	0.03
Delhi										ORF1a:K2775-	2	0.03
Delhi										ORF1a:V2776-	2	0.03
Delhi										ORF1a:T2777-	2	0.03
Delhi										ORF1a:L2778-	2	0.03
Delhi										ORF1a:V2779-	2	0.03
Delhi										ORF1a:F2780-	2	0.03
Delhi										ORF1a:L2781-	2	0.03
Delhi										ORF1a:F2782-	2	0.03
Delhi										ORF1a:S1587-	1	0.01
Delhi										ORF1a:M1588-	1	0.01
Delhi										ORF1a:T1589-	1	0.01
Delhi										ORF1a:Y1590-	1	0.01
Delhi										ORF1a:G1591-	1	0.01
Delhi										ORF1a:Q1592-	1	0.01
Delhi										ORF1a:Q1593-	1	0.01
Delhi										ORF1a:F1594-	1	0.01
Delhi										ORF1a:G1595-	1	0.01
Delhi										ORF1a:P1596-	1	0.01
Delhi										ORF1a:T1597-	1	0.01
Delhi										ORF1a:Y1598-	1	0.01
Delhi										ORF1a:L1599-	1	0.01
Delhi										ORF1a:D1600-	1	0.01
Delhi										ORF1a:G1601-	1	0.01
Delhi										ORF1a:A1602-	1	0.01
Delhi										ORF1a:D1603-	1	0.01
Delhi										ORF1a:V1604-	1	0.01
Delhi										ORF1a:T1605-	1	0.01
Delhi										ORF1a:K1606-	1	0.01
Delhi										ORF1a:I1607-	1	0.01
Delhi										ORF1a:K1608-	1	0.01
Delhi										ORF1a:S1612-	1	0.01
Delhi										ORF1a:H1613-	1	0.01
Delhi										ORF1a:E1614-	1	0.01

Delhi										ORF1a:G1615-	1	0.01
Delhi										ORF1a:K1616-	1	0.01
Delhi										ORF1a:T1617-	1	0.01
Delhi										ORF1a:F1618-	1	0.01
Delhi										ORF1a:Y1619-	1	0.01
Delhi										ORF1a:V1620-	1	0.01
Delhi										ORF1a:L1621-	1	0.01
Delhi										ORF1a:P1622-	1	0.01
Delhi										ORF1a:N1623-	1	0.01
Delhi										ORF1a:D1624-	1	0.01
Delhi										ORF1a:D1625-	1	0.01
Delhi										ORF1a:T1626-	1	0.01
Delhi										ORF1a:L1627-	1	0.01
Delhi										ORF1a:R1628-	1	0.01
Delhi										ORF1a:V1629-	1	0.01
Delhi										ORF1a:E1630-	1	0.01
Delhi										ORF1a:A1631-	1	0.01
Delhi										ORF1a:F1632-	1	0.01
Delhi										ORF1a:E1633-	1	0.01
Delhi										ORF1a:K1655-	1	0.01
Delhi										ORF1a:W1656-	1	0.01
Delhi										ORF1a:K1657-	1	0.01
Delhi										ORF1a:Y1658-	1	0.01
Delhi										ORF1a:P1659-	1	0.01
Delhi										ORF1a:Q1660-	1	0.01
Delhi										ORF1a:K1668-	1	0.01
Delhi										ORF1a:W1669-	1	0.01
Delhi										ORF1a:A1670-	1	0.01
Delhi										ORF1a:D1671-	1	0.01
Delhi										ORF1a:N1672-	1	0.01
Delhi										ORF1a:N1673-	1	0.01
Delhi										ORF1a:C1674-	1	0.01
Delhi										ORF1a:Y1675-	1	0.01
Delhi										ORF1a:L1676-	1	0.01
Delhi										ORF1a:A1677-	1	0.01
Delhi										ORF1a:T1678-	1	0.01
Delhi										ORF1a:A1679-	1	0.01
Delhi										ORF1a:E2112-	1	0.01
Delhi										ORF1a:L2113-	1	0.01
Delhi										ORF1a:S2114-	1	0.01
Delhi										ORF1a:L2234-	1	0.01
Delhi										ORF1a:L2235-	1	0.01
Delhi										ORF1a:L2236-	1	0.01
Delhi										ORF1a:R2267-	1	0.01
Delhi										ORF1a:E2268-	1	0.01
Delhi										ORF1a:G2269-	1	0.01
Delhi										ORF1a:Y2270-	1	0.01
Delhi										ORF1a:L2271-	1	0.01
Delhi										ORF1a:N2272-	1	0.01
Delhi										ORF1a:D2296-	1	0.01
Delhi										ORF1a:S2297-	1	0.01
Delhi										ORF1a:L2298-	1	0.01
Delhi										ORF1a:D2299-	1	0.01
Delhi										ORF1a:T2300-	1	0.01
Delhi										ORF1a:Y2301-	1	0.01
Delhi										ORF1a:P2302-	1	0.01
Delhi										ORF1a:S2303-	1	0.01
Delhi										ORF1a:L2304-	1	0.01
Delhi										ORF1a:E2305-	1	0.01
Delhi										ORF1a:T2306-	1	0.01
Delhi										ORF1a:I2307-	1	0.01
Delhi										ORF1a:Q2308-	1	0.01
Delhi										ORF1a:I2309-	1	0.01
Delhi										ORF1a:T2310-	1	0.01
Delhi										ORF1a:I2311-	1	0.01
Delhi										ORF1a:S2312-	1	0.01
Delhi										ORF1a:F2337-	1	0.01
Delhi										ORF1a:F2338-	1	0.01
Delhi										ORF1a:Y2339-	1	0.01
Delhi										ORF1b:F784-	1	0.01
Delhi										ORF1b:M785-	1	0.01
Delhi										ORF1b:S786-	1	0.01
Delhi										ORF1b:E787-	1	0.01

Delhi										ORF1b:A788-	1	0.01
Delhi										ORF1b:K789-	1	0.01
Delhi										ORF1b:C790-	1	0.01
Delhi										ORF1b:W791-	1	0.01
Delhi										ORF1b:T792-	1	0.01
Delhi										ORF1b:K798-	1	0.01
Delhi										ORF1b:G799-	1	0.01
Delhi										ORF1b:P800-	1	0.01
Delhi										ORF1b:H801-	1	0.01
Delhi										ORF1b:E802-	1	0.01
Delhi										ORF1b:F803-	1	0.01
Delhi										ORF1b:C804-	1	0.01
Delhi										ORF1b:S805-	1	0.01
Delhi										ORF1b:Q806-	1	0.01
Delhi										ORF1b:H807-	1	0.01
Delhi										ORF1b:T808-	1	0.01
Delhi										ORF1b:M809-	1	0.01
Delhi										ORF1b:G814-	1	0.01
Delhi										ORF1b:D815-	1	0.01
Delhi										ORF1b:D816-	1	0.01
Delhi										ORF1b:Y817-	1	0.01
Delhi										ORF1b:V818-	1	0.01
Delhi										ORF1b:Y819-	1	0.01
Delhi										ORF1b:L820-	1	0.01
Delhi										ORF1b:P821-	1	0.01
Delhi										ORF1b:Y822-	1	0.01
Delhi										ORF1b:P823-	1	0.01
Delhi										ORF1b:D824-	1	0.01
Delhi										ORF1b:P825-	1	0.01
Delhi										ORF1b:S826-	1	0.01
Delhi										ORF1b:R827-	1	0.01
Delhi										ORF1b:I828-	1	0.01
Delhi										ORF1b:L829-	1	0.01
Delhi										ORF1b:G830-	1	0.01
Delhi										ORF1b:A831-	1	0.01
Delhi										ORF1b:G832-	1	0.01
Delhi										ORF1b:C833-	1	0.01
Delhi										ORF1b:F834-	1	0.01
Delhi										ORF1b:V835-	1	0.01
Delhi										ORF1b:D836-	1	0.01
Delhi										ORF1b:D837-	1	0.01
Delhi										ORF1b:I847-	1	0.01
Delhi										ORF1b:E848-	1	0.01
Delhi										ORF1b:R849-	1	0.01
Delhi										ORF1b:F850-	1	0.01
Delhi										ORF1b:V851-	1	0.01
Delhi										ORF1b:S852-	1	0.01
Delhi										ORF1b:L853-	1	0.01
Delhi										ORF1b:A854-	1	0.01
Delhi										ORF1b:I855-	1	0.01
Delhi										ORF1b:D856-	1	0.01
Delhi										ORF1b:A857-	1	0.01
Delhi										ORF1b:Y858-	1	0.01
Delhi										ORF1b:P859-	1	0.01
Delhi										ORF1b:K862-	1	0.01
Delhi										ORF1b:H863-	1	0.01
Delhi										ORF1b:P864-	1	0.01
Delhi										ORF1b:N865-	1	0.01
Delhi										ORF1b:Q866-	1	0.01
Delhi										ORF1b:E867-	1	0.01
Delhi										ORF1b:Y868-	1	0.01
Delhi										ORF1b:A869-	1	0.01
Delhi										ORF1b:D870-	1	0.01
Delhi										ORF1b:V871-	1	0.01
Delhi										ORF1b:F872-	1	0.01
Delhi										ORF1b:L876-	1	0.01
Delhi										ORF1b:Q877-	1	0.01
Delhi										ORF1b:Y878-	1	0.01
Delhi										ORF1b:I879-	1	0.01
Delhi										ORF1b:R880-	1	0.01
Delhi										ORF1b:K881-	1	0.01
Delhi										ORF1b:L882-	1	0.01
Delhi										ORF1b:H883-	1	0.01

Delhi										ORF1b:D884-	1	0.01
Delhi										ORF1b:E885-	1	0.01
Delhi										ORF1b:L886-	1	0.01
Delhi										ORF1b:T887-	1	0.01
Delhi										ORF1b:G888-	1	0.01
Delhi										ORF1b:H889-	1	0.01
Delhi										ORF1b:M890-	1	0.01
Delhi										ORF1b:L891-	1	0.01
Delhi										ORF1b:D892-	1	0.01
Delhi										ORF1b:R905-	1	0.01
Delhi										ORF1b:Y906-	1	0.01
Delhi										ORF1b:W907-	1	0.01
Delhi										ORF1b:E908-	1	0.01
Delhi										ORF1b:P909-	1	0.01
Delhi										ORF1b:E910-	1	0.01
Delhi										ORF1b:F911-	1	0.01
Delhi										ORF1b:Y912-	1	0.01
Delhi										ORF1b:E913-	1	0.01
Delhi										ORF1b:A914-	1	0.01
Delhi										ORF1b:M915-	1	0.01
Delhi										ORF1b:Y916-	1	0.01
Delhi										ORF1b:T917-	1	0.01
Delhi										ORF1b:P918-	1	0.01
Delhi										ORF1b:H919-	1	0.01
Delhi										ORF1b:T920-	1	0.01
Delhi										ORF1b:V921-	1	0.01
Delhi										ORF1b:C1007-	1	0.01
Delhi										ORF1b:A1008-	1	0.01
Delhi										ORF1b:N1009-	1	0.01
Delhi										ORF1b:G1010-	1	0.01
Delhi										ORF1b:Q1011-	1	0.01
Delhi										ORF1b:G1014-	1	0.01
Delhi										ORF1b:L1015-	1	0.01
Delhi										ORF1b:Y1016-	1	0.01
Delhi										ORF1b:K1017-	1	0.01
Delhi										ORF1b:N1018-	1	0.01
Delhi										ORF1b:T1027-	1	0.01
Delhi										ORF1b:D1028-	1	0.01
Delhi										ORF1b:F1029-	1	0.01
Delhi										ORF1b:N1030-	1	0.01
Delhi										ORF1b:A1031-	1	0.01
Delhi										ORF1b:I1032-	1	0.01
Delhi										ORF1b:A1033-	1	0.01
Delhi										ORF1b:T1034-	1	0.01
Delhi										ORF1b:C1035-	1	0.01
Delhi										ORF1b:D1036-	1	0.01
Delhi										ORF1b:W1037-	1	0.01
Delhi										ORF1b:T1038-	1	0.01
Delhi										ORF1b:N1039-	1	0.01
Delhi										ORF1b:A1040-	1	0.01
Delhi										ORF1b:G1041-	1	0.01
Delhi										ORF1b:D1042-	1	0.01
Delhi										ORF1b:Y1043-	1	0.01
Delhi										ORF1b:I1044-	1	0.01
Delhi										ORF1b:L1045-	1	0.01
Delhi										ORF1b:A1046-	1	0.01
Delhi										ORF1b:N1047-	1	0.01
Delhi										ORF1b:T1048-	1	0.01
Delhi										ORF1b:C1049-	1	0.01
Delhi										ORF1b:F1056-	1	0.01
Delhi										ORF1b:A1057-	1	0.01
Delhi										ORF1b:A1058-	1	0.01
Delhi										ORF1b:E1059-	1	0.01
Delhi										ORF1b:T1060-	1	0.01
Delhi										ORF1b:L1061-	1	0.01
Delhi										ORF1b:K1062-	1	0.01
Delhi										ORF1b:A1063-	1	0.01
Delhi										ORF1b:T1064-	1	0.01
Delhi										ORF1b:E1065-	1	0.01
Delhi										ORF1b:E1066-	1	0.01
Delhi										ORF1b:T1067-	1	0.01
Delhi										ORF1b:F1068-	1	0.01
Delhi										ORF1b:K1069-	1	0.01

Delhi										ORF1b:L1070-	1	0.01
Delhi										ORF1b:S1071-	1	0.01
Delhi										ORF1b:Y1072-	1	0.01
Delhi										ORF1b:G1073-	1	0.01
Delhi										ORF1b:I1074-	1	0.01
Delhi										ORF1b:A1075-	1	0.01
Delhi										ORF1b:T1076-	1	0.01
Delhi										ORF1b:V1092-	1	0.01
Delhi										ORF1b:G1093-	1	0.01
Delhi										ORF1b:K1094-	1	0.01
Delhi										ORF1b:P1095-	1	0.01
Delhi										ORF1b:R1096-	1	0.01
Delhi										ORF1b:P1097-	1	0.01
Delhi										ORF1b:P1098-	1	0.01
Delhi										ORF1b:L1099-	1	0.01
Delhi										ORF1b:N1100-	1	0.01
Delhi										ORF1b:R1101-	1	0.01
Delhi										ORF1b:N1102-	1	0.01
Delhi										ORF1b:Y1103-	1	0.01
Delhi										ORF1b:V1104-	1	0.01
Delhi										ORF1b:F1105-	1	0.01
Delhi										ORF1b:T1106-	1	0.01
Delhi										ORF1b:G1107-	1	0.01
Delhi										ORF1b:Y1108-	1	0.01
Delhi										ORF1b:R1109-	1	0.01
Delhi										ORF1b:V1110-	1	0.01
Delhi										ORF1b:T1111-	1	0.01
Delhi										ORF1b:Q1117-	1	0.01
Delhi										ORF1b:I1118-	1	0.01
Delhi										ORF1b:G1119-	1	0.01
Delhi										ORF1b:E1120-	1	0.01
Delhi										ORF1b:Y1121-	1	0.01
Delhi										ORF1b:T1122-	1	0.01
Delhi										ORF1b:F1123-	1	0.01
Delhi										ORF1b:E1124-	1	0.01
Delhi										ORF1b:K1125-	1	0.01
Delhi										ORF1b:G1126-	1	0.01
Delhi										ORF1b:D1127-	1	0.01
Delhi										ORF1b:Y1128-	1	0.01
Delhi										ORF1b:G1129-	1	0.01
Delhi										ORF1b:D1130-	1	0.01
Delhi										ORF1b:A1131-	1	0.01
Delhi										ORF1b:V1132-	1	0.01
Delhi										ORF1b:V1133-	1	0.01
Delhi										ORF1b:Y1134-	1	0.01
Delhi										ORF1b:R1135-	1	0.01
Delhi										ORF1b:G1136-	1	0.01
Delhi										ORF1b:T1137-	1	0.01
Delhi										ORF1b:T1138-	1	0.01
Delhi										ORF1b:N1143-	1	0.01
Delhi										ORF1b:V1144-	1	0.01
Delhi										ORF1b:G1145-	1	0.01
Delhi										ORF1b:D1146-	1	0.01
Delhi										ORF1b:Y1147-	1	0.01
Delhi										ORF1b:F1148-	1	0.01
Delhi										ORF1b:V1149-	1	0.01
Delhi										ORF1b:L1150-	1	0.01
Delhi										ORF1b:T1151-	1	0.01
Delhi										ORF1b:S1152-	1	0.01
Delhi										ORF1b:H1153-	1	0.01
Delhi										ORF1b:T1154-	1	0.01
Delhi										ORF1b:A1160-	1	0.01
Delhi										ORF1b:P1161-	1	0.01
Delhi										ORF1b:T1162-	1	0.01
Delhi										ORF1b:L1163-	1	0.01
Delhi										ORF1b:V1164-	1	0.01
Delhi										ORF1b:P1165-	1	0.01
Delhi										ORF1b:Q1166-	1	0.01
Delhi										ORF1b:E1167-	1	0.01
Delhi										ORF1b:H1168-	1	0.01
Delhi										ORF1b:Y1169-	1	0.01
Delhi										ORF1b:V1170-	1	0.01
Delhi										ORF1b:R1171-	1	0.01

Delhi										ORF1b:I1172-	1	0.01
Delhi										ORF1b:D1183-	1	0.01
Delhi										ORF1b:E1184-	1	0.01
Delhi										ORF1b:F1185-	1	0.01
Delhi										ORF1b:S1186-	1	0.01
Delhi										ORF1b:S1187-	1	0.01
Delhi										ORF1b:N1188-	1	0.01
Delhi										ORF1b:V1189-	1	0.01
Delhi										ORF1b:A1190-	1	0.01
Delhi										ORF1b:N1191-	1	0.01
Delhi										ORF1b:Y1192-	1	0.01
Delhi										ORF1b:Q1193-	1	0.01
Delhi										ORF1b:K1194-	1	0.01
Delhi										ORF1b:V1195-	1	0.01
Delhi										ORF1b:G1205-	1	0.01
Delhi										ORF1b:P1206-	1	0.01
Delhi										ORF1b:P1207-	1	0.01
Delhi										ORF1b:G1208-	1	0.01
Delhi										ORF1b:T1209-	1	0.01
Delhi										ORF1b:G1210-	1	0.01
Delhi										ORF1b:K1211-	1	0.01
Delhi										ORF1b:S1212-	1	0.01
Delhi										ORF1b:H1213-	1	0.01
Delhi										ORF1b:F1214-	1	0.01
Delhi										ORF1b:A1215-	1	0.01
Delhi										ORF1b:I1216-	1	0.01
Delhi										ORF1b:G1217-	1	0.01
Delhi										ORF1b:A1225-	1	0.01
Delhi										ORF1b:R1226-	1	0.01
Delhi										ORF1b:I1227-	1	0.01
Delhi										ORF1b:V1228-	1	0.01
Delhi										ORF1b:Y1229-	1	0.01
Delhi										ORF1b:C1241-	1	0.01
Delhi										ORF1b:E1242-	1	0.01
Delhi										ORF1b:K1243-	1	0.01
Delhi										ORF1b:P1258-	1	0.01
Delhi										ORF1b:A1259-	1	0.01
Delhi										ORF1b:R1260-	1	0.01
Delhi										ORF1b:A1261-	1	0.01
Delhi										ORF1b:R1262-	1	0.01
Delhi										ORF1b:V1263-	1	0.01
Delhi										ORF1b:E1264-	1	0.01
Delhi										ORF1b:C1265-	1	0.01
Delhi										ORF1b:F1266-	1	0.01
Delhi										ORF1b:D1267-	1	0.01
Delhi										ORF1b:K1268-	1	0.01
Delhi										ORF1b:F1269-	1	0.01
Delhi										ORF1b:K1270-	1	0.01
Delhi										ORF1b:V1271-	1	0.01
Delhi										ORF1b:C1281-	1	0.01
Delhi										ORF1b:T1282-	1	0.01
Delhi										ORF1b:V1283-	1	0.01
Delhi										ORF1b:N1284-	1	0.01
Delhi										ORF1b:A1285-	1	0.01
Delhi										ORF1b:L1286-	1	0.01
Delhi										ORF1b:P1287-	1	0.01
Delhi										ORF1b:E1288-	1	0.01
Delhi										ORF1b:T1289-	1	0.01
Delhi										ORF1b:T1290-	1	0.01
Delhi										ORF1b:A1291-	1	0.01
Delhi										ORF1b:D1292-	1	0.01
Delhi										ORF1b:I1293-	1	0.01
Delhi										ORF1b:V1294-	1	0.01
Delhi										ORF1b:A1302-	1	0.01
Delhi										ORF1b:T1303-	1	0.01
Delhi										ORF1b:N1304-	1	0.01
Delhi										ORF1b:Y1305-	1	0.01
Delhi										ORF1b:D1306-	1	0.01
Delhi										ORF1b:L1307-	1	0.01
Delhi										ORF1b:S1308-	1	0.01
Delhi										ORF1b:V1309-	1	0.01
Delhi										ORF1b:V1310-	1	0.01
Delhi										ORF1b:N1311-	1	0.01

Delhi										**ORF1b:A1312-**	1	0.01
Delhi										**ORF1b:R1313-**	1	0.01
Delhi										**ORF1b:L1314-**	1	0.01
Delhi										**ORF1b:R1315-**	1	0.01
Delhi										**ORF1b:A1316-**	1	0.01
Delhi										**ORF1b:Y1321-**	1	0.01
Delhi										**ORF1b:I1322-**	1	0.01
Delhi										**ORF1b:G1323-**	1	0.01
Delhi										**ORF1b:D1324-**	1	0.01
Delhi										**ORF1b:P1325-**	1	0.01
Delhi										**ORF1b:A1326-**	1	0.01
Delhi										**ORF1b:Q1327-**	1	0.01
Delhi										**ORF1b:T1336-**	1	0.01
Delhi										**ORF1b:K1337-**	1	0.01
Delhi										**ORF1b:G1338-**	1	0.01
Delhi										**ORF1b:T1339-**	1	0.01
Delhi										**ORF1b:L1340-**	1	0.01
Delhi										**ORF1b:E1341-**	1	0.01
Delhi										**ORF1b:P1342-**	1	0.01
Delhi										**ORF1b:E1343-**	1	0.01
Delhi										**ORF1b:Y1344-**	1	0.01
Delhi										**ORF1b:F1345-**	1	0.01
Delhi										**ORF1b:N1346-**	1	0.01
Delhi										**ORF1b:S1347-**	1	0.01
Delhi										**ORF1b:V1348-**	1	0.01
Delhi										**ORF1b:C1349-**	1	0.01
Delhi										**ORF1b:I1355-**	1	0.01
Delhi										**ORF1b:G1356-**	1	0.01
Delhi										**ORF1b:P1357-**	1	0.01
Delhi										**ORF1b:D1358-**	1	0.01
Delhi										**ORF1b:M1359-**	1	0.01
Delhi										**ORF1b:F1360-**	1	0.01
Delhi										**ORF1b:L1361-**	1	0.01
Delhi										**ORF1b:G1362-**	1	0.01
Delhi										**ORF1b:T1363-**	1	0.01
Delhi										**ORF1b:C1364-**	1	0.01
Delhi										**ORF1b:R1365-**	1	0.01
Delhi										**ORF1b:R1366-**	1	0.01
Delhi										**ORF1b:D1389-**	1	0.01
Delhi										**ORF1b:K1390-**	1	0.01
Delhi										**ORF1b:S1391-**	1	0.01
Delhi										**ORF1b:A1392-**	1	0.01
Delhi										**ORF1b:Q1393-**	1	0.01
Delhi										**ORF1b:Y1399-**	1	0.01
Delhi										**ORF1b:K1400-**	1	0.01
Delhi										**ORF1b:G1401-**	1	0.01
Delhi										**ORF1b:V1402-**	1	0.01
Delhi										**ORF1b:I1403-**	1	0.01
Delhi										**ORF1b:T1404-**	1	0.01
Delhi										**ORF1b:H1405-**	1	0.01
Delhi										**ORF1b:D1406-**	1	0.01
Delhi										**ORF1b:V1407-**	1	0.01
Delhi										**ORF1b:S1408-**	1	0.01
Delhi										**ORF1b:S1409-**	1	0.01
Delhi										**ORF1b:A1410-**	1	0.01
Delhi										**ORF1b:I1411-**	1	0.01
Delhi										**ORF1b:N1412-**	1	0.01
Delhi										**ORF1b:R1413-**	1	0.01
Delhi										**ORF1b:P1414-**	1	0.01
Delhi										**ORF1b:Q1415-**	1	0.01
Delhi										**ORF1b:I1416-**	1	0.01
Delhi										**ORF1b:G1417-**	1	0.01
Delhi										**ORF1b:V1418-**	1	0.01
Delhi										**ORF1b:V1419-**	1	0.01
Delhi										**ORF1b:R1420-**	1	0.01
Delhi										**ORF1b:T1424-**	1	0.01
Delhi										**ORF1b:R1425-**	1	0.01
Delhi										**ORF1b:N1426-**	1	0.01
Delhi										**ORF1b:P1427-**	1	0.01
Delhi										**ORF1b:A1428-**	1	0.01
Delhi										**ORF1b:W1429-**	1	0.01
Delhi										**ORF1b:R1430-**	1	0.01
Delhi										**ORF1b:K1431-**	1	0.01

Delhi										ORF1b:A1432-	1	0.01
Delhi										ORF1b:V1433-	1	0.01
Delhi										ORF1b:F1434-	1	0.01
Delhi										ORF1b:I1435-	1	0.01
Delhi										ORF1b:S1436-	1	0.01
Delhi										ORF1b:P1437-	1	0.01
Delhi										ORF1b:Y1438-	1	0.01
Delhi										ORF1b:N1439-	1	0.01
Delhi										ORF1b:S1440-	1	0.01
Delhi										ORF1b:Q1441-	1	0.01
Delhi										ORF1b:N1442-	1	0.01
Delhi										ORF1b:A1443-	1	0.01
Delhi										ORF1b:V1444-	1	0.01
Delhi										ORF1b:A1445-	1	0.01
Delhi										ORF1b:G1450-	1	0.01
Delhi										ORF1b:L1451-	1	0.01
Delhi										ORF1b:P1452-	1	0.01
Delhi										ORF1b:T1453-	1	0.01
Delhi										ORF1b:Q1454-	1	0.01
Delhi										ORF1b:T1455-	1	0.01
Delhi										ORF1b:V1456-	1	0.01
Delhi										ORF1b:D1457-	1	0.01
Delhi										ORF1b:S1458-	1	0.01
Delhi										ORF1b:S1459-	1	0.01
Delhi										ORF1b:Q1460-	1	0.01
Delhi										ORF1b:G1461-	1	0.01
Delhi										ORF1b:S1462-	1	0.01
Delhi										ORF1b:E1463-	1	0.01
Delhi										ORF1b:Y1464-	1	0.01
Delhi										ORF1b:D1465-	1	0.01
Delhi										ORF1b:Y1466-	1	0.01
Delhi										ORF1b:V1467-	1	0.01
Delhi										ORF1b:E1474-	1	0.01
Delhi										ORF1b:T1475-	1	0.01
Delhi										ORF1b:A1476-	1	0.01
Delhi										ORF1b:H1477-	1	0.01
Delhi										ORF1b:S1478-	1	0.01
Delhi										ORF1b:C1479-	1	0.01
Delhi										ORF1b:N1480-	1	0.01
Delhi										ORF1b:V1481-	1	0.01
Delhi										ORF1b:N1482-	1	0.01
Delhi										ORF1b:R1483-	1	0.01
Delhi										ORF1b:F1484-	1	0.01
Delhi										ORF1b:S1500-	1	0.01
Delhi										ORF1b:D1501-	1	0.01
Delhi										ORF1b:R1502-	1	0.01
Delhi										ORF1b:D1503-	1	0.01
Delhi										ORF1b:L1504-	1	0.01
Delhi										ORF1b:Y1505-	1	0.01
Delhi										ORF1b:D1506-	1	0.01
Delhi										ORF1b:K1507-	1	0.01
Delhi										ORF1b:L1508-	1	0.01
Delhi										ORF1b:Q1509-	1	0.01
Delhi										ORF1b:F1510-	1	0.01
Delhi										ORF1b:E1514-	1	0.01
Delhi										ORF1b:I1515-	1	0.01
Delhi										ORF1b:P1516-	1	0.01
Delhi										ORF1b:R1517-	1	0.01
Delhi										ORF1b:R1518-	1	0.01
Delhi										ORF1b:N1519-	1	0.01
Delhi										ORF1b:V1520-	1	0.01
Delhi										ORF1b:A1521-	1	0.01
Delhi										ORF1b:T1522-	1	0.01
Delhi										ORF1b:L1523-	1	0.01
Delhi										ORF1b:Q1524-	1	0.01
Delhi										ORF1b:A1525-	1	0.01
Delhi										ORF1b:E1526-	1	0.01
Delhi										ORF1b:N1527-	1	0.01
Delhi										ORF1b:V1528-	1	0.01
Delhi										ORF1b:T1529-	1	0.01
Delhi										ORF1b:G1530-	1	0.01
Delhi										ORF1b:L1531-	1	0.01
Delhi										ORF1b:F1532-	1	0.01

Delhi										ORF1b:K1533-	1	0.01
Delhi										ORF1b:D1534-	1	0.01
Delhi										ORF1b:C1535-	1	0.01
Delhi										ORF1b:P1544-	1	0.01
Delhi										ORF1b:T1545-	1	0.01
Delhi										ORF1b:Q1546-	1	0.01
Delhi										ORF1b:A1547-	1	0.01
Delhi										ORF1b:P1548-	1	0.01
Delhi										ORF1b:C1563-	1	0.01
Delhi										ORF1b:V1564-	1	0.01
Delhi										ORF1b:D1565-	1	0.01
Delhi										ORF1b:I1566-	1	0.01
Delhi										ORF1b:P1567-	1	0.01
Delhi										ORF1b:G1568-	1	0.01
Delhi										ORF1b:I1569-	1	0.01
Delhi										ORF1b:P1570-	1	0.01
Delhi										ORF1b:K1571-	1	0.01
Delhi										ORF1b:D1572-	1	0.01
Delhi										ORF1b:M1573-	1	0.01
Delhi										ORF1b:G1583-	1	0.01
Delhi										ORF1b:F1584-	1	0.01
Delhi										ORF1b:K1585-	1	0.01
Delhi										ORF1b:M1586-	1	0.01
Delhi										ORF1b:N1587-	1	0.01
Delhi										ORF1b:Y1588-	1	0.01
Delhi										ORF1b:Q1589-	1	0.01
Delhi										ORF1b:V1590-	1	0.01
Delhi										ORF1b:N1591-	1	0.01
Delhi										ORF1b:G1592-	1	0.01
Delhi										ORF1b:Y1593-	1	0.01
Delhi										ORF1b:P1594-	1	0.01
Delhi										ORF1b:N1595-	1	0.01
Delhi										ORF1b:M1596-	1	0.01
Delhi										ORF1b:F1597-	1	0.01
Delhi										ORF1b:I1598-	1	0.01
Delhi										ORF1b:T1599-	1	0.01
Delhi										ORF1b:R1600-	1	0.01
Delhi										ORF1b:E1601-	1	0.01
Delhi										ORF1b:E1602-	1	0.01
Delhi										ORF1b:A1603-	1	0.01
Delhi										ORF1b:I1604-	1	0.01
Delhi										ORF1b:R1605-	1	0.01
Delhi										ORF1b:A1609-	1	0.01
Delhi										ORF1b:W1610-	1	0.01
Delhi										ORF1b:I1611-	1	0.01
Delhi										ORF1b:G1612-	1	0.01
Delhi										ORF1b:F1613-	1	0.01
Delhi										ORF1b:D1614-	1	0.01
Delhi										ORF1b:V1615-	1	0.01
Delhi										ORF1b:E1616-	1	0.01
Delhi										ORF1b:G1617-	1	0.01
Delhi										ORF1b:C1618-	1	0.01
Delhi										ORF1b:H1619-	1	0.01
Delhi										ORF1b:A1620-	1	0.01
Delhi										ORF1b:T1621-	1	0.01
Delhi										ORF1b:R1622-	1	0.01
Delhi										ORF1b:E1623-	1	0.01
Delhi										ORF1b:A1624-	1	0.01
Delhi										ORF1b:V1625-	1	0.01
Delhi										ORF1b:L1633-	1	0.01
Delhi										ORF1b:G1634-	1	0.01
Delhi										ORF1b:F1635-	1	0.01
Delhi										ORF1b:S1636-	1	0.01
Delhi										ORF1b:T1637-	1	0.01
Delhi										ORF1b:G1638-	1	0.01
Delhi										ORF1b:V1639-	1	0.01
Delhi										ORF1b:N1640-	1	0.01
Delhi										ORF1b:L1641-	1	0.01
Delhi										ORF1b:V1642-	1	0.01
Delhi										ORF1b:A1643-	1	0.01
Delhi										ORF1b:V1644-	1	0.01
Delhi										ORF1b:P1645-	1	0.01
Delhi										ORF1b:T1646-	1	0.01

Delhi										ORF1b:G1647-	1	0.01
Delhi										ORF1b:Y1648-	1	0.01
Delhi										ORF1b:N1653-	1	0.01
Delhi										ORF1b:N1654-	1	0.01
Delhi										ORF1b:T1655-	1	0.01
Delhi										ORF1b:D1656-	1	0.01
Delhi										ORF1b:F1657-	1	0.01
Delhi										ORF1b:S1658-	1	0.01
Delhi										ORF1b:R1659-	1	0.01
Delhi										ORF1b:V1660-	1	0.01
Delhi										ORF1b:S1661-	1	0.01
Delhi										ORF1b:A1662-	1	0.01
Delhi										ORF1b:K1663-	1	0.01
Delhi										ORF1b:P1664-	1	0.01
Delhi										ORF1b:P1665-	1	0.01
Delhi										ORF1b:P1666-	1	0.01
Delhi										ORF1b:G1667-	1	0.01
Delhi										ORF1b:D1668-	1	0.01
Delhi										ORF1b:Q1669-	1	0.01
Delhi										ORF1b:F1670-	1	0.01
Delhi										ORF1b:K1671-	1	0.01
Delhi										ORF1b:M1677-	1	0.01
Delhi										ORF1b:Y1678-	1	0.01
Delhi										ORF1b:K1679-	1	0.01
Delhi										ORF1b:G1680-	1	0.01
Delhi										ORF1b:L1681-	1	0.01
Delhi										ORF1b:P1682-	1	0.01
Delhi										ORF1b:W1683-	1	0.01
Delhi										ORF1b:V1691-	1	0.01
Delhi										ORF1b:Q1692-	1	0.01
Delhi										ORF1b:M1693-	1	0.01
Delhi										ORF1b:L1694-	1	0.01
Delhi										ORF1b:S1695-	1	0.01
Delhi										ORF1b:D1696-	1	0.01
Delhi										ORF1b:T1697-	1	0.01
Delhi										ORF1b:L1698-	1	0.01
Delhi										ORF1b:K1699-	1	0.01
Delhi										ORF1b:N1700-	1	0.01
Delhi										ORF1b:L1701-	1	0.01
Delhi										ORF1b:S1702-	1	0.01
Delhi										ORF1b:D1703-	1	0.01
Delhi										ORF1b:R1704-	1	0.01
Delhi										ORF1b:V1705-	1	0.01
Delhi										ORF1b:V1706-	1	0.01
Delhi										ORF1b:F1707-	1	0.01
Delhi										ORF1b:V1708-	1	0.01
Delhi										ORF1b:L1709-	1	0.01
Delhi										ORF1b:W1710-	1	0.01
Delhi										ORF1b:A1711-	1	0.01
Delhi										ORF1b:H1712-	1	0.01
Delhi										ORF1b:G1713-	1	0.01
Delhi										ORF1b:F1714-	1	0.01
Delhi										ORF1b:G1726-	1	0.01
Delhi										ORF1b:P1727-	1	0.01
Delhi										ORF1b:E1728-	1	0.01
Delhi										ORF1b:R1729-	1	0.01
Delhi										ORF1b:T1730-	1	0.01
Delhi										ORF1b:C1731-	1	0.01
Delhi										ORF1b:C1732-	1	0.01
Delhi										ORF1b:L1733-	1	0.01
Delhi										ORF1b:C1734-	1	0.01
Delhi										ORF1b:D1735-	1	0.01
Delhi										ORF1b:R1736-	1	0.01
Delhi										ORF1b:R1737-	1	0.01
Delhi										ORF1b:A1738-	1	0.01
Delhi										ORF1b:T1739-	1	0.01
Delhi										ORF1b:C1740-	1	0.01
Delhi										ORF1b:F1741-	1	0.01
Delhi										ORF1b:S1742-	1	0.01
Delhi										ORF1b:T1743-	1	0.01
Delhi										ORF1b:A1744-	1	0.01
Delhi										ORF1b:S1745-	1	0.01
Delhi										ORF1b:D1746-	1	0.01

Delhi										ORF1b:T1747-	1	0.01
Delhi										ORF1b:Y1748-	1	0.01
Delhi										ORF1b:A1749-	1	0.01
Delhi										ORF1b:G1756-	1	0.01
Delhi										ORF1b:F1757-	1	0.01
Delhi										ORF1b:D1758-	1	0.01
Delhi										ORF1b:Y1759-	1	0.01
Delhi										ORF1b:V1760-	1	0.01
Delhi										ORF1b:Y1761-	1	0.01
Delhi										ORF1b:N1762-	1	0.01
Delhi										ORF1b:P1763-	1	0.01
Delhi										ORF1b:F1764-	1	0.01
Delhi										ORF1b:M1765-	1	0.01
Delhi										ORF1b:N1780-	1	0.01
Delhi										ORF1b:H1781-	1	0.01
Delhi										ORF1b:D1782-	1	0.01
Delhi										ORF1b:L1783-	1	0.01
Delhi										ORF1b:Y1784-	1	0.01
Delhi										ORF1b:C1785-	1	0.01
Delhi										ORF1b:Q1786-	1	0.01
Delhi										ORF1b:V1787-	1	0.01
Delhi										ORF1b:H1788-	1	0.01
Delhi										ORF1b:G1789-	1	0.01
Delhi										ORF1b:N1790-	1	0.01
Delhi										ORF1b:A1791-	1	0.01
Delhi										ORF1b:H1792-	1	0.01
Delhi										ORF1b:V1793-	1	0.01
Delhi										ORF1b:A1794-	1	0.01
Delhi										ORF1b:S1795-	1	0.01
Delhi										ORF1b:C1796-	1	0.01
Delhi										ORF1b:D1797-	1	0.01
Delhi										ORF1b:H1807-	1	0.01
Delhi										ORF1b:E1808-	1	0.01
Delhi										ORF1b:C1809-	1	0.01
Delhi										ORF1b:F1810-	1	0.01
Delhi										ORF1b:V1811-	1	0.01
Delhi										ORF1b:K1812-	1	0.01
Delhi										ORF1b:R1813-	1	0.01
Delhi										ORF1b:V1814-	1	0.01
Delhi										ORF1b:D1815-	1	0.01
Delhi										ORF1b:W1816-	1	0.01
Delhi										ORF1b:T1817-	1	0.01
Delhi										ORF1b:I1818-	1	0.01
Delhi										ORF1b:E1819-	1	0.01
Delhi										ORF1b:Y1820-	1	0.01
Delhi										ORF1b:P1821-	1	0.01
Delhi										ORF1b:I1822-	1	0.01
Delhi										ORF1b:I1823-	1	0.01
Delhi										ORF1b:G1824-	1	0.01
Delhi										ORF1b:D1825-	1	0.01
Delhi										ORF1b:V1836-	1	0.01
Delhi										ORF1b:Q1837-	1	0.01
Delhi										ORF1b:H1838-	1	0.01
Delhi										ORF1b:M1839-	1	0.01
Delhi										ORF1b:V1840-	1	0.01
Delhi										ORF1b:V1841-	1	0.01
Delhi										ORF1b:K1842-	1	0.01
Delhi										ORF1b:A1843-	1	0.01
Delhi										ORF1b:A1844-	1	0.01
Delhi										ORF1b:L1845-	1	0.01
Delhi										ORF1b:L1846-	1	0.01
Delhi										ORF1b:A1847-	1	0.01
Delhi										ORF1b:D1848-	1	0.01
Delhi										ORF1b:K1849-	1	0.01
Delhi										ORF1b:F1850-	1	0.01
Delhi										ORF1b:P1851-	1	0.01
Delhi										ORF1b:V1852-	1	0.01
Delhi										ORF1b:D1855-	1	0.01
Delhi										ORF1b:I1856-	1	0.01
Delhi										ORF1b:G1857-	1	0.01
Delhi										ORF1b:N1858-	1	0.01
Delhi										ORF1b:P1859-	1	0.01
Delhi										ORF1b:K1860-	1	0.01

Delhi										**ORF1b:A1861-**	1	0.01
Delhi										**ORF1b:I1862-**	1	0.01
Delhi										**ORF1b:K1863-**	1	0.01
Delhi										**ORF1b:C1864-**	1	0.01
Delhi										**ORF1b:V1865-**	1	0.01
Delhi										**ORF1b:P1866-**	1	0.01
Delhi										**ORF1b:Q1867-**	1	0.01
Delhi										**ORF1b:A1868-**	1	0.01
Delhi										**ORF1b:D1869-**	1	0.01
Delhi										**ORF1b:F1874-**	1	0.01
Delhi										**ORF1b:Y1875-**	1	0.01
Delhi										**ORF1b:D1876-**	1	0.01
Delhi										**ORF1b:A1877-**	1	0.01
Delhi										**ORF1b:Q1878-**	1	0.01
Delhi										**ORF1b:P1879-**	1	0.01
Delhi										**ORF1b:C1880-**	1	0.01
Delhi										**ORF1b:S1881-**	1	0.01
Delhi										**ORF1b:E1888-**	1	0.01
Delhi										**ORF1b:E1889-**	1	0.01
Delhi										**ORF1b:L1890-**	1	0.01
Delhi										**ORF1b:F1891-**	1	0.01
Delhi										**ORF1b:Y1892-**	1	0.01
Delhi										**ORF1b:S1893-**	1	0.01
Delhi										**ORF1b:Y1894-**	1	0.01
Delhi										**ORF1b:A1895-**	1	0.01
Delhi										**ORF1b:T1896-**	1	0.01
Delhi										**ORF1b:H1897-**	1	0.01
Delhi										**ORF1b:S1898-**	1	0.01
Delhi										**ORF1b:D1899-**	1	0.01
Delhi										**ORF1b:K1900-**	1	0.01
Delhi										**ORF1b:F1901-**	1	0.01
Delhi										**ORF1b:T1902-**	1	0.01
Delhi										**ORF1b:D1903-**	1	0.01
Delhi										**ORF1b:G1904-**	1	0.01
Delhi										**ORF1b:V1905-**	1	0.01
Delhi										**ORF1b:C1906-**	1	0.01
Delhi										**ORF1b:W1909-**	1	0.01
Delhi										**ORF1b:N1910-**	1	0.01
Delhi										**ORF1b:C1911-**	1	0.01
Delhi										**ORF1b:N1912-**	1	0.01
Delhi										**ORF1b:V1913-**	1	0.01
Delhi										**ORF1b:D1914-**	1	0.01
Delhi										**ORF1b:R1915-**	1	0.01
Delhi										**ORF1b:R1924-**	1	0.01
Delhi										**ORF1b:F1925-**	1	0.01
Delhi										**ORF1b:D1926-**	1	0.01
Delhi										**ORF1b:T1927-**	1	0.01
Delhi										**ORF1b:R1928-**	1	0.01
Delhi										**ORF1b:V1929-**	1	0.01
Delhi										**ORF1b:L1930-**	1	0.01
Delhi										**ORF1b:S1931-**	1	0.01
Delhi										**ORF1b:N1932-**	1	0.01
Delhi										**ORF1b:L1933-**	1	0.01
Delhi										**ORF1b:N1934-**	1	0.01
Delhi										**ORF1b:L1935-**	1	0.01
Delhi										**ORF1b:P1936-**	1	0.01
Delhi										**ORF1b:G1937-**	1	0.01
Delhi										**ORF1b:C1938-**	1	0.01
Delhi										**ORF1b:D1939-**	1	0.01
Delhi										**ORF1b:G1940-**	1	0.01
Delhi										**ORF1b:G1941-**	1	0.01
Delhi										**ORF1b:S1942-**	1	0.01
Delhi										**ORF1b:L1943-**	1	0.01
Delhi										**S:L54-**	1	0.01
Delhi										**ORF1a:I2786-**	1	0.01
Delhi										**ORF1a:F2787-**	1	0.01
Delhi										**ORF1a:F2802-**	1	0.01
Delhi										**ORF1a:S2803-**	1	0.01
Delhi										**ORF1a:S2804-**	1	0.01
Delhi										**ORF1a:E2805-**	1	0.01
Delhi										**ORF1a:I2806-**	1	0.01
Delhi										**ORF1a:I2807-**	1	0.01
Delhi										**ORF1a:G2808-**	1	0.01

Delhi										ORF1a:Y2809-	1	0.01
Delhi										ORF1a:K2810-	1	0.01
Delhi										ORF1a:A2811-	1	0.01
Delhi										ORF1a:I2812-	1	0.01
Delhi										ORF1a:D2813-	1	0.01
Delhi										ORF1a:G2814-	1	0.01
Delhi										ORF1a:G2815-	1	0.01
Delhi										ORF1a:V2816-	1	0.01
Delhi										ORF1a:C2826-	1	0.01
Delhi										ORF1a:F2827-	1	0.01
Delhi										ORF1a:A2828-	1	0.01
Delhi										ORF1a:N2829-	1	0.01
Delhi										ORF1a:K2830-	1	0.01
Delhi										ORF1a:H2831-	1	0.01
Delhi										ORF1a:A2832-	1	0.01
Delhi										ORF1a:D2833-	1	0.01
Delhi										ORF1a:F2834-	1	0.01
Delhi										ORF1a:D2835-	1	0.01
Delhi										ORF1a:T2836-	1	0.01
Delhi										ORF1a:W2837-	1	0.01
Delhi										ORF1a:F2838-	1	0.01
Delhi										ORF1a:S2839-	1	0.01
Delhi										ORF1a:Q2840-	1	0.01
Delhi										ORF1a:I2858-	1	0.01
Delhi										ORF1a:T2859-	1	0.01
Delhi										ORF1a:R2860-	1	0.01
Delhi										ORF1a:E2861-	1	0.01
Delhi										ORF1a:V2862-	1	0.01
Delhi										ORF1a:G2863-	1	0.01
Delhi										ORF1a:F2864-	1	0.01
Delhi										ORF1a:V2865-	1	0.01
Delhi										ORF1a:V2866-	1	0.01
Delhi										ORF1a:P2867-	1	0.01
Delhi										ORF1a:G2868-	1	0.01
Delhi										ORF1a:L2869-	1	0.01
Delhi										ORF1a:P2870-	1	0.01
Delhi										ORF1a:G2871-	1	0.01
Delhi										ORF1a:T2872-	1	0.01
Delhi										ORF1a:I2873-	1	0.01
Delhi										ORF1a:I2903-	1	0.01
Delhi										ORF1a:E2904-	1	0.01
Delhi										ORF1a:Y2905-	1	0.01
Delhi										ORF1a:T2906-	1	0.01
Delhi										ORF1a:D2907-	1	0.01
Delhi										ORF1a:F2908-	1	0.01
Delhi										ORF1a:A2909-	1	0.01
Delhi										ORF1a:A2916-	1	0.01
Delhi										ORF1a:A2917-	1	0.01
Delhi										ORF1a:E2918-	1	0.01
Delhi										ORF1a:C2919-	1	0.01
Delhi										ORF1a:T2920-	1	0.01
Delhi										ORF1a:I2921-	1	0.01
Delhi										ORF1a:F2922-	1	0.01
Delhi										ORF1a:K2923-	1	0.01
Delhi										ORF1a:D2924-	1	0.01
Delhi										ORF1a:A2925-	1	0.01
Delhi										ORF1a:S2926-	1	0.01
Delhi										ORF1a:G2927-	1	0.01
Delhi										ORF1a:K2928-	1	0.01
Delhi										ORF1a:P2929-	1	0.01
Delhi										ORF1a:V2930-	1	0.01
Delhi										ORF1a:P2931-	1	0.01
Delhi										ORF1a:Y2932-	1	0.01
Delhi										ORF1a:C2933-	1	0.01
Delhi										ORF1a:Y2934-	1	0.01
Delhi										ORF1a:D2935-	1	0.01
Delhi										ORF1a:A2944-	1	0.01
Delhi										ORF1a:Y2945-	1	0.01
Delhi										ORF1a:E2946-	1	0.01
Delhi										ORF1a:S2947-	1	0.01
Delhi										ORF1a:L2948-	1	0.01
Delhi										ORF1a:R2949-	1	0.01
Delhi										ORF1a:P2950-	1	0.01

Delhi										ORF1a:D2951-	1	0.01
Delhi										ORF1a:T2952-	1	0.01
Delhi										ORF1a:R2953-	1	0.01
Delhi										ORF1a:M2957-	1	0.01
Delhi										ORF1a:D2958-	1	0.01
Delhi										ORF1a:G2959-	1	0.01
Delhi										ORF1a:S2960-	1	0.01
Delhi										ORF1a:I2961-	1	0.01
Delhi										ORF1a:I2962-	1	0.01
Delhi										ORF1a:Q2963-	1	0.01
Delhi										ORF1a:F2964-	1	0.01
Delhi										ORF1a:P2965-	1	0.01
Delhi										ORF1a:N2966-	1	0.01
Delhi										ORF1a:T2967-	1	0.01
Delhi										ORF1a:Y2968-	1	0.01
Delhi										ORF1a:F2979-	1	0.01
Delhi										ORF1a:D2980-	1	0.01
Delhi										ORF1a:S2981-	1	0.01
Delhi										ORF1a:E2982-	1	0.01
Delhi										ORF1a:Y2983-	1	0.01
Delhi										ORF1a:C2984-	1	0.01
Delhi										ORF1a:R2985-	1	0.01
Delhi										ORF1a:H2986-	1	0.01
Delhi										ORF1a:G2987-	1	0.01
Delhi										ORF1a:T2988-	1	0.01
Delhi										ORF1a:C2989-	1	0.01
Delhi										ORF1a:E2990-	1	0.01
Delhi										ORF1a:R2991-	1	0.01
Delhi										ORF1a:S2992-	1	0.01
Delhi										ORF1a:E2993-	1	0.01
Delhi										ORF1a:S2999-	1	0.01
Delhi										ORF1a:T3000-	1	0.01
Delhi										ORF1a:S3001-	1	0.01
Delhi										ORF1a:G3002-	1	0.01
Delhi										ORF1a:R3003-	1	0.01
Delhi										ORF1a:W3004-	1	0.01
Delhi										ORF1a:V3005-	1	0.01
Delhi										ORF1a:L3006-	1	0.01
Delhi										ORF1a:N3007-	1	0.01
Delhi										ORF1a:N3008-	1	0.01
Delhi										ORF1a:P3015-	1	0.01
Delhi										ORF1a:G3016-	1	0.01
Delhi										ORF1a:V3017-	1	0.01
Delhi										ORF1a:F3018-	1	0.01
Delhi										ORF1a:C3019-	1	0.01
Delhi										ORF1a:G3020-	1	0.01
Delhi										ORF1a:V3021-	1	0.01
Delhi										ORF1a:D3022-	1	0.01
Delhi										ORF1a:A3023-	1	0.01
Delhi										ORF1a:V3024-	1	0.01
Delhi										ORF1a:N3025-	1	0.01
Delhi										ORF1a:L3026-	1	0.01
Delhi										ORF1a:L3027-	1	0.01
Delhi										ORF1a:T3028-	1	0.01
Delhi										ORF1a:N3029-	1	0.01
Delhi										ORF1a:D3042-	1	0.01
Delhi										ORF1a:I3043-	1	0.01
Delhi										ORF1a:S3044-	1	0.01
Delhi										ORF1a:A3045-	1	0.01
Delhi										ORF1a:S3046-	1	0.01
Delhi										ORF1a:I3047-	1	0.01
Delhi										ORF1a:V3048-	1	0.01
Delhi										ORF1a:A3049-	1	0.01
Delhi										ORF1a:G3050-	1	0.01
Delhi										ORF1a:G3051-	1	0.01
Delhi										ORF1a:I3052-	1	0.01
Delhi										ORF1a:V3053-	1	0.01
Delhi										ORF1a:A3054-	1	0.01
Delhi										ORF1a:I3055-	1	0.01
Delhi										ORF1a:A3061-	1	0.01
Delhi										ORF1a:Y3062-	1	0.01
Delhi										ORF1a:Y3063-	1	0.01
Delhi										ORF1a:F3064-	1	0.01

Delhi										ORF1a:M3065-	1	0.01
Delhi										ORF1a:R3066-	1	0.01
Delhi										ORF1a:F3067-	1	0.01
Delhi										ORF1a:R3068-	1	0.01
Delhi										ORF1a:R3069-	1	0.01
Delhi										ORF1a:A3070-	1	0.01
Delhi										ORF1a:T3117-	1	0.01
Delhi										ORF1a:N3118-	1	0.01
Delhi										ORF1a:D3119-	1	0.01
Delhi										ORF1a:V3120-	1	0.01
Delhi										ORF1a:S3121-	1	0.01
Delhi										ORF1a:F3122-	1	0.01
Delhi										ORF1a:L3123-	1	0.01
Delhi										ORF1a:A3124-	1	0.01
Delhi										ORF1a:H3125-	1	0.01
Delhi										ORF1a:I3126-	1	0.01
Delhi										ORF1a:V3136-	1	0.01
Delhi										ORF1a:P3137-	1	0.01
Delhi										ORF1a:F3138-	1	0.01
Delhi										ORF1a:W3139-	1	0.01
Delhi										ORF1a:I3140-	1	0.01
Delhi										ORF1a:T3141-	1	0.01
Delhi										ORF1a:I3142-	1	0.01
Delhi										ORF1a:A3143-	1	0.01
Delhi										ORF1a:Y3144-	1	0.01
Delhi										ORF1a:I3145-	1	0.01
Delhi										ORF1a:I3146-	1	0.01
Delhi										ORF1a:C3147-	1	0.01
Delhi										ORF1a:T3150-	1	0.01
Delhi										ORF1a:K3151-	1	0.01
Delhi										ORF1a:H3152-	1	0.01
Delhi										ORF1a:S3171-	1	0.01
Delhi										ORF1a:F3172-	1	0.01
Delhi										ORF1a:S3173-	1	0.01
Delhi										ORF1a:T3174-	1	0.01
Delhi										ORF1a:F3175-	1	0.01
Delhi										ORF1a:E3176-	1	0.01
Delhi										ORF1a:E3177-	1	0.01
Delhi										ORF1a:A3178-	1	0.01
Delhi										ORF1a:A3179-	1	0.01
Delhi										ORF1a:L3180-	1	0.01
Delhi										ORF1a:C3181-	1	0.01
Delhi										ORF1a:T3182-	1	0.01
Delhi										ORF1a:F3183-	1	0.01
Delhi										ORF1a:K3192-	1	0.01
Delhi										ORF1a:L3193-	1	0.01
Delhi										ORF1a:R3194-	1	0.01
Delhi										ORF1a:S3195-	1	0.01
Delhi										ORF1a:D3196-	1	0.01
Delhi										ORF1a:V3197-	1	0.01
Delhi										ORF1a:L3198-	1	0.01
Delhi										ORF1a:L3199-	1	0.01
Delhi										ORF1a:P3200-	1	0.01
Delhi										ORF1a:L3201-	1	0.01
Delhi										ORF1a:T3202-	1	0.01
Delhi										ORF1a:Q3203-	1	0.01
Delhi										ORF1a:Y3204-	1	0.01
Delhi										ORF1a:N3205-	1	0.01
Delhi										ORF1a:R3206-	1	0.01
Delhi										ORF1a:Y3207-	1	0.01
Delhi										ORF1a:L3208-	1	0.01
Delhi										ORF1a:A3209-	1	0.01
Delhi										ORF1a:K3213-	1	0.01
Delhi										ORF1a:Y3214-	1	0.01
Delhi										ORF1a:K3215-	1	0.01
Delhi										ORF1a:Y3216-	1	0.01
Delhi										ORF1a:F3217-	1	0.01
Delhi										ORF1a:S3218-	1	0.01
Delhi										ORF1a:G3219-	1	0.01
Delhi										ORF1a:A3220-	1	0.01
Delhi										ORF1a:M3221-	1	0.01
Delhi										ORF1a:D3222-	1	0.01
Delhi										ORF1a:T3223-	1	0.01

Delhi										ORF1a:T3255-	1	0.01
Delhi										ORF1a:S3256-	1	0.01
Delhi										ORF1a:I3257-	1	0.01
Delhi										ORF1a:T3258-	1	0.01
Delhi										ORF1a:S3259-	1	0.01
Delhi										ORF1a:A3260-	1	0.01
Delhi										ORF1a:V3261-	1	0.01
Delhi										ORF1a:L3262-	1	0.01
Delhi										ORF1a:Q3263-	1	0.01
Delhi										ORF1a:S3264-	1	0.01
Delhi										ORF1a:G3265-	1	0.01
Delhi										ORF1a:F3266-	1	0.01
Delhi										ORF1a:R3267-	1	0.01
Delhi										ORF1a:K3268-	1	0.01
Delhi										ORF1a:M3269-	1	0.01
Delhi										ORF1a:W3294-	1	0.01
Delhi										ORF1a:L3295-	1	0.01
Delhi										ORF1a:D3296-	1	0.01
Delhi										ORF1a:D3297-	1	0.01
Delhi										ORF1a:V3298-	1	0.01
Delhi										ORF1a:V3299-	1	0.01
Delhi										ORF1a:Y3300-	1	0.01
Delhi										ORF1a:C3301-	1	0.01
Delhi										ORF1a:P3302-	1	0.01
Delhi										ORF1a:R3303-	1	0.01
Delhi										ORF1a:N3314-	1	0.01
Delhi										ORF1a:P3315-	1	0.01
Delhi										ORF1a:N3316-	1	0.01
Delhi										ORF1a:Y3317-	1	0.01
Delhi										ORF1a:E3318-	1	0.01
Delhi										ORF1a:D3319-	1	0.01
Delhi										ORF1a:Q3332-	1	0.01
Delhi										ORF1a:A3333-	1	0.01
Delhi										ORF1a:G3334-	1	0.01
Delhi										ORF1a:N3335-	1	0.01
Delhi										ORF1a:V3336-	1	0.01
Delhi										ORF1a:Q3337-	1	0.01
Delhi										ORF1a:L3338-	1	0.01
Delhi										ORF1a:R3339-	1	0.01
Delhi										ORF1a:V3340-	1	0.01
Delhi										ORF1a:I3341-	1	0.01
Delhi										ORF1a:G3342-	1	0.01
Delhi										ORF1a:H3343-	1	0.01
Delhi										ORF1a:S3344-	1	0.01
Delhi										ORF1a:K3353-	1	0.01
Delhi										ORF1a:V3354-	1	0.01
Delhi										ORF1a:D3355-	1	0.01
Delhi										ORF1a:T3356-	1	0.01
Delhi										ORF1a:A3357-	1	0.01
Delhi										ORF1a:N3358-	1	0.01
Delhi										ORF1a:P3359-	1	0.01
Delhi										ORF1a:K3360-	1	0.01
Delhi										ORF1a:T3361-	1	0.01
Delhi										ORF1a:P3362-	1	0.01
Delhi										ORF1a:K3363-	1	0.01
Delhi										ORF1a:Y3364-	1	0.01
Delhi										ORF1a:K3365-	1	0.01
Delhi										ORF1a:F3366-	1	0.01
Delhi										ORF1a:V3367-	1	0.01
Delhi										ORF1a:A3379-	1	0.01
Delhi										ORF1a:C3380-	1	0.01
Delhi										ORF1a:Y3381-	1	0.01
Delhi										ORF1a:N3382-	1	0.01
Delhi										ORF1a:G3383-	1	0.01
Delhi										ORF1a:S3384-	1	0.01
Delhi										ORF1a:P3385-	1	0.01
Delhi										ORF1a:S3386-	1	0.01
Delhi										ORF1a:G3387-	1	0.01
Delhi										ORF1a:V3388-	1	0.01
Delhi										ORF1a:Y3389-	1	0.01
Delhi										ORF1a:Q3390-	1	0.01
Delhi										ORF1a:C3391-	1	0.01
Delhi										ORF1a:A3392-	1	0.01

Delhi										ORF1a:M3393-	1	0.01
Delhi										ORF1a:R3394-	1	0.01
Delhi										ORF1a:P3395-	1	0.01
Delhi										ORF1a:K3400-	1	0.01
Delhi										ORF1a:G3401-	1	0.01
Delhi										ORF1a:S3402-	1	0.01
Delhi										ORF1a:F3403-	1	0.01
Delhi										ORF1a:L3404-	1	0.01
Delhi										ORF1a:N3405-	1	0.01
Delhi										ORF1a:G3406-	1	0.01
Delhi										ORF1a:N3414-	1	0.01
Delhi										ORF1a:I3415-	1	0.01
Delhi										ORF1a:D3416-	1	0.01
Delhi										ORF1a:Y3417-	1	0.01
Delhi										ORF1a:D3418-	1	0.01
Delhi										ORF1a:C3419-	1	0.01
Delhi										ORF1a:V3420-	1	0.01
Delhi										ORF1a:S3421-	1	0.01
Delhi										ORF1a:F3422-	1	0.01
Delhi										ORF1a:C3423-	1	0.01
Delhi										ORF1a:Y3424-	1	0.01
Delhi										ORF1a:M3425-	1	0.01
Delhi										ORF1a:H3426-	1	0.01
Delhi										ORF1a:H3427-	1	0.01
Delhi										ORF1a:M3428-	1	0.01
Delhi										ORF1a:E3429-	1	0.01
Delhi										ORF1a:L3430-	1	0.01
Delhi										ORF1a:P3431-	1	0.01
Delhi										ORF1a:T3432-	1	0.01
Delhi										ORF1a:G3433-	1	0.01
Delhi										ORF1a:T3453-	1	0.01
Delhi										ORF1a:A3454-	1	0.01
Delhi										ORF1a:Q3455-	1	0.01
Delhi										ORF1a:A3456-	1	0.01
Delhi										ORF1a:A3457-	1	0.01
Delhi										ORF1a:G3458-	1	0.01
Delhi										ORF1a:T3459-	1	0.01
Delhi										ORF1a:D3460-	1	0.01
Delhi										ORF1a:T3461-	1	0.01
Delhi										ORF1a:T3462-	1	0.01
Delhi										ORF1a:I3463-	1	0.01
Delhi										ORF1a:T3464-	1	0.01
Delhi										ORF1a:V3465-	1	0.01
Delhi										ORF1a:N3466-	1	0.01
Delhi										ORF1a:V3467-	1	0.01
Delhi										ORF1a:L3468-	1	0.01
Delhi										ORF1a:A3469-	1	0.01
Delhi										ORF1a:N3491-	1	0.01
Delhi										ORF1a:D3492-	1	0.01
Delhi										ORF1a:F3493-	1	0.01
Delhi										ORF1a:N3494-	1	0.01
Delhi										ORF1a:L3495-	1	0.01
Delhi										ORF1a:V3496-	1	0.01
Delhi										ORF1a:A3497-	1	0.01
Delhi										ORF1a:M3498-	1	0.01
Delhi										ORF1a:K3499-	1	0.01
Delhi										ORF1a:Y3500-	1	0.01
Delhi										ORF1a:N3501-	1	0.01
Delhi										ORF1a:Y3502-	1	0.01
Delhi										ORF1a:E3503-	1	0.01
Delhi										ORF1a:P3504-	1	0.01
Delhi										ORF1a:L3505-	1	0.01
Delhi										ORF1a:T3506-	1	0.01
Delhi										ORF1a:E3533-	1	0.01
Delhi										ORF1a:L3534-	1	0.01
Delhi										ORF1a:L3535-	1	0.01
Delhi										ORF1a:Q3536-	1	0.01
Delhi										ORF1a:N3537-	1	0.01
Delhi										ORF1a:G3538-	1	0.01
Delhi										ORF1a:M3539-	1	0.01
Delhi										ORF1a:N3540-	1	0.01
Delhi										ORF1a:G3541-	1	0.01
Delhi										ORF1a:R3542-	1	0.01

Delhi										**ORF1a:T3543-**	1	0.01
Delhi										**ORF1a:I3544-**	1	0.01
Delhi										**ORF1a:L3545-**	1	0.01
Delhi										**ORF1a:G3546-**	1	0.01
Delhi										**ORF1a:S3547-**	1	0.01
Delhi										**ORF1a:E3551-**	1	0.01
Delhi										**ORF1a:D3552-**	1	0.01
Delhi										**ORF1a:E3553-**	1	0.01
Delhi										**ORF1a:F3554-**	1	0.01
Delhi										**ORF1a:T3555-**	1	0.01
Delhi										**ORF1a:P3556-**	1	0.01
Delhi										**ORF1a:F3557-**	1	0.01
Delhi										**ORF1a:D3558-**	1	0.01
Delhi										**ORF1a:V3559-**	1	0.01
Delhi										**ORF1a:V3560-**	1	0.01
Delhi										**ORF1a:R3561-**	1	0.01
Delhi										**ORF1a:Q3562-**	1	0.01
Delhi										**ORF1a:C3563-**	1	0.01
Delhi										**ORF1a:S3564-**	1	0.01
Delhi										**ORF1a:G3565-**	1	0.01
Delhi										**ORF1a:V3566-**	1	0.01
Delhi										**ORF1a:T3567-**	1	0.01
Delhi										**ORF1a:F3568-**	1	0.01
Delhi										**ORF1a:K3573-**	1	0.01
Delhi										**ORF1a:R3574-**	1	0.01
Delhi										**ORF1a:T3575-**	1	0.01
Delhi										**ORF1a:I3576-**	1	0.01
Delhi										**ORF1a:K3577-**	1	0.01
Delhi										**ORF1a:G3578-**	1	0.01
Delhi										**ORF1a:T3579-**	1	0.01
Delhi										**ORF1a:H3580-**	1	0.01
Delhi										**ORF1a:H3581-**	1	0.01
Delhi										**ORF1a:W3582-**	1	0.01
Delhi										**ORF1a:L3583-**	1	0.01
Delhi										**ORF1a:L3641-**	1	0.01
Delhi										**ORF1a:P3642-**	1	0.01
Delhi										**ORF1a:S3643-**	1	0.01
Delhi										**ORF1a:L3644-**	1	0.01
Delhi										**ORF1a:A3645-**	1	0.01
Delhi										**ORF1a:T3646-**	1	0.01
Delhi										**ORF1a:V3647-**	1	0.01
Delhi										**ORF1a:A3648-**	1	0.01
Delhi										**ORF1a:Y3649-**	1	0.01
Delhi										**ORF1a:F3650-**	1	0.01
Delhi										**ORF1a:N3651-**	1	0.01
Delhi										**ORF1a:M3652-**	1	0.01
Delhi										**ORF1a:V3653-**	1	0.01
Delhi										**ORF1a:Y3654-**	1	0.01
Delhi										**ORF1a:M3655-**	1	0.01
Delhi										**ORF1a:P3656-**	1	0.01
Delhi										**ORF1a:A3657-**	1	0.01
Delhi										**ORF1a:K3678-**	1	0.01
Delhi										**ORF1a:L3679-**	1	0.01
Delhi										**ORF1a:K3680-**	1	0.01
Delhi										**ORF1a:D3681-**	1	0.01
Delhi										**ORF1a:C3682-**	1	0.01
Delhi										**ORF1a:V3683-**	1	0.01
Delhi										**ORF1a:M3684-**	1	0.01
Delhi										**ORF1a:Y3685-**	1	0.01
Delhi										**ORF1a:A3686-**	1	0.01
Delhi										**ORF1a:T3696-**	1	0.01
Delhi										**ORF1a:A3697-**	1	0.01
Delhi										**ORF1a:R3698-**	1	0.01
Delhi										**ORF1a:T3699-**	1	0.01
Delhi										**ORF1a:V3700-**	1	0.01
Delhi										**ORF1a:Y3701-**	1	0.01
Delhi										**ORF1a:D3702-**	1	0.01
Delhi										**ORF1a:D3703-**	1	0.01
Delhi										**ORF1a:G3704-**	1	0.01
Delhi										**ORF1a:A3705-**	1	0.01
Delhi										**ORF1a:R3706-**	1	0.01
Delhi										**ORF1a:R3707-**	1	0.01
Delhi										**ORF1a:V3708-**	1	0.01

**Delhi**	**Nucleotide deletions**											
**Delhi**	**Pre-Wave 1**			**Wave 1**			**Pre-Wave 2**			**Wave 2**		
Delhi	**509-523**	1	5.88	**21992-21994**	14	11.02	**28271**	239	20.02	**28271**	1062	27.82
Delhi	**516-518**	1	5.88	**11288-11296**	10	7.87	**21992-21994**	216	18.09	**28248-28253**	525	13.75
Delhi	**22709-22724**	1	5.88	**28271**	9	7.09	**21765-21770**	215	18.01	**21765-21770**	416	10.9
Delhi	**22730-22734**	1	5.88	**27948-27950**	8	6.3	**11288-11296**	211	17.67	**21992-21994**	413	10.82
Delhi	**21765-21770**	1	5.88	**28881-28884**	6	4.72	**8620**	121	10.13	**11288-11296**	384	10.06
Delhi	**28**	1	5.88	**21765-21770**	6	4.72	**28278-28280**	32	2.68	**22029-22034**	381	9.98
Delhi	**26401-26433**	1	5.88	**26161**	5	3.94	**27205-27207**	31	2.6	**8620**	261	6.84
Delhi	**26440-26444**	1	5.88	**686-694**	4	3.15	**29728-29776**	21	1.76	**27205-27207**	53	1.39
Delhi	**26449-26468**	1	5.88	**21980-21982**	4	3.15	**22283-22291**	11	0.92	**28278-28280**	52	1.36
Delhi	**5822-5834**	1	5.88	**26158-26161**	3	2.36	**19297-19433**	7	0.59	**29728-29776**	41	1.07
Delhi	**2591-2821**	1	5.88	**21993-21998**	3	2.36	**22349-22350**	5	0.42	**686-694**	25	0.65
Delhi	**19300**	1	5.88	**21972-21995**	2	1.57	**5261-5271**	5	0.42	**22283-22291**	19	0.5
Delhi	**13993**	1	5.88	**6350-6397**	2	1.57	**19300**	5	0.42	**19297-19298**	15	0.39
Delhi	**19297**	1	5.88	**27792-27793**	2	1.57	**19298**	4	0.34	**19300**	11	0.29
Delhi	**5277-5278**	1	5.88	**8620**	2	1.57	**5261-5264**	4	0.34	**25448-25476**	9	0.24
Delhi	**11396-11397**	1	5.88	**21983-21994**	2	1.57	**28248-28253**	4	0.34	**25519**	9	0.24
Delhi	**15808**	1	5.88	**19297-19433**	2	1.57	**21980-21982**	3	0.25	**19298**	8	0.21
Delhi				**28090-28095**	2	1.57	**22029-22034**	3	0.25	**515-520**	6	0.16
Delhi				**27792-27794**	2	1.57	**28065-28066**	2	0.17	**19298-19428**	5	0.13
Delhi				**29765-29771**	2	1.57	**222**	2	0.17	**19300-19548**	5	0.13
Delhi				**19402-19437**	2	1.57	**686-694**	2	0.17	**19555-19557**	5	0.13
Delhi				**6518-6523**	1	0.79	**28090-28095**	2	0.17	**3833-3835**	4	0.1
Delhi				**29756-29762**	1	0.79	**26161**	2	0.17	**27556-27624**	4	0.1
Delhi				**28254**	1	0.79	**19300-19306**	2	0.17	**28090-28095**	4	0.1
Delhi				**27792**	1	0.79	**5261-5266**	2	0.17	**26155-26157**	4	0.1
Delhi				**26155-26157**	1	0.79	**27514-27516**	2	0.17	**19298-19326**	4	0.1
Delhi				**9857-10009**	1	0.79	**19298-19465**	2	0.17	**5261-5264**	4	0.1
Delhi				**515-523**	1	0.79	**22348-22519**	2	0.17	**510-518**	3	0.08
Delhi				**21611-21649**	1	0.79	**27751-27762**	1	0.08	**518-520**	3	0.08
Delhi				**27579-27581**	1	0.79	**3332-3343**	1	0.08	**27887-27901**	3	0.08
Delhi				**26158-26178**	1	0.79	**29767-29776**	1	0.08	**6513-6515**	2	0.05
Delhi				**26485-26486**	1	0.79	**13401-13470**	1	0.08	**28896-28898**	2	0.05
Delhi				**27888-27899**	1	0.79	**21985-21993**	1	0.08	**19300-19304**	2	0.05
Delhi				**29711**	1	0.79	**28242-28246**	1	0.08	**27565-27616**	2	0.05
Delhi				**6506-6508**	1	0.79	**22352-22353**	1	0.08	**19298-19413**	2	0.05
Delhi				**21632-21634**	1	0.79	**21993-21998**	1	0.08	**22349-22350**	2	0.05
Delhi				**438-446**	1	0.79	**5263-5268**	1	0.08	**5019**	2	0.05
Delhi				**26359-26382**	1	0.79	**21148-21387**	1	0.08	**19336-19380**	2	0.05
Delhi				**27792-27800**	1	0.79	**21148-21197**	1	0.08	**19390-19391**	2	0.05
Delhi				**22190-22192**	1	0.79	**21148-21260**	1	0.08	**4070-4071**	2	0.05
Delhi				**5948-6043**	1	0.79	**5260-5268**	1	0.08	**28781-28791**	2	0.05
Delhi				**29726-29729**	1	0.79	**21456-21470**	1	0.08	**5261**	2	0.05
Delhi				**29730-29766**	1	0.79	**5277-5278**	1	0.08	**5261-5271**	2	0.05
Delhi				**25447-25476**	1	0.79	**1598-1603**	1	0.08	**15808**	2	0.05
Delhi				**1431-1433**	1	0.79	**27792**	1	0.08	**21719-21724**	1	0.03
Delhi				**6323-6349**	1	0.79	**28083-28087**	1	0.08	**29582-29587**	1	0.03
Delhi				**29728-29735**	1	0.79	**19297-19340**	1	0.08	**21556-21561**	1	0.03
Delhi				**29764-29772**	1	0.79	**19298-19428**	1	0.08	**21734-21739**	1	0.03
Delhi				**29738-29748**	1	0.79	**27792-27794**	1	0.08	**3336-3344**	1	0.03
Delhi				**27620-27623**	1	0.79	**29736-29759**	1	0.08	**21767-21772**	1	0.03
Delhi				**19285**	1	0.79	**22345**	1	0.08	**28270**	1	0.03
Delhi				**28252-28256**	1	0.79	**19298-19302**	1	0.08	**22877-22974**	1	0.03
Delhi				**27701-27702**	1	0.79	**19300-19474**	1	0.08	**27383-27398**	1	0.03
Delhi				**26158-26162**	1	0.79	**19300-19437**	1	0.08	**27406-27745**	1	0.03
Delhi				**27888-27935**	1	0.79	**19301-19416**	1	0.08	**29780-29787**	1	0.03
Delhi				**21430-21632**	1	0.79	**19298-19306**	1	0.08	**29792-29800**	1	0.03
Delhi							**21990-21995**	1	0.08	**19301-19416**	1	0.03
Delhi							**10174**	1	0.08	**19297-19555**	1	0.03
Delhi							**9863-9880**	1	0.08	**27565-27568**	1	0.03
Delhi							**29729-29774**	1	0.08	**27571-27581**	1	0.03
Delhi							**21911-21913**	1	0.08	**27578-27623**	1	0.03
Delhi							**21923-21925**	1	0.08	**5261-5266**	1	0.03
Delhi							**25346-25351**	1	0.08	**27388-27398**	1	0.03
Delhi							**21987-21995**	1	0.08	**19276-19277**	1	0.03
Delhi							**22285-22305**	1	0.08	**19285-19304**	1	0.03
Delhi										**21992-21993**	1	0.03
Delhi										**5260-5263**	1	0.03
Delhi										**27265-27290**	1	0.03
Delhi										**27694-27700**	1	0.03

Delhi										29726-29727	1	0.03
Delhi										19300-19306	1	0.03
Delhi										1431-1433	1	0.03
Delhi										5261-5278	1	0.03
Delhi										29726	1	0.03
Delhi										29729-29747	1	0.03
Delhi										27556-27625	1	0.03
Delhi										27911-27949	1	0.03
Delhi										21987-21995	1	0.03
Delhi										19298-19300	1	0.03
Delhi										19402-19407	1	0.03
Delhi										27571-27623	1	0.03
Delhi										19297-19340	1	0.03
Delhi										19351-19410	1	0.03
Delhi										19363-19430	1	0.03
Delhi										29728-29752	1	0.03
Delhi										21980-21982	1	0.03
Delhi										25393-25395	1	0.03
Delhi										22347-22480	1	0.03
Delhi										26161-26168	1	0.03
Delhi										28095	1	0.03
Delhi										5277	1	0.03
Delhi										2270	1	0.03
Delhi										19297-19304	1	0.03
Delhi										21719-21727	1	0.03
Delhi										28778	1	0.03
Delhi										11396-11397	1	0.03
**Karnataka**	**ORF1a**											
**Karnataka**	**Pre-Wave 1**			**Wave 1**			**Pre-Wave 2**			**Wave 2**		
Karnataka	**Mutation**	**Count**	**percent**	**Mutation**	**Count**	**percent**	**Mutation**	**Count**	**percent**	**Mutation**	**Count**	**percent**
Karnataka	**ORF1a:L3606F**	58	26.85	**ORF1a:T1001I**	101	37.55	**ORF1a:T1001I**	89	42.38	**ORF1a:T3646A**	680	66.93
Karnataka	**ORF1a:S318L**	49	22.69	**ORF1a:D3042N**	75	27.88	**ORF1a:D3042N**	40	19.05	**ORF1a:P2046L**	533	52.46
Karnataka	**ORF1a:T2016K**	42	19.44	**ORF1a:S318L**	29	10.78	**ORF1a:T3646A**	31	14.76	**ORF1a:V2930L**	530	52.17
Karnataka	**ORF1a:I1159M**	17	7.87	**ORF1a:L3606F**	22	8.18	**ORF1a:A1708D**	30	14.29	**ORF1a:A1306S**	528	51.97
Karnataka	**ORF1a:V2586G**	14	6.48	**ORF1a:A1812D**	21	7.81	**ORF1a:I2230T**	25	11.9	**ORF1a:T3255I**	526	51.77
Karnataka	**ORF1a:T1597I**	13	6.02	**ORF1a:A1708D**	15	5.58	**ORF1a:R848M**	21	10	**ORF1a:P2287S**	515	50.69
Karnataka	**ORF1a:A1812D**	11	5.09	**ORF1a:I2230T**	15	5.58	**ORF1a:R3993L**	19	9.05	**ORF1a:P1640L**	195	19.19
Karnataka	**ORF1a:D3042N**	9	4.17	**ORF1a:T4204A**	9	3.35	**ORF1a:F3677L**	15	7.14	**ORF1a:A3209V**	156	15.35
Karnataka	**ORF1a:V2453G**	9	4.17	**ORF1a:Y905H**	9	3.35	**ORF1a:K1247R**	12	5.71	**ORF1a:V3718A**	154	15.16
Karnataka	**ORF1a:N2454S**	9	4.17	**ORF1a:S944L**	8	2.97	**ORF1a:L2062F**	12	5.71	**ORF1a:T1567I**	129	12.7
Karnataka	**ORF1a:V1295F**	9	4.17	**ORF1a:V561A**	7	2.6	**ORF1a:T1567I**	12	5.71	**ORF1a:P309L**	103	10.14
Karnataka	**ORF1a:I114T**	9	4.17	**ORF1a:V28I**	5	1.86	**ORF1a:L730F**	11	5.24	**ORF1a:T1001I**	100	9.84
Karnataka	**ORF1a:R207C**	9	4.17	**ORF1a:S2103F**	4	1.49	**ORF1a:P309L**	11	5.24	**ORF1a:T3750I**	97	9.55
Karnataka	**ORF1a:V378I**	9	4.17	**ORF1a:M1014I**	4	1.49	**ORF1a:P2046L**	10	4.76	**ORF1a:A1708D**	76	7.48
Karnataka	**ORF1a:M2796I**	9	4.17	**ORF1a:K3979N**	4	1.49	**ORF1a:N363K**	9	4.29	**ORF1a:I2230T**	64	6.3
Karnataka	**ORF1a:V3968I**	9	4.17	**ORF1a:R398C**	4	1.49	**ORF1a:L3606F**	9	4.29	**ORF1a:F3138S**	45	4.43
Karnataka	**ORF1a:P971S**	8	3.7	**ORF1a:Q3966R**	4	1.49	**ORF1a:A1306S**	9	4.29	**ORF1a:D2980N**	44	4.33
Karnataka	**ORF1a:P2376L**	8	3.7	**ORF1a:L397I**	4	1.49	**ORF1a:P2287S**	9	4.29	**ORF1a:H3580Q**	41	4.04
Karnataka	**ORF1a:S40T**	7	3.24	**ORF1a:P1692S**	4	1.49	**ORF1a:V2930L**	9	4.29	**ORF1a:T4083M**	22	2.17
Karnataka	**ORF1a:N2671Y**	7	3.24	**ORF1a:A3620V**	4	1.49	**ORF1a:V54I**	8	3.81	**ORF1a:V54I**	20	1.97
Karnataka	**ORF1a:E3188V**	7	3.24	**ORF1a:V1169A**	3	1.12	**ORF1a:I404T**	8	3.81	**ORF1a:H2092Y**	20	1.97
Karnataka	**ORF1a:M3189A**	7	3.24	**ORF1a:A2098V**	3	1.12	**ORF1a:T814I**	8	3.81	**ORF1a:K261N**	17	1.67
Karnataka	**ORF1a:F2787C**	6	2.78	**ORF1a:A2994V**	3	1.12	**ORF1a:K1247N**	8	3.81	**ORF1a:V1169A**	17	1.67
Karnataka	**ORF1a:P1803S**	6	2.78	**ORF1a:G519S**	3	1.12	**ORF1a:P371L**	7	3.33	**ORF1a:L3606F**	16	1.57
Karnataka	**ORF1a:A339V**	5	2.31	**ORF1a:P309L**	3	1.12	**ORF1a:K3353R**	7	3.33	**ORF1a:K3353R**	16	1.57
Karnataka	**ORF1a:I1577L**	4	1.85	**ORF1a:K292N**	3	1.12	**ORF1a:A3209V**	7	3.33	**ORF1a:K2208N**	14	1.38
Karnataka	**ORF1a:L3800H**	4	1.85	**ORF1a:T3202M**	3	1.12	**ORF1a:T3255I**	7	3.33	**ORF1a:D3042N**	11	1.08
Karnataka	**ORF1a:V561A**	3	1.39	**ORF1a:K247E**	3	1.12	**ORF1a:P1640L**	7	3.33	**ORF1a:P4120S**	11	1.08
Karnataka	**ORF1a:R848S**	3	1.39	**ORF1a:T3567I**	3	1.12	**ORF1a:V561A**	6	2.86	**ORF1a:G519S**	11	1.08
Karnataka	**ORF1a:F1248S**	3	1.39	**ORF1a:T1854I**	3	1.12	**ORF1a:G3676S**	6	2.86	**ORF1a:L4182F**	10	0.98
Karnataka	**ORF1a:G2265V**	3	1.39	**ORF1a:P371L**	3	1.12	**ORF1a:K1655N**	5	2.38	**ORF1a:S2048F**	10	0.98
Karnataka	**ORF1a:I1877T**	3	1.39	**ORF1a:L4182F**	3	1.12	**ORF1a:S944L**	5	2.38	**ORF1a:T760I**	9	0.89
Karnataka	**ORF1a:T2463S**	3	1.39	**ORF1a:S2132G**	3	1.12	**ORF1a:T4083M**	5	2.38	**ORF1a:S944L**	7	0.69
Karnataka	**ORF1a:V3467A**	3	1.39	**ORF1a:L3027F**	3	1.12	**ORF1a:V1169A**	5	2.38	**ORF1a:M2259I**	7	0.69
Karnataka	**ORF1a:N1576H**	3	1.39	**ORF1a:D2980G**	3	1.12	**ORF1a:P3504L**	4	1.9	**ORF1a:S279F**	7	0.69
Karnataka	**ORF1a:P2134S**	2	0.93	**ORF1a:I4049V**	2	0.74	**ORF1a:T4355I**	4	1.9	**ORF1a:F3677L**	7	0.69
Karnataka	**ORF1a:P1096S**	2	0.93	**ORF1a:V3331I**	2	0.74	**ORF1a:S318L**	4	1.9	**ORF1a:N1662S**	7	0.69
Karnataka	**ORF1a:D1440G**	2	0.93	**ORF1a:T3506A**	2	0.74	**ORF1a:A3615V**	4	1.9	**ORF1a:S528L**	6	0.59
Karnataka	**ORF1a:R124C**	2	0.93	**ORF1a:S3344F**	2	0.74	**ORF1a:A1812D**	4	1.9	**ORF1a:A3070V**	6	0.59
Karnataka	**ORF1a:T1001I**	2	0.93	**ORF1a:A4094V**	2	0.74	**ORF1a:T1597I**	4	1.9	**ORF1a:P3395S**	6	0.59

Karnataka	**ORF1a:A1176S**	2	0.93	**ORF1a:S3547G**	2	0.74	**ORF1a:T4087I**	4	1.9	**ORF1a:D33G**	6	0.59
Karnataka	**ORF1a:W2316R**	2	0.93	**ORF1a:S2024L**	2	0.74	**ORF1a:M3752I**	4	1.9	**ORF1a:A2994V**	6	0.59
Karnataka	**ORF1a:V2276G**	2	0.93	**ORF1a:R1464W**	2	0.74	**ORF1a:T265I**	4	1.9	**ORF1a:T1597I**	5	0.49
Karnataka	**ORF1a:I4062M**	2	0.93	**ORF1a:T4088I**	2	0.74	**ORF1a:A599V**	4	1.9	**ORF1a:S2103F**	5	0.49
Karnataka	**ORF1a:P4063S**	2	0.93	**ORF1a:K3886R**	2	0.74	**ORF1a:V3718A**	4	1.9	**ORF1a:T3058I**	5	0.49
Karnataka	**ORF1a:L4064P**	2	0.93	**ORF1a:V3759F**	2	0.74	**ORF1a:P971L**	3	1.43	**ORF1a:G728C**	5	0.49
Karnataka	**ORF1a:N1576T**	2	0.93	**ORF1a:A3143V**	2	0.74	**ORF1a:S2488F**	3	1.43	**ORF1a:A498V**	5	0.49
Karnataka	**ORF1a:N3801Y**	2	0.93	**ORF1a:T753I**	2	0.74	**ORF1a:Q2702H**	3	1.43	**ORF1a:E235G**	4	0.39
Karnataka	**ORF1a:Q970H**	2	0.93	**ORF1a:P3613L**	2	0.74	**ORF1a:N4167S**	3	1.43	**ORF1a:P1921Q**	4	0.39
Karnataka	**ORF1a:E235G**	2	0.93	**ORF1a:G227S**	2	0.74	**ORF1a:T346I**	3	1.43	**ORF1a:L2062F**	4	0.39
Karnataka	**ORF1a:G3072C**	1	0.46	**ORF1a:E2K**	2	0.74	**ORF1a:A206V**	3	1.43	**ORF1a:D1532G**	4	0.39
Karnataka	**ORF1a:Q3893H**	1	0.46	**ORF1a:V3968I**	2	0.74	**ORF1a:T4031I**	3	1.43	**ORF1a:A486V**	4	0.39
Karnataka	**ORF1a:H3580Y**	1	0.46	**ORF1a:L3201F**	2	0.74	**ORF1a:R24C**	3	1.43	**ORF1a:S653L**	4	0.39
Karnataka	**ORF1a:F4196S**	1	0.46	**ORF1a:S2535L**	2	0.74	**ORF1a:L1130F**	3	1.43	**ORF1a:M3087I**	4	0.39
Karnataka	**ORF1a:K1230R**	1	0.46	**ORF1a:A3209V**	2	0.74	**ORF1a:T4174A**	3	1.43	**ORF1a:K1948T**	4	0.39
Karnataka	**ORF1a:E772A**	1	0.46	**ORF1a:G1155C**	2	0.74	**ORF1a:S1612L**	3	1.43	**ORF1a:S4398L**	4	0.39
Karnataka	**ORF1a:A773V**	1	0.46	**ORF1a:K2928N**	2	0.74	**ORF1a:T4204A**	3	1.43	**ORF1a:L730F**	4	0.39
Karnataka	**ORF1a:V774S**	1	0.46	**ORF1a:R1404C**	2	0.74	**ORF1a:L552F**	3	1.43	**ORF1a:P1010L**	4	0.39
Karnataka	**ORF1a:E775Y**	1	0.46	**ORF1a:K1817R**	2	0.74	**ORF1a:T3750I**	3	1.43	**ORF1a:T3284I**	4	0.39
Karnataka	**ORF1a:A776T**	1	0.46	**ORF1a:P1862S**	2	0.74	**ORF1a:S528L**	3	1.43	**ORF1a:R848M**	3	0.3
Karnataka	**ORF1a:C857L**	1	0.46	**ORF1a:Q2702H**	2	0.74	**ORF1a:Y1465C**	2	0.95	**ORF1a:S1188P**	3	0.3
Karnataka	**ORF1a:D2094C**	1	0.46	**ORF1a:R3993L**	2	0.74	**ORF1a:H417Y**	2	0.95	**ORF1a:K4176N**	3	0.3
Karnataka	**ORF1a:M2096L**	1	0.46	**ORF1a:N4167S**	2	0.74	**ORF1a:T2408I**	2	0.95	**ORF1a:H3076Y**	3	0.3
Karnataka	**ORF1a:A2097Y**	1	0.46	**ORF1a:L552F**	2	0.74	**ORF1a:E2070K**	2	0.95	**ORF1a:S443F**	3	0.3
Karnataka	**ORF1a:A2098T**	1	0.46	**ORF1a:S1029N**	2	0.74	**ORF1a:V2646L**	2	0.95	**ORF1a:D1554Y**	3	0.3
Karnataka	**ORF1a:K2511N**	1	0.46	**ORF1a:M3752I**	2	0.74	**ORF1a:P2134S**	2	0.95	**ORF1a:T4249I**	3	0.3
Karnataka	**ORF1a:S3884T**	1	0.46	**ORF1a:K1763N**	2	0.74	**ORF1a:S3884L**	2	0.95	**ORF1a:T1854I**	3	0.3
Karnataka	**ORF1a:R3993C**	1	0.46	**ORF1a:T4304I**	2	0.74	**ORF1a:N593K**	2	0.95	**ORF1a:A3333V**	3	0.3
Karnataka	**ORF1a:A565V**	1	0.46	**ORF1a:E1192D**	2	0.74	**ORF1a:S1029I**	2	0.95	**ORF1a:M3280I**	3	0.3
Karnataka	**ORF1a:R24C**	1	0.46	**ORF1a:P1879L**	2	0.74	**ORF1a:F2787V**	2	0.95	**ORF1a:V2613F**	3	0.3
Karnataka	**ORF1a:V464F**	1	0.46	**ORF1a:P2046S**	2	0.74	**ORF1a:T1637I**	2	0.95	**ORF1a:G2035V**	3	0.3
Karnataka	**ORF1a:M1083I**	1	0.46	**ORF1a:M3911I**	2	0.74	**ORF1a:S302F**	2	0.95	**ORF1a:S2500F**	3	0.3
Karnataka	**ORF1a:M3752I**	1	0.46	**ORF1a:P971L**	2	0.74	**ORF1a:G963C**	2	0.95	**ORF1a:V1071I**	3	0.3
Karnataka	**ORF1a:H13Y**	1	0.46	**ORF1a:Q3777R**	1	0.37	**ORF1a:M3655I**	2	0.95	**ORF1a:G1946S**	3	0.3
Karnataka	**ORF1a:M2565I**	1	0.46	**ORF1a:K2622N**	1	0.37	**ORF1a:L3667F**	2	0.95	**ORF1a:T2093I**	3	0.3
Karnataka	**ORF1a:A2994F**	1	0.46	**ORF1a:L642F**	1	0.37	**ORF1a:K292N**	2	0.95	**ORF1a:P2605S**	3	0.3
Karnataka	**ORF1a:A3548P**	1	0.46	**ORF1a:V1104I**	1	0.37	**ORF1a:T3202M**	2	0.95	**ORF1a:D203Y**	3	0.3
Karnataka	**ORF1a:P286L**	1	0.46	**ORF1a:T2648I**	1	0.37	**ORF1a:P3395L**	2	0.95	**ORF1a:L1270F**	3	0.3
Karnataka	**ORF1a:E961G**	1	0.46	**ORF1a:D644N**	1	0.37	**ORF1a:H3509Y**	2	0.95	**ORF1a:T1840I**	3	0.3
Karnataka	**ORF1a:V852L**	1	0.46	**ORF1a:V3524I**	1	0.37	**ORF1a:S212L**	2	0.95	**ORF1a:S2972F**	3	0.3
Karnataka	**ORF1a:A2587V**	1	0.46	**ORF1a:R99H**	1	0.37	**ORF1a:P1207L**	2	0.95	**ORF1a:L3116F**	3	0.3
Karnataka	**ORF1a:G519S**	1	0.46	**ORF1a:T1840I**	1	0.37	**ORF1a:I1276T**	2	0.95	**ORF1a:A1812D**	3	0.3
Karnataka	**ORF1a:S2015R**	1	0.46	**ORF1a:S2695R**	1	0.37	**ORF1a:A2529V**	2	0.95	**ORF1a:I4049V**	3	0.3
Karnataka	**ORF1a:K1655N**	1	0.46	**ORF1a:M3087I**	1	0.37	**ORF1a:P3504H**	2	0.95	**ORF1a:T350I**	3	0.3
Karnataka	**ORF1a:P1921L**	1	0.46	**ORF1a:V852L**	1	0.37	**ORF1a:M315I**	2	0.95	**ORF1a:Y359H**	3	0.3
Karnataka	**ORF1a:Y905H**	1	0.46	**ORF1a:T1761I**	1	0.37	**ORF1a:V1211F**	2	0.95	**ORF1a:W2014R**	3	0.3
Karnataka	**ORF1a:K3353R**	1	0.46	**ORF1a:A2587V**	1	0.37	**ORF1a:L3930F**	2	0.95	**ORF1a:K3979N**	3	0.3
Karnataka	**ORF1a:A1204V**	1	0.46	**ORF1a:T987P**	1	0.37	**ORF1a:T2007I**	2	0.95	**ORF1a:K1720N**	3	0.3
Karnataka	**ORF1a:D1088G**	1	0.46	**ORF1a:S2048F**	1	0.37	**ORF1a:P1921L**	2	0.95	**ORF1a:V3718F**	3	0.3
Karnataka	**ORF1a:T265I**	1	0.46	**ORF1a:G3757S**	1	0.37	**ORF1a:R287G**	2	0.95	**ORF1a:V868L**	3	0.3
Karnataka	**ORF1a:D1625V**	1	0.46	**ORF1a:S610L**	1	0.37	**ORF1a:D4344N**	2	0.95	**ORF1a:K2511N**	3	0.3
Karnataka	**ORF1a:L2781P**	1	0.46	**ORF1a:K1319T**	1	0.37	**ORF1a:M3087I**	2	0.95	**ORF1a:T2936I**	3	0.3
Karnataka	**ORF1a:Y4379***	1	0.46	**ORF1a:E2617K**	1	0.37	**ORF1a:G1073R**	2	0.95	**ORF1a:M2365I**	3	0.3
Karnataka	**ORF1a:C4381W**	1	0.46	**ORF1a:T4083M**	1	0.37	**ORF1a:S3885F**	1	0.48	**ORF1a:I114V**	3	0.3
Karnataka	**ORF1a:A3143V**	1	0.46	**ORF1a:E940D**	1	0.37	**ORF1a:G934D**	1	0.48	**ORF1a:T333M**	3	0.3
Karnataka	**ORF1a:E381D**	1	0.46	**ORF1a:D928Y**	1	0.37	**ORF1a:S1188L**	1	0.48	**ORF1a:M321V**	3	0.3
Karnataka	**ORF1a:G2118C**	1	0.46	**ORF1a:T708I**	1	0.37	**ORF1a:R301Q**	1	0.48	**ORF1a:K2497N**	2	0.2
Karnataka	**ORF1a:D4165E**	1	0.46	**ORF1a:Q2510R**	1	0.37	**ORF1a:A1679V**	1	0.48	**ORF1a:I3758V**	2	0.2
Karnataka	**ORF1a:V2629A**	1	0.46	**ORF1a:E386A**	1	0.37	**ORF1a:S2981F**	1	0.48	**ORF1a:G3676C**	2	0.2
Karnataka	**ORF1a:A1298V**	1	0.46	**ORF1a:T4010I**	1	0.37	**ORF1a:M1083I**	1	0.48	**ORF1a:D3681N**	2	0.2
Karnataka	**ORF1a:V757F**	1	0.46	**ORF1a:G30V**	1	0.37	**ORF1a:T403I**	1	0.48	**ORF1a:I222V**	2	0.2
Karnataka	**ORF1a:A2745V**	1	0.46	**ORF1a:S2731G**	1	0.37	**ORF1a:R550H**	1	0.48	**ORF1a:L1182S**	2	0.2
Karnataka	**ORF1a:S944L**	1	0.46	**ORF1a:P1541S**	1	0.37	**ORF1a:T602A**	1	0.48	**ORF1a:R4017K**	2	0.2
Karnataka	**ORF1a:D2627Y**	1	0.46	**ORF1a:C2459Y**	1	0.37	**ORF1a:K2589R**	1	0.48	**ORF1a:V1888I**	2	0.2
Karnataka	**ORF1a:F678S**	1	0.46	**ORF1a:M2096T**	1	0.37	**ORF1a:V3653F**	1	0.48	**ORF1a:P971S**	2	0.2
Karnataka	**ORF1a:A903V**	1	0.46	**ORF1a:K1077M**	1	0.37	**ORF1a:A4357V**	1	0.48	**ORF1a:K1280N**	2	0.2
Karnataka	**ORF1a:S549F**	1	0.46	**ORF1a:T4292I**	1	0.37	**ORF1a:I222V**	1	0.48	**ORF1a:T4031I**	2	0.2
Karnataka	**ORF1a:I3052V**	1	0.46	**ORF1a:M1588I**	1	0.37	**ORF1a:A1397V**	1	0.48	**ORF1a:L580F**	2	0.2
Karnataka	**ORF1a:I238T**	1	0.46	**ORF1a:A540V**	1	0.37	**ORF1a:S2900L**	1	0.48	**ORF1a:R3542C**	2	0.2
Karnataka	**ORF1a:I2368V**	1	0.46	**ORF1a:N1458S**	1	0.37	**ORF1a:S2273F**	1	0.48	**ORF1a:S2900L**	2	0.2
Karnataka	**ORF1a:P3359S**	1	0.46	**ORF1a:S2500F**	1	0.37	**ORF1a:H374Y**	1	0.48	**ORF1a:T1754A**	2	0.2
Karnataka	**ORF1a:V682L**	1	0.46	**ORF1a:I2712V**	1	0.37	**ORF1a:E2031K**	1	0.48	**ORF1a:G524S**	2	0.2
Karnataka	**ORF1a:R3368C**	1	0.46	**ORF1a:D335G**	1	0.37	**ORF1a:L3490F**	1	0.48	**ORF1a:A3963V**	2	0.2

Karnataka	**ORF1a:A206E**	1	0.46	**ORF1a:P769S**	1	0.37	**ORF1a:S4112I**	1	0.48	**ORF1a:T1168I**	2	0.2
Karnataka	**ORF1a:P2144L**	1	0.46	**ORF1a:S2844I**	1	0.37	**ORF1a:V2783F**	1	0.48	**ORF1a:T1543I**	2	0.2
Karnataka	**ORF1a:T666I**	1	0.46	**ORF1a:A482T**	1	0.37	**ORF1a:A211T**	1	0.48	**ORF1a:T2166I**	2	0.2
Karnataka	**ORF1a:N2162S**	1	0.46	**ORF1a:D1273E**	1	0.37	**ORF1a:H2092Y**	1	0.48	**ORF1a:A138D**	2	0.2
Karnataka	**ORF1a:P1158S**	1	0.46	**ORF1a:A3996S**	1	0.37	**ORF1a:L2572I**	1	0.48	**ORF1a:P3395L**	2	0.2
Karnataka	**ORF1a:D2492N**	1	0.46	**ORF1a:K1348N**	1	0.37	**ORF1a:V28I**	1	0.48	**ORF1a:G52S**	2	0.2
Karnataka	**ORF1a:L3123F**	1	0.46	**ORF1a:T609I**	1	0.37	**ORF1a:L1388V**	1	0.48	**ORF1a:N840S**	2	0.2
Karnataka	**ORF1a:E87K**	1	0.46	**ORF1a:T2280I**	1	0.37	**ORF1a:M2254I**	1	0.48	**ORF1a:L3338F**	2	0.2
Karnataka	**ORF1a:S1515F**	1	0.46	**ORF1a:V2061F**	1	0.37	**ORF1a:A3497V**	1	0.48	**ORF1a:G3676S**	2	0.2
Karnataka	**ORF1a:T1626A**	1	0.46	**ORF1a:E1209K**	1	0.37	**ORF1a:L1507F**	1	0.48	**ORF1a:A878T**	2	0.2
Karnataka	**ORF1a:T2898I**	1	0.46	**ORF1a:V2290I**	1	0.37	**ORF1a:A903S**	1	0.48	**ORF1a:G3372R**	2	0.2
Karnataka	**ORF1a:C3637F**	1	0.46	**ORF1a:A117T**	1	0.37	**ORF1a:P1147S**	1	0.48	**ORF1a:A1397V**	2	0.2
Karnataka	**ORF1a:M1586R**	1	0.46	**ORF1a:E797G**	1	0.37	**ORF1a:M1769V**	1	0.48	**ORF1a:P2046S**	2	0.2
Karnataka	**ORF1a:M2374I**	1	0.46	**ORF1a:I1274V**	1	0.37	**ORF1a:T3058I**	1	0.48	**ORF1a:K3832N**	2	0.2
Karnataka	**ORF1a:A2375S**	1	0.46	**ORF1a:S428G**	1	0.37	**ORF1a:K3839R**	1	0.48	**ORF1a:L2122F**	2	0.2
Karnataka	**ORF1a:S135G**	1	0.46	**ORF1a:A2618V**	1	0.37	**ORF1a:G442C**	1	0.48	**ORF1a:S3099L**	2	0.2
Karnataka	**ORF1a:Y136D**	1	0.46	**ORF1a:D1939G**	1	0.37	**ORF1a:T1998I**	1	0.48	**ORF1a:A2123S**	2	0.2
Karnataka	**ORF1a:Y3190C**	1	0.46	**ORF1a:P732S**	1	0.37	**ORF1a:K2511N**	1	0.48	**ORF1a:A1049V**	2	0.2
Karnataka	**ORF1a:K1655R**	1	0.46	**ORF1a:T814I**	1	0.37	**ORF1a:G445S**	1	0.48	**ORF1a:Q556K**	2	0.2
Karnataka	**ORF1a:A2460V**	1	0.46	**ORF1a:A2199T**	1	0.37	**ORF1a:A3705V**	1	0.48	**ORF1a:V4147I**	2	0.2
Karnataka	**ORF1a:G2461C**	1	0.46	**ORF1a:A3357V**	1	0.37	**ORF1a:Y118H**	1	0.48	**ORF1a:S2797F**	2	0.2
Karnataka	**ORF1a:S3885F**	1	0.46	**ORF1a:A1298V**	1	0.37	**ORF1a:A181T**	1	0.48	**ORF1a:P1596L**	2	0.2
Karnataka	**ORF1a:E235N**	1	0.46	**ORF1a:K1124N**	1	0.37	**ORF1a:D1625E**	1	0.48	**ORF1a:P309Q**	2	0.2
Karnataka	**ORF1a:G1073A**	1	0.46	**ORF1a:Y3300C**	1	0.37	**ORF1a:A3523V**	1	0.48	**ORF1a:P748H**	2	0.2
Karnataka	**ORF1a:C1889***	1	0.46	**ORF1a:M4009I**	1	0.37	**ORF1a:K247N**	1	0.48	**ORF1a:D1044Y**	2	0.2
Karnataka	**ORF1a:E235D**	1	0.46	**ORF1a:K322R**	1	0.37	**ORF1a:T1242I**	1	0.48	**ORF1a:V1019F**	2	0.2
Karnataka	**ORF1a:D1179H**	1	0.46	**ORF1a:G3072C**	1	0.37	**ORF1a:P927L**	1	0.48	**ORF1a:A903V**	2	0.2
Karnataka	**ORF1a:V1574L**	1	0.46	**ORF1a:A591V**	1	0.37	**ORF1a:L3027F**	1	0.48	**ORF1a:A1283V**	2	0.2
Karnataka	**ORF1a:I1577T**	1	0.46	**ORF1a:P1497L**	1	0.37	**ORF1a:L122F**	1	0.48	**ORF1a:T1822I**	2	0.2
Karnataka	**ORF1a:Y1776D**	1	0.46	**ORF1a:A1283V**	1	0.37	**ORF1a:P1472S**	1	0.48	**ORF1a:E539G**	2	0.2
Karnataka	**ORF1a:F1779L**	1	0.46	**ORF1a:K1202N**	1	0.37	**ORF1a:V303F**	1	0.48	**ORF1a:A482V**	2	0.2
Karnataka	**ORF1a:I1785R**	1	0.46	**ORF1a:G146S**	1	0.37	**ORF1a:G392C**	1	0.48	**ORF1a:A3958S**	2	0.2
Karnataka	**ORF1a:I1877K**	1	0.46	**ORF1a:Y182H**	1	0.37	**ORF1a:G519D**	1	0.48	**ORF1a:M585I**	2	0.2
Karnataka	**ORF1a:K1878T**	1	0.46	**ORF1a:S4398L**	1	0.37	**ORF1a:N869D**	1	0.48	**ORF1a:T1035I**	2	0.2
Karnataka	**ORF1a:C1889W**	1	0.46	**ORF1a:L1853F**	1	0.37	**ORF1a:H1545Y**	1	0.48	**ORF1a:T602A**	2	0.2
Karnataka	**ORF1a:A4067E**	1	0.46	**ORF1a:Y3649C**	1	0.37	**ORF1a:P2046S**	1	0.48	**ORF1a:A3045V**	2	0.2
Karnataka	**ORF1a:E1126G**	1	0.46	**ORF1a:I3758S**	1	0.37	**ORF1a:T2087I**	1	0.48	**ORF1a:S531N**	2	0.2
Karnataka	**ORF1a:N2275T**	1	0.46	**ORF1a:N189S**	1	0.37	**ORF1a:N2405S**	1	0.48	**ORF1a:T2154I**	2	0.2
Karnataka	**ORF1a:S2578P**	1	0.46	**ORF1a:L580F**	1	0.37	**ORF1a:T965I**	1	0.48	**ORF1a:P1207L**	2	0.2
Karnataka	**ORF1a:C3181Y**	1	0.46	**ORF1a:S3885F**	1	0.37	**ORF1a:M315V**	1	0.48	**ORF1a:P3504H**	2	0.2
Karnataka	**ORF1a:L3490R**	1	0.46	**ORF1a:G645S**	1	0.37	**ORF1a:T1542I**	1	0.48	**ORF1a:A427V**	2	0.2
Karnataka	**ORF1a:N3491T**	1	0.46	**ORF1a:D909N**	1	0.37	**ORF1a:A1283V**	1	0.48	**ORF1a:S1952L**	2	0.2
Karnataka	**ORF1a:L1379H**	1	0.46	**ORF1a:A1092V**	1	0.37	**ORF1a:P3504S**	1	0.48	**ORF1a:T2495I**	2	0.2
Karnataka	**ORF1a:M1588I**	1	0.46	**ORF1a:G1548D**	1	0.37	**ORF1a:A76V**	1	0.48	**ORF1a:T1017A**	2	0.2
Karnataka	**ORF1a:I2311N**	1	0.46	**ORF1a:F3957L**	1	0.37	**ORF1a:L3694F**	1	0.48	**ORF1a:G2667C**	2	0.2
Karnataka	**ORF1a:F2787L**	1	0.46	**ORF1a:A566V**	1	0.37	**ORF1a:T2952I**	1	0.48	**ORF1a:R24C**	2	0.2
Karnataka	**ORF1a:Y2788L**	1	0.46	**ORF1a:A964V**	1	0.37	**ORF1a:S2500F**	1	0.48	**ORF1a:S2517F**	2	0.2
Karnataka	**ORF1a:F3957S**	1	0.46	**ORF1a:V1211F**	1	0.37	**ORF1a:V3718F**	1	0.48	**ORF1a:E102K**	2	0.2
Karnataka	**ORF1a:A3958G**	1	0.46	**ORF1a:A1082V**	1	0.37	**ORF1a:W630C**	1	0.48	**ORF1a:P361L**	2	0.2
Karnataka	**ORF1a:H236N**	1	0.46	**ORF1a:A1812T**	1	0.37	**ORF1a:K2395Q**	1	0.48	**ORF1a:T1246I**	2	0.2
Karnataka	**ORF1a:A872P**	1	0.46	**ORF1a:H1545Y**	1	0.37	**ORF1a:E347D**	1	0.48	**ORF1a:D1639N**	2	0.2
Karnataka	**ORF1a:E972Q**	1	0.46	**ORF1a:C433R**	1	0.37	**ORF1a:G2207S**	1	0.48	**ORF1a:D3222N**	2	0.2
Karnataka	**ORF1a:E973V**	1	0.46	**ORF1a:R579K**	1	0.37	**ORF1a:A4257V**	1	0.48	**ORF1a:G3278S**	2	0.2
Karnataka	**ORF1a:M2194P**	1	0.46	**ORF1a:A1432S**	1	0.37	**ORF1a:T708I**	1	0.48	**ORF1a:L3691S**	2	0.2
Karnataka	**ORF1a:Y3098H**	1	0.46	**ORF1a:P2483L**	1	0.37	**ORF1a:S1189G**	1	0.48	**ORF1a:T4090I**	2	0.2
Karnataka				**ORF1a:K3353R**	1	0.37	**ORF1a:S1515F**	1	0.48	**ORF1a:T3202M**	2	0.2
Karnataka				**ORF1a:G519C**	1	0.37	**ORF1a:S944P**	1	0.48	**ORF1a:P1640F**	2	0.2
Karnataka				**ORF1a:A1473S**	1	0.37	**ORF1a:N2596S**	1	0.48	**ORF1a:V2715F**	2	0.2
Karnataka				**ORF1a:V874F**	1	0.37	**ORF1a:A3456V**	1	0.48	**ORF1a:W331C**	2	0.2
Karnataka				**ORF1a:P2144L**	1	0.37	**ORF1a:M585T**	1	0.48	**ORF1a:K2810R**	2	0.2
Karnataka				**ORF1a:P309S**	1	0.37	**ORF1a:S4398P**	1	0.48	**ORF1a:L552F**	2	0.2
Karnataka				**ORF1a:P3447L**	1	0.37	**ORF1a:I1128V**	1	0.48	**ORF1a:L451F**	2	0.2
Karnataka				**ORF1a:A4016V**	1	0.37	**ORF1a:E1614Q**	1	0.48	**ORF1a:I2765T**	2	0.2
Karnataka				**ORF1a:V3718F**	1	0.37	**ORF1a:K3861R**	1	0.48	**ORF1a:V1117I**	2	0.2
Karnataka				**ORF1a:T1055I**	1	0.37	**ORF1a:K261N**	1	0.48	**ORF1a:S3547I**	2	0.2
Karnataka				**ORF1a:P2221S**	1	0.37	**ORF1a:P2018S**	1	0.48	**ORF1a:S2972P**	2	0.2
Karnataka				**ORF1a:V2613F**	1	0.37	**ORF1a:V756F**	1	0.48	**ORF1a:T770I**	2	0.2
Karnataka				**ORF1a:S3099L**	1	0.37	**ORF1a:K1124E**	1	0.48	**ORF1a:S2352G**	2	0.2
Karnataka				**ORF1a:E1498G**	1	0.37	**ORF1a:P2260S**	1	0.48	**ORF1a:H3509Y**	2	0.2
Karnataka				**ORF1a:A698V**	1	0.37	**ORF1a:N2454S**	1	0.48	**ORF1a:S2488F**	2	0.2
Karnataka				**ORF1a:P2144S**	1	0.37	**ORF1a:T2910I**	1	0.48	**ORF1a:A79V**	2	0.2
Karnataka				**ORF1a:P3952S**	1	0.37	**ORF1a:I95T**	1	0.48	**ORF1a:L3483F**	2	0.2
Karnataka				**ORF1a:T3255I**	1	0.37	**ORF1a:G3578V**	1	0.48	**ORF1a:M315V**	2	0.2

Karnataka				**ORF1a:A1234V**	1	0.37	**ORF1a:N810D**	1	0.48	**ORF1a:I95V**	1	0.1
Karnataka				**ORF1a:S3517F**	1	0.37	**ORF1a:K3832N**	1	0.48	**ORF1a:M2957T**	1	0.1
Karnataka				**ORF1a:L3845V**	1	0.37	**ORF1a:N3501D**	1	0.48	**ORF1a:L4126F**	1	0.1
Karnataka				**ORF1a:L3930F**	1	0.37	**ORF1a:T1000I**	1	0.48	**ORF1a:T4364I**	1	0.1
Karnataka				**ORF1a:P959H**	1	0.37	**ORF1a:N2737S**	1	0.48	**ORF1a:E1384A**	1	0.1
Karnataka				**ORF1a:Y2392F**	1	0.37	**ORF1a:H110Y**	1	0.48	**ORF1a:P1284S**	1	0.1
Karnataka				**ORF1a:N997T**	1	0.37	**ORF1a:H1738Y**	1	0.48	**ORF1a:S2535L**	1	0.1
Karnataka				**ORF1a:P2134S**	1	0.37	**ORF1a:L2146F**	1	0.48	**ORF1a:A2745S**	1	0.1
Karnataka				**ORF1a:S3884L**	1	0.37	**ORF1a:T1682I**	1	0.48	**ORF1a:K3886R**	1	0.1
Karnataka				**ORF1a:T2419I**	1	0.37	**ORF1a:M4116I**	1	0.48	**ORF1a:H374Y**	1	0.1
Karnataka				**ORF1a:T1474I**	1	0.37	**ORF1a:T999I**	1	0.48	**ORF1a:S376L**	1	0.1
Karnataka				**ORF1a:I2227V**	1	0.37	**ORF1a:Q3777R**	1	0.48	**ORF1a:M321T**	1	0.1
Karnataka				**ORF1a:F2172L**	1	0.37	**ORF1a:T4161I**	1	0.48	**ORF1a:P1921L**	1	0.1
Karnataka				**ORF1a:T3589I**	1	0.37	**ORF1a:A307V**	1	0.48	**ORF1a:S2024L**	1	0.1
Karnataka				**ORF1a:A687V**	1	0.37	**ORF1a:M598I**	1	0.48	**ORF1a:E87D**	1	0.1
Karnataka				**ORF1a:S1856F**	1	0.37	**ORF1a:L631F**	1	0.48	**ORF1a:D1926E**	1	0.1
Karnataka				**ORF1a:L631F**	1	0.37	**ORF1a:T1474I**	1	0.48	**ORF1a:K141R**	1	0.1
Karnataka				**ORF1a:E4019D**	1	0.37	**ORF1a:I4049V**	1	0.48	**ORF1a:A967V**	1	0.1
Karnataka				**ORF1a:A599V**	1	0.37	**ORF1a:G150S**	1	0.48	**ORF1a:D4165Y**	1	0.1
Karnataka				**ORF1a:V2943F**	1	0.37	**ORF1a:S3344F**	1	0.48	**ORF1a:A3270V**	1	0.1
Karnataka				**ORF1a:T2093I**	1	0.37	**ORF1a:A656V**	1	0.48	**ORF1a:K2928N**	1	0.1
Karnataka				**ORF1a:A801V**	1	0.37	**ORF1a:D1554N**	1	0.48	**ORF1a:P4337L**	1	0.1
Karnataka				**ORF1a:T1589I**	1	0.37	**ORF1a:P309S**	1	0.48	**ORF1a:P1497S**	1	0.1
Karnataka				**ORF1a:V3595F**	1	0.37	**ORF1a:G989V**	1	0.48	**ORF1a:G295C**	1	0.1
Karnataka				**ORF1a:R848M**	1	0.37	**ORF1a:A4016V**	1	0.48	**ORF1a:S428G**	1	0.1
Karnataka				**ORF1a:T1760I**	1	0.37	**ORF1a:T2684I**	1	0.48	**ORF1a:E457K**	1	0.1
Karnataka				**ORF1a:S2488F**	1	0.37	**ORF1a:A4133V**	1	0.48	**ORF1a:V3349I**	1	0.1
Karnataka				**ORF1a:T3058I**	1	0.37	**ORF1a:E754Q**	1	0.48	**ORF1a:A4133V**	1	0.1
Karnataka				**ORF1a:A211T**	1	0.37	**ORF1a:A3143V**	1	0.48	**ORF1a:V3670F**	1	0.1
Karnataka				**ORF1a:V1056L**	1	0.37	**ORF1a:I3944V**	1	0.48	**ORF1a:A1698S**	1	0.1
Karnataka				**ORF1a:L1579F**	1	0.37	**ORF1a:V2061F**	1	0.48	**ORF1a:I473T**	1	0.1
Karnataka				**ORF1a:L3935M**	1	0.37	**ORF1a:S2378L**	1	0.48	**ORF1a:P1862S**	1	0.1
Karnataka				**ORF1a:A541V**	1	0.37	**ORF1a:T319I**	1	0.48	**ORF1a:M1586I**	1	0.1
Karnataka				**ORF1a:V306F**	1	0.37	**ORF1a:S2242F**	1	0.48	**ORF1a:L681F**	1	0.1
Karnataka				**ORF1a:K2108R**	1	0.37	**ORF1a:A2593T**	1	0.48	**ORF1a:K2533R**	1	0.1
Karnataka				**ORF1a:T654I**	1	0.37	**ORF1a:A3987V**	1	0.48	**ORF1a:L590F**	1	0.1
Karnataka				**ORF1a:P885L**	1	0.37	**ORF1a:F3486S**	1	0.48	**ORF1a:Q2510H**	1	0.1
Karnataka				**ORF1a:S1029I**	1	0.37	**ORF1a:V4369I**	1	0.48	**ORF1a:V1291F**	1	0.1
Karnataka				**ORF1a:T1168I**	1	0.37	**ORF1a:P1220S**	1	0.48	**ORF1a:L624F**	1	0.1
Karnataka				**ORF1a:K4198R**	1	0.37	**ORF1a:A1314V**	1	0.48	**ORF1a:E2112A**	1	0.1
Karnataka				**ORF1a:P271S**	1	0.37	**ORF1a:Q285L**	1	0.48	**ORF1a:G3848V**	1	0.1
Karnataka				**ORF1a:P892S**	1	0.37	**ORF1a:S2103F**	1	0.48	**ORF1a:T2648I**	1	0.1
Karnataka				**ORF1a:P1921L**	1	0.37	**ORF1a:D4344E**	1	0.48	**ORF1a:L177I**	1	0.1
Karnataka				**ORF1a:P2483S**	1	0.37				**ORF1a:M3627I**	1	0.1
Karnataka				**ORF1a:N3484K**	1	0.37				**ORF1a:K120N**	1	0.1
Karnataka				**ORF1a:Q1813H**	1	0.37				**ORF1a:K4348R**	1	0.1
Karnataka				**ORF1a:P3395L**	1	0.37				**ORF1a:N160T**	1	0.1
Karnataka				**ORF1a:A3686V**	1	0.37				**ORF1a:V3367I**	1	0.1
Karnataka				**ORF1a:G4222S**	1	0.37				**ORF1a:S318L**	1	0.1
Karnataka				**ORF1a:A1631V**	1	0.37				**ORF1a:I2712T**	1	0.1
Karnataka				**ORF1a:G2091C**	1	0.37				**ORF1a:M2796L**	1	0.1
Karnataka				**ORF1a:G4177R**	1	0.37				**ORF1a:T4129I**	1	0.1
Karnataka				**ORF1a:T265I**	1	0.37				**ORF1a:A1923V**	1	0.1
Karnataka				**ORF1a:L3352F**	1	0.37				**ORF1a:T4207I**	1	0.1
Karnataka				**ORF1a:Q3729R**	1	0.37				**ORF1a:R426H**	1	0.1
Karnataka				**ORF1a:A3657V**	1	0.37				**ORF1a:A2925V**	1	0.1
Karnataka				**ORF1a:A3969V**	1	0.37				**ORF1a:P361S**	1	0.1
Karnataka				**ORF1a:R124C**	1	0.37				**ORF1a:T4158I**	1	0.1
Karnataka				**ORF1a:A1397S**	1	0.37				**ORF1a:Y1814C**	1	0.1
Karnataka				**ORF1a:S212P**	1	0.37				**ORF1a:G3072C**	1	0.1
Karnataka				**ORF1a:S858Y**	1	0.37				**ORF1a:L3800F**	1	0.1
Karnataka				**ORF1a:L349F**	1	0.37				**ORF1a:T1277A**	1	0.1
Karnataka				**ORF1a:I404T**	1	0.37				**ORF1a:N2155T**	1	0.1
Karnataka				**ORF1a:H1113R**	1	0.37				**ORF1a:P777S**	1	0.1
Karnataka				**ORF1a:A3049V**	1	0.37				**ORF1a:T1022I**	1	0.1
Karnataka				**ORF1a:L1507F**	1	0.37				**ORF1a:G59C**	1	0.1
Karnataka				**ORF1a:S166C**	1	0.37				**ORF1a:W646C**	1	0.1
Karnataka				**ORF1a:V1583I**	1	0.37				**ORF1a:T2898I**	1	0.1
Karnataka				**ORF1a:S302F**	1	0.37				**ORF1a:D220N**	1	0.1
Karnataka				**ORF1a:P748L**	1	0.37				**ORF1a:V511F**	1	0.1
Karnataka				**ORF1a:I3306V**	1	0.37				**ORF1a:S1494A**	1	0.1
Karnataka				**ORF1a:V1299L**	1	0.37				**ORF1a:I1326T**	1	0.1
Karnataka				**ORF1a:M1378I**	1	0.37				**ORF1a:V2491A**	1	0.1

Karnataka				**ORF1a:P1207L**	1	0.37				**ORF1a:T4219I**	1	0.1
Karnataka				**ORF1a:P3504H**	1	0.37				**ORF1a:I3341V**	1	0.1
Karnataka				**ORF1a:N2147S**	1	0.37				**ORF1a:T609I**	1	0.1
Karnataka				**ORF1a:T2166I**	1	0.37				**ORF1a:A2994S**	1	0.1
Karnataka				**ORF1a:A2344V**	1	0.37				**ORF1a:V3510F**	1	0.1
Karnataka				**ORF1a:A4136V**	1	0.37				**ORF1a:L2620F**	1	0.1
Karnataka				**ORF1a:L688V**	1	0.37				**ORF1a:T568I**	1	0.1
Karnataka				**ORF1a:T1567I**	1	0.37				**ORF1a:E933K**	1	0.1
Karnataka				**ORF1a:N2603D**	1	0.37				**ORF1a:E1205K**	1	0.1
Karnataka				**ORF1a:K4014R**	1	0.37				**ORF1a:T2124I**	1	0.1
Karnataka				**ORF1a:L730F**	1	0.37				**ORF1a:A1397S**	1	0.1
Karnataka				**ORF1a:D589G**	1	0.37				**ORF1a:A2123V**	1	0.1
Karnataka				**ORF1a:E976D**	1	0.37				**ORF1a:E1136K**	1	0.1
Karnataka				**ORF1a:K2533R**	1	0.37				**ORF1a:A1851D**	1	0.1
Karnataka				**ORF1a:N2131D**	1	0.37				**ORF1a:G661S**	1	0.1
Karnataka				**ORF1a:H3076Y**	1	0.37				**ORF1a:P3447L**	1	0.1
Karnataka				**ORF1a:I2156V**	1	0.37				**ORF1a:G298D**	1	0.1
Karnataka				**ORF1a:V2588F**	1	0.37				**ORF1a:L2176M**	1	0.1
Karnataka				**ORF1a:F3138C**	1	0.37				**ORF1a:D1229G**	1	0.1
Karnataka				**ORF1a:P1158S**	1	0.37				**ORF1a:E972K**	1	0.1
Karnataka				**ORF1a:A3963V**	1	0.37				**ORF1a:G706C**	1	0.1
Karnataka				**ORF1a:L552I**	1	0.37				**ORF1a:G2294C**	1	0.1
Karnataka				**ORF1a:T2859I**	1	0.37				**ORF1a:V3975F**	1	0.1
Karnataka				**ORF1a:T3579I**	1	0.37				**ORF1a:P892S**	1	0.1
Karnataka				**ORF1a:V3653F**	1	0.37				**ORF1a:L2789S**	1	0.1
Karnataka				**ORF1a:G445V**	1	0.37				**ORF1a:Y3109H**	1	0.1
Karnataka				**ORF1a:M1586I**	1	0.37				**ORF1a:S2352R**	1	0.1
Karnataka				**ORF1a:T4129I**	1	0.37				**ORF1a:D3460N**	1	0.1
Karnataka				**ORF1a:V2646L**	1	0.37				**ORF1a:I3731V**	1	0.1
Karnataka				**ORF1a:A1043V**	1	0.37				**ORF1a:N126H**	1	0.1
Karnataka				**ORF1a:G1073R**	1	0.37				**ORF1a:T2967I**	1	0.1
Karnataka										**ORF1a:Q2574H**	1	0.1
Karnataka										**ORF1a:T1168A**	1	0.1
Karnataka										**ORF1a:V3048I**	1	0.1
Karnataka										**ORF1a:D981G**	1	0.1
Karnataka										**ORF1a:D1273G**	1	0.1
Karnataka										**ORF1a:S2273F**	1	0.1
Karnataka										**ORF1a:V3091I**	1	0.1
Karnataka										**ORF1a:G1125C**	1	0.1
Karnataka										**ORF1a:V1143F**	1	0.1
Karnataka										**ORF1a:V1751L**	1	0.1
Karnataka										**ORF1a:K2555N**	1	0.1
Karnataka										**ORF1a:T4161I**	1	0.1
Karnataka										**ORF1a:G1095R**	1	0.1
Karnataka										**ORF1a:Y2489H**	1	0.1
Karnataka										**ORF1a:T1242I**	1	0.1
Karnataka										**ORF1a:S3673N**	1	0.1
Karnataka										**ORF1a:C270F**	1	0.1
Karnataka										**ORF1a:T2274N**	1	0.1
Karnataka										**ORF1a:E754D**	1	0.1
Karnataka										**ORF1a:G826V**	1	0.1
Karnataka										**ORF1a:D828G**	1	0.1
Karnataka										**ORF1a:S2661F**	1	0.1
Karnataka										**ORF1a:I3618V**	1	0.1
Karnataka										**ORF1a:Q3859H**	1	0.1
Karnataka										**ORF1a:T512A**	1	0.1
Karnataka										**ORF1a:N2966S**	1	0.1
Karnataka										**ORF1a:V60F**	1	0.1
Karnataka										**ORF1a:C2210W**	1	0.1
Karnataka										**ORF1a:A1997V**	1	0.1
Karnataka										**ORF1a:T4217I**	1	0.1
Karnataka										**ORF1a:N310K**	1	0.1
Karnataka										**ORF1a:T2087I**	1	0.1
Karnataka										**ORF1a:V2051L**	1	0.1
Karnataka										**ORF1a:V1019I**	1	0.1
Karnataka										**ORF1a:A1043V**	1	0.1
Karnataka										**ORF1a:R1628L**	1	0.1
Karnataka										**ORF1a:R184C**	1	0.1
Karnataka										**ORF1a:P1692S**	1	0.1
Karnataka										**ORF1a:G1782C**	1	0.1
Karnataka										**ORF1a:V660A**	1	0.1
Karnataka										**ORF1a:V1211F**	1	0.1
Karnataka										**ORF1a:P1640S**	1	0.1

Karnataka										**ORF1a:P1977L**	1	0.1
Karnataka										**ORF1a:R2163L**	1	0.1
Karnataka										**ORF1a:V3976F**	1	0.1
Karnataka										**ORF1a:V3104I**	1	0.1
Karnataka										**ORF1a:A2249V**	1	0.1
Karnataka										**ORF1a:A4016V**	1	0.1
Karnataka										**ORF1a:N2767D**	1	0.1
Karnataka										**ORF1a:D1940Y**	1	0.1
Karnataka										**ORF1a:L1559F**	1	0.1
Karnataka										**ORF1a:V306F**	1	0.1
Karnataka										**ORF1a:V639A**	1	0.1
Karnataka										**ORF1a:S2285F**	1	0.1
Karnataka										**ORF1a:G493C**	1	0.1
Karnataka										**ORF1a:G1101V**	1	0.1
Karnataka										**ORF1a:T587I**	1	0.1
Karnataka										**ORF1a:R542L**	1	0.1
Karnataka										**ORF1a:L1680F**	1	0.1
Karnataka										**ORF1a:S3158G**	1	0.1
Karnataka										**ORF1a:K1208R**	1	0.1
Karnataka										**ORF1a:S1561F**	1	0.1
Karnataka										**ORF1a:G1591V**	1	0.1
Karnataka										**ORF1a:I2501V**	1	0.1
Karnataka										**ORF1a:T1241I**	1	0.1
Karnataka										**ORF1a:S1587L**	1	0.1
Karnataka										**ORF1a:V1199F**	1	0.1
Karnataka										**ORF1a:I2307V**	1	0.1
Karnataka										**ORF1a:Q314K**	1	0.1
Karnataka										**ORF1a:M2347V**	1	0.1
Karnataka										**ORF1a:R2115S**	1	0.1
Karnataka										**ORF1a:M2565I**	1	0.1
Karnataka										**ORF1a:I2385V**	1	0.1
Karnataka										**ORF1a:K2830Q**	1	0.1
Karnataka										**ORF1a:L2084I**	1	0.1
Karnataka										**ORF1a:G1850C**	1	0.1
Karnataka										**ORF1a:E940D**	1	0.1
Karnataka										**ORF1a:L1110F**	1	0.1
Karnataka										**ORF1a:V3053I**	1	0.1
Karnataka										**ORF1a:T1760I**	1	0.1
Karnataka										**ORF1a:V1020F**	1	0.1
Karnataka										**ORF1a:V1583F**	1	0.1
Karnataka										**ORF1a:T1653A**	1	0.1
Karnataka										**ORF1a:E1209D**	1	0.1
Karnataka										**ORF1a:V2783F**	1	0.1
Karnataka										**ORF1a:E995D**	1	0.1
Karnataka										**ORF1a:G514R**	1	0.1
Karnataka										**ORF1a:D691V**	1	0.1
Karnataka										**ORF1a:P1541S**	1	0.1
Karnataka										**ORF1a:V3653F**	1	0.1
Karnataka										**ORF1a:I2138V**	1	0.1
Karnataka										**ORF1a:P371L**	1	0.1
Karnataka										**ORF1a:W630C**	1	0.1
Karnataka										**ORF1a:N4167S**	1	0.1
Karnataka										**ORF1a:A208V**	1	0.1
Karnataka										**ORF1a:E913D**	1	0.1
Karnataka										**ORF1a:M920I**	1	0.1
Karnataka										**ORF1a:K2029N**	1	0.1
Karnataka										**ORF1a:N1137H**	1	0.1
Karnataka										**ORF1a:V3197L**	1	0.1
Karnataka										**ORF1a:P1786S**	1	0.1
Karnataka										**ORF1a:V2203L**	1	0.1
Karnataka										**ORF1a:T1303I**	1	0.1
Karnataka										**ORF1a:V823I**	1	0.1
Karnataka										**ORF1a:P4075S**	1	0.1
Karnataka										**ORF1a:A690V**	1	0.1
Karnataka										**ORF1a:K2204M**	1	0.1
Karnataka										**ORF1a:L2218S**	1	0.1
Karnataka										**ORF1a:T3032I**	1	0.1
Karnataka										**ORF1a:Y1489***	1	0.1
Karnataka										**ORF1a:S1517F**	1	0.1
Karnataka										**ORF1a:K1654N**	1	0.1
Karnataka										**ORF1a:P3359T**	1	0.1
Karnataka										**ORF1a:W2135R**	1	0.1
Karnataka										**ORF1a:N683S**	1	0.1

Karnataka										**ORF1a:N2736R**	1	0.1
Karnataka										**ORF1a:P1541L**	1	0.1
Karnataka										**ORF1a:T708I**	1	0.1
Karnataka										**ORF1a:T1017I**	1	0.1
Karnataka										**ORF1a:P2018L**	1	0.1
Karnataka										**ORF1a:T4355I**	1	0.1
Karnataka										**ORF1a:A3333S**	1	0.1
Karnataka										**ORF1a:L791P**	1	0.1
Karnataka										**ORF1a:S485R**	1	0.1
Karnataka										**ORF1a:G101D**	1	0.1
Karnataka										**ORF1a:Y185H**	1	0.1
Karnataka										**ORF1a:C720F**	1	0.1
Karnataka										**ORF1a:L969R**	1	0.1
Karnataka										**ORF1a:E1340D**	1	0.1
Karnataka										**ORF1a:K292N**	1	0.1
Karnataka										**ORF1a:D1036Y**	1	0.1
Karnataka										**ORF1a:V365I**	1	0.1
Karnataka										**ORF1a:G132V**	1	0.1
Karnataka										**ORF1a:M1771T**	1	0.1
Karnataka										**ORF1a:E754K**	1	0.1
Karnataka										**ORF1a:S1188L**	1	0.1
Karnataka										**ORF1a:K497R**	1	0.1
Karnataka										**ORF1a:I2961V**	1	0.1
Karnataka										**ORF1a:A372V**	1	0.1
Karnataka										**ORF1a:T3775A**	1	0.1
Karnataka										**ORF1a:P3371S**	1	0.1
Karnataka										**ORF1a:D2907G**	1	0.1
Karnataka										**ORF1a:P927L**	1	0.1
Karnataka										**ORF1a:T1908I**	1	0.1
Karnataka										**ORF1a:Q1943H**	1	0.1
Karnataka										**ORF1a:A3235V**	1	0.1
Karnataka										**ORF1a:S2242F**	1	0.1
Karnataka										**ORF1a:L4188S**	1	0.1
Karnataka										**ORF1a:T2791I**	1	0.1
Karnataka										**ORF1a:A3497T**	1	0.1
Karnataka										**ORF1a:A1432V**	1	0.1
Karnataka										**ORF1a:G2091D**	1	0.1
Karnataka										**ORF1a:P1921S**	1	0.1
Karnataka										**ORF1a:A3143V**	1	0.1
Karnataka										**ORF1a:L3313F**	1	0.1
Karnataka										**ORF1a:H1108Y**	1	0.1
Karnataka										**ORF1a:E1564A**	1	0.1
Karnataka										**ORF1a:G2545V**	1	0.1
Karnataka										**ORF1a:S3885F**	1	0.1
Karnataka										**ORF1a:G392S**	1	0.1
Karnataka										**ORF1a:A3457T**	1	0.1
Karnataka										**ORF1a:K225R**	1	0.1
Karnataka										**ORF1a:S166G**	1	0.1
Karnataka										**ORF1a:E1340K**	1	0.1
Karnataka										**ORF1a:T2274I**	1	0.1
Karnataka										**ORF1a:G993D**	1	0.1
Karnataka										**ORF1a:R3993L**	1	0.1
Karnataka										**ORF1a:E622K**	1	0.1
Karnataka										**ORF1a:T3356I**	1	0.1
Karnataka										**ORF1a:L3736P**	1	0.1
Karnataka										**ORF1a:T3090I**	1	0.1
Karnataka										**ORF1a:I740V**	1	0.1
Karnataka										**ORF1a:F1960L**	1	0.1
Karnataka										**ORF1a:R2336K**	1	0.1
Karnataka										**ORF1a:P4223L**	1	0.1
Karnataka										**ORF1a:D1962N**	1	0.1
Karnataka										**ORF1a:R299G**	1	0.1
Karnataka										**ORF1a:G2035R**	1	0.1
Karnataka										**ORF1a:A520V**	1	0.1
Karnataka										**ORF1a:V3524F**	1	0.1
Karnataka										**ORF1a:C2210F**	1	0.1
Karnataka										**ORF1a:T1881I**	1	0.1
Karnataka										**ORF1a:G2126S**	1	0.1
Karnataka										**ORF1a:S1520F**	1	0.1
Karnataka										**ORF1a:I2873M**	1	0.1
Karnataka										**ORF1a:I3476V**	1	0.1
Karnataka										**ORF1a:T3940I**	1	0.1
Karnataka										**ORF1a:T1240I**	1	0.1

Karnataka										**ORF1a:R724G**	1	0.1
Karnataka										**ORF1a:A690T**	1	0.1
Karnataka										**ORF1a:R232H**	1	0.1
Karnataka										**ORF1a:T677I**	1	0.1
Karnataka										**ORF1a:P1810L**	1	0.1
Karnataka										**ORF1a:V2019F**	1	0.1
Karnataka										**ORF1a:N2140D**	1	0.1
Karnataka										**ORF1a:K1655N**	1	0.1
Karnataka										**ORF1a:D147N**	1	0.1
Karnataka										**ORF1a:T50I**	1	0.1
Karnataka										**ORF1a:L1130F**	1	0.1
Karnataka										**ORF1a:I4291L**	1	0.1
Karnataka										**ORF1a:K1202N**	1	0.1
Karnataka										**ORF1a:T554I**	1	0.1
Karnataka										**ORF1a:K1855E**	1	0.1
Karnataka										**ORF1a:M731I**	1	0.1
Karnataka										**ORF1a:T2283I**	1	0.1
Karnataka										**ORF1a:D2299N**	1	0.1
Karnataka										**ORF1a:G661C**	1	0.1
Karnataka										**ORF1a:V3968I**	1	0.1
Karnataka										**ORF1a:L3338V**	1	0.1
Karnataka										**ORF1a:V1056L**	1	0.1
Karnataka										**ORF1a:G1764S**	1	0.1
Karnataka										**ORF1a:T2007I**	1	0.1
Karnataka										**ORF1a:A1473V**	1	0.1
Karnataka										**ORF1a:A260S**	1	0.1
Karnataka										**ORF1a:I280L**	1	0.1
Karnataka										**ORF1a:M2096I**	1	0.1
Karnataka										**ORF1a:H2698L**	1	0.1
Karnataka										**ORF1a:R1464W**	1	0.1
Karnataka										**ORF1a:A2828V**	1	0.1
Karnataka										**ORF1a:A3070T**	1	0.1
Karnataka										**ORF1a:I2786T**	1	0.1
Karnataka										**ORF1a:G3072S**	1	0.1
Karnataka										**ORF1a:D928Y**	1	0.1
Karnataka										**ORF1a:K2219N**	1	0.1
Karnataka										**ORF1a:K680N**	1	0.1
Karnataka										**ORF1a:A859V**	1	0.1
Karnataka										**ORF1a:E36D**	1	0.1
Karnataka										**ORF1a:T2153I**	1	0.1
Karnataka										**ORF1a:G1119S**	1	0.1
Karnataka										**ORF1a:V1295F**	1	0.1
Karnataka										**ORF1a:D953Y**	1	0.1
Karnataka										**ORF1a:R287S**	1	0.1
Karnataka										**ORF1a:E1413D**	1	0.1
Karnataka										**ORF1a:L3829F**	1	0.1
	**ORF1b**											
**Karnataka**	**Pre-Wave 1**			**Wave 1**			**Pre-Wave 2**			**Wave 2**		
Karnataka	**Mutation**	**Count**	**percent**	**Mutation**	**Count**	**percent**	**Mutation**	**Count**	**percent**	**Mutation**	**Count**	**percent**
Karnataka	**ORF1b:P314L**	150	69.44	**ORF1b:P314L**	265	98.51	**ORF1b:P314L**	199	94.76	**ORF1b:P314L**	1012	99.61
Karnataka	**ORF1b:A88V**	51	23.61	**ORF1b:L2523F**	82	30.48	**ORF1b:L2523F**	56	26.67	**ORF1b:P1000L**	687	67.62
Karnataka	**ORF1b:A1160T**	16	7.41	**ORF1b:A647S**	12	4.46	**ORF1b:M1352I**	23	10.95	**ORF1b:G662S**	614	60.43
Karnataka	**ORF1b:M2414I**	12	5.56	**ORF1b:V1092F**	7	2.6	**ORF1b:E1264D**	21	10	**ORF1b:A1918V**	531	52.26
Karnataka	**ORF1b:S1089A**	10	4.63	**ORF1b:G1561V**	5	1.86	**ORF1b:G662S**	20	9.52	**ORF1b:K2310R**	173	17.03
Karnataka	**ORF1b:D1869G**	9	4.17	**ORF1b:P218L**	5	1.86	**ORF1b:K2310R**	19	9.05	**ORF1b:M1352I**	141	13.88
Karnataka	**ORF1b:P369L**	9	4.17	**ORF1b:D1373Y**	5	1.86	**ORF1b:P1000L**	16	7.62	**ORF1b:S2312A**	86	8.46
Karnataka	**ORF1b:V1644L**	7	3.24	**ORF1b:D2142Y**	5	1.86	**ORF1b:A2306V**	15	7.14	**ORF1b:G1129C**	73	7.19
Karnataka	**ORF1b:A370G**	7	3.24	**ORF1b:K1383R**	5	1.86	**ORF1b:S2312A**	15	7.14	**ORF1b:H2285Y**	32	3.15
Karnataka	**ORF1b:M371L**	7	3.24	**ORF1b:T1655I**	5	1.86	**ORF1b:G1129C**	10	4.76	**ORF1b:P1570L**	26	2.56
Karnataka	**ORF1b:V2475G**	6	2.78	**ORF1b:A2131V**	4	1.49	**ORF1b:A1918V**	9	4.29	**ORF1b:L2523F**	23	2.26
Karnataka	**ORF1b:L1015***	4	1.85	**ORF1b:P314F**	4	1.49	**ORF1b:V666I**	6	2.86	**ORF1b:V666I**	16	1.57
Karnataka	**ORF1b:D2666E**	4	1.85	**ORF1b:P970L**	3	1.12	**ORF1b:M1156I**	5	2.38	**ORF1b:T2490M**	14	1.38
Karnataka	**ORF1b:V1092F**	3	1.39	**ORF1b:K2160N**	3	1.12	**ORF1b:K2557R**	5	2.38	**ORF1b:K2557R**	13	1.28
Karnataka	**ORF1b:S1159I**	3	1.39	**ORF1b:M2269I**	3	1.12	**ORF1b:P1821S**	5	2.38	**ORF1b:P218L**	12	1.18
Karnataka	**ORF1b:F2467S**	3	1.39	**ORF1b:K2557R**	3	1.12	**ORF1b:A2006V**	4	1.9	**ORF1b:P2116S**	11	1.08
Karnataka	**ORF1b:V2287I**	2	0.93	**ORF1b:T2432I**	2	0.74	**ORF1b:D51Y**	4	1.9	**ORF1b:K469N**	9	0.89
Karnataka	**ORF1b:R2257W**	2	0.93	**ORF1b:G1129C**	2	0.74	**ORF1b:Y2276C**	3	1.43	**ORF1b:S2312L**	9	0.89
Karnataka	**ORF1b:S1893F**	2	0.93	**ORF1b:M1573I**	2	0.74	**ORF1b:N734T**	3	1.43	**ORF1b:A373V**	8	0.79
Karnataka	**ORF1b:F2590Y**	2	0.93	**ORF1b:T2514I**	2	0.74	**ORF1b:P403S**	3	1.43	**ORF1b:P403S**	7	0.69
Karnataka	**ORF1b:L796F**	2	0.93	**ORF1b:A370V**	2	0.74	**ORF1b:L1314S**	3	1.43	**ORF1b:A1895T**	7	0.69

Karnataka	ORF1b:N1018T	2	0.93	ORF1b:A520V	2	0.74	ORF1b:T1137I	2	0.95	ORF1b:V2061L	7	0.69
Karnataka	ORF1b:N1113H	2	0.93	ORF1b:Q866K	2	0.74	ORF1b:A1643V	2	0.95	ORF1b:P1821S	6	0.59
Karnataka	ORF1b:A370V	2	0.93	ORF1b:I1579T	2	0.74	ORF1b:P1727L	2	0.95	ORF1b:R240S	5	0.49
Karnataka	ORF1b:G1568V	1	0.46	ORF1b:A2222V	2	0.74	ORF1b:P2531S	2	0.95	ORF1b:E1184D	5	0.49
Karnataka	ORF1b:P2321L	1	0.46	ORF1b:S1089L	2	0.74	ORF1b:I235T	2	0.95	ORF1b:T1545A	4	0.39
Karnataka	ORF1b:S2148F	1	0.46	ORF1b:Q2247H	2	0.74	ORF1b:W2586*	2	0.95	ORF1b:K1873N	4	0.39
Karnataka	ORF1b:V596A	1	0.46	ORF1b:D2666G	2	0.74	ORF1b:K50N	2	0.95	ORF1b:A2431V	4	0.39
Karnataka	ORF1b:V1649F	1	0.46	ORF1b:K1873N	2	0.74	ORF1b:D152Y	2	0.95	ORF1b:A1219S	4	0.39
Karnataka	ORF1b:T794I	1	0.46	ORF1b:A440V	2	0.74	ORF1b:P2434S	2	0.95	ORF1b:M1693I	4	0.39
Karnataka	ORF1b:P1223L	1	0.46	ORF1b:T1774I	2	0.74	ORF1b:P314F	2	0.95	ORF1b:M1839I	3	0.3
Karnataka	ORF1b:R107C	1	0.46	ORF1b:I2357M	2	0.74	ORF1b:S997P	2	0.95	ORF1b:R1315C	3	0.3
Karnataka	ORF1b:M897I	1	0.46	ORF1b:A545S	2	0.74	ORF1b:P1001S	2	0.95	ORF1b:M620I	3	0.3
Karnataka	ORF1b:W1090L	1	0.46	ORF1b:A1219T	2	0.74	ORF1b:S1023G	2	0.95	ORF1b:M1197I	3	0.3
Karnataka	ORF1b:E1091G	1	0.46	ORF1b:N487H	2	0.74	ORF1b:D729Y	2	0.95	ORF1b:V119I	3	0.3
Karnataka	ORF1b:V1092D	1	0.46	ORF1b:M2667I	2	0.74	ORF1b:A370V	2	0.95	ORF1b:I192V	3	0.3
Karnataka	ORF1b:G1093V	1	0.46	ORF1b:I1690V	2	0.74	ORF1b:P1570L	2	0.95	ORF1b:A1521V	3	0.3
Karnataka	ORF1b:K1094Y	1	0.46	ORF1b:L2017F	2	0.74	ORF1b:G1980E	1	0.48	ORF1b:A2513V	3	0.3
Karnataka	ORF1b:P1095K	1	0.46	ORF1b:I2136V	2	0.74	ORF1b:K1383R	1	0.48	ORF1b:A2222V	3	0.3
Karnataka	ORF1b:P1097Q	1	0.46	ORF1b:R2613C	2	0.74	ORF1b:S1661I	1	0.48	ORF1b:E1264D	3	0.3
Karnataka	ORF1b:G1940A	1	0.46	ORF1b:T1354I	2	0.74	ORF1b:L1701F	1	0.48	ORF1b:L966F	3	0.3
Karnataka	ORF1b:G1941V	1	0.46	ORF1b:L261F	2	0.74	ORF1b:A2222V	1	0.48	ORF1b:G2337S	3	0.3
Karnataka	ORF1b:A1949Y	1	0.46	ORF1b:E735D	2	0.74	ORF1b:L261F	1	0.48	ORF1b:D1306E	3	0.3
Karnataka	ORF1b:F1950T	1	0.46	ORF1b:P1645S	2	0.74	ORF1b:T2537I	1	0.48	ORF1b:G2610S	3	0.3
Karnataka	ORF1b:T1952L	1	0.46	ORF1b:V1649F	2	0.74	ORF1b:I1998V	1	0.48	ORF1b:V2371M	3	0.3
Karnataka	ORF1b:P1953R	1	0.46	ORF1b:Q813H	1	0.37	ORF1b:P160S	1	0.48	ORF1b:V1984L	3	0.3
Karnataka	ORF1b:Y2018Q	1	0.46	ORF1b:G977C	1	0.37	ORF1b:P2313L	1	0.48	ORF1b:M592I	3	0.3
Karnataka	ORF1b:L2019T	1	0.46	ORF1b:M371I	1	0.37	ORF1b:I2357M	1	0.48	ORF1b:D1042H	2	0.2
Karnataka	ORF1b:D2020I	1	0.46	ORF1b:F1657L	1	0.37	ORF1b:E913D	1	0.48	ORF1b:S961L	2	0.2
Karnataka	ORF1b:A2021*	1	0.46	ORF1b:D2183G	1	0.37	ORF1b:E2390K	1	0.48	ORF1b:T730I	2	0.2
Karnataka	ORF1b:N2023F	1	0.46	ORF1b:M1156I	1	0.37	ORF1b:A1302V	1	0.48	ORF1b:Q435R	2	0.2
Karnataka	ORF1b:M2024*	1	0.46	ORF1b:T797I	1	0.37	ORF1b:P218L	1	0.48	ORF1b:I2158L	2	0.2
Karnataka	ORF1b:M2025C	1	0.46	ORF1b:D870G	1	0.37	ORF1b:D2576G	1	0.48	ORF1b:P1095S	2	0.2
Karnataka	ORF1b:I2026C	1	0.46	ORF1b:D1848Y	1	0.37	ORF1b:E1728D	1	0.48	ORF1b:T2548I	2	0.2
Karnataka	ORF1b:S2027K	1	0.46	ORF1b:A1662V	1	0.37	ORF1b:D2179Y	1	0.48	ORF1b:N725D	2	0.2
Karnataka	ORF1b:A2028*	1	0.46	ORF1b:L1701F	1	0.37	ORF1b:R2613N	1	0.48	ORF1b:V1493L	2	0.2
Karnataka	ORF1b:F2030T	1	0.46	ORF1b:P1821S	1	0.37	ORF1b:P2256S	1	0.48	ORF1b:P1727L	2	0.2
Karnataka	ORF1b:S2031L	1	0.46	ORF1b:T1336I	1	0.37	ORF1b:T1453I	1	0.48	ORF1b:D131Y	2	0.2
Karnataka	ORF1b:L2032*	1	0.46	ORF1b:P1570L	1	0.37	ORF1b:T1050N	1	0.48	ORF1b:A1443V	2	0.2
Karnataka	ORF1b:R2049T	1	0.46	ORF1b:P909L	1	0.37	ORF1b:T1555I	1	0.48	ORF1b:V1271L	2	0.2
Karnataka	ORF1b:L2050G	1	0.46	ORF1b:M2462I	1	0.37	ORF1b:P1223L	1	0.48	ORF1b:D1183Y	2	0.2
Karnataka	ORF1b:Q2051*	1	0.46	ORF1b:T2376I	1	0.37	ORF1b:L1220I	1	0.48	ORF1b:M1156I	2	0.2
Karnataka	ORF1b:F2066K	1	0.46	ORF1b:T239I	1	0.37	ORF1b:S2363F	1	0.48	ORF1b:V248F	2	0.2
Karnataka	ORF1b:D2067S	1	0.46	ORF1b:C1880S	1	0.37	ORF1b:T1545A	1	0.48	ORF1b:A1884S	2	0.2
Karnataka	ORF1b:G2068*	1	0.46	ORF1b:T1274I	1	0.37	ORF1b:K1900R	1	0.48	ORF1b:T2163I	2	0.2
Karnataka	ORF1b:Q2069W	1	0.46	ORF1b:G2337V	1	0.37	ORF1b:D260N	1	0.48	ORF1b:V1706L	2	0.2
Karnataka	ORF1b:Q2070C	1	0.46	ORF1b:V1811I	1	0.37	ORF1b:A2513V	1	0.48	ORF1b:V1170F	2	0.2
Karnataka	ORF1b:G2071*	1	0.46	ORF1b:Q2556R	1	0.37	ORF1b:A1131S	1	0.48	ORF1b:H1838Y	2	0.2
Karnataka	ORF1b:E2072C	1	0.46	ORF1b:D837H	1	0.37	ORF1b:E269D	1	0.48	ORF1b:E1728D	2	0.2
Karnataka	ORF1b:V2073R	1	0.46	ORF1b:V2073L	1	0.37	ORF1b:D1127Y	1	0.48	ORF1b:T1522I	2	0.2
Karnataka	ORF1b:P2074I	1	0.46	ORF1b:A545V	1	0.37	ORF1b:Y2625Q	1	0.48	ORF1b:C1740F	2	0.2
Karnataka	ORF1b:S2076*	1	0.46	ORF1b:P2531S	1	0.37	ORF1b:F2627I	1	0.48	ORF1b:S2198I	2	0.2
Karnataka	ORF1b:I2077K	1	0.46	ORF1b:A1219S	1	0.37	ORF1b:W2628I	1	0.48	ORF1b:V1644I	2	0.2
Karnataka	ORF1b:I2078*	1	0.46	ORF1b:D1042G	1	0.37	ORF1b:N2630E	1	0.48	ORF1b:L1220F	2	0.2
Karnataka	ORF1b:T2098S	1	0.46	ORF1b:C455F	1	0.37	ORF1b:T2631N	1	0.48	ORF1b:A647S	2	0.2
Karnataka	ORF1b:L2100R	1	0.46	ORF1b:G662S	1	0.37	ORF1b:P2633R	1	0.48	ORF1b:T797I	2	0.2
Karnataka	ORF1b:P2101G	1	0.46	ORF1b:M1586V	1	0.37	ORF1b:I2634V	1	0.48	ORF1b:A517V	2	0.2
Karnataka	ORF1b:V2102E	1	0.46	ORF1b:D2333Y	1	0.37	ORF1b:Q2635V	1	0.48	ORF1b:E2544D	2	0.2
Karnataka	ORF1b:V2104Y	1	0.46	ORF1b:G2662S	1	0.37	ORF1b:Y2015H	1	0.48	ORF1b:T1162I	2	0.2
Karnataka	ORF1b:A2105C	1	0.46	ORF1b:S2312P	1	0.37	ORF1b:M2322K	1	0.48	ORF1b:P2321L	2	0.2
Karnataka	ORF1b:F2106D	1	0.46	ORF1b:A2222T	1	0.37	ORF1b:W2628R	1	0.48	ORF1b:D1381Y	2	0.2
Karnataka	ORF1b:E2107L	1	0.46	ORF1b:N2535D	1	0.37	ORF1b:V2685N	1	0.48	ORF1b:T17I	2	0.2
Karnataka	ORF1b:L2108G	1	0.46	ORF1b:V896I	1	0.37	ORF1b:V2686*	1	0.48	ORF1b:D1251N	2	0.2
Karnataka	ORF1b:W2109L	1	0.46	ORF1b:V2371L	1	0.37	ORF1b:G2610S	1	0.48	ORF1b:N1426Y	2	0.2
Karnataka	ORF1b:A2110Q	1	0.46	ORF1b:M1596I	1	0.37	ORF1b:H2285Y	1	0.48	ORF1b:L1681F	2	0.2
Karnataka	ORF1b:N2125C	1	0.46	ORF1b:T2099I	1	0.37	ORF1b:N2574S	1	0.48	ORF1b:R164H	2	0.2
Karnataka	ORF1b:L2126S	1	0.46	ORF1b:H2388Y	1	0.37	ORF1b:G814C	1	0.48	ORF1b:T1404M	2	0.2
Karnataka	ORF1b:G2127S	1	0.46	ORF1b:D2429N	1	0.37	ORF1b:E1514G	1	0.48	ORF1b:A576T	2	0.2
Karnataka	ORF1b:V2128T	1	0.46	ORF1b:Y23C	1	0.37	ORF1b:R2652S	1	0.48	ORF1b:T1333I	2	0.2
Karnataka	ORF1b:D2129Y	1	0.46	ORF1b:H2065Y	1	0.37	ORF1b:D731Y	1	0.48	ORF1b:P1452S	2	0.2
Karnataka	ORF1b:A2131D	1	0.46	ORF1b:D727Y	1	0.37	ORF1b:S1273L	1	0.48	ORF1b:T76I	2	0.2
Karnataka	ORF1b:A2132L	1	0.46	ORF1b:D1267N	1	0.37	ORF1b:M1573I	1	0.48	ORF1b:I1611V	2	0.2
Karnataka	ORF1b:N2133C	1	0.46	ORF1b:A2513V	1	0.37	ORF1b:V2061L	1	0.48	ORF1b:T2488M	2	0.2
Karnataka	ORF1b:G2151T	1	0.46	ORF1b:M1397I	1	0.37	ORF1b:V1649F	1	0.48	ORF1b:T1050N	2	0.2

Karnataka	**ORF1b:V2152H**	1	0.46	**ORF1b:Q2181H**	1	0.37	**ORF1b:I1690L**	1	0.48	**ORF1b:A2105S**	2	0.2
Karnataka	**ORF1b:M2155S**	1	0.46	**ORF1b:P1342S**	1	0.37	**ORF1b:V2117L**	1	0.48	**ORF1b:M2025I**	2	0.2
Karnataka	**ORF1b:T2156R**	1	0.46	**ORF1b:S2430I**	1	0.37	**ORF1b:V2073L**	1	0.48	**ORF1b:T1067I**	1	0.1
Karnataka	**ORF1b:D2157L**	1	0.46	**ORF1b:V2117L**	1	0.37	**ORF1b:N2535S**	1	0.48	**ORF1b:H1979Y**	1	0.1
Karnataka	**ORF1b:A2159***	1	0.46	**ORF1b:D2179Y**	1	0.37	**ORF1b:T393I**	1	0.48	**ORF1b:D2423Y**	1	0.1
Karnataka	**ORF1b:K2161C**	1	0.46	**ORF1b:K402R**	1	0.37	**ORF1b:V1149L**	1	0.48	**ORF1b:W153C**	1	0.1
Karnataka	**ORF1b:T2163Y**	1	0.46	**ORF1b:N129S**	1	0.37	**ORF1b:D1903Y**	1	0.48	**ORF1b:V1639A**	1	0.1
Karnataka	**ORF1b:E2164R**	1	0.46	**ORF1b:A1428S**	1	0.37	**ORF1b:Q1546H**	1	0.48	**ORF1b:S424R**	1	0.1
Karnataka	**ORF1b:T2165R**	1	0.46	**ORF1b:A1884S**	1	0.37	**ORF1b:K89E**	1	0.48	**ORF1b:H2011R**	1	0.1
Karnataka	**ORF1b:I2166***	1	0.46	**ORF1b:A2006V**	1	0.37				**ORF1b:V1021F**	1	0.1
Karnataka	**ORF1b:D2183***	1	0.46	**ORF1b:T861I**	1	0.37				**ORF1b:D1869G**	1	0.1
Karnataka	**ORF1b:L2184R**	1	0.46	**ORF1b:V1691A**	1	0.37				**ORF1b:A414V**	1	0.1
Karnataka	**ORF1b:N2187S**	1	0.46	**ORF1b:R188Q**	1	0.37				**ORF1b:R945K**	1	0.1
Karnataka	**ORF1b:A2188R**	1	0.46	**ORF1b:T1038I**	1	0.37				**ORF1b:F2353V**	1	0.1
Karnataka	**ORF1b:R2189K**	1	0.46	**ORF1b:T792I**	1	0.37				**ORF1b:D1825Y**	1	0.1
Karnataka	**ORF1b:G2191T**	1	0.46	**ORF1b:D436Y**	1	0.37				**ORF1b:C142Y**	1	0.1
Karnataka	**ORF1b:A2211Y**	1	0.46	**ORF1b:N1653D**	1	0.37				**ORF1b:T1050I**	1	0.1
Karnataka	**ORF1b:L2213E**	1	0.46	**ORF1b:P976L**	1	0.37				**ORF1b:H1168Y**	1	0.1
Karnataka	**ORF1b:N2214***	1	0.46	**ORF1b:D2361Y**	1	0.37				**ORF1b:S1273L**	1	0.1
Karnataka	**ORF1b:G2215K**	1	0.46	**ORF1b:P1427S**	1	0.37				**ORF1b:F92L**	1	0.1
Karnataka	**ORF1b:V2216F**	1	0.46	**ORF1b:R2308C**	1	0.37				**ORF1b:I527T**	1	0.1
Karnataka	**ORF1b:L2218R**	1	0.46	**ORF1b:M2025T**	1	0.37				**ORF1b:A1392T**	1	0.1
Karnataka	**ORF1b:V2236***	1	0.46	**ORF1b:P1675L**	1	0.37				**ORF1b:A88S**	1	0.1
Karnataka	**ORF1b:V2237T**	1	0.46	**ORF1b:V2060F**	1	0.37				**ORF1b:G2610N**	1	0.1
Karnataka	**ORF1b:Q2239E**	1	0.46	**ORF1b:P2633L**	1	0.37				**ORF1b:R524H**	1	0.1
Karnataka	**ORF1b:L2240S**	1	0.46	**ORF1b:T67I**	1	0.37				**ORF1b:E720D**	1	0.1
Karnataka	**ORF1b:P2241N**	1	0.46	**ORF1b:M657I**	1	0.37				**ORF1b:S316I**	1	0.1
Karnataka	**ORF1b:E2242G**	1	0.46	**ORF1b:L1504F**	1	0.37				**ORF1b:A1609S**	1	0.1
Karnataka	**ORF1b:T2243N**	1	0.46	**ORF1b:A2306V**	1	0.37				**ORF1b:P1594L**	1	0.1
Karnataka	**ORF1b:Y2244***	1	0.46	**ORF1b:V2061L**	1	0.37				**ORF1b:A1868V**	1	0.1
Karnataka	**ORF1b:T2246V**	1	0.46	**ORF1b:A1160V**	1	0.37				**ORF1b:S1089L**	1	0.1
Karnataka	**ORF1b:Q2247***	1	0.46							**ORF1b:R2613S**	1	0.1
Karnataka	**ORF1b:S2248I**	1	0.46							**ORF1b:Q1546L**	1	0.1
Karnataka	**ORF1b:N2250R**	1	0.46							**ORF1b:E2107D**	1	0.1
Karnataka	**ORF1b:Q2252C**	1	0.46							**ORF1b:I162V**	1	0.1
Karnataka	**ORF1b:E2253L**	1	0.46							**ORF1b:Q215H**	1	0.1
Karnataka	**ORF1b:F2254R**	1	0.46							**ORF1b:A2575V**	1	0.1
Karnataka	**ORF1b:K2255T**	1	0.46							**ORF1b:C554R**	1	0.1
Karnataka	**ORF1b:P2256Y**	1	0.46							**ORF1b:A370V**	1	0.1
Karnataka	**ORF1b:Q2259***	1	0.46							**ORF1b:I1921V**	1	0.1
Karnataka	**ORF1b:M2260S**	1	0.46							**ORF1b:V2117A**	1	0.1
Karnataka	**ORF1b:E2261V**	1	0.46							**ORF1b:V1841I**	1	0.1
Karnataka	**ORF1b:I2262R**	1	0.46							**ORF1b:I1598S**	1	0.1
Karnataka	**ORF1b:D2263W**	1	0.46							**ORF1b:P1879S**	1	0.1
Karnataka	**ORF1b:E2284***	1	0.46							**ORF1b:A1895V**	1	0.1
Karnataka	**ORF1b:H2285G**	1	0.46							**ORF1b:R240M**	1	0.1
Karnataka	**ORF1b:V2287S**	1	0.46							**ORF1b:L1846S**	1	0.1
Karnataka	**ORF1b:D2290Q**	1	0.46							**ORF1b:P2321S**	1	0.1
Karnataka	**ORF1b:F2291Y**	1	0.46							**ORF1b:A762S**	1	0.1
Karnataka	**ORF1b:I2303***	1	0.46							**ORF1b:T2453I**	1	0.1
Karnataka	**ORF1b:G2304K**	1	0.46							**ORF1b:I2509V**	1	0.1
Karnataka	**ORF1b:K2307N**	1	0.46							**ORF1b:H801Y**	1	0.1
Karnataka	**ORF1b:M2322I**	1	0.46							**ORF1b:K2579N**	1	0.1
Karnataka	**ORF1b:D2323***	1	0.46							**ORF1b:G170S**	1	0.1
Karnataka	**ORF1b:S2324V**	1	0.46							**ORF1b:M2667I**	1	0.1
Karnataka	**ORF1b:T2325C**	1	0.46							**ORF1b:G2068E**	1	0.1
Karnataka	**ORF1b:K2327F**	1	0.46							**ORF1b:Q813H**	1	0.1
Karnataka	**ORF1b:N2328C**	1	0.46							**ORF1b:A440V**	1	0.1
Karnataka	**ORF1b:V2345F**	1	0.46							**ORF1b:A2518T**	1	0.1
Karnataka	**ORF1b:D2347Y**	1	0.46							**ORF1b:V711A**	1	0.1
Karnataka	**ORF1b:L2348A**	1	0.46							**ORF1b:T2432I**	1	0.1
Karnataka	**ORF1b:K2367S**	1	0.46							**ORF1b:P2612T**	1	0.1
Karnataka	**ORF1b:K2385A**	1	0.46							**ORF1b:D51Y**	1	0.1
Karnataka	**ORF1b:D2386T**	1	0.46							**ORF1b:N1025S**	1	0.1
Karnataka	**ORF1b:H2388K**	1	0.46							**ORF1b:T2134N**	1	0.1
Karnataka	**ORF1b:Q2403***	1	0.46							**ORF1b:A176V**	1	0.1
Karnataka	**ORF1b:G2405S**	1	0.46							**ORF1b:A1643V**	1	0.1
Karnataka	**ORF1b:V2406K**	1	0.46							**ORF1b:V2178F**	1	0.1
Karnataka	**ORF1b:A2407L**	1	0.46							**ORF1b:I1598T**	1	0.1
Karnataka	**ORF1b:M2408W**	1	0.46							**ORF1b:S997P**	1	0.1
Karnataka	**ORF1b:P2409***	1	0.46							**ORF1b:D275Y**	1	0.1
Karnataka	**ORF1b:N2410***	1	0.46							**ORF1b:I623V**	1	0.1
Karnataka	**ORF1b:L2411C**	1	0.46							**ORF1b:V1811F**	1	0.1

Karnataka	**ORF1b:Y2412N**	1	0.46							**ORF1b:E735G**	1	0.1
Karnataka	**ORF1b:K2413I**	1	0.46							**ORF1b:D2270Y**	1	0.1
Karnataka	**ORF1b:M2414T**	1	0.46							**ORF1b:P1427S**	1	0.1
Karnataka	**ORF1b:Q2415***	1	0.46							**ORF1b:A1259T**	1	0.1
Karnataka	**ORF1b:M2417H**	1	0.46							**ORF1b:T1424I**	1	0.1
Karnataka	**ORF1b:L2418N**	1	0.46							**ORF1b:A869V**	1	0.1
Karnataka	**ORF1b:L2419D**	1	0.46							**ORF1b:R1052K**	1	0.1
Karnataka	**ORF1b:N2426H**	1	0.46							**ORF1b:I1515V**	1	0.1
Karnataka	**ORF1b:Y2427I**	1	0.46							**ORF1b:S2363F**	1	0.1
Karnataka	**ORF1b:G2428N**	1	0.46							**ORF1b:D1848Y**	1	0.1
Karnataka	**ORF1b:D2429I**	1	0.46							**ORF1b:V2237F**	1	0.1
Karnataka	**ORF1b:A2431C**	1	0.46							**ORF1b:V2368F**	1	0.1
Karnataka	**ORF1b:N2440***	1	0.46							**ORF1b:M1582I**	1	0.1
Karnataka	**ORF1b:V2441Y**	1	0.46							**ORF1b:H1213Y**	1	0.1
Karnataka	**ORF1b:A2442E**	1	0.46							**ORF1b:A1918I**	1	0.1
Karnataka	**ORF1b:K2443S**	1	0.46							**ORF1b:S1779N**	1	0.1
Karnataka	**ORF1b:Q2446F**	1	0.46							**ORF1b:A1033S**	1	0.1
Karnataka	**ORF1b:L2447W**	1	0.46							**ORF1b:A1877V**	1	0.1
Karnataka	**ORF1b:Q2449R**	1	0.46							**ORF1b:V138I**	1	0.1
Karnataka	**ORF1b:L2451S**	1	0.46							**ORF1b:Q1589H**	1	0.1
Karnataka	**ORF1b:N2452C**	1	0.46							**ORF1b:A549S**	1	0.1
Karnataka	**ORF1b:T2453F**	1	0.46							**ORF1b:E1242D**	1	0.1
Karnataka	**ORF1b:L2454K**	1	0.46							**ORF1b:G2297C**	1	0.1
Karnataka	**ORF1b:I2465V**	1	0.46							**ORF1b:D2183E**	1	0.1
Karnataka	**ORF1b:H2466A**	1	0.46							**ORF1b:P234S**	1	0.1
Karnataka	**ORF1b:F2467Y**	1	0.46							**ORF1b:E427G**	1	0.1
Karnataka	**ORF1b:P2487***	1	0.46							**ORF1b:P2256L**	1	0.1
Karnataka	**ORF1b:T2488M**	1	0.46							**ORF1b:V464F**	1	0.1
Karnataka	**ORF1b:T2507***	1	0.46							**ORF1b:G1450R**	1	0.1
Karnataka	**ORF1b:I2509***	1	0.46							**ORF1b:V1644L**	1	0.1
Karnataka	**ORF1b:G2510R**	1	0.46							**ORF1b:V1836I**	1	0.1
Karnataka	**ORF1b:D2511G**	1	0.46							**ORF1b:C2341F**	1	0.1
Karnataka	**ORF1b:C2512F**	1	0.46							**ORF1b:M2417I**	1	0.1
Karnataka	**ORF1b:A2513F**	1	0.46							**ORF1b:K1094R**	1	0.1
Karnataka	**ORF1b:T2514H**	1	0.46							**ORF1b:A2306S**	1	0.1
Karnataka	**ORF1b:V2515L**	1	0.46							**ORF1b:P1851S**	1	0.1
Karnataka	**ORF1b:K2532L**	1	0.46							**ORF1b:T1178I**	1	0.1
Karnataka	**ORF1b:T2533W**	1	0.46							**ORF1b:S2324I**	1	0.1
Karnataka	**ORF1b:K2534V**	1	0.46							**ORF1b:T1339I**	1	0.1
Karnataka	**ORF1b:N2535Y**	1	0.46							**ORF1b:L2213F**	1	0.1
Karnataka	**ORF1b:V2536T**	1	0.46							**ORF1b:I1498M**	1	0.1
Karnataka	**ORF1b:N2540C**	1	0.46							**ORF1b:S2339F**	1	0.1
Karnataka	**ORF1b:D2541***	1	0.46							**ORF1b:G1772C**	1	0.1
Karnataka	**ORF1b:K2543L**	1	0.46							**ORF1b:K1828N**	1	0.1
Karnataka	**ORF1b:E2544***	1	0.46							**ORF1b:S424C**	1	0.1
Karnataka	**ORF1b:G2545A**	1	0.46							**ORF1b:V486I**	1	0.1
Karnataka	**ORF1b:F2546H**	1	0.46							**ORF1b:V980F**	1	0.1
Karnataka	**ORF1b:F2547G**	1	0.46							**ORF1b:M2260V**	1	0.1
Karnataka	**ORF1b:A2559R**	1	0.46							**ORF1b:D729Y**	1	0.1
Karnataka	**ORF1b:L2560M**	1	0.46							**ORF1b:N1962S**	1	0.1
Karnataka	**ORF1b:G2561V**	1	0.46							**ORF1b:K1017R**	1	0.1
Karnataka	**ORF1b:G2562D**	1	0.46							**ORF1b:A366V**	1	0.1
Karnataka	**ORF1b:Y2288S**	1	0.46							**ORF1b:V1649F**	1	0.1
Karnataka	**ORF1b:V329F**	1	0.46							**ORF1b:H2146R**	1	0.1
Karnataka	**ORF1b:T1470I**	1	0.46							**ORF1b:K50N**	1	0.1
Karnataka	**ORF1b:A1877V**	1	0.46							**ORF1b:V1021I**	1	0.1
Karnataka	**ORF1b:L2017F**	1	0.46							**ORF1b:Q1546H**	1	0.1
Karnataka	**ORF1b:S2312L**	1	0.46							**ORF1b:M454I**	1	0.1
Karnataka	**ORF1b:D1899Y**	1	0.46							**ORF1b:K1849R**	1	0.1
Karnataka	**ORF1b:E1728D**	1	0.46							**ORF1b:M624L**	1	0.1
Karnataka	**ORF1b:D2318Y**	1	0.46							**ORF1b:V839L**	1	0.1
Karnataka	**ORF1b:D2323G**	1	0.46							**ORF1b:G2151S**	1	0.1
Karnataka	**ORF1b:Y78C**	1	0.46							**ORF1b:Q866R**	1	0.1
Karnataka	**ORF1b:T2156S**	1	0.46							**ORF1b:Q866K**	1	0.1
Karnataka	**ORF1b:M1573I**	1	0.46							**ORF1b:A647V**	1	0.1
Karnataka	**ORF1b:A2132V**	1	0.46							**ORF1b:G814V**	1	0.1
Karnataka	**ORF1b:R2629V**	1	0.46							**ORF1b:P1414S**	1	0.1
Karnataka	**ORF1b:N2630***	1	0.46							**ORF1b:M1573T**	1	0.1
Karnataka	**ORF1b:T2631E**	1	0.46							**ORF1b:A1225S**	1	0.1
Karnataka	**ORF1b:N2632T**	1	0.46							**ORF1b:I687S**	1	0.1
Karnataka	**ORF1b:P2633A**	1	0.46							**ORF1b:Y1944***	1	0.1
Karnataka	**ORF1b:A1847V**	1	0.46							**ORF1b:H2388R**	1	0.1
Karnataka	**ORF1b:G977S**	1	0.46							**ORF1b:I1468V**	1	0.1

Karnataka	**ORF1b:P2477A**	1	0.46							**ORF1b:E1808D**	1	0.1
Karnataka	**ORF1b:T981A**	1	0.46							**ORF1b:V1092F**	1	0.1
Karnataka	**ORF1b:L629F**	1	0.46							**ORF1b:Q435H**	1	0.1
Karnataka	**ORF1b:E1288D**	1	0.46							**ORF1b:H807Y**	1	0.1
Karnataka	**ORF1b:M454T**	1	0.46							**ORF1b:T1290P**	1	0.1
Karnataka	**ORF1b:M657I**	1	0.46							**ORF1b:P1664S**	1	0.1
Karnataka	**ORF1b:T2134F**	1	0.46							**ORF1b:H1087Y**	1	0.1
Karnataka	**ORF1b:I2136M**	1	0.46							**ORF1b:A1831V**	1	0.1
Karnataka	**ORF1b:N1102H**	1	0.46							**ORF1b:S2338L**	1	0.1
Karnataka	**ORF1b:I2346L**	1	0.46							**ORF1b:T310I**	1	0.1
Karnataka	**ORF1b:R2463S**	1	0.46							**ORF1b:I2524V**	1	0.1
Karnataka	**ORF1b:K1094I**	1	0.46							**ORF1b:D260G**	1	0.1
Karnataka	**ORF1b:H2571D**	1	0.46							**ORF1b:T250N**	1	0.1
Karnataka	**ORF1b:N2574K**	1	0.46							**ORF1b:A1620V**	1	0.1
Karnataka	**ORF1b:S2027***	1	0.46							**ORF1b:G814C**	1	0.1
Karnataka	**ORF1b:P611S**	1	0.46							**ORF1b:T1739I**	1	0.1
Karnataka	**ORF1b:T1424E**	1	0.46							**ORF1b:E269D**	1	0.1
Karnataka	**ORF1b:T2654A**	1	0.46							**ORF1b:N1519S**	1	0.1
Karnataka	**ORF1b:K1947T**	1	0.46							**ORF1b:I828T**	1	0.1
Karnataka	**ORF1b:V87F**	1	0.46							**ORF1b:A1326S**	1	0.1
Karnataka	**ORF1b:R712H**	1	0.46							**ORF1b:V2073L**	1	0.1
Karnataka	**ORF1b:P1727A**	1	0.46							**ORF1b:H604D**	1	0.1
Karnataka	**ORF1b:E1728A**	1	0.46							**ORF1b:T1139N**	1	0.1
Karnataka	**ORF1b:G2191C**	1	0.46							**ORF1b:V1870L**	1	0.1
Karnataka	**ORF1b:A576V**	1	0.46							**ORF1b:A1749V**	1	0.1
Karnataka	**ORF1b:S997L**	1	0.46							**ORF1b:P2531S**	1	0.1
Karnataka	**ORF1b:T2134A**	1	0.46							**ORF1b:L2560F**	1	0.1
Karnataka	**ORF1b:F2584I**	1	0.46							**ORF1b:R188Q**	1	0.1
Karnataka	**ORF1b:T903I**	1	0.46							**ORF1b:K1835N**	1	0.1
Karnataka	**ORF1b:S904Q**	1	0.46							**ORF1b:A2132V**	1	0.1
Karnataka	**ORF1b:R905K**	1	0.46							**ORF1b:V290F**	1	0.1
Karnataka	**ORF1b:Y906L**	1	0.46							**ORF1b:T1137I**	1	0.1
Karnataka	**ORF1b:T1019L**	1	0.46							**ORF1b:S2212I**	1	0.1
Karnataka	**ORF1b:K2255E**	1	0.46							**ORF1b:V2086F**	1	0.1
Karnataka	**ORF1b:L2641F**	1	0.46							**ORF1b:I2554L**	1	0.1
Karnataka	**ORF1b:K2650Q**	1	0.46							**ORF1b:V181I**	1	0.1
Karnataka										**ORF1b:G661V**	1	0.1
Karnataka										**ORF1b:A1291S**	1	0.1
Karnataka										**ORF1b:T1336I**	1	0.1
Karnataka										**ORF1b:V464I**	1	0.1
Karnataka										**ORF1b:P2169L**	1	0.1
Karnataka										**ORF1b:D275E**	1	0.1
Karnataka										**ORF1b:D2090Y**	1	0.1
Karnataka										**ORF1b:R2684I**	1	0.1
Karnataka										**ORF1b:P1570H**	1	0.1
Karnataka										**ORF1b:V2199F**	1	0.1
	**Spike (S)**											
**Karnataka**	**Pre-Wave 1**			**Wave 1**			**Pre-Wave 2**			**Wave 2**		
Karnataka	**Mutation**	**Count**	**percent**	**Mutation**	**Count**	**percent**	**Mutation**	**Count**	**percent**	**Mutation**	**Count**	**percent**
Karnataka	**S:D614G**	144	66.67	**S:D614G**	264	98.14	**S:D614G**	206	98.1	**S:D614G**	1010	99.41
Karnataka	**S:K77M**	7	3.24	**S:N440K**	73	27.14	**S:L452R**	39	18.57	**S:P681R**	869	85.53
Karnataka	**S:D848G**	6	2.78	**S:P681H**	26	9.67	**S:N440K**	37	17.62	**S:L452R**	860	84.65
Karnataka	**S:L849P**	6	2.78	**S:N501Y**	15	5.58	**S:P681R**	36	17.14	**S:T478K**	719	70.77
Karnataka	**S:D1084Y**	5	2.31	**S:T716I**	15	5.58	**S:P681H**	33	15.71	**S:T19R**	714	70.28
Karnataka	**S:D839Y**	3	1.39	**S:S982A**	15	5.58	**S:N501Y**	31	14.76	**S:D950N**	659	64.86
Karnataka	**S:R765L**	3	1.39	**S:D1118H**	15	5.58	**S:A570D**	30	14.29	**S:R158G**	459	45.18
Karnataka	**S:G184V**	3	1.39	**S:A570D**	14	5.2	**S:S982A**	29	13.81	**S:E484Q**	143	14.07
Karnataka	**S:A1078S**	2	0.93	**S:D138Y**	9	3.35	**S:D1118H**	29	13.81	**S:Q1071H**	123	12.11
Karnataka	**S:Q677H**	2	0.93	**S:T22I**	7	2.6	**S:T716I**	28	13.33	**S:E154K**	96	9.45
Karnataka	**S:K1045N**	2	0.93	**S:K1073N**	7	2.6	**S:E484Q**	23	10.95	**S:A222V**	89	8.76
Karnataka	**S:S98F**	1	0.46	**S:H49Y**	5	1.86	**S:Q1071H**	21	10	**S:G142D**	88	8.66
Karnataka	**S:M1229I**	1	0.46	**S:G257S**	4	1.49	**S:N679K**	19	9.05	**S:P681H**	80	7.87
Karnataka	**S:D138Y**	1	0.46	**S:Q613H**	4	1.49	**S:D950N**	17	8.1	**S:N501Y**	75	7.38
Karnataka	**S:N481D**	1	0.46	**S:A222V**	4	1.49	**S:T478K**	16	7.62	**S:A570D**	75	7.38
Karnataka	**S:D178N**	1	0.46	**S:D1139H**	3	1.12	**S:E154K**	12	5.71	**S:S982A**	75	7.38
Karnataka	**S:S967G**	1	0.46	**S:L242F**	3	1.12	**S:T19R**	10	4.76	**S:T716I**	74	7.28
Karnataka	**S:L981P**	1	0.46	**S:I1227T**	3	1.12	**S:E484K**	9	4.29	**S:D1118H**	73	7.19
Karnataka	**S:P1263L**	1	0.46	**S:Q677H**	3	1.12	**S:V382L**	9	4.29	**S:T95I**	66	6.5

Karnataka	**S:A701S**	1	0.46	**S:Q675H**	3	1.12	**S:D1153Y**	8	3.81	**S:H1101D**	47	4.63
Karnataka	**S:H49Y**	1	0.46	**S:G1219C**	3	1.12	**S:G1219C**	8	3.81	**S:V382L**	18	1.77
Karnataka	**S:C1250F**	1	0.46	**S:M153I**	3	1.12	**S:G257S**	7	3.33	**S:D1153Y**	18	1.77
Karnataka	**S:L368P**	1	0.46	**S:V213A**	3	1.12	**S:D138Y**	6	2.86	**S:Q218H**	15	1.48
Karnataka	**S:A647S**	1	0.46	**S:F490S**	3	1.12	**S:Q218H**	5	2.38	**S:K77T**	12	1.18
Karnataka	**S:P1162S**	1	0.46	**S:Q1208H**	2	0.74	**S:Q677H**	5	2.38	**S:V1104L**	10	0.98
Karnataka	**S:A243V**	1	0.46	**S:V1264L**	2	0.74	**S:H1101D**	5	2.38	**S:L18F**	10	0.98
Karnataka	**S:V36S**	1	0.46	**S:L5F**	2	0.74	**S:M1237I**	4	1.9	**S:A67V**	6	0.59
Karnataka	**S:H1048Q**	1	0.46	**S:T95I**	2	0.74	**S:F157L**	4	1.9	**S:Q677H**	6	0.59
Karnataka	**S:F565S**	1	0.46	**S:L176F**	2	0.74	**S:D80A**	4	1.9	**S:A653V**	5	0.49
Karnataka	**S:G566E**	1	0.46	**S:A520S**	2	0.74	**S:D215G**	4	1.9	**S:F490L**	5	0.49
Karnataka	**S:R567M**	1	0.46	**S:L18F**	2	0.74	**S:K417N**	4	1.9	**S:G446V**	5	0.49
Karnataka	**S:D568C**	1	0.46	**S:G75R**	2	0.74	**S:A701V**	4	1.9	**S:M1229I**	5	0.49
Karnataka	**S:A570R**	1	0.46	**S:T791I**	2	0.74	**S:L5F**	3	1.43	**S:E484K**	5	0.49
Karnataka	**S:T572A**	1	0.46	**S:T95N**	2	0.74	**S:A222V**	3	1.43	**S:N679K**	4	0.39
Karnataka	**S:E96D**	1	0.46	**S:L822F**	2	0.74	**S:E96G**	3	1.43	**S:N440K**	4	0.39
Karnataka	**S:I1179V**	1	0.46	**S:K1191N**	2	0.74	**S:A831S**	3	1.43	**S:D138Y**	4	0.39
Karnataka	**S:Q613H**	1	0.46	**S:P26S**	1	0.37	**S:S477N**	3	1.43	**S:D228Y**	4	0.39
Karnataka	**S:P728S**	1	0.46	**S:I1169F**	1	0.37	**S:T95N**	3	1.43	**S:M153I**	4	0.39
Karnataka	**S:F490S**	1	0.46	**S:T859I**	1	0.37	**S:K356M**	3	1.43	**S:S254F**	4	0.39
Karnataka	**S:I624T**	1	0.46	**S:D80Y**	1	0.37	**S:F490S**	2	0.95	**S:Q613H**	4	0.39
Karnataka	**S:D627H**	1	0.46	**S:A846S**	1	0.37	**S:V367F**	2	0.95	**S:S494P**	4	0.39
Karnataka	**S:M731I**	1	0.46	**S:S937T**	1	0.37	**S:D574Y**	2	0.95	**S:L176F**	3	0.3
Karnataka	**S:D936H**	1	0.46	**S:R246I**	1	0.37	**S:Q613H**	2	0.95	**S:L5F**	3	0.3
Karnataka	**S:D627P**	1	0.46	**S:M731I**	1	0.37	**S:A623S**	2	0.95	**S:T719S**	3	0.3
Karnataka	**S:D88H**	1	0.46	**S:D1118Y**	1	0.37	**S:Q675H**	2	0.95	**S:A1078S**	3	0.3
Karnataka	**S:S12F**	1	0.46	**S:V642I**	1	0.37	**S:L18F**	2	0.95	**S:S98F**	3	0.3
Karnataka	**S:L858F**	1	0.46	**S:I770V**	1	0.37	**S:T95I**	2	0.95	**S:K417N**	3	0.3
Karnataka	**S:F565L**	1	0.46	**S:G1267R**	1	0.37	**S:Q52R**	2	0.95	**S:V1228L**	3	0.3
Karnataka	**S:A27E**	1	0.46	**S:E654K**	1	0.37	**S:F888L**	2	0.95	**S:A1020S**	3	0.3
Karnataka	**S:S46T**	1	0.46	**S:V143F**	1	0.37	**S:R158G**	2	0.95	**S:T678I**	3	0.3
Karnataka	**S:Y28D**	1	0.46	**S:E1144Q**	1	0.37	**S:W152L**	2	0.95	**S:L1244F**	3	0.3
Karnataka	**S:A243P**	1	0.46	**S:V213G**	1	0.37	**S:N370S**	1	0.48	**S:A688V**	3	0.3
Karnataka	**S:L244F**	1	0.46	**S:P9S**	1	0.37	**S:V622F**	1	0.48	**S:A647S**	3	0.3
Karnataka	**S:Q1054H**	1	0.46	**S:E156Q**	1	0.37	**S:T29I**	1	0.48	**S:C1247F**	3	0.3
Karnataka	**S:S1055L**	1	0.46	**S:K187N**	1	0.37	**S:H245R**	1	0.48	**S:Q675H**	3	0.3
Karnataka	**S:S254C**	1	0.46	**S:I584V**	1	0.37	**S:K1191N**	1	0.48	**S:N1187D**	3	0.3
Karnataka				**S:S940F**	1	0.37	**S:S255F**	1	0.48	**S:D215Y**	3	0.3
Karnataka				**S:P681L**	1	0.37	**S:Q628K**	1	0.48	**S:A924S**	3	0.3
Karnataka				**S:D936Y**	1	0.37	**S:S151I**	1	0.48	**S:H245Y**	3	0.3
Karnataka				**S:H146Y**	1	0.37	**S:P1263L**	1	0.48	**S:D796H**	3	0.3
Karnataka				**S:K1073T**	1	0.37	**S:P631S**	1	0.48	**S:V3G**	2	0.2
Karnataka				**S:M1229I**	1	0.37	**S:L1244F**	1	0.48	**S:T572I**	2	0.2
Karnataka				**S:E1202Q**	1	0.37	**S:D936Y**	1	0.48	**S:G1246V**	2	0.2
Karnataka				**S:D1259Y**	1	0.37	**S:I834V**	1	0.48	**S:G181V**	2	0.2
Karnataka				**S:D574Y**	1	0.37	**S:P26R**	1	0.48	**S:S50L**	2	0.2
Karnataka				**S:A1078T**	1	0.37	**S:I105V**	1	0.48	**S:L455F**	2	0.2
Karnataka				**S:S12F**	1	0.37	**S:E156Q**	1	0.48	**S:V171F**	2	0.2
Karnataka				**S:E465D**	1	0.37	**S:A27S**	1	0.48	**S:Q414K**	2	0.2
Karnataka				**S:N679K**	1	0.37	**S:M153I**	1	0.48	**S:A419S**	2	0.2
Karnataka				**S:Q23R**	1	0.37	**S:A522P**	1	0.48	**S:D215G**	2	0.2
Karnataka				**S:C1247F**	1	0.37	**S:S939F**	1	0.48	**S:V36F**	2	0.2
Karnataka				**S:T323I**	1	0.37	**S:T20I**	1	0.48	**S:S929I**	2	0.2
Karnataka				**S:D253G**	1	0.37	**S:A1078S**	1	0.48	**S:S940F**	2	0.2
Karnataka				**S:F306L**	1	0.37	**S:H655Y**	1	0.48	**S:S680T**	2	0.2
Karnataka				**S:P1162S**	1	0.37	**S:L24F**	1	0.48	**S:K444N**	2	0.2
Karnataka				**S:S494L**	1	0.37	**S:P25V**	1	0.48	**S:S13T**	2	0.2
Karnataka				**S:E583D**	1	0.37	**S:R190K**	1	0.48	**S:L1063F**	2	0.2
Karnataka				**S:Q677P**	1	0.37	**S:L938F**	1	0.48	**S:W258L**	2	0.2
Karnataka				**S:V483A**	1	0.37	**S:L1049V**	1	0.48	**S:W152R**	2	0.2
Karnataka				**S:N556K**	1	0.37	**S:S884F**	1	0.48	**S:I1227M**	2	0.2
Karnataka				**S:D215H**	1	0.37	**S:F490L**	1	0.48	**S:F374L**	2	0.2
Karnataka				**S:A67V**	1	0.37	**S:G142D**	1	0.48	**S:E169Q**	2	0.2
Karnataka				**S:V642F**	1	0.37	**S:A653V**	1	0.48	**S:S256L**	2	0.2
Karnataka				**S:S477N**	1	0.37	**S:E96D**	1	0.48	**S:M177I**	2	0.2
Karnataka				**S:A647S**	1	0.37	**S:T76A**	1	0.48	**S:S12F**	2	0.2
Karnataka				**S:P863S**	1	0.37	**S:F79L**	1	0.48	**S:T250A**	2	0.2
Karnataka				**S:Q675R**	1	0.37	**S:D198H**	1	0.48	**S:M1237I**	2	0.2
Karnataka				**S:S1170A**	1	0.37	**S:I1114V**	1	0.48	**S:S494L**	2	0.2
Karnataka				**S:V308I**	1	0.37	**S:D808H**	1	0.48	**S:N185K**	2	0.2
Karnataka				**S:V1122L**	1	0.37	**S:L452M**	1	0.48	**S:W258R**	2	0.2
Karnataka				**S:E484Q**	1	0.37	**S:P1162L**	1	0.48	**S:A27S**	2	0.2
Karnataka				**S:H69Y**	1	0.37	**S:V213A**	1	0.48	**S:A694V**	2	0.2

Karnataka							**S:S494P**	1	0.48	**S:E1202Q**	1	0.1
Karnataka										**S:I210V**	1	0.1
Karnataka										**S:R214L**	1	0.1
Karnataka										**S:L1141W**	1	0.1
Karnataka										**S:V213L**	1	0.1
Karnataka										**S:T240I**	1	0.1
Karnataka										**S:G476S**	1	0.1
Karnataka										**S:G1219C**	1	0.1
Karnataka										**S:V308L**	1	0.1
Karnataka										**S:I203L**	1	0.1
Karnataka										**S:D420N**	1	0.1
Karnataka										**S:P209T**	1	0.1
Karnataka										**S:C1248F**	1	0.1
Karnataka										**S:A260S**	1	0.1
Karnataka										**S:G446D**	1	0.1
Karnataka										**S:G1099D**	1	0.1
Karnataka										**S:S45F**	1	0.1
Karnataka										**S:N280S**	1	0.1
Karnataka										**S:A397S**	1	0.1
Karnataka										**S:E96D**	1	0.1
Karnataka										**S:Y170S**	1	0.1
Karnataka										**S:W258C**	1	0.1
Karnataka										**S:V16L**	1	0.1
Karnataka										**S:E1111Q**	1	0.1
Karnataka										**S:P337L**	1	0.1
Karnataka										**S:S359G**	1	0.1
Karnataka										**S:Q1180K**	1	0.1
Karnataka										**S:E1262D**	1	0.1
Karnataka										**S:A1070V**	1	0.1
Karnataka										**S:P1162S**	1	0.1
Karnataka										**S:P1263L**	1	0.1
Karnataka										**S:R21T**	1	0.1
Karnataka										**S:S477I**	1	0.1
Karnataka										**S:S708F**	1	0.1
Karnataka										**S:D178G**	1	0.1
Karnataka										**S:V1176F**	1	0.1
Karnataka										**S:C1250F**	1	0.1
Karnataka										**S:R646L**	1	0.1
Karnataka										**S:T1117I**	1	0.1
Karnataka										**S:A522V**	1	0.1
Karnataka										**S:T1120I**	1	0.1
Karnataka										**S:P174S**	1	0.1
Karnataka										**S:R1014T**	1	0.1
Karnataka										**S:V1230L**	1	0.1
Karnataka										**S:L922S**	1	0.1
Karnataka										**S:E1188K**	1	0.1
Karnataka										**S:D138H**	1	0.1
Karnataka										**S:V512I**	1	0.1
Karnataka										**S:L8F**	1	0.1
Karnataka										**S:P217S**	1	0.1
Karnataka										**S:A871S**	1	0.1
Karnataka										**S:I1232V**	1	0.1
Karnataka										**S:Y380F**	1	0.1
Karnataka										**S:A1226V**	1	0.1
Karnataka										**S:G1085A**	1	0.1
Karnataka										**S:A783V**	1	0.1
Karnataka										**S:D1259Y**	1	0.1
Karnataka										**S:K1191N**	1	0.1
Karnataka										**S:E1207D**	1	0.1
Karnataka										**S:T22I**	1	0.1
Karnataka										**S:I100V**	1	0.1
Karnataka										**S:E1195Q**	1	0.1
Karnataka										**S:G257S**	1	0.1
Karnataka										**S:P26L**	1	0.1
Karnataka										**S:D80N**	1	0.1
Karnataka										**S:V826L**	1	0.1
Karnataka										**S:T572S**	1	0.1
Karnataka										**S:D1084Y**	1	0.1
Karnataka										**S:K1205N**	1	0.1
Karnataka										**S:P230S**	1	0.1
Karnataka										**S:V159A**	1	0.1
Karnataka										**S:A163V**	1	0.1
Karnataka										**S:N164L**	1	0.1

Karnataka										**S:N165H**	1	0.1
Karnataka										**S:C166S**	1	0.1
Karnataka										**S:Y170D**	1	0.1
Karnataka										**S:Q173L**	1	0.1
Karnataka										**S:K187S**	1	0.1
Karnataka										**S:L189V**	1	0.1
Karnataka										**S:R190T**	1	0.1
Karnataka										**S:E191W**	1	0.1
Karnataka										**S:V193H**	1	0.1
Karnataka										**S:F194V**	1	0.1
Karnataka										**S:K195S**	1	0.1
Karnataka										**S:D198F**	1	0.1
Karnataka										**S:I210T**	1	0.1
Karnataka										**S:N211Y**	1	0.1
Karnataka										**S:V16A**	1	0.1
Karnataka										**S:K733E**	1	0.1
Karnataka										**S:E1144V**	1	0.1
Karnataka										**S:I1130L**	1	0.1
Karnataka										**S:V1065A**	1	0.1
Karnataka										**S:V289L**	1	0.1
Karnataka										**S:A411S**	1	0.1
Karnataka										**S:L922W**	1	0.1
Karnataka										**S:A892S**	1	0.1
Karnataka										**S:S373L**	1	0.1
Karnataka										**S:E1150D**	1	0.1
Karnataka										**S:V401I**	1	0.1
Karnataka										**S:Q218R**	1	0.1
Karnataka										**S:S640F**	1	0.1
Karnataka										**S:A831V**	1	0.1
Karnataka										**S:V1068I**	1	0.1
Karnataka										**S:T618A**	1	0.1
Karnataka										**S:M177V**	1	0.1
Karnataka										**S:G142A**	1	0.1
Karnataka										**S:S94F**	1	0.1
Karnataka										**S:D427G**	1	0.1
Karnataka										**S:D574Y**	1	0.1
Karnataka										**S:I909V**	1	0.1
Karnataka										**S:V615F**	1	0.1
Karnataka										**S:E702V**	1	0.1
Karnataka										**S:T961M**	1	0.1
Karnataka										**S:T883I**	1	0.1
Karnataka										**S:E583D**	1	0.1
Karnataka										**S:A879S**	1	0.1
Karnataka										**S:S1252F**	1	0.1
Karnataka										**S:I670V**	1	0.1
Karnataka										**S:N148Y**	1	0.1
Karnataka										**S:W152L**	1	0.1
Karnataka										**S:V1264M**	1	0.1
Karnataka										**S:F32N**	1	0.1
Karnataka										**S:Q314K**	1	0.1
Karnataka										**S:R1107I**	1	0.1
Karnataka										**S:A831S**	1	0.1
	**Envelope (E)**											
**Karnataka**	**Pre-Wave 1**			**Wave 1**			**Pre-Wave 2**			**Wave 2**		
Karnataka	**Mutation**	**Count**	**percent**	**Mutation**	**Count**	**percent**	**Mutation**	**Count**	**percent**	**Mutation**	**Count**	**percent**
Karnataka	**E:V75F**	12	5.56	**E:V75F**	4	1.49	**E:A41V**	19	9.05	**E:V58F**	5	0.49
Karnataka	**E:I46V**	3	1.39	**E:L73F**	2	0.74	**E:V75F**	4	1.9	**E:S6T**	2	0.2
Karnataka				**E:S68F**	1	0.37	**E:P71L**	4	1.9	**E:L73F**	1	0.1
Karnataka				**E:V24A**	1	0.37	**E:L21F**	2	0.95	**E:V62F**	1	0.1
Karnataka				**E:V49L**	1	0.37	**E:V49L**	2	0.95	**E:V24A**	1	0.1
Karnataka							**E:C43F**	1	0.48	**E:S55F**	1	0.1
Karnataka							**E:T9I**	1	0.48	**E:A41V**	1	0.1
Karnataka										**E:D72E**	1	0.1
	**Membrane (M)**											
**Karnataka**	**Pre-Wave 1**			**Wave 1**			**Pre-Wave 2**			**Wave 2**		
Karnataka	**Mutation**	**Count**	**percent**	**Mutation**	**Count**	**percent**	**Mutation**	**Count**	**percent**	**Mutation**	**Count**	**percent**
Karnataka	**M:*223K**	4	1.85	**M:A2S**	6	2.23	**M:I82T**	19	9.05	**M:I82T**	730	71.85

Karnataka	**M:P123Q**	2	0.93	**M:W75S**	4	1.49	**M:I82S**	10	4.76	**M:I82S**	79	7.78
Karnataka	**M:S94I**	1	0.46	**M:H125Y**	1	0.37	**M:W75R**	3	1.43	**M:A2S**	14	1.38
Karnataka	**M:M1L**	1	0.46	**M:H148Y**	1	0.37	**M:A68S**	2	0.95	**M:F28L**	9	0.89
Karnataka	**M:A2G**	1	0.46	**M:V70L**	1	0.37	**M:D160Y**	1	0.48	**M:A85V**	5	0.49
Karnataka	**M:S4V**	1	0.46	**M:M84I**	1	0.37	**M:A69S**	1	0.48	**M:D209Y**	3	0.3
Karnataka	**M:N5Y**	1	0.46	**M:S4P**	1	0.37	**M:L29F**	1	0.48	**M:V23L**	3	0.3
Karnataka				**M:A38S**	1	0.37	**M:A63T**	1	0.48	**M:L29F**	2	0.2
Karnataka				**M:V66L**	1	0.37	**M:A2S**	1	0.48	**M:S214I**	2	0.2
Karnataka							**M:A85V**	1	0.48	**M:M109I**	2	0.2
Karnataka										**M:I144M**	1	0.1
Karnataka										**M:I217V**	1	0.1
Karnataka										**M:P123L**	1	0.1
Karnataka										**M:A2V**	1	0.1
Karnataka										**M:T127N**	1	0.1
Karnataka										**M:V10I**	1	0.1
Karnataka										**M:R158C**	1	0.1
Karnataka										**M:M84V**	1	0.1
Karnataka										**M:I128T**	1	0.1
Karnataka										**M:E12Q**	1	0.1
Karnataka										**M:W31L**	1	0.1
Karnataka										**M:C64F**	1	0.1
Karnataka										**M:V70F**	1	0.1
Karnataka										**M:G79A**	1	0.1
Karnataka										**M:C86F**	1	0.1
Karnataka										**M:T7I**	1	0.1
	**Nucleocapsid (N)**											
**Karnataka**	**Pre-Wave 1**			**Wave 1**			**Pre-Wave 2**			**Wave 2**		
Karnataka	**Mutation**	**Count**	**percent**	**Mutation**	**Count**	**percent**	**Mutation**	**Count**	**percent**	**Mutation**	**Count**	**percent**
Karnataka	**N:R203K**	80	37.04	**N:R203K**	88	32.71	**N:S2P**	59	28.1	**N:D377Y**	876	86.22
Karnataka	**N:G204R**	80	37.04	**N:G204R**	87	32.34	**N:G204R**	49	23.33	**N:D63G**	736	72.44
Karnataka	**N:P13L**	43	19.91	**N:S2P**	76	28.25	**N:R203K**	46	21.9	**N:R203M**	698	68.7
Karnataka	**N:S194L**	18	8.33	**N:S194L**	68	25.28	**N:S194L**	44	20.95	**N:G215C**	435	42.81
Karnataka	**N:Q9H**	10	4.63	**N:T379I**	34	12.64	**N:D377Y**	43	20.48	**N:D3L**	68	6.69
Karnataka	**N:P199S**	9	4.17	**N:D3L**	15	5.58	**N:R203M**	34	16.19	**N:R203K**	59	5.81
Karnataka	**N:A381S**	3	1.39	**N:L139F**	12	4.46	**N:D3L**	29	13.81	**N:G204R**	57	5.61
Karnataka	**N:S202N**	3	1.39	**N:R209I**	6	2.23	**N:S235F**	21	10	**N:S235F**	47	4.63
Karnataka	**N:S413I**	2	0.93	**N:S235F**	6	2.23	**N:D63G**	18	8.57	**N:R385K**	31	3.05
Karnataka	**N:G179V**	1	0.46	**N:D377Y**	4	1.49	**N:D3Y**	9	4.29	**N:S194L**	23	2.26
Karnataka	**N:A381T**	1	0.46	**N:R262H**	4	1.49	**N:G215C**	9	4.29	**N:D3Y**	22	2.17
Karnataka	**N:R185C**	1	0.46	**N:R209K**	2	0.74	**N:R209I**	6	2.86	**N:S2P**	20	1.97
Karnataka	**N:A152T**	1	0.46	**N:P20S**	2	0.74	**N:T205I**	5	2.38	**N:P142S**	12	1.18
Karnataka	**N:Q163L**	1	0.46	**N:T16M**	2	0.74	**N:V392L**	4	1.9	**N:T362I**	12	1.18
Karnataka	**N:R209I**	1	0.46	**N:R14L**	2	0.74	**N:T362I**	4	1.9	**N:G236V**	9	0.89
Karnataka	**N:P368L**	1	0.46	**N:Q9H**	2	0.74	**N:M411L**	4	1.9	**N:L161F**	7	0.69
Karnataka	**N:Q9L**	1	0.46	**N:H145Y**	2	0.74	**N:G236V**	4	1.9	**N:A208S**	5	0.49
Karnataka	**N:S183Y**	1	0.46	**N:Q229H**	2	0.74	**N:T379I**	3	1.43	**N:S206F**	5	0.49
Karnataka	**N:P151L**	1	0.46	**N:G18C**	2	0.74	**N:P13S**	2	0.95	**N:G120V**	3	0.3
Karnataka	**N:R14C**	1	0.46	**N:D348H**	2	0.74	**N:M234I**	2	0.95	**N:A359S**	3	0.3
Karnataka	**N:D225Y**	1	0.46	**N:Q281L**	1	0.37	**N:H145Y**	2	0.95	**N:A90S**	3	0.3
Karnataka	**N:N192Y**	1	0.46	**N:P383L**	1	0.37	**N:T366I**	2	0.95	**N:G212V**	3	0.3
Karnataka	**N:Q229H**	1	0.46	**N:T362I**	1	0.37	**N:S2Y**	2	0.95	**N:G18C**	3	0.3
Karnataka	**N:T271A**	1	0.46	**N:Q389H**	1	0.37	**N:Q9H**	2	0.95	**N:E253D**	3	0.3
Karnataka	**N:Q160***	1	0.46	**N:R195I**	1	0.37	**N:D371E**	2	0.95	**N:G30E**	3	0.3
Karnataka	**N:K370E**	1	0.46	**N:A414S**	1	0.37	**N:A12G**	2	0.95	**N:A182S**	3	0.3
Karnataka	**N:A211V**	1	0.46	**N:S193I**	1	0.37	**N:Q389H**	2	0.95	**N:D103Y**	3	0.3
Karnataka	**N:D144E**	1	0.46	**N:D22Y**	1	0.37	**N:L167F**	2	0.95	**N:A211V**	2	0.2
Karnataka	**N:T247S**	1	0.46	**N:P67L**	1	0.37	**N:Q418H**	1	0.48	**N:Q389H**	2	0.2
Karnataka	**N:S416T**	1	0.46	**N:P13T**	1	0.37	**N:S327L**	1	0.48	**N:T141I**	2	0.2
Karnataka	**N:L331F**	1	0.46	**N:M234I**	1	0.37	**N:T166I**	1	0.48	**N:D144H**	2	0.2
Karnataka	**N:D348E**	1	0.46	**N:T205I**	1	0.37	**N:P365L**	1	0.48	**N:L139F**	2	0.2
Karnataka	**N:S404P**	1	0.46	**N:P258S**	1	0.37	**N:G214S**	1	0.48	**N:G238C**	2	0.2
Karnataka	**N:K405E**	1	0.46	**N:A156S**	1	0.37	**N:S193I**	1	0.48	**N:N8Y**	2	0.2
Karnataka	**N:R189S**	1	0.46	**N:K373N**	1	0.37	**N:S202N**	1	0.48	**N:P13T**	2	0.2
Karnataka	**N:G236D**	1	0.46	**N:G236D**	1	0.37	**N:D348H**	1	0.48	**N:S193I**	2	0.2
Karnataka	**N:Y360S**	1	0.46	**N:M322I**	1	0.37	**N:E253D**	1	0.48	**N:A152S**	2	0.2
Karnataka	**N:A414L**	1	0.46	**N:D3Y**	1	0.37	**N:R319H**	1	0.48	**N:A152V**	2	0.2
Karnataka	**N:D415N**	1	0.46	**N:N192Y**	1	0.37	**N:D103Y**	1	0.48	**N:R209I**	2	0.2
Karnataka				**N:Q380R**	1	0.37	**N:N48Y**	1	0.48	**N:D348Y**	2	0.2
Karnataka				**N:A220V**	1	0.37	**N:Q306E**	1	0.48	**N:D81Y**	2	0.2

Karnataka				**N:A252S**	1	0.37	**N:Q409R**	1	0.48	**N:R209G**	2	0.2
Karnataka				**N:P67S**	1	0.37	**N:D128N**	1	0.48	**N:A156S**	2	0.2
Karnataka				**N:K369Q**	1	0.37	**N:P344S**	1	0.48	**N:H300Y**	2	0.2
Karnataka				**N:G34W**	1	0.37	**N:G18S**	1	0.48	**N:S2F**	2	0.2
Karnataka				**N:Q418H**	1	0.37	**N:A119S**	1	0.48	**N:S255F**	1	0.1
Karnataka				**N:P13R**	1	0.37	**N:D81Y**	1	0.48	**N:K374N**	1	0.1
Karnataka				**N:D371E**	1	0.37	**N:G19R**	1	0.48	**N:S184P**	1	0.1
Karnataka				**N:T135I**	1	0.37	**N:M210I**	1	0.48	**N:A398S**	1	0.1
Karnataka				**N:A359S**	1	0.37	**N:N8D**	1	0.48	**N:L139S**	1	0.1
Karnataka				**N:A211V**	1	0.37	**N:T135I**	1	0.48	**N:D22Y**	1	0.1
Karnataka				**N:V72I**	1	0.37	**N:R385K**	1	0.48	**N:A414D**	1	0.1
Karnataka				**N:V270L**	1	0.37	**N:S33I**	1	0.48	**N:K373N**	1	0.1
Karnataka				**N:K374N**	1	0.37	**N:T282I**	1	0.48	**N:D401H**	1	0.1
Karnataka				**N:R226K**	1	0.37	**N:L139F**	1	0.48	**N:G44C**	1	0.1
Karnataka							**N:G243V**	1	0.48	**N:T334I**	1	0.1
Karnataka										**N:G18V**	1	0.1
Karnataka										**N:G25V**	1	0.1
Karnataka										**N:Q43K**	1	0.1
Karnataka										**N:A90T**	1	0.1
Karnataka										**N:G204E**	1	0.1
Karnataka										**N:A376S**	1	0.1
Karnataka										**N:R14P**	1	0.1
Karnataka										**N:V270L**	1	0.1
Karnataka										**N:Q9H**	1	0.1
Karnataka										**N:R14L**	1	0.1
Karnataka										**N:A254S**	1	0.1
Karnataka										**N:N8D**	1	0.1
Karnataka										**N:H145Y**	1	0.1
Karnataka										**N:Q418H**	1	0.1
Karnataka										**N:D371E**	1	0.1
Karnataka										**N:M322V**	1	0.1
Karnataka										**N:P6S**	1	0.1
Karnataka										**N:A273S**	1	0.1
Karnataka										**N:Q163L**	1	0.1
Karnataka										**N:Q272L**	1	0.1
Karnataka										**N:R41Q**	1	0.1
Karnataka										**N:D402Y**	1	0.1
Karnataka										**N:G147S**	1	0.1
Karnataka										**N:D399N**	1	0.1
Karnataka										**N:D402H**	1	0.1
Karnataka										**N:A305G**	1	0.1
Karnataka										**N:R385I**	1	0.1
Karnataka										**N:S79N**	1	0.1
Karnataka										**N:A414S**	1	0.1
Karnataka										**N:T366I**	1	0.1
Karnataka										**N:P13S**	1	0.1
Karnataka										**N:K387N**	1	0.1
Karnataka										**N:P46S**	1	0.1
Karnataka										**N:Q294H**	1	0.1
Karnataka										**N:P80S**	1	0.1
Karnataka										**N:A311T**	1	0.1
Karnataka										**N:A218S**	1	0.1
Karnataka										**N:T166I**	1	0.1
Karnataka										**N:P365L**	1	0.1
Karnataka										**N:T24A**	1	0.1
Karnataka										**N:A397S**	1	0.1
Karnataka										**N:Q294K**	1	0.1
Karnataka										**N:S180C**	1	0.1
Karnataka										**N:D399Y**	1	0.1
Karnataka										**N:D343G**	1	0.1
Karnataka										**N:M210T**	1	0.1
Karnataka										**N:Q289H**	1	0.1
**State/Gene**	**ORF3a**											
**Karnataka**	**Pre-Wave 1**			**Wave 1**			**Pre-Wave 2**			**Wave 2**		
**Karnataka**	**Mutation**	**Count**	**percent**	**Mutation**	**Count**	**percent**	**Mutation**	**Count**	**percent**	**Mutation**	**Count**	**percent**
**Karnataka**	**ORF3a:Q57H**	26	52	**ORF3a:Q57H**	100	35.21	**ORF3a:Q57H**	67	23.18	**ORF3a:S26L**	743	75.89
**Karnataka**	**ORF3a:L52I**	7	14	**ORF3a:E261***	81	28.52	**ORF3a:E261***	59	20.42	**ORF3a:Q57H**	23	2.35
**Karnataka**	**ORF3a:P240S**	3	6	**ORF3a:T223I**	9	3.17	**ORF3a:S26L**	37	12.8	**ORF3a:E261***	21	2.15
**Karnataka**	**ORF3a:L15F**	3	6	**ORF3a:S216T**	5	1.76	**ORF3a:D173Y**	19	6.57	**ORF3a:W69C**	13	1.33
**Karnataka**	**ORF3a:V90F**	2	4	**ORF3a:D155Y**	4	1.41	**ORF3a:A110S**	18	6.23	**ORF3a:D238Y**	13	1.33
**Karnataka**	**ORF3a:G254***	2	4	**ORF3a:L108F**	4	1.41	**ORF3a:T223I**	11	3.81	**ORF3a:P240L**	12	1.23

Karnataka	ORF3a:G172V	2	4	ORF3a:L46F	4	1.41	ORF3a:L106F	8	2.77	ORF3a:T175I	6	0.61
Karnataka	ORF3a:G100C	2	4	ORF3a:A72S	4	1.41	ORF3a:S171L	5	1.73	ORF3a:N234I	5	0.51
Karnataka	ORF3a:I35T	1	2	ORF3a:V225F	4	1.41	ORF3a:W69C	4	1.38	ORF3a:A23S	5	0.51
Karnataka	ORF3a:P159S	1	2	ORF3a:G224C	3	1.06	ORF3a:W193R	3	1.04	ORF3a:A33S	5	0.51
Karnataka	ORF3a:A51P	1	2	ORF3a:A54V	2	0.7	ORF3a:G174V	3	1.04	ORF3a:W45L	4	0.41
Karnataka				ORF3a:W131C	2	0.7	ORF3a:G224C	3	1.04	ORF3a:G100C	4	0.41
Karnataka				ORF3a:A23V	2	0.7	ORF3a:T229I	3	1.04	ORF3a:T89I	4	0.41
Karnataka				ORF3a:S166L	2	0.7	ORF3a:G224R	3	1.04	ORF3a:D173Y	3	0.31
Karnataka				ORF3a:S171L	2	0.7	ORF3a:P240S	2	0.69	ORF3a:Q213K	3	0.31
Karnataka				ORF3a:G172A	2	0.7	ORF3a:T151I	2	0.69	ORF3a:K75N	3	0.31
Karnataka				ORF3a:G172C	2	0.7	ORF3a:G100C	2	0.69	ORF3a:G172V	3	0.31
Karnataka				ORF3a:W193R	2	0.7	ORF3a:S216T	2	0.69	ORF3a:R68I	3	0.31
Karnataka				ORF3a:G224R	2	0.7	ORF3a:S165F	2	0.69	ORF3a:Q185H	3	0.31
Karnataka				ORF3a:V202L	2	0.7	ORF3a:W131C	2	0.69	ORF3a:L219F	3	0.31
Karnataka				ORF3a:T89I	2	0.7	ORF3a:D155Y	2	0.69	ORF3a:A110S	3	0.31
Karnataka				ORF3a:F105L	1	0.35	ORF3a:G174C	2	0.69	ORF3a:A98V	2	0.2
Karnataka				ORF3a:K136Q	1	0.35	ORF3a:V202L	1	0.35	ORF3a:S74F	2	0.2
Karnataka				ORF3a:S60F	1	0.35	ORF3a:I63T	1	0.35	ORF3a:V259L	2	0.2
Karnataka				ORF3a:V13L	1	0.35	ORF3a:V13L	1	0.35	ORF3a:L94F	2	0.2
Karnataka				ORF3a:D2Y	1	0.35	ORF3a:H93Y	1	0.35	ORF3a:L53F	2	0.2
Karnataka				ORF3a:S74F	1	0.35	ORF3a:K136E	1	0.35	ORF3a:S165F	2	0.2
Karnataka				ORF3a:V55G	1	0.35	ORF3a:Q185H	1	0.35	ORF3a:N257D	2	0.2
Karnataka				ORF3a:G251V	1	0.35	ORF3a:I118T	1	0.35	ORF3a:V13L	2	0.2
Karnataka				ORF3a:L94F	1	0.35	ORF3a:W128L	1	0.35	ORF3a:L85F	2	0.2
Karnataka				ORF3a:L53F	1	0.35	ORF3a:T176I	1	0.35	ORF3a:Q38L	2	0.2
Karnataka				ORF3a:T217I	1	0.35	ORF3a:P267S	1	0.35	ORF3a:A143V	2	0.2
Karnataka				ORF3a:T271I	1	0.35	ORF3a:R126M	1	0.35	ORF3a:V256I	2	0.2
Karnataka				ORF3a:R68T	1	0.35	ORF3a:A99S	1	0.35	ORF3a:W128L	2	0.2
Karnataka				ORF3a:W128C	1	0.35	ORF3a:S180F	1	0.35	ORF3a:G100D	2	0.2
Karnataka				ORF3a:E181V	1	0.35	ORF3a:A110V	1	0.35	ORF3a:W193C	2	0.2
Karnataka				ORF3a:L106F	1	0.35	ORF3a:L94F	1	0.35	ORF3a:V202L	2	0.2
Karnataka				ORF3a:E266G	1	0.35	ORF3a:L108F	1	0.35	ORF3a:T24I	2	0.2
Karnataka				ORF3a:V55I	1	0.35	ORF3a:S92L	1	0.35	ORF3a:G49V	2	0.2
Karnataka				ORF3a:C81F	1	0.35	ORF3a:T175I	1	0.35	ORF3a:T221K	2	0.2
Karnataka				ORF3a:E19G	1	0.35	ORF3a:V255F	1	0.35	ORF3a:A54S	2	0.2
Karnataka				ORF3a:P104L	1	0.35	ORF3a:D238Y	1	0.35	ORF3a:R134C	2	0.2
Karnataka				ORF3a:D199Y	1	0.35	ORF3a:G18V	1	0.35	ORF3a:G44V	2	0.2
Karnataka				ORF3a:V163L	1	0.35	ORF3a:W45L	1	0.35	ORF3a:A31S	2	0.2
Karnataka				ORF3a:D173Y	1	0.35	ORF3a:N234I	1	0.35	ORF3a:L106R	2	0.2
Karnataka				ORF3a:L129F	1	0.35	ORF3a:T32I	1	0.35	ORF3a:V88L	2	0.2
Karnataka				ORF3a:P159S	1	0.35	ORF3a:M260I	1	0.35	ORF3a:S92L	1	0.1
Karnataka				ORF3a:Y184H	1	0.35	ORF3a:I20V	1	0.35	ORF3a:D27Y	1	0.1
Karnataka				ORF3a:T12I	1	0.35	ORF3a:E194D	1	0.35	ORF3a:S117I	1	0.1
Karnataka				ORF3a:T32N	1	0.35	ORF3a:R134L	1	0.35	ORF3a:Y211H	1	0.1
Karnataka				ORF3a:G172V	1	0.35	ORF3a:I124T	1	0.35	ORF3a:W131L	1	0.1
Karnataka				ORF3a:S216P	1	0.35	ORF3a:T34M	1	0.35	ORF3a:V97I	1	0.1
Karnataka				ORF3a:A33S	1	0.35				ORF3a:I123M	1	0.1
Karnataka				ORF3a:P262S	1	0.35				ORF3a:D155Y	1	0.1
Karnataka				ORF3a:G254V	1	0.35				ORF3a:L65I	1	0.1
Karnataka				ORF3a:G174V	1	0.35				ORF3a:K21N	1	0.1
Karnataka				ORF3a:D183Y	1	0.35				ORF3a:L147F	1	0.1
Karnataka				ORF3a:A31T	1	0.35				ORF3a:A103T	1	0.1
Karnataka				ORF3a:T151N	1	0.35				ORF3a:E241K	1	0.1
Karnataka				ORF3a:I10S	1	0.35				ORF3a:A54V	1	0.1
Karnataka				ORF3a:G100S	1	0.35				ORF3a:L106F	1	0.1
Karnataka				ORF3a:I10F	1	0.35				ORF3a:L41F	1	0.1
Karnataka				ORF3a:M260I	1	0.35				ORF3a:S272R	1	0.1
Karnataka				ORF3a:G172D	1	0.35				ORF3a:W131C	1	0.1
Karnataka				ORF3a:I20V	1	0.35				ORF3a:T64I	1	0.1
Karnataka										ORF3a:N137K	1	0.1
Karnataka										ORF3a:G100V	1	0.1
Karnataka										ORF3a:L94P	1	0.1
Karnataka										ORF3a:T223I	1	0.1
Karnataka										ORF3a:S171L	1	0.1
Karnataka										ORF3a:G224C	1	0.1
Karnataka										ORF3a:W193R	1	0.1
Karnataka										ORF3a:T268M	1	0.1
Karnataka										ORF3a:G174C	1	0.1
Karnataka										ORF3a:S74C	1	0.1
Karnataka										ORF3a:G251S	1	0.1
Karnataka										ORF3a:W131S	1	0.1
Karnataka										ORF3a:S60P	1	0.1
Karnataka										ORF3a:P104L	1	0.1

**Karnataka**										**ORF3a:Y107H**	1	0.1
**Karnataka**										**ORF3a:P25L**	1	0.1
**Karnataka**										**ORF3a:G44E**	1	0.1
**Karnataka**										**ORF3a:K16N**	1	0.1
**Karnataka**										**ORF3a:S60Y**	1	0.1
**Karnataka**										**ORF3a:L96I**	1	0.1
**Karnataka**										**ORF3a:I246T**	1	0.1
**Karnataka**										**ORF3a:D155H**	1	0.1
**Karnataka**										**ORF3a:S252L**	1	0.1
**Karnataka**										**ORF3a:T151I**	1	0.1
**Karnataka**										**ORF3a:D222Y**	1	0.1
**Karnataka**										**ORF3a:G172C**	1	0.1
**Karnataka**										**ORF3a:A103S**	1	0.1
**Karnataka**										**ORF3a:T170S**	1	0.1
**State/Gene**	**ORF6**											
**Karnataka**	**Pre-Wave 1**			**Wave 1**			**Pre-Wave 2**			**Wave 2**		
**Karnataka**	**Mutation**	**Count**	**percent**	**Mutation**	**Count**	**percent**	**Mutation**	**Count**	**percent**	**Mutation**	**Count**	**percent**
**Karnataka**	**ORF6:M1I**	1	33.33	**ORF6:D6N**	2	40	**ORF6:I33T**	13	72.22	**ORF6:I33T**	65	70.65
**Karnataka**	**ORF6:P57L**	1	33.33	**ORF6:D61Y**	1	20	**ORF6:R20S**	3	16.67	**ORF6:I18V**	6	6.52
**Karnataka**	**ORF6:E59D**	1	33.33	**ORF6:D61G**	1	20	**ORF6:F2L**	1	5.56	**ORF6:R20K**	4	4.35
**Karnataka**				**ORF6:V9F**	1	20	**ORF6:W27L**	1	5.56	**ORF6:D61Y**	3	3.26
**Karnataka**										**ORF6:I37V**	3	3.26
**Karnataka**										**ORF6:Y31F**	2	2.17
**Karnataka**										**ORF6:W27L**	2	2.17
**Karnataka**										**ORF6:K42N**	1	1.09
**Karnataka**										**ORF6:V24F**	1	1.09
**Karnataka**										**ORF6:V5F**	1	1.09
**Karnataka**										**ORF6:T21I**	1	1.09
**Karnataka**										**ORF6:D6N**	1	1.09
**Karnataka**										**ORF6:M58L**	1	1.09
**Karnataka**										**ORF6:R20S**	1	1.09
**State/Gene**	**ORF7a**											
**Karnataka**	**Pre-Wave 1**			**Wave 1**			**Pre-Wave 2**			**Wave 2**		
**Karnataka**	**Mutation**	**Count**	**percent**	**Mutation**	**Count**	**percent**	**Mutation**	**Count**	**percent**	**Mutation**	**Count**	**percent**
**Karnataka**	**ORF7a:G38V**	8	28.57	**ORF7a:V104F**	2	13.33	**ORF7a:V82A**	33	32.67	**ORF7a:V82A**	706	49.41
**Karnataka**	**ORF7a:T39K**	8	28.57	**ORF7a:A50V**	1	6.67	**ORF7a:V93I**	19	18.81	**ORF7a:T120I**	606	42.41
**Karnataka**	**ORF7a:Y40H**	8	28.57	**ORF7a:P84S**	1	6.67	**ORF7a:T120I**	15	14.85	**ORF7a:L116F**	46	3.22
**Karnataka**	**ORF7a:L102F**	3	10.71	**ORF7a:A105V**	1	6.67	**ORF7a:K119N**	8	7.92	**ORF7a:K119N**	12	0.84
**Karnataka**	**ORF7a:P34S**	1	3.57	**ORF7a:S37P**	1	6.67	**ORF7a:L116F**	5	4.95	**ORF7a:N43Y**	7	0.49
**Karnataka**				**ORF7a:A8V**	1	6.67	**ORF7a:D69Y**	4	3.96	**ORF7a:D51Y**	5	0.35
**Karnataka**				**ORF7a:A55V**	1	6.67	**ORF7a:T14N**	3	2.97	**ORF7a:G38V**	4	0.28
**Karnataka**				**ORF7a:V29I**	1	6.67	**ORF7a:L112F**	3	2.97	**ORF7a:V24F**	4	0.28
**Karnataka**				**ORF7a:V93I**	1	6.67	**ORF7a:S98F**	2	1.98	**ORF7a:V93I**	3	0.21
**Karnataka**				**ORF7a:T14N**	1	6.67	**ORF7a:P84S**	2	1.98	**ORF7a:Q62***	3	0.21
**Karnataka**				**ORF7a:P99L**	1	6.67	**ORF7a:N43Y**	2	1.98	**ORF7a:F63L**	2	0.14
**Karnataka**				**ORF7a:T120I**	1	6.67	**ORF7a:V24F**	1	0.99	**ORF7a:P34L**	2	0.14
**Karnataka**				**ORF7a:V24F**	1	6.67	**ORF7a:*122L**	1	0.99	**ORF7a:P99L**	2	0.14
**Karnataka**				**ORF7a:E22***	1	6.67	**ORF7a:E95Q**	1	0.99	**ORF7a:A55S**	2	0.14
**Karnataka**							**ORF7a:P99S**	1	0.99	**ORF7a:A50S**	2	0.14
**Karnataka**							**ORF7a:Q62H**	1	0.99	**ORF7a:Q76R**	1	0.07
**Karnataka**										**ORF7a:K2R**	1	0.07
**Karnataka**										**ORF7a:F6L**	1	0.07
**Karnataka**										**ORF7a:T11K**	1	0.07
**Karnataka**										**ORF7a:C58F**	1	0.07
**Karnataka**										**ORF7a:I110R**	1	0.07
**Karnataka**										**ORF7a:P45S**	1	0.07
**Karnataka**										**ORF7a:Q94***	1	0.07
**Karnataka**										**ORF7a:V29I**	1	0.07
**Karnataka**										**ORF7a:R78C**	1	0.07
**Karnataka**										**ORF7a:A50T**	1	0.07
**Karnataka**										**ORF7a:E92G**	1	0.07
**Karnataka**										**ORF7a:E41G**	1	0.07
**Karnataka**										**ORF7a:V24I**	1	0.07
**Karnataka**										**ORF7a:P84S**	1	0.07
**Karnataka**										**ORF7a:V93F**	1	0.07
**Karnataka**										**ORF7a:R89I**	1	0.07
**Karnataka**										**ORF7a:R25K**	1	0.07
**Karnataka**										**ORF7a:G38***	1	0.07
**Karnataka**										**ORF7a:T120V**	1	0.07
**Karnataka**										**ORF7a:R25I**	1	0.07
**Karnataka**										**ORF7a:K32E**	1	0.07

**Karnataka**										**ORF7a:E95K**	1	0.07
**State/Gene**	**ORF7b**											
**Karnataka**	**Pre-Wave 1**			**Wave 1**			**Pre-Wave 2**			**Wave 2**		
**Karnataka**	**Mutation**	**Count**	**percent**	**Mutation**	**Count**	**percent**	**Mutation**	**Count**	**percent**	**Mutation**	**Count**	**percent**
**Karnataka**	**ORF7b:S31L**	5	35.71	**ORF7b:H37R**	2	20	**ORF7b:T40I**	9	36	**ORF7b:T40I**	453	95.97
**Karnataka**	**ORF7b:Y10C**	3	21.43	**ORF7b:F16L**	1	10	**ORF7b:A15S**	4	16	**ORF7b:A15S**	5	1.06
**Karnataka**	**ORF7b:L11F**	1	7.14	**ORF7b:A43N**	1	10	**ORF7b:E39***	4	16	**ORF7b:A43V**	3	0.64
**Karnataka**	**ORF7b:Y10F**	1	7.14	**ORF7b:D8Y**	1	10	**ORF7b:S31L**	3	12	**ORF7b:C41F**	2	0.42
**Karnataka**	**ORF7b:V21F**	1	7.14	**ORF7b:F13L**	1	10	**ORF7b:L17V**	2	8	**ORF7b:D36Y**	2	0.42
**Karnataka**	**ORF7b:L6F**	1	7.14	**ORF7b:L14F**	1	10	**ORF7b:H42Y**	1	4	**ORF7b:A43S**	2	0.42
**Karnataka**	**ORF7b:I7V**	1	7.14	**ORF7b:L4F**	1	10	**ORF7b:M1I**	1	4	**ORF7b:E39***	2	0.42
**Karnataka**	**ORF7b:Y10H**	1	7.14	**ORF7b:A15V**	1	10	**ORF7b:I23T**	1	4	**ORF7b:V21I**	1	0.21
**Karnataka**				**ORF7b:T40I**	1	10				**ORF7b:Q35R**	1	0.21
**Karnataka**										**ORF7b:H37L**	1	0.21
**State/Gene**	**ORF8**											
**Karnataka**	**Pre-Wave 1**			**Wave 1**			**Pre-Wave 2**			**Wave 2**		
**Karnataka**	**Mutation**	**Count**	**percent**	**Mutation**	**Count**	**percent**	**Mutation**	**Count**	**percent**	**Mutation**	**Count**	**percent**
**Karnataka**	**ORF8:G8***	7	38.89	**ORF8:S97I**	77	36.15	**ORF8:S97I**	56	27.32	**ORF8:F120L**	70	15.32
**Karnataka**	**ORF8:L84S**	3	16.67	**ORF8:C20F**	46	21.6	**ORF8:Y73C**	28	13.66	**ORF8:Y73C**	69	15.1
**Karnataka**	**ORF8:V33F**	1	5.56	**ORF8:Q27***	16	7.51	**ORF8:R52I**	26	12.68	**ORF8:R52I**	65	14.22
**Karnataka**	**ORF8:E64D**	1	5.56	**ORF8:R52I**	15	7.04	**ORF8:Q27***	25	12.2	**ORF8:Q27***	63	13.79
**Karnataka**	**ORF8:A65S**	1	5.56	**ORF8:Y73C**	14	6.57	**ORF8:F120L**	14	6.83	**ORF8:D119V**	50	10.94
**Karnataka**	**ORF8:V99L**	1	5.56	**ORF8:A51V**	5	2.35	**ORF8:L95F**	9	4.39	**ORF8:S69L**	26	5.69
**Karnataka**	**ORF8:D119G**	1	5.56	**ORF8:I121L**	3	1.41	**ORF8:I121L**	8	3.9	**ORF8:K68***	19	4.16
**Karnataka**	**ORF8:R52I**	1	5.56	**ORF8:K68***	3	1.41	**ORF8:D119V**	8	3.9	**ORF8:S97I**	15	3.28
**Karnataka**	**ORF8:R115L**	1	5.56	**ORF8:Q18***	2	0.94	**ORF8:C20F**	7	3.41	**ORF8:G8V**	7	1.53
**Karnataka**	**ORF8:I121F**	1	5.56	**ORF8:E59***	2	0.94	**ORF8:A65V**	3	1.46	**ORF8:V62L**	6	1.31
**Karnataka**				**ORF8:S21I**	2	0.94	**ORF8:E106Q**	3	1.46	**ORF8:E106***	5	1.09
**Karnataka**				**ORF8:L95F**	2	0.94	**ORF8:K68***	3	1.46	**ORF8:E19***	5	1.09
**Karnataka**				**ORF8:I9T**	2	0.94	**ORF8:L84S**	2	0.98	**ORF8:F16S**	4	0.88
**Karnataka**				**ORF8:G66D**	2	0.94	**ORF8:E92K**	2	0.98	**ORF8:I10V**	4	0.88
**Karnataka**				**ORF8:L57S**	2	0.94	**ORF8:Q18***	2	0.98	**ORF8:I47V**	4	0.88
**Karnataka**				**ORF8:V62L**	2	0.94	**ORF8:E110***	1	0.49	**ORF8:Q23L**	3	0.66
**Karnataka**				**ORF8:Q29L**	1	0.47	**ORF8:K68E**	1	0.49	**ORF8:P85L**	2	0.44
**Karnataka**				**ORF8:G8V**	1	0.47	**ORF8:S69L**	1	0.49	**ORF8:P38L**	2	0.44
**Karnataka**				**ORF8:V5F**	1	0.47	**ORF8:Q29L**	1	0.49	**ORF8:G77C**	2	0.44
**Karnataka**				**ORF8:T11S**	1	0.47	**ORF8:A55T**	1	0.49	**ORF8:W45L**	2	0.44
**Karnataka**				**ORF8:G66V**	1	0.47	**ORF8:A65T**	1	0.49	**ORF8:R115L**	2	0.44
**Karnataka**				**ORF8:I47T**	1	0.47	**ORF8:I47V**	1	0.49	**ORF8:L118V**	2	0.44
**Karnataka**				**ORF8:V32L**	1	0.47	**ORF8:V62L**	1	0.49	**ORF8:D75Y**	2	0.44
**Karnataka**				**ORF8:E106***	1	0.47	**ORF8:P70T**	1	0.49	**ORF8:A51V**	2	0.44
**Karnataka**				**ORF8:A65V**	1	0.47				**ORF8:P36L**	1	0.22
**Karnataka**				**ORF8:V13A**	1	0.47				**ORF8:D35Y**	1	0.22
**Karnataka**				**ORF8:P38L**	1	0.47				**ORF8:Y42***	1	0.22
**Karnataka**				**ORF8:W45L**	1	0.47				**ORF8:Q72H**	1	0.22
**Karnataka**				**ORF8:D34Y**	1	0.47				**ORF8:V32L**	1	0.22
**Karnataka**				**ORF8:D119Y**	1	0.47				**ORF8:G66C**	1	0.22
**Karnataka**				**ORF8:T26I**	1	0.47				**ORF8:D63N**	1	0.22
**Karnataka**				**ORF8:I10T**	1	0.47				**ORF8:I10T**	1	0.22
**Karnataka**				**ORF8:S67F**	1	0.47				**ORF8:E106D**	1	0.22
**Karnataka**				**ORF8:E106Q**	1	0.47				**ORF8:V100L**	1	0.22
**Karnataka**										**ORF8:C37F**	1	0.22
**Karnataka**										**ORF8:W45S**	1	0.22
**Karnataka**										**ORF8:C20F**	1	0.22
**Karnataka**										**ORF8:T26I**	1	0.22
**Karnataka**										**ORF8:S67F**	1	0.22
**Karnataka**										**ORF8:A15V**	1	0.22
**Karnataka**										**ORF8:A65V**	1	0.22
**Karnataka**										**ORF8:W45C**	1	0.22
**Karnataka**										**ORF8:F3L**	1	0.22
**Karnataka**										**ORF8:L95F**	1	0.22
**Karnataka**										**ORF8:D75G**	1	0.22
**Karnataka**										**ORF8:D113E**	1	0.22
**Karnataka**										**ORF8:E64***	1	0.22
**Karnataka**										**ORF8:G8E**	1	0.22
**Karnataka**										**ORF8:C90F**	1	0.22
**Karnataka**										**ORF8:I39T**	1	0.22
**State/Gene**	**ORF9b**											
**Karnataka**	**Pre-Wave 1**			**Wave 1**			**Pre-Wave 2**			**Wave 2**		
**Karnataka**	**Mutation**	**Count**	**percent**	**Mutation**	**Count**	**percent**	**Mutation**	**Count**	**percent**	**Mutation**	**Count**	**percent**

Karnataka	ORF9b:P10S	43	79.63	ORF9b:R13C	2	13.33	ORF9b:T60A	18	62.07	ORF9b:T60A	736	94.85
Karnataka	ORF9b:S6I	10	18.52	ORF9b:A11S	2	13.33	ORF9b:R32P	2	6.9	ORF9b:P3L	5	0.64
Karnataka	ORF9b:S6C	1	1.85	ORF9b:S6I	2	13.33	ORF9b:S6I	2	6.9	ORF9b:E86D	3	0.39
Karnataka				ORF9b:L14F	2	13.33	ORF9b:H9D	2	6.9	ORF9b:L14F	3	0.39
Karnataka				ORF9b:V37A	1	6.67	ORF9b:R13H	1	3.45	ORF9b:E27K	3	0.39
Karnataka				ORF9b:Q18H	1	6.67	ORF9b:M8I	1	3.45	ORF9b:K4N	2	0.26
Karnataka				ORF9b:L64F	1	6.67	ORF9b:S6N	1	3.45	ORF9b:R32L	2	0.26
Karnataka				ORF9b:H9Q	1	6.67	ORF9b:Q77H	1	3.45	ORF9b:R13L	2	0.26
Karnataka				ORF9b:R32L	1	6.67	ORF9b:V30L	1	3.45	ORF9b:H9Q	2	0.26
Karnataka				ORF9b:P10A	1	6.67				ORF9b:L61S	2	0.26
Karnataka				ORF9b:P39L	1	6.67				ORF9b:Q77H	2	0.26
Karnataka										ORF9b:Q18H	1	0.13
Karnataka										ORF9b:K40N	1	0.13
Karnataka										ORF9b:V15L	1	0.13
Karnataka										ORF9b:A22S	1	0.13
Karnataka										ORF9b:G38V	1	0.13
Karnataka										ORF9b:T83I	1	0.13
Karnataka										ORF9b:A11P	1	0.13
Karnataka										ORF9b:S6I	1	0.13
Karnataka										ORF9b:A11S	1	0.13
Karnataka										ORF9b:G38S	1	0.13
Karnataka										ORF9b:D33G	1	0.13
Karnataka										ORF9b:V76I	1	0.13
Karnataka										ORF9b:S53L	1	0.13
Karnataka										ORF9b:R32H	1	0.13
Karnataka	Amino acid deletions											
Karnataka	Pre-Wave 1			Wave 1			Pre-Wave 2			Wave 2		
Karnataka	Mutation	Count	percent	Mutation	Count	percent	Mutation	Count	percent	Mutation	Count	percent
Karnataka	ORF1a:K141-	2	0.48	S:Y144-	16	15.84	S:Y144-	16	8.99	ORF8:D119-	510	18.88
Karnataka	ORF1a:S142-	2	0.48	ORF1a:S3675-	15	14.85	ORF1a:S3675-	15	8.43	ORF8:F120-	510	18.88
Karnataka	ORF1a:F143-	2	0.48	ORF1a:G3676-	15	14.85	ORF1a:G3676-	15	8.43	S:E156-	459	16.99
Karnataka	ORF1a:V84-	2	0.48	ORF1a:F3677-	15	14.85	ORF1a:F3677-	15	8.43	S:F157-	459	16.99
Karnataka	ORF1a:M85-	2	0.48	S:H69-	15	14.85	S:H69-	13	7.3	S:P57-	26	0.96
Karnataka	ORF1b:L1943-	1	0.24	S:V70-	15	14.85	S:V70-	13	7.3	S:F58-	25	0.93
Karnataka	ORF1b:Y1944-	1	0.24	ORF3a:M1-	1	0.99	ORF1b:I2687-	2	1.12	S:Y144-	25	0.93
Karnataka	ORF1b:V1945-	1	0.24	S:L242-	1	0.99	ORF1b:S2688-	2	1.12	S:H69-	24	0.89
Karnataka	ORF1b:N1946-	1	0.24	S:A243-	1	0.99	ORF1b:S2689-	2	1.12	S:Q52-	24	0.89
Karnataka	ORF1b:K1947-	1	0.24	ORF1a:V84-	1	0.99	ORF1b:D2690-	2	1.12	S:D53-	24	0.89
Karnataka	ORF1b:H1948-	1	0.24	ORF1a:M85-	1	0.99	ORF1b:V2691-	2	1.12	ORF1a:S3675-	23	0.85
Karnataka	ORF1b:G2005-	1	0.24	ORF1a:V868-	1	0.99	ORF1b:L2692-	2	1.12	ORF1a:G3676-	23	0.85
Karnataka	ORF1b:A2006-	1	0.24	ORF1a:S2083-	1	0.99	ORF1b:V2693-	2	1.12	ORF1a:F3677-	23	0.85
Karnataka	ORF1b:V2007-	1	0.24	ORF1a:L2084-	1	0.99	ORF1b:N2694-	2	1.12	S:V70-	23	0.85
Karnataka	ORF1b:C2008-	1	0.24	ORF1a:K2085-	1	0.99	ORF1b:N2695-	2	1.12	ORF1a:R2115-	7	0.26
Karnataka	ORF1b:R2009-	1	0.24	ORF1a:I2086-	1	0.99	ORF1b:*2696-	2	1.12	ORF1a:V2116-	7	0.26
Karnataka	ORF1b:H2010-	1	0.24				ORF8:D119-	2	1.12	ORF1a:S2114-	6	0.22
Karnataka	ORF1b:H2011-	1	0.24				ORF8:F120-	2	1.12	ORF1a:L2117-	6	0.22
Karnataka	ORF1b:A2012-	1	0.24				S:E156-	2	1.12	ORF1a:G2118-	6	0.22
Karnataka	ORF1b:N2013-	1	0.24				S:F157-	2	1.12	ORF1a:I2107-	5	0.19
Karnataka	ORF1b:E2014-	1	0.24				ORF1a:H83-	1	0.56	ORF1a:K2108-	5	0.19
Karnataka	ORF1b:Y2015-	1	0.24				ORF1a:V84-	1	0.56	ORF1a:K2109-	5	0.19
Karnataka	ORF1b:R2016-	1	0.24				ORF1a:M85-	1	0.56	ORF1a:P2110-	5	0.19
Karnataka	ORF1b:V2034-	1	0.24				ORF1a:V86-	1	0.56	ORF1a:N2111-	5	0.19
Karnataka	ORF1b:Y2035-	1	0.24				ORF1a:E87-	1	0.56	ORF1a:E2112-	5	0.19
Karnataka	ORF1b:K2036-	1	0.24				ORF3a:V255-	1	0.56	ORF1a:L2113-	5	0.19
Karnataka	ORF1b:Q2037-	1	0.24				ORF8:G66-	1	0.56	ORF1a:M85-	5	0.19
Karnataka	ORF1b:F2038-	1	0.24				ORF8:S67-	1	0.56	ORF1a:K141-	5	0.19
Karnataka	ORF1b:D2039-	1	0.24				S:L241-	1	0.56	ORF1a:S142-	5	0.19
Karnataka	ORF1b:T2040-	1	0.24				S:L242-	1	0.56	ORF1a:F143-	5	0.19
Karnataka	ORF1b:Y2041-	1	0.24				S:A243-	1	0.56	ORF1a:A2129-	4	0.15
Karnataka	ORF1b:N2042-	1	0.24				ORF1b:Y2619-	1	0.56	ORF1a:V2130-	4	0.15
Karnataka	ORF1b:L2043-	1	0.24				ORF1b:V2620-	1	0.56	ORF1a:L2095-	3	0.11
Karnataka	ORF1b:W2044-	1	0.24				ORF1b:M2621-	1	0.56	ORF1a:M2096-	3	0.11
Karnataka	ORF1b:N2045-	1	0.24				ORF1b:H2622-	1	0.56	ORF1a:A2097-	3	0.11
Karnataka	ORF1b:T2046-	1	0.24				ORF1b:A2623-	1	0.56	ORF1a:A2098-	3	0.11
Karnataka	ORF1b:F2047-	1	0.24				ORF1b:N2624-	1	0.56	ORF1a:Y2099-	3	0.11
Karnataka	ORF1b:E2054-	1	0.24				ORF1b:K2646-	1	0.56	ORF1a:V2100-	3	0.11
Karnataka	ORF1b:N2055-	1	0.24				ORF1b:F2647-	1	0.56	ORF1a:D2101-	3	0.11
Karnataka	ORF1b:V2056-	1	0.24				ORF1b:P2648-	1	0.56	ORF1a:N2102-	3	0.11
Karnataka	ORF1b:A2057-	1	0.24				ORF1b:L2649-	1	0.56	ORF1a:S2103-	3	0.11
Karnataka	ORF1b:F2058-	1	0.24				ORF1b:K2650-	1	0.56	ORF1a:S2104-	3	0.11

Karnataka	ORF1b:N2059-	1	0.24				ORF1b:L2651-	1	0.56	ORF1a:L2105-	3	0.11
Karnataka	ORF1b:V2060-	1	0.24				ORF1b:R2652-	1	0.56	ORF1a:T2106-	3	0.11
Karnataka	ORF1b:V2061-	1	0.24				ORF1b:G2653-	1	0.56	ORF1a:L2119-	3	0.11
Karnataka	ORF1b:N2062-	1	0.24				ORF1b:T2654-	1	0.56	ORF1a:K2120-	3	0.11
Karnataka	ORF1b:K2063-	1	0.24				ORF1b:A2655-	1	0.56	ORF1a:T2121-	3	0.11
Karnataka	ORF1b:G2064-	1	0.24				ORF1b:V2656-	1	0.56	ORF1a:L2122-	3	0.11
Karnataka	ORF1b:V2082-	1	0.24				ORF1b:M2657-	1	0.56	ORF1a:A2123-	3	0.11
Karnataka	ORF1b:Y2083-	1	0.24				ORF1b:S2658-	1	0.56	ORF1a:T2124-	3	0.11
Karnataka	ORF1b:T2084-	1	0.24				ORF1b:L2659-	1	0.56	ORF1a:H2125-	3	0.11
Karnataka	ORF1b:K2085-	1	0.24				ORF1b:K2660-	1	0.56	ORF1a:G2126-	3	0.11
Karnataka	ORF1b:V2086-	1	0.24				ORF1b:E2661-	1	0.56	ORF1a:L2127-	3	0.11
Karnataka	ORF1b:D2087-	1	0.24				ORF1b:G2662-	1	0.56	ORF1a:A2128-	3	0.11
Karnataka	ORF1b:G2088-	1	0.24				ORF1b:Q2663-	1	0.56	ORF1a:V84-	3	0.11
Karnataka	ORF1b:V2089-	1	0.24				ORF1b:I2664-	1	0.56	N:R209-	2	0.07
Karnataka	ORF1b:D2090-	1	0.24				ORF1b:N2665-	1	0.56	N:M210-	2	0.07
Karnataka	ORF1b:V2091-	1	0.24				ORF1b:D2666-	1	0.56	N:A211-	2	0.07
Karnataka	ORF1b:E2092-	1	0.24				ORF1b:M2667-	1	0.56	N:G212-	2	0.07
Karnataka	ORF1b:L2093-	1	0.24				ORF1b:I2668-	1	0.56	S:N679-	2	0.07
Karnataka	ORF1b:F2094-	1	0.24				ORF1b:L2669-	1	0.56	ORF3a:V255-	2	0.07
Karnataka	ORF1b:E2095-	1	0.24				ORF1b:S2670-	1	0.56	S:K150-	2	0.07
Karnataka	ORF1b:N2096-	1	0.24				ORF1b:L2671-	1	0.56	S:S151-	2	0.07
Karnataka	ORF1b:K2097-	1	0.24				ORF1b:L2672-	1	0.56	S:L56-	2	0.07
Karnataka	ORF1b:N2113-	1	0.24				ORF1b:S2673-	1	0.56	ORF1a:V86-	2	0.07
Karnataka	ORF1b:I2114-	1	0.24				ORF1b:K2674-	1	0.56	ORF1a:N2131-	2	0.07
Karnataka	ORF1b:K2115-	1	0.24				ORF1b:G2675-	1	0.56	ORF1a:S2132-	2	0.07
Karnataka	ORF1b:P2116-	1	0.24				ORF1b:R2676-	1	0.56	ORF1a:V2133-	2	0.07
Karnataka	ORF1b:V2117-	1	0.24				ORF1b:L2677-	1	0.56	ORF1a:P2134-	2	0.07
Karnataka	ORF1b:P2118-	1	0.24				ORF1b:I2678-	1	0.56	ORF1a:W2135-	2	0.07
Karnataka	ORF1b:E2119-	1	0.24				ORF1b:I2679-	1	0.56	ORF1a:D2136-	2	0.07
Karnataka	ORF1b:V2120-	1	0.24				ORF1b:R2680-	1	0.56	ORF1a:T2137-	2	0.07
Karnataka	ORF1b:K2121-	1	0.24				ORF1b:E2681-	1	0.56	ORF1a:I2138-	2	0.07
Karnataka	ORF1b:I2122-	1	0.24				ORF1b:N2682-	1	0.56	S:M1-	2	0.07
Karnataka	ORF1b:L2123-	1	0.24				ORF1b:N2683-	1	0.56	S:F2-	2	0.07
Karnataka	ORF1b:N2124-	1	0.24				ORF1b:R2684-	1	0.56	S:V47-	2	0.07
Karnataka	ORF1b:V2135-	1	0.24				ORF1b:V2685-	1	0.56	S:L48-	2	0.07
Karnataka	ORF1b:I2136-	1	0.24				ORF1b:V2686-	1	0.56	S:S50-	2	0.07
Karnataka	ORF1b:W2137-	1	0.24				ORF1a:K2085-	1	0.56	S:T51-	2	0.07
Karnataka	ORF1b:D2138-	1	0.24				ORF1a:I2086-	1	0.56	S:V62-	2	0.07
Karnataka	ORF1b:Y2139-	1	0.24				ORF1a:G445-	1	0.56	S:T63-	2	0.07
Karnataka	ORF1b:K2140-	1	0.24				ORF1a:L446-	1	0.56	S:I68-	2	0.07
Karnataka	ORF1b:R2141-	1	0.24				ORF8:E19-	1	0.56	N:A208-	2	0.07
Karnataka	ORF1b:D2142-	1	0.24							ORF8:M1-	2	0.07
Karnataka	ORF1b:A2143-	1	0.24							ORF8:K2-	2	0.07
Karnataka	ORF1b:P2144-	1	0.24							ORF1b:N591-	1	0.04
Karnataka	ORF1b:A2145-	1	0.24							ORF1b:M592-	1	0.04
Karnataka	ORF1b:H2146-	1	0.24							ORF1b:L593-	1	0.04
Karnataka	ORF1b:I2147-	1	0.24							ORF1b:K594-	1	0.04
Karnataka	ORF1b:S2148-	1	0.24							ORF1b:T595-	1	0.04
Karnataka	ORF1b:T2149-	1	0.24							ORF1b:V596-	1	0.04
Karnataka	ORF1b:I2150-	1	0.24							ORF1b:Y597-	1	0.04
Karnataka	ORF1b:A2168-	1	0.24							ORF1b:S598-	1	0.04
Karnataka	ORF1b:P2169-	1	0.24							ORF1b:D599-	1	0.04
Karnataka	ORF1b:L2170-	1	0.24							ORF1b:V600-	1	0.04
Karnataka	ORF1b:T2171-	1	0.24							ORF1b:E601-	1	0.04
Karnataka	ORF1b:V2172-	1	0.24							ORF1b:N602-	1	0.04
Karnataka	ORF1b:F2173-	1	0.24							ORF1b:P603-	1	0.04
Karnataka	ORF1b:F2174-	1	0.24							ORF1b:H604-	1	0.04
Karnataka	ORF1b:D2175-	1	0.24							ORF1b:L605-	1	0.04
Karnataka	ORF1b:G2176-	1	0.24							ORF1b:M606-	1	0.04
Karnataka	ORF1b:R2177-	1	0.24							ORF1b:G607-	1	0.04
Karnataka	ORF1b:V2178-	1	0.24							ORF1b:W608-	1	0.04
Karnataka	ORF1b:D2179-	1	0.24							ORF1b:D609-	1	0.04
Karnataka	ORF1b:G2180-	1	0.24							ORF1b:Y610-	1	0.04
Karnataka	ORF1b:Q2181-	1	0.24							ORF1b:P611-	1	0.04
Karnataka	ORF1b:V2182-	1	0.24							ORF1b:K612-	1	0.04
Karnataka	ORF1b:L2193-	1	0.24							ORF1b:C613-	1	0.04
Karnataka	ORF1b:I2194-	1	0.24							ORF1b:D614-	1	0.04
Karnataka	ORF1b:T2195-	1	0.24							ORF1b:K2200-	1	0.04
Karnataka	ORF1b:E2196-	1	0.24							ORF1a:L3198-	1	0.04
Karnataka	ORF1b:G2197-	1	0.24							ORF1a:N1094-	1	0.04
Karnataka	ORF1b:S2198-	1	0.24							ORF1a:V2766-	1	0.04
Karnataka	ORF1b:V2199-	1	0.24							ORF1a:N2082-	1	0.04
Karnataka	ORF1b:K2200-	1	0.24							ORF1a:S2083-	1	0.04

Karnataka	ORF1b:G2201-	1	0.24							ORF1a:N2081-	1	0.04
Karnataka	ORF1b:L2202-	1	0.24							ORF1a:A2139-	1	0.04
Karnataka	ORF1b:Q2203-	1	0.24							ORF1a:N2140-	1	0.04
Karnataka	ORF1b:P2204-	1	0.24							ORF1a:Y2141-	1	0.04
Karnataka	ORF1b:S2205-	1	0.24							ORF1a:A2142-	1	0.04
Karnataka	ORF1b:V2206-	1	0.24							ORF1a:K2143-	1	0.04
Karnataka	ORF1b:G2207-	1	0.24							ORF1a:P2144-	1	0.04
Karnataka	ORF1b:P2208-	1	0.24							ORF1a:F2145-	1	0.04
Karnataka	ORF1b:K2209-	1	0.24							ORF1a:L2146-	1	0.04
Karnataka	ORF1b:G2220-	1	0.24							ORF1a:N2147-	1	0.04
Karnataka	ORF1b:E2221-	1	0.24							ORF1a:K2148-	1	0.04
Karnataka	ORF1b:A2222-	1	0.24							ORF1a:V2149-	1	0.04
Karnataka	ORF1b:V2223-	1	0.24							ORF1a:V2150-	1	0.04
Karnataka	ORF1b:K2224-	1	0.24							ORF1a:S2151-	1	0.04
Karnataka	ORF1b:T2225-	1	0.24							ORF1a:T2152-	1	0.04
Karnataka	ORF1b:Q2226-	1	0.24							ORF1a:T2153-	1	0.04
Karnataka	ORF1b:F2227-	1	0.24							ORF1a:T2154-	1	0.04
Karnataka	ORF1b:N2228-	1	0.24							ORF1a:N2155-	1	0.04
Karnataka	ORF1b:Y2229-	1	0.24							ORF1a:I2156-	1	0.04
Karnataka	ORF1b:Y2230-	1	0.24							ORF1a:V2157-	1	0.04
Karnataka	ORF1b:K2231-	1	0.24							ORF1a:T2158-	1	0.04
Karnataka	ORF1b:K2232-	1	0.24							ORF1a:R2159-	1	0.04
Karnataka	ORF1b:V2233-	1	0.24							ORF1a:C2160-	1	0.04
Karnataka	ORF1b:D2234-	1	0.24							ORF1a:L2161-	1	0.04
Karnataka	ORF1b:G2235-	1	0.24							ORF1a:N2162-	1	0.04
Karnataka	ORF1b:L2265-	1	0.24							ORF1a:R2163-	1	0.04
Karnataka	ORF1b:E2266-	1	0.24							ORF1a:V2164-	1	0.04
Karnataka	ORF1b:L2267-	1	0.24							ORF1a:C2165-	1	0.04
Karnataka	ORF1b:A2268-	1	0.24							ORF1a:T2166-	1	0.04
Karnataka	ORF1b:M2269-	1	0.24							ORF1a:N2167-	1	0.04
Karnataka	ORF1b:D2270-	1	0.24							ORF1a:Y2168-	1	0.04
Karnataka	ORF1b:E2271-	1	0.24							ORF1a:M2169-	1	0.04
Karnataka	ORF1b:F2272-	1	0.24							ORF1a:P2170-	1	0.04
Karnataka	ORF1b:I2273-	1	0.24							ORF1a:Y2171-	1	0.04
Karnataka	ORF1b:E2274-	1	0.24							ORF1a:F2172-	1	0.04
Karnataka	ORF1b:R2275-	1	0.24							ORF1a:F2173-	1	0.04
Karnataka	ORF1b:Y2276-	1	0.24							ORF1a:T2174-	1	0.04
Karnataka	ORF1b:K2277-	1	0.24							ORF1a:L2175-	1	0.04
Karnataka	ORF1b:L2278-	1	0.24							ORF1a:L2176-	1	0.04
Karnataka	ORF1b:E2279-	1	0.24							ORF1a:L2177-	1	0.04
Karnataka	ORF1b:G2280-	1	0.24							ORF1a:Q2178-	1	0.04
Karnataka	ORF1b:Y2281-	1	0.24							ORF1a:L2179-	1	0.04
Karnataka	ORF1b:A2282-	1	0.24							ORF1a:C2180-	1	0.04
Karnataka	ORF1b:H2293-	1	0.24							ORF1a:T2181-	1	0.04
Karnataka	ORF1b:S2294-	1	0.24							ORF1a:F2182-	1	0.04
Karnataka	ORF1b:Q2295-	1	0.24							ORF1a:T2183-	1	0.04
Karnataka	ORF1b:L2296-	1	0.24							ORF1a:R2184-	1	0.04
Karnataka	ORF1b:G2297-	1	0.24							ORF1a:S2185-	1	0.04
Karnataka	ORF1b:G2298-	1	0.24							ORF1a:T2186-	1	0.04
Karnataka	ORF1b:L2299-	1	0.24							ORF1a:N2187-	1	0.04
Karnataka	ORF1b:H2300-	1	0.24							ORF1a:S2188-	1	0.04
Karnataka	ORF1b:L2301-	1	0.24							ORF1a:R2189-	1	0.04
Karnataka	ORF1b:L2302-	1	0.24							ORF1a:I2190-	1	0.04
Karnataka	ORF1b:K2310-	1	0.24							ORF1a:K2191-	1	0.04
Karnataka	ORF1b:E2311-	1	0.24							ORF1a:A2192-	1	0.04
Karnataka	ORF1b:S2312-	1	0.24							ORF1a:S2193-	1	0.04
Karnataka	ORF1b:P2313-	1	0.24							ORF1a:M2194-	1	0.04
Karnataka	ORF1b:F2314-	1	0.24							ORF1a:P2195-	1	0.04
Karnataka	ORF1b:E2315-	1	0.24							ORF1a:T2196-	1	0.04
Karnataka	ORF1b:L2316-	1	0.24							ORF1a:T2197-	1	0.04
Karnataka	ORF1b:E2317-	1	0.24							ORF1a:I2198-	1	0.04
Karnataka	ORF1b:D2318-	1	0.24							ORF1a:A2199-	1	0.04
Karnataka	ORF1b:F2319-	1	0.24							ORF1a:K2200-	1	0.04
Karnataka	ORF1b:I2320-	1	0.24							ORF1a:N2201-	1	0.04
Karnataka	ORF1b:P2321-	1	0.24							ORF1a:T2202-	1	0.04
Karnataka	ORF1b:T2332-	1	0.24							ORF1a:V2203-	1	0.04
Karnataka	ORF1b:D2333-	1	0.24							ORF1a:K2204-	1	0.04
Karnataka	ORF1b:A2334-	1	0.24							ORF1a:S2205-	1	0.04
Karnataka	ORF1b:Q2335-	1	0.24							ORF1a:V2206-	1	0.04
Karnataka	ORF1b:T2336-	1	0.24							ORF1a:G2207-	1	0.04
Karnataka	ORF1b:G2337-	1	0.24							ORF1a:K2208-	1	0.04
Karnataka	ORF1b:S2338-	1	0.24							ORF1a:F2209-	1	0.04
Karnataka	ORF1b:S2339-	1	0.24							ORF1a:C2210-	1	0.04

Karnataka	ORF1b:K2340-	1	0.24							ORF1b:S1942-	1	0.04
Karnataka	ORF1b:C2341-	1	0.24							ORF1b:L1943-	1	0.04
Karnataka	ORF1b:V2342-	1	0.24							ORF1b:Y1944-	1	0.04
Karnataka	ORF1b:L2350-	1	0.24							ORF1b:V1945-	1	0.04
Karnataka	ORF1b:D2351-	1	0.24							ORF1b:N1946-	1	0.04
Karnataka	ORF1b:D2352-	1	0.24							ORF1b:K1947-	1	0.04
Karnataka	ORF1b:F2353-	1	0.24							ORF1b:H1948-	1	0.04
Karnataka	ORF1b:V2354-	1	0.24							ORF1b:A1949-	1	0.04
Karnataka	ORF1b:E2355-	1	0.24							ORF1b:F1950-	1	0.04
Karnataka	ORF1b:I2356-	1	0.24							ORF1b:H1951-	1	0.04
Karnataka	ORF1b:I2357-	1	0.24							ORF1a:K3499-	1	0.04
Karnataka	ORF1b:K2358-	1	0.24							ORF1a:Y3500-	1	0.04
Karnataka	ORF1b:S2359-	1	0.24							ORF1a:N3501-	1	0.04
Karnataka	ORF1b:Q2360-	1	0.24							ORF1a:Y3502-	1	0.04
Karnataka	ORF1b:D2361-	1	0.24							ORF1a:E3503-	1	0.04
Karnataka	ORF1b:L2362-	1	0.24							ORF1a:P3504-	1	0.04
Karnataka	ORF1b:S2363-	1	0.24							ORF1a:L3505-	1	0.04
Karnataka	ORF1b:V2364-	1	0.24							ORF1a:T3506-	1	0.04
Karnataka	ORF1b:V2369-	1	0.24							ORF1a:Q3507-	1	0.04
Karnataka	ORF1b:K2370-	1	0.24							ORF1a:D3508-	1	0.04
Karnataka	ORF1b:V2371-	1	0.24							ORF1a:H3509-	1	0.04
Karnataka	ORF1b:T2372-	1	0.24							ORF1a:V3510-	1	0.04
Karnataka	ORF1b:I2373-	1	0.24							ORF1a:D3511-	1	0.04
Karnataka	ORF1b:D2374-	1	0.24							ORF1a:I3512-	1	0.04
Karnataka	ORF1b:Y2375-	1	0.24							ORF1a:L3513-	1	0.04
Karnataka	ORF1b:T2376-	1	0.24							ORF1a:G3514-	1	0.04
Karnataka	ORF1b:E2377-	1	0.24							ORF1a:P3515-	1	0.04
Karnataka	ORF1b:I2378-	1	0.24							ORF1a:L3516-	1	0.04
Karnataka	ORF1b:S2379-	1	0.24							ORF1a:S3517-	1	0.04
Karnataka	ORF1b:F2380-	1	0.24							ORF1a:A3518-	1	0.04
Karnataka	ORF1b:M2381-	1	0.24							ORF1a:Q3519-	1	0.04
Karnataka	ORF1b:L2382-	1	0.24							ORF1a:T3520-	1	0.04
Karnataka	ORF1b:W2383-	1	0.24							ORF1a:G3521-	1	0.04
Karnataka	ORF1b:C2384-	1	0.24							ORF1a:I3522-	1	0.04
Karnataka	ORF1b:E2390-	1	0.24							ORF1a:A3523-	1	0.04
Karnataka	ORF1b:T2391-	1	0.24							ORF1a:V3524-	1	0.04
Karnataka	ORF1b:F2392-	1	0.24							ORF1a:L3525-	1	0.04
Karnataka	ORF1b:Y2393-	1	0.24							ORF1a:D3526-	1	0.04
Karnataka	ORF1b:P2394-	1	0.24							ORF1a:M3527-	1	0.04
Karnataka	ORF1b:K2395-	1	0.24							ORF1a:C3528-	1	0.04
Karnataka	ORF1b:L2396-	1	0.24							ORF1a:A3529-	1	0.04
Karnataka	ORF1b:Q2397-	1	0.24							ORF1a:S3530-	1	0.04
Karnataka	ORF1b:S2398-	1	0.24							ORF1a:L3531-	1	0.04
Karnataka	ORF1b:S2399-	1	0.24							S:V3-	1	0.04
Karnataka	ORF1b:Q2400-	1	0.24							S:F4-	1	0.04
Karnataka	ORF1b:A2401-	1	0.24							S:L5-	1	0.04
Karnataka	ORF1b:W2402-	1	0.24							S:V6-	1	0.04
Karnataka	ORF1b:C2422-	1	0.24							S:L7-	1	0.04
Karnataka	ORF1b:D2423-	1	0.24							S:L8-	1	0.04
Karnataka	ORF1b:L2424-	1	0.24							S:P9-	1	0.04
Karnataka	ORF1b:Q2425-	1	0.24							S:L10-	1	0.04
Karnataka	ORF1b:P2434-	1	0.24							S:V11-	1	0.04
Karnataka	ORF1b:K2435-	1	0.24							S:S12-	1	0.04
Karnataka	ORF1b:G2436-	1	0.24							S:S13-	1	0.04
Karnataka	ORF1b:I2437-	1	0.24							S:Q14-	1	0.04
Karnataka	ORF1b:M2438-	1	0.24							S:C15-	1	0.04
Karnataka	ORF1b:M2439-	1	0.24							S:V16-	1	0.04
Karnataka	ORF1b:L2456-	1	0.24							S:N17-	1	0.04
Karnataka	ORF1b:A2457-	1	0.24							S:L18-	1	0.04
Karnataka	ORF1b:V2458-	1	0.24							S:T19-	1	0.04
Karnataka	ORF1b:P2459-	1	0.24							S:T20-	1	0.04
Karnataka	ORF1b:Y2460-	1	0.24							S:R21-	1	0.04
Karnataka	ORF1b:N2461-	1	0.24							S:T22-	1	0.04
Karnataka	ORF1b:M2462-	1	0.24							S:Q23-	1	0.04
Karnataka	ORF1b:R2463-	1	0.24							S:L24-	1	0.04
Karnataka	ORF1b:A2469-	1	0.24							S:P25-	1	0.04
Karnataka	ORF1b:G2470-	1	0.24							S:P26-	1	0.04
Karnataka	ORF1b:S2471-	1	0.24							S:A27-	1	0.04
Karnataka	ORF1b:D2472-	1	0.24							S:Y28-	1	0.04
Karnataka	ORF1b:K2473-	1	0.24							S:T29-	1	0.04
Karnataka	ORF1b:G2474-	1	0.24							S:N30-	1	0.04
Karnataka	ORF1b:V2475-	1	0.24							S:S31-	1	0.04
Karnataka	ORF1b:A2476-	1	0.24							S:F32-	1	0.04

Karnataka	ORF1b:P2477-	1	0.24							S:T33-	1	0.04
Karnataka	ORF1b:G2478-	1	0.24							S:R34-	1	0.04
Karnataka	ORF1b:T2479-	1	0.24							S:G35-	1	0.04
Karnataka	ORF1b:A2480-	1	0.24							S:V36-	1	0.04
Karnataka	ORF1b:V2481-	1	0.24							S:Y37-	1	0.04
Karnataka	ORF1b:L2482-	1	0.24							S:Y38-	1	0.04
Karnataka	ORF1b:R2483-	1	0.24							S:P39-	1	0.04
Karnataka	ORF1b:Q2484-	1	0.24							S:D40-	1	0.04
Karnataka	ORF1b:W2485-	1	0.24							S:K41-	1	0.04
Karnataka	ORF1b:L2486-	1	0.24							S:V42-	1	0.04
Karnataka	ORF1b:T2490-	1	0.24							S:F43-	1	0.04
Karnataka	ORF1b:L2491-	1	0.24							S:R44-	1	0.04
Karnataka	ORF1b:L2492-	1	0.24							S:S45-	1	0.04
Karnataka	ORF1b:V2493-	1	0.24							S:S46-	1	0.04
Karnataka	ORF1b:D2494-	1	0.24							S:H49-	1	0.04
Karnataka	ORF1b:S2495-	1	0.24							S:L54-	1	0.04
Karnataka	ORF1b:D2496-	1	0.24							S:F55-	1	0.04
Karnataka	ORF1b:L2497-	1	0.24							S:F59-	1	0.04
Karnataka	ORF1b:N2498-	1	0.24							S:S60-	1	0.04
Karnataka	ORF1b:D2499-	1	0.24							S:N61-	1	0.04
Karnataka	ORF1b:F2500-	1	0.24							S:W64-	1	0.04
Karnataka	ORF1b:V2501-	1	0.24							S:F65-	1	0.04
Karnataka	ORF1b:S2502-	1	0.24							S:H66-	1	0.04
Karnataka	ORF1b:D2503-	1	0.24							S:A67-	1	0.04
Karnataka	ORF1b:A2504-	1	0.24							S:S71-	1	0.04
Karnataka	ORF1b:D2505-	1	0.24							S:G72-	1	0.04
Karnataka	ORF1b:T2517-	1	0.24							S:T73-	1	0.04
Karnataka	ORF1b:A2518-	1	0.24							S:N74-	1	0.04
Karnataka	ORF1b:N2519-	1	0.24							S:G75-	1	0.04
Karnataka	ORF1b:K2520-	1	0.24							S:T76-	1	0.04
Karnataka	ORF1b:W2521-	1	0.24							S:K77-	1	0.04
Karnataka	ORF1b:D2522-	1	0.24							S:R78-	1	0.04
Karnataka	ORF1b:L2523-	1	0.24							S:F79-	1	0.04
Karnataka	ORF1b:I2524-	1	0.24							S:D80-	1	0.04
Karnataka	ORF1b:I2525-	1	0.24							S:N81-	1	0.04
Karnataka	ORF1b:S2526-	1	0.24							S:P82-	1	0.04
Karnataka	ORF1b:D2527-	1	0.24							S:V83-	1	0.04
Karnataka	ORF1b:M2528-	1	0.24							S:L84-	1	0.04
Karnataka	ORF1b:Y2529-	1	0.24							S:P85-	1	0.04
Karnataka	ORF1b:D2530-	1	0.24							S:F86-	1	0.04
Karnataka	ORF1b:P2531-	1	0.24							S:N87-	1	0.04
Karnataka	ORF1b:Y2549-	1	0.24							S:D88-	1	0.04
Karnataka	ORF1b:I2550-	1	0.24							S:G89-	1	0.04
Karnataka	ORF1b:C2551-	1	0.24							S:V90-	1	0.04
Karnataka	ORF1b:G2552-	1	0.24							S:Y91-	1	0.04
Karnataka	ORF1b:F2553-	1	0.24							S:F92-	1	0.04
Karnataka	ORF1b:I2554-	1	0.24							S:A93-	1	0.04
Karnataka	ORF1b:Q2555-	1	0.24							S:S94-	1	0.04
Karnataka	ORF1b:Q2556-	1	0.24							S:T95-	1	0.04
Karnataka	ORF1b:K2557-	1	0.24							S:E96-	1	0.04
Karnataka	ORF1b:S2597-	1	0.24							S:K97-	1	0.04
Karnataka	ORF1b:S2598-	1	0.24							S:S98-	1	0.04
Karnataka	ORF1b:S2599-	1	0.24							S:N99-	1	0.04
Karnataka	ORF1b:E2600-	1	0.24							S:I100-	1	0.04
Karnataka	ORF1b:A2601-	1	0.24							S:I101-	1	0.04
Karnataka	ORF1b:F2602-	1	0.24							S:R102-	1	0.04
Karnataka	ORF1b:L2603-	1	0.24							S:G103-	1	0.04
Karnataka	ORF1b:I2604-	1	0.24							S:W104-	1	0.04
Karnataka	ORF1b:G2605-	1	0.24							S:I105-	1	0.04
Karnataka	ORF1b:C2606-	1	0.24							S:F106-	1	0.04
Karnataka	ORF1b:N2607-	1	0.24							S:G107-	1	0.04
Karnataka	ORF1b:Y2608-	1	0.24							S:T108-	1	0.04
Karnataka	ORF1b:L2609-	1	0.24							S:T109-	1	0.04
Karnataka	ORF1b:G2610-	1	0.24							S:L110-	1	0.04
Karnataka	ORF1b:K2611-	1	0.24							S:D111-	1	0.04
Karnataka	ORF1b:P2612-	1	0.24							S:S112-	1	0.04
Karnataka	ORF1b:R2613-	1	0.24							S:K113-	1	0.04
Karnataka	ORF1b:E2614-	1	0.24							S:T114-	1	0.04
Karnataka	ORF1b:Q2615-	1	0.24							S:Q115-	1	0.04
Karnataka	ORF1b:I2616-	1	0.24							S:S116-	1	0.04
Karnataka	ORF1b:D2617-	1	0.24							S:L117-	1	0.04
Karnataka	ORF1b:G2618-	1	0.24							S:L118-	1	0.04
Karnataka	ORF1b:Y2619-	1	0.24							S:I119-	1	0.04

Karnataka	ORF1b:V2620-	1	0.24							S:V120-	1	0.04
Karnataka	ORF1b:M2621-	1	0.24							S:N121-	1	0.04
Karnataka	ORF1b:H2622-	1	0.24							S:N122-	1	0.04
Karnataka	ORF1b:A2623-	1	0.24							S:A123-	1	0.04
Karnataka	ORF1b:N2624-	1	0.24							S:T124-	1	0.04
Karnataka	ORF1b:Y2625-	1	0.24							S:N125-	1	0.04
Karnataka	ORF1b:I2626-	1	0.24							S:V126-	1	0.04
Karnataka	ORF1b:F2627-	1	0.24							S:V127-	1	0.04
Karnataka	ORF1b:W2628-	1	0.24							S:I128-	1	0.04
Karnataka	ORF1b:R2629-	1	0.24							S:K129-	1	0.04
Karnataka	ORF1b:N2630-	1	0.24							S:V130-	1	0.04
Karnataka	ORF1b:T2631-	1	0.24							S:C131-	1	0.04
Karnataka	ORF1b:N2632-	1	0.24							S:E132-	1	0.04
Karnataka	ORF1b:P2633-	1	0.24							S:F168-	1	0.04
Karnataka	ORF1b:I2634-	1	0.24							S:L176-	1	0.04
Karnataka	ORF1b:Q2635-	1	0.24							S:M177-	1	0.04
Karnataka	ORF1b:L2636-	1	0.24							S:D178-	1	0.04
Karnataka	ORF1b:S2637-	1	0.24							S:L179-	1	0.04
Karnataka	ORF1b:S2638-	1	0.24							S:E180-	1	0.04
Karnataka	ORF1b:Y2639-	1	0.24							S:G181-	1	0.04
Karnataka	ORF1b:S2640-	1	0.24							S:K182-	1	0.04
Karnataka	ORF1b:L2641-	1	0.24							S:Q183-	1	0.04
Karnataka	ORF1b:F2642-	1	0.24							S:G184-	1	0.04
Karnataka	ORF1b:D2643-	1	0.24							S:N185-	1	0.04
Karnataka	ORF1b:M2644-	1	0.24							S:Y200-	1	0.04
Karnataka	ORF1b:S2645-	1	0.24							S:F201-	1	0.04
Karnataka	ORF1b:K2646-	1	0.24							S:K202-	1	0.04
Karnataka	ORF1b:F2647-	1	0.24							S:I203-	1	0.04
Karnataka	ORF1b:P2648-	1	0.24							S:Y204-	1	0.04
Karnataka	ORF1b:L2649-	1	0.24							S:S205-	1	0.04
Karnataka	ORF1b:K2650-	1	0.24							S:K206-	1	0.04
Karnataka	ORF1b:L2651-	1	0.24							S:H207-	1	0.04
Karnataka	ORF1b:R2652-	1	0.24							ORF7a:I103-	1	0.04
Karnataka	ORF1b:G2653-	1	0.24							ORF7a:V104-	1	0.04
Karnataka	ORF1b:T2654-	1	0.24							ORF7a:F63-	1	0.04
Karnataka	ORF1b:A2655-	1	0.24							ORF7a:A64-	1	0.04
Karnataka	ORF1b:V2656-	1	0.24							ORF7a:F65-	1	0.04
Karnataka	ORF1b:M2657-	1	0.24							ORF7a:A66-	1	0.04
Karnataka	ORF1b:S2658-	1	0.24							ORF7a:C67-	1	0.04
Karnataka	ORF1b:L2659-	1	0.24							ORF7a:P68-	1	0.04
Karnataka	ORF1b:K2660-	1	0.24							ORF7a:D69-	1	0.04
Karnataka	ORF1b:E2661-	1	0.24							ORF7a:G70-	1	0.04
Karnataka	ORF1b:G2662-	1	0.24							ORF7a:V71-	1	0.04
Karnataka	ORF1b:Q2663-	1	0.24							ORF7a:K72-	1	0.04
Karnataka	ORF1b:I2664-	1	0.24							ORF7a:H73-	1	0.04
Karnataka	ORF1b:N2665-	1	0.24							ORF7a:V74-	1	0.04
Karnataka	ORF1b:D2666-	1	0.24							ORF7a:Y75-	1	0.04
Karnataka	ORF1b:M2667-	1	0.24							ORF7a:Q76-	1	0.04
Karnataka	ORF1b:I2668-	1	0.24							ORF7a:L77-	1	0.04
Karnataka	ORF1b:L2669-	1	0.24							S:V143-	1	0.04
Karnataka	ORF1b:S2670-	1	0.24									
Karnataka	ORF1b:L2671-	1	0.24									
Karnataka	ORF1b:L2672-	1	0.24									
Karnataka	ORF1b:S2673-	1	0.24									
Karnataka	ORF1b:K2674-	1	0.24									
Karnataka	ORF1b:G2675-	1	0.24									
Karnataka	ORF1b:R2676-	1	0.24									
Karnataka	ORF1b:L2677-	1	0.24									
Karnataka	ORF1b:I2678-	1	0.24									
Karnataka	ORF1b:I2679-	1	0.24									
Karnataka	ORF1b:R2680-	1	0.24									
Karnataka	ORF1b:E2681-	1	0.24									
Karnataka	ORF1b:N2682-	1	0.24									
Karnataka	ORF1b:N2683-	1	0.24									
Karnataka	ORF1b:R2684-	1	0.24									
Karnataka	ORF1b:V2685-	1	0.24									
Karnataka	ORF1b:V2686-	1	0.24									
Karnataka	ORF1b:I2687-	1	0.24									
Karnataka	ORF1b:S2688-	1	0.24									
Karnataka	ORF1b:S2689-	1	0.24									
Karnataka	ORF1b:D2690-	1	0.24									
Karnataka	ORF1b:V2691-	1	0.24									
Karnataka	ORF1b:L2692-	1	0.24									

Karnataka	ORF1b:V2693-	1	0.24									
Karnataka	ORF1b:N2694-	1	0.24									
Karnataka	ORF1b:N2695-	1	0.24									
Karnataka	ORF1b:*2696-	1	0.24									
Karnataka	ORF1a:G82-	1	0.24									
Karnataka	ORF1a:H83-	1	0.24									
Karnataka	ORF1a:V86-	1	0.24									
Karnataka	Nucleotide deletions											
Karnataka	Pre-Wave 1			Wave 1			Pre-Wave 2			Wave 2		
Karnataka	Mutation	Count	percent	Mutation	Count	percent	Mutation	Count	percent	Mutation	Count	percent
	36-44	9	8.49	21992-21994	16	27.59	21992-21994	18	20	28271	624	34.82
	29698-29705	8	7.55	11288-11296	15	25.86	11288-11296	16	17.78	28248-28253	511	28.52
	23	3	2.83	21765-21770	15	25.86	21765-21770	15	16.67	22029-22034	462	25.78
	25-37	3	2.83	29738-29748	2	3.45	28271	8	8.89	21992-21994	26	1.45
	29687-29783	3	2.83	28182-28221	1	1.72	18349	2	2.22	21734-21739	23	1.28
	29792-29831	3	2.83	26170-26174	1	1.72	29730-29768	2	2.22	11288-11296	23	1.28
	22-30	3	2.83	25393-25395	1	1.72	22029-22034	2	2.22	21765-21770	22	1.23
	29868	3	2.83	22289-22294	1	1.72	28248-28253	2	2.22	21719-21724	22	1.23
	25-43	2	1.89	26158-26161	1	1.72	512-526	1	1.11	686-694	5	0.28
	23489	2	1.89	27268-27292	1	1.72	19458	1	1.11	6551-6622	2	0.11
	11083	2	1.89	29729-29759	1	1.72	18656	1	1.11	6584-6655	2	0.11
	21-24	2	1.89	515-520	1	1.72	29867	1	1.11	28898-28909	2	0.11
	29861-29867	2	1.89	2867-2869	1	1.72	3566	1	1.11	23598-23600	2	0.11
	686-694	2	1.89	6512-6523	1	1.72	4068	1	1.11	515-520	2	0.11
	28	2	1.89				6167	1	1.11	26155-26157	2	0.11
	29687	2	1.89				11858-11859	1	1.11	22013-22018	2	0.11
	29673-29674	1	0.94				14570-14577	1	1.11	19291-19386	2	0.11
	29864-29867	1	0.94				23036	1	1.11	19502-19506	2	0.11
	23762-23765	1	0.94				27784	1	1.11	27887-27901	2	0.11
	29849	1	0.94				22430-22431	1	1.11	28896-28898	2	0.11
	29860	1	0.94				29278	1	1.11	241-242	2	0.11
	21403-21415	1	0.94				26155-26157	1	1.11	6548-6619	1	0.06
	21556-21568	1	0.94				28090-28095	1	1.11	21680-21732	1	0.06
	23666-23667	1	0.94				22283-22291	1	1.11	21782-22084	1	0.06
	29712-29716	1	0.94				782	1	1.11	21830-22202	1	0.06
	29866-29867	1	0.94				19300	1	1.11	15238-15309	1	0.06
	15-43	1	0.94				29000	1	1.11	20065-20067	1	0.06
	27933-28220	1	0.94				21166-21297	1	1.11	29724-29775	1	0.06
	22-24	1	0.94				21436-21465	1	1.11	21731-21734	1	0.06
	14-21	1	0.94				4865-4965	1	1.11	26161-26177	1	0.06
	25-34	1	0.94				6518-6523	1	1.11	27792-27793	1	0.06
	8336-8355	1	0.94				1598-1603	1	1.11	6605-6613	1	0.06
	19295-19303	1	0.94				27948-27950	1	1.11	29741-29743	1	0.06
	19312-19379	1	0.94							9857-9859	1	0.06
	21235-21457	1	0.94							3546-3548	1	0.06
	7010-7049	1	0.94							21714-21715	1	0.06
	25-32	1	0.94							21556-21561	1	0.06
	11-19	1	0.94							8562-8564	1	0.06
	11096-11100	1	0.94							518-520	1	0.06
	16636-16645	1	0.94							27888-27893	1	0.06
	16651-16678	1	0.94							21728-21732	1	0.06
	21403-21408	1	0.94							6510-6515	1	0.06
	8837-8853	1	0.94							21728-21733	1	0.06
	75-76	1	0.94							6506-6508	1	0.06
	4529	1	0.94							28233-28238	1	0.06
	4677-4691	1	0.94							21731-21736	1	0.06
	4701-4712	1	0.94							521-523	1	0.06
	4718-4729	1	0.94							6608-6895	1	0.06
	4745-4748	1	0.94							4835-4935	1	0.06
	4757-4878	1	0.94							19291-19320	1	0.06
	4889-4937	1	0.94							10766-10864	1	0.06
	6701-6705	1	0.94							21776-21871	1	0.06
	6715-6716	1	0.94							21888-21889	1	0.06
	6722-6724	1	0.94							21917-22193	1	0.06
	10929-11009	1	0.94							10797-10969	1	0.06
	23359-23387	1	0.94							21436-21554	1	0.06
	23396-23435	1	0.94							2585-2700	1	0.06
	26287-26292	1	0.94							21621-21622	1	0.06
	26296-26297	1	0.94							21638-21699	1	0.06
	29863-29865	1	0.94							27700-27705	1	0.06
	32-36	1	0.94							515-523	1	0.06
	23256-23259	1	0.94							27580-27624	1	0.06

	**29687-29689**	1	0.94							**21701-21706**	1	0.06
	**21352-21353**	1	0.94							**21710-21715**	1	0.06
	**519-524**	1	0.94							**6654-6683**	1	0.06
	**4**	1	0.94							**516-518**	1	0.06
	**509-523**	1	0.94							**21717-21722**	1	0.06
	**6875-6930**	1	0.94							**21656-21661**	1	0.06
	**6884-6950**	1	0.94							**21767-21772**	1	0.06
	**21656**	1	0.94							**21749-21754**	1	0.06
	**29783**	1	0.94							**29723-29775**	1	0.06
Maharashtra	**ORF1a**											
Maharashtra	**Pre-Wave 1**			**Wave 1**			**Pre-Wave 2**			**Wave 2**		
Maharashtra	**Mutation**	**Count**	**percent**	**Mutation**	**Count**	**percent**	**Mutation**	**Count**	**percent**	**Mutation**	**Count**	**percent**
Maharashtra	**ORF1a:A1812D**	404	58.89	**ORF1a:A1812D**	480	61.3	**ORF1a:T3646A**	182	27.74	**ORF1a:T3646A**	1867	52.77
Maharashtra	**ORF1a:L3606F**	50	7.29	**ORF1a:V3718F**	51	6.51	**ORF1a:T1567I**	177	26.98	**ORF1a:T1567I**	1500	42.4
Maharashtra	**ORF1a:T2016K**	44	6.41	**ORF1a:L3606F**	42	5.36	**ORF1a:T1001I**	128	19.51	**ORF1a:P1640L**	777	21.96
Maharashtra	**ORF1a:Q2702H**	17	2.48	**ORF1a:Q2702H**	30	3.83	**ORF1a:A1812D**	69	10.52	**ORF1a:V3718A**	662	18.71
Maharashtra	**ORF1a:P2739S**	17	2.48	**ORF1a:A1049V**	21	2.68	**ORF1a:D3042N**	40	6.1	**ORF1a:A3209V**	657	18.57
Maharashtra	**ORF1a:G519S**	13	1.9	**ORF1a:V561A**	20	2.55	**ORF1a:L3606F**	34	5.18	**ORF1a:T3750I**	417	11.79
Maharashtra	**ORF1a:Y4077H**	11	1.6	**ORF1a:N2498H**	19	2.43	**ORF1a:V561A**	26	3.96	**ORF1a:T3255I**	368	10.4
Maharashtra	**ORF1a:L204F**	10	1.46	**ORF1a:T3589A**	19	2.43	**ORF1a:I2230T**	24	3.66	**ORF1a:V2930L**	366	10.34
Maharashtra	**ORF1a:A3615V**	9	1.31	**ORF1a:S1520F**	14	1.79	**ORF1a:M902V**	24	3.66	**ORF1a:A1306S**	363	10.26
Maharashtra	**ORF1a:D1939G**	9	1.31	**ORF1a:K2143R**	14	1.79	**ORF1a:A1708D**	23	3.51	**ORF1a:P2046L**	363	10.26
Maharashtra	**ORF1a:S2015R**	9	1.31	**ORF1a:T1001I**	13	1.66	**ORF1a:V2586G**	23	3.51	**ORF1a:P2287S**	358	10.12
Maharashtra	**ORF1a:S3913L**	9	1.31	**ORF1a:A690V**	13	1.66	**ORF1a:V3653F**	22	3.35	**ORF1a:P309L**	348	9.84
Maharashtra	**ORF1a:S2193F**	8	1.17	**ORF1a:D3042N**	12	1.53	**ORF1a:K3353R**	19	2.9	**ORF1a:T1001I**	193	5.46
Maharashtra	**ORF1a:V3718F**	7	1.02	**ORF1a:H2659Y**	12	1.53	**ORF1a:Q3777R**	17	2.59	**ORF1a:A3457S**	117	3.31
Maharashtra	**ORF1a:A690V**	6	0.87	**ORF1a:P1803S**	12	1.53	**ORF1a:S838I**	16	2.44	**ORF1a:T2648I**	115	3.25
Maharashtra	**ORF1a:A339V**	6	0.87	**ORF1a:P309S**	11	1.4	**ORF1a:M3752I**	15	2.29	**ORF1a:A1812D**	114	3.22
Maharashtra	**ORF1a:V378I**	6	0.87	**ORF1a:A4016V**	10	1.28	**ORF1a:V54I**	14	2.13	**ORF1a:F3138S**	106	3
Maharashtra	**ORF1a:T4031I**	6	0.87	**ORF1a:A1314T**	10	1.28	**ORF1a:V3718F**	14	2.13	**ORF1a:A3686V**	106	3
Maharashtra	**ORF1a:T951I**	5	0.73	**ORF1a:K3353R**	10	1.28	**ORF1a:T2648I**	14	2.13	**ORF1a:A2344V**	105	2.97
Maharashtra	**ORF1a:A1679V**	5	0.73	**ORF1a:L204F**	10	1.28	**ORF1a:A3457S**	14	2.13	**ORF1a:D2980N**	102	2.88
Maharashtra	**ORF1a:D2060Y**	5	0.73	**ORF1a:Y4077H**	9	1.15	**ORF1a:A3686V**	14	2.13	**ORF1a:A1708D**	96	2.71
Maharashtra	**ORF1a:V2783F**	5	0.73	**ORF1a:A3523V**	9	1.15	**ORF1a:A4016V**	13	1.98	**ORF1a:V54I**	95	2.69
Maharashtra	**ORF1a:D4043G**	5	0.73	**ORF1a:H1160Y**	9	1.15	**ORF1a:M315V**	12	1.83	**ORF1a:I2230T**	93	2.63
Maharashtra	**ORF1a:Y925C**	5	0.73	**ORF1a:T4090I**	8	1.02	**ORF1a:S1361F**	12	1.83	**ORF1a:L3606F**	88	2.49
Maharashtra	**ORF1a:R398C**	5	0.73	**ORF1a:S2151G**	8	1.02	**ORF1a:A2344V**	12	1.83	**ORF1a:H3580Q**	83	2.35
Maharashtra	**ORF1a:P1472S**	4	0.58	**ORF1a:V2586G**	7	0.89	**ORF1a:S302F**	12	1.83	**ORF1a:M315V**	82	2.32
Maharashtra	**ORF1a:K3577R**	4	0.58	**ORF1a:N854D**	7	0.89	**ORF1a:P309S**	11	1.68	**ORF1a:A819T**	60	1.7
Maharashtra	**ORF1a:P380L**	4	0.58	**ORF1a:S1188L**	6	0.77	**ORF1a:P1640L**	10	1.52	**ORF1a:H2092Y**	48	1.36
Maharashtra	**ORF1a:F536C**	4	0.58	**ORF1a:A2249V**	6	0.77	**ORF1a:P309L**	8	1.22	**ORF1a:K3353R**	41	1.16
Maharashtra	**ORF1a:R1628C**	4	0.58	**ORF1a:M3655V**	6	0.77	**ORF1a:T2183I**	8	1.22	**ORF1a:F3677L**	39	1.1
Maharashtra	**ORF1a:R3993H**	4	0.58	**ORF1a:E1498V**	6	0.77	**ORF1a:A819T**	8	1.22	**ORF1a:I222V**	36	1.02
Maharashtra	**ORF1a:V561A**	4	0.58	**ORF1a:M3669I**	6	0.77	**ORF1a:G3676C**	7	1.07	**ORF1a:L730F**	35	0.99
Maharashtra	**ORF1a:S40T**	4	0.58	**ORF1a:M3425I**	6	0.77	**ORF1a:V3759F**	7	1.07	**ORF1a:D3042N**	33	0.93
Maharashtra	**ORF1a:N2671Y**	4	0.58	**ORF1a:P1921L**	6	0.77	**ORF1a:A1352V**	7	1.07	**ORF1a:A1397V**	31	0.88
Maharashtra	**ORF1a:G524S**	4	0.58	**ORF1a:V1751M**	6	0.77	**ORF1a:L1131F**	7	1.07	**ORF1a:L3027F**	31	0.88
Maharashtra	**ORF1a:L3483F**	4	0.58	**ORF1a:T265I**	6	0.77	**ORF1a:P971L**	6	0.91	**ORF1a:A3620V**	28	0.79
Maharashtra	**ORF1a:M2957T**	4	0.58	**ORF1a:T1538I**	5	0.64	**ORF1a:L3027F**	6	0.91	**ORF1a:A1352T**	24	0.68
Maharashtra	**ORF1a:A687V**	4	0.58	**ORF1a:V111L**	5	0.64	**ORF1a:S1612L**	6	0.91	**ORF1a:M3752I**	24	0.68
Maharashtra	**ORF1a:P1786L**	3	0.44	**ORF1a:K1202N**	5	0.64	**ORF1a:E633D**	6	0.91	**ORF1a:L3930F**	23	0.65
Maharashtra	**ORF1a:S1587L**	3	0.44	**ORF1a:A2832V**	5	0.64	**ORF1a:Q1140L**	6	0.91	**ORF1a:L2062F**	23	0.65
Maharashtra	**ORF1a:G1288V**	3	0.44	**ORF1a:T3716A**	5	0.64	**ORF1a:E102A**	6	0.91	**ORF1a:Y2845L**	21	0.59
Maharashtra	**ORF1a:I4308M**	3	0.44	**ORF1a:E976K**	5	0.64	**ORF1a:T554N**	6	0.91	**ORF1a:M902V**	21	0.59
Maharashtra	**ORF1a:A872T**	3	0.44	**ORF1a:T253A**	5	0.64	**ORF1a:T2152I**	6	0.91	**ORF1a:V561A**	20	0.57
Maharashtra	**ORF1a:Q1918H**	3	0.44	**ORF1a:M3761V**	5	0.64	**ORF1a:K2511N**	5	0.76	**ORF1a:A4257T**	20	0.57
Maharashtra	**ORF1a:A307V**	3	0.44	**ORF1a:E913D**	5	0.64	**ORF1a:P1692S**	5	0.76	**ORF1a:T2007I**	20	0.57
Maharashtra	**ORF1a:A2994F**	3	0.44	**ORF1a:E933G**	4	0.51	**ORF1a:T1881I**	5	0.76	**ORF1a:E1377L**	20	0.57
Maharashtra	**ORF1a:A2123V**	3	0.44	**ORF1a:S2103F**	4	0.51	**ORF1a:I404T**	5	0.76	**ORF1a:M3934V**	20	0.57
Maharashtra	**ORF1a:V2453G**	3	0.44	**ORF1a:T3255I**	4	0.51	**ORF1a:Q3777H**	5	0.76	**ORF1a:A1649I**	19	0.54
Maharashtra	**ORF1a:N2454S**	3	0.44	**ORF1a:V3653F**	4	0.51	**ORF1a:V2862L**	5	0.76	**ORF1a:V2586G**	19	0.54
Maharashtra	**ORF1a:E3188V**	3	0.44	**ORF1a:G2241S**	4	0.51	**ORF1a:P1921S**	5	0.76	**ORF1a:I3587G**	18	0.51
Maharashtra	**ORF1a:M3189A**	3	0.44	**ORF1a:T568I**	4	0.51	**ORF1a:T2823I**	5	0.76	**ORF1a:A1679V**	18	0.51
Maharashtra	**ORF1a:S318L**	3	0.44	**ORF1a:D928Y**	4	0.51	**ORF1a:P1147S**	5	0.76	**ORF1a:M2365I**	18	0.51
Maharashtra	**ORF1a:H2659Y**	3	0.44	**ORF1a:M3752I**	4	0.51	**ORF1a:S2103F**	5	0.76	**ORF1a:D1440Q**	17	0.48
Maharashtra	**ORF1a:V2061F**	3	0.44	**ORF1a:T1881I**	4	0.51	**ORF1a:S2493I**	4	0.61	**ORF1a:G2509L**	17	0.48
Maharashtra	**ORF1a:A1314T**	3	0.44	**ORF1a:T4129I**	4	0.51	**ORF1a:M3934I**	4	0.61	**ORF1a:Q3777R**	17	0.48
Maharashtra	**ORF1a:S1520F**	3	0.44	**ORF1a:Y3722C**	4	0.51	**ORF1a:A1679V**	4	0.61	**ORF1a:E1564A**	17	0.48
Maharashtra	**ORF1a:H110Y**	3	0.44	**ORF1a:P2046L**	4	0.51	**ORF1a:V3097A**	4	0.61	**ORF1a:D1903T**	17	0.48
Maharashtra	**ORF1a:A2129V**	3	0.44	**ORF1a:V60I**	4	0.51	**ORF1a:K1247N**	4	0.61	**ORF1a:H1108Y**	17	0.48

Maharashtra	**ORF1a:T568I**	2	0.29	**ORF1a:P1640S**	4	0.51	**ORF1a:V1056L**	4	0.61	**ORF1a:M3087I**	16	0.45
Maharashtra	**ORF1a:V3892I**	2	0.29	**ORF1a:G2269C**	4	0.51	**ORF1a:L3935M**	4	0.61	**ORF1a:K2497N**	16	0.45
Maharashtra	**ORF1a:V3660L**	2	0.29	**ORF1a:V3892I**	4	0.51	**ORF1a:L3667F**	4	0.61	**ORF1a:D1275***	16	0.45
Maharashtra	**ORF1a:T2183I**	2	0.29	**ORF1a:S3913L**	4	0.51	**ORF1a:T170I**	4	0.61	**ORF1a:S2312L**	16	0.45
Maharashtra	**ORF1a:M3087I**	2	0.29	**ORF1a:S1856Y**	4	0.51	**ORF1a:L681F**	4	0.61	**ORF1a:G3412L**	15	0.42
Maharashtra	**ORF1a:P153L**	2	0.29	**ORF1a:K680N**	4	0.51	**ORF1a:P959S**	4	0.61	**ORF1a:H3233***	15	0.42
Maharashtra	**ORF1a:A3523T**	2	0.29	**ORF1a:T1240A**	4	0.51	**ORF1a:A3454V**	4	0.61	**ORF1a:A3235Q**	15	0.42
Maharashtra	**ORF1a:M85V**	2	0.29	**ORF1a:T2283I**	4	0.51	**ORF1a:A4162V**	4	0.61	**ORF1a:K3236R**	15	0.42
Maharashtra	**ORF1a:T1682P**	2	0.29	**ORF1a:S901R**	4	0.51	**ORF1a:S944L**	4	0.61	**ORF1a:A3237S**	15	0.42
Maharashtra	**ORF1a:K1837L**	2	0.29	**ORF1a:P1692S**	4	0.51	**ORF1a:V2133I**	4	0.61	**ORF1a:L3238C**	15	0.42
Maharashtra	**ORF1a:H1838V**	2	0.29	**ORF1a:T4083M**	3	0.38	**ORF1a:S4398L**	4	0.61	**ORF1a:N3239L**	15	0.42
Maharashtra	**ORF1a:K1842V**	2	0.29	**ORF1a:Q3777R**	3	0.38	**ORF1a:T1241I**	4	0.61	**ORF1a:D3240L**	15	0.42
Maharashtra	**ORF1a:E1843V**	2	0.29	**ORF1a:T814I**	3	0.38	**ORF1a:S376P**	4	0.61	**ORF1a:F3241S**	15	0.42
Maharashtra	**ORF1a:I1877K**	2	0.29	**ORF1a:A4277T**	3	0.38	**ORF1a:T3255I**	4	0.61	**ORF1a:N3243R**	15	0.42
Maharashtra	**ORF1a:P1879T**	2	0.29	**ORF1a:V274A**	3	0.38	**ORF1a:K2589R**	3	0.46	**ORF1a:S3244K**	15	0.42
Maharashtra	**ORF1a:Y1882Q**	2	0.29	**ORF1a:P4075S**	3	0.38	**ORF1a:E995D**	3	0.46	**ORF1a:A1314L**	15	0.42
Maharashtra	**ORF1a:P1919I**	2	0.29	**ORF1a:P286S**	3	0.38	**ORF1a:A2832V**	3	0.46	**ORF1a:A1316C**	15	0.42
Maharashtra	**ORF1a:Y1920Q**	2	0.29	**ORF1a:A591V**	3	0.38	**ORF1a:T2408I**	3	0.46	**ORF1a:L1317***	15	0.42
Maharashtra	**ORF1a:P1921T**	2	0.29	**ORF1a:P2110S**	3	0.38	**ORF1a:T2274I**	3	0.46	**ORF1a:C1349V**	15	0.42
Maharashtra	**ORF1a:N1922Q**	2	0.29	**ORF1a:K3979N**	3	0.38	**ORF1a:A735S**	3	0.46	**ORF1a:K1350P**	15	0.42
Maharashtra	**ORF1a:N2065Y**	2	0.29	**ORF1a:V3759F**	3	0.38	**ORF1a:G963C**	3	0.46	**ORF1a:S1351F**	15	0.42
Maharashtra	**ORF1a:V2066R**	2	0.29	**ORF1a:V2061F**	3	0.38	**ORF1a:V3475I**	3	0.46	**ORF1a:I1355H**	15	0.42
Maharashtra	**ORF1a:T2069F**	2	0.29	**ORF1a:G445S**	3	0.38	**ORF1a:K4232R**	3	0.46	**ORF1a:P1357L**	15	0.42
Maharashtra	**ORF1a:E2070L**	2	0.29	**ORF1a:E2049K**	3	0.38	**ORF1a:C4381W**	3	0.46	**ORF1a:T1370R**	15	0.42
Maharashtra	**ORF1a:V2071S**	2	0.29	**ORF1a:L3935M**	3	0.38	**ORF1a:L642F**	3	0.46	**ORF1a:N1374F**	15	0.42
Maharashtra	**ORF1a:G2073Q**	2	0.29	**ORF1a:A3956V**	3	0.38	**ORF1a:L3338F**	3	0.46	**ORF1a:M1389K**	15	0.42
Maharashtra	**ORF1a:D2074Q**	2	0.29	**ORF1a:S74W**	3	0.38	**ORF1a:G150S**	3	0.46	**ORF1a:T1401***	15	0.42
Maharashtra	**ORF1a:E2112L**	2	0.29	**ORF1a:E1223G**	3	0.38	**ORF1a:K1202N**	3	0.46	**ORF1a:I1402F**	15	0.42
Maharashtra	**ORF1a:P2144L**	2	0.29	**ORF1a:A2123V**	3	0.38	**ORF1a:T3308I**	3	0.46	**ORF1a:R1404L**	15	0.42
Maharashtra	**ORF1a:N2147T**	2	0.29	**ORF1a:A540V**	3	0.38	**ORF1a:V1025L**	3	0.46	**ORF1a:Y1423T**	15	0.42
Maharashtra	**ORF1a:T2174V**	2	0.29	**ORF1a:S279F**	3	0.38	**ORF1a:M4009V**	3	0.46	**ORF1a:G1531E**	15	0.42
Maharashtra	**ORF1a:A2279***	2	0.29	**ORF1a:H388Y**	3	0.38	**ORF1a:A1997V**	3	0.46	**ORF1a:D1532V**	15	0.42
Maharashtra	**ORF1a:S2297K**	2	0.29	**ORF1a:Y1337N**	3	0.38	**ORF1a:V2816A**	3	0.46	**ORF1a:K1533I**	15	0.42
Maharashtra	**ORF1a:T2335W**	2	0.29	**ORF1a:P2685S**	3	0.38	**ORF1a:V721N**	3	0.46	**ORF1a:S1534K**	15	0.42
Maharashtra	**ORF1a:R2336H**	2	0.29	**ORF1a:R24C**	3	0.38	**ORF1a:M1312I**	3	0.46	**ORF1a:Y1537I**	15	0.42
Maharashtra	**ORF1a:F2337I**	2	0.29	**ORF1a:P943S**	3	0.38	**ORF1a:K2497N**	3	0.46	**ORF1a:H1613Y**	15	0.42
Maharashtra	**ORF1a:Y2339F**	2	0.29	**ORF1a:H1108Y**	3	0.38	**ORF1a:L3930F**	3	0.46	**ORF1a:R3164L**	15	0.42
Maharashtra	**ORF1a:V2340S**	2	0.29	**ORF1a:D827N**	3	0.38	**ORF1a:A1074T**	3	0.46	**ORF1a:N3168P**	14	0.4
Maharashtra	**ORF1a:L2343F**	2	0.29	**ORF1a:L1249F**	3	0.38	**ORF1a:E102K**	2	0.3	**ORF1a:G3169L**	14	0.4
Maharashtra	**ORF1a:A2344S**	2	0.29	**ORF1a:A482V**	3	0.38	**ORF1a:Q607H**	2	0.3	**ORF1a:N3186K**	14	0.4
Maharashtra	**ORF1a:M2347L**	2	0.29	**ORF1a:H2125Y**	3	0.38	**ORF1a:I476T**	2	0.3	**ORF1a:T3710C**	14	0.4
Maharashtra	**ORF1a:A3958V**	2	0.29	**ORF1a:G2035R**	3	0.38	**ORF1a:P2046L**	2	0.3	**ORF1a:S1506L**	14	0.4
Maharashtra	**ORF1a:S4398L**	2	0.29	**ORF1a:L733I**	2	0.26	**ORF1a:R1464W**	2	0.3	**ORF1a:G1369M**	14	0.4
Maharashtra	**ORF1a:A4394V**	2	0.29	**ORF1a:E37D**	2	0.26	**ORF1a:G3278S**	2	0.3	**ORF1a:V1371S**	14	0.4
Maharashtra	**ORF1a:K1655R**	2	0.29	**ORF1a:K3577R**	2	0.26	**ORF1a:E767A**	2	0.3	**ORF1a:S1372K**	14	0.4
Maharashtra	**ORF1a:F2787C**	2	0.29	**ORF1a:T4364I**	2	0.26	**ORF1a:K1781R**	2	0.3	**ORF1a:W1373K**	14	0.4
Maharashtra	**ORF1a:P286S**	2	0.29	**ORF1a:V1295F**	2	0.26	**ORF1a:K1655N**	2	0.3	**ORF1a:R1376E**	14	0.4
Maharashtra	**ORF1a:S2500F**	2	0.29	**ORF1a:A427T**	2	0.26	**ORF1a:N1139S**	2	0.3	**ORF1a:M1378F**	14	0.4
Maharashtra	**ORF1a:P2055L**	2	0.29	**ORF1a:M315V**	2	0.26	**ORF1a:S2578Y**	2	0.3	**ORF1a:A1380G**	14	0.4
Maharashtra	**ORF1a:E3441D**	2	0.29	**ORF1a:T2877I**	2	0.26	**ORF1a:K680N**	2	0.3	**ORF1a:H1381I**	14	0.4
Maharashtra	**ORF1a:C3790F**	2	0.29	**ORF1a:S3344F**	2	0.26	**ORF1a:E1013D**	2	0.3	**ORF1a:A1382C**	14	0.4
Maharashtra	**ORF1a:T2300I**	2	0.29	**ORF1a:V3420L**	2	0.26	**ORF1a:F2780L**	2	0.3	**ORF1a:E1384S**	14	0.4
Maharashtra	**ORF1a:S481F**	2	0.29	**ORF1a:A4254V**	2	0.26	**ORF1a:A4257T**	2	0.3	**ORF1a:T1385V**	14	0.4
Maharashtra	**ORF1a:L1852F**	2	0.29	**ORF1a:M3221I**	2	0.26	**ORF1a:M1014I**	2	0.3	**ORF1a:R1386W**	14	0.4
Maharashtra	**ORF1a:G2091S**	2	0.29	**ORF1a:K322R**	2	0.26	**ORF1a:K3979N**	2	0.3	**ORF1a:P1449K**	14	0.4
Maharashtra	**ORF1a:H2092V**	2	0.29	**ORF1a:L1853F**	2	0.26	**ORF1a:V1058I**	2	0.3	**ORF1a:N2216***	14	0.4
Maharashtra	**ORF1a:H3580Q**	2	0.29	**ORF1a:S2517F**	2	0.26	**ORF1a:L3591I**	2	0.3	**ORF1a:P2144L**	14	0.4
Maharashtra	**ORF1a:A2192T**	2	0.29	**ORF1a:I3052V**	2	0.26	**ORF1a:M3221I**	2	0.3	**ORF1a:Y1425T**	14	0.4
Maharashtra	**ORF1a:K338R**	2	0.29	**ORF1a:L730F**	2	0.26	**ORF1a:S2015I**	2	0.3	**ORF1a:T1426P**	14	0.4
Maharashtra	**ORF1a:I453V**	2	0.29	**ORF1a:F2328L**	2	0.26	**ORF1a:T265I**	2	0.3	**ORF1a:S1427V**	14	0.4
Maharashtra	**ORF1a:D1089Y**	2	0.29	**ORF1a:T1788M**	2	0.26	**ORF1a:S3913L**	2	0.3	**ORF1a:N1442***	14	0.4
Maharashtra	**ORF1a:S2246P**	2	0.29	**ORF1a:A3209V**	2	0.26	**ORF1a:A4092V**	2	0.3	**ORF1a:E1443R**	14	0.4
Maharashtra	**ORF1a:A2249V**	2	0.29	**ORF1a:A1997V**	2	0.26	**ORF1a:P892S**	2	0.3	**ORF1a:T1444H**	14	0.4
Maharashtra	**ORF1a:L4P**	2	0.29	**ORF1a:K3499R**	2	0.26	**ORF1a:A656V**	2	0.3	**ORF1a:V1446S**	14	0.4
Maharashtra	**ORF1a:F2173L**	2	0.29	**ORF1a:P4220L**	2	0.26	**ORF1a:L3116F**	2	0.3	**ORF1a:E1549L**	14	0.4
Maharashtra	**ORF1a:V4361G**	2	0.29	**ORF1a:N1995K**	2	0.26	**ORF1a:V3968F**	2	0.3	**ORF1a:M2347L**	13	0.37
Maharashtra	**ORF1a:K1350R**	2	0.29	**ORF1a:A2587V**	2	0.26	**ORF1a:Q4100H**	2	0.3	**ORF1a:D2467V**	13	0.37
Maharashtra	**ORF1a:A76V**	2	0.29	**ORF1a:P1018L**	2	0.26	**ORF1a:A4394V**	2	0.3	**ORF1a:E2468L**	13	0.37
Maharashtra	**ORF1a:P732H**	2	0.29	**ORF1a:R1628C**	2	0.26	**ORF1a:D795N**	2	0.3	**ORF1a:A2470V**	13	0.37
Maharashtra	**ORF1a:A3454V**	2	0.29	**ORF1a:V86F**	2	0.26	**ORF1a:L3711F**	2	0.3	**ORF1a:R2471H**	13	0.37
Maharashtra	**ORF1a:I2227M**	2	0.29	**ORF1a:A385V**	2	0.26	**ORF1a:T4364I**	2	0.3	**ORF1a:D2472L**	13	0.37
Maharashtra	**ORF1a:A2279V**	2	0.29	**ORF1a:S1106N**	2	0.26	**ORF1a:A3209V**	2	0.3	**ORF1a:A2557L**	13	0.37
Maharashtra	**ORF1a:D828Y**	2	0.29	**ORF1a:M3652I**	2	0.26	**ORF1a:V3718A**	2	0.3	**ORF1a:S2558C**	13	0.37

Maharashtra	**ORF1a:A3070V**	1	0.15	**ORF1a:A307V**	2	0.26	**ORF1a:T554G**	2	0.3	**ORF1a:Q2563N**	13	0.37
Maharashtra	**ORF1a:I671T**	1	0.15	**ORF1a:K2849E**	2	0.26	**ORF1a:A555R**	2	0.3	**ORF1a:M2565Y**	13	0.37
Maharashtra	**ORF1a:P2144S**	1	0.15	**ORF1a:V1056L**	2	0.26	**ORF1a:Q556Y**	2	0.3	**ORF1a:Q2567Y**	13	0.37
Maharashtra	**ORF1a:I476V**	1	0.15	**ORF1a:T2183I**	2	0.26	**ORF1a:S558N**	2	0.3	**ORF1a:P2568***	13	0.37
Maharashtra	**ORF1a:P2079L**	1	0.15	**ORF1a:S2500F**	2	0.26	**ORF1a:V559T**	2	0.3	**ORF1a:Q2749***	13	0.37
Maharashtra	**ORF1a:I431M**	1	0.15	**ORF1a:P2055L**	2	0.26	**ORF1a:V627***	2	0.3	**ORF1a:Y3115I**	13	0.37
Maharashtra	**ORF1a:L4111F**	1	0.15	**ORF1a:D4043G**	2	0.26	**ORF1a:D629A**	2	0.3	**ORF1a:H3435L**	13	0.37
Maharashtra	**ORF1a:L3796F**	1	0.15	**ORF1a:I473S**	2	0.26	**ORF1a:W630***	2	0.3	**ORF1a:A3735L**	13	0.37
Maharashtra	**ORF1a:C4381Y**	1	0.15	**ORF1a:S2493I**	2	0.26	**ORF1a:L631R**	2	0.3	**ORF1a:D3247T**	13	0.37
Maharashtra	**ORF1a:S1133N**	1	0.15	**ORF1a:A2139V**	2	0.26	**ORF1a:E633V**	2	0.3	**ORF1a:V3248N**	13	0.37
Maharashtra	**ORF1a:E471G**	1	0.15	**ORF1a:L681F**	2	0.26	**ORF1a:K634***	2	0.3	**ORF1a:F2598N**	13	0.37
Maharashtra	**ORF1a:M2096K**	1	0.15	**ORF1a:I2138V**	2	0.26	**ORF1a:F635G**	2	0.3	**ORF1a:T2800N**	13	0.37
Maharashtra	**ORF1a:T3058I**	1	0.15	**ORF1a:W2769C**	2	0.26	**ORF1a:K636R**	2	0.3	**ORF1a:E2805R**	13	0.37
Maharashtra	**ORF1a:S1905F**	1	0.15	**ORF1a:T708I**	2	0.26	**ORF1a:E637C**	2	0.3	**ORF1a:I2807Q**	13	0.37
Maharashtra	**ORF1a:A516V**	1	0.15	**ORF1a:D2627A**	2	0.26	**ORF1a:G638R**	2	0.3	**ORF1a:G2987L**	13	0.37
Maharashtra	**ORF1a:A3379V**	1	0.15	**ORF1a:L2367***	2	0.26	**ORF1a:E640S**	2	0.3	**ORF1a:T2988***	13	0.37
Maharashtra	**ORF1a:R426C**	1	0.15	**ORF1a:M2254I**	2	0.26	**ORF1a:F641***	2	0.3	**ORF1a:C2989K**	13	0.37
Maharashtra	**ORF1a:E1766D**	1	0.15	**ORF1a:L2778F**	2	0.26	**ORF1a:L642R**	2	0.3	**ORF1a:E2990I**	13	0.37
Maharashtra	**ORF1a:S279T**	1	0.15	**ORF1a:V3475I**	2	0.26	**ORF1a:D644L**	2	0.3	**ORF1a:V3005***	13	0.37
Maharashtra	**ORF1a:G295V**	1	0.15	**ORF1a:T403I**	2	0.26	**ORF1a:A656N**	2	0.3	**ORF1a:N3007L**	13	0.37
Maharashtra	**ORF1a:V3917F**	1	0.15	**ORF1a:M2957T**	2	0.26	**ORF1a:E658***	2	0.3	**ORF1a:N3008Q**	13	0.37
Maharashtra	**ORF1a:L1683I**	1	0.15	**ORF1a:T1682I**	2	0.26	**ORF1a:V660C**	2	0.3	**ORF1a:D3009I**	13	0.37
Maharashtra	**ORF1a:S279F**	1	0.15	**ORF1a:L2146F**	2	0.26	**ORF1a:G661K**	2	0.3	**ORF1a:Y3010F**	13	0.37
Maharashtra	**ORF1a:V675A**	1	0.15	**ORF1a:A3143V**	2	0.26	**ORF1a:Q663N**	2	0.3	**ORF1a:Y3011T**	13	0.37
Maharashtra	**ORF1a:R724I**	1	0.15	**ORF1a:I1259S**	2	0.26	**ORF1a:I664***	2	0.3	**ORF1a:L3026F**	13	0.37
Maharashtra	**ORF1a:E2112A**	1	0.15	**ORF1a:T554I**	2	0.26	**ORF1a:V665G**	2	0.3	**ORF1a:N3118Y**	13	0.37
Maharashtra	**ORF1a:P3952S**	1	0.15	**ORF1a:P2144L**	2	0.26	**ORF1a:T666E**	2	0.3	**ORF1a:D3119S**	13	0.37
Maharashtra	**ORF1a:H3076Y**	1	0.15	**ORF1a:T2906I**	2	0.26	**ORF1a:L700C**	2	0.3	**ORF1a:S944L**	13	0.37
Maharashtra	**ORF1a:T4207I**	1	0.15	**ORF1a:Q1198R**	2	0.26	**ORF1a:K701H**	2	0.3	**ORF1a:G519S**	13	0.37
Maharashtra	**ORF1a:M1586N**	1	0.15	**ORF1a:P309L**	2	0.26	**ORF1a:L716I**	2	0.3	**ORF1a:P1213L**	13	0.37
Maharashtra	**ORF1a:D1603L**	1	0.15	**ORF1a:H1113Y**	2	0.26	**ORF1a:Y717V**	2	0.3	**ORF1a:T1035I**	13	0.37
Maharashtra	**ORF1a:T1605M**	1	0.15	**ORF1a:S4398L**	2	0.26	**ORF1a:R718Q**	2	0.3	**ORF1a:E1136K**	13	0.37
Maharashtra	**ORF1a:I1607V**	1	0.15	**ORF1a:P971L**	2	0.26	**ORF1a:E738R**	2	0.3	**ORF1a:N1137T**	13	0.37
Maharashtra	**ORF1a:P1609H**	1	0.15	**ORF1a:D1554Y**	2	0.26	**ORF1a:I739N**	2	0.3	**ORF1a:Q1140F**	13	0.37
Maharashtra	**ORF1a:H1610F**	1	0.15	**ORF1a:G1119C**	2	0.26	**ORF1a:I740Y**	2	0.3	**ORF1a:H1141L**	13	0.37
Maharashtra	**ORF1a:N1611M**	1	0.15	**ORF1a:S2132G**	2	0.26	**ORF1a:F741L**	2	0.3	**ORF1a:E1142R**	13	0.37
Maharashtra	**ORF1a:S1612F**	1	0.15	**ORF1a:E1311G**	2	0.26	**ORF1a:E743R**	2	0.3	**ORF1a:L1255M**	13	0.37
Maharashtra	**ORF1a:H1613Y**	1	0.15	**ORF1a:I616T**	2	0.26	**ORF1a:E745S**	2	0.3	**ORF1a:Y1256A**	13	0.37
Maharashtra	**ORF1a:E1614L**	1	0.15	**ORF1a:T999I**	2	0.26	**ORF1a:T746C**	2	0.3	**ORF1a:Y1511I**	13	0.37
Maharashtra	**ORF1a:G1615M**	1	0.15	**ORF1a:V2795I**	2	0.26	**ORF1a:S838D**	2	0.3	**ORF1a:H1652T**	13	0.37
Maharashtra	**ORF1a:K1616M**	1	0.15	**ORF1a:M902V**	2	0.26	**ORF1a:V839***	2	0.3	**ORF1a:T1653H**	13	0.37
Maharashtra	**ORF1a:F1618L**	1	0.15	**ORF1a:L1131F**	2	0.26	**ORF1a:N840***	2	0.3	**ORF1a:R1746L**	13	0.37
Maharashtra	**ORF1a:P1622R**	1	0.15	**ORF1a:V561F**	2	0.26	**ORF1a:I841S**	2	0.3	**ORF1a:V2066R**	13	0.37
Maharashtra	**ORF1a:N1623L**	1	0.15	**ORF1a:E246G**	2	0.26	**ORF1a:Q991S**	2	0.3	**ORF1a:F1867L**	13	0.37
Maharashtra	**ORF1a:D1624L**	1	0.15	**ORF1a:I652T**	2	0.26	**ORF1a:F1028C**	2	0.3	**ORF1a:S302F**	13	0.37
Maharashtra	**ORF1a:D1625S**	1	0.15	**ORF1a:S2242F**	2	0.26	**ORF1a:G1030D**	2	0.3	**ORF1a:Y2397L**	12	0.34
Maharashtra	**ORF1a:M1647Q**	1	0.15	**ORF1a:G519S**	2	0.26	**ORF1a:K1033F**	2	0.3	**ORF1a:V2750H**	12	0.34
Maharashtra	**ORF1a:S1648L**	1	0.15	**ORF1a:R207C**	2	0.26	**ORF1a:L1034K**	2	0.3	**ORF1a:V2751L**	12	0.34
Maharashtra	**ORF1a:A1649I**	1	0.15	**ORF1a:K1781E**	2	0.26	**ORF1a:D1036Y**	2	0.3	**ORF1a:N2752R**	12	0.34
Maharashtra	**ORF1a:H1652T**	1	0.15	**ORF1a:P4337S**	2	0.26	**ORF1a:N1037***	2	0.3	**ORF1a:Q2772L**	12	0.34
Maharashtra	**ORF1a:T1653H**	1	0.15	**ORF1a:T4087I**	2	0.26	**ORF1a:V1038Q**	2	0.3	**ORF1a:P2899***	12	0.34
Maharashtra	**ORF1a:K1655L**	1	0.15	**ORF1a:G3072C**	2	0.26	**ORF1a:Y1039C**	2	0.3	**ORF1a:T2977G**	12	0.34
Maharashtra	**ORF1a:W1656M**	1	0.15	**ORF1a:S443P**	2	0.26	**ORF1a:H1067***	2	0.3	**ORF1a:G2995L**	12	0.34
Maharashtra	**ORF1a:K1657V**	1	0.15	**ORF1a:V1994I**	2	0.26	**ORF1a:L1131***	2	0.3	**ORF1a:H3327W**	12	0.34
Maharashtra	**ORF1a:Y1658***	1	0.15	**ORF1a:L3919F**	2	0.26	**ORF1a:L1148F**	2	0.3	**ORF1a:N3328Y**	12	0.34
Maharashtra	**ORF1a:P1659L**	1	0.15	**ORF1a:D691N**	2	0.26	**ORF1a:L1149***	2	0.3	**ORF1a:F3329R**	12	0.34
Maharashtra	**ORF1a:Q1660L**	1	0.15	**ORF1a:S838I**	2	0.26	**ORF1a:G1152R**	2	0.3	**ORF1a:Q3346F**	12	0.34
Maharashtra	**ORF1a:V1661L**	1	0.15	**ORF1a:T319I**	2	0.26	**ORF1a:I1153S**	2	0.3	**ORF1a:C3348S**	12	0.34
Maharashtra	**ORF1a:Y1675Q**	1	0.15	**ORF1a:S2048F**	2	0.26	**ORF1a:F1154S**	2	0.3	**ORF1a:V3349G**	12	0.34
Maharashtra	**ORF1a:L1676I**	1	0.15	**ORF1a:P276S**	2	0.26	**ORF1a:G1155T**	2	0.3	**ORF1a:G3437Q**	12	0.34
Maharashtra	**ORF1a:A1677T**	1	0.15	**ORF1a:E2042A**	2	0.26	**ORF1a:A1156C**	2	0.3	**ORF1a:W3481L**	12	0.34
Maharashtra	**ORF1a:L1680I**	1	0.15	**ORF1a:P1265S**	2	0.26	**ORF1a:D1157T**	2	0.3	**ORF1a:F3482L**	12	0.34
Maharashtra	**ORF1a:Q1684S**	1	0.15	**ORF1a:T4164I**	2	0.26	**ORF1a:P1158I**	2	0.3	**ORF1a:A3571R**	12	0.34
Maharashtra	**ORF1a:Q1685L**	1	0.15	**ORF1a:T1022I**	2	0.26	**ORF1a:H1160I**	2	0.3	**ORF1a:T3665I**	12	0.34
Maharashtra	**ORF1a:E1687H**	1	0.15	**ORF1a:E1200G**	2	0.26	**ORF1a:L1162C**	2	0.3	**ORF1a:K3213G**	12	0.34
Maharashtra	**ORF1a:Y1699L**	1	0.15	**ORF1a:G989V**	1	0.13	**ORF1a:V1164Y**	2	0.3	**ORF1a:C3232N**	12	0.34
Maharashtra	**ORF1a:R1701K**	1	0.15	**ORF1a:T1760K**	1	0.13	**ORF1a:S1905L**	2	0.3	**ORF1a:N1651S**	12	0.34
Maharashtra	**ORF1a:A1702M**	1	0.15	**ORF1a:M2719I**	1	0.13	**ORF1a:S2237N**	2	0.3	**ORF1a:L1797D**	12	0.34
Maharashtra	**ORF1a:R1703L**	1	0.15	**ORF1a:R550H**	1	0.13	**ORF1a:G2269L**	2	0.3	**ORF1a:Y1827***	12	0.34
Maharashtra	**ORF1a:A1704I**	1	0.15	**ORF1a:D1481G**	1	0.13	**ORF1a:N2275***	2	0.3	**ORF1a:G1829Y**	12	0.34
Maharashtra	**ORF1a:G1705T**	1	0.15	**ORF1a:T1840I**	1	0.13	**ORF1a:V2276C**	2	0.3	**ORF1a:M2347F**	12	0.34
Maharashtra	**ORF1a:K1720P**	1	0.15	**ORF1a:L3915F**	1	0.13	**ORF1a:Q3455K**	2	0.3	**ORF1a:F2351C**	12	0.34
Maharashtra	**ORF1a:G1723L**	1	0.15	**ORF1a:G1095R**	1	0.13	**ORF1a:Y2266N**	2	0.3	**ORF1a:Y2353T**	12	0.34

Maharashtra	**ORF1a:L1725K**	1	0.15	**ORF1a:T4164A**	1	0.13	**ORF1a:G3541E**	2	0.3	**ORF1a:A2508D**	12	0.34
Maharashtra	**ORF1a:G1726Q**	1	0.15	**ORF1a:K564N**	1	0.13	**ORF1a:T1035I**	2	0.3	**ORF1a:Q2510***	12	0.34
Maharashtra	**ORF1a:D1727***	1	0.15	**ORF1a:T1626I**	1	0.13	**ORF1a:R1628C**	2	0.3	**ORF1a:H2516F**	12	0.34
Maharashtra	**ORF1a:S1743T**	1	0.15	**ORF1a:K2771E**	1	0.13	**ORF1a:E2607K**	2	0.3	**ORF1a:N2628C**	12	0.34
Maharashtra	**ORF1a:R1746L**	1	0.15	**ORF1a:P3952S**	1	0.13	**ORF1a:K722***	2	0.3	**ORF1a:I2634S**	12	0.34
Maharashtra	**ORF1a:L1748D**	1	0.15	**ORF1a:R287S**	1	0.13	**ORF1a:H1580R**	2	0.3	**ORF1a:A2636S**	12	0.34
Maharashtra	**ORF1a:V1768H**	1	0.15	**ORF1a:V3097A**	1	0.13	**ORF1a:V757F**	2	0.3	**ORF1a:Q2702G**	12	0.34
Maharashtra	**ORF1a:M1769F**	1	0.15	**ORF1a:S1612L**	1	0.13	**ORF1a:P2792R**	2	0.3	**ORF1a:V2703S**	12	0.34
Maharashtra	**ORF1a:Y1770L**	1	0.15	**ORF1a:G2241D**	1	0.13	**ORF1a:V2892F**	2	0.3	**ORF1a:A2704K**	12	0.34
Maharashtra	**ORF1a:G1772N**	1	0.15	**ORF1a:S2242***	1	0.13	**ORF1a:L477W**	2	0.3	**ORF1a:D2824Q**	12	0.34
Maharashtra	**ORF1a:T1773N**	1	0.15	**ORF1a:L2243R**	1	0.13	**ORF1a:V5I**	2	0.3	**ORF1a:H2883A**	12	0.34
Maharashtra	**ORF1a:K1791R**	1	0.15	**ORF1a:I2244T**	1	0.13	**ORF1a:P6F**	2	0.3	**ORF1a:K2923W**	12	0.34
Maharashtra	**ORF1a:Q1792K**	1	0.15	**ORF1a:Y2245***	1	0.13	**ORF1a:G7C**	2	0.3	**ORF1a:D2924***	12	0.34
Maharashtra	**ORF1a:A1793V**	1	0.15	**ORF1a:S2246A**	1	0.13	**ORF1a:T829I**	2	0.3	**ORF1a:A3045T**	12	0.34
Maharashtra	**ORF1a:T1794F**	1	0.15	**ORF1a:S2261I**	1	0.13	**ORF1a:T1496I**	2	0.3	**ORF1a:S3046Y**	12	0.34
Maharashtra	**ORF1a:K1795R**	1	0.15	**ORF1a:Y2262N**	1	0.13	**ORF1a:V721I**	2	0.3	**ORF1a:I3047W**	12	0.34
Maharashtra	**ORF1a:G1819S**	1	0.15	**ORF1a:C2263S**	1	0.13	**ORF1a:R550H**	2	0.3	**ORF1a:V3048C**	12	0.34
Maharashtra	**ORF1a:F1867L**	1	0.15	**ORF1a:T2264K**	1	0.13	**ORF1a:N522H**	2	0.3	**ORF1a:A3049F**	12	0.34
Maharashtra	**ORF1a:Y1868L**	1	0.15	**ORF1a:G2265N**	1	0.13	**ORF1a:L27F**	2	0.3	**ORF1a:G3051H**	12	0.34
Maharashtra	**ORF1a:K1869R**	1	0.15	**ORF1a:Y2266H**	1	0.13	**ORF1a:G744E**	2	0.3	**ORF1a:V3056N**	12	0.34
Maharashtra	**ORF1a:E1870M**	1	0.15	**ORF1a:P2965L**	1	0.13	**ORF1a:V3130A**	2	0.3	**ORF1a:V3057M**	12	0.34
Maharashtra	**ORF1a:N1871F**	1	0.15	**ORF1a:F3628K**	1	0.13	**ORF1a:S1857L**	2	0.3	**ORF1a:T3058P**	12	0.34
Maharashtra	**ORF1a:D1903T**	1	0.15	**ORF1a:V3629I**	1	0.13	**ORF1a:G445S**	2	0.3	**ORF1a:L3191V**	12	0.34
Maharashtra	**ORF1a:N1904L**	1	0.15	**ORF1a:Y4080C**	1	0.13	**ORF1a:T2154I**	2	0.3	**ORF1a:L3193***	12	0.34
Maharashtra	**ORF1a:F1907Q**	1	0.15	**ORF1a:L3027I**	1	0.13	**ORF1a:T3258I**	2	0.3	**ORF1a:L3262H**	12	0.34
Maharashtra	**ORF1a:F1937I**	1	0.15	**ORF1a:V1747I**	1	0.13	**ORF1a:F548D**	2	0.3	**ORF1a:Q3263L**	12	0.34
Maharashtra	**ORF1a:A1938I**	1	0.15	**ORF1a:K4226R**	1	0.13	**ORF1a:S549Q**	2	0.3	**ORF1a:A3454***	12	0.34
Maharashtra	**ORF1a:D1939L**	1	0.15	**ORF1a:D589G**	1	0.13	**ORF1a:T551L**	2	0.3	**ORF1a:D3460S**	12	0.34
Maharashtra	**ORF1a:D1940S**	1	0.15	**ORF1a:Q611H**	1	0.13	**ORF1a:S713L**	2	0.3	**ORF1a:V3718F**	12	0.34
Maharashtra	**ORF1a:Q1943L**	1	0.15	**ORF1a:F651V**	1	0.13	**ORF1a:V3593F**	2	0.3	**ORF1a:S485I**	12	0.34
Maharashtra	**ORF1a:T1945Q**	1	0.15	**ORF1a:R1318I**	1	0.13	**ORF1a:S984I**	2	0.3	**ORF1a:P4223L**	12	0.34
Maharashtra	**ORF1a:G1946E**	1	0.15	**ORF1a:L2518F**	1	0.13	**ORF1a:N840D**	2	0.3	**ORF1a:L1034***	12	0.34
Maharashtra	**ORF1a:Y1947S**	1	0.15	**ORF1a:L4191S**	1	0.13	**ORF1a:G1155C**	2	0.3	**ORF1a:D1036V**	12	0.34
Maharashtra	**ORF1a:K1948L**	1	0.15	**ORF1a:A2832S**	1	0.13	**ORF1a:H3509R**	2	0.3	**ORF1a:N1037L**	12	0.34
Maharashtra	**ORF1a:K1981T**	1	0.15	**ORF1a:A3889V**	1	0.13	**ORF1a:T1881K**	2	0.3	**ORF1a:Y1039V**	12	0.34
Maharashtra	**ORF1a:G1982T**	1	0.15	**ORF1a:S212L**	1	0.13	**ORF1a:L1914S**	2	0.3	**ORF1a:K1041***	12	0.34
Maharashtra	**ORF1a:A1983H**	1	0.15	**ORF1a:T1277I**	1	0.13	**ORF1a:Q2348***	2	0.3	**ORF1a:V1052L**	12	0.34
Maharashtra	**ORF1a:K1984P**	1	0.15	**ORF1a:S2911L**	1	0.13	**ORF1a:N2256I**	2	0.3	**ORF1a:A1111S**	12	0.34
Maharashtra	**ORF1a:H1987R**	1	0.15	**ORF1a:H2986Y**	1	0.13	**ORF1a:V874F**	2	0.3	**ORF1a:K1112A**	12	0.34
Maharashtra	**ORF1a:P1989L**	1	0.15	**ORF1a:P3613H**	1	0.13	**ORF1a:A1074V**	2	0.3	**ORF1a:H1113Q**	12	0.34
Maharashtra	**ORF1a:N2006R**	1	0.15	**ORF1a:P1147S**	1	0.13	**ORF1a:S2132G**	2	0.3	**ORF1a:C1114M**	12	0.34
Maharashtra	**ORF1a:T2007I**	1	0.15	**ORF1a:T4371A**	1	0.13	**ORF1a:A478H**	2	0.3	**ORF1a:H1116T**	12	0.34
Maharashtra	**ORF1a:W2008N**	1	0.15	**ORF1a:A4277V**	1	0.13	**ORF1a:M789I**	2	0.3	**ORF1a:V1117K**	12	0.34
Maharashtra	**ORF1a:C2009Q**	1	0.15	**ORF1a:T1000I**	1	0.13	**ORF1a:L450I**	2	0.3	**ORF1a:S1188K**	12	0.34
Maharashtra	**ORF1a:L2062I**	1	0.15	**ORF1a:I2227V**	1	0.13	**ORF1a:I648T**	2	0.3	**ORF1a:F1190K**	12	0.34
Maharashtra	**ORF1a:E2063L**	1	0.15	**ORF1a:P1596L**	1	0.13	**ORF1a:S2242F**	2	0.3	**ORF1a:E1192N**	12	0.34
Maharashtra	**ORF1a:C2064P**	1	0.15	**ORF1a:E113G**	1	0.13	**ORF1a:T1538I**	2	0.3	**ORF1a:M1193K**	12	0.34
Maharashtra	**ORF1a:G2091M**	1	0.15	**ORF1a:P626L**	1	0.13	**ORF1a:L552I**	2	0.3	**ORF1a:K1194R**	12	0.34
Maharashtra	**ORF1a:H2092***	1	0.15	**ORF1a:T2124I**	1	0.13	**ORF1a:A3143V**	2	0.3	**ORF1a:D1258F**	12	0.34
Maharashtra	**ORF1a:D2094I**	1	0.15	**ORF1a:D1513G**	1	0.13	**ORF1a:S2312L**	2	0.3	**ORF1a:G1261Q**	12	0.34
Maharashtra	**ORF1a:N2111V**	1	0.15	**ORF1a:L4064I**	1	0.13	**ORF1a:T614I**	2	0.3	**ORF1a:N1262I**	12	0.34
Maharashtra	**ORF1a:S2114L**	1	0.15	**ORF1a:T1444I**	1	0.13	**ORF1a:A427V**	2	0.3	**ORF1a:H1264P**	12	0.34
Maharashtra	**ORF1a:V2116N**	1	0.15	**ORF1a:A4094V**	1	0.13	**ORF1a:A3987V**	2	0.3	**ORF1a:P1265L**	12	0.34
Maharashtra	**ORF1a:G2118***	1	0.15	**ORF1a:T1637I**	1	0.13	**ORF1a:E289D**	2	0.3	**ORF1a:D1266L**	12	0.34
Maharashtra	**ORF1a:A2128L**	1	0.15	**ORF1a:H3509Y**	1	0.13	**ORF1a:H388Y**	2	0.3	**ORF1a:S1267L**	12	0.34
Maharashtra	**ORF1a:A2129I**	1	0.15	**ORF1a:A1679V**	1	0.13	**ORF1a:G2265V**	2	0.3	**ORF1a:A1268V**	12	0.34
Maharashtra	**ORF1a:V2150Q**	1	0.15	**ORF1a:T2988I**	1	0.13	**ORF1a:M3087I**	2	0.3	**ORF1a:V1271T**	12	0.34
Maharashtra	**ORF1a:S2151L**	1	0.15	**ORF1a:K4176M**	1	0.13	**ORF1a:T1542I**	2	0.3	**ORF1a:F1278M**	12	0.34
Maharashtra	**ORF1a:T2152L**	1	0.15	**ORF1a:A537S**	1	0.13	**ORF1a:M297I**	2	0.3	**ORF1a:D1603L**	12	0.34
Maharashtra	**ORF1a:C2160***	1	0.15	**ORF1a:L2869F**	1	0.13	**ORF1a:P959L**	2	0.3	**ORF1a:N1611M**	12	0.34
Maharashtra	**ORF1a:N2162H**	1	0.15	**ORF1a:A4357V**	1	0.13	**ORF1a:Q1021H**	2	0.3	**ORF1a:S1612F**	12	0.34
Maharashtra	**ORF1a:R2163G**	1	0.15	**ORF1a:S245P**	1	0.13	**ORF1a:P1921L**	2	0.3	**ORF1a:E1614L**	12	0.34
Maharashtra	**ORF1a:C2165***	1	0.15	**ORF1a:A3705V**	1	0.13	**ORF1a:S2797F**	2	0.3	**ORF1a:G1615M**	12	0.34
Maharashtra	**ORF1a:N2167L**	1	0.15	**ORF1a:L4386F**	1	0.13	**ORF1a:I601V**	2	0.3	**ORF1a:K1616M**	12	0.34
Maharashtra	**ORF1a:Y2168L**	1	0.15	**ORF1a:T1328N**	1	0.13	**ORF1a:A3497V**	2	0.3	**ORF1a:F1618L**	12	0.34
Maharashtra	**ORF1a:M2169Y**	1	0.15	**ORF1a:D2101Y**	1	0.13	**ORF1a:H3580Y**	2	0.3	**ORF1a:K1655L**	12	0.34
Maharashtra	**ORF1a:P2170C**	1	0.15	**ORF1a:P271S**	1	0.13	**ORF1a:L705F**	2	0.3	**ORF1a:W1656M**	12	0.34
Maharashtra	**ORF1a:F2172N**	1	0.15	**ORF1a:G345D**	1	0.13	**ORF1a:F2350V**	2	0.3	**ORF1a:K1657V**	12	0.34
Maharashtra	**ORF1a:F2173C**	1	0.15	**ORF1a:V1222F**	1	0.13	**ORF1a:L2367K**	2	0.3	**ORF1a:Y1658***	12	0.34
Maharashtra	**ORF1a:Y2270C**	1	0.15	**ORF1a:P197S**	1	0.13	**ORF1a:P2739L**	2	0.3	**ORF1a:P1659L**	12	0.34
Maharashtra	**ORF1a:N2272L**	1	0.15	**ORF1a:G379E**	1	0.13	**ORF1a:P2287S**	2	0.3	**ORF1a:Q1660L**	12	0.34
Maharashtra	**ORF1a:S2273T**	1	0.15	**ORF1a:T1558I**	1	0.13	**ORF1a:A1314V**	2	0.3	**ORF1a:V1661L**	12	0.34
Maharashtra	**ORF1a:T2274V**	1	0.15	**ORF1a:M3655I**	1	0.13	**ORF1a:K798N**	2	0.3	**ORF1a:Y1675Q**	12	0.34

Maharashtra	**ORF1a:N2275L**	1	0.15	**ORF1a:S2255F**	1	0.13	**ORF1a:A2279K**	2	0.3	**ORF1a:L1676I**	12	0.34
Maharashtra	**ORF1a:S2293I**	1	0.15	**ORF1a:V28I**	1	0.13	**ORF1a:Y2353K**	2	0.3	**ORF1a:A1677T**	12	0.34
Maharashtra	**ORF1a:G2294L**	1	0.15	**ORF1a:P2605S**	1	0.13	**ORF1a:F2354K**	2	0.3	**ORF1a:L1680I**	12	0.34
Maharashtra	**ORF1a:D2296***	1	0.15	**ORF1a:M85I**	1	0.13	**ORF1a:V721L**	2	0.3	**ORF1a:T1682P**	12	0.34
Maharashtra	**ORF1a:D2299Y**	1	0.15	**ORF1a:A2614V**	1	0.13	**ORF1a:G706K**	2	0.3	**ORF1a:Q1684S**	12	0.34
Maharashtra	**ORF1a:T2300K**	1	0.15	**ORF1a:V306F**	1	0.13	**ORF1a:A405V**	2	0.3	**ORF1a:Q1685L**	12	0.34
Maharashtra	**ORF1a:Y2301L**	1	0.15	**ORF1a:L1270F**	1	0.13	**ORF1a:K2761N**	2	0.3	**ORF1a:E1687H**	12	0.34
Maharashtra	**ORF1a:L2364Y**	1	0.15	**ORF1a:S1733I**	1	0.13	**ORF1a:V3420L**	2	0.3	**ORF1a:Y1699L**	12	0.34
Maharashtra	**ORF1a:M2365I**	1	0.15	**ORF1a:M3312I**	1	0.13	**ORF1a:A2249T**	2	0.3	**ORF1a:R1701K**	12	0.34
Maharashtra	**ORF1a:W2366L**	1	0.15	**ORF1a:T1754I**	1	0.13	**ORF1a:V1550F**	2	0.3	**ORF1a:A1702M**	12	0.34
Maharashtra	**ORF1a:I2368V**	1	0.15	**ORF1a:A3497V**	1	0.13	**ORF1a:P197S**	1	0.15	**ORF1a:R1703L**	12	0.34
Maharashtra	**ORF1a:N2370Q**	1	0.15	**ORF1a:G442C**	1	0.13	**ORF1a:A1397T**	1	0.15	**ORF1a:A1704I**	12	0.34
Maharashtra	**ORF1a:V2381W**	1	0.15	**ORF1a:A656V**	1	0.13	**ORF1a:S2926F**	1	0.15	**ORF1a:G1705T**	12	0.34
Maharashtra	**ORF1a:R2382L**	1	0.15	**ORF1a:A1670T**	1	0.13	**ORF1a:P3096S**	1	0.15	**ORF1a:L1748D**	12	0.34
Maharashtra	**ORF1a:M2383E**	1	0.15	**ORF1a:T2300I**	1	0.13	**ORF1a:G1073R**	1	0.15	**ORF1a:P1989***	12	0.34
Maharashtra	**ORF1a:Y2384C**	1	0.15	**ORF1a:P926L**	1	0.13	**ORF1a:K1219E**	1	0.15	**ORF1a:W1992S**	12	0.34
Maharashtra	**ORF1a:C2452M**	1	0.15	**ORF1a:K1720N**	1	0.13	**ORF1a:N2023H**	1	0.15	**ORF1a:L2874C**	12	0.34
Maharashtra	**ORF1a:V2453W**	1	0.15	**ORF1a:M3934I**	1	0.13	**ORF1a:K2533R**	1	0.15	**ORF1a:T2877H**	12	0.34
Maharashtra	**ORF1a:N2454Y**	1	0.15	**ORF1a:G353C**	1	0.13	**ORF1a:I740V**	1	0.15	**ORF1a:G2879***	12	0.34
Maharashtra	**ORF1a:T4065I**	1	0.15	**ORF1a:M1586I**	1	0.13	**ORF1a:P2144L**	1	0.15	**ORF1a:D2880W**	12	0.34
Maharashtra	**ORF1a:L730F**	1	0.15	**ORF1a:A3209T**	1	0.13	**ORF1a:M3661I**	1	0.15	**ORF1a:F2881***	12	0.34
Maharashtra	**ORF1a:P3362S**	1	0.15	**ORF1a:D466G**	1	0.13	**ORF1a:L360S**	1	0.15	**ORF1a:G1819S**	12	0.34
Maharashtra	**ORF1a:A3987V**	1	0.15	**ORF1a:L642F**	1	0.13	**ORF1a:S2273F**	1	0.15	**ORF1a:N2147T**	12	0.34
Maharashtra	**ORF1a:K4069R**	1	0.15	**ORF1a:A4394V**	1	0.13	**ORF1a:T4083M**	1	0.15	**ORF1a:T2174V**	12	0.34
Maharashtra	**ORF1a:T749A**	1	0.15	**ORF1a:G706S**	1	0.13	**ORF1a:L730F**	1	0.15	**ORF1a:A2344S**	12	0.34
Maharashtra	**ORF1a:I601V**	1	0.15	**ORF1a:A3610V**	1	0.13	**ORF1a:A2891V**	1	0.15	**ORF1a:S2487T**	12	0.34
Maharashtra	**ORF1a:D1127N**	1	0.15	**ORF1a:A1473N**	1	0.13	**ORF1a:I2501T**	1	0.15	**ORF1a:Y2489L**	12	0.34
Maharashtra	**ORF1a:N615S**	1	0.15	**ORF1a:Y2245H**	1	0.13	**ORF1a:M4241I**	1	0.15	**ORF1a:M3539V**	12	0.34
Maharashtra	**ORF1a:S2552P**	1	0.15	**ORF1a:T3058I**	1	0.13	**ORF1a:P4389L**	1	0.15	**ORF1a:K1655N**	12	0.34
Maharashtra	**ORF1a:I2307T**	1	0.15	**ORF1a:P959S**	1	0.13	**ORF1a:A967V**	1	0.15	**ORF1a:L494F**	12	0.34
Maharashtra	**ORF1a:D3728N**	1	0.15	**ORF1a:R1170H**	1	0.13	**ORF1a:G445C**	1	0.15	**ORF1a:D1933G**	12	0.34
Maharashtra	**ORF1a:A3143V**	1	0.15	**ORF1a:H2799Y**	1	0.13	**ORF1a:T708K**	1	0.15	**ORF1a:H2799S**	11	0.31
Maharashtra	**ORF1a:L3930F**	1	0.15	**ORF1a:N363H**	1	0.13	**ORF1a:H1113Y**	1	0.15	**ORF1a:T2800***	11	0.31
Maharashtra	**ORF1a:G989S**	1	0.15	**ORF1a:A486V**	1	0.13	**ORF1a:G3051S**	1	0.15	**ORF1a:N2847H**	11	0.31
Maharashtra	**ORF1a:I3693T**	1	0.15	**ORF1a:P769H**	1	0.13	**ORF1a:V2766F**	1	0.15	**ORF1a:D2848G**	11	0.31
Maharashtra	**ORF1a:T4164I**	1	0.15	**ORF1a:I1502F**	1	0.13	**ORF1a:A3235S**	1	0.15	**ORF1a:K2849L**	11	0.31
Maharashtra	**ORF1a:L2620I**	1	0.15	**ORF1a:T1760I**	1	0.13	**ORF1a:Y3685H**	1	0.15	**ORF1a:R2974K**	11	0.31
Maharashtra	**ORF1a:A385V**	1	0.15	**ORF1a:K2004R**	1	0.13	**ORF1a:A3705V**	1	0.15	**ORF1a:F3241I**	11	0.31
Maharashtra	**ORF1a:V2276G**	1	0.15	**ORF1a:A4262V**	1	0.13	**ORF1a:K701E**	1	0.15	**ORF1a:N3243T**	11	0.31
Maharashtra	**ORF1a:D2467G**	1	0.15	**ORF1a:S1587L**	1	0.13	**ORF1a:A702S**	1	0.15	**ORF1a:G3245V**	11	0.31
Maharashtra	**ORF1a:L3594G**	1	0.15	**ORF1a:D147G**	1	0.13	**ORF1a:D75G**	1	0.15	**ORF1a:S3246T**	11	0.31
Maharashtra	**ORF1a:Q4100H**	1	0.15	**ORF1a:G150A**	1	0.13	**ORF1a:A76T**	1	0.15	**ORF1a:D3247Q**	11	0.31
Maharashtra	**ORF1a:D1585E**	1	0.15	**ORF1a:S318R**	1	0.13	**ORF1a:R77P**	1	0.15	**ORF1a:A3270L**	11	0.31
Maharashtra	**ORF1a:C2694R**	1	0.15	**ORF1a:T319H**	1	0.13	**ORF1a:T78L**	1	0.15	**ORF1a:L3320T**	11	0.31
Maharashtra	**ORF1a:A1716S**	1	0.15	**ORF1a:M807R**	1	0.13	**ORF1a:Y241H**	1	0.15	**ORF1a:R3323M**	11	0.31
Maharashtra	**ORF1a:T4158I**	1	0.15	**ORF1a:G1069K**	1	0.13	**ORF1a:E452G**	1	0.15	**ORF1a:S3325I**	11	0.31
Maharashtra	**ORF1a:S2562T**	1	0.15	**ORF1a:G1070K**	1	0.13	**ORF1a:T551R**	1	0.15	**ORF1a:N3326S**	11	0.31
Maharashtra	**ORF1a:A4285V**	1	0.15	**ORF1a:V1071M**	1	0.13	**ORF1a:L552V**	1	0.15	**ORF1a:I3476***	11	0.31
Maharashtra	**ORF1a:F871L**	1	0.15	**ORF1a:T1678S**	1	0.13	**ORF1a:E553Y**	1	0.15	**ORF1a:N3477L**	11	0.31
Maharashtra	**ORF1a:A872D**	1	0.15	**ORF1a:A1679G**	1	0.13	**ORF1a:T554S**	1	0.15	**ORF1a:D3479C**	11	0.31
Maharashtra	**ORF1a:A968V**	1	0.15	**ORF1a:A1951S**	1	0.13	**ORF1a:A555E**	1	0.15	**ORF1a:R3480T**	11	0.31
Maharashtra	**ORF1a:K2078Q**	1	0.15	**ORF1a:F2641A**	1	0.13	**ORF1a:M1771R**	1	0.15	**ORF1a:G3521***	11	0.31
Maharashtra	**ORF1a:Y2331H**	1	0.15	**ORF1a:M3393I**	1	0.13	**ORF1a:Y2266S**	1	0.15	**ORF1a:A3523C**	11	0.31
Maharashtra	**ORF1a:G334S**	1	0.15	**ORF1a:V3572M**	1	0.13	**ORF1a:R2267I**	1	0.15	**ORF1a:M3664W**	11	0.31
Maharashtra	**ORF1a:E863Q**	1	0.15	**ORF1a:K4176N**	1	0.13	**ORF1a:S2273P**	1	0.15	**ORF1a:D3668I**	11	0.31
Maharashtra	**ORF1a:T1269N**	1	0.15	**ORF1a:L4185***	1	0.13	**ORF1a:T2274C**	1	0.15	**ORF1a:M3669L**	11	0.31
Maharashtra	**ORF1a:N1576T**	1	0.15	**ORF1a:S531R**	1	0.13	**ORF1a:N2275S**	1	0.15	**ORF1a:D3671C**	11	0.31
Maharashtra	**ORF1a:I1577L**	1	0.15	**ORF1a:K1280N**	1	0.13	**ORF1a:C3563N**	1	0.15	**ORF1a:T3672L**	11	0.31
Maharashtra	**ORF1a:H1818Y**	1	0.15	**ORF1a:A1712V**	1	0.13	**ORF1a:S3564T**	1	0.15	**ORF1a:S3673V**	11	0.31
Maharashtra	**ORF1a:T2463S**	1	0.15	**ORF1a:A1397V**	1	0.13	**ORF1a:G3565V**	1	0.15	**ORF1a:S3687***	11	0.31
Maharashtra	**ORF1a:V3467A**	1	0.15	**ORF1a:L3352F**	1	0.13	**ORF1a:V3566C**	1	0.15	**ORF1a:A3688K**	11	0.31
Maharashtra	**ORF1a:N3477I**	1	0.15	**ORF1a:V86I**	1	0.13	**ORF1a:F3568V**	1	0.15	**ORF1a:V3689T**	11	0.31
Maharashtra	**ORF1a:Y3701C**	1	0.15	**ORF1a:Y2099C**	1	0.13	**ORF1a:Q3569C**	1	0.15	**ORF1a:T1794F**	11	0.31
Maharashtra	**ORF1a:L3800H**	1	0.15	**ORF1a:A3969V**	1	0.13	**ORF1a:S3570G**	1	0.15	**ORF1a:E1826L**	11	0.31
Maharashtra	**ORF1a:N3801Y**	1	0.15	**ORF1a:A3615V**	1	0.13	**ORF1a:A3571M**	1	0.15	**ORF1a:N2596R**	11	0.31
Maharashtra	**ORF1a:A4067E**	1	0.15	**ORF1a:D978H**	1	0.13	**ORF1a:V3572W**	1	0.15	**ORF1a:S2599G**	11	0.31
Maharashtra	**ORF1a:I1577T**	1	0.15	**ORF1a:V2421A**	1	0.13	**ORF1a:T12H**	1	0.15	**ORF1a:S2600K**	11	0.31
Maharashtra	**ORF1a:A1432V**	1	0.15	**ORF1a:E633D**	1	0.13	**ORF1a:H13T**	1	0.15	**ORF1a:F2602Q**	11	0.31
Maharashtra	**ORF1a:S1188L**	1	0.15	**ORF1a:D953Y**	1	0.13	**ORF1a:I1206L**	1	0.15	**ORF1a:A2616K**	11	0.31
Maharashtra	**ORF1a:A2554V**	1	0.15	**ORF1a:G645S**	1	0.13	**ORF1a:P1207L**	1	0.15	**ORF1a:S2631F**	11	0.31
Maharashtra	**ORF1a:L2778F**	1	0.15	**ORF1a:A478T**	1	0.13	**ORF1a:T1626I**	1	0.15	**ORF1a:T2632Y**	11	0.31
Maharashtra	**ORF1a:T1844N**	1	0.15	**ORF1a:P3395L**	1	0.13	**ORF1a:T2197I**	1	0.15	**ORF1a:G2689C**	11	0.31
Maharashtra	**ORF1a:R3574G**	1	0.15	**ORF1a:T1605I**	1	0.13	**ORF1a:K3981***	1	0.15	**ORF1a:S2803C**	11	0.31

Maharashtra	**ORF1a:M315V**	1	0.15	**ORF1a:A4314S**	1	0.13	**ORF1a:G524W**	1	0.15	**ORF1a:P2886T**	11	0.31
Maharashtra	**ORF1a:M1586I**	1	0.15	**ORF1a:R226M**	1	0.13	**ORF1a:K2761L**	1	0.15	**ORF1a:R2887***	11	0.31
Maharashtra	**ORF1a:A4016V**	1	0.15	**ORF1a:V365F**	1	0.13	**ORF1a:Y1337N**	1	0.15	**ORF1a:V2888S**	11	0.31
Maharashtra	**ORF1a:H2520Y**	1	0.15	**ORF1a:K4021R**	1	0.13	**ORF1a:E2990K**	1	0.15	**ORF1a:V3053Y**	11	0.31
Maharashtra	**ORF1a:T1854I**	1	0.15	**ORF1a:T12I**	1	0.13	**ORF1a:R2991D**	1	0.15	**ORF1a:A3054R**	11	0.31
Maharashtra	**ORF1a:A239E**	1	0.15	**ORF1a:L1853I**	1	0.13	**ORF1a:G3433K**	1	0.15	**ORF1a:I3055S**	11	0.31
Maharashtra	**ORF1a:W240R**	1	0.15	**ORF1a:T1854K**	1	0.13	**ORF1a:V3434I**	1	0.15	**ORF1a:Q3452F**	11	0.31
Maharashtra	**ORF1a:V2290I**	1	0.15	**ORF1a:K1855P**	1	0.13	**ORF1a:H3435K**	1	0.15	**ORF1a:T3453C**	11	0.31
Maharashtra	**ORF1a:V3136E**	1	0.15	**ORF1a:S1856G**	1	0.13	**ORF1a:A3436T**	1	0.15	**ORF1a:A3457N**	11	0.31
Maharashtra	**ORF1a:P3656L**	1	0.15	**ORF1a:S1857T**	1	0.13	**ORF1a:G3437T**	1	0.15	**ORF1a:G3458S**	11	0.31
Maharashtra	**ORF1a:A3657G**	1	0.15	**ORF1a:E1858C**	1	0.13	**ORF1a:T3438K**	1	0.15	**ORF1a:T3461S**	11	0.31
Maharashtra	**ORF1a:W3659L**	1	0.15	**ORF1a:Y1859P**	1	0.13	**ORF1a:D3439N**	1	0.15	**ORF1a:T3462W**	11	0.31
Maharashtra	**ORF1a:R207C**	1	0.15	**ORF1a:P971S**	1	0.13	**ORF1a:W3709C**	1	0.15	**ORF1a:I3463Y**	11	0.31
Maharashtra	**ORF1a:M2796I**	1	0.15	**ORF1a:T2274I**	1	0.13	**ORF1a:T3710G**	1	0.15	**ORF1a:T3464G**	11	0.31
Maharashtra	**ORF1a:W3128***	1	0.15	**ORF1a:K2622N**	1	0.13	**ORF1a:L122F**	1	0.15	**ORF1a:V3465H**	11	0.31
Maharashtra	**ORF1a:M3131V**	1	0.15	**ORF1a:I2490T**	1	0.13	**ORF1a:P1207T**	1	0.15	**ORF1a:D3492S**	11	0.31
Maharashtra	**ORF1a:G1336D**	1	0.15	**ORF1a:L3829F**	1	0.13	**ORF1a:L1388I**	1	0.15	**ORF1a:F3493***	11	0.31
Maharashtra	**ORF1a:P1541A**	1	0.15	**ORF1a:E743G**	1	0.13	**ORF1a:G295C**	1	0.15	**ORF1a:N3494***	11	0.31
Maharashtra	**ORF1a:R1729T**	1	0.15	**ORF1a:N2140I**	1	0.13	**ORF1a:L3644F**	1	0.15	**ORF1a:V3496***	11	0.31
Maharashtra	**ORF1a:A2909S**	1	0.15	**ORF1a:V2206T**	1	0.13	**ORF1a:I547P**	1	0.15	**ORF1a:A3497P**	11	0.31
Maharashtra	**ORF1a:F3614L**	1	0.15	**ORF1a:A2279T**	1	0.13	**ORF1a:F548L**	1	0.15	**ORF1a:M3498C**	11	0.31
Maharashtra	**ORF1a:A3615S**	1	0.15	**ORF1a:T2777K**	1	0.13	**ORF1a:S549F**	1	0.15	**ORF1a:K3499G**	11	0.31
Maharashtra	**ORF1a:V1722D**	1	0.15	**ORF1a:Y2788***	1	0.13	**ORF1a:R550L**	1	0.15	**ORF1a:M2259I**	11	0.31
Maharashtra	**ORF1a:G1723S**	1	0.15	**ORF1a:I2790L**	1	0.13	**ORF1a:G415S**	1	0.15	**ORF1a:Y1489H**	11	0.31
Maharashtra	**ORF1a:R4331C**	1	0.15	**ORF1a:T2791I**	1	0.13	**ORF1a:A668V**	1	0.15	**ORF1a:K1529L**	11	0.31
Maharashtra	**ORF1a:V2372I**	1	0.15	**ORF1a:P2792L**	1	0.13	**ORF1a:A1812N**	1	0.15	**ORF1a:G1601N**	11	0.31
Maharashtra	**ORF1a:C2288F**	1	0.15	**ORF1a:V2793I**	1	0.13	**ORF1a:Q3599H**	1	0.15	**ORF1a:A1602S**	11	0.31
Maharashtra	**ORF1a:F2633L**	1	0.15	**ORF1a:H2794I**	1	0.13	**ORF1a:T866I**	1	0.15	**ORF1a:T1605M**	11	0.31
Maharashtra	**ORF1a:M3312I**	1	0.15	**ORF1a:W3888***	1	0.13	**ORF1a:T1854I**	1	0.15	**ORF1a:I1607V**	11	0.31
Maharashtra	**ORF1a:Y3803C**	1	0.15	**ORF1a:V1118A**	1	0.13	**ORF1a:Q4099L**	1	0.15	**ORF1a:P1609H**	11	0.31
Maharashtra	**ORF1a:Q4397***	1	0.15	**ORF1a:S1520P**	1	0.13	**ORF1a:A2944V**	1	0.15	**ORF1a:H1610F**	11	0.31
Maharashtra	**ORF1a:P765S**	1	0.15	**ORF1a:G4178D**	1	0.13	**ORF1a:E237K**	1	0.15	**ORF1a:P1622R**	11	0.31
Maharashtra	**ORF1a:R3993C**	1	0.15	**ORF1a:T753I**	1	0.13	**ORF1a:S2193F**	1	0.15	**ORF1a:N1623L**	11	0.31
Maharashtra	**ORF1a:G133C**	1	0.15	**ORF1a:L3806***	1	0.13	**ORF1a:A2855V**	1	0.15	**ORF1a:D1624L**	11	0.31
Maharashtra	**ORF1a:E870G**	1	0.15	**ORF1a:K1470Q**	1	0.13	**ORF1a:S1029G**	1	0.15	**ORF1a:D1625S**	11	0.31
Maharashtra	**ORF1a:M2096L**	1	0.15	**ORF1a:V2866A**	1	0.13	**ORF1a:I1232T**	1	0.15	**ORF1a:T1854R**	11	0.31
Maharashtra	**ORF1a:S2695T**	1	0.15	**ORF1a:A3730S**	1	0.13	**ORF1a:N508Y**	1	0.15	**ORF1a:D1962***	11	0.31
Maharashtra	**ORF1a:A2925P**	1	0.15	**ORF1a:D144E**	1	0.13	**ORF1a:Q1918H**	1	0.15	**ORF1a:N2228K**	11	0.31
Maharashtra	**ORF1a:M789A**	1	0.15	**ORF1a:T2280I**	1	0.13	**ORF1a:L3606C**	1	0.15	**ORF1a:I2231P**	11	0.31
Maharashtra	**ORF1a:L790M**	1	0.15	**ORF1a:V3629N**	1	0.13	**ORF1a:E37Y**	1	0.15	**ORF1a:A2345I**	11	0.31
Maharashtra	**ORF1a:L791***	1	0.15	**ORF1a:H3631K**	1	0.13	**ORF1a:L39A**	1	0.15	**ORF1a:I2346V**	11	0.31
Maharashtra	**ORF1a:E792***	1	0.15	**ORF1a:H3633K**	1	0.13	**ORF1a:S40L**	1	0.15	**ORF1a:E2422L**	11	0.31
Maharashtra	**ORF1a:I793V**	1	0.15	**ORF1a:F3770L**	1	0.13	**ORF1a:R43***	1	0.15	**ORF1a:C2423Q**	11	0.31
Maharashtra	**ORF1a:L1546P**	1	0.15	**ORF1a:Y3765***	1	0.13	**ORF1a:Q44***	1	0.15	**ORF1a:T2425***	11	0.31
Maharashtra	**ORF1a:V1550G**	1	0.15	**ORF1a:I404V**	1	0.13	**ORF1a:H45K**	1	0.15	**ORF1a:I2426***	11	0.31
Maharashtra	**ORF1a:I1551N**	1	0.15	**ORF1a:E1293D**	1	0.13	**ORF1a:R73S**	1	0.15	**ORF1a:V2427S**	11	0.31
Maharashtra	**ORF1a:E1630V**	1	0.15	**ORF1a:S2926F**	1	0.13	**ORF1a:D75N**	1	0.15	**ORF1a:G2429K**	11	0.31
Maharashtra	**ORF1a:A1631C**	1	0.15	**ORF1a:L393F**	1	0.13	**ORF1a:A76V**	1	0.15	**ORF1a:V2430S**	11	0.31
Maharashtra	**ORF1a:A668T**	1	0.15	**ORF1a:K2108R**	1	0.13	**ORF1a:V86L**	1	0.15	**ORF1a:A2832V**	11	0.31
Maharashtra	**ORF1a:E1384***	1	0.15	**ORF1a:M2380I**	1	0.13	**ORF1a:L107V**	1	0.15	**ORF1a:G1723L**	11	0.31
Maharashtra	**ORF1a:V2052M**	1	0.15	**ORF1a:A1234V**	1	0.13	**ORF1a:P109R**	1	0.15	**ORF1a:L1725K**	11	0.31
Maharashtra	**ORF1a:D3450G**	1	0.15	**ORF1a:D1939G**	1	0.13	**ORF1a:K120L**	1	0.15	**ORF1a:G1726Q**	11	0.31
Maharashtra	**ORF1a:G3746D**	1	0.15	**ORF1a:S1561F**	1	0.13	**ORF1a:L122S**	1	0.15	**ORF1a:D1727***	11	0.31
Maharashtra	**ORF1a:S479F**	1	0.15	**ORF1a:G2322L**	1	0.13	**ORF1a:R124I**	1	0.15	**ORF1a:S1743T**	11	0.31
Maharashtra	**ORF1a:R643K**	1	0.15	**ORF1a:V2324N**	1	0.13	**ORF1a:G150K**	1	0.15	**ORF1a:K1984P**	11	0.31
Maharashtra	**ORF1a:M3131L**	1	0.15	**ORF1a:A2325G**	1	0.13	**ORF1a:R175G**	1	0.15	**ORF1a:H1987R**	11	0.31
Maharashtra	**ORF1a:V3136I**	1	0.15	**ORF1a:E2326I**	1	0.13	**ORF1a:E176T**	1	0.15	**ORF1a:P1989L**	11	0.31
Maharashtra	**ORF1a:A478S**	1	0.15	**ORF1a:W2327***	1	0.13	**ORF1a:G179A**	1	0.15	**ORF1a:N2065Y**	11	0.31
Maharashtra	**ORF1a:L3116F**	1	0.15	**ORF1a:A2330L**	1	0.13	**ORF1a:G180M**	1	0.15	**ORF1a:T2069F**	11	0.31
Maharashtra	**ORF1a:K699L**	1	0.15	**ORF1a:Y2331A**	1	0.13	**ORF1a:A181S**	1	0.15	**ORF1a:E2070L**	11	0.31
Maharashtra	**ORF1a:K701D**	1	0.15	**ORF1a:I2332***	1	0.13	**ORF1a:Y182I**	1	0.15	**ORF1a:V2071S**	11	0.31
Maharashtra	**ORF1a:A702T**	1	0.15	**ORF1a:T2335W**	1	0.13	**ORF1a:R184T**	1	0.15	**ORF1a:G2073Q**	11	0.31
Maharashtra	**ORF1a:L703Y**	1	0.15	**ORF1a:R2336H**	1	0.13	**ORF1a:Y185S**	1	0.15	**ORF1a:D2074Q**	11	0.31
Maharashtra	**ORF1a:N704P**	1	0.15	**ORF1a:F2337I**	1	0.13	**ORF1a:G209L**	1	0.15	**ORF1a:Y2217R**	11	0.31
Maharashtra	**ORF1a:L705S**	1	0.15	**ORF1a:Y2339F**	1	0.13	**ORF1a:K210M**	1	0.15	**ORF1a:K2219H**	11	0.31
Maharashtra	**ORF1a:G706L**	1	0.15	**ORF1a:V2340S**	1	0.13	**ORF1a:S212N**	1	0.15	**ORF1a:S2220L**	11	0.31
Maharashtra	**ORF1a:K527N**	1	0.15	**ORF1a:L2343D**	1	0.13	**ORF1a:C213W**	1	0.15	**ORF1a:P2221I**	11	0.31
Maharashtra	**ORF1a:C1932I**	1	0.15	**ORF1a:A2344W**	1	0.13	**ORF1a:D223L**	1	0.15	**ORF1a:N2222I**	11	0.31
Maharashtra	**ORF1a:P1950S**	1	0.15	**ORF1a:A2345L**	1	0.13	**ORF1a:K225L**	1	0.15	**ORF1a:F2223***	11	0.31
Maharashtra	**ORF1a:V54A**	1	0.15	**ORF1a:I2346Q**	1	0.13	**ORF1a:Q252G**	1	0.15	**ORF1a:K2225H**	11	0.31
Maharashtra	**ORF1a:F499L**	1	0.15	**ORF1a:M2347Q**	1	0.13	**ORF1a:P254R**	1	0.15	**ORF1a:T2335W**	11	0.31
Maharashtra	**ORF1a:E755K**	1	0.15	**ORF1a:A2142S**	1	0.13	**ORF1a:F255N**	1	0.15	**ORF1a:R2336H**	11	0.31
Maharashtra	**ORF1a:T3741A**	1	0.15	**ORF1a:K1305N**	1	0.13	**ORF1a:E256V**	1	0.15	**ORF1a:F2337I**	11	0.31

Maharashtra	ORF1a:N1335D	1	0.15	ORF1a:S4382I	1	0.13	ORF1a:I257L	1	0.15	ORF1a:Y2339F	11	0.31
Maharashtra	ORF1a:T1543K	1	0.15	ORF1a:D1939N	1	0.13	ORF1a:L259R	1	0.15	ORF1a:V2340S	11	0.31
Maharashtra	ORF1a:A2925D	1	0.15	ORF1a:S3913P	1	0.13	ORF1a:P286L	1	0.15	ORF1a:L2343F	11	0.31
Maharashtra	ORF1a:Q3826H	1	0.15	ORF1a:G192S	1	0.13	ORF1a:R287W	1	0.15	ORF1a:E2112L	11	0.31
Maharashtra	ORF1a:T265I	1	0.15	ORF1a:R226K	1	0.13	ORF1a:M315F	1	0.15	ORF1a:T1552F	11	0.31
Maharashtra	ORF1a:A3474V	1	0.15	ORF1a:L2253F	1	0.13	ORF1a:C316D	1	0.15	ORF1a:D1554L	11	0.31
Maharashtra	ORF1a:V3475I	1	0.15	ORF1a:K3832N	1	0.13	ORF1a:T319K	1	0.15	ORF1a:N1555*	11	0.31
Maharashtra	ORF1a:I1839V	1	0.15	ORF1a:R2818S	1	0.13	ORF1a:F343H	1	0.15	ORF1a:L1556E	11	0.31
Maharashtra	ORF1a:Q362K	1	0.15	ORF1a:W3128*	1	0.13	ORF1a:C344G	1	0.15	ORF1a:T1558*	11	0.31
Maharashtra	ORF1a:F651S	1	0.15	ORF1a:E233K	1	0.13	ORF1a:G345R	1	0.15	ORF1a:L1559G	11	0.31
Maharashtra	ORF1a:L552Q	1	0.15	ORF1a:L2105F	1	0.13	ORF1a:T346R	1	0.15	ORF1a:K2589R	11	0.31
Maharashtra	ORF1a:F641V	1	0.15	ORF1a:H2799D	1	0.13	ORF1a:E347A	1	0.15	ORF1a:V2629Q	10	0.28
Maharashtra	ORF1a:M2380I	1	0.15	ORF1a:T2936I	1	0.13	ORF1a:N348I	1	0.15	ORF1a:T2648G	10	0.28
Maharashtra	ORF1a:G82V	1	0.15	ORF1a:A535V	1	0.13	ORF1a:T350L	1	0.15	ORF1a:D2650N	10	0.28
Maharashtra	ORF1a:K634N	1	0.15	ORF1a:V2613F	1	0.13	ORF1a:E352P	1	0.15	ORF1a:V2652L	10	0.28
Maharashtra	ORF1a:L446P	1	0.15	ORF1a:S1027G	1	0.13	ORF1a:G353L	1	0.15	ORF1a:E2653N	10	0.28
Maharashtra	ORF1a:V2061I	1	0.15	ORF1a:S481F	1	0.13	ORF1a:E377I	1	0.15	ORF1a:L2655*	10	0.28
Maharashtra	ORF1a:V4181I	1	0.15	ORF1a:L1243M	1	0.13	ORF1a:G379L	1	0.15	ORF1a:N2708V	10	0.28
Maharashtra	ORF1a:S1433A	1	0.15	ORF1a:A2249T	1	0.13	ORF1a:R402N	1	0.15	ORF1a:H2883E	10	0.28
Maharashtra	ORF1a:T4204N	1	0.15	ORF1a:R3821K	1	0.13	ORF1a:P425H	1	0.15	ORF1a:P2886V	10	0.28
Maharashtra	ORF1a:K120N	1	0.15	ORF1a:Y3822N	1	0.13	ORF1a:D448T	1	0.15	ORF1a:R2887Q	10	0.28
Maharashtra	ORF1a:K4348R	1	0.15	ORF1a:L1852F	1	0.13	ORF1a:N449P	1	0.15	ORF1a:V2888L	10	0.28
Maharashtra	ORF1a:P3371S	1	0.15	ORF1a:G2258C	1	0.13	ORF1a:L450K	1	0.15	ORF1a:F2889V	10	0.28
Maharashtra	ORF1a:T1682I	1	0.15	ORF1a:A76*	1	0.13	ORF1a:L451R	1	0.15	ORF1a:S2890T	10	0.28
Maharashtra	ORF1a:Q4306H	1	0.15	ORF1a:R77H	1	0.13	ORF1a:L477F	1	0.15	ORF1a:A2891S	10	0.28
Maharashtra	ORF1a:M3425I	1	0.15	ORF1a:T78S	1	0.13	ORF1a:A478F	1	0.15	ORF1a:A3237L	10	0.28
Maharashtra	ORF1a:A3523V	1	0.15	ORF1a:A79P	1	0.13	ORF1a:S479C	1	0.15	ORF1a:N3239V	10	0.28
Maharashtra	ORF1a:M3761V	1	0.15	ORF1a:P80R	1	0.13	ORF1a:Y496H	1	0.15	ORF1a:D3240V	10	0.28
Maharashtra	ORF1a:P80S	1	0.15	ORF1a:H81V	1	0.13	ORF1a:A498C	1	0.15	ORF1a:Y3250P	10	0.28
Maharashtra	ORF1a:D264E	1	0.15	ORF1a:G82F	1	0.13	ORF1a:K500C	1	0.15	ORF1a:Q3251L	10	0.28
Maharashtra	ORF1a:S2242F	1	0.15	ORF1a:T3453I	1	0.13	ORF1a:Q501G	1	0.15	ORF1a:P3252S	10	0.28
Maharashtra	ORF1a:T2952I	1	0.15	ORF1a:A903V	1	0.13	ORF1a:I502N	1	0.15	ORF1a:C3408L	10	0.28
Maharashtra	ORF1a:I3779V	1	0.15	ORF1a:F1154L	1	0.13	ORF1a:V503C	1	0.15	ORF1a:G3409L	10	0.28
Maharashtra	ORF1a:P1213L	1	0.15	ORF1a:T3665I	1	0.13	ORF1a:S505R	1	0.15	ORF1a:S3410R	10	0.28
Maharashtra	ORF1a:E1217G	1	0.15	ORF1a:K3929N	1	0.13	ORF1a:C506F	1	0.15	ORF1a:D3439L	10	0.28
Maharashtra	ORF1a:K3979N	1	0.15	ORF1a:G524V	1	0.13	ORF1a:N508*	1	0.15	ORF1a:E3441T	10	0.28
Maharashtra	ORF1a:M2194I	1	0.15	ORF1a:A2690V	1	0.13	ORF1a:K510*	1	0.15	ORF1a:N3443K	10	0.28
Maharashtra	ORF1a:A3379S	1	0.15	ORF1a:T587I	1	0.13	ORF1a:V511S	1	0.15	ORF1a:F3444Q	10	0.28
Maharashtra	ORF1a:S3099L	1	0.15	ORF1a:L2250R	1	0.13	ORF1a:T512Y	1	0.15	ORF1a:Y3445H	10	0.28
Maharashtra	ORF1a:S3547N	1	0.15	ORF1a:A2279*	1	0.13	ORF1a:G514R	1	0.15	ORF1a:G3446K	10	0.28
Maharashtra	ORF1a:D3864Y	1	0.15	ORF1a:N2187K	1	0.13	ORF1a:E525S	1	0.15	ORF1a:F3448L	10	0.28
Maharashtra	ORF1a:E3882*	1	0.15	ORF1a:T2800I	1	0.13	ORF1a:Q526*	1	0.15	ORF1a:D3450R	10	0.28
Maharashtra	ORF1a:S2303Y	1	0.15	ORF1a:Y3744C	1	0.13	ORF1a:S528R	1	0.15	ORF1a:R3451T	10	0.28
Maharashtra	ORF1a:V606F	1	0.15	ORF1a:V3283A	1	0.13	ORF1a:I529C	1	0.15	ORF1a:W3470L	10	0.28
Maharashtra	ORF1a:T609I	1	0.15	ORF1a:I476N	1	0.13	ORF1a:P532S	1	0.15	ORF1a:Y3472Q	10	0.28
Maharashtra	ORF1a:S610L	1	0.15	ORF1a:A1151G	1	0.13	ORF1a:S546C	1	0.15	ORF1a:A3473L	10	0.28
Maharashtra	ORF1a:P3359H	1	0.15	ORF1a:G1152C	1	0.13	ORF1a:F548C	1	0.15	ORF1a:A3474M	10	0.28
Maharashtra	ORF1a:D1897A	1	0.15	ORF1a:F2387Y	1	0.13	ORF1a:S549I	1	0.15	ORF1a:V3475F	10	0.28
Maharashtra	ORF1a:Y1285H	1	0.15	ORF1a:N2878K	1	0.13	ORF1a:T551F	1	0.15	ORF1a:L3711M	10	0.28
Maharashtra	ORF1a:P1330H	1	0.15	ORF1a:G2879W	1	0.13	ORF1a:L552T	1	0.15	ORF1a:N3713M	10	0.28
Maharashtra	ORF1a:G1331K	1	0.15	ORF1a:D2880*	1	0.13	ORF1a:M584W	1	0.15	ORF1a:F509L	10	0.28
Maharashtra	ORF1a:Q1332L	1	0.15	ORF1a:F2881L	1	0.13	ORF1a:M585N	1	0.15	ORF1a:V511L	10	0.28
Maharashtra	ORF1a:G1333L	1	0.15	ORF1a:N3609I	1	0.13	ORF1a:S588V	1	0.15	ORF1a:T512Q	10	0.28
Maharashtra	ORF1a:L1334S	1	0.15	ORF1a:S308L	1	0.13	ORF1a:D589F	1	0.15	ORF1a:G514E	10	0.28
Maharashtra	ORF1a:N1335L	1	0.15	ORF1a:P1497S	1	0.13	ORF1a:L590T	1	0.15	ORF1a:A516L	10	0.28
Maharashtra	ORF1a:G1336H	1	0.15	ORF1a:N786I	1	0.13	ORF1a:A591E	1	0.15	ORF1a:A735S	10	0.28
Maharashtra	ORF1a:K1470*	1	0.15	ORF1a:D3450T	1	0.13	ORF1a:N594G	1	0.15	ORF1a:L4111F	10	0.28
Maharashtra	ORF1a:V1471E	1	0.15	ORF1a:R3451A	1	0.13	ORF1a:V596S	1	0.15	ORF1a:L1650K	10	0.28
Maharashtra	ORF1a:T2093K	1	0.15	ORF1a:T3807N	1	0.13	ORF1a:I693D	1	0.15	ORF1a:T1653*	10	0.28
Maharashtra	ORF1a:D2094G	1	0.15	ORF1a:I3768N	1	0.13	ORF1a:I695L	1	0.15	ORF1a:N1662E	10	0.28
Maharashtra	ORF1a:S2900L	1	0.15	ORF1a:N363T	1	0.13	ORF1a:G696*	1	0.15	ORF1a:G1663I	10	0.28
Maharashtra	ORF1a:T3749R	1	0.15	ORF1a:V3595G	1	0.13	ORF1a:G697A	1	0.15	ORF1a:L1664P	10	0.28
Maharashtra	ORF1a:M4071I	1	0.15	ORF1a:P1596S	1	0.13	ORF1a:A698C	1	0.15	ORF1a:I1714L	10	0.28
Maharashtra	ORF1a:W4096C	1	0.15	ORF1a:M2606I	1	0.13	ORF1a:A776N	1	0.15	ORF1a:V1768D	10	0.28
Maharashtra	ORF1a:E4097H	1	0.15	ORF1a:Y1646H	1	0.13	ORF1a:P777W	1	0.15	ORF1a:M1769N	10	0.28
Maharashtra	ORF1a:S4398C	1	0.15	ORF1a:T1093I	1	0.13	ORF1a:L778*	1	0.15	ORF1a:Y1770P	10	0.28
Maharashtra	ORF1a:L4400S	1	0.15	ORF1a:G1119S	1	0.13	ORF1a:V779F	1	0.15	ORF1a:M1771*	10	0.28
Maharashtra	ORF1a:L1681Q	1	0.15	ORF1a:I1159V	1	0.13	ORF1a:G780T	1	0.15	ORF1a:K1795C	10	0.28
Maharashtra	ORF1a:E2031V	1	0.15	ORF1a:G2207S	1	0.13	ORF1a:P782Y	1	0.15	ORF1a:K2548V	10	0.28
Maharashtra	ORF1a:D2032H	1	0.15	ORF1a:T1426N	1	0.13	ORF1a:P804R	1	0.15	ORF1a:D2801N	10	0.28
Maharashtra	ORF1a:S2193T	1	0.15	ORF1a:T3258I	1	0.13	ORF1a:M806R	1	0.15	ORF1a:F2802T	10	0.28
Maharashtra	ORF1a:G989V	1	0.15	ORF1a:Q1224L	1	0.13	ORF1a:M807R	1	0.15	ORF1a:T2920N	10	0.28
Maharashtra	ORF1a:P1096L	1	0.15	ORF1a:F2780L	1	0.13	ORF1a:V808C	1	0.15	ORF1a:E2982L	10	0.28

Maharashtra	**ORF1a:S3989F**	1	0.15	**ORF1a:A2344V**	1	0.13	**ORF1a:Q834K**	1	0.15	**ORF1a:Y2983***	10	0.28
Maharashtra	**ORF1a:M789I**	1	0.15	**ORF1a:V424I**	1	0.13	**ORF1a:V868***	1	0.15	**ORF1a:C2984A**	10	0.28
Maharashtra	**ORF1a:Q1813H**	1	0.15	**ORF1a:M2365I**	1	0.13	**ORF1a:N869***	1	0.15	**ORF1a:D953Y**	10	0.28
Maharashtra	**ORF1a:A2529V**	1	0.15	**ORF1a:L1016I**	1	0.13	**ORF1a:F871V**	1	0.15	**ORF1a:M3934I**	10	0.28
Maharashtra	**ORF1a:A206V**	1	0.15	**ORF1a:T1055I**	1	0.13	**ORF1a:A872L**	1	0.15	**ORF1a:V3875A**	10	0.28
Maharashtra	**ORF1a:K492R**	1	0.15	**ORF1a:T965I**	1	0.13	**ORF1a:V874N**	1	0.15	**ORF1a:A421L**	10	0.28
Maharashtra	**ORF1a:A2587V**	1	0.15	**ORF1a:S1218I**	1	0.13	**ORF1a:V875F**	1	0.15	**ORF1a:G603L**	10	0.28
Maharashtra	**ORF1a:A1473V**	1	0.15	**ORF1a:P1158L**	1	0.13	**ORF1a:L893I**	1	0.15	**ORF1a:G604Q**	10	0.28
Maharashtra	**ORF1a:G2294C**	1	0.15	**ORF1a:M584V**	1	0.13	**ORF1a:G894***	1	0.15	**ORF1a:Q607***	10	0.28
Maharashtra	**ORF1a:P1147S**	1	0.15	**ORF1a:V1020I**	1	0.13	**ORF1a:Y906I**	1	0.15	**ORF1a:K636L**	10	0.28
Maharashtra	**ORF1a:A4257V**	1	0.15	**ORF1a:G2927S**	1	0.13	**ORF1a:L907G**	1	0.15	**ORF1a:G638T**	10	0.28
Maharashtra	**ORF1a:T1093A**	1	0.15	**ORF1a:Q1813E**	1	0.13	**ORF1a:M920T**	1	0.15	**ORF1a:E640G**	10	0.28
Maharashtra	**ORF1a:A1923S**	1	0.15	**ORF1a:E37G**	1	0.13	**ORF1a:C922V**	1	0.15	**ORF1a:F641K**	10	0.28
Maharashtra	**ORF1a:F2864V**	1	0.15	**ORF1a:A555V**	1	0.13	**ORF1a:S923L**	1	0.15	**ORF1a:P765K**	10	0.28
Maharashtra	**ORF1a:L3352F**	1	0.15	**ORF1a:E940G**	1	0.13	**ORF1a:E942L**	1	0.15	**ORF1a:S1218R**	10	0.28
Maharashtra	**ORF1a:T12I**	1	0.15	**ORF1a:I601T**	1	0.13	**ORF1a:T945V**	1	0.15	**ORF1a:P1220L**	10	0.28
Maharashtra	**ORF1a:V1056L**	1	0.15	**ORF1a:T781I**	1	0.13	**ORF1a:Q946W**	1	0.15	**ORF1a:F1248I**	10	0.28
Maharashtra	**ORF1a:G618C**	1	0.15	**ORF1a:P2018S**	1	0.13	**ORF1a:E948***	1	0.15	**ORF1a:K1512L**	10	0.28
Maharashtra	**ORF1a:P1640L**	1	0.15	**ORF1a:M2096K**	1	0.13	**ORF1a:Y949R**	1	0.15	**ORF1a:S2024I**	10	0.28
Maharashtra	**ORF1a:D3416G**	1	0.15	**ORF1a:L64F**	1	0.13	**ORF1a:G950***	1	0.15	**ORF1a:F2025K**	10	0.28
Maharashtra	**ORF1a:P765L**	1	0.15	**ORF1a:A520T**	1	0.13	**ORF1a:T951L**	1	0.15	**ORF1a:D2026F**	10	0.28
Maharashtra	**ORF1a:S2224F**	1	0.15	**ORF1a:A2994V**	1	0.13	**ORF1a:E952P**	1	0.15	**ORF1a:L2028***	10	0.28
Maharashtra	**ORF1a:V1211F**	1	0.15	**ORF1a:V106I**	1	0.13	**ORF1a:D953R**	1	0.15	**ORF1a:K2029C**	10	0.28
Maharashtra	**ORF1a:P371S**	1	0.15	**ORF1a:T4219I**	1	0.13	**ORF1a:D954***	1	0.15	**ORF1a:S2030T**	10	0.28
Maharashtra	**ORF1a:I1274F**	1	0.15	**ORF1a:T2825I**	1	0.13	**ORF1a:Y955T**	1	0.15	**ORF1a:D2032V**	10	0.28
Maharashtra	**ORF1a:G49C**	1	0.15	**ORF1a:E1205D**	1	0.13	**ORF1a:Q956F**	1	0.15	**ORF1a:A2033R**	10	0.28
Maharashtra	**ORF1a:E1498V**	1	0.15	**ORF1a:T2106I**	1	0.13	**ORF1a:K958I**	1	0.15	**ORF1a:Q2034G**	10	0.28
Maharashtra	**ORF1a:T2093I**	1	0.15	**ORF1a:S2285F**	1	0.13	**ORF1a:P959W**	1	0.15	**ORF1a:G2035R**	10	0.28
Maharashtra	**ORF1a:G728C**	1	0.15	**ORF1a:V337F**	1	0.13	**ORF1a:L960C**	1	0.15	**ORF1a:M2036A**	10	0.28
Maharashtra	**ORF1a:A1283V**	1	0.15	**ORF1a:L3951F**	1	0.13	**ORF1a:E961H**	1	0.15	**ORF1a:D2037G**	10	0.28
Maharashtra	**ORF1a:R24C**	1	0.15	**ORF1a:M3129V**	1	0.13	**ORF1a:A964C**	1	0.15	**ORF1a:A2040***	10	0.28
Maharashtra	**ORF1a:N1324S**	1	0.15	**ORF1a:S3949N**	1	0.13	**ORF1a:T965S**	1	0.15	**ORF1a:C2041R**	10	0.28
Maharashtra	**ORF1a:V116M**	1	0.15	**ORF1a:D1885N**	1	0.13	**ORF1a:V988***	1	0.15	**ORF1a:E2042S**	10	0.28
Maharashtra	**ORF1a:T403I**	1	0.15	**ORF1a:L4182F**	1	0.13	**ORF1a:G1069Y**	1	0.15	**ORF1a:D2043S**	10	0.28
Maharashtra	**ORF1a:D335G**	1	0.15	**ORF1a:I453T**	1	0.13	**ORF1a:G1070***	1	0.15	**ORF1a:L2044G**	10	0.28
Maharashtra	**ORF1a:P4220L**	1	0.15	**ORF1a:R3480S**	1	0.13	**ORF1a:V1071Q**	1	0.15	**ORF1a:S2103G**	10	0.28
Maharashtra	**ORF1a:F1178L**	1	0.15	**ORF1a:N3491K**	1	0.13	**ORF1a:A1072C**	1	0.15	**ORF1a:S2104C**	10	0.28
Maharashtra	**ORF1a:D1179I**	1	0.15	**ORF1a:R3993C**	1	0.13	**ORF1a:G1073H**	1	0.15	**ORF1a:F2145***	10	0.28
Maharashtra	**ORF1a:N1181I**	1	0.15	**ORF1a:M1014I**	1	0.13	**ORF1a:C1103***	1	0.15	**ORF1a:K2148Y**	10	0.28
Maharashtra	**ORF1a:L1182S**	1	0.15	**ORF1a:M1769I**	1	0.13	**ORF1a:V1104T**	1	0.15	**ORF1a:V2149***	10	0.28
Maharashtra	**ORF1a:Y1183M**	1	0.15	**ORF1a:M3345I**	1	0.13	**ORF1a:Y1183S**	1	0.15	**ORF1a:V2150H**	10	0.28
Maharashtra	**ORF1a:D1184T**	1	0.15	**ORF1a:A3061V**	1	0.13	**ORF1a:D1184H**	1	0.15	**ORF1a:T2152Y**	10	0.28
Maharashtra	**ORF1a:K1185N**	1	0.15	**ORF1a:L4126I**	1	0.13	**ORF1a:L1186C**	1	0.15	**ORF1a:L2177Y**	10	0.28
Maharashtra	**ORF1a:S1188K**	1	0.15	**ORF1a:E2031D**	1	0.13	**ORF1a:V1187L**	1	0.15	**ORF1a:S2188***	10	0.28
Maharashtra	**ORF1a:F1190K**	1	0.15	**ORF1a:E4002D**	1	0.13	**ORF1a:S1188L**	1	0.15	**ORF1a:R2189S**	10	0.28
Maharashtra	**ORF1a:E1192N**	1	0.15	**ORF1a:T677I**	1	0.13	**ORF1a:F1190C**	1	0.15	**ORF1a:K2191Y**	10	0.28
Maharashtra	**ORF1a:M1193K**	1	0.15	**ORF1a:R579K**	1	0.13	**ORF1a:E1192***	1	0.15	**ORF1a:C2210E**	10	0.28
Maharashtra	**ORF1a:K1194R**	1	0.15	**ORF1a:P2046S**	1	0.13	**ORF1a:M1193***	1	0.15	**ORF1a:L2211V**	10	0.28
Maharashtra	**ORF1a:E1196L**	1	0.15	**ORF1a:T1822I**	1	0.13	**ORF1a:V1211***	1	0.15	**ORF1a:E2212T**	10	0.28
Maharashtra	**ORF1a:Q1227L**	1	0.15	**ORF1a:G2269R**	1	0.13	**ORF1a:P1213F**	1	0.15	**ORF1a:A2213***	10	0.28
Maharashtra	**ORF1a:D1228V**	1	0.15	**ORF1a:C4381W**	1	0.13	**ORF1a:F1214S**	1	0.15	**ORF1a:S2214F**	10	0.28
Maharashtra	**ORF1a:D1229L**	1	0.15	**ORF1a:T2936A**	1	0.13	**ORF1a:I1215***	1	0.15	**ORF1a:Y2217T**	10	0.28
Maharashtra	**ORF1a:L1255M**	1	0.15	**ORF1a:T1567I**	1	0.13	**ORF1a:T1240K**	1	0.15	**ORF1a:L2218D**	10	0.28
Maharashtra	**ORF1a:Y1256A**	1	0.15	**ORF1a:T3646A**	1	0.13	**ORF1a:T1242R**	1	0.15	**ORF1a:S2220Y**	10	0.28
Maharashtra	**ORF1a:D1273F**	1	0.15	**ORF1a:D2299***	1	0.13	**ORF1a:L1243***	1	0.15	**ORF1a:P2221Y**	10	0.28
Maharashtra	**ORF1a:D1275Q**	1	0.15	**ORF1a:T2300H**	1	0.13	**ORF1a:E1244***	1	0.15	**ORF1a:F2223L**	10	0.28
Maharashtra	**ORF1a:T1277H**	1	0.15	**ORF1a:S610L**	1	0.13	**ORF1a:T1246R**	1	0.15	**ORF1a:S2224V**	10	0.28
Maharashtra	**ORF1a:T1309Y**	1	0.15	**ORF1a:S318L**	1	0.13	**ORF1a:S1272***	1	0.15	**ORF1a:K2225F**	10	0.28
Maharashtra	**ORF1a:E1311R**	1	0.15	**ORF1a:L2179F**	1	0.13	**ORF1a:D1273***	1	0.15	**ORF1a:L2226T**	10	0.28
Maharashtra	**ORF1a:M1312Q**	1	0.15	**ORF1a:H110Y**	1	0.13	**ORF1a:I1274H**	1	0.15	**ORF1a:I2229C**	10	0.28
Maharashtra	**ORF1a:A1314Y**	1	0.15	**ORF1a:T4031I**	1	0.13	**ORF1a:D1275***	1	0.15	**ORF1a:I2230L**	10	0.28
Maharashtra	**ORF1a:K1315N**	1	0.15	**ORF1a:E37K**	1	0.13	**ORF1a:I1276H**	1	0.15	**ORF1a:W2232R**	10	0.28
Maharashtra	**ORF1a:A1316H**	1	0.15	**ORF1a:T2068A**	1	0.13	**ORF1a:T1277H**	1	0.15	**ORF1a:L2234F**	10	0.28
Maharashtra	**ORF1a:Q1332S**	1	0.15	**ORF1a:A1298V**	1	0.13	**ORF1a:L1317N**	1	0.15	**ORF1a:L2235N**	10	0.28
Maharashtra	**ORF1a:C1349F**	1	0.15	**ORF1a:K2200N**	1	0.13	**ORF1a:T1327Q**	1	0.15	**ORF1a:L2524F**	10	0.28
Maharashtra	**ORF1a:A1352T**	1	0.15	**ORF1a:M1588R**	1	0.13	**ORF1a:T1328L**	1	0.15	**ORF1a:D2525C**	10	0.28
Maharashtra	**ORF1a:F1353I**	1	0.15	**ORF1a:D1962E**	1	0.13	**ORF1a:Q1332S**	1	0.15	**ORF1a:N2526***	10	0.28
Maharashtra	**ORF1a:I1355Y**	1	0.15	**ORF1a:D335G**	1	0.13	**ORF1a:C1349F**	1	0.15	**ORF1a:I2774***	10	0.28
Maharashtra	**ORF1a:P1357***	1	0.15	**ORF1a:L730I**	1	0.13	**ORF1a:A1352T**	1	0.15	**ORF1a:T2846P**	10	0.28
Maharashtra	**ORF1a:S1358***	1	0.15	**ORF1a:A1473V**	1	0.13	**ORF1a:F1353I**	1	0.15	**ORF1a:N2847I**	10	0.28
Maharashtra	**ORF1a:I1359E**	1	0.15	**ORF1a:K1655N**	1	0.13	**ORF1a:I1355Y**	1	0.15	**ORF1a:I2895***	10	0.28
Maharashtra	**ORF1a:I1360A**	1	0.15	**ORF1a:L107F**	1	0.13	**ORF1a:P1357***	1	0.15	**ORF1a:S2900H**	10	0.28
Maharashtra	**ORF1a:S1361R**	1	0.15	**ORF1a:C667Y**	1	0.13	**ORF1a:S1358***	1	0.15	**ORF1a:G3265W**	10	0.28

Maharashtra	**ORF1a:R1386C**	1	0.15	**ORF1a:V1211F**	1	0.13	**ORF1a:I1359E**	1	0.15	**ORF1a:R3267***	10	0.28
Maharashtra	**ORF1a:K1387F**	1	0.15	**ORF1a:I238T**	1	0.13	**ORF1a:I1360A**	1	0.15	**ORF1a:M3269N**	10	0.28
Maharashtra	**ORF1a:D1417N**	1	0.15	**ORF1a:S302F**	1	0.13	**ORF1a:S1361R**	1	0.15	**ORF1a:A3270G**	10	0.28
Maharashtra	**ORF1a:G1419T**	1	0.15	**ORF1a:S994N**	1	0.13	**ORF1a:L1375C**	1	0.15	**ORF1a:F3271I**	10	0.28
Maharashtra	**ORF1a:R1421***	1	0.15	**ORF1a:T2181I**	1	0.13	**ORF1a:R1376F**	1	0.15	**ORF1a:M3345T**	10	0.28
Maharashtra	**ORF1a:F1422I**	1	0.15	**ORF1a:V116L**	1	0.13	**ORF1a:E1377L**	1	0.15	**ORF1a:N3347T**	10	0.28
Maharashtra	**ORF1a:Y1423***	1	0.15	**ORF1a:T224I**	1	0.13	**ORF1a:M1378E**	1	0.15	**ORF1a:C3348F**	10	0.28
Maharashtra	**ORF1a:F1424G**	1	0.15	**ORF1a:T3058A**	1	0.13	**ORF1a:L1379F**	1	0.15	**ORF1a:V3349Y**	10	0.28
Maharashtra	**ORF1a:S1427Y**	1	0.15	**ORF1a:Y621C**	1	0.13	**ORF1a:H1381R**	1	0.15	**ORF1a:L3350A**	10	0.28
Maharashtra	**ORF1a:K1428Q**	1	0.15	**ORF1a:E1209D**	1	0.13	**ORF1a:A1382N**	1	0.15	**ORF1a:K1837L**	10	0.28
Maharashtra	**ORF1a:T1429H**	1	0.15	**ORF1a:E102A**	1	0.13	**ORF1a:E1383A**	1	0.15	**ORF1a:D1849N**	10	0.28
Maharashtra	**ORF1a:V1431***	1	0.15	**ORF1a:T2121I**	1	0.13	**ORF1a:E1384C**	1	0.15	**ORF1a:Q1918H**	10	0.28
Maharashtra	**ORF1a:A1432R**	1	0.15	**ORF1a:S3099L**	1	0.13	**ORF1a:G1419T**	1	0.15	**ORF1a:P1919I**	10	0.28
Maharashtra	**ORF1a:L1434K**	1	0.15	**ORF1a:T2648A**	1	0.13	**ORF1a:R1421***	1	0.15	**ORF1a:Y1920Q**	10	0.28
Maharashtra	**ORF1a:I1435***	1	0.15	**ORF1a:A3688T**	1	0.13	**ORF1a:F1422I**	1	0.15	**ORF1a:P1921T**	10	0.28
Maharashtra	**ORF1a:T1437N**	1	0.15	**ORF1a:N1442D**	1	0.13	**ORF1a:Y1423***	1	0.15	**ORF1a:N1922Q**	10	0.28
Maharashtra	**ORF1a:L1438A**	1	0.15	**ORF1a:A2710V**	1	0.13	**ORF1a:F1424G**	1	0.15	**ORF1a:Y2245***	10	0.28
Maharashtra	**ORF1a:N1439T**	1	0.15	**ORF1a:L3935Q**	1	0.13	**ORF1a:S1427Y**	1	0.15	**ORF1a:T2484K**	10	0.28
Maharashtra	**ORF1a:D1440W**	1	0.15	**ORF1a:A2856V**	1	0.13	**ORF1a:K1428Q**	1	0.15	**ORF1a:S3121D**	10	0.28
Maharashtra	**ORF1a:N1442C**	1	0.15	**ORF1a:L1130F**	1	0.13	**ORF1a:T1429H**	1	0.15	**ORF1a:F3122G**	10	0.28
Maharashtra	**ORF1a:E1443N**	1	0.15	**ORF1a:A3571V**	1	0.13	**ORF1a:V1431***	1	0.15	**ORF1a:L3123Y**	10	0.28
Maharashtra	**ORF1a:S1468W**	1	0.15	**ORF1a:T1246I**	1	0.13	**ORF1a:A1432R**	1	0.15	**ORF1a:A3124V**	10	0.28
Maharashtra	**ORF1a:V1471F**	1	0.15	**ORF1a:P1472S**	1	0.13	**ORF1a:L1434K**	1	0.15	**ORF1a:T999I**	10	0.28
Maharashtra	**ORF1a:P1472G**	1	0.15	**ORF1a:L590F**	1	0.13	**ORF1a:I1435***	1	0.15	**ORF1a:V1320L**	10	0.28
Maharashtra	**ORF1a:A1473R**	1	0.15	**ORF1a:Q2510L**	1	0.13	**ORF1a:T1437N**	1	0.15	**ORF1a:P1321***	10	0.28
Maharashtra	**ORF1a:T1474S**	1	0.15	**ORF1a:L3930F**	1	0.13	**ORF1a:L1438A**	1	0.15	**ORF1a:T1322E**	10	0.28
Maharashtra	**ORF1a:V1475C**	1	0.15	**ORF1a:S505P**	1	0.13	**ORF1a:N1439T**	1	0.15	**ORF1a:D1323K**	10	0.28
Maharashtra	**ORF1a:K1533F**	1	0.15	**ORF1a:A1043V**	1	0.13	**ORF1a:D1440W**	1	0.15	**ORF1a:N1324C**	10	0.28
Maharashtra	**ORF1a:S1534W**	1	0.15	**ORF1a:T2859I**	1	0.13	**ORF1a:N1442C**	1	0.15	**ORF1a:Y1325Q**	10	0.28
Maharashtra	**ORF1a:V1535T**	1	0.15	**ORF1a:A537T**	1	0.13	**ORF1a:E1443N**	1	0.15	**ORF1a:I1326Q**	10	0.28
Maharashtra	**ORF1a:Y1536I**	1	0.15	**ORF1a:A687V**	1	0.13	**ORF1a:S1468W**	1	0.15	**ORF1a:K1347V**	10	0.28
Maharashtra	**ORF1a:N1540Y**	1	0.15	**ORF1a:D1127N**	1	0.13	**ORF1a:V1471F**	1	0.15	**ORF1a:G1456L**	10	0.28
Maharashtra	**ORF1a:P1541H**	1	0.15	**ORF1a:A1204V**	1	0.13	**ORF1a:P1472G**	1	0.15	**ORF1a:N1458Q**	10	0.28
Maharashtra	**ORF1a:T1542L**	1	0.15	**ORF1a:F3375L**	1	0.13	**ORF1a:A1473R**	1	0.15	**ORF1a:L1459C**	10	0.28
Maharashtra	**ORF1a:T1543***	1	0.15	**ORF1a:A2375V**	1	0.13	**ORF1a:T1474S**	1	0.15	**ORF1a:E1460H**	10	0.28
Maharashtra	**ORF1a:F1544Q**	1	0.15	**ORF1a:T987A**	1	0.13	**ORF1a:V1475C**	1	0.15	**ORF1a:E1461L**	10	0.28
Maharashtra	**ORF1a:H1545S**	1	0.15	**ORF1a:I431M**	1	0.13	**ORF1a:A1812V**	1	0.15	**ORF1a:A1463M**	10	0.28
Maharashtra	**ORF1a:L1546***	1	0.15	**ORF1a:S2114F**	1	0.13	**ORF1a:Q1813S**	1	0.15	**ORF1a:R1464***	10	0.28
Maharashtra	**ORF1a:D1575Y**	1	0.15	**ORF1a:S2535L**	1	0.13	**ORF1a:Y1814M**	1	0.15	**ORF1a:Y1465H**	10	0.28
Maharashtra	**ORF1a:F1804S**	1	0.15	**ORF1a:V2588F**	1	0.13	**ORF1a:E1815N**	1	0.15	**ORF1a:R1467L**	10	0.28
Maharashtra	**ORF1a:Y1831L**	1	0.15	**ORF1a:V2866M**	1	0.13	**ORF1a:K1817L**	1	0.15	**ORF1a:S1468F**	10	0.28
Maharashtra	**ORF1a:Q1832S**	1	0.15	**ORF1a:S3244L**	1	0.13	**ORF1a:H1818V**	1	0.15	**ORF1a:M1014V**	10	0.28
Maharashtra	**ORF1a:C1833M**	1	0.15				**ORF1a:G1819S**	1	0.15	**ORF1a:K351R**	10	0.28
Maharashtra	**ORF1a:G1834V**	1	0.15				**ORF1a:K1837L**	1	0.15	**ORF1a:W2366L**	9	0.25
Maharashtra	**ORF1a:Y1836T**	1	0.15				**ORF1a:H1838V**	1	0.15	**ORF1a:R2420N**	9	0.25
Maharashtra	**ORF1a:D1849N**	1	0.15				**ORF1a:K1842V**	1	0.15	**ORF1a:K2442P**	9	0.25
Maharashtra	**ORF1a:G1850I**	1	0.15				**ORF1a:E1843V**	1	0.15	**ORF1a:G2443F**	9	0.25
Maharashtra	**ORF1a:A1851***	1	0.15				**ORF1a:D1849N**	1	0.15	**ORF1a:F2444M**	9	0.25
Maharashtra	**ORF1a:T1854K**	1	0.15				**ORF1a:G1850I**	1	0.15	**ORF1a:C2445S**	9	0.25
Maharashtra	**ORF1a:E1909N**	1	0.15				**ORF1a:A1851***	1	0.15	**ORF1a:K2446M**	9	0.25
Maharashtra	**ORF1a:P1911L**	1	0.15				**ORF1a:T1854K**	1	0.15	**ORF1a:L2571M**	9	0.25
Maharashtra	**ORF1a:D1913L**	1	0.15				**ORF1a:I1877K**	1	0.15	**ORF1a:A2587I**	9	0.25
Maharashtra	**ORF1a:L1914Y**	1	0.15				**ORF1a:P1879T**	1	0.15	**ORF1a:K2589I**	9	0.25
Maharashtra	**ORF1a:V1915Q**	1	0.15				**ORF1a:K1883L**	1	0.15	**ORF1a:M2590R**	9	0.25
Maharashtra	**ORF1a:P1916T**	1	0.15				**ORF1a:L1884***	1	0.15	**ORF1a:E2607H**	9	0.25
Maharashtra	**ORF1a:N1942I**	1	0.15				**ORF1a:D1885I**	1	0.15	**ORF1a:K2608Q**	9	0.25
Maharashtra	**ORF1a:Q1943I**	1	0.15				**ORF1a:V1887W**	1	0.15	**ORF1a:K2610L**	9	0.25
Maharashtra	**ORF1a:T1945S**	1	0.15				**ORF1a:V1888C**	1	0.15	**ORF1a:R2726L**	9	0.25
Maharashtra	**ORF1a:G1946L**	1	0.15				**ORF1a:F1907C**	1	0.15	**ORF1a:Q2728R**	9	0.25
Maharashtra	**ORF1a:K1949L**	1	0.15				**ORF1a:V1931K**	1	0.15	**ORF1a:D2819Q**	9	0.25
Maharashtra	**ORF1a:P1950H**	1	0.15				**ORF1a:C1932P**	1	0.15	**ORF1a:A2821L**	9	0.25
Maharashtra	**ORF1a:A1951F**	1	0.15				**ORF1a:D1933V**	1	0.15	**ORF1a:S2822V**	9	0.25
Maharashtra	**ORF1a:R1953L**	1	0.15				**ORF1a:E1954W**	1	0.15	**ORF1a:T2823L**	9	0.25
Maharashtra	**ORF1a:E1954T**	1	0.15				**ORF1a:K1956***	1	0.15	**ORF1a:D2824L**	9	0.25
Maharashtra	**ORF1a:L1955***	1	0.15				**ORF1a:V1957E**	1	0.15	**ORF1a:R2841N**	9	0.25
Maharashtra	**ORF1a:K1956M**	1	0.15				**ORF1a:F1959C**	1	0.15	**ORF1a:G2842M**	9	0.25
Maharashtra	**ORF1a:G1982M**	1	0.15				**ORF1a:P1961Y**	1	0.15	**ORF1a:G2843L**	9	0.25
Maharashtra	**ORF1a:A1983W**	1	0.15				**ORF1a:D1962***	1	0.15	**ORF1a:S2844I**	9	0.25
Maharashtra	**ORF1a:K1984W**	1	0.15				**ORF1a:K1984***	1	0.15	**ORF1a:R2875V**	9	0.25
Maharashtra	**ORF1a:H1987I**	1	0.15				**ORF1a:L1985T**	1	0.15	**ORF1a:T2877F**	9	0.25
Maharashtra	**ORF1a:C2012R**	1	0.15				**ORF1a:K1988S**	1	0.15	**ORF1a:N2878C**	9	0.25
Maharashtra	**ORF1a:E2042Q**	1	0.15				**ORF1a:P1989***	1	0.15	**ORF1a:G2879I**	9	0.25
Maharashtra	**ORF1a:D2043S**	1	0.15				**ORF1a:W1992S**	1	0.15	**ORF1a:D2880S**	9	0.25

Maharashtra	**ORF1a:L2105***	1	0.15				**ORF1a:S2015***	1	0.15	**ORF1a:F2881Y**	9	0.25
Maharashtra	**ORF1a:K2108***	1	0.15				**ORF1a:P2018L**	1	0.15	**ORF1a:G2893T**	9	0.25
Maharashtra	**ORF1a:N2111L**	1	0.15				**ORF1a:E2020Y**	1	0.15	**ORF1a:N2894H**	9	0.25
Maharashtra	**ORF1a:S2114M**	1	0.15				**ORF1a:K2029S**	1	0.15	**ORF1a:I2895H**	9	0.25
Maharashtra	**ORF1a:R2115V**	1	0.15				**ORF1a:S2030L**	1	0.15	**ORF1a:C2896Q**	9	0.25
Maharashtra	**ORF1a:V2116***	1	0.15				**ORF1a:Q2058S**	1	0.15	**ORF1a:Y2897N**	9	0.25
Maharashtra	**ORF1a:G2118L**	1	0.15				**ORF1a:D2060T**	1	0.15	**ORF1a:T2898L**	9	0.25
Maharashtra	**ORF1a:V2149G**	1	0.15				**ORF1a:V2061S**	1	0.15	**ORF1a:K2901T**	9	0.25
Maharashtra	**ORF1a:S2151***	1	0.15				**ORF1a:E2112C**	1	0.15	**ORF1a:E2970T**	9	0.25
Maharashtra	**ORF1a:F2172V**	1	0.15				**ORF1a:L2113Y**	1	0.15	**ORF1a:G2971P**	9	0.25
Maharashtra	**ORF1a:L2176I**	1	0.15				**ORF1a:M2169I**	1	0.15	**ORF1a:S2972T**	9	0.25
Maharashtra	**ORF1a:Q2178E**	1	0.15				**ORF1a:P2170V**	1	0.15	**ORF1a:V2973L**	9	0.25
Maharashtra	**ORF1a:C2180K**	1	0.15				**ORF1a:I2227S**	1	0.15	**ORF1a:C2997V**	9	0.25
Maharashtra	**ORF1a:T2181H**	1	0.15				**ORF1a:N2228R**	1	0.15	**ORF1a:Y3010G**	9	0.25
Maharashtra	**ORF1a:F2182L**	1	0.15				**ORF1a:I2229G**	1	0.15	**ORF1a:P3037H**	9	0.25
Maharashtra	**ORF1a:T2183C**	1	0.15				**ORF1a:I2230F**	1	0.15	**ORF1a:I3038L**	9	0.25
Maharashtra	**ORF1a:S2185L**	1	0.15				**ORF1a:W2232F**	1	0.15	**ORF1a:G3039***	9	0.25
Maharashtra	**ORF1a:T2186L**	1	0.15				**ORF1a:L2234N**	1	0.15	**ORF1a:A3040***	9	0.25
Maharashtra	**ORF1a:N2187***	1	0.15				**ORF1a:V2238R**	1	0.15	**ORF1a:K3162***	9	0.25
Maharashtra	**ORF1a:S2188Q**	1	0.15				**ORF1a:Y2266F**	1	0.15	**ORF1a:R3164D**	9	0.25
Maharashtra	**ORF1a:N2216L**	1	0.15				**ORF1a:S2558C**	1	0.15	**ORF1a:G3274H**	9	0.25
Maharashtra	**ORF1a:Y2217L**	1	0.15				**ORF1a:Q2563N**	1	0.15	**ORF1a:K3275L**	9	0.25
Maharashtra	**ORF1a:L2218R**	1	0.15				**ORF1a:M2565Y**	1	0.15	**ORF1a:E3277K**	9	0.25
Maharashtra	**ORF1a:K2219V**	1	0.15				**ORF1a:Q2567Y**	1	0.15	**ORF1a:G3278L**	9	0.25
Maharashtra	**ORF1a:P2221V**	1	0.15				**ORF1a:P2568***	1	0.15	**ORF1a:C3279R**	9	0.25
Maharashtra	**ORF1a:K2225H**	1	0.15				**ORF1a:L2570R**	1	0.15	**ORF1a:M3280V**	9	0.25
Maharashtra	**ORF1a:Y2245***	1	0.15				**ORF1a:L2571H**	1	0.15	**ORF1a:Q3282L**	9	0.25
Maharashtra	**ORF1a:A2248C**	1	0.15				**ORF1a:L2572***	1	0.15	**ORF1a:T3284V**	9	0.25
Maharashtra	**ORF1a:A2249L**	1	0.15				**ORF1a:D2573C**	1	0.15	**ORF1a:C3285Q**	9	0.25
Maharashtra	**ORF1a:G2251T**	1	0.15				**ORF1a:Q2574L**	1	0.15	**ORF1a:G3286L**	9	0.25
Maharashtra	**ORF1a:Y2281L**	1	0.15				**ORF1a:A2575M**	1	0.15	**ORF1a:T3287H**	9	0.25
Maharashtra	**ORF1a:C2282L**	1	0.15				**ORF1a:Y2594I**	1	0.15	**ORF1a:T3288L**	9	0.25
Maharashtra	**ORF1a:T2283M**	1	0.15				**ORF1a:D2627F**	1	0.15	**ORF1a:L3290V**	9	0.25
Maharashtra	**ORF1a:G2284S**	1	0.15				**ORF1a:N2628Q**	1	0.15	**ORF1a:N3291F**	9	0.25
Maharashtra	**ORF1a:S2285L**	1	0.15				**ORF1a:V2629Q**	1	0.15	**ORF1a:P3447Q**	9	0.25
Maharashtra	**ORF1a:I2286L**	1	0.15				**ORF1a:E2653K**	1	0.15	**ORF1a:G3514L**	9	0.25
Maharashtra	**ORF1a:P2287Q**	1	0.15				**ORF1a:C2654G**	1	0.15	**ORF1a:P3515K**	9	0.25
Maharashtra	**ORF1a:C2288P**	1	0.15				**ORF1a:K2656***	1	0.15	**ORF1a:S3517E**	9	0.25
Maharashtra	**ORF1a:S2289T**	1	0.15				**ORF1a:R2686***	1	0.15	**ORF1a:A3518L**	9	0.25
Maharashtra	**ORF1a:C2291L**	1	0.15				**ORF1a:D2687***	1	0.15	**ORF1a:Q3519P**	9	0.25
Maharashtra	**ORF1a:S2293***	1	0.15				**ORF1a:G2689Y**	1	0.15	**ORF1a:T3520F**	9	0.25
Maharashtra	**ORF1a:G2294K**	1	0.15				**ORF1a:C2691H**	1	0.15	**ORF1a:L3585T**	9	0.25
Maharashtra	**ORF1a:D2296Y**	1	0.15				**ORF1a:I2692L**	1	0.15	**ORF1a:E913L**	9	0.25
Maharashtra	**ORF1a:A2320P**	1	0.15				**ORF1a:D2693***	1	0.15	**ORF1a:E3188C**	9	0.25
Maharashtra	**ORF1a:G2322H**	1	0.15				**ORF1a:C2694Q**	1	0.15	**ORF1a:M3189Y**	9	0.25
Maharashtra	**ORF1a:A2330N**	1	0.15				**ORF1a:R2697S**	1	0.15	**ORF1a:C3784L**	9	0.25
Maharashtra	**ORF1a:Y2331G**	1	0.15				**ORF1a:H2698Y**	1	0.15	**ORF1a:G3787R**	9	0.25
Maharashtra	**ORF1a:L2333G**	1	0.15				**ORF1a:I2699***	1	0.15	**ORF1a:Y3788L**	9	0.25
Maharashtra	**ORF1a:I2368Y**	1	0.15				**ORF1a:N2700C**	1	0.15	**ORF1a:C3792L**	9	0.25
Maharashtra	**ORF1a:N2370L**	1	0.15				**ORF1a:Q2702G**	1	0.15	**ORF1a:F3794L**	9	0.25
Maharashtra	**ORF1a:Y2397L**	1	0.15				**ORF1a:V2703S**	1	0.15	**ORF1a:G3795L**	9	0.25
Maharashtra	**ORF1a:V2398H**	1	0.15				**ORF1a:A2704K**	1	0.15	**ORF1a:F3797W**	9	0.25
Maharashtra	**ORF1a:R2417I**	1	0.15				**ORF1a:N2708V**	1	0.15	**ORF1a:C3798P**	9	0.25
Maharashtra	**ORF1a:A2418H**	1	0.15				**ORF1a:N2736V**	1	0.15	**ORF1a:G1723N**	9	0.25
Maharashtra	**ORF1a:T2419Q**	1	0.15				**ORF1a:N2737***	1	0.15	**ORF1a:A2355Y**	9	0.25
Maharashtra	**ORF1a:R2420L**	1	0.15				**ORF1a:L2738T**	1	0.15	**ORF1a:V2356***	9	0.25
Maharashtra	**ORF1a:E2422***	1	0.15				**ORF1a:P2739T**	1	0.15	**ORF1a:H2357***	9	0.25
Maharashtra	**ORF1a:T2424V**	1	0.15				**ORF1a:F2740T**	1	0.15	**ORF1a:N2937I**	9	0.25
Maharashtra	**ORF1a:I2426N**	1	0.15				**ORF1a:W2769***	1	0.15	**ORF1a:V2938L**	9	0.25
Maharashtra	**ORF1a:N2428I**	1	0.15				**ORF1a:A2784E**	1	0.15	**ORF1a:I3322***	9	0.25
Maharashtra	**ORF1a:G2429E**	1	0.15				**ORF1a:I2786Y**	1	0.15	**ORF1a:K3353***	9	0.25
Maharashtra	**ORF1a:V2430Q**	1	0.15				**ORF1a:V2914W**	1	0.15	**ORF1a:N3358S**	9	0.25
Maharashtra	**ORF1a:R2431Q**	1	0.15				**ORF1a:A2916Y**	1	0.15	**ORF1a:G3437W**	9	0.25
Maharashtra	**ORF1a:R2432E**	1	0.15				**ORF1a:A2917H**	1	0.15	**ORF1a:V3467Y**	9	0.25
Maharashtra	**ORF1a:Y2435A**	1	0.15				**ORF1a:E2918I**	1	0.15	**ORF1a:L3468Y**	9	0.25
Maharashtra	**ORF1a:V2436N**	1	0.15				**ORF1a:C2919V**	1	0.15	**ORF1a:A3469S**	9	0.25
Maharashtra	**ORF1a:A2438T**	1	0.15				**ORF1a:T2920M**	1	0.15	**ORF1a:W3470***	9	0.25
Maharashtra	**ORF1a:N2439I**	1	0.15				**ORF1a:D2951M**	1	0.15	**ORF1a:L3471C**	9	0.25
Maharashtra	**ORF1a:D2467V**	1	0.15				**ORF1a:T2952Y**	1	0.15	**ORF1a:Y3472F**	9	0.25
Maharashtra	**ORF1a:E2468L**	1	0.15				**ORF1a:R2953***	1	0.15	**ORF1a:A3473S**	9	0.25
Maharashtra	**ORF1a:A2470V**	1	0.15				**ORF1a:Y2954K**	1	0.15	**ORF1a:A3474L**	9	0.25
Maharashtra	**ORF1a:R2471H**	1	0.15				**ORF1a:T2977G**	1	0.15	**ORF1a:T3489Y**	9	0.25
Maharashtra	**ORF1a:D2472L**	1	0.15				**ORF1a:G2995L**	1	0.15	**ORF1a:L3490H**	9	0.25

Maharashtra	**ORF1a:Q2486L**	1	0.15				**ORF1a:C2997M**	1	0.15	**ORF1a:G3676C**	9	0.25
Maharashtra	**ORF1a:S2487T**	1	0.15				**ORF1a:V2998I**	1	0.15	**ORF1a:L3116F**	9	0.25
Maharashtra	**ORF1a:Y2489L**	1	0.15				**ORF1a:S2999I**	1	0.15	**ORF1a:A690V**	9	0.25
Maharashtra	**ORF1a:G2509L**	1	0.15				**ORF1a:A3023***	1	0.15	**ORF1a:E2049D**	9	0.25
Maharashtra	**ORF1a:K2511L**	1	0.15				**ORF1a:V3024F**	1	0.15	**ORF1a:K3866H**	9	0.25
Maharashtra	**ORF1a:Y2513***	1	0.15				**ORF1a:A3054***	1	0.15	**ORF1a:V3870L**	9	0.25
Maharashtra	**ORF1a:E2514T**	1	0.15				**ORF1a:I3055L**	1	0.15	**ORF1a:V3871T**	9	0.25
Maharashtra	**ORF1a:R2515T**	1	0.15				**ORF1a:T3058E**	1	0.15	**ORF1a:L3873S**	9	0.25
Maharashtra	**ORF1a:H2516***	1	0.15				**ORF1a:C3059L**	1	0.15	**ORF1a:S3874F**	9	0.25
Maharashtra	**ORF1a:S2517E**	1	0.15				**ORF1a:A3061V**	1	0.15	**ORF1a:L3876T**	9	0.25
Maharashtra	**ORF1a:A2557L**	1	0.15				**ORF1a:Y3062N**	1	0.15	**ORF1a:Q3877T**	9	0.25
Maharashtra	**ORF1a:S2558C**	1	0.15				**ORF1a:Y3063T**	1	0.15	**ORF1a:L3879S**	9	0.25
Maharashtra	**ORF1a:Q2563N**	1	0.15				**ORF1a:F3064V**	1	0.15	**ORF1a:C433L**	9	0.25
Maharashtra	**ORF1a:M2565Y**	1	0.15				**ORF1a:V3091***	1	0.15	**ORF1a:N434A**	9	0.25
Maharashtra	**ORF1a:Q2567Y**	1	0.15				**ORF1a:C3093P**	1	0.15	**ORF1a:H435L**	9	0.25
Maharashtra	**ORF1a:P2568***	1	0.15				**ORF1a:T3095I**	1	0.15	**ORF1a:G437***	9	0.25
Maharashtra	**ORF1a:L2570R**	1	0.15				**ORF1a:P3096L**	1	0.15	**ORF1a:R643L**	9	0.25
Maharashtra	**ORF1a:L2571H**	1	0.15				**ORF1a:V3097Y**	1	0.15	**ORF1a:D644N**	9	0.25
Maharashtra	**ORF1a:L2572***	1	0.15				**ORF1a:F3100L**	1	0.15	**ORF1a:G645L**	9	0.25
Maharashtra	**ORF1a:D2573C**	1	0.15				**ORF1a:L3101C**	1	0.15	**ORF1a:W646S**	9	0.25
Maharashtra	**ORF1a:Q2574L**	1	0.15				**ORF1a:P3102H**	1	0.15	**ORF1a:E647Q**	9	0.25
Maharashtra	**ORF1a:A2575M**	1	0.15				**ORF1a:G3103S**	1	0.15	**ORF1a:I648P**	9	0.25
Maharashtra	**ORF1a:Y2594I**	1	0.15				**ORF1a:V3104L**	1	0.15	**ORF1a:S713L**	9	0.25
Maharashtra	**ORF1a:N2596L**	1	0.15				**ORF1a:Y3109L**	1	0.15	**ORF1a:K714S**	9	0.25
Maharashtra	**ORF1a:L2620Y**	1	0.15				**ORF1a:V3130M**	1	0.15	**ORF1a:G715R**	9	0.25
Maharashtra	**ORF1a:A2621Q**	1	0.15				**ORF1a:T3133L**	1	0.15	**ORF1a:K722E**	9	0.25
Maharashtra	**ORF1a:V2624Q**	1	0.15				**ORF1a:P3134F**	1	0.15	**ORF1a:K737R**	9	0.25
Maharashtra	**ORF1a:S2625Q**	1	0.15				**ORF1a:L3135***	1	0.15	**ORF1a:L883W**	9	0.25
Maharashtra	**ORF1a:D2627G**	1	0.15				**ORF1a:V3136H**	1	0.15	**ORF1a:P885M**	9	0.25
Maharashtra	**ORF1a:N2628K**	1	0.15				**ORF1a:P3137I**	1	0.15	**ORF1a:V886L**	9	0.25
Maharashtra	**ORF1a:V2629G**	1	0.15				**ORF1a:W3139S**	1	0.15	**ORF1a:E888***	9	0.25
Maharashtra	**ORF1a:Q2702L**	1	0.15				**ORF1a:T3141G**	1	0.15	**ORF1a:L889K**	9	0.25
Maharashtra	**ORF1a:A2704L**	1	0.15				**ORF1a:I3142S**	1	0.15	**ORF1a:V1222K**	9	0.25
Maharashtra	**ORF1a:R2730I**	1	0.15				**ORF1a:A3143L**	1	0.15	**ORF1a:E1223M**	9	0.25
Maharashtra	**ORF1a:A2732C**	1	0.15				**ORF1a:Y3144V**	1	0.15	**ORF1a:Q1224I**	9	0.25
Maharashtra	**ORF1a:A2733H**	1	0.15				**ORF1a:L3161T**	1	0.15	**ORF1a:T1241S**	9	0.25
Maharashtra	**ORF1a:K2734C**	1	0.15				**ORF1a:K3162***	1	0.15	**ORF1a:T1242K**	9	0.25
Maharashtra	**ORF1a:K2735L**	1	0.15				**ORF1a:R3164D**	1	0.15	**ORF1a:E1244V**	9	0.25
Maharashtra	**ORF1a:P2739D**	1	0.15				**ORF1a:N3168P**	1	0.15	**ORF1a:E1245L**	9	0.25
Maharashtra	**ORF1a:F2740K**	1	0.15				**ORF1a:G3169L**	1	0.15	**ORF1a:T1246K**	9	0.25
Maharashtra	**ORF1a:K2741L**	1	0.15				**ORF1a:N3186K**	1	0.15	**ORF1a:D1273L**	9	0.25
Maharashtra	**ORF1a:T2743M**	1	0.15				**ORF1a:E3188C**	1	0.15	**ORF1a:I1274S**	9	0.25
Maharashtra	**ORF1a:C2744L**	1	0.15				**ORF1a:M3189Y**	1	0.15	**ORF1a:I1276R**	9	0.25
Maharashtra	**ORF1a:A2745***	1	0.15				**ORF1a:Y3211R**	1	0.15	**ORF1a:T1277K**	9	0.25
Maharashtra	**ORF1a:T2746Q**	1	0.15				**ORF1a:K3236Q**	1	0.15	**ORF1a:A1508I**	9	0.25
Maharashtra	**ORF1a:T2747Q**	1	0.15				**ORF1a:A3237V**	1	0.15	**ORF1a:S1510P**	9	0.25
Maharashtra	**ORF1a:Q2749***	1	0.15				**ORF1a:N3239M**	1	0.15	**ORF1a:F1527N**	9	0.25
Maharashtra	**ORF1a:V2750H**	1	0.15				**ORF1a:D3240F**	1	0.15	**ORF1a:M1647Q**	9	0.25
Maharashtra	**ORF1a:V2751L**	1	0.15				**ORF1a:S3242T**	1	0.15	**ORF1a:S1648L**	9	0.25
Maharashtra	**ORF1a:N2752R**	1	0.15				**ORF1a:F3266H**	1	0.15	**ORF1a:V1768H**	9	0.25
Maharashtra	**ORF1a:Q2772L**	1	0.15				**ORF1a:R3267H**	1	0.15	**ORF1a:M1769F**	9	0.25
Maharashtra	**ORF1a:V2776L**	1	0.15				**ORF1a:M3269P**	1	0.15	**ORF1a:Y1770L**	9	0.25
Maharashtra	**ORF1a:T2777H**	1	0.15				**ORF1a:A3270L**	1	0.15	**ORF1a:G1772N**	9	0.25
Maharashtra	**ORF1a:V2779***	1	0.15				**ORF1a:F3271S**	1	0.15	**ORF1a:T1773N**	9	0.25
Maharashtra	**ORF1a:F2780H**	1	0.15				**ORF1a:G3274H**	1	0.15	**ORF1a:T1881S**	9	0.25
Maharashtra	**ORF1a:H2799S**	1	0.15				**ORF1a:K3275L**	1	0.15	**ORF1a:K1883L**	9	0.25
Maharashtra	**ORF1a:T2800***	1	0.15				**ORF1a:E3277K**	1	0.15	**ORF1a:L1884***	9	0.25
Maharashtra	**ORF1a:D2819L**	1	0.15				**ORF1a:G3278L**	1	0.15	**ORF1a:D1885I**	9	0.25
Maharashtra	**ORF1a:Y2845L**	1	0.15				**ORF1a:C3279R**	1	0.15	**ORF1a:V1887W**	9	0.25
Maharashtra	**ORF1a:N2847H**	1	0.15				**ORF1a:M3280V**	1	0.15	**ORF1a:V1888C**	9	0.25
Maharashtra	**ORF1a:D2848G**	1	0.15				**ORF1a:Q3282L**	1	0.15	**ORF1a:K1901R**	9	0.25
Maharashtra	**ORF1a:K2849L**	1	0.15				**ORF1a:T3284V**	1	0.15	**ORF1a:K1902A**	9	0.25
Maharashtra	**ORF1a:C2851W**	1	0.15				**ORF1a:C3285Q**	1	0.15	**ORF1a:S1905***	9	0.25
Maharashtra	**ORF1a:P2852V**	1	0.15				**ORF1a:G3286L**	1	0.15	**ORF1a:Y1906S**	9	0.25
Maharashtra	**ORF1a:D2880S**	1	0.15				**ORF1a:T3287H**	1	0.15	**ORF1a:F1907C**	9	0.25
Maharashtra	**ORF1a:F2881C**	1	0.15				**ORF1a:T3288L**	1	0.15	**ORF1a:P1911T**	9	0.25
Maharashtra	**ORF1a:H2883V**	1	0.15				**ORF1a:L3290V**	1	0.15	**ORF1a:F1928S**	9	0.25
Maharashtra	**ORF1a:F2884C**	1	0.15				**ORF1a:N3291F**	1	0.15	**ORF1a:D2717Q**	9	0.25
Maharashtra	**ORF1a:P2886A**	1	0.15				**ORF1a:C3307M**	1	0.15	**ORF1a:F2718H**	9	0.25
Maharashtra	**ORF1a:F2889T**	1	0.15				**ORF1a:N3335F**	1	0.15	**ORF1a:M2719C**	9	0.25
Maharashtra	**ORF1a:A2909V**	1	0.15				**ORF1a:Q3337S**	1	0.15	**ORF1a:S2720F**	9	0.25
Maharashtra	**ORF1a:S2911H**	1	0.15				**ORF1a:R3368L**	1	0.15	**ORF1a:L2721D**	9	0.25
Maharashtra	**ORF1a:A2912H**	1	0.15				**ORF1a:G3372F**	1	0.15	**ORF1a:S2722M**	9	0.25

Maharashtra	**ORF1a:C2913Q**	1	0.15				**ORF1a:T3374C**	1	0.15	**ORF1a:Q2724R**	9	0.25
Maharashtra	**ORF1a:V2914N**	1	0.15				**ORF1a:F3375***	1	0.15	**ORF1a:L2725***	9	0.25
Maharashtra	**ORF1a:A2916***	1	0.15				**ORF1a:S3376L**	1	0.15	**ORF1a:I3737R**	9	0.25
Maharashtra	**ORF1a:A2917S**	1	0.15				**ORF1a:A3379V**	1	0.15	**ORF1a:A1793V**	9	0.25
Maharashtra	**ORF1a:E2918T**	1	0.15				**ORF1a:C3380F**	1	0.15	**ORF1a:K1795R**	9	0.25
Maharashtra	**ORF1a:C2919L**	1	0.15				**ORF1a:Y3381T**	1	0.15	**ORF1a:H1838V**	9	0.25
Maharashtra	**ORF1a:S2947V**	1	0.15				**ORF1a:F3403V**	1	0.15	**ORF1a:K1842V**	9	0.25
Maharashtra	**ORF1a:L2948M**	1	0.15				**ORF1a:N3405***	1	0.15	**ORF1a:E1843V**	9	0.25
Maharashtra	**ORF1a:R2949I**	1	0.15				**ORF1a:H3435L**	1	0.15	**ORF1a:I1877K**	9	0.25
Maharashtra	**ORF1a:D2951M**	1	0.15				**ORF1a:A3436F**	1	0.15	**ORF1a:P1879T**	9	0.25
Maharashtra	**ORF1a:T2952Y**	1	0.15				**ORF1a:G3437V**	1	0.15	**ORF1a:Y1882Q**	9	0.25
Maharashtra	**ORF1a:R2953***	1	0.15				**ORF1a:D3439C**	1	0.15	**ORF1a:Q1943L**	9	0.25
Maharashtra	**ORF1a:Y2954K**	1	0.15				**ORF1a:L3440T**	1	0.15	**ORF1a:T1945Q**	9	0.25
Maharashtra	**ORF1a:E2993T**	1	0.15				**ORF1a:E3441I**	1	0.15	**ORF1a:G1946E**	9	0.25
Maharashtra	**ORF1a:A2994V**	1	0.15				**ORF1a:G3442W**	1	0.15	**ORF1a:Y1947S**	9	0.25
Maharashtra	**ORF1a:C2997F**	1	0.15				**ORF1a:F3444Y**	1	0.15	**ORF1a:K1948L**	9	0.25
Maharashtra	**ORF1a:S3013Y**	1	0.15				**ORF1a:Y3445Q**	1	0.15	**ORF1a:K1981T**	9	0.25
Maharashtra	**ORF1a:P3015L**	1	0.15				**ORF1a:G3446L**	1	0.15	**ORF1a:G1982T**	9	0.25
Maharashtra	**ORF1a:G3016V**	1	0.15				**ORF1a:P3447E**	1	0.15	**ORF1a:A1983H**	9	0.25
Maharashtra	**ORF1a:F3018I**	1	0.15				**ORF1a:V3449M**	1	0.15	**ORF1a:H2092***	9	0.25
Maharashtra	**ORF1a:G3020L**	1	0.15				**ORF1a:D3450L**	1	0.15	**ORF1a:K2901***	9	0.25
Maharashtra	**ORF1a:V3021H**	1	0.15				**ORF1a:R3451A**	1	0.15	**ORF1a:A1851V**	9	0.25
Maharashtra	**ORF1a:D3022H**	1	0.15				**ORF1a:A3454***	1	0.15	**ORF1a:I3142K**	9	0.25
Maharashtra	**ORF1a:A3023***	1	0.15				**ORF1a:A3456V**	1	0.15	**ORF1a:R3163F**	9	0.25
Maharashtra	**ORF1a:V3024F**	1	0.15				**ORF1a:A3457T**	1	0.15	**ORF1a:V3165L**	9	0.25
Maharashtra	**ORF1a:A3054***	1	0.15				**ORF1a:G3458F**	1	0.15	**ORF1a:F3167L**	9	0.25
Maharashtra	**ORF1a:I3055L**	1	0.15				**ORF1a:T3459M**	1	0.15	**ORF1a:N3168***	9	0.25
Maharashtra	**ORF1a:T3058E**	1	0.15				**ORF1a:T3461L**	1	0.15	**ORF1a:G3169***	9	0.25
Maharashtra	**ORF1a:C3059L**	1	0.15				**ORF1a:T3462L**	1	0.15	**ORF1a:V3170L**	9	0.25
Maharashtra	**ORF1a:A3061V**	1	0.15				**ORF1a:I3463L**	1	0.15	**ORF1a:S3171P**	9	0.25
Maharashtra	**ORF1a:Y3062N**	1	0.15				**ORF1a:V3465Q**	1	0.15	**ORF1a:F3172K**	9	0.25
Maharashtra	**ORF1a:Y3063T**	1	0.15				**ORF1a:N3466V**	1	0.15	**ORF1a:S3173E**	9	0.25
Maharashtra	**ORF1a:F3064V**	1	0.15				**ORF1a:L3468S**	1	0.15	**ORF1a:T3058I**	9	0.25
Maharashtra	**ORF1a:V3091***	1	0.15				**ORF1a:A3469Q**	1	0.15	**ORF1a:V3653F**	9	0.25
Maharashtra	**ORF1a:C3093P**	1	0.15				**ORF1a:W3470S**	1	0.15	**ORF1a:E1413D**	9	0.25
Maharashtra	**ORF1a:T3095I**	1	0.15				**ORF1a:L3471I**	1	0.15	**ORF1a:V1056L**	9	0.25
Maharashtra	**ORF1a:P3096L**	1	0.15				**ORF1a:L3490H**	1	0.15	**ORF1a:V2398H**	8	0.23
Maharashtra	**ORF1a:V3097Y**	1	0.15				**ORF1a:D3492S**	1	0.15	**ORF1a:G2534***	8	0.23
Maharashtra	**ORF1a:F3100L**	1	0.15				**ORF1a:F3493***	1	0.15	**ORF1a:S2535F**	8	0.23
Maharashtra	**ORF1a:L3101C**	1	0.15				**ORF1a:N3494***	1	0.15	**ORF1a:P2537M**	8	0.23
Maharashtra	**ORF1a:P3102H**	1	0.15				**ORF1a:V3496***	1	0.15	**ORF1a:I2538V**	8	0.23
Maharashtra	**ORF1a:G3103S**	1	0.15				**ORF1a:A3497P**	1	0.15	**ORF1a:V2540Q**	8	0.23
Maharashtra	**ORF1a:V3104L**	1	0.15				**ORF1a:M3498C**	1	0.15	**ORF1a:I2541N**	8	0.23
Maharashtra	**ORF1a:Y3109L**	1	0.15				**ORF1a:K3499G**	1	0.15	**ORF1a:A2710L**	8	0.23
Maharashtra	**ORF1a:V3130M**	1	0.15				**ORF1a:S3517K**	1	0.15	**ORF1a:L2711M**	8	0.23
Maharashtra	**ORF1a:T3133Q**	1	0.15				**ORF1a:A3518R**	1	0.15	**ORF1a:I2712R**	8	0.23
Maharashtra	**ORF1a:P3134L**	1	0.15				**ORF1a:Q3519I**	1	0.15	**ORF1a:W2713R**	8	0.23
Maharashtra	**ORF1a:K3162***	1	0.15				**ORF1a:L3549M**	1	0.15	**ORF1a:N2714***	8	0.23
Maharashtra	**ORF1a:R3164D**	1	0.15				**ORF1a:E3551R**	1	0.15	**ORF1a:V2715Q**	8	0.23
Maharashtra	**ORF1a:N3168P**	1	0.15				**ORF1a:D3552C**	1	0.15	**ORF1a:D2717V**	8	0.23
Maharashtra	**ORF1a:G3169L**	1	0.15				**ORF1a:E3553Y**	1	0.15	**ORF1a:F2718T**	8	0.23
Maharashtra	**ORF1a:N3186K**	1	0.15				**ORF1a:R3561P**	1	0.15	**ORF1a:M2719T**	8	0.23
Maharashtra	**ORF1a:E3188K**	1	0.15				**ORF1a:Q3562K**	1	0.15	**ORF1a:S2720L**	8	0.23
Maharashtra	**ORF1a:M3189***	1	0.15				**ORF1a:G3565E**	1	0.15	**ORF1a:S2722Y**	8	0.23
Maharashtra	**ORF1a:I3476***	1	0.15				**ORF1a:V3566K**	1	0.15	**ORF1a:E2723V**	8	0.23
Maharashtra	**ORF1a:N3477L**	1	0.15				**ORF1a:T3567N**	1	0.15	**ORF1a:Q2724V**	8	0.23
Maharashtra	**ORF1a:D3479C**	1	0.15				**ORF1a:F3568N**	1	0.15	**ORF1a:E2940L**	8	0.23
Maharashtra	**ORF1a:R3480T**	1	0.15				**ORF1a:K737I**	1	0.15	**ORF1a:G2941M**	8	0.23
Maharashtra	**ORF1a:W3481L**	1	0.15				**ORF1a:T2277H**	1	0.15	**ORF1a:S2942K**	8	0.23
Maharashtra	**ORF1a:F3482L**	1	0.15				**ORF1a:K3886R**	1	0.15	**ORF1a:V2955A**	8	0.23
Maharashtra	**ORF1a:N3484***	1	0.15				**ORF1a:L451F**	1	0.15	**ORF1a:T3095Y**	8	0.23
Maharashtra	**ORF1a:R3485M**	1	0.15				**ORF1a:I2346N**	1	0.15	**ORF1a:P3096S**	8	0.23
Maharashtra	**ORF1a:F3486E**	1	0.15				**ORF1a:M2347T**	1	0.15	**ORF1a:Y3098***	8	0.23
Maharashtra	**ORF1a:T3489I**	1	0.15				**ORF1a:S1924I**	1	0.15	**ORF1a:S3099H**	8	0.23
Maharashtra	**ORF1a:L3490M**	1	0.15				**ORF1a:L628F**	1	0.15	**ORF1a:F3100Q**	8	0.23
Maharashtra	**ORF1a:S3517***	1	0.15				**ORF1a:A1702V**	1	0.15	**ORF1a:L3101F**	8	0.23
Maharashtra	**ORF1a:A3518H**	1	0.15				**ORF1a:G1705D**	1	0.15	**ORF1a:P3102T**	8	0.23
Maharashtra	**ORF1a:Q3519K**	1	0.15				**ORF1a:V3197L**	1	0.15	**ORF1a:G3103H**	8	0.23
Maharashtra	**ORF1a:G3521M**	1	0.15				**ORF1a:N4256D**	1	0.15	**ORF1a:V3104S**	8	0.23
Maharashtra	**ORF1a:I3522L**	1	0.15				**ORF1a:L477S**	1	0.15	**ORF1a:S3106L**	8	0.23
Maharashtra	**ORF1a:V3524Y**	1	0.15				**ORF1a:G524Y**	1	0.15	**ORF1a:I3108F**	8	0.23
Maharashtra	**ORF1a:L3525***	1	0.15				**ORF1a:E525D**	1	0.15	**ORF1a:Y3109I**	8	0.23
Maharashtra	**ORF1a:M3527L**	1	0.15				**ORF1a:S2220T**	1	0.15	**ORF1a:Y3111L**	8	0.23

Maharashtra	**ORF1a:C3528F**	1	0.15				**ORF1a:P2221L**	1	0.15	**ORF1a:L3112F**	8	0.23
Maharashtra	**ORF1a:A3529L**	1	0.15				**ORF1a:I2227V**	1	0.15	**ORF1a:T3133Q**	8	0.23
Maharashtra	**ORF1a:S3530L**	1	0.15				**ORF1a:N2228K**	1	0.15	**ORF1a:P3134L**	8	0.23
Maharashtra	**ORF1a:L3531K**	1	0.15				**ORF1a:I2229T**	1	0.15	**ORF1a:P3200***	8	0.23
Maharashtra	**ORF1a:K3532L**	1	0.15				**ORF1a:I2230K**	1	0.15	**ORF1a:P3272H**	8	0.23
Maharashtra	**ORF1a:Q3536Y**	1	0.15				**ORF1a:C2263N**	1	0.15	**ORF1a:H3304M**	8	0.23
Maharashtra	**ORF1a:N3537***	1	0.15				**ORF1a:T2264I**	1	0.15	**ORF1a:V3305T**	8	0.23
Maharashtra	**ORF1a:G3538K**	1	0.15				**ORF1a:G2265Y**	1	0.15	**ORF1a:I3306***	8	0.23
Maharashtra	**ORF1a:F3568E**	1	0.15				**ORF1a:Y2266H**	1	0.15	**ORF1a:C3307F**	8	0.23
Maharashtra	**ORF1a:A3571R**	1	0.15				**ORF1a:I1785T**	1	0.15	**ORF1a:S3309V**	8	0.23
Maharashtra	**ORF1a:K3573H**	1	0.15				**ORF1a:F1867L**	1	0.15	**ORF1a:E3310Q**	8	0.23
Maharashtra	**ORF1a:R3574T**	1	0.15				**ORF1a:N3484K**	1	0.15	**ORF1a:P3371S**	8	0.23
Maharashtra	**ORF1a:I3576G**	1	0.15				**ORF1a:A702T**	1	0.15	**ORF1a:D3526H**	8	0.23
Maharashtra	**ORF1a:K3577C**	1	0.15				**ORF1a:L729Q**	1	0.15	**ORF1a:M3527***	8	0.23
Maharashtra	**ORF1a:G3578Y**	1	0.15				**ORF1a:M3655I**	1	0.15	**ORF1a:C3528K**	8	0.23
Maharashtra	**ORF1a:T3579S**	1	0.15				**ORF1a:G519Y**	1	0.15	**ORF1a:A3529N**	8	0.23
Maharashtra	**ORF1a:H3581F**	1	0.15				**ORF1a:A520D**	1	0.15	**ORF1a:S3530Y**	8	0.23
Maharashtra	**ORF1a:W3582***	1	0.15				**ORF1a:I476Y**	1	0.15	**ORF1a:L3531C**	8	0.23
Maharashtra	**ORF1a:L3584V**	1	0.15				**ORF1a:Y3788***	1	0.15	**ORF1a:L3584H**	8	0.23
Maharashtra	**ORF1a:T3586N**	1	0.15				**ORF1a:I3146S**	1	0.15	**ORF1a:L3588C**	8	0.23
Maharashtra	**ORF1a:I3587G**	1	0.15				**ORF1a:V23I**	1	0.15	**ORF1a:T3589Y**	8	0.23
Maharashtra	**ORF1a:T3589C**	1	0.15				**ORF1a:A2001V**	1	0.15	**ORF1a:L3591Q**	8	0.23
Maharashtra	**ORF1a:P3613F**	1	0.15				**ORF1a:S2246P**	1	0.15	**ORF1a:L3592F**	8	0.23
Maharashtra	**ORF1a:A3615C**	1	0.15				**ORF1a:V2324I**	1	0.15	**ORF1a:V3593***	8	0.23
Maharashtra	**ORF1a:G3617S**	1	0.15				**ORF1a:A1151L**	1	0.15	**ORF1a:Q3596L**	8	0.23
Maharashtra	**ORF1a:I3618N**	1	0.15				**ORF1a:G1152C**	1	0.15	**ORF1a:S3597N**	8	0.23
Maharashtra	**ORF1a:A3620S**	1	0.15				**ORF1a:D1939***	1	0.15	**ORF1a:T3598G**	8	0.23
Maharashtra	**ORF1a:K3632F**	1	0.15				**ORF1a:D1940***	1	0.15	**ORF1a:Q3599L**	8	0.23
Maharashtra	**ORF1a:H3633S**	1	0.15				**ORF1a:G1946C**	1	0.15	**ORF1a:W3600C**	8	0.23
Maharashtra	**ORF1a:A3634V**	1	0.15				**ORF1a:A3615G**	1	0.15	**ORF1a:S212L**	8	0.23
Maharashtra	**ORF1a:F3635C**	1	0.15				**ORF1a:M3616L**	1	0.15	**ORF1a:K225L**	8	0.23
Maharashtra	**ORF1a:L3636F**	1	0.15				**ORF1a:G2265T**	1	0.15	**ORF1a:D335I**	8	0.23
Maharashtra	**ORF1a:L3638Y**	1	0.15				**ORF1a:Y2266D**	1	0.15	**ORF1a:I3768Y**	8	0.23
Maharashtra	**ORF1a:F3639L**	1	0.15				**ORF1a:V1299L**	1	0.15	**ORF1a:R3805T**	8	0.23
Maharashtra	**ORF1a:M3664W**	1	0.15				**ORF1a:R3451M**	1	0.15	**ORF1a:L3806Q**	8	0.23
Maharashtra	**ORF1a:T3665I**	1	0.15				**ORF1a:G1723S**	1	0.15	**ORF1a:T3807P**	8	0.23
Maharashtra	**ORF1a:D3668I**	1	0.15				**ORF1a:L494F**	1	0.15	**ORF1a:S4112T**	8	0.23
Maharashtra	**ORF1a:M3669L**	1	0.15				**ORF1a:F2358V**	1	0.15	**ORF1a:E4113***	8	0.23
Maharashtra	**ORF1a:D3671C**	1	0.15				**ORF1a:M731T**	1	0.15	**ORF1a:I4114F**	8	0.23
Maharashtra	**ORF1a:T3672L**	1	0.15				**ORF1a:C2239F**	1	0.15	**ORF1a:L4131S**	8	0.23
Maharashtra	**ORF1a:S3673V**	1	0.15				**ORF1a:L2240T**	1	0.15	**ORF1a:R4132Y**	8	0.23
Maharashtra	**ORF1a:S3687***	1	0.15				**ORF1a:G2241E**	1	0.15	**ORF1a:A4133C**	8	0.23
Maharashtra	**ORF1a:A3688K**	1	0.15				**ORF1a:E1293G**	1	0.15	**ORF1a:A4155C**	8	0.23
Maharashtra	**ORF1a:V3689T**	1	0.15				**ORF1a:F8I**	1	0.15	**ORF1a:S1361R**	8	0.23
Maharashtra	**ORF1a:R3707S**	1	0.15				**ORF1a:T78Y**	1	0.15	**ORF1a:T1773C**	8	0.23
Maharashtra	**ORF1a:W3709D**	1	0.15				**ORF1a:E2053G**	1	0.15	**ORF1a:L1774R**	8	0.23
Maharashtra	**ORF1a:L3711Y**	1	0.15				**ORF1a:V1199M**	1	0.15	**ORF1a:E1340D**	8	0.23
Maharashtra	**ORF1a:M3712E**	1	0.15				**ORF1a:E1200K**	1	0.15	**ORF1a:V721I**	8	0.23
Maharashtra	**ORF1a:N3713C**	1	0.15				**ORF1a:V2203F**	1	0.15	**ORF1a:G2971Y**	8	0.23
Maharashtra	**ORF1a:V3714L**	1	0.15				**ORF1a:A4136V**	1	0.15	**ORF1a:Y3811***	8	0.23
Maharashtra	**ORF1a:L3715D**	1	0.15				**ORF1a:M2374I**	1	0.15	**ORF1a:D3812I**	8	0.23
Maharashtra	**ORF1a:V3747Y**	1	0.15				**ORF1a:G1119C**	1	0.15	**ORF1a:M3862R**	8	0.23
Maharashtra	**ORF1a:T3749C**	1	0.15				**ORF1a:G49D**	1	0.15	**ORF1a:S3863C**	8	0.23
Maharashtra	**ORF1a:T3750***	1	0.15				**ORF1a:I1128T**	1	0.15	**ORF1a:D3864K**	8	0.23
Maharashtra	**ORF1a:C3766L**	1	0.15				**ORF1a:A2637T**	1	0.15	**ORF1a:C3867I**	8	0.23
Maharashtra	**ORF1a:I3768Y**	1	0.15				**ORF1a:S4245N**	1	0.15	**ORF1a:T3868S**	8	0.23
Maharashtra	**ORF1a:C3784L**	1	0.15				**ORF1a:V4369I**	1	0.15	**ORF1a:E377I**	8	0.23
Maharashtra	**ORF1a:G3787R**	1	0.15				**ORF1a:S2246L**	1	0.15	**ORF1a:G379L**	8	0.23
Maharashtra	**ORF1a:Y3788L**	1	0.15				**ORF1a:M2365I**	1	0.15	**ORF1a:K394N**	8	0.23
Maharashtra	**ORF1a:C3792L**	1	0.15				**ORF1a:G137C**	1	0.15	**ORF1a:G408E**	8	0.23
Maharashtra	**ORF1a:F3794L**	1	0.15				**ORF1a:A1473V**	1	0.15	**ORF1a:C409A**	8	0.23
Maharashtra	**ORF1a:G3795L**	1	0.15				**ORF1a:T2967I**	1	0.15	**ORF1a:F411C**	8	0.23
Maharashtra	**ORF1a:F3797W**	1	0.15				**ORF1a:E472D**	1	0.15	**ORF1a:Y422M**	8	0.23
Maharashtra	**ORF1a:C3798P**	1	0.15				**ORF1a:A1043V**	1	0.15	**ORF1a:W423L**	8	0.23
Maharashtra	**ORF1a:R402C**	1	0.15				**ORF1a:Q2034R**	1	0.15	**ORF1a:P425A**	8	0.23
Maharashtra	**ORF1a:T891I**	1	0.15				**ORF1a:L1110F**	1	0.15	**ORF1a:R426I**	8	0.23
Maharashtra	**ORF1a:P2483H**	1	0.15				**ORF1a:T1682I**	1	0.15	**ORF1a:A427T**	8	0.23
Maharashtra	**ORF1a:V2613F**	1	0.15				**ORF1a:K2928N**	1	0.15	**ORF1a:A429V**	8	0.23
Maharashtra	**ORF1a:E933G**	1	0.15				**ORF1a:T882I**	1	0.15	**ORF1a:N430P**	8	0.23
Maharashtra	**ORF1a:G706C**	1	0.15				**ORF1a:P1810S**	1	0.15	**ORF1a:T677I**	8	0.23
Maharashtra	**ORF1a:Q768R**	1	0.15				**ORF1a:I3576T**	1	0.15	**ORF1a:F678N**	8	0.23
Maharashtra	**ORF1a:M3527I**	1	0.15				**ORF1a:N1042S**	1	0.15	**ORF1a:K680W**	8	0.23
Maharashtra	**ORF1a:M4241V**	1	0.15				**ORF1a:R2471G**	1	0.15	**ORF1a:V682C**	8	0.23

Maharashtra	**ORF1a:K714N**	1	0.15				**ORF1a:P1640H**	1	0.15	**ORF1a:N683V**	8	0.23
Maharashtra	**ORF1a:V751L**	1	0.15				**ORF1a:E553L**	1	0.15	**ORF1a:K684L**	8	0.23
Maharashtra	**ORF1a:K3353R**	1	0.15				**ORF1a:T554Y**	1	0.15	**ORF1a:F685T**	8	0.23
Maharashtra	**ORF1a:T1678I**	1	0.15				**ORF1a:A555D**	1	0.15	**ORF1a:A687S**	8	0.23
Maharashtra	**ORF1a:M2036T**	1	0.15				**ORF1a:N557F**	1	0.15	**ORF1a:K701V**	8	0.23
Maharashtra	**ORF1a:T2121P**	1	0.15				**ORF1a:V559R**	1	0.15	**ORF1a:A702E**	8	0.23
Maharashtra	**ORF1a:Y3389***	1	0.15				**ORF1a:V561P**	1	0.15	**ORF1a:G706P**	8	0.23
Maharashtra	**ORF1a:S4093P**	1	0.15				**ORF1a:S574***	1	0.15	**ORF1a:E707***	8	0.23
Maharashtra	**ORF1a:L4185V**	1	0.15				**ORF1a:R579H**	1	0.15	**ORF1a:T708I**	8	0.23
Maharashtra	**ORF1a:S4398T**	1	0.15				**ORF1a:D589W**	1	0.15	**ORF1a:F709***	8	0.23
Maharashtra	**ORF1a:K1655N**	1	0.15				**ORF1a:A591L**	1	0.15	**ORF1a:T711K**	8	0.23
Maharashtra	**ORF1a:A2587S**	1	0.15				**ORF1a:N593I**	1	0.15	**ORF1a:L716T**	8	0.23
Maharashtra	**ORF1a:K1230N**	1	0.15				**ORF1a:N594***	1	0.15	**ORF1a:Y717Q**	8	0.23
Maharashtra	**ORF1a:C191F**	1	0.15				**ORF1a:A599***	1	0.15	**ORF1a:K719D**	8	0.23
Maharashtra	**ORF1a:A1679T**	1	0.15				**ORF1a:Y600W**	1	0.15	**ORF1a:V721T**	8	0.23
Maharashtra	**ORF1a:I2190S**	1	0.15				**ORF1a:I601P**	1	0.15	**ORF1a:L729V**	8	0.23
Maharashtra	**ORF1a:A2199P**	1	0.15				**ORF1a:G603L**	1	0.15	**ORF1a:M731N**	8	0.23
Maharashtra	**ORF1a:K2200N**	1	0.15				**ORF1a:G604Q**	1	0.15	**ORF1a:K734S**	8	0.23
Maharashtra	**ORF1a:N2201Y**	1	0.15				**ORF1a:Q607***	1	0.15	**ORF1a:P736***	8	0.23
Maharashtra	**ORF1a:T2202N**	1	0.15				**ORF1a:K625N**	1	0.15	**ORF1a:K851S**	8	0.23
Maharashtra	**ORF1a:V2203I**	1	0.15				**ORF1a:V627S**	1	0.15	**ORF1a:V852L**	8	0.23
Maharashtra	**ORF1a:K2204Y**	1	0.15				**ORF1a:D629I**	1	0.15	**ORF1a:K856M**	8	0.23
Maharashtra	**ORF1a:S2205Q**	1	0.15				**ORF1a:W630G**	1	0.15	**ORF1a:C857R**	8	0.23
Maharashtra	**ORF1a:V2206F**	1	0.15				**ORF1a:E632V**	1	0.15	**ORF1a:N1109M**	8	0.23
Maharashtra	**ORF1a:G2207R**	1	0.15				**ORF1a:E633***	1	0.15	**ORF1a:F1214R**	8	0.23
Maharashtra	**ORF1a:F2209I**	1	0.15				**ORF1a:K634S**	1	0.15	**ORF1a:I1215F**	8	0.23
Maharashtra	**ORF1a:E2212V**	1	0.15				**ORF1a:K636L**	1	0.15	**ORF1a:T1216L**	8	0.23
Maharashtra	**ORF1a:A2213T**	1	0.15				**ORF1a:G638T**	1	0.15	**ORF1a:E1217K**	8	0.23
Maharashtra	**ORF1a:F2215N**	1	0.15				**ORF1a:E640G**	1	0.15	**ORF1a:M1312A**	8	0.23
Maharashtra	**ORF1a:S2220***	1	0.15				**ORF1a:F641K**	1	0.15	**ORF1a:E1909K**	8	0.23
Maharashtra	**ORF1a:P2221E**	1	0.15				**ORF1a:R643L**	1	0.15	**ORF1a:Q1910P**	8	0.23
Maharashtra	**ORF1a:N2222G**	1	0.15				**ORF1a:D644N**	1	0.15	**ORF1a:K1988S**	8	0.23
Maharashtra	**ORF1a:F2223M**	1	0.15				**ORF1a:G645L**	1	0.15	**ORF1a:P2018S**	8	0.23
Maharashtra	**ORF1a:S2224P**	1	0.15				**ORF1a:W646S**	1	0.15	**ORF1a:G2294H**	8	0.23
Maharashtra	**ORF1a:N2228D**	1	0.15				**ORF1a:E647Q**	1	0.15	**ORF1a:R2432M**	8	0.23
Maharashtra	**ORF1a:I2230R**	1	0.15				**ORF1a:I648P**	1	0.15	**ORF1a:S2433Y**	8	0.23
Maharashtra	**ORF1a:I2231T**	1	0.15				**ORF1a:I664L**	1	0.15	**ORF1a:F2434N**	8	0.23
Maharashtra	**ORF1a:T3741I**	1	0.15				**ORF1a:T666K**	1	0.15	**ORF1a:M3312H**	8	0.23
Maharashtra	**ORF1a:N3833T**	1	0.15				**ORF1a:C667L**	1	0.15	**ORF1a:N3314***	8	0.23
Maharashtra	**ORF1a:S3834P**	1	0.15				**ORF1a:A668S**	1	0.15	**ORF1a:P3315R**	8	0.23
Maharashtra	**ORF1a:I3835V**	1	0.15				**ORF1a:K669V**	1	0.15	**ORF1a:N3316H**	8	0.23
Maharashtra	**ORF1a:L729Q**	1	0.15				**ORF1a:E670D**	1	0.15	**ORF1a:Y3317A**	8	0.23
Maharashtra	**ORF1a:G1333A**	1	0.15				**ORF1a:I671K**	1	0.15	**ORF1a:E3318***	8	0.23
Maharashtra	**ORF1a:Q1593P**	1	0.15				**ORF1a:K672L**	1	0.15	**ORF1a:D3319P**	8	0.23
Maharashtra	**ORF1a:F1594S**	1	0.15				**ORF1a:E673S**	1	0.15	**ORF1a:L3320***	8	0.23
Maharashtra	**ORF1a:G1595C**	1	0.15				**ORF1a:S674P**	1	0.15	**ORF1a:K1791R**	8	0.23
Maharashtra	**ORF1a:M1771K**	1	0.15				**ORF1a:T677I**	1	0.15	**ORF1a:Q1792K**	8	0.23
Maharashtra	**ORF1a:V2469D**	1	0.15				**ORF1a:F678N**	1	0.15	**ORF1a:N1904L**	8	0.23
Maharashtra	**ORF1a:F2641L**	1	0.15				**ORF1a:K680W**	1	0.15	**ORF1a:F1907Q**	8	0.23
Maharashtra	**ORF1a:I2812N**	1	0.15				**ORF1a:V682C**	1	0.15	**ORF1a:G2118L**	8	0.23
Maharashtra	**ORF1a:M3131R**	1	0.15				**ORF1a:N683V**	1	0.15	**ORF1a:V2149G**	8	0.23
Maharashtra	**ORF1a:R3480M**	1	0.15				**ORF1a:K684L**	1	0.15	**ORF1a:S2151***	8	0.23
Maharashtra	**ORF1a:T2967I**	1	0.15				**ORF1a:F685T**	1	0.15	**ORF1a:F2172V**	8	0.23
Maharashtra	**ORF1a:L1853F**	1	0.15				**ORF1a:A687S**	1	0.15	**ORF1a:I3738C**	8	0.23
Maharashtra	**ORF1a:T2274I**	1	0.15				**ORF1a:K701V**	1	0.15	**ORF1a:P3359S**	8	0.23
Maharashtra	**ORF1a:M920I**	1	0.15				**ORF1a:A702E**	1	0.15	**ORF1a:P1054F**	8	0.23
Maharashtra	**ORF1a:P2221S**	1	0.15				**ORF1a:G706P**	1	0.15	**ORF1a:L1599M**	8	0.23
Maharashtra	**ORF1a:K4039R**	1	0.15				**ORF1a:E707***	1	0.15	**ORF1a:Y1868R**	8	0.23
Maharashtra	**ORF1a:V2019F**	1	0.15				**ORF1a:T708I**	1	0.15	**ORF1a:K1869I**	8	0.23
Maharashtra	**ORF1a:T999I**	1	0.15				**ORF1a:F709***	1	0.15	**ORF1a:E1870Q**	8	0.23
Maharashtra							**ORF1a:T711K**	1	0.15	**ORF1a:N1871R**	8	0.23
Maharashtra							**ORF1a:K714S**	1	0.15	**ORF1a:T2106S**	8	0.23
Maharashtra							**ORF1a:G715R**	1	0.15	**ORF1a:A2128R**	8	0.23
Maharashtra							**ORF1a:L716T**	1	0.15	**ORF1a:A2129F**	8	0.23
Maharashtra							**ORF1a:Y717Q**	1	0.15	**ORF1a:V2130E**	8	0.23
Maharashtra							**ORF1a:K719D**	1	0.15	**ORF1a:T2898R**	8	0.23
Maharashtra							**ORF1a:V721T**	1	0.15	**ORF1a:P2899V**	8	0.23
Maharashtra							**ORF1a:K722E**	1	0.15	**ORF1a:H3343C**	8	0.23
Maharashtra							**ORF1a:L729V**	1	0.15	**ORF1a:F3639L**	8	0.23
Maharashtra							**ORF1a:M731N**	1	0.15	**ORF1a:M1647V**	8	0.23
Maharashtra							**ORF1a:K734S**	1	0.15	**ORF1a:T512A**	8	0.23
Maharashtra							**ORF1a:P736***	1	0.15	**ORF1a:K2849R**	8	0.23
Maharashtra							**ORF1a:K737R**	1	0.15	**ORF1a:A1043V**	8	0.23

Maharashtra							**ORF1a:I739K**	1	0.15	**ORF1a:D1962Y**	8	0.23
Maharashtra							**ORF1a:I740H**	1	0.15	**ORF1a:K669N**	8	0.23
Maharashtra							**ORF1a:V757P**	1	0.15	**ORF1a:N2498L**	7	0.2
Maharashtra							**ORF1a:L758Q**	1	0.15	**ORF1a:S2517I**	7	0.2
Maharashtra							**ORF1a:T760N**	1	0.15	**ORF1a:D2525I**	7	0.2
Maharashtra							**ORF1a:G761N**	1	0.15	**ORF1a:N2526L**	7	0.2
Maharashtra							**ORF1a:D762L**	1	0.15	**ORF1a:K2533L**	7	0.2
Maharashtra							**ORF1a:Q764V**	1	0.15	**ORF1a:K2775C**	7	0.2
Maharashtra							**ORF1a:P765K**	1	0.15	**ORF1a:F2780L**	7	0.2
Maharashtra							**ORF1a:G780L**	1	0.15	**ORF1a:R3012***	7	0.2
Maharashtra							**ORF1a:P782G**	1	0.15	**ORF1a:S3013M**	7	0.2
Maharashtra							**ORF1a:V783L**	1	0.15	**ORF1a:M3030***	7	0.2
Maharashtra							**ORF1a:G818I**	1	0.15	**ORF1a:F3031I**	7	0.2
Maharashtra							**ORF1a:A819I**	1	0.15	**ORF1a:T3032Y**	7	0.2
Maharashtra							**ORF1a:K822L**	1	0.15	**ORF1a:P3033L**	7	0.2
Maharashtra							**ORF1a:V823***	1	0.15	**ORF1a:C3059I**	7	0.2
Maharashtra							**ORF1a:T824***	1	0.15	**ORF1a:F3071***	7	0.2
Maharashtra							**ORF1a:F825K**	1	0.15	**ORF1a:E3073L**	7	0.2
Maharashtra							**ORF1a:G826C**	1	0.15	**ORF1a:Y3074E**	7	0.2
Maharashtra							**ORF1a:D827K**	1	0.15	**ORF1a:S3075E**	7	0.2
Maharashtra							**ORF1a:D828V**	1	0.15	**ORF1a:H3076L**	7	0.2
Maharashtra							**ORF1a:I849L**	1	0.15	**ORF1a:V3077L**	7	0.2
Maharashtra							**ORF1a:D850P**	1	0.15	**ORF1a:A3079N**	7	0.2
Maharashtra							**ORF1a:K851I**	1	0.15	**ORF1a:F3080T**	7	0.2
Maharashtra							**ORF1a:V852Q**	1	0.15	**ORF1a:N3081V**	7	0.2
Maharashtra							**ORF1a:C873S**	1	0.15	**ORF1a:T3082M**	7	0.2
Maharashtra							**ORF1a:T882L**	1	0.15	**ORF1a:L3083***	7	0.2
Maharashtra							**ORF1a:L883W**	1	0.15	**ORF1a:F3085P**	7	0.2
Maharashtra							**ORF1a:P885M**	1	0.15	**ORF1a:S3088L**	7	0.2
Maharashtra							**ORF1a:V886L**	1	0.15	**ORF1a:F3089Y**	7	0.2
Maharashtra							**ORF1a:E888***	1	0.15	**ORF1a:T3090Y**	7	0.2
Maharashtra							**ORF1a:L889K**	1	0.15	**ORF1a:V3091S**	7	0.2
Maharashtra							**ORF1a:T891W**	1	0.15	**ORF1a:Q3373S**	7	0.2
Maharashtra							**ORF1a:P892A**	1	0.15	**ORF1a:N3484L**	7	0.2
Maharashtra							**ORF1a:G894I**	1	0.15	**ORF1a:R3485L**	7	0.2
Maharashtra							**ORF1a:I895***	1	0.15	**ORF1a:F3486M**	7	0.2
Maharashtra							**ORF1a:D896M**	1	0.15	**ORF1a:T3488L**	7	0.2
Maharashtra							**ORF1a:L897S**	1	0.15	**ORF1a:L3602F**	7	0.2
Maharashtra							**ORF1a:D898G**	1	0.15	**ORF1a:M3733L**	7	0.2
Maharashtra							**ORF1a:E899V**	1	0.15	**ORF1a:S3946L**	7	0.2
Maharashtra							**ORF1a:S901L**	1	0.15	**ORF1a:W4096L**	7	0.2
Maharashtra							**ORF1a:M902H**	1	0.15	**ORF1a:G209L**	7	0.2
Maharashtra							**ORF1a:A903T**	1	0.15	**ORF1a:L317A**	7	0.2
Maharashtra							**ORF1a:A917M**	1	0.15	**ORF1a:S318F**	7	0.2
Maharashtra							**ORF1a:H919Q**	1	0.15	**ORF1a:T319Q**	7	0.2
Maharashtra							**ORF1a:E961L**	1	0.15	**ORF1a:W331***	7	0.2
Maharashtra							**ORF1a:D996V**	1	0.15	**ORF1a:Q332S**	7	0.2
Maharashtra							**ORF1a:Q998K**	1	0.15	**ORF1a:T333V**	7	0.2
Maharashtra							**ORF1a:T1000R**	1	0.15	**ORF1a:G334I**	7	0.2
Maharashtra							**ORF1a:Q1003R**	1	0.15	**ORF1a:F336V**	7	0.2
Maharashtra							**ORF1a:T1004Q**	1	0.15	**ORF1a:D589W**	7	0.2
Maharashtra							**ORF1a:I1005L**	1	0.15	**ORF1a:A591L**	7	0.2
Maharashtra							**ORF1a:V1006L**	1	0.15	**ORF1a:N593I**	7	0.2
Maharashtra							**ORF1a:E1007F**	1	0.15	**ORF1a:N594***	7	0.2
Maharashtra							**ORF1a:V1008K**	1	0.15	**ORF1a:N3484***	7	0.2
Maharashtra							**ORF1a:P1010H**	1	0.15	**ORF1a:R3485M**	7	0.2
Maharashtra							**ORF1a:S1029F**	1	0.15	**ORF1a:F3486E**	7	0.2
Maharashtra							**ORF1a:G1030R**	1	0.15	**ORF1a:F3604T**	7	0.2
Maharashtra							**ORF1a:Y1031L**	1	0.15	**ORF1a:L3606C**	7	0.2
Maharashtra							**ORF1a:L1034***	1	0.15	**ORF1a:T4079I**	7	0.2
Maharashtra							**ORF1a:D1036V**	1	0.15	**ORF1a:V4109G**	7	0.2
Maharashtra							**ORF1a:N1037L**	1	0.15	**ORF1a:M4152***	7	0.2
Maharashtra							**ORF1a:Y1039V**	1	0.15	**ORF1a:A4156T**	7	0.2
Maharashtra							**ORF1a:K1041***	1	0.15	**ORF1a:G4157T**	7	0.2
Maharashtra							**ORF1a:V1052L**	1	0.15	**ORF1a:E1196L**	7	0.2
Maharashtra							**ORF1a:P1054F**	1	0.15	**ORF1a:F1353I**	7	0.2
Maharashtra							**ORF1a:T1093L**	1	0.15	**ORF1a:I1355Y**	7	0.2
Maharashtra							**ORF1a:G1095D**	1	0.15	**ORF1a:P1357***	7	0.2
Maharashtra							**ORF1a:P1096H**	1	0.15	**ORF1a:S1358***	7	0.2
Maharashtra							**ORF1a:A1111S**	1	0.15	**ORF1a:I1359E**	7	0.2
Maharashtra							**ORF1a:K1112A**	1	0.15	**ORF1a:I1360A**	7	0.2
Maharashtra							**ORF1a:H1113Q**	1	0.15	**ORF1a:V1393N**	7	0.2
Maharashtra							**ORF1a:C1114M**	1	0.15	**ORF1a:E1394Y**	7	0.2

Maharashtra							**ORF1a:H1116T**	1	0.15	**ORF1a:K1396A**	7	0.2
Maharashtra							**ORF1a:V1117K**	1	0.15	**ORF1a:A1397***	7	0.2
Maharashtra							**ORF1a:E1136K**	1	0.15	**ORF1a:D1575Y**	7	0.2
Maharashtra							**ORF1a:N1137T**	1	0.15	**ORF1a:I1577S**	7	0.2
Maharashtra							**ORF1a:Q1140F**	1	0.15	**ORF1a:N1578R**	7	0.2
Maharashtra							**ORF1a:H1141L**	1	0.15	**ORF1a:L1579Q**	7	0.2
Maharashtra							**ORF1a:E1142R**	1	0.15	**ORF1a:T1820S**	7	0.2
Maharashtra							**ORF1a:A1146V**	1	0.15	**ORF1a:F1821T**	7	0.2
Maharashtra							**ORF1a:P1147F**	1	0.15	**ORF1a:A1824W**	7	0.2
Maharashtra							**ORF1a:D1167L**	1	0.15	**ORF1a:Q2486K**	7	0.2
Maharashtra							**ORF1a:S1188K**	1	0.15	**ORF1a:D2582G**	7	0.2
Maharashtra							**ORF1a:F1190K**	1	0.15	**ORF1a:A2584C**	7	0.2
Maharashtra							**ORF1a:E1192N**	1	0.15	**ORF1a:E2585S**	7	0.2
Maharashtra							**ORF1a:M1193K**	1	0.15	**ORF1a:V2586***	7	0.2
Maharashtra							**ORF1a:K1194R**	1	0.15	**ORF1a:A2587N**	7	0.2
Maharashtra							**ORF1a:K1212L**	1	0.15	**ORF1a:K2589***	7	0.2
Maharashtra							**ORF1a:P1213R**	1	0.15	**ORF1a:M2590C**	7	0.2
Maharashtra							**ORF1a:I1215N**	1	0.15	**ORF1a:F2591L**	7	0.2
Maharashtra							**ORF1a:T1216R**	1	0.15	**ORF1a:D2592R**	7	0.2
Maharashtra							**ORF1a:S1218N**	1	0.15	**ORF1a:A2593***	7	0.2
Maharashtra							**ORF1a:D1229M**	1	0.15	**ORF1a:Q2660R**	7	0.2
Maharashtra							**ORF1a:K1230I**	1	0.15	**ORF1a:Y3109L**	7	0.2
Maharashtra							**ORF1a:H110Y**	1	0.15	**ORF1a:G3051S**	7	0.2
Maharashtra							**ORF1a:T2124I**	1	0.15	**ORF1a:T2846I**	7	0.2
Maharashtra							**ORF1a:S2900L**	1	0.15	**ORF1a:V1295G**	7	0.2
Maharashtra							**ORF1a:I581W**	1	0.15	**ORF1a:M1647I**	7	0.2
Maharashtra							**ORF1a:D582N**	1	0.15	**ORF1a:P309S**	7	0.2
Maharashtra							**ORF1a:A583F**	1	0.15	**ORF1a:I453T**	7	0.2
Maharashtra							**ORF1a:M584T**	1	0.15	**ORF1a:A3856C**	7	0.2
Maharashtra							**ORF1a:M585V**	1	0.15	**ORF1a:Q3859***	7	0.2
Maharashtra							**ORF1a:V605C**	1	0.15	**ORF1a:S3860N**	7	0.2
Maharashtra							**ORF1a:V606N**	1	0.15	**ORF1a:K3861V**	7	0.2
Maharashtra							**ORF1a:Q607G**	1	0.15	**ORF1a:E3882G**	7	0.2
Maharashtra							**ORF1a:T609H**	1	0.15	**ORF1a:P3952T**	7	0.2
Maharashtra							**ORF1a:S610Y**	1	0.15	**ORF1a:L3086F**	7	0.2
Maharashtra							**ORF1a:Q611R**	1	0.15	**ORF1a:G445V**	7	0.2
Maharashtra							**ORF1a:L613K**	1	0.15	**ORF1a:V757P**	7	0.2
Maharashtra							**ORF1a:K623Q**	1	0.15	**ORF1a:L758Q**	7	0.2
Maharashtra							**ORF1a:L624T**	1	0.15	**ORF1a:E855L**	7	0.2
Maharashtra							**ORF1a:K625R**	1	0.15	**ORF1a:S1029F**	7	0.2
Maharashtra							**ORF1a:F685S**	1	0.15	**ORF1a:G1030R**	7	0.2
Maharashtra							**ORF1a:L686D**	1	0.15	**ORF1a:Y1031L**	7	0.2
Maharashtra							**ORF1a:A687I**	1	0.15	**ORF1a:V1299L**	7	0.2
Maharashtra							**ORF1a:C689***	1	0.15	**ORF1a:V2019L**	7	0.2
Maharashtra							**ORF1a:D691C**	1	0.15	**ORF1a:A2388S**	7	0.2
Maharashtra							**ORF1a:S692K**	1	0.15	**ORF1a:S2389Y**	7	0.2
Maharashtra							**ORF1a:I693***	1	0.15	**ORF1a:F2390G**	7	0.2
Maharashtra							**ORF1a:I695F**	1	0.15	**ORF1a:Y2391***	7	0.2
Maharashtra							**ORF1a:G697***	1	0.15	**ORF1a:Y2392N**	7	0.2
Maharashtra							**ORF1a:A698N**	1	0.15	**ORF1a:W2394R**	7	0.2
Maharashtra							**ORF1a:K699I**	1	0.15	**ORF1a:K2395L**	7	0.2
Maharashtra							**ORF1a:C720T**	1	0.15	**ORF1a:S2396***	7	0.2
Maharashtra							**ORF1a:V721H**	1	0.15	**ORF1a:Y2397F**	7	0.2
Maharashtra							**ORF1a:K722A**	1	0.15	**ORF1a:V2398I**	7	0.2
Maharashtra							**ORF1a:P748E**	1	0.15	**ORF1a:H2399N**	7	0.2
Maharashtra							**ORF1a:T749N**	1	0.15	**ORF1a:V2400L**	7	0.2
Maharashtra							**ORF1a:E750W**	1	0.15	**ORF1a:V2401Y**	7	0.2
Maharashtra							**ORF1a:V751***	1	0.15	**ORF1a:V2436L**	7	0.2
Maharashtra							**ORF1a:L752F**	1	0.15	**ORF1a:Y2437L**	7	0.2
Maharashtra							**ORF1a:E772I**	1	0.15	**ORF1a:A2438Q**	7	0.2
Maharashtra							**ORF1a:A773G**	1	0.15	**ORF1a:N2439T**	7	0.2
Maharashtra							**ORF1a:V774W**	1	0.15	**ORF1a:G2440T**	7	0.2
Maharashtra							**ORF1a:E775Y**	1	0.15	**ORF1a:G2441Q**	7	0.2
Maharashtra							**ORF1a:A776T**	1	0.15	**ORF1a:K2442L**	7	0.2
Maharashtra							**ORF1a:P777S**	1	0.15	**ORF1a:G2443E**	7	0.2
Maharashtra							**ORF1a:D795Y**	1	0.15	**ORF1a:F2444L**	7	0.2
Maharashtra							**ORF1a:T796***	1	0.15	**ORF1a:K2446***	7	0.2
Maharashtra							**ORF1a:E797R**	1	0.15	**ORF1a:T2463***	7	0.2
Maharashtra							**ORF1a:K798A**	1	0.15	**ORF1a:F2464Y**	7	0.2
Maharashtra							**ORF1a:C800V**	1	0.15	**ORF1a:D2467C**	7	0.2
Maharashtra							**ORF1a:V808H**	1	0.15	**ORF1a:S2556L**	7	0.2
Maharashtra							**ORF1a:N810Q**	1	0.15	**ORF1a:Y2561V**	7	0.2
Maharashtra							**ORF1a:N811R**	1	0.15	**ORF1a:Q2574T**	7	0.2

Maharashtra							**ORF1a:T812R**	1	0.15	**ORF1a:A2575Y**	7	0.2
Maharashtra							**ORF1a:F813C**	1	0.15	**ORF1a:L2576T**	7	0.2
Maharashtra							**ORF1a:L815N**	1	0.15	**ORF1a:S2578T**	7	0.2
Maharashtra							**ORF1a:G835***	1	0.15	**ORF1a:D2579R**	7	0.2
Maharashtra							**ORF1a:Y836***	1	0.15	**ORF1a:V2580S**	7	0.2
Maharashtra							**ORF1a:G865***	1	0.15	**ORF1a:G2667S**	7	0.2
Maharashtra							**ORF1a:T866***	1	0.15	**ORF1a:D2668I**	7	0.2
Maharashtra							**ORF1a:N869H**	1	0.15	**ORF1a:S2669***	7	0.2
Maharashtra							**ORF1a:G993N**	1	0.15	**ORF1a:C2670H**	7	0.2
Maharashtra							**ORF1a:S994Y**	1	0.15	**ORF1a:N2671R**	7	0.2
Maharashtra							**ORF1a:E995Y**	1	0.15	**ORF1a:N2672S**	7	0.2
Maharashtra							**ORF1a:D996S**	1	0.15	**ORF1a:D2693L**	7	0.2
Maharashtra							**ORF1a:Q998G**	1	0.15	**ORF1a:R2697S**	7	0.2
Maharashtra							**ORF1a:T999S**	1	0.15	**ORF1a:H2698Y**	7	0.2
Maharashtra							**ORF1a:T1001S**	1	0.15	**ORF1a:I2699***	7	0.2
Maharashtra							**ORF1a:Q1021R**	1	0.15	**ORF1a:N2700C**	7	0.2
Maharashtra							**ORF1a:T1022D**	1	0.15	**ORF1a:T2823***	7	0.2
Maharashtra							**ORF1a:I1023G**	1	0.15	**ORF1a:A2944***	7	0.2
Maharashtra							**ORF1a:E1024T**	1	0.15	**ORF1a:E2946Q**	7	0.2
Maharashtra							**ORF1a:V1025Y**	1	0.15	**ORF1a:S2947C**	7	0.2
Maharashtra							**ORF1a:N1026T**	1	0.15	**ORF1a:L2948T**	7	0.2
Maharashtra							**ORF1a:N1059G**	1	0.15	**ORF1a:E2970Y**	7	0.2
Maharashtra							**ORF1a:A1060K**	1	0.15	**ORF1a:G3072Y**	7	0.2
Maharashtra							**ORF1a:A1061T**	1	0.15	**ORF1a:V3091L**	7	0.2
Maharashtra							**ORF1a:V1063R**	1	0.15	**ORF1a:C3093I**	7	0.2
Maharashtra							**ORF1a:Y1064S**	1	0.15	**ORF1a:L3110W**	7	0.2
Maharashtra							**ORF1a:I1091W**	1	0.15	**ORF1a:Y3111C**	7	0.2
Maharashtra							**ORF1a:A1092T**	1	0.15	**ORF1a:T3113F**	7	0.2
Maharashtra							**ORF1a:L1105***	1	0.15	**ORF1a:F3114C**	7	0.2
Maharashtra							**ORF1a:H1108W**	1	0.15	**ORF1a:I3306S**	7	0.2
Maharashtra							**ORF1a:N1109***	1	0.15	**ORF1a:C3307K**	7	0.2
Maharashtra							**ORF1a:A1111P**	1	0.15	**ORF1a:S3309C**	7	0.2
Maharashtra							**ORF1a:D1127C**	1	0.15	**ORF1a:E3310D**	7	0.2
Maharashtra							**ORF1a:I1128***	1	0.15	**ORF1a:D3311L**	7	0.2
Maharashtra							**ORF1a:R1170S**	1	0.15	**ORF1a:D3972V**	7	0.2
Maharashtra							**ORF1a:T1171H**	1	0.15	**ORF1a:S3973E**	7	0.2
Maharashtra							**ORF1a:N1172K**	1	0.15	**ORF1a:F1937I**	7	0.2
Maharashtra							**ORF1a:V1173C**	1	0.15	**ORF1a:A1938I**	7	0.2
Maharashtra							**ORF1a:Y1174L**	1	0.15	**ORF1a:D1939L**	7	0.2
Maharashtra							**ORF1a:A1176S**	1	0.15	**ORF1a:D1940S**	7	0.2
Maharashtra							**ORF1a:V1177C**	1	0.15	**ORF1a:S2030I**	7	0.2
Maharashtra							**ORF1a:D1585T**	1	0.15	**ORF1a:E2031L**	7	0.2
Maharashtra							**ORF1a:T1605L**	1	0.15	**ORF1a:D2032P**	7	0.2
Maharashtra							**ORF1a:K1608H**	1	0.15	**ORF1a:K3351H**	7	0.2
Maharashtra							**ORF1a:P1609M**	1	0.15	**ORF1a:Y3211R**	7	0.2
Maharashtra							**ORF1a:H1610K**	1	0.15	**ORF1a:K2108***	7	0.2
Maharashtra							**ORF1a:N1611V**	1	0.15	**ORF1a:N2111L**	7	0.2
Maharashtra							**ORF1a:S1612K**	1	0.15	**ORF1a:S2114M**	7	0.2
Maharashtra							**ORF1a:E1614L**	1	0.15	**ORF1a:R2115V**	7	0.2
Maharashtra							**ORF1a:G1615C**	1	0.15	**ORF1a:V2116***	7	0.2
Maharashtra							**ORF1a:K1616F**	1	0.15	**ORF1a:L2176I**	7	0.2
Maharashtra							**ORF1a:V3298L**	1	0.15	**ORF1a:Q2178E**	7	0.2
Maharashtra							**ORF1a:V2149F**	1	0.15	**ORF1a:C2180K**	7	0.2
Maharashtra							**ORF1a:S2625F**	1	0.15	**ORF1a:T2181H**	7	0.2
Maharashtra							**ORF1a:G993D**	1	0.15	**ORF1a:F2182L**	7	0.2
Maharashtra							**ORF1a:S3547G**	1	0.15	**ORF1a:T2183C**	7	0.2
Maharashtra							**ORF1a:K734E**	1	0.15	**ORF1a:S2185L**	7	0.2
Maharashtra							**ORF1a:L454F**	1	0.15	**ORF1a:T2186L**	7	0.2
Maharashtra							**ORF1a:T814S**	1	0.15	**ORF1a:N2187***	7	0.2
Maharashtra							**ORF1a:C857Y**	1	0.15	**ORF1a:S2188Q**	7	0.2
Maharashtra							**ORF1a:S858F**	1	0.15	**ORF1a:C2210L**	7	0.2
Maharashtra							**ORF1a:A859I**	1	0.15	**ORF1a:E2212R**	7	0.2
Maharashtra							**ORF1a:Y860N**	1	0.15	**ORF1a:A2213V**	7	0.2
Maharashtra							**ORF1a:T861P**	1	0.15	**ORF1a:F2215V**	7	0.2
Maharashtra							**ORF1a:V862F**	1	0.15	**ORF1a:G2251T**	7	0.2
Maharashtra							**ORF1a:E870Q**	1	0.15	**ORF1a:K1720P**	7	0.2
Maharashtra							**ORF1a:K1763N**	1	0.15	**ORF1a:Y1846F**	7	0.2
Maharashtra							**ORF1a:F2633L**	1	0.15	**ORF1a:C1847***	7	0.2
Maharashtra							**ORF1a:L3806V**	1	0.15	**ORF1a:I1848R**	7	0.2
Maharashtra							**ORF1a:M1389K**	1	0.15	**ORF1a:G1850F**	7	0.2
Maharashtra							**ORF1a:S1189K**	1	0.15	**ORF1a:L1853H**	7	0.2
Maharashtra							**ORF1a:F1190L**	1	0.15	**ORF1a:K1855R**	7	0.2
Maharashtra							**ORF1a:T151I**	1	0.15	**ORF1a:S1856C**	7	0.2

Maharashtra							**ORF1a:S391F**	1	0.15	**ORF1a:S1857F**	7	0.2
Maharashtra							**ORF1a:A698K**	1	0.15	**ORF1a:E1858T**	7	0.2
Maharashtra							**ORF1a:T1864F**	1	0.15	**ORF1a:Y1899L**	7	0.2
Maharashtra							**ORF1a:V1866I**	1	0.15	**ORF1a:F2740E**	7	0.2
Maharashtra							**ORF1a:F1867I**	1	0.15	**ORF1a:K2741***	7	0.2
Maharashtra							**ORF1a:I1877L**	1	0.15	**ORF1a:G2762N**	7	0.2
Maharashtra							**ORF1a:P1879L**	1	0.15	**ORF1a:G2763N**	7	0.2
Maharashtra							**ORF1a:V1880S**	1	0.15	**ORF1a:I2765A**	7	0.2
Maharashtra							**ORF1a:Y1882L**	1	0.15	**ORF1a:K2775S**	7	0.2
Maharashtra							**ORF1a:K1883A**	1	0.15	**ORF1a:V2776Y**	7	0.2
Maharashtra							**ORF1a:L1884F**	1	0.15	**ORF1a:L2778C**	7	0.2
Maharashtra							**ORF1a:G1886M**	1	0.15	**ORF1a:D3355***	7	0.2
Maharashtra							**ORF1a:E1891R**	1	0.15	**ORF1a:T3356V**	7	0.2
Maharashtra							**ORF1a:K1895Q**	1	0.15	**ORF1a:A3357C**	7	0.2
Maharashtra							**ORF1a:D1897A**	1	0.15	**ORF1a:P3359H**	7	0.2
Maharashtra							**ORF1a:N1898A**	1	0.15	**ORF1a:K3360S**	7	0.2
Maharashtra							**ORF1a:Y1899L**	1	0.15	**ORF1a:P3362R**	7	0.2
Maharashtra							**ORF1a:Y1900***	1	0.15	**ORF1a:K3363T**	7	0.2
Maharashtra							**ORF1a:K1901N**	1	0.15	**ORF1a:Y3364D**	7	0.2
Maharashtra							**ORF1a:D1903N**	1	0.15	**ORF1a:K3365F**	7	0.2
Maharashtra							**ORF1a:N1904C**	1	0.15	**ORF1a:P3385V**	7	0.2
Maharashtra							**ORF1a:Y1906S**	1	0.15	**ORF1a:G3387F**	7	0.2
Maharashtra							**ORF1a:F1907Y**	1	0.15	**ORF1a:V3388H**	7	0.2
Maharashtra							**ORF1a:T1908N**	1	0.15	**ORF1a:M315I**	7	0.2
Maharashtra							**ORF1a:E1909N**	1	0.15	**ORF1a:T1297V**	7	0.2
Maharashtra							**ORF1a:Q1910C**	1	0.15	**ORF1a:G1644***	7	0.2
Maharashtra							**ORF1a:D1913S**	1	0.15	**ORF1a:R1645V**	7	0.2
Maharashtra							**ORF1a:P1916Q**	1	0.15	**ORF1a:Y1646H**	7	0.2
Maharashtra							**ORF1a:P1919T**	1	0.15	**ORF1a:F1690V**	7	0.2
Maharashtra							**ORF1a:Q4397E**	1	0.15	**ORF1a:P1692T**	7	0.2
Maharashtra							**ORF1a:V1728I**	1	0.15	**ORF1a:A1694L**	7	0.2
Maharashtra							**ORF1a:L2333Q**	1	0.15	**ORF1a:G1705S**	7	0.2
Maharashtra							**ORF1a:F2334C**	1	0.15	**ORF1a:E1706K**	7	0.2
Maharashtra							**ORF1a:G2342K**	1	0.15	**ORF1a:A1707G**	7	0.2
Maharashtra							**ORF1a:L2343I**	1	0.15	**ORF1a:A1708W**	7	0.2
Maharashtra							**ORF1a:M2347A**	1	0.15	**ORF1a:N1709***	7	0.2
Maharashtra							**ORF1a:Y2353N**	1	0.15	**ORF1a:F1710S**	7	0.2
Maharashtra							**ORF1a:F2354P**	1	0.15	**ORF1a:A1712***	7	0.2
Maharashtra							**ORF1a:A3615V**	1	0.15	**ORF1a:L1715C**	7	0.2
Maharashtra							**ORF1a:G696V**	1	0.15	**ORF1a:A1716T**	7	0.2
Maharashtra							**ORF1a:G697S**	1	0.15	**ORF1a:A1146V**	7	0.2
Maharashtra							**ORF1a:A698P**	1	0.15	**ORF1a:P1147F**	7	0.2
Maharashtra							**ORF1a:L700F**	1	0.15	**ORF1a:D1167L**	7	0.2
Maharashtra							**ORF1a:N704S**	1	0.15	**ORF1a:L552I**	7	0.2
Maharashtra							**ORF1a:F709S**	1	0.15	**ORF1a:S4398L**	7	0.2
Maharashtra							**ORF1a:V710T**	1	0.15	**ORF1a:T1241I**	7	0.2
Maharashtra							**ORF1a:P1879S**	1	0.15	**ORF1a:L3667F**	6	0.17
Maharashtra							**ORF1a:Y1882***	1	0.15	**ORF1a:V2776S**	6	0.17
Maharashtra							**ORF1a:K1883E**	1	0.15	**ORF1a:T2777F**	6	0.17
Maharashtra							**ORF1a:C1889S**	1	0.15	**ORF1a:V2779L**	6	0.17
Maharashtra							**ORF1a:T1890I**	1	0.15	**ORF1a:L2789S**	6	0.17
Maharashtra							**ORF1a:E1891I**	1	0.15	**ORF1a:T2791***	6	0.17
Maharashtra							**ORF1a:D1893V**	1	0.15	**ORF1a:P2792***	6	0.17
Maharashtra							**ORF1a:P1911L**	1	0.15	**ORF1a:V2793H**	6	0.17
Maharashtra							**ORF1a:D1913N**	1	0.15	**ORF1a:H2794L**	6	0.17
Maharashtra							**ORF1a:Q556K**	1	0.15	**ORF1a:V2795F**	6	0.17
Maharashtra							**ORF1a:Q575R**	1	0.15	**ORF1a:P2852***	6	0.17
Maharashtra							**ORF1a:V1583F**	1	0.15	**ORF1a:S2911L**	6	0.17
Maharashtra							**ORF1a:M2259I**	1	0.15	**ORF1a:A2912Q**	6	0.17
Maharashtra							**ORF1a:V2750I**	1	0.15	**ORF1a:C2913H**	6	0.17
Maharashtra							**ORF1a:E471D**	1	0.15	**ORF1a:V2914Q**	6	0.17
Maharashtra							**ORF1a:E472Y**	1	0.15	**ORF1a:T2936L**	6	0.17
Maharashtra							**ORF1a:I473T**	1	0.15	**ORF1a:N2937L**	6	0.17
Maharashtra							**ORF1a:A478T**	1	0.15	**ORF1a:Q3127L**	6	0.17
Maharashtra							**ORF1a:F480***	1	0.15	**ORF1a:W3128M**	6	0.17
Maharashtra							**ORF1a:T554I**	1	0.15	**ORF1a:V3130F**	6	0.17
Maharashtra							**ORF1a:E632D**	1	0.15	**ORF1a:M3131L**	6	0.17
Maharashtra							**ORF1a:K1405N**	1	0.15	**ORF1a:M3221I**	6	0.17
Maharashtra							**ORF1a:Y1406I**	1	0.15	**ORF1a:Y3226I**	6	0.17
Maharashtra							**ORF1a:M2254K**	1	0.15	**ORF1a:R3227L**	6	0.17
Maharashtra							**ORF1a:G2258D**	1	0.15	**ORF1a:E3228V**	6	0.17
Maharashtra							**ORF1a:P2260T**	1	0.15	**ORF1a:A3229E**	6	0.17
Maharashtra							**ORF1a:C2263***	1	0.15	**ORF1a:A3230Q**	6	0.17

Maharashtra							**ORF1a:L2271E**	1	0.15	**ORF1a:C3231W**	6	0.17
Maharashtra							**ORF1a:T2274S**	1	0.15	**ORF1a:C3232I**	6	0.17
Maharashtra							**ORF1a:S2289K**	1	0.15	**ORF1a:H3233Q**	6	0.17
Maharashtra							**ORF1a:V2290N**	1	0.15	**ORF1a:F3605Y**	6	0.17
Maharashtra							**ORF1a:M2606I**	1	0.15	**ORF1a:A3620C**	6	0.17
Maharashtra							**ORF1a:G3827I**	1	0.15	**ORF1a:A3625S**	6	0.17
Maharashtra							**ORF1a:Y799N**	1	0.15	**ORF1a:V3858L**	6	0.17
Maharashtra							**ORF1a:A1268V**	1	0.15	**ORF1a:A3939T**	6	0.17
Maharashtra							**ORF1a:A2355K**	1	0.15	**ORF1a:L3941G**	6	0.17
Maharashtra							**ORF1a:I238V**	1	0.15	**ORF1a:F143L**	6	0.17
Maharashtra							**ORF1a:T2183V**	1	0.15	**ORF1a:A181S**	6	0.17
Maharashtra							**ORF1a:R2184K**	1	0.15	**ORF1a:Y182I**	6	0.17
Maharashtra							**ORF1a:M2259L**	1	0.15	**ORF1a:R184T**	6	0.17
Maharashtra							**ORF1a:E472R**	1	0.15	**ORF1a:Y185S**	6	0.17
Maharashtra							**ORF1a:M2347I**	1	0.15	**ORF1a:D203A**	6	0.17
Maharashtra							**ORF1a:L2367***	1	0.15	**ORF1a:A208L**	6	0.17
Maharashtra							**ORF1a:F3611L**	1	0.15	**ORF1a:N313H**	6	0.17
Maharashtra							**ORF1a:P1961F**	1	0.15	**ORF1a:N449V**	6	0.17
Maharashtra							**ORF1a:L1986***	1	0.15	**ORF1a:V488K**	6	0.17
Maharashtra							**ORF1a:P2005F**	1	0.15	**ORF1a:E489H**	6	0.17
Maharashtra							**ORF1a:P3447H**	1	0.15	**ORF1a:T490S**	6	0.17
Maharashtra							**ORF1a:F3448I**	1	0.15	**ORF1a:V491N**	6	0.17
Maharashtra							**ORF1a:V3449I**	1	0.15	**ORF1a:G493L**	6	0.17
Maharashtra							**ORF1a:S1221L**	1	0.15	**ORF1a:S531V**	6	0.17
Maharashtra							**ORF1a:E1245R**	1	0.15	**ORF1a:A535D**	6	0.17
Maharashtra							**ORF1a:M2606L**	1	0.15	**ORF1a:F536Q**	6	0.17
Maharashtra							**ORF1a:Y576C**	1	0.15	**ORF1a:A537F**	6	0.17
Maharashtra							**ORF1a:V682L**	1	0.15	**ORF1a:E539P**	6	0.17
Maharashtra							**ORF1a:N683E**	1	0.15	**ORF1a:Q563L**	6	0.17
Maharashtra							**ORF1a:F685L**	1	0.15	**ORF1a:I3148L**	6	0.17
Maharashtra							**ORF1a:V23A**	1	0.15	**ORF1a:I4074V**	6	0.17
Maharashtra							**ORF1a:V35T**	1	0.15	**ORF1a:P4075***	6	0.17
Maharashtra							**ORF1a:T78S**	1	0.15	**ORF1a:D4076W**	6	0.17
Maharashtra							**ORF1a:A79P**	1	0.15	**ORF1a:Q4099S**	6	0.17
Maharashtra							**ORF1a:P80K**	1	0.15	**ORF1a:Q4100I**	6	0.17
Maharashtra							**ORF1a:H81P**	1	0.15	**ORF1a:M1193***	6	0.17
Maharashtra							**ORF1a:G82R**	1	0.15	**ORF1a:V1236***	6	0.17
Maharashtra							**ORF1a:T1474I**	1	0.15	**ORF1a:T1277H**	6	0.17
Maharashtra							**ORF1a:H2092Y**	1	0.15	**ORF1a:L1375C**	6	0.17
Maharashtra							**ORF1a:A773V**	1	0.15	**ORF1a:R1376F**	6	0.17
Maharashtra							**ORF1a:V774F**	1	0.15	**ORF1a:M1378E**	6	0.17
Maharashtra							**ORF1a:L2250***	1	0.15	**ORF1a:L1379F**	6	0.17
Maharashtra							**ORF1a:Y2281***	1	0.15	**ORF1a:H1381R**	6	0.17
Maharashtra							**ORF1a:C2282***	1	0.15	**ORF1a:A1382N**	6	0.17
Maharashtra							**ORF1a:T2283N**	1	0.15	**ORF1a:E1383A**	6	0.17
Maharashtra							**ORF1a:I2359S**	1	0.15	**ORF1a:E1384C**	6	0.17
Maharashtra							**ORF1a:S17R**	1	0.15	**ORF1a:T1573G**	6	0.17
Maharashtra							**ORF1a:Q22R**	1	0.15	**ORF1a:Q1582T**	6	0.17
Maharashtra							**ORF1a:V23F**	1	0.15	**ORF1a:P1596W**	6	0.17
Maharashtra							**ORF1a:D25K**	1	0.15	**ORF1a:N2482***	6	0.17
Maharashtra							**ORF1a:V26A**	1	0.15	**ORF1a:P2483K**	6	0.17
Maharashtra							**ORF1a:V28H**	1	0.15	**ORF1a:D2485N**	6	0.17
Maharashtra							**ORF1a:R29L**	1	0.15	**ORF1a:S2488Y**	6	0.17
Maharashtra							**ORF1a:G30T**	1	0.15	**ORF1a:Y2489***	6	0.17
Maharashtra							**ORF1a:T78R**	1	0.15	**ORF1a:I2490P**	6	0.17
Maharashtra							**ORF1a:K2395E**	1	0.15	**ORF1a:D2492P**	6	0.17
Maharashtra							**ORF1a:N2361K**	1	0.15	**ORF1a:V2494L**	6	0.17
Maharashtra							**ORF1a:S2362I**	1	0.15	**ORF1a:T2495L**	6	0.17
Maharashtra							**ORF1a:W2363K**	1	0.15	**ORF1a:V2496***	6	0.17
Maharashtra							**ORF1a:L2364I**	1	0.15	**ORF1a:K2497***	6	0.17
Maharashtra							**ORF1a:V721F**	1	0.15	**ORF1a:N2498S**	6	0.17
Maharashtra							**ORF1a:C3093***	1	0.15	**ORF1a:G2499W**	6	0.17
Maharashtra							**ORF1a:L3094***	1	0.15	**ORF1a:A3061L**	6	0.17
Maharashtra							**ORF1a:T727I**	1	0.15	**ORF1a:Y3062F**	6	0.17
Maharashtra							**ORF1a:G728Y**	1	0.15	**ORF1a:H3076E**	6	0.17
Maharashtra							**ORF1a:F2354I**	1	0.15	**ORF1a:A3079I**	6	0.17
Maharashtra							**ORF1a:L1191W**	1	0.15	**ORF1a:F3080H**	6	0.17
Maharashtra							**ORF1a:E1192K**	1	0.15	**ORF1a:N3081C**	6	0.17
Maharashtra							**ORF1a:I523V**	1	0.15	**ORF1a:L3086N**	6	0.17
Maharashtra							**ORF1a:V2356L**	1	0.15	**ORF1a:M3087T**	6	0.17
Maharashtra							**ORF1a:L3210F**	1	0.15	**ORF1a:Y3111I**	6	0.17
Maharashtra							**ORF1a:K3353N**	1	0.15	**ORF1a:Y3115F**	6	0.17
Maharashtra							**ORF1a:A3648V**	1	0.15	**ORF1a:L3116S**	6	0.17

Maharashtra							**ORF1a:T3665I**	1	0.15	**ORF1a:L204F**	6	0.17
Maharashtra							**ORF1a:A2828V**	1	0.15	**ORF1a:R24C**	6	0.17
Maharashtra							**ORF1a:E942V**	1	0.15	**ORF1a:T3488I**	6	0.17
Maharashtra							**ORF1a:G1125S**	1	0.15	**ORF1a:T2823I**	6	0.17
Maharashtra							**ORF1a:G3072C**	1	0.15	**ORF1a:A3901***	6	0.17
Maharashtra							**ORF1a:E670K**	1	0.15	**ORF1a:R4022Y**	6	0.17
Maharashtra							**ORF1a:T224I**	1	0.15	**ORF1a:K4176T**	6	0.17
Maharashtra							**ORF1a:S610L**	1	0.15	**ORF1a:G4178I**	6	0.17
Maharashtra							**ORF1a:I2057T**	1	0.15	**ORF1a:F4230R**	6	0.17
Maharashtra							**ORF1a:H2659Y**	1	0.15	**ORF1a:A2584V**	6	0.17
Maharashtra							**ORF1a:R1170H**	1	0.15	**ORF1a:C373***	6	0.17
Maharashtra							**ORF1a:D1513G**	1	0.15	**ORF1a:H374D**	6	0.17
Maharashtra							**ORF1a:Q1140H**	1	0.15	**ORF1a:N375L**	6	0.17
Maharashtra							**ORF1a:A1938S**	1	0.15	**ORF1a:A599***	6	0.17
Maharashtra							**ORF1a:S3983F**	1	0.15	**ORF1a:Y600W**	6	0.17
Maharashtra							**ORF1a:A2128D**	1	0.15	**ORF1a:I601P**	6	0.17
Maharashtra							**ORF1a:K680M**	1	0.15	**ORF1a:I664L**	6	0.17
Maharashtra							**ORF1a:E797K**	1	0.15	**ORF1a:T666K**	6	0.17
Maharashtra							**ORF1a:E942A**	1	0.15	**ORF1a:C667L**	6	0.17
Maharashtra							**ORF1a:S3173N**	1	0.15	**ORF1a:A668S**	6	0.17
Maharashtra							**ORF1a:T814I**	1	0.15	**ORF1a:K669V**	6	0.17
Maharashtra							**ORF1a:N462T**	1	0.15	**ORF1a:E670D**	6	0.17
Maharashtra							**ORF1a:F1138I**	1	0.15	**ORF1a:I671K**	6	0.17
Maharashtra							**ORF1a:W2232C**	1	0.15	**ORF1a:K672L**	6	0.17
Maharashtra							**ORF1a:L2234I**	1	0.15	**ORF1a:E673S**	6	0.17
Maharashtra							**ORF1a:S1666F**	1	0.15	**ORF1a:S674P**	6	0.17
Maharashtra							**ORF1a:R426L**	1	0.15	**ORF1a:I739K**	6	0.17
Maharashtra							**ORF1a:K714N**	1	0.15	**ORF1a:I740H**	6	0.17
Maharashtra							**ORF1a:M3280I**	1	0.15	**ORF1a:T760N**	6	0.17
Maharashtra							**ORF1a:M1647I**	1	0.15	**ORF1a:G761N**	6	0.17
Maharashtra							**ORF1a:M3539V**	1	0.15	**ORF1a:D762L**	6	0.17
Maharashtra							**ORF1a:A498P**	1	0.15	**ORF1a:Q764V**	6	0.17
Maharashtra							**ORF1a:G445V**	1	0.15	**ORF1a:T861P**	6	0.17
Maharashtra							**ORF1a:P1480L**	1	0.15	**ORF1a:T882L**	6	0.17
Maharashtra							**ORF1a:Y2489H**	1	0.15	**ORF1a:T891W**	6	0.17
Maharashtra							**ORF1a:D203N**	1	0.15	**ORF1a:P892A**	6	0.17
Maharashtra							**ORF1a:A260S**	1	0.15	**ORF1a:G894I**	6	0.17
Maharashtra							**ORF1a:T1788M**	1	0.15	**ORF1a:I895***	6	0.17
Maharashtra							**ORF1a:Y1285H**	1	0.15	**ORF1a:D896M**	6	0.17
Maharashtra							**ORF1a:V38A**	1	0.15	**ORF1a:L897S**	6	0.17
Maharashtra							**ORF1a:M3780I**	1	0.15	**ORF1a:D898G**	6	0.17
Maharashtra							**ORF1a:S1561F**	1	0.15	**ORF1a:E899V**	6	0.17
Maharashtra							**ORF1a:P2221S**	1	0.15	**ORF1a:S901L**	6	0.17
Maharashtra							**ORF1a:A3079S**	1	0.15	**ORF1a:M902H**	6	0.17
Maharashtra							**ORF1a:K816R**	1	0.15	**ORF1a:A903T**	6	0.17
Maharashtra							**ORF1a:T1543S**	1	0.15	**ORF1a:E961L**	6	0.17
Maharashtra							**ORF1a:A2696V**	1	0.15	**ORF1a:Q1003R**	6	0.17
Maharashtra							**ORF1a:D1554Y**	1	0.15	**ORF1a:I1005L**	6	0.17
Maharashtra							**ORF1a:V1211F**	1	0.15	**ORF1a:P1010H**	6	0.17
Maharashtra							**ORF1a:T2859I**	1	0.15	**ORF1a:G1095D**	6	0.17
Maharashtra							**ORF1a:Y2384***	1	0.15	**ORF1a:P1096H**	6	0.17
Maharashtra							**ORF1a:I2385V**	1	0.15	**ORF1a:L1145M**	6	0.17
Maharashtra							**ORF1a:F2386C**	1	0.15	**ORF1a:A1146K**	6	0.17
Maharashtra							**ORF1a:A1342S**	1	0.15	**ORF1a:P1147I**	6	0.17
Maharashtra							**ORF1a:G3578S**	1	0.15	**ORF1a:F1821***	6	0.17
Maharashtra							**ORF1a:T4164I**	1	0.15	**ORF1a:C1823***	6	0.17
Maharashtra							**ORF1a:A339T**	1	0.15	**ORF1a:V1931I**	6	0.17
Maharashtra							**ORF1a:A1283V**	1	0.15	**ORF1a:C2009***	6	0.17
Maharashtra							**ORF1a:D930Y**	1	0.15	**ORF1a:I2010T**	6	0.17
Maharashtra							**ORF1a:A540V**	1	0.15	**ORF1a:R2011K**	6	0.17
Maharashtra							**ORF1a:L2572I**	1	0.15	**ORF1a:C2012Y**	6	0.17
Maharashtra							**ORF1a:S1478T**	1	0.15	**ORF1a:W2014V**	6	0.17
Maharashtra							**ORF1a:S2024L**	1	0.15	**ORF1a:S2015Y**	6	0.17
Maharashtra							**ORF1a:A4314S**	1	0.15	**ORF1a:K2017L**	6	0.17
Maharashtra							**ORF1a:T609I**	1	0.15	**ORF1a:K2067H**	6	0.17
Maharashtra							**ORF1a:D1939A**	1	0.15	**ORF1a:I2086***	6	0.17
Maharashtra							**ORF1a:L2062I**	1	0.15	**ORF1a:I2156V**	6	0.17
Maharashtra							**ORF1a:A2128T**	1	0.15	**ORF1a:V2157F**	6	0.17
Maharashtra							**ORF1a:H2092D**	1	0.15	**ORF1a:T2158K**	6	0.17
Maharashtra							**ORF1a:Q663K**	1	0.15	**ORF1a:R2159P**	6	0.17
Maharashtra							**ORF1a:G728C**	1	0.15	**ORF1a:T2174Y**	6	0.17
Maharashtra							**ORF1a:M3934V**	1	0.15	**ORF1a:L2175***	6	0.17
Maharashtra							**ORF1a:L3951F**	1	0.15	**ORF1a:Q2178A**	6	0.17

Maharashtra							**ORF1a:D953Y**	1	0.15	**ORF1a:C2180F**	6	0.17
Maharashtra							**ORF1a:Q4110L**	1	0.15	**ORF1a:T2181L**	6	0.17
Maharashtra							**ORF1a:C1235Y**	1	0.15	**ORF1a:F2182Y**	6	0.17
Maharashtra							**ORF1a:I2359Y**	1	0.15	**ORF1a:T2183F**	6	0.17
Maharashtra							**ORF1a:L2869F**	1	0.15	**ORF1a:R2184I**	6	0.17
Maharashtra							**ORF1a:A239V**	1	0.15	**ORF1a:S2185A**	6	0.17
Maharashtra							**ORF1a:K2200N**	1	0.15	**ORF1a:S2255L**	6	0.17
Maharashtra							**ORF1a:E910D**	1	0.15	**ORF1a:S2289L**	6	0.17
Maharashtra							**ORF1a:G696Y**	1	0.15	**ORF1a:V2290***	6	0.17
Maharashtra							**ORF1a:G697R**	1	0.15	**ORF1a:K2497F**	6	0.17
Maharashtra							**ORF1a:P361T**	1	0.15	**ORF1a:G2499H**	6	0.17
Maharashtra							**ORF1a:P4290S**	1	0.15	**ORF1a:S2500R**	6	0.17
Maharashtra							**ORF1a:L2253F**	1	0.15	**ORF1a:I2501***	6	0.17
Maharashtra							**ORF1a:M2254N**	1	0.15	**ORF1a:H2502***	6	0.17
Maharashtra							**ORF1a:S2255V**	1	0.15	**ORF1a:L2503C**	6	0.17
Maharashtra							**ORF1a:N2256Y**	1	0.15	**ORF1a:F2505W**	6	0.17
Maharashtra							**ORF1a:P309T**	1	0.15	**ORF1a:D2506S**	6	0.17
Maharashtra							**ORF1a:L2349V**	1	0.15	**ORF1a:M2683D**	6	0.17
Maharashtra							**ORF1a:M3780K**	1	0.15	**ORF1a:R2686***	6	0.17
Maharashtra							**ORF1a:V3782L**	1	0.15	**ORF1a:D2687P**	6	0.17
Maharashtra							**ORF1a:Y3783D**	1	0.15	**ORF1a:L2688W**	6	0.17
Maharashtra							**ORF1a:M3823L**	1	0.15	**ORF1a:A2690L**	6	0.17
Maharashtra							**ORF1a:L729I**	1	0.15	**ORF1a:C2691Y**	6	0.17
Maharashtra							**ORF1a:A1156S**	1	0.15	**ORF1a:I2692***	6	0.17
Maharashtra							**ORF1a:R1404C**	1	0.15	**ORF1a:C2694***	6	0.17
Maharashtra							**ORF1a:Y2099F**	1	0.15	**ORF1a:S2695C**	6	0.17
Maharashtra							**ORF1a:D571Y**	1	0.15	**ORF1a:F2827***	6	0.17
Maharashtra							**ORF1a:G715N**	1	0.15	**ORF1a:A2828F**	6	0.17
Maharashtra							**ORF1a:C720F**	1	0.15	**ORF1a:N2829***	6	0.17
Maharashtra							**ORF1a:V721Y**	1	0.15	**ORF1a:K2830H**	6	0.17
Maharashtra							**ORF1a:T1945N**	1	0.15	**ORF1a:H2831M**	6	0.17
Maharashtra							**ORF1a:A3518V**	1	0.15	**ORF1a:D2833***	6	0.17
Maharashtra							**ORF1a:T2846I**	1	0.15	**ORF1a:F2834P**	6	0.17
Maharashtra							**ORF1a:I222V**	1	0.15	**ORF1a:D2835A**	6	0.17
Maharashtra							**ORF1a:I473S**	1	0.15	**ORF1a:T2836W**	6	0.17
Maharashtra							**ORF1a:W612C**	1	0.15	**ORF1a:F2838***	6	0.17
Maharashtra							**ORF1a:E933G**	1	0.15	**ORF1a:S2839L**	6	0.17
Maharashtra							**ORF1a:A1306S**	1	0.15	**ORF1a:Q2840Y**	6	0.17
Maharashtra							**ORF1a:V2930L**	1	0.15	**ORF1a:R2841***	6	0.17
Maharashtra							**ORF1a:I3146T**	1	0.15	**ORF1a:G2842***	6	0.17
Maharashtra							**ORF1a:M174I**	1	0.15	**ORF1a:G2843Q**	6	0.17
Maharashtra							**ORF1a:T3058I**	1	0.15	**ORF1a:A3070L**	6	0.17
Maharashtra							**ORF1a:A599V**	1	0.15	**ORF1a:Q3562K**	6	0.17
Maharashtra							**ORF1a:C3059S**	1	0.15	**ORF1a:G3565E**	6	0.17
Maharashtra							**ORF1a:I3306V**	1	0.15	**ORF1a:V3566K**	6	0.17
Maharashtra							**ORF1a:A3523V**	1	0.15	**ORF1a:T3567N**	6	0.17
Maharashtra							**ORF1a:V1393M**	1	0.15	**ORF1a:F3568N**	6	0.17
Maharashtra							**ORF1a:P1497L**	1	0.15	**ORF1a:L3588Y**	6	0.17
Maharashtra							**ORF1a:M2565I**	1	0.15	**ORF1a:S3590P**	6	0.17
Maharashtra							**ORF1a:I3779V**	1	0.15	**ORF1a:L3592V**	6	0.17
Maharashtra							**ORF1a:A859V**	1	0.15	**ORF1a:L3594F**	6	0.17
Maharashtra							**ORF1a:P2792L**	1	0.15	**ORF1a:V3595S**	6	0.17
Maharashtra							**ORF1a:S318L**	1	0.15	**ORF1a:Q3596F**	6	0.17
Maharashtra							**ORF1a:Q2639L**	1	0.15	**ORF1a:T3598P**	6	0.17
Maharashtra							**ORF1a:T1589I**	1	0.15	**ORF1a:Q3599E**	6	0.17
Maharashtra							**ORF1a:I3758V**	1	0.15	**ORF1a:W3600Y**	6	0.17
Maharashtra							**ORF1a:W2327***	1	0.15	**ORF1a:V3763Q**	6	0.17
Maharashtra							**ORF1a:S3884L**	1	0.15	**ORF1a:E3764R**	6	0.17
Maharashtra							**ORF1a:P3952S**	1	0.15	**ORF1a:A2279***	6	0.17
Maharashtra							**ORF1a:T1017I**	1	0.15	**ORF1a:H3076Y**	6	0.17
Maharashtra							**ORF1a:D996Y**	1	0.15	**ORF1a:E1954W**	6	0.17
Maharashtra							**ORF1a:S1733G**	1	0.15	**ORF1a:P1961Y**	6	0.17
Maharashtra							**ORF1a:D3936Y**	1	0.15	**ORF1a:K1984***	6	0.17
Maharashtra							**ORF1a:F2354S**	1	0.15	**ORF1a:L1985T**	6	0.17
Maharashtra							**ORF1a:L177F**	1	0.15	**ORF1a:S2015***	6	0.17
Maharashtra							**ORF1a:L1162T**	1	0.15	**ORF1a:H2659Y**	6	0.17
Maharashtra							**ORF1a:R1163K**	1	0.15	**ORF1a:A498V**	6	0.17
Maharashtra							**ORF1a:V1164D**	1	0.15	**ORF1a:K680N**	6	0.17
Maharashtra							**ORF1a:L446F**	1	0.15	**ORF1a:D629L**	6	0.17
Maharashtra							**ORF1a:A482S**	1	0.15	**ORF1a:W630A**	6	0.17
Maharashtra							**ORF1a:S483P**	1	0.15	**ORF1a:E632F**	6	0.17
Maharashtra							**ORF1a:M1378I**	1	0.15	**ORF1a:E633M**	6	0.17
Maharashtra							**ORF1a:C2210Y**	1	0.15	**ORF1a:K915T**	6	0.17

Maharashtra							**ORF1a:L2211***	1	0.15	**ORF1a:A917Q**	6	0.17
Maharashtra							**ORF1a:E2212K**	1	0.15	**ORF1a:S918M**	6	0.17
Maharashtra							**ORF1a:Y3063S**	1	0.15	**ORF1a:H919R**	6	0.17
Maharashtra							**ORF1a:P3767L**	1	0.15	**ORF1a:G1101I**	6	0.17
Maharashtra							**ORF1a:S1978F**	1	0.15	**ORF1a:S1102T**	6	0.17
Maharashtra							**ORF1a:S3099L**	1	0.15	**ORF1a:C1103***	6	0.17
Maharashtra							**ORF1a:A702N**	1	0.15	**ORF1a:V1104L**	6	0.17
Maharashtra							**ORF1a:T1168I**	1	0.15	**ORF1a:S1106M**	6	0.17
Maharashtra							**ORF1a:Y1325***	1	0.15	**ORF1a:G1107D**	6	0.17
Maharashtra							**ORF1a:I1326M**	1	0.15	**ORF1a:V2027Q**	6	0.17
Maharashtra							**ORF1a:T2016I**	1	0.15	**ORF1a:L2105***	6	0.17
Maharashtra							**ORF1a:A2192H**	1	0.15	**ORF1a:A2248C**	6	0.17
Maharashtra							**ORF1a:A2785V**	1	0.15	**ORF1a:A2249L**	6	0.17
Maharashtra							**ORF1a:I2786V**	1	0.15	**ORF1a:L2343H**	6	0.17
Maharashtra							**ORF1a:V3057A**	1	0.15	**ORF1a:E3906F**	6	0.17
Maharashtra							**ORF1a:M3131N**	1	0.15	**ORF1a:E4019Y**	6	0.17
Maharashtra							**ORF1a:F3132T**	1	0.15	**ORF1a:K2315F**	6	0.17
Maharashtra							**ORF1a:T3287A**	1	0.15	**ORF1a:G3292N**	6	0.17
Maharashtra							**ORF1a:S2237K**	1	0.15	**ORF1a:G3433L**	6	0.17
Maharashtra							**ORF1a:V2238K**	1	0.15	**ORF1a:V3434L**	6	0.17
Maharashtra							**ORF1a:T2264K**	1	0.15	**ORF1a:T1637S**	6	0.17
Maharashtra							**ORF1a:G2265K**	1	0.15	**ORF1a:D1639C**	6	0.17
Maharashtra							**ORF1a:S2352K**	1	0.15	**ORF1a:P1640***	6	0.17
Maharashtra							**ORF1a:A2098V**	1	0.15	**ORF1a:S1641G**	6	0.17
Maharashtra							**ORF1a:A702V**	1	0.15	**ORF1a:L1643G**	6	0.17
Maharashtra							**ORF1a:L703Q**	1	0.15	**ORF1a:V2776L**	6	0.17
Maharashtra							**ORF1a:N704K**	1	0.15	**ORF1a:T2777H**	6	0.17
Maharashtra							**ORF1a:L705V**	1	0.15	**ORF1a:V2779***	6	0.17
Maharashtra							**ORF1a:G2265S**	1	0.15	**ORF1a:F2780H**	6	0.17
Maharashtra							**ORF1a:V544L**	1	0.15	**ORF1a:A2855C**	6	0.17
Maharashtra							**ORF1a:A1204T**	1	0.15	**ORF1a:A2909V**	6	0.17
Maharashtra							**ORF1a:V2356F**	1	0.15	**ORF1a:L3638Y**	6	0.17
Maharashtra							**ORF1a:H2357T**	1	0.15	**ORF1a:T1682S**	6	0.17
Maharashtra							**ORF1a:F2358K**	1	0.15	**ORF1a:L1683C**	6	0.17
Maharashtra							**ORF1a:G697Q**	1	0.15	**ORF1a:Q1684H**	6	0.17
Maharashtra							**ORF1a:T1761I**	1	0.15	**ORF1a:Q1685C**	6	0.17
Maharashtra							**ORF1a:G1294S**	1	0.15	**ORF1a:C1718***	6	0.17
Maharashtra							**ORF1a:T1597N**	1	0.15	**ORF1a:N1719C**	6	0.17
Maharashtra							**ORF1a:F3677L**	1	0.15	**ORF1a:K1720***	6	0.17
Maharashtra							**ORF1a:N1662S**	1	0.15	**ORF1a:T1721R**	6	0.17
Maharashtra							**ORF1a:T3284I**	1	0.15	**ORF1a:V1722N**	6	0.17
Maharashtra							**ORF1a:C3766***	1	0.15	**ORF1a:D1742V**	6	0.17
Maharashtra							**ORF1a:G3773N**	1	0.15	**ORF1a:C1744E**	6	0.17
Maharashtra							**ORF1a:N272I**	1	0.15	**ORF1a:K1745R**	6	0.17
Maharashtra							**ORF1a:E1205M**	1	0.15	**ORF1a:R1746G**	6	0.17
Maharashtra							**ORF1a:T2632I**	1	0.15	**ORF1a:L1748***	6	0.17
Maharashtra							**ORF1a:S1189G**	1	0.15	**ORF1a:Q1758L**	6	0.17
Maharashtra							**ORF1a:S1515F**	1	0.15	**ORF1a:Q1759W**	6	0.17
Maharashtra							**ORF1a:M315I**	1	0.15	**ORF1a:V3759F**	6	0.17
Maharashtra							**ORF1a:L1853F**	1	0.15	**ORF1a:S2048F**	6	0.17
Maharashtra							**ORF1a:E3932A**	1	0.15	**ORF1a:A486V**	6	0.17
Maharashtra							**ORF1a:P305S**	1	0.15	**ORF1a:V3097A**	6	0.17
Maharashtra							**ORF1a:A2199T**	1	0.15	**ORF1a:R2336K**	6	0.17
Maharashtra							**ORF1a:K2200Y**	1	0.15	**ORF1a:T3940I**	6	0.17
Maharashtra							**ORF1a:V627I**	1	0.15	**ORF1a:L1131F**	6	0.17
Maharashtra							**ORF1a:A2832S**	1	0.15	**ORF1a:L2039F**	6	0.17
Maharashtra							**ORF1a:G3286C**	1	0.15	**ORF1a:M1771I**	6	0.17
Maharashtra							**ORF1a:A4277T**	1	0.15	**ORF1a:T4087I**	6	0.17
Maharashtra							**ORF1a:R124C**	1	0.15	**ORF1a:N1137H**	6	0.17
Maharashtra							**ORF1a:V2393I**	1	0.15	**ORF1a:L1853F**	6	0.17
Maharashtra							**ORF1a:G415D**	1	0.15	**ORF1a:P1921Q**	6	0.17
Maharashtra							**ORF1a:V2061F**	1	0.15	**ORF1a:K2029N**	5	0.14
Maharashtra							**ORF1a:R184C**	1	0.15	**ORF1a:W2363Y**	5	0.14
Maharashtra							**ORF1a:L716F**	1	0.15	**ORF1a:L2364I**	5	0.14
Maharashtra							**ORF1a:E913D**	1	0.15	**ORF1a:M2365L**	5	0.14
Maharashtra							**ORF1a:L2062F**	1	0.15	**ORF1a:L2367V**	5	0.14
Maharashtra							**ORF1a:E2468T**	1	0.15	**ORF1a:I2369L**	5	0.14
Maharashtra							**ORF1a:V2469I**	1	0.15	**ORF1a:N2370G**	5	0.14
Maharashtra							**ORF1a:A2470T**	1	0.15	**ORF1a:V2372C**	5	0.14
Maharashtra							**ORF1a:Q2660R**	1	0.15	**ORF1a:Q2373G**	5	0.14
Maharashtra							**ORF1a:K1753R**	1	0.15	**ORF1a:M2374***	5	0.14
Maharashtra							**ORF1a:S1587L**	1	0.15	**ORF1a:A2375***	5	0.14
Maharashtra							**ORF1a:A776D**	1	0.15	**ORF1a:P2376L**	5	0.14

Maharashtra							**ORF1a:P777L**	1	0.15	**ORF1a:N2416***	5	0.14
Maharashtra							**ORF1a:L778F**	1	0.15	**ORF1a:R2417C**	5	0.14
Maharashtra							**ORF1a:V779I**	1	0.15	**ORF1a:A2418V**	5	0.14
Maharashtra							**ORF1a:V783F**	1	0.15	**ORF1a:Y2677K**	5	0.14
Maharashtra							**ORF1a:C784F**	1	0.15	**ORF1a:N2678L**	5	0.14
Maharashtra							**ORF1a:A803L**	1	0.15	**ORF1a:E2681L**	5	0.14
Maharashtra							**ORF1a:T2777K**	1	0.15	**ORF1a:N2682V**	5	0.14
Maharashtra							**ORF1a:L2778N**	1	0.15	**ORF1a:M2683L**	5	0.14
Maharashtra							**ORF1a:S2804N**	1	0.15	**ORF1a:K2705V**	5	0.14
Maharashtra							**ORF1a:E2805K**	1	0.15	**ORF1a:S2706V**	5	0.14
Maharashtra							**ORF1a:G3446K**	1	0.15	**ORF1a:H2707R**	5	0.14
Maharashtra							**ORF1a:A3474N**	1	0.15	**ORF1a:C2851S**	5	0.14
Maharashtra							**ORF1a:V3475K**	1	0.15	**ORF1a:L2853Q**	5	0.14
Maharashtra										**ORF1a:I2854E**	5	0.14
Maharashtra										**ORF1a:A2855K**	5	0.14
Maharashtra										**ORF1a:A2856W**	5	0.14
Maharashtra										**ORF1a:E3188M**	5	0.14
Maharashtra										**ORF1a:M3189C**	5	0.14
Maharashtra										**ORF1a:L3191Y**	5	0.14
Maharashtra										**ORF1a:K3192L**	5	0.14
Maharashtra										**ORF1a:S3195N**	5	0.14
Maharashtra										**ORF1a:D3196I**	5	0.14
Maharashtra										**ORF1a:V3197I**	5	0.14
Maharashtra										**ORF1a:L3198D**	5	0.14
Maharashtra										**ORF1a:L3199T**	5	0.14
Maharashtra										**ORF1a:A3220F**	5	0.14
Maharashtra										**ORF1a:D3222I**	5	0.14
Maharashtra										**ORF1a:T3223S**	5	0.14
Maharashtra										**ORF1a:K3351I**	5	0.14
Maharashtra										**ORF1a:I3369H**	5	0.14
Maharashtra										**ORF1a:Q3370L**	5	0.14
Maharashtra										**ORF1a:G3372I**	5	0.14
Maharashtra										**ORF1a:T3374L**	5	0.14
Maharashtra										**ORF1a:S3376H**	5	0.14
Maharashtra										**ORF1a:V3377H**	5	0.14
Maharashtra										**ORF1a:N3396V**	5	0.14
Maharashtra										**ORF1a:Y3607L**	5	0.14
Maharashtra										**ORF1a:E3608L**	5	0.14
Maharashtra										**ORF1a:F3611L**	5	0.14
Maharashtra										**ORF1a:M3616L**	5	0.14
Maharashtra										**ORF1a:Y3719I**	5	0.14
Maharashtra										**ORF1a:W3734L**	5	0.14
Maharashtra										**ORF1a:I3737T**	5	0.14
Maharashtra										**ORF1a:C3778S**	5	0.14
Maharashtra										**ORF1a:S3916R**	5	0.14
Maharashtra										**ORF1a:L3919***	5	0.14
Maharashtra										**ORF1a:S3920T**	5	0.14
Maharashtra										**ORF1a:M3921***	5	0.14
Maharashtra										**ORF1a:Q3922T**	5	0.14
Maharashtra										**ORF1a:G3923S**	5	0.14
Maharashtra										**ORF1a:A3924F**	5	0.14
Maharashtra										**ORF1a:L3930K**	5	0.14
Maharashtra										**ORF1a:R3938W**	5	0.14
Maharashtra										**ORF1a:A3943P**	5	0.14
Maharashtra										**ORF1a:I3944Y**	5	0.14
Maharashtra										**ORF1a:A3945K**	5	0.14
Maharashtra										**ORF1a:E3947***	5	0.14
Maharashtra										**ORF1a:F3948P**	5	0.14
Maharashtra										**ORF1a:S3949Q**	5	0.14
Maharashtra										**ORF1a:V3976I**	5	0.14
Maharashtra										**ORF1a:K3979L**	5	0.14
Maharashtra										**ORF1a:L3980F**	5	0.14
Maharashtra										**ORF1a:K3981L**	5	0.14
Maharashtra										**ORF1a:N3985T**	5	0.14
Maharashtra										**ORF1a:K4000W**	5	0.14
Maharashtra										**ORF1a:E4002I**	5	0.14
Maharashtra										**ORF1a:Q4011L**	5	0.14
Maharashtra										**ORF1a:M4012***	5	0.14
Maharashtra										**ORF1a:Y4013P**	5	0.14
Maharashtra										**ORF1a:Q4015C**	5	0.14
Maharashtra										**ORF1a:A4016I**	5	0.14
Maharashtra										**ORF1a:R4017N**	5	0.14
Maharashtra										**ORF1a:S4018R**	5	0.14

Maharashtra										ORF1a:E4019L	5	0.14
Maharashtra										ORF1a:K4021L	5	0.14
Maharashtra										ORF1a:H165V	5	0.14
Maharashtra										ORF1a:N178T	5	0.14
Maharashtra										ORF1a:G179E	5	0.14
Maharashtra										ORF1a:Y304E	5	0.14
Maharashtra										ORF1a:P305F	5	0.14
Maharashtra										ORF1a:V306D	5	0.14
Maharashtra										ORF1a:A307L	5	0.14
Maharashtra										ORF1a:P309I	5	0.14
Maharashtra										ORF1a:N310Q	5	0.14
Maharashtra										ORF1a:E311L	5	0.14
Maharashtra										ORF1a:C312R	5	0.14
Maharashtra										ORF1a:R579H	5	0.14
Maharashtra										ORF1a:N840*	5	0.14
Maharashtra										ORF1a:M3087H	5	0.14
Maharashtra										ORF1a:T3113V	5	0.14
Maharashtra										ORF1a:T3117G	5	0.14
Maharashtra										ORF1a:N3118W	5	0.14
Maharashtra										ORF1a:D3119L	5	0.14
Maharashtra										ORF1a:V3120C	5	0.14
Maharashtra										ORF1a:F3122H	5	0.14
Maharashtra										ORF1a:K3678*	5	0.14
Maharashtra										ORF1a:L3679A	5	0.14
Maharashtra										ORF1a:V3700N	5	0.14
Maharashtra										ORF1a:Y3701P	5	0.14
Maharashtra										ORF1a:D3702Y	5	0.14
Maharashtra										ORF1a:G3704S	5	0.14
Maharashtra										ORF1a:A3705K	5	0.14
Maharashtra										ORF1a:R3706N	5	0.14
Maharashtra										ORF1a:R3707C	5	0.14
Maharashtra										ORF1a:M3712E	5	0.14
Maharashtra										ORF1a:L3715D	5	0.14
Maharashtra										ORF1a:E4097C	5	0.14
Maharashtra										ORF1a:L1131*	5	0.14
Maharashtra										ORF1a:V1164Y	5	0.14
Maharashtra										ORF1a:T1474S	5	0.14
Maharashtra										ORF1a:T1597S	5	0.14
Maharashtra										ORF1a:Y1598N	5	0.14
Maharashtra										ORF1a:D1600F	5	0.14
Maharashtra										ORF1a:T1854I	5	0.14
Maharashtra										ORF1a:V2436N	5	0.14
Maharashtra										ORF1a:D2573Q	5	0.14
Maharashtra										ORF1a:Q2574S	5	0.14
Maharashtra										ORF1a:L2576Y	5	0.14
Maharashtra										ORF1a:D2579*	5	0.14
Maharashtra										ORF1a:V2580*	5	0.14
Maharashtra										ORF1a:G2581C	5	0.14
Maharashtra										ORF1a:E2653R	5	0.14
Maharashtra										ORF1a:L2655C	5	0.14
Maharashtra										ORF1a:K2656*	5	0.14
Maharashtra										ORF1a:L2657M	5	0.14
Maharashtra										ORF1a:N2678*	5	0.14
Maharashtra										ORF1a:V2680H	5	0.14
Maharashtra										ORF1a:E2681D	5	0.14
Maharashtra										ORF1a:N2682T	5	0.14
Maharashtra										ORF1a:M2683P	5	0.14
Maharashtra										ORF1a:T2684*	5	0.14
Maharashtra										ORF1a:R2686W	5	0.14
Maharashtra										ORF1a:D2687C	5	0.14
Maharashtra										ORF1a:C2691S	5	0.14
Maharashtra										ORF1a:I2692Y	5	0.14
Maharashtra										ORF1a:D2693*	5	0.14
Maharashtra										ORF1a:T753I	5	0.14
Maharashtra										ORF1a:L3829F	5	0.14
Maharashtra										ORF1a:T760I	5	0.14
Maharashtra										ORF1a:L4182F	5	0.14
Maharashtra										ORF1a:P2929L	5	0.14
Maharashtra										ORF1a:M1586I	5	0.14
Maharashtra										ORF1a:S3950N	5	0.14
Maharashtra										ORF1a:T4159D	5	0.14
Maharashtra										ORF1a:Q4160V	5	0.14
Maharashtra										ORF1a:T4161L	5	0.14

Maharashtra										**ORF1a:A4162C**	5	0.14
Maharashtra										**ORF1a:T4164L**	5	0.14
Maharashtra										**ORF1a:D4165Q**	5	0.14
Maharashtra										**ORF1a:D4166H**	5	0.14
Maharashtra										**ORF1a:A4168K**	5	0.14
Maharashtra										**ORF1a:L4169G**	5	0.14
Maharashtra										**ORF1a:A4170R**	5	0.14
Maharashtra										**ORF1a:Y4171***	5	0.14
Maharashtra										**ORF1a:Y4172V**	5	0.14
Maharashtra										**ORF1a:N4173C**	5	0.14
Maharashtra										**ORF1a:T4175C**	5	0.14
Maharashtra										**ORF1a:G4177V**	5	0.14
Maharashtra										**ORF1a:G4222T**	5	0.14
Maharashtra										**ORF1a:K4224***	5	0.14
Maharashtra										**ORF1a:K4226C**	5	0.14
Maharashtra										**ORF1a:D144A**	5	0.14
Maharashtra										**ORF1a:D223L**	5	0.14
Maharashtra										**ORF1a:A260L**	5	0.14
Maharashtra										**ORF1a:A372K**	5	0.14
Maharashtra										**ORF1a:E444K**	5	0.14
Maharashtra										**ORF1a:D448T**	5	0.14
Maharashtra										**ORF1a:L450F**	5	0.14
Maharashtra										**ORF1a:P532L**	5	0.14
Maharashtra										**ORF1a:S574***	5	0.14
Maharashtra										**ORF1a:Y860L**	5	0.14
Maharashtra										**ORF1a:V862I**	5	0.14
Maharashtra										**ORF1a:E863Q**	5	0.14
Maharashtra										**ORF1a:G865S**	5	0.14
Maharashtra										**ORF1a:T866S**	5	0.14
Maharashtra										**ORF1a:E867P**	5	0.14
Maharashtra										**ORF1a:A917M**	5	0.14
Maharashtra										**ORF1a:H919Q**	5	0.14
Maharashtra										**ORF1a:D996V**	5	0.14
Maharashtra										**ORF1a:Q998K**	5	0.14
Maharashtra										**ORF1a:T1000R**	5	0.14
Maharashtra										**ORF1a:T1004Q**	5	0.14
Maharashtra										**ORF1a:V1006L**	5	0.14
Maharashtra										**ORF1a:E1007F**	5	0.14
Maharashtra										**ORF1a:V1008K**	5	0.14
Maharashtra										**ORF1a:T1297K**	5	0.14
Maharashtra										**ORF1a:A1298R**	5	0.14
Maharashtra										**ORF1a:F1930Q**	5	0.14
Maharashtra										**ORF1a:D1933***	5	0.14
Maharashtra										**ORF1a:N1934***	5	0.14
Maharashtra										**ORF1a:I1935F**	5	0.14
Maharashtra										**ORF1a:F1937P**	5	0.14
Maharashtra										**ORF1a:A1938V**	5	0.14
Maharashtra										**ORF1a:D1939N**	5	0.14
Maharashtra										**ORF1a:D1940W**	5	0.14
Maharashtra										**ORF1a:Q1943***	5	0.14
Maharashtra										**ORF1a:L1944E**	5	0.14
Maharashtra										**ORF1a:G1946C**	5	0.14
Maharashtra										**ORF1a:Y1947F**	5	0.14
Maharashtra										**ORF1a:K1949R**	5	0.14
Maharashtra										**ORF1a:P1950A**	5	0.14
Maharashtra										**ORF1a:A1951***	5	0.14
Maharashtra										**ORF1a:T1958Y**	5	0.14
Maharashtra										**ORF1a:F1959I**	5	0.14
Maharashtra										**ORF1a:A2732C**	5	0.14
Maharashtra										**ORF1a:Y3502T**	5	0.14
Maharashtra										**ORF1a:E3503R**	5	0.14
Maharashtra										**ORF1a:S3517C**	5	0.14
Maharashtra										**ORF1a:A3518***	5	0.14
Maharashtra										**ORF1a:Q3519H**	5	0.14
Maharashtra										**ORF1a:G3521F**	5	0.14
Maharashtra										**ORF1a:G3541K**	5	0.14
Maharashtra										**ORF1a:T3543F**	5	0.14
Maharashtra										**ORF1a:V4102S**	5	0.14
Maharashtra										**ORF1a:D4103T**	5	0.14
Maharashtra										**ORF1a:A4104***	5	0.14
Maharashtra										**ORF1a:D4105***	5	0.14
Maharashtra										**ORF1a:S4106N**	5	0.14
Maharashtra										**ORF1a:K4107***	5	0.14

Maharashtra										**ORF1a:I4108Y**	5	0.14
Maharashtra										**ORF1a:N2006R**	5	0.14
Maharashtra										**ORF1a:W2008N**	5	0.14
Maharashtra										**ORF1a:C2009Q**	5	0.14
Maharashtra										**ORF1a:G2091M**	5	0.14
Maharashtra										**ORF1a:D2094I**	5	0.14
Maharashtra										**ORF1a:A2199S**	5	0.14
Maharashtra										**ORF1a:Y2504M**	5	0.14
Maharashtra										**ORF1a:F2505K**	5	0.14
Maharashtra										**ORF1a:F3403V**	5	0.14
Maharashtra										**ORF1a:N3405***	5	0.14
Maharashtra										**ORF1a:Y1868L**	5	0.14
Maharashtra										**ORF1a:K1869R**	5	0.14
Maharashtra										**ORF1a:E1870M**	5	0.14
Maharashtra										**ORF1a:N1871F**	5	0.14
Maharashtra										**ORF1a:S2293I**	5	0.14
Maharashtra										**ORF1a:G2294L**	5	0.14
Maharashtra										**ORF1a:D2296***	5	0.14
Maharashtra										**ORF1a:S2297K**	5	0.14
Maharashtra										**ORF1a:D2299Y**	5	0.14
Maharashtra										**ORF1a:T2300K**	5	0.14
Maharashtra										**ORF1a:Y2301L**	5	0.14
Maharashtra										**ORF1a:Y3154V**	5	0.14
Maharashtra										**ORF1a:W3155F**	5	0.14
Maharashtra										**ORF1a:F3156P**	5	0.14
Maharashtra										**ORF1a:F3157Q**	5	0.14
Maharashtra										**ORF1a:N3159I**	5	0.14
Maharashtra										**ORF1a:Y3160S**	5	0.14
Maharashtra										**ORF1a:L3161I**	5	0.14
Maharashtra										**ORF1a:K3162G**	5	0.14
Maharashtra										**ORF1a:R3163S**	5	0.14
Maharashtra										**ORF1a:V1931K**	5	0.14
Maharashtra										**ORF1a:C1932P**	5	0.14
Maharashtra										**ORF1a:D1933V**	5	0.14
Maharashtra										**ORF1a:K1956***	5	0.14
Maharashtra										**ORF1a:V1957E**	5	0.14
Maharashtra										**ORF1a:F1959C**	5	0.14
Maharashtra										**ORF1a:L446T**	5	0.14
Maharashtra										**ORF1a:N447I**	5	0.14
Maharashtra										**ORF1a:D448Q**	5	0.14
Maharashtra										**ORF1a:F908I**	5	0.14
Maharashtra										**ORF1a:D909***	5	0.14
Maharashtra										**ORF1a:E910M**	5	0.14
Maharashtra										**ORF1a:Q1003K**	5	0.14
Maharashtra										**ORF1a:I1005A**	5	0.14
Maharashtra										**ORF1a:E1007R**	5	0.14
Maharashtra										**ORF1a:V1008T**	5	0.14
Maharashtra										**ORF1a:Q1009I**	5	0.14
Maharashtra										**ORF1a:P1010R**	5	0.14
Maharashtra										**ORF1a:E1013L**	5	0.14
Maharashtra										**ORF1a:M1014F**	5	0.14
Maharashtra										**ORF1a:E1015R**	5	0.14
Maharashtra										**ORF1a:K1098N**	5	0.14
Maharashtra										**ORF1a:V1099L**	5	0.14
Maharashtra										**ORF1a:G1100M**	5	0.14
Maharashtra										**ORF1a:M3661I**	5	0.14
Maharashtra										**ORF1a:Q4015G**	5	0.14
Maharashtra										**ORF1a:A4016***	5	0.14
Maharashtra										**ORF1a:R4017S**	5	0.14
Maharashtra										**ORF1a:K4021C**	5	0.14
Maharashtra										**ORF1a:N2272L**	5	0.14
Maharashtra										**ORF1a:F2338H**	5	0.14
Maharashtra										**ORF1a:Y2339***	5	0.14
Maharashtra										**ORF1a:D2582I**	5	0.14
Maharashtra										**ORF1a:K2589Y**	5	0.14
Maharashtra										**ORF1a:M2590V**	5	0.14
Maharashtra										**ORF1a:D2592I**	5	0.14
Maharashtra										**ORF1a:A2593N**	5	0.14
Maharashtra										**ORF1a:Y2594F**	5	0.14
Maharashtra										**ORF1a:V2595***	5	0.14
Maharashtra										**ORF1a:I3341W**	5	0.14
Maharashtra										**ORF1a:G3342***	5	0.14
Maharashtra										**ORF1a:C3408***	5	0.14

Maharashtra										ORF1a:S3410L	5	0.14
Maharashtra										ORF1a:V3411C	5	0.14
Maharashtra										ORF1a:L3584V	5	0.14
Maharashtra										ORF1a:V3740Y	5	0.14
Maharashtra										ORF1a:T3741N	5	0.14
Maharashtra										ORF1a:S3742C	5	0.14
Maharashtra										ORF1a:N3743H	5	0.14
Maharashtra										ORF1a:Y3744V	5	0.14
Maharashtra										ORF1a:S3745F	5	0.14
Maharashtra										ORF1a:L4070I	5	0.14
Maharashtra										ORF1a:V4073Y	5	0.14
Maharashtra										ORF1a:C2851W	5	0.14
Maharashtra										ORF1a:P2852V	5	0.14
Maharashtra										ORF1a:I2854S	5	0.14
Maharashtra										ORF1a:A2856L	5	0.14
Maharashtra										ORF1a:Y2504K	5	0.14
Maharashtra										ORF1a:F2505L	5	0.14
Maharashtra										ORF1a:D2506V	5	0.14
Maharashtra										ORF1a:A2508R	5	0.14
Maharashtra										ORF1a:Q2510M	5	0.14
Maharashtra										ORF1a:Y2513*	5	0.14
Maharashtra										ORF1a:Y1634*	5	0.14
Maharashtra										ORF1a:M2259R	5	0.14
Maharashtra										ORF1a:V3475I	5	0.14
Maharashtra										ORF1a:L4391I	5	0.14
Maharashtra										ORF1a:T609I	5	0.14
Maharashtra										ORF1a:T2124I	5	0.14
Maharashtra										ORF1a:T1597I	5	0.14
Maharashtra										ORF1a:K202R	5	0.14
Maharashtra										ORF1a:I2858V	5	0.14
Maharashtra										ORF1a:V2613F	5	0.14
Maharashtra										ORF1a:L580F	5	0.14
Maharashtra										ORF1a:V1629F	5	0.14
Maharashtra										ORF1a:E2861R	5	0.14
Maharashtra										ORF1a:V2862S	5	0.14
Maharashtra										ORF1a:N2140I	5	0.14
Maharashtra										ORF1a:T554I	5	0.14
Maharashtra										ORF1a:S376L	5	0.14
Maharashtra										ORF1a:T4364I	5	0.14
Maharashtra										ORF1a:P1921L	5	0.14
Maharashtra										ORF1a:K261N	5	0.14
Maharashtra										ORF1a:A1283V	5	0.14
Maharashtra										ORF1a:T1246A	5	0.14
Maharashtra										ORF1a:M3652I	5	0.14
Maharashtra										ORF1a:L2778F	5	0.14
Maharashtra										ORF1a:T4083M	5	0.14
Maharashtra										ORF1a:V1057F	5	0.14
Maharashtra										ORF1a:P3395L	5	0.14
Maharashtra										ORF1a:V1169A	4	0.11
Maharashtra										ORF1a:V3751I	4	0.11
Maharashtra										ORF1a:T350I	4	0.11
Maharashtra										ORF1a:P1497S	4	0.11
Maharashtra										ORF1a:L3161T	4	0.11
Maharashtra										ORF1a:Q3507W	4	0.11
Maharashtra										ORF1a:D3508L	4	0.11
Maharashtra										ORF1a:H3509*	4	0.11
Maharashtra										ORF1a:V3510S	4	0.11
Maharashtra										ORF1a:D3511T	4	0.11
Maharashtra										ORF1a:A3548H	4	0.11
Maharashtra										ORF1a:N3609W	4	0.11
Maharashtra										ORF1a:A3610V	4	0.11
Maharashtra										ORF1a:P3613L	4	0.11
Maharashtra										ORF1a:F3614C	4	0.11
Maharashtra										ORF1a:A3615L	4	0.11
Maharashtra										ORF1a:G3617L	4	0.11
Maharashtra										ORF1a:I3618Q	4	0.11
Maharashtra										ORF1a:I3619*	4	0.11
Maharashtra										ORF1a:M3621L	4	0.11
Maharashtra										ORF1a:A3623N	4	0.11
Maharashtra										ORF1a:F3624I	4	0.11
Maharashtra										ORF1a:M3627H	4	0.11
Maharashtra										ORF1a:F3635L	4	0.11
Maharashtra										ORF1a:R3662S	4	0.11

Maharashtra										**ORF1a:T3716M**	4	0.11
Maharashtra										**ORF1a:V3718***	4	0.11
Maharashtra										**ORF1a:V3721P**	4	0.11
Maharashtra										**ORF1a:Y3722F**	4	0.11
Maharashtra										**ORF1a:Y3723P**	4	0.11
Maharashtra										**ORF1a:G3724C**	4	0.11
Maharashtra										**ORF1a:N3725G**	4	0.11
Maharashtra										**ORF1a:A3726L**	4	0.11
Maharashtra										**ORF1a:I3731***	4	0.11
Maharashtra										**ORF1a:I3738T**	4	0.11
Maharashtra										**ORF1a:S3739Q**	4	0.11
Maharashtra										**ORF1a:T3741***	4	0.11
Maharashtra										**ORF1a:S3742L**	4	0.11
Maharashtra										**ORF1a:N3743Q**	4	0.11
Maharashtra										**ORF1a:Y3744L**	4	0.11
Maharashtra										**ORF1a:G3746C**	4	0.11
Maharashtra										**ORF1a:V3747F**	4	0.11
Maharashtra										**ORF1a:V3748W**	4	0.11
Maharashtra										**ORF1a:T3749P**	4	0.11
Maharashtra										**ORF1a:T3750E**	4	0.11
Maharashtra										**ORF1a:M3752L**	4	0.11
Maharashtra										**ORF1a:Q3777F**	4	0.11
Maharashtra										**ORF1a:I3779S**	4	0.11
Maharashtra										**ORF1a:M3780***	4	0.11
Maharashtra										**ORF1a:Y3783I**	4	0.11
Maharashtra										**ORF1a:C3784H**	4	0.11
Maharashtra										**ORF1a:G3787A**	4	0.11
Maharashtra										**ORF1a:Y3788S**	4	0.11
Maharashtra										**ORF1a:C3790V**	4	0.11
Maharashtra										**ORF1a:T3791Y**	4	0.11
Maharashtra										**ORF1a:C3792S**	4	0.11
Maharashtra										**ORF1a:F3794A**	4	0.11
Maharashtra										**ORF1a:G3795T**	4	0.11
Maharashtra										**ORF1a:F3797D**	4	0.11
Maharashtra										**ORF1a:C3798***	4	0.11
Maharashtra										**ORF1a:E3819V**	4	0.11
Maharashtra										**ORF1a:R3821D**	4	0.11
Maharashtra										**ORF1a:M3823S**	4	0.11
Maharashtra										**ORF1a:N3824H**	4	0.11
Maharashtra										**ORF1a:S3825P**	4	0.11
Maharashtra										**ORF1a:Q3826R**	4	0.11
Maharashtra										**ORF1a:G3827I**	4	0.11
Maharashtra										**ORF1a:L3828A**	4	0.11
Maharashtra										**ORF1a:L3829***	4	0.11
Maharashtra										**ORF1a:P3830M**	4	0.11
Maharashtra										**ORF1a:K3832S**	4	0.11
Maharashtra										**ORF1a:K3839T**	4	0.11
Maharashtra										**ORF1a:I3842C**	4	0.11
Maharashtra										**ORF1a:K3843W**	4	0.11
Maharashtra										**ORF1a:L3844V**	4	0.11
Maharashtra										**ORF1a:T3857N**	4	0.11
Maharashtra										**ORF1a:Q3859V**	4	0.11
Maharashtra										**ORF1a:A3907T**	4	0.11
Maharashtra										**ORF1a:Q4110K**	4	0.11
Maharashtra										**ORF1a:N4121K**	4	0.11
Maharashtra										**ORF1a:Q575R**	4	0.11
Maharashtra										**ORF1a:L205M**	4	0.11
Maharashtra										**ORF1a:R207T**	4	0.11
Maharashtra										**ORF1a:K210S**	4	0.11
Maharashtra										**ORF1a:Q252G**	4	0.11
Maharashtra										**ORF1a:P254R**	4	0.11
Maharashtra										**ORF1a:F255N**	4	0.11
Maharashtra										**ORF1a:E256V**	4	0.11
Maharashtra										**ORF1a:I257L**	4	0.11
Maharashtra										**ORF1a:L259R**	4	0.11
Maharashtra										**ORF1a:D466L**	4	0.11
Maharashtra										**ORF1a:F467V**	4	0.11
Maharashtra										**ORF1a:K468T**	4	0.11
Maharashtra										**ORF1a:E471L**	4	0.11
Maharashtra										**ORF1a:E472M**	4	0.11
Maharashtra										**ORF1a:I473K**	4	0.11
Maharashtra										**ORF1a:A474R**	4	0.11
Maharashtra										**ORF1a:I475S**	4	0.11

Maharashtra										**ORF1a:I476P**	4	0.11
Maharashtra										**ORF1a:A566L**	4	0.11
Maharashtra										**ORF1a:I567L**	4	0.11
Maharashtra										**ORF1a:T568K**	4	0.11
Maharashtra										**ORF1a:D571H**	4	0.11
Maharashtra										**ORF1a:G572S**	4	0.11
Maharashtra										**ORF1a:S574H**	4	0.11
Maharashtra										**ORF1a:Q575***	4	0.11
Maharashtra										**ORF1a:Y576D**	4	0.11
Maharashtra										**ORF1a:Q991S**	4	0.11
Maharashtra										**ORF1a:T3489I**	4	0.11
Maharashtra										**ORF1a:L3490M**	4	0.11
Maharashtra										**ORF1a:D3681L**	4	0.11
Maharashtra										**ORF1a:V3683Y**	4	0.11
Maharashtra										**ORF1a:M3684V**	4	0.11
Maharashtra										**ORF1a:Y3685C**	4	0.11
Maharashtra										**ORF1a:A3686I**	4	0.11
Maharashtra										**ORF1a:A3688C**	4	0.11
Maharashtra										**ORF1a:V3689S**	4	0.11
Maharashtra										**ORF1a:L3711***	4	0.11
Maharashtra										**ORF1a:N3713S**	4	0.11
Maharashtra										**ORF1a:T1001S**	4	0.11
Maharashtra										**ORF1a:H1067***	4	0.11
Maharashtra										**ORF1a:V1104T**	4	0.11
Maharashtra										**ORF1a:L1148F**	4	0.11
Maharashtra										**ORF1a:L1149***	4	0.11
Maharashtra										**ORF1a:G1152R**	4	0.11
Maharashtra										**ORF1a:I1153S**	4	0.11
Maharashtra										**ORF1a:F1154S**	4	0.11
Maharashtra										**ORF1a:G1155T**	4	0.11
Maharashtra										**ORF1a:A1156C**	4	0.11
Maharashtra										**ORF1a:D1157T**	4	0.11
Maharashtra										**ORF1a:P1158I**	4	0.11
Maharashtra										**ORF1a:H1160I**	4	0.11
Maharashtra										**ORF1a:L1162C**	4	0.11
Maharashtra										**ORF1a:T1246R**	4	0.11
Maharashtra										**ORF1a:L1249V**	4	0.11
Maharashtra										**ORF1a:I1276H**	4	0.11
Maharashtra										**ORF1a:Q1332S**	4	0.11
Maharashtra										**ORF1a:L1346F**	4	0.11
Maharashtra										**ORF1a:K1350H**	4	0.11
Maharashtra										**ORF1a:Y1418***	4	0.11
Maharashtra										**ORF1a:E1443N**	4	0.11
Maharashtra										**ORF1a:V1471F**	4	0.11
Maharashtra										**ORF1a:P1472G**	4	0.11
Maharashtra										**ORF1a:A1473R**	4	0.11
Maharashtra										**ORF1a:V1475C**	4	0.11
Maharashtra										**ORF1a:S1478L**	4	0.11
Maharashtra										**ORF1a:G1524C**	4	0.11
Maharashtra										**ORF1a:A1712L**	4	0.11
Maharashtra										**ORF1a:N1749V**	4	0.11
Maharashtra										**ORF1a:V1750S**	4	0.11
Maharashtra										**ORF1a:V1751T**	4	0.11
Maharashtra										**ORF1a:K1753Q**	4	0.11
Maharashtra										**ORF1a:T1754F**	4	0.11
Maharashtra										**ORF1a:C1755R**	4	0.11
Maharashtra										**ORF1a:G1756F**	4	0.11
Maharashtra										**ORF1a:Q1757L**	4	0.11
Maharashtra										**ORF1a:Q1759K**	4	0.11
Maharashtra										**ORF1a:T1760S**	4	0.11
Maharashtra										**ORF1a:T1761L**	4	0.11
Maharashtra										**ORF1a:L1762E**	4	0.11
Maharashtra										**ORF1a:K1763R**	4	0.11
Maharashtra										**ORF1a:E1766T**	4	0.11
Maharashtra										**ORF1a:S2942L**	4	0.11
Maharashtra										**ORF1a:Y2945H**	4	0.11
Maharashtra										**ORF1a:E2946G**	4	0.11
Maharashtra										**ORF1a:S2947W**	4	0.11
Maharashtra										**ORF1a:L2969Y**	4	0.11
Maharashtra										**ORF1a:E2970S**	4	0.11
Maharashtra										**ORF1a:G2971I**	4	0.11
Maharashtra										**ORF1a:A3143L**	4	0.11
Maharashtra										**ORF1a:I3145N**	4	0.11

Maharashtra										**ORF1a:N3291Y**	4	0.11
Maharashtra										**ORF1a:D3319H**	4	0.11
Maharashtra										**ORF1a:T614I**	4	0.11
Maharashtra										**ORF1a:S1361F**	4	0.11
Maharashtra										**ORF1a:Y2968H**	4	0.11
Maharashtra										**ORF1a:V2892F**	4	0.11
Maharashtra										**ORF1a:T866I**	4	0.11
Maharashtra										**ORF1a:A1352V**	4	0.11
Maharashtra										**ORF1a:Q3942***	4	0.11
Maharashtra										**ORF1a:A3943R**	4	0.11
Maharashtra										**ORF1a:I3944N**	4	0.11
Maharashtra										**ORF1a:S3946G**	4	0.11
Maharashtra										**ORF1a:E3947Q**	4	0.11
Maharashtra										**ORF1a:F3948Q**	4	0.11
Maharashtra										**ORF1a:S3949G**	4	0.11
Maharashtra										**ORF1a:P371S**	4	0.11
Maharashtra										**ORF1a:E57***	4	0.11
Maharashtra										**ORF1a:Y118V**	4	0.11
Maharashtra										**ORF1a:K120T**	4	0.11
Maharashtra										**ORF1a:N128I**	4	0.11
Maharashtra										**ORF1a:A138T**	4	0.11
Maharashtra										**ORF1a:I238T**	4	0.11
Maharashtra										**ORF1a:W240A**	4	0.11
Maharashtra										**ORF1a:Y241M**	4	0.11
Maharashtra										**ORF1a:T242N**	4	0.11
Maharashtra										**ORF1a:E243C**	4	0.11
Maharashtra										**ORF1a:K258H**	4	0.11
Maharashtra										**ORF1a:K262L**	4	0.11
Maharashtra										**ORF1a:F263N**	4	0.11
Maharashtra										**ORF1a:D264W**	4	0.11
Maharashtra										**ORF1a:T265Q**	4	0.11
Maharashtra										**ORF1a:F266R**	4	0.11
Maharashtra										**ORF1a:E269N**	4	0.11
Maharashtra										**ORF1a:C270V**	4	0.11
Maharashtra										**ORF1a:P271Q**	4	0.11
Maharashtra										**ORF1a:N272I**	4	0.11
Maharashtra										**ORF1a:F273L**	4	0.11
Maharashtra										**ORF1a:V274Y**	4	0.11
Maharashtra										**ORF1a:L277F**	4	0.11
Maharashtra										**ORF1a:G440E**	4	0.11
Maharashtra										**ORF1a:E441K**	4	0.11
Maharashtra										**ORF1a:G442V**	4	0.11
Maharashtra										**ORF1a:S443P**	4	0.11
Maharashtra										**ORF1a:N447M**	4	0.11
Maharashtra										**ORF1a:N449T**	4	0.11
Maharashtra										**ORF1a:E452K**	4	0.11
Maharashtra										**ORF1a:I453Y**	4	0.11
Maharashtra										**ORF1a:L454S**	4	0.11
Maharashtra										**ORF1a:Q455K**	4	0.11
Maharashtra										**ORF1a:E457R**	4	0.11
Maharashtra										**ORF1a:N460T**	4	0.11
Maharashtra										**ORF1a:I461L**	4	0.11
Maharashtra										**ORF1a:D495L**	4	0.11
Maharashtra										**ORF1a:Y496W**	4	0.11
Maharashtra										**ORF1a:A498L**	4	0.11
Maharashtra										**ORF1a:F499***	4	0.11
Maharashtra										**ORF1a:Q501V**	4	0.11
Maharashtra										**ORF1a:I502W**	4	0.11
Maharashtra										**ORF1a:V503I**	4	0.11
Maharashtra										**ORF1a:E504I**	4	0.11
Maharashtra										**ORF1a:S505K**	4	0.11
Maharashtra										**ORF1a:C506H**	4	0.11
Maharashtra										**ORF1a:G507S**	4	0.11
Maharashtra										**ORF1a:Q526V**	4	0.11
Maharashtra										**ORF1a:K527P**	4	0.11
Maharashtra										**ORF1a:S528G**	4	0.11
Maharashtra										**ORF1a:S531R**	4	0.11
Maharashtra										**ORF1a:L552V**	4	0.11
Maharashtra										**ORF1a:E553L**	4	0.11
Maharashtra										**ORF1a:T554Y**	4	0.11
Maharashtra										**ORF1a:A555D**	4	0.11
Maharashtra										**ORF1a:N557F**	4	0.11
Maharashtra										**ORF1a:V559R**	4	0.11

Maharashtra										**ORF1a:V561P**	4	0.11
Maharashtra										**ORF1a:K625N**	4	0.11
Maharashtra										**ORF1a:V627S**	4	0.11
Maharashtra										**ORF1a:D629I**	4	0.11
Maharashtra										**ORF1a:W630G**	4	0.11
Maharashtra										**ORF1a:E632V**	4	0.11
Maharashtra										**ORF1a:E633***	4	0.11
Maharashtra										**ORF1a:K634S**	4	0.11
Maharashtra										**ORF1a:G780L**	4	0.11
Maharashtra										**ORF1a:P782G**	4	0.11
Maharashtra										**ORF1a:V783L**	4	0.11
Maharashtra										**ORF1a:T1093L**	4	0.11
Maharashtra										**ORF1a:K1185***	4	0.11
Maharashtra										**ORF1a:I1435***	4	0.11
Maharashtra										**ORF1a:G1451L**	4	0.11
Maharashtra										**ORF1a:Y1452L**	4	0.11
Maharashtra										**ORF1a:V1453Q**	4	0.11
Maharashtra										**ORF1a:T1454C**	4	0.11
Maharashtra										**ORF1a:H1580W**	4	0.11
Maharashtra										**ORF1a:D1849T**	4	0.11
Maharashtra										**ORF1a:K2771N**	4	0.11
Maharashtra										**ORF1a:F3770***	4	0.11
Maharashtra										**ORF1a:I3771Y**	4	0.11
Maharashtra										**ORF1a:L3781S**	4	0.11
Maharashtra										**ORF1a:F4196T**	4	0.11
Maharashtra										**ORF1a:P4197G**	4	0.11
Maharashtra										**ORF1a:K4198F**	4	0.11
Maharashtra										**ORF1a:S4199E**	4	0.11
Maharashtra										**ORF1a:D4200M**	4	0.11
Maharashtra										**ORF1a:P2195E**	4	0.11
Maharashtra										**ORF1a:T2196V**	4	0.11
Maharashtra										**ORF1a:T2197Q**	4	0.11
Maharashtra										**ORF1a:K2200V**	4	0.11
Maharashtra										**ORF1a:T2202F**	4	0.11
Maharashtra										**ORF1a:A2248Q**	4	0.11
Maharashtra										**ORF1a:A2249P**	4	0.11
Maharashtra										**ORF1a:G2251A**	4	0.11
Maharashtra										**ORF1a:V2252C**	4	0.11
Maharashtra										**ORF1a:M2254L**	4	0.11
Maharashtra										**ORF1a:S2255T**	4	0.11
Maharashtra										**ORF1a:N2256V**	4	0.11
Maharashtra										**ORF1a:I2278E**	4	0.11
Maharashtra										**ORF1a:A2279K**	4	0.11
Maharashtra										**ORF1a:T2280A**	4	0.11
Maharashtra										**ORF1a:Y2281I**	4	0.11
Maharashtra										**ORF1a:C2282***	4	0.11
Maharashtra										**ORF1a:S2285***	4	0.11
Maharashtra										**ORF1a:E2305***	4	0.11
Maharashtra										**ORF1a:W2327***	4	0.11
Maharashtra										**ORF1a:F2328L**	4	0.11
Maharashtra										**ORF1a:A2330L**	4	0.11
Maharashtra										**ORF1a:Y2331A**	4	0.11
Maharashtra										**ORF1a:I2332***	4	0.11
Maharashtra										**ORF1a:I2368Y**	4	0.11
Maharashtra										**ORF1a:N2370L**	4	0.11
Maharashtra										**ORF1a:R2417I**	4	0.11
Maharashtra										**ORF1a:A2418H**	4	0.11
Maharashtra										**ORF1a:T2419Q**	4	0.11
Maharashtra										**ORF1a:R2420L**	4	0.11
Maharashtra										**ORF1a:E2422***	4	0.11
Maharashtra										**ORF1a:T2424V**	4	0.11
Maharashtra										**ORF1a:I2426N**	4	0.11
Maharashtra										**ORF1a:N2428I**	4	0.11
Maharashtra										**ORF1a:G2429E**	4	0.11
Maharashtra										**ORF1a:V2430Q**	4	0.11
Maharashtra										**ORF1a:R2431Q**	4	0.11
Maharashtra										**ORF1a:R2432E**	4	0.11
Maharashtra										**ORF1a:Y2435A**	4	0.11
Maharashtra										**ORF1a:L2570R**	4	0.11
Maharashtra										**ORF1a:L2571H**	4	0.11
Maharashtra										**ORF1a:L2572***	4	0.11
Maharashtra										**ORF1a:D2573C**	4	0.11
Maharashtra										**ORF1a:Q2574L**	4	0.11

Maharashtra										**ORF1a:A2575M**	4	0.11
Maharashtra										**ORF1a:V2955L**	4	0.11
Maharashtra										**ORF1a:T3374S**	4	0.11
Maharashtra										**ORF1a:F3375I**	4	0.11
Maharashtra										**ORF1a:S3402N**	4	0.11
Maharashtra										**ORF1a:N3651D**	4	0.11
Maharashtra										**ORF1a:L2062I**	4	0.11
Maharashtra										**ORF1a:E2063L**	4	0.11
Maharashtra										**ORF1a:C2064P**	4	0.11
Maharashtra										**ORF1a:N2111V**	4	0.11
Maharashtra										**ORF1a:S2114L**	4	0.11
Maharashtra										**ORF1a:V2116N**	4	0.11
Maharashtra										**ORF1a:G2118***	4	0.11
Maharashtra										**ORF1a:A2128L**	4	0.11
Maharashtra										**ORF1a:A2129I**	4	0.11
Maharashtra										**ORF1a:V2150Q**	4	0.11
Maharashtra										**ORF1a:S2151L**	4	0.11
Maharashtra										**ORF1a:T2152L**	4	0.11
Maharashtra										**ORF1a:C2160***	4	0.11
Maharashtra										**ORF1a:N2162H**	4	0.11
Maharashtra										**ORF1a:R2163G**	4	0.11
Maharashtra										**ORF1a:C2165***	4	0.11
Maharashtra										**ORF1a:N2167L**	4	0.11
Maharashtra										**ORF1a:Y2168L**	4	0.11
Maharashtra										**ORF1a:M2169Y**	4	0.11
Maharashtra										**ORF1a:P2170C**	4	0.11
Maharashtra										**ORF1a:F2172N**	4	0.11
Maharashtra										**ORF1a:F2173C**	4	0.11
Maharashtra										**ORF1a:G4255W**	4	0.11
Maharashtra										**ORF1a:T1000I**	4	0.11
Maharashtra										**ORF1a:S2104N**	4	0.11
Maharashtra										**ORF1a:M1378I**	4	0.11
Maharashtra										**ORF1a:A656V**	4	0.11
Maharashtra										**ORF1a:A3392V**	4	0.11
Maharashtra										**ORF1a:P1640S**	4	0.11
Maharashtra										**ORF1a:F2458S**	4	0.11
Maharashtra										**ORF1a:K2478E**	4	0.11
Maharashtra										**ORF1a:A1793D**	4	0.11
Maharashtra										**ORF1a:N2065S**	4	0.11
Maharashtra										**ORF1a:V2066Y**	4	0.11
Maharashtra										**ORF1a:S2273Y**	4	0.11
Maharashtra										**ORF1a:T2274W**	4	0.11
Maharashtra										**ORF1a:N2275F**	4	0.11
Maharashtra										**ORF1a:V2276Y**	4	0.11
Maharashtra										**ORF1a:S2313F**	4	0.11
Maharashtra										**ORF1a:F2334S**	4	0.11
Maharashtra										**ORF1a:T2335C**	4	0.11
Maharashtra										**ORF1a:L2341C**	4	0.11
Maharashtra										**ORF1a:G2342N**	4	0.11
Maharashtra										**ORF1a:E2550L**	4	0.11
Maharashtra										**ORF1a:E2551L**	4	0.11
Maharashtra										**ORF1a:S2552Q**	4	0.11
Maharashtra										**ORF1a:D3903F**	4	0.11
Maharashtra										**ORF1a:A3907C**	4	0.11
Maharashtra										**ORF1a:A3955***	4	0.11
Maharashtra										**ORF1a:T4066R**	4	0.11
Maharashtra										**ORF1a:A4067L**	4	0.11
Maharashtra										**ORF1a:A4068***	4	0.11
Maharashtra										**ORF1a:K4069H**	4	0.11
Maharashtra										**ORF1a:M4071***	4	0.11
Maharashtra										**ORF1a:V4072K**	4	0.11
Maharashtra										**ORF1a:S2911H**	4	0.11
Maharashtra										**ORF1a:A2912H**	4	0.11
Maharashtra										**ORF1a:C2913Q**	4	0.11
Maharashtra										**ORF1a:V2914N**	4	0.11
Maharashtra										**ORF1a:A2916***	4	0.11
Maharashtra										**ORF1a:A2917S**	4	0.11
Maharashtra										**ORF1a:E2918T**	4	0.11
Maharashtra										**ORF1a:C2919L**	4	0.11
Maharashtra										**ORF1a:F3568E**	4	0.11
Maharashtra										**ORF1a:K3573H**	4	0.11
Maharashtra										**ORF1a:R3574T**	4	0.11
Maharashtra										**ORF1a:I3576G**	4	0.11

Maharashtra										**ORF1a:K3577C**	4	0.11
Maharashtra										**ORF1a:G3578Y**	4	0.11
Maharashtra										**ORF1a:T3579S**	4	0.11
Maharashtra										**ORF1a:H3581F**	4	0.11
Maharashtra										**ORF1a:W3582***	4	0.11
Maharashtra										**ORF1a:T3586N**	4	0.11
Maharashtra										**ORF1a:T3589C**	4	0.11
Maharashtra										**ORF1a:A3615C**	4	0.11
Maharashtra										**ORF1a:G3617S**	4	0.11
Maharashtra										**ORF1a:I3618N**	4	0.11
Maharashtra										**ORF1a:A3620S**	4	0.11
Maharashtra										**ORF1a:K3632F**	4	0.11
Maharashtra										**ORF1a:H3633S**	4	0.11
Maharashtra										**ORF1a:A3634V**	4	0.11
Maharashtra										**ORF1a:F3635C**	4	0.11
Maharashtra										**ORF1a:L3636F**	4	0.11
Maharashtra										**ORF1a:T3716L**	4	0.11
Maharashtra										**ORF1a:A3023***	4	0.11
Maharashtra										**ORF1a:V3024F**	4	0.11
Maharashtra										**ORF1a:M585L**	4	0.11
Maharashtra										**ORF1a:F586***	4	0.11
Maharashtra										**ORF1a:T587C**	4	0.11
Maharashtra										**ORF1a:D589H**	4	0.11
Maharashtra										**ORF1a:T760L**	4	0.11
Maharashtra										**ORF1a:G761V**	4	0.11
Maharashtra										**ORF1a:D762K**	4	0.11
Maharashtra										**ORF1a:Q764L**	4	0.11
Maharashtra										**ORF1a:A776H**	4	0.11
Maharashtra										**ORF1a:P777W**	4	0.11
Maharashtra										**ORF1a:G780H**	4	0.11
Maharashtra										**ORF1a:T781Q**	4	0.11
Maharashtra										**ORF1a:P782F**	4	0.11
Maharashtra										**ORF1a:Y860S**	4	0.11
Maharashtra										**ORF1a:P943M**	4	0.11
Maharashtra										**ORF1a:S944I**	4	0.11
Maharashtra										**ORF1a:D978L**	4	0.11
Maharashtra										**ORF1a:W979L**	4	0.11
Maharashtra										**ORF1a:A1508K**	4	0.11
Maharashtra										**ORF1a:G1509P**	4	0.11
Maharashtra										**ORF1a:E1526N**	4	0.11
Maharashtra										**ORF1a:P2018L**	4	0.11
Maharashtra										**ORF1a:E2020Y**	4	0.11
Maharashtra										**ORF1a:S2022***	4	0.11
Maharashtra										**ORF1a:V2066S**	4	0.11
Maharashtra										**ORF1a:K2067Y**	4	0.11
Maharashtra										**ORF1a:T2068H**	4	0.11
Maharashtra										**ORF1a:Y2171V**	4	0.11
Maharashtra										**ORF1a:F2173K**	4	0.11
Maharashtra										**ORF1a:T2174P**	4	0.11
Maharashtra										**ORF1a:L2175C**	4	0.11
Maharashtra										**ORF1a:Q2178***	4	0.11
Maharashtra										**ORF1a:C2180Y**	4	0.11
Maharashtra										**ORF1a:T2181K**	4	0.11
Maharashtra										**ORF1a:T2186***	4	0.11
Maharashtra										**ORF1a:G2258N**	4	0.11
Maharashtra										**ORF1a:P2260C**	4	0.11
Maharashtra										**ORF1a:S2261F**	4	0.11
Maharashtra										**ORF1a:Y2262R**	4	0.11
Maharashtra										**ORF1a:T2264E**	4	0.11
Maharashtra										**ORF1a:G2265L**	4	0.11
Maharashtra										**ORF1a:R2267***	4	0.11
Maharashtra										**ORF1a:E2268C**	4	0.11
Maharashtra										**ORF1a:G2269H**	4	0.11
Maharashtra										**ORF1a:L2271C**	4	0.11
Maharashtra										**ORF1a:T4204A**	4	0.11
Maharashtra										**ORF1a:G2258C**	4	0.11
Maharashtra										**ORF1a:T2016K**	4	0.11
Maharashtra										**ORF1a:M585T**	4	0.11
Maharashtra										**ORF1a:T265I**	4	0.11
Maharashtra										**ORF1a:K1132N**	4	0.11
Maharashtra										**ORF1a:K497N**	4	0.11
Maharashtra										**ORF1a:A1151V**	4	0.11
Maharashtra										**ORF1a:L3238I**	4	0.11

Maharashtra										**ORF1a:T4065I**	4	0.11
Maharashtra										**ORF1a:Y1635***	4	0.11
Maharashtra										**ORF1a:G2091I**	4	0.11
Maharashtra										**ORF1a:T2093W**	4	0.11
Maharashtra										**ORF1a:D2094L**	4	0.11
Maharashtra										**ORF1a:A2139L**	4	0.11
Maharashtra										**ORF1a:Y2141M**	4	0.11
Maharashtra										**ORF1a:A2142L**	4	0.11
Maharashtra										**ORF1a:K2143S**	4	0.11
Maharashtra										**ORF1a:L2364Y**	4	0.11
Maharashtra										**ORF1a:I2368V**	4	0.11
Maharashtra										**ORF1a:N2370W**	4	0.11
Maharashtra										**ORF1a:W2394I**	4	0.11
Maharashtra										**ORF1a:K2395H**	4	0.11
Maharashtra										**ORF1a:S2396Q**	4	0.11
Maharashtra										**ORF1a:H2399***	4	0.11
Maharashtra										**ORF1a:V2400C**	4	0.11
Maharashtra										**ORF1a:E2514T**	4	0.11
Maharashtra										**ORF1a:R2515T**	4	0.11
Maharashtra										**ORF1a:H2516***	4	0.11
Maharashtra										**ORF1a:S2517E**	4	0.11
Maharashtra										**ORF1a:V1295L**	4	0.11
Maharashtra										**ORF1a:T1309Y**	4	0.11
Maharashtra										**ORF1a:D981Y**	4	0.11
Maharashtra										**ORF1a:V1295I**	4	0.11
Maharashtra										**ORF1a:L3591I**	4	0.11
Maharashtra										**ORF1a:M2036I**	4	0.11
Maharashtra										**ORF1a:S1857L**	4	0.11
Maharashtra										**ORF1a:Y196H**	4	0.11
Maharashtra										**ORF1a:A482V**	4	0.11
Maharashtra										**ORF1a:A307V**	4	0.11
Maharashtra										**ORF1a:L3644F**	4	0.11
Maharashtra										**ORF1a:S3885F**	4	0.11
Maharashtra										**ORF1a:A385V**	4	0.11
Maharashtra										**ORF1a:T1242I**	4	0.11
Maharashtra										**ORF1a:T2069I**	4	0.11
Maharashtra										**ORF1a:S2352N**	4	0.11
Maharashtra										**ORF1a:S2114F**	4	0.11
Maharashtra										**ORF1a:K1305N**	4	0.11
Maharashtra										**ORF1a:G192D**	4	0.11
Maharashtra										**ORF1a:V1750L**	4	0.11
Maharashtra										**ORF1a:M3129T**	4	0.11
Maharashtra										**ORF1a:S3949N**	4	0.11
Maharashtra										**ORF1a:V3892A**	4	0.11
Maharashtra										**ORF1a:K3979N**	4	0.11
Maharashtra										**ORF1a:P361T**	4	0.11
Maharashtra										**ORF1a:R2189K**	4	0.11
Maharashtra										**ORF1a:T3543I**	4	0.11
Maharashtra										**ORF1a:A1670V**	4	0.11
Maharashtra										**ORF1a:T1444A**	4	0.11
Maharashtra										**ORF1a:A2355S**	4	0.11
Maharashtra										**ORF1a:S1612L**	4	0.11
Maharashtra										**ORF1a:T2283I**	4	0.11
Maharashtra										**ORF1a:K120N**	4	0.11
Maharashtra										**ORF1a:H45Y**	4	0.11
Maharashtra										**ORF1a:P1158S**	4	0.11
Maharashtra										**ORF1a:K3630C**	3	0.08
Maharashtra										**ORF1a:H3631F**	3	0.08
Maharashtra										**ORF1a:K3632C**	3	0.08
Maharashtra										**ORF1a:H3633Y**	3	0.08
Maharashtra										**ORF1a:A3634L**	3	0.08
Maharashtra										**ORF1a:C3637P**	3	0.08
Maharashtra										**ORF1a:F3639***	3	0.08
Maharashtra										**ORF1a:S3658I**	3	0.08
Maharashtra										**ORF1a:W3659L**	3	0.08
Maharashtra										**ORF1a:V3660I**	3	0.08
Maharashtra										**ORF1a:M3661W**	3	0.08
Maharashtra										**ORF1a:Y3793T**	3	0.08
Maharashtra										**ORF1a:T3904Y**	3	0.08
Maharashtra										**ORF1a:E3906M**	3	0.08
Maharashtra										**ORF1a:E3909S**	3	0.08
Maharashtra										**ORF1a:K3910***	3	0.08
Maharashtra										**ORF1a:M3911L**	3	0.08

Maharashtra										**ORF1a:V3912K**	3	0.08
Maharashtra										**ORF1a:S3913I**	3	0.08
Maharashtra										**ORF1a:A3955V**	3	0.08
Maharashtra										**ORF1a:A3956P**	3	0.08
Maharashtra										**ORF1a:A3958H**	3	0.08
Maharashtra										**ORF1a:T3959H**	3	0.08
Maharashtra										**ORF1a:A3960M**	3	0.08
Maharashtra										**ORF1a:E4097M**	3	0.08
Maharashtra										**ORF1a:D4117F**	3	0.08
Maharashtra										**ORF1a:S4119L**	3	0.08
Maharashtra										**ORF1a:P4120V**	3	0.08
Maharashtra										**ORF1a:A4123V**	3	0.08
Maharashtra										**ORF1a:P4125Q**	3	0.08
Maharashtra										**ORF1a:I4127***	3	0.08
Maharashtra										**ORF1a:V4128G**	3	0.08
Maharashtra										**ORF1a:T4129P**	3	0.08
Maharashtra										**ORF1a:A4130I**	3	0.08
Maharashtra										**ORF1a:R4132L**	3	0.08
Maharashtra										**ORF1a:A4133S**	3	0.08
Maharashtra										**ORF1a:L4149Y**	3	0.08
Maharashtra										**ORF1a:Q4151I**	3	0.08
Maharashtra										**ORF1a:C4154L**	3	0.08
Maharashtra										**ORF1a:G4177Q**	3	0.08
Maharashtra										**ORF1a:T163M**	3	0.08
Maharashtra										**ORF1a:S166N**	3	0.08
Maharashtra										**ORF1a:G168C**	3	0.08
Maharashtra										**ORF1a:E176S**	3	0.08
Maharashtra										**ORF1a:C213K**	3	0.08
Maharashtra										**ORF1a:S279V**	3	0.08
Maharashtra										**ORF1a:I280Q**	3	0.08
Maharashtra										**ORF1a:D629A**	3	0.08
Maharashtra										**ORF1a:W630***	3	0.08
Maharashtra										**ORF1a:L631R**	3	0.08
Maharashtra										**ORF1a:E633V**	3	0.08
Maharashtra										**ORF1a:K634***	3	0.08
Maharashtra										**ORF1a:F635G**	3	0.08
Maharashtra										**ORF1a:K636R**	3	0.08
Maharashtra										**ORF1a:E637C**	3	0.08
Maharashtra										**ORF1a:G638R**	3	0.08
Maharashtra										**ORF1a:E640S**	3	0.08
Maharashtra										**ORF1a:F641***	3	0.08
Maharashtra										**ORF1a:L642R**	3	0.08
Maharashtra										**ORF1a:D644L**	3	0.08
Maharashtra										**ORF1a:A656N**	3	0.08
Maharashtra										**ORF1a:E658***	3	0.08
Maharashtra										**ORF1a:E743R**	3	0.08
Maharashtra										**ORF1a:D795Y**	3	0.08
Maharashtra										**ORF1a:C800V**	3	0.08
Maharashtra										**ORF1a:S838D**	3	0.08
Maharashtra										**ORF1a:V839***	3	0.08
Maharashtra										**ORF1a:I841S**	3	0.08
Maharashtra										**ORF1a:G865***	3	0.08
Maharashtra										**ORF1a:T866***	3	0.08
Maharashtra										**ORF1a:D909V**	3	0.08
Maharashtra										**ORF1a:S911Y**	3	0.08
Maharashtra										**ORF1a:K915F**	3	0.08
Maharashtra										**ORF1a:A917P**	3	0.08
Maharashtra										**ORF1a:F3080V**	3	0.08
Maharashtra										**ORF1a:L3083V**	3	0.08
Maharashtra										**ORF1a:L3084M**	3	0.08
Maharashtra										**ORF1a:F3085***	3	0.08
Maharashtra										**ORF1a:S3088Q**	3	0.08
Maharashtra										**ORF1a:Y3109H**	3	0.08
Maharashtra										**ORF1a:L3110S**	3	0.08
Maharashtra										**ORF1a:I3146***	3	0.08
Maharashtra										**ORF1a:C3147Y**	3	0.08
Maharashtra										**ORF1a:M3627V**	3	0.08
Maharashtra										**ORF1a:L3754G**	3	0.08
Maharashtra										**ORF1a:A3755Q**	3	0.08
Maharashtra										**ORF1a:K4176N**	3	0.08
Maharashtra										**ORF1a:A903H**	3	0.08
Maharashtra										**ORF1a:G950***	3	0.08
Maharashtra										**ORF1a:G993N**	3	0.08

Maharashtra										**ORF1a:S994Y**	3	0.08
Maharashtra										**ORF1a:E995Y**	3	0.08
Maharashtra										**ORF1a:D996S**	3	0.08
Maharashtra										**ORF1a:Q998G**	3	0.08
Maharashtra										**ORF1a:T999S**	3	0.08
Maharashtra										**ORF1a:Q1021R**	3	0.08
Maharashtra										**ORF1a:T1022D**	3	0.08
Maharashtra										**ORF1a:I1023G**	3	0.08
Maharashtra										**ORF1a:E1024T**	3	0.08
Maharashtra										**ORF1a:V1025Y**	3	0.08
Maharashtra										**ORF1a:N1026T**	3	0.08
Maharashtra										**ORF1a:F1028C**	3	0.08
Maharashtra										**ORF1a:G1030D**	3	0.08
Maharashtra										**ORF1a:K1033F**	3	0.08
Maharashtra										**ORF1a:L1034K**	3	0.08
Maharashtra										**ORF1a:D1036Y**	3	0.08
Maharashtra										**ORF1a:N1037***	3	0.08
Maharashtra										**ORF1a:V1038Q**	3	0.08
Maharashtra										**ORF1a:Y1039C**	3	0.08
Maharashtra										**ORF1a:G1069Y**	3	0.08
Maharashtra										**ORF1a:G1070***	3	0.08
Maharashtra										**ORF1a:V1071Q**	3	0.08
Maharashtra										**ORF1a:A1072C**	3	0.08
Maharashtra										**ORF1a:G1073H**	3	0.08
Maharashtra										**ORF1a:V1099R**	3	0.08
Maharashtra										**ORF1a:G1100T**	3	0.08
Maharashtra										**ORF1a:G1101Q**	3	0.08
Maharashtra										**ORF1a:Y1183S**	3	0.08
Maharashtra										**ORF1a:S1188L**	3	0.08
Maharashtra										**ORF1a:E1192***	3	0.08
Maharashtra										**ORF1a:K1197***	3	0.08
Maharashtra										**ORF1a:V1199T**	3	0.08
Maharashtra										**ORF1a:E1200C**	3	0.08
Maharashtra										**ORF1a:Q1201F**	3	0.08
Maharashtra										**ORF1a:D1228Y**	3	0.08
Maharashtra										**ORF1a:D1229N**	3	0.08
Maharashtra										**ORF1a:K1230***	3	0.08
Maharashtra										**ORF1a:I1232***	3	0.08
Maharashtra										**ORF1a:K1233T**	3	0.08
Maharashtra										**ORF1a:A1234F**	3	0.08
Maharashtra										**ORF1a:C1235S**	3	0.08
Maharashtra										**ORF1a:E1237T**	3	0.08
Maharashtra										**ORF1a:V1239K**	3	0.08
Maharashtra										**ORF1a:T1241R**	3	0.08
Maharashtra										**ORF1a:T1242***	3	0.08
Maharashtra										**ORF1a:L1243***	3	0.08
Maharashtra										**ORF1a:E1245H**	3	0.08
Maharashtra										**ORF1a:F1248L**	3	0.08
Maharashtra										**ORF1a:L1317N**	3	0.08
Maharashtra										**ORF1a:T1327Q**	3	0.08
Maharashtra										**ORF1a:T1328L**	3	0.08
Maharashtra										**ORF1a:N1436L**	3	0.08
Maharashtra										**ORF1a:T1437L**	3	0.08
Maharashtra										**ORF1a:N1439H**	3	0.08
Maharashtra										**ORF1a:L1441***	3	0.08
Maharashtra										**ORF1a:T1444C**	3	0.08
Maharashtra										**ORF1a:L1445S**	3	0.08
Maharashtra										**ORF1a:L1627***	3	0.08
Maharashtra										**ORF1a:A1707C**	3	0.08
Maharashtra										**ORF1a:A1708S**	3	0.08
Maharashtra										**ORF1a:N1709T**	3	0.08
Maharashtra										**ORF1a:F1710R**	3	0.08
Maharashtra										**ORF1a:A1716***	3	0.08
Maharashtra										**ORF1a:Y1717***	3	0.08
Maharashtra										**ORF1a:C1718D**	3	0.08
Maharashtra										**ORF1a:N1719S**	3	0.08
Maharashtra										**ORF1a:K1720R**	3	0.08
Maharashtra										**ORF1a:T1721***	3	0.08
Maharashtra										**ORF1a:P1786S**	3	0.08
Maharashtra										**ORF1a:L1853R**	3	0.08
Maharashtra										**ORF1a:K1855Q**	3	0.08
Maharashtra										**ORF1a:S1856R**	3	0.08
Maharashtra										**ORF1a:E1858Y**	3	0.08

Maharashtra										**ORF1a:K1883Q**	3	0.08
Maharashtra										**ORF1a:D1885V**	3	0.08
Maharashtra										**ORF1a:M2374L**	3	0.08
Maharashtra										**ORF1a:P2376C**	3	0.08
Maharashtra										**ORF1a:E2940H**	3	0.08
Maharashtra										**ORF1a:G2941T**	3	0.08
Maharashtra										**ORF1a:V2943C**	3	0.08
Maharashtra										**ORF1a:T3133D**	3	0.08
Maharashtra										**ORF1a:P3134G**	3	0.08
Maharashtra										**ORF1a:L3135Y**	3	0.08
Maharashtra										**ORF1a:P3137H**	3	0.08
Maharashtra										**ORF1a:F3138T**	3	0.08
Maharashtra										**ORF1a:W3139F**	3	0.08
Maharashtra										**ORF1a:I3140S**	3	0.08
Maharashtra										**ORF1a:I3142F**	3	0.08
Maharashtra										**ORF1a:Y3144D**	3	0.08
Maharashtra										**ORF1a:I3146N**	3	0.08
Maharashtra										**ORF1a:S3149Y**	3	0.08
Maharashtra										**ORF1a:T3150H**	3	0.08
Maharashtra										**ORF1a:K3151L**	3	0.08
Maharashtra										**ORF1a:H3152Y**	3	0.08
Maharashtra										**ORF1a:Y3154H**	3	0.08
Maharashtra										**ORF1a:W3155K**	3	0.08
Maharashtra										**ORF1a:F3156A**	3	0.08
Maharashtra										**ORF1a:D3196K**	3	0.08
Maharashtra										**ORF1a:L3198S**	3	0.08
Maharashtra										**ORF1a:L3201***	3	0.08
Maharashtra										**ORF1a:T3202V**	3	0.08
Maharashtra										**ORF1a:Y3204V**	3	0.08
Maharashtra										**ORF1a:N3205F**	3	0.08
Maharashtra										**ORF1a:R3206***	3	0.08
Maharashtra										**ORF1a:Y3207W**	3	0.08
Maharashtra										**ORF1a:L3208S**	3	0.08
Maharashtra										**ORF1a:A3209N**	3	0.08
Maharashtra										**ORF1a:L3210G**	3	0.08
Maharashtra										**ORF1a:V3411***	3	0.08
Maharashtra										**ORF1a:V186I**	3	0.08
Maharashtra										**ORF1a:A2249V**	3	0.08
Maharashtra										**ORF1a:A2345V**	3	0.08
Maharashtra										**ORF1a:S40K**	3	0.08
Maharashtra										**ORF1a:E1293R**	3	0.08
Maharashtra										**ORF1a:V274L**	3	0.08
Maharashtra										**ORF1a:T3710I**	3	0.08
Maharashtra										**ORF1a:Y799N**	3	0.08
Maharashtra										**ORF1a:A2554V**	3	0.08
Maharashtra										**ORF1a:T3361A**	3	0.08
Maharashtra										**ORF1a:S2015I**	3	0.08
Maharashtra										**ORF1a:K4232R**	3	0.08
Maharashtra										**ORF1a:Q998H**	3	0.08
Maharashtra										**ORF1a:H3233Y**	3	0.08
Maharashtra										**ORF1a:Y4013G**	3	0.08
Maharashtra										**ORF1a:D4043R**	3	0.08
Maharashtra										**ORF1a:A4044W**	3	0.08
Maharashtra										**ORF1a:E55A**	3	0.08
Maharashtra										**ORF1a:V56***	3	0.08
Maharashtra										**ORF1a:R77V**	3	0.08
Maharashtra										**ORF1a:A79M**	3	0.08
Maharashtra										**ORF1a:L107V**	3	0.08
Maharashtra										**ORF1a:V108R**	3	0.08
Maharashtra										**ORF1a:A117F**	3	0.08
Maharashtra										**ORF1a:G130T**	3	0.08
Maharashtra										**ORF1a:G132P**	3	0.08
Maharashtra										**ORF1a:G133I**	3	0.08
Maharashtra										**ORF1a:H134***	3	0.08
Maharashtra										**ORF1a:Y136H**	3	0.08
Maharashtra										**ORF1a:G137L**	3	0.08
Maharashtra										**ORF1a:D139***	3	0.08
Maharashtra										**ORF1a:L140A**	3	0.08
Maharashtra										**ORF1a:K141T**	3	0.08
Maharashtra										**ORF1a:A206L**	3	0.08
Maharashtra										**ORF1a:R207S**	3	0.08
Maharashtra										**ORF1a:S279Q**	3	0.08
Maharashtra										**ORF1a:I280G**	3	0.08

Maharashtra										**ORF1a:I281L**	3	0.08
Maharashtra										**ORF1a:K367V**	3	0.08
Maharashtra										**ORF1a:I368T**	3	0.08
Maharashtra										**ORF1a:C370I**	3	0.08
Maharashtra										**ORF1a:P371Q**	3	0.08
Maharashtra										**ORF1a:V488F**	3	0.08
Maharashtra										**ORF1a:P959L**	3	0.08
Maharashtra										**ORF1a:A1072M**	3	0.08
Maharashtra										**ORF1a:G1073F**	3	0.08
Maharashtra										**ORF1a:A1485F**	3	0.08
Maharashtra										**ORF1a:Q1582C**	3	0.08
Maharashtra										**ORF1a:V1583Q**	3	0.08
Maharashtra										**ORF1a:V1584***	3	0.08
Maharashtra										**ORF1a:D1585H**	3	0.08
Maharashtra										**ORF1a:F1804S**	3	0.08
Maharashtra										**ORF1a:N1898L**	3	0.08
Maharashtra										**ORF1a:Y1900H**	3	0.08
Maharashtra										**ORF1a:T2106I**	3	0.08
Maharashtra										**ORF1a:T2335I**	3	0.08
Maharashtra										**ORF1a:R2336Y**	3	0.08
Maharashtra										**ORF1a:F2337S**	3	0.08
Maharashtra										**ORF1a:Y2339H**	3	0.08
Maharashtra										**ORF1a:V2340***	3	0.08
Maharashtra										**ORF1a:L2341V**	3	0.08
Maharashtra										**ORF1a:G2342F**	3	0.08
Maharashtra										**ORF1a:S2547I**	3	0.08
Maharashtra										**ORF1a:K2548I**	3	0.08
Maharashtra										**ORF1a:E2550K**	3	0.08
Maharashtra										**ORF1a:E2551I**	3	0.08
Maharashtra										**ORF1a:S2553V**	3	0.08
Maharashtra										**ORF1a:A2554C**	3	0.08
Maharashtra										**ORF1a:K2555L**	3	0.08
Maharashtra										**ORF1a:A2557Q**	3	0.08
Maharashtra										**ORF1a:V2559A**	3	0.08
Maharashtra										**ORF1a:S2731***	3	0.08
Maharashtra										**ORF1a:P2739T**	3	0.08
Maharashtra										**ORF1a:V2750***	3	0.08
Maharashtra										**ORF1a:L2770***	3	0.08
Maharashtra										**ORF1a:Q2772C**	3	0.08
Maharashtra										**ORF1a:L2773***	3	0.08
Maharashtra										**ORF1a:K2775L**	3	0.08
Maharashtra										**ORF1a:T2777E**	3	0.08
Maharashtra										**ORF1a:L2778A**	3	0.08
Maharashtra										**ORF1a:I3369V**	3	0.08
Maharashtra										**ORF1a:N1578K**	3	0.08
Maharashtra										**ORF1a:S1856F**	3	0.08
Maharashtra										**ORF1a:G2284V**	3	0.08
Maharashtra										**ORF1a:C2459T**	3	0.08
Maharashtra										**ORF1a:A2460I**	3	0.08
Maharashtra										**ORF1a:S2462I**	3	0.08
Maharashtra										**ORF1a:T2463V**	3	0.08
Maharashtra										**ORF1a:F2464L**	3	0.08
Maharashtra										**ORF1a:S2658L**	3	0.08
Maharashtra										**ORF1a:H2659A**	3	0.08
Maharashtra										**ORF1a:Q2660I**	3	0.08
Maharashtra										**ORF1a:S2661V**	3	0.08
Maharashtra										**ORF1a:D2662V**	3	0.08
Maharashtra										**ORF1a:V3130M**	3	0.08
Maharashtra										**ORF1a:P3504W**	3	0.08
Maharashtra										**ORF1a:M3652A**	3	0.08
Maharashtra										**ORF1a:V3653Y**	3	0.08
Maharashtra										**ORF1a:K798N**	3	0.08
Maharashtra										**ORF1a:E995D**	3	0.08
Maharashtra										**ORF1a:S2661F**	3	0.08
Maharashtra										**ORF1a:T1344R**	3	0.08
Maharashtra										**ORF1a:C316Q**	3	0.08
Maharashtra										**ORF1a:S383N**	3	0.08
Maharashtra										**ORF1a:G407***	3	0.08
Maharashtra										**ORF1a:E673L**	3	0.08
Maharashtra										**ORF1a:D1167F**	3	0.08
Maharashtra										**ORF1a:T1168L**	3	0.08
Maharashtra										**ORF1a:R1170L**	3	0.08
Maharashtra										**ORF1a:N1172L**	3	0.08

Maharashtra										**ORF1a:V1173Y**	3	0.08
Maharashtra										**ORF1a:Y1174I**	3	0.08
Maharashtra										**ORF1a:A1176***	3	0.08
Maharashtra										**ORF1a:V1177E**	3	0.08
Maharashtra										**ORF1a:D1179V**	3	0.08
Maharashtra										**ORF1a:K1180***	3	0.08
Maharashtra										**ORF1a:N1181I**	3	0.08
Maharashtra										**ORF1a:Q1227L**	3	0.08
Maharashtra										**ORF1a:D1228V**	3	0.08
Maharashtra										**ORF1a:D1229L**	3	0.08
Maharashtra										**ORF1a:V4250N**	3	0.08
Maharashtra										**ORF1a:C1755S**	3	0.08
Maharashtra										**ORF1a:G1756R**	3	0.08
Maharashtra										**ORF1a:Q1758P**	3	0.08
Maharashtra										**ORF1a:Q1759L**	3	0.08
Maharashtra										**ORF1a:T1760R**	3	0.08
Maharashtra										**ORF1a:T1761V**	3	0.08
Maharashtra										**ORF1a:L1762***	3	0.08
Maharashtra										**ORF1a:D3992G**	3	0.08
Maharashtra										**ORF1a:R3993K**	3	0.08
Maharashtra										**ORF1a:I4061K**	3	0.08
Maharashtra										**ORF1a:I4062R**	3	0.08
Maharashtra										**ORF1a:P4063W**	3	0.08
Maharashtra										**ORF1a:D1273H**	3	0.08
Maharashtra										**ORF1a:I1276W**	3	0.08
Maharashtra										**ORF1a:T1277V**	3	0.08
Maharashtra										**ORF1a:K1280F**	3	0.08
Maharashtra										**ORF1a:G1294R**	3	0.08
Maharashtra										**ORF1a:N1611W**	3	0.08
Maharashtra										**ORF1a:N2623F**	3	0.08
Maharashtra										**ORF1a:V2624Q**	3	0.08
Maharashtra										**ORF1a:S2625Q**	3	0.08
Maharashtra										**ORF1a:D2627G**	3	0.08
Maharashtra										**ORF1a:N2628K**	3	0.08
Maharashtra										**ORF1a:V2629G**	3	0.08
Maharashtra										**ORF1a:Y2677L**	3	0.08
Maharashtra										**ORF1a:N2678V**	3	0.08
Maharashtra										**ORF1a:K2679L**	3	0.08
Maharashtra										**ORF1a:A2704L**	3	0.08
Maharashtra										**ORF1a:R2730I**	3	0.08
Maharashtra										**ORF1a:A2733H**	3	0.08
Maharashtra										**ORF1a:K2734C**	3	0.08
Maharashtra										**ORF1a:K2735L**	3	0.08
Maharashtra										**ORF1a:P2739D**	3	0.08
Maharashtra										**ORF1a:F2740K**	3	0.08
Maharashtra										**ORF1a:K2741L**	3	0.08
Maharashtra										**ORF1a:T2743M**	3	0.08
Maharashtra										**ORF1a:C2744L**	3	0.08
Maharashtra										**ORF1a:A2745***	3	0.08
Maharashtra										**ORF1a:T2746Q**	3	0.08
Maharashtra										**ORF1a:T2747Q**	3	0.08
Maharashtra										**ORF1a:H2883V**	3	0.08
Maharashtra										**ORF1a:F2884Q**	3	0.08
Maharashtra										**ORF1a:F2889T**	3	0.08
Maharashtra										**ORF1a:P2931M**	3	0.08
Maharashtra										**ORF1a:Y2932I**	3	0.08
Maharashtra										**ORF1a:C2933P**	3	0.08
Maharashtra										**ORF1a:Y2934M**	3	0.08
Maharashtra										**ORF1a:D2935Y**	3	0.08
Maharashtra										**ORF1a:T2936***	3	0.08
Maharashtra										**ORF1a:N2937K**	3	0.08
Maharashtra										**ORF1a:Y3300T**	3	0.08
Maharashtra										**ORF1a:D3319L**	3	0.08
Maharashtra										**ORF1a:S3517***	3	0.08
Maharashtra										**ORF1a:A3518H**	3	0.08
Maharashtra										**ORF1a:Q3519K**	3	0.08
Maharashtra										**ORF1a:T3555V**	3	0.08
Maharashtra										**ORF1a:F3557Y**	3	0.08
Maharashtra										**ORF1a:D3558W**	3	0.08
Maharashtra										**ORF1a:R3561V**	3	0.08
Maharashtra										**ORF1a:Q3562L**	3	0.08
Maharashtra										**ORF1a:C3563L**	3	0.08
Maharashtra										**ORF1a:G3565K**	3	0.08

Maharashtra										**ORF1a:T3567K**	3	0.08
Maharashtra										**ORF1a:P3613F**	3	0.08
Maharashtra										**ORF1a:C2997M**	3	0.08
Maharashtra										**ORF1a:V2998I**	3	0.08
Maharashtra										**ORF1a:S2999I**	3	0.08
Maharashtra										**ORF1a:A3054***	3	0.08
Maharashtra										**ORF1a:T3058E**	3	0.08
Maharashtra										**ORF1a:C3059L**	3	0.08
Maharashtra										**ORF1a:M789T**	3	0.08
Maharashtra										**ORF1a:L790G**	3	0.08
Maharashtra										**ORF1a:E792T**	3	0.08
Maharashtra										**ORF1a:I793Q**	3	0.08
Maharashtra										**ORF1a:D795S**	3	0.08
Maharashtra										**ORF1a:T861S**	3	0.08
Maharashtra										**ORF1a:V862P**	3	0.08
Maharashtra										**ORF1a:E863V**	3	0.08
Maharashtra										**ORF1a:T1552***	3	0.08
Maharashtra										**ORF1a:F1553M**	3	0.08
Maharashtra										**ORF1a:D1554V**	3	0.08
Maharashtra										**ORF1a:N1555K**	3	0.08
Maharashtra										**ORF1a:K1557S**	3	0.08
Maharashtra										**ORF1a:T1558P**	3	0.08
Maharashtra										**ORF1a:L1560T**	3	0.08
Maharashtra										**ORF1a:S1561I**	3	0.08
Maharashtra										**ORF1a:V1584N**	3	0.08
Maharashtra										**ORF1a:S2900L**	3	0.08
Maharashtra										**ORF1a:T1840I**	3	0.08
Maharashtra										**ORF1a:G3578S**	3	0.08
Maharashtra										**ORF1a:Q4140H**	3	0.08
Maharashtra										**ORF1a:N1898Y**	3	0.08
Maharashtra										**ORF1a:L2364F**	3	0.08
Maharashtra										**ORF1a:P4211S**	3	0.08
Maharashtra										**ORF1a:W1669R**	3	0.08
Maharashtra										**ORF1a:C3301S**	3	0.08
Maharashtra										**ORF1a:S318L**	3	0.08
Maharashtra										**ORF1a:K3353N**	3	0.08
Maharashtra										**ORF1a:T2936I**	3	0.08
Maharashtra										**ORF1a:V2061F**	3	0.08
Maharashtra										**ORF1a:E102A**	3	0.08
Maharashtra										**ORF1a:Q3777H**	3	0.08
Maharashtra										**ORF1a:P2079L**	3	0.08
Maharashtra										**ORF1a:S142L**	3	0.08
Maharashtra										**ORF1a:D75N**	3	0.08
Maharashtra										**ORF1a:T1538I**	3	0.08
Maharashtra										**ORF1a:T1401I**	3	0.08
Maharashtra										**ORF1a:L1556F**	3	0.08
Maharashtra										**ORF1a:S838I**	3	0.08
Maharashtra										**ORF1a:A4016V**	3	0.08
Maharashtra										**ORF1a:E913D**	3	0.08
Maharashtra										**ORF1a:A2355V**	3	0.08
Maharashtra										**ORF1a:H2357N**	3	0.08
Maharashtra										**ORF1a:F2358N**	3	0.08
Maharashtra										**ORF1a:I2359***	3	0.08
Maharashtra										**ORF1a:Y2384E**	3	0.08
Maharashtra										**ORF1a:I2385K**	3	0.08
Maharashtra										**ORF1a:F2386L**	3	0.08
Maharashtra										**ORF1a:F2387C**	3	0.08
Maharashtra										**ORF1a:M2410C**	3	0.08
Maharashtra										**ORF1a:M2411R**	3	0.08
Maharashtra										**ORF1a:C2412R**	3	0.08
Maharashtra										**ORF1a:Y2413L**	3	0.08
Maharashtra										**ORF1a:K2414***	3	0.08
Maharashtra										**ORF1a:R2415F**	3	0.08
Maharashtra										**ORF1a:N2416I**	3	0.08
Maharashtra										**ORF1a:R2417N**	3	0.08
Maharashtra										**ORF1a:A2418L**	3	0.08
Maharashtra										**ORF1a:T2419Y**	3	0.08
Maharashtra										**ORF1a:R2420D**	3	0.08
Maharashtra										**ORF1a:N2454L**	3	0.08
Maharashtra										**ORF1a:C2455L**	3	0.08
Maharashtra										**ORF1a:D2456Q**	3	0.08
Maharashtra										**ORF1a:G2927T**	3	0.08
Maharashtra										**ORF1a:K2928I**	3	0.08

Maharashtra										ORF1a:P2931*	3	0.08
Maharashtra										ORF1a:M2957L	3	0.08
Maharashtra										ORF1a:D2958Y	3	0.08
Maharashtra										ORF1a:G2959Y	3	0.08
Maharashtra										ORF1a:I2962*	3	0.08
Maharashtra										ORF1a:Q2963R	3	0.08
Maharashtra										ORF1a:S3273I	3	0.08
Maharashtra										ORF1a:G3274W	3	0.08
Maharashtra										ORF1a:K3275*	3	0.08
Maharashtra										ORF1a:V3276S	3	0.08
Maharashtra										ORF1a:E3277*	3	0.08
Maharashtra										ORF1a:L3295C	3	0.08
Maharashtra										ORF1a:E3318L	3	0.08
Maharashtra										ORF1a:S2500F	3	0.08
Maharashtra										ORF1a:V756F	3	0.08
Maharashtra										ORF1a:N1181C	3	0.08
Maharashtra										ORF1a:Y1183L	3	0.08
Maharashtra										ORF1a:D1184S	3	0.08
Maharashtra										ORF1a:K1185C	3	0.08
Maharashtra										ORF1a:V1187*	3	0.08
Maharashtra										ORF1a:S1188*	3	0.08
Maharashtra										ORF1a:S1189K	3	0.08
Maharashtra										ORF1a:F1190S	3	0.08
Maharashtra										ORF1a:E1205A	3	0.08
Maharashtra										ORF1a:P1207Y	3	0.08
Maharashtra										ORF1a:K1208N	3	0.08
Maharashtra										ORF1a:E1209*	3	0.08
Maharashtra										ORF1a:E1210K	3	0.08
Maharashtra										ORF1a:V1211*	3	0.08
Maharashtra										ORF1a:K1212T	3	0.08
Maharashtra										ORF1a:P1213F	3	0.08
Maharashtra										ORF1a:F1214S	3	0.08
Maharashtra										ORF1a:I1215*	3	0.08
Maharashtra										ORF1a:A1234T	3	0.08
Maharashtra										ORF1a:C1235R	3	0.08
Maharashtra										ORF1a:E1237*	3	0.08
Maharashtra										ORF1a:T1240P	3	0.08
Maharashtra										ORF1a:T1241H	3	0.08
Maharashtra										ORF1a:T1242R	3	0.08
Maharashtra										ORF1a:L1243K	3	0.08
Maharashtra										ORF1a:E1244L	3	0.08
Maharashtra										ORF1a:E1245V	3	0.08
Maharashtra										ORF1a:K1281E	3	0.08
Maharashtra										ORF1a:D1282R	3	0.08
Maharashtra										ORF1a:A1283C	3	0.08
Maharashtra										ORF1a:P1284S	3	0.08
Maharashtra										ORF1a:Y1285I	3	0.08
Maharashtra										ORF1a:I1286Y	3	0.08
Maharashtra										ORF1a:V1287S	3	0.08
Maharashtra										ORF1a:M1312K	3	0.08
Maharashtra										ORF1a:L1313S	3	0.08
Maharashtra										ORF1a:P1330N	3	0.08
Maharashtra										ORF1a:G1331R	3	0.08
Maharashtra										ORF1a:G1333L	3	0.08
Maharashtra										ORF1a:L1334Y	3	0.08
Maharashtra										ORF1a:V1431*	3	0.08
Maharashtra										ORF1a:A1432R	3	0.08
Maharashtra										ORF1a:L1434K	3	0.08
Maharashtra										ORF1a:L1469C	3	0.08
Maharashtra										ORF1a:K1470S	3	0.08
Maharashtra										ORF1a:L1546*	3	0.08
Maharashtra										ORF1a:D1547*	3	0.08
Maharashtra										ORF1a:G1548S	3	0.08
Maharashtra										ORF1a:E1549Y	3	0.08
Maharashtra										ORF1a:V1550H	3	0.08
Maharashtra										ORF1a:T1552S	3	0.08
Maharashtra										ORF1a:T1572*	3	0.08
Maharashtra										ORF1a:T3488S	3	0.08
Maharashtra										ORF1a:A3548M	3	0.08
Maharashtra										ORF1a:S1510F	3	0.08
Maharashtra										ORF1a:T1240I	3	0.08
Maharashtra										ORF1a:H1818Y	3	0.08
Maharashtra										ORF1a:S2389L	3	0.08

Maharashtra										**ORF1a:T2087I**	3	0.08
Maharashtra										**ORF1a:P2079S**	3	0.08
Maharashtra										**ORF1a:Q1140H**	3	0.08
Maharashtra										**ORF1a:L2367***	3	0.08
Maharashtra										**ORF1a:A2891V**	3	0.08
Maharashtra										**ORF1a:S3099L**	3	0.08
Maharashtra										**ORF1a:E444D**	3	0.08
Maharashtra										**ORF1a:R3066K**	3	0.08
Maharashtra										**ORF1a:S2972F**	3	0.08
Maharashtra										**ORF1a:T3202A**	3	0.08
Maharashtra										**ORF1a:P943S**	3	0.08
Maharashtra										**ORF1a:P2685T**	3	0.08
Maharashtra										**ORF1a:A1111P**	3	0.08
Maharashtra										**ORF1a:D1127C**	3	0.08
Maharashtra										**ORF1a:I1128***	3	0.08
Maharashtra										**ORF1a:D1481S**	3	0.08
Maharashtra										**ORF1a:G1488T**	3	0.08
Maharashtra										**ORF1a:Y1489***	3	0.08
Maharashtra										**ORF1a:L1490R**	3	0.08
Maharashtra										**ORF1a:A3454V**	3	0.08
Maharashtra										**ORF1a:A4357V**	3	0.08
Maharashtra										**ORF1a:R3267K**	3	0.08
Maharashtra										**ORF1a:T2183I**	3	0.08
Maharashtra										**ORF1a:H325Q**	3	0.08
Maharashtra										**ORF1a:P2079T**	3	0.08
Maharashtra										**ORF1a:T4249I**	3	0.08
Maharashtra										**ORF1a:T882N**	3	0.08
Maharashtra										**ORF1a:H2125Y**	3	0.08
Maharashtra										**ORF1a:V627F**	3	0.08
Maharashtra										**ORF1a:M1312I**	3	0.08
Maharashtra										**ORF1a:E797V**	3	0.08
Maharashtra										**ORF1a:L1896W**	3	0.08
Maharashtra										**ORF1a:D203Y**	3	0.08
Maharashtra										**ORF1a:L3352F**	3	0.08
Maharashtra										**ORF1a:Q607H**	3	0.08
Maharashtra										**ORF1a:P892L**	3	0.08
Maharashtra										**ORF1a:A3571V**	3	0.08
Maharashtra										**ORF1a:T2615I**	3	0.08
Maharashtra										**ORF1a:S1534I**	3	0.08
Maharashtra										**ORF1a:F3785L**	3	0.08
Maharashtra										**ORF1a:P3134S**	3	0.08
Maharashtra										**ORF1a:V3048I**	3	0.08
Maharashtra										**ORF1a:G1155C**	3	0.08
Maharashtra										**ORF1a:T1543I**	3	0.08
Maharashtra										**ORF1a:S2103F**	3	0.08
Maharashtra										**ORF1a:A540V**	3	0.08
Maharashtra										**ORF1a:P927L**	3	0.08
Maharashtra										**ORF1a:S2822F**	3	0.08
Maharashtra										**ORF1a:M1448K**	3	0.08
Maharashtra										**ORF1a:T403I**	3	0.08
Maharashtra										**ORF1a:Q2702H**	3	0.08
Maharashtra										**ORF1a:L92F**	3	0.08
Maharashtra										**ORF1a:S1221P**	3	0.08
Maharashtra										**ORF1a:P2568S**	3	0.08
Maharashtra										**ORF1a:S1027N**	2	0.06
Maharashtra										**ORF1a:A3730S**	2	0.06
Maharashtra										**ORF1a:H2502K**	2	0.06
Maharashtra										**ORF1a:Q2510V**	2	0.06
Maharashtra										**ORF1a:T2512R**	2	0.06
Maharashtra										**ORF1a:Y2513L**	2	0.06
Maharashtra										**ORF1a:E2514M**	2	0.06
Maharashtra										**ORF1a:R2515K**	2	0.06
Maharashtra										**ORF1a:H2516D**	2	0.06
Maharashtra										**ORF1a:T3772L**	2	0.06
Maharashtra										**ORF1a:G3773S**	2	0.06
Maharashtra										**ORF1a:N3774I**	2	0.06
Maharashtra										**ORF1a:T3775A**	2	0.06
Maharashtra										**ORF1a:M3862***	2	0.06
Maharashtra										**ORF1a:E3882Y**	2	0.06
Maharashtra										**ORF1a:M4032R**	2	0.06
Maharashtra										**ORF1a:L4033G**	2	0.06
Maharashtra										**ORF1a:F4034Q**	2	0.06
Maharashtra										**ORF1a:T4035K**	2	0.06

Maharashtra										**ORF1a:M4036L**	2	0.06
Maharashtra										**ORF1a:R4038T**	2	0.06
Maharashtra										**ORF1a:K4039T**	2	0.06
Maharashtra										**ORF1a:N4060Q**	2	0.06
Maharashtra										**ORF1a:I4061Q**	2	0.06
Maharashtra										**ORF1a:I4062Q**	2	0.06
Maharashtra										**ORF1a:A4094H**	2	0.06
Maharashtra										**ORF1a:I4098H**	2	0.06
Maharashtra										**ORF1a:A4155L**	2	0.06
Maharashtra										**ORF1a:G4157L**	2	0.06
Maharashtra										**ORF1a:T4158M**	2	0.06
Maharashtra										**ORF1a:Q4160M**	2	0.06
Maharashtra										**ORF1a:T4161R**	2	0.06
Maharashtra										**ORF1a:A4162***	2	0.06
Maharashtra										**ORF1a:C4163L**	2	0.06
Maharashtra										**ORF1a:K4176Q**	2	0.06
Maharashtra										**ORF1a:G4178R**	2	0.06
Maharashtra										**ORF1a:R4179E**	2	0.06
Maharashtra										**ORF1a:F4180V**	2	0.06
Maharashtra										**ORF1a:V4181G**	2	0.06
Maharashtra										**ORF1a:A4183Y**	2	0.06
Maharashtra										**ORF1a:S4186D**	2	0.06
Maharashtra										**ORF1a:D4187S**	2	0.06
Maharashtra										**ORF1a:I4205R**	2	0.06
Maharashtra										**ORF1a:Y4206V**	2	0.06
Maharashtra										**ORF1a:T4207M**	2	0.06
Maharashtra										**ORF1a:I4231L**	2	0.06
Maharashtra										**ORF1a:G4233V**	2	0.06
Maharashtra										**ORF1a:N4235W**	2	0.06
Maharashtra										**ORF1a:N4236Y**	2	0.06
Maharashtra										**ORF1a:T4249V**	2	0.06
Maharashtra										**ORF1a:R4251***	2	0.06
Maharashtra										**ORF1a:Q3966R**	2	0.06
Maharashtra										**ORF1a:S142A**	2	0.06
Maharashtra										**ORF1a:Q663N**	2	0.06
Maharashtra										**ORF1a:I664***	2	0.06
Maharashtra										**ORF1a:V665G**	2	0.06
Maharashtra										**ORF1a:T666E**	2	0.06
Maharashtra										**ORF1a:F685S**	2	0.06
Maharashtra										**ORF1a:L686D**	2	0.06
Maharashtra										**ORF1a:A687I**	2	0.06
Maharashtra										**ORF1a:C689***	2	0.06
Maharashtra										**ORF1a:D691C**	2	0.06
Maharashtra										**ORF1a:S692K**	2	0.06
Maharashtra										**ORF1a:I693***	2	0.06
Maharashtra										**ORF1a:I695F**	2	0.06
Maharashtra										**ORF1a:G697***	2	0.06
Maharashtra										**ORF1a:A698N**	2	0.06
Maharashtra										**ORF1a:K699I**	2	0.06
Maharashtra										**ORF1a:L700C**	2	0.06
Maharashtra										**ORF1a:K701H**	2	0.06
Maharashtra										**ORF1a:L716I**	2	0.06
Maharashtra										**ORF1a:Y717V**	2	0.06
Maharashtra										**ORF1a:R718Q**	2	0.06
Maharashtra										**ORF1a:C720T**	2	0.06
Maharashtra										**ORF1a:V721H**	2	0.06
Maharashtra										**ORF1a:K722A**	2	0.06
Maharashtra										**ORF1a:E738R**	2	0.06
Maharashtra										**ORF1a:I739N**	2	0.06
Maharashtra										**ORF1a:I740Y**	2	0.06
Maharashtra										**ORF1a:F741L**	2	0.06
Maharashtra										**ORF1a:E745S**	2	0.06
Maharashtra										**ORF1a:T746C**	2	0.06
Maharashtra										**ORF1a:P748E**	2	0.06
Maharashtra										**ORF1a:T749N**	2	0.06
Maharashtra										**ORF1a:E750W**	2	0.06
Maharashtra										**ORF1a:V751***	2	0.06
Maharashtra										**ORF1a:L752F**	2	0.06
Maharashtra										**ORF1a:E772I**	2	0.06
Maharashtra										**ORF1a:A773G**	2	0.06
Maharashtra										**ORF1a:V774W**	2	0.06
Maharashtra										**ORF1a:E775Y**	2	0.06
Maharashtra										**ORF1a:A776T**	2	0.06

Maharashtra										**ORF1a:P777S**	2	0.06
Maharashtra										**ORF1a:T796***	2	0.06
Maharashtra										**ORF1a:E797R**	2	0.06
Maharashtra										**ORF1a:K798A**	2	0.06
Maharashtra										**ORF1a:V808H**	2	0.06
Maharashtra										**ORF1a:N811R**	2	0.06
Maharashtra										**ORF1a:T812R**	2	0.06
Maharashtra										**ORF1a:F813C**	2	0.06
Maharashtra										**ORF1a:L815N**	2	0.06
Maharashtra										**ORF1a:G835***	2	0.06
Maharashtra										**ORF1a:Y836***	2	0.06
Maharashtra										**ORF1a:I880H**	2	0.06
Maharashtra										**ORF1a:Y905G**	2	0.06
Maharashtra										**ORF1a:Y906H**	2	0.06
Maharashtra										**ORF1a:L907***	2	0.06
Maharashtra										**ORF1a:E961T**	2	0.06
Maharashtra										**ORF1a:S984R**	2	0.06
Maharashtra										**ORF1a:Q985R**	2	0.06
Maharashtra										**ORF1a:Q986R**	2	0.06
Maharashtra										**ORF1a:T987L**	2	0.06
Maharashtra										**ORF1a:T1001Y**	2	0.06
Maharashtra										**ORF1a:S2488F**	2	0.06
Maharashtra										**ORF1a:Y3811F**	2	0.06
Maharashtra										**ORF1a:R3880N**	2	0.06
Maharashtra										**ORF1a:E3882S**	2	0.06
Maharashtra										**ORF1a:P3952M**	2	0.06
Maharashtra										**ORF1a:N3970R**	2	0.06
Maharashtra										**ORF1a:G3971L**	2	0.06
Maharashtra										**ORF1a:D3972L**	2	0.06
Maharashtra										**ORF1a:S3973L**	2	0.06
Maharashtra										**ORF1a:E3974M**	2	0.06
Maharashtra										**ORF1a:T4159L**	2	0.06
Maharashtra										**ORF1a:T4175H**	2	0.06
Maharashtra										**ORF1a:G4177K**	2	0.06
Maharashtra										**ORF1a:F4180***	2	0.06
Maharashtra										**ORF1a:M4241T**	2	0.06
Maharashtra										**ORF1a:V4242W**	2	0.06
Maharashtra										**ORF1a:L4243***	2	0.06
Maharashtra										**ORF1a:G4244F**	2	0.06
Maharashtra										**ORF1a:L4246C**	2	0.06
Maharashtra										**ORF1a:A4247H**	2	0.06
Maharashtra										**ORF1a:A4248S**	2	0.06
Maharashtra										**ORF1a:G4287S**	2	0.06
Maharashtra										**ORF1a:G4288***	2	0.06
Maharashtra										**ORF1a:Q4289W**	2	0.06
Maharashtra										**ORF1a:P4290G**	2	0.06
Maharashtra										**ORF1a:I4291T**	2	0.06
Maharashtra										**ORF1a:V4295***	2	0.06
Maharashtra										**ORF1a:K4296D**	2	0.06
Maharashtra										**ORF1a:M4297V**	2	0.06
Maharashtra										**ORF1a:L4298V**	2	0.06
Maharashtra										**ORF1a:C4299Y**	2	0.06
Maharashtra										**ORF1a:F4321Q**	2	0.06
Maharashtra										**ORF1a:G4322Y**	2	0.06
Maharashtra										**ORF1a:A4324S**	2	0.06
Maharashtra										**ORF1a:S4325R**	2	0.06
Maharashtra										**ORF1a:C4326I**	2	0.06
Maharashtra										**ORF1a:C4327L**	2	0.06
Maharashtra										**ORF1a:L4328W**	2	0.06
Maharashtra										**ORF1a:Y4329W**	2	0.06
Maharashtra										**ORF1a:R4331I**	2	0.06
Maharashtra										**ORF1a:C4332V**	2	0.06
Maharashtra										**ORF1a:H4333L**	2	0.06
Maharashtra										**ORF1a:I4334S**	2	0.06
Maharashtra										**ORF1a:D4335V**	2	0.06
Maharashtra										**ORF1a:H4336L**	2	0.06
Maharashtra										**ORF1a:D4359***	2	0.06
Maharashtra										**ORF1a:V4361C**	2	0.06
Maharashtra										**ORF1a:T4364Y**	2	0.06
Maharashtra										**ORF1a:L4365T**	2	0.06
Maharashtra										**ORF1a:K4366***	2	0.06
Maharashtra										**ORF1a:N4367K**	2	0.06
Maharashtra										**ORF1a:T4368H**	2	0.06

Maharashtra										**ORF1a:V4369S**	2	0.06
Maharashtra										**ORF1a:H1141Y**	2	0.06
Maharashtra										**ORF1a:Q1129C**	2	0.06
Maharashtra										**ORF1a:D1184H**	2	0.06
Maharashtra										**ORF1a:L1186C**	2	0.06
Maharashtra										**ORF1a:V1187L**	2	0.06
Maharashtra										**ORF1a:F1190C**	2	0.06
Maharashtra										**ORF1a:K1781C**	2	0.06
Maharashtra										**ORF1a:G1782Y**	2	0.06
Maharashtra										**ORF1a:Q1784K**	2	0.06
Maharashtra										**ORF1a:C1787S**	2	0.06
Maharashtra										**ORF1a:C1789T**	2	0.06
Maharashtra										**ORF1a:Q1792V**	2	0.06
Maharashtra										**ORF1a:A1793T**	2	0.06
Maharashtra										**ORF1a:C2282L**	2	0.06
Maharashtra										**ORF1a:G2342C**	2	0.06
Maharashtra										**ORF1a:L2343C**	2	0.06
Maharashtra										**ORF1a:Q2373H**	2	0.06
Maharashtra										**ORF1a:A2375L**	2	0.06
Maharashtra										**ORF1a:I2541***	2	0.06
Maharashtra										**ORF1a:V2542R**	2	0.06
Maharashtra										**ORF1a:D2544C**	2	0.06
Maharashtra										**ORF1a:G2545K**	2	0.06
Maharashtra										**ORF1a:K2546I**	2	0.06
Maharashtra										**ORF1a:T2777***	2	0.06
Maharashtra										**ORF1a:D3355C**	2	0.06
Maharashtra										**ORF1a:T3356S**	2	0.06
Maharashtra										**ORF1a:A3357H**	2	0.06
Maharashtra										**ORF1a:P3359T**	2	0.06
Maharashtra										**ORF1a:K3360R**	2	0.06
Maharashtra										**ORF1a:Q3373D**	2	0.06
Maharashtra										**ORF1a:T3374F**	2	0.06
Maharashtra										**ORF1a:L3378P**	2	0.06
Maharashtra										**ORF1a:A3379M**	2	0.06
Maharashtra										**ORF1a:E797K**	2	0.06
Maharashtra										**ORF1a:T4174I**	2	0.06
Maharashtra										**ORF1a:K2511N**	2	0.06
Maharashtra										**ORF1a:T4090I**	2	0.06
Maharashtra										**ORF1a:V365I**	2	0.06
Maharashtra										**ORF1a:W630C**	2	0.06
Maharashtra										**ORF1a:S1029G**	2	0.06
Maharashtra										**ORF1a:E87D**	2	0.06
Maharashtra										**ORF1a:I404T**	2	0.06
Maharashtra										**ORF1a:T814I**	2	0.06
Maharashtra										**ORF1a:K1247N**	2	0.06
Maharashtra										**ORF1a:Q1292K**	2	0.06
Maharashtra										**ORF1a:E1363D**	2	0.06
Maharashtra										**ORF1a:M3129I**	2	0.06
Maharashtra										**ORF1a:R207C**	2	0.06
Maharashtra										**ORF1a:H388Y**	2	0.06
Maharashtra										**ORF1a:V1222I**	2	0.06
Maharashtra										**ORF1a:A1631V**	2	0.06
Maharashtra										**ORF1a:Q1201K**	2	0.06
Maharashtra										**ORF1a:A2584E**	2	0.06
Maharashtra										**ORF1a:Y3803D**	2	0.06
Maharashtra										**ORF1a:V649F**	2	0.06
Maharashtra										**ORF1a:R1464L**	2	0.06
Maharashtra										**ORF1a:S2303F**	2	0.06
Maharashtra										**ORF1a:N348K**	2	0.06
Maharashtra										**ORF1a:P4075S**	2	0.06
Maharashtra										**ORF1a:P2537L**	2	0.06
Maharashtra										**ORF1a:R3163G**	2	0.06
Maharashtra										**ORF1a:Y3649C**	2	0.06
Maharashtra										**ORF1a:D850N**	2	0.06
Maharashtra										**ORF1a:A2891T**	2	0.06
Maharashtra										**ORF1a:E347D**	2	0.06
Maharashtra										**ORF1a:R3993H**	2	0.06
Maharashtra										**ORF1a:A4394V**	2	0.06
Maharashtra										**ORF1a:Y3803N**	2	0.06
Maharashtra										**ORF1a:K798S**	2	0.06
Maharashtra										**ORF1a:K3982C**	2	0.06
Maharashtra										**ORF1a:L3984***	2	0.06
Maharashtra										**ORF1a:N3985K**	2	0.06

Maharashtra										**ORF1a:Q4011***	2	0.06
Maharashtra										**ORF1a:M4012V**	2	0.06
Maharashtra										**ORF1a:R4017***	2	0.06
Maharashtra										**ORF1a:D4020G**	2	0.06
Maharashtra										**ORF1a:N4046C**	2	0.06
Maharashtra										**ORF1a:N4047S**	2	0.06
Maharashtra										**ORF1a:I4048L**	2	0.06
Maharashtra										**ORF1a:I4049E**	2	0.06
Maharashtra										**ORF1a:N4050H**	2	0.06
Maharashtra										**ORF1a:D4200T**	2	0.06
Maharashtra										**ORF1a:A2129V**	2	0.06
Maharashtra										**ORF1a:R426H**	2	0.06
Maharashtra										**ORF1a:D3240A**	2	0.06
Maharashtra										**ORF1a:P3359L**	2	0.06
Maharashtra										**ORF1a:I4062L**	2	0.06
Maharashtra										**ORF1a:V38I**	2	0.06
Maharashtra										**ORF1a:E41M**	2	0.06
Maharashtra										**ORF1a:G59L**	2	0.06
Maharashtra										**ORF1a:V60N**	2	0.06
Maharashtra										**ORF1a:L61S**	2	0.06
Maharashtra										**ORF1a:K72M**	2	0.06
Maharashtra										**ORF1a:R73C**	2	0.06
Maharashtra										**ORF1a:D75S**	2	0.06
Maharashtra										**ORF1a:A76N**	2	0.06
Maharashtra										**ORF1a:T78R**	2	0.06
Maharashtra										**ORF1a:P80L**	2	0.06
Maharashtra										**ORF1a:H81E**	2	0.06
Maharashtra										**ORF1a:G82L**	2	0.06
Maharashtra										**ORF1a:V84L**	2	0.06
Maharashtra										**ORF1a:E87M**	2	0.06
Maharashtra										**ORF1a:E91W**	2	0.06
Maharashtra										**ORF1a:E93S**	2	0.06
Maharashtra										**ORF1a:G94W**	2	0.06
Maharashtra										**ORF1a:I95***	2	0.06
Maharashtra										**ORF1a:Y97N**	2	0.06
Maharashtra										**ORF1a:G98S**	2	0.06
Maharashtra										**ORF1a:R99K**	2	0.06
Maharashtra										**ORF1a:S100A**	2	0.06
Maharashtra										**ORF1a:G101F**	2	0.06
Maharashtra										**ORF1a:E102S**	2	0.06
Maharashtra										**ORF1a:L104V**	2	0.06
Maharashtra										**ORF1a:G105V**	2	0.06
Maharashtra										**ORF1a:P109H**	2	0.06
Maharashtra										**ORF1a:H110L**	2	0.06
Maharashtra										**ORF1a:G112T**	2	0.06
Maharashtra										**ORF1a:E113A**	2	0.06
Maharashtra										**ORF1a:I114R**	2	0.06
Maharashtra										**ORF1a:P115F**	2	0.06
Maharashtra										**ORF1a:V116F**	2	0.06
Maharashtra										**ORF1a:L122I**	2	0.06
Maharashtra										**ORF1a:L123K**	2	0.06
Maharashtra										**ORF1a:R124E**	2	0.06
Maharashtra										**ORF1a:K125L**	2	0.06
Maharashtra										**ORF1a:N126V**	2	0.06
Maharashtra										**ORF1a:G127A**	2	0.06
Maharashtra										**ORF1a:K129V**	2	0.06
Maharashtra										**ORF1a:W161I**	2	0.06
Maharashtra										**ORF1a:N162L**	2	0.06
Maharashtra										**ORF1a:C200A**	2	0.06
Maharashtra										**ORF1a:I201L**	2	0.06
Maharashtra										**ORF1a:K202M**	2	0.06
Maharashtra										**ORF1a:L204T**	2	0.06
Maharashtra										**ORF1a:F406P**	2	0.06
Maharashtra										**ORF1a:G407L**	2	0.06
Maharashtra										**ORF1a:A478M**	2	0.06
Maharashtra										**ORF1a:S479K**	2	0.06
Maharashtra										**ORF1a:F480R**	2	0.06
Maharashtra										**ORF1a:A482P**	2	0.06
Maharashtra										**ORF1a:S483L**	2	0.06
Maharashtra										**ORF1a:T484F**	2	0.06
Maharashtra										**ORF1a:S485W**	2	0.06
Maharashtra										**ORF1a:F487L**	2	0.06
Maharashtra										**ORF1a:E489L**	2	0.06

Maharashtra										**ORF1a:T490L**	2	0.06
Maharashtra										**ORF1a:V491P**	2	0.06
Maharashtra										**ORF1a:K492Q**	2	0.06
Maharashtra										**ORF1a:G493V**	2	0.06
Maharashtra										**ORF1a:G817Q**	2	0.06
Maharashtra										**ORF1a:G818T**	2	0.06
Maharashtra										**ORF1a:A819I**	2	0.06
Maharashtra										**ORF1a:K822L**	2	0.06
Maharashtra										**ORF1a:V823***	2	0.06
Maharashtra										**ORF1a:T824***	2	0.06
Maharashtra										**ORF1a:F825K**	2	0.06
Maharashtra										**ORF1a:G826C**	2	0.06
Maharashtra										**ORF1a:D827K**	2	0.06
Maharashtra										**ORF1a:D828V**	2	0.06
Maharashtra										**ORF1a:S838R**	2	0.06
Maharashtra										**ORF1a:G957P**	2	0.06
Maharashtra										**ORF1a:K958L**	2	0.06
Maharashtra										**ORF1a:A1151L**	2	0.06
Maharashtra										**ORF1a:E1341R**	2	0.06
Maharashtra										**ORF1a:A1342V**	2	0.06
Maharashtra										**ORF1a:K1343***	2	0.06
Maharashtra										**ORF1a:T1344M**	2	0.06
Maharashtra										**ORF1a:S1476***	2	0.06
Maharashtra										**ORF1a:D1481C**	2	0.06
Maharashtra										**ORF1a:A1482Q**	2	0.06
Maharashtra										**ORF1a:Q1799C**	2	0.06
Maharashtra										**ORF1a:E1801H**	2	0.06
Maharashtra										**ORF1a:S1802H**	2	0.06
Maharashtra										**ORF1a:P1803L**	2	0.06
Maharashtra										**ORF1a:T1840W**	2	0.06
Maharashtra										**ORF1a:K1842L**	2	0.06
Maharashtra										**ORF1a:E1843***	2	0.06
Maharashtra										**ORF1a:L1845R**	2	0.06
Maharashtra										**ORF1a:Y1846R**	2	0.06
Maharashtra										**ORF1a:I1848F**	2	0.06
Maharashtra										**ORF1a:G1850Y**	2	0.06
Maharashtra										**ORF1a:A1851K**	2	0.06
Maharashtra										**ORF1a:L1852V**	2	0.06
Maharashtra										**ORF1a:K1855I**	2	0.06
Maharashtra										**ORF1a:S1856Q**	2	0.06
Maharashtra										**ORF1a:S1857R**	2	0.06
Maharashtra										**ORF1a:E1858S**	2	0.06
Maharashtra										**ORF1a:K1860Y**	2	0.06
Maharashtra										**ORF1a:P1862C**	2	0.06
Maharashtra										**ORF1a:I1863F**	2	0.06
Maharashtra										**ORF1a:T1864L**	2	0.06
Maharashtra										**ORF1a:D1865Q**	2	0.06
Maharashtra										**ORF1a:V1866R**	2	0.06
Maharashtra										**ORF1a:F1867K**	2	0.06
Maharashtra										**ORF1a:Y1868Q**	2	0.06
Maharashtra										**ORF1a:K1869L**	2	0.06
Maharashtra										**ORF1a:E1870H**	2	0.06
Maharashtra										**ORF1a:S1872N**	2	0.06
Maharashtra										**ORF1a:Y1873H**	2	0.06
Maharashtra										**ORF1a:T1874K**	2	0.06
Maharashtra										**ORF1a:N2065R**	2	0.06
Maharashtra										**ORF1a:T2068Y**	2	0.06
Maharashtra										**ORF1a:T2124***	2	0.06
Maharashtra										**ORF1a:H2125C**	2	0.06
Maharashtra										**ORF1a:G2126P**	2	0.06
Maharashtra										**ORF1a:L2146Y**	2	0.06
Maharashtra										**ORF1a:C2263W**	2	0.06
Maharashtra										**ORF1a:T2264L**	2	0.06
Maharashtra										**ORF1a:G2265Q**	2	0.06
Maharashtra										**ORF1a:Y2266R**	2	0.06
Maharashtra										**ORF1a:E2268L**	2	0.06
Maharashtra										**ORF1a:G2269F**	2	0.06
Maharashtra										**ORF1a:Y2270E**	2	0.06
Maharashtra										**ORF1a:C2282Y**	2	0.06
Maharashtra										**ORF1a:D2296S**	2	0.06
Maharashtra										**ORF1a:S2297F**	2	0.06
Maharashtra										**ORF1a:L2298F**	2	0.06
Maharashtra										**ORF1a:D2299R**	2	0.06

Maharashtra										**ORF1a:T2300N**	2	0.06
Maharashtra										**ORF1a:P2302T**	2	0.06
Maharashtra										**ORF1a:S2303N**	2	0.06
Maharashtra										**ORF1a:L2304Y**	2	0.06
Maharashtra										**ORF1a:E2305H**	2	0.06
Maharashtra										**ORF1a:T2306F**	2	0.06
Maharashtra										**ORF1a:D2662***	2	0.06
Maharashtra										**ORF1a:N2682H**	2	0.06
Maharashtra										**ORF1a:Q2728T**	2	0.06
Maharashtra										**ORF1a:I2729N**	2	0.06
Maharashtra										**ORF1a:R2730T**	2	0.06
Maharashtra										**ORF1a:A2733C**	2	0.06
Maharashtra										**ORF1a:K2734***	2	0.06
Maharashtra										**ORF1a:N2736E**	2	0.06
Maharashtra										**ORF1a:N2737***	2	0.06
Maharashtra										**ORF1a:V2753K**	2	0.06
Maharashtra										**ORF1a:V2754D**	2	0.06
Maharashtra										**ORF1a:T2755S**	2	0.06
Maharashtra										**ORF1a:N2767***	2	0.06
Maharashtra										**ORF1a:N2768G**	2	0.06
Maharashtra										**ORF1a:H2799N**	2	0.06
Maharashtra										**ORF1a:D2801T**	2	0.06
Maharashtra										**ORF1a:F2802C**	2	0.06
Maharashtra										**ORF1a:S2804H**	2	0.06
Maharashtra										**ORF1a:D2819Y**	2	0.06
Maharashtra										**ORF1a:I2820L**	2	0.06
Maharashtra										**ORF1a:A2821F**	2	0.06
Maharashtra										**ORF1a:S2822C**	2	0.06
Maharashtra										**ORF1a:Y2897S**	2	0.06
Maharashtra										**ORF1a:T2898W**	2	0.06
Maharashtra										**ORF1a:K2901L**	2	0.06
Maharashtra										**ORF1a:V2914S**	2	0.06
Maharashtra										**ORF1a:A2916C**	2	0.06
Maharashtra										**ORF1a:A2917F**	2	0.06
Maharashtra										**ORF1a:E2918G**	2	0.06
Maharashtra										**ORF1a:T2920R**	2	0.06
Maharashtra										**ORF1a:I2921C**	2	0.06
Maharashtra										**ORF1a:Y2968W**	2	0.06
Maharashtra										**ORF1a:V2973S**	2	0.06
Maharashtra										**ORF1a:R2974G**	2	0.06
Maharashtra										**ORF1a:V2975N**	2	0.06
Maharashtra										**ORF1a:V2976N**	2	0.06
Maharashtra										**ORF1a:T2977F**	2	0.06
Maharashtra										**ORF1a:T2978***	2	0.06
Maharashtra										**ORF1a:D2980***	2	0.06
Maharashtra										**ORF1a:S2981V**	2	0.06
Maharashtra										**ORF1a:G3274***	2	0.06
Maharashtra										**ORF1a:K3275G**	2	0.06
Maharashtra										**ORF1a:V3276L**	2	0.06
Maharashtra										**ORF1a:E3277Y**	2	0.06
Maharashtra										**ORF1a:L3290S**	2	0.06
Maharashtra										**ORF1a:K3353S**	2	0.06
Maharashtra										**ORF1a:V3354***	2	0.06
Maharashtra										**ORF1a:F3366***	2	0.06
Maharashtra										**ORF1a:R3368***	2	0.06
Maharashtra										**ORF1a:Q3370C**	2	0.06
Maharashtra										**ORF1a:G3372H**	2	0.06
Maharashtra										**ORF1a:G3387Y**	2	0.06
Maharashtra										**ORF1a:V3388E**	2	0.06
Maharashtra										**ORF1a:Y3389A**	2	0.06
Maharashtra										**ORF1a:C3423P**	2	0.06
Maharashtra										**ORF1a:V3560R**	2	0.06
Maharashtra										**ORF1a:I3618Y**	2	0.06
Maharashtra										**ORF1a:I3619Y**	2	0.06
Maharashtra										**ORF1a:M3621Y**	2	0.06
Maharashtra										**ORF1a:S3622V**	2	0.06
Maharashtra										**ORF1a:A3623C**	2	0.06
Maharashtra										**ORF1a:L3641C**	2	0.06
Maharashtra										**ORF1a:P3642I**	2	0.06
Maharashtra										**ORF1a:S3949Y**	2	0.06
Maharashtra										**ORF1a:A1649Q**	2	0.06
Maharashtra										**ORF1a:G1850I**	2	0.06
Maharashtra										**ORF1a:A1851***	2	0.06

Maharashtra										**ORF1a:T1854K**	2	0.06
Maharashtra										**ORF1a:V2595Q**	2	0.06
Maharashtra										**ORF1a:N2596W**	2	0.06
Maharashtra										**ORF1a:T2597K**	2	0.06
Maharashtra										**ORF1a:M3129L**	2	0.06
Maharashtra										**ORF1a:T3459L**	2	0.06
Maharashtra										**ORF1a:D3460L**	2	0.06
Maharashtra										**ORF1a:D3526S**	2	0.06
Maharashtra										**ORF1a:M3527F**	2	0.06
Maharashtra										**ORF1a:S3530K**	2	0.06
Maharashtra										**ORF1a:L3531W**	2	0.06
Maharashtra										**ORF1a:K3532Y**	2	0.06
Maharashtra										**ORF1a:V3566Y**	2	0.06
Maharashtra										**ORF1a:F3677Y**	2	0.06
Maharashtra										**ORF1a:K3678G**	2	0.06
Maharashtra										**ORF1a:L3679***	2	0.06
Maharashtra										**ORF1a:K3680Y**	2	0.06
Maharashtra										**ORF1a:D3681***	2	0.06
Maharashtra										**ORF1a:C3682F**	2	0.06
Maharashtra										**ORF1a:M3684P**	2	0.06
Maharashtra										**ORF1a:A3686D**	2	0.06
Maharashtra										**ORF1a:Q4253T**	2	0.06
Maharashtra										**ORF1a:A4254S**	2	0.06
Maharashtra										**ORF1a:F70L**	2	0.06
Maharashtra										**ORF1a:L631F**	2	0.06
Maharashtra										**ORF1a:T4164I**	2	0.06
Maharashtra										**ORF1a:G2814S**	2	0.06
Maharashtra										**ORF1a:E1341D**	2	0.06
Maharashtra										**ORF1a:T2016I**	2	0.06
Maharashtra										**ORF1a:D335N**	2	0.06
Maharashtra										**ORF1a:M585E**	2	0.06
Maharashtra										**ORF1a:T587H**	2	0.06
Maharashtra										**ORF1a:E670L**	2	0.06
Maharashtra										**ORF1a:I671V**	2	0.06
Maharashtra										**ORF1a:Q676***	2	0.06
Maharashtra										**ORF1a:M789K**	2	0.06
Maharashtra										**ORF1a:F813V**	2	0.06
Maharashtra										**ORF1a:T814P**	2	0.06
Maharashtra										**ORF1a:T829A**	2	0.06
Maharashtra										**ORF1a:A878S**	2	0.06
Maharashtra										**ORF1a:I880Q**	2	0.06
Maharashtra										**ORF1a:T882Y**	2	0.06
Maharashtra										**ORF1a:T904H**	2	0.06
Maharashtra										**ORF1a:Y905W**	2	0.06
Maharashtra										**ORF1a:Y906A**	2	0.06
Maharashtra										**ORF1a:Y1183W**	2	0.06
Maharashtra										**ORF1a:D1184K**	2	0.06
Maharashtra										**ORF1a:L1186R**	2	0.06
Maharashtra										**ORF1a:R1225S**	2	0.06
Maharashtra										**ORF1a:E1909N**	2	0.06
Maharashtra										**ORF1a:P1911L**	2	0.06
Maharashtra										**ORF1a:D1913L**	2	0.06
Maharashtra										**ORF1a:L1914Y**	2	0.06
Maharashtra										**ORF1a:V1915Q**	2	0.06
Maharashtra										**ORF1a:P1916T**	2	0.06
Maharashtra										**ORF1a:Q2308L**	2	0.06
Maharashtra										**ORF1a:I2309***	2	0.06
Maharashtra										**ORF1a:I2311L**	2	0.06
Maharashtra										**ORF1a:L2341F**	2	0.06
Maharashtra										**ORF1a:A2732T**	2	0.06
Maharashtra										**ORF1a:A2733T**	2	0.06
Maharashtra										**ORF1a:W2837***	2	0.06
Maharashtra										**ORF1a:T3579Y**	2	0.06
Maharashtra										**ORF1a:H3580T**	2	0.06
Maharashtra										**ORF1a:R2860K**	2	0.06
Maharashtra										**ORF1a:N126T**	2	0.06
Maharashtra										**ORF1a:G127V**	2	0.06
Maharashtra										**ORF1a:D144E**	2	0.06
Maharashtra										**ORF1a:G345Y**	2	0.06
Maharashtra										**ORF1a:T346L**	2	0.06
Maharashtra										**ORF1a:E347W**	2	0.06
Maharashtra										**ORF1a:N348L**	2	0.06
Maharashtra										**ORF1a:H374A**	2	0.06

Maharashtra										**ORF1a:N375***	2	0.06
Maharashtra										**ORF1a:E377C**	2	0.06
Maharashtra										**ORF1a:V378R**	2	0.06
Maharashtra										**ORF1a:G379I**	2	0.06
Maharashtra										**ORF1a:E381***	2	0.06
Maharashtra										**ORF1a:H382***	2	0.06
Maharashtra										**ORF1a:S383I**	2	0.06
Maharashtra										**ORF1a:L384W**	2	0.06
Maharashtra										**ORF1a:A385L**	2	0.06
Maharashtra										**ORF1a:Y387N**	2	0.06
Maharashtra										**ORF1a:A405S**	2	0.06
Maharashtra										**ORF1a:F406S**	2	0.06
Maharashtra										**ORF1a:C420L**	2	0.06
Maharashtra										**ORF1a:Y422C**	2	0.06
Maharashtra										**ORF1a:V424L**	2	0.06
Maharashtra										**ORF1a:N508***	2	0.06
Maharashtra										**ORF1a:K510***	2	0.06
Maharashtra										**ORF1a:V511S**	2	0.06
Maharashtra										**ORF1a:T512Y**	2	0.06
Maharashtra										**ORF1a:G514R**	2	0.06
Maharashtra										**ORF1a:E525S**	2	0.06
Maharashtra										**ORF1a:Q526***	2	0.06
Maharashtra										**ORF1a:S528R**	2	0.06
Maharashtra										**ORF1a:I529C**	2	0.06
Maharashtra										**ORF1a:P532S**	2	0.06
Maharashtra										**ORF1a:S546C**	2	0.06
Maharashtra										**ORF1a:F548C**	2	0.06
Maharashtra										**ORF1a:S549I**	2	0.06
Maharashtra										**ORF1a:T551F**	2	0.06
Maharashtra										**ORF1a:L552T**	2	0.06
Maharashtra										**ORF1a:T554G**	2	0.06
Maharashtra										**ORF1a:A555R**	2	0.06
Maharashtra										**ORF1a:Q556Y**	2	0.06
Maharashtra										**ORF1a:S558N**	2	0.06
Maharashtra										**ORF1a:V559T**	2	0.06
Maharashtra										**ORF1a:V627***	2	0.06
Maharashtra										**ORF1a:P892S**	2	0.06
Maharashtra										**ORF1a:T945I**	2	0.06
Maharashtra										**ORF1a:Q946***	2	0.06
Maharashtra										**ORF1a:Y947V**	2	0.06
Maharashtra										**ORF1a:E948W**	2	0.06
Maharashtra										**ORF1a:G1456D**	2	0.06
Maharashtra										**ORF1a:E1503G**	2	0.06
Maharashtra										**ORF1a:T1504P**	2	0.06
Maharashtra										**ORF1a:Q2486L**	2	0.06
Maharashtra										**ORF1a:D2492S**	2	0.06
Maharashtra										**ORF1a:S2493I**	2	0.06
Maharashtra										**ORF1a:V2494F**	2	0.06
Maharashtra										**ORF1a:S2519I**	2	0.06
Maharashtra										**ORF1a:H2520K**	2	0.06
Maharashtra										**ORF1a:F2521L**	2	0.06
Maharashtra										**ORF1a:Y2594I**	2	0.06
Maharashtra										**ORF1a:A2621S**	2	0.06
Maharashtra										**ORF1a:A2701L**	2	0.06
Maharashtra										**ORF1a:Q2702T**	2	0.06
Maharashtra										**ORF1a:D2819L**	2	0.06
Maharashtra										**ORF1a:R3012I**	2	0.06
Maharashtra										**ORF1a:S3013Y**	2	0.06
Maharashtra										**ORF1a:T3058L**	2	0.06
Maharashtra										**ORF1a:C3059***	2	0.06
Maharashtra										**ORF1a:L3086T**	2	0.06
Maharashtra										**ORF1a:A3143***	2	0.06
Maharashtra										**ORF1a:I3145V**	2	0.06
Maharashtra										**ORF1a:T3432L**	2	0.06
Maharashtra										**ORF1a:G3433F**	2	0.06
Maharashtra										**ORF1a:D3526***	2	0.06
Maharashtra										**ORF1a:F3554D**	2	0.06
Maharashtra										**ORF1a:T3749Y**	2	0.06
Maharashtra										**ORF1a:T3750N**	2	0.06
Maharashtra										**ORF1a:I1359L**	2	0.06
Maharashtra										**ORF1a:L1650G**	2	0.06
Maharashtra										**ORF1a:R3805L**	2	0.06
Maharashtra										**ORF1a:V4147C**	2	0.06

Maharashtra										**ORF1a:A4148C**	2	0.06
Maharashtra										**ORF1a:L4149I**	2	0.06
Maharashtra										**ORF1a:K3236Q**	2	0.06
Maharashtra										**ORF1a:A3237V**	2	0.06
Maharashtra										**ORF1a:N3239M**	2	0.06
Maharashtra										**ORF1a:D3240F**	2	0.06
Maharashtra										**ORF1a:S3242T**	2	0.06
Maharashtra										**ORF1a:A3456V**	2	0.06
Maharashtra										**ORF1a:L140I**	2	0.06
Maharashtra										**ORF1a:T163S**	2	0.06
Maharashtra										**ORF1a:K164C**	2	0.06
Maharashtra										**ORF1a:A1074I**	2	0.06
Maharashtra										**ORF1a:S1587W**	2	0.06
Maharashtra										**ORF1a:M1588T**	2	0.06
Maharashtra										**ORF1a:W2316R**	2	0.06
Maharashtra										**ORF1a:D2317N**	2	0.06
Maharashtra										**ORF1a:L2318Y**	2	0.06
Maharashtra										**ORF1a:A2320V**	2	0.06
Maharashtra										**ORF1a:R2336S**	2	0.06
Maharashtra										**ORF1a:A1049V**	2	0.06
Maharashtra										**ORF1a:A1176V**	2	0.06
Maharashtra										**ORF1a:N3725S**	2	0.06
Maharashtra										**ORF1a:D992Y**	2	0.06
Maharashtra										**ORF1a:E1013D**	2	0.06
Maharashtra										**ORF1a:Y3160C**	2	0.06
Maharashtra										**ORF1a:F3605L**	2	0.06
Maharashtra										**ORF1a:H1545Y**	2	0.06
Maharashtra										**ORF1a:G1125C**	2	0.06
Maharashtra										**ORF1a:V2149F**	2	0.06
Maharashtra										**ORF1a:P4120S**	2	0.06
Maharashtra										**ORF1a:H1113Y**	2	0.06
Maharashtra										**ORF1a:S391F**	2	0.06
Maharashtra										**ORF1a:T1055I**	2	0.06
Maharashtra										**ORF1a:W646C**	2	0.06
Maharashtra										**ORF1a:S1133G**	2	0.06
Maharashtra										**ORF1a:L3338F**	2	0.06
Maharashtra										**ORF1a:P4337S**	2	0.06
Maharashtra										**ORF1a:K672N**	2	0.06
Maharashtra										**ORF1a:E933D**	2	0.06
Maharashtra										**ORF1a:A2587V**	2	0.06
Maharashtra										**ORF1a:S2535L**	2	0.06
Maharashtra										**ORF1a:S428G**	2	0.06
Maharashtra										**ORF1a:T1474I**	2	0.06
Maharashtra										**ORF1a:V1887F**	2	0.06
Maharashtra										**ORF1a:H1500R**	2	0.06
Maharashtra										**ORF1a:P765L**	2	0.06
Maharashtra										**ORF1a:N3484D**	2	0.06
Maharashtra										**ORF1a:A2460V**	2	0.06
Maharashtra										**ORF1a:L802F**	2	0.06
Maharashtra										**ORF1a:N313S**	2	0.06
Maharashtra										**ORF1a:Q2574R**	2	0.06
Maharashtra										**ORF1a:D335V**	2	0.06
Maharashtra										**ORF1a:K1668***	2	0.06
Maharashtra										**ORF1a:L2956F**	2	0.06
Maharashtra										**ORF1a:F1154Y**	2	0.06
Maharashtra										**ORF1a:I4334T**	2	0.06
Maharashtra										**ORF1a:T2021I**	2	0.06
Maharashtra										**ORF1a:L790F**	2	0.06
Maharashtra										**ORF1a:K1281R**	2	0.06
Maharashtra										**ORF1a:V620I**	2	0.06
Maharashtra										**ORF1a:A2614V**	2	0.06
Maharashtra										**ORF1a:T1822I**	2	0.06
Maharashtra										**ORF1a:N2405D**	2	0.06
Maharashtra										**ORF1a:P3395S**	2	0.06
Maharashtra										**ORF1a:Y1337N**	2	0.06
Maharashtra										**ORF1a:L2236V**	2	0.06
Maharashtra										**ORF1a:F143G**	2	0.06
Maharashtra										**ORF1a:P2221S**	2	0.06
Maharashtra										**ORF1a:L107F**	2	0.06
Maharashtra										**ORF1a:S1978F**	2	0.06
Maharashtra										**ORF1a:A3969V**	2	0.06
Maharashtra										**ORF1a:V1570H**	2	0.06
Maharashtra										**ORF1a:T2277L**	2	0.06

Maharashtra										**ORF1a:I2278L**	2	0.06
Maharashtra										**ORF1a:A2279Y**	2	0.06
Maharashtra										**ORF1a:T2280W**	2	0.06
Maharashtra										**ORF1a:P1609K**	2	0.06
Maharashtra										**ORF1a:C1847Q**	2	0.06
Maharashtra										**ORF1a:I1848N**	2	0.06
Maharashtra										**ORF1a:G1850K**	2	0.06
Maharashtra										**ORF1a:K1855M**	2	0.06
Maharashtra										**ORF1a:R207V**	2	0.06
Maharashtra										**ORF1a:G209V**	2	0.06
Maharashtra										**ORF1a:S318R**	2	0.06
Maharashtra										**ORF1a:T319H**	2	0.06
Maharashtra										**ORF1a:T356Y**	2	0.06
Maharashtra										**ORF1a:C357P**	2	0.06
Maharashtra										**ORF1a:G358K**	2	0.06
Maharashtra										**ORF1a:Y359M**	2	0.06
Maharashtra										**ORF1a:I463L**	2	0.06
Maharashtra										**ORF1a:V464M**	2	0.06
Maharashtra										**ORF1a:G465K**	2	0.06
Maharashtra										**ORF1a:D466R**	2	0.06
Maharashtra										**ORF1a:F467S**	2	0.06
Maharashtra										**ORF1a:K468P**	2	0.06
Maharashtra										**ORF1a:T829L**	2	0.06
Maharashtra										**ORF1a:S2797F**	2	0.06
Maharashtra										**ORF1a:S2273F**	2	0.06
Maharashtra										**ORF1a:R1386C**	2	0.06
Maharashtra										**ORF1a:L1149I**	2	0.06
Maharashtra										**ORF1a:A1420***	2	0.06
Maharashtra										**ORF1a:R1421N**	2	0.06
Maharashtra										**ORF1a:F1422N**	2	0.06
Maharashtra										**ORF1a:Y1423C**	2	0.06
Maharashtra										**ORF1a:F1424S**	2	0.06
Maharashtra										**ORF1a:Y1425V**	2	0.06
Maharashtra										**ORF1a:S1427Y**	2	0.06
Maharashtra										**ORF1a:K1428Q**	2	0.06
Maharashtra										**ORF1a:T1429H**	2	0.06
Maharashtra										**ORF1a:I1505L**	2	0.06
Maharashtra										**ORF1a:S1506V**	2	0.06
Maharashtra										**ORF1a:V86L**	2	0.06
Maharashtra										**ORF1a:L320Q**	2	0.06
Maharashtra										**ORF1a:K322N**	2	0.06
Maharashtra										**ORF1a:C323A**	2	0.06
Maharashtra										**ORF1a:D324T**	2	0.06
Maharashtra										**ORF1a:H325K**	2	0.06
Maharashtra										**ORF1a:V1968L**	2	0.06
Maharashtra										**ORF1a:A1969I**	2	0.06
Maharashtra										**ORF1a:D1971N**	2	0.06
Maharashtra										**ORF1a:Y1972T**	2	0.06
Maharashtra										**ORF1a:K1973T**	2	0.06
Maharashtra										**ORF1a:W3709D**	2	0.06
Maharashtra										**ORF1a:L4033H**	2	0.06
Maharashtra										**ORF1a:F4034S**	2	0.06
Maharashtra										**ORF1a:L2146F**	2	0.06
Maharashtra										**ORF1a:D571E**	2	0.06
Maharashtra										**ORF1a:T619N**	2	0.06
Maharashtra										**ORF1a:T554N**	2	0.06
Maharashtra										**ORF1a:T2152I**	2	0.06
Maharashtra										**ORF1a:F1960L**	2	0.06
Maharashtra										**ORF1a:A206T**	2	0.06
Maharashtra										**ORF1a:K1396R**	2	0.06
Maharashtra										**ORF1a:S3244L**	2	0.06
Maharashtra										**ORF1a:D3972G**	2	0.06
Maharashtra										**ORF1a:M789I**	2	0.06
Maharashtra										**ORF1a:I1091T**	2	0.06
Maharashtra										**ORF1a:T4355I**	2	0.06
Maharashtra										**ORF1a:K2928N**	2	0.06
Maharashtra										**ORF1a:S100N**	2	0.06
Maharashtra										**ORF1a:T2791I**	2	0.06
Maharashtra										**ORF1a:L3060F**	2	0.06
Maharashtra										**ORF1a:S505F**	2	0.06
Maharashtra										**ORF1a:Y229H**	2	0.06
Maharashtra										**ORF1a:P197S**	2	0.06
Maharashtra										**ORF1a:G150C**	2	0.06

Maharashtra										**ORF1a:V1287A**	2	0.06
Maharashtra										**ORF1a:S2193F**	2	0.06
Maharashtra										**ORF1a:G2258D**	2	0.06
Maharashtra										**ORF1a:A3497V**	2	0.06
Maharashtra										**ORF1a:Q2563H**	2	0.06
Maharashtra										**ORF1a:A735V**	2	0.06
Maharashtra										**ORF1a:D2880E**	2	0.06
Maharashtra										**ORF1a:A2033T**	2	0.06
Maharashtra										**ORF1a:R1225I**	2	0.06
Maharashtra										**ORF1a:V1431I**	2	0.06
Maharashtra										**ORF1a:G2265V**	2	0.06
Maharashtra										**ORF1a:K247N**	2	0.06
Maharashtra										**ORF1a:I3944M**	2	0.06
Maharashtra										**ORF1a:T2836I**	2	0.06
Maharashtra										**ORF1a:M2957T**	2	0.06
Maharashtra										**ORF1a:D1926N**	2	0.06
Maharashtra										**ORF1a:G295V**	2	0.06
Maharashtra										**ORF1a:D335E**	2	0.06
Maharashtra										**ORF1a:V1187F**	2	0.06
Maharashtra										**ORF1a:I3693V**	2	0.06
Maharashtra										**ORF1a:M2410V**	2	0.06
Maharashtra										**ORF1a:T3117N**	2	0.06
Maharashtra										**ORF1a:T2877I**	2	0.06
Maharashtra										**ORF1a:V1063R**	2	0.06
Maharashtra										**ORF1a:Y1064S**	2	0.06
Maharashtra										**ORF1a:F1178S**	2	0.06
Maharashtra										**ORF1a:D1179H**	2	0.06
Maharashtra										**ORF1a:D1289***	2	0.06
Maharashtra										**ORF1a:V1290C**	2	0.06
Maharashtra										**ORF1a:V1291C**	2	0.06
Maharashtra										**ORF1a:Q1292S**	2	0.06
Maharashtra										**ORF1a:T1303C**	2	0.06
Maharashtra										**ORF1a:K1304F**	2	0.06
Maharashtra										**ORF1a:A1306C**	2	0.06
Maharashtra										**ORF1a:G1307C**	2	0.06
Maharashtra										**ORF1a:P1472***	2	0.06
Maharashtra										**ORF1a:A1473C**	2	0.06
Maharashtra										**ORF1a:T1474C**	2	0.06
Maharashtra										**ORF1a:V1475Y**	2	0.06
Maharashtra										**ORF1a:S1478***	2	0.06
Maharashtra										**ORF1a:S1479W**	2	0.06
Maharashtra										**ORF1a:P1480L**	2	0.06
Maharashtra										**ORF1a:A1482Y**	2	0.06
Maharashtra										**ORF1a:V1483F**	2	0.06
Maharashtra										**ORF1a:T1484F**	2	0.06
Maharashtra										**ORF1a:Y1486***	2	0.06
Maharashtra										**ORF1a:P1497L**	2	0.06
Maharashtra										**ORF1a:T1581G**	2	0.06
Maharashtra										**ORF1a:Q1582H**	2	0.06
Maharashtra										**ORF1a:F3624C**	2	0.06
Maharashtra										**ORF1a:A3625I**	2	0.06
Maharashtra										**ORF1a:M3626S**	2	0.06
Maharashtra										**ORF1a:M3627L**	2	0.06
Maharashtra										**ORF1a:A3645C**	2	0.06
Maharashtra										**ORF1a:T3646P**	2	0.06
Maharashtra										**ORF1a:S1587L**	2	0.06
Maharashtra										**ORF1a:T3308I**	2	0.06
Maharashtra										**ORF1a:D1554Y**	2	0.06
Maharashtra										**ORF1a:A2325V**	2	0.06
Maharashtra										**ORF1a:T654I**	2	0.06
Maharashtra										**ORF1a:G963D**	2	0.06
Maharashtra										**ORF1a:L705F**	2	0.06
Maharashtra										**ORF1a:V14A**	2	0.06
Maharashtra										**ORF1a:D1229G**	2	0.06
Maharashtra										**ORF1a:P1018S**	2	0.06
Maharashtra										**ORF1a:V106I**	2	0.06
Maharashtra										**ORF1a:S1733G**	2	0.06
Maharashtra										**ORF1a:T2274I**	2	0.06
Maharashtra										**ORF1a:T2166I**	2	0.06
Maharashtra										**ORF1a:A2696V**	2	0.06
Maharashtra										**ORF1a:E1205D**	2	0.06
Maharashtra										**ORF1a:D1323G**	2	0.06
Maharashtra										**ORF1a:G440R**	2	0.06

Maharashtra										ORF1a:V306F	2	0.06
Maharashtra										ORF1a:V660A	2	0.06
Maharashtra										ORF1a:S3C	2	0.06
Maharashtra										ORF1a:G728C	2	0.06
Maharashtra										ORF1a:K1231R	2	0.06
Maharashtra										ORF1a:V2491I	2	0.06
Maharashtra										ORF1a:E102K	2	0.06
Maharashtra										ORF1a:G392C	2	0.06
Maharashtra										ORF1a:T3579I	2	0.06
Maharashtra										ORF1a:G82S	2	0.06
Maharashtra										ORF1a:T4311A	2	0.06
Maharashtra										ORF1a:G415S	2	0.06
Maharashtra										ORF1a:M1A	2	0.06
Maharashtra										ORF1a:E2K	2	0.06
Maharashtra										ORF1a:S3R	2	0.06
Maharashtra										ORF1a:V5F	2	0.06
Maharashtra										ORF1a:V60A	2	0.06
Maharashtra										ORF1a:L3781I	2	0.06
Maharashtra										ORF1a:D996Y	2	0.06
Maharashtra										ORF1a:L3919F	2	0.06
Maharashtra										ORF1a:E1614A	2	0.06
Maharashtra										ORF1a:A2001T	2	0.06
Maharashtra										ORF1a:A3070V	2	0.06
Maharashtra										ORF1a:G1119S	2	0.06
Maharashtra										ORF1a:G137S	2	0.06
Maharashtra										ORF1a:I1355V	2	0.06
Maharashtra										ORF1a:V1058I	2	0.06
Maharashtra										ORF1a:T1542I	2	0.06
Maharashtra										ORF1a:P1692S	2	0.06
Maharashtra										ORF1a:A2994V	2	0.06
Maharashtra										ORF1a:T3150I	2	0.06
Maharashtra										ORF1a:N1081K	2	0.06
Maharashtra										ORF1a:E3608A	2	0.06
Maharashtra										ORF1a:T2800I	2	0.06
Maharashtra										ORF1a:V111L	2	0.06
Maharashtra										ORF1a:P748L	2	0.06
Maharashtra										ORF1a:T1430A	2	0.06
Maharashtra										ORF1a:P971L	2	0.06
Maharashtra										ORF1a:K1233E	2	0.06
Maharashtra										ORF1a:I1153F	2	0.06
Maharashtra										ORF1a:A1082L	2	0.06
Maharashtra										ORF1a:P19R	2	0.06
Maharashtra										ORF1a:L1105*	2	0.06
Maharashtra										ORF1a:H1108W	2	0.06
Maharashtra										ORF1a:N1109*	2	0.06
Maharashtra										ORF1a:N2275L	2	0.06
Maharashtra										ORF1a:G2667I	2	0.06
Maharashtra										ORF1a:D2668C	2	0.06
Maharashtra										ORF1a:I2873C	2	0.06
Maharashtra										ORF1a:A3049L	2	0.06
Maharashtra										ORF1a:G3050P	2	0.06
Maharashtra										ORF1a:G3051T	2	0.06
Maharashtra										ORF1a:V3053L	2	0.06
Maharashtra										ORF1a:I3055G	2	0.06
Maharashtra										ORF1a:V3056L	2	0.06
Maharashtra										ORF1a:V3057E	2	0.06
Maharashtra										ORF1a:M3626V	2	0.06
Maharashtra										ORF1a:M3627C	2	0.06
Maharashtra										ORF1a:T4302I	2	0.06
Maharashtra										ORF1a:P4389S	2	0.06
Maharashtra										ORF1a:R1566S	2	0.06
Maharashtra										ORF1a:S483F	2	0.06
Maharashtra										ORF1a:A2856V	2	0.06
Maharashtra										ORF1a:G1095R	2	0.06
Maharashtra										ORF1a:T4159I	2	0.06
Maharashtra										ORF1a:V1046A	2	0.06
Maharashtra										ORF1a:Q1592R	2	0.06
Maharashtra										ORF1a:S2352G	2	0.06
Maharashtra										ORF1a:R3993L	2	0.06
Maharashtra										ORF1a:L3210F	2	0.06
Maharashtra										ORF1a:T3224N	2	0.06
Maharashtra										ORF1a:H2357Y	2	0.06
Maharashtra										ORF1a:V2130F	2	0.06

Maharashtra										**ORF1a:T4371A**	2	0.06
Maharashtra										**ORF1a:S2024L**	2	0.06
Maharashtra										**ORF1a:D1513E**	2	0.06
Maharashtra										**ORF1a:L1559I**	2	0.06
Maharashtra										**ORF1a:K2017R**	2	0.06
Maharashtra										**ORF1a:A3924V**	2	0.06
Maharashtra										**ORF1a:S2517F**	2	0.06
Maharashtra										**ORF1a:T1874A**	2	0.06
Maharashtra										**ORF1a:V116M**	2	0.06
Maharashtra										**ORF1a:K3832N**	2	0.06
Maharashtra										**ORF1a:V2019F**	2	0.06
Maharashtra										**ORF1a:I3771T**	2	0.06
Maharashtra										**ORF1a:E2112A**	2	0.06
Maharashtra										**ORF1a:E933K**	2	0.06
Maharashtra										**ORF1a:L2869F**	2	0.06
Maharashtra										**ORF1a:K1348N**	2	0.06
Maharashtra										**ORF1a:G2808E**	2	0.06
Maharashtra										**ORF1a:M3425I**	2	0.06
Maharashtra										**ORF1a:D4335N**	2	0.06
Maharashtra										**ORF1a:E743D**	2	0.06
Maharashtra										**ORF1a:L2902I**	2	0.06
Maharashtra										**ORF1a:D2136N**	2	0.06
Maharashtra										**ORF1a:A2784V**	2	0.06
Maharashtra										**ORF1a:M3997I**	2	0.06
Maharashtra										**ORF1a:S558F**	2	0.06
Maharashtra										**ORF1a:N2894S**	2	0.06
Maharashtra										**ORF1a:T842I**	2	0.06
Maharashtra										**ORF1a:V2865I**	2	0.06
Maharashtra										**ORF1a:T4258I**	2	0.06
Maharashtra										**ORF1a:R3368H**	2	0.06
Maharashtra										**ORF1a:A3548V**	2	0.06
Maharashtra										**ORF1a:D1157A**	2	0.06
Maharashtra										**ORF1a:G950V**	2	0.06
Maharashtra										**ORF1a:Q2510H**	2	0.06
Maharashtra										**ORF1a:V2090F**	2	0.06
Maharashtra										**ORF1a:T4265I**	2	0.06
Maharashtra										**ORF1a:F678I**	2	0.06
Maharashtra										**ORF1a:S531N**	2	0.06
Maharashtra										**ORF1a:S2285F**	2	0.06
Maharashtra										**ORF1a:G993D**	2	0.06
Maharashtra										**ORF1a:V3975F**	2	0.06
Maharashtra										**ORF1a:S2474L**	1	0.03
Maharashtra										**ORF1a:V2496S**	1	0.03
Maharashtra										**ORF1a:K2497L**	1	0.03
Maharashtra										**ORF1a:G2499T**	1	0.03
Maharashtra										**ORF1a:F3157V**	1	0.03
Maharashtra										**ORF1a:N3159V**	1	0.03
Maharashtra										**ORF1a:Y3160I**	1	0.03
Maharashtra										**ORF1a:F4230H**	1	0.03
Maharashtra										**ORF1a:Q4253Y**	1	0.03
Maharashtra										**ORF1a:A4254K**	1	0.03
Maharashtra										**ORF1a:G4255L**	1	0.03
Maharashtra										**ORF1a:N4256G**	1	0.03
Maharashtra										**ORF1a:A1397T**	1	0.03
Maharashtra										**ORF1a:A3357T**	1	0.03
Maharashtra										**ORF1a:G137***	1	0.03
Maharashtra										**ORF1a:D139T**	1	0.03
Maharashtra										**ORF1a:L140S**	1	0.03
Maharashtra										**ORF1a:K141L**	1	0.03
Maharashtra										**ORF1a:D144I**	1	0.03
Maharashtra										**ORF1a:T350K**	1	0.03
Maharashtra										**ORF1a:V366P**	1	0.03
Maharashtra										**ORF1a:K367L**	1	0.03
Maharashtra										**ORF1a:I368L**	1	0.03
Maharashtra										**ORF1a:Y369V**	1	0.03
Maharashtra										**ORF1a:G604F**	1	0.03
Maharashtra										**ORF1a:V605S**	1	0.03
Maharashtra										**ORF1a:Q607D**	1	0.03
Maharashtra										**ORF1a:L608F**	1	0.03
Maharashtra										**ORF1a:V660C**	1	0.03
Maharashtra										**ORF1a:G661K**	1	0.03
Maharashtra										**ORF1a:N810***	1	0.03
Maharashtra										**ORF1a:T1000N**	1	0.03

Maharashtra										**ORF1a:K1197E**	1	0.03
Maharashtra										**ORF1a:A3079L**	1	0.03
Maharashtra										**ORF1a:Q3251H**	1	0.03
Maharashtra										**ORF1a:G3546H**	1	0.03
Maharashtra										**ORF1a:S3547L**	1	0.03
Maharashtra										**ORF1a:A3548L**	1	0.03
Maharashtra										**ORF1a:L3549M**	1	0.03
Maharashtra										**ORF1a:E3551L**	1	0.03
Maharashtra										**ORF1a:M3626F**	1	0.03
Maharashtra										**ORF1a:A3634T**	1	0.03
Maharashtra										**ORF1a:L3636S**	1	0.03
Maharashtra										**ORF1a:L3640***	1	0.03
Maharashtra										**ORF1a:L3641Y**	1	0.03
Maharashtra										**ORF1a:P3642G**	1	0.03
Maharashtra										**ORF1a:S3643L**	1	0.03
Maharashtra										**ORF1a:L3644Y**	1	0.03
Maharashtra										**ORF1a:T3665C**	1	0.03
Maharashtra										**ORF1a:W3666***	1	0.03
Maharashtra										**ORF1a:Y3783L**	1	0.03
Maharashtra										**ORF1a:G3809W**	1	0.03
Maharashtra										**ORF1a:V3810C**	1	0.03
Maharashtra										**ORF1a:S3884F**	1	0.03
Maharashtra										**ORF1a:K3886***	1	0.03
Maharashtra										**ORF1a:D3897K**	1	0.03
Maharashtra										**ORF1a:S3913K**	1	0.03
Maharashtra										**ORF1a:L3914P**	1	0.03
Maharashtra										**ORF1a:N4046H**	1	0.03
Maharashtra										**ORF1a:I4048T**	1	0.03
Maharashtra										**ORF1a:I4049S**	1	0.03
Maharashtra										**ORF1a:N4050Y**	1	0.03
Maharashtra										**ORF1a:P4220I**	1	0.03
Maharashtra										**ORF1a:G4222Q**	1	0.03
Maharashtra										**ORF1a:V4225***	1	0.03
Maharashtra										**ORF1a:K4226R**	1	0.03
Maharashtra										**ORF1a:A4257S**	1	0.03
Maharashtra										**ORF1a:E4259S**	1	0.03
Maharashtra										**ORF1a:V4260W**	1	0.03
Maharashtra										**ORF1a:P4261E**	1	0.03
Maharashtra										**ORF1a:A4262H**	1	0.03
Maharashtra										**ORF1a:D4282L**	1	0.03
Maharashtra										**ORF1a:Y4283Q**	1	0.03
Maharashtra										**ORF1a:L4284R**	1	0.03
Maharashtra										**ORF1a:A4285L**	1	0.03
Maharashtra										**ORF1a:P4339T**	1	0.03
Maharashtra										**ORF1a:K4340Y**	1	0.03
Maharashtra										**ORF1a:G4341N**	1	0.03
Maharashtra										**ORF1a:F4342L**	1	0.03
Maharashtra										**ORF1a:N4358***	1	0.03
Maharashtra										**ORF1a:C4370L**	1	0.03
Maharashtra										**ORF1a:T4371Y**	1	0.03
Maharashtra										**ORF1a:L883S**	1	0.03
Maharashtra										**ORF1a:Q884S**	1	0.03
Maharashtra										**ORF1a:S887W**	1	0.03
Maharashtra										**ORF1a:E888A**	1	0.03
Maharashtra										**ORF1a:L890I**	1	0.03
Maharashtra										**ORF1a:T891***	1	0.03
Maharashtra										**ORF1a:P892M**	1	0.03
Maharashtra										**ORF1a:L893S**	1	0.03
Maharashtra										**ORF1a:I895V**	1	0.03
Maharashtra										**ORF1a:D896W**	1	0.03
Maharashtra										**ORF1a:Y905T**	1	0.03
Maharashtra										**ORF1a:C922M**	1	0.03
Maharashtra										**ORF1a:E948P**	1	0.03
Maharashtra										**ORF1a:Y949R**	1	0.03
Maharashtra										**ORF1a:P1911K**	1	0.03
Maharashtra										**ORF1a:I1912R**	1	0.03
Maharashtra										**ORF1a:D1913K**	1	0.03
Maharashtra										**ORF1a:D1926R**	1	0.03
Maharashtra										**ORF1a:N1927***	1	0.03
Maharashtra										**ORF1a:Y2281V**	1	0.03
Maharashtra										**ORF1a:L2341D**	1	0.03
Maharashtra										**ORF1a:A2344N**	1	0.03
Maharashtra										**ORF1a:V2401I**	1	0.03

Maharashtra										**ORF1a:D2402L**	1	0.03
Maharashtra										**ORF1a:G2403L**	1	0.03
Maharashtra										**ORF1a:N2405M**	1	0.03
Maharashtra										**ORF1a:S2406E**	1	0.03
Maharashtra										**ORF1a:S2407K**	1	0.03
Maharashtra										**ORF1a:T2408L**	1	0.03
Maharashtra										**ORF1a:S2462T**	1	0.03
Maharashtra										**ORF1a:I2481V**	1	0.03
Maharashtra										**ORF1a:Q2724***	1	0.03
Maharashtra										**ORF1a:R2748F**	1	0.03
Maharashtra										**ORF1a:V2751***	1	0.03
Maharashtra										**ORF1a:V2776***	1	0.03
Maharashtra										**ORF1a:L2882F**	1	0.03
Maharashtra										**ORF1a:S2890Y**	1	0.03
Maharashtra										**ORF1a:A2891R**	1	0.03
Maharashtra										**ORF1a:A2916H**	1	0.03
Maharashtra										**ORF1a:A2917***	1	0.03
Maharashtra										**ORF1a:E2918L**	1	0.03
Maharashtra										**ORF1a:S3158L**	1	0.03
Maharashtra										**ORF1a:N3159L**	1	0.03
Maharashtra										**ORF1a:Y3160V**	1	0.03
Maharashtra										**ORF1a:K3162F**	1	0.03
Maharashtra										**ORF1a:R3163***	1	0.03
Maharashtra										**ORF1a:L3180S**	1	0.03
Maharashtra										**ORF1a:T3182A**	1	0.03
Maharashtra										**ORF1a:F3183V**	1	0.03
Maharashtra										**ORF1a:L3184H**	1	0.03
Maharashtra										**ORF1a:Y3190F**	1	0.03
Maharashtra										**ORF1a:L3330T**	1	0.03
Maharashtra										**ORF1a:V3331F**	1	0.03
Maharashtra										**ORF1a:Q3332Y**	1	0.03
Maharashtra										**ORF1a:N3405E**	1	0.03
Maharashtra										**ORF1a:G3406A**	1	0.03
Maharashtra										**ORF1a:S3407Q**	1	0.03
Maharashtra										**ORF1a:C3408F**	1	0.03
Maharashtra										**ORF1a:G3409H**	1	0.03
Maharashtra										**ORF1a:S3410Y**	1	0.03
Maharashtra										**ORF1a:D1585N**	1	0.03
Maharashtra										**ORF1a:F1618***	1	0.03
Maharashtra										**ORF1a:Y1619N**	1	0.03
Maharashtra										**ORF1a:S3601F**	1	0.03
Maharashtra										**ORF1a:A537V**	1	0.03
Maharashtra										**ORF1a:T3904I**	1	0.03
Maharashtra										**ORF1a:G227S**	1	0.03
Maharashtra										**ORF1a:L2367R**	1	0.03
Maharashtra										**ORF1a:Q1943H**	1	0.03
Maharashtra										**ORF1a:E36H**	1	0.03
Maharashtra										**ORF1a:E37G**	1	0.03
Maharashtra										**ORF1a:L39S**	1	0.03
Maharashtra										**ORF1a:A181Q**	1	0.03
Maharashtra										**ORF1a:G334R**	1	0.03
Maharashtra										**ORF1a:F336L**	1	0.03
Maharashtra										**ORF1a:V337I**	1	0.03
Maharashtra										**ORF1a:K338***	1	0.03
Maharashtra										**ORF1a:V35I**	1	0.03
Maharashtra										**ORF1a:T727N**	1	0.03
Maharashtra										**ORF1a:G989S**	1	0.03
Maharashtra										**ORF1a:T1242N**	1	0.03
Maharashtra										**ORF1a:L1243I**	1	0.03
Maharashtra										**ORF1a:E1244K**	1	0.03
Maharashtra										**ORF1a:E1245I**	1	0.03
Maharashtra										**ORF1a:K1247V**	1	0.03
Maharashtra										**ORF1a:F1248M**	1	0.03
Maharashtra										**ORF1a:L1852F**	1	0.03
Maharashtra										**ORF1a:G1861L**	1	0.03
Maharashtra										**ORF1a:P1862Q**	1	0.03
Maharashtra										**ORF1a:I3043T**	1	0.03
Maharashtra										**ORF1a:K4000H**	1	0.03
Maharashtra										**ORF1a:L4001S**	1	0.03
Maharashtra										**ORF1a:E4002F**	1	0.03
Maharashtra										**ORF1a:K4003G**	1	0.03
Maharashtra										**ORF1a:M4004W**	1	0.03
Maharashtra										**ORF1a:A4005L**	1	0.03

Maharashtra										**ORF1a:D4006I**	1	0.03
Maharashtra										**ORF1a:Q4007V**	1	0.03
Maharashtra										**ORF1a:A4008G**	1	0.03
Maharashtra										**ORF1a:M4009V**	1	0.03
Maharashtra										**ORF1a:T4087L**	1	0.03
Maharashtra										**ORF1a:T4088H**	1	0.03
Maharashtra										**ORF1a:F4089C**	1	0.03
Maharashtra										**ORF1a:T4090N**	1	0.03
Maharashtra										**ORF1a:Y4091N**	1	0.03
Maharashtra										**ORF1a:A4092Y**	1	0.03
Maharashtra										**ORF1a:S4093Q**	1	0.03
Maharashtra										**ORF1a:G1288D**	1	0.03
Maharashtra										**ORF1a:D1289V**	1	0.03
Maharashtra										**ORF1a:V1291S**	1	0.03
Maharashtra										**ORF1a:T1297L**	1	0.03
Maharashtra										**ORF1a:K1364N**	1	0.03
Maharashtra										**ORF1a:S2030L**	1	0.03
Maharashtra										**ORF1a:R226S**	1	0.03
Maharashtra										**ORF1a:R184C**	1	0.03
Maharashtra										**ORF1a:V3718I**	1	0.03
Maharashtra										**ORF1a:A3040V**	1	0.03
Maharashtra										**ORF1a:A427V**	1	0.03
Maharashtra										**ORF1a:N3537D**	1	0.03
Maharashtra										**ORF1a:T1168I**	1	0.03
Maharashtra										**ORF1a:P2483L**	1	0.03
Maharashtra										**ORF1a:W2837R**	1	0.03
Maharashtra										**ORF1a:R550H**	1	0.03
Maharashtra										**ORF1a:S1106N**	1	0.03
Maharashtra										**ORF1a:A591T**	1	0.03
Maharashtra										**ORF1a:M2719I**	1	0.03
Maharashtra										**ORF1a:E972D**	1	0.03
Maharashtra										**ORF1a:G358C**	1	0.03
Maharashtra										**ORF1a:A2529V**	1	0.03
Maharashtra										**ORF1a:D4218E**	1	0.03
Maharashtra										**ORF1a:R1464W**	1	0.03
Maharashtra										**ORF1a:Q1224H**	1	0.03
Maharashtra										**ORF1a:I3737V**	1	0.03
Maharashtra										**ORF1a:V1583F**	1	0.03
Maharashtra										**ORF1a:L1130F**	1	0.03
Maharashtra										**ORF1a:I473S**	1	0.03
Maharashtra										**ORF1a:T2532I**	1	0.03
Maharashtra										**ORF1a:L454R**	1	0.03
Maharashtra										**ORF1a:I2569V**	1	0.03
Maharashtra										**ORF1a:K2078E**	1	0.03
Maharashtra										**ORF1a:L3330F**	1	0.03
Maharashtra										**ORF1a:Q1799K**	1	0.03
Maharashtra										**ORF1a:E347K**	1	0.03
Maharashtra										**ORF1a:T727I**	1	0.03
Maharashtra										**ORF1a:L16I**	1	0.03
Maharashtra										**ORF1a:K1495R**	1	0.03
Maharashtra										**ORF1a:S2466G**	1	0.03
Maharashtra										**ORF1a:P2480L**	1	0.03
Maharashtra										**ORF1a:I2481L**	1	0.03
Maharashtra										**ORF1a:P2483C**	1	0.03
Maharashtra										**ORF1a:T2484S**	1	0.03
Maharashtra										**ORF1a:K2533A**	1	0.03
Maharashtra										**ORF1a:G2534T**	1	0.03
Maharashtra										**ORF1a:L2536S**	1	0.03
Maharashtra										**ORF1a:I2541L**	1	0.03
Maharashtra										**ORF1a:V2542P**	1	0.03
Maharashtra										**ORF1a:F2543A**	1	0.03
Maharashtra										**ORF1a:D2544Q**	1	0.03
Maharashtra										**ORF1a:G2545N**	1	0.03
Maharashtra										**ORF1a:K2546V**	1	0.03
Maharashtra										**ORF1a:K2548Q**	1	0.03
Maharashtra										**ORF1a:C2549L**	1	0.03
Maharashtra										**ORF1a:E2550T**	1	0.03
Maharashtra										**ORF1a:E2551Q**	1	0.03
Maharashtra										**ORF1a:S2553I**	1	0.03
Maharashtra										**ORF1a:K2555***	1	0.03
Maharashtra										**ORF1a:S2556F**	1	0.03
Maharashtra										**ORF1a:S2558I**	1	0.03
Maharashtra										**ORF1a:V2559L**	1	0.03

Maharashtra										**ORF1a:Y2560I**	1	0.03
Maharashtra										**ORF1a:S2562H**	1	0.03
Maharashtra										**ORF1a:P2568T**	1	0.03
Maharashtra										**ORF1a:L2570T**	1	0.03
Maharashtra										**ORF1a:L2572I**	1	0.03
Maharashtra										**ORF1a:S2599Q**	1	0.03
Maharashtra										**ORF1a:S2600***	1	0.03
Maharashtra										**ORF1a:N2603S**	1	0.03
Maharashtra										**ORF1a:E2653A**	1	0.03
Maharashtra										**ORF1a:A2690D**	1	0.03
Maharashtra										**ORF1a:C2691H**	1	0.03
Maharashtra										**ORF1a:I2692V**	1	0.03
Maharashtra										**ORF1a:N3335T**	1	0.03
Maharashtra										**ORF1a:H3125Y**	1	0.03
Maharashtra										**ORF1a:V3708S**	1	0.03
Maharashtra										**ORF1a:W3709P**	1	0.03
Maharashtra										**ORF1a:L3711A**	1	0.03
Maharashtra										**ORF1a:M3712I**	1	0.03
Maharashtra										**ORF1a:V464F**	1	0.03
Maharashtra										**ORF1a:C2826Y**	1	0.03
Maharashtra										**ORF1a:N4082S**	1	0.03
Maharashtra										**ORF1a:N2038K**	1	0.03
Maharashtra										**ORF1a:A2139V**	1	0.03
Maharashtra										**ORF1a:V2051L**	1	0.03
Maharashtra										**ORF1a:K798T**	1	0.03
Maharashtra										**ORF1a:A773I**	1	0.03
Maharashtra										**ORF1a:V774K**	1	0.03
Maharashtra										**ORF1a:E775D**	1	0.03
Maharashtra										**ORF1a:Y1354L**	1	0.03
Maharashtra										**ORF1a:G3971S**	1	0.03
Maharashtra										**ORF1a:S674N**	1	0.03
Maharashtra										**ORF1a:S2695I**	1	0.03
Maharashtra										**ORF1a:A4183T**	1	0.03
Maharashtra										**ORF1a:T2777I**	1	0.03
Maharashtra										**ORF1a:D2592N**	1	0.03
Maharashtra										**ORF1a:F924L**	1	0.03
Maharashtra										**ORF1a:A3456S**	1	0.03
Maharashtra										**ORF1a:C3798R**	1	0.03
Maharashtra										**ORF1a:A3730F**	1	0.03
Maharashtra										**ORF1a:V3751H**	1	0.03
Maharashtra										**ORF1a:M3752V**	1	0.03
Maharashtra										**ORF1a:Q3922N**	1	0.03
Maharashtra										**ORF1a:D3972***	1	0.03
Maharashtra										**ORF1a:Q4015D**	1	0.03
Maharashtra										**ORF1a:A4016G**	1	0.03
Maharashtra										**ORF1a:T4083G**	1	0.03
Maharashtra										**ORF1a:D4085V**	1	0.03
Maharashtra										**ORF1a:A4247Q**	1	0.03
Maharashtra										**ORF1a:A4248P**	1	0.03
Maharashtra										**ORF1a:T4249K**	1	0.03
Maharashtra										**ORF1a:V4250***	1	0.03
Maharashtra										**ORF1a:E289A**	1	0.03
Maharashtra										**ORF1a:T2846A**	1	0.03
Maharashtra										**ORF1a:C1833F**	1	0.03
Maharashtra										**ORF1a:M315K**	1	0.03
Maharashtra										**ORF1a:A486H**	1	0.03
Maharashtra										**ORF1a:A1074T**	1	0.03
Maharashtra										**ORF1a:S1150T**	1	0.03
Maharashtra										**ORF1a:G1152Y**	1	0.03
Maharashtra										**ORF1a:R1163***	1	0.03
Maharashtra										**ORF1a:V1164E**	1	0.03
Maharashtra										**ORF1a:C1165F**	1	0.03
Maharashtra										**ORF1a:D1167S**	1	0.03
Maharashtra										**ORF1a:V1291H**	1	0.03
Maharashtra										**ORF1a:Q1292I**	1	0.03
Maharashtra										**ORF1a:E1293***	1	0.03
Maharashtra										**ORF1a:G1294W**	1	0.03
Maharashtra										**ORF1a:V1320***	1	0.03
Maharashtra										**ORF1a:Y1337L**	1	0.03
Maharashtra										**ORF1a:V1339R**	1	0.03
Maharashtra										**ORF1a:E1340V**	1	0.03
Maharashtra										**ORF1a:T1430P**	1	0.03
Maharashtra										**ORF1a:A1432K**	1	0.03

Maharashtra										**ORF1a:S1433Q**	1	0.03
Maharashtra										**ORF1a:N1436R**	1	0.03
Maharashtra										**ORF1a:T1437H**	1	0.03
Maharashtra										**ORF1a:E1443S**	1	0.03
Maharashtra										**ORF1a:L1445H**	1	0.03
Maharashtra										**ORF1a:V1446L**	1	0.03
Maharashtra										**ORF1a:V1475I**	1	0.03
Maharashtra										**ORF1a:V1477D**	1	0.03
Maharashtra										**ORF1a:P1480K**	1	0.03
Maharashtra										**ORF1a:V1483L**	1	0.03
Maharashtra										**ORF1a:T1484Q**	1	0.03
Maharashtra										**ORF1a:Y1486L**	1	0.03
Maharashtra										**ORF1a:N1487F**	1	0.03
Maharashtra										**ORF1a:G1488L**	1	0.03
Maharashtra										**ORF1a:T1504N**	1	0.03
Maharashtra										**ORF1a:N1555T**	1	0.03
Maharashtra										**ORF1a:L1556***	1	0.03
Maharashtra										**ORF1a:K1557M**	1	0.03
Maharashtra										**ORF1a:T1558V**	1	0.03
Maharashtra										**ORF1a:L1559K**	1	0.03
Maharashtra										**ORF1a:A1707Q**	1	0.03
Maharashtra										**ORF1a:A1708G**	1	0.03
Maharashtra										**ORF1a:N1709L**	1	0.03
Maharashtra										**ORF1a:F1710V**	1	0.03
Maharashtra										**ORF1a:C1711K**	1	0.03
Maharashtra										**ORF1a:E1730Q**	1	0.03
Maharashtra										**ORF1a:T1731***	1	0.03
Maharashtra										**ORF1a:M1732V**	1	0.03
Maharashtra										**ORF1a:Y1734I**	1	0.03
Maharashtra										**ORF1a:F1736A**	1	0.03
Maharashtra										**ORF1a:Q1737K**	1	0.03
Maharashtra										**ORF1a:H1738E**	1	0.03
Maharashtra										**ORF1a:A1739S**	1	0.03
Maharashtra										**ORF1a:N1740***	1	0.03
Maharashtra										**ORF1a:L1741T**	1	0.03
Maharashtra										**ORF1a:D1742W**	1	0.03
Maharashtra										**ORF1a:S1743C**	1	0.03
Maharashtra										**ORF1a:C1744V**	1	0.03
Maharashtra										**ORF1a:G1790R**	1	0.03
Maharashtra										**ORF1a:A1793N**	1	0.03
Maharashtra										**ORF1a:T1794I**	1	0.03
Maharashtra										**ORF1a:K1795***	1	0.03
Maharashtra										**ORF1a:V1798***	1	0.03
Maharashtra										**ORF1a:A3645F**	1	0.03
Maharashtra										**ORF1a:T3646V**	1	0.03
Maharashtra										**ORF1a:V3647F**	1	0.03
Maharashtra										**ORF1a:A3648V**	1	0.03
Maharashtra										**ORF1a:Y3649T**	1	0.03
Maharashtra										**ORF1a:N3651G**	1	0.03
Maharashtra										**ORF1a:M3652D**	1	0.03
Maharashtra										**ORF1a:V3653A**	1	0.03
Maharashtra										**ORF1a:L3667Y**	1	0.03
Maharashtra										**ORF1a:M3733W**	1	0.03
Maharashtra										**ORF1a:W3734***	1	0.03
Maharashtra										**ORF1a:A3735C**	1	0.03
Maharashtra										**ORF1a:L3736F**	1	0.03
Maharashtra										**ORF1a:I3738S**	1	0.03
Maharashtra										**ORF1a:T3741G**	1	0.03
Maharashtra										**ORF1a:N3743S**	1	0.03
Maharashtra										**ORF1a:S3745N**	1	0.03
Maharashtra										**ORF1a:V3747H**	1	0.03
Maharashtra										**ORF1a:M3761F**	1	0.03
Maharashtra										**ORF1a:R3805C**	1	0.03
Maharashtra										**ORF1a:T3807***	1	0.03
Maharashtra										**ORF1a:G3809L**	1	0.03
Maharashtra										**ORF1a:V3810S**	1	0.03
Maharashtra										**ORF1a:D3812Y**	1	0.03
Maharashtra										**ORF1a:Y3813T**	1	0.03
Maharashtra										**ORF1a:L3814G**	1	0.03
Maharashtra										**ORF1a:S3816***	1	0.03
Maharashtra										**ORF1a:T3817I**	1	0.03
Maharashtra										**ORF1a:Q3818Y**	1	0.03
Maharashtra										**ORF1a:I3853W**	1	0.03

Maharashtra										**ORF1a:K3854W**	1	0.03
Maharashtra										**ORF1a:V3855Q**	1	0.03
Maharashtra										**ORF1a:A3856T**	1	0.03
Maharashtra										**ORF1a:T3857L**	1	0.03
Maharashtra										**ORF1a:V3858Y**	1	0.03
Maharashtra										**ORF1a:V3865S**	1	0.03
Maharashtra										**ORF1a:D3903N**	1	0.03
Maharashtra										**ORF1a:T3904G**	1	0.03
Maharashtra										**ORF1a:T3905F**	1	0.03
Maharashtra										**ORF1a:E3906T**	1	0.03
Maharashtra										**ORF1a:D3936L**	1	0.03
Maharashtra										**ORF1a:N3937***	1	0.03
Maharashtra										**ORF1a:A3939N**	1	0.03
Maharashtra										**ORF1a:T3940A**	1	0.03
Maharashtra										**ORF1a:A3943Q**	1	0.03
Maharashtra										**ORF1a:I3944G**	1	0.03
Maharashtra										**ORF1a:A3945N**	1	0.03
Maharashtra										**ORF1a:E3947T**	1	0.03
Maharashtra										**ORF1a:F3948S**	1	0.03
Maharashtra										**ORF1a:P3952C**	1	0.03
Maharashtra										**ORF1a:Y3954R**	1	0.03
Maharashtra										**ORF1a:A3955S**	1	0.03
Maharashtra										**ORF1a:A3956L**	1	0.03
Maharashtra										**ORF1a:F3957***	1	0.03
Maharashtra										**ORF1a:N4060C**	1	0.03
Maharashtra										**ORF1a:I4061S**	1	0.03
Maharashtra										**ORF1a:P4063E**	1	0.03
Maharashtra										**ORF1a:L4064H**	1	0.03
Maharashtra										**ORF1a:K2120R**	1	0.03
Maharashtra										**ORF1a:T2121N**	1	0.03
Maharashtra										**ORF1a:A2123V**	1	0.03
Maharashtra										**ORF1a:T2124S**	1	0.03
Maharashtra										**ORF1a:H2125L**	1	0.03
Maharashtra										**ORF1a:Q2373W**	1	0.03
Maharashtra										**ORF1a:A2375E**	1	0.03
Maharashtra										**ORF1a:I2377T**	1	0.03
Maharashtra										**ORF1a:N2623Y**	1	0.03
Maharashtra										**ORF1a:V2624L**	1	0.03
Maharashtra										**ORF1a:S2625L**	1	0.03
Maharashtra										**ORF1a:D2627F**	1	0.03
Maharashtra										**ORF1a:N2628Q**	1	0.03
Maharashtra										**ORF1a:P2685V**	1	0.03
Maharashtra										**ORF1a:G2871K**	1	0.03
Maharashtra										**ORF1a:T2872W**	1	0.03
Maharashtra										**ORF1a:I2873V**	1	0.03
Maharashtra										**ORF1a:T3543***	1	0.03
Maharashtra										**ORF1a:V3708L**	1	0.03
Maharashtra										**ORF1a:W3709***	1	0.03
Maharashtra										**ORF1a:T3710S**	1	0.03
Maharashtra										**ORF1a:M3761Y**	1	0.03
Maharashtra										**ORF1a:C3762V**	1	0.03
Maharashtra										**ORF1a:V3763F**	1	0.03
Maharashtra										**ORF1a:L1774F**	1	0.03
Maharashtra										**ORF1a:S1775L**	1	0.03
Maharashtra										**ORF1a:Y1776M**	1	0.03
Maharashtra										**ORF1a:E1777N**	1	0.03
Maharashtra										**ORF1a:Q1778N**	1	0.03
Maharashtra										**ORF1a:F1779L**	1	0.03
Maharashtra										**ORF1a:K1780R**	1	0.03
Maharashtra										**ORF1a:Y4227H**	1	0.03
Maharashtra										**ORF1a:Y4229K**	1	0.03
Maharashtra										**ORF1a:F4230V**	1	0.03
Maharashtra										**ORF1a:G4233***	1	0.03
Maharashtra										**ORF1a:L4234S**	1	0.03
Maharashtra										**ORF1a:N4235I**	1	0.03
Maharashtra										**ORF1a:N4236I**	1	0.03
Maharashtra										**ORF1a:N4238R**	1	0.03
Maharashtra										**ORF1a:R4239Y**	1	0.03
Maharashtra										**ORF1a:L4252S**	1	0.03
Maharashtra										**ORF1a:V4274I**	1	0.03
Maharashtra										**ORF1a:P2480S**	1	0.03
Maharashtra										**ORF1a:T602A**	1	0.03
Maharashtra										**ORF1a:K1981R**	1	0.03

Maharashtra										**ORF1a:P1147S**	1	0.03
Maharashtra										**ORF1a:N997H**	1	0.03
Maharashtra										**ORF1a:D4085A**	1	0.03
Maharashtra										**ORF1a:I3587T**	1	0.03
Maharashtra										**ORF1a:A4247V**	1	0.03
Maharashtra										**ORF1a:T1310L**	1	0.03
Maharashtra										**ORF1a:E1311V**	1	0.03
Maharashtra										**ORF1a:V1320P**	1	0.03
Maharashtra										**ORF1a:P1321L**	1	0.03
Maharashtra										**ORF1a:G1456I**	1	0.03
Maharashtra										**ORF1a:L1457***	1	0.03
Maharashtra										**ORF1a:N1458D**	1	0.03
Maharashtra										**ORF1a:P2018V**	1	0.03
Maharashtra										**ORF1a:V2019***	1	0.03
Maharashtra										**ORF1a:E2020C**	1	0.03
Maharashtra										**ORF1a:D2043R**	1	0.03
Maharashtra										**ORF1a:L2044S**	1	0.03
Maharashtra										**ORF1a:P2046T**	1	0.03
Maharashtra										**ORF1a:A2593V**	1	0.03
Maharashtra										**ORF1a:P3134H**	1	0.03
Maharashtra										**ORF1a:L3180***	1	0.03
Maharashtra										**ORF1a:C3181W**	1	0.03
Maharashtra										**ORF1a:T3182C**	1	0.03
Maharashtra										**ORF1a:L3185R**	1	0.03
Maharashtra										**ORF1a:R3194V**	1	0.03
Maharashtra										**ORF1a:P3252***	1	0.03
Maharashtra										**ORF1a:P3253L**	1	0.03
Maharashtra										**ORF1a:T3255N**	1	0.03
Maharashtra										**ORF1a:M2194I**	1	0.03
Maharashtra										**ORF1a:S3570G**	1	0.03
Maharashtra										**ORF1a:P1879L**	1	0.03
Maharashtra										**ORF1a:F2173V**	1	0.03
Maharashtra										**ORF1a:Y359A**	1	0.03
Maharashtra										**ORF1a:P361V**	1	0.03
Maharashtra										**ORF1a:G408K**	1	0.03
Maharashtra										**ORF1a:C409P**	1	0.03
Maharashtra										**ORF1a:V410F**	1	0.03
Maharashtra										**ORF1a:S412V**	1	0.03
Maharashtra										**ORF1a:Y413R**	1	0.03
Maharashtra										**ORF1a:C416F**	1	0.03
Maharashtra										**ORF1a:G432V**	1	0.03
Maharashtra										**ORF1a:K1197R**	1	0.03
Maharashtra										**ORF1a:Q1198F**	1	0.03
Maharashtra										**ORF1a:V1199L**	1	0.03
Maharashtra										**ORF1a:E1200K**	1	0.03
Maharashtra										**ORF1a:Q1201R**	1	0.03
Maharashtra										**ORF1a:D1273F**	1	0.03
Maharashtra										**ORF1a:D1275Q**	1	0.03
Maharashtra										**ORF1a:N1898I**	1	0.03
Maharashtra										**ORF1a:D1939I**	1	0.03
Maharashtra										**ORF1a:D1940I**	1	0.03
Maharashtra										**ORF1a:D2299V**	1	0.03
Maharashtra										**ORF1a:T2300V**	1	0.03
Maharashtra										**ORF1a:Y2301F**	1	0.03
Maharashtra										**ORF1a:P2302V**	1	0.03
Maharashtra										**ORF1a:S2303L**	1	0.03
Maharashtra										**ORF1a:L2304V**	1	0.03
Maharashtra										**ORF1a:E2305V**	1	0.03
Maharashtra										**ORF1a:T2306***	1	0.03
Maharashtra										**ORF1a:F2337C**	1	0.03
Maharashtra										**ORF1a:F2338R**	1	0.03
Maharashtra										**ORF1a:Y2339V**	1	0.03
Maharashtra										**ORF1a:L2609G**	1	0.03
Maharashtra										**ORF1a:K2622Q**	1	0.03
Maharashtra										**ORF1a:V2624T**	1	0.03
Maharashtra										**ORF1a:L2626C**	1	0.03
Maharashtra										**ORF1a:D2627N**	1	0.03
Maharashtra										**ORF1a:V2629R**	1	0.03
Maharashtra										**ORF1a:L2630S**	1	0.03
Maharashtra										**ORF1a:S2631***	1	0.03
Maharashtra										**ORF1a:I2729V**	1	0.03
Maharashtra										**ORF1a:R2730***	1	0.03
Maharashtra										**ORF1a:S2731T**	1	0.03

Maharashtra										**ORF1a:K2735T**	1	0.03
Maharashtra										**ORF1a:L2738Y**	1	0.03
Maharashtra										**ORF1a:P2739***	1	0.03
Maharashtra										**ORF1a:F2740T**	1	0.03
Maharashtra										**ORF1a:K2741S**	1	0.03
Maharashtra										**ORF1a:L2742C**	1	0.03
Maharashtra										**ORF1a:T2743***	1	0.03
Maharashtra										**ORF1a:V2766C**	1	0.03
Maharashtra										**ORF1a:V2973C**	1	0.03
Maharashtra										**ORF1a:R2974***	1	0.03
Maharashtra										**ORF1a:V2975S**	1	0.03
Maharashtra										**ORF1a:V2976G**	1	0.03
Maharashtra										**ORF1a:T2977N**	1	0.03
Maharashtra										**ORF1a:T2978N**	1	0.03
Maharashtra										**ORF1a:D2980Y**	1	0.03
Maharashtra										**ORF1a:S3658L**	1	0.03
Maharashtra										**ORF1a:W3659***	1	0.03
Maharashtra										**ORF1a:V3660L**	1	0.03
Maharashtra										**ORF1a:R3662L**	1	0.03
Maharashtra										**ORF1a:W3734V**	1	0.03
Maharashtra										**ORF1a:A3735M**	1	0.03
Maharashtra										**ORF1a:I3737***	1	0.03
Maharashtra										**ORF1a:M3752C**	1	0.03
Maharashtra										**ORF1a:A4068C**	1	0.03
Maharashtra										**ORF1a:K4069S**	1	0.03
Maharashtra										**ORF1a:A4156W**	1	0.03
Maharashtra										**ORF1a:G4157H**	1	0.03
Maharashtra										**ORF1a:T4174H**	1	0.03
Maharashtra										**ORF1a:T4175N**	1	0.03
Maharashtra										**ORF1a:C4270L**	1	0.03
Maharashtra										**ORF1a:A4271C**	1	0.03
Maharashtra										**ORF1a:A4273C**	1	0.03
Maharashtra										**ORF1a:V4274C**	1	0.03
Maharashtra										**ORF1a:D4275R**	1	0.03
Maharashtra										**ORF1a:A4276C**	1	0.03
Maharashtra										**ORF1a:A4277C**	1	0.03
Maharashtra										**ORF1a:K4278***	1	0.03
Maharashtra										**ORF1a:V4372I**	1	0.03
Maharashtra										**ORF1a:E1209K**	1	0.03
Maharashtra										**ORF1a:C2694G**	1	0.03
Maharashtra										**ORF1a:S2706K**	1	0.03
Maharashtra										**ORF1a:H2707S**	1	0.03
Maharashtra										**ORF1a:N2708Q**	1	0.03
Maharashtra										**ORF1a:I2709H**	1	0.03
Maharashtra										**ORF1a:A2710C**	1	0.03
Maharashtra										**ORF1a:L2711F**	1	0.03
Maharashtra										**ORF1a:I2712D**	1	0.03
Maharashtra										**ORF1a:W2713M**	1	0.03
Maharashtra										**ORF1a:P2792S**	1	0.03
Maharashtra										**ORF1a:H3581P**	1	0.03
Maharashtra										**ORF1a:W3582L**	1	0.03
Maharashtra										**ORF1a:L3583V**	1	0.03
Maharashtra										**ORF1a:G2808***	1	0.03
Maharashtra										**ORF1a:Y2809V**	1	0.03
Maharashtra										**ORF1a:A2811S**	1	0.03
Maharashtra										**ORF1a:I2812***	1	0.03
Maharashtra										**ORF1a:G2815L**	1	0.03
Maharashtra										**ORF1a:K2830***	1	0.03
Maharashtra										**ORF1a:C2851H**	1	0.03
Maharashtra										**ORF1a:I2854L**	1	0.03
Maharashtra										**ORF1a:A2855Q**	1	0.03
Maharashtra										**ORF1a:A2856S**	1	0.03
Maharashtra										**ORF1a:V2857***	1	0.03
Maharashtra										**ORF1a:I2858Q**	1	0.03
Maharashtra										**ORF1a:T2859E**	1	0.03
Maharashtra										**ORF1a:E2861W**	1	0.03
Maharashtra										**ORF1a:K3632S**	1	0.03
Maharashtra										**ORF1a:H3633V**	1	0.03
Maharashtra										**ORF1a:A3634C**	1	0.03
Maharashtra										**ORF1a:L3636C**	1	0.03
Maharashtra										**ORF1a:C3637Y**	1	0.03
Maharashtra										**ORF1a:L3641P**	1	0.03
Maharashtra										**ORF1a:P3642C**	1	0.03

Maharashtra										**ORF1a:G146F**	1	0.03
Maharashtra										**ORF1a:D147K**	1	0.03
Maharashtra										**ORF1a:E148K**	1	0.03
Maharashtra										**ORF1a:L149T**	1	0.03
Maharashtra										**ORF1a:G179S**	1	0.03
Maharashtra										**ORF1a:G180V**	1	0.03
Maharashtra										**ORF1a:A211T**	1	0.03
Maharashtra										**ORF1a:C213S**	1	0.03
Maharashtra										**ORF1a:Q252V**	1	0.03
Maharashtra										**ORF1a:T253H**	1	0.03
Maharashtra										**ORF1a:P254G**	1	0.03
Maharashtra										**ORF1a:F255T**	1	0.03
Maharashtra										**ORF1a:E256F**	1	0.03
Maharashtra										**ORF1a:I257***	1	0.03
Maharashtra										**ORF1a:L259E**	1	0.03
Maharashtra										**ORF1a:K261***	1	0.03
Maharashtra										**ORF1a:K262I**	1	0.03
Maharashtra										**ORF1a:F263A**	1	0.03
Maharashtra										**ORF1a:D294Y**	1	0.03
Maharashtra										**ORF1a:C312***	1	0.03
Maharashtra										**ORF1a:Q314S**	1	0.03
Maharashtra										**ORF1a:T319N**	1	0.03
Maharashtra										**ORF1a:L320F**	1	0.03
Maharashtra										**ORF1a:T340D**	1	0.03
Maharashtra										**ORF1a:C341G**	1	0.03
Maharashtra										**ORF1a:E342R**	1	0.03
Maharashtra										**ORF1a:A498C**	1	0.03
Maharashtra										**ORF1a:K500C**	1	0.03
Maharashtra										**ORF1a:Q501G**	1	0.03
Maharashtra										**ORF1a:I502N**	1	0.03
Maharashtra										**ORF1a:V503C**	1	0.03
Maharashtra										**ORF1a:S505R**	1	0.03
Maharashtra										**ORF1a:C506F**	1	0.03
Maharashtra										**ORF1a:M584W**	1	0.03
Maharashtra										**ORF1a:M585N**	1	0.03
Maharashtra										**ORF1a:S588V**	1	0.03
Maharashtra										**ORF1a:D589F**	1	0.03
Maharashtra										**ORF1a:L590T**	1	0.03
Maharashtra										**ORF1a:A591E**	1	0.03
Maharashtra										**ORF1a:N594G**	1	0.03
Maharashtra										**ORF1a:V597D**	1	0.03
Maharashtra										**ORF1a:M598F**	1	0.03
Maharashtra										**ORF1a:V660***	1	0.03
Maharashtra										**ORF1a:L688E**	1	0.03
Maharashtra										**ORF1a:A690S**	1	0.03
Maharashtra										**ORF1a:V710***	1	0.03
Maharashtra										**ORF1a:H712***	1	0.03
Maharashtra										**ORF1a:P765C**	1	0.03
Maharashtra										**ORF1a:Q768W**	1	0.03
Maharashtra										**ORF1a:P769Y**	1	0.03
Maharashtra										**ORF1a:E772L**	1	0.03
Maharashtra										**ORF1a:A773Y**	1	0.03
Maharashtra										**ORF1a:V774***	1	0.03
Maharashtra										**ORF1a:E775R**	1	0.03
Maharashtra										**ORF1a:A803H**	1	0.03
Maharashtra										**ORF1a:P804T**	1	0.03
Maharashtra										**ORF1a:N805Q**	1	0.03
Maharashtra										**ORF1a:M806R**	1	0.03
Maharashtra										**ORF1a:M807R**	1	0.03
Maharashtra										**ORF1a:V808C**	1	0.03
Maharashtra										**ORF1a:Q834K**	1	0.03
Maharashtra										**ORF1a:N869K**	1	0.03
Maharashtra										**ORF1a:E870N**	1	0.03
Maharashtra										**ORF1a:L893I**	1	0.03
Maharashtra										**ORF1a:G894***	1	0.03
Maharashtra										**ORF1a:Y906F**	1	0.03
Maharashtra										**ORF1a:L907R**	1	0.03
Maharashtra										**ORF1a:F908***	1	0.03
Maharashtra										**ORF1a:E942I**	1	0.03
Maharashtra										**ORF1a:P943N**	1	0.03
Maharashtra										**ORF1a:Q990R**	1	0.03
Maharashtra										**ORF1a:D992***	1	0.03
Maharashtra										**ORF1a:S994Q**	1	0.03

Maharashtra										**ORF1a:E995S**	1	0.03
Maharashtra										**ORF1a:C1103W**	1	0.03
Maharashtra										**ORF1a:G1107A**	1	0.03
Maharashtra										**ORF1a:H1108Q**	1	0.03
Maharashtra										**ORF1a:A1111T**	1	0.03
Maharashtra										**ORF1a:Y1135V**	1	0.03
Maharashtra										**ORF1a:E1223R**	1	0.03
Maharashtra										**ORF1a:Q1224K**	1	0.03
Maharashtra										**ORF1a:C1718N**	1	0.03
Maharashtra										**ORF1a:F1821S**	1	0.03
Maharashtra										**ORF1a:C1823T**	1	0.03
Maharashtra										**ORF1a:A1824C**	1	0.03
Maharashtra										**ORF1a:E1826V**	1	0.03
Maharashtra										**ORF1a:N1830I**	1	0.03
Maharashtra										**ORF1a:Q1832L**	1	0.03
Maharashtra										**ORF1a:G1834L**	1	0.03
Maharashtra										**ORF1a:Y1836R**	1	0.03
Maharashtra										**ORF1a:K1837R**	1	0.03
Maharashtra										**ORF1a:H1838C**	1	0.03
Maharashtra										**ORF1a:I1839F**	1	0.03
Maharashtra										**ORF1a:S1841Y**	1	0.03
Maharashtra										**ORF1a:Y1882S**	1	0.03
Maharashtra										**ORF1a:K1883Y**	1	0.03
Maharashtra										**ORF1a:K1980G**	1	0.03
Maharashtra										**ORF1a:K1981M**	1	0.03
Maharashtra										**ORF1a:G1982L**	1	0.03
Maharashtra										**ORF1a:A1983T**	1	0.03
Maharashtra										**ORF1a:K1984M**	1	0.03
Maharashtra										**ORF1a:L1985Q**	1	0.03
Maharashtra										**ORF1a:H1987I**	1	0.03
Maharashtra										**ORF1a:G2035L**	1	0.03
Maharashtra										**ORF1a:D2037***	1	0.03
Maharashtra										**ORF1a:K2085***	1	0.03
Maharashtra										**ORF1a:I2086E**	1	0.03
Maharashtra										**ORF1a:S2237***	1	0.03
Maharashtra										**ORF1a:S2312Q**	1	0.03
Maharashtra										**ORF1a:L2333G**	1	0.03
Maharashtra										**ORF1a:N2523C**	1	0.03
Maharashtra										**ORF1a:D2525L**	1	0.03
Maharashtra										**ORF1a:N2526M**	1	0.03
Maharashtra										**ORF1a:I1360M**	1	0.03
Maharashtra										**ORF1a:N1362S**	1	0.03
Maharashtra										**ORF1a:E1363K**	1	0.03
Maharashtra										**ORF1a:Q1365F**	1	0.03
Maharashtra										**ORF1a:E1366L**	1	0.03
Maharashtra										**ORF1a:I1367E**	1	0.03
Maharashtra										**ORF1a:F1501R**	1	0.03
Maharashtra										**ORF1a:E1503M**	1	0.03
Maharashtra										**ORF1a:T1504V**	1	0.03
Maharashtra										**ORF1a:Y1646L**	1	0.03
Maharashtra										**ORF1a:M1647L**	1	0.03
Maharashtra										**ORF1a:A1649S**	1	0.03
Maharashtra										**ORF1a:A1677M**	1	0.03
Maharashtra										**ORF1a:T1678V**	1	0.03
Maharashtra										**ORF1a:A1679***	1	0.03
Maharashtra										**ORF1a:T1682L**	1	0.03
Maharashtra										**ORF1a:L1683N**	1	0.03
Maharashtra										**ORF1a:Q1684G**	1	0.03
Maharashtra										**ORF1a:E1687T**	1	0.03
Maharashtra										**ORF1a:L1688T**	1	0.03
Maharashtra										**ORF1a:K1689V**	1	0.03
Maharashtra										**ORF1a:F1690I**	1	0.03
Maharashtra										**ORF1a:N1691L**	1	0.03
Maharashtra										**ORF1a:P1693Q**	1	0.03
Maharashtra										**ORF1a:A1694G**	1	0.03
Maharashtra										**ORF1a:G1723K**	1	0.03
Maharashtra										**ORF1a:E1724L**	1	0.03
Maharashtra										**ORF1a:G1726T**	1	0.03
Maharashtra										**ORF1a:D1727F**	1	0.03
Maharashtra										**ORF1a:R1729***	1	0.03
Maharashtra										**ORF1a:E1730V**	1	0.03
Maharashtra										**ORF1a:F1930I**	1	0.03
Maharashtra										**ORF1a:D3511S**	1	0.03

Maharashtra										**ORF1a:T3716F**	1	0.03
Maharashtra										**ORF1a:L3717H**	1	0.03
Maharashtra										**ORF1a:V3747Y**	1	0.03
Maharashtra										**ORF1a:T3749C**	1	0.03
Maharashtra										**ORF1a:T3750***	1	0.03
Maharashtra										**ORF1a:C3766L**	1	0.03
Maharashtra										**ORF1a:C3798L**	1	0.03
Maharashtra										**ORF1a:L3799R**	1	0.03
Maharashtra										**ORF1a:N3801F**	1	0.03
Maharashtra										**ORF1a:R3802L**	1	0.03
Maharashtra										**ORF1a:F3804L**	1	0.03
Maharashtra										**ORF1a:T3807Y**	1	0.03
Maharashtra										**ORF1a:L3808T**	1	0.03
Maharashtra										**ORF1a:L3814T**	1	0.03
Maharashtra										**ORF1a:V3815T**	1	0.03
Maharashtra										**ORF1a:S3816P**	1	0.03
Maharashtra										**ORF1a:G3846N**	1	0.03
Maharashtra										**ORF1a:G3848R**	1	0.03
Maharashtra										**ORF1a:G3849C**	1	0.03
Maharashtra										**ORF1a:P3851V**	1	0.03
Maharashtra										**ORF1a:C3852H**	1	0.03
Maharashtra										**ORF1a:V3870S**	1	0.03
Maharashtra										**ORF1a:V3871L**	1	0.03
Maharashtra										**ORF1a:L3872T**	1	0.03
Maharashtra										**ORF1a:W3888F**	1	0.03
Maharashtra										**ORF1a:C3891***	1	0.03
Maharashtra										**ORF1a:V3892H**	1	0.03
Maharashtra										**ORF1a:Q3893S**	1	0.03
Maharashtra										**ORF1a:L3914A**	1	0.03
Maharashtra										**ORF1a:S3916***	1	0.03
Maharashtra										**ORF1a:V3917R**	1	0.03
Maharashtra										**ORF1a:L3918N**	1	0.03
Maharashtra										**ORF1a:L3919A**	1	0.03
Maharashtra										**ORF1a:S3920G**	1	0.03
Maharashtra										**ORF1a:M3921Q**	1	0.03
Maharashtra										**ORF1a:A3955W**	1	0.03
Maharashtra										**ORF1a:A3956***	1	0.03
Maharashtra										**ORF1a:L3980***	1	0.03
Maharashtra										**ORF1a:K3981S**	1	0.03
Maharashtra										**ORF1a:Q4015Y**	1	0.03
Maharashtra										**ORF1a:D4043L**	1	0.03
Maharashtra										**ORF1a:A4044E**	1	0.03
Maharashtra										**ORF1a:L4045H**	1	0.03
Maharashtra										**ORF1a:N4047T**	1	0.03
Maharashtra										**ORF1a:I4048S**	1	0.03
Maharashtra										**ORF1a:I4049Y**	1	0.03
Maharashtra										**ORF1a:D4103L**	1	0.03
Maharashtra										**ORF1a:A4104C**	1	0.03
Maharashtra										**ORF1a:D4105I**	1	0.03
Maharashtra										**ORF1a:K4107Q**	1	0.03
Maharashtra										**ORF1a:I4108F**	1	0.03
Maharashtra										**ORF1a:V4109T**	1	0.03
Maharashtra										**ORF1a:Q4110***	1	0.03
Maharashtra										**ORF1a:E4113M**	1	0.03
Maharashtra										**ORF1a:I4114A**	1	0.03
Maharashtra										**ORF1a:M4116Y**	1	0.03
Maharashtra										**ORF1a:D4117C**	1	0.03
Maharashtra										**ORF1a:P4120F**	1	0.03
Maharashtra										**ORF1a:L4122G**	1	0.03
Maharashtra										**ORF1a:A4123Q**	1	0.03
Maharashtra										**ORF1a:W4124F**	1	0.03
Maharashtra										**ORF1a:P4125C**	1	0.03
Maharashtra										**ORF1a:L4126C**	1	0.03
Maharashtra										**ORF1a:I4127Q**	1	0.03
Maharashtra										**ORF1a:V4128I**	1	0.03
Maharashtra										**ORF1a:A4130E**	1	0.03
Maharashtra										**ORF1a:L4131***	1	0.03
Maharashtra										**ORF1a:R4132***	1	0.03
Maharashtra										**ORF1a:N4134***	1	0.03
Maharashtra										**ORF1a:A2438T**	1	0.03
Maharashtra										**ORF1a:N2439I**	1	0.03
Maharashtra										**ORF1a:Q2702L**	1	0.03
Maharashtra										**ORF1a:D2951M**	1	0.03

Maharashtra										**ORF1a:T2952Y**	1	0.03
Maharashtra										**ORF1a:R2953***	1	0.03
Maharashtra										**ORF1a:Y2954K**	1	0.03
Maharashtra										**ORF1a:I3055L**	1	0.03
Maharashtra										**ORF1a:A3061V**	1	0.03
Maharashtra										**ORF1a:Y3062N**	1	0.03
Maharashtra										**ORF1a:Y3063T**	1	0.03
Maharashtra										**ORF1a:F3064V**	1	0.03
Maharashtra										**ORF1a:V3091***	1	0.03
Maharashtra										**ORF1a:C3093P**	1	0.03
Maharashtra										**ORF1a:T3095I**	1	0.03
Maharashtra										**ORF1a:P3096L**	1	0.03
Maharashtra										**ORF1a:V3097Y**	1	0.03
Maharashtra										**ORF1a:F3100L**	1	0.03
Maharashtra										**ORF1a:L3101C**	1	0.03
Maharashtra										**ORF1a:P3102H**	1	0.03
Maharashtra										**ORF1a:G3103S**	1	0.03
Maharashtra										**ORF1a:V3104L**	1	0.03
Maharashtra										**ORF1a:T3133L**	1	0.03
Maharashtra										**ORF1a:P3134F**	1	0.03
Maharashtra										**ORF1a:L3135***	1	0.03
Maharashtra										**ORF1a:V3136H**	1	0.03
Maharashtra										**ORF1a:P3137I**	1	0.03
Maharashtra										**ORF1a:W3139S**	1	0.03
Maharashtra										**ORF1a:T3141G**	1	0.03
Maharashtra										**ORF1a:I3142S**	1	0.03
Maharashtra										**ORF1a:Y3144V**	1	0.03
Maharashtra										**ORF1a:F3266H**	1	0.03
Maharashtra										**ORF1a:R3267H**	1	0.03
Maharashtra										**ORF1a:M3269P**	1	0.03
Maharashtra										**ORF1a:F3271S**	1	0.03
Maharashtra										**ORF1a:C3307M**	1	0.03
Maharashtra										**ORF1a:N3335F**	1	0.03
Maharashtra										**ORF1a:Q3337S**	1	0.03
Maharashtra										**ORF1a:R3368L**	1	0.03
Maharashtra										**ORF1a:G3372F**	1	0.03
Maharashtra										**ORF1a:T3374C**	1	0.03
Maharashtra										**ORF1a:F3375***	1	0.03
Maharashtra										**ORF1a:S3376L**	1	0.03
Maharashtra										**ORF1a:A3379V**	1	0.03
Maharashtra										**ORF1a:C3380F**	1	0.03
Maharashtra										**ORF1a:Y3381T**	1	0.03
Maharashtra										**ORF1a:A3436F**	1	0.03
Maharashtra										**ORF1a:G3437V**	1	0.03
Maharashtra										**ORF1a:D3439C**	1	0.03
Maharashtra										**ORF1a:L3440T**	1	0.03
Maharashtra										**ORF1a:E3441I**	1	0.03
Maharashtra										**ORF1a:G3442W**	1	0.03
Maharashtra										**ORF1a:F3444Y**	1	0.03
Maharashtra										**ORF1a:Y3445Q**	1	0.03
Maharashtra										**ORF1a:G3446L**	1	0.03
Maharashtra										**ORF1a:P3447E**	1	0.03
Maharashtra										**ORF1a:V3449M**	1	0.03
Maharashtra										**ORF1a:D3450L**	1	0.03
Maharashtra										**ORF1a:R3451A**	1	0.03
Maharashtra										**ORF1a:Q3455K**	1	0.03
Maharashtra										**ORF1a:A3457T**	1	0.03
Maharashtra										**ORF1a:G3458F**	1	0.03
Maharashtra										**ORF1a:T3459M**	1	0.03
Maharashtra										**ORF1a:G3521M**	1	0.03
Maharashtra										**ORF1a:I3522L**	1	0.03
Maharashtra										**ORF1a:A3523T**	1	0.03
Maharashtra										**ORF1a:V3524Y**	1	0.03
Maharashtra										**ORF1a:L3525***	1	0.03
Maharashtra										**ORF1a:M3527L**	1	0.03
Maharashtra										**ORF1a:C3528F**	1	0.03
Maharashtra										**ORF1a:A3529L**	1	0.03
Maharashtra										**ORF1a:S3530L**	1	0.03
Maharashtra										**ORF1a:L3531K**	1	0.03
Maharashtra										**ORF1a:K3532L**	1	0.03
Maharashtra										**ORF1a:Q3536Y**	1	0.03
Maharashtra										**ORF1a:N3537***	1	0.03
Maharashtra										**ORF1a:G3538K**	1	0.03

Maharashtra										**ORF1a:M3621I**	1	0.03
Maharashtra										**ORF1a:A3623L**	1	0.03
Maharashtra										**ORF1a:F3650Y**	1	0.03
Maharashtra										**ORF1a:D3681R**	1	0.03
Maharashtra										**ORF1a:C3682L**	1	0.03
Maharashtra										**ORF1a:V3683C**	1	0.03
Maharashtra										**ORF1a:M3684Y**	1	0.03
Maharashtra										**ORF1a:Y3685V**	1	0.03
Maharashtra										**ORF1a:A3686***	1	0.03
Maharashtra										**ORF1a:E1564N**	1	0.03
Maharashtra										**ORF1a:V1565S**	1	0.03
Maharashtra										**ORF1a:T1605***	1	0.03
Maharashtra										**ORF1a:K1606N**	1	0.03
Maharashtra										**ORF1a:E1630L**	1	0.03
Maharashtra										**ORF1a:A1631C**	1	0.03
Maharashtra										**ORF1a:E1633G**	1	0.03
Maharashtra										**ORF1a:Y1635V**	1	0.03
Maharashtra										**ORF1a:F2334I**	1	0.03
Maharashtra										**ORF1a:T2335Y**	1	0.03
Maharashtra										**ORF1a:G2342L**	1	0.03
Maharashtra										**ORF1a:A2344T**	1	0.03
Maharashtra										**ORF1a:A2345W**	1	0.03
Maharashtra										**ORF1a:P371L**	1	0.03
Maharashtra										**ORF1a:N4167S**	1	0.03
Maharashtra										**ORF1a:Q362K**	1	0.03
Maharashtra										**ORF1a:D982G**	1	0.03
Maharashtra										**ORF1a:V596I**	1	0.03
Maharashtra										**ORF1a:H110Y**	1	0.03
Maharashtra										**ORF1a:L1507F**	1	0.03
Maharashtra										**ORF1a:P1921S**	1	0.03
Maharashtra										**ORF1a:Q2749R**	1	0.03
Maharashtra										**ORF1a:V2750L**	1	0.03
Maharashtra										**ORF1a:V2779W**	1	0.03
Maharashtra										**ORF1a:Y1598F**	1	0.03
Maharashtra										**ORF1a:T1617I**	1	0.03
Maharashtra										**ORF1a:I1667N**	1	0.03
Maharashtra										**ORF1a:N1927D**	1	0.03
Maharashtra										**ORF1a:L1944***	1	0.03
Maharashtra										**ORF1a:T1945L**	1	0.03
Maharashtra										**ORF1a:I1153Y**	1	0.03
Maharashtra										**ORF1a:R1163K**	1	0.03
Maharashtra										**ORF1a:N1922R**	1	0.03
Maharashtra										**ORF1a:N1927K**	1	0.03
Maharashtra										**ORF1a:G445S**	1	0.03
Maharashtra										**ORF1a:G728H**	1	0.03
Maharashtra										**ORF1a:L729S**	1	0.03
Maharashtra										**ORF1a:S2553D**	1	0.03
Maharashtra										**ORF1a:T4175K**	1	0.03
Maharashtra										**ORF1a:G2294C**	1	0.03
Maharashtra										**ORF1a:D2296G**	1	0.03
Maharashtra										**ORF1a:D2299I**	1	0.03
Maharashtra										**ORF1a:D2317Y**	1	0.03
Maharashtra										**ORF1a:L2318S**	1	0.03
Maharashtra										**ORF1a:F2321A**	1	0.03
Maharashtra										**ORF1a:A2325R**	1	0.03
Maharashtra										**ORF1a:T4031I**	1	0.03
Maharashtra										**ORF1a:D2835N**	1	0.03
Maharashtra										**ORF1a:S3149P**	1	0.03
Maharashtra										**ORF1a:Q1198L**	1	0.03
Maharashtra										**ORF1a:V1199F**	1	0.03
Maharashtra										**ORF1a:E1200A**	1	0.03
Maharashtra										**ORF1a:A1204S**	1	0.03
Maharashtra										**ORF1a:I1002F**	1	0.03
Maharashtra										**ORF1a:T1004P**	1	0.03
Maharashtra										**ORF1a:D3479G**	1	0.03
Maharashtra										**ORF1a:R3480K**	1	0.03
Maharashtra										**ORF1a:W3481Q**	1	0.03
Maharashtra										**ORF1a:S984N**	1	0.03
Maharashtra										**ORF1a:T2197L**	1	0.03
Maharashtra										**ORF1a:I2198N**	1	0.03
Maharashtra										**ORF1a:K2219N**	1	0.03
Maharashtra										**ORF1a:P2221T**	1	0.03
Maharashtra										**ORF1a:F2223V**	1	0.03

Maharashtra										**ORF1a:S2224T**	1	0.03
Maharashtra										**ORF1a:K2225N**	1	0.03
Maharashtra										**ORF1a:L2226C**	1	0.03
Maharashtra										**ORF1a:I2227T**	1	0.03
Maharashtra										**ORF1a:N2228G**	1	0.03
Maharashtra										**ORF1a:I2229S**	1	0.03
Maharashtra										**ORF1a:L2234V**	1	0.03
Maharashtra										**ORF1a:E1013G**	1	0.03
Maharashtra										**ORF1a:D877Y**	1	0.03
Maharashtra										**ORF1a:E937***	1	0.03
Maharashtra										**ORF1a:E2G**	1	0.03
Maharashtra										**ORF1a:G2342A**	1	0.03
Maharashtra										**ORF1a:E65***	1	0.03
Maharashtra										**ORF1a:L123F**	1	0.03
Maharashtra										**ORF1a:L2536F**	1	0.03
Maharashtra										**ORF1a:I1276T**	1	0.03
Maharashtra										**ORF1a:P1803S**	1	0.03
Maharashtra										**ORF1a:G379R**	1	0.03
Maharashtra										**ORF1a:K798V**	1	0.03
Maharashtra										**ORF1a:I3476V**	1	0.03
Maharashtra										**ORF1a:V3892I**	1	0.03
Maharashtra										**ORF1a:S2104I**	1	0.03
Maharashtra										**ORF1a:K72P**	1	0.03
Maharashtra										**ORF1a:R73K**	1	0.03
Maharashtra										**ORF1a:A76D**	1	0.03
Maharashtra										**ORF1a:E87R**	1	0.03
Maharashtra										**ORF1a:L88S**	1	0.03
Maharashtra										**ORF1a:V89A**	1	0.03
Maharashtra										**ORF1a:A90P**	1	0.03
Maharashtra										**ORF1a:E91***	1	0.03
Maharashtra										**ORF1a:I95H**	1	0.03
Maharashtra										**ORF1a:Y97L**	1	0.03
Maharashtra										**ORF1a:G98L**	1	0.03
Maharashtra										**ORF1a:L570V**	1	0.03
Maharashtra										**ORF1a:V121F**	1	0.03
Maharashtra										**ORF1a:V14L**	1	0.03
Maharashtra										**ORF1a:S1952L**	1	0.03
Maharashtra										**ORF1a:T3000I**	1	0.03
Maharashtra										**ORF1a:I2774V**	1	0.03
Maharashtra										**ORF1a:P1220S**	1	0.03
Maharashtra										**ORF1a:L2122F**	1	0.03
Maharashtra										**ORF1a:K1895N**	1	0.03
Maharashtra										**ORF1a:T1573L**	1	0.03
Maharashtra										**ORF1a:V1574T**	1	0.03
Maharashtra										**ORF1a:D1575S**	1	0.03
Maharashtra										**ORF1a:N1576T**	1	0.03
Maharashtra										**ORF1a:I1577R**	1	0.03
Maharashtra										**ORF1a:H1580***	1	0.03
Maharashtra										**ORF1a:T1581H**	1	0.03
Maharashtra										**ORF1a:G1591S**	1	0.03
Maharashtra										**ORF1a:Q1592N**	1	0.03
Maharashtra										**ORF1a:Q1593L**	1	0.03
Maharashtra										**ORF1a:D1885P**	1	0.03
Maharashtra										**ORF1a:G1886***	1	0.03
Maharashtra										**ORF1a:V1888G**	1	0.03
Maharashtra										**ORF1a:F1960G**	1	0.03
Maharashtra										**ORF1a:K399N**	1	0.03
Maharashtra										**ORF1a:T1572S**	1	0.03
Maharashtra										**ORF1a:V1574R**	1	0.03
Maharashtra										**ORF1a:D1575K**	1	0.03
Maharashtra										**ORF1a:N1576L**	1	0.03
Maharashtra										**ORF1a:I1577W**	1	0.03
Maharashtra										**ORF1a:K1608V**	1	0.03
Maharashtra										**ORF1a:L1748G**	1	0.03
Maharashtra										**ORF1a:Y2281L**	1	0.03
Maharashtra										**ORF1a:T2283M**	1	0.03
Maharashtra										**ORF1a:G2284S**	1	0.03
Maharashtra										**ORF1a:S2285L**	1	0.03
Maharashtra										**ORF1a:I2286L**	1	0.03
Maharashtra										**ORF1a:P2287Q**	1	0.03
Maharashtra										**ORF1a:C2288P**	1	0.03
Maharashtra										**ORF1a:S2289T**	1	0.03
Maharashtra										**ORF1a:C2291T**	1	0.03

Maharashtra										**ORF1a:C2694T**	1	0.03
Maharashtra										**ORF1a:T3090I**	1	0.03
Maharashtra										**ORF1a:L205***	1	0.03
Maharashtra										**ORF1a:A206H**	1	0.03
Maharashtra										**ORF1a:L320***	1	0.03
Maharashtra										**ORF1a:D324A**	1	0.03
Maharashtra										**ORF1a:H325F**	1	0.03
Maharashtra										**ORF1a:C326Q**	1	0.03
Maharashtra										**ORF1a:G327L**	1	0.03
Maharashtra										**ORF1a:E328S**	1	0.03
Maharashtra										**ORF1a:T329***	1	0.03
Maharashtra										**ORF1a:W331K**	1	0.03
Maharashtra										**ORF1a:Q332P**	1	0.03
Maharashtra										**ORF1a:T333L**	1	0.03
Maharashtra										**ORF1a:G334A**	1	0.03
Maharashtra										**ORF1a:Y3211S**	1	0.03
Maharashtra										**ORF1a:A2375V**	1	0.03
Maharashtra										**ORF1a:I2786F**	1	0.03
Maharashtra										**ORF1a:F2787L**	1	0.03
Maharashtra										**ORF1a:Y2788F**	1	0.03
Maharashtra										**ORF1a:L2789N**	1	0.03
Maharashtra										**ORF1a:I2790N**	1	0.03
Maharashtra										**ORF1a:T2898***	1	0.03
Maharashtra										**ORF1a:P2899C**	1	0.03
Maharashtra										**ORF1a:K2901S**	1	0.03
Maharashtra										**ORF1a:V2943L**	1	0.03
Maharashtra										**ORF1a:G3265C**	1	0.03
Maharashtra										**ORF1a:G1101S**	1	0.03
Maharashtra										**ORF1a:G3846S**	1	0.03
Maharashtra										**ORF1a:K1936I**	1	0.03
Maharashtra										**ORF1a:I1970C**	1	0.03
Maharashtra										**ORF1a:D1971G**	1	0.03
Maharashtra										**ORF1a:K1973***	1	0.03
Maharashtra										**ORF1a:H1974L**	1	0.03
Maharashtra										**ORF1a:Y1975***	1	0.03
Maharashtra										**ORF1a:P1977R**	1	0.03
Maharashtra										**ORF1a:D3311E**	1	0.03
Maharashtra										**ORF1a:G572P**	1	0.03
Maharashtra										**ORF1a:I573L**	1	0.03
Maharashtra										**ORF1a:V665L**	1	0.03
Maharashtra										**ORF1a:T666V**	1	0.03
Maharashtra										**ORF1a:C667K**	1	0.03
Maharashtra										**ORF1a:A668L**	1	0.03
Maharashtra										**ORF1a:K669S**	1	0.03
Maharashtra										**ORF1a:E670V**	1	0.03
Maharashtra										**ORF1a:I671D**	1	0.03
Maharashtra										**ORF1a:V675P**	1	0.03
Maharashtra										**ORF1a:Q676V**	1	0.03
Maharashtra										**ORF1a:G1152***	1	0.03
Maharashtra										**ORF1a:I1153E**	1	0.03
Maharashtra										**ORF1a:G1155V**	1	0.03
Maharashtra										**ORF1a:A1156***	1	0.03
Maharashtra										**ORF1a:D1157I**	1	0.03
Maharashtra										**ORF1a:P1158L**	1	0.03
Maharashtra										**ORF1a:I1159S**	1	0.03
Maharashtra										**ORF1a:P1472C**	1	0.03
Maharashtra										**ORF1a:A1473Y**	1	0.03
Maharashtra										**ORF1a:V1477W**	1	0.03
Maharashtra										**ORF1a:E1498Y**	1	0.03
Maharashtra										**ORF1a:E1499F**	1	0.03
Maharashtra										**ORF1a:H1500F**	1	0.03
Maharashtra										**ORF1a:I1502***	1	0.03
Maharashtra										**ORF1a:E1503N**	1	0.03
Maharashtra										**ORF1a:R3323I**	1	0.03
Maharashtra										**ORF1a:K3324I**	1	0.03
Maharashtra										**ORF1a:G52A**	1	0.03
Maharashtra										**ORF1a:I71K**	1	0.03
Maharashtra										**ORF1a:R73S**	1	0.03
Maharashtra										**ORF1a:A76V**	1	0.03
Maharashtra										**ORF1a:P109R**	1	0.03
Maharashtra										**ORF1a:P115L**	1	0.03
Maharashtra										**ORF1a:G179A**	1	0.03
Maharashtra										**ORF1a:G180M**	1	0.03

Maharashtra										**ORF1a:S212N**	1	0.03
Maharashtra										**ORF1a:C213W**	1	0.03
Maharashtra										**ORF1a:P286L**	1	0.03
Maharashtra										**ORF1a:R287W**	1	0.03
Maharashtra										**ORF1a:M297D**	1	0.03
Maharashtra										**ORF1a:G298L**	1	0.03
Maharashtra										**ORF1a:R299S**	1	0.03
Maharashtra										**ORF1a:T350L**	1	0.03
Maharashtra										**ORF1a:E352P**	1	0.03
Maharashtra										**ORF1a:G353L**	1	0.03
Maharashtra										**ORF1a:S1478F**	1	0.03
Maharashtra										**ORF1a:S1479F**	1	0.03
Maharashtra										**ORF1a:P1480T**	1	0.03
Maharashtra										**ORF1a:L2341N**	1	0.03
Maharashtra										**ORF1a:K2395T**	1	0.03
Maharashtra										**ORF1a:Y2397S**	1	0.03
Maharashtra										**ORF1a:V2398L**	1	0.03
Maharashtra										**ORF1a:N2523E**	1	0.03
Maharashtra										**ORF1a:F2979K**	1	0.03
Maharashtra										**ORF1a:W3004Q**	1	0.03
Maharashtra										**ORF1a:V3005K**	1	0.03
Maharashtra										**ORF1a:N3007V**	1	0.03
Maharashtra										**ORF1a:N3008F**	1	0.03
Maharashtra										**ORF1a:D3009V**	1	0.03
Maharashtra										**ORF1a:Y3011L**	1	0.03
Maharashtra										**ORF1a:R3012L**	1	0.03
Maharashtra										**ORF1a:S3013V**	1	0.03
Maharashtra										**ORF1a:L3014V**	1	0.03
Maharashtra										**ORF1a:P3015D**	1	0.03
Maharashtra										**ORF1a:V3017L**	1	0.03
Maharashtra										**ORF1a:F3018I**	1	0.03
Maharashtra										**ORF1a:G3020L**	1	0.03
Maharashtra										**ORF1a:V3021H**	1	0.03
Maharashtra										**ORF1a:D3022H**	1	0.03
Maharashtra										**ORF1a:E3073Y**	1	0.03
Maharashtra										**ORF1a:G3265H**	1	0.03
Maharashtra										**ORF1a:F3266L**	1	0.03
Maharashtra										**ORF1a:R3267V**	1	0.03
Maharashtra										**ORF1a:M3269L**	1	0.03
Maharashtra										**ORF1a:A3270R**	1	0.03
Maharashtra										**ORF1a:F3271V**	1	0.03
Maharashtra										**ORF1a:P3272V**	1	0.03
Maharashtra										**ORF1a:S3273W**	1	0.03
Maharashtra										**ORF1a:G3274Y**	1	0.03
Maharashtra										**ORF1a:D3296***	1	0.03
Maharashtra										**ORF1a:D3297L**	1	0.03
Maharashtra										**ORF1a:Q3519R**	1	0.03
Maharashtra										**ORF1a:G3521R**	1	0.03
Maharashtra										**ORF1a:M3652F**	1	0.03
Maharashtra										**ORF1a:L3711Y**	1	0.03
Maharashtra										**ORF1a:N3713C**	1	0.03
Maharashtra										**ORF1a:V3714L**	1	0.03
Maharashtra										**ORF1a:L3915C**	1	0.03
Maharashtra										**ORF1a:M4036T**	1	0.03
Maharashtra										**ORF1a:I4061V**	1	0.03
Maharashtra										**ORF1a:I4062F**	1	0.03
Maharashtra										**ORF1a:L4064***	1	0.03
Maharashtra										**ORF1a:A4067H**	1	0.03
Maharashtra										**ORF1a:A4068I**	1	0.03
Maharashtra										**ORF1a:M4071R**	1	0.03
Maharashtra										**ORF1a:I4098V**	1	0.03
Maharashtra										**ORF1a:A4156L**	1	0.03
Maharashtra										**ORF1a:G4157M**	1	0.03
Maharashtra										**ORF1a:A4170M**	1	0.03
Maharashtra										**ORF1a:Y4171R**	1	0.03
Maharashtra										**ORF1a:Y4172***	1	0.03
Maharashtra										**ORF1a:N4173L**	1	0.03
Maharashtra										**ORF1a:G4178Q**	1	0.03
Maharashtra										**ORF1a:F4180E**	1	0.03
Maharashtra										**ORF1a:G4201L**	1	0.03
Maharashtra										**ORF1a:T4202V**	1	0.03
Maharashtra										**ORF1a:T4204L**	1	0.03
Maharashtra										**ORF1a:I4205L**	1	0.03

Maharashtra										ORF1a:Y4206Q	1	0.03
Maharashtra										ORF1a:E4208H	1	0.03
Maharashtra										ORF1a:Y1489V	1	0.03
Maharashtra										ORF1a:P3447H	1	0.03
Maharashtra										ORF1a:L3692C	1	0.03
Maharashtra										ORF1a:I3693M	1	0.03
Maharashtra										ORF1a:L3694I	1	0.03
Maharashtra										ORF1a:M3695S	1	0.03
Maharashtra										ORF1a:T3696C	1	0.03
Maharashtra										ORF1a:A3697S	1	0.03
Maharashtra										ORF1a:R3698V	1	0.03
Maharashtra										ORF1a:M3712L	1	0.03
Maharashtra										ORF1a:N3713*	1	0.03
Maharashtra										ORF1a:V3714S	1	0.03
Maharashtra										ORF1a:T2872M	1	0.03
Maharashtra										ORF1a:I1159L	1	0.03
Maharashtra										ORF1a:V3420L	1	0.03
Maharashtra										ORF1a:L3674F	1	0.03
Maharashtra										ORF1a:T3356P	1	0.03
Maharashtra										ORF1a:G645V	1	0.03
Maharashtra										ORF1a:I2286T	1	0.03
Maharashtra										ORF1a:M3761I	1	0.03
Maharashtra										ORF1a:Y1859H	1	0.03
Maharashtra										ORF1a:I1206V	1	0.03
Maharashtra										ORF1a:N3725Y	1	0.03
Maharashtra										ORF1a:A876T	1	0.03
Maharashtra										ORF1a:E772D	1	0.03
Maharashtra										ORF1a:D2835G	1	0.03
Maharashtra										ORF1a:L3941*	1	0.03
Maharashtra										ORF1a:V3968A	1	0.03
Maharashtra										ORF1a:N3970Y	1	0.03
Maharashtra										ORF1a:G3971D	1	0.03
Maharashtra										ORF1a:S3973R	1	0.03
Maharashtra										ORF1a:V3975L	1	0.03
Maharashtra										ORF1a:V3976N	1	0.03
Maharashtra										ORF1a:L3977S	1	0.03
Maharashtra										ORF1a:K3978E	1	0.03
Maharashtra										ORF1a:F685L	1	0.03
Maharashtra										ORF1a:S2242F	1	0.03
Maharashtra										ORF1a:I3108V	1	0.03
Maharashtra										ORF1a:V488L	1	0.03
Maharashtra										ORF1a:M584I	1	0.03
Maharashtra										ORF1a:Y799D	1	0.03
Maharashtra										ORF1a:Q4189H	1	0.03
Maharashtra										ORF1a:V2047F	1	0.03
Maharashtra										ORF1a:D1554A	1	0.03
Maharashtra										ORF1a:R1701G	1	0.03
Maharashtra										ORF1a:M321T	1	0.03
Maharashtra										ORF1a:C3790F	1	0.03
Maharashtra										ORF1a:E217G	1	0.03
Maharashtra										ORF1a:K634N	1	0.03
Maharashtra										ORF1a:P1786L	1	0.03
Maharashtra										ORF1a:K2395Q	1	0.03
Maharashtra										ORF1a:P959S	1	0.03
Maharashtra										ORF1a:T346I	1	0.03
Maharashtra										ORF1a:V2943F	1	0.03
Maharashtra										ORF1a:S40L	1	0.03
Maharashtra										ORF1a:V414I	1	0.03
Maharashtra										ORF1a:K367T	1	0.03
Maharashtra										ORF1a:K141D	1	0.03
Maharashtra										ORF1a:C3790Y	1	0.03
Maharashtra										ORF1a:V3782I	1	0.03
Maharashtra										ORF1a:I2368L	1	0.03
Maharashtra										ORF1a:I2961V	1	0.03
Maharashtra										ORF1a:V4181I	1	0.03
Maharashtra										ORF1a:E940G	1	0.03
Maharashtra										ORF1a:A1156V	1	0.03
Maharashtra										ORF1a:N1662S	1	0.03
Maharashtra										ORF1a:S771I	1	0.03
Maharashtra										ORF1a:Y182H	1	0.03
Maharashtra										ORF1a:G442S	1	0.03
Maharashtra										ORF1a:R3662C	1	0.03
Maharashtra										ORF1a:P4220L	1	0.03

Maharashtra										**ORF1a:L259F**	1	0.03
Maharashtra										**ORF1a:E939G**	1	0.03
Maharashtra										**ORF1a:V1056***	1	0.03
Maharashtra										**ORF1a:V1057T**	1	0.03
Maharashtra										**ORF1a:V1058W**	1	0.03
Maharashtra										**ORF1a:N1059R**	1	0.03
Maharashtra										**ORF1a:A1060R**	1	0.03
Maharashtra										**ORF1a:A1061C**	1	0.03
Maharashtra										**ORF1a:N1062C**	1	0.03
Maharashtra										**ORF1a:A1092S**	1	0.03
Maharashtra										**ORF1a:T1093***	1	0.03
Maharashtra										**ORF1a:N1094I**	1	0.03
Maharashtra										**ORF1a:G1095***	1	0.03
Maharashtra										**ORF1a:P1096***	1	0.03
Maharashtra										**ORF1a:G1107S**	1	0.03
Maharashtra										**ORF1a:H1108C**	1	0.03
Maharashtra										**ORF1a:N1109C**	1	0.03
Maharashtra										**ORF1a:L1110R**	1	0.03
Maharashtra										**ORF1a:E1413***	1	0.03
Maharashtra										**ORF1a:I1435L**	1	0.03
Maharashtra										**ORF1a:N1436H**	1	0.03
Maharashtra										**ORF1a:T1437Q**	1	0.03
Maharashtra										**ORF1a:L1438***	1	0.03
Maharashtra										**ORF1a:D1440N**	1	0.03
Maharashtra										**ORF1a:L1441C**	1	0.03
Maharashtra										**ORF1a:N1442S**	1	0.03
Maharashtra										**ORF1a:E1443V**	1	0.03
Maharashtra										**ORF1a:V1446C**	1	0.03
Maharashtra										**ORF1a:T1447N**	1	0.03
Maharashtra										**ORF1a:M1448T**	1	0.03
Maharashtra										**ORF1a:P1449W**	1	0.03
Maharashtra										**ORF1a:R1464K**	1	0.03
Maharashtra										**ORF1a:Y1465F**	1	0.03
Maharashtra										**ORF1a:M1466G**	1	0.03
Maharashtra										**ORF1a:S1492F**	1	0.03
Maharashtra										**ORF1a:S1493Y**	1	0.03
Maharashtra										**ORF1a:S1494***	1	0.03
Maharashtra										**ORF1a:K1495N**	1	0.03
Maharashtra										**ORF1a:T1496H**	1	0.03
Maharashtra										**ORF1a:E1498T**	1	0.03
Maharashtra										**ORF1a:E1499C**	1	0.03
Maharashtra										**ORF1a:H1500W**	1	0.03
Maharashtra										**ORF1a:I1502L**	1	0.03
Maharashtra										**ORF1a:E1503***	1	0.03
Maharashtra										**ORF1a:T1504R**	1	0.03
Maharashtra										**ORF1a:L3640V**	1	0.03
Maharashtra										**ORF1a:L3641T**	1	0.03
Maharashtra										**ORF1a:P3642F**	1	0.03
Maharashtra										**ORF1a:L1955F**	1	0.03
Maharashtra										**ORF1a:Y4077I**	1	0.03
Maharashtra										**ORF1a:N4078A**	1	0.03
Maharashtra										**ORF1a:T4079M**	1	0.03
Maharashtra										**ORF1a:S4145***	1	0.03
Maharashtra										**ORF1a:P4146S**	1	0.03
Maharashtra										**ORF1a:T1216I**	1	0.03
Maharashtra										**ORF1a:M1586N**	1	0.03
Maharashtra										**ORF1a:T1605Q**	1	0.03
Maharashtra										**ORF1a:K1606L**	1	0.03
Maharashtra										**ORF1a:K1608L**	1	0.03
Maharashtra										**ORF1a:H1610***	1	0.03
Maharashtra										**ORF1a:R2887F**	1	0.03
Maharashtra										**ORF1a:V2888A**	1	0.03
Maharashtra										**ORF1a:S2890F**	1	0.03
Maharashtra										**ORF1a:V2892***	1	0.03
Maharashtra										**ORF1a:G2893S**	1	0.03
Maharashtra										**ORF1a:N2894F**	1	0.03
Maharashtra										**ORF1a:R2949***	1	0.03
Maharashtra										**ORF1a:P2950K**	1	0.03
Maharashtra										**ORF1a:D2951F**	1	0.03
Maharashtra										**ORF1a:R2953A**	1	0.03
Maharashtra										**ORF1a:V2955G**	1	0.03
Maharashtra										**ORF1a:L2956W**	1	0.03
Maharashtra										**ORF1a:A3045R**	1	0.03

Maharashtra										ORF1a:I1206T	1	0.03
Maharashtra										ORF1a:E647G	1	0.03
Maharashtra										ORF1a:Y2099D	1	0.03
Maharashtra										ORF1a:D2525N	1	0.03
Maharashtra										ORF1a:S1561F	1	0.03
Maharashtra										ORF1a:E1614K	1	0.03
Maharashtra										ORF1a:T224N	1	0.03
Maharashtra										ORF1a:A2855V	1	0.03
Maharashtra										ORF1a:I114T	1	0.03
Maharashtra										ORF1a:R4017K	1	0.03
Maharashtra										ORF1a:K292R	1	0.03
Maharashtra										ORF1a:N704K	1	0.03
Maharashtra										ORF1a:G706S	1	0.03
Maharashtra										ORF1a:S3088F	1	0.03
Maharashtra										ORF1a:D264E	1	0.03
Maharashtra										ORF1a:T265L	1	0.03
Maharashtra										ORF1a:F266D	1	0.03
Maharashtra										ORF1a:P271T	1	0.03
Maharashtra										ORF1a:N272D	1	0.03
Maharashtra										ORF1a:R301L	1	0.03
Maharashtra										ORF1a:S302P	1	0.03
Maharashtra										ORF1a:E311*	1	0.03
Maharashtra										ORF1a:C312S	1	0.03
Maharashtra										ORF1a:M315A	1	0.03
Maharashtra										ORF1a:C316F	1	0.03
Maharashtra										ORF1a:M2383L	1	0.03
Maharashtra										ORF1a:W3128C	1	0.03
Maharashtra										ORF1a:W3139L	1	0.03
Maharashtra										ORF1a:I3140*	1	0.03
Maharashtra										ORF1a:T3646R	1	0.03
Maharashtra										ORF1a:V3647E	1	0.03
Maharashtra										ORF1a:A3648G	1	0.03
Maharashtra										ORF1a:Y3649N	1	0.03
Maharashtra										ORF1a:F3650K	1	0.03
Maharashtra										ORF1a:M3661K	1	0.03
Maharashtra										ORF1a:I3663N	1	0.03
Maharashtra										ORF1a:S2205N	1	0.03
Maharashtra										ORF1a:V2206L	1	0.03
Maharashtra										ORF1a:F2215L	1	0.03
Maharashtra										ORF1a:I2227V	1	0.03
Maharashtra										ORF1a:I2229T	1	0.03
Maharashtra										ORF1a:I2230K	1	0.03
Maharashtra										ORF1a:I2231L	1	0.03
Maharashtra										ORF1a:W2232*	1	0.03
Maharashtra										ORF1a:F2233Y	1	0.03
Maharashtra										ORF1a:L2235S	1	0.03
Maharashtra										ORF1a:E2268K	1	0.03
Maharashtra										ORF1a:L2295I	1	0.03
Maharashtra										ORF1a:F3366I	1	0.03
Maharashtra										ORF1a:V3367C	1	0.03
Maharashtra										ORF1a:R3368Y	1	0.03
Maharashtra										ORF1a:S2844F	1	0.03
Maharashtra										ORF1a:Y2845C	1	0.03
Maharashtra										ORF1a:V26L	1	0.03
Maharashtra										ORF1a:R73Q	1	0.03
Maharashtra										ORF1a:S74A	1	0.03
Maharashtra										ORF1a:S723F	1	0.03
Maharashtra										ORF1a:R724K	1	0.03
Maharashtra										ORF1a:G7I	1	0.03
Maharashtra										ORF1a:G82K	1	0.03
Maharashtra										ORF1a:F3183L	1	0.03
Maharashtra										ORF1a:V56P	1	0.03
Maharashtra										ORF1a:A2732V	1	0.03
Maharashtra										ORF1a:F679L	1	0.03
Maharashtra										ORF1a:V4369I	1	0.03
Maharashtra										ORF1a:T3398I	1	0.03
Maharashtra										ORF1a:E37K	1	0.03
Maharashtra										ORF1a:N389K	1	0.03
Maharashtra										ORF1a:A1204V	1	0.03
Maharashtra										ORF1a:T3487I	1	0.03
Maharashtra										ORF1a:A2199K	1	0.03
Maharashtra										ORF1a:K2200R	1	0.03
Maharashtra										ORF1a:L2039P	1	0.03

Maharashtra										**ORF1a:S2132G**	1	0.03
Maharashtra										**ORF1a:T1876I**	1	0.03
Maharashtra										**ORF1a:I3035S**	1	0.03
Maharashtra										**ORF1a:L4246S**	1	0.03
Maharashtra										**ORF1a:E2088K**	1	0.03
Maharashtra										**ORF1a:L1182F**	1	0.03
Maharashtra										**ORF1a:T4026I**	1	0.03
Maharashtra										**ORF1a:K881R**	1	0.03
Maharashtra										**ORF1a:H1067Y**	1	0.03
Maharashtra										**ORF1a:M1T**	1	0.03
Maharashtra										**ORF1a:V5L**	1	0.03
Maharashtra										**ORF1a:P6Q**	1	0.03
Maharashtra										**ORF1a:G7S**	1	0.03
Maharashtra										**ORF1a:L27R**	1	0.03
Maharashtra										**ORF1a:V28A**	1	0.03
Maharashtra										**ORF1a:E870D**	1	0.03
Maharashtra										**ORF1a:E797Y**	1	0.03
Maharashtra										**ORF1a:K798***	1	0.03
Maharashtra										**ORF1a:F2354V**	1	0.03
Maharashtra										**ORF1a:Y185H**	1	0.03
Maharashtra										**ORF1a:V186N**	1	0.03
Maharashtra										**ORF1a:D187M**	1	0.03
Maharashtra										**ORF1a:K1280N**	1	0.03
Maharashtra										**ORF1a:Q986K**	1	0.03
Maharashtra										**ORF1a:H1160Y**	1	0.03
Maharashtra										**ORF1a:K4198R**	1	0.03
Maharashtra										**ORF1a:V675I**	1	0.03
Maharashtra										**ORF1a:T2124A**	1	0.03
Maharashtra										**ORF1a:L3550S**	1	0.03
Maharashtra										**ORF1a:L788H**	1	0.03
Maharashtra										**ORF1a:L2146P**	1	0.03
Maharashtra										**ORF1a:P769S**	1	0.03
Maharashtra										**ORF1a:I2873M**	1	0.03
Maharashtra										**ORF1a:A3945S**	1	0.03
Maharashtra										**ORF1a:Y241C**	1	0.03
Maharashtra										**ORF1a:V2252F**	1	0.03
Maharashtra										**ORF1a:I1935T**	1	0.03
Maharashtra										**ORF1a:K1124N**	1	0.03
Maharashtra										**ORF1a:F3804S**	1	0.03
Maharashtra										**ORF1a:F941C**	1	0.03
Maharashtra										**ORF1a:K2923R**	1	0.03
Maharashtra										**ORF1a:P4197S**	1	0.03
Maharashtra										**ORF1a:F941S**	1	0.03
Maharashtra										**ORF1a:L1270F**	1	0.03
Maharashtra										**ORF1a:L730I**	1	0.03
Maharashtra										**ORF1a:E55D**	1	0.03
Maharashtra										**ORF1a:L469F**	1	0.03
Maharashtra										**ORF1a:P4211L**	1	0.03
Maharashtra										**ORF1a:Q1227H**	1	0.03
Maharashtra										**ORF1a:M2254I**	1	0.03
Maharashtra										**ORF1a:L1680F**	1	0.03
Maharashtra										**ORF1a:G2640E**	1	0.03
Maharashtra										**ORF1a:D589A**	1	0.03
Maharashtra										**ORF1a:F1028I**	1	0.03
Maharashtra										**ORF1a:L2318***	1	0.03
Maharashtra										**ORF1a:F2209I**	1	0.03
Maharashtra										**ORF1a:T3141A**	1	0.03
Maharashtra										**ORF1a:G7N**	1	0.03
Maharashtra										**ORF1a:N9Y**	1	0.03
Maharashtra										**ORF1a:E10***	1	0.03
Maharashtra										**ORF1a:K11A**	1	0.03
Maharashtra										**ORF1a:H13S**	1	0.03
Maharashtra										**ORF1a:Q15I**	1	0.03
Maharashtra										**ORF1a:L16F**	1	0.03
Maharashtra										**ORF1a:S17K**	1	0.03
Maharashtra										**ORF1a:L18M**	1	0.03
Maharashtra										**ORF1a:V20P**	1	0.03
Maharashtra										**ORF1a:L21P**	1	0.03
Maharashtra										**ORF1a:Q22R**	1	0.03
Maharashtra										**ORF1a:V23S**	1	0.03
Maharashtra										**ORF1a:R24L**	1	0.03
Maharashtra										**ORF1a:D25Q**	1	0.03
Maharashtra										**ORF1a:V26S**	1	0.03

Maharashtra										**ORF1a:L27H**	1	0.03
Maharashtra										**ORF1a:G30A**	1	0.03
Maharashtra										**ORF1a:F31R**	1	0.03
Maharashtra										**ORF1a:G32R**	1	0.03
Maharashtra										**ORF1a:D33E**	1	0.03
Maharashtra										**ORF1a:S34P**	1	0.03
Maharashtra										**ORF1a:E36K**	1	0.03
Maharashtra										**ORF1a:E37Q**	1	0.03
Maharashtra										**ORF1a:V38A**	1	0.03
Maharashtra										**ORF1a:L39N**	1	0.03
Maharashtra										**ORF1a:S40***	1	0.03
Maharashtra										**ORF1a:E41V**	1	0.03
Maharashtra										**ORF1a:A42G**	1	0.03
Maharashtra										**ORF1a:Q44V**	1	0.03
Maharashtra										**ORF1a:H45F**	1	0.03
Maharashtra										**ORF1a:V56T**	1	0.03
Maharashtra										**ORF1a:E57R**	1	0.03
Maharashtra										**ORF1a:K58D**	1	0.03
Maharashtra										**ORF1a:G59K**	1	0.03
Maharashtra										**ORF1a:P62H**	1	0.03
Maharashtra										**ORF1a:H81F**	1	0.03
Maharashtra										**ORF1a:L1368V**	1	0.03
Maharashtra										**ORF1a:G1369P**	1	0.03
Maharashtra										**ORF1a:V1371N**	1	0.03
Maharashtra										**ORF1a:W1373C**	1	0.03
Maharashtra										**ORF1a:L1375S**	1	0.03
Maharashtra										**ORF1a:R1376L**	1	0.03
Maharashtra										**ORF1a:L1379I**	1	0.03
Maharashtra										**ORF1a:K1396M**	1	0.03
Maharashtra										**ORF1a:F3769N**	1	0.03
Maharashtra										**ORF1a:I3779T**	1	0.03
Maharashtra										**ORF1a:M3780K**	1	0.03
Maharashtra										**ORF1a:V3782A**	1	0.03
Maharashtra										**ORF1a:P361S**	1	0.03
Maharashtra										**ORF1a:N9D**	1	0.03
Maharashtra										**ORF1a:T1395N**	1	0.03
Maharashtra										**ORF1a:H2831R**	1	0.03
Maharashtra										**ORF1a:P3447S**	1	0.03
Maharashtra										**ORF1a:V606I**	1	0.03
Maharashtra										**ORF1a:T12I**	1	0.03
Maharashtra										**ORF1a:V2522I**	1	0.03
Maharashtra										**ORF1a:L2609F**	1	0.03
Maharashtra										**ORF1a:K3632N**	1	0.03
Maharashtra										**ORF1a:A773D**	1	0.03
Maharashtra										**ORF1a:V774T**	1	0.03
Maharashtra										**ORF1a:V2652I**	1	0.03
Maharashtra										**ORF1a:L700H**	1	0.03
Maharashtra										**ORF1a:L703K**	1	0.03
Maharashtra										**ORF1a:P305S**	1	0.03
Maharashtra										**ORF1a:E553A**	1	0.03
Maharashtra										**ORF1a:Y2171F**	1	0.03
Maharashtra										**ORF1a:V597I**	1	0.03
Maharashtra										**ORF1a:T183A**	1	0.03
Maharashtra										**ORF1a:G2118D**	1	0.03
Maharashtra										**ORF1a:N2523T**	1	0.03
Maharashtra										**ORF1a:V1211F**	1	0.03
Maharashtra										**ORF1a:T2859I**	1	0.03
Maharashtra										**ORF1a:H2799Y**	1	0.03
Maharashtra										**ORF1a:A474T**	1	0.03
Maharashtra										**ORF1a:T746S**	1	0.03
Maharashtra										**ORF1a:I4049V**	1	0.03
Maharashtra										**ORF1a:D1167G**	1	0.03
Maharashtra										**ORF1a:C4381***	1	0.03
Maharashtra										**ORF1a:D2980G**	1	0.03
Maharashtra										**ORF1a:M3652V**	1	0.03
Maharashtra										**ORF1a:P380S**	1	0.03
Maharashtra										**ORF1a:Y249V**	1	0.03
Maharashtra										**ORF1a:E1826G**	1	0.03
Maharashtra										**ORF1a:S984I**	1	0.03
Maharashtra										**ORF1a:T2181I**	1	0.03
Maharashtra										**ORF1a:V3572M**	1	0.03
Maharashtra										**ORF1a:V4057I**	1	0.03
Maharashtra										**ORF1a:I1259T**	1	0.03

Maharashtra										**ORF1a:V3336I**	1	0.03
Maharashtra										**ORF1a:A4396V**	1	0.03
Maharashtra										**ORF1a:A4396T**	1	0.03
Maharashtra										**ORF1a:A1268S**	1	0.03
Maharashtra										**ORF1a:V2164I**	1	0.03
Maharashtra										**ORF1a:F4269L**	1	0.03
Maharashtra										**ORF1a:T1017I**	1	0.03
Maharashtra										**ORF1a:M1588I**	1	0.03
Maharashtra										**ORF1a:D1940N**	1	0.03
Maharashtra										**ORF1a:R3802S**	1	0.03
Maharashtra										**ORF1a:P2852S**	1	0.03
Maharashtra										**ORF1a:S3001N**	1	0.03
Maharashtra										**ORF1a:A687V**	1	0.03
Maharashtra										**ORF1a:R287S**	1	0.03
Maharashtra										**ORF1a:R3394M**	1	0.03
Maharashtra										**ORF1a:S2722F**	1	0.03
Maharashtra										**ORF1a:R4387H**	1	0.03
Maharashtra										**ORF1a:S2992A**	1	0.03
Maharashtra										**ORF1a:A3945V**	1	0.03
Maharashtra										**ORF1a:P271S**	1	0.03
Maharashtra										**ORF1a:F2979I**	1	0.03
Maharashtra										**ORF1a:T2495I**	1	0.03
Maharashtra										**ORF1a:L446F**	1	0.03
Maharashtra										**ORF1a:E2031K**	1	0.03
Maharashtra										**ORF1a:V3925I**	1	0.03
Maharashtra										**ORF1a:V1236F**	1	0.03
Maharashtra										**ORF1a:N508D**	1	0.03
Maharashtra										**ORF1a:V3559I**	1	0.03
Maharashtra										**ORF1a:V424A**	1	0.03
Maharashtra										**ORF1a:N1335S**	1	0.03
Maharashtra										**ORF1a:V35A**	1	0.03
Maharashtra										**ORF1a:A3645V**	1	0.03
Maharashtra										**ORF1a:E939***	1	0.03
Maharashtra										**ORF1a:S2132R**	1	0.03
Maharashtra										**ORF1a:L642F**	1	0.03
Maharashtra										**ORF1a:V3759G**	1	0.03
Maharashtra										**ORF1a:S216F**	1	0.03
Maharashtra										**ORF1a:A2821S**	1	0.03
Maharashtra										**ORF1a:A1151T**	1	0.03
Maharashtra										**ORF1a:G1152K**	1	0.03
Maharashtra										**ORF1a:D1179F**	1	0.03
Maharashtra										**ORF1a:K1180A**	1	0.03
Maharashtra										**ORF1a:N1181Q**	1	0.03
Maharashtra										**ORF1a:L1182M**	1	0.03
Maharashtra										**ORF1a:D1184T**	1	0.03
Maharashtra										**ORF1a:A1923***	1	0.03
Maharashtra										**ORF1a:F1925***	1	0.03
Maharashtra										**ORF1a:D1926***	1	0.03
Maharashtra										**ORF1a:Q2728V**	1	0.03
Maharashtra										**ORF1a:R2730V**	1	0.03
Maharashtra										**ORF1a:K2734T**	1	0.03
Maharashtra										**ORF1a:N2736T**	1	0.03
Maharashtra										**ORF1a:V2753Y**	1	0.03
Maharashtra										**ORF1a:V2754***	1	0.03
Maharashtra										**ORF1a:C2851L**	1	0.03
Maharashtra										**ORF1a:L2853I**	1	0.03
Maharashtra										**ORF1a:I2854D**	1	0.03
Maharashtra										**ORF1a:A2856C**	1	0.03
Maharashtra										**ORF1a:V2857S**	1	0.03
Maharashtra										**ORF1a:I2858H**	1	0.03
Maharashtra										**ORF1a:T2859N**	1	0.03
Maharashtra										**ORF1a:V3248***	1	0.03
Maharashtra										**ORF1a:P3272T**	1	0.03
Maharashtra										**ORF1a:K3400E**	1	0.03
Maharashtra										**ORF1a:G3401A**	1	0.03
Maharashtra										**ORF1a:S3402Q**	1	0.03
Maharashtra										**ORF1a:L3404R**	1	0.03
Maharashtra										**ORF1a:N3405L**	1	0.03
Maharashtra										**ORF1a:G3406***	1	0.03
Maharashtra										**ORF1a:S3407L**	1	0.03
Maharashtra										**ORF1a:P3431L**	1	0.03
Maharashtra										**ORF1a:T3432F**	1	0.03
Maharashtra										**ORF1a:N3609C**	1	0.03

Maharashtra										ORF1a:A3610L	1	0.03
Maharashtra										ORF1a:P3613Q	1	0.03
Maharashtra										ORF1a:F3614*	1	0.03
Maharashtra										ORF1a:G3746F	1	0.03
Maharashtra										ORF1a:T3905C	1	0.03
Maharashtra										ORF1a:M3921H	1	0.03
Maharashtra										ORF1a:Q3922A	1	0.03
Maharashtra										ORF1a:D3936*	1	0.03
Maharashtra										ORF1a:N3937R	1	0.03
Maharashtra										ORF1a:R3938N	1	0.03
Maharashtra										ORF1a:T3940G	1	0.03
Maharashtra										ORF1a:L3941Q	1	0.03
Maharashtra										ORF1a:A3943I	1	0.03
Maharashtra										ORF1a:A3945C	1	0.03
Maharashtra										ORF1a:E3947F	1	0.03
Maharashtra										ORF1a:F3948C	1	0.03
Maharashtra										ORF1a:S3950C	1	0.03
Maharashtra										ORF1a:L3951S	1	0.03
Maharashtra										ORF1a:P3952R	1	0.03
Maharashtra										ORF1a:Y3954L	1	0.03
Maharashtra										ORF1a:M4012E	1	0.03
Maharashtra										ORF1a:Q4015S	1	0.03
Maharashtra										ORF1a:A4016Y	1	0.03
Maharashtra										ORF1a:S4018C	1	0.03
Maharashtra										ORF1a:D4020A	1	0.03
Maharashtra										ORF1a:K4021D	1	0.03
Maharashtra										ORF1a:R4022N	1	0.03
Maharashtra										ORF1a:D4041F	1	0.03
Maharashtra										ORF1a:N4042H	1	0.03
Maharashtra										ORF1a:D4043Y	1	0.03
Maharashtra										ORF1a:I4062C	1	0.03
Maharashtra										ORF1a:P4063S	1	0.03
Maharashtra										ORF1a:P4220R	1	0.03
Maharashtra										ORF1a:K4221S	1	0.03
Maharashtra										ORF1a:G4222*	1	0.03
Maharashtra										ORF1a:P4223S	1	0.03
Maharashtra										ORF1a:K4224E	1	0.03
Maharashtra										ORF1a:K4226F	1	0.03
Maharashtra										ORF1a:Y4227I	1	0.03
Maharashtra										ORF1a:F4269A	1	0.03
Maharashtra										ORF1a:A4271Q	1	0.03
Maharashtra										ORF1a:N4338L	1	0.03
Maharashtra										ORF1a:G4341S	1	0.03
Maharashtra										ORF1a:F4342*	1	0.03
Maharashtra										ORF1a:C4343R	1	0.03
Maharashtra										ORF1a:D4344I	1	0.03
Maharashtra										ORF1a:K4346*	1	0.03
Maharashtra										ORF1a:G4347L	1	0.03
Maharashtra										ORF1a:P4353R	1	0.03
Maharashtra										ORF1a:T4354*	1	0.03
Maharashtra										ORF1a:T4355V	1	0.03
Maharashtra										ORF1a:A4357T	1	0.03
Maharashtra										ORF1a:Y1183F	1	0.03
Maharashtra										ORF1a:D1184A	1	0.03
Maharashtra										ORF1a:K1185Q	1	0.03
Maharashtra										ORF1a:L1186M	1	0.03
Maharashtra										ORF1a:V1187S	1	0.03
Maharashtra										ORF1a:S1188T	1	0.03
Maharashtra										ORF1a:S1189*	1	0.03
Maharashtra										ORF1a:F1190L	1	0.03
Maharashtra										ORF1a:L1191F	1	0.03
Maharashtra										ORF1a:E1311R	1	0.03
Maharashtra										ORF1a:M1312Q	1	0.03
Maharashtra										ORF1a:A1314Y	1	0.03
Maharashtra										ORF1a:K1315N	1	0.03
Maharashtra										ORF1a:A1316H	1	0.03
Maharashtra										ORF1a:R1421*	1	0.03
Maharashtra										ORF1a:F1422G	1	0.03
Maharashtra										ORF1a:F1424*	1	0.03
Maharashtra										ORF1a:Y1425N	1	0.03
Maharashtra										ORF1a:K1428V	1	0.03
Maharashtra										ORF1a:T1437N	1	0.03
Maharashtra										ORF1a:L1438A	1	0.03

Maharashtra										**ORF1a:N1439T**	1	0.03
Maharashtra										**ORF1a:D1440W**	1	0.03
Maharashtra										**ORF1a:N1442C**	1	0.03
Maharashtra										**ORF1a:S1468W**	1	0.03
Maharashtra										**ORF1a:S1478Y**	1	0.03
Maharashtra										**ORF1a:S1479E**	1	0.03
Maharashtra										**ORF1a:P1480I**	1	0.03
Maharashtra										**ORF1a:V1483S**	1	0.03
Maharashtra										**ORF1a:T1484A**	1	0.03
Maharashtra										**ORF1a:A1485S**	1	0.03
Maharashtra										**ORF1a:S1515F**	1	0.03
Maharashtra										**ORF1a:G1518***	1	0.03
Maharashtra										**ORF1a:Q1519E**	1	0.03
Maharashtra										**ORF1a:S1520R**	1	0.03
Maharashtra										**ORF1a:T1521***	1	0.03
Maharashtra										**ORF1a:Q1522***	1	0.03
Maharashtra										**ORF1a:L1523K**	1	0.03
Maharashtra										**ORF1a:P1609W**	1	0.03
Maharashtra										**ORF1a:H1610S**	1	0.03
Maharashtra										**ORF1a:Y1831F**	1	0.03
Maharashtra										**ORF1a:Q1832***	1	0.03
Maharashtra										**ORF1a:C1833R**	1	0.03
Maharashtra										**ORF1a:G1834N**	1	0.03
Maharashtra										**ORF1a:H1835F**	1	0.03
Maharashtra										**ORF1a:Y1836V**	1	0.03
Maharashtra										**ORF1a:I1863R**	1	0.03
Maharashtra										**ORF1a:T1864R**	1	0.03
Maharashtra										**ORF1a:D1865C**	1	0.03
Maharashtra										**ORF1a:V1866F**	1	0.03
Maharashtra										**ORF1a:F1867T**	1	0.03
Maharashtra										**ORF1a:E1870K**	1	0.03
Maharashtra										**ORF1a:N1871T**	1	0.03
Maharashtra										**ORF1a:D1897F**	1	0.03
Maharashtra										**ORF1a:Y1899F**	1	0.03
Maharashtra										**ORF1a:P2018Y**	1	0.03
Maharashtra										**ORF1a:E2020V**	1	0.03
Maharashtra										**ORF1a:T2021Y**	1	0.03
Maharashtra										**ORF1a:S2022T**	1	0.03
Maharashtra										**ORF1a:N2023L**	1	0.03
Maharashtra										**ORF1a:F2025L**	1	0.03
Maharashtra										**ORF1a:D2026E**	1	0.03
Maharashtra										**ORF1a:V2027H**	1	0.03
Maharashtra										**ORF1a:L2028K**	1	0.03
Maharashtra										**ORF1a:K2029T**	1	0.03
Maharashtra										**ORF1a:I2057R**	1	0.03
Maharashtra										**ORF1a:Q2058S**	1	0.03
Maharashtra										**ORF1a:D2060T**	1	0.03
Maharashtra										**ORF1a:V2061S**	1	0.03
Maharashtra										**ORF1a:T2310P**	1	0.03
Maharashtra										**ORF1a:S2313L**	1	0.03
Maharashtra										**ORF1a:F2314***	1	0.03
Maharashtra										**ORF1a:W2316N**	1	0.03
Maharashtra										**ORF1a:I2346Q**	1	0.03
Maharashtra										**ORF1a:M2347Q**	1	0.03
Maharashtra										**ORF1a:V2629N**	1	0.03
Maharashtra										**ORF1a:K2798C**	1	0.03
Maharashtra										**ORF1a:H2799L**	1	0.03
Maharashtra										**ORF1a:N2847L**	1	0.03
Maharashtra										**ORF1a:P2867A**	1	0.03
Maharashtra										**ORF1a:G2868Q**	1	0.03
Maharashtra										**ORF1a:P2870M**	1	0.03
Maharashtra										**ORF1a:G2871V**	1	0.03
Maharashtra										**ORF1a:F3604L**	1	0.03
Maharashtra										**ORF1a:G661Q**	1	0.03
Maharashtra										**ORF1a:N810Q**	1	0.03
Maharashtra										**ORF1a:N1059G**	1	0.03
Maharashtra										**ORF1a:A1060K**	1	0.03
Maharashtra										**ORF1a:A1061T**	1	0.03
Maharashtra										**ORF1a:I1091W**	1	0.03
Maharashtra										**ORF1a:A1092T**	1	0.03
Maharashtra										**ORF1a:S2273V**	1	0.03
Maharashtra										**ORF1a:Y3793I**	1	0.03
Maharashtra										**ORF1a:L3873***	1	0.03

Maharashtra										**ORF1a:L3876H**	1	0.03
Maharashtra										**ORF1a:Q3878G**	1	0.03
Maharashtra										**ORF1a:P4058T**	1	0.03
Maharashtra										**ORF1a:D4076***	1	0.03
Maharashtra										**ORF1a:Y4077W**	1	0.03
Maharashtra										**ORF1a:N4078L**	1	0.03
Maharashtra										**ORF1a:T4079S**	1	0.03
Maharashtra										**ORF1a:K4081Q**	1	0.03
Maharashtra										**ORF1a:N4082Q**	1	0.03
Maharashtra										**ORF1a:G49D**	1	0.03
Maharashtra										**ORF1a:E1047A**	1	0.03
Maharashtra										**ORF1a:D1089E**	1	0.03
Maharashtra										**ORF1a:Y1090N**	1	0.03
Maharashtra										**ORF1a:V2324N**	1	0.03
Maharashtra										**ORF1a:A2325G**	1	0.03
Maharashtra										**ORF1a:E2326I**	1	0.03
Maharashtra										**ORF1a:M297Q**	1	0.03
Maharashtra										**ORF1a:R299L**	1	0.03
Maharashtra										**ORF1a:I300K**	1	0.03
Maharashtra										**ORF1a:S302W**	1	0.03
Maharashtra										**ORF1a:A1809S**	1	0.03
Maharashtra										**ORF1a:P1810T**	1	0.03
Maharashtra										**ORF1a:P1811T**	1	0.03
Maharashtra										**ORF1a:A1812C**	1	0.03
Maharashtra										**ORF1a:N449P**	1	0.03
Maharashtra										**ORF1a:L450K**	1	0.03
Maharashtra										**ORF1a:L451R**	1	0.03
Maharashtra										**ORF1a:S485G**	1	0.03
Maharashtra										**ORF1a:A486I**	1	0.03
Maharashtra										**ORF1a:E489C**	1	0.03
Maharashtra										**ORF1a:T490F**	1	0.03
Maharashtra										**ORF1a:V491H**	1	0.03
Maharashtra										**ORF1a:E504***	1	0.03
Maharashtra										**ORF1a:C506L**	1	0.03
Maharashtra										**ORF1a:G507W**	1	0.03
Maharashtra										**ORF1a:I581W**	1	0.03
Maharashtra										**ORF1a:D582N**	1	0.03
Maharashtra										**ORF1a:A583F**	1	0.03
Maharashtra										**ORF1a:M584T**	1	0.03
Maharashtra										**ORF1a:M585V**	1	0.03
Maharashtra										**ORF1a:V605C**	1	0.03
Maharashtra										**ORF1a:V606N**	1	0.03
Maharashtra										**ORF1a:Q607G**	1	0.03
Maharashtra										**ORF1a:T609H**	1	0.03
Maharashtra										**ORF1a:S610Y**	1	0.03
Maharashtra										**ORF1a:Q611R**	1	0.03
Maharashtra										**ORF1a:L613K**	1	0.03
Maharashtra										**ORF1a:K623Q**	1	0.03
Maharashtra										**ORF1a:L624T**	1	0.03
Maharashtra										**ORF1a:K625R**	1	0.03
Maharashtra										**ORF1a:V757S**	1	0.03
Maharashtra										**ORF1a:L758***	1	0.03
Maharashtra										**ORF1a:T814H**	1	0.03
Maharashtra										**ORF1a:K816I**	1	0.03
Maharashtra										**ORF1a:G817***	1	0.03
Maharashtra										**ORF1a:G818W**	1	0.03
Maharashtra										**ORF1a:A819***	1	0.03
Maharashtra										**ORF1a:P820Q**	1	0.03
Maharashtra										**ORF1a:D828C**	1	0.03
Maharashtra										**ORF1a:T829K**	1	0.03
Maharashtra										**ORF1a:I831T**	1	0.03
Maharashtra										**ORF1a:E832R**	1	0.03
Maharashtra										**ORF1a:I849***	1	0.03
Maharashtra										**ORF1a:D850I**	1	0.03
Maharashtra										**ORF1a:E855Q**	1	0.03
Maharashtra										**ORF1a:C857***	1	0.03
Maharashtra										**ORF1a:S858M**	1	0.03
Maharashtra										**ORF1a:A859S**	1	0.03
Maharashtra										**ORF1a:Y906W**	1	0.03
Maharashtra										**ORF1a:F908H**	1	0.03
Maharashtra										**ORF1a:D909I**	1	0.03
Maharashtra										**ORF1a:E910C**	1	0.03
Maharashtra										**ORF1a:S911I**	1	0.03

Maharashtra										**ORF1a:G912V**	1	0.03
Maharashtra										**ORF1a:E942K**	1	0.03
Maharashtra										**ORF1a:W979N**	1	0.03
Maharashtra										**ORF1a:T1001A**	1	0.03
Maharashtra										**ORF1a:I1002V**	1	0.03
Maharashtra										**ORF1a:V1006R**	1	0.03
Maharashtra										**ORF1a:E1007W**	1	0.03
Maharashtra										**ORF1a:V1008N**	1	0.03
Maharashtra										**ORF1a:Q1009L**	1	0.03
Maharashtra										**ORF1a:K1077M**	1	0.03
Maharashtra										**ORF1a:A1078I**	1	0.03
Maharashtra										**ORF1a:N1080***	1	0.03
Maharashtra										**ORF1a:N1081L**	1	0.03
Maharashtra										**ORF1a:H1500I**	1	0.03
Maharashtra										**ORF1a:F1501K**	1	0.03
Maharashtra										**ORF1a:V3925Q**	1	0.03
Maharashtra										**ORF1a:D3926Q**	1	0.03
Maharashtra										**ORF1a:I3927G**	1	0.03
Maharashtra										**ORF1a:K4081L**	1	0.03
Maharashtra										**ORF1a:N4082C**	1	0.03
Maharashtra										**ORF1a:T4083I**	1	0.03
Maharashtra										**ORF1a:C4084S**	1	0.03
Maharashtra										**ORF1a:D4085I**	1	0.03
Maharashtra										**ORF1a:V2276F**	1	0.03
Maharashtra										**ORF1a:V3976F**	1	0.03
Maharashtra										**ORF1a:R402C**	1	0.03
Maharashtra										**ORF1a:K1230N**	1	0.03
Maharashtra										**ORF1a:N1540T**	1	0.03
Maharashtra										**ORF1a:N375D**	1	0.03
Maharashtra										**ORF1a:S3983F**	1	0.03
Maharashtra										**ORF1a:H2659Q**	1	0.03
Maharashtra										**ORF1a:D294G**	1	0.03
Maharashtra										**ORF1a:G59N**	1	0.03
Maharashtra										**ORF1a:L61F**	1	0.03
Maharashtra										**ORF1a:P62F**	1	0.03
Maharashtra										**ORF1a:F70S**	1	0.03
Maharashtra										**ORF1a:I71T**	1	0.03
Maharashtra										**ORF1a:H3435C**	1	0.03
Maharashtra										**ORF1a:G3437S**	1	0.03
Maharashtra										**ORF1a:T3438M**	1	0.03
Maharashtra										**ORF1a:D3439N**	1	0.03
Maharashtra										**ORF1a:A3457V**	1	0.03
Maharashtra										**ORF1a:T3459N**	1	0.03
Maharashtra										**ORF1a:T3462M**	1	0.03
Maharashtra										**ORF1a:I3463V**	1	0.03
Maharashtra										**ORF1a:K4281S**	1	0.03
Maharashtra										**ORF1a:Y4283W**	1	0.03
Maharashtra										**ORF1a:A4285T**	1	0.03
Maharashtra										**ORF1a:P4290I**	1	0.03
Maharashtra										**ORF1a:T4302F**	1	0.03
Maharashtra										**ORF1a:G4303V**	1	0.03
Maharashtra										**ORF1a:T4304S**	1	0.03
Maharashtra										**ORF1a:G4305F**	1	0.03
Maharashtra										**ORF1a:Q4306S**	1	0.03
Maharashtra										**ORF1a:A4307S**	1	0.03
Maharashtra										**ORF1a:P4312A**	1	0.03
Maharashtra										**ORF1a:E4313K**	1	0.03
Maharashtra										**ORF1a:M4316T**	1	0.03
Maharashtra										**ORF1a:D4317V**	1	0.03
Maharashtra										**ORF1a:Q4318E**	1	0.03
Maharashtra										**ORF1a:Y4329H**	1	0.03
Maharashtra										**ORF1a:C4330F**	1	0.03
Maharashtra										**ORF1a:R4331C**	1	0.03
Maharashtra										**ORF1a:V649I**	1	0.03
Maharashtra										**ORF1a:V1798L**	1	0.03
Maharashtra										**ORF1a:D75G**	1	0.03
Maharashtra										**ORF1a:P80A**	1	0.03
Maharashtra										**ORF1a:H81T**	1	0.03
Maharashtra										**ORF1a:H83S**	1	0.03
Maharashtra										**ORF1a:V84F**	1	0.03
Maharashtra										**ORF1a:M85I**	1	0.03
Maharashtra										**ORF1a:V86T**	1	0.03
Maharashtra										**ORF1a:E87V**	1	0.03

Maharashtra										**ORF1a:V89T**	1	0.03
Maharashtra										**ORF1a:A90K**	1	0.03
Maharashtra										**ORF1a:G98E**	1	0.03
Maharashtra										**ORF1a:R99P**	1	0.03
Maharashtra										**ORF1a:S100C**	1	0.03
Maharashtra										**ORF1a:T103P**	1	0.03
Maharashtra										**ORF1a:V111E**	1	0.03
Maharashtra										**ORF1a:E113Q**	1	0.03
Maharashtra										**ORF1a:I114G**	1	0.03
Maharashtra										**ORF1a:P115Q**	1	0.03
Maharashtra										**ORF1a:A117S**	1	0.03
Maharashtra										**ORF1a:Y118H**	1	0.03
Maharashtra										**ORF1a:R119H**	1	0.03
Maharashtra										**ORF1a:K120Y**	1	0.03
Maharashtra										**ORF1a:D139V**	1	0.03
Maharashtra										**ORF1a:K141N**	1	0.03
Maharashtra										**ORF1a:L145F**	1	0.03
Maharashtra										**ORF1a:G146C**	1	0.03
Maharashtra										**ORF1a:D147Y**	1	0.03
Maharashtra										**ORF1a:E148Q**	1	0.03
Maharashtra										**ORF1a:T183N**	1	0.03
Maharashtra										**ORF1a:R1628C**	1	0.03
Maharashtra										**ORF1a:C270F**	1	0.03
Maharashtra										**ORF1a:K291T**	1	0.03
Maharashtra										**ORF1a:I3306L**	1	0.03
Maharashtra										**ORF1a:H1580S**	1	0.03
Maharashtra										**ORF1a:Q1582R**	1	0.03
Maharashtra										**ORF1a:V1583K**	1	0.03
Maharashtra										**ORF1a:V1584L**	1	0.03
Maharashtra										**ORF1a:D1585W**	1	0.03
Maharashtra										**ORF1a:M1586T**	1	0.03
Maharashtra										**ORF1a:Y2270C**	1	0.03
Maharashtra										**ORF1a:S2273T**	1	0.03
Maharashtra										**ORF1a:T2274V**	1	0.03
Maharashtra										**ORF1a:R3542W**	1	0.03
Maharashtra										**ORF1a:V4260M**	1	0.03
Maharashtra										**ORF1a:C1114F**	1	0.03
Maharashtra										**ORF1a:T1874I**	1	0.03
Maharashtra										**ORF1a:L454I**	1	0.03
Maharashtra										**ORF1a:L624I**	1	0.03
Maharashtra										**ORF1a:V3057L**	1	0.03
Maharashtra										**ORF1a:P3200S**	1	0.03
Maharashtra										**ORF1a:L3198P**	1	0.03
Maharashtra										**ORF1a:Y3211***	1	0.03
Maharashtra										**ORF1a:K3213H**	1	0.03
Maharashtra										**ORF1a:Y3214S**	1	0.03
Maharashtra										**ORF1a:K3215A**	1	0.03
Maharashtra										**ORF1a:Y3226***	1	0.03
Maharashtra										**ORF1a:R3227G**	1	0.03
Maharashtra										**ORF1a:E3228D**	1	0.03
Maharashtra										**ORF1a:A3229R**	1	0.03
Maharashtra										**ORF1a:A3230G**	1	0.03
Maharashtra										**ORF1a:C3231L**	1	0.03
Maharashtra										**ORF1a:C3232W**	1	0.03
Maharashtra										**ORF1a:H3233W**	1	0.03
Maharashtra										**ORF1a:A3235V**	1	0.03
Maharashtra										**ORF1a:D3240N**	1	0.03
Maharashtra										**ORF1a:S3242R**	1	0.03
Maharashtra										**ORF1a:S3244***	1	0.03
Maharashtra										**ORF1a:D3247E**	1	0.03
Maharashtra										**ORF1a:L3249I**	1	0.03
Maharashtra										**ORF1a:G3278P**	1	0.03
Maharashtra										**ORF1a:H1580Y**	1	0.03
Maharashtra										**ORF1a:E867D**	1	0.03
Maharashtra										**ORF1a:F8L**	1	0.03
Maharashtra										**ORF1a:I1153V**	1	0.03
Maharashtra										**ORF1a:K3363N**	1	0.03
Maharashtra										**ORF1a:Y2266N**	1	0.03
Maharashtra										**ORF1a:L733***	1	0.03
Maharashtra										**ORF1a:E4097K**	1	0.03
Maharashtra										**ORF1a:I2359S**	1	0.03
Maharashtra										**ORF1a:S2360M**	1	0.03
Maharashtra										**ORF1a:I2190T**	1	0.03

Maharashtra										**ORF1a:G2265I**	1	0.03
Maharashtra										**ORF1a:W2366M**	1	0.03
Maharashtra										**ORF1a:S248R**	1	0.03
Maharashtra										**ORF1a:I1159T**	1	0.03
Maharashtra										**ORF1a:A859V**	1	0.03
Maharashtra										**ORF1a:R1163G**	1	0.03
Maharashtra										**ORF1a:L2503F**	1	0.03
Maharashtra										**ORF1a:I95T**	1	0.03
Maharashtra										**ORF1a:L3915F**	1	0.03
Maharashtra										**ORF1a:K394Q**	1	0.03
Maharashtra										**ORF1a:T333M**	1	0.03
Maharashtra										**ORF1a:A3730V**	1	0.03
Maharashtra										**ORF1a:C1103R**	1	0.03
Maharashtra										**ORF1a:C2826***	1	0.03
Maharashtra										**ORF1a:A2618S**	1	0.03
Maharashtra										**ORF1a:S2911P**	1	0.03
Maharashtra										**ORF1a:K3351R**	1	0.03
Maharashtra										**ORF1a:K225N**	1	0.03
Maharashtra										**ORF1a:G392V**	1	0.03
Maharashtra										**ORF1a:I3148V**	1	0.03
Maharashtra										**ORF1a:L2874S**	1	0.03
Maharashtra										**ORF1a:I695N**	1	0.03
Maharashtra										**ORF1a:V410M**	1	0.03
Maharashtra										**ORF1a:V511I**	1	0.03
Maharashtra										**ORF1a:G2241S**	1	0.03
Maharashtra										**ORF1a:P1977S**	1	0.03
Maharashtra										**ORF1a:T2121I**	1	0.03
Maharashtra										**ORF1a:I693T**	1	0.03
Maharashtra										**ORF1a:L16T**	1	0.03
Maharashtra										**ORF1a:M1466H**	1	0.03
Maharashtra										**ORF1a:Y3803C**	1	0.03
Maharashtra										**ORF1a:G298S**	1	0.03
Maharashtra										**ORF1a:T4088A**	1	0.03
Maharashtra										**ORF1a:T1246I**	1	0.03
Maharashtra										**ORF1a:P804S**	1	0.03
Maharashtra										**ORF1a:T2264K**	1	0.03
Maharashtra										**ORF1a:G2265N**	1	0.03
Maharashtra										**ORF1a:A3497S**	1	0.03
Maharashtra										**ORF1a:Y2413C**	1	0.03
Maharashtra										**ORF1a:S167N**	1	0.03
Maharashtra										**ORF1a:S610L**	1	0.03
Maharashtra										**ORF1a:T3868I**	1	0.03
Maharashtra										**ORF1a:R2638Q**	1	0.03
Maharashtra										**ORF1a:A3523V**	1	0.03
Maharashtra										**ORF1a:A239V**	1	0.03
Maharashtra										**ORF1a:I300R**	1	0.03
Maharashtra										**ORF1a:R301K**	1	0.03
Maharashtra										**ORF1a:A486L**	1	0.03
Maharashtra										**ORF1a:D1088H**	1	0.03
Maharashtra										**ORF1a:D1089S**	1	0.03
Maharashtra										**ORF1a:I1091***	1	0.03
Maharashtra										**ORF1a:A1092W**	1	0.03
Maharashtra										**ORF1a:L1149R**	1	0.03
Maharashtra										**ORF1a:A1151Y**	1	0.03
Maharashtra										**ORF1a:G1152T**	1	0.03
Maharashtra										**ORF1a:H1160K**	1	0.03
Maharashtra										**ORF1a:L1375R**	1	0.03
Maharashtra										**ORF1a:E1377N**	1	0.03
Maharashtra										**ORF1a:M1378T**	1	0.03
Maharashtra										**ORF1a:L1379Q**	1	0.03
Maharashtra										**ORF1a:A1380I**	1	0.03
Maharashtra										**ORF1a:H1381N**	1	0.03
Maharashtra										**ORF1a:V1416***	1	0.03
Maharashtra										**ORF1a:D1417G**	1	0.03
Maharashtra										**ORF1a:G1419Q**	1	0.03
Maharashtra										**ORF1a:D1513F**	1	0.03
Maharashtra										**ORF1a:G1524T**	1	0.03
Maharashtra										**ORF1a:E1526C**	1	0.03
Maharashtra										**ORF1a:F1527I**	1	0.03
Maharashtra										**ORF1a:G1595I**	1	0.03
Maharashtra										**ORF1a:E1614***	1	0.03
Maharashtra										**ORF1a:G1615N**	1	0.03
Maharashtra										**ORF1a:F1618S**	1	0.03

Maharashtra										**ORF1a:Y1619***	1	0.03
Maharashtra										**ORF1a:V1620F**	1	0.03
Maharashtra										**ORF1a:L1621T**	1	0.03
Maharashtra										**ORF1a:P1622***	1	0.03
Maharashtra										**ORF1a:N1623R**	1	0.03
Maharashtra										**ORF1a:D1624***	1	0.03
Maharashtra										**ORF1a:D1625N**	1	0.03
Maharashtra										**ORF1a:T1626I**	1	0.03
Maharashtra										**ORF1a:R1628***	1	0.03
Maharashtra										**ORF1a:T1773R**	1	0.03
Maharashtra										**ORF1a:L1774C**	1	0.03
Maharashtra										**ORF1a:K1795L**	1	0.03
Maharashtra										**ORF1a:Q1799W**	1	0.03
Maharashtra										**ORF1a:Q1800***	1	0.03
Maharashtra										**ORF1a:E1801T**	1	0.03
Maharashtra										**ORF1a:P1803C**	1	0.03
Maharashtra										**ORF1a:I694S**	1	0.03
Maharashtra										**ORF1a:K4281N**	1	0.03
Maharashtra										**ORF1a:N1324I**	1	0.03
Maharashtra										**ORF1a:I3052V**	1	0.03
Maharashtra										**ORF1a:Q1140R**	1	0.03
Maharashtra										**ORF1a:S3075N**	1	0.03
Maharashtra										**ORF1a:G445C**	1	0.03
Maharashtra										**ORF1a:A1234V**	1	0.03
Maharashtra										**ORF1a:T2181A**	1	0.03
Maharashtra										**ORF1a:S2224F**	1	0.03
Maharashtra										**ORF1a:K714N**	1	0.03
Maharashtra										**ORF1a:F1779V**	1	0.03
Maharashtra										**ORF1a:E725K**	1	0.03
Maharashtra										**ORF1a:L890F**	1	0.03
Maharashtra										**ORF1a:C2239Y**	1	0.03
Maharashtra										**ORF1a:A2142V**	1	0.03
Maharashtra										**ORF1a:D2043N**	1	0.03
Maharashtra										**ORF1a:C3419R**	1	0.03
Maharashtra										**ORF1a:M3527I**	1	0.03
Maharashtra										**ORF1a:T602I**	1	0.03
Maharashtra										**ORF1a:P361L**	1	0.03
Maharashtra										**ORF1a:D1282Y**	1	0.03
Maharashtra										**ORF1a:I2231N**	1	0.03
Maharashtra										**ORF1a:S1666N**	1	0.03
Maharashtra										**ORF1a:L1375F**	1	0.03
Maharashtra										**ORF1a:E913G**	1	0.03
Maharashtra										**ORF1a:G957S**	1	0.03
Maharashtra										**ORF1a:D1229A**	1	0.03
Maharashtra										**ORF1a:R398C**	1	0.03
Maharashtra										**ORF1a:H712L**	1	0.03
Maharashtra										**ORF1a:S713T**	1	0.03
Maharashtra										**ORF1a:Y717F**	1	0.03
Maharashtra										**ORF1a:R718***	1	0.03
Maharashtra										**ORF1a:K719W**	1	0.03
Maharashtra										**ORF1a:L733C**	1	0.03
Maharashtra										**ORF1a:T3791S**	1	0.03
Maharashtra										**ORF1a:L716F**	1	0.03
Maharashtra										**ORF1a:Y717S**	1	0.03
Maharashtra										**ORF1a:R718F**	1	0.03
Maharashtra										**ORF1a:K719T**	1	0.03
Maharashtra										**ORF1a:C720S**	1	0.03
Maharashtra										**ORF1a:L729C**	1	0.03
Maharashtra										**ORF1a:L730S**	1	0.03
Maharashtra										**ORF1a:M731K**	1	0.03
Maharashtra										**ORF1a:M3684I**	1	0.03
Maharashtra										**ORF1a:H81R**	1	0.03
Maharashtra										**ORF1a:T2093I**	1	0.03
Maharashtra										**ORF1a:M2796I**	1	0.03
Maharashtra										**ORF1a:S901I**	1	0.03
Maharashtra										**ORF1a:C1349S**	1	0.03
Maharashtra										**ORF1a:T3287I**	1	0.03
Maharashtra										**ORF1a:T770I**	1	0.03
Maharashtra										**ORF1a:C3766F**	1	0.03
Maharashtra										**ORF1a:T965I**	1	0.03
Maharashtra										**ORF1a:M2347E**	1	0.03
Maharashtra										**ORF1a:Q2348H**	1	0.03
Maharashtra										**ORF1a:D139N**	1	0.03

Maharashtra										**ORF1a:V1020F**	1	0.03
Maharashtra										**ORF1a:K1929N**	1	0.03
Maharashtra										**ORF1a:V4101F**	1	0.03
Maharashtra										**ORF1a:I1863V**	1	0.03
Maharashtra										**ORF1a:S34F**	1	0.03
Maharashtra										**ORF1a:E755K**	1	0.03
Maharashtra										**ORF1a:R2336M**	1	0.03
Maharashtra										**ORF1a:L2333I**	1	0.03
Maharashtra										**ORF1a:F2334L**	1	0.03
Maharashtra										**ORF1a:T2335M**	1	0.03
Maharashtra										**ORF1a:R2336W**	1	0.03
Maharashtra										**ORF1a:S2695N**	1	0.03
Maharashtra										**ORF1a:P380T**	1	0.03
Maharashtra										**ORF1a:P885S**	1	0.03
Maharashtra										**ORF1a:G2207S**	1	0.03
Maharashtra										**ORF1a:T1004A**	1	0.03
Maharashtra										**ORF1a:T4161I**	1	0.03
Maharashtra										**ORF1a:G728S**	1	0.03
Maharashtra										**ORF1a:I1259V**	1	0.03
Maharashtra										**ORF1a:T1761N**	1	0.03
Maharashtra										**ORF1a:L2620I**	1	0.03
Maharashtra										**ORF1a:L3799V**	1	0.03
Maharashtra										**ORF1a:D930Y**	1	0.03
Maharashtra										**ORF1a:K1396N**	1	0.03
Maharashtra										**ORF1a:A3357V**	1	0.03
Maharashtra										**ORF1a:P2739L**	1	0.03
Maharashtra										**ORF1a:K719N**	1	0.03
Maharashtra										**ORF1a:C720L**	1	0.03
Maharashtra										**ORF1a:V2130A**	1	0.03
Maharashtra										**ORF1a:A4136V**	1	0.03
Maharashtra										**ORF1a:F2354S**	1	0.03
Maharashtra										**ORF1a:L628F**	1	0.03
Maharashtra										**ORF1a:V3449F**	1	0.03
Maharashtra										**ORF1a:Y1465C**	1	0.03
Maharashtra										**ORF1a:G4378V**	1	0.03
Maharashtra										**ORF1a:T1754I**	1	0.03
Maharashtra										**ORF1a:M3655I**	1	0.03
Maharashtra										**ORF1a:G661S**	1	0.03
Maharashtra										**ORF1a:I3731T**	1	0.03
Maharashtra										**ORF1a:Y3722C**	1	0.03
Maharashtra										**ORF1a:A1204T**	1	0.03
Maharashtra										**ORF1a:F1553V**	1	0.03
Maharashtra										**ORF1a:G638S**	1	0.03
Maharashtra										**ORF1a:S3916A**	1	0.03
Maharashtra										**ORF1a:A917T**	1	0.03
Maharashtra										**ORF1a:E948D**	1	0.03
Maharashtra										**ORF1a:D2101Y**	1	0.03
Maharashtra										**ORF1a:A2279P**	1	0.03
Maharashtra										**ORF1a:A903V**	1	0.03
Maharashtra										**ORF1a:I1301L**	1	0.03
Maharashtra										**ORF1a:H134R**	1	0.03
Maharashtra										**ORF1a:E972K**	1	0.03
Maharashtra										**ORF1a:K338R**	1	0.03
Maharashtra										**ORF1a:A339V**	1	0.03
Maharashtra										**ORF1a:G604C**	1	0.03
Maharashtra										**ORF1a:A2636V**	1	0.03
Maharashtra										**ORF1a:V3968F**	1	0.03
Maharashtra										**ORF1a:A2279E**	1	0.03
Maharashtra										**ORF1a:F2358S**	1	0.03
Maharashtra										**ORF1a:G696Y**	1	0.03
Maharashtra										**ORF1a:I1286M**	1	0.03
Maharashtra										**ORF1a:T2967I**	1	0.03
Maharashtra										**ORF1a:T882I**	1	0.03
Maharashtra										**ORF1a:A2690V**	1	0.03
Maharashtra										**ORF1a:E1724D**	1	0.03
Maharashtra										**ORF1a:L4391F**	1	0.03
Maharashtra										**ORF1a:V3298I**	1	0.03
Maharashtra										**ORF1a:R2949L**	1	0.03
Maharashtra										**ORF1a:M3065I**	1	0.03
Maharashtra										**ORF1a:F3067T**	1	0.03
Maharashtra										**ORF1a:R3068K**	1	0.03
Maharashtra										**ORF1a:R3069K**	1	0.03
Maharashtra										**ORF1a:K1895T**	1	0.03

Maharashtra										**ORF1a:N1262S**	1	0.03
Maharashtra										**ORF1a:A486T**	1	0.03
Maharashtra										**ORF1a:T4354I**	1	0.03
Maharashtra										**ORF1a:E441G**	1	0.03
Maharashtra										**ORF1a:G3827V**	1	0.03
Maharashtra										**ORF1a:N1871S**	1	0.03
Maharashtra										**ORF1a:M297I**	1	0.03
Maharashtra										**ORF1a:V439F**	1	0.03
Maharashtra										**ORF1a:V3349I**	1	0.03
Maharashtra										**ORF1a:E633D**	1	0.03
Maharashtra										**ORF1a:V86F**	1	0.03
Maharashtra										**ORF1a:G696S**	1	0.03
Maharashtra										**ORF1a:P2685S**	1	0.03
Maharashtra										**ORF1a:P971S**	1	0.03
Maharashtra										**ORF1a:Y2489H**	1	0.03
Maharashtra										**ORF1a:T2174A**	1	0.03
Maharashtra										**ORF1a:C2210F**	1	0.03
Maharashtra										**ORF1a:V2072I**	1	0.03
Maharashtra										**ORF1a:T3182N**	1	0.03
Maharashtra										**ORF1a:S74L**	1	0.03
Maharashtra										**ORF1a:N313K**	1	0.03
Maharashtra										**ORF1a:A1380S**	1	0.03
Maharashtra										**ORF1a:I3618V**	1	0.03
Maharashtra										**ORF1a:P782L**	1	0.03
Maharashtra										**ORF1a:T1250I**	1	0.03
Maharashtra										**ORF1a:S3564A**	1	0.03
Maharashtra										**ORF1a:D1275N**	1	0.03
Maharashtra										**ORF1a:K500T**	1	0.03
Maharashtra										**ORF1a:K1208E**	1	0.03
Maharashtra										**ORF1a:K1077N**	1	0.03
Maharashtra										**ORF1a:S1517F**	1	0.03
Maharashtra										**ORF1a:P193S**	1	0.03
Maharashtra										**ORF1a:K2200N**	1	0.03
Maharashtra										**ORF1a:Y3500C**	1	0.03
Maharashtra										**ORF1a:L2084I**	1	0.03
Maharashtra										**ORF1a:T484I**	1	0.03
Maharashtra										**ORF1a:D2592G**	1	0.03
Maharashtra										**ORF1a:L360V**	1	0.03
Maharashtra										**ORF1a:G1100S**	1	0.03
Maharashtra										**ORF1a:Y3765C**	1	0.03
Maharashtra										**ORF1a:T2906S**	1	0.03
Maharashtra										**ORF1a:A1923T**	1	0.03
Maharashtra										**ORF1a:D1624Y**	1	0.03
Maharashtra										**ORF1a:V1583N**	1	0.03
Maharashtra										**ORF1a:I3731F**	1	0.03
Maharashtra										**ORF1a:S3732V**	1	0.03
Maharashtra										**ORF1a:N2201I**	1	0.03
Maharashtra										**ORF1a:G2241M**	1	0.03
Maharashtra										**ORF1a:S2242P**	1	0.03
Maharashtra										**ORF1a:L3694F**	1	0.03
Maharashtra										**ORF1a:D4200G**	1	0.03
Maharashtra										**ORF1a:I1551T**	1	0.03
	**ORF1b**											
**Maharashtra**	**Pre-Wave 1**			**Wave 1**			**Pre-Wave 2**			**Wave 2**		
Maharashtra	**Mutation**	**Count**	**percent**	**Mutation**	**Count**	**percent**	**Mutation**	**Count**	**percent**	**Mutation**	**Count**	**percent**
Maharashtra	**ORF1b:P314L**	599	87.32	**ORF1b:P314L**	763	97.45	**ORF1b:P314L**	559	85.21	**ORF1b:P314L**	3377	95.45
Maharashtra	**ORF1b:A88V**	45	6.56	**ORF1b:A2306V**	73	9.32	**ORF1b:M1352I**	192	29.27	**ORF1b:K2310R**	1628	46.01
Maharashtra	**ORF1b:A2132V**	12	1.75	**ORF1b:A2132V**	42	5.36	**ORF1b:K2310R**	190	28.96	**ORF1b:M1352I**	1508	42.62
Maharashtra	**ORF1b:V2287I**	11	1.6	**ORF1b:Q813H**	22	2.81	**ORF1b:S2312A**	134	20.43	**ORF1b:G662S**	1062	30.02
Maharashtra	**ORF1b:I214M**	9	1.31	**ORF1b:L2523F**	12	1.53	**ORF1b:G1129C**	120	18.29	**ORF1b:P1000L**	1061	29.99
Maharashtra	**ORF1b:K1873N**	8	1.17	**ORF1b:P2394T**	12	1.53	**ORF1b:L2523F**	111	16.92	**ORF1b:S2312A**	787	22.24
Maharashtra	**ORF1b:H1087Y**	6	0.87	**ORF1b:Y2276C**	11	1.4	**ORF1b:P800R**	84	12.8	**ORF1b:G1129C**	691	19.53
Maharashtra	**ORF1b:M747I**	6	0.87	**ORF1b:V2475G**	10	1.28	**ORF1b:C804G**	57	8.69	**ORF1b:A1918V**	358	10.12
Maharashtra	**ORF1b:V1961F**	6	0.87	**ORF1b:P2477A**	9	1.15	**ORF1b:G799R**	55	8.38	**ORF1b:H2285Y**	140	3.96
Maharashtra	**ORF1b:P1325S**	6	0.87	**ORF1b:T797I**	8	1.02	**ORF1b:V2475G**	38	5.79	**ORF1b:P2116S**	135	3.82
Maharashtra	**ORF1b:G2191C**	5	0.73	**ORF1b:H1087Y**	8	1.02	**ORF1b:H801D**	29	4.42	**ORF1b:K469N**	125	3.53
Maharashtra	**ORF1b:T1529A**	5	0.73	**ORF1b:V596A**	7	0.89	**ORF1b:P2116S**	25	3.81	**ORF1b:P1570L**	115	3.25
Maharashtra	**ORF1b:P2477A**	5	0.73	**ORF1b:P2169S**	7	0.89	**ORF1b:Q1393L**	23	3.51	**ORF1b:T2490M**	109	3.08
Maharashtra	**ORF1b:P2256S**	4	0.58	**ORF1b:I2687V**	7	0.89	**ORF1b:A1428V**	23	3.51	**ORF1b:L2523F**	100	2.83
Maharashtra	**ORF1b:K2557R**	4	0.58	**ORF1b:E1079D**	7	0.89	**ORF1b:T1545A**	18	2.74	**ORF1b:V666I**	88	2.49

Maharashtra	**ORF1b:M1156I**	4	0.58	**ORF1b:K2557R**	6	0.77	**ORF1b:F803I**	16	2.44	**ORF1b:T1050N**	74	2.09
Maharashtra	**ORF1b:V596A**	4	0.58	**ORF1b:H2388Y**	6	0.77	**ORF1b:A2306V**	15	2.29	**ORF1b:T1545A**	72	2.04
Maharashtra	**ORF1b:S638I**	4	0.58	**ORF1b:M1156I**	6	0.77	**ORF1b:T2490M**	14	2.13	**ORF1b:M1839I**	66	1.87
Maharashtra	**ORF1b:R2257G**	4	0.58	**ORF1b:D1869Y**	6	0.77	**ORF1b:K1828N**	9	1.37	**ORF1b:K2557R**	36	1.02
Maharashtra	**ORF1b:K1812R**	4	0.58	**ORF1b:S638I**	6	0.77	**ORF1b:T1050N**	8	1.22	**ORF1b:K1507R**	30	0.85
Maharashtra	**ORF1b:V2117L**	4	0.58	**ORF1b:G2191C**	6	0.77	**ORF1b:T2163I**	8	1.22	**ORF1b:K1383R**	27	0.76
Maharashtra	**ORF1b:G2207A**	4	0.58	**ORF1b:M1573I**	5	0.64	**ORF1b:K2557R**	8	1.22	**ORF1b:G2337S**	27	0.76
Maharashtra	**ORF1b:A370V**	4	0.58	**ORF1b:I214M**	5	0.64	**ORF1b:L1504F**	7	1.07	**ORF1b:L1504F**	24	0.68
Maharashtra	**ORF1b:G1217S**	4	0.58	**ORF1b:L1504F**	5	0.64	**ORF1b:T1050I**	7	1.07	**ORF1b:P800R**	23	0.65
Maharashtra	**ORF1b:M2667I**	4	0.58	**ORF1b:P864S**	5	0.64	**ORF1b:L2577F**	7	1.07	**ORF1b:A1291S**	23	0.65
Maharashtra	**ORF1b:V2493I**	4	0.58	**ORF1b:I2273V**	5	0.64	**ORF1b:D2142Y**	7	1.07	**ORF1b:P314F**	20	0.57
Maharashtra	**ORF1b:Q2070R**	3	0.44	**ORF1b:N1962S**	5	0.64	**ORF1b:L796H**	6	0.91	**ORF1b:Q1393L**	19	0.54
Maharashtra	**ORF1b:V1149L**	3	0.44	**ORF1b:A2143V**	4	0.51	**ORF1b:S1089L**	5	0.76	**ORF1b:T634I**	18	0.51
Maharashtra	**ORF1b:R1315C**	3	0.44	**ORF1b:T310I**	4	0.51	**ORF1b:S1182L**	5	0.76	**ORF1b:I1002V**	18	0.51
Maharashtra	**ORF1b:G765C**	3	0.44	**ORF1b:H883Y**	4	0.51	**ORF1b:P218L**	5	0.76	**ORF1b:A2306V**	17	0.48
Maharashtra	**ORF1b:V2223L**	3	0.44	**ORF1b:T1404M**	4	0.51	**ORF1b:P2477A**	5	0.76	**ORF1b:T1050I**	16	0.45
Maharashtra	**ORF1b:H247Y**	3	0.44	**ORF1b:T393I**	4	0.51	**ORF1b:T797A**	5	0.76	**ORF1b:A1895T**	14	0.4
Maharashtra	**ORF1b:P2256L**	3	0.44	**ORF1b:T2165M**	4	0.51	**ORF1b:A2168V**	5	0.76	**ORF1b:V579L**	14	0.4
Maharashtra	**ORF1b:K709N**	3	0.44	**ORF1b:M747I**	3	0.38	**ORF1b:A1219S**	5	0.76	**ORF1b:Q2226H**	14	0.4
Maharashtra	**ORF1b:V1133I**	2	0.29	**ORF1b:A2585S**	3	0.38	**ORF1b:K1383R**	5	0.76	**ORF1b:T1336I**	14	0.4
Maharashtra	**ORF1b:T1511I**	2	0.29	**ORF1b:M1397I**	3	0.38	**ORF1b:T1555I**	5	0.76	**ORF1b:H1087Y**	13	0.37
Maharashtra	**ORF1b:M2414V**	2	0.29	**ORF1b:G1772V**	3	0.38	**ORF1b:I235T**	5	0.76	**ORF1b:V1271L**	13	0.37
Maharashtra	**ORF1b:A1476V**	2	0.29	**ORF1b:K2160N**	3	0.38	**ORF1b:V666I**	5	0.76	**ORF1b:A86S**	13	0.37
Maharashtra	**ORF1b:G2436C**	2	0.29	**ORF1b:D2270Y**	3	0.38	**ORF1b:S638T**	4	0.61	**ORF1b:H604Y**	11	0.31
Maharashtra	**ORF1b:A2306V**	2	0.29	**ORF1b:P825L**	3	0.38	**ORF1b:K2224N**	4	0.61	**ORF1b:V1493L**	11	0.31
Maharashtra	**ORF1b:A520S**	2	0.29	**ORF1b:T2488M**	3	0.38	**ORF1b:V1407F**	4	0.61	**ORF1b:T1511I**	10	0.28
Maharashtra	**ORF1b:V2061L**	2	0.29	**ORF1b:M1839I**	3	0.38	**ORF1b:A1831T**	4	0.61	**ORF1b:S1089L**	10	0.28
Maharashtra	**ORF1b:A1868V**	2	0.29	**ORF1b:Q1393L**	3	0.38	**ORF1b:G662S**	4	0.61	**ORF1b:V2223L**	10	0.28
Maharashtra	**ORF1b:D2666E**	2	0.29	**ORF1b:A1428V**	3	0.38	**ORF1b:P1000L**	4	0.61	**ORF1b:K2111T**	10	0.28
Maharashtra	**ORF1b:L796F**	2	0.29	**ORF1b:M1596I**	3	0.38	**ORF1b:R1517K**	4	0.61	**ORF1b:M1596I**	10	0.28
Maharashtra	**ORF1b:N1426K**	2	0.29	**ORF1b:M1582I**	3	0.38	**ORF1b:N1776V**	4	0.61	**ORF1b:A1428V**	10	0.28
Maharashtra	**ORF1b:V767E**	2	0.29	**ORF1b:E2221Q**	3	0.38	**ORF1b:A1884N**	4	0.61	**ORF1b:V1538L**	9	0.25
Maharashtra	**ORF1b:D2472Y**	2	0.29	**ORF1b:K82R**	3	0.38	**ORF1b:Y1344H**	4	0.61	**ORF1b:A2431V**	9	0.25
Maharashtra	**ORF1b:A1219S**	2	0.29	**ORF1b:A88V**	3	0.38	**ORF1b:K2160N**	4	0.61	**ORF1b:P1095S**	9	0.25
Maharashtra	**ORF1b:I1690L**	2	0.29	**ORF1b:A1443V**	3	0.38	**ORF1b:G1667R**	3	0.46	**ORF1b:T1162I**	9	0.25
Maharashtra	**ORF1b:P2648S**	2	0.29	**ORF1b:P976L**	3	0.38	**ORF1b:S2198I**	3	0.46	**ORF1b:T1540I**	8	0.23
Maharashtra	**ORF1b:T1019I**	2	0.29	**ORF1b:L2577F**	3	0.38	**ORF1b:S2363F**	3	0.46	**ORF1b:D2142Y**	8	0.23
Maharashtra	**ORF1b:P2169S**	2	0.29	**ORF1b:A1521V**	3	0.38	**ORF1b:R2613N**	3	0.46	**ORF1b:A1033S**	8	0.23
Maharashtra	**ORF1b:G814C**	2	0.29	**ORF1b:D2090Y**	3	0.38	**ORF1b:F1741L**	3	0.46	**ORF1b:V980F**	8	0.23
Maharashtra	**ORF1b:K50N**	2	0.29	**ORF1b:L1681F**	3	0.38	**ORF1b:M2269I**	3	0.46	**ORF1b:V1407F**	8	0.23
Maharashtra	**ORF1b:P2531S**	2	0.29	**ORF1b:D144Y**	3	0.38	**ORF1b:C1734***	3	0.46	**ORF1b:L796H**	8	0.23
Maharashtra	**ORF1b:H604Y**	2	0.29	**ORF1b:I235T**	3	0.38	**ORF1b:M1839I**	3	0.46	**ORF1b:P2313L**	8	0.23
Maharashtra	**ORF1b:V839I**	2	0.29	**ORF1b:K469N**	2	0.26	**ORF1b:A241V**	3	0.46	**ORF1b:D1848Y**	8	0.23
Maharashtra	**ORF1b:A397V**	1	0.15	**ORF1b:R2257S**	2	0.26	**ORF1b:V2178F**	3	0.46	**ORF1b:V1961L**	8	0.23
Maharashtra	**ORF1b:T1137I**	1	0.15	**ORF1b:K1812R**	2	0.26	**ORF1b:V345L**	3	0.46	**ORF1b:N1962L**	8	0.23
Maharashtra	**ORF1b:L886***	1	0.15	**ORF1b:V2086F**	2	0.26	**ORF1b:E802D**	3	0.46	**ORF1b:S2379L**	8	0.23
Maharashtra	**ORF1b:L1248F**	1	0.15	**ORF1b:N1919S**	2	0.26	**ORF1b:L966F**	3	0.46	**ORF1b:A545V**	8	0.23
Maharashtra	**ORF1b:K2063N**	1	0.15	**ORF1b:V329I**	2	0.26	**ORF1b:P1570L**	3	0.46	**ORF1b:V1092F**	8	0.23
Maharashtra	**ORF1b:K2310N**	1	0.15	**ORF1b:K2063N**	2	0.26	**ORF1b:T1404G**	3	0.46	**ORF1b:A941V**	7	0.2
Maharashtra	**ORF1b:A8P**	1	0.15	**ORF1b:T2163I**	2	0.26	**ORF1b:N1426K**	3	0.46	**ORF1b:V1644L**	7	0.2
Maharashtra	**ORF1b:A434V**	1	0.15	**ORF1b:M371I**	2	0.26	**ORF1b:P1427***	3	0.46	**ORF1b:P1223S**	7	0.2
Maharashtra	**ORF1b:A1369V**	1	0.15	**ORF1b:M1352I**	2	0.26	**ORF1b:A1643V**	3	0.46	**ORF1b:S904L**	7	0.2
Maharashtra	**ORF1b:Q2295H**	1	0.15	**ORF1b:A1219S**	2	0.26	**ORF1b:V2117L**	3	0.46	**ORF1b:L1220F**	7	0.2
Maharashtra	**ORF1b:I2626V**	1	0.15	**ORF1b:A1521T**	2	0.26	**ORF1b:T2432I**	3	0.46	**ORF1b:M1156I**	7	0.2
Maharashtra	**ORF1b:G2337V**	1	0.15	**ORF1b:M2269I**	2	0.26	**ORF1b:M659I**	3	0.46	**ORF1b:R1517K**	7	0.2
Maharashtra	**ORF1b:K429N**	1	0.15	**ORF1b:N55D**	2	0.26	**ORF1b:S904L**	3	0.46	**ORF1b:Y1944F**	7	0.2
Maharashtra	**ORF1b:D1506N**	1	0.15	**ORF1b:I2114N**	2	0.26	**ORF1b:H1213Y**	3	0.46	**ORF1b:D1988V**	7	0.2
Maharashtra	**ORF1b:K1507T**	1	0.15	**ORF1b:S2339F**	2	0.26	**ORF1b:K1812R**	3	0.46	**ORF1b:V1990N**	7	0.2
Maharashtra	**ORF1b:E2544D**	1	0.15	**ORF1b:K2310N**	2	0.26	**ORF1b:I2357M**	3	0.46	**ORF1b:P1991T**	7	0.2
Maharashtra	**ORF1b:G2545F**	1	0.15	**ORF1b:T2099I**	2	0.26	**ORF1b:Y147L**	2	0.3	**ORF1b:K1993Q**	7	0.2
Maharashtra	**ORF1b:Q2226H**	1	0.15	**ORF1b:T1336I**	2	0.26	**ORF1b:N149S**	2	0.3	**ORF1b:S1994F**	7	0.2
Maharashtra	**ORF1b:V2369G**	1	0.15	**ORF1b:P2256L**	2	0.26	**ORF1b:V1279F**	2	0.3	**ORF1b:A1995R**	7	0.2
Maharashtra	**ORF1b:Y1176H**	1	0.15	**ORF1b:D1848Y**	2	0.26	**ORF1b:K1900R**	2	0.3	**ORF1b:T1996W**	7	0.2
Maharashtra	**ORF1b:Q813H**	1	0.15	**ORF1b:S640L**	2	0.26	**ORF1b:M1397I**	2	0.3	**ORF1b:Y2035G**	7	0.2
Maharashtra	**ORF1b:E269D**	1	0.15	**ORF1b:D2576E**	2	0.26	**ORF1b:P2612L**	2	0.3	**ORF1b:K2036L**	7	0.2
Maharashtra	**ORF1b:P976S**	1	0.15	**ORF1b:K50N**	2	0.26	**ORF1b:E2390Q**	2	0.3	**ORF1b:F2047I**	7	0.2
Maharashtra	**ORF1b:V871I**	1	0.15	**ORF1b:V2060F**	2	0.26	**ORF1b:N1946***	2	0.3	**ORF1b:T2048***	7	0.2
Maharashtra	**ORF1b:H1550Y**	1	0.15	**ORF1b:E2377***	2	0.26	**ORF1b:D870Y**	2	0.3	**ORF1b:R2049Y**	7	0.2
Maharashtra	**ORF1b:S1661I**	1	0.15	**ORF1b:L2316F**	2	0.26	**ORF1b:S961P**	2	0.3	**ORF1b:Q2051***	7	0.2
Maharashtra	**ORF1b:S2399I**	1	0.15	**ORF1b:Y1176H**	2	0.26	**ORF1b:A86S**	2	0.3	**ORF1b:S2052P**	7	0.2
Maharashtra	**ORF1b:R1078L**	1	0.15	**ORF1b:S2430I**	2	0.26	**ORF1b:A167S**	2	0.3	**ORF1b:F2106Q**	7	0.2
Maharashtra	**ORF1b:M620I**	1	0.15	**ORF1b:K1828N**	2	0.26	**ORF1b:N288K**	2	0.3	**ORF1b:E2107H**	7	0.2
Maharashtra	**ORF1b:D2617N**	1	0.15	**ORF1b:T132I**	2	0.26	**ORF1b:G662A**	2	0.3	**ORF1b:L2108***	7	0.2

Maharashtra	**ORF1b:T794I**	1	0.15	**ORF1b:H1318R**	2	0.26	**ORF1b:F803N**	2	0.3	**ORF1b:W2109T**	7	0.2
Maharashtra	**ORF1b:K798R**	1	0.15	**ORF1b:M2414I**	2	0.26	**ORF1b:D2090Y**	2	0.3	**ORF1b:A2110S**	7	0.2
Maharashtra	**ORF1b:K2421N**	1	0.15	**ORF1b:T2134N**	2	0.26	**ORF1b:S2363A**	2	0.3	**ORF1b:D2138L**	7	0.2
Maharashtra	**ORF1b:K1957R**	1	0.15	**ORF1b:V2073I**	2	0.26	**ORF1b:T1274I**	2	0.3	**ORF1b:Y2139Q**	7	0.2
Maharashtra	**ORF1b:G1107C**	1	0.15	**ORF1b:S1182L**	2	0.26	**ORF1b:Y1380K**	2	0.3	**ORF1b:M2155S**	7	0.2
Maharashtra	**ORF1b:V1271L**	1	0.15	**ORF1b:K1873N**	2	0.26	**ORF1b:D1381T**	2	0.3	**ORF1b:D2157Y**	7	0.2
Maharashtra	**ORF1b:A1432V**	1	0.15	**ORF1b:A370S**	2	0.26	**ORF1b:K1383Q**	2	0.3	**ORF1b:A2211T**	7	0.2
Maharashtra	**ORF1b:A2222V**	1	0.15	**ORF1b:M1197I**	2	0.26	**ORF1b:K1385N**	2	0.3	**ORF1b:G99V**	7	0.2
Maharashtra	**ORF1b:L2251V**	1	0.15	**ORF1b:K1383R**	2	0.26	**ORF1b:H1387L**	2	0.3	**ORF1b:D2020Y**	7	0.2
Maharashtra	**ORF1b:S316I**	1	0.15	**ORF1b:P1727L**	2	0.26	**ORF1b:K1396M**	2	0.3	**ORF1b:A2132V**	7	0.2
Maharashtra	**ORF1b:R2652K**	1	0.15	**ORF1b:S2198I**	2	0.26	**ORF1b:Y1399H**	2	0.3	**ORF1b:V2475G**	7	0.2
Maharashtra	**ORF1b:T634I**	1	0.15	**ORF1b:C2341F**	2	0.26	**ORF1b:K1400L**	2	0.3	**ORF1b:D2576G**	6	0.17
Maharashtra	**ORF1b:S640L**	1	0.15	**ORF1b:A941V**	2	0.26	**ORF1b:G1401Q**	2	0.3	**ORF1b:Q2635H**	6	0.17
Maharashtra	**ORF1b:L2672R**	1	0.15	**ORF1b:P2256S**	1	0.13	**ORF1b:V1402L**	2	0.3	**ORF1b:M897I**	6	0.17
Maharashtra	**ORF1b:R2684***	1	0.15	**ORF1b:I1566K**	1	0.13	**ORF1b:I1403T**	2	0.3	**ORF1b:V2102***	6	0.17
Maharashtra	**ORF1b:L2692V**	1	0.15	**ORF1b:V1945A**	1	0.13	**ORF1b:V1456T**	2	0.3	**ORF1b:N1776V**	6	0.17
Maharashtra	**ORF1b:N2694I**	1	0.15	**ORF1b:D1988A**	1	0.13	**ORF1b:D1457N**	2	0.3	**ORF1b:K1812P**	6	0.17
Maharashtra	**ORF1b:M2322I**	1	0.15	**ORF1b:P1287S**	1	0.13	**ORF1b:S1459H**	2	0.3	**ORF1b:W1816Y**	6	0.17
Maharashtra	**ORF1b:S2324Y**	1	0.15	**ORF1b:V1760F**	1	0.13	**ORF1b:Q1460I**	2	0.3	**ORF1b:T1817***	6	0.17
Maharashtra	**ORF1b:D2270E**	1	0.15	**ORF1b:D2179Y**	1	0.13	**ORF1b:G1461H**	2	0.3	**ORF1b:E1819S**	6	0.17
Maharashtra	**ORF1b:I2273S**	1	0.15	**ORF1b:S961P**	1	0.13	**ORF1b:R1483H**	2	0.3	**ORF1b:P1879***	6	0.17
Maharashtra	**ORF1b:Y1944F**	1	0.15	**ORF1b:D295V**	1	0.13	**ORF1b:F1484***	2	0.3	**ORF1b:P1953S**	6	0.17
Maharashtra	**ORF1b:F2291V**	1	0.15	**ORF1b:R296Q**	1	0.13	**ORF1b:V1486S**	2	0.3	**ORF1b:A1954F**	6	0.17
Maharashtra	**ORF1b:G418C**	1	0.15	**ORF1b:C297H**	1	0.13	**ORF1b:A1487S**	2	0.3	**ORF1b:F1955***	6	0.17
Maharashtra	**ORF1b:F2173C**	1	0.15	**ORF1b:I324A**	1	0.13	**ORF1b:I1488L**	2	0.3	**ORF1b:D1956***	6	0.17
Maharashtra	**ORF1b:G2235D**	1	0.15	**ORF1b:F325L**	1	0.13	**ORF1b:T1489L**	2	0.3	**ORF1b:S1958T**	6	0.17
Maharashtra	**ORF1b:Y1200H**	1	0.15	**ORF1b:V326***	1	0.13	**ORF1b:R1490***	2	0.3	**ORF1b:A1959I**	6	0.17
Maharashtra	**ORF1b:I2158M**	1	0.15	**ORF1b:D327S**	1	0.13	**ORF1b:A1491C**	2	0.3	**ORF1b:K1964***	6	0.17
Maharashtra	**ORF1b:V1945G**	1	0.15	**ORF1b:P330Q**	1	0.13	**ORF1b:V1493Q**	2	0.3	**ORF1b:K1981S**	6	0.17
Maharashtra	**ORF1b:P369L**	1	0.15	**ORF1b:F331L**	1	0.13	**ORF1b:G1494I**	2	0.3	**ORF1b:Q1982M**	6	0.17
Maharashtra	**ORF1b:A370G**	1	0.15	**ORF1b:V332K**	1	0.13	**ORF1b:I1495***	2	0.3	**ORF1b:V1983***	6	0.17
Maharashtra	**ORF1b:N1018T**	1	0.15	**ORF1b:V333L**	1	0.13	**ORF1b:L1496C**	2	0.3	**ORF1b:D1986C**	6	0.17
Maharashtra	**ORF1b:S1089A**	1	0.15	**ORF1b:S334Q**	1	0.13	**ORF1b:L1513V**	2	0.3	**ORF1b:I1987Y**	6	0.17
Maharashtra	**ORF1b:I2026L**	1	0.15	**ORF1b:T335M**	1	0.13	**ORF1b:E1514A**	2	0.3	**ORF1b:N2055K**	6	0.17
Maharashtra	**ORF1b:R270S**	1	0.15	**ORF1b:G336E**	1	0.13	**ORF1b:P1516Y**	2	0.3	**ORF1b:V2056C**	6	0.17
Maharashtra	**ORF1b:M1397I**	1	0.15	**ORF1b:Y337H**	1	0.13	**ORF1b:R1518S**	2	0.3	**ORF1b:A2057G**	6	0.17
Maharashtra	**ORF1b:L1179F**	1	0.15	**ORF1b:D795A**	1	0.13	**ORF1b:N1519***	2	0.3	**ORF1b:N2059***	6	0.17
Maharashtra	**ORF1b:T310I**	1	0.15	**ORF1b:G799R**	1	0.13	**ORF1b:V1520N**	2	0.3	**ORF1b:V2060C**	6	0.17
Maharashtra	**ORF1b:M657I**	1	0.15	**ORF1b:F803I**	1	0.13	**ORF1b:A1521S**	2	0.3	**ORF1b:V2061C**	6	0.17
Maharashtra	**ORF1b:T917A**	1	0.15	**ORF1b:C804G**	1	0.13	**ORF1b:L1523***	2	0.3	**ORF1b:N2062K**	6	0.17
Maharashtra	**ORF1b:P1427L**	1	0.15	**ORF1b:V896I**	1	0.13	**ORF1b:Q1524E**	2	0.3	**ORF1b:K2063***	6	0.17
Maharashtra	**ORF1b:Y1464C**	1	0.15	**ORF1b:S1779I**	1	0.13	**ORF1b:A1525C**	2	0.3	**ORF1b:H2065S**	6	0.17
Maharashtra	**ORF1b:T1522I**	1	0.15	**ORF1b:T861I**	1	0.13	**ORF1b:E1526G**	2	0.3	**ORF1b:T2195I**	6	0.17
Maharashtra	**ORF1b:S1187N**	1	0.15	**ORF1b:V2236A**	1	0.13	**ORF1b:V1528F**	2	0.3	**ORF1b:E2196C**	6	0.17
Maharashtra	**ORF1b:P255L**	1	0.15	**ORF1b:G662V**	1	0.13	**ORF1b:L1531N**	2	0.3	**ORF1b:G2197R**	6	0.17
Maharashtra	**ORF1b:Y256G**	1	0.15	**ORF1b:E735D**	1	0.13	**ORF1b:F1532H**	2	0.3	**ORF1b:S997L**	6	0.17
Maharashtra	**ORF1b:I257V**	1	0.15	**ORF1b:E2614D**	1	0.13	**ORF1b:K1533W**	2	0.3	**ORF1b:T1555I**	6	0.17
Maharashtra	**ORF1b:V691A**	1	0.15	**ORF1b:G2151D**	1	0.13	**ORF1b:D1534V**	2	0.3	**ORF1b:V464F**	6	0.17
Maharashtra	**ORF1b:C939S**	1	0.15	**ORF1b:P160S**	1	0.13	**ORF1b:C1535T**	2	0.3	**ORF1b:T2163I**	6	0.17
Maharashtra	**ORF1b:I1181P**	1	0.15	**ORF1b:A2513V**	1	0.13	**ORF1b:D1554Y**	2	0.3	**ORF1b:S2338L**	5	0.14
Maharashtra	**ORF1b:V2120S**	1	0.15	**ORF1b:D870Y**	1	0.13	**ORF1b:K1556G**	2	0.3	**ORF1b:A1884S**	5	0.14
Maharashtra	**ORF1b:Y2393H**	1	0.15	**ORF1b:R1813H**	1	0.13	**ORF1b:F1557T**	2	0.3	**ORF1b:T2488M**	5	0.14
Maharashtra	**ORF1b:G2562V**	1	0.15	**ORF1b:T1050I**	1	0.13	**ORF1b:K1558Y**	2	0.3	**ORF1b:P218L**	5	0.14
Maharashtra	**ORF1b:N2695I**	1	0.15	**ORF1b:S1661I**	1	0.13	**ORF1b:E1560P**	2	0.3	**ORF1b:S1182L**	5	0.14
Maharashtra	**ORF1b:D58N**	1	0.15	**ORF1b:I527T**	1	0.13	**ORF1b:G1561Q**	2	0.3	**ORF1b:T310I**	5	0.14
Maharashtra	**ORF1b:E427L**	1	0.15	**ORF1b:G1129V**	1	0.13	**ORF1b:L1562C**	2	0.3	**ORF1b:M657I**	5	0.14
Maharashtra	**ORF1b:V1092A**	1	0.15	**ORF1b:M1197V**	1	0.13	**ORF1b:C1563***	2	0.3	**ORF1b:S2212I**	5	0.14
Maharashtra	**ORF1b:D91T**	1	0.15	**ORF1b:Y256H**	1	0.13	**ORF1b:V1564H**	2	0.3	**ORF1b:N696F**	5	0.14
Maharashtra	**ORF1b:D131N**	1	0.15	**ORF1b:L238V**	1	0.13	**ORF1b:D1565***	2	0.3	**ORF1b:A697Y**	5	0.14
Maharashtra	**ORF1b:D436H**	1	0.15	**ORF1b:A647S**	1	0.13	**ORF1b:P1567K**	2	0.3	**ORF1b:Y710M**	5	0.14
Maharashtra	**ORF1b:G205L**	1	0.15	**ORF1b:G2127S**	1	0.13	**ORF1b:G1568T**	2	0.3	**ORF1b:R712T**	5	0.14
Maharashtra	**ORF1b:C478S**	1	0.15	**ORF1b:S1089L**	1	0.13	**ORF1b:I1569H**	2	0.3	**ORF1b:N713K**	5	0.14
Maharashtra	**ORF1b:G2215E**	1	0.15	**ORF1b:V345L**	1	0.13	**ORF1b:K1571Y**	2	0.3	**ORF1b:E793L**	5	0.14
Maharashtra	**ORF1b:G799E**	1	0.15	**ORF1b:T1637I**	1	0.13	**ORF1b:M1573G**	2	0.3	**ORF1b:T794K**	5	0.14
Maharashtra	**ORF1b:P800R**	1	0.15	**ORF1b:P1095S**	1	0.13	**ORF1b:T1574F**	2	0.3	**ORF1b:Y894M**	5	0.14
Maharashtra	**ORF1b:T385M**	1	0.15	**ORF1b:I1355T**	1	0.13	**ORF1b:Y1575***	2	0.3	**ORF1b:V896***	5	0.14
Maharashtra	**ORF1b:C386***	1	0.15	**ORF1b:E1091A**	1	0.13	**ORF1b:R1576N**	2	0.3	**ORF1b:M897Q**	5	0.14
Maharashtra	**ORF1b:F387M**	1	0.15	**ORF1b:K151N**	1	0.13	**ORF1b:R1577E**	2	0.3	**ORF1b:L898D**	5	0.14
Maharashtra	**ORF1b:S388R**	1	0.15	**ORF1b:Q2070H**	1	0.13	**ORF1b:I1579S**	2	0.3	**ORF1b:D901L**	5	0.14
Maharashtra	**ORF1b:T547I**	1	0.15	**ORF1b:A1918V**	1	0.13	**ORF1b:E1601***	2	0.3	**ORF1b:N902S**	5	0.14
Maharashtra	**ORF1b:E802C**	1	0.15	**ORF1b:R1729C**	1	0.13	**ORF1b:E1602W**	2	0.3	**ORF1b:T903F**	5	0.14
Maharashtra	**ORF1b:F803C**	1	0.15	**ORF1b:I1611V**	1	0.13	**ORF1b:A1603L**	2	0.3	**ORF1b:S904M**	5	0.14
Maharashtra	**ORF1b:A1131S**	1	0.15	**ORF1b:P1664S**	1	0.13	**ORF1b:I1604P**	2	0.3	**ORF1b:P918L**	5	0.14

Maharashtra	**ORF1b:K1199N**	1	0.15	**ORF1b:D146G**	1	0.13	**ORF1b:R1605***	2	0.3	**ORF1b:H919C**	5	0.14
Maharashtra	**ORF1b:T1473A**	1	0.15	**ORF1b:Q2238R**	1	0.13	**ORF1b:D1656***	2	0.3	**ORF1b:L922F**	5	0.14
Maharashtra	**ORF1b:A1476S**	1	0.15	**ORF1b:T2455M**	1	0.13	**ORF1b:F1657***	2	0.3	**ORF1b:Q923F**	5	0.14
Maharashtra	**ORF1b:M1839I**	1	0.15	**ORF1b:R1687C**	1	0.13	**ORF1b:S1658Y**	2	0.3	**ORF1b:I1416T**	5	0.14
Maharashtra	**ORF1b:N288K**	1	0.15	**ORF1b:S2312L**	1	0.13	**ORF1b:V1660R**	2	0.3	**ORF1b:M1197I**	5	0.14
Maharashtra	**ORF1b:K1388E**	1	0.15	**ORF1b:M657V**	1	0.13	**ORF1b:A1662I**	2	0.3	**ORF1b:S967Q**	5	0.14
Maharashtra	**ORF1b:C1740A**	1	0.15	**ORF1b:T1739I**	1	0.13	**ORF1b:K1663***	2	0.3	**ORF1b:Y2018S**	5	0.14
Maharashtra	**ORF1b:F1741C**	1	0.15	**ORF1b:R2613C**	1	0.13	**ORF1b:P1664T**	2	0.3	**ORF1b:A2028C**	5	0.14
Maharashtra	**ORF1b:S1742P**	1	0.15	**ORF1b:N734D**	1	0.13	**ORF1b:P1666T**	2	0.3	**ORF1b:T1139N**	5	0.14
Maharashtra	**ORF1b:T1743P**	1	0.15	**ORF1b:V1649F**	1	0.13	**ORF1b:G1667Y**	2	0.3	**ORF1b:A1831H**	5	0.14
Maharashtra	**ORF1b:A1744H**	1	0.15	**ORF1b:I257T**	1	0.13	**ORF1b:D1668V**	2	0.3	**ORF1b:A1832G**	5	0.14
Maharashtra	**ORF1b:S1745***	1	0.15	**ORF1b:R1262C**	1	0.13	**ORF1b:F1670R**	2	0.3	**ORF1b:D1855I**	5	0.14
Maharashtra	**ORF1b:D1746S**	1	0.15	**ORF1b:F1510C**	1	0.13	**ORF1b:K1671T**	2	0.3	**ORF1b:A1877S**	5	0.14
Maharashtra	**ORF1b:T1747V**	1	0.15	**ORF1b:T1511A**	1	0.13	**ORF1b:H1672S**	2	0.3	**ORF1b:Q1878S**	5	0.14
Maharashtra	**ORF1b:D2175Y**	1	0.15	**ORF1b:S1512G**	1	0.13	**ORF1b:I1674E**	2	0.3	**ORF1b:D1882L**	5	0.14
Maharashtra	**ORF1b:T2332S**	1	0.15	**ORF1b:E1514L**	1	0.13	**ORF1b:P1675C**	2	0.3	**ORF1b:K1883***	5	0.14
Maharashtra	**ORF1b:D249Y**	1	0.15	**ORF1b:N1595***	1	0.13	**ORF1b:L1676S**	2	0.3	**ORF1b:A1884N**	5	0.14
Maharashtra	**ORF1b:G170V**	1	0.15	**ORF1b:G1940F**	1	0.13	**ORF1b:M1677A**	2	0.3	**ORF1b:Y1885R**	5	0.14
Maharashtra	**ORF1b:N291F**	1	0.15	**ORF1b:H2293Q**	1	0.13	**ORF1b:L1698***	2	0.3	**ORF1b:K1886R**	5	0.14
Maharashtra	**ORF1b:C292N**	1	0.15	**ORF1b:C130S**	1	0.13	**ORF1b:N1700S**	2	0.3	**ORF1b:N1910Q**	5	0.14
Maharashtra	**ORF1b:L293M**	1	0.15	**ORF1b:M1693I**	1	0.13	**ORF1b:F1714T**	2	0.3	**ORF1b:F1950P**	5	0.14
Maharashtra	**ORF1b:P1000L**	1	0.15	**ORF1b:G703V**	1	0.13	**ORF1b:E1715W**	2	0.3	**ORF1b:K2255L**	5	0.14
Maharashtra	**ORF1b:M1499I**	1	0.15	**ORF1b:V871I**	1	0.13	**ORF1b:T1717***	2	0.3	**ORF1b:P2256R**	5	0.14
Maharashtra	**ORF1b:I2194N**	1	0.15	**ORF1b:S2198C**	1	0.13	**ORF1b:S1718V**	2	0.3	**ORF1b:R2257I**	5	0.14
Maharashtra	**ORF1b:D152N**	1	0.15	**ORF1b:D1127Y**	1	0.13	**ORF1b:M1719D**	2	0.3	**ORF1b:Q2259Y**	5	0.14
Maharashtra	**ORF1b:V1945I**	1	0.15	**ORF1b:H716Y**	1	0.13	**ORF1b:K1720I**	2	0.3	**ORF1b:M2260G**	5	0.14
Maharashtra	**ORF1b:V1493L**	1	0.15	**ORF1b:G2297V**	1	0.13	**ORF1b:F1722E**	2	0.3	**ORF1b:E2261***	5	0.14
Maharashtra	**ORF1b:R164H**	1	0.15	**ORF1b:A2559T**	1	0.13	**ORF1b:C1731F**	2	0.3	**ORF1b:A2268H**	5	0.14
Maharashtra	**ORF1b:Q108L**	1	0.15	**ORF1b:D2333N**	1	0.13	**ORF1b:L1733E**	2	0.3	**ORF1b:M2269***	5	0.14
Maharashtra	**ORF1b:R109F**	1	0.15	**ORF1b:C1732S**	1	0.13	**ORF1b:C1734N**	2	0.3	**ORF1b:D2270T**	5	0.14
Maharashtra	**ORF1b:S309T**	1	0.15	**ORF1b:D884Y**	1	0.13	**ORF1b:D1735R**	2	0.3	**ORF1b:E2271V**	5	0.14
Maharashtra	**ORF1b:S904L**	1	0.15	**ORF1b:E2355K**	1	0.13	**ORF1b:R1736T**	2	0.3	**ORF1b:F2272***	5	0.14
Maharashtra	**ORF1b:T11A**	1	0.15	**ORF1b:N865S**	1	0.13	**ORF1b:R1737***	2	0.3	**ORF1b:E2274R**	5	0.14
Maharashtra	**ORF1b:P1664S**	1	0.15	**ORF1b:T1162L**	1	0.13	**ORF1b:T1739H**	2	0.3	**ORF1b:Y2276L**	5	0.14
Maharashtra	**ORF1b:P2633L**	1	0.15	**ORF1b:V1164F**	1	0.13	**ORF1b:C1740L**	2	0.3	**ORF1b:K2277C**	5	0.14
Maharashtra	**ORF1b:I1598V**	1	0.15	**ORF1b:P1165Q**	1	0.13	**ORF1b:T1743L**	2	0.3	**ORF1b:E2279R**	5	0.14
Maharashtra	**ORF1b:T1333I**	1	0.15	**ORF1b:L2019I**	1	0.13	**ORF1b:T1774***	2	0.3	**ORF1b:G2280T**	5	0.14
Maharashtra	**ORF1b:V2691F**	1	0.15	**ORF1b:D2020K**	1	0.13	**ORF1b:G1775S**	2	0.3	**ORF1b:Q2295R**	5	0.14
Maharashtra	**ORF1b:L1220F**	1	0.15	**ORF1b:A2021I**	1	0.13	**ORF1b:Q1778S**	2	0.3	**ORF1b:W2383C**	5	0.14
Maharashtra	**ORF1b:I1690V**	1	0.15	**ORF1b:V1309A**	1	0.13	**ORF1b:N1780P**	2	0.3	**ORF1b:T1621I**	5	0.14
Maharashtra	**ORF1b:P864S**	1	0.15	**ORF1b:V1021I**	1	0.13	**ORF1b:H1781W**	2	0.3	**ORF1b:T730I**	5	0.14
Maharashtra	**ORF1b:I847V**	1	0.15	**ORF1b:M1677I**	1	0.13	**ORF1b:D1782***	2	0.3	**ORF1b:A302S**	5	0.14
Maharashtra	**ORF1b:L1681F**	1	0.15	**ORF1b:V2117L**	1	0.13	**ORF1b:L1783C**	2	0.3	**ORF1b:A1219S**	5	0.14
Maharashtra	**ORF1b:V2182L**	1	0.15	**ORF1b:G2489D**	1	0.13	**ORF1b:Y1784T**	2	0.3	**ORF1b:A690S**	5	0.14
Maharashtra	**ORF1b:T797I**	1	0.15	**ORF1b:R109H**	1	0.13	**ORF1b:Q1786S**	2	0.3	**ORF1b:A414V**	5	0.14
Maharashtra	**ORF1b:H1838Y**	1	0.15	**ORF1b:E1977Q**	1	0.13	**ORF1b:V1787***	2	0.3	**ORF1b:D1988G**	5	0.14
Maharashtra	**ORF1b:V2691I**	1	0.15	**ORF1b:K996E**	1	0.13	**ORF1b:H1788L**	2	0.3	**ORF1b:A1798S**	5	0.14
Maharashtra	**ORF1b:A37T**	1	0.15	**ORF1b:L1248F**	1	0.13	**ORF1b:G1789***	2	0.3	**ORF1b:A2559S**	5	0.14
Maharashtra	**ORF1b:I2286V**	1	0.15	**ORF1b:C804I**	1	0.13	**ORF1b:N1790C**	2	0.3	**ORF1b:D2090Y**	5	0.14
Maharashtra	**ORF1b:T730K**	1	0.15	**ORF1b:H807L**	1	0.13	**ORF1b:A1791N**	2	0.3	**ORF1b:D2067Y**	5	0.14
Maharashtra	**ORF1b:S2205F**	1	0.15	**ORF1b:P823***	1	0.13	**ORF1b:A1794S**	2	0.3	**ORF1b:K1835N**	4	0.11
Maharashtra	**ORF1b:K1724R**	1	0.15	**ORF1b:D824R**	1	0.13	**ORF1b:K1812P**	2	0.3	**ORF1b:K709N**	4	0.11
Maharashtra	**ORF1b:S1958N**	1	0.15	**ORF1b:P825T**	1	0.13	**ORF1b:W1816Y**	2	0.3	**ORF1b:M1693I**	4	0.11
Maharashtra	**ORF1b:A1476L**	1	0.15	**ORF1b:R827***	1	0.13	**ORF1b:T1817***	2	0.3	**ORF1b:Q813K**	4	0.11
Maharashtra	**ORF1b:M2644I**	1	0.15	**ORF1b:H883T**	1	0.13	**ORF1b:E1819S**	2	0.3	**ORF1b:T1076I**	4	0.11
Maharashtra	**ORF1b:N1039T**	1	0.15	**ORF1b:D884***	1	0.13	**ORF1b:A1831H**	2	0.3	**ORF1b:K667M**	4	0.11
Maharashtra	**ORF1b:A1160T**	1	0.15	**ORF1b:E885V**	1	0.13	**ORF1b:A1832G**	2	0.3	**ORF1b:P976L**	4	0.11
Maharashtra	**ORF1b:R1G**	1	0.15	**ORF1b:T887***	1	0.13	**ORF1b:V1852***	2	0.3	**ORF1b:L845***	4	0.11
Maharashtra	**ORF1b:V2I**	1	0.15	**ORF1b:A1057C**	1	0.13	**ORF1b:L1853S**	2	0.3	**ORF1b:E848M**	4	0.11
Maharashtra	**ORF1b:T1404M**	1	0.15	**ORF1b:A1058S**	1	0.13	**ORF1b:H1854C**	2	0.3	**ORF1b:R849L**	4	0.11
Maharashtra	**ORF1b:Q1509P**	1	0.15	**ORF1b:E1059K**	1	0.13	**ORF1b:D1855I**	2	0.3	**ORF1b:F850T**	4	0.11
Maharashtra	**ORF1b:S1942N**	1	0.15	**ORF1b:T1637R**	1	0.13	**ORF1b:A1877S**	2	0.3	**ORF1b:V851H**	4	0.11
Maharashtra	**ORF1b:S2205Y**	1	0.15	**ORF1b:N1640M**	1	0.13	**ORF1b:Q1878S**	2	0.3	**ORF1b:S852L**	4	0.11
Maharashtra	**ORF1b:G2207P**	1	0.15	**ORF1b:V1642L**	1	0.13	**ORF1b:P1879***	2	0.3	**ORF1b:A854N**	4	0.11
Maharashtra	**ORF1b:H2388P**	1	0.15	**ORF1b:A1643L**	1	0.13	**ORF1b:D1882L**	2	0.3	**ORF1b:V1110L**	4	0.11
Maharashtra	**ORF1b:V2389P**	1	0.15	**ORF1b:G1647T**	1	0.13	**ORF1b:K1883***	2	0.3	**ORF1b:R944H**	4	0.11
Maharashtra	**ORF1b:E2390K**	1	0.15	**ORF1b:F1670R**	1	0.13	**ORF1b:Y1885R**	2	0.3	**ORF1b:C953S**	4	0.11
Maharashtra	**ORF1b:L2017V**	1	0.15	**ORF1b:K1671T**	1	0.13	**ORF1b:K1886R**	2	0.3	**ORF1b:D955Q**	4	0.11
Maharashtra	**ORF1b:V2371L**	1	0.15	**ORF1b:H1672S**	1	0.13	**ORF1b:E1889I**	2	0.3	**ORF1b:V957H**	4	0.11
Maharashtra	**ORF1b:K82R**	1	0.15	**ORF1b:P1675C**	1	0.13	**ORF1b:G1904L**	2	0.3	**ORF1b:S959N**	4	0.11
Maharashtra	**ORF1b:D1465Y**	1	0.15	**ORF1b:F1714T**	1	0.13	**ORF1b:V1905C**	2	0.3	**ORF1b:T960***	4	0.11
Maharashtra	**ORF1b:I1032V**	1	0.15	**ORF1b:E1715W**	1	0.13	**ORF1b:C1906H**	2	0.3	**ORF1b:H962C**	4	0.11
Maharashtra	**ORF1b:P1177L**	1	0.15	**ORF1b:T1717***	1	0.13	**ORF1b:L1907T**	2	0.3	**ORF1b:K963L**	4	0.11

Maharashtra	**ORF1b:L1504F**	1	0.15	**ORF1b:S1718V**	1	0.13	**ORF1b:W1909***	2	0.3	**ORF1b:V965M**	4	0.11
Maharashtra	**ORF1b:A2513V**	1	0.15	**ORF1b:M1719D**	1	0.13	**ORF1b:N1910Q**	2	0.3	**ORF1b:S1942C**	4	0.11
Maharashtra	**ORF1b:S1893F**	1	0.15	**ORF1b:K1720I**	1	0.13	**ORF1b:C1911I**	2	0.3	**ORF1b:Y1944V**	4	0.11
Maharashtra	**ORF1b:M2414I**	1	0.15	**ORF1b:F1722E**	1	0.13	**ORF1b:N1912H**	2	0.3	**ORF1b:K1947M**	4	0.11
Maharashtra	**ORF1b:K2160N**	1	0.15	**ORF1b:T1774C**	1	0.13	**ORF1b:V1913R**	2	0.3	**ORF1b:A1949S**	4	0.11
Maharashtra	**ORF1b:V1760F**	1	0.15	**ORF1b:G1775E**	1	0.13	**ORF1b:D1914W**	2	0.3	**ORF1b:F1950T**	4	0.11
Maharashtra	**ORF1b:S2339F**	1	0.15	**ORF1b:Q1778***	1	0.13	**ORF1b:R1915C**	2	0.3	**ORF1b:D1986***	4	0.11
Maharashtra	**ORF1b:A647S**	1	0.15	**ORF1b:S1779C**	1	0.13	**ORF1b:Y1916M**	2	0.3	**ORF1b:D1988M**	4	0.11
Maharashtra	**ORF1b:N377C**	1	0.15	**ORF1b:H1792***	1	0.13	**ORF1b:A1918I**	2	0.3	**ORF1b:R2009***	4	0.11
Maharashtra	**ORF1b:L378T**	1	0.15	**ORF1b:A1794S**	1	0.13	**ORF1b:N1919L**	2	0.3	**ORF1b:H2011V**	4	0.11
Maharashtra	**ORF1b:D381A**	1	0.15	**ORF1b:E1888L**	1	0.13	**ORF1b:S1920E**	2	0.3	**ORF1b:N2013I**	4	0.11
Maharashtra	**ORF1b:K382L**	1	0.15	**ORF1b:V1922Q**	1	0.13	**ORF1b:I1921L**	2	0.3	**ORF1b:E2014***	4	0.11
Maharashtra	**ORF1b:E422N**	1	0.15	**ORF1b:Q1965W**	1	0.13	**ORF1b:V1922Q**	2	0.3	**ORF1b:Y2015V**	4	0.11
Maharashtra	**ORF1b:G423T**	1	0.15	**ORF1b:P1967H**	1	0.13	**ORF1b:G2215K**	2	0.3	**ORF1b:R2016V**	4	0.11
Maharashtra	**ORF1b:Y446L**	1	0.15	**ORF1b:F2038H**	1	0.13	**ORF1b:K2255L**	2	0.3	**ORF1b:L2019V**	4	0.11
Maharashtra	**ORF1b:Y447L**	1	0.15	**ORF1b:D2039***	1	0.13	**ORF1b:H604Y**	2	0.3	**ORF1b:A2021I**	4	0.11
Maharashtra	**ORF1b:N450Y**	1	0.15	**ORF1b:I2150R**	1	0.13	**ORF1b:H1948C**	2	0.3	**ORF1b:Y2022M**	4	0.11
Maharashtra	**ORF1b:P452***	1	0.15	**ORF1b:V2152R**	1	0.13	**ORF1b:A1949I**	2	0.3	**ORF1b:N2023L**	4	0.11
Maharashtra	**ORF1b:T453L**	1	0.15	**ORF1b:M2155S**	1	0.13	**ORF1b:F1950P**	2	0.3	**ORF1b:M2025C**	4	0.11
Maharashtra	**ORF1b:M454K**	1	0.15	**ORF1b:D2157Y**	1	0.13	**ORF1b:P1953S**	2	0.3	**ORF1b:Y2041N**	4	0.11
Maharashtra	**ORF1b:C455L**	1	0.15	**ORF1b:A2159S**	1	0.13	**ORF1b:A1954F**	2	0.3	**ORF1b:W2044I**	4	0.11
Maharashtra	**ORF1b:D456L**	1	0.15	**ORF1b:K2160R**	1	0.13	**ORF1b:F1955***	2	0.3	**ORF1b:N2045T**	4	0.11
Maharashtra	**ORF1b:R458S**	1	0.15	**ORF1b:P2162N**	1	0.13	**ORF1b:D1956***	2	0.3	**ORF1b:T2046S**	4	0.11
Maharashtra	**ORF1b:Q459T**	1	0.15	**ORF1b:Q2239L**	1	0.13	**ORF1b:S1958T**	2	0.3	**ORF1b:F2047G**	4	0.11
Maharashtra	**ORF1b:K499E**	1	0.15	**ORF1b:P2241***	1	0.13	**ORF1b:A1959I**	2	0.3	**ORF1b:R2049L**	4	0.11
Maharashtra	**ORF1b:W500L**	1	0.15	**ORF1b:T2243R**	1	0.13	**ORF1b:V1961L**	2	0.3	**ORF1b:I2077L**	4	0.11
Maharashtra	**ORF1b:G501***	1	0.15	**ORF1b:Y2244I**	1	0.13	**ORF1b:N1962L**	2	0.3	**ORF1b:Y1140***	4	0.11
Maharashtra	**ORF1b:K502G**	1	0.15	**ORF1b:F2245***	1	0.13	**ORF1b:K1964***	2	0.3	**ORF1b:F1469L**	4	0.11
Maharashtra	**ORF1b:A503S**	1	0.15	**ORF1b:S2248E**	1	0.13	**ORF1b:S1779I**	2	0.3	**ORF1b:T1470K**	4	0.11
Maharashtra	**ORF1b:Y521C**	1	0.15	**ORF1b:R2249S**	1	0.13	**ORF1b:A2585P**	2	0.3	**ORF1b:T1472L**	4	0.11
Maharashtra	**ORF1b:K523F**	1	0.15	**ORF1b:Y2281N**	1	0.13	**ORF1b:V345***	2	0.3	**ORF1b:N1485L**	4	0.11
Maharashtra	**ORF1b:K542S**	1	0.15	**ORF1b:A2282***	1	0.13	**ORF1b:R1096K**	2	0.3	**ORF1b:A1487M**	4	0.11
Maharashtra	**ORF1b:R544D**	1	0.15	**ORF1b:E2284L**	1	0.13	**ORF1b:R1226H**	2	0.3	**ORF1b:I1488***	4	0.11
Maharashtra	**ORF1b:A545Q**	1	0.15	**ORF1b:H2285R**	1	0.13	**ORF1b:G799T**	2	0.3	**ORF1b:R1490F**	4	0.11
Maharashtra	**ORF1b:R546***	1	0.15	**ORF1b:V2287S**	1	0.13	**ORF1b:L2577I**	2	0.3	**ORF1b:K1492***	4	0.11
Maharashtra	**ORF1b:A549S**	1	0.15	**ORF1b:D2290R**	1	0.13	**ORF1b:F1557C**	2	0.3	**ORF1b:V1493C**	4	0.11
Maharashtra	**ORF1b:G550S**	1	0.15	**ORF1b:S2292I**	1	0.13	**ORF1b:P1223S**	2	0.3	**ORF1b:G1494L**	4	0.11
Maharashtra	**ORF1b:V551K**	1	0.15	**ORF1b:H2293C**	1	0.13	**ORF1b:P1936S**	2	0.3	**ORF1b:L1496E**	4	0.11
Maharashtra	**ORF1b:S552I**	1	0.15	**ORF1b:Q2295F**	1	0.13	**ORF1b:L1220F**	2	0.3	**ORF1b:C1497T**	4	0.11
Maharashtra	**ORF1b:V579E**	1	0.15	**ORF1b:L2296***	1	0.13	**ORF1b:P1342S**	2	0.3	**ORF1b:I1498F**	4	0.11
Maharashtra	**ORF1b:E601K**	1	0.15	**ORF1b:L2396C**	1	0.13	**ORF1b:G1450K**	2	0.3	**ORF1b:E1514Q**	4	0.11
Maharashtra	**ORF1b:L605G**	1	0.15	**ORF1b:G2428S**	1	0.13	**ORF1b:V1467L**	2	0.3	**ORF1b:I1515V**	4	0.11
Maharashtra	**ORF1b:M606L**	1	0.15	**ORF1b:D2429C**	1	0.13	**ORF1b:F1469L**	2	0.3	**ORF1b:P1516L**	4	0.11
Maharashtra	**ORF1b:W608***	1	0.15	**ORF1b:S2430F**	1	0.13	**ORF1b:T1470K**	2	0.3	**ORF1b:R1518F**	4	0.11
Maharashtra	**ORF1b:D609N**	1	0.15	**ORF1b:A2431K**	1	0.13	**ORF1b:T1472L**	2	0.3	**ORF1b:N1519H**	4	0.11
Maharashtra	**ORF1b:H633G**	1	0.15	**ORF1b:G2474V**	1	0.13	**ORF1b:N1485L**	2	0.3	**ORF1b:A1521G**	4	0.11
Maharashtra	**ORF1b:T634L**	1	0.15	**ORF1b:P2477Y**	1	0.13	**ORF1b:A1487M**	2	0.3	**ORF1b:T1522M**	4	0.11
Maharashtra	**ORF1b:V711K**	1	0.15	**ORF1b:T2479Y**	1	0.13	**ORF1b:I1488***	2	0.3	**ORF1b:L1523W**	4	0.11
Maharashtra	**ORF1b:R712***	1	0.15	**ORF1b:V2481H**	1	0.13	**ORF1b:R1490F**	2	0.3	**ORF1b:F1532Y**	4	0.11
Maharashtra	**ORF1b:N713R**	1	0.15	**ORF1b:R2483H**	1	0.13	**ORF1b:K1492***	2	0.3	**ORF1b:C1535S**	4	0.11
Maharashtra	**ORF1b:L714C**	1	0.15	**ORF1b:Q2484L**	1	0.13	**ORF1b:V1493C**	2	0.3	**ORF1b:S1536L**	4	0.11
Maharashtra	**ORF1b:Q715***	1	0.15	**ORF1b:L2486V**	1	0.13	**ORF1b:G1494L**	2	0.3	**ORF1b:V1538Y**	4	0.11
Maharashtra	**ORF1b:Y719C**	1	0.15	**ORF1b:P2487Y**	1	0.13	**ORF1b:L1496E**	2	0.3	**ORF1b:T1540L**	4	0.11
Maharashtra	**ORF1b:C721***	1	0.15	**ORF1b:E2570A**	1	0.13	**ORF1b:C1497T**	2	0.3	**ORF1b:G1541H**	4	0.11
Maharashtra	**ORF1b:L722V**	1	0.15	**ORF1b:H2571S**	1	0.13	**ORF1b:I1498F**	2	0.3	**ORF1b:L1542R**	4	0.11
Maharashtra	**ORF1b:Y723L**	1	0.15	**ORF1b:R383V**	1	0.13	**ORF1b:E1514Q**	2	0.3	**ORF1b:T1549L**	4	0.11
Maharashtra	**ORF1b:H743I**	1	0.15	**ORF1b:T384A**	1	0.13	**ORF1b:I1515V**	2	0.3	**ORF1b:L1551T**	4	0.11
Maharashtra	**ORF1b:S745A**	1	0.15	**ORF1b:T385R**	1	0.13	**ORF1b:P1516L**	2	0.3	**ORF1b:D1554L**	4	0.11
Maharashtra	**ORF1b:M746***	1	0.15	**ORF1b:S388F**	1	0.13	**ORF1b:R1518F**	2	0.3	**ORF1b:F1557L**	4	0.11
Maharashtra	**ORF1b:M747T**	1	0.15	**ORF1b:R2613H**	1	0.13	**ORF1b:N1519H**	2	0.3	**ORF1b:T1559V**	4	0.11
Maharashtra	**ORF1b:I748F**	1	0.15	**ORF1b:D2666E**	1	0.13	**ORF1b:A1521G**	2	0.3	**ORF1b:E1560Y**	4	0.11
Maharashtra	**ORF1b:S750N**	1	0.15	**ORF1b:V345Q**	1	0.13	**ORF1b:T1522M**	2	0.3	**ORF1b:G1561V**	4	0.11
Maharashtra	**ORF1b:Y761S**	1	0.15	**ORF1b:S2212I**	1	0.13	**ORF1b:L1523W**	2	0.3	**ORF1b:V1852***	4	0.11
Maharashtra	**ORF1b:A762R**	1	0.15	**ORF1b:P1975S**	1	0.13	**ORF1b:F1532Y**	2	0.3	**ORF1b:L1853S**	4	0.11
Maharashtra	**ORF1b:Q764S**	1	0.15	**ORF1b:Q2295H**	1	0.13	**ORF1b:C1535S**	2	0.3	**ORF1b:H1854C**	4	0.11
Maharashtra	**ORF1b:V767***	1	0.15	**ORF1b:I2626V**	1	0.13	**ORF1b:S1536L**	2	0.3	**ORF1b:E1889I**	4	0.11
Maharashtra	**ORF1b:A768V**	1	0.15	**ORF1b:K771R**	1	0.13	**ORF1b:V1538Y**	2	0.3	**ORF1b:G1904L**	4	0.11
Maharashtra	**ORF1b:V776L**	1	0.15	**ORF1b:M2414V**	1	0.13	**ORF1b:T1540L**	2	0.3	**ORF1b:A1949I**	4	0.11
Maharashtra	**ORF1b:Y778S**	1	0.15	**ORF1b:R1P**	1	0.13	**ORF1b:G1541H**	2	0.3	**ORF1b:R2016C**	4	0.11
Maharashtra	**ORF1b:Y779K**	1	0.15	**ORF1b:F433Y**	1	0.13	**ORF1b:L1542R**	2	0.3	**ORF1b:Y2018D**	4	0.11
Maharashtra	**ORF1b:W791L**	1	0.15	**ORF1b:R270S**	1	0.13	**ORF1b:T1549L**	2	0.3	**ORF1b:L2032S**	4	0.11
Maharashtra	**ORF1b:T792L**	1	0.15	**ORF1b:N902S**	1	0.13	**ORF1b:L1551T**	2	0.3	**ORF1b:I2136L**	4	0.11
Maharashtra	**ORF1b:E793S**	1	0.15	**ORF1b:E2266I**	1	0.13	**ORF1b:D1554L**	2	0.3	**ORF1b:W2137G**	4	0.11

Maharashtra	ORF1b:D836R	1	0.15	ORF1b:I770K	1	0.13	ORF1b:F1557L	2	0.3	ORF1b:F1148N	4	0.11
Maharashtra	ORF1b:D837S	1	0.15	ORF1b:I1074V	1	0.13	ORF1b:T1559V	2	0.3	ORF1b:V1149N	4	0.11
Maharashtra	ORF1b:V839K	1	0.15	ORF1b:H743N	1	0.13	ORF1b:E1560Y	2	0.3	ORF1b:S1408*	4	0.11
Maharashtra	ORF1b:K840N	1	0.15	ORF1b:V222I	1	0.13	ORF1b:G1561V	2	0.3	ORF1b:S1409Q	4	0.11
Maharashtra	ORF1b:T841P	1	0.15	ORF1b:D1668N	1	0.13	ORF1b:R1576K	2	0.3	ORF1b:I1411T	4	0.11
Maharashtra	ORF1b:D842R	1	0.15	ORF1b:N347I	1	0.13	ORF1b:R1577*	2	0.3	ORF1b:Q1415Y	4	0.11
Maharashtra	ORF1b:P859D	1	0.15	ORF1b:S2027L	1	0.13	ORF1b:L1578I	2	0.3	ORF1b:G1417*	4	0.11
Maharashtra	ORF1b:L860*	1	0.15	ORF1b:R188Q	1	0.13	ORF1b:S1580K	2	0.3	ORF1b:V1418P	4	0.11
Maharashtra	ORF1b:H883S	1	0.15	ORF1b:K421R	1	0.13	ORF1b:M1581L	2	0.3	ORF1b:V1419C	4	0.11
Maharashtra	ORF1b:D884F	1	0.15	ORF1b:G814C	1	0.13	ORF1b:R1622S	2	0.3	ORF1b:R1420L	4	0.11
Maharashtra	ORF1b:E885V	1	0.15	ORF1b:T1540I	1	0.13	ORF1b:E1623M	2	0.3	ORF1b:N1439K	4	0.11
Maharashtra	ORF1b:G888I	1	0.15	ORF1b:E1124A	1	0.13	ORF1b:A1624S	2	0.3	ORF1b:R1483H	4	0.11
Maharashtra	ORF1b:M890K	1	0.15	ORF1b:E1184D	1	0.13	ORF1b:V1625R	2	0.3	ORF1b:F1484*	4	0.11
Maharashtra	ORF1b:L891K	1	0.15	ORF1b:M592I	1	0.13	ORF1b:T1627V	2	0.3	ORF1b:V1486S	4	0.11
Maharashtra	ORF1b:D892A	1	0.15	ORF1b:V1271L	1	0.13	ORF1b:N1628M	2	0.3	ORF1b:A1487S	4	0.11
Maharashtra	ORF1b:M893T	1	0.15	ORF1b:V2223L	1	0.13	ORF1b:P1630F	2	0.3	ORF1b:I1488L	4	0.11
Maharashtra	ORF1b:Y894*	1	0.15	ORF1b:T385M	1	0.13	ORF1b:D1650L	2	0.3	ORF1b:T1489L	4	0.11
Maharashtra	ORF1b:S895*	1	0.15	ORF1b:D436N	1	0.13	ORF1b:P1652V	2	0.3	ORF1b:R1490*	4	0.11
Maharashtra	ORF1b:M897V	1	0.15	ORF1b:L1099F	1	0.13	ORF1b:N1653L	2	0.3	ORF1b:A1491C	4	0.11
Maharashtra	ORF1b:M915*	1	0.15	ORF1b:K2231N	1	0.13	ORF1b:Q1692G	2	0.3	ORF1b:V1493Q	4	0.11
Maharashtra	ORF1b:Y916G	1	0.15	ORF1b:E2266A	1	0.13	ORF1b:L1694I	2	0.3	ORF1b:G1494I	4	0.11
Maharashtra	ORF1b:T917Y	1	0.15	ORF1b:P2394S	1	0.13	ORF1b:D1696L	2	0.3	ORF1b:I1495*	4	0.11
Maharashtra	ORF1b:P918V	1	0.15	ORF1b:K2115E	1	0.13	ORF1b:L1698E	2	0.3	ORF1b:L1496C	4	0.11
Maharashtra	ORF1b:V921A	1	0.15	ORF1b:C688F	1	0.13	ORF1b:K1699S	2	0.3	ORF1b:R1576N	4	0.11
Maharashtra	ORF1b:L922Y	1	0.15	ORF1b:G662S	1	0.13	ORF1b:N1700Y	2	0.3	ORF1b:R1577E	4	0.11
Maharashtra	ORF1b:Q923S	1	0.15	ORF1b:I1257V	1	0.13	ORF1b:H1712Y	2	0.3	ORF1b:I1579S	4	0.11
Maharashtra	ORF1b:A924L	1	0.15	ORF1b:A167T	1	0.13	ORF1b:F1714H	2	0.3	ORF1b:P1666T	4	0.11
Maharashtra	ORF1b:V925T	1	0.15	ORF1b:V1133F	1	0.13	ORF1b:E1715M	2	0.3	ORF1b:G1667Y	4	0.11
Maharashtra	ORF1b:A927C	1	0.15	ORF1b:R1315C	1	0.13	ORF1b:L1716A	2	0.3	ORF1b:D1668V	4	0.11
Maharashtra	ORF1b:C928W	1	0.15	ORF1b:Q1441H	1	0.13	ORF1b:T1717L	2	0.3	ORF1b:F1670R	4	0.11
Maharashtra	ORF1b:V929G	1	0.15	ORF1b:S1745P	1	0.13	ORF1b:M1719*	2	0.3	ORF1b:K1671T	4	0.11
Maharashtra	ORF1b:R938Q	1	0.15	ORF1b:L1551F	1	0.13	ORF1b:R1736L	2	0.3	ORF1b:H1672S	4	0.11
Maharashtra	ORF1b:C939F	1	0.15	ORF1b:M1586I	1	0.13	ORF1b:R1737S	2	0.3	ORF1b:I1674E	4	0.11
Maharashtra	ORF1b:G940T	1	0.15	ORF1b:A1643V	1	0.13	ORF1b:F1757L	2	0.3	ORF1b:P1675C	4	0.11
Maharashtra	ORF1b:A941D	1	0.15	ORF1b:T17I	1	0.13	ORF1b:D1758M	2	0.3	ORF1b:L1676S	4	0.11
Maharashtra	ORF1b:C942F	1	0.15	ORF1b:V2691I	1	0.13	ORF1b:Y1759P	2	0.3	ORF1b:M1677A	4	0.11
Maharashtra	ORF1b:R944L	1	0.15	ORF1b:A37T	1	0.13	ORF1b:Y1761N	2	0.3	ORF1b:F1714T	4	0.11
Maharashtra	ORF1b:D955C	1	0.15	ORF1b:I2286V	1	0.13	ORF1b:A1798M	2	0.3	ORF1b:E1715W	4	0.11
Maharashtra	ORF1b:V957I	1	0.15	ORF1b:R1171I	1	0.13	ORF1b:I1799V	2	0.3	ORF1b:T1717*	4	0.11
Maharashtra	ORF1b:I958N	1	0.15	ORF1b:A1476V	1	0.13	ORF1b:T1801H	2	0.3	ORF1b:S1718V	4	0.11
Maharashtra	ORF1b:S959I	1	0.15	ORF1b:M746I	1	0.13	ORF1b:R1802M	2	0.3	ORF1b:M1719D	4	0.11
Maharashtra	ORF1b:S961*	1	0.15	ORF1b:T2084I	1	0.13	ORF1b:C1803*	2	0.3	ORF1b:K1720I	4	0.11
Maharashtra	ORF1b:H962I	1	0.15	ORF1b:A520V	1	0.13	ORF1b:A1805V	2	0.3	ORF1b:F1722E	4	0.11
Maharashtra	ORF1b:K963S	1	0.15	ORF1b:M115I	1	0.13	ORF1b:D1825G	2	0.3	ORF1b:C1731F	4	0.11
Maharashtra	ORF1b:L966C	1	0.15	ORF1b:V2458A	1	0.13	ORF1b:E1826L	2	0.3	ORF1b:L1733E	4	0.11
Maharashtra	ORF1b:S967*	1	0.15	ORF1b:K82E	1	0.13	ORF1b:A1832W	2	0.3	ORF1b:C1734N	4	0.11
Maharashtra	ORF1b:V968S	1	0.15	ORF1b:L2213F	1	0.13	ORF1b:C1833L	2	0.3	ORF1b:D1735R	4	0.11
Maharashtra	ORF1b:N969V	1	0.15	ORF1b:A2431V	1	0.13	ORF1b:R1834L	2	0.3	ORF1b:R1736T	4	0.11
Maharashtra	ORF1b:P970C	1	0.15	ORF1b:P2477L	1	0.13	ORF1b:E1871Y	2	0.3	ORF1b:R1737*	4	0.11
Maharashtra	ORF1b:Y971L	1	0.15	ORF1b:G1129C	1	0.13	ORF1b:W1872L	2	0.3	ORF1b:T1739H	4	0.11
Maharashtra	ORF1b:V972Q	1	0.15	ORF1b:K2310R	1	0.13	ORF1b:F1874L	2	0.3	ORF1b:C1740L	4	0.11
Maharashtra	ORF1b:N974S	1	0.15	ORF1b:S2312A	1	0.13	ORF1b:Y1875V	2	0.3	ORF1b:F1741L	4	0.11
Maharashtra	ORF1b:A975R	1	0.15	ORF1b:A1895V	1	0.13	ORF1b:D1876V	2	0.3	ORF1b:T1743L	4	0.11
Maharashtra	ORF1b:P976L	1	0.15	ORF1b:P2116S	1	0.13	ORF1b:A1877T	2	0.3	ORF1b:T1774*	4	0.11
Maharashtra	ORF1b:G977*	1	0.15	ORF1b:T1111N	1	0.13	ORF1b:Q1878K	2	0.3	ORF1b:G1775S	4	0.11
Maharashtra	ORF1b:D979H	1	0.15	ORF1b:A2143S	1	0.13	ORF1b:P1879L	2	0.3	ORF1b:Q1778S	4	0.11
Maharashtra	ORF1b:V980R	1	0.15	ORF1b:T1599A	1	0.13	ORF1b:C1880I	2	0.3	ORF1b:N1780P	4	0.11
Maharashtra	ORF1b:T981C	1	0.15	ORF1b:P218L	1	0.13	ORF1b:S1881K	2	0.3	ORF1b:H1781W	4	0.11
Maharashtra	ORF1b:V983S	1	0.15	ORF1b:G1568C	1	0.13	ORF1b:D1882*	2	0.3	ORF1b:D1782*	4	0.11
Maharashtra	ORF1b:Q985L	1	0.15	ORF1b:V2120A	1	0.13	ORF1b:K1886S	2	0.3	ORF1b:L1783C	4	0.11
Maharashtra	ORF1b:Y987L	1	0.15	ORF1b:V2371L	1	0.13	ORF1b:F1891M	2	0.3	ORF1b:Y1784T	4	0.11
Maharashtra	ORF1b:A1008W	1	0.15	ORF1b:K2097R	1	0.13	ORF1b:Y1892P	2	0.3	ORF1b:Q1786S	4	0.11
Maharashtra	ORF1b:N1009*	1	0.15	ORF1b:I2568M	1	0.13	ORF1b:S1893H	2	0.3	ORF1b:V1787*	4	0.11
Maharashtra	ORF1b:G1010R	1	0.15	ORF1b:I2320T	1	0.13	ORF1b:Y1894I	2	0.3	ORF1b:H1788L	4	0.11
Maharashtra	ORF1b:Q1011*	1	0.15	ORF1b:L1220F	1	0.13	ORF1b:A1895L	2	0.3	ORF1b:G1789*	4	0.11
Maharashtra	ORF1b:V1012C	1	0.15	ORF1b:T1290I	1	0.13	ORF1b:H1897N	2	0.3	ORF1b:N1790C	4	0.11
Maharashtra	ORF1b:F1013Y	1	0.15	ORF1b:T1050N	1	0.13	ORF1b:C1911Q	2	0.3	ORF1b:A1791N	4	0.11
Maharashtra	ORF1b:G1014*	1	0.15	ORF1b:M2462I	1	0.13	ORF1b:N1912M	2	0.3	ORF1b:A1794S	4	0.11
Maharashtra	ORF1b:Y1016*	1	0.15	ORF1b:F308L	1	0.13	ORF1b:D1914Y	2	0.3	ORF1b:L2213K	4	0.11
Maharashtra	ORF1b:K1017C	1	0.15	ORF1b:R1577K	1	0.13	ORF1b:R1915A	2	0.3	ORF1b:Q2239L	4	0.11
Maharashtra	ORF1b:T1019C	1	0.15	ORF1b:P1567L	1	0.13	ORF1b:P1917I	2	0.3	ORF1b:P2241*	4	0.11
Maharashtra	ORF1b:C1020N	1	0.15	ORF1b:V1686L	1	0.13	ORF1b:N1919M	2	0.3	ORF1b:T2243R	4	0.11
Maharashtra	ORF1b:V1021M	1	0.15	ORF1b:M454I	1	0.13	ORF1b:P1001*	2	0.3	ORF1b:Y2244I	4	0.11

Maharashtra	**ORF1b:G1022***	1	0.15	**ORF1b:I1498M**	1	0.13	**ORF1b:S2338L**	2	0.3	**ORF1b:F2245***	4	0.11
Maharashtra	**ORF1b:S1023L**	1	0.15	**ORF1b:M809I**	1	0.13	**ORF1b:F803Y**	2	0.3	**ORF1b:S2248E**	4	0.11
Maharashtra	**ORF1b:T1048K**	1	0.15	**ORF1b:S2312P**	1	0.13	**ORF1b:A647S**	2	0.3	**ORF1b:R2249S**	4	0.11
Maharashtra	**ORF1b:G1073D**	1	0.15	**ORF1b:V2061L**	1	0.13	**ORF1b:L293F**	2	0.3	**ORF1b:L2251G**	4	0.11
Maharashtra	**ORF1b:A1075***	1	0.15	**ORF1b:D131Y**	1	0.13	**ORF1b:T2084I**	2	0.3	**ORF1b:Q2252N**	4	0.11
Maharashtra	**ORF1b:P1098W**	1	0.15	**ORF1b:A2407V**	1	0.13	**ORF1b:V1195I**	2	0.3	**ORF1b:E2253***	4	0.11
Maharashtra	**ORF1b:N1100S**	1	0.15	**ORF1b:R1813L**	1	0.13	**ORF1b:R2257S**	2	0.3	**ORF1b:G2297S**	4	0.11
Maharashtra	**ORF1b:R1101C**	1	0.15	**ORF1b:H1619Y**	1	0.13	**ORF1b:N1039K**	2	0.3	**ORF1b:G2298***	4	0.11
Maharashtra	**ORF1b:K1112***	1	0.15	**ORF1b:Q1589R**	1	0.13	**ORF1b:E1184D**	2	0.3	**ORF1b:L2299T**	4	0.11
Maharashtra	**ORF1b:D1869L**	1	0.15	**ORF1b:V2128M**	1	0.13	**ORF1b:R2613H**	2	0.3	**ORF1b:H2300F**	4	0.11
Maharashtra	**ORF1b:E1871V**	1	0.15	**ORF1b:L621F**	1	0.13	**ORF1b:P976S**	2	0.3	**ORF1b:L2301***	4	0.11
Maharashtra	**ORF1b:W1872T**	1	0.15	**ORF1b:I1488T**	1	0.13	**ORF1b:K469N**	2	0.3	**ORF1b:L2302G**	4	0.11
Maharashtra	**ORF1b:K1886L**	1	0.15	**ORF1b:Q2635H**	1	0.13	**ORF1b:R1737L**	2	0.3	**ORF1b:G2304T**	4	0.11
Maharashtra	**ORF1b:E1888K**	1	0.15	**ORF1b:I192V**	1	0.13	**ORF1b:P2313L**	2	0.3	**ORF1b:L2305F**	4	0.11
Maharashtra	**ORF1b:E1889***	1	0.15	**ORF1b:H346Y**	1	0.13	**ORF1b:P1223L**	2	0.3	**ORF1b:A2306***	4	0.11
Maharashtra	**ORF1b:L1890K**	1	0.15	**ORF1b:I1227M**	1	0.13	**ORF1b:T1404I**	2	0.3	**ORF1b:K2307I**	4	0.11
Maharashtra	**ORF1b:F1891N**	1	0.15	**ORF1b:S2338L**	1	0.13	**ORF1b:K1507R**	2	0.3	**ORF1b:F2309R**	4	0.11
Maharashtra	**ORF1b:D1914Y**	1	0.15	**ORF1b:R240G**	1	0.13	**ORF1b:A2334V**	1	0.15	**ORF1b:K2310F**	4	0.11
Maharashtra	**ORF1b:R1915F**	1	0.15	**ORF1b:Y868F**	1	0.13	**ORF1b:N1946D**	1	0.15	**ORF1b:E2311Y**	4	0.11
Maharashtra	**ORF1b:Y1916G**	1	0.15	**ORF1b:S1898F**	1	0.13	**ORF1b:K82R**	1	0.15	**ORF1b:C2341***	4	0.11
Maharashtra	**ORF1b:P1917I**	1	0.15	**ORF1b:S1159I**	1	0.13	**ORF1b:R2308C**	1	0.15	**ORF1b:D2352V**	4	0.11
Maharashtra	**ORF1b:N1919M**	1	0.15	**ORF1b:V2073L**	1	0.13	**ORF1b:A2211V**	1	0.15	**ORF1b:V2354P**	4	0.11
Maharashtra	**ORF1b:N1932D**	1	0.15	**ORF1b:A434S**	1	0.13	**ORF1b:L2213F**	1	0.15	**ORF1b:E2355R**	4	0.11
Maharashtra	**ORF1b:N1934T**	1	0.15	**ORF1b:L966F**	1	0.13	**ORF1b:A1160V**	1	0.15	**ORF1b:I2356F**	4	0.11
Maharashtra	**ORF1b:P1953S**	1	0.15	**ORF1b:P1000L**	1	0.13	**ORF1b:T2537I**	1	0.15	**ORF1b:K2358C**	4	0.11
Maharashtra	**ORF1b:A1954F**	1	0.15				**ORF1b:V1092F**	1	0.15	**ORF1b:I1539T**	4	0.11
Maharashtra	**ORF1b:F1955***	1	0.15				**ORF1b:L273Q**	1	0.15	**ORF1b:L966F**	4	0.11
Maharashtra	**ORF1b:D1956***	1	0.15				**ORF1b:D275G**	1	0.15	**ORF1b:G1667R**	4	0.11
Maharashtra	**ORF1b:S1958T**	1	0.15				**ORF1b:R276W**	1	0.15	**ORF1b:V2117L**	4	0.11
Maharashtra	**ORF1b:A1959I**	1	0.15				**ORF1b:F278V**	1	0.15	**ORF1b:V2515I**	4	0.11
Maharashtra	**ORF1b:V1961L**	1	0.15				**ORF1b:K279***	1	0.15	**ORF1b:L1641I**	4	0.11
Maharashtra	**ORF1b:N1962L**	1	0.15				**ORF1b:Y280S**	1	0.15	**ORF1b:T1404M**	4	0.11
Maharashtra	**ORF1b:K1964***	1	0.15				**ORF1b:W281Q**	1	0.15	**ORF1b:Y1176H**	4	0.11
Maharashtra	**ORF1b:P1975L**	1	0.15				**ORF1b:D282Y**	1	0.15	**ORF1b:T1637I**	4	0.11
Maharashtra	**ORF1b:L2649F**	1	0.15				**ORF1b:Q283***	1	0.15	**ORF1b:A1369S**	4	0.11
Maharashtra	**ORF1b:L2508F**	1	0.15				**ORF1b:H286R**	1	0.15	**ORF1b:D1869Y**	4	0.11
Maharashtra	**ORF1b:M454I**	1	0.15				**ORF1b:P287S**	1	0.15	**ORF1b:D870Y**	4	0.11
Maharashtra	**ORF1b:K429A**	1	0.15				**ORF1b:C289S**	1	0.15	**ORF1b:V1983M**	4	0.11
Maharashtra	**ORF1b:W1683***	1	0.15				**ORF1b:V290F**	1	0.15	**ORF1b:S1985A**	4	0.11
Maharashtra	**ORF1b:A2211T**	1	0.15				**ORF1b:F317S**	1	0.15	**ORF1b:D1986V**	4	0.11
Maharashtra	**ORF1b:T284I**	1	0.15				**ORF1b:G336S**	1	0.15	**ORF1b:I1987C**	4	0.11
Maharashtra	**ORF1b:V1144F**	1	0.15				**ORF1b:H338F**	1	0.15	**ORF1b:Y1989C**	4	0.11
Maharashtra	**ORF1b:V1320L**	1	0.15				**ORF1b:L1733P**	1	0.15	**ORF1b:M1499I**	4	0.11
Maharashtra	**ORF1b:I1856K**	1	0.15				**ORF1b:R1736K**	1	0.15	**ORF1b:V1170F**	4	0.11
Maharashtra	**ORF1b:M2025I**	1	0.15				**ORF1b:R1737Y**	1	0.15	**ORF1b:L2560F**	4	0.11
Maharashtra	**ORF1b:P2118T**	1	0.15				**ORF1b:T1743S**	1	0.15	**ORF1b:D1939Y**	4	0.11
Maharashtra	**ORF1b:A2211P**	1	0.15				**ORF1b:A1744V**	1	0.15	**ORF1b:G799R**	4	0.11
Maharashtra	**ORF1b:V1660I**	1	0.15				**ORF1b:S1745I**	1	0.15	**ORF1b:I235T**	4	0.11
Maharashtra	**ORF1b:V839L**	1	0.15				**ORF1b:D1746E**	1	0.15	**ORF1b:T2048I**	4	0.11
Maharashtra	**ORF1b:M1573I**	1	0.15				**ORF1b:T1747I**	1	0.15	**ORF1b:A1525S**	4	0.11
Maharashtra	**ORF1b:M371I**	1	0.15				**ORF1b:Y1748F**	1	0.15	**ORF1b:Q1546H**	4	0.11
Maharashtra							**ORF1b:A1749R**	1	0.15	**ORF1b:K2063N**	4	0.11
Maharashtra							**ORF1b:H90Q**	1	0.15	**ORF1b:A1643V**	4	0.11
Maharashtra							**ORF1b:F433L**	1	0.15	**ORF1b:D2270Y**	4	0.11
Maharashtra							**ORF1b:V767A**	1	0.15	**ORF1b:V1814F**	4	0.11
Maharashtra							**ORF1b:G2029C**	1	0.15	**ORF1b:D2183N**	3	0.08
Maharashtra							**ORF1b:S2031R**	1	0.15	**ORF1b:E885D**	3	0.08
Maharashtra							**ORF1b:H2388Y**	1	0.15	**ORF1b:F1722C**	3	0.08
Maharashtra							**ORF1b:V2389L**	1	0.15	**ORF1b:P85L**	3	0.08
Maharashtra							**ORF1b:I324M**	1	0.15	**ORF1b:A520V**	3	0.08
Maharashtra							**ORF1b:V333F**	1	0.15	**ORF1b:I257V**	3	0.08
Maharashtra							**ORF1b:G336V**	1	0.15	**ORF1b:T15I**	3	0.08
Maharashtra							**ORF1b:Y337S**	1	0.15	**ORF1b:V225L**	3	0.08
Maharashtra							**ORF1b:F339L**	1	0.15	**ORF1b:A1831T**	3	0.08
Maharashtra							**ORF1b:R340Q**	1	0.15	**ORF1b:H801Y**	3	0.08
Maharashtra							**ORF1b:V1149L**	1	0.15	**ORF1b:Y1344H**	3	0.08
Maharashtra							**ORF1b:F2272V**	1	0.15	**ORF1b:S2292I**	3	0.08
Maharashtra							**ORF1b:P2404M**	1	0.15	**ORF1b:T797A**	3	0.08
Maharashtra							**ORF1b:W2628S**	1	0.15	**ORF1b:L1681F**	3	0.08
Maharashtra							**ORF1b:R2629Q**	1	0.15	**ORF1b:G669Q**	3	0.08
Maharashtra							**ORF1b:A1131S**	1	0.15	**ORF1b:G670L**	3	0.08
Maharashtra							**ORF1b:T1339S**	1	0.15	**ORF1b:T671L**	3	0.08
Maharashtra							**ORF1b:D1825Y**	1	0.15	**ORF1b:S672M**	3	0.08

Maharashtra							**ORF1b:I2158L**	1	0.15	**ORF1b:S673L**	3	0.08
Maharashtra							**ORF1b:K50N**	1	0.15	**ORF1b:G674I**	3	0.08
Maharashtra							**ORF1b:V839L**	1	0.15	**ORF1b:D675V**	3	0.08
Maharashtra							**ORF1b:P1368S**	1	0.15	**ORF1b:A676F**	3	0.08
Maharashtra							**ORF1b:A1369S**	1	0.15	**ORF1b:T677L**	3	0.08
Maharashtra							**ORF1b:I1371L**	1	0.15	**ORF1b:N694F**	3	0.08
Maharashtra							**ORF1b:D1373G**	1	0.15	**ORF1b:Q715V**	3	0.08
Maharashtra							**ORF1b:T1374V**	1	0.15	**ORF1b:H716S**	3	0.08
Maharashtra							**ORF1b:S1376L**	1	0.15	**ORF1b:R717I**	3	0.08
Maharashtra							**ORF1b:A1377K**	1	0.15	**ORF1b:L718E**	3	0.08
Maharashtra							**ORF1b:L1378H**	1	0.15	**ORF1b:Y719I**	3	0.08
Maharashtra							**ORF1b:V1379I**	1	0.15	**ORF1b:C721M**	3	0.08
Maharashtra							**ORF1b:R1430***	1	0.15	**ORF1b:Y737T**	3	0.08
Maharashtra							**ORF1b:K1431F**	1	0.15	**ORF1b:A738Q**	3	0.08
Maharashtra							**ORF1b:A1432T**	1	0.15	**ORF1b:Y739T**	3	0.08
Maharashtra							**ORF1b:V1433E**	1	0.15	**ORF1b:R741***	3	0.08
Maharashtra							**ORF1b:F1434C**	1	0.15	**ORF1b:K742M**	3	0.08
Maharashtra							**ORF1b:I1435C**	1	0.15	**ORF1b:H743S**	3	0.08
Maharashtra							**ORF1b:S1446L**	1	0.15	**ORF1b:S745L**	3	0.08
Maharashtra							**ORF1b:I1448D**	1	0.15	**ORF1b:M746T**	3	0.08
Maharashtra							**ORF1b:L1449F**	1	0.15	**ORF1b:I748L**	3	0.08
Maharashtra							**ORF1b:F1925A**	1	0.15	**ORF1b:L766C**	3	0.08
Maharashtra							**ORF1b:D1926I**	1	0.15	**ORF1b:A768F**	3	0.08
Maharashtra							**ORF1b:T1927***	1	0.15	**ORF1b:S769I**	3	0.08
Maharashtra							**ORF1b:R1928P**	1	0.15	**ORF1b:N1039K**	3	0.08
Maharashtra							**ORF1b:V1929***	1	0.15	**ORF1b:M1677I**	3	0.08
Maharashtra							**ORF1b:S1931A**	1	0.15	**ORF1b:P946V**	3	0.08
Maharashtra							**ORF1b:N1932W**	1	0.15	**ORF1b:F947N**	3	0.08
Maharashtra							**ORF1b:N1934***	1	0.15	**ORF1b:L948A**	3	0.08
Maharashtra							**ORF1b:L1935W**	1	0.15	**ORF1b:C949V**	3	0.08
Maharashtra							**ORF1b:P1936W**	1	0.15	**ORF1b:C950T**	3	0.08
Maharashtra							**ORF1b:G1937Q**	1	0.15	**ORF1b:K951T**	3	0.08
Maharashtra							**ORF1b:C1938F**	1	0.15	**ORF1b:C952M**	3	0.08
Maharashtra							**ORF1b:M2024W**	1	0.15	**ORF1b:G1010T**	3	0.08
Maharashtra							**ORF1b:M2025L**	1	0.15	**ORF1b:Q1011H**	3	0.08
Maharashtra							**ORF1b:I2026***	1	0.15	**ORF1b:V1012***	3	0.08
Maharashtra							**ORF1b:S2027L**	1	0.15	**ORF1b:G1014***	3	0.08
Maharashtra							**ORF1b:A2028V**	1	0.15	**ORF1b:L1015C**	3	0.08
Maharashtra							**ORF1b:W2044L**	1	0.15	**ORF1b:K1017***	3	0.08
Maharashtra							**ORF1b:N2045Q**	1	0.15	**ORF1b:N1018L**	3	0.08
Maharashtra							**ORF1b:F2047I**	1	0.15	**ORF1b:T1019***	3	0.08
Maharashtra							**ORF1b:T2048***	1	0.15	**ORF1b:L1055T**	3	0.08
Maharashtra							**ORF1b:R2049Y**	1	0.15	**ORF1b:F1056Q**	3	0.08
Maharashtra							**ORF1b:Q2051***	1	0.15	**ORF1b:A1058F**	3	0.08
Maharashtra							**ORF1b:S2052P**	1	0.15	**ORF1b:E1059C**	3	0.08
Maharashtra							**ORF1b:N2055K**	1	0.15	**ORF1b:T1060S**	3	0.08
Maharashtra							**ORF1b:V2056C**	1	0.15	**ORF1b:L1061R**	3	0.08
Maharashtra							**ORF1b:A2057G**	1	0.15	**ORF1b:K1062N**	3	0.08
Maharashtra							**ORF1b:N2059***	1	0.15	**ORF1b:L1070Q**	3	0.08
Maharashtra							**ORF1b:V2060C**	1	0.15	**ORF1b:G1073D**	3	0.08
Maharashtra							**ORF1b:V2061C**	1	0.15	**ORF1b:A1075***	3	0.08
Maharashtra							**ORF1b:N2062K**	1	0.15	**ORF1b:P1098K**	3	0.08
Maharashtra							**ORF1b:K2063***	1	0.15	**ORF1b:R1101***	3	0.08
Maharashtra							**ORF1b:H2065S**	1	0.15	**ORF1b:N1102K**	3	0.08
Maharashtra							**ORF1b:F2106Q**	1	0.15	**ORF1b:Y1103Q**	3	0.08
Maharashtra							**ORF1b:E2107H**	1	0.15	**ORF1b:V1104***	3	0.08
Maharashtra							**ORF1b:L2108***	1	0.15	**ORF1b:F1105S**	3	0.08
Maharashtra							**ORF1b:W2109T**	1	0.15	**ORF1b:G1107N**	3	0.08
Maharashtra							**ORF1b:A2110S**	1	0.15	**ORF1b:Y1108R**	3	0.08
Maharashtra							**ORF1b:K2111T**	1	0.15	**ORF1b:A1805S**	3	0.08
Maharashtra							**ORF1b:V2128C**	1	0.15	**ORF1b:D1825G**	3	0.08
Maharashtra							**ORF1b:I2136L**	1	0.15	**ORF1b:E1826L**	3	0.08
Maharashtra							**ORF1b:W2137G**	1	0.15	**ORF1b:K1828N**	3	0.08
Maharashtra							**ORF1b:D2138L**	1	0.15	**ORF1b:A2057D**	3	0.08
Maharashtra							**ORF1b:Y2139Q**	1	0.15	**ORF1b:N2059R**	3	0.08
Maharashtra							**ORF1b:M2155S**	1	0.15	**ORF1b:V2061R**	3	0.08
Maharashtra							**ORF1b:D2157Y**	1	0.15	**ORF1b:N2062V**	3	0.08
Maharashtra							**ORF1b:V2172N**	1	0.15	**ORF1b:G2064Y**	3	0.08
Maharashtra							**ORF1b:F2173***	1	0.15	**ORF1b:H2065Q**	3	0.08
Maharashtra							**ORF1b:F2174N**	1	0.15	**ORF1b:V2075L**	3	0.08
Maharashtra							**ORF1b:G2176S**	1	0.15	**ORF1b:V2082L**	3	0.08
Maharashtra							**ORF1b:T2195I**	1	0.15	**ORF1b:Y2083F**	3	0.08
Maharashtra							**ORF1b:E2196C**	1	0.15	**ORF1b:K2085Q**	3	0.08

Maharashtra							**ORF1b:G2197R**	1	0.15	**ORF1b:V2086K**	3	0.08
Maharashtra							**ORF1b:A2211T**	1	0.15	**ORF1b:D2087L**	3	0.08
Maharashtra							**ORF1b:L2213S**	1	0.15	**ORF1b:G2088M**	3	0.08
Maharashtra							**ORF1b:N2214R**	1	0.15	**ORF1b:P2101M**	3	0.08
Maharashtra							**ORF1b:V2216N**	1	0.15	**ORF1b:V2104L**	3	0.08
Maharashtra							**ORF1b:Q2239L**	1	0.15	**ORF1b:A2105S**	3	0.08
Maharashtra							**ORF1b:P2241***	1	0.15	**ORF1b:E2107G**	3	0.08
Maharashtra							**ORF1b:T2243R**	1	0.15	**ORF1b:W2109S**	3	0.08
Maharashtra							**ORF1b:Y2244I**	1	0.15	**ORF1b:D2138I**	3	0.08
Maharashtra							**ORF1b:F2245***	1	0.15	**ORF1b:K2140L**	3	0.08
Maharashtra							**ORF1b:S2248E**	1	0.15	**ORF1b:R2141L**	3	0.08
Maharashtra							**ORF1b:R2249S**	1	0.15	**ORF1b:D2142L**	3	0.08
Maharashtra							**ORF1b:L2251G**	1	0.15	**ORF1b:A2143V**	3	0.08
Maharashtra							**ORF1b:Q2252N**	1	0.15	**ORF1b:P2144F**	3	0.08
Maharashtra							**ORF1b:E2253***	1	0.15	**ORF1b:A2145V**	3	0.08
Maharashtra							**ORF1b:P2256R**	1	0.15	**ORF1b:H2146L**	3	0.08
Maharashtra							**ORF1b:R2257I**	1	0.15	**ORF1b:I2147***	3	0.08
Maharashtra							**ORF1b:Q2259Y**	1	0.15	**ORF1b:S2148L**	3	0.08
Maharashtra							**ORF1b:M2260G**	1	0.15	**ORF1b:A2168***	3	0.08
Maharashtra							**ORF1b:E2261***	1	0.15	**ORF1b:T2171M**	3	0.08
Maharashtra							**ORF1b:A2268H**	1	0.15	**ORF1b:L2492F**	3	0.08
Maharashtra							**ORF1b:M2269***	1	0.15	**ORF1b:D982V**	3	0.08
Maharashtra							**ORF1b:D2270T**	1	0.15	**ORF1b:V983M**	3	0.08
Maharashtra							**ORF1b:E2271V**	1	0.15	**ORF1b:T984S**	3	0.08
Maharashtra							**ORF1b:F2272***	1	0.15	**ORF1b:L986M**	3	0.08
Maharashtra							**ORF1b:E2274R**	1	0.15	**ORF1b:Y987***	3	0.08
Maharashtra							**ORF1b:Y2276L**	1	0.15	**ORF1b:S1003N**	3	0.08
Maharashtra							**ORF1b:K2277C**	1	0.15	**ORF1b:G1014V**	3	0.08
Maharashtra							**ORF1b:E2279R**	1	0.15	**ORF1b:N1030V**	3	0.08
Maharashtra							**ORF1b:G2280T**	1	0.15	**ORF1b:A1033I**	3	0.08
Maharashtra							**ORF1b:Q2295R**	1	0.15	**ORF1b:C1035F**	3	0.08
Maharashtra							**ORF1b:K2307***	1	0.15	**ORF1b:D1036***	3	0.08
Maharashtra							**ORF1b:R2308T**	1	0.15	**ORF1b:W1037L**	3	0.08
Maharashtra							**ORF1b:K2310***	1	0.15	**ORF1b:E1059P**	3	0.08
Maharashtra							**ORF1b:E2311G**	1	0.15	**ORF1b:T1060V**	3	0.08
Maharashtra							**ORF1b:S2312T**	1	0.15	**ORF1b:V1116L**	3	0.08
Maharashtra							**ORF1b:V2686T**	1	0.15	**ORF1b:G1119***	3	0.08
Maharashtra							**ORF1b:I2687T**	1	0.15	**ORF1b:Y1121S**	3	0.08
Maharashtra							**ORF1b:S2688E**	1	0.15	**ORF1b:F1123P**	3	0.08
Maharashtra							**ORF1b:S2689L**	1	0.15	**ORF1b:E1124L**	3	0.08
Maharashtra							**ORF1b:D2690L**	1	0.15	**ORF1b:K1141M**	3	0.08
Maharashtra							**ORF1b:V2691F**	1	0.15	**ORF1b:P1157I**	3	0.08
Maharashtra							**ORF1b:T1540I**	1	0.15	**ORF1b:G1174M**	3	0.08
Maharashtra							**ORF1b:V1945***	1	0.15	**ORF1b:P1177T**	3	0.08
Maharashtra							**ORF1b:K1947T**	1	0.15	**ORF1b:T1178Q**	3	0.08
Maharashtra							**ORF1b:K1981S**	1	0.15	**ORF1b:L1179H**	3	0.08
Maharashtra							**ORF1b:Q1982M**	1	0.15	**ORF1b:N1180S**	3	0.08
Maharashtra							**ORF1b:V1983***	1	0.15	**ORF1b:F1185Q**	3	0.08
Maharashtra							**ORF1b:D1986R**	1	0.15	**ORF1b:S1186M**	3	0.08
Maharashtra							**ORF1b:I1987Y**	1	0.15	**ORF1b:N1188F**	3	0.08
Maharashtra							**ORF1b:D1988R**	1	0.15	**ORF1b:V1189L**	3	0.08
Maharashtra							**ORF1b:Y1989L**	1	0.15	**ORF1b:N1191M**	3	0.08
Maharashtra							**ORF1b:V1990***	1	0.15	**ORF1b:Y1192L**	3	0.08
Maharashtra							**ORF1b:E1977D**	1	0.15	**ORF1b:T1202I**	3	0.08
Maharashtra							**ORF1b:A2585S**	1	0.15	**ORF1b:F1214S**	3	0.08
Maharashtra							**ORF1b:V1945I**	1	0.15	**ORF1b:A1215R**	3	0.08
Maharashtra							**ORF1b:R524H**	1	0.15	**ORF1b:I1216D**	3	0.08
Maharashtra							**ORF1b:D2234I**	1	0.15	**ORF1b:G1217H**	3	0.08
Maharashtra							**ORF1b:G2235H**	1	0.15	**ORF1b:A1219V**	3	0.08
Maharashtra							**ORF1b:N347I**	1	0.15	**ORF1b:V1237Q**	3	0.08
Maharashtra							**ORF1b:Q348C**	1	0.15	**ORF1b:D1238L**	3	0.08
Maharashtra							**ORF1b:D349***	1	0.15	**ORF1b:C1241M**	3	0.08
Maharashtra							**ORF1b:G2582E**	1	0.15	**ORF1b:E1242P**	3	0.08
Maharashtra							**ORF1b:G2207A**	1	0.15	**ORF1b:K1243L**	3	0.08
Maharashtra							**ORF1b:M1596I**	1	0.15	**ORF1b:A1244L**	3	0.08
Maharashtra							**ORF1b:G2582R**	1	0.15	**ORF1b:L1245M**	3	0.08
Maharashtra							**ORF1b:H2583S**	1	0.15	**ORF1b:K1246H**	3	0.08
Maharashtra							**ORF1b:F2584V**	1	0.15	**ORF1b:P1249***	3	0.08
Maharashtra							**ORF1b:H641Y**	1	0.15	**ORF1b:A1261N**	3	0.08
Maharashtra							**ORF1b:L2382I**	1	0.15	**ORF1b:R1262V**	3	0.08
Maharashtra							**ORF1b:L2558***	1	0.15	**ORF1b:C1265L**	3	0.08
Maharashtra							**ORF1b:S1089***	1	0.15	**ORF1b:F1266Y**	3	0.08
Maharashtra							**ORF1b:V1961F**	1	0.15	**ORF1b:D1267L**	3	0.08

Maharashtra							**ORF1b:E2261K**	1	0.15	**ORF1b:K1268H**	3	0.08
Maharashtra							**ORF1b:S1003G**	1	0.15	**ORF1b:F1269V**	3	0.08
Maharashtra							**ORF1b:D2183N**	1	0.15	**ORF1b:K1270L**	3	0.08
Maharashtra							**ORF1b:L2350F**	1	0.15	**ORF1b:N1272***	3	0.08
Maharashtra							**ORF1b:D2351I**	1	0.15	**ORF1b:T1274V**	3	0.08
Maharashtra							**ORF1b:D2352N**	1	0.15	**ORF1b:E1276M**	3	0.08
Maharashtra							**ORF1b:F325L**	1	0.15	**ORF1b:Q1277H**	3	0.08
Maharashtra							**ORF1b:V326F**	1	0.15	**ORF1b:Y1278C**	3	0.08
Maharashtra							**ORF1b:T797M**	1	0.15	**ORF1b:V1279L**	3	0.08
Maharashtra							**ORF1b:K798T**	1	0.15	**ORF1b:F1280R**	3	0.08
Maharashtra							**ORF1b:D126N**	1	0.15	**ORF1b:C1281R**	3	0.08
Maharashtra							**ORF1b:D2576Y**	1	0.15	**ORF1b:T1282Q**	3	0.08
Maharashtra							**ORF1b:D1127Y**	1	0.15	**ORF1b:V1283Q**	3	0.08
Maharashtra							**ORF1b:T2548I**	1	0.15	**ORF1b:N1284I**	3	0.08
Maharashtra							**ORF1b:S220N**	1	0.15	**ORF1b:A1285***	3	0.08
Maharashtra							**ORF1b:V2656I**	1	0.15	**ORF1b:D1306V**	3	0.08
Maharashtra							**ORF1b:M1499I**	1	0.15	**ORF1b:Q1327T**	3	0.08
Maharashtra							**ORF1b:P1682S**	1	0.15	**ORF1b:L1328M**	3	0.08
Maharashtra							**ORF1b:G170C**	1	0.15	**ORF1b:P1329C**	3	0.08
Maharashtra							**ORF1b:A1798S**	1	0.15	**ORF1b:A1330T**	3	0.08
Maharashtra							**ORF1b:P1427L**	1	0.15	**ORF1b:P1331L**	3	0.08
Maharashtra							**ORF1b:G128D**	1	0.15	**ORF1b:R1332A**	3	0.08
Maharashtra							**ORF1b:P1570S**	1	0.15	**ORF1b:V1379I**	3	0.08
Maharashtra							**ORF1b:M657V**	1	0.15	**ORF1b:Y1380K**	3	0.08
Maharashtra							**ORF1b:S1942C**	1	0.15	**ORF1b:D1381T**	3	0.08
Maharashtra							**ORF1b:Y1944V**	1	0.15	**ORF1b:K1383Q**	3	0.08
Maharashtra							**ORF1b:K1947M**	1	0.15	**ORF1b:K1385N**	3	0.08
Maharashtra							**ORF1b:A1949S**	1	0.15	**ORF1b:H1387L**	3	0.08
Maharashtra							**ORF1b:F1950T**	1	0.15	**ORF1b:K1396M**	3	0.08
Maharashtra							**ORF1b:F2030A**	1	0.15	**ORF1b:Y1399H**	3	0.08
Maharashtra							**ORF1b:S2031***	1	0.15	**ORF1b:K1400L**	3	0.08
Maharashtra							**ORF1b:W2033V**	1	0.15	**ORF1b:G1401Q**	3	0.08
Maharashtra							**ORF1b:R945D**	1	0.15	**ORF1b:V1402L**	3	0.08
Maharashtra							**ORF1b:P946V**	1	0.15	**ORF1b:I1403T**	3	0.08
Maharashtra							**ORF1b:F947V**	1	0.15	**ORF1b:T1404G**	3	0.08
Maharashtra							**ORF1b:C949V**	1	0.15	**ORF1b:N1426K**	3	0.08
Maharashtra							**ORF1b:C950T**	1	0.15	**ORF1b:P1427***	3	0.08
Maharashtra							**ORF1b:K951T**	1	0.15	**ORF1b:G1450K**	3	0.08
Maharashtra							**ORF1b:C952M**	1	0.15	**ORF1b:V1467L**	3	0.08
Maharashtra							**ORF1b:C953S**	1	0.15	**ORF1b:R1576K**	3	0.08
Maharashtra							**ORF1b:D955Q**	1	0.15	**ORF1b:R1577***	3	0.08
Maharashtra							**ORF1b:V957H**	1	0.15	**ORF1b:L1578I**	3	0.08
Maharashtra							**ORF1b:S959N**	1	0.15	**ORF1b:S1580K**	3	0.08
Maharashtra							**ORF1b:T960***	1	0.15	**ORF1b:M1581L**	3	0.08
Maharashtra							**ORF1b:H962C**	1	0.15	**ORF1b:R1622S**	3	0.08
Maharashtra							**ORF1b:K963L**	1	0.15	**ORF1b:E1623M**	3	0.08
Maharashtra							**ORF1b:V965M**	1	0.15	**ORF1b:A1624S**	3	0.08
Maharashtra							**ORF1b:S967Q**	1	0.15	**ORF1b:V1625R**	3	0.08
Maharashtra							**ORF1b:T981***	1	0.15	**ORF1b:T1627V**	3	0.08
Maharashtra							**ORF1b:D982E**	1	0.15	**ORF1b:N1628M**	3	0.08
Maharashtra							**ORF1b:T984***	1	0.15	**ORF1b:P1630F**	3	0.08
Maharashtra							**ORF1b:Q985A**	1	0.15	**ORF1b:D1650L**	3	0.08
Maharashtra							**ORF1b:S1003***	1	0.15	**ORF1b:Q1692G**	3	0.08
Maharashtra							**ORF1b:P1005S**	1	0.15	**ORF1b:L1694I**	3	0.08
Maharashtra							**ORF1b:L1006I**	1	0.15	**ORF1b:D1696L**	3	0.08
Maharashtra							**ORF1b:P2256L**	1	0.15	**ORF1b:L1698E**	3	0.08
Maharashtra							**ORF1b:S2258K**	1	0.15	**ORF1b:K1699S**	3	0.08
Maharashtra							**ORF1b:Q2259W**	1	0.15	**ORF1b:N1700Y**	3	0.08
Maharashtra							**ORF1b:M2260K**	1	0.15	**ORF1b:V1905C**	3	0.08
Maharashtra							**ORF1b:E2261L**	1	0.15	**ORF1b:C1906H**	3	0.08
Maharashtra							**ORF1b:D2263S**	1	0.15	**ORF1b:L1907T**	3	0.08
Maharashtra							**ORF1b:F2264***	1	0.15	**ORF1b:W1909***	3	0.08
Maharashtra							**ORF1b:L2265N**	1	0.15	**ORF1b:C1911I**	3	0.08
Maharashtra							**ORF1b:E2266***	1	0.15	**ORF1b:N1912H**	3	0.08
Maharashtra							**ORF1b:A2268W**	1	0.15	**ORF1b:V1913R**	3	0.08
Maharashtra							**ORF1b:D2270N**	1	0.15	**ORF1b:D1914W**	3	0.08
Maharashtra							**ORF1b:E2271S**	1	0.15	**ORF1b:R1915C**	3	0.08
Maharashtra							**ORF1b:F2272L**	1	0.15	**ORF1b:Y1916M**	3	0.08
Maharashtra							**ORF1b:T2336V**	1	0.15	**ORF1b:A1918I**	3	0.08
Maharashtra							**ORF1b:G2337H**	1	0.15	**ORF1b:N1919L**	3	0.08
Maharashtra							**ORF1b:K2340V**	1	0.15	**ORF1b:S1920E**	3	0.08
Maharashtra							**ORF1b:S2344Y**	1	0.15	**ORF1b:I1921L**	3	0.08
Maharashtra							**ORF1b:K2358P**	1	0.15	**ORF1b:V1922Q**	3	0.08

Maharashtra							**ORF1b:S2359R**	1	0.15	**ORF1b:N1946C**	3	0.08
Maharashtra							**ORF1b:Q2360F**	1	0.15	**ORF1b:H1948C**	3	0.08
Maharashtra							**ORF1b:D2361I**	1	0.15	**ORF1b:V2128C**	3	0.08
Maharashtra							**ORF1b:L2362C**	1	0.15	**ORF1b:V2172N**	3	0.08
Maharashtra							**ORF1b:I2373F**	1	0.15	**ORF1b:F2173***	3	0.08
Maharashtra							**ORF1b:D2374***	1	0.15	**ORF1b:F2174N**	3	0.08
Maharashtra							**ORF1b:Y2375G**	1	0.15	**ORF1b:G2176S**	3	0.08
Maharashtra							**ORF1b:Q2449N**	1	0.15	**ORF1b:Q1204F**	3	0.08
Maharashtra							**ORF1b:Y2450I**	1	0.15	**ORF1b:G1205Y**	3	0.08
Maharashtra							**ORF1b:L2451S**	1	0.15	**ORF1b:P1206T**	3	0.08
Maharashtra							**ORF1b:N2452C**	1	0.15	**ORF1b:G1210S**	3	0.08
Maharashtra							**ORF1b:G2468***	1	0.15	**ORF1b:K1211L**	3	0.08
Maharashtra							**ORF1b:A2469Y**	1	0.15	**ORF1b:S1212L**	3	0.08
Maharashtra							**ORF1b:G2470E**	1	0.15	**ORF1b:H1213P**	3	0.08
Maharashtra							**ORF1b:D2472K**	1	0.15	**ORF1b:V1279C**	3	0.08
Maharashtra							**ORF1b:K2473T**	1	0.15	**ORF1b:F1280K**	3	0.08
Maharashtra							**ORF1b:G2474V**	1	0.15	**ORF1b:V1294A**	3	0.08
Maharashtra							**ORF1b:P2477Y**	1	0.15	**ORF1b:V1295***	3	0.08
Maharashtra							**ORF1b:T2479Y**	1	0.15	**ORF1b:F1296D**	3	0.08
Maharashtra							**ORF1b:V2481A**	1	0.15	**ORF1b:L1334C**	3	0.08
Maharashtra							**ORF1b:L2482C**	1	0.15	**ORF1b:I1569H**	3	0.08
Maharashtra							**ORF1b:V2501R**	1	0.15	**ORF1b:K1571Y**	3	0.08
Maharashtra							**ORF1b:D2503L**	1	0.15	**ORF1b:M1573G**	3	0.08
Maharashtra							**ORF1b:A2504C**	1	0.15	**ORF1b:T1574F**	3	0.08
Maharashtra							**ORF1b:D2505N**	1	0.15	**ORF1b:Y1575***	3	0.08
Maharashtra							**ORF1b:S2506C**	1	0.15	**ORF1b:E1601***	3	0.08
Maharashtra							**ORF1b:I2525Y**	1	0.15	**ORF1b:E1602W**	3	0.08
Maharashtra							**ORF1b:M2528***	1	0.15	**ORF1b:A1603L**	3	0.08
Maharashtra							**ORF1b:Y2529K**	1	0.15	**ORF1b:I1604P**	3	0.08
Maharashtra							**ORF1b:D2530C**	1	0.15	**ORF1b:R1605***	3	0.08
Maharashtra							**ORF1b:P2531Y**	1	0.15	**ORF1b:I1998***	3	0.08
Maharashtra							**ORF1b:T2533R**	1	0.15	**ORF1b:R2000S**	3	0.08
Maharashtra							**ORF1b:D2541***	1	0.15	**ORF1b:N2002***	3	0.08
Maharashtra							**ORF1b:S2542L**	1	0.15	**ORF1b:L2003***	3	0.08
Maharashtra							**ORF1b:K2543***	1	0.15	**ORF1b:G2004V**	3	0.08
Maharashtra							**ORF1b:E2544R**	1	0.15	**ORF1b:G2005Q**	3	0.08
Maharashtra							**ORF1b:K2557H**	1	0.15	**ORF1b:A2006I**	3	0.08
Maharashtra							**ORF1b:A2559H**	1	0.15	**ORF1b:C2008S**	3	0.08
Maharashtra							**ORF1b:G2562Y**	1	0.15	**ORF1b:S2031C**	3	0.08
Maharashtra							**ORF1b:S2563K**	1	0.15	**ORF1b:W2033L**	3	0.08
Maharashtra							**ORF1b:W2628K**	1	0.15	**ORF1b:V2104***	3	0.08
Maharashtra							**ORF1b:R2629S**	1	0.15	**ORF1b:L2123G**	3	0.08
Maharashtra							**ORF1b:S2638V**	1	0.15	**ORF1b:N2124E**	3	0.08
Maharashtra							**ORF1b:Y2639V**	1	0.15	**ORF1b:A2159D**	3	0.08
Maharashtra							**ORF1b:S2640F**	1	0.15	**ORF1b:K2160***	3	0.08
Maharashtra							**ORF1b:P2648L**	1	0.15	**ORF1b:V2236T**	3	0.08
Maharashtra							**ORF1b:K2650L**	1	0.15	**ORF1b:L2396I**	3	0.08
Maharashtra							**ORF1b:L2651C**	1	0.15	**ORF1b:Q2397***	3	0.08
Maharashtra							**ORF1b:R2652L**	1	0.15	**ORF1b:M2408T**	3	0.08
Maharashtra							**ORF1b:G2653***	1	0.15	**ORF1b:P2409G**	3	0.08
Maharashtra							**ORF1b:T2654K**	1	0.15	**ORF1b:N2410C**	3	0.08
Maharashtra							**ORF1b:A2655K**	1	0.15	**ORF1b:L2411C**	3	0.08
Maharashtra							**ORF1b:M2657K**	1	0.15	**ORF1b:V1170T**	3	0.08
Maharashtra							**ORF1b:S2670***	1	0.15	**ORF1b:R1171S**	3	0.08
Maharashtra							**ORF1b:L1378***	1	0.15	**ORF1b:I1172A**	3	0.08
Maharashtra							**ORF1b:Y1944C**	1	0.15	**ORF1b:G1174W**	3	0.08
Maharashtra							**ORF1b:V2216A**	1	0.15	**ORF1b:Y1176I**	3	0.08
Maharashtra							**ORF1b:C1020L**	1	0.15	**ORF1b:S1881I**	3	0.08
Maharashtra							**ORF1b:V1021M**	1	0.15	**ORF1b:A176V**	3	0.08
Maharashtra							**ORF1b:G1022Q**	1	0.15	**ORF1b:P1821S**	3	0.08
Maharashtra							**ORF1b:S1023L**	1	0.15	**ORF1b:A647S**	3	0.08
Maharashtra							**ORF1b:D1024Q**	1	0.15	**ORF1b:T1522I**	3	0.08
Maharashtra							**ORF1b:N1025H**	1	0.15	**ORF1b:P2612L**	3	0.08
Maharashtra							**ORF1b:D1028G**	1	0.15	**ORF1b:S961L**	3	0.08
Maharashtra							**ORF1b:F1029Q**	1	0.15	**ORF1b:Y449H**	3	0.08
Maharashtra							**ORF1b:N1030M**	1	0.15	**ORF1b:R1600S**	3	0.08
Maharashtra							**ORF1b:A1031L**	1	0.15	**ORF1b:D1381Y**	3	0.08
Maharashtra							**ORF1b:I1032V**	1	0.15	**ORF1b:V2178F**	3	0.08
Maharashtra							**ORF1b:A1033I**	1	0.15	**ORF1b:R2112L**	3	0.08
Maharashtra							**ORF1b:C1035F**	1	0.15	**ORF1b:I2114Q**	3	0.08
Maharashtra							**ORF1b:D1036***	1	0.15	**ORF1b:K2115Y**	3	0.08
Maharashtra							**ORF1b:W1037L**	1	0.15	**ORF1b:P2116Q**	3	0.08
Maharashtra							**ORF1b:E1059P**	1	0.15	**ORF1b:V2117R**	3	0.08

Maharashtra							ORF1b:T1060V	1	0.15	ORF1b:P2118*	3	0.08
Maharashtra							ORF1b:G1073C	1	0.15	ORF1b:E2119K	3	0.08
Maharashtra							ORF1b:I1074C	1	0.15	ORF1b:V2120Y	3	0.08
Maharashtra							ORF1b:A1075L	1	0.15	ORF1b:K2121S	3	0.08
Maharashtra							ORF1b:V1092H	1	0.15	ORF1b:D1656*	3	0.08
Maharashtra							ORF1b:T1453I	1	0.15	ORF1b:F1657*	3	0.08
Maharashtra							ORF1b:Q1546H	1	0.15	ORF1b:S1658Y	3	0.08
Maharashtra							ORF1b:G2004C	1	0.15	ORF1b:V1660R	3	0.08
Maharashtra							ORF1b:V1132I	1	0.15	ORF1b:A1662I	3	0.08
Maharashtra							ORF1b:V345F	1	0.15	ORF1b:K1663*	3	0.08
Maharashtra							ORF1b:M657I	1	0.15	ORF1b:P1664T	3	0.08
Maharashtra							ORF1b:F2590L	1	0.15	ORF1b:L1698*	3	0.08
Maharashtra							ORF1b:V2591I	1	0.15	ORF1b:N1700S	3	0.08
Maharashtra							ORF1b:A25L	1	0.15	ORF1b:K1873N	3	0.08
Maharashtra							ORF1b:E802G	1	0.15	ORF1b:T385M	3	0.08
Maharashtra							ORF1b:A2211S	1	0.15	ORF1b:Y337H	3	0.08
Maharashtra							ORF1b:S2212T	1	0.15	ORF1b:T1230I	3	0.08
Maharashtra							ORF1b:N2214S	1	0.15	ORF1b:T310K	3	0.08
Maharashtra							ORF1b:V2233F	1	0.15	ORF1b:K1689N	3	0.08
Maharashtra							ORF1b:D2234Q	1	0.15	ORF1b:D1389Y	3	0.08
Maharashtra							ORF1b:G2235V	1	0.15	ORF1b:E1977D	3	0.08
Maharashtra							ORF1b:V2236I	1	0.15	ORF1b:G1129F	3	0.08
Maharashtra							ORF1b:Q2238G	1	0.15	ORF1b:T582I	3	0.08
Maharashtra							ORF1b:L2240H	1	0.15	ORF1b:H1606Y	3	0.08
Maharashtra							ORF1b:P2241H	1	0.15	ORF1b:V1723L	3	0.08
Maharashtra							ORF1b:E2242Q	1	0.15	ORF1b:V1456T	3	0.08
Maharashtra							ORF1b:T2243L	1	0.15	ORF1b:D1457N	3	0.08
Maharashtra							ORF1b:I2273L	1	0.15	ORF1b:S1459H	3	0.08
Maharashtra							ORF1b:E2274*	1	0.15	ORF1b:Q1460I	3	0.08
Maharashtra							ORF1b:R2275*	1	0.15	ORF1b:G1461H	3	0.08
Maharashtra							ORF1b:M2408A	1	0.15	ORF1b:L1513V	3	0.08
Maharashtra							ORF1b:Y2427C	1	0.15	ORF1b:E1514A	3	0.08
Maharashtra							ORF1b:G2428V	1	0.15	ORF1b:P1516Y	3	0.08
Maharashtra							ORF1b:G2436D	1	0.15	ORF1b:R1518S	3	0.08
Maharashtra							ORF1b:I2437T	1	0.15	ORF1b:N1519*	3	0.08
Maharashtra							ORF1b:M2438V	1	0.15	ORF1b:V1520N	3	0.08
Maharashtra							ORF1b:M2439E	1	0.15	ORF1b:A1521S	3	0.08
Maharashtra							ORF1b:N2440Y	1	0.15	ORF1b:L1523*	3	0.08
Maharashtra							ORF1b:V2441I	1	0.15	ORF1b:Q1524E	3	0.08
Maharashtra							ORF1b:A2442L	1	0.15	ORF1b:A1525C	3	0.08
Maharashtra							ORF1b:K2443R	1	0.15	ORF1b:E1526G	3	0.08
Maharashtra							ORF1b:Y2444H	1	0.15	ORF1b:V1528F	3	0.08
Maharashtra							ORF1b:T2445S	1	0.15	ORF1b:L1531N	3	0.08
Maharashtra							ORF1b:Q2446S	1	0.15	ORF1b:F1532H	3	0.08
Maharashtra							ORF1b:Q2449L	1	0.15	ORF1b:K1533W	3	0.08
Maharashtra							ORF1b:L2451K	1	0.15	ORF1b:D1534V	3	0.08
Maharashtra							ORF1b:N2452I	1	0.15	ORF1b:C1535T	3	0.08
Maharashtra							ORF1b:T2453*	1	0.15	ORF1b:G587C	3	0.08
Maharashtra							ORF1b:L2454S	1	0.15	ORF1b:A2585S	3	0.08
Maharashtra							ORF1b:L2456S	1	0.15	ORF1b:E2390Q	3	0.08
Maharashtra							ORF1b:A2457S	1	0.15	ORF1b:K2579N	3	0.08
Maharashtra							ORF1b:F2467V	1	0.15	ORF1b:M1581I	3	0.08
Maharashtra							ORF1b:A2469N	1	0.15	ORF1b:P1001S	3	0.08
Maharashtra							ORF1b:G2470H	1	0.15	ORF1b:K82R	3	0.08
Maharashtra							ORF1b:S2471C	1	0.15	ORF1b:S638I	3	0.08
Maharashtra							ORF1b:D2472L	1	0.15	ORF1b:T132I	3	0.08
Maharashtra							ORF1b:G2474T	1	0.15	ORF1b:D729Y	3	0.08
Maharashtra							ORF1b:V2475A	1	0.15	ORF1b:H2388Y	3	0.08
Maharashtra							ORF1b:A2476V	1	0.15	ORF1b:L627F	3	0.08
Maharashtra							ORF1b:T2479A	1	0.15	ORF1b:R240S	3	0.08
Maharashtra							ORF1b:A2480T	1	0.15	ORF1b:G1338C	3	0.08
Maharashtra							ORF1b:V2481P	1	0.15	ORF1b:S1942V	3	0.08
Maharashtra							ORF1b:R2629Y	1	0.15	ORF1b:S2563T	3	0.08
Maharashtra							ORF1b:H286T	1	0.15	ORF1b:A1291V	3	0.08
Maharashtra							ORF1b:P287C	1	0.15	ORF1b:Q348H	3	0.08
Maharashtra							ORF1b:C289W	1	0.15	ORF1b:V1787F	3	0.08
Maharashtra							ORF1b:V290E	1	0.15	ORF1b:V776A	3	0.08
Maharashtra							ORF1b:N291H	1	0.15	ORF1b:R1078L	2	0.06
Maharashtra							ORF1b:L293R	1	0.15	ORF1b:R1315C	2	0.06
Maharashtra							ORF1b:D295A	1	0.15	ORF1b:S997P	2	0.06
Maharashtra							ORF1b:R296E	1	0.15	ORF1b:S1023G	2	0.06
Maharashtra							ORF1b:L299C	1	0.15	ORF1b:H1979Y	2	0.06
Maharashtra							ORF1b:C301L	1	0.15	ORF1b:G1583S	2	0.06

Maharashtra							**ORF1b:A302T**	1	0.15	**ORF1b:F2309S**	2	0.06
Maharashtra							**ORF1b:N303S**	1	0.15	**ORF1b:E735D**	2	0.06
Maharashtra							**ORF1b:R322E**	1	0.15	**ORF1b:A1219T**	2	0.06
Maharashtra							**ORF1b:V345I**	1	0.15	**ORF1b:H1213Y**	2	0.06
Maharashtra							**ORF1b:R2275C**	1	0.15	**ORF1b:N189S**	2	0.06
Maharashtra							**ORF1b:P1851S**	1	0.15	**ORF1b:T1173I**	2	0.06
Maharashtra							**ORF1b:D2511V**	1	0.15	**ORF1b:D1267Y**	2	0.06
Maharashtra							**ORF1b:N2630Q**	1	0.15	**ORF1b:V2073L**	2	0.06
Maharashtra							**ORF1b:T2631L**	1	0.15	**ORF1b:A1521V**	2	0.06
Maharashtra							**ORF1b:P2633W**	1	0.15	**ORF1b:E802I**	2	0.06
Maharashtra							**ORF1b:M2644I**	1	0.15	**ORF1b:L1701F**	2	0.06
Maharashtra							**ORF1b:S2645T**	1	0.15	**ORF1b:R712H**	2	0.06
Maharashtra							**ORF1b:K2646I**	1	0.15	**ORF1b:T917A**	2	0.06
Maharashtra							**ORF1b:F2647Y**	1	0.15	**ORF1b:A434V**	2	0.06
Maharashtra							**ORF1b:L2651S**	1	0.15	**ORF1b:C804G**	2	0.06
Maharashtra							**ORF1b:M2657L**	1	0.15	**ORF1b:T253I**	2	0.06
Maharashtra							**ORF1b:Y166L**	1	0.15	**ORF1b:V2152I**	2	0.06
Maharashtra							**ORF1b:A167R**	1	0.15	**ORF1b:R944L**	2	0.06
Maharashtra							**ORF1b:R172S**	1	0.15	**ORF1b:D955C**	2	0.06
Maharashtra							**ORF1b:V332R**	1	0.15	**ORF1b:V957I**	2	0.06
Maharashtra							**ORF1b:V333A**	1	0.15	**ORF1b:I958N**	2	0.06
Maharashtra							**ORF1b:Y337G**	1	0.15	**ORF1b:S959I**	2	0.06
Maharashtra							**ORF1b:A2006T**	1	0.15	**ORF1b:S961***	2	0.06
Maharashtra							**ORF1b:V2007I**	1	0.15	**ORF1b:H962I**	2	0.06
Maharashtra							**ORF1b:R2009T**	1	0.15	**ORF1b:K963S**	2	0.06
Maharashtra							**ORF1b:N2023S**	1	0.15	**ORF1b:L966C**	2	0.06
Maharashtra							**ORF1b:K2036R**	1	0.15	**ORF1b:S967***	2	0.06
Maharashtra							**ORF1b:I2077T**	1	0.15	**ORF1b:V968S**	2	0.06
Maharashtra							**ORF1b:I2078T**	1	0.15	**ORF1b:N969V**	2	0.06
Maharashtra							**ORF1b:N2079K**	1	0.15	**ORF1b:P970C**	2	0.06
Maharashtra							**ORF1b:N2080F**	1	0.15	**ORF1b:Y971L**	2	0.06
Maharashtra							**ORF1b:T2081N**	1	0.15	**ORF1b:V972Q**	2	0.06
Maharashtra							**ORF1b:V2082G**	1	0.15	**ORF1b:N974S**	2	0.06
Maharashtra							**ORF1b:K2085R**	1	0.15	**ORF1b:A975R**	2	0.06
Maharashtra							**ORF1b:V2086I**	1	0.15	**ORF1b:G977***	2	0.06
Maharashtra							**ORF1b:V345G**	1	0.15	**ORF1b:D979H**	2	0.06
Maharashtra							**ORF1b:D2175Y**	1	0.15	**ORF1b:V980R**	2	0.06
Maharashtra							**ORF1b:L796T**	1	0.15	**ORF1b:T981C**	2	0.06
Maharashtra							**ORF1b:D795A**	1	0.15	**ORF1b:V983S**	2	0.06
Maharashtra							**ORF1b:G1217C**	1	0.15	**ORF1b:Q985E**	2	0.06
Maharashtra							**ORF1b:H1807Q**	1	0.15	**ORF1b:Y987L**	2	0.06
Maharashtra							**ORF1b:E1808K**	1	0.15	**ORF1b:P1001***	2	0.06
Maharashtra							**ORF1b:C1809F**	1	0.15	**ORF1b:A545Q**	2	0.06
Maharashtra							**ORF1b:F1810I**	1	0.15	**ORF1b:G550L**	2	0.06
Maharashtra							**ORF1b:V1811Y**	1	0.15	**ORF1b:M557I**	2	0.06
Maharashtra							**ORF1b:R1813L**	1	0.15	**ORF1b:V811N**	2	0.06
Maharashtra							**ORF1b:M2581T**	1	0.15	**ORF1b:K812I**	2	0.06
Maharashtra							**ORF1b:G2582L**	1	0.15	**ORF1b:V818C**	2	0.06
Maharashtra							**ORF1b:F2584C**	1	0.15	**ORF1b:Y819***	2	0.06
Maharashtra							**ORF1b:W2586P**	1	0.15	**ORF1b:P821***	2	0.06
Maharashtra							**ORF1b:W2587C**	1	0.15	**ORF1b:Y822G**	2	0.06
Maharashtra							**ORF1b:T2588E**	1	0.15	**ORF1b:D842A**	2	0.06
Maharashtra							**ORF1b:H247Y**	1	0.15	**ORF1b:G843V**	2	0.06
Maharashtra							**ORF1b:A373V**	1	0.15	**ORF1b:T844L**	2	0.06
Maharashtra							**ORF1b:K179N**	1	0.15	**ORF1b:Q435H**	2	0.06
Maharashtra							**ORF1b:T180I**	1	0.15	**ORF1b:M624V**	2	0.06
Maharashtra							**ORF1b:V181I**	1	0.15	**ORF1b:V628I**	2	0.06
Maharashtra							**ORF1b:G1093V**	1	0.15	**ORF1b:A690L**	2	0.06
Maharashtra							**ORF1b:P1095N**	1	0.15	**ORF1b:V691L**	2	0.06
Maharashtra							**ORF1b:R1096S**	1	0.15	**ORF1b:T692M**	2	0.06
Maharashtra							**ORF1b:P1097T**	1	0.15	**ORF1b:A693L**	2	0.06
Maharashtra							**ORF1b:V2I**	1	0.15	**ORF1b:N694I**	2	0.06
Maharashtra							**ORF1b:V548L**	1	0.15	**ORF1b:Q715I**	2	0.06
Maharashtra							**ORF1b:F1722C**	1	0.15	**ORF1b:H716E**	2	0.06
Maharashtra							**ORF1b:H801Y**	1	0.15	**ORF1b:R717M**	2	0.06
Maharashtra							**ORF1b:D295Y**	1	0.15	**ORF1b:Y719T**	2	0.06
Maharashtra							**ORF1b:T2171I**	1	0.15	**ORF1b:E720Q**	2	0.06
Maharashtra							**ORF1b:P85S**	1	0.15	**ORF1b:C721T**	2	0.06
Maharashtra							**ORF1b:V2182L**	1	0.15	**ORF1b:S745***	2	0.06
Maharashtra							**ORF1b:V2371L**	1	0.15	**ORF1b:G765L**	2	0.06
Maharashtra							**ORF1b:K1724R**	1	0.15	**ORF1b:V767C**	2	0.06
Maharashtra							**ORF1b:M454I**	1	0.15	**ORF1b:A768V**	2	0.06
Maharashtra							**ORF1b:A1190V**	1	0.15	**ORF1b:L810R**	2	0.06

Maharashtra							**ORF1b:T1739I**	1	0.15	**ORF1b:R827M**	2	0.06
Maharashtra							**ORF1b:R2613C**	1	0.15	**ORF1b:G990V**	2	0.06
Maharashtra							**ORF1b:P2169S**	1	0.15	**ORF1b:A1040***	2	0.06
Maharashtra							**ORF1b:D51Y**	1	0.15	**ORF1b:G1041H**	2	0.06
Maharashtra							**ORF1b:H2285Y**	1	0.15	**ORF1b:D1042L**	2	0.06
Maharashtra							**ORF1b:T797D**	1	0.15	**ORF1b:L1053***	2	0.06
Maharashtra							**ORF1b:K798***	1	0.15	**ORF1b:I1823L**	2	0.06
Maharashtra							**ORF1b:G799D**	1	0.15	**ORF1b:G1824T**	2	0.06
Maharashtra							**ORF1b:E802I**	1	0.15	**ORF1b:A1832W**	2	0.06
Maharashtra							**ORF1b:A167T**	1	0.15	**ORF1b:C1833L**	2	0.06
Maharashtra							**ORF1b:I2616T**	1	0.15	**ORF1b:R1834L**	2	0.06
Maharashtra							**ORF1b:D2617I**	1	0.15	**ORF1b:E1871Y**	2	0.06
Maharashtra							**ORF1b:G2618D**	1	0.15	**ORF1b:W1872L**	2	0.06
Maharashtra							**ORF1b:D1024A**	1	0.15	**ORF1b:E1888K**	2	0.06
Maharashtra							**ORF1b:V225L**	1	0.15	**ORF1b:E1889***	2	0.06
Maharashtra							**ORF1b:A545V**	1	0.15	**ORF1b:L1890K**	2	0.06
Maharashtra							**ORF1b:G662V**	1	0.15	**ORF1b:F1891N**	2	0.06
Maharashtra							**ORF1b:E2614D**	1	0.15	**ORF1b:D1914Y**	2	0.06
Maharashtra							**ORF1b:L1248F**	1	0.15	**ORF1b:R1915F**	2	0.06
Maharashtra							**ORF1b:G1010E**	1	0.15	**ORF1b:Y1916G**	2	0.06
Maharashtra							**ORF1b:Q561H**	1	0.15	**ORF1b:P1917I**	2	0.06
Maharashtra							**ORF1b:S1893F**	1	0.15	**ORF1b:N1919M**	2	0.06
Maharashtra							**ORF1b:A302S**	1	0.15	**ORF1b:N1962Q**	2	0.06
Maharashtra							**ORF1b:D1381Y**	1	0.15	**ORF1b:V1983H**	2	0.06
Maharashtra							**ORF1b:L2123I**	1	0.15	**ORF1b:R2189K**	2	0.06
Maharashtra							**ORF1b:M371I**	1	0.15	**ORF1b:N2190***	2	0.06
Maharashtra							**ORF1b:E735D**	1	0.15	**ORF1b:G2191T**	2	0.06
Maharashtra							**ORF1b:T1739A**	1	0.15	**ORF1b:V2192Y**	2	0.06
Maharashtra							**ORF1b:S2212I**	1	0.15	**ORF1b:I2194E**	2	0.06
Maharashtra							**ORF1b:A1291S**	1	0.15	**ORF1b:T2195M**	2	0.06
Maharashtra							**ORF1b:N2133S**	1	0.15	**ORF1b:E2196P**	2	0.06
Maharashtra							**ORF1b:V344S**	1	0.15	**ORF1b:G2197V**	2	0.06
Maharashtra							**ORF1b:T17I**	1	0.15	**ORF1b:S2198M**	2	0.06
Maharashtra							**ORF1b:A2132V**	1	0.15	**ORF1b:K2200F**	2	0.06
Maharashtra							**ORF1b:L2382F**	1	0.15	**ORF1b:G2201L**	2	0.06
Maharashtra							**ORF1b:D349V**	1	0.15	**ORF1b:G2215K**	2	0.06
Maharashtra							**ORF1b:A414V**	1	0.15	**ORF1b:A2222L**	2	0.06
Maharashtra							**ORF1b:V1905I**	1	0.15	**ORF1b:Y2276N**	2	0.06
Maharashtra							**ORF1b:T730I**	1	0.15	**ORF1b:L2302V**	2	0.06
Maharashtra							**ORF1b:M1693I**	1	0.15	**ORF1b:G2304H**	2	0.06
Maharashtra							**ORF1b:P1967L**	1	0.15	**ORF1b:E2317N**	2	0.06
Maharashtra							**ORF1b:N1962S**	1	0.15	**ORF1b:D2318***	2	0.06
Maharashtra							**ORF1b:Q2635H**	1	0.15	**ORF1b:F2319K**	2	0.06
Maharashtra							**ORF1b:G191C**	1	0.15	**ORF1b:P2321L**	2	0.06
Maharashtra							**ORF1b:V896I**	1	0.15	**ORF1b:M2322F**	2	0.06
Maharashtra							**ORF1b:P1567L**	1	0.15	**ORF1b:D2323L**	2	0.06
Maharashtra							**ORF1b:V463L**	1	0.15	**ORF1b:S2324W**	2	0.06
Maharashtra							**ORF1b:A1476S**	1	0.15	**ORF1b:T2336V**	2	0.06
Maharashtra							**ORF1b:D1572N**	1	0.15	**ORF1b:G2337H**	2	0.06
Maharashtra							**ORF1b:S1881I**	1	0.15	**ORF1b:K2340V**	2	0.06
Maharashtra							**ORF1b:T1067I**	1	0.15	**ORF1b:D2351I**	2	0.06
Maharashtra							**ORF1b:A1918V**	1	0.15	**ORF1b:D2352Y**	2	0.06
Maharashtra							**ORF1b:F2309S**	1	0.15	**ORF1b:F2353Y**	2	0.06
Maharashtra							**ORF1b:N543L**	1	0.15	**ORF1b:V2354L**	2	0.06
Maharashtra							**ORF1b:R544V**	1	0.15	**ORF1b:E2355M**	2	0.06
Maharashtra							**ORF1b:A545S**	1	0.15	**ORF1b:A2504T**	2	0.06
Maharashtra							**ORF1b:R546L**	1	0.15	**ORF1b:D2505Y**	2	0.06
Maharashtra							**ORF1b:T547S**	1	0.15	**ORF1b:T2507***	2	0.06
Maharashtra							**ORF1b:A549V**	1	0.15	**ORF1b:L2508***	2	0.06
Maharashtra							**ORF1b:G550L**	1	0.15	**ORF1b:I2509M**	2	0.06
Maharashtra							**ORF1b:V551***	1	0.15	**ORF1b:E2311K**	2	0.06
Maharashtra							**ORF1b:S552P**	1	0.15	**ORF1b:S1082M**	2	0.06
Maharashtra							**ORF1b:C554D**	1	0.15	**ORF1b:D1083V**	2	0.06
Maharashtra							**ORF1b:T556F**	1	0.15	**ORF1b:R1084L**	2	0.06
Maharashtra							**ORF1b:M557I**	1	0.15	**ORF1b:E1085L**	2	0.06
Maharashtra							**ORF1b:T558K**	1	0.15	**ORF1b:R1135K**	2	0.06
Maharashtra							**ORF1b:T577M**	1	0.15	**ORF1b:G1136V**	2	0.06
Maharashtra							**ORF1b:I580G**	1	0.15	**ORF1b:S1376V**	2	0.06
Maharashtra							**ORF1b:G581T**	1	0.15	**ORF1b:A1377L**	2	0.06
Maharashtra							**ORF1b:L593***	1	0.15	**ORF1b:P1652V**	2	0.06
Maharashtra							**ORF1b:D614***	1	0.15	**ORF1b:N1653L**	2	0.06
Maharashtra							**ORF1b:R615H**	1	0.15	**ORF1b:H1712Y**	2	0.06
Maharashtra							**ORF1b:M617***	1	0.15	**ORF1b:F1714H**	2	0.06

Maharashtra							**ORF1b:P618N**	1	0.15	**ORF1b:E1715M**	2	0.06
Maharashtra							**ORF1b:N619Y**	1	0.15	**ORF1b:L1716A**	2	0.06
Maharashtra							**ORF1b:M620G**	1	0.15	**ORF1b:T1717L**	2	0.06
Maharashtra							**ORF1b:T635C**	1	0.15	**ORF1b:M1719***	2	0.06
Maharashtra							**ORF1b:C636S**	1	0.15	**ORF1b:C1734***	2	0.06
Maharashtra							**ORF1b:V653***	1	0.15	**ORF1b:R1736L**	2	0.06
Maharashtra							**ORF1b:L654I**	1	0.15	**ORF1b:R1737S**	2	0.06
Maharashtra							**ORF1b:E656***	1	0.15	**ORF1b:F1757L**	2	0.06
Maharashtra							**ORF1b:M657***	1	0.15	**ORF1b:D1758M**	2	0.06
Maharashtra							**ORF1b:T677***	1	0.15	**ORF1b:Y1759P**	2	0.06
Maharashtra							**ORF1b:L699S**	1	0.15	**ORF1b:Y1761N**	2	0.06
Maharashtra							**ORF1b:T701C**	1	0.15	**ORF1b:A1794V**	2	0.06
Maharashtra							**ORF1b:D702H**	1	0.15	**ORF1b:V1133L**	2	0.06
Maharashtra							**ORF1b:H716C**	1	0.15	**ORF1b:Y1134P**	2	0.06
Maharashtra							**ORF1b:Y719***	1	0.15	**ORF1b:A1831***	2	0.06
Maharashtra							**ORF1b:E720V**	1	0.15	**ORF1b:A1832S**	2	0.06
Maharashtra							**ORF1b:C721S**	1	0.15	**ORF1b:N1932C**	2	0.06
Maharashtra							**ORF1b:Y723***	1	0.15	**ORF1b:T2084***	2	0.06
Maharashtra							**ORF1b:R724K**	1	0.15	**ORF1b:K2161H**	2	0.06
Maharashtra							**ORF1b:N725***	1	0.15	**ORF1b:P2162S**	2	0.06
Maharashtra							**ORF1b:D727***	1	0.15	**ORF1b:T2163Q**	2	0.06
Maharashtra							**ORF1b:D729L**	1	0.15	**ORF1b:I2166N**	2	0.06
Maharashtra							**ORF1b:T730R**	1	0.15	**ORF1b:C2167***	2	0.06
Maharashtra							**ORF1b:D731I**	1	0.15	**ORF1b:A2168N**	2	0.06
Maharashtra							**ORF1b:V733A**	1	0.15	**ORF1b:P2169D**	2	0.06
Maharashtra							**ORF1b:N734***	1	0.15	**ORF1b:L2193T**	2	0.06
Maharashtra							**ORF1b:E735T**	1	0.15	**ORF1b:Q1669H**	2	0.06
Maharashtra							**ORF1b:S750N**	1	0.15	**ORF1b:L1248F**	2	0.06
Maharashtra							**ORF1b:A753H**	1	0.15	**ORF1b:S1942G**	2	0.06
Maharashtra							**ORF1b:V754L**	1	0.15	**ORF1b:T903A**	2	0.06
Maharashtra							**ORF1b:V755C**	1	0.15	**ORF1b:H2583R**	2	0.06
Maharashtra							**ORF1b:C756I**	1	0.15	**ORF1b:S1893F**	2	0.06
Maharashtra							**ORF1b:F757S**	1	0.15	**ORF1b:T701I**	2	0.06
Maharashtra							**ORF1b:N758R**	1	0.15	**ORF1b:N2630I**	2	0.06
Maharashtra							**ORF1b:T760S**	1	0.15	**ORF1b:I2634N**	2	0.06
Maharashtra							**ORF1b:Y761G**	1	0.15	**ORF1b:L820F**	2	0.06
Maharashtra							**ORF1b:A762***	1	0.15	**ORF1b:D2423N**	2	0.06
Maharashtra							**ORF1b:S763H**	1	0.15	**ORF1b:T1655I**	2	0.06
Maharashtra							**ORF1b:Q764K**	1	0.15	**ORF1b:S1942I**	2	0.06
Maharashtra							**ORF1b:G765E**	1	0.15	**ORF1b:D58A**	2	0.06
Maharashtra							**ORF1b:V767***	1	0.15	**ORF1b:A1131V**	2	0.06
Maharashtra							**ORF1b:A768V**	1	0.15	**ORF1b:M659I**	2	0.06
Maharashtra							**ORF1b:V776L**	1	0.15	**ORF1b:P2633S**	2	0.06
Maharashtra							**ORF1b:Y778S**	1	0.15	**ORF1b:W2628G**	2	0.06
Maharashtra							**ORF1b:Y779K**	1	0.15	**ORF1b:T393I**	2	0.06
Maharashtra							**ORF1b:W791R**	1	0.15	**ORF1b:E269Q**	2	0.06
Maharashtra							**ORF1b:E793S**	1	0.15	**ORF1b:V1132F**	2	0.06
Maharashtra							**ORF1b:T794***	1	0.15	**ORF1b:D1130E**	2	0.06
Maharashtra							**ORF1b:D795I**	1	0.15	**ORF1b:H247Y**	2	0.06
Maharashtra							**ORF1b:I847***	1	0.15	**ORF1b:D2423Y**	2	0.06
Maharashtra							**ORF1b:E848Y**	1	0.15	**ORF1b:N1587D**	2	0.06
Maharashtra							**ORF1b:F850Y**	1	0.15	**ORF1b:T1137I**	2	0.06
Maharashtra							**ORF1b:V851R**	1	0.15	**ORF1b:I2525V**	2	0.06
Maharashtra							**ORF1b:S852C**	1	0.15	**ORF1b:E2614G**	2	0.06
Maharashtra							**ORF1b:H883S**	1	0.15	**ORF1b:D1899Y**	2	0.06
Maharashtra							**ORF1b:D884F**	1	0.15	**ORF1b:E427G**	2	0.06
Maharashtra							**ORF1b:E885V**	1	0.15	**ORF1b:S2312L**	2	0.06
Maharashtra							**ORF1b:G888I**	1	0.15	**ORF1b:P2477A**	2	0.06
Maharashtra							**ORF1b:M890K**	1	0.15	**ORF1b:S334***	2	0.06
Maharashtra							**ORF1b:L891K**	1	0.15	**ORF1b:A2268S**	2	0.06
Maharashtra							**ORF1b:D892A**	1	0.15	**ORF1b:F1960Q**	2	0.06
Maharashtra							**ORF1b:M893T**	1	0.15	**ORF1b:L1963I**	2	0.06
Maharashtra							**ORF1b:Y894***	1	0.15	**ORF1b:Q1965V**	2	0.06
Maharashtra							**ORF1b:S895***	1	0.15	**ORF1b:P2162L**	2	0.06
Maharashtra							**ORF1b:M897V**	1	0.15	**ORF1b:I2373S**	2	0.06
Maharashtra							**ORF1b:Y916G**	1	0.15	**ORF1b:M2381***	2	0.06
Maharashtra							**ORF1b:P918V**	1	0.15	**ORF1b:W2383L**	2	0.06
Maharashtra							**ORF1b:V921A**	1	0.15	**ORF1b:C2384P**	2	0.06
Maharashtra							**ORF1b:L922Y**	1	0.15	**ORF1b:P2394I**	2	0.06
Maharashtra							**ORF1b:Q923S**	1	0.15	**ORF1b:K2395T**	2	0.06
Maharashtra							**ORF1b:A924L**	1	0.15	**ORF1b:L2213S**	2	0.06
Maharashtra							**ORF1b:V925T**	1	0.15	**ORF1b:N696D**	2	0.06
Maharashtra							**ORF1b:A927C**	1	0.15	**ORF1b:P2633A**	2	0.06

Maharashtra							**ORF1b:C928W**	1	0.15	**ORF1b:D1251V**	2	0.06
Maharashtra							**ORF1b:V929G**	1	0.15	**ORF1b:T243I**	2	0.06
Maharashtra							**ORF1b:R938Q**	1	0.15	**ORF1b:G2068R**	2	0.06
Maharashtra							**ORF1b:C939F**	1	0.15	**ORF1b:P1287S**	2	0.06
Maharashtra							**ORF1b:G940T**	1	0.15	**ORF1b:L2669S**	2	0.06
Maharashtra							**ORF1b:A941D**	1	0.15	**ORF1b:H1979S**	2	0.06
Maharashtra							**ORF1b:C942F**	1	0.15	**ORF1b:N2632S**	2	0.06
Maharashtra							**ORF1b:R944L**	1	0.15	**ORF1b:E720K**	2	0.06
Maharashtra							**ORF1b:D955C**	1	0.15	**ORF1b:R296K**	2	0.06
Maharashtra							**ORF1b:V957I**	1	0.15	**ORF1b:T1064N**	2	0.06
Maharashtra							**ORF1b:I958N**	1	0.15	**ORF1b:Y1784H**	2	0.06
Maharashtra							**ORF1b:S959I**	1	0.15	**ORF1b:I1690T**	2	0.06
Maharashtra							**ORF1b:S961***	1	0.15	**ORF1b:V1691S**	2	0.06
Maharashtra							**ORF1b:H962I**	1	0.15	**ORF1b:Q1692***	2	0.06
Maharashtra							**ORF1b:K963S**	1	0.15	**ORF1b:L1694S**	2	0.06
Maharashtra							**ORF1b:L966C**	1	0.15	**ORF1b:D1746K**	2	0.06
Maharashtra							**ORF1b:S967***	1	0.15	**ORF1b:T1747P**	2	0.06
Maharashtra							**ORF1b:V968S**	1	0.15	**ORF1b:Y1748C**	2	0.06
Maharashtra							**ORF1b:N969V**	1	0.15	**ORF1b:C1750H**	2	0.06
Maharashtra							**ORF1b:P970C**	1	0.15	**ORF1b:Q1878H**	2	0.06
Maharashtra							**ORF1b:Y971L**	1	0.15	**ORF1b:N2124H**	2	0.06
Maharashtra							**ORF1b:V972Q**	1	0.15	**ORF1b:D2142N**	2	0.06
Maharashtra							**ORF1b:N974S**	1	0.15	**ORF1b:S2294G**	2	0.06
Maharashtra							**ORF1b:A975R**	1	0.15	**ORF1b:A1057S**	2	0.06
Maharashtra							**ORF1b:P976L**	1	0.15	**ORF1b:V2685F**	2	0.06
Maharashtra							**ORF1b:G977***	1	0.15	**ORF1b:V396F**	2	0.06
Maharashtra							**ORF1b:D979H**	1	0.15	**ORF1b:D436G**	2	0.06
Maharashtra							**ORF1b:V980R**	1	0.15	**ORF1b:M1197V**	2	0.06
Maharashtra							**ORF1b:T981C**	1	0.15	**ORF1b:S2495L**	2	0.06
Maharashtra							**ORF1b:V983S**	1	0.15	**ORF1b:A1491V**	2	0.06
Maharashtra							**ORF1b:Q985E**	1	0.15	**ORF1b:V2371L**	2	0.06
Maharashtra							**ORF1b:Y987L**	1	0.15	**ORF1b:Y1593H**	2	0.06
Maharashtra							**ORF1b:D1024N**	1	0.15	**ORF1b:K1125R**	2	0.06
Maharashtra							**ORF1b:N1025M**	1	0.15	**ORF1b:I1227M**	2	0.06
Maharashtra							**ORF1b:V1026***	1	0.15	**ORF1b:V1649F**	2	0.06
Maharashtra							**ORF1b:T1027L**	1	0.15	**ORF1b:E1242V**	2	0.06
Maharashtra							**ORF1b:T1048K**	1	0.15	**ORF1b:I2077V**	2	0.06
Maharashtra							**ORF1b:K1062Q**	1	0.15	**ORF1b:D161Y**	2	0.06
Maharashtra							**ORF1b:T1064F**	1	0.15	**ORF1b:T2537I**	2	0.06
Maharashtra							**ORF1b:E1065C**	1	0.15	**ORF1b:V839L**	2	0.06
Maharashtra							**ORF1b:E1066S**	1	0.15	**ORF1b:Q1441H**	2	0.06
Maharashtra							**ORF1b:T1067R**	1	0.15	**ORF1b:G1940S**	2	0.06
Maharashtra							**ORF1b:F1068N**	1	0.15	**ORF1b:L1943***	2	0.06
Maharashtra							**ORF1b:K1069A**	1	0.15	**ORF1b:G2561R**	2	0.06
Maharashtra							**ORF1b:L1070Q**	1	0.15	**ORF1b:D275Y**	2	0.06
Maharashtra							**ORF1b:G1073D**	1	0.15	**ORF1b:L2213F**	2	0.06
Maharashtra							**ORF1b:A1075***	1	0.15	**ORF1b:L1633I**	2	0.06
Maharashtra							**ORF1b:P1097L**	1	0.15	**ORF1b:D1183Y**	2	0.06
Maharashtra							**ORF1b:P1098C**	1	0.15	**ORF1b:P12S**	2	0.06
Maharashtra							**ORF1b:R1109W**	1	0.15	**ORF1b:T2432I**	2	0.06
Maharashtra							**ORF1b:V1110L**	1	0.15	**ORF1b:S1003G**	2	0.06
Maharashtra							**ORF1b:T1111S**	1	0.15	**ORF1b:K1812R**	2	0.06
Maharashtra							**ORF1b:K1112C**	1	0.15	**ORF1b:R1226H**	2	0.06
Maharashtra							**ORF1b:S1114***	1	0.15	**ORF1b:Y23H**	2	0.06
Maharashtra							**ORF1b:V1144L**	1	0.15	**ORF1b:V2233F**	2	0.06
Maharashtra							**ORF1b:G1145P**	1	0.15	**ORF1b:M620I**	2	0.06
Maharashtra							**ORF1b:D1146R**	1	0.15	**ORF1b:T1067I**	2	0.06
Maharashtra							**ORF1b:F1148N**	1	0.15	**ORF1b:K705R**	2	0.06
Maharashtra							**ORF1b:V1149N**	1	0.15	**ORF1b:V1642F**	2	0.06
Maharashtra							**ORF1b:V1170T**	1	0.15	**ORF1b:I1569T**	2	0.06
Maharashtra							**ORF1b:R1171S**	1	0.15	**ORF1b:M809V**	2	0.06
Maharashtra							**ORF1b:I1172A**	1	0.15	**ORF1b:A2513S**	2	0.06
Maharashtra							**ORF1b:G1174W**	1	0.15	**ORF1b:Q1509H**	2	0.06
Maharashtra							**ORF1b:Y1176I**	1	0.15	**ORF1b:A2222S**	2	0.06
Maharashtra							**ORF1b:V1189K**	1	0.15	**ORF1b:V1840F**	2	0.06
Maharashtra							**ORF1b:A1190G**	1	0.15	**ORF1b:G1940L**	2	0.06
Maharashtra							**ORF1b:N1191W**	1	0.15	**ORF1b:G1937V**	2	0.06
Maharashtra							**ORF1b:Q1193A**	1	0.15	**ORF1b:G2561V**	2	0.06
Maharashtra							**ORF1b:Q1204F**	1	0.15	**ORF1b:E2317R**	2	0.06
Maharashtra							**ORF1b:G1205Y**	1	0.15	**ORF1b:V1021F**	2	0.06
Maharashtra							**ORF1b:P1206T**	1	0.15	**ORF1b:M187I**	2	0.06
Maharashtra							**ORF1b:G1210S**	1	0.15	**ORF1b:S1273L**	2	0.06
Maharashtra							**ORF1b:K1211L**	1	0.15	**ORF1b:G1107D**	2	0.06

Maharashtra							**ORF1b:S1212L**	1	0.15	**ORF1b:Y1108H**	2	0.06
Maharashtra							**ORF1b:H1213P**	1	0.15	**ORF1b:R1109H**	2	0.06
Maharashtra							**ORF1b:A1235S**	1	0.15	**ORF1b:F1908E**	2	0.06
Maharashtra							**ORF1b:A1236V**	1	0.15	**ORF1b:W1909L**	2	0.06
Maharashtra							**ORF1b:V1237Y**	1	0.15	**ORF1b:N1919R**	2	0.06
Maharashtra							**ORF1b:D1238S**	1	0.15	**ORF1b:S1920***	2	0.06
Maharashtra							**ORF1b:A1239L**	1	0.15	**ORF1b:V1922S**	2	0.06
Maharashtra							**ORF1b:C1241G**	1	0.15	**ORF1b:R1924***	2	0.06
Maharashtra							**ORF1b:E1242I**	1	0.15	**ORF1b:D1926I**	2	0.06
Maharashtra							**ORF1b:P1258F**	1	0.15	**ORF1b:T1927***	2	0.06
Maharashtra							**ORF1b:R1260S**	1	0.15	**ORF1b:R1928P**	2	0.06
Maharashtra							**ORF1b:A1261C**	1	0.15	**ORF1b:V1929***	2	0.06
Maharashtra							**ORF1b:V1283F**	1	0.15	**ORF1b:S1931A**	2	0.06
Maharashtra							**ORF1b:A1285C**	1	0.15	**ORF1b:N1932W**	2	0.06
Maharashtra							**ORF1b:L1307***	1	0.15	**ORF1b:K2160N**	2	0.06
Maharashtra							**ORF1b:S1308***	1	0.15	**ORF1b:P1000H**	2	0.06
Maharashtra							**ORF1b:V1309N**	1	0.15	**ORF1b:V1144L**	2	0.06
Maharashtra							**ORF1b:V1310F**	1	0.15	**ORF1b:G1145P**	2	0.06
Maharashtra							**ORF1b:P1329I**	1	0.15	**ORF1b:D1146R**	2	0.06
Maharashtra							**ORF1b:S1408***	1	0.15	**ORF1b:V1189K**	2	0.06
Maharashtra							**ORF1b:S1409Q**	1	0.15	**ORF1b:A1190G**	2	0.06
Maharashtra							**ORF1b:I1411T**	1	0.15	**ORF1b:N1191W**	2	0.06
Maharashtra							**ORF1b:Q1415Y**	1	0.15	**ORF1b:Q1193A**	2	0.06
Maharashtra							**ORF1b:I1416T**	1	0.15	**ORF1b:A1235S**	2	0.06
Maharashtra							**ORF1b:G1417***	1	0.15	**ORF1b:A1236V**	2	0.06
Maharashtra							**ORF1b:V1418P**	1	0.15	**ORF1b:V1237Y**	2	0.06
Maharashtra							**ORF1b:V1419C**	1	0.15	**ORF1b:D1238S**	2	0.06
Maharashtra							**ORF1b:R1420L**	1	0.15	**ORF1b:A1239L**	2	0.06
Maharashtra							**ORF1b:N1439K**	1	0.15	**ORF1b:C1241G**	2	0.06
Maharashtra							**ORF1b:Y1944F**	1	0.15	**ORF1b:E1242I**	2	0.06
Maharashtra							**ORF1b:N1946C**	1	0.15	**ORF1b:P1258F**	2	0.06
Maharashtra							**ORF1b:P1975M**	1	0.15	**ORF1b:R1260S**	2	0.06
Maharashtra							**ORF1b:C1976***	1	0.15	**ORF1b:A1261C**	2	0.06
Maharashtra							**ORF1b:E1977V**	1	0.15	**ORF1b:S1273F**	2	0.06
Maharashtra							**ORF1b:G1980W**	1	0.15	**ORF1b:T1274***	2	0.06
Maharashtra							**ORF1b:Q1982T**	1	0.15	**ORF1b:L1275***	2	0.06
Maharashtra							**ORF1b:V1983I**	1	0.15	**ORF1b:E1276I**	2	0.06
Maharashtra							**ORF1b:W2383C**	1	0.15	**ORF1b:K1317C**	2	0.06
Maharashtra							**ORF1b:V1705I**	1	0.15	**ORF1b:H1318C**	2	0.06
Maharashtra							**ORF1b:M809I**	1	0.15	**ORF1b:Y1319Q**	2	0.06
Maharashtra							**ORF1b:I1032N**	1	0.15	**ORF1b:V1320C**	2	0.06
Maharashtra							**ORF1b:S2052G**	1	0.15	**ORF1b:Y1321Q**	2	0.06
Maharashtra							**ORF1b:M2581L**	1	0.15	**ORF1b:G1323T**	2	0.06
Maharashtra							**ORF1b:G2582***	1	0.15	**ORF1b:D1324C**	2	0.06
Maharashtra							**ORF1b:Q813H**	1	0.15	**ORF1b:P1325***	2	0.06
Maharashtra							**ORF1b:L1351F**	1	0.15	**ORF1b:Q1327L**	2	0.06
Maharashtra							**ORF1b:L1701F**	1	0.15	**ORF1b:L1328C**	2	0.06
Maharashtra							**ORF1b:P2633L**	1	0.15	**ORF1b:P1329V**	2	0.06
Maharashtra							**ORF1b:D1028N**	1	0.15	**ORF1b:A1330H**	2	0.06
Maharashtra							**ORF1b:A2575S**	1	0.15	**ORF1b:P1331W**	2	0.06
Maharashtra							**ORF1b:H1087Y**	1	0.15	**ORF1b:T1333P**	2	0.06
Maharashtra							**ORF1b:T1522I**	1	0.15	**ORF1b:L1335S**	2	0.06
Maharashtra							**ORF1b:A434V**	1	0.15	**ORF1b:K1337T**	2	0.06
Maharashtra							**ORF1b:I2262M**	1	0.15	**ORF1b:L1340***	2	0.06
Maharashtra							**ORF1b:V1538L**	1	0.15	**ORF1b:E1341T**	2	0.06
Maharashtra							**ORF1b:D260N**	1	0.15	**ORF1b:P1342Y**	2	0.06
Maharashtra							**ORF1b:Y1200C**	1	0.15	**ORF1b:Y1344N**	2	0.06
Maharashtra							**ORF1b:V1945L**	1	0.15	**ORF1b:F1345Y**	2	0.06
Maharashtra							**ORF1b:G2151D**	1	0.15	**ORF1b:N1346R**	2	0.06
Maharashtra							**ORF1b:D349N**	1	0.15	**ORF1b:D1554Y**	2	0.06
Maharashtra							**ORF1b:A176S**	1	0.15	**ORF1b:K1556G**	2	0.06
Maharashtra							**ORF1b:V767L**	1	0.15	**ORF1b:F1557T**	2	0.06
Maharashtra							**ORF1b:K1141R**	1	0.15	**ORF1b:K1558Y**	2	0.06
Maharashtra							**ORF1b:A1443V**	1	0.15	**ORF1b:E1560P**	2	0.06
Maharashtra							**ORF1b:I770M**	1	0.15	**ORF1b:G1561Q**	2	0.06
Maharashtra							**ORF1b:K2063N**	1	0.15	**ORF1b:L1562C**	2	0.06
Maharashtra							**ORF1b:S334E**	1	0.15	**ORF1b:C1563***	2	0.06
Maharashtra							**ORF1b:G336T**	1	0.15	**ORF1b:V1564H**	2	0.06
Maharashtra							**ORF1b:M2417I**	1	0.15	**ORF1b:D1565***	2	0.06
Maharashtra							**ORF1b:A1791V**	1	0.15	**ORF1b:P1567K**	2	0.06
Maharashtra							**ORF1b:T76I**	1	0.15	**ORF1b:G1568T**	2	0.06
Maharashtra							**ORF1b:V426I**	1	0.15	**ORF1b:S1942D**	2	0.06
Maharashtra							**ORF1b:E427S**	1	0.15	**ORF1b:A2028Y**	2	0.06

Maharashtra							ORF1b:L428T	1	0.15	ORF1b:T1354I	2	0.06
Maharashtra							ORF1b:M1586I	1	0.15	ORF1b:E1184D	2	0.06
Maharashtra							ORF1b:T2332K	1	0.15	ORF1b:T899I	2	0.06
Maharashtra							ORF1b:E2377K	1	0.15	ORF1b:H2293R	2	0.06
Maharashtra										ORF1b:G2610N	2	0.06
Maharashtra										ORF1b:A576S	2	0.06
Maharashtra										ORF1b:Y710D	2	0.06
Maharashtra										ORF1b:A2513V	2	0.06
Maharashtra										ORF1b:Y2281S	2	0.06
Maharashtra										ORF1b:D1306E	2	0.06
Maharashtra										ORF1b:H66Y	2	0.06
Maharashtra										ORF1b:L261F	2	0.06
Maharashtra										ORF1b:P1249F	2	0.06
Maharashtra										ORF1b:W153C	2	0.06
Maharashtra										ORF1b:A1410S	2	0.06
Maharashtra										ORF1b:D131Y	2	0.06
Maharashtra										ORF1b:R2652M	2	0.06
Maharashtra										ORF1b:F1891L	2	0.06
Maharashtra										ORF1b:A1432V	2	0.06
Maharashtra										ORF1b:V2368F	2	0.06
Maharashtra										ORF1b:G2197S	2	0.06
Maharashtra										ORF1b:D1028G	2	0.06
Maharashtra										ORF1b:P1727L	2	0.06
Maharashtra										ORF1b:S1159I	2	0.06
Maharashtra										ORF1b:T2040N	2	0.06
Maharashtra										ORF1b:S2258G	2	0.06
Maharashtra										ORF1b:T1178I	2	0.06
Maharashtra										ORF1b:A1547S	2	0.06
Maharashtra										ORF1b:N2410S	2	0.06
Maharashtra										ORF1b:T797I	2	0.06
Maharashtra										ORF1b:I1293V	2	0.06
Maharashtra										ORF1b:G2191C	2	0.06
Maharashtra										ORF1b:F308L	2	0.06
Maharashtra										ORF1b:A1954G	2	0.06
Maharashtra										ORF1b:F1955C	2	0.06
Maharashtra										ORF1b:C2001G	2	0.06
Maharashtra										ORF1b:N2002S	2	0.06
Maharashtra										ORF1b:F2030C	2	0.06
Maharashtra										ORF1b:T1289A	1	0.03
Maharashtra										ORF1b:T1067S	1	0.03
Maharashtra										ORF1b:F1068S	1	0.03
Maharashtra										ORF1b:S1071L	1	0.03
Maharashtra										ORF1b:Y1072R	1	0.03
Maharashtra										ORF1b:G1073R	1	0.03
Maharashtra										ORF1b:I1074H	1	0.03
Maharashtra										ORF1b:A1075L	1	0.03
Maharashtra										ORF1b:T1076N	1	0.03
Maharashtra										ORF1b:V1077C	1	0.03
Maharashtra										ORF1b:E1079M	1	0.03
Maharashtra										ORF1b:S2198I	1	0.03
Maharashtra										ORF1b:T1559I	1	0.03
Maharashtra										ORF1b:V1945I	1	0.03
Maharashtra										ORF1b:E2014K	1	0.03
Maharashtra										ORF1b:A2407S	1	0.03
Maharashtra										ORF1b:M2667I	1	0.03
Maharashtra										ORF1b:P1427L	1	0.03
Maharashtra										ORF1b:I2147M	1	0.03
Maharashtra										ORF1b:S316N	1	0.03
Maharashtra										ORF1b:F317G	1	0.03
Maharashtra										ORF1b:G318T	1	0.03
Maharashtra										ORF1b:L320S	1	0.03
Maharashtra										ORF1b:A2559V	1	0.03
Maharashtra										ORF1b:E1242D	1	0.03
Maharashtra										ORF1b:T111A	1	0.03
Maharashtra										ORF1b:G2151S	1	0.03
Maharashtra										ORF1b:T2084I	1	0.03
Maharashtra										ORF1b:L1676F	1	0.03
Maharashtra										ORF1b:P1664S	1	0.03
Maharashtra										ORF1b:K1383E	1	0.03
Maharashtra										ORF1b:P1165S	1	0.03
Maharashtra										ORF1b:V87A	1	0.03
Maharashtra										ORF1b:F325Y	1	0.03
Maharashtra										ORF1b:I1118V	1	0.03

Maharashtra										**ORF1b:Y1989H**	1	0.03
Maharashtra										**ORF1b:G14F**	1	0.03
Maharashtra										**ORF1b:G16A**	1	0.03
Maharashtra										**ORF1b:T19L**	1	0.03
Maharashtra										**ORF1b:D20***	1	0.03
Maharashtra										**ORF1b:V21S**	1	0.03
Maharashtra										**ORF1b:G799T**	1	0.03
Maharashtra										**ORF1b:F803L**	1	0.03
Maharashtra										**ORF1b:K798N**	1	0.03
Maharashtra										**ORF1b:G799E**	1	0.03
Maharashtra										**ORF1b:M606I**	1	0.03
Maharashtra										**ORF1b:D53Y**	1	0.03
Maharashtra										**ORF1b:R1577K**	1	0.03
Maharashtra										**ORF1b:I162V**	1	0.03
Maharashtra										**ORF1b:K254N**	1	0.03
Maharashtra										**ORF1b:Q806H**	1	0.03
Maharashtra										**ORF1b:A869V**	1	0.03
Maharashtra										**ORF1b:L810C**	1	0.03
Maharashtra										**ORF1b:I847***	1	0.03
Maharashtra										**ORF1b:E848Y**	1	0.03
Maharashtra										**ORF1b:F850Y**	1	0.03
Maharashtra										**ORF1b:V851R**	1	0.03
Maharashtra										**ORF1b:S852C**	1	0.03
Maharashtra										**ORF1b:H883S**	1	0.03
Maharashtra										**ORF1b:D884F**	1	0.03
Maharashtra										**ORF1b:E885V**	1	0.03
Maharashtra										**ORF1b:G888I**	1	0.03
Maharashtra										**ORF1b:M890K**	1	0.03
Maharashtra										**ORF1b:L891K**	1	0.03
Maharashtra										**ORF1b:D892A**	1	0.03
Maharashtra										**ORF1b:M893T**	1	0.03
Maharashtra										**ORF1b:Y894***	1	0.03
Maharashtra										**ORF1b:S895***	1	0.03
Maharashtra										**ORF1b:M897V**	1	0.03
Maharashtra										**ORF1b:Y916G**	1	0.03
Maharashtra										**ORF1b:P918V**	1	0.03
Maharashtra										**ORF1b:V921A**	1	0.03
Maharashtra										**ORF1b:L922Y**	1	0.03
Maharashtra										**ORF1b:Q923S**	1	0.03
Maharashtra										**ORF1b:A924L**	1	0.03
Maharashtra										**ORF1b:V925T**	1	0.03
Maharashtra										**ORF1b:A927C**	1	0.03
Maharashtra										**ORF1b:C928W**	1	0.03
Maharashtra										**ORF1b:V929G**	1	0.03
Maharashtra										**ORF1b:R938Q**	1	0.03
Maharashtra										**ORF1b:C939F**	1	0.03
Maharashtra										**ORF1b:G940T**	1	0.03
Maharashtra										**ORF1b:A941D**	1	0.03
Maharashtra										**ORF1b:C942F**	1	0.03
Maharashtra										**ORF1b:D1024N**	1	0.03
Maharashtra										**ORF1b:N1025M**	1	0.03
Maharashtra										**ORF1b:V1026***	1	0.03
Maharashtra										**ORF1b:T1027L**	1	0.03
Maharashtra										**ORF1b:A1040S**	1	0.03
Maharashtra										**ORF1b:F413L**	1	0.03
Maharashtra										**ORF1b:A414R**	1	0.03
Maharashtra										**ORF1b:F419L**	1	0.03
Maharashtra										**ORF1b:F420R**	1	0.03
Maharashtra										**ORF1b:E427L**	1	0.03
Maharashtra										**ORF1b:A439S**	1	0.03
Maharashtra										**ORF1b:L451M**	1	0.03
Maharashtra										**ORF1b:P452T**	1	0.03
Maharashtra										**ORF1b:M454I**	1	0.03
Maharashtra										**ORF1b:C455V**	1	0.03
Maharashtra										**ORF1b:D456I**	1	0.03
Maharashtra										**ORF1b:R458Y**	1	0.03
Maharashtra										**ORF1b:F462I**	1	0.03
Maharashtra										**ORF1b:V463S**	1	0.03
Maharashtra										**ORF1b:V464T**	1	0.03
Maharashtra										**ORF1b:E465L**	1	0.03
Maharashtra										**ORF1b:V466I**	1	0.03
Maharashtra										**ORF1b:N482T**	1	0.03
Maharashtra										**ORF1b:Q483M**	1	0.03

Maharashtra										**ORF1b:I485A**	1	0.03
Maharashtra										**ORF1b:N487L**	1	0.03
Maharashtra										**ORF1b:N488M**	1	0.03
Maharashtra										**ORF1b:D490T**	1	0.03
Maharashtra										**ORF1b:A493N**	1	0.03
Maharashtra										**ORF1b:A503V**	1	0.03
Maharashtra										**ORF1b:Y537***	1	0.03
Maharashtra										**ORF1b:A538I**	1	0.03
Maharashtra										**ORF1b:I539L**	1	0.03
Maharashtra										**ORF1b:A541M**	1	0.03
Maharashtra										**ORF1b:K542P**	1	0.03
Maharashtra										**ORF1b:N543L**	1	0.03
Maharashtra										**ORF1b:R544V**	1	0.03
Maharashtra										**ORF1b:T547S**	1	0.03
Maharashtra										**ORF1b:A549V**	1	0.03
Maharashtra										**ORF1b:V551***	1	0.03
Maharashtra										**ORF1b:S552P**	1	0.03
Maharashtra										**ORF1b:C554D**	1	0.03
Maharashtra										**ORF1b:T556F**	1	0.03
Maharashtra										**ORF1b:T558K**	1	0.03
Maharashtra										**ORF1b:T577M**	1	0.03
Maharashtra										**ORF1b:I580G**	1	0.03
Maharashtra										**ORF1b:G581T**	1	0.03
Maharashtra										**ORF1b:L593***	1	0.03
Maharashtra										**ORF1b:V596L**	1	0.03
Maharashtra										**ORF1b:Y597T**	1	0.03
Maharashtra										**ORF1b:S598L**	1	0.03
Maharashtra										**ORF1b:D599W**	1	0.03
Maharashtra										**ORF1b:M617G**	1	0.03
Maharashtra										**ORF1b:P618I**	1	0.03
Maharashtra										**ORF1b:N619I**	1	0.03
Maharashtra										**ORF1b:M620L**	1	0.03
Maharashtra										**ORF1b:L621N**	1	0.03
Maharashtra										**ORF1b:R622V**	1	0.03
Maharashtra										**ORF1b:M624E**	1	0.03
Maharashtra										**ORF1b:A625P**	1	0.03
Maharashtra										**ORF1b:S626C**	1	0.03
Maharashtra										**ORF1b:L629V**	1	0.03
Maharashtra										**ORF1b:A651H**	1	0.03
Maharashtra										**ORF1b:Q652T**	1	0.03
Maharashtra										**ORF1b:L654S**	1	0.03
Maharashtra										**ORF1b:S655I**	1	0.03
Maharashtra										**ORF1b:E656D**	1	0.03
Maharashtra										**ORF1b:M657***	1	0.03
Maharashtra										**ORF1b:V658L**	1	0.03
Maharashtra										**ORF1b:C660S**	1	0.03
Maharashtra										**ORF1b:G661V**	1	0.03
Maharashtra										**ORF1b:G662L**	1	0.03
Maharashtra										**ORF1b:S663K**	1	0.03
Maharashtra										**ORF1b:L664Y**	1	0.03
Maharashtra										**ORF1b:Y665***	1	0.03
Maharashtra										**ORF1b:S1003***	1	0.03
Maharashtra										**ORF1b:P1005S**	1	0.03
Maharashtra										**ORF1b:L1006I**	1	0.03
Maharashtra										**ORF1b:L1086I**	1	0.03
Maharashtra										**ORF1b:G1561S**	1	0.03
Maharashtra										**ORF1b:R1315L**	1	0.03
Maharashtra										**ORF1b:K151R**	1	0.03
Maharashtra										**ORF1b:A391S**	1	0.03
Maharashtra										**ORF1b:A2575V**	1	0.03
Maharashtra										**ORF1b:P909H**	1	0.03
Maharashtra										**ORF1b:K1724R**	1	0.03
Maharashtra										**ORF1b:T1122H**	1	0.03
Maharashtra										**ORF1b:F1123L**	1	0.03
Maharashtra										**ORF1b:E1124***	1	0.03
Maharashtra										**ORF1b:G1126R**	1	0.03
Maharashtra										**ORF1b:D1127***	1	0.03
Maharashtra										**ORF1b:Y1128L**	1	0.03
Maharashtra										**ORF1b:R2112Y**	1	0.03
Maharashtra										**ORF1b:N2113S**	1	0.03
Maharashtra										**ORF1b:P2204K**	1	0.03
Maharashtra										**ORF1b:S2205V**	1	0.03
Maharashtra										**ORF1b:G2207L**	1	0.03

Maharashtra										**ORF1b:P2208K**	1	0.03
Maharashtra										**ORF1b:K2209V**	1	0.03
Maharashtra										**ORF1b:Q2210Y**	1	0.03
Maharashtra										**ORF1b:A2211N**	1	0.03
Maharashtra										**ORF1b:S2212H**	1	0.03
Maharashtra										**ORF1b:V2216L**	1	0.03
Maharashtra										**ORF1b:T2217V**	1	0.03
Maharashtra										**ORF1b:G2220R**	1	0.03
Maharashtra										**ORF1b:E2221K**	1	0.03
Maharashtra										**ORF1b:V2223M**	1	0.03
Maharashtra										**ORF1b:K2224V**	1	0.03
Maharashtra										**ORF1b:T2225L**	1	0.03
Maharashtra										**ORF1b:Q2226S**	1	0.03
Maharashtra										**ORF1b:F2227N**	1	0.03
Maharashtra										**ORF1b:Y2230L**	1	0.03
Maharashtra										**ORF1b:F2245L**	1	0.03
Maharashtra										**ORF1b:Q2247L**	1	0.03
Maharashtra										**ORF1b:S2248L**	1	0.03
Maharashtra										**ORF1b:N2250K**	1	0.03
Maharashtra										**ORF1b:E2274S**	1	0.03
Maharashtra										**ORF1b:R2275***	1	0.03
Maharashtra										**ORF1b:K2277***	1	0.03
Maharashtra										**ORF1b:H2300I**	1	0.03
Maharashtra										**ORF1b:I2357R**	1	0.03
Maharashtra										**ORF1b:K2358L**	1	0.03
Maharashtra										**ORF1b:Q2360K**	1	0.03
Maharashtra										**ORF1b:D2361***	1	0.03
Maharashtra										**ORF1b:Y2375L**	1	0.03
Maharashtra										**ORF1b:E2377I**	1	0.03
Maharashtra										**ORF1b:I2378Q**	1	0.03
Maharashtra										**ORF1b:S2379K**	1	0.03
Maharashtra										**ORF1b:M2381H**	1	0.03
Maharashtra										**ORF1b:T2391C**	1	0.03
Maharashtra										**ORF1b:Y2393G**	1	0.03
Maharashtra										**ORF1b:P2394V**	1	0.03
Maharashtra										**ORF1b:M2417C**	1	0.03
Maharashtra										**ORF1b:L2419I**	1	0.03
Maharashtra										**ORF1b:E2420F**	1	0.03
Maharashtra										**ORF1b:K2421T**	1	0.03
Maharashtra										**ORF1b:C2422K**	1	0.03
Maharashtra										**ORF1b:D2423C**	1	0.03
Maharashtra										**ORF1b:L2424K**	1	0.03
Maharashtra										**ORF1b:Q2425E**	1	0.03
Maharashtra										**ORF1b:N2426C**	1	0.03
Maharashtra										**ORF1b:G2428***	1	0.03
Maharashtra										**ORF1b:D2429K**	1	0.03
Maharashtra										**ORF1b:K2435F**	1	0.03
Maharashtra										**ORF1b:G2436K**	1	0.03
Maharashtra										**ORF1b:M2439V**	1	0.03
Maharashtra										**ORF1b:N2440I**	1	0.03
Maharashtra										**ORF1b:A2442Q**	1	0.03
Maharashtra										**ORF1b:K2443H**	1	0.03
Maharashtra										**ORF1b:T2445H**	1	0.03
Maharashtra										**ORF1b:Q2446***	1	0.03
Maharashtra										**ORF1b:C2448Y**	1	0.03
Maharashtra										**ORF1b:Q2449P**	1	0.03
Maharashtra										**ORF1b:Y2450I**	1	0.03
Maharashtra										**ORF1b:L2451I**	1	0.03
Maharashtra										**ORF1b:N2452***	1	0.03
Maharashtra										**ORF1b:T2453E**	1	0.03
Maharashtra										**ORF1b:T2455Y**	1	0.03
Maharashtra										**ORF1b:L2456I**	1	0.03
Maharashtra										**ORF1b:A2457L**	1	0.03
Maharashtra										**ORF1b:A2480L**	1	0.03
Maharashtra										**ORF1b:R2483I**	1	0.03
Maharashtra										**ORF1b:Q2484K**	1	0.03
Maharashtra										**ORF1b:W2485E**	1	0.03
Maharashtra										**ORF1b:P2487H**	1	0.03
Maharashtra										**ORF1b:T2488Q**	1	0.03
Maharashtra										**ORF1b:G2489V**	1	0.03
Maharashtra										**ORF1b:T2490Q**	1	0.03
Maharashtra										**ORF1b:V2493***	1	0.03
Maharashtra										**ORF1b:D2496L**	1	0.03

Maharashtra										**ORF1b:L2497***	1	0.03
Maharashtra										**ORF1b:N2498C**	1	0.03
Maharashtra										**ORF1b:D2499R**	1	0.03
Maharashtra										**ORF1b:V2501N**	1	0.03
Maharashtra										**ORF1b:S2502F**	1	0.03
Maharashtra										**ORF1b:Q2295H**	1	0.03
Maharashtra										**ORF1b:Y2619C**	1	0.03
Maharashtra										**ORF1b:V2656I**	1	0.03
Maharashtra										**ORF1b:F1004Y**	1	0.03
Maharashtra										**ORF1b:K1115L**	1	0.03
Maharashtra										**ORF1b:Q1117V**	1	0.03
Maharashtra										**ORF1b:H1792M**	1	0.03
Maharashtra										**ORF1b:K2209Q**	1	0.03
Maharashtra										**ORF1b:Q2210T**	1	0.03
Maharashtra										**ORF1b:T547A**	1	0.03
Maharashtra										**ORF1b:V548P**	1	0.03
Maharashtra										**ORF1b:A549***	1	0.03
Maharashtra										**ORF1b:I553L**	1	0.03
Maharashtra										**ORF1b:C554S**	1	0.03
Maharashtra										**ORF1b:S555V**	1	0.03
Maharashtra										**ORF1b:T556V**	1	0.03
Maharashtra										**ORF1b:M557Y**	1	0.03
Maharashtra										**ORF1b:T558D**	1	0.03
Maharashtra										**ORF1b:N559Q**	1	0.03
Maharashtra										**ORF1b:R560***	1	0.03
Maharashtra										**ORF1b:G843A**	1	0.03
Maharashtra										**ORF1b:T844V**	1	0.03
Maharashtra										**ORF1b:K862H**	1	0.03
Maharashtra										**ORF1b:H863L**	1	0.03
Maharashtra										**ORF1b:P864L**	1	0.03
Maharashtra										**ORF1b:H873I**	1	0.03
Maharashtra										**ORF1b:R1096***	1	0.03
Maharashtra										**ORF1b:V1132C**	1	0.03
Maharashtra										**ORF1b:H1153A**	1	0.03
Maharashtra										**ORF1b:V1155R**	1	0.03
Maharashtra										**ORF1b:M1156A**	1	0.03
Maharashtra										**ORF1b:P1157L**	1	0.03
Maharashtra										**ORF1b:L1158C**	1	0.03
Maharashtra										**ORF1b:S1159***	1	0.03
Maharashtra										**ORF1b:A1160N**	1	0.03
Maharashtra										**ORF1b:P1161Y**	1	0.03
Maharashtra										**ORF1b:T1162W**	1	0.03
Maharashtra										**ORF1b:V1164I**	1	0.03
Maharashtra										**ORF1b:Q1166N**	1	0.03
Maharashtra										**ORF1b:E1167T**	1	0.03
Maharashtra										**ORF1b:H1168Q**	1	0.03
Maharashtra										**ORF1b:V1170L**	1	0.03
Maharashtra										**ORF1b:K1194***	1	0.03
Maharashtra										**ORF1b:G1196F**	1	0.03
Maharashtra										**ORF1b:M1197***	1	0.03
Maharashtra										**ORF1b:K1199W**	1	0.03
Maharashtra										**ORF1b:S1201A**	1	0.03
Maharashtra										**ORF1b:T1202K**	1	0.03
Maharashtra										**ORF1b:L1203V**	1	0.03
Maharashtra										**ORF1b:E1298C**	1	0.03
Maharashtra										**ORF1b:I1299Q**	1	0.03
Maharashtra										**ORF1b:S1300C**	1	0.03
Maharashtra										**ORF1b:M1301Q**	1	0.03
Maharashtra										**ORF1b:A1302I**	1	0.03
Maharashtra										**ORF1b:N1304C**	1	0.03
Maharashtra										**ORF1b:Y1305***	1	0.03
Maharashtra										**ORF1b:D1306A**	1	0.03
Maharashtra										**ORF1b:S1308C**	1	0.03
Maharashtra										**ORF1b:A1326H**	1	0.03
Maharashtra										**ORF1b:Q1327W**	1	0.03
Maharashtra										**ORF1b:L1328R**	1	0.03
Maharashtra										**ORF1b:A1330C**	1	0.03
Maharashtra										**ORF1b:P1331S**	1	0.03
Maharashtra										**ORF1b:R1332I**	1	0.03
Maharashtra										**ORF1b:L1335T**	1	0.03
Maharashtra										**ORF1b:K1337V**	1	0.03
Maharashtra										**ORF1b:G1338***	1	0.03
Maharashtra										**ORF1b:L1340Y**	1	0.03

Maharashtra										**ORF1b:C1364N**	1	0.03
Maharashtra										**ORF1b:R1365Y**	1	0.03
Maharashtra										**ORF1b:H1405***	1	0.03
Maharashtra										**ORF1b:D1406N**	1	0.03
Maharashtra										**ORF1b:Q1441T**	1	0.03
Maharashtra										**ORF1b:A1443S**	1	0.03
Maharashtra										**ORF1b:V1444N**	1	0.03
Maharashtra										**ORF1b:A1445C**	1	0.03
Maharashtra										**ORF1b:S1446***	1	0.03
Maharashtra										**ORF1b:K1447F**	1	0.03
Maharashtra										**ORF1b:L1449T**	1	0.03
Maharashtra										**ORF1b:T1470R**	1	0.03
Maharashtra										**ORF1b:Q1471I**	1	0.03
Maharashtra										**ORF1b:T1472***	1	0.03
Maharashtra										**ORF1b:T1473L**	1	0.03
Maharashtra										**ORF1b:E1474C**	1	0.03
Maharashtra										**ORF1b:T1475H**	1	0.03
Maharashtra										**ORF1b:A1476I**	1	0.03
Maharashtra										**ORF1b:A1521R**	1	0.03
Maharashtra										**ORF1b:T1540***	1	0.03
Maharashtra										**ORF1b:G1541R**	1	0.03
Maharashtra										**ORF1b:P1544***	1	0.03
Maharashtra										**ORF1b:T1545I**	1	0.03
Maharashtra										**ORF1b:T1697C**	1	0.03
Maharashtra										**ORF1b:H1712M**	1	0.03
Maharashtra										**ORF1b:T1902R**	1	0.03
Maharashtra										**ORF1b:D1903***	1	0.03
Maharashtra										**ORF1b:G1904I**	1	0.03
Maharashtra										**ORF1b:V1905S**	1	0.03
Maharashtra										**ORF1b:L1907***	1	0.03
Maharashtra										**ORF1b:S1931H**	1	0.03
Maharashtra										**ORF1b:N1934C**	1	0.03
Maharashtra										**ORF1b:L1935K**	1	0.03
Maharashtra										**ORF1b:P1936***	1	0.03
Maharashtra										**ORF1b:G1937T**	1	0.03
Maharashtra										**ORF1b:F2185R**	1	0.03
Maharashtra										**ORF1b:N2187***	1	0.03
Maharashtra										**ORF1b:A2188C**	1	0.03
Maharashtra										**ORF1b:R2189***	1	0.03
Maharashtra										**ORF1b:N2190R**	1	0.03
Maharashtra										**ORF1b:G2191F**	1	0.03
Maharashtra										**ORF1b:V2192T**	1	0.03
Maharashtra										**ORF1b:G2207C**	1	0.03
Maharashtra										**ORF1b:P2208R**	1	0.03
Maharashtra										**ORF1b:K2209S**	1	0.03
Maharashtra										**ORF1b:F2380N**	1	0.03
Maharashtra										**ORF1b:M2381I**	1	0.03
Maharashtra										**ORF1b:T2432V**	1	0.03
Maharashtra										**ORF1b:L2433***	1	0.03
Maharashtra										**ORF1b:Q2449I**	1	0.03
Maharashtra										**ORF1b:L2451S**	1	0.03
Maharashtra										**ORF1b:N2452C**	1	0.03
Maharashtra										**ORF1b:G2468***	1	0.03
Maharashtra										**ORF1b:A2469Y**	1	0.03
Maharashtra										**ORF1b:G2470E**	1	0.03
Maharashtra										**ORF1b:D2472K**	1	0.03
Maharashtra										**ORF1b:K2473T**	1	0.03
Maharashtra										**ORF1b:G2474V**	1	0.03
Maharashtra										**ORF1b:P2477Y**	1	0.03
Maharashtra										**ORF1b:T2479Y**	1	0.03
Maharashtra										**ORF1b:V2481A**	1	0.03
Maharashtra										**ORF1b:L2482C**	1	0.03
Maharashtra										**ORF1b:V2501R**	1	0.03
Maharashtra										**ORF1b:D2503C**	1	0.03
Maharashtra										**ORF1b:T1178N**	1	0.03
Maharashtra										**ORF1b:L1179T**	1	0.03
Maharashtra										**ORF1b:N1180Q**	1	0.03
Maharashtra										**ORF1b:I1181Y**	1	0.03
Maharashtra										**ORF1b:F2058D**	1	0.03
Maharashtra										**ORF1b:N2059F**	1	0.03
Maharashtra										**ORF1b:V2060R**	1	0.03
Maharashtra										**ORF1b:N2062***	1	0.03
Maharashtra										**ORF1b:V2082***	1	0.03

Maharashtra										**ORF1b:Y2083S**	1	0.03
Maharashtra										**ORF1b:G2180***	1	0.03
Maharashtra										**ORF1b:Q2181S**	1	0.03
Maharashtra										**ORF1b:V2182***	1	0.03
Maharashtra										**ORF1b:D2183W**	1	0.03
Maharashtra										**ORF1b:L2184S**	1	0.03
Maharashtra										**ORF1b:F2185S**	1	0.03
Maharashtra										**ORF1b:G2215T**	1	0.03
Maharashtra										**ORF1b:T2217Q**	1	0.03
Maharashtra										**ORF1b:I2219L**	1	0.03
Maharashtra										**ORF1b:G2220***	1	0.03
Maharashtra										**ORF1b:V2233S**	1	0.03
Maharashtra										**ORF1b:D2234C**	1	0.03
Maharashtra										**ORF1b:V1133F**	1	0.03
Maharashtra										**ORF1b:V1164M**	1	0.03
Maharashtra										**ORF1b:T984A**	1	0.03
Maharashtra										**ORF1b:V1961G**	1	0.03
Maharashtra										**ORF1b:E1977V**	1	0.03
Maharashtra										**ORF1b:Q1982G**	1	0.03
Maharashtra										**ORF1b:V1983R**	1	0.03
Maharashtra										**ORF1b:T1996K**	1	0.03
Maharashtra										**ORF1b:C1997H**	1	0.03
Maharashtra										**ORF1b:G2029W**	1	0.03
Maharashtra										**ORF1b:F2030L**	1	0.03
Maharashtra										**ORF1b:S2031Y**	1	0.03
Maharashtra										**ORF1b:V1984M**	1	0.03
Maharashtra										**ORF1b:P1991L**	1	0.03
Maharashtra										**ORF1b:R2000H**	1	0.03
Maharashtra										**ORF1b:T2156L**	1	0.03
Maharashtra										**ORF1b:Y1944D**	1	0.03
Maharashtra										**ORF1b:T1952P**	1	0.03
Maharashtra										**ORF1b:P1953H**	1	0.03
Maharashtra										**ORF1b:C2008Y**	1	0.03
Maharashtra										**ORF1b:I2616K**	1	0.03
Maharashtra										**ORF1b:S2312T**	1	0.03
Maharashtra										**ORF1b:H2571R**	1	0.03
Maharashtra										**ORF1b:V2591C**	1	0.03
Maharashtra										**ORF1b:R2009S**	1	0.03
Maharashtra										**ORF1b:N2328T**	1	0.03
Maharashtra										**ORF1b:L1943T**	1	0.03
Maharashtra										**ORF1b:S1978F**	1	0.03
Maharashtra										**ORF1b:S2638F**	1	0.03
Maharashtra										**ORF1b:Y2639H**	1	0.03
Maharashtra										**ORF1b:V1961D**	1	0.03
Maharashtra										**ORF1b:R2613N**	1	0.03
Maharashtra										**ORF1b:Q2635C**	1	0.03
Maharashtra										**ORF1b:S2637F**	1	0.03
Maharashtra										**ORF1b:Y2639C**	1	0.03
Maharashtra										**ORF1b:R2629S**	1	0.03
Maharashtra										**ORF1b:N2632Y**	1	0.03
Maharashtra										**ORF1b:I2194L**	1	0.03
Maharashtra										**ORF1b:T2195R**	1	0.03
Maharashtra										**ORF1b:L2202G**	1	0.03
Maharashtra										**ORF1b:P2162Q**	1	0.03
Maharashtra										**ORF1b:T2163L**	1	0.03
Maharashtra										**ORF1b:V2237D**	1	0.03
Maharashtra										**ORF1b:Q2238G**	1	0.03
Maharashtra										**ORF1b:G1530Q**	1	0.03
Maharashtra										**ORF1b:C2551F**	1	0.03
Maharashtra										**ORF1b:F433V**	1	0.03
Maharashtra										**ORF1b:D2690S**	1	0.03
Maharashtra										**ORF1b:P909L**	1	0.03
Maharashtra										**ORF1b:T2654M**	1	0.03
Maharashtra										**ORF1b:M2657Y**	1	0.03
Maharashtra										**ORF1b:S2658V**	1	0.03
Maharashtra										**ORF1b:S375F**	1	0.03
Maharashtra										**ORF1b:D2333T**	1	0.03
Maharashtra										**ORF1b:A2334L**	1	0.03
Maharashtra										**ORF1b:Q2335E**	1	0.03
Maharashtra										**ORF1b:H2388Q**	1	0.03
Maharashtra										**ORF1b:V2389I**	1	0.03
Maharashtra										**ORF1b:E2390N**	1	0.03
Maharashtra										**ORF1b:T2391L**	1	0.03

Maharashtra										**ORF1b:V345***	1	0.03
Maharashtra										**ORF1b:T1774S**	1	0.03
Maharashtra										**ORF1b:G1775T**	1	0.03
Maharashtra										**ORF1b:H286T**	1	0.03
Maharashtra										**ORF1b:D349N**	1	0.03
Maharashtra										**ORF1b:T1817A**	1	0.03
Maharashtra										**ORF1b:V345F**	1	0.03
Maharashtra										**ORF1b:V332F**	1	0.03
Maharashtra										**ORF1b:T335I**	1	0.03
Maharashtra										**ORF1b:G336C**	1	0.03
Maharashtra										**ORF1b:Y337I**	1	0.03
Maharashtra										**ORF1b:H338P**	1	0.03
Maharashtra										**ORF1b:F339L**	1	0.03
Maharashtra										**ORF1b:D349A**	1	0.03
Maharashtra										**ORF1b:L1088P**	1	0.03
Maharashtra										**ORF1b:S1089F**	1	0.03
Maharashtra										**ORF1b:W1090R**	1	0.03
Maharashtra										**ORF1b:I1766V**	1	0.03
Maharashtra										**ORF1b:S2359Y**	1	0.03
Maharashtra										**ORF1b:I136T**	1	0.03
Maharashtra										**ORF1b:F95I**	1	0.03
Maharashtra										**ORF1b:H801D**	1	0.03
Maharashtra										**ORF1b:S2673I**	1	0.03
Maharashtra										**ORF1b:S2359A**	1	0.03
Maharashtra										**ORF1b:K491R**	1	0.03
Maharashtra										**ORF1b:A440V**	1	0.03
Maharashtra										**ORF1b:D1373N**	1	0.03
Maharashtra										**ORF1b:R2613S**	1	0.03
Maharashtra										**ORF1b:M2025T**	1	0.03
Maharashtra										**ORF1b:V1092A**	1	0.03
Maharashtra										**ORF1b:S220N**	1	0.03
Maharashtra										**ORF1b:R1262H**	1	0.03
Maharashtra										**ORF1b:S1931T**	1	0.03
Maharashtra										**ORF1b:S2148Y**	1	0.03
Maharashtra										**ORF1b:T2149L**	1	0.03
Maharashtra										**ORF1b:I2150L**	1	0.03
Maharashtra										**ORF1b:G2151L**	1	0.03
Maharashtra										**ORF1b:K2160F**	1	0.03
Maharashtra										**ORF1b:K2161V**	1	0.03
Maharashtra										**ORF1b:T2163***	1	0.03
Maharashtra										**ORF1b:E2164L**	1	0.03
Maharashtra										**ORF1b:Y2244C**	1	0.03
Maharashtra										**ORF1b:F2245P**	1	0.03
Maharashtra										**ORF1b:Q2247I**	1	0.03
Maharashtra										**ORF1b:S2248T**	1	0.03
Maharashtra										**ORF1b:R2249***	1	0.03
Maharashtra										**ORF1b:V1922P**	1	0.03
Maharashtra										**ORF1b:C1923L**	1	0.03
Maharashtra										**ORF1b:R1924F**	1	0.03
Maharashtra										**ORF1b:F1925V**	1	0.03
Maharashtra										**ORF1b:T1927L**	1	0.03
Maharashtra										**ORF1b:R1928T**	1	0.03
Maharashtra										**ORF1b:V1929L**	1	0.03
Maharashtra										**ORF1b:L1930E**	1	0.03
Maharashtra										**ORF1b:S1931C**	1	0.03
Maharashtra										**ORF1b:N1932Y**	1	0.03
Maharashtra										**ORF1b:N1934T**	1	0.03
Maharashtra										**ORF1b:G1941T**	1	0.03
Maharashtra										**ORF1b:P2204L**	1	0.03
Maharashtra										**ORF1b:S2205***	1	0.03
Maharashtra										**ORF1b:G2207P**	1	0.03
Maharashtra										**ORF1b:P2208N**	1	0.03
Maharashtra										**ORF1b:P2241V**	1	0.03
Maharashtra										**ORF1b:T2243I**	1	0.03
Maharashtra										**ORF1b:D2263K**	1	0.03
Maharashtra										**ORF1b:F2264N**	1	0.03
Maharashtra										**ORF1b:E2266***	1	0.03
Maharashtra										**ORF1b:A2268W**	1	0.03
Maharashtra										**ORF1b:K2277S**	1	0.03
Maharashtra										**ORF1b:E2279N**	1	0.03
Maharashtra										**ORF1b:Y2281I**	1	0.03
Maharashtra										**ORF1b:A2282S**	1	0.03
Maharashtra										**ORF1b:E2284M**	1	0.03

Maharashtra										**ORF1b:H2285E**	1	0.03
Maharashtra										**ORF1b:K2358Y**	1	0.03
Maharashtra										**ORF1b:S2359L**	1	0.03
Maharashtra										**ORF1b:Q2360***	1	0.03
Maharashtra										**ORF1b:D2361F**	1	0.03
Maharashtra										**ORF1b:S2363R**	1	0.03
Maharashtra										**ORF1b:V2364L**	1	0.03
Maharashtra										**ORF1b:V2365Q**	1	0.03
Maharashtra										**ORF1b:M2414V**	1	0.03
Maharashtra										**ORF1b:Q2415***	1	0.03
Maharashtra										**ORF1b:R2416P**	1	0.03
Maharashtra										**ORF1b:M2417S**	1	0.03
Maharashtra										**ORF1b:L2418K**	1	0.03
Maharashtra										**ORF1b:G2428C**	1	0.03
Maharashtra										**ORF1b:P1368S**	1	0.03
Maharashtra										**ORF1b:I1371L**	1	0.03
Maharashtra										**ORF1b:D1373G**	1	0.03
Maharashtra										**ORF1b:T1374V**	1	0.03
Maharashtra										**ORF1b:S1376L**	1	0.03
Maharashtra										**ORF1b:A1377K**	1	0.03
Maharashtra										**ORF1b:L1378H**	1	0.03
Maharashtra										**ORF1b:M1581***	1	0.03
Maharashtra										**ORF1b:M1582W**	1	0.03
Maharashtra										**ORF1b:G1583L**	1	0.03
Maharashtra										**ORF1b:F1584P**	1	0.03
Maharashtra										**ORF1b:K1585***	1	0.03
Maharashtra										**ORF1b:M1586P**	1	0.03
Maharashtra										**ORF1b:N1587V**	1	0.03
Maharashtra										**ORF1b:V1607C**	1	0.03
Maharashtra										**ORF1b:N2214R**	1	0.03
Maharashtra										**ORF1b:V2216N**	1	0.03
Maharashtra										**ORF1b:K2413V**	1	0.03
Maharashtra										**ORF1b:M2414***	1	0.03
Maharashtra										**ORF1b:Q2415P**	1	0.03
Maharashtra										**ORF1b:R2416S**	1	0.03
Maharashtra										**ORF1b:M2417K**	1	0.03
Maharashtra										**ORF1b:L2419W**	1	0.03
Maharashtra										**ORF1b:E2420***	1	0.03
Maharashtra										**ORF1b:K2421***	1	0.03
Maharashtra										**ORF1b:V2441I**	1	0.03
Maharashtra										**ORF1b:A2442F**	1	0.03
Maharashtra										**ORF1b:A86V**	1	0.03
Maharashtra										**ORF1b:T1289M**	1	0.03
Maharashtra										**ORF1b:G328C**	1	0.03
Maharashtra										**ORF1b:H2571Y**	1	0.03
Maharashtra										**ORF1b:S2599***	1	0.03
Maharashtra										**ORF1b:E2600V**	1	0.03
Maharashtra										**ORF1b:A2601M**	1	0.03
Maharashtra										**ORF1b:F2602S**	1	0.03
Maharashtra										**ORF1b:I2604E**	1	0.03
Maharashtra										**ORF1b:G2605E**	1	0.03
Maharashtra										**ORF1b:C2606Y**	1	0.03
Maharashtra										**ORF1b:G2618C**	1	0.03
Maharashtra										**ORF1b:T2569N**	1	0.03
Maharashtra										**ORF1b:R2000Q**	1	0.03
Maharashtra										**ORF1b:L1943K**	1	0.03
Maharashtra										**ORF1b:K1947***	1	0.03
Maharashtra										**ORF1b:Q1965K**	1	0.03
Maharashtra										**ORF1b:G1010E**	1	0.03
Maharashtra										**ORF1b:V1080L**	1	0.03
Maharashtra										**ORF1b:V486I**	1	0.03
Maharashtra										**ORF1b:L56S**	1	0.03
Maharashtra										**ORF1b:R270S**	1	0.03
Maharashtra										**ORF1b:P1851S**	1	0.03
Maharashtra										**ORF1b:R1704S**	1	0.03
Maharashtra										**ORF1b:G2280S**	1	0.03
Maharashtra										**ORF1b:D2179Y**	1	0.03
Maharashtra										**ORF1b:Y285H**	1	0.03
Maharashtra										**ORF1b:D294E**	1	0.03
Maharashtra										**ORF1b:D295N**	1	0.03
Maharashtra										**ORF1b:C297***	1	0.03
Maharashtra										**ORF1b:I298S**	1	0.03
Maharashtra										**ORF1b:C301N**	1	0.03

Maharashtra										**ORF1b:N303E**	1	0.03
Maharashtra										**ORF1b:F304C**	1	0.03
Maharashtra										**ORF1b:F308S**	1	0.03
Maharashtra										**ORF1b:R322I**	1	0.03
Maharashtra										**ORF1b:K323P**	1	0.03
Maharashtra										**ORF1b:V326K**	1	0.03
Maharashtra										**ORF1b:D327I**	1	0.03
Maharashtra										**ORF1b:G328T**	1	0.03
Maharashtra										**ORF1b:P330K**	1	0.03
Maharashtra										**ORF1b:S334L**	1	0.03
Maharashtra										**ORF1b:A167V**	1	0.03
Maharashtra										**ORF1b:T1470A**	1	0.03
Maharashtra										**ORF1b:M2462I**	1	0.03
Maharashtra										**ORF1b:Y1944***	1	0.03
Maharashtra										**ORF1b:I2026S**	1	0.03
Maharashtra										**ORF1b:S2027L**	1	0.03
Maharashtra										**ORF1b:V1133M**	1	0.03
Maharashtra										**ORF1b:Y1134L**	1	0.03
Maharashtra										**ORF1b:R1135L**	1	0.03
Maharashtra										**ORF1b:G1136F**	1	0.03
Maharashtra										**ORF1b:T1545S**	1	0.03
Maharashtra										**ORF1b:T1952Q**	1	0.03
Maharashtra										**ORF1b:A1954T**	1	0.03
Maharashtra										**ORF1b:E2539D**	1	0.03
Maharashtra										**ORF1b:G1937***	1	0.03
Maharashtra										**ORF1b:C1938L**	1	0.03
Maharashtra										**ORF1b:S1779N**	1	0.03
Maharashtra										**ORF1b:G1937C**	1	0.03
Maharashtra										**ORF1b:C1938H**	1	0.03
Maharashtra										**ORF1b:Y1944H**	1	0.03
Maharashtra										**ORF1b:V1945L**	1	0.03
Maharashtra										**ORF1b:G2561I**	1	0.03
Maharashtra										**ORF1b:G2562Q**	1	0.03
Maharashtra										**ORF1b:D2175Y**	1	0.03
Maharashtra										**ORF1b:L1099H**	1	0.03
Maharashtra										**ORF1b:Y1222C**	1	0.03
Maharashtra										**ORF1b:R240G**	1	0.03
Maharashtra										**ORF1b:S2324I**	1	0.03
Maharashtra										**ORF1b:Y819H**	1	0.03
Maharashtra										**ORF1b:A1428S**	1	0.03
Maharashtra										**ORF1b:K179Q**	1	0.03
Maharashtra										**ORF1b:D2270E**	1	0.03
Maharashtra										**ORF1b:A788V**	1	0.03
Maharashtra										**ORF1b:K2367E**	1	0.03
Maharashtra										**ORF1b:P970L**	1	0.03
Maharashtra										**ORF1b:A549S**	1	0.03
Maharashtra										**ORF1b:K1957R**	1	0.03
Maharashtra										**ORF1b:V2086F**	1	0.03
Maharashtra										**ORF1b:A1259T**	1	0.03
Maharashtra										**ORF1b:L1935F**	1	0.03
Maharashtra										**ORF1b:V2691F**	1	0.03
Maharashtra										**ORF1b:A2222V**	1	0.03
Maharashtra										**ORF1b:P1427S**	1	0.03
Maharashtra										**ORF1b:S2689G**	1	0.03
Maharashtra										**ORF1b:P1675L**	1	0.03
Maharashtra										**ORF1b:P2169S**	1	0.03
Maharashtra										**ORF1b:I958M**	1	0.03
Maharashtra										**ORF1b:D1127Y**	1	0.03
Maharashtra										**ORF1b:T2548N**	1	0.03
Maharashtra										**ORF1b:D145A**	1	0.03
Maharashtra										**ORF1b:E802G**	1	0.03
Maharashtra										**ORF1b:V222I**	1	0.03
Maharashtra										**ORF1b:E910D**	1	0.03
Maharashtra										**ORF1b:Y1399C**	1	0.03
Maharashtra										**ORF1b:M2025I**	1	0.03
Maharashtra										**ORF1b:M2381V**	1	0.03
Maharashtra										**ORF1b:D1882G**	1	0.03
Maharashtra										**ORF1b:V1984L**	1	0.03
Maharashtra										**ORF1b:I1579T**	1	0.03
Maharashtra										**ORF1b:S1186F**	1	0.03
Maharashtra										**ORF1b:D1973N**	1	0.03
Maharashtra										**ORF1b:S2359F**	1	0.03
Maharashtra										**ORF1b:L1218I**	1	0.03

Maharashtra										**ORF1b:N488T**	1	0.03
Maharashtra										**ORF1b:K568***	1	0.03
Maharashtra										**ORF1b:D1899N**	1	0.03
Maharashtra										**ORF1b:G2280V**	1	0.03
Maharashtra										**ORF1b:P2633***	1	0.03
Maharashtra										**ORF1b:A2006K**	1	0.03
Maharashtra										**ORF1b:R2009N**	1	0.03
Maharashtra										**ORF1b:E2092A**	1	0.03
Maharashtra										**ORF1b:G2197C**	1	0.03
Maharashtra										**ORF1b:T2171N**	1	0.03
Maharashtra										**ORF1b:I2286V**	1	0.03
Maharashtra										**ORF1b:V333M**	1	0.03
Maharashtra										**ORF1b:T335F**	1	0.03
Maharashtra										**ORF1b:G336T**	1	0.03
Maharashtra										**ORF1b:Y337S**	1	0.03
Maharashtra										**ORF1b:V344D**	1	0.03
Maharashtra										**ORF1b:V345M**	1	0.03
Maharashtra										**ORF1b:H346Y**	1	0.03
Maharashtra										**ORF1b:D349T**	1	0.03
Maharashtra										**ORF1b:I479V**	1	0.03
Maharashtra										**ORF1b:R2189S**	1	0.03
Maharashtra										**ORF1b:V5A**	1	0.03
Maharashtra										**ORF1b:S6G**	1	0.03
Maharashtra										**ORF1b:A7T**	1	0.03
Maharashtra										**ORF1b:S18V**	1	0.03
Maharashtra										**ORF1b:K2310S**	1	0.03
Maharashtra										**ORF1b:A2168V**	1	0.03
Maharashtra										**ORF1b:K82N**	1	0.03
Maharashtra										**ORF1b:P2074L**	1	0.03
Maharashtra										**ORF1b:V2365I**	1	0.03
Maharashtra										**ORF1b:S2339F**	1	0.03
Maharashtra										**ORF1b:C1938S**	1	0.03
Maharashtra										**ORF1b:D1939N**	1	0.03
Maharashtra										**ORF1b:G2562L**	1	0.03
Maharashtra										**ORF1b:S2563P**	1	0.03
Maharashtra										**ORF1b:N2695L**	1	0.03
Maharashtra										**ORF1b:W907F**	1	0.03
Maharashtra										**ORF1b:E908C**	1	0.03
Maharashtra										**ORF1b:P909N**	1	0.03
Maharashtra										**ORF1b:F911I**	1	0.03
Maharashtra										**ORF1b:W2628C**	1	0.03
Maharashtra										**ORF1b:L2316F**	1	0.03
Maharashtra										**ORF1b:G661C**	1	0.03
Maharashtra										**ORF1b:P2321S**	1	0.03
Maharashtra										**ORF1b:I2634V**	1	0.03
Maharashtra										**ORF1b:I1579V**	1	0.03
Maharashtra										**ORF1b:V1170A**	1	0.03
Maharashtra										**ORF1b:M1573I**	1	0.03
Maharashtra										**ORF1b:Y1319H**	1	0.03
Maharashtra										**ORF1b:D2472N**	1	0.03
Maharashtra										**ORF1b:L1179F**	1	0.03
Maharashtra										**ORF1b:A2596T**	1	0.03
Maharashtra										**ORF1b:N480S**	1	0.03
Maharashtra										**ORF1b:L2017F**	1	0.03
Maharashtra										**ORF1b:V2237F**	1	0.03
Maharashtra										**ORF1b:Y365H**	1	0.03
Maharashtra										**ORF1b:V1983I**	1	0.03
Maharashtra										**ORF1b:R741H**	1	0.03
Maharashtra										**ORF1b:A88T**	1	0.03
Maharashtra										**ORF1b:S316I**	1	0.03
Maharashtra										**ORF1b:T2514I**	1	0.03
Maharashtra										**ORF1b:R1813H**	1	0.03
Maharashtra										**ORF1b:V2060I**	1	0.03
Maharashtra										**ORF1b:H430Q**	1	0.03
Maharashtra										**ORF1b:L829I**	1	0.03
Maharashtra										**ORF1b:V1865L**	1	0.03
Maharashtra										**ORF1b:I2114V**	1	0.03
Maharashtra										**ORF1b:T197A**	1	0.03
Maharashtra										**ORF1b:V2371A**	1	0.03
Maharashtra										**ORF1b:T2372D**	1	0.03
Maharashtra										**ORF1b:I2373Y**	1	0.03
Maharashtra										**ORF1b:D2374***	1	0.03
Maharashtra										**ORF1b:F1004H**	1	0.03

Maharashtra										**ORF1b:A1063D**	1	0.03
Maharashtra										**ORF1b:T1064S**	1	0.03
Maharashtra										**ORF1b:E1065S**	1	0.03
Maharashtra										**ORF1b:E1066F**	1	0.03
Maharashtra										**ORF1b:T1067L**	1	0.03
Maharashtra										**ORF1b:F1068Q**	1	0.03
Maharashtra										**ORF1b:T1106L**	1	0.03
Maharashtra										**ORF1b:A1798M**	1	0.03
Maharashtra										**ORF1b:I1799V**	1	0.03
Maharashtra										**ORF1b:T1801H**	1	0.03
Maharashtra										**ORF1b:R1802M**	1	0.03
Maharashtra										**ORF1b:C1803***	1	0.03
Maharashtra										**ORF1b:A1805V**	1	0.03
Maharashtra										**ORF1b:N1830T**	1	0.03
Maharashtra										**ORF1b:N2113Q**	1	0.03
Maharashtra										**ORF1b:I2114H**	1	0.03
Maharashtra										**ORF1b:K2115***	1	0.03
Maharashtra										**ORF1b:P2116T**	1	0.03
Maharashtra										**ORF1b:G2289E**	1	0.03
Maharashtra										**ORF1b:G2191K**	1	0.03
Maharashtra										**ORF1b:V2192***	1	0.03
Maharashtra										**ORF1b:I2194Y**	1	0.03
Maharashtra										**ORF1b:T2195L**	1	0.03
Maharashtra										**ORF1b:G2197M**	1	0.03
Maharashtra										**ORF1b:S2198P**	1	0.03
Maharashtra										**ORF1b:Y1016***	1	0.03
Maharashtra										**ORF1b:W1429G**	1	0.03
Maharashtra										**ORF1b:R1430E**	1	0.03
Maharashtra										**ORF1b:V1444Y**	1	0.03
Maharashtra										**ORF1b:A1445Q**	1	0.03
Maharashtra										**ORF1b:S1446L**	1	0.03
Maharashtra										**ORF1b:I1448L**	1	0.03
Maharashtra										**ORF1b:P1652L**	1	0.03
Maharashtra										**ORF1b:F1722V**	1	0.03
Maharashtra										**ORF1b:V1723Y**	1	0.03
Maharashtra										**ORF1b:K1724V**	1	0.03
Maharashtra										**ORF1b:C1785L**	1	0.03
Maharashtra										**ORF1b:H1788T**	1	0.03
Maharashtra										**ORF1b:G1789Y**	1	0.03
Maharashtra										**ORF1b:N1790K**	1	0.03
Maharashtra										**ORF1b:H1792L**	1	0.03
Maharashtra										**ORF1b:V1793G**	1	0.03
Maharashtra										**ORF1b:V1905L**	1	0.03
Maharashtra										**ORF1b:I2078***	1	0.03
Maharashtra										**ORF1b:N2079W**	1	0.03
Maharashtra										**ORF1b:N2080T**	1	0.03
Maharashtra										**ORF1b:N2103I**	1	0.03
Maharashtra										**ORF1b:A2131G**	1	0.03
Maharashtra										**ORF1b:A2132E**	1	0.03
Maharashtra										**ORF1b:V2135D**	1	0.03
Maharashtra										**ORF1b:A2159***	1	0.03
Maharashtra										**ORF1b:K2161D**	1	0.03
Maharashtra										**ORF1b:T2163C**	1	0.03
Maharashtra										**ORF1b:E2164T**	1	0.03
Maharashtra										**ORF1b:I2166H**	1	0.03
Maharashtra										**ORF1b:V2371M**	1	0.03
Maharashtra										**ORF1b:I1002L**	1	0.03
Maharashtra										**ORF1b:S1003V**	1	0.03
Maharashtra										**ORF1b:P1005F**	1	0.03
Maharashtra										**ORF1b:C1007V**	1	0.03
Maharashtra										**ORF1b:G1136K**	1	0.03
Maharashtra										**ORF1b:T1137V**	1	0.03
Maharashtra										**ORF1b:T1139M**	1	0.03
Maharashtra										**ORF1b:K1141C**	1	0.03
Maharashtra										**ORF1b:L1142C**	1	0.03
Maharashtra										**ORF1b:N1143C**	1	0.03
Maharashtra										**ORF1b:D1899I**	1	0.03
Maharashtra										**ORF1b:K1900L**	1	0.03
Maharashtra										**ORF1b:T1902L**	1	0.03
Maharashtra										**ORF1b:D1903E**	1	0.03
Maharashtra										**ORF1b:V1905Q**	1	0.03
Maharashtra										**ORF1b:L1907R**	1	0.03
Maharashtra										**ORF1b:F1908***	1	0.03

Maharashtra										**ORF1b:W1909I**	1	0.03
Maharashtra										**ORF1b:N1910S**	1	0.03
Maharashtra										**ORF1b:Q1011L**	1	0.03
Maharashtra										**ORF1b:R1096K**	1	0.03
Maharashtra										**ORF1b:P1097L**	1	0.03
Maharashtra										**ORF1b:P1098C**	1	0.03
Maharashtra										**ORF1b:R1109W**	1	0.03
Maharashtra										**ORF1b:T1111S**	1	0.03
Maharashtra										**ORF1b:K1112C**	1	0.03
Maharashtra										**ORF1b:S1114***	1	0.03
Maharashtra										**ORF1b:V1870M**	1	0.03
Maharashtra										**ORF1b:W1872V**	1	0.03
Maharashtra										**ORF1b:K1873L**	1	0.03
Maharashtra										**ORF1b:F1874***	1	0.03
Maharashtra										**ORF1b:Y1875C**	1	0.03
Maharashtra										**ORF1b:D1876T**	1	0.03
Maharashtra										**ORF1b:N1946***	1	0.03
Maharashtra										**ORF1b:K1947W**	1	0.03
Maharashtra										**ORF1b:H1948W**	1	0.03
Maharashtra										**ORF1b:A1949Q**	1	0.03
Maharashtra										**ORF1b:N2214***	1	0.03
Maharashtra										**ORF1b:G2215***	1	0.03
Maharashtra										**ORF1b:D508N**	1	0.03
Maharashtra										**ORF1b:V1706L**	1	0.03
Maharashtra										**ORF1b:S2031N**	1	0.03
Maharashtra										**ORF1b:S1978V**	1	0.03
Maharashtra										**ORF1b:S1942E**	1	0.03
Maharashtra										**ORF1b:A2028V**	1	0.03
Maharashtra										**ORF1b:A1959L**	1	0.03
Maharashtra										**ORF1b:E2274D**	1	0.03
Maharashtra										**ORF1b:R2275E**	1	0.03
Maharashtra										**ORF1b:A2028N**	1	0.03
Maharashtra										**ORF1b:H2583P**	1	0.03
Maharashtra										**ORF1b:F2584R**	1	0.03
Maharashtra										**ORF1b:G1980A**	1	0.03
Maharashtra										**ORF1b:K1981I**	1	0.03
Maharashtra										**ORF1b:Q1982***	1	0.03
Maharashtra										**ORF1b:C1997S**	1	0.03
Maharashtra										**ORF1b:I1998V**	1	0.03
Maharashtra										**ORF1b:V1092I**	1	0.03
Maharashtra										**ORF1b:V596I**	1	0.03
Maharashtra										**ORF1b:H919R**	1	0.03
Maharashtra										**ORF1b:T17I**	1	0.03
Maharashtra										**ORF1b:V2192R**	1	0.03
Maharashtra										**ORF1b:K2310***	1	0.03
Maharashtra										**ORF1b:E2311G**	1	0.03
Maharashtra										**ORF1b:P2313T**	1	0.03
Maharashtra										**ORF1b:E2315***	1	0.03
Maharashtra										**ORF1b:L2316I**	1	0.03
Maharashtra										**ORF1b:D2318R**	1	0.03
Maharashtra										**ORF1b:I2331H**	1	0.03
Maharashtra										**ORF1b:T2332N**	1	0.03
Maharashtra										**ORF1b:D2333S**	1	0.03
Maharashtra										**ORF1b:Q2335H**	1	0.03
Maharashtra										**ORF1b:G1937S**	1	0.03
Maharashtra										**ORF1b:C1938V**	1	0.03
Maharashtra										**ORF1b:D1939V**	1	0.03
Maharashtra										**ORF1b:E269D**	1	0.03
Maharashtra										**ORF1b:G2561T**	1	0.03
Maharashtra										**ORF1b:G2562N**	1	0.03
Maharashtra										**ORF1b:G2562C**	1	0.03
Maharashtra										**ORF1b:G2561D**	1	0.03
Maharashtra										**ORF1b:G2562S**	1	0.03
Maharashtra										**ORF1b:C1938F**	1	0.03
Maharashtra										**ORF1b:G2561E**	1	0.03
Maharashtra										**ORF1b:A1432S**	1	0.03
Maharashtra										**ORF1b:Y993H**	1	0.03
Maharashtra										**ORF1b:M592I**	1	0.03
Maharashtra										**ORF1b:Y1466H**	1	0.03
Maharashtra										**ORF1b:K2340N**	1	0.03
Maharashtra										**ORF1b:Q483H**	1	0.03
Maharashtra										**ORF1b:E52G**	1	0.03
Maharashtra										**ORF1b:A167T**	1	0.03

Maharashtra										**ORF1b:V2685A**	1	0.03
Maharashtra										**ORF1b:V2369I**	1	0.03
Maharashtra										**ORF1b:H1168Y**	1	0.03
Maharashtra										**ORF1b:L2577F**	1	0.03
Maharashtra										**ORF1b:D51N**	1	0.03
Maharashtra										**ORF1b:K1194N**	1	0.03
Maharashtra										**ORF1b:H801P**	1	0.03
Maharashtra										**ORF1b:A7V**	1	0.03
Maharashtra										**ORF1b:D161G**	1	0.03
Maharashtra										**ORF1b:A1302V**	1	0.03
Maharashtra										**ORF1b:V102A**	1	0.03
Maharashtra										**ORF1b:V896I**	1	0.03
Maharashtra										**ORF1b:A1877V**	1	0.03
Maharashtra										**ORF1b:A1662V**	1	0.03
Maharashtra										**ORF1b:A2431T**	1	0.03
Maharashtra										**ORF1b:M1765I**	1	0.03
Maharashtra										**ORF1b:A1215S**	1	0.03
Maharashtra										**ORF1b:T76I**	1	0.03
Maharashtra										**ORF1b:A1620V**	1	0.03
Maharashtra										**ORF1b:S2363F**	1	0.03
Maharashtra										**ORF1b:F1969L**	1	0.03
Maharashtra										**ORF1b:V321L**	1	0.03
Maharashtra										**ORF1b:D152Y**	1	0.03
Maharashtra										**ORF1b:A1225T**	1	0.03
Maharashtra										**ORF1b:R2016***	1	0.03
Maharashtra										**ORF1b:A2012S**	1	0.03
Maharashtra										**ORF1b:A2028M**	1	0.03
Maharashtra										**ORF1b:S1942N**	1	0.03
Maharashtra										**ORF1b:T2569Q**	1	0.03
Maharashtra										**ORF1b:K2579M**	1	0.03
Maharashtra										**ORF1b:G1941A**	1	0.03
Maharashtra										**ORF1b:L2651I**	1	0.03
Maharashtra										**ORF1b:D884N**	1	0.03
Maharashtra										**ORF1b:R1813L**	1	0.03
Maharashtra										**ORF1b:V119I**	1	0.03
Maharashtra										**ORF1b:T1339I**	1	0.03
Maharashtra										**ORF1b:S69A**	1	0.03
Maharashtra										**ORF1b:W1751K**	1	0.03
Maharashtra										**ORF1b:E2014D**	1	0.03
Maharashtra										**ORF1b:M747I**	1	0.03
Maharashtra										**ORF1b:P909S**	1	0.03
Maharashtra										**ORF1b:A2596V**	1	0.03
Maharashtra										**ORF1b:E1264D**	1	0.03
Maharashtra										**ORF1b:M2581I**	1	0.03
Maharashtra										**ORF1b:G2582L**	1	0.03
Maharashtra										**ORF1b:R1600C**	1	0.03
Maharashtra										**ORF1b:V345L**	1	0.03
Maharashtra										**ORF1b:L2558F**	1	0.03
Maharashtra										**ORF1b:E52K**	1	0.03
Maharashtra										**ORF1b:P218T**	1	0.03
Maharashtra										**ORF1b:D2361Y**	1	0.03
Maharashtra										**ORF1b:M2417I**	1	0.03
Maharashtra										**ORF1b:D2142H**	1	0.03
Maharashtra										**ORF1b:G1174D**	1	0.03
Maharashtra										**ORF1b:G1756E**	1	0.03
Maharashtra										**ORF1b:G219S**	1	0.03
Maharashtra										**ORF1b:T1274I**	1	0.03
Maharashtra										**ORF1b:Q215P**	1	0.03
Maharashtra										**ORF1b:R2308C**	1	0.03
Maharashtra										**ORF1b:P2313S**	1	0.03
Maharashtra										**ORF1b:P1342S**	1	0.03
Maharashtra										**ORF1b:A927V**	1	0.03
Maharashtra										**ORF1b:V2086G**	1	0.03
Maharashtra										**ORF1b:T453I**	1	0.03
Maharashtra										**ORF1b:S663L**	1	0.03
Maharashtra										**ORF1b:P1917L**	1	0.03
Maharashtra										**ORF1b:L1220I**	1	0.03
Maharashtra										**ORF1b:A88V**	1	0.03
Maharashtra										**ORF1b:I527V**	1	0.03
Maharashtra										**ORF1b:D275G**	1	0.03
Maharashtra										**ORF1b:A373V**	1	0.03
Maharashtra										**ORF1b:G2201D**	1	0.03
Maharashtra										**ORF1b:H1550N**	1	0.03

Maharashtra										**ORF1b:D294N**	1	0.03
Maharashtra										**ORF1b:D295Y**	1	0.03
Maharashtra										**ORF1b:W2573R**	1	0.03
Maharashtra										**ORF1b:G703V**	1	0.03
Maharashtra										**ORF1b:S1273A**	1	0.03
Maharashtra										**ORF1b:T2453I**	1	0.03
Maharashtra										**ORF1b:A1432T**	1	0.03
Maharashtra										**ORF1b:S2339Y**	1	0.03
Maharashtra										**ORF1b:I2687V**	1	0.03
Maharashtra										**ORF1b:D1782G**	1	0.03
Maharashtra										**ORF1b:W1771C**	1	0.03
Maharashtra										**ORF1b:V1865I**	1	0.03
Maharashtra										**ORF1b:R1109C**	1	0.03
Maharashtra										**ORF1b:T861I**	1	0.03
Maharashtra										**ORF1b:L505F**	1	0.03
Maharashtra										**ORF1b:Y1200C**	1	0.03
Maharashtra										**ORF1b:Y2276C**	1	0.03
Maharashtra										**ORF1b:N1188D**	1	0.03
Maharashtra										**ORF1b:L1220P**	1	0.03
Maharashtra										**ORF1b:S1898F**	1	0.03
Maharashtra										**ORF1b:D2323Y**	1	0.03
Maharashtra										**ORF1b:A2457S**	1	0.03
Maharashtra										**ORF1b:P2531S**	1	0.03
Maharashtra										**ORF1b:S598I**	1	0.03
Maharashtra										**ORF1b:A2057V**	1	0.03
Maharashtra										**ORF1b:G2510C**	1	0.03
Maharashtra										**ORF1b:D1042H**	1	0.03
Maharashtra										**ORF1b:E1343K**	1	0.03
Maharashtra										**ORF1b:S1974N**	1	0.03
Maharashtra										**ORF1b:N734I**	1	0.03
Maharashtra										**ORF1b:D1899E**	1	0.03
Maharashtra										**ORF1b:A2028E**	1	0.03
Maharashtra										**ORF1b:G2029R**	1	0.03
Maharashtra										**ORF1b:F2030V**	1	0.03
Maharashtra										**ORF1b:A2028S**	1	0.03
Maharashtra										**ORF1b:S1958I**	1	0.03
Maharashtra										**ORF1b:P1975L**	1	0.03
Maharashtra										**ORF1b:R2016S**	1	0.03
Maharashtra										**ORF1b:L2017P**	1	0.03
Maharashtra										**ORF1b:Y2018C**	1	0.03
Maharashtra										**ORF1b:L1992***	1	0.03
Maharashtra										**ORF1b:C1997N**	1	0.03
Maharashtra										**ORF1b:S2027I**	1	0.03
Maharashtra										**ORF1b:A2028T**	1	0.03
Maharashtra										**ORF1b:I1987V**	1	0.03
Maharashtra										**ORF1b:Y1989Q**	1	0.03
Maharashtra										**ORF1b:A2028H**	1	0.03
Maharashtra										**ORF1b:T1952K**	1	0.03
Maharashtra										**ORF1b:S2031G**	1	0.03
Maharashtra										**ORF1b:G2610D**	1	0.03
Maharashtra										**ORF1b:K2611E**	1	0.03
Maharashtra										**ORF1b:I2566L**	1	0.03
Maharashtra										**ORF1b:F2174S**	1	0.03
Maharashtra										**ORF1b:P2633E**	1	0.03
Maharashtra										**ORF1b:G1941C**	1	0.03
Maharashtra										**ORF1b:C2008D**	1	0.03
Maharashtra										**ORF1b:L1943V**	1	0.03
Maharashtra										**ORF1b:V165I**	1	0.03
Maharashtra										**ORF1b:D445E**	1	0.03

	**Spike (S)**											
**Maharashtra**	**Pre-Wave 1**			**Wave 1**			**Pre-Wave 2**			**Wave 2**		
Maharashtra	**Mutation**	**Count**	**percent**	**Mutation**	**Count**	**percent**	**Mutation**	**Count**	**percent**	**Mutation**	**Count**	**percent**
Maharashtra	**S:D614G**	613	89.36	**S:D614G**	775	98.98	**S:D614G**	579	88.26	**S:D614G**	3252	91.92
Maharashtra	**S:T29I**	17	2.48	**S:P681H**	65	8.3	**S:L452R**	180	27.44	**S:P681R**	2641	74.65
Maharashtra	**S:Q677H**	10	1.46	**S:Q677H**	36	4.6	**S:P681R**	163	24.85	**S:L452R**	2611	73.8
Maharashtra	**S:A1078S**	7	1.02	**S:Q675H**	21	2.68	**S:E484Q**	152	23.17	**S:E484Q**	1551	43.84
Maharashtra	**S:D138Y**	7	1.02	**S:L18F**	17	2.17	**S:Q1071H**	115	17.53	**S:T19R**	1189	33.61
Maharashtra	**S:T22I**	6	0.87	**S:A771S**	16	2.04	**S:N440K**	89	13.57	**S:G142D**	1047	29.59
Maharashtra	**S:Q675H**	6	0.87	**S:N440K**	11	1.4	**S:E154K**	89	13.57	**S:T478K**	1033	29.2

Maharashtra	**S:G181A**	5	0.73	**S:L5F**	11	1.4	**S:T95I**	89	13.57	**S:E154K**	937	26.48
Maharashtra	**S:L5F**	5	0.73	**S:S98F**	11	1.4	**S:G142D**	80	12.2	**S:Q1071H**	799	22.58
Maharashtra	**S:R102I**	5	0.73	**S:D138Y**	8	1.02	**S:Q677H**	55	8.38	**S:T95I**	778	21.99
Maharashtra	**S:A520S**	3	0.44	**S:S943P**	7	0.89	**S:P681H**	53	8.08	**S:H1101D**	662	18.71
Maharashtra	**S:A879V**	3	0.44	**S:T1117I**	7	0.89	**S:L18F**	50	7.62	**S:D950N**	559	15.8
Maharashtra	**S:Q14H**	3	0.44	**S:Q677R**	7	0.89	**S:H1101D**	44	6.71	**S:A222V**	366	10.34
Maharashtra	**S:G1124V**	2	0.29	**S:T22A**	7	0.89	**S:T716I**	32	4.88	**S:R158G**	162	4.58
Maharashtra	**S:R214L**	2	0.29	**S:N679H**	6	0.77	**S:D1118H**	32	4.88	**S:P681H**	120	3.39
Maharashtra	**S:Q239R**	2	0.29	**S:S939F**	6	0.77	**S:Q675H**	31	4.73	**S:E484K**	109	3.08
Maharashtra	**S:A243S**	2	0.29	**S:T20I**	5	0.64	**S:N501Y**	31	4.73	**S:N501Y**	104	2.94
Maharashtra	**S:A845S**	2	0.29	**S:H49Y**	5	0.64	**S:A570D**	31	4.73	**S:Q677H**	102	2.88
Maharashtra	**S:Q836L**	2	0.29	**S:R214L**	5	0.64	**S:S982A**	30	4.57	**S:L18F**	102	2.88
Maharashtra	**S:V1176F**	2	0.29	**S:G75R**	4	0.51	**S:D138Y**	28	4.27	**S:S982A**	100	2.83
Maharashtra	**S:D1260Y**	2	0.29	**S:E484K**	4	0.51	**S:S12F**	24	3.66	**S:A570D**	99	2.8
Maharashtra	**S:R21I**	2	0.29	**S:H655Y**	4	0.51	**S:A27S**	20	3.05	**S:A27S**	98	2.77
Maharashtra	**S:D848G**	2	0.29	**S:N185D**	4	0.51	**S:E484K**	14	2.13	**S:T716I**	98	2.77
Maharashtra	**S:L849P**	2	0.29	**S:E156Q**	4	0.51	**S:Q613H**	14	2.13	**S:D1118H**	95	2.69
Maharashtra	**S:F43L**	2	0.29	**S:G769V**	4	0.51	**S:S477N**	9	1.37	**S:Q675H**	93	2.63
Maharashtra	**S:I1081V**	2	0.29	**S:H1101Y**	4	0.51	**S:D950N**	9	1.37	**S:V382L**	88	2.49
Maharashtra	**S:I1179N**	2	0.29	**S:Q613H**	3	0.38	**S:T19R**	9	1.37	**S:K77T**	86	2.43
Maharashtra	**S:D1163H**	2	0.29	**S:S943T**	3	0.38	**S:V382L**	8	1.22	**S:D1153Y**	85	2.4
Maharashtra	**S:D808N**	2	0.29	**S:S254F**	3	0.38	**S:D1153Y**	8	1.22	**S:N440K**	77	2.18
Maharashtra	**S:M1237I**	2	0.29	**S:W64L**	3	0.38	**S:A222V**	8	1.22	**S:Q218H**	66	1.87
Maharashtra	**S:S151I**	2	0.29	**S:D215H**	3	0.38	**S:A1078S**	7	1.07	**S:D138Y**	38	1.07
Maharashtra	**S:A623S**	2	0.29	**S:P26L**	3	0.38	**S:W258R**	7	1.07	**S:Q1113K**	27	0.76
Maharashtra	**S:R408I**	1	0.15	**S:G184A**	3	0.38	**S:F157S**	7	1.07	**S:C851F**	26	0.73
Maharashtra	**S:A930V**	1	0.15	**S:F186S**	3	0.38	**S:V1040F**	7	1.07	**S:V1264L**	26	0.73
Maharashtra	**S:A694V**	1	0.15	**S:V915I**	3	0.38	**S:S98F**	5	0.76	**S:V159I**	25	0.71
Maharashtra	**S:I1225F**	1	0.15	**S:A1078S**	3	0.38	**S:R78M**	5	0.76	**S:T761F**	25	0.71
Maharashtra	**S:S46L**	1	0.15	**S:R190K**	3	0.38	**S:R158G**	5	0.76	**S:Q762N**	25	0.71
Maharashtra	**S:V47I**	1	0.15	**S:F797C**	3	0.38	**S:Q23R**	4	0.61	**S:L763W**	25	0.71
Maharashtra	**S:P812S**	1	0.15	**S:L54F**	3	0.38	**S:Q218H**	4	0.61	**S:R765S**	25	0.71
Maharashtra	**S:S50L**	1	0.15	**S:E484Q**	3	0.38	**S:T385I**	4	0.61	**S:A766C**	25	0.71
Maharashtra	**S:F1220L**	1	0.15	**S:L452R**	3	0.38	**S:A879S**	4	0.61	**S:L767***	25	0.71
Maharashtra	**S:G476S**	1	0.15	**S:S813I**	3	0.38	**S:E1072K**	4	0.61	**S:A845V**	25	0.71
Maharashtra	**S:T553I**	1	0.15	**S:D80Y**	2	0.26	**S:H49Y**	4	0.61	**S:I850C**	25	0.71
Maharashtra	**S:N211S**	1	0.15	**S:Q1208H**	2	0.26	**S:V687L**	4	0.61	**S:A944R**	25	0.71
Maharashtra	**S:S221L**	1	0.15	**S:A27S**	2	0.26	**S:D80Y**	4	0.61	**S:T531N**	25	0.71
Maharashtra	**S:R1014K**	1	0.15	**S:Q52H**	2	0.26	**S:Y28H**	4	0.61	**S:N532C**	25	0.71
Maharashtra	**S:T1009I**	1	0.15	**S:K1266R**	2	0.26	**S:A701V**	3	0.46	**S:V534W**	25	0.71
Maharashtra	**S:E583D**	1	0.15	**S:A892S**	2	0.26	**S:N1187H**	3	0.46	**S:K535T**	25	0.71
Maharashtra	**S:Y144H**	1	0.15	**S:V308L**	2	0.26	**S:T478K**	3	0.46	**S:N536***	25	0.71
Maharashtra	**S:R214H**	1	0.15	**S:T719S**	2	0.26	**S:L5F**	3	0.46	**S:C538V**	25	0.71
Maharashtra	**S:A783S**	1	0.15	**S:G72R**	2	0.26	**S:Y145H**	3	0.46	**S:V539Y**	25	0.71
Maharashtra	**S:E471D**	1	0.15	**S:Q23R**	2	0.26	**S:V1228L**	3	0.46	**S:N540***	25	0.71
Maharashtra	**S:S929I**	1	0.15	**S:M153I**	2	0.26	**S:V1122L**	3	0.46	**S:N542W**	25	0.71
Maharashtra	**S:Q1071E**	1	0.15	**S:P812S**	2	0.26	**S:S254F**	3	0.46	**S:A846***	24	0.68
Maharashtra	**S:L54F**	1	0.15	**S:V227A**	2	0.26	**S:E1202Q**	3	0.46	**S:D848P**	24	0.68
Maharashtra	**S:D574Y**	1	0.15	**S:G799S**	2	0.26	**S:I909V**	3	0.46	**S:L849Y**	24	0.68
Maharashtra	**S:L244F**	1	0.15	**S:R346K**	2	0.26	**S:M153I**	3	0.46	**S:Q409F**	24	0.68
Maharashtra	**S:Q1054H**	1	0.15	**S:V6F**	2	0.26	**S:V3G**	3	0.46	**S:E484S**	24	0.68
Maharashtra	**S:S1055L**	1	0.15	**S:E1262D**	2	0.26	**S:A262S**	3	0.46	**S:T573***	24	0.68
Maharashtra	**S:V62G**	1	0.15	**S:A419S**	2	0.26	**S:V83F**	3	0.46	**S:E918T**	23	0.65
Maharashtra	**S:T638S**	1	0.15	**S:P384L**	2	0.26	**S:N679K**	2	0.3	**S:I233S**	23	0.65
Maharashtra	**S:D215Y**	1	0.15	**S:T286I**	2	0.26	**S:N450K**	2	0.3	**S:G431***	23	0.65
Maharashtra	**S:G502R**	1	0.15	**S:S477I**	2	0.26	**S:D1084G**	2	0.3	**S:V433***	23	0.65
Maharashtra	**S:V503L**	1	0.15	**S:A845S**	2	0.26	**S:V70A**	2	0.3	**S:K733S**	23	0.65
Maharashtra	**S:T20I**	1	0.15	**S:A684V**	2	0.26	**S:E780Q**	2	0.3	**S:E1072K**	23	0.65
Maharashtra	**S:D111N**	1	0.15	**S:P209S**	2	0.26	**S:K1245Y**	2	0.3	**S:S12F**	23	0.65
Maharashtra	**S:G75V**	1	0.15	**S:S12F**	2	0.26	**S:D80N**	2	0.3	**S:I235F**	22	0.62
Maharashtra	**S:A522S**	1	0.15	**S:S640F**	2	0.26	**S:N81Y**	2	0.3	**S:R237C**	22	0.62
Maharashtra	**S:L176F**	1	0.15	**S:F220V**	2	0.26	**S:D80A**	2	0.3	**S:Q239T**	22	0.62
Maharashtra	**S:P384S**	1	0.15	**S:V367I**	2	0.26	**S:D215G**	2	0.3	**S:T240***	22	0.62
Maharashtra	**S:L18F**	1	0.15	**S:T299I**	2	0.26	**S:K417N**	2	0.3	**S:L241K**	22	0.62
Maharashtra	**S:A27S**	1	0.15	**S:V83F**	1	0.13	**S:V622F**	2	0.3	**S:G252F**	22	0.62
Maharashtra	**S:K558N**	1	0.15	**S:W258R**	1	0.13	**S:S943T**	2	0.3	**S:Y279W**	22	0.62
Maharashtra	**S:F797C**	1	0.15	**S:L303F**	1	0.13	**S:V1230L**	2	0.3	**S:Q690***	22	0.62
Maharashtra	**S:M731R**	1	0.15	**S:R357I**	1	0.13	**S:G639S**	2	0.3	**S:M697F**	22	0.62
Maharashtra	**S:D1041G**	1	0.15	**S:I358T**	1	0.13	**S:T114L**	2	0.3	**S:D737F**	22	0.62
Maharashtra	**S:G1046S**	1	0.15	**S:C361S**	1	0.13	**S:V159I**	2	0.3	**S:C738N**	22	0.62
Maharashtra	**S:P82L**	1	0.15	**S:A363I**	1	0.13	**S:Y160I**	2	0.3	**S:T739***	22	0.62
Maharashtra	**S:E484Q**	1	0.15	**S:L387A**	1	0.13	**S:S161A**	2	0.3	**S:Y756Q**	22	0.62
Maharashtra	**S:A93V**	1	0.15	**S:C391R**	1	0.13	**S:S162L**	2	0.3	**S:G757Q**	22	0.62

Maharashtra	**S:G404K**	1	0.15	**S:F392S**	1	0.13	**S:A163L**	2	0.3	**S:A684***	21	0.59
Maharashtra	**S:D405C**	1	0.15	**S:T393F**	1	0.13	**S:D178S**	2	0.3	**S:H1101Y**	20	0.57
Maharashtra	**S:G1044***	1	0.15	**S:V395Y**	1	0.13	**S:E180S**	2	0.3	**S:Q183T**	20	0.57
Maharashtra	**S:V407R**	1	0.15	**S:Y396S**	1	0.13	**S:G181L**	2	0.3	**S:N185***	20	0.57
Maharashtra	**S:W258***	1	0.15	**S:A397I**	1	0.13	**S:K182F**	2	0.3	**S:Y28H**	19	0.54
Maharashtra	**S:Y145V**	1	0.15	**S:V407L**	1	0.13	**S:Q183L**	2	0.3	**S:G181R**	19	0.54
Maharashtra	**S:H146G**	1	0.15	**S:R408K**	1	0.13	**S:G184W**	2	0.3	**S:G219***	19	0.54
Maharashtra	**S:K147E**	1	0.15	**S:Q409S**	1	0.13	**S:N185T**	2	0.3	**S:D198L**	18	0.51
Maharashtra	**S:N148G**	1	0.15	**S:K1181N**	1	0.13	**S:F186L**	2	0.3	**S:G639Y**	18	0.51
Maharashtra	**S:N149G**	1	0.15	**S:A262S**	1	0.13	**S:N188E**	2	0.3	**S:E180***	18	0.51
Maharashtra	**S:K150G**	1	0.15	**S:A647S**	1	0.13	**S:L189N**	2	0.3	**S:K195***	18	0.51
Maharashtra	**S:S151N**	1	0.15	**S:G219S**	1	0.13	**S:F194I**	2	0.3	**S:L223***	18	0.51
Maharashtra	**S:L216I**	1	0.15	**S:T1077G**	1	0.13	**S:K195S**	2	0.3	**S:E224S**	18	0.51
Maharashtra	**S:S637T**	1	0.15	**S:P1079A**	1	0.13	**S:N196K**	2	0.3	**S:I664Q**	17	0.48
Maharashtra	**S:Q1005H**	1	0.15	**S:N1125D**	1	0.13	**S:D198L**	2	0.3	**S:D80Y**	17	0.48
Maharashtra	**S:L938***	1	0.15	**S:S691F**	1	0.13	**S:Y200L**	2	0.3	**S:S943P**	17	0.48
Maharashtra	**S:S939P**	1	0.15	**S:P809S**	1	0.13	**S:F201K**	2	0.3	**S:G946W**	17	0.48
Maharashtra	**S:S940P**	1	0.15	**S:Q1201H**	1	0.13	**S:K202Y**	2	0.3	**S:L948T**	17	0.48
Maharashtra	**S:T941L**	1	0.15	**S:G769R**	1	0.13	**S:Y204L**	2	0.3	**S:Q949S**	17	0.48
Maharashtra	**S:A942S**	1	0.15	**S:Q183R**	1	0.13	**S:K206T**	2	0.3	**S:N188S**	17	0.48
Maharashtra	**S:D215V**	1	0.15	**S:K77R**	1	0.13	**S:H207R**	2	0.3	**S:L189***	17	0.48
Maharashtra	**S:L216C**	1	0.15	**S:D867A**	1	0.13	**S:T208L**	2	0.3	**S:R190G**	17	0.48
Maharashtra	**S:L7I**	1	0.15	**S:M1237I**	1	0.13	**S:P209L**	2	0.3	**S:E191I**	17	0.48
Maharashtra	**S:R634S**	1	0.15	**S:W64C**	1	0.13	**S:N211***	2	0.3	**S:F192C**	17	0.48
Maharashtra	**S:D574H**	1	0.15	**S:T76I**	1	0.13	**S:L212C**	2	0.3	**S:N188E**	16	0.45
Maharashtra	**S:E654A**	1	0.15	**S:R158S**	1	0.13	**S:R214I**	2	0.3	**S:L189N**	16	0.45
Maharashtra	**S:A846S**	1	0.15	**S:N1074D**	1	0.13	**S:D215S**	2	0.3	**S:F194I**	16	0.45
Maharashtra	**S:G75R**	1	0.15	**S:G946V**	1	0.13	**S:L226R**	2	0.3	**S:K195S**	16	0.45
Maharashtra	**S:T22A**	1	0.15	**S:T1273I**	1	0.13	**S:D228V**	2	0.3	**S:N196K**	16	0.45
Maharashtra	**S:S939F**	1	0.15	**S:V1122L**	1	0.13	**S:L241S**	2	0.3	**S:Y266Q**	16	0.45
Maharashtra	**S:T1117I**	1	0.15	**S:S247I**	1	0.13	**S:A243***	2	0.3	**S:Y269Q**	16	0.45
Maharashtra	**S:D290Y**	1	0.15	**S:N540S**	1	0.13	**S:H245L**	2	0.3	**S:P897C**	16	0.45
Maharashtra	**S:P9Q**	1	0.15	**S:A263V**	1	0.13	**S:R246V**	2	0.3	**S:F898Y**	16	0.45
Maharashtra	**S:V308E**	1	0.15	**S:A1016T**	1	0.13	**S:S247I**	2	0.3	**S:I909L**	16	0.45
Maharashtra	**S:Y91K**	1	0.15	**S:T572N**	1	0.13	**S:Y248L**	2	0.3	**S:G910***	16	0.45
Maharashtra	**S:G885A**	1	0.15	**S:L517H**	1	0.13	**S:Y266Q**	2	0.3	**S:A845S**	16	0.45
Maharashtra	**S:G1099V**	1	0.15	**S:T747I**	1	0.13	**S:Y269Q**	2	0.3	**S:Y423W**	16	0.45
Maharashtra	**S:S162I**	1	0.15	**S:D80N**	1	0.13	**S:C291M**	2	0.3	**S:L492T**	16	0.45
Maharashtra	**S:S929T**	1	0.15	**S:S112L**	1	0.13	**S:A292K**	2	0.3	**S:Q493H**	16	0.45
Maharashtra	**S:S373L**	1	0.15	**S:P812L**	1	0.13	**S:L293M**	2	0.3	**S:S494***	16	0.45
Maharashtra	**S:M1029I**	1	0.15	**S:Y789N**	1	0.13	**S:D294E**	2	0.3	**S:Y495W**	16	0.45
Maharashtra	**S:A701S**	1	0.15	**S:D88Y**	1	0.13	**S:I664Q**	2	0.3	**S:G496C**	16	0.45
Maharashtra	**S:D936H**	1	0.15	**S:V433N**	1	0.13	**S:P26S**	2	0.3	**S:F497W**	16	0.45
Maharashtra	**S:I235T**	1	0.15	**S:K811M**	1	0.13	**S:E583Q**	2	0.3	**S:Q498L**	16	0.45
Maharashtra	**S:D80Y**	1	0.15	**S:S816P**	1	0.13	**S:P330S**	2	0.3	**S:V512I**	16	0.45
Maharashtra	**S:T678I**	1	0.15	**S:N960S**	1	0.13	**S:P812L**	2	0.3	**S:Y200L**	15	0.42
Maharashtra	**S:D1199Y**	1	0.15	**S:T961L**	1	0.13	**S:W152L**	2	0.3	**S:F201K**	15	0.42
Maharashtra	**S:A672V**	1	0.15	**S:L962D**	1	0.13	**S:M153L**	2	0.3	**S:K202Y**	15	0.42
Maharashtra	**S:V159I**	1	0.15	**S:V963L**	1	0.13	**S:S813I**	2	0.3	**S:Y204L**	15	0.42
Maharashtra	**S:Y160I**	1	0.15	**S:D936Y**	1	0.13	**S:T29I**	2	0.3	**S:K206T**	15	0.42
Maharashtra	**S:S161A**	1	0.15	**S:T22I**	1	0.13	**S:S939F**	2	0.3	**S:H207R**	15	0.42
Maharashtra	**S:S162L**	1	0.15	**S:A67V**	1	0.13	**S:T827H**	2	0.3	**S:T208L**	15	0.42
Maharashtra	**S:A163L**	1	0.15	**S:A701V**	1	0.13	**S:C851T**	2	0.3	**S:P209L**	15	0.42
Maharashtra	**S:D178S**	1	0.15	**S:V1228L**	1	0.13	**S:A876S**	2	0.3	**S:N211***	15	0.42
Maharashtra	**S:E180S**	1	0.15	**S:G75D**	1	0.13	**S:A879V**	2	0.3	**S:L212C**	15	0.42
Maharashtra	**S:G181L**	1	0.15	**S:A1070V**	1	0.13	**S:V911R**	2	0.3	**S:L241S**	15	0.42
Maharashtra	**S:K182F**	1	0.15	**S:D578Y**	1	0.13	**S:Q913K**	2	0.3	**S:I332Q**	15	0.42
Maharashtra	**S:Q183L**	1	0.15	**S:K1191N**	1	0.13	**S:V915***	2	0.3	**S:T333Q**	15	0.42
Maharashtra	**S:G184W**	1	0.15	**S:M153T**	1	0.13	**S:Y917P**	2	0.3	**S:T385L**	15	0.42
Maharashtra	**S:N185T**	1	0.15	**S:A222S**	1	0.13	**S:E918T**	2	0.3	**S:K386L**	15	0.42
Maharashtra	**S:F186L**	1	0.15	**S:T573I**	1	0.13	**S:S937V**	2	0.3	**S:E868C**	15	0.42
Maharashtra	**S:N188E**	1	0.15	**S:A263S**	1	0.13	**S:S939L**	2	0.3	**S:M869S**	15	0.42
Maharashtra	**S:L189N**	1	0.15	**S:A522S**	1	0.13	**S:S940A**	2	0.3	**S:A871H**	15	0.42
Maharashtra	**S:F194I**	1	0.15	**S:L1224F**	1	0.13	**S:T941K**	2	0.3	**S:Q872F**	15	0.42
Maharashtra	**S:K195S**	1	0.15	**S:R78S**	1	0.13	**S:A942F**	2	0.3	**S:Y873C**	15	0.42
Maharashtra	**S:N196K**	1	0.15	**S:F32I**	1	0.13	**S:S943K**	2	0.3	**S:T883V**	15	0.42
Maharashtra	**S:D198L**	1	0.15	**S:T51F**	1	0.13	**S:A944T**	2	0.3	**S:W886N**	15	0.42
Maharashtra	**S:Y200L**	1	0.15	**S:D53I**	1	0.13	**S:L945H**	2	0.3	**S:T912***	15	0.42
Maharashtra	**S:F201K**	1	0.15	**S:F55S**	1	0.13	**S:G946F**	2	0.3	**S:Q913W**	15	0.42
Maharashtra	**S:K202Y**	1	0.15	**S:L56F**	1	0.13	**S:K947L**	2	0.3	**S:N914Y**	15	0.42
Maharashtra	**S:Y204L**	1	0.15	**S:P57T**	1	0.13	**S:L948P**	2	0.3	**S:V915W**	15	0.42
Maharashtra	**S:K206T**	1	0.15	**S:F59N**	1	0.13	**S:D950Q**	2	0.3	**S:L916S**	15	0.42
Maharashtra	**S:H207R**	1	0.15	**S:K77C**	1	0.13	**S:V952H**	2	0.3	**S:N919E**	15	0.42

Maharashtra	**S:T208L**	1	0.15	**S:R78L**	1	0.13	**S:N953L**	2	0.3	**S:Q920C**	15	0.42
Maharashtra	**S:P209L**	1	0.15	**S:F79W**	1	0.13	**S:Q954E**	2	0.3	**S:K921S**	15	0.42
Maharashtra	**S:N211***	1	0.15	**S:N81W**	1	0.13	**S:A956T**	2	0.3	**S:I923W**	15	0.42
Maharashtra	**S:L212C**	1	0.15	**S:P82C**	1	0.13	**S:P1162L**	2	0.3	**S:A924Q**	15	0.42
Maharashtra	**S:R214I**	1	0.15	**S:V83L**	1	0.13	**S:M177I**	2	0.3	**S:Q613H**	15	0.42
Maharashtra	**S:D215S**	1	0.15	**S:L84F**	1	0.13	**S:E406K**	2	0.3	**S:L5F**	15	0.42
Maharashtra	**S:D228R**	1	0.15	**S:P85C**	1	0.13	**S:L176F**	2	0.3	**S:R328P**	15	0.42
Maharashtra	**S:L229V**	1	0.15	**S:N87H**	1	0.13	**S:F79S**	2	0.3	**S:F347W**	15	0.42
Maharashtra	**S:P230F**	1	0.15	**S:D88***	1	0.13	**S:T307I**	2	0.3	**S:A348***	15	0.42
Maharashtra	**S:I231R**	1	0.15	**S:G89E**	1	0.13	**S:D1260Y**	2	0.3	**S:V367Q**	15	0.42
Maharashtra	**S:G232L**	1	0.15	**S:I105***	1	0.13	**S:L517H**	2	0.3	**S:F456E**	15	0.42
Maharashtra	**S:I233***	1	0.15	**S:F106H**	1	0.13	**S:S698L**	2	0.3	**S:K458Y**	15	0.42
Maharashtra	**S:T236E**	1	0.15	**S:G107N**	1	0.13	**S:D215Y**	2	0.3	**S:S459F**	15	0.42
Maharashtra	**S:R237V**	1	0.15	**S:T108K**	1	0.13	**S:L1063F**	2	0.3	**S:Y489W**	15	0.42
Maharashtra	**S:F238I**	1	0.15	**S:T109R**	1	0.13	**S:V1060L**	2	0.3	**S:D198H**	15	0.42
Maharashtra	**S:Q239***	1	0.15	**S:S112V**	1	0.13	**S:L948F**	2	0.3	**S:G199A**	15	0.42
Maharashtra	**S:T240L**	1	0.15	**S:K113P**	1	0.13	**S:W258L**	2	0.3	**S:G1219V**	15	0.42
Maharashtra	**S:L242V**	1	0.15	**S:K129L**	1	0.13	**S:N1192H**	2	0.3	**S:Y160I**	14	0.4
Maharashtra	**S:A243I**	1	0.15	**S:V130***	1	0.13	**S:S659L**	2	0.3	**S:S161A**	14	0.4
Maharashtra	**S:Y265L**	1	0.15	**S:C131***	1	0.13	**S:R214H**	2	0.3	**S:S162L**	14	0.4
Maharashtra	**S:Y266Q**	1	0.15	**S:E132S**	1	0.13	**S:G75S**	1	0.15	**S:A163L**	14	0.4
Maharashtra	**S:Y269Q**	1	0.15	**S:F133I**	1	0.13	**S:C1236F**	1	0.15	**S:D178S**	14	0.4
Maharashtra	**S:V289K**	1	0.15	**S:Q134F**	1	0.13	**S:S477T**	1	0.15	**S:E180S**	14	0.4
Maharashtra	**S:D290M**	1	0.15	**S:F135G**	1	0.13	**S:I68T**	1	0.15	**S:G181L**	14	0.4
Maharashtra	**S:C291E**	1	0.15	**S:N137L**	1	0.13	**S:P85T**	1	0.15	**S:K182F**	14	0.4
Maharashtra	**S:A292P**	1	0.15	**S:D138L**	1	0.13	**S:F86I**	1	0.15	**S:Q183L**	14	0.4
Maharashtra	**S:D294Q**	1	0.15	**S:H146Q**	1	0.13	**S:Y170L**	1	0.15	**S:G184W**	14	0.4
Maharashtra	**S:P295M**	1	0.15	**S:N148Q**	1	0.13	**S:V171N**	1	0.15	**S:N185T**	14	0.4
Maharashtra	**S:S297***	1	0.15	**S:N149Q**	1	0.13	**S:S172A**	1	0.15	**S:F186L**	14	0.4
Maharashtra	**S:E298T**	1	0.15	**S:T167L**	1	0.13	**S:L922F**	1	0.15	**S:R214I**	14	0.4
Maharashtra	**S:T299V**	1	0.15	**S:E169***	1	0.13	**S:I923G**	1	0.15	**S:D215S**	14	0.4
Maharashtra	**S:K300H**	1	0.15	**S:Y170C**	1	0.13	**S:Q926K**	1	0.15	**S:Y365A**	14	0.4
Maharashtra	**S:C301L**	1	0.15	**S:V171E**	1	0.13	**S:F927L**	1	0.15	**S:S366T**	14	0.4
Maharashtra	**S:P330S**	1	0.15	**S:S172***	1	0.13	**S:G1223D**	1	0.15	**S:Y369L**	14	0.4
Maharashtra	**S:I332Q**	1	0.15	**S:Q173L**	1	0.13	**S:V6F**	1	0.15	**S:A419I**	14	0.4
Maharashtra	**S:T333Q**	1	0.15	**S:P174H**	1	0.13	**S:T95N**	1	0.15	**S:G447I**	14	0.4
Maharashtra	**S:R346G**	1	0.15	**S:L176***	1	0.13	**S:I818D**	1	0.15	**S:Q784R**	14	0.4
Maharashtra	**S:F347R**	1	0.15	**S:M177I**	1	0.13	**S:H1088P**	1	0.15	**S:V785Q**	14	0.4
Maharashtra	**S:A348E**	1	0.15	**S:D178C**	1	0.13	**S:K1045N**	1	0.15	**S:G798W**	14	0.4
Maharashtra	**S:Y365A**	1	0.15	**S:E180***	1	0.13	**S:F133C**	1	0.15	**S:G799W**	14	0.4
Maharashtra	**S:S366T**	1	0.15	**S:G181R**	1	0.13	**S:F135S**	1	0.15	**S:N801***	14	0.4
Maharashtra	**S:Y369L**	1	0.15	**S:Q183T**	1	0.13	**S:D138C**	1	0.15	**S:S803K**	14	0.4
Maharashtra	**S:N370I**	1	0.15	**S:N185***	1	0.13	**S:P139N**	1	0.15	**S:K825E**	14	0.4
Maharashtra	**S:S371I**	1	0.15	**S:N188S**	1	0.13	**S:F140H**	1	0.15	**S:R319L**	14	0.4
Maharashtra	**S:A372L**	1	0.15	**S:L189***	1	0.13	**S:L141D**	1	0.15	**S:V320***	14	0.4
Maharashtra	**S:F374L**	1	0.15	**S:R190G**	1	0.13	**S:F565I**	1	0.15	**S:Q321S**	14	0.4
Maharashtra	**S:T376V**	1	0.15	**S:E191I**	1	0.13	**S:D578Y**	1	0.15	**S:E324N**	14	0.4
Maharashtra	**S:F377M**	1	0.15	**S:F192C**	1	0.13	**S:P812S**	1	0.15	**S:S325R**	14	0.4
Maharashtra	**S:K378E**	1	0.15	**S:K195***	1	0.13	**S:C1250F**	1	0.15	**S:Y369***	14	0.4
Maharashtra	**S:T385L**	1	0.15	**S:V213F**	1	0.13	**S:I105V**	1	0.15	**S:N370V**	14	0.4
Maharashtra	**S:K386L**	1	0.15	**S:R214***	1	0.13	**S:P561S**	1	0.15	**S:S371L**	14	0.4
Maharashtra	**S:D389H**	1	0.15	**S:D215A**	1	0.13	**S:T734I**	1	0.15	**S:A372W**	14	0.4
Maharashtra	**S:G404***	1	0.15	**S:L216H**	1	0.13	**S:E1182Q**	1	0.15	**S:F374V**	14	0.4
Maharashtra	**S:D405L**	1	0.15	**S:P217A**	1	0.13	**S:Y145D**	1	0.15	**S:D428C**	14	0.4
Maharashtra	**S:R408M**	1	0.15	**S:Q218Y**	1	0.13	**S:Q14K**	1	0.15	**S:F429***	14	0.4
Maharashtra	**S:Q409K**	1	0.15	**S:G219***	1	0.13	**S:I105L**	1	0.15	**S:T430L**	14	0.4
Maharashtra	**S:I410S**	1	0.15	**S:L223***	1	0.13	**S:F106R**	1	0.15	**S:C432L**	14	0.4
Maharashtra	**S:A411D**	1	0.15	**S:E224S**	1	0.13	**S:G107S**	1	0.15	**S:A435G**	14	0.4
Maharashtra	**S:P412K**	1	0.15	**S:L226F**	1	0.13	**S:T108L**	1	0.15	**S:N487I**	14	0.4
Maharashtra	**S:G413S**	1	0.15	**S:V227G**	1	0.13	**S:D111L**	1	0.15	**S:L513Q**	14	0.4
Maharashtra	**S:Q414L**	1	0.15	**S:D228F**	1	0.13	**S:S112I**	1	0.15	**S:V595S**	14	0.4
Maharashtra	**S:T415Q**	1	0.15	**S:L229R**	1	0.13	**S:K113T**	1	0.15	**S:S596***	14	0.4
Maharashtra	**S:A419I**	1	0.15	**S:P230T**	1	0.13	**S:Q115L**	1	0.15	**S:V597H**	14	0.4
Maharashtra	**S:D420I**	1	0.15	**S:I233S**	1	0.13	**S:S116M**	1	0.15	**S:I598Y**	14	0.4
Maharashtra	**S:Y421I**	1	0.15	**S:I235F**	1	0.13	**S:I128L**	1	0.15	**S:A653V**	14	0.4
Maharashtra	**S:K424***	1	0.15	**S:R237C**	1	0.13	**S:V130S**	1	0.15	**S:E654***	14	0.4
Maharashtra	**S:G447I**	1	0.15	**S:Q239T**	1	0.13	**S:C131V**	1	0.15	**S:A688S**	14	0.4
Maharashtra	**S:N448L**	1	0.15	**S:T240***	1	0.13	**S:E132N**	1	0.15	**S:Q52R**	14	0.4
Maharashtra	**S:Y449T**	1	0.15	**S:L241K**	1	0.13	**S:Q134N**	1	0.15	**S:F888L**	14	0.4
Maharashtra	**S:N450I**	1	0.15	**S:G252F**	1	0.13	**S:C136V**	1	0.15	**S:A243***	13	0.37
Maharashtra	**S:Y451L**	1	0.15	**S:R273L**	1	0.13	**S:N137M**	1	0.15	**S:H245L**	13	0.37
Maharashtra	**S:L452I**	1	0.15	**S:T274L**	1	0.13	**S:D138I**	1	0.15	**S:R246V**	13	0.37
Maharashtra	**S:Y453L**	1	0.15	**S:F275C**	1	0.13	**S:P139H**	1	0.15	**S:S247I**	13	0.37

Maharashtra	**S:F456S**	1	0.15	**S:L276G**	1	0.13	**S:M153V**	1	0.15	**S:Y248L**	13	0.37
Maharashtra	**S:R457L**	1	0.15	**S:Y279W**	1	0.13	**S:E154S**	1	0.15	**S:F347R**	13	0.37
Maharashtra	**S:K458I**	1	0.15	**S:A288H**	1	0.13	**S:S297H**	1	0.15	**S:N370I**	13	0.37
Maharashtra	**S:S469L**	1	0.15	**S:V289Y**	1	0.13	**S:E298C**	1	0.15	**S:S371I**	13	0.37
Maharashtra	**S:T470R**	1	0.15	**S:D290R**	1	0.13	**S:T299R**	1	0.15	**S:A372L**	13	0.37
Maharashtra	**S:Y473F**	1	0.15	**S:T716I**	1	0.13	**S:C301R**	1	0.15	**S:F374L**	13	0.37
Maharashtra	**S:F490N**	1	0.15	**S:F2L**	1	0.13	**S:T302N**	1	0.15	**S:T376V**	13	0.37
Maharashtra	**S:V511M**	1	0.15	**S:I834N**	1	0.13	**S:R319L**	1	0.15	**S:F377M**	13	0.37
Maharashtra	**S:S514L**	1	0.15	**S:E780Q**	1	0.13	**S:V320***	1	0.15	**S:V3G**	13	0.37
Maharashtra	**S:E516V**	1	0.15	**S:R21I**	1	0.13	**S:Q321S**	1	0.15	**S:S943T**	13	0.37
Maharashtra	**S:L517D**	1	0.15	**S:I1183L**	1	0.13	**S:E324N**	1	0.15	**S:D287H**	13	0.37
Maharashtra	**S:G550L**	1	0.15	**S:S816T**	1	0.13	**S:S325R**	1	0.15	**S:A288Y**	13	0.37
Maharashtra	**S:V551S**	1	0.15	**S:V952I**	1	0.13	**S:R328P**	1	0.15	**S:V289R**	13	0.37
Maharashtra	**S:E554K**	1	0.15	**S:T33I**	1	0.13	**S:F347W**	1	0.15	**S:D290C**	13	0.37
Maharashtra	**S:I569S**	1	0.15	**S:F1052L**	1	0.13	**S:A348***	1	0.15	**S:A292R**	13	0.37
Maharashtra	**S:T573***	1	0.15	**S:D1259Y**	1	0.13	**S:V367Q**	1	0.15	**S:Y453W**	13	0.37
Maharashtra	**S:V597Y**	1	0.15	**S:I1179V**	1	0.13	**S:Y369***	1	0.15	**S:R454***	13	0.37
Maharashtra	**S:I598F**	1	0.15	**S:V1264L**	1	0.13	**S:N370V**	1	0.15	**S:N481***	13	0.37
Maharashtra	**S:T599***	1	0.15	**S:T307I**	1	0.13	**S:S371L**	1	0.15	**S:G482N**	13	0.37
Maharashtra	**S:T638Y**	1	0.15	**S:D427A**	1	0.13	**S:A372W**	1	0.15	**S:V483L**	13	0.37
Maharashtra	**S:G639L**	1	0.15	**S:K558N**	1	0.13	**S:F374V**	1	0.15	**S:F486T**	13	0.37
Maharashtra	**S:S640A**	1	0.15	**S:S256L**	1	0.13	**S:Q409F**	1	0.15	**S:C488I**	13	0.37
Maharashtra	**S:N641C**	1	0.15	**S:G75V**	1	0.13	**S:Y423W**	1	0.15	**S:V642R**	13	0.37
Maharashtra	**S:V642L**	1	0.15	**S:F490S**	1	0.13	**S:D428C**	1	0.15	**S:F643G**	13	0.37
Maharashtra	**S:T645Y**	1	0.15	**S:A846S**	1	0.13	**S:F429***	1	0.15	**S:Q644***	13	0.37
Maharashtra	**S:A647F**	1	0.15	**S:W64R**	1	0.13	**S:T430L**	1	0.15	**S:V642L**	13	0.37
Maharashtra	**S:G648***	1	0.15	**S:G1219C**	1	0.13	**S:G431***	1	0.15	**S:G652***	13	0.37
Maharashtra	**S:G652***	1	0.15	**S:T1238N**	1	0.13	**S:C432L**	1	0.15	**S:L176S**	13	0.37
Maharashtra	**S:A653V**	1	0.15	**S:S443A**	1	0.13	**S:V433***	1	0.15	**S:L226R**	12	0.34
Maharashtra	**S:E654***	1	0.15	**S:T167S**	1	0.13	**S:A435G**	1	0.15	**S:D228V**	12	0.34
Maharashtra	**S:T678W**	1	0.15	**S:V1104L**	1	0.13	**S:Y453W**	1	0.15	**S:R346G**	12	0.34
Maharashtra	**S:N679C**	1	0.15	**S:A243S**	1	0.13	**S:R454***	1	0.15	**S:A348E**	12	0.34
Maharashtra	**S:S680R**	1	0.15	**S:V341I**	1	0.13	**S:F456E**	1	0.15	**S:K378E**	12	0.34
Maharashtra	**S:P681Y**	1	0.15	**S:A520S**	1	0.13	**S:K458Y**	1	0.15	**S:R403K**	12	0.34
Maharashtra	**S:R682M**	1	0.15	**S:K1073N**	1	0.13	**S:S459F**	1	0.15	**S:D420I**	12	0.34
Maharashtra	**S:A684***	1	0.15	**S:T95I**	1	0.13	**S:N481***	1	0.15	**S:Y421I**	12	0.34
Maharashtra	**S:R685L**	1	0.15	**S:E154K**	1	0.13	**S:G482N**	1	0.15	**S:N448L**	12	0.34
Maharashtra	**S:V687D**	1	0.15	**S:A263E**	1	0.13	**S:V483L**	1	0.15	**S:Y449T**	12	0.34
Maharashtra	**S:A688S**	1	0.15	**S:P681R**	1	0.13	**S:E484S**	1	0.15	**S:N450I**	12	0.34
Maharashtra	**S:S689D**	1	0.15	**S:N1187H**	1	0.13	**S:F486T**	1	0.15	**S:Y451L**	12	0.34
Maharashtra	**S:Q690***	1	0.15	**S:S162I**	1	0.13	**S:N487I**	1	0.15	**S:L452I**	12	0.34
Maharashtra	**S:S691F**	1	0.15	**S:L216F**	1	0.13	**S:C488I**	1	0.15	**S:Y453L**	12	0.34
Maharashtra	**S:I692S**	1	0.15	**S:F392L**	1	0.13	**S:Y489W**	1	0.15	**S:S477N**	12	0.34
Maharashtra	**S:I693S**	1	0.15	**S:A123S**	1	0.13	**S:L492T**	1	0.15	**S:F275L**	12	0.34
Maharashtra	**S:Y695G**	1	0.15	**S:G639S**	1	0.13	**S:Q493H**	1	0.15	**S:L277***	12	0.34
Maharashtra	**S:M697F**	1	0.15	**S:V483F**	1	0.13	**S:S494***	1	0.15	**S:D663Q**	12	0.34
Maharashtra	**S:K733S**	1	0.15	**S:P1162L**	1	0.13	**S:Y495W**	1	0.15	**S:P665L**	12	0.34
Maharashtra	**S:I742Y**	1	0.15	**S:G1267R**	1	0.13	**S:G496C**	1	0.15	**S:I693W**	12	0.34
Maharashtra	**S:C743D**	1	0.15	**S:L945F**	1	0.13	**S:F497W**	1	0.15	**S:A694C**	12	0.34
Maharashtra	**S:G744Q**	1	0.15	**S:I794T**	1	0.13	**S:Q498L**	1	0.15	**S:Y695R**	12	0.34
Maharashtra	**S:S746I**	1	0.15	**S:L176F**	1	0.13	**S:V512I**	1	0.15	**S:T696K**	12	0.34
Maharashtra	**S:T747S**	1	0.15	**S:S1252F**	1	0.13	**S:L513Q**	1	0.15	**S:T716R**	12	0.34
Maharashtra	**S:E748R**	1	0.15	**S:A1020V**	1	0.13	**S:T531N**	1	0.15	**S:Q804N**	12	0.34
Maharashtra	**S:C749L**	1	0.15	**S:D253N**	1	0.13	**S:N532C**	1	0.15	**S:S161L**	12	0.34
Maharashtra	**S:S750Y**	1	0.15	**S:A222V**	1	0.13	**S:V534W**	1	0.15	**S:A831V**	12	0.34
Maharashtra	**S:L752V**	1	0.15	**S:A694T**	1	0.13	**S:K535T**	1	0.15	**S:S98F**	12	0.34
Maharashtra	**S:L753H**	1	0.15	**S:A879V**	1	0.13	**S:N536***	1	0.15	**S:K310S**	11	0.31
Maharashtra	**S:D848L**	1	0.15	**S:D839A**	1	0.13	**S:C538V**	1	0.15	**S:G311I**	11	0.31
Maharashtra	**S:I850F**	1	0.15	**S:P479S**	1	0.13	**S:V539Y**	1	0.15	**S:I312K**	11	0.31
Maharashtra	**S:Q872H**	1	0.15				**S:N540***	1	0.15	**S:Y313L**	11	0.31
Maharashtra	**S:Y873L**	1	0.15				**S:N542W**	1	0.15	**S:Q314L**	11	0.31
Maharashtra	**S:T874C**	1	0.15				**S:T573***	1	0.15	**S:T323L**	11	0.31
Maharashtra	**S:A876S**	1	0.15				**S:V595S**	1	0.15	**S:Y396***	11	0.31
Maharashtra	**S:V911R**	1	0.15				**S:S596***	1	0.15	**S:A397M**	11	0.31
Maharashtra	**S:Q913K**	1	0.15				**S:V597H**	1	0.15	**S:D398I**	11	0.31
Maharashtra	**S:V915***	1	0.15				**S:I598Y**	1	0.15	**S:F400E**	11	0.31
Maharashtra	**S:Y917P**	1	0.15				**S:G639Y**	1	0.15	**S:I402M**	11	0.31
Maharashtra	**S:E918T**	1	0.15				**S:V642R**	1	0.15	**S:G404S**	11	0.31
Maharashtra	**S:S937V**	1	0.15				**S:F643G**	1	0.15	**S:E406K**	11	0.31
Maharashtra	**S:S939L**	1	0.15				**S:Q644***	1	0.15	**S:V407S**	11	0.31
Maharashtra	**S:S940A**	1	0.15				**S:D663Q**	1	0.15	**S:R408L**	11	0.31
Maharashtra	**S:T941K**	1	0.15				**S:P665L**	1	0.15	**S:L959C**	11	0.31
Maharashtra	**S:A942F**	1	0.15				**S:P1263S**	1	0.15	**S:N960***	11	0.31

Maharashtra	**S:S943K**	1	0.15				**S:T430I**	1	0.15	**S:L962T**	11	0.31
Maharashtra	**S:A944T**	1	0.15				**S:K814E**	1	0.15	**S:V963***	11	0.31
Maharashtra	**S:L945H**	1	0.15				**S:V1176F**	1	0.15	**S:K964L**	11	0.31
Maharashtra	**S:G946F**	1	0.15				**S:A924D**	1	0.15	**S:L966F**	11	0.31
Maharashtra	**S:K947L**	1	0.15				**S:N925K**	1	0.15	**S:S967W**	11	0.31
Maharashtra	**S:L948P**	1	0.15				**S:Q926N**	1	0.15	**S:D1041***	11	0.31
Maharashtra	**S:D950Q**	1	0.15				**S:Q954K**	1	0.15	**S:C1043L**	11	0.31
Maharashtra	**S:V952H**	1	0.15				**S:C166S**	1	0.15	**S:G1044W**	11	0.31
Maharashtra	**S:N953L**	1	0.15				**S:T167I**	1	0.15	**S:H1048V**	11	0.31
Maharashtra	**S:Q954E**	1	0.15				**S:E169G**	1	0.15	**S:M1050P**	11	0.31
Maharashtra	**S:A956T**	1	0.15				**S:L176I**	1	0.15	**S:N1187H**	11	0.31
Maharashtra	**S:Q957R**	1	0.15				**S:M177T**	1	0.15	**S:P681D**	11	0.31
Maharashtra	**S:A958L**	1	0.15				**S:I794F**	1	0.15	**S:R682S**	11	0.31
Maharashtra	**S:T961N**	1	0.15				**S:A1070V**	1	0.15	**S:R683D**	11	0.31
Maharashtra	**S:V963A**	1	0.15				**S:L84I**	1	0.15	**S:R685F**	11	0.31
Maharashtra	**S:K964P**	1	0.15				**S:A783E**	1	0.15	**S:V687S**	11	0.31
Maharashtra	**S:Q965I**	1	0.15				**S:Q784K**	1	0.15	**S:N501H**	11	0.31
Maharashtra	**S:S967F**	1	0.15				**S:V785K**	1	0.15	**S:G502***	11	0.31
Maharashtra	**S:S968Q**	1	0.15				**S:Q787K**	1	0.15	**S:V503W**	11	0.31
Maharashtra	**S:N969V**	1	0.15				**S:S816L**	1	0.15	**S:G504C**	11	0.31
Maharashtra	**S:G971***	1	0.15				**S:A243S**	1	0.15	**S:Y505W**	11	0.31
Maharashtra	**S:A972M**	1	0.15				**S:G946L**	1	0.15	**S:Q506L**	11	0.31
Maharashtra	**S:S975F**	1	0.15				**S:A924V**	1	0.15	**S:R567K**	11	0.31
Maharashtra	**S:V976H**	1	0.15				**S:S494P**	1	0.15	**S:I569S**	11	0.31
Maharashtra	**S:L977V**	1	0.15				**S:F106I**	1	0.15	**S:Q774R**	11	0.31
Maharashtra	**S:N978L**	1	0.15				**S:G107R**	1	0.15	**S:D775Q**	11	0.31
Maharashtra	**S:D979T**	1	0.15				**S:T109R**	1	0.15	**S:S803T**	11	0.31
Maharashtra	**S:I980K**	1	0.15				**S:L110I**	1	0.15	**S:K814T**	11	0.31
Maharashtra	**S:S982R**	1	0.15				**S:D111N**	1	0.15	**S:K835Q**	11	0.31
Maharashtra	**S:R983L**	1	0.15				**S:K113E**	1	0.15	**S:Y837S**	11	0.31
Maharashtra	**S:L984K**	1	0.15				**S:Q115T**	1	0.15	**S:G838D**	11	0.31
Maharashtra	**S:D985C**	1	0.15				**S:S116F**	1	0.15	**S:D839T**	11	0.31
Maharashtra	**S:V987L**	1	0.15				**S:L117H**	1	0.15	**S:R158***	11	0.31
Maharashtra	**S:E988I**	1	0.15				**S:L118P**	1	0.15	**S:Y160C**	11	0.31
Maharashtra	**S:A989G**	1	0.15				**S:I119T**	1	0.15	**S:M177Y**	11	0.31
Maharashtra	**S:E990***	1	0.15				**S:D1163Y**	1	0.15	**S:D178G**	11	0.31
Maharashtra	**S:V991S**	1	0.15				**S:H519P**	1	0.15	**S:L179P**	11	0.31
Maharashtra	**S:I993A**	1	0.15				**S:H146Y**	1	0.15	**S:S297H**	11	0.31
Maharashtra	**S:R1014L**	1	0.15				**S:G769V**	1	0.15	**S:E298C**	11	0.31
Maharashtra	**S:A1015L**	1	0.15				**S:A706S**	1	0.15	**S:T299R**	11	0.31
Maharashtra	**S:A1016L**	1	0.15				**S:V159F**	1	0.15	**S:C301R**	11	0.31
Maharashtra	**S:E1017L**	1	0.15				**S:N679S**	1	0.15	**S:T302N**	11	0.31
Maharashtra	**S:I1018K**	1	0.15				**S:S708F**	1	0.15	**S:I332P**	11	0.31
Maharashtra	**S:R1019C**	1	0.15				**S:Y144***	1	0.15	**S:C336V**	11	0.31
Maharashtra	**S:A1020Q**	1	0.15				**S:Y145N**	1	0.15	**S:G339W**	11	0.31
Maharashtra	**S:A1022V**	1	0.15				**S:R319G**	1	0.15	**S:E340***	11	0.31
Maharashtra	**S:N1023Y**	1	0.15				**S:N824K**	1	0.15	**S:V341S**	11	0.31
Maharashtra	**S:A1025D**	1	0.15				**S:V826***	1	0.15	**S:N354***	11	0.31
Maharashtra	**S:A1026N**	1	0.15				**S:A829V**	1	0.15	**S:K356H**	11	0.31
Maharashtra	**S:T1027Q**	1	0.15				**S:D830I**	1	0.15	**S:R357Q**	11	0.31
Maharashtra	**S:M1029E**	1	0.15				**S:G832L**	1	0.15	**S:S359C**	11	0.31
Maharashtra	**S:S1030L**	1	0.15				**S:F833V**	1	0.15	**S:N360I**	11	0.31
Maharashtra	**S:E1031I**	1	0.15				**S:R847L**	1	0.15	**S:H655Y**	11	0.31
Maharashtra	**S:C1032F**	1	0.15				**S:D848L**	1	0.15	**S:E1202Q**	11	0.31
Maharashtra	**S:S1055I**	1	0.15				**S:I850E**	1	0.15	**S:M1237I**	11	0.31
Maharashtra	**S:G1167S**	1	0.15				**S:A852S**	1	0.15	**S:T109A**	10	0.28
Maharashtra	**S:D1168Y**	1	0.15				**S:Q853F**	1	0.15	**S:C291M**	10	0.28
Maharashtra	**S:A1190K**	1	0.15				**S:K854V**	1	0.15	**S:A292K**	10	0.28
Maharashtra	**S:K1191R**	1	0.15				**S:F855H**	1	0.15	**S:L293M**	10	0.28
Maharashtra	**S:P1213R**	1	0.15				**S:N856K**	1	0.15	**S:D294E**	10	0.28
Maharashtra	**S:W1214F**	1	0.15				**S:G857S**	1	0.15	**S:F456S**	10	0.28
Maharashtra	**S:I1216S**	1	0.15				**S:V860A**	1	0.15	**S:R457L**	10	0.28
Maharashtra	**S:F1220D**	1	0.15				**S:P862K**	1	0.15	**S:K458I**	10	0.28
Maharashtra	**S:I1221N**	1	0.15				**S:P863***	1	0.15	**S:S469L**	10	0.28
Maharashtra	**S:A1222Y**	1	0.15				**S:T866N**	1	0.15	**S:T470R**	10	0.28
Maharashtra	**S:G1223A**	1	0.15				**S:D867T**	1	0.15	**S:Y473F**	10	0.28
Maharashtra	**S:K1245Q**	1	0.15				**S:E868L**	1	0.15	**S:V511M**	10	0.28
Maharashtra	**S:C1247L**	1	0.15				**S:M869L**	1	0.15	**S:S514L**	10	0.28
Maharashtra	**S:T1238I**	1	0.15				**S:I870H**	1	0.15	**S:E516V**	10	0.28
Maharashtra	**S:Y38G**	1	0.15				**S:A871C**	1	0.15	**S:L517D**	10	0.28
Maharashtra	**S:P39L**	1	0.15				**S:Q872***	1	0.15	**S:E583L**	10	0.28
Maharashtra	**S:D40T**	1	0.15				**S:Y873R**	1	0.15	**S:V1008H**	10	0.28
Maharashtra	**S:K41S**	1	0.15				**S:T874V**	1	0.15	**S:T1009R**	10	0.28

Maharashtra	S:V42Y	1	0.15				S:S875Q	1	0.15	S:E1031F	10	0.28
Maharashtra	S:R44W	1	0.15				S:Q957R	1	0.15	S:A1078S	10	0.28
Maharashtra	S:S46P	1	0.15				S:A958L	1	0.15	S:V826L	10	0.28
Maharashtra	S:V47L	1	0.15				S:T961N	1	0.15	S:K529T	10	0.28
Maharashtra	S:H49C	1	0.15				S:V963A	1	0.15	S:D568V	10	0.28
Maharashtra	S:R905S	1	0.15				S:K964P	1	0.15	S:V597Y	10	0.28
Maharashtra	S:Q218H	1	0.15				S:Q965I	1	0.15	S:I598F	10	0.28
Maharashtra	S:G769V	1	0.15				S:S967F	1	0.15	S:T599*	10	0.28
Maharashtra	S:Y91L	1	0.15				S:S968Q	1	0.15	S:T638Y	10	0.28
Maharashtra	S:F347I	1	0.15				S:N969V	1	0.15	S:G639L	10	0.28
Maharashtra	S:A348P	1	0.15				S:G971*	1	0.15	S:S640A	10	0.28
Maharashtra	S:T430N	1	0.15				S:A972M	1	0.15	S:N641C	10	0.28
Maharashtra	S:G431S	1	0.15				S:S975F	1	0.15	S:T645Y	10	0.28
Maharashtra	S:K811R	1	0.15				S:V976H	1	0.15	S:A647F	10	0.28
Maharashtra	S:F86P	1	0.15				S:L977V	1	0.15	S:G648*	10	0.28
Maharashtra	S:N87G	1	0.15				S:N978L	1	0.15	S:T678W	10	0.28
Maharashtra	S:D88N	1	0.15				S:D979T	1	0.15	S:N679C	10	0.28
Maharashtra	S:N165S	1	0.15				S:I980K	1	0.15	S:S680R	10	0.28
Maharashtra	S:S758C	1	0.15				S:S982R	1	0.15	S:P681Y	10	0.28
Maharashtra	S:A771L	1	0.15				S:R983L	1	0.15	S:R682M	10	0.28
Maharashtra	S:E773Q	1	0.15				S:L984K	1	0.15	S:R685L	10	0.28
Maharashtra	S:G261R	1	0.15				S:D985C	1	0.15	S:V687D	10	0.28
Maharashtra	S:V1133I	1	0.15				S:R1000*	1	0.15	S:S689D	10	0.28
Maharashtra	S:H655Y	1	0.15				S:L1001S	1	0.15	S:S691F	10	0.28
Maharashtra	S:L938F	1	0.15				S:S1003*	1	0.15	S:I692S	10	0.28
Maharashtra							S:I1018L	1	0.15	S:I693S	10	0.28
Maharashtra							S:R1019Q	1	0.15	S:Y695G	10	0.28
Maharashtra							S:A1020K	1	0.15	S:T778L	10	0.28
Maharashtra							S:A1022E	1	0.15	S:G842C	10	0.28
Maharashtra							S:N1023L	1	0.15	S:D843T	10	0.28
Maharashtra							S:A1025L	1	0.15	S:I844K	10	0.28
Maharashtra							S:A1026I	1	0.15	S:S875F	10	0.28
Maharashtra							S:T1027L	1	0.15	S:L878C	10	0.28
Maharashtra							S:K1028L	1	0.15	S:Q913E	10	0.28
Maharashtra							S:M1029L	1	0.15	S:N914C	10	0.28
Maharashtra							S:S1030L	1	0.15	S:V915S	10	0.28
Maharashtra							S:E1031K	1	0.15	S:Y917*	10	0.28
Maharashtra							S:K1038V	1	0.15	S:F329L	10	0.28
Maharashtra							S:R1039Y	1	0.15	S:V362I	10	0.28
Maharashtra							S:V1040L	1	0.15	S:A363F	10	0.28
Maharashtra							S:F1042C	1	0.15	S:D364H	10	0.28
Maharashtra							S:C1043P	1	0.15	S:Y365F	10	0.28
Maharashtra							S:G1044S	1	0.15	S:S366*	10	0.28
Maharashtra							S:K1045L	1	0.15	S:S459*	10	0.28
Maharashtra							S:G1046S	1	0.15	S:L226F	10	0.28
Maharashtra							S:Y1047Q	1	0.15	S:D228F	10	0.28
Maharashtra							S:D1084M	1	0.15	S:L229R	10	0.28
Maharashtra							S:G1085E	1	0.15	S:P230T	10	0.28
Maharashtra							S:A1087H	1	0.15	S:T572I	10	0.28
Maharashtra							S:H1088T	1	0.15	S:L54F	9	0.25
Maharashtra							S:G1093L	1	0.15	S:E298L	9	0.25
Maharashtra							S:F1095K	1	0.15	S:T299*	9	0.25
Maharashtra							S:N1098K	1	0.15	S:V308K	9	0.25
Maharashtra							S:G1124H	1	0.15	S:K424A	9	0.25
Maharashtra							S:N1125L	1	0.15	S:K444*	9	0.25
Maharashtra							S:D1127L	1	0.15	S:V445L	9	0.25
Maharashtra							S:V1129Q	1	0.15	S:K558S	9	0.25
Maharashtra							S:I1130F	1	0.15	S:I584M	9	0.25
Maharashtra							S:G1131M	1	0.15	S:E654K	9	0.25
Maharashtra							S:V1133L	1	0.15	S:S968C	9	0.25
Maharashtra							S:N1134C	1	0.15	S:R983K	9	0.25
Maharashtra							S:D1153H	1	0.15	S:L984C	9	0.25
Maharashtra							S:K1154Q	1	0.15	S:D985F	9	0.25
Maharashtra							S:Y1155M	1	0.15	S:Y1007D	9	0.25
Maharashtra							S:F1156L	1	0.15	S:I105V	9	0.25
Maharashtra							S:K1157I	1	0.15	S:R21T	9	0.25
Maharashtra							S:N1158*	1	0.15	S:L877W	9	0.25
Maharashtra							S:H1159V	1	0.15	S:A879R	9	0.25
Maharashtra							S:D1163A	1	0.15	S:G880C	9	0.25
Maharashtra							S:V1164L	1	0.15	S:T881C	9	0.25
Maharashtra							S:D1165M	1	0.15	S:G908T	9	0.25
Maharashtra							S:K1181T	1	0.15	S:G910S	9	0.25
Maharashtra							S:E1182A	1	0.15	S:V911Y	9	0.25

Maharashtra							**S:I1183S**	1	0.15	**S:N919W**	9	0.25
Maharashtra							**S:D1184M**	1	0.15	**S:A942N**	9	0.25
Maharashtra							**S:N1187P**	1	0.15	**S:E154***	9	0.25
Maharashtra							**S:E1188R**	1	0.15	**S:R328***	9	0.25
Maharashtra							**S:V1189I**	1	0.15	**S:P330***	9	0.25
Maharashtra							**S:A1190***	1	0.15	**S:N331S**	9	0.25
Maharashtra							**S:K1191M**	1	0.15	**S:K424S**	9	0.25
Maharashtra							**S:N1194L**	1	0.15	**S:P426E**	9	0.25
Maharashtra							**S:P1213M**	1	0.15	**S:D427F**	9	0.25
Maharashtra							**S:W1214S**	1	0.15	**S:D428***	9	0.25
Maharashtra							**S:Y1215S**	1	0.15	**S:F429Q**	9	0.25
Maharashtra							**S:W1217***	1	0.15	**S:T430S**	9	0.25
Maharashtra							**S:L1218N**	1	0.15	**S:C432F**	9	0.25
Maharashtra							**S:F1220***	1	0.15	**S:I434G**	9	0.25
Maharashtra							**S:I1221L**	1	0.15	**S:A435W**	9	0.25
Maharashtra							**S:G1223***	1	0.15	**S:Y144M**	9	0.25
Maharashtra							**S:I1225F**	1	0.15	**S:Y145I**	9	0.25
Maharashtra							**S:I1227V**	1	0.15	**S:M153V**	9	0.25
Maharashtra							**S:V1228***	1	0.15	**S:E154S**	9	0.25
Maharashtra							**S:M1229P**	1	0.15	**S:W152L**	9	0.25
Maharashtra							**S:L118I**	1	0.15	**S:Q134N**	9	0.25
Maharashtra							**S:T874I**	1	0.15	**S:N148T**	8	0.23
Maharashtra							**S:C361S**	1	0.15	**S:N149T**	8	0.23
Maharashtra							**S:A363I**	1	0.15	**S:C649V**	8	0.23
Maharashtra							**S:T376K**	1	0.15	**S:I651M**	8	0.23
Maharashtra							**S:F377A**	1	0.15	**S:G652F**	8	0.23
Maharashtra							**S:G381R**	1	0.15	**S:A653F**	8	0.23
Maharashtra							**S:V382S**	1	0.15	**S:N658M**	8	0.23
Maharashtra							**S:P384N**	1	0.15	**S:C662L**	8	0.23
Maharashtra							**S:T385Y**	1	0.15	**S:D663V**	8	0.23
Maharashtra							**S:K386S**	1	0.15	**S:A713Q**	8	0.23
Maharashtra							**S:L387I**	1	0.15	**S:I714L**	8	0.23
Maharashtra							**S:A397L**	1	0.15	**S:P715L**	8	0.23
Maharashtra							**S:D398K**	1	0.15	**S:N717L**	8	0.23
Maharashtra							**S:V407M**	1	0.15	**S:F718I**	8	0.23
Maharashtra							**S:F797C**	1	0.15	**S:I720L**	8	0.23
Maharashtra							**S:G799S**	1	0.15	**S:S721L**	8	0.23
Maharashtra							**S:F4S**	1	0.15	**S:V722P**	8	0.23
Maharashtra							**S:I805M**	1	0.15	**S:T723Y**	8	0.23
Maharashtra							**S:M153T**	1	0.15	**S:T724P**	8	0.23
Maharashtra							**S:H1083Y**	1	0.15	**S:E725Q**	8	0.23
Maharashtra							**S:I923N**	1	0.15	**S:V308L**	8	0.23
Maharashtra							**S:A570S**	1	0.15	**S:L226***	8	0.23
Maharashtra							**S:R847T**	1	0.15	**S:V227G**	8	0.23
Maharashtra							**S:D936H**	1	0.15	**S:P1162L**	8	0.23
Maharashtra							**S:K1073N**	1	0.15	**S:L752I**	8	0.23
Maharashtra							**S:R78N**	1	0.15	**S:L753Q**	8	0.23
Maharashtra							**S:F855V**	1	0.15	**S:Q755N**	8	0.23
Maharashtra							**S:G1219V**	1	0.15	**S:Y756A**	8	0.23
Maharashtra							**S:D138G**	1	0.15	**S:G757A**	8	0.23
Maharashtra							**S:I923E**	1	0.15	**S:S758I**	8	0.23
Maharashtra							**S:M1229I**	1	0.15	**S:V911R**	8	0.23
Maharashtra							**S:E309Q**	1	0.15	**S:Q913K**	8	0.23
Maharashtra							**S:V1264L**	1	0.15	**S:V915***	8	0.23
Maharashtra							**S:W152R**	1	0.15	**S:Y917P**	8	0.23
Maharashtra							**S:P589S**	1	0.15	**S:G946F**	8	0.23
Maharashtra							**S:E702Q**	1	0.15	**S:Q762L**	8	0.23
Maharashtra							**S:G1219C**	1	0.15	**S:R765K**	8	0.23
Maharashtra							**S:S13C**	1	0.15	**S:A766T**	8	0.23
Maharashtra							**S:D574Y**	1	0.15	**S:L767K**	8	0.23
Maharashtra							**S:I788V**	1	0.15	**S:G769P**	8	0.23
Maharashtra							**S:Y789F**	1	0.15	**S:I770K**	8	0.23
Maharashtra							**S:K97N**	1	0.15	**S:A771K**	8	0.23
Maharashtra							**S:R21T**	1	0.15	**S:V772F**	8	0.23
Maharashtra							**S:D253N**	1	0.15	**S:E773L**	8	0.23
Maharashtra							**S:P26L**	1	0.15	**S:Q774H**	8	0.23
Maharashtra							**S:K1191N**	1	0.15	**S:D775K**	8	0.23
Maharashtra							**S:A1070S**	1	0.15	**S:K776S**	8	0.23
Maharashtra							**S:G1267R**	1	0.15	**S:T778K**	8	0.23
Maharashtra							**S:A845V**	1	0.15	**S:Q779F**	8	0.23
Maharashtra							**S:S943P**	1	0.15	**S:E780T**	8	0.23
Maharashtra							**S:C1248F**	1	0.15	**S:V781K**	8	0.23
Maharashtra							**S:E1150Q**	1	0.15	**S:F782H**	8	0.23

Maharashtra							**S:D950H**	1	0.15	**S:A783H**	8	0.23
Maharashtra							**S:T1009I**	1	0.15	**S:V785L**	8	0.23
Maharashtra							**S:L141F**	1	0.15	**S:M153I**	7	0.2
Maharashtra							**S:A27V**	1	0.15	**S:Y144I**	7	0.2
Maharashtra							**S:G142V**	1	0.15	**S:Y145T**	7	0.2
Maharashtra							**S:V143F**	1	0.15	**S:H146T**	7	0.2
Maharashtra							**S:Y144I**	1	0.15	**S:Q580L**	7	0.2
Maharashtra							**S:Y145T**	1	0.15	**S:A609R**	7	0.2
Maharashtra							**S:H146T**	1	0.15	**S:G648Q**	7	0.2
Maharashtra							**S:N148T**	1	0.15	**S:S686L**	7	0.2
Maharashtra							**S:N149T**	1	0.15	**S:P728I**	7	0.2
Maharashtra							**S:E298L**	1	0.15	**S:S730Q**	7	0.2
Maharashtra							**S:T299***	1	0.15	**S:M731C**	7	0.2
Maharashtra							**S:V308K**	1	0.15	**S:F1052V**	7	0.2
Maharashtra							**S:K310S**	1	0.15	**S:P1053S**	7	0.2
Maharashtra							**S:G311I**	1	0.15	**S:Q1054T**	7	0.2
Maharashtra							**S:I312K**	1	0.15	**S:H49Y**	7	0.2
Maharashtra							**S:Y313L**	1	0.15	**S:V1104L**	7	0.2
Maharashtra							**S:Q314L**	1	0.15	**S:T114L**	7	0.2
Maharashtra							**S:T323L**	1	0.15	**S:N148***	7	0.2
Maharashtra							**S:I332Q**	1	0.15	**S:N149V**	7	0.2
Maharashtra							**S:T333Q**	1	0.15	**S:K150Q**	7	0.2
Maharashtra							**S:R346G**	1	0.15	**S:T167L**	7	0.2
Maharashtra							**S:F347R**	1	0.15	**S:E169***	7	0.2
Maharashtra							**S:A348E**	1	0.15	**S:Y170C**	7	0.2
Maharashtra							**S:Y365A**	1	0.15	**S:V171E**	7	0.2
Maharashtra							**S:S366T**	1	0.15	**S:S172***	7	0.2
Maharashtra							**S:Y369L**	1	0.15	**S:Q173L**	7	0.2
Maharashtra							**S:N370I**	1	0.15	**S:P174H**	7	0.2
Maharashtra							**S:S371I**	1	0.15	**S:L176***	7	0.2
Maharashtra							**S:A372L**	1	0.15	**S:M177I**	7	0.2
Maharashtra							**S:F374L**	1	0.15	**S:D178C**	7	0.2
Maharashtra							**S:T376V**	1	0.15	**S:S71L**	7	0.2
Maharashtra							**S:F377M**	1	0.15	**S:T108L**	7	0.2
Maharashtra							**S:K378E**	1	0.15	**S:D1084N**	7	0.2
Maharashtra							**S:T385L**	1	0.15	**S:G1085C**	7	0.2
Maharashtra							**S:K386L**	1	0.15	**S:K1086S**	7	0.2
Maharashtra							**S:Y396***	1	0.15	**S:A1087C**	7	0.2
Maharashtra							**S:A397M**	1	0.15	**S:T63L**	7	0.2
Maharashtra							**S:D398I**	1	0.15	**S:D138I**	7	0.2
Maharashtra							**S:F400E**	1	0.15	**S:P139H**	7	0.2
Maharashtra							**S:I402M**	1	0.15	**S:T791I**	7	0.2
Maharashtra							**S:R403K**	1	0.15	**S:A27V**	7	0.2
Maharashtra							**S:G404S**	1	0.15	**S:S750V**	7	0.2
Maharashtra							**S:V407S**	1	0.15	**S:N751V**	7	0.2
Maharashtra							**S:R408L**	1	0.15	**S:Q853L**	7	0.2
Maharashtra							**S:A419I**	1	0.15	**S:K854L**	7	0.2
Maharashtra							**S:D420I**	1	0.15	**S:N856C**	7	0.2
Maharashtra							**S:Y421I**	1	0.15	**S:G857H**	7	0.2
Maharashtra							**S:K424A**	1	0.15	**S:T874C**	7	0.2
Maharashtra							**S:K444***	1	0.15	**S:A876S**	7	0.2
Maharashtra							**S:V445L**	1	0.15	**S:A879V**	7	0.2
Maharashtra							**S:G447I**	1	0.15	**S:L948P**	7	0.2
Maharashtra							**S:N448L**	1	0.15	**S:D950Q**	7	0.2
Maharashtra							**S:Y449T**	1	0.15	**S:V952H**	7	0.2
Maharashtra							**S:N450I**	1	0.15	**S:N953L**	7	0.2
Maharashtra							**S:Y451L**	1	0.15	**S:Q954E**	7	0.2
Maharashtra							**S:L452I**	1	0.15	**S:T761***	7	0.2
Maharashtra							**S:Y453L**	1	0.15	**S:A846L**	7	0.2
Maharashtra							**S:F456S**	1	0.15	**S:R847V**	7	0.2
Maharashtra							**S:R457L**	1	0.15	**S:D848I**	7	0.2
Maharashtra							**S:K458I**	1	0.15	**S:F157S**	7	0.2
Maharashtra							**S:S469L**	1	0.15	**S:P26S**	6	0.17
Maharashtra							**S:T470R**	1	0.15	**S:A879S**	6	0.17
Maharashtra							**S:Y473F**	1	0.15	**S:I598Q**	6	0.17
Maharashtra							**S:A475L**	1	0.15	**S:T599I**	6	0.17
Maharashtra							**S:G476K**	1	0.15	**S:P600L**	6	0.17
Maharashtra							**S:C480I**	1	0.15	**S:G601L**	6	0.17
Maharashtra							**S:N481R**	1	0.15	**S:N603R**	6	0.17
Maharashtra							**S:G482P**	1	0.15	**S:T604L**	6	0.17
Maharashtra							**S:E484A**	1	0.15	**S:S605L**	6	0.17
Maharashtra							**S:G485H**	1	0.15	**S:N606F**	6	0.17
Maharashtra							**S:F486L**	1	0.15	**S:Q607F**	6	0.17

Maharashtra							**S:N487V**	1	0.15	**S:V608I**	6	0.17
Maharashtra							**S:C488M**	1	0.15	**S:A626T**	6	0.17
Maharashtra							**S:Y489V**	1	0.15	**S:D627A**	6	0.17
Maharashtra							**S:V511M**	1	0.15	**S:A684L**	6	0.17
Maharashtra							**S:S514L**	1	0.15	**S:T696N**	6	0.17
Maharashtra							**S:E516V**	1	0.15	**S:M697P**	6	0.17
Maharashtra							**S:L517D**	1	0.15	**S:G1059W**	6	0.17
Maharashtra							**S:K558S**	1	0.15	**S:V1060C**	6	0.17
Maharashtra							**S:Q580L**	1	0.15	**S:V1061S**	6	0.17
Maharashtra							**S:E583L**	1	0.15	**S:F1062L**	6	0.17
Maharashtra							**S:I584M**	1	0.15	**S:H1064A**	6	0.17
Maharashtra							**S:I598Q**	1	0.15	**S:V1065C**	6	0.17
Maharashtra							**S:T599I**	1	0.15	**S:T1066D**	6	0.17
Maharashtra							**S:P600L**	1	0.15	**S:Y1067L**	6	0.17
Maharashtra							**S:G601L**	1	0.15	**S:V1068C**	6	0.17
Maharashtra							**S:N603R**	1	0.15	**S:A1070C**	6	0.17
Maharashtra							**S:T604L**	1	0.15	**S:Q1071T**	6	0.17
Maharashtra							**S:S605L**	1	0.15	**S:E583D**	6	0.17
Maharashtra							**S:N606F**	1	0.15	**S:G181V**	6	0.17
Maharashtra							**S:Q607F**	1	0.15	**S:T547I**	6	0.17
Maharashtra							**S:V608I**	1	0.15	**S:W258L**	6	0.17
Maharashtra							**S:A609R**	1	0.15	**S:G75V**	6	0.17
Maharashtra							**S:A626T**	1	0.15	**S:F306L**	6	0.17
Maharashtra							**S:D627A**	1	0.15	**S:P330S**	6	0.17
Maharashtra							**S:G648Q**	1	0.15	**S:T109L**	6	0.17
Maharashtra							**S:C649V**	1	0.15	**S:K113L**	6	0.17
Maharashtra							**S:I651M**	1	0.15	**S:S116L**	6	0.17
Maharashtra							**S:G652F**	1	0.15	**S:K150H**	6	0.17
Maharashtra							**S:A653F**	1	0.15	**S:S151F**	6	0.17
Maharashtra							**S:E654K**	1	0.15	**S:L141N**	6	0.17
Maharashtra							**S:N658M**	1	0.15	**S:G142F**	6	0.17
Maharashtra							**S:E661P**	1	0.15	**S:N1098K**	6	0.17
Maharashtra							**S:C662L**	1	0.15	**S:N61M**	6	0.17
Maharashtra							**S:D663V**	1	0.15	**S:W64G**	6	0.17
Maharashtra							**S:P665V**	1	0.15	**S:C136V**	6	0.17
Maharashtra							**S:I666Y**	1	0.15	**S:N137M**	6	0.17
Maharashtra							**S:G667A**	1	0.15	**S:T307I**	6	0.17
Maharashtra							**S:A668L**	1	0.15	**S:F79S**	6	0.17
Maharashtra							**S:G669V**	1	0.15	**S:A67V**	6	0.17
Maharashtra							**S:A684L**	1	0.15	**S:G257V**	6	0.17
Maharashtra							**S:S686L**	1	0.15	**S:A475L**	6	0.17
Maharashtra							**S:T696N**	1	0.15	**S:G476K**	6	0.17
Maharashtra							**S:M697P**	1	0.15	**S:C749F**	6	0.17
Maharashtra							**S:A713Q**	1	0.15	**S:C851T**	6	0.17
Maharashtra							**S:I714L**	1	0.15	**S:S937V**	6	0.17
Maharashtra							**S:P715L**	1	0.15	**S:S939L**	6	0.17
Maharashtra							**S:N717L**	1	0.15	**S:S940A**	6	0.17
Maharashtra							**S:F718I**	1	0.15	**S:T941K**	6	0.17
Maharashtra							**S:I720L**	1	0.15	**S:A942F**	6	0.17
Maharashtra							**S:S721L**	1	0.15	**S:S943K**	6	0.17
Maharashtra							**S:V722P**	1	0.15	**S:A944T**	6	0.17
Maharashtra							**S:T723Y**	1	0.15	**S:L945H**	6	0.17
Maharashtra							**S:T724P**	1	0.15	**S:K947L**	6	0.17
Maharashtra							**S:E725Q**	1	0.15	**S:I850L**	6	0.17
Maharashtra							**S:P728I**	1	0.15	**S:D80N**	6	0.17
Maharashtra							**S:S730Q**	1	0.15	**S:P209T**	6	0.17
Maharashtra							**S:M731C**	1	0.15	**S:E661P**	5	0.14
Maharashtra							**S:C749F**	1	0.15	**S:P665V**	5	0.14
Maharashtra							**S:S750V**	1	0.15	**S:I666Y**	5	0.14
Maharashtra							**S:N751V**	1	0.15	**S:G667A**	5	0.14
Maharashtra							**S:L752I**	1	0.15	**S:A668L**	5	0.14
Maharashtra							**S:L753Q**	1	0.15	**S:G669V**	5	0.14
Maharashtra							**S:Q755N**	1	0.15	**S:V143F**	5	0.14
Maharashtra							**S:Y756A**	1	0.15	**S:S151V**	5	0.14
Maharashtra							**S:G757A**	1	0.15	**S:W152G**	5	0.14
Maharashtra							**S:S758I**	1	0.15	**S:D294Q**	5	0.14
Maharashtra							**S:T761***	1	0.15	**S:P295M**	5	0.14
Maharashtra							**S:Q762L**	1	0.15	**S:S297***	5	0.14
Maharashtra							**S:R765K**	1	0.15	**S:E298T**	5	0.14
Maharashtra							**S:A766T**	1	0.15	**S:T299V**	5	0.14
Maharashtra							**S:L767K**	1	0.15	**S:K300H**	5	0.14
Maharashtra							**S:G769P**	1	0.15	**S:C301L**	5	0.14
Maharashtra							**S:I770K**	1	0.15	**S:D442Y**	5	0.14

Maharashtra							**S:A771K**	1	0.15	**S:D111I**	5	0.14
Maharashtra							**S:V772F**	1	0.15	**S:S112T**	5	0.14
Maharashtra							**S:E773L**	1	0.15	**S:Q115M**	5	0.14
Maharashtra							**S:Q774H**	1	0.15	**S:I119K**	5	0.14
Maharashtra							**S:D775K**	1	0.15	**S:V120S**	5	0.14
Maharashtra							**S:K776S**	1	0.15	**S:N121V**	5	0.14
Maharashtra							**S:T778K**	1	0.15	**S:A123F**	5	0.14
Maharashtra							**S:Q779F**	1	0.15	**S:M153E**	5	0.14
Maharashtra							**S:E780T**	1	0.15	**S:S155L**	5	0.14
Maharashtra							**S:V781K**	1	0.15	**S:E156H**	5	0.14
Maharashtra							**S:F782H**	1	0.15	**S:G769A**	5	0.14
Maharashtra							**S:A783H**	1	0.15	**S:I105L**	5	0.14
Maharashtra							**S:V785L**	1	0.15	**S:F106R**	5	0.14
Maharashtra							**S:D843V**	1	0.15	**S:G107S**	5	0.14
Maharashtra							**S:A846L**	1	0.15	**S:M153F**	5	0.14
Maharashtra							**S:R847V**	1	0.15	**S:S155C**	5	0.14
Maharashtra							**S:D848I**	1	0.15	**S:G1099W**	5	0.14
Maharashtra							**S:I850L**	1	0.15	**S:T1100H**	5	0.14
Maharashtra							**S:Q853L**	1	0.15	**S:H1101N**	5	0.14
Maharashtra							**S:K854L**	1	0.15	**S:V62L**	5	0.14
Maharashtra							**S:N856C**	1	0.15	**S:V1228L**	5	0.14
Maharashtra							**S:G857H**	1	0.15	**S:R21I**	5	0.14
Maharashtra							**S:T874C**	1	0.15	**S:K417N**	5	0.14
Maharashtra							**S:A956K**	1	0.15	**S:A701V**	5	0.14
Maharashtra							**S:Q957H**	1	0.15	**S:A1087S**	5	0.14
Maharashtra							**S:L959C**	1	0.15	**S:E484A**	5	0.14
Maharashtra							**S:N960***	1	0.15	**S:F486L**	5	0.14
Maharashtra							**S:L962T**	1	0.15	**S:D843V**	5	0.14
Maharashtra							**S:V963***	1	0.15	**S:V1040F**	5	0.14
Maharashtra							**S:K964L**	1	0.15	**S:D442V**	5	0.14
Maharashtra							**S:L966F**	1	0.15	**S:T29I**	4	0.11
Maharashtra							**S:S967W**	1	0.15	**S:N137C**	4	0.11
Maharashtra							**S:S968G**	1	0.15	**S:P139***	4	0.11
Maharashtra							**S:R983K**	1	0.15	**S:F140S**	4	0.11
Maharashtra							**S:L984C**	1	0.15	**S:Y489S**	4	0.11
Maharashtra							**S:D985F**	1	0.15	**S:F490N**	4	0.11
Maharashtra							**S:Y1007D**	1	0.15	**S:I909V**	4	0.11
Maharashtra							**S:V1008H**	1	0.15	**S:V687L**	4	0.11
Maharashtra							**S:T1009R**	1	0.15	**S:P1263L**	4	0.11
Maharashtra							**S:E1031F**	1	0.15	**S:A1070V**	4	0.11
Maharashtra							**S:D1041***	1	0.15	**S:G142V**	4	0.11
Maharashtra							**S:C1043L**	1	0.15	**S:M153W**	4	0.11
Maharashtra							**S:G1044W**	1	0.15	**S:S155V**	4	0.11
Maharashtra							**S:H1048V**	1	0.15	**S:V159S**	4	0.11
Maharashtra							**S:M1050P**	1	0.15	**S:M177W**	4	0.11
Maharashtra							**S:F1052V**	1	0.15	**S:D178T**	4	0.11
Maharashtra							**S:P1053S**	1	0.15	**S:S305L**	4	0.11
Maharashtra							**S:Q1054T**	1	0.15	**S:L441R**	4	0.11
Maharashtra							**S:G1059W**	1	0.15	**S:T124N**	4	0.11
Maharashtra							**S:V1060C**	1	0.15	**S:N125F**	4	0.11
Maharashtra							**S:V1061S**	1	0.15	**S:V127M**	4	0.11
Maharashtra							**S:F1062L**	1	0.15	**S:A694V**	4	0.11
Maharashtra							**S:H1064A**	1	0.15	**S:P85V**	4	0.11
Maharashtra							**S:V1065C**	1	0.15	**S:W104P**	4	0.11
Maharashtra							**S:T1066D**	1	0.15	**S:N440***	4	0.11
Maharashtra							**S:Y1067L**	1	0.15	**S:S443L**	4	0.11
Maharashtra							**S:V1068C**	1	0.15	**S:Y636R**	4	0.11
Maharashtra							**S:A1070C**	1	0.15	**S:N148Q**	4	0.11
Maharashtra							**S:Q1071T**	1	0.15	**S:N149Q**	4	0.11
Maharashtra							**S:N440I**	1	0.15	**S:F65S**	4	0.11
Maharashtra							**S:L441I**	1	0.15	**S:H66M**	4	0.11
Maharashtra							**S:S443C**	1	0.15	**S:A67L**	4	0.11
Maharashtra							**S:K444F**	1	0.15	**S:I68Y**	4	0.11
Maharashtra							**S:V445I**	1	0.15	**S:H69M**	4	0.11
Maharashtra							**S:G446A**	1	0.15	**S:V70S**	4	0.11
Maharashtra							**S:G447C**	1	0.15	**S:D80A**	4	0.11
Maharashtra							**S:L441R**	1	0.15	**S:D215G**	4	0.11
Maharashtra							**S:D442V**	1	0.15	**S:C1243F**	4	0.11
Maharashtra							**S:T732A**	1	0.15	**S:V1122L**	4	0.11
Maharashtra							**S:G446V**	1	0.15	**S:C480I**	4	0.11
Maharashtra							**S:I233V**	1	0.15	**S:N481R**	4	0.11
Maharashtra							**S:P561L**	1	0.15	**S:G482P**	4	0.11
Maharashtra							**S:D1199N**	1	0.15	**S:G485H**	4	0.11

Maharashtra							**S:A688V**	1	0.15	**S:N487V**	4	0.11
Maharashtra							**S:W152C**	1	0.15	**S:C488M**	4	0.11
Maharashtra							**S:A706V**	1	0.15	**S:Y489V**	4	0.11
Maharashtra							**S:E725D**	1	0.15	**S:T827H**	4	0.11
Maharashtra							**S:D1139Y**	1	0.15	**S:A956T**	4	0.11
Maharashtra							**S:G181T**	1	0.15	**S:Q957R**	4	0.11
Maharashtra							**S:R190S**	1	0.15	**S:A958L**	4	0.11
Maharashtra							**S:D614N**	1	0.15	**S:K1038V**	4	0.11
Maharashtra							**S:T859I**	1	0.15	**S:Y1047Q**	4	0.11
Maharashtra							**S:G75V**	1	0.15	**S:F1062S**	4	0.11
Maharashtra							**S:I896T**	1	0.15	**S:F1103L**	4	0.11
Maharashtra							**S:T19I**	1	0.15	**S:N81Y**	4	0.11
Maharashtra							**S:D228H**	1	0.15	**S:D574Y**	4	0.11
Maharashtra							**S:P1162S**	1	0.15	**S:K444N**	4	0.11
Maharashtra							**S:L452M**	1	0.15	**S:P82T**	4	0.11
Maharashtra							**S:Y204H**	1	0.15	**S:D442F**	4	0.11
Maharashtra							**S:N928Y**	1	0.15	**S:S443F**	4	0.11
Maharashtra							**S:F168I**	1	0.15	**S:S1097C**	4	0.11
Maharashtra							**S:Y1155F**	1	0.15	**S:H1101K**	4	0.11
Maharashtra							**S:H655Y**	1	0.15	**S:W1102E**	4	0.11
Maharashtra							**S:E554Q**	1	0.15	**S:T1105***	4	0.11
Maharashtra							**S:F238V**	1	0.15	**S:Q1106T**	4	0.11
Maharashtra							**S:N1125D**	1	0.15	**S:R1107T**	4	0.11
Maharashtra							**S:R237K**	1	0.15	**S:F1109H**	4	0.11
Maharashtra							**S:A831V**	1	0.15	**S:E1111Y**	4	0.11
Maharashtra							**S:M1237I**	1	0.15	**S:P1112R**	4	0.11
Maharashtra							**S:S704L**	1	0.15	**S:I1114H**	4	0.11
Maharashtra										**S:V83F**	4	0.11
Maharashtra										**S:E554D**	3	0.08
Maharashtra										**S:R246T**	3	0.08
Maharashtra										**S:L1265F**	3	0.08
Maharashtra										**S:K1191N**	3	0.08
Maharashtra										**S:S94F**	3	0.08
Maharashtra										**S:D228***	3	0.08
Maharashtra										**S:L229H**	3	0.08
Maharashtra										**S:P230H**	3	0.08
Maharashtra										**S:I231***	3	0.08
Maharashtra										**S:G232V**	3	0.08
Maharashtra										**S:R158M**	3	0.08
Maharashtra										**S:Y160L**	3	0.08
Maharashtra										**S:P174L**	3	0.08
Maharashtra										**S:S45T**	3	0.08
Maharashtra										**S:S46C**	3	0.08
Maharashtra										**S:V47S**	3	0.08
Maharashtra										**S:L48Y**	3	0.08
Maharashtra										**S:H49L**	3	0.08
Maharashtra										**S:K77M**	3	0.08
Maharashtra										**S:Q271M**	3	0.08
Maharashtra										**S:P272W**	3	0.08
Maharashtra										**S:R273V**	3	0.08
Maharashtra										**S:T274I**	3	0.08
Maharashtra										**S:L276N**	3	0.08
Maharashtra										**S:D290M**	3	0.08
Maharashtra										**S:C291E**	3	0.08
Maharashtra										**S:A292P**	3	0.08
Maharashtra										**S:C336P**	3	0.08
Maharashtra										**S:F338D**	3	0.08
Maharashtra										**S:G339L**	3	0.08
Maharashtra										**S:E340H**	3	0.08
Maharashtra										**S:V341L**	3	0.08
Maharashtra										**S:A352M**	3	0.08
Maharashtra										**S:W353L**	3	0.08
Maharashtra										**S:N354G**	3	0.08
Maharashtra										**S:R355T**	3	0.08
Maharashtra										**S:K356G**	3	0.08
Maharashtra										**S:I358E**	3	0.08
Maharashtra										**S:N536Q**	3	0.08
Maharashtra										**S:N641R**	3	0.08
Maharashtra										**S:Q644N**	3	0.08
Maharashtra										**S:T645R**	3	0.08
Maharashtra										**S:R646G**	3	0.08
Maharashtra										**S:A647***	3	0.08
Maharashtra										**S:V126F**	3	0.08

Maharashtra										**S:K458N**	3	0.08
Maharashtra										**S:C488V**	3	0.08
Maharashtra										**S:H146Q**	3	0.08
Maharashtra										**S:V83L**	3	0.08
Maharashtra										**S:S112I**	3	0.08
Maharashtra										**S:A163V**	3	0.08
Maharashtra										**S:P9L**	3	0.08
Maharashtra										**S:S477I**	3	0.08
Maharashtra										**S:V16I**	3	0.08
Maharashtra										**S:S255F**	3	0.08
Maharashtra										**S:D1163Y**	3	0.08
Maharashtra										**S:L303F**	3	0.08
Maharashtra										**S:R408M**	3	0.08
Maharashtra										**S:Q409K**	3	0.08
Maharashtra										**S:I410S**	3	0.08
Maharashtra										**S:A411D**	3	0.08
Maharashtra										**S:P412K**	3	0.08
Maharashtra										**S:G413S**	3	0.08
Maharashtra										**S:Q414L**	3	0.08
Maharashtra										**S:T415Q**	3	0.08
Maharashtra										**S:K424***	3	0.08
Maharashtra										**S:E661A**	3	0.08
Maharashtra										**S:A956K**	3	0.08
Maharashtra										**S:Q957H**	3	0.08
Maharashtra										**S:L141W**	3	0.08
Maharashtra										**S:V193I**	3	0.08
Maharashtra										**S:V976N**	3	0.08
Maharashtra										**S:N978A**	3	0.08
Maharashtra										**S:D979P**	3	0.08
Maharashtra										**S:S982L**	3	0.08
Maharashtra										**S:I993K**	3	0.08
Maharashtra										**S:D994C**	3	0.08
Maharashtra										**S:R995K**	3	0.08
Maharashtra										**S:R1000***	3	0.08
Maharashtra										**S:L1001S**	3	0.08
Maharashtra										**S:S1003***	3	0.08
Maharashtra										**S:I1018L**	3	0.08
Maharashtra										**S:R1019Q**	3	0.08
Maharashtra										**S:A1020K**	3	0.08
Maharashtra										**S:A1022E**	3	0.08
Maharashtra										**S:N1023L**	3	0.08
Maharashtra										**S:A1025L**	3	0.08
Maharashtra										**S:A1026I**	3	0.08
Maharashtra										**S:T1027L**	3	0.08
Maharashtra										**S:K1028L**	3	0.08
Maharashtra										**S:M1029L**	3	0.08
Maharashtra										**S:S1030L**	3	0.08
Maharashtra										**S:E1031K**	3	0.08
Maharashtra										**S:R1039Y**	3	0.08
Maharashtra										**S:V1040L**	3	0.08
Maharashtra										**S:F1042C**	3	0.08
Maharashtra										**S:C1043P**	3	0.08
Maharashtra										**S:G1044S**	3	0.08
Maharashtra										**S:K1045L**	3	0.08
Maharashtra										**S:G1046S**	3	0.08
Maharashtra										**S:P1079R**	3	0.08
Maharashtra										**S:A1080K**	3	0.08
Maharashtra										**S:I1081E**	3	0.08
Maharashtra										**S:C1082L**	3	0.08
Maharashtra										**S:Y144L**	3	0.08
Maharashtra										**S:P384S**	3	0.08
Maharashtra										**S:V1176F**	3	0.08
Maharashtra										**S:T676I**	3	0.08
Maharashtra										**S:I233V**	3	0.08
Maharashtra										**S:T22S**	3	0.08
Maharashtra										**S:V6F**	3	0.08
Maharashtra										**S:A890V**	3	0.08
Maharashtra										**S:V1065L**	3	0.08
Maharashtra										**S:L176F**	3	0.08
Maharashtra										**S:N439I**	3	0.08
Maharashtra										**S:K444L**	3	0.08
Maharashtra										**S:V445F**	3	0.08
Maharashtra										**S:D796H**	3	0.08

Maharashtra										**S:D1084G**	3	0.08
Maharashtra										**S:L441I**	3	0.08
Maharashtra										**S:G1093L**	3	0.08
Maharashtra										**S:F1121C**	3	0.08
Maharashtra										**S:S1123N**	3	0.08
Maharashtra										**S:G1124R**	3	0.08
Maharashtra										**S:L1141Q**	3	0.08
Maharashtra										**S:P1143H**	3	0.08
Maharashtra										**S:E1144S**	3	0.08
Maharashtra										**S:D1146***	3	0.08
Maharashtra										**S:K1149***	3	0.08
Maharashtra										**S:V615I**	3	0.08
Maharashtra										**S:S939F**	3	0.08
Maharashtra										**S:V120A**	2	0.06
Maharashtra										**S:K795N**	2	0.06
Maharashtra										**S:G1219C**	2	0.06
Maharashtra										**S:G769V**	2	0.06
Maharashtra										**S:S929I**	2	0.06
Maharashtra										**S:G181A**	2	0.06
Maharashtra										**S:G946V**	2	0.06
Maharashtra										**S:S494P**	2	0.06
Maharashtra										**S:D1260N**	2	0.06
Maharashtra										**S:D138N**	2	0.06
Maharashtra										**S:F392L**	2	0.06
Maharashtra										**S:D1146H**	2	0.06
Maharashtra										**S:P217C**	2	0.06
Maharashtra										**S:R237***	2	0.06
Maharashtra										**S:F238V**	2	0.06
Maharashtra										**S:Q239L**	2	0.06
Maharashtra										**S:A243L**	2	0.06
Maharashtra										**S:Q271E**	2	0.06
Maharashtra										**S:K444I**	2	0.06
Maharashtra										**S:T124E**	2	0.06
Maharashtra										**S:N125D**	2	0.06
Maharashtra										**S:V126P**	2	0.06
Maharashtra										**S:N137L**	2	0.06
Maharashtra										**S:D138L**	2	0.06
Maharashtra										**S:S640F**	2	0.06
Maharashtra										**S:N439K**	2	0.06
Maharashtra										**S:Q1208H**	2	0.06
Maharashtra										**S:T19H**	2	0.06
Maharashtra										**S:V36L**	2	0.06
Maharashtra										**S:Y37I**	2	0.06
Maharashtra										**S:Y38L**	2	0.06
Maharashtra										**S:P39S**	2	0.06
Maharashtra										**S:D40H**	2	0.06
Maharashtra										**S:K41V**	2	0.06
Maharashtra										**S:T51F**	2	0.06
Maharashtra										**S:Q52P**	2	0.06
Maharashtra										**S:D53M**	2	0.06
Maharashtra										**S:T73S**	2	0.06
Maharashtra										**S:N74M**	2	0.06
Maharashtra										**S:G75L**	2	0.06
Maharashtra										**S:T76Y**	2	0.06
Maharashtra										**S:R78S**	2	0.06
Maharashtra										**S:F79L**	2	0.06
Maharashtra										**S:D80G**	2	0.06
Maharashtra										**S:N81P**	2	0.06
Maharashtra										**S:P82M**	2	0.06
Maharashtra										**S:R102L**	2	0.06
Maharashtra										**S:G103L**	2	0.06
Maharashtra										**S:D420N**	2	0.06
Maharashtra										**S:N422Q**	2	0.06
Maharashtra										**S:Y423M**	2	0.06
Maharashtra										**S:K424I**	2	0.06
Maharashtra										**S:D442***	2	0.06
Maharashtra										**S:K444P**	2	0.06
Maharashtra										**S:G446***	2	0.06
Maharashtra										**S:N448V**	2	0.06
Maharashtra										**S:Y449***	2	0.06
Maharashtra										**S:N450E**	2	0.06
Maharashtra										**S:Y451V**	2	0.06
Maharashtra										**S:L452***	2	0.06

Maharashtra										**S:Y453S**	2	0.06
Maharashtra										**S:R454Q**	2	0.06
Maharashtra										**S:L455T**	2	0.06
Maharashtra										**S:T470***	2	0.06
Maharashtra										**S:I472R**	2	0.06
Maharashtra										**S:F515N**	2	0.06
Maharashtra										**S:E516C**	2	0.06
Maharashtra										**S:S530W**	2	0.06
Maharashtra										**S:N532***	2	0.06
Maharashtra										**S:L533K**	2	0.06
Maharashtra										**S:K537M**	2	0.06
Maharashtra										**S:T549Q**	2	0.06
Maharashtra										**S:G550F**	2	0.06
Maharashtra										**S:V551Q**	2	0.06
Maharashtra										**S:T553Q**	2	0.06
Maharashtra										**S:E554W**	2	0.06
Maharashtra										**S:S555F**	2	0.06
Maharashtra										**S:K557Q**	2	0.06
Maharashtra										**S:F559V**	2	0.06
Maharashtra										**S:L560S**	2	0.06
Maharashtra										**S:P561A**	2	0.06
Maharashtra										**S:D571P**	2	0.06
Maharashtra										**S:P589Q**	2	0.06
Maharashtra										**S:T602Y**	2	0.06
Maharashtra										**S:S605R**	2	0.06
Maharashtra										**S:L611K**	2	0.06
Maharashtra										**S:Q613C**	2	0.06
Maharashtra										**S:D614C**	2	0.06
Maharashtra										**S:V615Y**	2	0.06
Maharashtra										**S:N616S**	2	0.06
Maharashtra										**S:G648T**	2	0.06
Maharashtra										**S:Q218Y**	2	0.06
Maharashtra										**S:C1236F**	2	0.06
Maharashtra										**S:G72K**	2	0.06
Maharashtra										**S:N74L**	2	0.06
Maharashtra										**S:K97L**	2	0.06
Maharashtra										**S:Q115Y**	2	0.06
Maharashtra										**S:F275R**	2	0.06
Maharashtra										**S:K304***	2	0.06
Maharashtra										**S:T430***	2	0.06
Maharashtra										**S:G667S**	2	0.06
Maharashtra										**S:C671***	2	0.06
Maharashtra										**S:N122F**	2	0.06
Maharashtra										**S:A123E**	2	0.06
Maharashtra										**S:T124D**	2	0.06
Maharashtra										**S:N125P**	2	0.06
Maharashtra										**S:C131***	2	0.06
Maharashtra										**S:D111L**	2	0.06
Maharashtra										**S:K113T**	2	0.06
Maharashtra										**S:Q115L**	2	0.06
Maharashtra										**S:S116M**	2	0.06
Maharashtra										**S:I128L**	2	0.06
Maharashtra										**S:V130S**	2	0.06
Maharashtra										**S:C131V**	2	0.06
Maharashtra										**S:E132N**	2	0.06
Maharashtra										**S:L461***	2	0.06
Maharashtra										**S:K462N**	2	0.06
Maharashtra										**S:P463L**	2	0.06
Maharashtra										**S:F464S**	2	0.06
Maharashtra										**S:E465G**	2	0.06
Maharashtra										**S:S477***	2	0.06
Maharashtra										**S:T478Q**	2	0.06
Maharashtra										**S:E132***	2	0.06
Maharashtra										**S:F133R**	2	0.06
Maharashtra										**S:Q134Y**	2	0.06
Maharashtra										**S:F135***	2	0.06
Maharashtra										**S:Y145D**	2	0.06
Maharashtra										**S:H146G**	2	0.06
Maharashtra										**S:V90F**	2	0.06
Maharashtra										**S:A1020S**	2	0.06
Maharashtra										**S:H69Y**	2	0.06
Maharashtra										**S:I1179V**	2	0.06
Maharashtra										**S:A1020V**	2	0.06

Maharashtra										**S:T95N**	2	0.06
Maharashtra										**S:E96D**	2	0.06
Maharashtra										**S:E1188D**	2	0.06
Maharashtra										**S:T1238I**	2	0.06
Maharashtra										**S:E702Q**	2	0.06
Maharashtra										**S:A411V**	2	0.06
Maharashtra										**S:F817L**	2	0.06
Maharashtra										**S:I105N**	2	0.06
Maharashtra										**S:V159F**	2	0.06
Maharashtra										**S:F490V**	2	0.06
Maharashtra										**S:T299K**	2	0.06
Maharashtra										**S:V308Q**	2	0.06
Maharashtra										**S:T208M**	2	0.06
Maharashtra										**S:L229F**	2	0.06
Maharashtra										**S:W152S**	2	0.06
Maharashtra										**S:D389H**	2	0.06
Maharashtra										**S:G404***	2	0.06
Maharashtra										**S:D405L**	2	0.06
Maharashtra										**S:S968G**	2	0.06
Maharashtra										**S:R190C**	2	0.06
Maharashtra										**S:E191L**	2	0.06
Maharashtra										**S:F192R**	2	0.06
Maharashtra										**S:F194L**	2	0.06
Maharashtra										**S:K195M**	2	0.06
Maharashtra										**S:N196V**	2	0.06
Maharashtra										**S:G199K**	2	0.06
Maharashtra										**S:N211L**	2	0.06
Maharashtra										**S:L212S**	2	0.06
Maharashtra										**S:V213T**	2	0.06
Maharashtra										**S:D215L**	2	0.06
Maharashtra										**S:P217I**	2	0.06
Maharashtra										**S:Q218***	2	0.06
Maharashtra										**S:G219C**	2	0.06
Maharashtra										**S:F220V**	2	0.06
Maharashtra										**S:S221I**	2	0.06
Maharashtra										**S:A222S**	2	0.06
Maharashtra										**S:E224R**	2	0.06
Maharashtra										**S:P225V**	2	0.06
Maharashtra										**S:V227R**	2	0.06
Maharashtra										**S:Q853T**	2	0.06
Maharashtra										**S:Q134I**	2	0.06
Maharashtra										**S:F135L**	2	0.06
Maharashtra										**S:C136***	2	0.06
Maharashtra										**S:N137***	2	0.06
Maharashtra										**S:D138S**	2	0.06
Maharashtra										**S:P139I**	2	0.06
Maharashtra										**S:V143C**	2	0.06
Maharashtra										**S:I203A**	2	0.06
Maharashtra										**S:P1079S**	2	0.06
Maharashtra										**S:C1247F**	2	0.06
Maharashtra										**S:R102G**	2	0.06
Maharashtra										**S:Y145H**	2	0.06
Maharashtra										**S:T76I**	2	0.06
Maharashtra										**S:A520S**	2	0.06
Maharashtra										**S:S494L**	2	0.06
Maharashtra										**S:A93S**	2	0.06
Maharashtra										**S:I1216T**	2	0.06
Maharashtra										**S:V445I**	2	0.06
Maharashtra										**S:K1205R**	2	0.06
Maharashtra										**S:S640P**	2	0.06
Maharashtra										**S:P25S**	2	0.06
Maharashtra										**S:S13N**	2	0.06
Maharashtra										**S:A348S**	2	0.06
Maharashtra										**S:Q957L**	2	0.06
Maharashtra										**S:T95***	2	0.06
Maharashtra										**S:K97V**	2	0.06
Maharashtra										**S:T114Y**	2	0.06
Maharashtra										**S:D138H**	2	0.06
Maharashtra										**S:N211S**	2	0.06
Maharashtra										**S:F2L**	2	0.06
Maharashtra										**S:V622F**	2	0.06
Maharashtra										**S:I468V**	2	0.06
Maharashtra										**S:D1118Y**	2	0.06

Maharashtra										**S:A575S**	2	0.06
Maharashtra										**S:A264S**	2	0.06
Maharashtra										**S:A262V**	2	0.06
Maharashtra										**S:S940F**	2	0.06
Maharashtra										**S:V90I**	2	0.06
Maharashtra										**S:N394I**	2	0.06
Maharashtra										**S:F175L**	2	0.06
Maharashtra										**S:L1224M**	2	0.06
Maharashtra										**S:T859I**	2	0.06
Maharashtra										**S:N439T**	2	0.06
Maharashtra										**S:L441V**	2	0.06
Maharashtra										**S:S443Y**	2	0.06
Maharashtra										**S:N440Y**	2	0.06
Maharashtra										**S:A260S**	2	0.06
Maharashtra										**S:Y837H**	2	0.06
Maharashtra										**S:G1167V**	2	0.06
Maharashtra										**S:D1260Y**	2	0.06
Maharashtra										**S:L296P**	2	0.06
Maharashtra										**S:F1089S**	2	0.06
Maharashtra										**S:P1090***	2	0.06
Maharashtra										**S:E1092C**	2	0.06
Maharashtra										**S:V1094C**	2	0.06
Maharashtra										**S:D364L**	2	0.06
Maharashtra										**S:N437W**	2	0.06
Maharashtra										**S:S438***	2	0.06
Maharashtra										**S:N439L**	2	0.06
Maharashtra										**S:L452P**	2	0.06
Maharashtra										**S:S711K**	2	0.06
Maharashtra										**S:F823N**	2	0.06
Maharashtra										**S:K825M**	2	0.06
Maharashtra										**S:T941V**	2	0.06
Maharashtra										**S:A942L**	2	0.06
Maharashtra										**S:S943L**	2	0.06
Maharashtra										**S:L945K**	2	0.06
Maharashtra										**S:T961N**	2	0.06
Maharashtra										**S:V963A**	2	0.06
Maharashtra										**S:K964P**	2	0.06
Maharashtra										**S:Q965I**	2	0.06
Maharashtra										**S:S967F**	2	0.06
Maharashtra										**S:S968Q**	2	0.06
Maharashtra										**S:N969V**	2	0.06
Maharashtra										**S:G971***	2	0.06
Maharashtra										**S:A972M**	2	0.06
Maharashtra										**S:D1084C**	2	0.06
Maharashtra										**S:G1085F**	2	0.06
Maharashtra										**S:A1087W**	2	0.06
Maharashtra										**S:V1094T**	2	0.06
Maharashtra										**S:F1095L**	2	0.06
Maharashtra										**S:R1019C**	2	0.06
Maharashtra										**S:N99L**	2	0.06
Maharashtra										**S:G103R**	2	0.06
Maharashtra										**S:I105S**	2	0.06
Maharashtra										**S:F106P**	2	0.06
Maharashtra										**S:G107Y**	2	0.06
Maharashtra										**S:N148M**	2	0.06
Maharashtra										**S:N149I**	2	0.06
Maharashtra										**S:N81D**	2	0.06
Maharashtra										**S:S155R**	2	0.06
Maharashtra										**S:T124I**	2	0.06
Maharashtra										**S:W152R**	2	0.06
Maharashtra										**S:E156S**	2	0.06
Maharashtra										**S:V503F**	2	0.06
Maharashtra										**S:S721T**	2	0.06
Maharashtra										**S:Y1067F**	2	0.06
Maharashtra										**S:E96Q**	2	0.06
Maharashtra										**S:S1242N**	2	0.06
Maharashtra										**S:N282I**	2	0.06
Maharashtra										**S:L8F**	2	0.06
Maharashtra										**S:E654Q**	2	0.06
Maharashtra										**S:M1229I**	2	0.06
Maharashtra										**S:G261V**	2	0.06
Maharashtra										**S:M153L**	2	0.06
Maharashtra										**S:D1153H**	2	0.06

Maharashtra										**S:D1168E**	2	0.06
Maharashtra										**S:P251H**	2	0.06
Maharashtra										**S:A958S**	2	0.06
Maharashtra										**S:K1045N**	2	0.06
Maharashtra										**S:C1248S**	1	0.03
Maharashtra										**S:A899S**	1	0.03
Maharashtra										**S:L1063F**	1	0.03
Maharashtra										**S:L753F**	1	0.03
Maharashtra										**S:Q755Y**	1	0.03
Maharashtra										**S:Y756T**	1	0.03
Maharashtra										**S:G757I**	1	0.03
Maharashtra										**S:S758K**	1	0.03
Maharashtra										**S:F759P**	1	0.03
Maharashtra										**S:E1072R**	1	0.03
Maharashtra										**S:N1074E**	1	0.03
Maharashtra										**S:F1075L**	1	0.03
Maharashtra										**S:T1076H**	1	0.03
Maharashtra										**S:T1077N**	1	0.03
Maharashtra										**S:N536T**	1	0.03
Maharashtra										**S:G842S**	1	0.03
Maharashtra										**S:P1162S**	1	0.03
Maharashtra										**S:S680Y**	1	0.03
Maharashtra										**S:S162Y**	1	0.03
Maharashtra										**S:A163F**	1	0.03
Maharashtra										**S:C166V**	1	0.03
Maharashtra										**S:Q949E**	1	0.03
Maharashtra										**S:T22I**	1	0.03
Maharashtra										**S:S884F**	1	0.03
Maharashtra										**S:S680F**	1	0.03
Maharashtra										**S:L938I**	1	0.03
Maharashtra										**S:K77R**	1	0.03
Maharashtra										**S:A623V**	1	0.03
Maharashtra										**S:V70F**	1	0.03
Maharashtra										**S:L1049I**	1	0.03
Maharashtra										**S:C1250F**	1	0.03
Maharashtra										**S:V126I**	1	0.03
Maharashtra										**S:Q14H**	1	0.03
Maharashtra										**S:Y453F**	1	0.03
Maharashtra										**S:F490S**	1	0.03
Maharashtra										**S:Q926H**	1	0.03
Maharashtra										**S:I818Y**	1	0.03
Maharashtra										**S:C136R**	1	0.03
Maharashtra										**S:N137T**	1	0.03
Maharashtra										**S:F140T**	1	0.03
Maharashtra										**S:G142I**	1	0.03
Maharashtra										**S:W152C**	1	0.03
Maharashtra										**S:M153G**	1	0.03
Maharashtra										**S:V308I**	1	0.03
Maharashtra										**S:E224I**	1	0.03
Maharashtra										**S:P225S**	1	0.03
Maharashtra										**S:N370W**	1	0.03
Maharashtra										**S:A372V**	1	0.03
Maharashtra										**S:A397***	1	0.03
Maharashtra										**S:D398***	1	0.03
Maharashtra										**S:G416L**	1	0.03
Maharashtra										**S:K417***	1	0.03
Maharashtra										**S:A419T**	1	0.03
Maharashtra										**S:D420R**	1	0.03
Maharashtra										**S:Y421***	1	0.03
Maharashtra										**S:N422F**	1	0.03
Maharashtra										**S:K424R**	1	0.03
Maharashtra										**S:N121L**	1	0.03
Maharashtra										**S:N122R**	1	0.03
Maharashtra										**S:F175A**	1	0.03
Maharashtra										**S:M177S**	1	0.03
Maharashtra										**S:D178L**	1	0.03
Maharashtra										**S:L179F**	1	0.03
Maharashtra										**S:E180Y**	1	0.03
Maharashtra										**S:G652R**	1	0.03
Maharashtra										**S:A653G**	1	0.03
Maharashtra										**S:T109Y**	1	0.03
Maharashtra										**S:A1190S**	1	0.03
Maharashtra										**S:P384H**	1	0.03

Maharashtra										**S:N17S**	1	0.03
Maharashtra										**S:A288V**	1	0.03
Maharashtra										**S:W104F**	1	0.03
Maharashtra										**S:I128P**	1	0.03
Maharashtra										**S:K129T**	1	0.03
Maharashtra										**S:V130Y**	1	0.03
Maharashtra										**S:E132I**	1	0.03
Maharashtra										**S:Y204F**	1	0.03
Maharashtra										**S:S205***	1	0.03
Maharashtra										**S:K206A**	1	0.03
Maharashtra										**S:T73L**	1	0.03
Maharashtra										**S:D111R**	1	0.03
Maharashtra										**S:L226N**	1	0.03
Maharashtra										**S:R457F**	1	0.03
Maharashtra										**S:F592P**	1	0.03
Maharashtra										**S:G594C**	1	0.03
Maharashtra										**S:V595C**	1	0.03
Maharashtra										**S:R634S**	1	0.03
Maharashtra										**S:V635C**	1	0.03
Maharashtra										**S:I664***	1	0.03
Maharashtra										**S:P665F**	1	0.03
Maharashtra										**S:I666S**	1	0.03
Maharashtra										**S:I670T**	1	0.03
Maharashtra										**S:A672C**	1	0.03
Maharashtra										**S:M697***	1	0.03
Maharashtra										**S:F800T**	1	0.03
Maharashtra										**S:L877R**	1	0.03
Maharashtra										**S:A879C**	1	0.03
Maharashtra										**S:G880I**	1	0.03
Maharashtra										**S:I882N**	1	0.03
Maharashtra										**S:N657W**	1	0.03
Maharashtra										**S:N658C**	1	0.03
Maharashtra										**S:S659R**	1	0.03
Maharashtra										**S:P681M**	1	0.03
Maharashtra										**S:R683S**	1	0.03
Maharashtra										**S:R685G**	1	0.03
Maharashtra										**S:S686T**	1	0.03
Maharashtra										**S:V687***	1	0.03
Maharashtra										**S:A688C**	1	0.03
Maharashtra										**S:V127S**	1	0.03
Maharashtra										**S:K129L**	1	0.03
Maharashtra										**S:V130***	1	0.03
Maharashtra										**S:E132S**	1	0.03
Maharashtra										**S:F133I**	1	0.03
Maharashtra										**S:Q134F**	1	0.03
Maharashtra										**S:F135G**	1	0.03
Maharashtra										**S:I100F**	1	0.03
Maharashtra										**S:V127P**	1	0.03
Maharashtra										**S:I128T**	1	0.03
Maharashtra										**S:K129Y**	1	0.03
Maharashtra										**S:V130C**	1	0.03
Maharashtra										**S:G142***	1	0.03
Maharashtra										**S:G648I**	1	0.03
Maharashtra										**S:Y674A**	1	0.03
Maharashtra										**S:Q675G**	1	0.03
Maharashtra										**S:T696***	1	0.03
Maharashtra										**S:M697C**	1	0.03
Maharashtra										**S:Q52H**	1	0.03
Maharashtra										**S:L1200F**	1	0.03
Maharashtra										**S:E1144Q**	1	0.03
Maharashtra										**S:L7V**	1	0.03
Maharashtra										**S:V70I**	1	0.03
Maharashtra										**S:V159L**	1	0.03
Maharashtra										**S:S98Y**	1	0.03
Maharashtra										**S:G257R**	1	0.03
Maharashtra										**S:A123P**	1	0.03
Maharashtra										**S:F140I**	1	0.03
Maharashtra										**S:T63S**	1	0.03
Maharashtra										**S:N122I**	1	0.03
Maharashtra										**S:V320T**	1	0.03
Maharashtra										**S:F86I**	1	0.03
Maharashtra										**S:N87Y**	1	0.03
Maharashtra										**S:M153D**	1	0.03

Maharashtra										**S:A262S**	1	0.03
Maharashtra										**S:Q23R**	1	0.03
Maharashtra										**S:Q690H**	1	0.03
Maharashtra										**S:S698L**	1	0.03
Maharashtra										**S:R158Q**	1	0.03
Maharashtra										**S:C166D**	1	0.03
Maharashtra										**S:L141M**	1	0.03
Maharashtra										**S:S813I**	1	0.03
Maharashtra										**S:R815E**	1	0.03
Maharashtra										**S:S816V**	1	0.03
Maharashtra										**S:K304E**	1	0.03
Maharashtra										**S:F2I**	1	0.03
Maharashtra										**S:D627N**	1	0.03
Maharashtra										**S:M153S**	1	0.03
Maharashtra										**S:L242V**	1	0.03
Maharashtra										**S:A243I**	1	0.03
Maharashtra										**S:Y265L**	1	0.03
Maharashtra										**S:V289K**	1	0.03
Maharashtra										**S:H625T**	1	0.03
Maharashtra										**S:D627L**	1	0.03
Maharashtra										**S:Q762C**	1	0.03
Maharashtra										**S:L763F**	1	0.03
Maharashtra										**S:G769W**	1	0.03
Maharashtra										**S:I770N**	1	0.03
Maharashtra										**S:A771S**	1	0.03
Maharashtra										**S:D839V**	1	0.03
Maharashtra										**S:C840A**	1	0.03
Maharashtra										**S:L849V**	1	0.03
Maharashtra										**S:A852C**	1	0.03
Maharashtra										**S:F855V**	1	0.03
Maharashtra										**S:N856***	1	0.03
Maharashtra										**S:G857R**	1	0.03
Maharashtra										**S:L858P**	1	0.03
Maharashtra										**S:T859Y**	1	0.03
Maharashtra										**S:V860C**	1	0.03
Maharashtra										**S:E661D**	1	0.03
Maharashtra										**S:S161V**	1	0.03
Maharashtra										**S:A163S**	1	0.03
Maharashtra										**S:N164E**	1	0.03
Maharashtra										**S:S60P**	1	0.03
Maharashtra										**S:V213F**	1	0.03
Maharashtra										**S:R214***	1	0.03
Maharashtra										**S:D215A**	1	0.03
Maharashtra										**S:L216H**	1	0.03
Maharashtra										**S:P217A**	1	0.03
Maharashtra										**S:L226I**	1	0.03
Maharashtra										**S:S704L**	1	0.03
Maharashtra										**S:V62S**	1	0.03
Maharashtra										**S:Y144***	1	0.03
Maharashtra										**S:Y145N**	1	0.03
Maharashtra										**S:I742H**	1	0.03
Maharashtra										**S:I233T**	1	0.03
Maharashtra										**S:C851M**	1	0.03
Maharashtra										**S:L141G**	1	0.03
Maharashtra										**S:G142C**	1	0.03
Maharashtra										**S:V143L**	1	0.03
Maharashtra										**S:Y145P**	1	0.03
Maharashtra										**S:K187***	1	0.03
Maharashtra										**S:N188W**	1	0.03
Maharashtra										**S:R190F**	1	0.03
Maharashtra										**S:E191***	1	0.03
Maharashtra										**S:F192N**	1	0.03
Maharashtra										**S:N196A**	1	0.03
Maharashtra										**S:I197H**	1	0.03
Maharashtra										**S:D198A**	1	0.03
Maharashtra										**S:G199Y**	1	0.03
Maharashtra										**S:Y200***	1	0.03
Maharashtra										**S:K202S**	1	0.03
Maharashtra										**S:Y204***	1	0.03
Maharashtra										**S:Q218S**	1	0.03
Maharashtra										**S:S221F**	1	0.03
Maharashtra										**S:A222G**	1	0.03
Maharashtra										**S:L226W**	1	0.03

Maharashtra										**S:V227F**	1	0.03
Maharashtra										**S:D228K**	1	0.03
Maharashtra										**S:Y369C**	1	0.03
Maharashtra										**S:N370C**	1	0.03
Maharashtra										**S:L387Y**	1	0.03
Maharashtra										**S:N388***	1	0.03
Maharashtra										**S:D389C**	1	0.03
Maharashtra										**S:R78M**	1	0.03
Maharashtra										**S:E96G**	1	0.03
Maharashtra										**S:W886L**	1	0.03
Maharashtra										**S:E309Q**	1	0.03
Maharashtra										**S:K150R**	1	0.03
Maharashtra										**S:S98***	1	0.03
Maharashtra										**S:N99H**	1	0.03
Maharashtra										**S:I100N**	1	0.03
Maharashtra										**S:I101K**	1	0.03
Maharashtra										**S:S112F**	1	0.03
Maharashtra										**S:K113W**	1	0.03
Maharashtra										**S:S116F**	1	0.03
Maharashtra										**S:L117R**	1	0.03
Maharashtra										**S:Y145L**	1	0.03
Maharashtra										**S:H146P**	1	0.03
Maharashtra										**S:E169S**	1	0.03
Maharashtra										**S:K187Q**	1	0.03
Maharashtra										**S:N188K**	1	0.03
Maharashtra										**S:L189S**	1	0.03
Maharashtra										**S:R190***	1	0.03
Maharashtra										**S:E191G**	1	0.03
Maharashtra										**S:F192I**	1	0.03
Maharashtra										**S:S162I**	1	0.03
Maharashtra										**S:T393L**	1	0.03
Maharashtra										**S:S816K**	1	0.03
Maharashtra										**S:D138G**	1	0.03
Maharashtra										**S:C760L**	1	0.03
Maharashtra										**S:P384L**	1	0.03
Maharashtra										**S:S1242T**	1	0.03
Maharashtra										**S:T1120I**	1	0.03
Maharashtra										**S:T1009I**	1	0.03
Maharashtra										**S:T676A**	1	0.03
Maharashtra										**S:A263E**	1	0.03
Maharashtra										**S:S875A**	1	0.03
Maharashtra										**S:T1238N**	1	0.03
Maharashtra										**S:A852V**	1	0.03
Maharashtra										**S:T1077G**	1	0.03
Maharashtra										**S:P1079A**	1	0.03
Maharashtra										**S:A344F**	1	0.03
Maharashtra										**S:R346K**	1	0.03
Maharashtra										**S:S349Q**	1	0.03
Maharashtra										**S:Y351C**	1	0.03
Maharashtra										**S:A352N**	1	0.03
Maharashtra										**S:L455F**	1	0.03
Maharashtra										**S:V213L**	1	0.03
Maharashtra										**S:K206M**	1	0.03
Maharashtra										**S:N1023K**	1	0.03
Maharashtra										**S:G476S**	1	0.03
Maharashtra										**S:V47L**	1	0.03
Maharashtra										**S:G504V**	1	0.03
Maharashtra										**S:M177K**	1	0.03
Maharashtra										**S:K854N**	1	0.03
Maharashtra										**S:N679K**	1	0.03
Maharashtra										**S:A871S**	1	0.03
Maharashtra										**S:Q1071L**	1	0.03
Maharashtra										**S:L118R**	1	0.03
Maharashtra										**S:I712V**	1	0.03
Maharashtra										**S:P812L**	1	0.03
Maharashtra										**S:A647S**	1	0.03
Maharashtra										**S:V36F**	1	0.03
Maharashtra										**S:V635I**	1	0.03
Maharashtra										**S:A243S**	1	0.03
Maharashtra										**S:T385I**	1	0.03
Maharashtra										**S:T430I**	1	0.03
Maharashtra										**S:N440I**	1	0.03
Maharashtra										**S:L441Y**	1	0.03

Maharashtra										**S:S443C**	1	0.03
Maharashtra										**S:K444F**	1	0.03
Maharashtra										**S:V445C**	1	0.03
Maharashtra										**S:N440S**	1	0.03
Maharashtra										**S:V445Y**	1	0.03
Maharashtra										**S:K444Y**	1	0.03
Maharashtra										**S:N440T**	1	0.03
Maharashtra										**S:V445A**	1	0.03
Maharashtra										**S:S255C**	1	0.03
Maharashtra										**S:S256D**	1	0.03
Maharashtra										**S:G257S**	1	0.03
Maharashtra										**S:A288S**	1	0.03
Maharashtra										**S:A647V**	1	0.03
Maharashtra										**S:S255L**	1	0.03
Maharashtra										**S:S256F**	1	0.03
Maharashtra										**S:W258S**	1	0.03
Maharashtra										**S:T259W**	1	0.03
Maharashtra										**S:A260G**	1	0.03
Maharashtra										**S:L441S**	1	0.03
Maharashtra										**S:K444M**	1	0.03
Maharashtra										**S:Q314R**	1	0.03
Maharashtra										**S:S94A**	1	0.03
Maharashtra										**S:R34H**	1	0.03
Maharashtra										**S:A522S**	1	0.03
Maharashtra										**S:S221L**	1	0.03
Maharashtra										**S:Q218R**	1	0.03
Maharashtra										**S:A570S**	1	0.03
Maharashtra										**S:I818V**	1	0.03
Maharashtra										**S:V987F**	1	0.03
Maharashtra										**S:P295N**	1	0.03
Maharashtra										**S:V826H**	1	0.03
Maharashtra										**S:T827L**	1	0.03
Maharashtra										**S:A829K**	1	0.03
Maharashtra										**S:D830I**	1	0.03
Maharashtra										**S:A831Y**	1	0.03
Maharashtra										**S:G832F**	1	0.03
Maharashtra										**S:F833S**	1	0.03
Maharashtra										**S:I834T**	1	0.03
Maharashtra										**S:Q836***	1	0.03
Maharashtra										**S:G838L**	1	0.03
Maharashtra										**S:D839Q**	1	0.03
Maharashtra										**S:C840M**	1	0.03
Maharashtra										**S:G842A**	1	0.03
Maharashtra										**S:D843S**	1	0.03
Maharashtra										**S:I844S**	1	0.03
Maharashtra										**S:A845N**	1	0.03
Maharashtra										**S:A846N**	1	0.03
Maharashtra										**S:D848***	1	0.03
Maharashtra										**S:K1154S**	1	0.03
Maharashtra										**S:F1156I**	1	0.03
Maharashtra										**S:K1157T**	1	0.03
Maharashtra										**S:V62F**	1	0.03
Maharashtra										**S:Y837I**	1	0.03
Maharashtra										**S:Q115***	1	0.03
Maharashtra										**S:L118***	1	0.03
Maharashtra										**S:F201Q**	1	0.03
Maharashtra										**S:L229S**	1	0.03
Maharashtra										**S:I231S**	1	0.03
Maharashtra										**S:V362***	1	0.03
Maharashtra										**S:A363V**	1	0.03
Maharashtra										**S:Y365W**	1	0.03
Maharashtra										**S:L368S**	1	0.03
Maharashtra										**S:Q409S**	1	0.03
Maharashtra										**S:I410R**	1	0.03
Maharashtra										**S:G485V**	1	0.03
Maharashtra										**S:Y489T**	1	0.03
Maharashtra										**S:T553A**	1	0.03
Maharashtra										**S:I584T**	1	0.03
Maharashtra										**S:D627R**	1	0.03
Maharashtra										**S:Q628V**	1	0.03
Maharashtra										**S:N710Q**	1	0.03
Maharashtra										**S:P728V**	1	0.03
Maharashtra										**S:P793H**	1	0.03

Maharashtra										**S:I794Q**	1	0.03
Maharashtra										**S:D796Q**	1	0.03
Maharashtra										**S:F797A**	1	0.03
Maharashtra										**S:G798R**	1	0.03
Maharashtra										**S:D820H**	1	0.03
Maharashtra										**S:I850S**	1	0.03
Maharashtra										**S:A852G**	1	0.03
Maharashtra										**S:I993F**	1	0.03
Maharashtra										**S:D994H**	1	0.03
Maharashtra										**S:R995V**	1	0.03
Maharashtra										**S:I997T**	1	0.03
Maharashtra										**S:T998K**	1	0.03
Maharashtra										**S:G999L**	1	0.03
Maharashtra										**S:Q1002L**	1	0.03
Maharashtra										**S:S1003E**	1	0.03
Maharashtra										**S:M1029K**	1	0.03
Maharashtra										**S:C1032L**	1	0.03
Maharashtra										**S:V1033L**	1	0.03
Maharashtra										**S:G1035S**	1	0.03
Maharashtra										**S:Q1036L**	1	0.03
Maharashtra										**S:E1072V**	1	0.03
Maharashtra										**S:K1073***	1	0.03
Maharashtra										**S:N1074S**	1	0.03
Maharashtra										**S:T1077L**	1	0.03
Maharashtra										**S:A1080***	1	0.03
Maharashtra										**S:I1081R**	1	0.03
Maharashtra										**S:H1083L**	1	0.03
Maharashtra										**S:V1128C**	1	0.03
Maharashtra										**S:G1131Q**	1	0.03
Maharashtra										**S:I1132G**	1	0.03
Maharashtra										**S:V1133G**	1	0.03
Maharashtra										**S:N1134V**	1	0.03
Maharashtra										**S:R319***	1	0.03
Maharashtra										**S:V320L**	1	0.03
Maharashtra										**S:Q321***	1	0.03
Maharashtra										**S:P322S**	1	0.03
Maharashtra										**S:E324Y**	1	0.03
Maharashtra										**S:S325K**	1	0.03
Maharashtra										**S:I326L**	1	0.03
Maharashtra										**S:V656R**	1	0.03
Maharashtra										**S:N657G**	1	0.03
Maharashtra										**S:N658***	1	0.03
Maharashtra										**S:E748W**	1	0.03
Maharashtra										**S:C749***	1	0.03
Maharashtra										**S:S750F**	1	0.03
Maharashtra										**S:L752***	1	0.03
Maharashtra										**S:L753M**	1	0.03
Maharashtra										**S:L754Q**	1	0.03
Maharashtra										**S:L296S**	1	0.03
Maharashtra										**S:S297L**	1	0.03
Maharashtra										**S:E298***	1	0.03
Maharashtra										**S:C301E**	1	0.03
Maharashtra										**S:T302S**	1	0.03
Maharashtra										**S:L303I**	1	0.03
Maharashtra										**S:H655Q**	1	0.03
Maharashtra										**S:F823S**	1	0.03
Maharashtra										**S:L841N**	1	0.03
Maharashtra										**S:G842M**	1	0.03
Maharashtra										**S:S975F**	1	0.03
Maharashtra										**S:V976H**	1	0.03
Maharashtra										**S:L977V**	1	0.03
Maharashtra										**S:N978L**	1	0.03
Maharashtra										**S:D979T**	1	0.03
Maharashtra										**S:I980K**	1	0.03
Maharashtra										**S:S982R**	1	0.03
Maharashtra										**S:R983L**	1	0.03
Maharashtra										**S:L984K**	1	0.03
Maharashtra										**S:D985C**	1	0.03
Maharashtra										**S:V987L**	1	0.03
Maharashtra										**S:E988I**	1	0.03
Maharashtra										**S:A989G**	1	0.03
Maharashtra										**S:E990***	1	0.03
Maharashtra										**S:V991S**	1	0.03

Maharashtra										**S:I993A**	1	0.03
Maharashtra										**S:R1014L**	1	0.03
Maharashtra										**S:A1015L**	1	0.03
Maharashtra										**S:A1016L**	1	0.03
Maharashtra										**S:E1017L**	1	0.03
Maharashtra										**S:I1018K**	1	0.03
Maharashtra										**S:A1020Q**	1	0.03
Maharashtra										**S:A1022V**	1	0.03
Maharashtra										**S:N1023Y**	1	0.03
Maharashtra										**S:A1025D**	1	0.03
Maharashtra										**S:A1026N**	1	0.03
Maharashtra										**S:T1027Q**	1	0.03
Maharashtra										**S:N1074K**	1	0.03
Maharashtra										**S:F1075R**	1	0.03
Maharashtra										**S:E1092F**	1	0.03
Maharashtra										**S:F1095K**	1	0.03
Maharashtra										**S:N1119L**	1	0.03
Maharashtra										**S:T1120C**	1	0.03
Maharashtra										**S:F1121L**	1	0.03
Maharashtra										**S:Y1138L**	1	0.03
Maharashtra										**S:D1139C**	1	0.03
Maharashtra										**S:T76S**	1	0.03
Maharashtra										**S:F92T**	1	0.03
Maharashtra										**S:A93L**	1	0.03
Maharashtra										**S:W104A**	1	0.03
Maharashtra										**S:I105G**	1	0.03
Maharashtra										**S:G107L**	1	0.03
Maharashtra										**S:T108V**	1	0.03
Maharashtra										**S:D111***	1	0.03
Maharashtra										**S:K113R**	1	0.03
Maharashtra										**S:T114R**	1	0.03
Maharashtra										**S:Q115P**	1	0.03
Maharashtra										**S:I598L**	1	0.03
Maharashtra										**S:N360A**	1	0.03
Maharashtra										**S:C361T**	1	0.03
Maharashtra										**S:A363P**	1	0.03
Maharashtra										**S:Y365L**	1	0.03
Maharashtra										**S:V382M**	1	0.03
Maharashtra										**S:S383E**	1	0.03
Maharashtra										**S:P384C**	1	0.03
Maharashtra										**S:D389***	1	0.03
Maharashtra										**S:N532S**	1	0.03
Maharashtra										**S:L533M**	1	0.03
Maharashtra										**S:K535***	1	0.03
Maharashtra										**S:K537A**	1	0.03
Maharashtra										**S:C538Q**	1	0.03
Maharashtra										**S:V551F**	1	0.03
Maharashtra										**S:T553L**	1	0.03
Maharashtra										**S:E554S**	1	0.03
Maharashtra										**S:S555L**	1	0.03
Maharashtra										**S:N556T**	1	0.03
Maharashtra										**S:A609L**	1	0.03
Maharashtra										**S:V610L**	1	0.03
Maharashtra										**S:Y612F**	1	0.03
Maharashtra										**S:Q613M**	1	0.03
Maharashtra										**S:D614Q**	1	0.03
Maharashtra										**S:C617L**	1	0.03
Maharashtra										**S:T618L**	1	0.03
Maharashtra										**S:E619L**	1	0.03
Maharashtra										**S:V620L**	1	0.03
Maharashtra										**S:P621G**	1	0.03
Maharashtra										**S:A623F**	1	0.03
Maharashtra										**S:A647L**	1	0.03
Maharashtra										**S:S686C**	1	0.03
Maharashtra										**S:V687H**	1	0.03
Maharashtra										**S:A688L**	1	0.03
Maharashtra										**S:S689V**	1	0.03
Maharashtra										**S:V1008D**	1	0.03
Maharashtra										**S:T1009H**	1	0.03
Maharashtra										**S:Q1010R**	1	0.03
Maharashtra										**S:L1012T**	1	0.03
Maharashtra										**S:I1013S**	1	0.03
Maharashtra										**S:R1014K**	1	0.03

Maharashtra										**S:A1015F**	1	0.03
Maharashtra										**S:E1017D**	1	0.03
Maharashtra										**S:A1020D**	1	0.03
Maharashtra										**S:A1022***	1	0.03
Maharashtra										**S:A1056C**	1	0.03
Maharashtra										**S:H1058C**	1	0.03
Maharashtra										**S:G1059T**	1	0.03
Maharashtra										**S:V1060R**	1	0.03
Maharashtra										**S:V1061K**	1	0.03
Maharashtra										**S:F1062E**	1	0.03
Maharashtra										**S:P1079N**	1	0.03
Maharashtra										**S:A1080C**	1	0.03
Maharashtra										**S:I1081S**	1	0.03
Maharashtra										**S:G1171C**	1	0.03
Maharashtra										**S:E96L**	1	0.03
Maharashtra										**S:S98L**	1	0.03
Maharashtra										**S:I100***	1	0.03
Maharashtra										**S:T95G**	1	0.03
Maharashtra										**S:E96F**	1	0.03
Maharashtra										**S:S98V**	1	0.03
Maharashtra										**S:I100L**	1	0.03
Maharashtra										**S:A668D**	1	0.03
Maharashtra										**S:G669S**	1	0.03
Maharashtra										**S:I670D**	1	0.03
Maharashtra										**S:A672F**	1	0.03
Maharashtra										**S:Y160V**	1	0.03
Maharashtra										**S:R246F**	1	0.03
Maharashtra										**S:S247F**	1	0.03
Maharashtra										**S:Y248R**	1	0.03
Maharashtra										**S:T250D**	1	0.03
Maharashtra										**S:P251S**	1	0.03
Maharashtra										**S:G252W**	1	0.03
Maharashtra										**S:D253C**	1	0.03
Maharashtra										**S:S254C**	1	0.03
Maharashtra										**S:T20N**	1	0.03
Maharashtra										**S:T250I**	1	0.03
Maharashtra										**S:S155E**	1	0.03
Maharashtra										**S:G232C**	1	0.03
Maharashtra										**S:T114I**	1	0.03
Maharashtra										**S:S116K**	1	0.03
Maharashtra										**S:L117W**	1	0.03
Maharashtra										**S:L118I**	1	0.03
Maharashtra										**S:V120I**	1	0.03
Maharashtra										**S:A123N**	1	0.03
Maharashtra										**S:N125S**	1	0.03
Maharashtra										**S:V126S**	1	0.03
Maharashtra										**S:L1197F**	1	0.03
Maharashtra										**S:A1174V**	1	0.03
Maharashtra										**S:V70C**	1	0.03
Maharashtra										**S:G72W**	1	0.03
Maharashtra										**S:T73D**	1	0.03
Maharashtra										**S:N74Q**	1	0.03
Maharashtra										**S:R158E**	1	0.03
Maharashtra										**S:T385Y**	1	0.03
Maharashtra										**S:K386***	1	0.03
Maharashtra										**S:V341I**	1	0.03
Maharashtra										**S:I410D**	1	0.03
Maharashtra										**S:P1069S**	1	0.03
Maharashtra										**S:K1245N**	1	0.03
Maharashtra										**S:S151G**	1	0.03
Maharashtra										**S:K147Q**	1	0.03
Maharashtra										**S:R815N**	1	0.03
Maharashtra										**S:S816F**	1	0.03
Maharashtra										**S:V382F**	1	0.03
Maharashtra										**S:N1178D**	1	0.03
Maharashtra										**S:C15F**	1	0.03
Maharashtra										**S:T604N**	1	0.03
Maharashtra										**S:P26L**	1	0.03
Maharashtra										**S:D287I**	1	0.03
Maharashtra										**S:S297P**	1	0.03
Maharashtra										**S:N487K**	1	0.03
Maharashtra										**S:K786R**	1	0.03
Maharashtra										**S:R408K**	1	0.03

Maharashtra										**S:T1076I**	1	0.03
Maharashtra										**S:T33I**	1	0.03
Maharashtra										**S:A419S**	1	0.03
Maharashtra										**S:H245Y**	1	0.03
Maharashtra										**S:I119L**	1	0.03
Maharashtra										**S:K378D**	1	0.03
Maharashtra										**S:G1167S**	1	0.03
Maharashtra										**S:D1168Y**	1	0.03
Maharashtra										**S:N1192R**	1	0.03
Maharashtra										**S:L1193N**	1	0.03
Maharashtra										**S:N1194***	1	0.03
Maharashtra										**S:E1195P**	1	0.03
Maharashtra										**S:S1196P**	1	0.03
Maharashtra										**S:L1197Q**	1	0.03
Maharashtra										**S:I1198***	1	0.03
Maharashtra										**S:D1199G**	1	0.03
Maharashtra										**S:L1200C**	1	0.03
Maharashtra										**S:L1203T**	1	0.03
Maharashtra										**S:G1204W**	1	0.03
Maharashtra										**S:Y1206V**	1	0.03
Maharashtra										**S:E1207***	1	0.03
Maharashtra										**S:Q1208A**	1	0.03
Maharashtra										**S:Y1209V**	1	0.03
Maharashtra										**S:I1210Y**	1	0.03
Maharashtra										**S:W1212M**	1	0.03
Maharashtra										**S:P1213A**	1	0.03
Maharashtra										**S:W1214M**	1	0.03
Maharashtra										**S:Y1215V**	1	0.03
Maharashtra										**S:I1216H**	1	0.03
Maharashtra										**S:W1217L**	1	0.03
Maharashtra										**S:L1218A**	1	0.03
Maharashtra										**S:G1219R**	1	0.03
Maharashtra										**S:I1221Y**	1	0.03
Maharashtra										**S:A1222S**	1	0.03
Maharashtra										**S:G1223W**	1	0.03
Maharashtra										**S:I1225D**	1	0.03
Maharashtra										**S:A1226C**	1	0.03
Maharashtra										**S:I1227H**	1	0.03
Maharashtra										**S:V1228I**	1	0.03
Maharashtra										**S:D571F**	1	0.03
Maharashtra										**S:T572P**	1	0.03
Maharashtra										**S:D574I**	1	0.03
Maharashtra										**S:A575W**	1	0.03
Maharashtra										**S:V576Q**	1	0.03
Maharashtra										**S:D936N**	1	0.03
Maharashtra										**S:V83C**	1	0.03
Maharashtra										**S:P85C**	1	0.03
Maharashtra										**S:N87H**	1	0.03
Maharashtra										**S:D88***	1	0.03
Maharashtra										**S:G89E**	1	0.03
Maharashtra										**S:L110***	1	0.03
Maharashtra										**S:D111H**	1	0.03
Maharashtra										**S:S112N**	1	0.03
Maharashtra										**S:G72Y**	1	0.03
Maharashtra										**S:S94P**	1	0.03
Maharashtra										**S:E96N**	1	0.03
Maharashtra										**S:T109R**	1	0.03
Maharashtra										**S:G142Q**	1	0.03
Maharashtra										**S:V143K**	1	0.03
Maharashtra										**S:T63N**	1	0.03
Maharashtra										**S:M177V**	1	0.03
Maharashtra										**S:P25L**	1	0.03
Maharashtra										**S:N658Y**	1	0.03
Maharashtra										**S:V193L**	1	0.03
Maharashtra										**S:S1252F**	1	0.03
Maharashtra										**S:T573I**	1	0.03
Maharashtra										**S:A1070S**	1	0.03
Maharashtra										**S:H146R**	1	0.03
Maharashtra										**S:F106L**	1	0.03
Maharashtra										**S:F65V**	1	0.03
Maharashtra										**S:R78E**	1	0.03
Maharashtra										**S:M177L**	1	0.03
Maharashtra										**S:V70G**	1	0.03

Maharashtra										**S:T73I**	1	0.03
Maharashtra										**S:N74F**	1	0.03
Maharashtra										**S:S161W**	1	0.03
Maharashtra										**S:S162K**	1	0.03
Maharashtra										**S:R319E**	1	0.03
Maharashtra										**S:V320I**	1	0.03
Maharashtra										**S:S689G**	1	0.03
Maharashtra										**S:I870V**	1	0.03
Maharashtra										**S:V367F**	1	0.03
Maharashtra										**S:T333I**	1	0.03
Maharashtra										**S:A684V**	1	0.03
Maharashtra										**S:L179V**	1	0.03
Maharashtra										**S:W152E**	1	0.03
Maharashtra										**S:M153K**	1	0.03
Maharashtra										**S:S155N**	1	0.03
Maharashtra										**S:C166F**	1	0.03
Maharashtra										**S:T167Y**	1	0.03
Maharashtra										**S:E406Q**	1	0.03
Maharashtra										**S:S708F**	1	0.03
Maharashtra										**S:R102I**	1	0.03
Maharashtra										**S:H245R**	1	0.03
Maharashtra										**S:D1146Y**	1	0.03
Maharashtra										**S:A522V**	1	0.03
Maharashtra										**S:T1117I**	1	0.03
Maharashtra										**S:A684T**	1	0.03
Maharashtra										**S:T549A**	1	0.03
Maharashtra										**S:V860L**	1	0.03
Maharashtra										**S:A67S**	1	0.03
Maharashtra										**S:G413R**	1	0.03
Maharashtra										**S:P499T**	1	0.03
Maharashtra										**S:T19K**	1	0.03
Maharashtra										**S:R21K**	1	0.03
Maharashtra										**S:H69N**	1	0.03
Maharashtra										**S:V70K**	1	0.03
Maharashtra										**S:S71C**	1	0.03
Maharashtra										**S:N74E**	1	0.03
Maharashtra										**S:G75D**	1	0.03
Maharashtra										**S:T76L**	1	0.03
Maharashtra										**S:R408S**	1	0.03
Maharashtra										**S:M740I**	1	0.03
Maharashtra										**S:D1084Y**	1	0.03
Maharashtra										**S:A243T**	1	0.03
Maharashtra										**S:R815K**	1	0.03
Maharashtra										**S:E1111K**	1	0.03
Maharashtra										**S:C1235F**	1	0.03
Maharashtra										**S:V127I**	1	0.03
Maharashtra										**S:V1060I**	1	0.03
Maharashtra										**S:L141F**	1	0.03
Maharashtra										**S:I1114V**	1	0.03
Maharashtra										**S:D737V**	1	0.03
Maharashtra										**S:G1223S**	1	0.03
Maharashtra										**S:L1234F**	1	0.03
Maharashtra										**S:N164I**	1	0.03
Maharashtra										**S:F86P**	1	0.03
Maharashtra										**S:R78G**	1	0.03
Maharashtra										**S:F59C**	1	0.03
Maharashtra										**S:Y269F**	1	0.03
Maharashtra										**S:L277I**	1	0.03
Maharashtra										**S:S116V**	1	0.03
Maharashtra										**S:T73P**	1	0.03
Maharashtra										**S:A262G**	1	0.03
Maharashtra										**S:L461F**	1	0.03
Maharashtra										**S:F79G**	1	0.03
	**Envelope (E)**											
**Maharashtra**	**Pre-Wave 1**			**Wave 1**			**Pre-Wave 2**			**Wave 2**		
Maharashtra	**Mutation**	**Count**	**percent**	**Mutation**	**Count**	**percent**	**Mutation**	**Count**	**percent**	**Mutation**	**Count**	**percent**
Maharashtra	**E:S68F**	18	2.62	**E:S68F**	28	3.58	**E:P71L**	2	0.3	**E:L21F**	16	0.45
Maharashtra	**E:R38G**	7	1.02	**E:L21F**	7	0.89	**E:V62F**	2	0.3	**E:V62F**	15	0.42
Maharashtra	**E:L73F**	4	0.58	**E:V49L**	1	0.13	**E:V49L**	2	0.3	**E:P71L**	8	0.23
Maharashtra	**E:L37T**	2	0.29	**E:L73F**	1	0.13	**E:S68F**	2	0.3	**E:V49L**	6	0.17

Maharashtra	E:R38*	2	0.29	E:D72Y	1	0.13	E:L28A	1	0.15	E:C43F	3	0.08
Maharashtra	E:L37S	1	0.15	E:L73V	1	0.13	E:S55F	1	0.15	E:P71S	3	0.08
Maharashtra	E:R38L	1	0.15	E:V75*	1	0.13	E:L37V	1	0.15	E:V58F	2	0.06
Maharashtra	E:A36I	1	0.15	E:T9I	1	0.13	E:R38T	1	0.15	E:S55F	2	0.06
Maharashtra	E:T35A	1	0.15	E:C43R	1	0.13	E:I33R	1	0.15	E:F26S	2	0.06
Maharashtra	E:V29S	1	0.15	E:Y42C	1	0.13	E:L34T	1	0.15	E:A36V	1	0.03
Maharashtra	E:T30S	1	0.15	E:C43T	1	0.13	E:V47I	1	0.15	E:V75L	1	0.03
Maharashtra	E:C44*	1	0.15	E:N45C	1	0.13	E:R61L	1	0.15	E:A36L	1	0.03
Maharashtra	E:L31P	1	0.15	E:I46G	1	0.13	E:T30A	1	0.15	E:P71T	1	0.03
Maharashtra	E:V29G	1	0.15	E:V58F	1	0.13				E:T35A	1	0.03
Maharashtra	E:L65M	1	0.15							E:D72G	1	0.03
Maharashtra	E:L21F	1	0.15							E:I33M	1	0.03
Maharashtra	E:R38C	1	0.15							E:N66T	1	0.03
Maharashtra	E:A32*	1	0.15							E:D72Y	1	0.03
Maharashtra	E:L34I	1	0.15							E:S68F	1	0.03
Maharashtra	E:A36G	1	0.15							E:P54S	1	0.03
Maharashtra	E:L37H	1	0.15							E:N48K	1	0.03
Maharashtra										E:F26L	1	0.03
Maharashtra										E:L37S	1	0.03
Maharashtra										E:T35F	1	0.03
Maharashtra										E:L37F	1	0.03
	Membrane (M)											
Maharashtra	Pre-Wave 1			Wave 1			Pre-Wave 2			Wave 2		
Maharashtra	Mutation	Count	percent	Mutation	Count	percent	Mutation	Count	percent	Mutation	Count	percent
Maharashtra	M:L29F	3	0.44	M:W55L	5	0.64	M:I82S	114	17.38	M:I82T	1199	33.89
Maharashtra	M:D3A	2	0.29	M:L29F	2	0.26	M:L22I	3	0.46	M:I82S	707	19.98
Maharashtra	M:S136N	2	0.29	M:D163E	2	0.26	M:I82T	3	0.46	M:F28L	5	0.14
Maharashtra	M:V66L	1	0.15	M:A2S	1	0.13	M:R101K	2	0.3	M:M91L	5	0.14
Maharashtra	M:A69S	1	0.15	M:L17F	1	0.13	M:A2S	2	0.3	M:L29F	4	0.11
Maharashtra	M:T77N	1	0.15	M:Y199S	1	0.13	M:R131I	2	0.3	M:A2S	4	0.11
Maharashtra	M:S4C	1	0.15	M:R200W	1	0.13	M:I73L	2	0.3	M:V70F	3	0.08
Maharashtra	M:D3G	1	0.15	M:I201S	1	0.13	M:L17I	2	0.3	M:S94G	3	0.08
Maharashtra	M:H125Y	1	0.15	M:S94I	1	0.13	M:C33F	2	0.3	M:W75L	3	0.08
Maharashtra				M:D160Y	1	0.13	M:D209Y	2	0.3	M:V60L	3	0.08
Maharashtra				M:H155Y	1	0.13	M:L87F	1	0.15	M:V70L	3	0.08
Maharashtra				M:E11K	1	0.13	M:L62T	1	0.15	M:M109I	2	0.06
Maharashtra				M:L35K	1	0.13	M:L102Q	1	0.15	M:V23L	2	0.06
Maharashtra				M:G78C	1	0.13	M:F103L	1	0.15	M:A142V	2	0.06
Maharashtra				M:I82S	1	0.13	M:A104K	1	0.15	M:R146H	2	0.06
Maharashtra				M:V23L	1	0.13	M:R105L	1	0.15	M:D160Y	2	0.06
Maharashtra							M:A63T	1	0.15	M:D163E	2	0.06
Maharashtra							M:W55*	1	0.15	M:I97V	2	0.06
Maharashtra							M:L87P	1	0.15	M:A85S	2	0.06
Maharashtra							M:G89E	1	0.15	M:L34F	2	0.06
Maharashtra							M:M91Q	1	0.15	M:I76V	2	0.06
Maharashtra							M:W92L	1	0.15	M:C64F	2	0.06
Maharashtra							M:S94H	1	0.15	M:L87F	1	0.03
Maharashtra							M:Y95C	1	0.15	M:C86F	1	0.03
Maharashtra							M:R101Q	1	0.15	M:A98S	1	0.03
Maharashtra							M:L102T	1	0.15	M:D209Y	1	0.03
Maharashtra							M:F103V	1	0.15	M:G189S	1	0.03
Maharashtra							M:A104C	1	0.15	M:T77S	1	0.03
Maharashtra							M:R105A	1	0.15	M:V10A	1	0.03
Maharashtra							M:L102F	1	0.15	M:M1I	1	0.03
Maharashtra							M:M1V	1	0.15	M:A81S	1	0.03
Maharashtra							M:A2I	1	0.15	M:K15N	1	0.03
Maharashtra							M:D3G	1	0.15	M:V66L	1	0.03
Maharashtra							M:W20Y	1	0.15	M:D160G	1	0.03
Maharashtra							M:S4F	1	0.15	M:R158H	1	0.03
Maharashtra							M:A85V	1	0.15	M:S136N	1	0.03
Maharashtra							M:E12K	1	0.15	M:H125Y	1	0.03
Maharashtra							M:G6N	1	0.15	M:T77H	1	0.03
Maharashtra							M:T7N	1	0.15	M:R158L	1	0.03
Maharashtra							M:E18*	1	0.15	M:L22I	1	0.03
Maharashtra							M:Q19*	1	0.15	M:D209H	1	0.03
Maharashtra							M:K14*	1	0.15	M:R105H	1	0.03
Maharashtra							M:S111K	1	0.15	M:L87I	1	0.03
Maharashtra							M:V70F	1	0.15	M:C33F	1	0.03
Maharashtra							M:V70L	1	0.15	M:S211F	1	0.03

Maharashtra							**M:R186C**	1	0.15	**M:K162N**	1	0.03
Maharashtra							**M:I128V**	1	0.15	**M:M84I**	1	0.03
Maharashtra							**M:A98S**	1	0.15	**M:S211P**	1	0.03
Maharashtra							**M:L29F**	1	0.15	**M:I201V**	1	0.03
Maharashtra							**M:L124F**	1	0.15	**M:D190E**	1	0.03
Maharashtra							**M:H125Y**	1	0.15	**M:H155Y**	1	0.03
Maharashtra							**M:V10N**	1	0.15	**M:F100S**	1	0.03
Maharashtra							**M:E11H**	1	0.15	**M:R174L**	1	0.03
Maharashtra										**M:T30I**	1	0.03
Maharashtra										**M:I82A**	1	0.03
Maharashtra										**M:D3Y**	1	0.03
Maharashtra										**M:T175M**	1	0.03
Maharashtra										**M:Q185H**	1	0.03
	**Nucleocapsid (N)**											
**Maharashtra**	**Pre-Wave 1**			**Wave 1**			**Pre-Wave 2**			**Wave 2**		
Maharashtra	**Mutation**	**Count**	**percent**	**Mutation**	**Count**	**percent**	**Mutation**	**Count**	**percent**	**Mutation**	**Count**	**percent**
Maharashtra	**N:R203K**	437	63.7	**N:R203K**	570	72.8	**N:D377Y**	221	33.69	**N:R203M**	2849	80.53
Maharashtra	**N:G204R**	437	63.7	**N:G204R**	567	72.41	**N:R203M**	214	32.62	**N:D377Y**	2699	76.29
Maharashtra	**N:P13L**	44	6.41	**N:S194L**	74	9.45	**N:S194L**	194	29.57	**N:D63G**	839	23.71
Maharashtra	**N:T334I**	15	2.19	**N:D371V**	26	3.32	**N:R203K**	183	27.9	**N:G215C**	364	10.29
Maharashtra	**N:S194L**	11	1.6	**N:T205I**	24	3.07	**N:G204R**	182	27.74	**N:D3Y**	279	7.89
Maharashtra	**N:S202N**	9	1.31	**N:Q9K**	22	2.81	**N:S2P**	120	18.29	**N:R203K**	259	7.32
Maharashtra	**N:D371V**	8	1.17	**N:K387R**	17	2.17	**N:D3Y**	33	5.03	**N:G204R**	256	7.24
Maharashtra	**N:S33I**	6	0.87	**N:S2P**	13	1.66	**N:S235F**	31	4.73	**N:S194L**	180	5.09
Maharashtra	**N:S188P**	6	0.87	**N:M210I**	11	1.4	**N:D3L**	29	4.42	**N:R385K**	120	3.39
Maharashtra	**N:K387R**	5	0.73	**N:V72I**	9	1.15	**N:R209I**	27	4.12	**N:P67S**	116	3.28
Maharashtra	**N:R209T**	4	0.58	**N:R209T**	9	1.15	**N:A211V**	20	3.05	**N:S2P**	110	3.11
Maharashtra	**N:R189H**	4	0.58	**N:R209I**	7	0.89	**N:P67S**	20	3.05	**N:S235F**	99	2.8
Maharashtra	**N:R195I**	2	0.29	**N:D128Y**	6	0.77	**N:M234I**	6	0.91	**N:D3L**	45	1.27
Maharashtra	**N:D399Y**	2	0.29	**N:A398V**	6	0.77	**N:A138S**	6	0.91	**N:T205I**	23	0.65
Maharashtra	**N:G19R**	2	0.29	**N:R203M**	6	0.77	**N:R92S**	6	0.91	**N:R209I**	21	0.59
Maharashtra	**N:T205I**	2	0.29	**N:D377Y**	5	0.64	**N:D144Y**	5	0.76	**N:P13S**	17	0.48
Maharashtra	**N:K370N**	2	0.29	**N:M234L**	5	0.64	**N:T141I**	5	0.76	**N:P13L**	14	0.4
Maharashtra	**N:D371Y**	2	0.29	**N:S202I**	4	0.51	**N:T379I**	4	0.61	**N:A12G**	14	0.4
Maharashtra	**N:D128Y**	2	0.29	**N:G25C**	4	0.51	**N:A220V**	4	0.61	**N:D144H**	14	0.4
Maharashtra	**N:G212C**	2	0.29	**N:P13L**	4	0.51	**N:P13S**	3	0.46	**N:A211V**	13	0.37
Maharashtra	**N:A35T**	2	0.29	**N:A398S**	4	0.51	**N:T205I**	3	0.46	**N:T366I**	13	0.37
Maharashtra	**N:V72I**	2	0.29	**N:T141I**	4	0.51	**N:P13L**	3	0.46	**N:M234I**	11	0.31
Maharashtra	**N:T271I**	1	0.15	**N:K374N**	3	0.38	**N:D63G**	3	0.46	**N:S193I**	10	0.28
Maharashtra	**N:F363L**	1	0.15	**N:S413I**	3	0.38	**N:S190I**	3	0.46	**N:P168Q**	10	0.28
Maharashtra	**N:G30V**	1	0.15	**N:D3Y**	3	0.38	**N:A119S**	3	0.46	**N:L139F**	10	0.28
Maharashtra	**N:Q272H**	1	0.15	**N:S190I**	3	0.38	**N:R191L**	2	0.3	**N:G238C**	9	0.25
Maharashtra	**N:T379I**	1	0.15	**N:G215C**	3	0.38	**N:V392L**	2	0.3	**N:S180I**	9	0.25
Maharashtra	**N:D402Y**	1	0.15	**N:A359S**	3	0.38	**N:P168S**	2	0.3	**N:A359S**	8	0.23
Maharashtra	**N:S193I**	1	0.15	**N:P383L**	3	0.38	**N:I320F**	2	0.3	**N:M210I**	7	0.2
Maharashtra	**N:P13T**	1	0.15	**N:Q70R**	3	0.38	**N:G238C**	2	0.3	**N:E367G**	7	0.2
Maharashtra	**N:T247S**	1	0.15	**N:A211V**	3	0.38	**N:A134V**	2	0.3	**N:M1L**	7	0.2
Maharashtra	**N:T282I**	1	0.15	**N:P383S**	3	0.38	**N:G215S**	2	0.3	**N:T362I**	7	0.2
Maharashtra	**N:A414L**	1	0.15	**N:D402Y**	3	0.38	**N:A182S**	2	0.3	**N:A152S**	7	0.2
Maharashtra	**N:D415N**	1	0.15	**N:T198I**	2	0.26	**N:L121F**	2	0.3	**N:T141I**	7	0.2
Maharashtra	**N:K372E**	1	0.15	**N:Q289H**	2	0.26	**N:R209T**	2	0.3	**N:T135I**	6	0.17
Maharashtra	**N:A35G**	1	0.15	**N:R191L**	2	0.26	**N:A119V**	2	0.3	**N:P383L**	6	0.17
Maharashtra	**N:M234I**	1	0.15	**N:T205A**	2	0.26	**N:T135I**	2	0.3	**N:G238V**	6	0.17
Maharashtra	**N:S235V**	1	0.15	**N:A414T**	2	0.26	**N:S193I**	2	0.3	**N:A182S**	6	0.17
Maharashtra	**N:Y360S**	1	0.15	**N:Q9H**	2	0.26	**N:D103Y**	2	0.3	**N:T271I**	6	0.17
Maharashtra	**N:S413I**	1	0.15	**N:E290D**	2	0.26	**N:M210I**	2	0.3	**N:S33I**	6	0.17
Maharashtra	**N:P46S**	1	0.15	**N:D401Y**	2	0.26	**N:D144H**	2	0.3	**N:A398V**	5	0.14
Maharashtra	**N:A414T**	1	0.15	**N:S201N**	2	0.26	**N:S327L**	2	0.3	**N:A119S**	5	0.14
Maharashtra	**N:S26I**	1	0.15	**N:T24I**	2	0.26	**N:L139F**	2	0.3	**N:R32H**	5	0.14
Maharashtra	**N:G243C**	1	0.15	**N:H300Y**	2	0.26	**N:A152S**	2	0.3	**N:Q409L**	5	0.14
Maharashtra	**N:P207L**	1	0.15	**N:S193I**	2	0.26	**N:K373R**	2	0.3	**N:T379I**	5	0.14
Maharashtra	**N:T296I**	1	0.15	**N:A252S**	2	0.26	**N:K374R**	2	0.3	**N:D144Y**	5	0.14
Maharashtra	**N:D3Y**	1	0.15	**N:T379I**	1	0.13	**N:K375R**	2	0.3	**N:G129D**	5	0.14
Maharashtra	**N:M210I**	1	0.15	**N:P20H**	1	0.13	**N:D399Y**	2	0.3	**N:P162C**	5	0.14
Maharashtra	**N:Q28L**	1	0.15	**N:Q303H**	1	0.13	**N:P6L**	2	0.3	**N:S413I**	5	0.14
Maharashtra	**N:S183F**	1	0.15	**N:P365S**	1	0.13	**N:P13T**	2	0.3	**N:T24I**	5	0.14
Maharashtra	**N:P383L**	1	0.15	**N:T247I**	1	0.13	**N:I94V**	1	0.15	**N:G30A**	5	0.14
Maharashtra	**N:D343H**	1	0.15	**N:P13T**	1	0.13	**N:E118Q**	1	0.15	**N:N269V**	4	0.11
Maharashtra	**N:I320M**	1	0.15	**N:P344S**	1	0.13	**N:R32S**	1	0.15	**N:Q294N**	4	0.11
Maharashtra	**N:T141I**	1	0.15	**N:R95C**	1	0.13	**N:A398V**	1	0.15	**N:A381S**	4	0.11

Maharashtra	**N:R203M**	1	0.15	**N:G214V**	1	0.13	**N:V270L**	1	0.15	**N:T417A**	4	0.11
Maharashtra	**N:P344S**	1	0.15	**N:N48S**	1	0.13	**N:R191H**	1	0.15	**N:A90S**	4	0.11
Maharashtra	**N:K375N**	1	0.15	**N:A35V**	1	0.13	**N:S26K**	1	0.15	**N:R88K**	4	0.11
Maharashtra	**N:P383S**	1	0.15	**N:R40L**	1	0.13	**N:N27T**	1	0.15	**N:R89L**	4	0.11
Maharashtra	**N:S197***	1	0.15	**N:A12S**	1	0.13	**N:N29S**	1	0.15	**N:G116T**	4	0.11
Maharashtra	**N:L221F**	1	0.15	**N:Q229H**	1	0.13	**N:T329L**	1	0.15	**N:P117***	4	0.11
Maharashtra	**N:D402E**	1	0.15	**N:I320M**	1	0.13	**N:Q70R**	1	0.15	**N:A119L**	4	0.11
Maharashtra	**N:F403D**	1	0.15	**N:S232I**	1	0.13	**N:P199L**	1	0.15	**N:N126Q**	4	0.11
Maharashtra	**N:T135I**	1	0.15	**N:E378Q**	1	0.13	**N:P162S**	1	0.15	**N:D128L**	4	0.11
Maharashtra	**N:R209I**	1	0.15	**N:R195I**	1	0.13	**N:W330L**	1	0.15	**N:I130F**	4	0.11
Maharashtra	**N:A156S**	1	0.15	**N:S188P**	1	0.13	**N:T271I**	1	0.15	**N:I131P**	4	0.11
Maharashtra	**N:Q389H**	1	0.15	**N:S193N**	1	0.13	**N:S413I**	1	0.15	**N:W132M**	4	0.11
Maharashtra	**N:R185C**	1	0.15	**N:G204L**	1	0.13	**N:Q384H**	1	0.15	**N:A134L**	4	0.11
Maharashtra	**N:R32C**	1	0.15	**N:S232R**	1	0.13	**N:I320L**	1	0.15	**N:E136K**	4	0.11
Maharashtra	**N:D377Y**	1	0.15	**N:E280G**	1	0.13	**N:Y333C**	1	0.15	**N:G137T**	4	0.11
Maharashtra	**N:W330L**	1	0.15	**N:Q281T**	1	0.13	**N:G335I**	1	0.15	**N:N154A**	4	0.11
Maharashtra	**N:K373N**	1	0.15	**N:T282N**	1	0.13	**N:D371E**	1	0.15	**N:A155P**	4	0.11
Maharashtra	**N:F403C**	1	0.15	**N:G284T**	1	0.13	**N:D144G**	1	0.15	**N:V158L**	4	0.11
Maharashtra	**N:R177K**	1	0.15	**N:N285R**	1	0.13	**N:G200D**	1	0.15	**N:L161H**	4	0.11
Maharashtra	**N:T16M**	1	0.15	**N:F286N**	1	0.13	**N:Y360F**	1	0.15	**N:G164K**	4	0.11
Maharashtra				**N:G287***	1	0.13	**N:D402Y**	1	0.15	**N:T165A**	4	0.11
Maharashtra				**N:D288L**	1	0.13	**N:Q294K**	1	0.15	**N:T166S**	4	0.11
Maharashtra				**N:E290T**	1	0.13	**N:P279L**	1	0.15	**N:L167T**	4	0.11
Maharashtra				**N:I292A**	1	0.13	**N:A173V**	1	0.15	**N:F171A**	4	0.11
Maharashtra				**N:R293A**	1	0.13	**N:E378Q**	1	0.15	**N:Y172E**	4	0.11
Maharashtra				**N:Q294N**	1	0.13	**N:Q281K**	1	0.15	**N:R189N**	4	0.11
Maharashtra				**N:G295C**	1	0.13	**N:T271A**	1	0.15	**N:K372Q**	4	0.11
Maharashtra				**N:H300I**	1	0.13	**N:P258S**	1	0.15	**N:A308S**	4	0.11
Maharashtra				**N:W301C**	1	0.13	**N:D343I**	1	0.15	**N:G25C**	4	0.11
Maharashtra				**N:I320H**	1	0.13	**N:A119P**	1	0.15	**N:G212V**	4	0.11
Maharashtra				**N:G97S**	1	0.13	**N:A119T**	1	0.15	**N:G96C**	4	0.11
Maharashtra				**N:D98Q**	1	0.13	**N:S193V**	1	0.15	**N:A12S**	4	0.11
Maharashtra				**N:G99S**	1	0.13	**N:G69E**	1	0.15	**N:A156S**	4	0.11
Maharashtra				**N:K102V**	1	0.13	**N:Q70K**	1	0.15	**N:H300Y**	4	0.11
Maharashtra				**N:D103F**	1	0.13	**N:D343H**	1	0.15	**N:R191H**	4	0.11
Maharashtra				**N:Y123M**	1	0.13	**N:H300Y**	1	0.15	**N:A311T**	4	0.11
Maharashtra				**N:A125C**	1	0.13	**N:Q9H**	1	0.15	**N:D216A**	4	0.11
Maharashtra				**N:E136***	1	0.13	**N:R32C**	1	0.15	**N:P368A**	4	0.11
Maharashtra				**N:A138S**	1	0.13	**N:P142S**	1	0.15	**N:P279S**	4	0.11
Maharashtra				**N:N140E**	1	0.13	**N:R195I**	1	0.15	**N:A173V**	4	0.11
Maharashtra				**N:T141Y**	1	0.13	**N:T135N**	1	0.15	**N:P365S**	4	0.11
Maharashtra				**N:P142T**	1	0.13	**N:P122L**	1	0.15	**N:S197P**	4	0.11
Maharashtra				**N:P151R**	1	0.13	**N:I320C**	1	0.15	**N:R226K**	4	0.11
Maharashtra				**N:L167S**	1	0.13	**N:I320H**	1	0.15	**N:T263S**	3	0.08
Maharashtra				**N:P168S**	1	0.13	**N:V270I**	1	0.15	**N:A264L**	3	0.08
Maharashtra				**N:K169R**	1	0.13	**N:A155V**	1	0.15	**N:T265G**	3	0.08
Maharashtra				**N:G170N**	1	0.13	**N:*420Q**	1	0.15	**N:G284R**	3	0.08
Maharashtra				**N:F171N**	1	0.13	**N:S202N**	1	0.15	**N:F286***	3	0.08
Maharashtra				**N:Y172I**	1	0.13	**N:T325I**	1	0.15	**N:G287S**	3	0.08
Maharashtra				**N:G175Q**	1	0.13	**N:P151L**	1	0.15	**N:Q289K**	3	0.08
Maharashtra				**N:S176R**	1	0.13	**N:G215C**	1	0.15	**N:R293T**	3	0.08
Maharashtra				**N:R185Q**	1	0.13	**N:A419S**	1	0.15	**N:G295I**	3	0.08
Maharashtra				**N:S188L**	1	0.13	**N:A35S**	1	0.15	**N:T296G**	3	0.08
Maharashtra				**N:R189F**	1	0.13	**N:Q39L**	1	0.15	**N:D297R**	3	0.08
Maharashtra				**N:R191F**	1	0.13	**N:D22G**	1	0.15	**N:Y298K**	3	0.08
Maharashtra				**N:S194R**	1	0.13	**N:G30R**	1	0.15	**N:K299L**	3	0.08
Maharashtra				**N:R195Q**	1	0.13	**N:M411I**	1	0.15	**N:W301N**	3	0.08
Maharashtra				**N:N196Q**	1	0.13	**N:G18S**	1	0.15	**N:P302L**	3	0.08
Maharashtra				**N:S197***	1	0.13	**N:A217S**	1	0.15	**N:Q303P**	3	0.08
Maharashtra				**N:P199N**	1	0.13	**N:E367Q**	1	0.15	**N:I304P**	3	0.08
Maharashtra				**N:G200F**	1	0.13	**N:T334I**	1	0.15	**N:Q306L**	3	0.08
Maharashtra				**N:A211C**	1	0.13	**N:A376T**	1	0.15	**N:F307Q**	3	0.08
Maharashtra				**N:G212***	1	0.13	**N:A90S**	1	0.15	**N:A308R**	3	0.08
Maharashtra				**N:G215F**	1	0.13	**N:R203S**	1	0.15	**N:P309S**	3	0.08
Maharashtra				**N:D216A**	1	0.13	**N:M234V**	1	0.15	**N:T271L**	3	0.08
Maharashtra				**N:N228***	1	0.13	**N:Q389H**	1	0.15	**N:L221F**	3	0.08
Maharashtra				**N:L230I**	1	0.13	**N:K373N**	1	0.15	**N:R195I**	3	0.08
Maharashtra				**N:V246T**	1	0.13				**N:A12D**	3	0.08
Maharashtra				**N:K248C**	1	0.13				**N:R14Q**	3	0.08
Maharashtra				**N:K249***	1	0.13				**N:K370R**	3	0.08
Maharashtra				**N:S250G**	1	0.13				**N:G204V**	3	0.08
Maharashtra				**N:A251F**	1	0.13				**N:Q389L**	3	0.08
Maharashtra				**N:A252***	1	0.13				**N:D402Y**	3	0.08

Maharashtra				**N:K256A**	1	0.13				**N:Q9H**	3	0.08
Maharashtra				**N:A273T**	1	0.13				**N:P396S**	3	0.08
Maharashtra				**N:F274W**	1	0.13				**N:D348Y**	3	0.08
Maharashtra				**N:G275S**	1	0.13				**N:R95S**	3	0.08
Maharashtra				**N:R277T**	1	0.13				**N:M210Q**	3	0.08
Maharashtra				**N:G278N**	1	0.13				**N:D225L**	3	0.08
Maharashtra				**N:G284R**	1	0.13				**N:L227A**	3	0.08
Maharashtra				**N:N11S**	1	0.13				**N:N228K**	3	0.08
Maharashtra				**N:D341Y**	1	0.13				**N:Q229C**	3	0.08
Maharashtra				**N:P326R**	1	0.13				**N:S232K**	3	0.08
Maharashtra				**N:P151L**	1	0.13				**N:F286N**	3	0.08
Maharashtra				**N:M234I**	1	0.13				**N:G287***	3	0.08
Maharashtra				**N:L167F**	1	0.13				**N:D288L**	3	0.08
Maharashtra				**N:G212V**	1	0.13				**N:E290T**	3	0.08
Maharashtra				**N:A173V**	1	0.13				**N:P365T**	3	0.08
Maharashtra				**N:S327L**	1	0.13				**N:G215F**	3	0.08
Maharashtra				**N:K375N**	1	0.13				**N:N228***	3	0.08
Maharashtra				**N:M210V**	1	0.13				**N:L230I**	3	0.08
Maharashtra				**N:I337M**	1	0.13				**N:K375R**	3	0.08
Maharashtra				**N:A152T**	1	0.13				**N:P6L**	3	0.08
Maharashtra				**N:G30R**	1	0.13				**N:Q28H**	3	0.08
Maharashtra				**N:A218T**	1	0.13				**N:L230F**	3	0.08
Maharashtra				**N:S183T**	1	0.13				**N:P168S**	3	0.08
Maharashtra				**N:A308S**	1	0.13				**N:R14C**	3	0.08
Maharashtra				**N:D343N**	1	0.13				**N:P13T**	3	0.08
Maharashtra				**N:A119S**	1	0.13				**N:S2M**	3	0.08
Maharashtra				**N:T334I**	1	0.13				**N:D348H**	3	0.08
Maharashtra				**N:S193T**	1	0.13				**N:D371V**	3	0.08
Maharashtra				**N:A125S**	1	0.13				**N:P46S**	3	0.08
Maharashtra				**N:E323K**	1	0.13				**N:K256L**	2	0.06
Maharashtra				**N:F307I**	1	0.13				**N:K257L**	2	0.06
Maharashtra				**N:P80R**	1	0.13				**N:P258L**	2	0.06
Maharashtra				**N:S2F**	1	0.13				**N:Q260L**	2	0.06
Maharashtra										**N:K261L**	2	0.06
Maharashtra										**N:Y268D**	2	0.06
Maharashtra										**N:V350S**	2	0.06
Maharashtra										**N:V392L**	2	0.06
Maharashtra										**N:P162L**	2	0.06
Maharashtra										**N:M322R**	2	0.06
Maharashtra										**N:S193G**	2	0.06
Maharashtra										**N:Y268C**	2	0.06
Maharashtra										**N:V270T**	2	0.06
Maharashtra										**N:Q272Y**	2	0.06
Maharashtra										**N:F274L**	2	0.06
Maharashtra										**N:G18V**	2	0.06
Maharashtra										**N:Q239K**	2	0.06
Maharashtra										**N:R10P**	2	0.06
Maharashtra										**N:R40C**	2	0.06
Maharashtra										**N:G243S**	2	0.06
Maharashtra										**N:Q83R**	2	0.06
Maharashtra										**N:G321C**	2	0.06
Maharashtra										**N:A35S**	2	0.06
Maharashtra										**N:M1I**	2	0.06
Maharashtra										**N:D3E**	2	0.06
Maharashtra										**N:N4M**	2	0.06
Maharashtra										**N:G5H**	2	0.06
Maharashtra										**N:Q7A**	2	0.06
Maharashtra										**N:N8L**	2	0.06
Maharashtra										**N:Q9R**	2	0.06
Maharashtra										**N:R10L**	2	0.06
Maharashtra										**N:N11V**	2	0.06
Maharashtra										**N:N29V**	2	0.06
Maharashtra										**N:P42D**	2	0.06
Maharashtra										**N:L45S**	2	0.06
Maharashtra										**N:Q58L**	2	0.06
Maharashtra										**N:H59N**	2	0.06
Maharashtra										**N:G60S**	2	0.06
Maharashtra										**N:K61L**	2	0.06
Maharashtra										**N:L64K**	2	0.06
Maharashtra										**N:K65A**	2	0.06
Maharashtra										**N:G71L**	2	0.06
Maharashtra										**N:V72T**	2	0.06
Maharashtra										**N:N75M**	2	0.06

Maharashtra										**N:M101***	2	0.06
Maharashtra										**N:D103G**	2	0.06
Maharashtra										**N:L104I**	2	0.06
Maharashtra										**N:S201Q**	2	0.06
Maharashtra										**N:S202Q**	2	0.06
Maharashtra										**N:R203***	2	0.06
Maharashtra										**N:T282I**	2	0.06
Maharashtra										**N:E367D**	2	0.06
Maharashtra										**N:P368R**	2	0.06
Maharashtra										**N:Q281L**	2	0.06
Maharashtra										**N:A211S**	2	0.06
Maharashtra										**N:D63Y**	2	0.06
Maharashtra										**N:S21L**	2	0.06
Maharashtra										**N:R41L**	2	0.06
Maharashtra										**N:E378V**	2	0.06
Maharashtra										**N:R36L**	2	0.06
Maharashtra										**N:P365L**	2	0.06
Maharashtra										**N:G214D**	2	0.06
Maharashtra										**N:A376V**	2	0.06
Maharashtra										**N:R92***	2	0.06
Maharashtra										**N:R93K**	2	0.06
Maharashtra										**N:G96V**	2	0.06
Maharashtra										**N:G97Q**	2	0.06
Maharashtra										**N:L113I**	2	0.06
Maharashtra										**N:G114S**	2	0.06
Maharashtra										**N:L219V**	2	0.06
Maharashtra										**N:A220M**	2	0.06
Maharashtra										**N:E231V**	2	0.06
Maharashtra										**N:K233A**	2	0.06
Maharashtra										**N:M234N**	2	0.06
Maharashtra										**N:S235N**	2	0.06
Maharashtra										**N:G236N**	2	0.06
Maharashtra										**N:E253K**	2	0.06
Maharashtra										**N:A254L**	2	0.06
Maharashtra										**N:I320F**	2	0.06
Maharashtra										**N:M322N**	2	0.06
Maharashtra										**N:E323V**	2	0.06
Maharashtra										**N:T366N**	2	0.06
Maharashtra										**N:E367R**	2	0.06
Maharashtra										**N:K369***	2	0.06
Maharashtra										**N:D371G**	2	0.06
Maharashtra										**N:K374E**	2	0.06
Maharashtra										**N:K375E**	2	0.06
Maharashtra										**N:A376G**	2	0.06
Maharashtra										**N:A267S**	2	0.06
Maharashtra										**N:S186Q**	2	0.06
Maharashtra										**N:R209Q**	2	0.06
Maharashtra										**N:A211***	2	0.06
Maharashtra										**N:G214C**	2	0.06
Maharashtra										**N:V246T**	2	0.06
Maharashtra										**N:R277W**	2	0.06
Maharashtra										**N:E280G**	2	0.06
Maharashtra										**N:Q281T**	2	0.06
Maharashtra										**N:T282N**	2	0.06
Maharashtra										**N:G284T**	2	0.06
Maharashtra										**N:N285R**	2	0.06
Maharashtra										**N:E367Q**	2	0.06
Maharashtra										**N:Q7K**	2	0.06
Maharashtra										**N:S37P**	2	0.06
Maharashtra										**N:R191C**	2	0.06
Maharashtra										**N:V270L**	2	0.06
Maharashtra										**N:A419S**	2	0.06
Maharashtra										**N:R195T**	2	0.06
Maharashtra										**N:A252S**	2	0.06
Maharashtra										**N:E367A**	2	0.06
Maharashtra										**N:S202T**	2	0.06
Maharashtra										**N:Q70***	2	0.06
Maharashtra										**N:V72L**	2	0.06
Maharashtra										**N:S206P**	2	0.06
Maharashtra										**N:A90T**	2	0.06
Maharashtra										**N:N345Y**	2	0.06
Maharashtra										**N:D348I**	2	0.06
Maharashtra										**N:Q380S**	2	0.06

Maharashtra										**N:L331F**	2	0.06
Maharashtra										**N:M411Q**	2	0.06
Maharashtra										**N:P326S**	2	0.06
Maharashtra										**N:D63R**	2	0.06
Maharashtra										**N:G214V**	2	0.06
Maharashtra										**N:D128Y**	2	0.06
Maharashtra										**N:A134V**	2	0.06
Maharashtra										**N:S416L**	2	0.06
Maharashtra										**N:A414S**	2	0.06
Maharashtra										**N:Q43K**	2	0.06
Maharashtra										**N:P368Q**	2	0.06
Maharashtra										**N:A254R**	1	0.03
Maharashtra										**N:S255N**	1	0.03
Maharashtra										**N:V324Q**	1	0.03
Maharashtra										**N:T325V**	1	0.03
Maharashtra										**N:K347C**	1	0.03
Maharashtra										**N:D348***	1	0.03
Maharashtra										**N:Q349I**	1	0.03
Maharashtra										**N:E367H**	1	0.03
Maharashtra										**N:P368T**	1	0.03
Maharashtra										**N:E253D**	1	0.03
Maharashtra										**N:D371N**	1	0.03
Maharashtra										**N:S318K**	1	0.03
Maharashtra										**N:R319S**	1	0.03
Maharashtra										**N:I320H**	1	0.03
Maharashtra										**N:G321L**	1	0.03
Maharashtra										**N:W330R**	1	0.03
Maharashtra										**N:L331G**	1	0.03
Maharashtra										**N:T332***	1	0.03
Maharashtra										**N:Y333P**	1	0.03
Maharashtra										**N:G335Q**	1	0.03
Maharashtra										**N:A336V**	1	0.03
Maharashtra										**N:I337P**	1	0.03
Maharashtra										**N:K338Q**	1	0.03
Maharashtra										**N:G44V**	1	0.03
Maharashtra										**N:W330L**	1	0.03
Maharashtra										**N:L339F**	1	0.03
Maharashtra										**N:S255P**	1	0.03
Maharashtra										**N:D81Y**	1	0.03
Maharashtra										**N:P142S**	1	0.03
Maharashtra										**N:G204L**	1	0.03
Maharashtra										**N:G214S**	1	0.03
Maharashtra										**N:D22Y**	1	0.03
Maharashtra										**N:A55S**	1	0.03
Maharashtra										**N:Q229P**	1	0.03
Maharashtra										**N:F66L**	1	0.03
Maharashtra										**N:A90N**	1	0.03
Maharashtra										**N:S105G**	1	0.03
Maharashtra										**N:P106L**	1	0.03
Maharashtra										**N:W108S**	1	0.03
Maharashtra										**N:Y109F**	1	0.03
Maharashtra										**N:F110I**	1	0.03
Maharashtra										**N:L400F**	1	0.03
Maharashtra										**N:S202I**	1	0.03
Maharashtra										**N:D63N**	1	0.03
Maharashtra										**N:H300R**	1	0.03
Maharashtra										**N:P6S**	1	0.03
Maharashtra										**N:P326L**	1	0.03
Maharashtra										**N:G179V**	1	0.03
Maharashtra										**N:Q406H**	1	0.03
Maharashtra										**N:K369Q**	1	0.03
Maharashtra										**N:D377N**	1	0.03
Maharashtra										**N:D341P**	1	0.03
Maharashtra										**N:T247S**	1	0.03
Maharashtra										**N:T57I**	1	0.03
Maharashtra										**N:Q58R**	1	0.03
Maharashtra										**N:A119V**	1	0.03
Maharashtra										**N:W52P**	1	0.03
Maharashtra										**N:D348V**	1	0.03
Maharashtra										**N:Q349H**	1	0.03
Maharashtra										**N:T362H**	1	0.03
Maharashtra										**N:F363S**	1	0.03
Maharashtra										**N:P364H**	1	0.03

Maharashtra										**N:P365Q**	1	0.03
Maharashtra										**N:T366Q**	1	0.03
Maharashtra										**N:E367S**	1	0.03
Maharashtra										**N:P368L**	1	0.03
Maharashtra										**N:K372A**	1	0.03
Maharashtra										**N:K374Q**	1	0.03
Maharashtra										**N:Q349R**	1	0.03
Maharashtra										**N:Q241R**	1	0.03
Maharashtra										**N:V392T**	1	0.03
Maharashtra										**N:T393R**	1	0.03
Maharashtra										**N:L394I**	1	0.03
Maharashtra										**N:L395H**	1	0.03
Maharashtra										**N:A398Q**	1	0.03
Maharashtra										**N:N154S**	1	0.03
Maharashtra										**N:T391I**	1	0.03
Maharashtra										**N:E323R**	1	0.03
Maharashtra										**N:P326V**	1	0.03
Maharashtra										**N:V350L**	1	0.03
Maharashtra										**N:I351F**	1	0.03
Maharashtra										**N:N354S**	1	0.03
Maharashtra										**N:K355L**	1	0.03
Maharashtra										**N:K374G**	1	0.03
Maharashtra										**N:D377***	1	0.03
Maharashtra										**N:T379S**	1	0.03
Maharashtra										**N:Q380***	1	0.03
Maharashtra										**N:A218S**	1	0.03
Maharashtra										**N:K369T**	1	0.03
Maharashtra										**N:N8D**	1	0.03
Maharashtra										**N:G212A**	1	0.03
Maharashtra										**N:N213V**	1	0.03
Maharashtra										**N:G215L**	1	0.03
Maharashtra										**N:A217M**	1	0.03
Maharashtra										**N:T271V**	1	0.03
Maharashtra										**N:S312A**	1	0.03
Maharashtra										**N:F314W**	1	0.03
Maharashtra										**N:F315H**	1	0.03
Maharashtra										**N:M317S**	1	0.03
Maharashtra										**N:S318H**	1	0.03
Maharashtra										**N:R319T**	1	0.03
Maharashtra										**N:Q163K**	1	0.03
Maharashtra										**N:G164E**	1	0.03
Maharashtra										**N:T165Q**	1	0.03
Maharashtra										**N:T166H**	1	0.03
Maharashtra										**N:L167C**	1	0.03
Maharashtra										**N:G170V**	1	0.03
Maharashtra										**N:F171L**	1	0.03
Maharashtra										**N:Y172R**	1	0.03
Maharashtra										**N:A173R**	1	0.03
Maharashtra										**N:E174R**	1	0.03
Maharashtra										**N:G175E**	1	0.03
Maharashtra										**N:S176Q**	1	0.03
Maharashtra										**N:K248E**	1	0.03
Maharashtra										**N:K249A**	1	0.03
Maharashtra										**N:A267T**	1	0.03
Maharashtra										**N:N269S**	1	0.03
Maharashtra										**N:V270R**	1	0.03
Maharashtra										**N:Q272N**	1	0.03
Maharashtra										**N:A273P**	1	0.03
Maharashtra										**N:F274R**	1	0.03
Maharashtra										**N:G275K**	1	0.03
Maharashtra										**N:R276F**	1	0.03
Maharashtra										**N:I292A**	1	0.03
Maharashtra										**N:R293A**	1	0.03
Maharashtra										**N:G295C**	1	0.03
Maharashtra										**N:H300I**	1	0.03
Maharashtra										**N:W301C**	1	0.03
Maharashtra										**N:K374N**	1	0.03
Maharashtra										**N:D377E**	1	0.03
Maharashtra										**N:D399N**	1	0.03
Maharashtra										**N:L400C**	1	0.03
Maharashtra										**N:D402R**	1	0.03
Maharashtra										**N:S404G**	1	0.03
Maharashtra										**N:K405***	1	0.03

Maharashtra										**N:Q406F**	1	0.03
Maharashtra										**N:Q409T**	1	0.03
Maharashtra										**N:S410I**	1	0.03
Maharashtra										**N:R203S**	1	0.03
Maharashtra										**N:S250F**	1	0.03
Maharashtra										**N:Q163R**	1	0.03
Maharashtra										**N:G164R**	1	0.03
Maharashtra										**N:Q244K**	1	0.03
Maharashtra										**N:T245S**	1	0.03
Maharashtra										**N:V246S**	1	0.03
Maharashtra										**N:E118R**	1	0.03
Maharashtra										**N:A119G**	1	0.03
Maharashtra										**N:E231K**	1	0.03
Maharashtra										**N:K233S**	1	0.03
Maharashtra										**N:S318A**	1	0.03
Maharashtra										**N:S187G**	1	0.03
Maharashtra										**N:R189L**	1	0.03
Maharashtra										**N:S193P**	1	0.03
Maharashtra										**N:S194Y**	1	0.03
Maharashtra										**N:R195D**	1	0.03
Maharashtra										**N:M322T**	1	0.03
Maharashtra										**N:L395S**	1	0.03
Maharashtra										**N:L395F**	1	0.03
Maharashtra										**N:A397V**	1	0.03
Maharashtra										**N:D402V**	1	0.03
Maharashtra										**N:A419V**	1	0.03
Maharashtra										**N:A208S**	1	0.03
Maharashtra										**N:H145Y**	1	0.03
Maharashtra										**N:A155S**	1	0.03
Maharashtra										**N:S190I**	1	0.03
Maharashtra										**N:P368S**	1	0.03
Maharashtra										**N:S410F**	1	0.03
Maharashtra										**N:R191L**	1	0.03
Maharashtra										**N:A173S**	1	0.03
Maharashtra										**N:A381D**	1	0.03
Maharashtra										**N:Q389K**	1	0.03
Maharashtra										**N:Q386R**	1	0.03
Maharashtra										**N:K387R**	1	0.03
Maharashtra										**N:T91A**	1	0.03
Maharashtra										**N:R92M**	1	0.03
Maharashtra										**N:G284A**	1	0.03
Maharashtra										**N:N285D**	1	0.03
Maharashtra										**N:D297***	1	0.03
Maharashtra										**N:Y298***	1	0.03
Maharashtra										**N:K299Q**	1	0.03
Maharashtra										**N:H300F**	1	0.03
Maharashtra										**N:K373N**	1	0.03
Maharashtra										**N:V350I**	1	0.03
Maharashtra										**N:Q244R**	1	0.03
Maharashtra										**N:D401Y**	1	0.03
Maharashtra										**N:G164L**	1	0.03
Maharashtra										**N:P162R**	1	0.03
Maharashtra										**N:G164P**	1	0.03
Maharashtra										**N:T166L**	1	0.03
Maharashtra										**N:R203I**	1	0.03
Maharashtra										**N:D343H**	1	0.03
Maharashtra										**N:N213S**	1	0.03
Maharashtra										**N:S183F**	1	0.03
Maharashtra										**N:S206F**	1	0.03
Maharashtra										**N:N196Q**	1	0.03
Maharashtra										**N:S197Q**	1	0.03
Maharashtra										**N:T198***	1	0.03
Maharashtra										**N:P199N**	1	0.03
Maharashtra										**N:G200F**	1	0.03
Maharashtra										**N:A211C**	1	0.03
Maharashtra										**N:G212***	1	0.03
Maharashtra										**N:S206A**	1	0.03
Maharashtra										**N:P207M**	1	0.03
Maharashtra										**N:R209V**	1	0.03
Maharashtra										**N:T247V**	1	0.03
Maharashtra										**N:K249L**	1	0.03
Maharashtra										**N:A251F**	1	0.03
Maharashtra										**N:A252***	1	0.03

Maharashtra										**N:K256A**	1	0.03
Maharashtra										**N:A273T**	1	0.03
Maharashtra										**N:F274W**	1	0.03
Maharashtra										**N:G275S**	1	0.03
Maharashtra										**N:A313H**	1	0.03
Maharashtra										**N:F314T**	1	0.03
Maharashtra										**N:K338N**	1	0.03
Maharashtra										**N:L339V**	1	0.03
Maharashtra										**N:D340V**	1	0.03
Maharashtra										**N:K342E**	1	0.03
Maharashtra										**N:D343***	1	0.03
Maharashtra										**N:P344A**	1	0.03
Maharashtra										**N:F346***	1	0.03
Maharashtra										**N:K347R**	1	0.03
Maharashtra										**N:V350N**	1	0.03
Maharashtra										**N:T16Q**	1	0.03
Maharashtra										**N:F17W**	1	0.03
Maharashtra										**N:G18Q**	1	0.03
Maharashtra										**N:G19***	1	0.03
Maharashtra										**N:S21E**	1	0.03
Maharashtra										**N:D22W**	1	0.03
Maharashtra										**N:S23R**	1	0.03
Maharashtra										**N:G25Q**	1	0.03
Maharashtra										**N:S26W**	1	0.03
Maharashtra										**N:N27G**	1	0.03
Maharashtra										**N:Q28A**	1	0.03
Maharashtra										**N:N29I**	1	0.03
Maharashtra										**N:G30K**	1	0.03
Maharashtra										**N:E31T**	1	0.03
Maharashtra										**N:R32T**	1	0.03
Maharashtra										**N:S51P**	1	0.03
Maharashtra										**N:W52R**	1	0.03
Maharashtra										**N:A55H**	1	0.03
Maharashtra										**N:L56S**	1	0.03
Maharashtra										**N:G69W**	1	0.03
Maharashtra										**N:V72R**	1	0.03
Maharashtra										**N:I74P**	1	0.03
Maharashtra										**N:R93***	1	0.03
Maharashtra										**N:I94N**	1	0.03
Maharashtra										**N:R95E**	1	0.03
Maharashtra										**N:G96R**	1	0.03
Maharashtra										**N:G97S**	1	0.03
Maharashtra										**N:D98Q**	1	0.03
Maharashtra										**N:G99S**	1	0.03
Maharashtra										**N:K102V**	1	0.03
Maharashtra										**N:D103F**	1	0.03
Maharashtra										**N:Y123M**	1	0.03
Maharashtra										**N:A125C**	1	0.03
Maharashtra										**N:E136***	1	0.03
Maharashtra										**N:A138S**	1	0.03
Maharashtra										**N:N140E**	1	0.03
Maharashtra										**N:T141Y**	1	0.03
Maharashtra										**N:P142T**	1	0.03
Maharashtra										**N:P151R**	1	0.03
Maharashtra										**N:G178R**	1	0.03
Maharashtra										**N:G179R**	1	0.03
Maharashtra										**N:S180E**	1	0.03
Maharashtra										**N:S184R**	1	0.03
Maharashtra										**N:E378M**	1	0.03
Maharashtra										**N:T379K**	1	0.03
Maharashtra										**N:P383T**	1	0.03
Maharashtra										**N:Q384A**	1	0.03
Maharashtra										**N:G34V**	1	0.03
Maharashtra										**N:A35L**	1	0.03
Maharashtra										**N:S51C**	1	0.03
Maharashtra										**N:W52***	1	0.03
Maharashtra										**N:S26G**	1	0.03
Maharashtra										**N:D82C**	1	0.03
Maharashtra										**N:Q83H**	1	0.03
Maharashtra										**N:I84V**	1	0.03
Maharashtra										**N:G85E**	1	0.03
Maharashtra										**N:Y86***	1	0.03
Maharashtra										**N:Y87E**	1	0.03

Maharashtra										**N:R89***	1	0.03
Maharashtra										**N:I94S**	1	0.03
Maharashtra										**N:N192D**	1	0.03
Maharashtra										**N:S193L**	1	0.03
Maharashtra										**N:R195V**	1	0.03
Maharashtra										**N:N196Y**	1	0.03
Maharashtra										**N:T205S**	1	0.03
Maharashtra										**N:D341G**	1	0.03
Maharashtra										**N:P142L**	1	0.03
Maharashtra										**N:T334I**	1	0.03
Maharashtra										**N:I94V**	1	0.03
Maharashtra										**N:A12P**	1	0.03
Maharashtra										**N:E231N**	1	0.03
Maharashtra										**N:S232N**	1	0.03
Maharashtra										**N:P207Q**	1	0.03
Maharashtra										**N:R209A**	1	0.03
Maharashtra										**N:S312L**	1	0.03
Maharashtra										**N:L331M**	1	0.03
Maharashtra										**N:I351H**	1	0.03
Maharashtra										**N:L352F**	1	0.03
Maharashtra										**N:L353A**	1	0.03
Maharashtra										**N:N354E**	1	0.03
Maharashtra										**N:G129S**	1	0.03
Maharashtra										**N:D399Y**	1	0.03
Maharashtra										**N:Q229H**	1	0.03
Maharashtra										**N:A155V**	1	0.03
Maharashtra										**N:A313S**	1	0.03
Maharashtra										**N:S412G**	1	0.03
Maharashtra										**N:D371T**	1	0.03
Maharashtra										**N:T417N**	1	0.03
Maharashtra										**N:K369E**	1	0.03
Maharashtra										**N:D415G**	1	0.03
Maharashtra										**N:K373R**	1	0.03
Maharashtra										**N:K374R**	1	0.03
Maharashtra										**N:A376P**	1	0.03
Maharashtra										**N:Q384R**	1	0.03
Maharashtra										**N:Q409D**	1	0.03
Maharashtra										**N:Q28E**	1	0.03
Maharashtra										**N:N29S**	1	0.03
Maharashtra										**N:R32P**	1	0.03
Maharashtra										**N:P46R**	1	0.03
Maharashtra										**N:N47S**	1	0.03
Maharashtra										**N:N48I**	1	0.03
Maharashtra										**N:T49I**	1	0.03
Maharashtra										**N:A50G**	1	0.03
Maharashtra										**N:E62N**	1	0.03
Maharashtra										**N:Q244P**	1	0.03
Maharashtra										**N:A376T**	1	0.03
Maharashtra										**N:Q163***	1	0.03
Maharashtra										**N:T166A**	1	0.03
Maharashtra										**N:R385I**	1	0.03
Maharashtra										**N:Q306P**	1	0.03
Maharashtra										**N:F307I**	1	0.03
Maharashtra										**N:S188L**	1	0.03
Maharashtra										**N:N8Y**	1	0.03
Maharashtra										**N:R95C**	1	0.03
Maharashtra										**N:R92S**	1	0.03
Maharashtra										**N:G34W**	1	0.03
Maharashtra										**N:T24N**	1	0.03
Maharashtra										**N:G5A**	1	0.03
Maharashtra										**N:R14L**	1	0.03
Maharashtra										**N:A35V**	1	0.03
Maharashtra										**N:M101K**	1	0.03
Maharashtra										**N:D103S**	1	0.03
Maharashtra										**N:N154Y**	1	0.03
Maharashtra										**N:T325I**	1	0.03
Maharashtra										**N:P344L**	1	0.03
Maharashtra										**N:G34R**	1	0.03
Maharashtra										**N:P151S**	1	0.03
Maharashtra										**N:G124S**	1	0.03
Maharashtra										**N:Q294K**	1	0.03
Maharashtra										**N:G18C**	1	0.03
Maharashtra										**N:P383S**	1	0.03

Maharashtra										N:K370E	1	0.03
Maharashtra										N:S413E	1	0.03
Maharashtra										N:A414G	1	0.03
Maharashtra										N:A381T	1	0.03
Maharashtra										N:A398P	1	0.03
Maharashtra										N:F403R	1	0.03
Maharashtra										N:K387T	1	0.03
Maharashtra										N:K388T	1	0.03
Maharashtra										N:K388Q	1	0.03
Maharashtra										N:L394V	1	0.03
Maharashtra										N:L395T	1	0.03
Maharashtra										N:K370N	1	0.03
Maharashtra										N:D371S	1	0.03
Maharashtra										N:K373T	1	0.03
Maharashtra										N:T393I	1	0.03
**State/Gene**	**ORF3a**											
**Maharashtra**	**Pre-Wave 1**			**Wave 1**			**Pre-Wave 2**			**Wave 2**		
**Maharashtra**	**Mutation**	**Count**	**percent**	**Mutation**	**Count**	**percent**	**Mutation**	**Count**	**percent**	**Mutation**	**Count**	**percent**
**Maharashtra**	**ORF3a:Q57H**	30	18.99	**ORF3a:Q57H**	65	17.24	**ORF3a:Q57H**	205	19.16	**ORF3a:S26L**	2730	66.44
**Maharashtra**	**ORF3a:G100C**	22	13.92	**ORF3a:G224V**	23	6.1	**ORF3a:S26L**	195	18.22	**ORF3a:Q57H**	170	4.14
**Maharashtra**	**ORF3a:G254***	11	6.96	**ORF3a:L65F**	20	5.31	**ORF3a:E261***	124	11.59	**ORF3a:E261***	110	2.68
**Maharashtra**	**ORF3a:A23V**	6	3.8	**ORF3a:T223I**	17	4.51	**ORF3a:T223I**	50	4.67	**ORF3a:T223I**	53	1.29
**Maharashtra**	**ORF3a:I35T**	5	3.16	**ORF3a:E181K**	15	3.98	**ORF3a:G224C**	21	1.96	**ORF3a:G100D**	49	1.19
**Maharashtra**	**ORF3a:A54S**	4	2.53	**ORF3a:E261***	13	3.45	**ORF3a:R6D**	19	1.78	**ORF3a:L85F**	28	0.68
**Maharashtra**	**ORF3a:V55G**	4	2.53	**ORF3a:T89I**	11	2.92	**ORF3a:I7L**	19	1.78	**ORF3a:G172C**	26	0.63
**Maharashtra**	**ORF3a:G224V**	4	2.53	**ORF3a:N144Y**	8	2.12	**ORF3a:T9R**	19	1.78	**ORF3a:A23V**	24	0.58
**Maharashtra**	**ORF3a:W149L**	4	2.53	**ORF3a:A23S**	7	1.86	**ORF3a:G11F**	19	1.78	**ORF3a:S171L**	22	0.54
**Maharashtra**	**ORF3a:P240L**	3	1.9	**ORF3a:T268M**	6	1.59	**ORF3a:K21G**	19	1.78	**ORF3a:D155Y**	21	0.51
**Maharashtra**	**ORF3a:P159S**	3	1.9	**ORF3a:L106F**	6	1.59	**ORF3a:D22T**	19	1.78	**ORF3a:G224C**	20	0.49
**Maharashtra**	**ORF3a:K21N**	2	1.27	**ORF3a:A54S**	5	1.33	**ORF3a:A23V**	19	1.78	**ORF3a:A31D**	19	0.46
**Maharashtra**	**ORF3a:W131S**	2	1.27	**ORF3a:L275F**	5	1.33	**ORF3a:P25G**	19	1.78	**ORF3a:A33D**	19	0.46
**Maharashtra**	**ORF3a:D155Y**	2	1.27	**ORF3a:A72T**	5	1.33	**ORF3a:D27R**	19	1.78	**ORF3a:R6D**	18	0.44
**Maharashtra**	**ORF3a:V112F**	2	1.27	**ORF3a:L53F**	4	1.06	**ORF3a:F28Y**	19	1.78	**ORF3a:I7L**	18	0.44
**Maharashtra**	**ORF3a:L83F**	2	1.27	**ORF3a:L46F**	4	1.06	**ORF3a:V29C**	19	1.78	**ORF3a:T9R**	18	0.44
**Maharashtra**	**ORF3a:L85F**	2	1.27	**ORF3a:V50F**	4	1.06	**ORF3a:R30N**	19	1.78	**ORF3a:G11F**	18	0.44
**Maharashtra**	**ORF3a:W131C**	2	1.27	**ORF3a:G254***	4	1.06	**ORF3a:A31D**	19	1.78	**ORF3a:K21G**	18	0.44
**Maharashtra**	**ORF3a:L275F**	2	1.27	**ORF3a:V112I**	4	1.06	**ORF3a:A33D**	19	1.78	**ORF3a:D22T**	18	0.44
**Maharashtra**	**ORF3a:S171L**	2	1.27	**ORF3a:I20T**	4	1.06	**ORF3a:T151I**	9	0.84	**ORF3a:P25G**	18	0.44
**Maharashtra**	**ORF3a:W69C**	2	1.27	**ORF3a:A103S**	4	1.06	**ORF3a:G174V**	9	0.84	**ORF3a:D27R**	18	0.44
**Maharashtra**	**ORF3a:H78Y**	2	1.27	**ORF3a:W131C**	3	0.8	**ORF3a:L106F**	8	0.75	**ORF3a:F28Y**	18	0.44
**Maharashtra**	**ORF3a:A33S**	1	0.63	**ORF3a:D155Y**	3	0.8	**ORF3a:S171L**	8	0.75	**ORF3a:V29C**	18	0.44
**Maharashtra**	**ORF3a:V259L**	1	0.63	**ORF3a:Q185H**	3	0.8	**ORF3a:S216T**	6	0.56	**ORF3a:R30N**	18	0.44
**Maharashtra**	**ORF3a:A110V**	1	0.63	**ORF3a:V55G**	3	0.8	**ORF3a:G172V**	6	0.56	**ORF3a:D142G**	15	0.37
**Maharashtra**	**ORF3a:P240S**	1	0.63	**ORF3a:L73F**	3	0.8	**ORF3a:T268M**	6	0.56	**ORF3a:V202L**	14	0.34
**Maharashtra**	**ORF3a:S165F**	1	0.63	**ORF3a:A110V**	3	0.8	**ORF3a:D142G**	5	0.47	**ORF3a:W131C**	14	0.34
**Maharashtra**	**ORF3a:D222Y**	1	0.63	**ORF3a:L52F**	3	0.8	**ORF3a:D155Y**	5	0.47	**ORF3a:W45L**	13	0.32
**Maharashtra**	**ORF3a:V225F**	1	0.63	**ORF3a:L85F**	3	0.8	**ORF3a:L46F**	5	0.47	**ORF3a:D183Y**	13	0.32
**Maharashtra**	**ORF3a:L108F**	1	0.63	**ORF3a:L108F**	3	0.8	**ORF3a:V77F**	4	0.37	**ORF3a:A110S**	12	0.29
**Maharashtra**	**ORF3a:L108I**	1	0.63	**ORF3a:T151I**	3	0.8	**ORF3a:W149G**	4	0.37	**ORF3a:D27Y**	12	0.29
**Maharashtra**	**ORF3a:K16N**	1	0.63	**ORF3a:W69S**	3	0.8	**ORF3a:D238G**	4	0.37	**ORF3a:A54S**	9	0.22
**Maharashtra**	**ORF3a:L85V**	1	0.63	**ORF3a:D22Y**	3	0.8	**ORF3a:Q17L**	4	0.37	**ORF3a:K67N**	9	0.22
**Maharashtra**	**ORF3a:S26L**	1	0.63	**ORF3a:T221K**	3	0.8	**ORF3a:T175I**	4	0.37	**ORF3a:G174V**	9	0.22
**Maharashtra**	**ORF3a:L140F**	1	0.63	**ORF3a:G100C**	2	0.53	**ORF3a:G224V**	4	0.37	**ORF3a:D173G**	8	0.19
**Maharashtra**	**ORF3a:L95F**	1	0.63	**ORF3a:V202L**	2	0.53	**ORF3a:V202L**	3	0.28	**ORF3a:V225F**	7	0.17
**Maharashtra**	**ORF3a:T176I**	1	0.63	**ORF3a:D27Y**	2	0.53	**ORF3a:V237I**	3	0.28	**ORF3a:V13L**	7	0.17
**Maharashtra**	**ORF3a:Y184H**	1	0.63	**ORF3a:E226K**	2	0.53	**ORF3a:W131C**	3	0.28	**ORF3a:G18V**	7	0.17
**Maharashtra**	**ORF3a:G251V**	1	0.63	**ORF3a:D142H**	2	0.53	**ORF3a:V273L**	3	0.28	**ORF3a:L106F**	6	0.15
**Maharashtra**	**ORF3a:A103V**	1	0.63	**ORF3a:L140F**	2	0.53	**ORF3a:H78Y**	3	0.28	**ORF3a:L106R**	6	0.15
**Maharashtra**	**ORF3a:R30H**	1	0.63	**ORF3a:L41I**	2	0.53	**ORF3a:A33S**	3	0.28	**ORF3a:I37L**	6	0.15
**Maharashtra**	**ORF3a:S58I**	1	0.63	**ORF3a:H227Y**	2	0.53	**ORF3a:L85F**	3	0.28	**ORF3a:S180F**	6	0.15
**Maharashtra**	**ORF3a:L96F**	1	0.63	**ORF3a:P240L**	2	0.53	**ORF3a:V55G**	3	0.28	**ORF3a:L53F**	6	0.15
**Maharashtra**	**ORF3a:A23S**	1	0.63	**ORF3a:P159S**	2	0.53	**ORF3a:G100C**	3	0.28	**ORF3a:K136E**	6	0.15
**Maharashtra**	**ORF3a:A103S**	1	0.63	**ORF3a:R134H**	2	0.53	**ORF3a:A59S**	2	0.19	**ORF3a:P240S**	6	0.15
**Maharashtra**	**ORF3a:T151I**	1	0.63	**ORF3a:G172R**	2	0.53	**ORF3a:W69C**	2	0.19	**ORF3a:A23S**	6	0.15
**Maharashtra**	**ORF3a:A72T**	1	0.63	**ORF3a:F105S**	2	0.53	**ORF3a:A23S**	2	0.19	**ORF3a:M260I**	5	0.12
**Maharashtra**	**ORF3a:I37L**	1	0.63	**ORF3a:V121A**	2	0.53	**ORF3a:G18C**	2	0.19	**ORF3a:L52F**	5	0.12
**Maharashtra**	**ORF3a:G49V**	1	0.63	**ORF3a:G224C**	2	0.53	**ORF3a:G100D**	2	0.19	**ORF3a:P240L**	5	0.12
**Maharashtra**	**ORF3a:R134C**	1	0.63	**ORF3a:V77F**	2	0.53	**ORF3a:N82D**	2	0.19	**ORF3a:W131L**	5	0.12
**Maharashtra**	**ORF3a:M125I**	1	0.63	**ORF3a:I35T**	2	0.53	**ORF3a:E242K**	2	0.19	**ORF3a:V90F**	5	0.12
**Maharashtra**	**ORF3a:A72S**	1	0.63	**ORF3a:K21E**	2	0.53	**ORF3a:G254R**	2	0.19	**ORF3a:Q57R**	5	0.12
**Maharashtra**	**ORF3a:S209L**	1	0.63	**ORF3a:A23T**	2	0.53	**ORF3a:T32I**	2	0.19	**ORF3a:F43L**	5	0.12

Maharashtra	ORF3a:K75N	1	0.63	ORF3a:Y107H	2	0.53	ORF3a:G224R	2	0.19	ORF3a:L65F	5	0.12
Maharashtra	ORF3a:P42H	1	0.63	ORF3a:A143T	1	0.27	ORF3a:V77I	2	0.19	ORF3a:W69C	5	0.12
Maharashtra	ORF3a:V121L	1	0.63	ORF3a:T221A	1	0.27	ORF3a:Q185H	2	0.19	ORF3a:A103S	5	0.12
Maharashtra	ORF3a:Y107H	1	0.63	ORF3a:W149G	1	0.27	ORF3a:S74F	2	0.19	ORF3a:W193R	5	0.12
Maharashtra	ORF3a:Q57R	1	0.63	ORF3a:S216T	1	0.27	ORF3a:R134H	2	0.19	ORF3a:R68I	5	0.12
Maharashtra	ORF3a:W128L	1	0.63	ORF3a:G254R	1	0.27	ORF3a:T89I	2	0.19	ORF3a:T151I	4	0.1
Maharashtra	ORF3a:L275M	1	0.63	ORF3a:N257D	1	0.27	ORF3a:S166L	2	0.19	ORF3a:V77I	4	0.1
Maharashtra	ORF3a:V80F	1	0.63	ORF3a:R68I	1	0.27	ORF3a:L106R	1	0.09	ORF3a:T14I	4	0.1
Maharashtra	ORF3a:V112I	1	0.63	ORF3a:S40T	1	0.27	ORF3a:T170S	1	0.09	ORF3a:G254R	4	0.1
Maharashtra				ORF3a:L94F	1	0.27	ORF3a:L15F	1	0.09	ORF3a:Y184H	4	0.1
Maharashtra				ORF3a:Q57N	1	0.27	ORF3a:K67N	1	0.09	ORF3a:G224R	4	0.1
Maharashtra				ORF3a:V13I	1	0.27	ORF3a:P104L	1	0.09	ORF3a:W128L	4	0.1
Maharashtra				ORF3a:V256G	1	0.27	ORF3a:Y107H	1	0.09	ORF3a:G172V	4	0.1
Maharashtra				ORF3a:L41F	1	0.27	ORF3a:D173G	1	0.09	ORF3a:Q185H	4	0.1
Maharashtra				ORF3a:W193R	1	0.27	ORF3a:W45L	1	0.09	ORF3a:D27H	4	0.1
Maharashtra				ORF3a:H78Y	1	0.27	ORF3a:H78Q	1	0.09	ORF3a:L15F	3	0.07
Maharashtra				ORF3a:L95C	1	0.27	ORF3a:F79K	1	0.09	ORF3a:H227Y	3	0.07
Maharashtra				ORF3a:Y109L	1	0.27	ORF3a:Q245R	1	0.09	ORF3a:H78Y	3	0.07
Maharashtra				ORF3a:Y113K	1	0.27	ORF3a:S60L	1	0.09	ORF3a:G100C	3	0.07
Maharashtra				ORF3a:F114P	1	0.27	ORF3a:G76S	1	0.09	ORF3a:A99S	3	0.07
Maharashtra				ORF3a:Q116F	1	0.27	ORF3a:Y264H	1	0.09	ORF3a:S216T	3	0.07
Maharashtra				ORF3a:N119G	1	0.27	ORF3a:M260I	1	0.09	ORF3a:T175I	3	0.07
Maharashtra				ORF3a:S135A	1	0.27	ORF3a:S40L	1	0.09	ORF3a:K235I	3	0.07
Maharashtra				ORF3a:K136G	1	0.27	ORF3a:Q213L	1	0.09	ORF3a:V48F	3	0.07
Maharashtra				ORF3a:P138S	1	0.27	ORF3a:G172R	1	0.09	ORF3a:A110V	3	0.07
Maharashtra				ORF3a:A31T	1	0.27	ORF3a:Y184H	1	0.09	ORF3a:V201I	3	0.07
Maharashtra				ORF3a:L101F	1	0.27	ORF3a:T223F	1	0.09	ORF3a:L108F	3	0.07
Maharashtra				ORF3a:P42H	1	0.27	ORF3a:S180F	1	0.09	ORF3a:L140F	3	0.07
Maharashtra				ORF3a:S209L	1	0.27	ORF3a:D27Y	1	0.09	ORF3a:S40L	3	0.07
Maharashtra				ORF3a:G172S	1	0.27	ORF3a:E261Q	1	0.09	ORF3a:H182Y	3	0.07
Maharashtra				ORF3a:I179N	1	0.27	ORF3a:V13C	1	0.09	ORF3a:H182R	3	0.07
Maharashtra				ORF3a:S74F	1	0.27	ORF3a:T14N	1	0.09	ORF3a:T12A	3	0.07
Maharashtra				ORF3a:S166L	1	0.27	ORF3a:A99T	1	0.09	ORF3a:P42L	3	0.07
Maharashtra				ORF3a:D210N	1	0.27	ORF3a:I10T	1	0.09	ORF3a:A33S	3	0.07
Maharashtra				ORF3a:M1I	1	0.27	ORF3a:L65P	1	0.09	ORF3a:P262T	3	0.07
Maharashtra				ORF3a:P25L	1	0.27	ORF3a:Y141H	1	0.09	ORF3a:H93Y	3	0.07
Maharashtra				ORF3a:V97R	1	0.27	ORF3a:H93Y	1	0.09	ORF3a:V77F	3	0.07
Maharashtra				ORF3a:E239D	1	0.27	ORF3a:H93Q	1	0.09	ORF3a:V112F	3	0.07
Maharashtra				ORF3a:S171L	1	0.27	ORF3a:H182Y	1	0.09	ORF3a:L94F	3	0.07
Maharashtra				ORF3a:L83F	1	0.27	ORF3a:K67T	1	0.09	ORF3a:I47V	3	0.07
Maharashtra				ORF3a:T176I	1	0.27	ORF3a:A31T	1	0.09	ORF3a:G254*	3	0.07
Maharashtra				ORF3a:A72S	1	0.27	ORF3a:L101F	1	0.09	ORF3a:A39V	2	0.05
Maharashtra				ORF3a:L147F	1	0.27	ORF3a:T223N	1	0.09	ORF3a:S74F	2	0.05
Maharashtra				ORF3a:V88A	1	0.27	ORF3a:G11E	1	0.09	ORF3a:I20V	2	0.05
Maharashtra				ORF3a:A110S	1	0.27	ORF3a:T24I	1	0.09	ORF3a:S166L	2	0.05
Maharashtra				ORF3a:W149L	1	0.27	ORF3a:E239D	1	0.09	ORF3a:K66T	2	0.05
Maharashtra				ORF3a:Y141D	1	0.27	ORF3a:V202I	1	0.09	ORF3a:A54V	2	0.05
Maharashtra				ORF3a:V50I	1	0.27	ORF3a:V225F	1	0.09	ORF3a:G11R	2	0.05
Maharashtra				ORF3a:L73H	1	0.27	ORF3a:K61R	1	0.09	ORF3a:V88L	2	0.05
Maharashtra				ORF3a:T223N	1	0.27	ORF3a:G174C	1	0.09	ORF3a:M260N	2	0.05
Maharashtra				ORF3a:P262S	1	0.27	ORF3a:G172C	1	0.09	ORF3a:D222E	2	0.05
Maharashtra				ORF3a:I37V	1	0.27	ORF3a:A143S	1	0.09	ORF3a:I10S	2	0.05
Maharashtra				ORF3a:L15F	1	0.27	ORF3a:D173N	1	0.09	ORF3a:L94R	2	0.05
Maharashtra				ORF3a:T175I	1	0.27	ORF3a:A99S	1	0.09	ORF3a:A39S	2	0.05
Maharashtra				ORF3a:S26L	1	0.27	ORF3a:V90F	1	0.09	ORF3a:V273L	2	0.05
Maharashtra				ORF3a:D210Y	1	0.27	ORF3a:T12I	1	0.09	ORF3a:M1I	2	0.05
Maharashtra				ORF3a:A143S	1	0.27	ORF3a:T14I	1	0.09	ORF3a:E239D	2	0.05
Maharashtra				ORF3a:V112F	1	0.27	ORF3a:K136E	1	0.09	ORF3a:E102Q	2	0.05
Maharashtra				ORF3a:L106R	1	0.27	ORF3a:L83F	1	0.09	ORF3a:G174C	2	0.05
Maharashtra				ORF3a:P274L	1	0.27	ORF3a:A54S	1	0.09	ORF3a:K16N	2	0.05
Maharashtra				ORF3a:G254V	1	0.27	ORF3a:W128C	1	0.09	ORF3a:D173Y	2	0.05
Maharashtra				ORF3a:R30H	1	0.27	ORF3a:Q213K	1	0.09	ORF3a:A59T	2	0.05
Maharashtra				ORF3a:A99S	1	0.27	ORF3a:L41P	1	0.09	ORF3a:P25L	2	0.05
Maharashtra				ORF3a:Q17L	1	0.27	ORF3a:S195P	1	0.09	ORF3a:H182Q	2	0.05
Maharashtra				ORF3a:K16N	1	0.27	ORF3a:E194G	1	0.09	ORF3a:W69L	2	0.05
Maharashtra							ORF3a:D27A	1	0.09	ORF3a:T64I	2	0.05
Maharashtra										ORF3a:L101F	2	0.05
Maharashtra										ORF3a:V55F	2	0.05
Maharashtra										ORF3a:N152Y	2	0.05
Maharashtra										ORF3a:I37T	2	0.05
Maharashtra										ORF3a:I10T	2	0.05
Maharashtra										ORF3a:L41I	2	0.05
Maharashtra										ORF3a:V163L	2	0.05

**Maharashtra**										**ORF3a:K67R**	2	0.05
**Maharashtra**										**ORF3a:T32A**	2	0.05
**Maharashtra**										**ORF3a:T221A**	2	0.05
**Maharashtra**										**ORF3a:V55A**	2	0.05
**Maharashtra**										**ORF3a:R68K**	2	0.05
**Maharashtra**										**ORF3a:G224V**	2	0.05
**Maharashtra**										**ORF3a:V259L**	2	0.05
**Maharashtra**										**ORF3a:G188V**	1	0.02
**Maharashtra**										**ORF3a:N82K**	1	0.02
**Maharashtra**										**ORF3a:D199K**	1	0.02
**Maharashtra**										**ORF3a:C200T**	1	0.02
**Maharashtra**										**ORF3a:P267N**	1	0.02
**Maharashtra**										**ORF3a:L41P**	1	0.02
**Maharashtra**										**ORF3a:Q245H**	1	0.02
**Maharashtra**										**ORF3a:G172S**	1	0.02
**Maharashtra**										**ORF3a:K61L**	1	0.02
**Maharashtra**										**ORF3a:R134H**	1	0.02
**Maharashtra**										**ORF3a:S209A**	1	0.02
**Maharashtra**										**ORF3a:L108V**	1	0.02
**Maharashtra**										**ORF3a:V202A**	1	0.02
**Maharashtra**										**ORF3a:S253F**	1	0.02
**Maharashtra**										**ORF3a:D142N**	1	0.02
**Maharashtra**										**ORF3a:H243Y**	1	0.02
**Maharashtra**										**ORF3a:P104L**	1	0.02
**Maharashtra**										**ORF3a:A99V**	1	0.02
**Maharashtra**										**ORF3a:W45C**	1	0.02
**Maharashtra**										**ORF3a:W69D**	1	0.02
**Maharashtra**										**ORF3a:Q70G**	1	0.02
**Maharashtra**										**ORF3a:L71N**	1	0.02
**Maharashtra**										**ORF3a:A72***	1	0.02
**Maharashtra**										**ORF3a:L73H**	1	0.02
**Maharashtra**										**ORF3a:K75P**	1	0.02
**Maharashtra**										**ORF3a:G76R**	1	0.02
**Maharashtra**										**ORF3a:L84F**	1	0.02
**Maharashtra**										**ORF3a:L85T**	1	0.02
**Maharashtra**										**ORF3a:V88A**	1	0.02
**Maharashtra**										**ORF3a:V90C**	1	0.02
**Maharashtra**										**ORF3a:Y91C**	1	0.02
**Maharashtra**										**ORF3a:S92C**	1	0.02
**Maharashtra**										**ORF3a:H93C**	1	0.02
**Maharashtra**										**ORF3a:Y107A**	1	0.02
**Maharashtra**										**ORF3a:Y109K**	1	0.02
**Maharashtra**										**ORF3a:A110P**	1	0.02
**Maharashtra**										**ORF3a:L127***	1	0.02
**Maharashtra**										**ORF3a:W128T**	1	0.02
**Maharashtra**										**ORF3a:P159V**	1	0.02
**Maharashtra**										**ORF3a:Y160T**	1	0.02
**Maharashtra**										**ORF3a:N161T**	1	0.02
**Maharashtra**										**ORF3a:S162I**	1	0.02
**Maharashtra**										**ORF3a:T164Y**	1	0.02
**Maharashtra**										**ORF3a:S165L**	1	0.02
**Maharashtra**										**ORF3a:S166T**	1	0.02
**Maharashtra**										**ORF3a:I169Q**	1	0.02
**Maharashtra**										**ORF3a:T170Q**	1	0.02
**Maharashtra**										**ORF3a:G172Y**	1	0.02
**Maharashtra**										**ORF3a:D173F**	1	0.02
**Maharashtra**										**ORF3a:G174***	1	0.02
**Maharashtra**										**ORF3a:T176***	1	0.02
**Maharashtra**										**ORF3a:S177L**	1	0.02
**Maharashtra**										**ORF3a:K192D**	1	0.02
**Maharashtra**										**ORF3a:E194W**	1	0.02
**Maharashtra**										**ORF3a:S195L**	1	0.02
**Maharashtra**										**ORF3a:G196Y**	1	0.02
**Maharashtra**										**ORF3a:V197***	1	0.02
**Maharashtra**										**ORF3a:D199H**	1	0.02
**Maharashtra**										**ORF3a:C200F**	1	0.02
**Maharashtra**										**ORF3a:V201R**	1	0.02
**Maharashtra**										**ORF3a:H204P**	1	0.02
**Maharashtra**										**ORF3a:S205A**	1	0.02
**Maharashtra**										**ORF3a:Y206V**	1	0.02
**Maharashtra**										**ORF3a:F207L**	1	0.02
**Maharashtra**										**ORF3a:T208N**	1	0.02
**Maharashtra**										**ORF3a:D210I**	1	0.02

**Maharashtra**										**ORF3a:Y211E**	1	0.02
**Maharashtra**										**ORF3a:E226R**	1	0.02
**Maharashtra**										**ORF3a:V228W**	1	0.02
**Maharashtra**										**ORF3a:T229C**	1	0.02
**Maharashtra**										**ORF3a:F230***	1	0.02
**Maharashtra**										**ORF3a:F231T**	1	0.02
**Maharashtra**										**ORF3a:I232C**	1	0.02
**Maharashtra**										**ORF3a:I249S**	1	0.02
**Maharashtra**										**ORF3a:D250C**	1	0.02
**Maharashtra**										**ORF3a:G251***	1	0.02
**Maharashtra**										**ORF3a:V29F**	1	0.02
**Maharashtra**										**ORF3a:A59V**	1	0.02
**Maharashtra**										**ORF3a:Q213K**	1	0.02
**Maharashtra**										**ORF3a:S165F**	1	0.02
**Maharashtra**										**ORF3a:L95S**	1	0.02
**Maharashtra**										**ORF3a:G187C**	1	0.02
**Maharashtra**										**ORF3a:L127F**	1	0.02
**Maharashtra**										**ORF3a:S135P**	1	0.02
**Maharashtra**										**ORF3a:T223N**	1	0.02
**Maharashtra**										**ORF3a:L115F**	1	0.02
**Maharashtra**										**ORF3a:T9I**	1	0.02
**Maharashtra**										**ORF3a:T34A**	1	0.02
**Maharashtra**										**ORF3a:M260T**	1	0.02
**Maharashtra**										**ORF3a:P25H**	1	0.02
**Maharashtra**										**ORF3a:A72T**	1	0.02
**Maharashtra**										**ORF3a:D210Y**	1	0.02
**Maharashtra**										**ORF3a:Y189C**	1	0.02
**Maharashtra**										**ORF3a:Y264C**	1	0.02
**Maharashtra**										**ORF3a:L86F**	1	0.02
**Maharashtra**										**ORF3a:S177N**	1	0.02
**Maharashtra**										**ORF3a:V197L**	1	0.02
**Maharashtra**										**ORF3a:A59S**	1	0.02
**Maharashtra**										**ORF3a:T32I**	1	0.02
**Maharashtra**										**ORF3a:M125I**	1	0.02
**Maharashtra**										**ORF3a:A98S**	1	0.02
**Maharashtra**										**ORF3a:E19K**	1	0.02
**Maharashtra**										**ORF3a:L3F**	1	0.02
**Maharashtra**										**ORF3a:F105Y**	1	0.02
**Maharashtra**										**ORF3a:R134C**	1	0.02
**Maharashtra**										**ORF3a:K61R**	1	0.02
**Maharashtra**										**ORF3a:L219F**	1	0.02
**Maharashtra**										**ORF3a:I63T**	1	0.02
**Maharashtra**										**ORF3a:F87S**	1	0.02
**Maharashtra**										**ORF3a:E241D**	1	0.02
**Maharashtra**										**ORF3a:T89I**	1	0.02
**Maharashtra**										**ORF3a:L52P**	1	0.02
**Maharashtra**										**ORF3a:T221K**	1	0.02
**Maharashtra**										**ORF3a:I35T**	1	0.02
**Maharashtra**										**ORF3a:K75N**	1	0.02
**Maharashtra**										**ORF3a:I10V**	1	0.02
**Maharashtra**										**ORF3a:V29S**	1	0.02
**Maharashtra**										**ORF3a:G44R**	1	0.02
**Maharashtra**										**ORF3a:W45M**	1	0.02
**Maharashtra**										**ORF3a:L46A**	1	0.02
**Maharashtra**										**ORF3a:I47Y**	1	0.02
**Maharashtra**										**ORF3a:V48C**	1	0.02
**Maharashtra**										**ORF3a:G49W**	1	0.02
**Maharashtra**										**ORF3a:V50R**	1	0.02
**Maharashtra**										**ORF3a:A51C**	1	0.02
**Maharashtra**										**ORF3a:L52T**	1	0.02
**Maharashtra**										**ORF3a:L53S**	1	0.02
**Maharashtra**										**ORF3a:A54C**	1	0.02
**Maharashtra**										**ORF3a:V55C**	1	0.02
**Maharashtra**										**ORF3a:Q57S**	1	0.02
**Maharashtra**										**ORF3a:I186N**	1	0.02
**Maharashtra**										**ORF3a:G188N**	1	0.02
**Maharashtra**										**ORF3a:Y189L**	1	0.02
**Maharashtra**										**ORF3a:T190E**	1	0.02
**Maharashtra**										**ORF3a:E191***	1	0.02
**Maharashtra**										**ORF3a:S209V**	1	0.02
**Maharashtra**										**ORF3a:D210L**	1	0.02
**Maharashtra**										**ORF3a:Q213T**	1	0.02
**Maharashtra**										**ORF3a:L214V**	1	0.02

**Maharashtra**										**ORF3a:Y215T**	1	0.02
**Maharashtra**										**ORF3a:T217L**	1	0.02
**Maharashtra**										**ORF3a:L219T**	1	0.02
**Maharashtra**										**ORF3a:S220I**	1	0.02
**Maharashtra**										**ORF3a:E241N**	1	0.02
**Maharashtra**										**ORF3a:E242S**	1	0.02
**Maharashtra**										**ORF3a:P258S**	1	0.02
**Maharashtra**										**ORF3a:V259S**	1	0.02
**Maharashtra**										**ORF3a:L95V**	1	0.02
**Maharashtra**										**ORF3a:I158V**	1	0.02
**Maharashtra**										**ORF3a:E242L**	1	0.02
**Maharashtra**										**ORF3a:L46F**	1	0.02
**Maharashtra**										**ORF3a:E241***	1	0.02
**Maharashtra**										**ORF3a:S58C**	1	0.02
**Maharashtra**										**ORF3a:I246V**	1	0.02
**Maharashtra**										**ORF3a:K21N**	1	0.02
**Maharashtra**										**ORF3a:D210A**	1	0.02
**Maharashtra**										**ORF3a:I62T**	1	0.02
**Maharashtra**										**ORF3a:R68T**	1	0.02
**Maharashtra**										**ORF3a:Q213R**	1	0.02
**Maharashtra**										**ORF3a:M260V**	1	0.02
**Maharashtra**										**ORF3a:T271S**	1	0.02
**Maharashtra**										**ORF3a:A39T**	1	0.02
**Maharashtra**										**ORF3a:S272I**	1	0.02
**Maharashtra**										**ORF3a:I47T**	1	0.02
**Maharashtra**										**ORF3a:A98T**	1	0.02
**Maharashtra**										**ORF3a:S58N**	1	0.02
**Maharashtra**										**ORF3a:V197I**	1	0.02
**State/Gene**	**ORF6**											
**Maharashtra**	**Pre-Wave 1**			**Wave 1**			**Pre-Wave 2**			**Wave 2**		
**Maharashtra**	**Mutation**	**Count**	**percent**	**Mutation**	**Count**	**percent**	**Mutation**	**Count**	**percent**	**Mutation**	**Count**	**percent**
**Maharashtra**	**ORF6:E13D**	3	21.43	**ORF6:K42N**	5	33.33	**ORF6:I33T**	68	75.56	**ORF6:I33T**	817	90.38
**Maharashtra**	**ORF6:E13G**	2	14.29	**ORF6:I26V**	1	6.67	**ORF6:Q51L**	4	4.44	**ORF6:E54***	11	1.22
**Maharashtra**	**ORF6:V5I**	2	14.29	**ORF6:I18S**	1	6.67	**ORF6:D53Y**	3	3.33	**ORF6:W27L**	8	0.88
**Maharashtra**	**ORF6:F2S**	1	7.14	**ORF6:V5I**	1	6.67	**ORF6:I32T**	3	3.33	**ORF6:R20K**	6	0.66
**Maharashtra**	**ORF6:T21S**	1	7.14	**ORF6:F7S**	1	6.67	**ORF6:L52S**	3	3.33	**ORF6:D6Y**	6	0.66
**Maharashtra**	**ORF6:I33L**	1	7.14	**ORF6:H3Y**	1	6.67	**ORF6:F2S**	2	2.22	**ORF6:T21N**	5	0.55
**Maharashtra**	**ORF6:V5F**	1	7.14	**ORF6:W27L**	1	6.67	**ORF6:W27L**	2	2.22	**ORF6:D53Y**	4	0.44
**Maharashtra**	**ORF6:M58I**	1	7.14	**ORF6:D6G**	1	6.67	**ORF6:D53K**	1	1.11	**ORF6:D61Y**	4	0.44
**Maharashtra**	**ORF6:E59Q**	1	7.14	**ORF6:E54***	1	6.67	**ORF6:E55***	1	1.11	**ORF6:N47S**	3	0.33
**Maharashtra**	**ORF6:V9F**	1	7.14	**ORF6:D61H**	1	6.67	**ORF6:E54***	1	1.11	**ORF6:T10I**	3	0.33
**Maharashtra**				**ORF6:N34S**	1	6.67	**ORF6:R20S**	1	1.11	**ORF6:D61C**	2	0.22
**Maharashtra**							**ORF6:D30Y**	1	1.11	**ORF6:Q8H**	2	0.22
**Maharashtra**										**ORF6:F2L**	2	0.22
**Maharashtra**										**ORF6:V24F**	2	0.22
**Maharashtra**										**ORF6:V9F**	2	0.22
**Maharashtra**										**ORF6:D61L**	2	0.22
**Maharashtra**										**ORF6:P57L**	2	0.22
**Maharashtra**										**ORF6:Y31H**	2	0.22
**Maharashtra**										**ORF6:E55***	2	0.22
**Maharashtra**										**ORF6:F2M**	2	0.22
**Maharashtra**										**ORF6:M1I**	2	0.22
**Maharashtra**										**ORF6:T21I**	2	0.22
**Maharashtra**										**ORF6:L29F**	2	0.22
**Maharashtra**										**ORF6:H3N**	1	0.11
**Maharashtra**										**ORF6:D53V**	1	0.11
**Maharashtra**										**ORF6:S43P**	1	0.11
**Maharashtra**										**ORF6:I60V**	1	0.11
**Maharashtra**										**ORF6:V5F**	1	0.11
**Maharashtra**										**ORF6:D61H**	1	0.11
**Maharashtra**										**ORF6:D6N**	1	0.11
**Maharashtra**										**ORF6:K42M**	1	0.11
**Maharashtra**										**ORF6:W27C**	1	0.11
**Maharashtra**										**ORF6:Q56R**	1	0.11
**Maharashtra**										**ORF6:Y49H**	1	0.11
**State/Gene**	**ORF7a**											
**Maharashtra**	**Pre-Wave 1**			**Wave 1**			**Pre-Wave 2**			**Wave 2**		
**Maharashtra**	**Mutation**	**Count**	**percent**	**Mutation**	**Count**	**percent**	**Mutation**	**Count**	**percent**	**Mutation**	**Count**	**percent**
**Maharashtra**	**ORF7a:V104F**	14	30.43	**ORF7a:V104F**	18	32.73	**ORF7a:V82A**	207	77.82	**ORF7a:V82A**	2642	58.78
**Maharashtra**	**ORF7a:E95K**	2	4.35	**ORF7a:S83L**	4	7.27	**ORF7a:D69E**	6	2.26	**ORF7a:T120I**	1136	25.27
**Maharashtra**	**ORF7a:T120I**	2	4.35	**ORF7a:R25T**	4	7.27	**ORF7a:K119N**	5	1.88	**ORF7a:L116F**	247	5.49

Maharashtra	ORF7a:V24F	2	4.35	ORF7a:T28I	3	5.45	ORF7a:S83L	4	1.5	ORF7a:K119N	86	1.91
Maharashtra	ORF7a:G38V	2	4.35	ORF7a:F87I	3	5.45	ORF7a:T11I	3	1.13	ORF7a:H73Y	76	1.69
Maharashtra	ORF7a:T39K	2	4.35	ORF7a:I10T	3	5.45	ORF7a:T14I	3	1.13	ORF7a:N43Y	17	0.38
Maharashtra	ORF7a:Y40H	2	4.35	ORF7a:A8V	2	3.64	ORF7a:N43Y	3	1.13	ORF7a:E95K	15	0.33
Maharashtra	ORF7a:E22D	2	4.35	ORF7a:P84S	1	1.82	ORF7a:V29L	2	0.75	ORF7a:V104F	14	0.31
Maharashtra	ORF7a:T39I	2	4.35	ORF7a:H47Y	1	1.82	ORF7a:R25I	2	0.75	ORF7a:V93F	11	0.24
Maharashtra	ORF7a:E121A	1	2.17	ORF7a:T115R	1	1.82	ORF7a:H73Y	2	0.75	ORF7a:A105S	10	0.22
Maharashtra	ORF7a:E92D	1	2.17	ORF7a:L116F	1	1.82	ORF7a:R78G	2	0.75	ORF7a:E121*	10	0.22
Maharashtra	ORF7a:A66V	1	2.17	ORF7a:C35F	1	1.82	ORF7a:T120I	2	0.75	ORF7a:T11I	10	0.22
Maharashtra	ORF7a:P48R	1	2.17	ORF7a:L96F	1	1.82	ORF7a:A105V	2	0.75	ORF7a:P84L	8	0.18
Maharashtra	ORF7a:H47L	1	2.17	ORF7a:P84L	1	1.82	ORF7a:E95K	1	0.38	ORF7a:E41D	7	0.16
Maharashtra	ORF7a:A50V	1	2.17	ORF7a:D69G	1	1.82	ORF7a:P48L	1	0.38	ORF7a:T14I	7	0.16
Maharashtra	ORF7a:N52T	1	2.17	ORF7a:E95G	1	1.82	ORF7a:L49V	1	0.38	ORF7a:V24F	6	0.13
Maharashtra	ORF7a:K53Q	1	2.17	ORF7a:S81L	1	1.82	ORF7a:L77*	1	0.38	ORF7a:F87Y	6	0.13
Maharashtra	ORF7a:Q90H	1	2.17	ORF7a:K2E	1	1.82	ORF7a:P99Q	1	0.38	ORF7a:R80I	6	0.13
Maharashtra	ORF7a:T111A	1	2.17	ORF7a:A66S	1	1.82	ORF7a:V104F	1	0.38	ORF7a:A105V	5	0.11
Maharashtra	ORF7a:H73D	1	2.17	ORF7a:E22D	1	1.82	ORF7a:R80T	1	0.38	ORF7a:T39I	4	0.09
Maharashtra	ORF7a:S37F	1	2.17	ORF7a:V82A	1	1.82	ORF7a:A13T	1	0.38	ORF7a:R118I	4	0.09
Maharashtra	ORF7a:T39E	1	2.17	ORF7a:E95K	1	1.82	ORF7a:V108L	1	0.38	ORF7a:L12F	4	0.09
Maharashtra	ORF7a:Y40N	1	2.17	ORF7a:T115I	1	1.82	ORF7a:K32T	1	0.38	ORF7a:P48S	4	0.09
Maharashtra	ORF7a:M1I	1	2.17	ORF7a:F87V	1	1.82	ORF7a:T115R	1	0.38	ORF7a:F46L	4	0.09
Maharashtra	ORF7a:T61I	1	2.17	ORF7a:A79V	1	1.82	ORF7a:V104I	1	0.38	ORF7a:Q62*	4	0.09
Maharashtra							ORF7a:A106V	1	0.38	ORF7a:S83L	3	0.07
Maharashtra							ORF7a:L49S	1	0.38	ORF7a:E121Q	3	0.07
Maharashtra							ORF7a:A64S	1	0.38	ORF7a:F63L	3	0.07
Maharashtra							ORF7a:E95Q	1	0.38	ORF7a:G38V	3	0.07
Maharashtra							ORF7a:R25K	1	0.38	ORF7a:H47Y	3	0.07
Maharashtra							ORF7a:V24I	1	0.38	ORF7a:T61I	3	0.07
Maharashtra							ORF7a:E92*	1	0.38	ORF7a:E91*	3	0.07
Maharashtra							ORF7a:A55S	1	0.38	ORF7a:*122L	3	0.07
Maharashtra							ORF7a:F114L	1	0.38	ORF7a:R25I	3	0.07
Maharashtra							ORF7a:P34S	1	0.38	ORF7a:A79V	3	0.07
Maharashtra							ORF7a:P45R	1	0.38	ORF7a:Y75H	2	0.04
Maharashtra										ORF7a:G38*	2	0.04
Maharashtra										ORF7a:P84S	2	0.04
Maharashtra										ORF7a:I10L	2	0.04
Maharashtra										ORF7a:F54L	2	0.04
Maharashtra										ORF7a:A66V	2	0.04
Maharashtra										ORF7a:A66S	2	0.04
Maharashtra										ORF7a:Q94H	2	0.04
Maharashtra										ORF7a:E95*	2	0.04
Maharashtra										ORF7a:Q62L	2	0.04
Maharashtra										ORF7a:P34L	2	0.04
Maharashtra										ORF7a:A55S	2	0.04
Maharashtra										ORF7a:P99L	2	0.04
Maharashtra										ORF7a:S81L	2	0.04
Maharashtra										ORF7a:R89I	2	0.04
Maharashtra										ORF7a:A105E	2	0.04
Maharashtra										ORF7a:A64S	2	0.04
Maharashtra										ORF7a:E16D	2	0.04
Maharashtra										ORF7a:V71L	2	0.04
Maharashtra										ORF7a:P48H	1	0.02
Maharashtra										ORF7a:L102F	1	0.02
Maharashtra										ORF7a:V74F	1	0.02
Maharashtra										ORF7a:T57A	1	0.02
Maharashtra										ORF7a:E33*	1	0.02
Maharashtra										ORF7a:T11K	1	0.02
Maharashtra										ORF7a:V93I	1	0.02
Maharashtra										ORF7a:K119*	1	0.02
Maharashtra										ORF7a:C67*	1	0.02
Maharashtra										ORF7a:P68S	1	0.02
Maharashtra										ORF7a:Q76V	1	0.02
Maharashtra										ORF7a:R118Q	1	0.02
Maharashtra										ORF7a:K53S	1	0.02
Maharashtra										ORF7a:F54*	1	0.02
Maharashtra										ORF7a:A55*	1	0.02
Maharashtra										ORF7a:L56Q	1	0.02
Maharashtra										ORF7a:T57I	1	0.02
Maharashtra										ORF7a:I103L	1	0.02
Maharashtra										ORF7a:L112H	1	0.02
Maharashtra										ORF7a:E91R	1	0.02
Maharashtra										ORF7a:E92G	1	0.02
Maharashtra										ORF7a:E91K	1	0.02

**Maharashtra**										**ORF7a:T120S**	1	0.02
**Maharashtra**										**ORF7a:H73L**	1	0.02
**Maharashtra**										**ORF7a:A105R**	1	0.02
**Maharashtra**										**ORF7a:A106Q**	1	0.02
**Maharashtra**										**ORF7a:I107L**	1	0.02
**Maharashtra**										**ORF7a:I10T**	1	0.02
**Maharashtra**										**ORF7a:E95Q**	1	0.02
**Maharashtra**										**ORF7a:C67W**	1	0.02
**Maharashtra**										**ORF7a:S81***	1	0.02
**Maharashtra**										**ORF7a:F114L**	1	0.02
**Maharashtra**										**ORF7a:Q76H**	1	0.02
**Maharashtra**										**ORF7a:Y97F**	1	0.02
**Maharashtra**										**ORF7a:N43S**	1	0.02
**Maharashtra**										**ORF7a:T111N**	1	0.02
**Maharashtra**										**ORF7a:L112F**	1	0.02
**Maharashtra**										**ORF7a:K117Q**	1	0.02
**Maharashtra**										**ORF7a:D69N**	1	0.02
**Maharashtra**										**ORF7a:Q94K**	1	0.02
**Maharashtra**										**ORF7a:R78C**	1	0.02
**Maharashtra**										**ORF7a:Q90***	1	0.02
**Maharashtra**										**ORF7a:G38E**	1	0.02
**Maharashtra**										**ORF7a:Q76***	1	0.02
**Maharashtra**										**ORF7a:Q94P**	1	0.02
**Maharashtra**										**ORF7a:A79P**	1	0.02
**Maharashtra**										**ORF7a:Y40S**	1	0.02
**Maharashtra**										**ORF7a:E41L**	1	0.02
**Maharashtra**										**ORF7a:Q21***	1	0.02
**Maharashtra**										**ORF7a:P34S**	1	0.02
**Maharashtra**										**ORF7a:R78L**	1	0.02
**Maharashtra**										**ORF7a:V24L**	1	0.02
**Maharashtra**										**ORF7a:Q94L**	1	0.02
**Maharashtra**										**ORF7a:I4T**	1	0.02
**Maharashtra**										**ORF7a:L86M**	1	0.02
**Maharashtra**										**ORF7a:Y97H**	1	0.02
**Maharashtra**										**ORF7a:S98T**	1	0.02
**Maharashtra**										**ORF7a:K53N**	1	0.02
**Maharashtra**										**ORF7a:A55Q**	1	0.02
**Maharashtra**										**ORF7a:R80Q**	1	0.02
**Maharashtra**										**ORF7a:E22D**	1	0.02
**Maharashtra**										**ORF7a:C35***	1	0.02
**Maharashtra**										**ORF7a:P45L**	1	0.02
**Maharashtra**										**ORF7a:T115I**	1	0.02
**Maharashtra**										**ORF7a:Y97***	1	0.02
**Maharashtra**										**ORF7a:K2E**	1	0.02
**Maharashtra**										**ORF7a:N52K**	1	0.02
**Maharashtra**										**ORF7a:C113R**	1	0.02
**Maharashtra**										**ORF7a:P99S**	1	0.02
**Maharashtra**										**ORF7a:V74A**	1	0.02
**Maharashtra**										**ORF7a:T120K**	1	0.02
**Maharashtra**										**ORF7a:R118K**	1	0.02
**Maharashtra**										**ORF7a:A64T**	1	0.02
**Maharashtra**										**ORF7a:V82T**	1	0.02
**Maharashtra**										**ORF7a:N52H**	1	0.02
**Maharashtra**										**ORF7a:K53Q**	1	0.02
**Maharashtra**										**ORF7a:Q90K**	1	0.02
**Maharashtra**										**ORF7a:E91D**	1	0.02
**Maharashtra**										**ORF7a:E92R**	1	0.02
**Maharashtra**										**ORF7a:V93M**	1	0.02
**Maharashtra**										**ORF7a:Q94I**	1	0.02
**Maharashtra**										**ORF7a:K117I**	1	0.02
**Maharashtra**										**ORF7a:R118E**	1	0.02
**Maharashtra**										**ORF7a:E92K**	1	0.02
**State/Gene**	**ORF7b**											
**Maharashtra**	**Pre-Wave 1**			**Wave 1**			**Pre-Wave 2**			**Wave 2**		
**Maharashtra**	**Mutation**	**Count**	**percent**	**Mutation**	**Count**	**percent**	**Mutation**	**Count**	**percent**	**Mutation**	**Count**	**percent**
**Maharashtra**	**ORF7b:S31L**	5	33.33	**ORF7b:I23T**	9	36	**ORF7b:H37Y**	3	8.82	**ORF7b:T40I**	372	83.6
**Maharashtra**	**ORF7b:F19L**	3	20	**ORF7b:E39***	4	16	**ORF7b:E33***	3	8.82	**ORF7b:A15S**	16	3.6
**Maharashtra**	**ORF7b:W29L**	1	6.67	**ORF7b:M24I**	3	12	**ORF7b:A15S**	3	8.82	**ORF7b:E39***	10	2.25
**Maharashtra**	**ORF7b:L6F**	1	6.67	**ORF7b:S31L**	3	12	**ORF7b:E39***	3	8.82	**ORF7b:M1I**	4	0.9
**Maharashtra**	**ORF7b:A43V**	1	6.67	**ORF7b:S5L**	3	12	**ORF7b:H37L**	2	5.88	**ORF7b:L14S**	4	0.9
**Maharashtra**	**ORF7b:T40I**	1	6.67	**ORF7b:D8Y**	1	4	**ORF7b:T40I**	2	5.88	**ORF7b:H37Y**	3	0.67
**Maharashtra**	**ORF7b:H42Y**	1	6.67	**ORF7b:N38S**	1	4	**ORF7b:I23T**	2	5.88	**ORF7b:F13L**	3	0.67

**Maharashtra**	**ORF7b:I27T**	1	6.67	**ORF7b:D36G**	1	4	**ORF7b:I7V**	1	2.94	**ORF7b:H42Q**	3	0.67
**Maharashtra**	**ORF7b:T40P**	1	6.67				**ORF7b:E3***	1	2.94	**ORF7b:C41F**	3	0.67
**Maharashtra**							**ORF7b:S31T**	1	2.94	**ORF7b:H37R**	2	0.45
**Maharashtra**							**ORF7b:Y10C**	1	2.94	**ORF7b:F16L**	2	0.45
**Maharashtra**							**ORF7b:L32F**	1	2.94	**ORF7b:I27T**	1	0.22
**Maharashtra**							**ORF7b:M24I**	1	2.94	**ORF7b:N38S**	1	0.22
**Maharashtra**							**ORF7b:L4F**	1	2.94	**ORF7b:I2N**	1	0.22
**Maharashtra**							**ORF7b:C41F**	1	2.94	**ORF7b:A43T**	1	0.22
**Maharashtra**							**ORF7b:S5L**	1	2.94	**ORF7b:C41V**	1	0.22
**Maharashtra**							**ORF7b:A15I**	1	2.94	**ORF7b:S5T**	1	0.22
**Maharashtra**							**ORF7b:I2N**	1	2.94	**ORF7b:A43S**	1	0.22
**Maharashtra**							**ORF7b:M1V**	1	2.94	**ORF7b:A43L**	1	0.22
**Maharashtra**							**ORF7b:L22S**	1	2.94	**ORF7b:W29C**	1	0.22
**Maharashtra**							**ORF7b:A43L**	1	2.94	**ORF7b:E33***	1	0.22
**Maharashtra**							**ORF7b:H42Q**	1	2.94	**ORF7b:*44L**	1	0.22
**Maharashtra**							**ORF7b:L6M**	1	2.94	**ORF7b:H37L**	1	0.22
**Maharashtra**										**ORF7b:L32F**	1	0.22
**Maharashtra**										**ORF7b:L18P**	1	0.22
**Maharashtra**										**ORF7b:F9S**	1	0.22
**Maharashtra**										**ORF7b:I2K**	1	0.22
**Maharashtra**										**ORF7b:L14F**	1	0.22
**Maharashtra**										**ORF7b:A15T**	1	0.22
**Maharashtra**										**ORF7b:I7N**	1	0.22
**Maharashtra**										**ORF7b:F13S**	1	0.22
**Maharashtra**										**ORF7b:M24I**	1	0.22
**Maharashtra**										**ORF7b:L11S**	1	0.22
**Maharashtra**										**ORF7b:C12H**	1	0.22
**State/Gene**	**ORF8**											
**Maharashtra**	**Pre-Wave 1**			**Wave 1**			**Pre-Wave 2**			**Wave 2**		
**Maharashtra**	**Mutation**	**Count**	**percent**	**Mutation**	**Count**	**percent**	**Mutation**	**Count**	**percent**	**Mutation**	**Count**	**percent**
**Maharashtra**	**ORF8:L84S**	11	20	**ORF8:E106Q**	68	35.05	**ORF8:S97I**	122	31.52	**ORF8:S69L**	265	23.27
**Maharashtra**	**ORF8:V62A**	5	9.09	**ORF8:V62L**	27	13.92	**ORF8:Q27***	29	7.49	**ORF8:T26I**	127	11.15
**Maharashtra**	**ORF8:T87I**	4	7.27	**ORF8:S97I**	14	7.22	**ORF8:R52I**	29	7.49	**ORF8:S97I**	117	10.27
**Maharashtra**	**ORF8:V116F**	4	7.27	**ORF8:T87I**	10	5.15	**ORF8:Y73C**	29	7.49	**ORF8:Y73C**	104	9.13
**Maharashtra**	**ORF8:V62L**	3	5.45	**ORF8:I121L**	8	4.12	**ORF8:S69L**	25	6.46	**ORF8:R52I**	101	8.87
**Maharashtra**	**ORF8:I121F**	2	3.64	**ORF8:A51S**	8	4.12	**ORF8:T26I**	22	5.68	**ORF8:Q27***	98	8.6
**Maharashtra**	**ORF8:E59***	2	3.64	**ORF8:S69P**	7	3.61	**ORF8:E106Q**	15	3.88	**ORF8:G8V**	61	5.36
**Maharashtra**	**ORF8:C25F**	2	3.64	**ORF8:F120L**	6	3.09	**ORF8:I121L**	10	2.58	**ORF8:Q18***	20	1.76
**Maharashtra**	**ORF8:Q18***	2	3.64	**ORF8:G66A**	5	2.58	**ORF8:V62L**	6	1.55	**ORF8:K68***	19	1.67
**Maharashtra**	**ORF8:S54L**	2	3.64	**ORF8:G8***	3	1.55	**ORF8:C25F**	5	1.29	**ORF8:I121L**	16	1.4
**Maharashtra**	**ORF8:R52I**	1	1.82	**ORF8:D34G**	3	1.55	**ORF8:L95F**	5	1.29	**ORF8:E106Q**	12	1.05
**Maharashtra**	**ORF8:E106***	1	1.82	**ORF8:W45***	2	1.03	**ORF8:V49L**	5	1.29	**ORF8:I10T**	12	1.05
**Maharashtra**	**ORF8:G8R**	1	1.82	**ORF8:C20F**	2	1.03	**ORF8:K68***	5	1.29	**ORF8:F120L**	10	0.88
**Maharashtra**	**ORF8:V117F**	1	1.82	**ORF8:E106***	2	1.03	**ORF8:P93S**	4	1.03	**ORF8:V62L**	9	0.79
**Maharashtra**	**ORF8:Q72H**	1	1.82	**ORF8:P93S**	2	1.03	**ORF8:G8E**	3	0.78	**ORF8:L95F**	9	0.79
**Maharashtra**	**ORF8:I121S**	1	1.82	**ORF8:I9T**	2	1.03	**ORF8:F120L**	3	0.78	**ORF8:K68E**	7	0.61
**Maharashtra**	**ORF8:I71S**	1	1.82	**ORF8:S103L**	2	1.03	**ORF8:I9V**	3	0.78	**ORF8:F120V**	6	0.53
**Maharashtra**	**ORF8:I74S**	1	1.82	**ORF8:V62F**	1	0.52	**ORF8:H17Y**	3	0.78	**ORF8:P93S**	6	0.53
**Maharashtra**	**ORF8:A51S**	1	1.82	**ORF8:H112L**	1	0.52	**ORF8:E110***	2	0.52	**ORF8:M1I**	6	0.53
**Maharashtra**	**ORF8:S69P**	1	1.82	**ORF8:E64D**	1	0.52	**ORF8:A65V**	2	0.52	**ORF8:A65S**	6	0.53
**Maharashtra**	**ORF8:Y73N**	1	1.82	**ORF8:E64***	1	0.52	**ORF8:T11I**	2	0.52	**ORF8:E110***	4	0.35
**Maharashtra**	**ORF8:A51V**	1	1.82	**ORF8:G8E**	1	0.52	**ORF8:D119E**	2	0.52	**ORF8:A65V**	4	0.35
**Maharashtra**	**ORF8:A14V**	1	1.82	**ORF8:P70L**	1	0.52	**ORF8:Q29***	1	0.26	**ORF8:F16S**	4	0.35
**Maharashtra**	**ORF8:G8***	1	1.82	**ORF8:E110***	1	0.52	**ORF8:C20F**	1	0.26	**ORF8:C37F**	4	0.35
**Maharashtra**	**ORF8:S67Y**	1	1.82	**ORF8:G66V**	1	0.52	**ORF8:W45L**	1	0.26	**ORF8:P36S**	4	0.35
**Maharashtra**	**ORF8:K68E**	1	1.82	**ORF8:P36L**	1	0.52	**ORF8:Q91R**	1	0.26	**ORF8:A15S**	3	0.26
**Maharashtra**	**ORF8:L98F**	1	1.82	**ORF8:Q27***	1	0.52	**ORF8:L118V**	1	0.26	**ORF8:T11I**	3	0.26
**Maharashtra**	**ORF8:P30T**	1	1.82	**ORF8:P85S**	1	0.52	**ORF8:L109V**	1	0.26	**ORF8:E106***	3	0.26
**Maharashtra**				**ORF8:I39V**	1	0.52	**ORF8:E110L**	1	0.26	**ORF8:D34Y**	3	0.26
**Maharashtra**				**ORF8:V116P**	1	0.52	**ORF8:Y111***	1	0.26	**ORF8:L118F**	3	0.26
**Maharashtra**				**ORF8:G77V**	1	0.52	**ORF8:H112R**	1	0.26	**ORF8:S43F**	3	0.26
**Maharashtra**				**ORF8:V62A**	1	0.52	**ORF8:D113L**	1	0.26	**ORF8:E106D**	3	0.26
**Maharashtra**				**ORF8:C61F**	1	0.52	**ORF8:V114F**	1	0.26	**ORF8:V49I**	3	0.26
**Maharashtra**				**ORF8:Y31***	1	0.52	**ORF8:V117C**	1	0.26	**ORF8:Q29***	2	0.18
**Maharashtra**				**ORF8:K68R**	1	0.52	**ORF8:L118F**	1	0.26	**ORF8:A55E**	2	0.18
**Maharashtra**				**ORF8:L4F**	1	0.52	**ORF8:D119R**	1	0.26	**ORF8:S54A**	2	0.18
**Maharashtra**				**ORF8:P56S**	1	0.52	**ORF8:D119I**	1	0.26	**ORF8:W45L**	2	0.18
**Maharashtra**				**ORF8:Q72H**	1	0.52	**ORF8:I121***	1	0.26	**ORF8:C83F**	2	0.18
**Maharashtra**				**ORF8:E19***	1	0.52	**ORF8:L60S**	1	0.26	**ORF8:T11A**	2	0.18
**Maharashtra**				**ORF8:T26P**	1	0.52	**ORF8:E19***	1	0.26	**ORF8:N89D**	2	0.18
**Maharashtra**							**ORF8:F108L**	1	0.26	**ORF8:L22***	2	0.18
**Maharashtra**							**ORF8:G8V**	1	0.26	**ORF8:P70L**	2	0.18

**Maharashtra**							**ORF8:A51T**	1	0.26	**ORF8:A51V**	2	0.18
**Maharashtra**							**ORF8:P36L**	1	0.26	**ORF8:C90F**	2	0.18
**Maharashtra**							**ORF8:E64D**	1	0.26	**ORF8:D75E**	1	0.09
**Maharashtra**							**ORF8:R101H**	1	0.26	**ORF8:G8R**	1	0.09
**Maharashtra**							**ORF8:V5F**	1	0.26	**ORF8:K53T**	1	0.09
**Maharashtra**							**ORF8:G77C**	1	0.26	**ORF8:C20F**	1	0.09
**Maharashtra**							**ORF8:C37F**	1	0.26	**ORF8:C25F**	1	0.09
**Maharashtra**							**ORF8:I10V**	1	0.26	**ORF8:F41S**	1	0.09
**Maharashtra**							**ORF8:L84***	1	0.26	**ORF8:V49***	1	0.09
**Maharashtra**							**ORF8:F86Y**	1	0.26	**ORF8:D34H**	1	0.09
**Maharashtra**							**ORF8:T87V**	1	0.26	**ORF8:V99I**	1	0.09
**Maharashtra**							**ORF8:I88V**	1	0.26	**ORF8:A65T**	1	0.09
**Maharashtra**							**ORF8:N89D**	1	0.26	**ORF8:H112L**	1	0.09
**Maharashtra**							**ORF8:C90D**	1	0.26	**ORF8:Y46D**	1	0.09
**Maharashtra**							**ORF8:Q91P**	1	0.26	**ORF8:A65E**	1	0.09
**Maharashtra**							**ORF8:P93C**	1	0.26	**ORF8:F3S**	1	0.09
**Maharashtra**							**ORF8:K68R**	1	0.26	**ORF8:E64***	1	0.09
**Maharashtra**							**ORF8:D119N**	1	0.26	**ORF8:C102***	1	0.09
**Maharashtra**							**ORF8:F120A**	1	0.26	**ORF8:H112I**	1	0.09
**Maharashtra**							**ORF8:L84S**	1	0.26	**ORF8:D113M**	1	0.09
**Maharashtra**							**ORF8:F120V**	1	0.26	**ORF8:V114T**	1	0.09
**Maharashtra**							**ORF8:T87I**	1	0.26	**ORF8:R115F**	1	0.09
**Maharashtra**							**ORF8:E106***	1	0.26	**ORF8:V117L**	1	0.09
**Maharashtra**							**ORF8:C37T**	1	0.26	**ORF8:D119***	1	0.09
**Maharashtra**							**ORF8:P38N**	1	0.26	**ORF8:K44I**	1	0.09
**Maharashtra**							**ORF8:L57S**	1	0.26	**ORF8:L22S**	1	0.09
**Maharashtra**							**ORF8:E59K**	1	0.26	**ORF8:E19K**	1	0.09
**Maharashtra**							**ORF8:E64G**	1	0.26	**ORF8:P38L**	1	0.09
**Maharashtra**							**ORF8:G66D**	1	0.26	**ORF8:G66V**	1	0.09
**Maharashtra**							**ORF8:S67F**	1	0.26	**ORF8:V62M**	1	0.09
**Maharashtra**							**ORF8:K68L**	1	0.26	**ORF8:V81F**	1	0.09
**Maharashtra**							**ORF8:S69A**	1	0.26	**ORF8:Q29L**	1	0.09
**Maharashtra**							**ORF8:I71T**	1	0.26	**ORF8:P93L**	1	0.09
**Maharashtra**							**ORF8:Q72L**	1	0.26	**ORF8:R115C**	1	0.09
**Maharashtra**							**ORF8:Q29L**	1	0.26	**ORF8:K44***	1	0.09
**Maharashtra**							**ORF8:Y42H**	1	0.26	**ORF8:E64D**	1	0.09
**Maharashtra**										**ORF8:I10V**	1	0.09
**Maharashtra**										**ORF8:I88F**	1	0.09
**Maharashtra**										**ORF8:N89Y**	1	0.09
**Maharashtra**										**ORF8:C90N**	1	0.09
**Maharashtra**										**ORF8:Q91***	1	0.09
**Maharashtra**										**ORF8:E92L**	1	0.09
**Maharashtra**										**ORF8:K94V**	1	0.09
**Maharashtra**										**ORF8:G96***	1	0.09
**Maharashtra**										**ORF8:S97R**	1	0.09
**Maharashtra**										**ORF8:V114F**	1	0.09
**Maharashtra**										**ORF8:V117C**	1	0.09
**Maharashtra**										**ORF8:D119R**	1	0.09
**Maharashtra**										**ORF8:I71N**	1	0.09
**Maharashtra**										**ORF8:C102R**	1	0.09
**Maharashtra**										**ORF8:D75Y**	1	0.09
**Maharashtra**										**ORF8:S67F**	1	0.09
**Maharashtra**										**ORF8:A51S**	1	0.09
**Maharashtra**										**ORF8:A55V**	1	0.09
**Maharashtra**										**ORF8:S103L**	1	0.09
**Maharashtra**										**ORF8:V13I**	1	0.09
**Maharashtra**										**ORF8:Q72R**	1	0.09
**Maharashtra**										**ORF8:D63N**	1	0.09
**Maharashtra**										**ORF8:L7***	1	0.09
**Maharashtra**										**ORF8:D34V**	1	0.09
**Maharashtra**										**ORF8:I74F**	1	0.09
**Maharashtra**										**ORF8:P93T**	1	0.09
**Maharashtra**										**ORF8:Q23H**	1	0.09
**Maharashtra**										**ORF8:L84S**	1	0.09
**State/Gene**	**ORF9b**											
**Maharashtra**	**Pre-Wave 1**			**Wave 1**			**Pre-Wave 2**			**Wave 2**		
**Maharashtra**	**Mutation**	**Count**	**percent**	**Mutation**	**Count**	**percent**	**Mutation**	**Count**	**percent**	**Mutation**	**Count**	**percent**
**Maharashtra**	**ORF9b:P10S**	44	73.33	**ORF9b:P3L**	14	30.43	**ORF9b:R32L**	28	40.58	**ORF9b:T60A**	843	77.62
**Maharashtra**	**ORF9b:V30L**	6	10	**ORF9b:R32L**	6	13.04	**ORF9b:R13H**	5	7.25	**ORF9b:R32L**	57	5.25
**Maharashtra**	**ORF9b:A75V**	2	3.33	**ORF9b:P10S**	4	8.7	**ORF9b:P10S**	4	5.8	**ORF9b:P10S**	15	1.38
**Maharashtra**	**ORF9b:I5T**	1	1.67	**ORF9b:K67E**	3	6.52	**ORF9b:T60A**	3	4.35	**ORF9b:H9D**	14	1.29
**Maharashtra**	**ORF9b:E27***	1	1.67	**ORF9b:S6I**	2	4.35	**ORF9b:D89V**	2	2.9	**ORF9b:A68V**	7	0.64

**Maharashtra**	**ORF9b:H9Q**	1	1.67	**ORF9b:A57V**	2	4.35	**ORF9b:R13L**	2	2.9	**ORF9b:Q34L**	7	0.64
**Maharashtra**	**ORF9b:R32G**	1	1.67	**ORF9b:A75V**	2	4.35	**ORF9b:S50L**	2	2.9	**ORF9b:A11V**	6	0.55
**Maharashtra**	**ORF9b:V23L**	1	1.67	**ORF9b:P17T**	1	2.17	**ORF9b:P3S**	2	2.9	**ORF9b:V30L**	6	0.55
**Maharashtra**	**ORF9b:R25***	1	1.67	**ORF9b:P3H**	1	2.17	**ORF9b:H9Q**	2	2.9	**ORF9b:R13L**	5	0.46
**Maharashtra**	**ORF9b:P3L**	1	1.67	**ORF9b:H9Q**	1	2.17	**ORF9b:N28K**	1	1.45	**ORF9b:A29T**	5	0.46
**Maharashtra**	**ORF9b:R13C**	1	1.67	**ORF9b:R32P**	1	2.17	**ORF9b:A68V**	1	1.45	**ORF9b:M8I**	5	0.46
**Maharashtra**				**ORF9b:I45V**	1	2.17	**ORF9b:V23K**	1	1.45	**ORF9b:E27Q**	5	0.46
**Maharashtra**				**ORF9b:R32C**	1	2.17	**ORF9b:T24H**	1	1.45	**ORF9b:E86D**	4	0.37
**Maharashtra**				**ORF9b:V37F**	1	2.17	**ORF9b:R25S**	1	1.45	**ORF9b:S6N**	4	0.37
**Maharashtra**				**ORF9b:M8I**	1	2.17	**ORF9b:M26L**	1	1.45	**ORF9b:T72I**	3	0.28
**Maharashtra**				**ORF9b:M8V**	1	2.17	**ORF9b:K67E**	1	1.45	**ORF9b:K59N**	3	0.28
**Maharashtra**				**ORF9b:P10L**	1	2.17	**ORF9b:E65A**	1	1.45	**ORF9b:A57V**	3	0.28
**Maharashtra**				**ORF9b:A68V**	1	2.17	**ORF9b:D66R**	1	1.45	**ORF9b:P3S**	3	0.28
**Maharashtra**				**ORF9b:M26I**	1	2.17	**ORF9b:S6I**	1	1.45	**ORF9b:R25I**	3	0.28
**Maharashtra**				**ORF9b:Q77E**	1	2.17	**ORF9b:P3H**	1	1.45	**ORF9b:D66G**	3	0.28
**Maharashtra**							**ORF9b:N36Y**	1	1.45	**ORF9b:H9Q**	3	0.28
**Maharashtra**							**ORF9b:I19V**	1	1.45	**ORF9b:D89G**	2	0.18
**Maharashtra**							**ORF9b:M26I**	1	1.45	**ORF9b:V15L**	2	0.18
**Maharashtra**							**ORF9b:A57V**	1	1.45	**ORF9b:E7Q**	2	0.18
**Maharashtra**							**ORF9b:D89E**	1	1.45	**ORF9b:P17H**	2	0.18
**Maharashtra**							**ORF9b:S6N**	1	1.45	**ORF9b:G38V**	2	0.18
**Maharashtra**							**ORF9b:E86D**	1	1.45	**ORF9b:K80E**	2	0.18
**Maharashtra**							**ORF9b:A11V**	1	1.45	**ORF9b:A75V**	2	0.18
**Maharashtra**										**ORF9b:P10L**	2	0.18
**Maharashtra**										**ORF9b:Q70L**	2	0.18
**Maharashtra**										**ORF9b:Q18***	2	0.18
**Maharashtra**										**ORF9b:S6I**	2	0.18
**Maharashtra**										**ORF9b:P39L**	2	0.18
**Maharashtra**										**ORF9b:G38C**	2	0.18
**Maharashtra**										**ORF9b:D33Y**	2	0.18
**Maharashtra**										**ORF9b:M26T**	2	0.18
**Maharashtra**										**ORF9b:V41F**	1	0.09
**Maharashtra**										**ORF9b:Q77H**	1	0.09
**Maharashtra**										**ORF9b:K67R**	1	0.09
**Maharashtra**										**ORF9b:Q18H**	1	0.09
**Maharashtra**										**ORF9b:L87I**	1	0.09
**Maharashtra**										**ORF9b:R13H**	1	0.09
**Maharashtra**										**ORF9b:S53***	1	0.09
**Maharashtra**										**ORF9b:L54F**	1	0.09
**Maharashtra**										**ORF9b:N55V**	1	0.09
**Maharashtra**										**ORF9b:I45T**	1	0.09
**Maharashtra**										**ORF9b:G49H**	1	0.09
**Maharashtra**										**ORF9b:A82V**	1	0.09
**Maharashtra**										**ORF9b:A22S**	1	0.09
**Maharashtra**										**ORF9b:D89C**	1	0.09
**Maharashtra**										**ORF9b:S6T**	1	0.09
**Maharashtra**										**ORF9b:L61S**	1	0.09
**Maharashtra**										**ORF9b:G31Y**	1	0.09
**Maharashtra**										**ORF9b:L48V**	1	0.09
**Maharashtra**										**ORF9b:G49N**	1	0.09
**Maharashtra**										**ORF9b:A22E**	1	0.09
**Maharashtra**										**ORF9b:V23G**	1	0.09
**Maharashtra**										**ORF9b:T24I**	1	0.09
**Maharashtra**										**ORF9b:T79A**	1	0.09
**Maharashtra**										**ORF9b:K80M**	1	0.09
**Maharashtra**										**ORF9b:A82S**	1	0.09
**Maharashtra**										**ORF9b:T83E**	1	0.09
**Maharashtra**										**ORF9b:T84S**	1	0.09
**Maharashtra**										**ORF9b:E85G**	1	0.09
**Maharashtra**										**ORF9b:L87P**	1	0.09
**Maharashtra**										**ORF9b:E90A**	1	0.09
**Maharashtra**										**ORF9b:F91V**	1	0.09
**Maharashtra**										**ORF9b:D33G**	1	0.09
**Maharashtra**										**ORF9b:M26A**	1	0.09
**Maharashtra**										**ORF9b:E27V**	1	0.09
**Maharashtra**										**ORF9b:A29Q**	1	0.09
**Maharashtra**										**ORF9b:P43A**	1	0.09
**Maharashtra**										**ORF9b:I44V**	1	0.09
**Maharashtra**										**ORF9b:I45L**	1	0.09
**Maharashtra**										**ORF9b:R47G**	1	0.09
**Maharashtra**										**ORF9b:K59T**	1	0.09
**Maharashtra**										**ORF9b:K4N**	1	0.09
**Maharashtra**										**ORF9b:D89V**	1	0.09

Maharashtra										ORF9b:L21M	1	0.09
Maharashtra										ORF9b:D2H	1	0.09
Maharashtra										ORF9b:T83I	1	0.09
Maharashtra										ORF9b:M8T	1	0.09
Maharashtra										ORF9b:A11S	1	0.09
Maharashtra										ORF9b:R32C	1	0.09
Maharashtra										ORF9b:*98R	1	0.09
Maharashtra										ORF9b:E7A	1	0.09
Maharashtra										ORF9b:S50L	1	0.09
Maharashtra										ORF9b:L14F	1	0.09
State/Gene	Amino acid deletions											
Maharashtra	Pre-Wave 1			Wave 1			Pre-Wave 2			Wave 2		
Maharashtra	Mutation	Count	percent	Mutation	Count	percent	Mutation	Count	percent	Mutation	Count	percent
Maharashtra	E:A36-	3	0.1	S:Y144-	7	0.47	S:Y144-	22	0.33	ORF8:D119-	268	0.35
Maharashtra	E:L37-	3	0.1	ORF1a:G82-	3	0.2	ORF3a:L3-	20	0.3	ORF8:F120-	268	0.35
Maharashtra	ORF1a:V84-	2	0.07	ORF1a:H83-	3	0.2	ORF3a:F4-	20	0.3	S:E156-	173	0.23
Maharashtra	ORF1a:T1788-	2	0.07	ORF1a:V84-	3	0.2	S:H69-	19	0.29	S:F157-	172	0.23
Maharashtra	ORF1a:C1789-	2	0.07	ORF1a:M85-	3	0.2	S:V70-	19	0.29	S:Y144-	63	0.08
Maharashtra	ORF1a:G1790-	2	0.07	ORF1a:V86-	3	0.2	ORF3a:M1-	19	0.29	S:N343-	41	0.05
Maharashtra	ORF1a:Q1800-	2	0.07	ORF3a:V255-	2	0.13	ORF3a:D2-	19	0.29	S:V350-	41	0.05
Maharashtra	ORF1a:E1801-	2	0.07	ORF7a:Q62-	2	0.13	ORF3a:V13-	19	0.29	S:Y351-	41	0.05
Maharashtra	ORF1a:S1802-	2	0.07	ORF7a:F63-	2	0.13	ORF3a:T14-	19	0.29	S:Y380-	41	0.05
Maharashtra	ORF1a:P1803-	2	0.07	ORF7a:A64-	2	0.13	ORF3a:L15-	19	0.29	S:G381-	41	0.05
Maharashtra	ORF1a:M1807-	2	0.07	ORF7a:F65-	2	0.13	ORF3a:K16-	19	0.29	S:F392-	40	0.05
Maharashtra	ORF1a:S1808-	2	0.07	ORF7a:A66-	2	0.13	ORF3a:Q17-	19	0.29	S:V395-	40	0.05
Maharashtra	ORF1a:A1809-	2	0.07	ORF7a:C67-	2	0.13	ORF3a:G18-	19	0.29	S:G257-	39	0.05
Maharashtra	ORF1a:P1810-	2	0.07	ORF7a:P68-	2	0.13	ORF3a:E19-	19	0.29	S:W258-	39	0.05
Maharashtra	ORF1a:P1811-	2	0.07	ORF7a:D69-	2	0.13	ORF3a:I35-	19	0.29	S:T259-	39	0.05
Maharashtra	ORF1a:A1812-	2	0.07	ORF7a:G70-	2	0.13	ORF3a:P36-	19	0.29	S:A260-	39	0.05
Maharashtra	ORF1a:Q1813-	2	0.07	ORF7a:V71-	2	0.13	ORF3a:I37-	19	0.29	S:G261-	39	0.05
Maharashtra	ORF1a:Y1814-	2	0.07	ORF7a:K72-	2	0.13	ORF3a:Q38-	19	0.29	S:A262-	39	0.05
Maharashtra	ORF1a:E1815-	2	0.07	ORF7a:H73-	2	0.13	ORF3a:A39-	19	0.29	S:A263-	39	0.05
Maharashtra	ORF1a:L1816-	2	0.07	ORF7a:V74-	2	0.13	ORF1a:S3675-	12	0.18	S:A264-	39	0.05
Maharashtra	ORF1a:K1817-	2	0.07	ORF7a:Y75-	2	0.13	ORF1a:G3676-	12	0.18	S:E281-	39	0.05
Maharashtra	ORF1a:F1821-	2	0.07	ORF7a:Q76-	2	0.13	ORF1a:F3677-	12	0.18	S:N282-	39	0.05
Maharashtra	ORF1a:T1822-	2	0.07	ORF7a:L77-	2	0.13	S:E156-	5	0.08	S:G283-	39	0.05
Maharashtra	ORF1a:C1823-	2	0.07	ORF7a:R78-	2	0.13	S:F157-	5	0.08	S:T284-	39	0.05
Maharashtra	ORF1a:A1824-	2	0.07	ORF7a:A79-	2	0.13	ORF1b:D1406-	4	0.06	S:I285-	39	0.05
Maharashtra	ORF1a:S1825-	2	0.07	N:K266-	2	0.13	ORF1b:F1422-	4	0.06	S:T286-	39	0.05
Maharashtra	ORF1a:E1826-	2	0.07	N:A267-	2	0.13	ORF1b:L1423-	4	0.06	S:V382-	39	0.05
Maharashtra	ORF1a:Y1827-	2	0.07	N:Y268-	2	0.13	ORF1b:T1424-	4	0.06	S:S383-	39	0.05
Maharashtra	ORF1a:T1828-	2	0.07	N:N269-	2	0.13	ORF1b:R1425-	4	0.06	S:P384-	39	0.05
Maharashtra	ORF1a:G1829-	2	0.07	N:V270-	2	0.13	ORF1b:P1437-	4	0.06	S:T393-	39	0.05
Maharashtra	ORF1a:C1847-	2	0.07	S:L141-	2	0.13	ORF1b:Y1438-	4	0.06	S:N394-	39	0.05
Maharashtra	ORF1a:S1857-	2	0.07	S:G142-	2	0.13	ORF1b:Q1441-	4	0.06	S:N165-	38	0.05
Maharashtra	ORF1a:E1858-	2	0.07	S:V143-	2	0.13	ORF1b:N1442-	4	0.06	S:C166-	38	0.05
Maharashtra	ORF1a:Y1859-	2	0.07	S:A243-	2	0.13	ORF1b:A1443-	4	0.06	S:T250-	38	0.05
Maharashtra	ORF1a:K1860-	2	0.07	N:A252-	1	0.07	ORF1b:V1444-	4	0.06	S:P251-	38	0.05
Maharashtra	ORF1a:G1861-	2	0.07	N:E253-	1	0.07	ORF1b:A1445-	4	0.06	S:Y265-	38	0.05
Maharashtra	ORF1a:P1862-	2	0.07	S:T376-	1	0.07	ORF1b:P1452-	4	0.06	S:A352-	38	0.05
Maharashtra	ORF1a:I1863-	2	0.07	S:F377-	1	0.07	ORF1b:T1453-	4	0.06	S:W353-	38	0.05
Maharashtra	ORF1a:T1864-	2	0.07	S:K378-	1	0.07	ORF1b:Q1454-	4	0.06	S:N437-	38	0.05
Maharashtra	ORF1a:D1865-	2	0.07	S:C379-	1	0.07	ORF1b:E1463-	4	0.06	S:S438-	38	0.05
Maharashtra	ORF1a:Y1873-	2	0.07	S:Y380-	1	0.07	ORF1b:Y1464-	4	0.06	S:N439-	38	0.05
Maharashtra	ORF1a:T1874-	2	0.07	S:G381-	1	0.07	ORF1b:D1465-	4	0.06	S:D467-	38	0.05
Maharashtra	ORF1a:T1875-	2	0.07	S:V382-	1	0.07	ORF1b:Y1466-	4	0.06	S:I468-	38	0.05
Maharashtra	ORF1a:D1885-	2	0.07	S:S383-	1	0.07	ORF1b:E1474-	4	0.06	S:H69-	37	0.05
Maharashtra	ORF1a:G1886-	2	0.07	S:P384-	1	0.07	ORF1b:T1475-	4	0.06	S:S256-	37	0.05
Maharashtra	ORF1a:V1887-	2	0.07	S:T385-	1	0.07	ORF1b:A1476-	4	0.06	S:F306-	37	0.05
Maharashtra	ORF1a:V1888-	2	0.07	E:*76-	1	0.07	ORF1b:H1477-	4	0.06	S:T307-	37	0.05
Maharashtra	ORF1a:C1889-	2	0.07	ORF3a:V97-	1	0.07	ORF1b:S1478-	4	0.06	S:P412-	37	0.05
Maharashtra	ORF1a:T1890-	2	0.07	ORF3a:A98-	1	0.07	ORF1b:C1479-	4	0.06	S:G413-	37	0.05
Maharashtra	ORF1a:E1891-	2	0.07	ORF3a:A99-	1	0.07	ORF1b:N1480-	4	0.06	S:D442-	37	0.05
Maharashtra	ORF1a:I1892-	2	0.07	ORF3a:G100-	1	0.07	ORF1b:V1481-	4	0.06	S:S443-	37	0.05
Maharashtra	ORF1a:D1893-	2	0.07	ORF3a:L101-	1	0.07	ORF1b:S1500-	4	0.06	S:Y508-	37	0.05
Maharashtra	ORF1a:P1894-	2	0.07	ORF3a:E102-	1	0.07	ORF1b:D1501-	4	0.06	S:R509-	37	0.05
Maharashtra	ORF1a:K1895-	2	0.07	ORF3a:A103-	1	0.07	ORF1b:R1502-	4	0.06	S:V510-	37	0.05
Maharashtra	ORF1a:L1896-	2	0.07	ORF3a:P104-	1	0.07	ORF1b:D1503-	4	0.06	S:H519-	37	0.05
Maharashtra	ORF1a:D1897-	2	0.07	ORF3a:F105-	1	0.07	ORF1b:L1504-	4	0.06	S:A520-	37	0.05
Maharashtra	ORF1a:N1898-	2	0.07	ORF3a:L106-	1	0.07	ORF1b:Y1505-	4	0.06	S:P521-	37	0.05
Maharashtra	ORF1a:Y1899-	2	0.07	ORF3a:Y107-	1	0.07	ORF1b:D1506-	4	0.06	S:A522-	37	0.05

Maharashtra	ORF1a:Y1900-	2	0.07	ORF3a:V112-	1	0.07	ORF1b:K1507-	4	0.06	S:T523-	37	0.05
Maharashtra	ORF1a:K1901-	2	0.07	ORF3a:V121-	1	0.07	ORF1b:L1508-	4	0.06	S:V524-	37	0.05
Maharashtra	ORF1a:K1902-	2	0.07	ORF3a:R122-	1	0.07	ORF1b:Q1509-	4	0.06	S:C525-	37	0.05
Maharashtra	ORF1a:F1925-	2	0.07	ORF3a:I123-	1	0.07	ORF1b:F1510-	4	0.06	S:G526-	37	0.05
Maharashtra	ORF1a:D1926-	2	0.07	ORF3a:I124-	1	0.07	ORF1b:P1544-	4	0.06	S:P527-	37	0.05
Maharashtra	ORF1a:N1927-	2	0.07	ORF3a:M125-	1	0.07	ORF1b:T1545-	4	0.06	S:K528-	37	0.05
Maharashtra	ORF1a:F1928-	2	0.07	ORF3a:R126-	1	0.07	ORF1b:Q1546-	4	0.06	S:G545-	37	0.05
Maharashtra	ORF1a:K1929-	2	0.07	ORF3a:L127-	1	0.07	ORF1b:A1547-	4	0.06	S:G548-	37	0.05
Maharashtra	ORF1a:F1930-	2	0.07	ORF3a:W128-	1	0.07	ORF1b:P1548-	4	0.06	S:Q563-	37	0.05
Maharashtra	ORF1a:V1931-	2	0.07	ORF3a:L129-	1	0.07	ORF1b:G1583-	4	0.06	S:Q564-	37	0.05
Maharashtra	ORF1a:C1932-	2	0.07	ORF3a:C130-	1	0.07	ORF1b:F1584-	4	0.06	S:F565-	37	0.05
Maharashtra	ORF1a:D1933-	2	0.07	ORF3a:W131-	1	0.07	ORF1b:K1585-	4	0.06	S:G566-	37	0.05
Maharashtra	ORF1a:N1934-	2	0.07	ORF3a:K132-	1	0.07	ORF1b:M1586-	4	0.06	S:P579-	37	0.05
Maharashtra	ORF1a:I1935-	2	0.07	ORF3a:C133-	1	0.07	ORF1b:N1587-	4	0.06	S:D586-	37	0.05
Maharashtra	ORF1a:K1936-	2	0.07	ORF3a:R134-	1	0.07	ORF1b:Y1588-	4	0.06	S:I587-	37	0.05
Maharashtra	ORF1a:T1958-	2	0.07	ORF3a:Y141-	1	0.07	ORF1b:Q1589-	4	0.06	S:P631-	37	0.05
Maharashtra	ORF1a:F1959-	2	0.07	ORF3a:D142-	1	0.07	ORF1b:V1590-	4	0.06	S:T632-	37	0.05
Maharashtra	ORF1a:F1960-	2	0.07	ORF3a:A143-	1	0.07	ORF1b:N1591-	4	0.06	S:W633-	37	0.05
Maharashtra	ORF1a:P1961-	2	0.07	ORF3a:N144-	1	0.07	ORF1b:G1592-	4	0.06	S:V70-	36	0.05
Maharashtra	ORF1a:D1962-	2	0.07	ORF3a:Y145-	1	0.07	ORF1b:Y1593-	4	0.06	S:R273-	36	0.05
Maharashtra	ORF1a:G1965-	2	0.07	ORF3a:F146-	1	0.07	ORF1b:P1594-	4	0.06	S:D389-	36	0.05
Maharashtra	ORF1a:D1966-	2	0.07	ORF3a:L147-	1	0.07	ORF1b:N1595-	4	0.06	S:Q414-	36	0.05
Maharashtra	ORF1a:V1967-	2	0.07	ORF3a:C148-	1	0.07	ORF1b:M1596-	4	0.06	S:T415-	36	0.05
Maharashtra	ORF1a:V1968-	2	0.07	ORF3a:W149-	1	0.07	ORF1b:F1597-	4	0.06	S:N440-	36	0.05
Maharashtra	ORF1a:A1969-	2	0.07	ORF1a:M1-	1	0.07	ORF1b:I1598-	4	0.06	S:K462-	36	0.05
Maharashtra	ORF1a:I1970-	2	0.07	ORF1a:E2-	1	0.07	ORF1b:T1599-	4	0.06	S:P463-	36	0.05
Maharashtra	ORF1a:D1971-	2	0.07	ORF1a:S3-	1	0.07	ORF1b:R1600-	4	0.06	S:F464-	36	0.05
Maharashtra	ORF1a:Y1972-	2	0.07	ORF1a:L4-	1	0.07	ORF1b:A1609-	4	0.06	S:E465-	36	0.05
Maharashtra	ORF1a:K1973-	2	0.07	ORF1a:V5-	1	0.07	ORF1b:W1610-	4	0.06	S:R466-	36	0.05
Maharashtra	ORF1a:H1974-	2	0.07	ORF1a:P6-	1	0.07	ORF1b:I1611-	4	0.06	S:P479-	36	0.05
Maharashtra	ORF1a:Y1975-	2	0.07	ORF1a:G7-	1	0.07	ORF1b:G1612-	4	0.06	S:A575-	36	0.05
Maharashtra	ORF1a:T1976-	2	0.07	ORF1a:F8-	1	0.07	ORF1b:F1613-	4	0.06	S:V576-	36	0.05
Maharashtra	ORF1a:P1977-	2	0.07	N:T271-	1	0.07	ORF1b:D1614-	4	0.06	S:R577-	36	0.05
Maharashtra	ORF1a:S1978-	2	0.07	N:Q272-	1	0.07	ORF1b:V1615-	4	0.06	S:D578-	36	0.05
Maharashtra	ORF1a:F1979-	2	0.07	N:A273-	1	0.07	ORF1b:E1616-	4	0.06	S:S591-	36	0.05
Maharashtra	ORF1a:K1980-	2	0.07	N:F274-	1	0.07	ORF1b:G1617-	4	0.06	S:F592-	36	0.05
Maharashtra	ORF1a:V1991-	2	0.07	N:G275-	1	0.07	ORF1b:C1618-	4	0.06	S:G593-	36	0.05
Maharashtra	ORF1a:W1992-	2	0.07	N:R276-	1	0.07	ORF1b:H1619-	4	0.06	S:G594-	36	0.05
Maharashtra	ORF1a:H1993-	2	0.07	N:R277-	1	0.07	ORF1b:A1620-	4	0.06	S:E619-	36	0.05
Maharashtra	ORF1a:V1994-	2	0.07	N:D297-	1	0.07	ORF1b:T1621-	4	0.06	S:V620-	36	0.05
Maharashtra	ORF1a:N1995-	2	0.07	N:Y298-	1	0.07	ORF1b:L1633-	4	0.06	S:P621-	36	0.05
Maharashtra	ORF1a:N1996-	2	0.07	N:K299-	1	0.07	ORF1b:G1634-	4	0.06	S:V622-	36	0.05
Maharashtra	ORF1a:A1997-	2	0.07	N:I304-	1	0.07	ORF1b:F1635-	4	0.06	S:A623-	36	0.05
Maharashtra	ORF1a:T1998-	2	0.07	N:A305-	1	0.07	ORF1b:S1636-	4	0.06	S:I624-	36	0.05
Maharashtra	ORF1a:N1999-	2	0.07	N:Q306-	1	0.07	ORF1b:T1637-	4	0.06	S:H625-	36	0.05
Maharashtra	ORF1a:K2000-	2	0.07	N:F307-	1	0.07	ORF1b:G1638-	4	0.06	S:T630-	36	0.05
Maharashtra	ORF1a:A2001-	2	0.07	N:A308-	1	0.07	ORF1b:V1639-	4	0.06	S:R634-	36	0.05
Maharashtra	ORF1a:T2002-	2	0.07	N:P309-	1	0.07	ORF1b:N1640-	4	0.06	S:V635-	36	0.05
Maharashtra	ORF1a:Y2003-	2	0.07	N:S310-	1	0.07	ORF1b:L1641-	4	0.06	S:S305-	35	0.05
Maharashtra	ORF1a:W2014-	2	0.07	N:A311-	1	0.07	ORF1b:V1642-	4	0.06	S:L441-	35	0.05
Maharashtra	ORF1a:S2015-	2	0.07	N:S312-	1	0.07	ORF1b:V1644-	4	0.06	S:T549-	35	0.05
Maharashtra	ORF1a:T2016-	2	0.07	N:A313-	1	0.07	ORF1b:P1645-	4	0.06	S:G550-	35	0.05
Maharashtra	ORF1a:K2017-	2	0.07	N:F314-	1	0.07	ORF1b:T1646-	4	0.06	S:P561-	35	0.05
Maharashtra	ORF1a:P2018-	2	0.07	N:F315-	1	0.07	ORF1b:G1647-	4	0.06	S:F562-	35	0.05
Maharashtra	ORF1a:V2019-	2	0.07	N:G316-	1	0.07	ORF1b:Y1648-	4	0.06	S:T588-	35	0.05
Maharashtra	ORF1a:E2020-	2	0.07	N:M317-	1	0.07	ORF1b:G1680-	4	0.06	S:P589-	35	0.05
Maharashtra	ORF1a:T2021-	2	0.07	N:S318-	1	0.07	ORF1b:L1681-	4	0.06	S:C590-	35	0.05
Maharashtra	ORF1a:S2022-	2	0.07	ORF1a:S2313-	1	0.07	ORF1b:P1682-	4	0.06	S:Q271-	34	0.04
Maharashtra	ORF1a:N2023-	2	0.07	ORF1a:F2314-	1	0.07	ORF1b:W1683-	4	0.06	S:P272-	34	0.04
Maharashtra	ORF1a:S2024-	2	0.07	ORF1a:K2315-	1	0.07	ORF1b:N1684-	4	0.06	S:T274-	34	0.04
Maharashtra	ORF1a:F2025-	2	0.07	ORF1a:W2316-	1	0.07	ORF1b:V1685-	4	0.06	S:T478-	34	0.04
Maharashtra	ORF1a:D2026-	2	0.07	ORF1a:D2317-	1	0.07	ORF1b:V1686-	4	0.06	S:N556-	34	0.04
Maharashtra	ORF1a:V2027-	2	0.07	ORF1a:L2318-	1	0.07	ORF1b:R1687-	4	0.06	S:Q613-	34	0.04
Maharashtra	ORF1a:S2030-	2	0.07	ORF1a:T2319-	1	0.07	ORF1b:I1688-	4	0.06	S:D614-	34	0.04
Maharashtra	ORF1a:E2031-	2	0.07	ORF1a:A2320-	1	0.07	ORF1b:K1689-	4	0.06	S:V615-	34	0.04
Maharashtra	ORF1a:D2032-	2	0.07	ORF1a:F2321-	1	0.07	ORF1b:I1690-	4	0.06	S:N616-	34	0.04
Maharashtra	ORF1a:A2033-	2	0.07	N:I94-	1	0.07	ORF1b:V1691-	4	0.06	S:Q787-	34	0.04
Maharashtra	ORF1a:Q2034-	2	0.07	N:R95-	1	0.07	ORF1b:D1703-	4	0.06	S:I788-	34	0.04
Maharashtra	ORF1a:G2035-	2	0.07	N:G96-	1	0.07	ORF1b:R1704-	4	0.06	S:Y789-	34	0.04
Maharashtra	ORF1a:M2036-	2	0.07	N:S105-	1	0.07	ORF1b:V1705-	4	0.06	S:P807-	34	0.04
Maharashtra	ORF1a:D2037-	2	0.07	N:P106-	1	0.07	ORF1b:V1706-	4	0.06	S:D808-	34	0.04
Maharashtra	ORF1a:N2038-	2	0.07	N:R107-	1	0.07	ORF1b:F1707-	4	0.06	S:P809-	34	0.04
Maharashtra	ORF1a:L2039-	2	0.07	N:W108-	1	0.07	ORF1b:V1708-	4	0.06	S:S810-	34	0.04

Maharashtra	ORF1a:A2040-	2	0.07	N:Y109-	1	0.07	ORF1b:L1709-	4	0.06	S:K811-	34	0.04
Maharashtra	ORF1a:C2041-	2	0.07	N:F110-	1	0.07	ORF1b:W1710-	4	0.06	S:P812-	34	0.04
Maharashtra	ORF1a:V2047-	2	0.07	N:Y111-	1	0.07	ORF1b:A1711-	4	0.06	S:S813-	34	0.04
Maharashtra	ORF1a:S2048-	2	0.07	N:Y112-	1	0.07	ORF1b:K1724-	4	0.06	S:I818-	34	0.04
Maharashtra	ORF1a:E2049-	2	0.07	N:L113-	1	0.07	ORF1b:I1725-	4	0.06	S:E819-	34	0.04
Maharashtra	ORF1a:E2050-	2	0.07	N:G114-	1	0.07	ORF1b:G1726-	4	0.06	S:K304-	33	0.04
Maharashtra	ORF1a:V2051-	2	0.07	N:T115-	1	0.07	ORF1b:P1727-	4	0.06	S:V551-	33	0.04
Maharashtra	ORF1a:V2052-	2	0.07	N:G116-	1	0.07	ORF1b:E1728-	4	0.06	S:L552-	33	0.04
Maharashtra	ORF1a:E2053-	2	0.07	N:P117-	1	0.07	ORF1b:R1729-	4	0.06	S:T553-	33	0.04
Maharashtra	ORF1a:N2054-	2	0.07	N:E118-	1	0.07	ORF1b:T1730-	4	0.06	S:E554-	33	0.04
Maharashtra	ORF1a:P2055-	2	0.07	N:A119-	1	0.07	ORF1b:D1746-	4	0.06	S:Y612-	33	0.04
Maharashtra	ORF1a:T2056-	2	0.07	N:G120-	1	0.07	ORF1b:T1747-	4	0.06	S:C671-	33	0.04
Maharashtra	ORF1a:I2057-	2	0.07	N:K127-	1	0.07	ORF1b:Y1748-	4	0.06	S:A672-	33	0.04
Maharashtra	ORF1a:Q2058-	2	0.07	N:D128-	1	0.07	ORF1b:A1749-	4	0.06	S:Y674-	33	0.04
Maharashtra	ORF1a:K2059-	2	0.07	N:G129-	1	0.07	ORF1b:C1750-	4	0.06	S:Q675-	33	0.04
Maharashtra	ORF1a:D2060-	2	0.07	N:I130-	1	0.07	ORF1b:W1751-	4	0.06	S:K790-	33	0.04
Maharashtra	ORF1a:V2061-	2	0.07	N:I131-	1	0.07	ORF1b:H1752-	4	0.06	S:T791-	33	0.04
Maharashtra	ORF1a:L2077-	2	0.07	N:W132-	1	0.07	ORF1b:H1753-	4	0.06	S:P792-	33	0.04
Maharashtra	ORF1a:K2078-	2	0.07	N:V133-	1	0.07	ORF1b:S1754-	4	0.06	S:P793-	33	0.04
Maharashtra	ORF1a:P2079-	2	0.07	N:A134-	1	0.07	ORF1b:I1755-	4	0.06	S:I794-	33	0.04
Maharashtra	ORF1a:A2080-	2	0.07	N:T135-	1	0.07	ORF1b:G1756-	4	0.06	S:K795-	33	0.04
Maharashtra	ORF1a:N2081-	2	0.07	N:D144-	1	0.07	ORF1b:P1763-	4	0.06	S:D796-	33	0.04
Maharashtra	ORF1a:N2082-	2	0.07	N:H145-	1	0.07	ORF1b:I1766-	4	0.06	S:D820-	33	0.04
Maharashtra	ORF1a:S2083-	2	0.07	N:I146-	1	0.07	ORF1b:D1767-	4	0.06	S:A829-	33	0.04
Maharashtra	ORF1a:L2084-	2	0.07	N:G147-	1	0.07	ORF1b:V1768-	4	0.06	S:D830-	33	0.04
Maharashtra	ORF1a:K2085-	2	0.07	N:T148-	1	0.07	ORF1b:Q1769-	4	0.06	S:A831-	33	0.04
Maharashtra	ORF1a:I2086-	2	0.07	N:N153-	1	0.07	ORF1b:Q1770-	4	0.06	S:G832-	33	0.04
Maharashtra	ORF1a:T2087-	2	0.07	N:N154-	1	0.07	ORF1b:W1771-	4	0.06	S:F833-	33	0.04
Maharashtra	ORF1a:E2088-	2	0.07	N:A155-	1	0.07	ORF1b:D1797-	4	0.06	S:I834-	33	0.04
Maharashtra	ORF1a:E2089-	2	0.07	N:A156-	1	0.07	ORF1b:H1807-	4	0.06	S:F888-	33	0.04
Maharashtra	ORF1a:A2097-	2	0.07	N:I157-	1	0.07	ORF1b:E1808-	4	0.06	S:G889-	33	0.04
Maharashtra	ORF1a:A2098-	2	0.07	N:V158-	1	0.07	ORF1b:C1809-	4	0.06	S:A890-	33	0.04
Maharashtra	ORF1a:Y2099-	2	0.07	N:L159-	1	0.07	ORF1b:F1810-	4	0.06	S:G891-	33	0.04
Maharashtra	ORF1a:V2100-	2	0.07	N:Q160-	1	0.07	ORF1b:V1811-	4	0.06	S:A892-	33	0.04
Maharashtra	ORF1a:D2101-	2	0.07	N:L161-	1	0.07	ORF1b:P1821-	4	0.06	S:A893-	33	0.04
Maharashtra	ORF1a:N2102-	2	0.07	N:P162-	1	0.07	ORF1b:I1822-	4	0.06	S:L894-	33	0.04
Maharashtra	ORF1a:S2103-	2	0.07	N:Q163-	1	0.07	ORF1b:I1823-	4	0.06	S:Q895-	33	0.04
Maharashtra	ORF1a:S2104-	2	0.07	N:G164-	1	0.07	ORF1b:G1824-	4	0.06	S:M900-	33	0.04
Maharashtra	ORF1a:K2120-	2	0.07	N:G178-	1	0.07	ORF1b:V1836-	4	0.06	S:Q901-	33	0.04
Maharashtra	ORF1a:T2121-	2	0.07	N:G179-	1	0.07	ORF1b:Q1837-	4	0.06	S:M902-	33	0.04
Maharashtra	ORF1a:L2122-	2	0.07	N:S180-	1	0.07	ORF1b:H1838-	4	0.06	S:A903-	33	0.04
Maharashtra	ORF1a:A2123-	2	0.07	N:Q181-	1	0.07	ORF1b:M1839-	4	0.06	S:Y904-	33	0.04
Maharashtra	ORF1a:T2124-	2	0.07	N:A182-	1	0.07	ORF1b:V1840-	4	0.06	S:R905-	33	0.04
Maharashtra	ORF1a:H2125-	2	0.07	N:S183-	1	0.07	ORF1b:V1841-	4	0.06	S:F906-	33	0.04
Maharashtra	ORF1a:G2126-	2	0.07	N:S184-	1	0.07	ORF1b:K1842-	4	0.06	S:Q926-	33	0.04
Maharashtra	ORF1a:N2131-	2	0.07	N:S202-	1	0.07	ORF1b:A1843-	4	0.06	S:F927-	33	0.04
Maharashtra	ORF1a:S2132-	2	0.07	N:R203-	1	0.07	ORF1b:A1844-	4	0.06	S:N928-	33	0.04
Maharashtra	ORF1a:V2133-	2	0.07	N:G204-	1	0.07	ORF1b:L1845-	4	0.06	S:S929-	33	0.04
Maharashtra	ORF1a:P2134-	2	0.07	N:T205-	1	0.07	ORF1b:L1846-	4	0.06	S:A930-	33	0.04
Maharashtra	ORF1a:W2135-	2	0.07	N:S206-	1	0.07	ORF1b:A1847-	4	0.06	S:I931-	33	0.04
Maharashtra	ORF1a:D2136-	2	0.07	N:P207-	1	0.07	ORF1b:D1848-	4	0.06	S:G932-	33	0.04
Maharashtra	ORF1a:T2137-	2	0.07	N:A208-	1	0.07	ORF1b:K1849-	4	0.06	S:K933-	33	0.04
Maharashtra	ORF1a:I2138-	2	0.07	N:R209-	1	0.07	ORF1b:F1850-	4	0.06	S:I934-	33	0.04
Maharashtra	ORF1a:A2139-	2	0.07	N:M210-	1	0.07	ORF1b:P1851-	4	0.06	S:Q935-	33	0.04
Maharashtra	ORF1a:N2140-	2	0.07	N:L219-	1	0.07	ORF1b:G1857-	4	0.06	S:D936-	33	0.04
Maharashtra	ORF1a:Y2141-	2	0.07	N:A220-	1	0.07	ORF1b:N1858-	4	0.06	ORF1a:G3676-	32	0.04
Maharashtra	ORF1a:A2142-	2	0.07	N:L221-	1	0.07	ORF1b:P1859-	4	0.06	ORF1a:F3677-	32	0.04
Maharashtra	ORF1a:K2143-	2	0.07	N:L222-	1	0.07	ORF1b:K1860-	4	0.06	S:G476-	32	0.04
Maharashtra	ORF1a:T2154-	2	0.07	N:L223-	1	0.07	ORF1b:A1861-	4	0.06	S:C480-	32	0.04
Maharashtra	ORF1a:N2155-	2	0.07	N:L224-	1	0.07	ORF1b:I1862-	4	0.06	S:G700-	32	0.04
Maharashtra	ORF1a:I2156-	2	0.07	N:D225-	1	0.07	ORF1b:K1863-	4	0.06	S:A701-	32	0.04
Maharashtra	ORF1a:V2157-	2	0.07	N:R226-	1	0.07	ORF1b:C1864-	4	0.06	S:E702-	32	0.04
Maharashtra	ORF1a:T2158-	2	0.07	N:L227-	1	0.07	ORF1b:V1865-	4	0.06	S:N703-	32	0.04
Maharashtra	ORF1a:R2159-	2	0.07	N:S232-	1	0.07	ORF1b:P1866-	4	0.06	S:S704-	32	0.04
Maharashtra	ORF1a:K2191-	2	0.07	N:K233-	1	0.07	ORF1b:Q1867-	4	0.06	S:V705-	32	0.04
Maharashtra	ORF1a:A2192-	2	0.07	N:M234-	1	0.07	ORF1b:A1868-	4	0.06	S:A706-	32	0.04
Maharashtra	ORF1a:S2193-	2	0.07	N:S235-	1	0.07	ORF1b:D1869-	4	0.06	S:Y707-	32	0.04
Maharashtra	ORF1a:M2254-	2	0.07	N:G236-	1	0.07	ORF1b:E1888-	4	0.06	S:S708-	32	0.04
Maharashtra	ORF1a:S2255-	2	0.07	N:K237-	1	0.07	ORF1b:D1899-	4	0.06	S:N709-	32	0.04
Maharashtra	ORF1a:N2256-	2	0.07	N:G238-	1	0.07	ORF1b:K1900-	4	0.06	S:C743-	32	0.04
Maharashtra	ORF1a:L2257-	2	0.07	N:Q239-	1	0.07	ORF1b:F1901-	4	0.06	S:G744-	32	0.04
Maharashtra	ORF1a:G2258-	2	0.07	N:Q240-	1	0.07	ORF1b:T1902-	4	0.06	S:D745-	32	0.04
Maharashtra	ORF1a:M2259-	2	0.07	N:Q241-	1	0.07	ORF1b:D1903-	4	0.06	S:S746-	32	0.04

Maharashtra	ORF1a:P2260-	2	0.07	N:Q242-	1	0.07	S:Y145-	3	0.05	S:T747-	32	0.04
Maharashtra	ORF1a:S2261-	2	0.07	N:G243-	1	0.07	S:H146-	3	0.05	S:E748-	32	0.04
Maharashtra	ORF1a:Y2262-	2	0.07	N:Q244-	1	0.07	S:S151-	3	0.05	S:T859-	32	0.04
Maharashtra	ORF1a:C2263-	2	0.07	N:P258-	1	0.07	ORF1b:D1389-	3	0.05	S:V860-	32	0.04
Maharashtra	ORF1a:T2264-	2	0.07	N:R259-	1	0.07	ORF1b:K1390-	3	0.05	S:L861-	32	0.04
Maharashtra	ORF1a:G2265-	2	0.07	N:Q260-	1	0.07	ORF1b:S1391-	3	0.05	S:P862-	32	0.04
Maharashtra	ORF1a:Y2266-	2	0.07	N:K261-	1	0.07	ORF1b:A1392-	3	0.05	S:P863-	32	0.04
Maharashtra	ORF1a:R2267-	2	0.07	N:R262-	1	0.07	ORF1b:Q1393-	3	0.05	S:L864-	32	0.04
Maharashtra	ORF1a:E2268-	2	0.07	N:T263-	1	0.07	ORF1b:V1407-	3	0.05	S:L865-	32	0.04
Maharashtra	ORF1a:G2269-	2	0.07	N:A264-	1	0.07	ORF1b:S1408-	3	0.05	S:T167-	31	0.04
Maharashtra	ORF1a:S2303-	2	0.07	N:T265-	1	0.07	ORF1b:S1409-	3	0.05	S:V171-	31	0.04
Maharashtra	ORF1a:L2304-	2	0.07	N:E280-	1	0.07	ORF1b:A1410-	3	0.05	S:S172-	31	0.04
Maharashtra	ORF1a:E2305-	2	0.07	N:Q281-	1	0.07	ORF1b:I1411-	3	0.05	S:Q173-	31	0.04
Maharashtra	ORF1a:T2306-	2	0.07	N:T282-	1	0.07	ORF1b:N1412-	3	0.05	S:P217-	31	0.04
Maharashtra	ORF1a:I2307-	2	0.07	N:Q283-	1	0.07	ORF1b:R1413-	3	0.05	S:P426-	31	0.04
Maharashtra	ORF1a:Q2308-	2	0.07	ORF1b:G670-	1	0.07	ORF1b:P1414-	3	0.05	S:N710-	31	0.04
Maharashtra	ORF1a:I2309-	2	0.07	ORF1b:T671-	1	0.07	ORF1b:Q1415-	3	0.05	S:Y741-	31	0.04
Maharashtra	ORF1a:T2310-	2	0.07	ORF1b:S672-	1	0.07	ORF1b:I1416-	3	0.05	S:I742-	31	0.04
Maharashtra	ORF1a:I2311-	2	0.07	ORF1b:S673-	1	0.07	ORF1b:G1417-	3	0.05	S:L821-	31	0.04
Maharashtra	ORF1a:S2312-	2	0.07	ORF1b:G674-	1	0.07	ORF1b:V1418-	3	0.05	S:L822-	31	0.04
Maharashtra	ORF1a:S2313-	2	0.07	ORF1b:D675-	1	0.07	ORF1b:V1419-	3	0.05	S:F823-	31	0.04
Maharashtra	ORF1a:F2314-	2	0.07	ORF1b:A676-	1	0.07	ORF1b:R1420-	3	0.05	S:N824-	31	0.04
Maharashtra	ORF1a:K2315-	2	0.07	ORF1b:T677-	1	0.07	ORF1b:E1421-	3	0.05	S:Q607-	31	0.04
Maharashtra	ORF1a:W2316-	2	0.07	ORF1b:T678-	1	0.07	ORF1b:N1439-	3	0.05	S:F168-	30	0.04
Maharashtra	ORF1a:D2317-	2	0.07	ORF1b:A679-	1	0.07	ORF1b:S1440-	3	0.05	S:Q218-	30	0.04
Maharashtra	ORF1a:L2318-	2	0.07	ORF1b:Y680-	1	0.07	ORF1a:A537-	3	0.05	S:S477-	30	0.04
Maharashtra	ORF1a:T2319-	2	0.07	ORF1b:A681-	1	0.07	ORF1a:S538-	3	0.05	S:S673-	30	0.04
Maharashtra	ORF1a:V2324-	2	0.07	ORF1b:N682-	1	0.07	ORF1a:E539-	3	0.05	S:S711-	30	0.04
Maharashtra	ORF1a:A2325-	2	0.07	ORF1b:S683-	1	0.07	ORF1a:A540-	3	0.05	S:K310-	30	0.04
Maharashtra	ORF1a:E2326-	2	0.07	ORF1b:V684-	1	0.07	ORF1a:A541-	3	0.05	S:G311-	30	0.04
Maharashtra	ORF1a:W2327-	2	0.07	ORF1b:F685-	1	0.07	ORF1a:R542-	3	0.05	S:I312-	30	0.04
Maharashtra	ORF1a:F2328-	2	0.07	ORF1b:N686-	1	0.07	ORF1a:V543-	3	0.05	S:Y313-	30	0.04
Maharashtra	ORF1a:L2349-	2	0.07	ORF1b:I687-	1	0.07	ORF1a:V544-	3	0.05	S:Q314-	30	0.04
Maharashtra	ORF1a:F2350-	2	0.07	ORF1b:C688-	1	0.07	ORF1a:R545-	3	0.05	S:T315-	30	0.04
Maharashtra	ORF1a:F2351-	2	0.07	ORF1b:Q689-	1	0.07	ORF1a:Q563-	3	0.05	S:Q580-	30	0.04
Maharashtra	ORF1a:S2352-	2	0.07	ORF1b:A690-	1	0.07	ORF1a:K564-	3	0.05	S:A626-	30	0.04
Maharashtra	ORF1a:Y2353-	2	0.07	ORF1b:V691-	1	0.07	ORF1a:A565-	3	0.05	S:L629-	30	0.04
Maharashtra	ORF1a:F2354-	2	0.07	ORF1b:T692-	1	0.07	ORF1a:A566-	3	0.05	S:E169-	29	0.04
Maharashtra	ORF1a:A2355-	2	0.07	ORF1b:A693-	1	0.07	ORF1a:I567-	3	0.05	S:Y170-	29	0.04
Maharashtra	ORF1a:V2356-	2	0.07	ORF1b:N694-	1	0.07	ORF1a:T568-	3	0.05	S:N360-	29	0.04
Maharashtra	ORF1a:H2357-	2	0.07	ORF1b:V695-	1	0.07	ORF1a:I569-	3	0.05	S:C361-	29	0.04
Maharashtra	ORF1a:F2358-	2	0.07	ORF1b:N696-	1	0.07	ORF1a:L570-	3	0.05	S:V362-	29	0.04
Maharashtra	ORF1a:I2359-	2	0.07	ORF1b:A697-	1	0.07	ORF1a:D571-	3	0.05	S:A363-	29	0.04
Maharashtra	ORF1a:S2360-	2	0.07	ORF1b:L698-	1	0.07	ORF1a:G572-	3	0.05	S:D364-	29	0.04
Maharashtra	ORF1a:N2361-	2	0.07	ORF1b:L699-	1	0.07	ORF1a:I573-	3	0.05	S:L828-	29	0.04
Maharashtra	ORF1a:S2362-	2	0.07	ORF1b:S700-	1	0.07	ORF1a:M598-	3	0.05	S:A344-	29	0.04
Maharashtra	ORF1a:W2363-	2	0.07	ORF1b:T701-	1	0.07	ORF1a:N615-	3	0.05	S:T345-	29	0.04
Maharashtra	ORF1a:Q2373-	2	0.07	ORF1b:D702-	1	0.07	ORF1a:I616-	3	0.05	S:R346-	29	0.04
Maharashtra	ORF1a:M2374-	2	0.07	ORF1b:G703-	1	0.07	ORF1a:F617-	3	0.05	S:F400-	29	0.04
Maharashtra	ORF1a:A2375-	2	0.07	ORF1b:N704-	1	0.07	ORF1a:G618-	3	0.05	S:V401-	29	0.04
Maharashtra	ORF1a:P2376-	2	0.07	ORF1b:K705-	1	0.07	ORF1a:T619-	3	0.05	S:I402-	29	0.04
Maharashtra	ORF1a:A2379-	2	0.07	ORF1b:I706-	1	0.07	ORF1a:V620-	3	0.05	S:R403-	29	0.04
Maharashtra	ORF1a:M2380-	2	0.07	ORF1b:A707-	1	0.07	ORF1a:Y621-	3	0.05	S:L546-	29	0.04
Maharashtra	S:Y144-	1	0.03	ORF1b:D708-	1	0.07	ORF1a:E622-	3	0.05	S:T547-	29	0.04
Maharashtra	ORF1a:G82-	1	0.03	ORF1b:K709-	1	0.07	ORF1a:K650-	3	0.05	S:T602-	29	0.04
Maharashtra	ORF1a:H83-	1	0.03	ORF1b:Y710-	1	0.07	ORF1a:F651-	3	0.05	S:N603-	29	0.04
Maharashtra	ORF1a:M1588-	1	0.03	ORF1b:V711-	1	0.07	ORF1a:I652-	3	0.05	S:T604-	29	0.04
Maharashtra	ORF1a:T1589-	1	0.03	ORF1b:R712-	1	0.07	ORF1a:S653-	3	0.05	S:S605-	29	0.04
Maharashtra	ORF1a:Y1590-	1	0.03	ORF1b:N713-	1	0.07	ORF1a:T654-	3	0.05	S:N606-	29	0.04
Maharashtra	ORF1a:G1591-	1	0.03	ORF1b:L714-	1	0.07	ORF1a:C655-	3	0.05	S:V608-	29	0.04
Maharashtra	ORF1a:Q1592-	1	0.03	ORF1b:Q715-	1	0.07	ORF1a:R724-	3	0.05	S:A609-	29	0.04
Maharashtra	ORF1a:Q1593-	1	0.03	ORF1b:H716-	1	0.07	ORF1a:E725-	3	0.05	S:V610-	29	0.04
Maharashtra	ORF1a:F1594-	1	0.03	ORF1b:R717-	1	0.07	ORF1a:E726-	3	0.05	S:D627-	29	0.04
Maharashtra	ORF1a:G1595-	1	0.03	ORF1b:L718-	1	0.07	ORF1a:T727-	3	0.05	S:Q628-	29	0.04
Maharashtra	ORF1a:P1596-	1	0.03	ORF1b:Y719-	1	0.07	ORF1a:G728-	3	0.05	ORF1a:S3675-	28	0.04
Maharashtra	ORF1a:T1597-	1	0.03	ORF1b:E720-	1	0.07	ORF1a:E754-	3	0.05	S:P174-	28	0.04
Maharashtra	ORF1a:Y1598-	1	0.03	ORF1b:C721-	1	0.07	ORF1a:E755-	3	0.05	S:P330-	28	0.04
Maharashtra	ORF1a:L1599-	1	0.03	ORF1b:L722-	1	0.07	ORF1a:V756-	3	0.05	S:V308-	28	0.04
Maharashtra	ORF1a:D1600-	1	0.03	ORF1b:Y723-	1	0.07	ORF1a:E767-	3	0.05	S:T376-	28	0.04
Maharashtra	ORF1a:G1601-	1	0.03	ORF1b:R724-	1	0.07	ORF1a:Q768-	3	0.05	S:F377-	28	0.04
Maharashtra	ORF1a:A1602-	1	0.03	ORF1b:N725-	1	0.07	ORF1a:P769-	3	0.05	S:K378-	28	0.04
Maharashtra	ORF1a:L1627-	1	0.03	ORF1b:R726-	1	0.07	ORF1a:I785-	3	0.05	S:C379-	28	0.04
Maharashtra	ORF1a:R1628-	1	0.03	ORF1b:D727-	1	0.07	ORF1a:N786-	3	0.05	S:Y396-	28	0.04

Maharashtra	ORF1a:V1629-	1	0.03	ORF1b:V728-	1	0.07	ORF1a:G787-	3	0.05	S:A397-	28	0.04
Maharashtra	ORF1a:E1630-	1	0.03	ORF1b:D729-	1	0.07	ORF1a:L788-	3	0.05	S:D398-	28	0.04
Maharashtra	ORF1a:A1631-	1	0.03	ORF1b:E735-	1	0.07	ORF1a:M789-	3	0.05	S:S399-	28	0.04
Maharashtra	ORF1a:F1632-	1	0.03	ORF1b:F736-	1	0.07	ORF1a:L790-	3	0.05	S:K444-	28	0.04
Maharashtra	ORF1a:E1633-	1	0.03	ORF1b:Y737-	1	0.07	ORF1a:L791-	3	0.05	S:V445-	28	0.04
Maharashtra	ORF1a:Y1634-	1	0.03	ORF1b:A738-	1	0.07	ORF1a:E792-	3	0.05	S:S469-	28	0.04
Maharashtra	ORF1a:Y1635-	1	0.03	ORF1b:Y739-	1	0.07	ORF1a:I793-	3	0.05	S:Q474-	28	0.04
Maharashtra	ORF1a:H1636-	1	0.03	ORF1b:L740-	1	0.07	ORF1a:K794-	3	0.05	S:A475-	28	0.04
Maharashtra	ORF1a:D1639-	1	0.03	ORF1b:R741-	1	0.07	ORF1a:V830-	3	0.05	S:G667-	28	0.04
Maharashtra	ORF1a:P1640-	1	0.03	ORF1b:K742-	1	0.07	ORF1a:I831-	3	0.05	S:A668-	28	0.04
Maharashtra	ORF1a:S1641-	1	0.03	ORF1b:H743-	1	0.07	ORF1a:E832-	3	0.05	S:G669-	28	0.04
Maharashtra	ORF1a:F1642-	1	0.03	ORF1b:F744-	1	0.07	ORF1a:V833-	3	0.05	S:I670-	28	0.04
Maharashtra	ORF1a:L1643-	1	0.03	ORF1b:S745-	1	0.07	ORF1a:F843-	3	0.05	ORF1a:F2908-	27	0.04
Maharashtra	ORF1a:G1644-	1	0.03	ORF1b:M746-	1	0.07	ORF1a:E844-	3	0.05	ORF1a:P2965-	27	0.04
Maharashtra	ORF1a:R1645-	1	0.03	ORF1b:M747-	1	0.07	ORF1a:L845-	3	0.05	ORF1a:N2966-	27	0.04
Maharashtra	ORF1a:Y1646-	1	0.03	ORF1b:I748-	1	0.07	ORF1a:D846-	3	0.05	ORF1a:T2967-	27	0.04
Maharashtra	ORF1a:L1664-	1	0.03	ORF1b:L749-	1	0.07	ORF1a:E847-	3	0.05	ORF1a:I3126-	27	0.04
Maharashtra	ORF1a:T1665-	1	0.03	ORF1b:S750-	1	0.07	ORF1a:E855-	3	0.05	ORF1a:E3176-	27	0.04
Maharashtra	ORF1a:S1666-	1	0.03	ORF1b:D751-	1	0.07	ORF1a:K856-	3	0.05	ORF1a:E3177-	27	0.04
Maharashtra	ORF1a:I1667-	1	0.03	ORF1b:D752-	1	0.07	ORF1a:C857-	3	0.05	ORF1a:A3178-	27	0.04
Maharashtra	ORF1a:K1668-	1	0.03	ORF1b:A753-	1	0.07	ORF1a:S858-	3	0.05	ORF1a:A3179-	27	0.04
Maharashtra	ORF1a:W1669-	1	0.03	ORF1b:V754-	1	0.07	ORF1a:A859-	3	0.05	ORF1a:K3215-	27	0.04
Maharashtra	ORF1a:A1670-	1	0.03	ORF1b:V755-	1	0.07	ORF1a:Y860-	3	0.05	ORF1a:Y3216-	27	0.04
Maharashtra	ORF1a:D1671-	1	0.03	ORF1b:C756-	1	0.07	ORF1a:T861-	3	0.05	ORF1a:F3217-	27	0.04
Maharashtra	ORF1a:N1672-	1	0.03	ORF1b:F757-	1	0.07	ORF1a:V862-	3	0.05	ORF1a:S3218-	27	0.04
Maharashtra	ORF1a:N1673-	1	0.03	ORF1b:N758-	1	0.07	ORF1a:E863-	3	0.05	ORF1a:G3219-	27	0.04
Maharashtra	ORF1a:C1674-	1	0.03	ORF1b:S759-	1	0.07	ORF1a:L864-	3	0.05	S:P337-	27	0.04
Maharashtra	ORF1a:K1689-	1	0.03	ORF1b:T760-	1	0.07	ORF1a:A967-	3	0.05	S:F338-	27	0.04
Maharashtra	ORF1a:F1690-	1	0.03	ORF1b:Y761-	1	0.07	ORF1a:A968-	3	0.05	S:G339-	27	0.04
Maharashtra	ORF1a:N1691-	1	0.03	ORF1b:A762-	1	0.07	ORF1a:Q970-	3	0.05	S:E340-	27	0.04
Maharashtra	ORF1a:P1692-	1	0.03	ORF1b:S763-	1	0.07	ORF1a:P971-	3	0.05	S:V341-	27	0.04
Maharashtra	ORF1a:P1693-	1	0.03	ORF1b:Q764-	1	0.07	ORF1a:E972-	3	0.05	S:F342-	27	0.04
Maharashtra	ORF1a:A1694-	1	0.03	ORF1b:G765-	1	0.07	ORF1a:E973-	3	0.05	S:N354-	27	0.04
Maharashtra	ORF1a:L1695-	1	0.03	ORF1b:L766-	1	0.07	ORF1a:E974-	3	0.05	S:R355-	27	0.04
Maharashtra	ORF1a:Q1696-	1	0.03	ORF1b:V767-	1	0.07	ORF1a:Q975-	3	0.05	S:K356-	27	0.04
Maharashtra	ORF1a:D1697-	1	0.03	ORF1b:A768-	1	0.07	ORF1a:E976-	3	0.05	S:R357-	27	0.04
Maharashtra	ORF1a:A1698-	1	0.03	ORF1b:S769-	1	0.07	ORF1a:E977-	3	0.05	S:I358-	27	0.04
Maharashtra	ORF1a:A1707-	1	0.03	ORF1b:I770-	1	0.07	ORF1a:D978-	3	0.05	S:S359-	27	0.04
Maharashtra	ORF1a:A1708-	1	0.03	ORF1b:K771-	1	0.07	ORF1a:W979-	3	0.05	S:R567-	27	0.04
Maharashtra	ORF1a:N1709-	1	0.03	ORF1b:N772-	1	0.07	ORF1a:D981-	3	0.05	S:D568-	27	0.04
Maharashtra	ORF1a:F1710-	1	0.03	ORF1b:F773-	1	0.07	ORF1a:D982-	3	0.05	S:S937-	27	0.04
Maharashtra	ORF1a:C1711-	1	0.03	ORF1b:K774-	1	0.07	ORF1a:D983-	3	0.05	S:L938-	27	0.04
Maharashtra	ORF1a:A1712-	1	0.03	ORF1b:S775-	1	0.07	ORF1a:S984-	3	0.05	S:S939-	27	0.04
Maharashtra	ORF1a:L1713-	1	0.03	ORF1b:V776-	1	0.07	ORF1a:Q985-	3	0.05	S:S940-	27	0.04
Maharashtra	ORF1a:I1714-	1	0.03	ORF1b:L777-	1	0.07	ORF1a:Q986-	3	0.05	S:G404-	27	0.04
Maharashtra	ORF1a:L1715-	1	0.03	ORF1b:Y778-	1	0.07	ORF1a:E1013-	3	0.05	S:D405-	27	0.04
Maharashtra	ORF1a:A1716-	1	0.03	ORF1b:Y779-	1	0.07	ORF1a:M1014-	3	0.05	S:V511-	27	0.04
Maharashtra	ORF1a:Y1717-	1	0.03	ORF1b:Q780-	1	0.07	ORF1a:E1015-	3	0.05	S:L518-	27	0.04
Maharashtra	ORF1a:C1718-	1	0.03	ORF1b:N781-	1	0.07	ORF1a:L1016-	3	0.05	S:T581-	27	0.04
Maharashtra	ORF1a:N1719-	1	0.03	ORF1b:N782-	1	0.07	ORF1a:P1018-	3	0.05	S:L582-	27	0.04
Maharashtra	ORF1a:R1729-	1	0.03	ORF1b:V783-	1	0.07	ORF1a:V1019-	3	0.05	S:E583-	27	0.04
Maharashtra	ORF1a:E1730-	1	0.03	ORF1b:F784-	1	0.07	ORF1a:V1020-	3	0.05	S:I584-	27	0.04
Maharashtra	ORF1a:T1731-	1	0.03	ORF1b:M785-	1	0.07	ORF1a:A1043-	3	0.05	S:L585-	27	0.04
Maharashtra	ORF1a:M1732-	1	0.03	ORF1b:S786-	1	0.07	ORF1a:D1044-	3	0.05	S:V656-	27	0.04
Maharashtra	ORF1a:S1733-	1	0.03	ORF1b:E787-	1	0.07	ORF1a:I1045-	3	0.05	S:N657-	27	0.04
Maharashtra	ORF1a:Y1734-	1	0.03	ORF1b:A788-	1	0.07	ORF1a:V1046-	3	0.05	S:N658-	27	0.04
Maharashtra	ORF1a:L1735-	1	0.03	ORF1b:K789-	1	0.07	ORF1a:E1047-	3	0.05	S:S659-	27	0.04
Maharashtra	ORF1a:F1736-	1	0.03	ORF1b:C790-	1	0.07	ORF1a:E1048-	3	0.05	S:Y660-	27	0.04
Maharashtra	ORF1a:Q1737-	1	0.03	ORF1b:W791-	1	0.07	ORF1a:A1049-	3	0.05	S:E661-	27	0.04
Maharashtra	ORF1a:H1738-	1	0.03	ORF1b:T792-	1	0.07	ORF1a:V1056-	3	0.05	ORF1a:N2530-	26	0.03
Maharashtra	ORF1a:A1739-	1	0.03	ORF1b:E793-	1	0.07	ORF1a:V1057-	3	0.05	ORF1a:N2531-	26	0.03
Maharashtra	ORF1a:N1740-	1	0.03	ORF1b:T794-	1	0.07	ORF1a:V1058-	3	0.05	ORF1a:E2904-	26	0.03
Maharashtra	ORF1a:L1741-	1	0.03	ORF1b:D795-	1	0.07	ORF1a:A1078-	3	0.05	ORF1a:Y2905-	26	0.03
Maharashtra	ORF1a:D1742-	1	0.03	ORF1b:L796-	1	0.07	ORF1a:T1079-	3	0.05	ORF1a:T2906-	26	0.03
Maharashtra	ORF1a:V1750-	1	0.03	ORF1b:T797-	1	0.07	ORF1a:N1080-	3	0.05	ORF1a:D2907-	26	0.03
Maharashtra	ORF1a:V1751-	1	0.03	ORF1b:K798-	1	0.07	ORF1a:N1081-	3	0.05	ORF1a:E2993-	26	0.03
Maharashtra	ORF1a:C1752-	1	0.03	ORF1b:G799-	1	0.07	ORF1a:A1082-	3	0.05	ORF1a:S3001-	26	0.03
Maharashtra	ORF1a:K1753-	1	0.03	ORF1b:P800-	1	0.07	ORF1a:M1083-	3	0.05	ORF1a:G3002-	26	0.03
Maharashtra	ORF1a:T1754-	1	0.03	ORF1b:H801-	1	0.07	ORF1a:Q1084-	3	0.05	ORF1a:R3003-	26	0.03
Maharashtra	ORF1a:C1755-	1	0.03	ORF1b:E802-	1	0.07	ORF1a:V1085-	3	0.05	ORF1a:D3042-	26	0.03

Maharashtra	ORF1a:G1756-	1	0.03	ORF1b:F803-	1	0.07	ORF1a:E1086-	3	0.05	ORF1a:I3043-	26	0.03
Maharashtra	ORF1a:Q1757-	1	0.03	ORF1b:V811-	1	0.07	ORF1a:S1087-	3	0.05	ORF1a:R3066-	26	0.03
Maharashtra	ORF1a:Q1758-	1	0.03	ORF1b:K812-	1	0.07	ORF1a:D1088-	3	0.05	ORF1a:F3067-	26	0.03
Maharashtra	ORF1a:Q1759-	1	0.03	ORF1b:Q813-	1	0.07	ORF1a:D1089-	3	0.05	ORF1a:R3068-	26	0.03
Maharashtra	ORF1a:T1760-	1	0.03	ORF1b:G814-	1	0.07	ORF1a:V1099-	3	0.05	ORF1a:R3069-	26	0.03
Maharashtra	ORF1a:T1761-	1	0.03	ORF1b:D815-	1	0.07	ORF1a:G1100-	3	0.05	ORF1a:T3255-	26	0.03
Maharashtra	ORF1a:L1762-	1	0.03	ORF1b:D816-	1	0.07	ORF1a:G1101-	3	0.05	ORF1a:S3256-	26	0.03
Maharashtra	ORF1a:K1763-	1	0.03	ORF1b:Y817-	1	0.07	ORF1a:G1119-	3	0.05	ORF1a:I3257-	26	0.03
Maharashtra	ORF1a:G1764-	1	0.03	ORF1b:V818-	1	0.07	ORF1a:P1120-	3	0.05	ORF1a:T3258-	26	0.03
Maharashtra	ORF1a:V1765-	1	0.03	ORF1b:Y819-	1	0.07	ORF1a:N1121-	3	0.05	ORF1a:S3259-	26	0.03
Maharashtra	ORF1a:E1766-	1	0.03	ORF1b:G830-	1	0.07	ORF1a:V1122-	3	0.05	S:D287-	26	0.03
Maharashtra	ORF1a:S1775-	1	0.03	ORF1b:A831-	1	0.07	ORF1a:N1123-	3	0.05	S:A288-	26	0.03
Maharashtra	ORF1a:Y1776-	1	0.03	ORF1b:G832-	1	0.07	ORF1a:K1124-	3	0.05	S:R319-	26	0.03
Maharashtra	ORF1a:E1777-	1	0.03	ORF1b:C833-	1	0.07	ORF1a:G1125-	3	0.05	S:V320-	26	0.03
Maharashtra	ORF1a:Q1778-	1	0.03	ORF1b:F834-	1	0.07	ORF1a:E1126-	3	0.05	S:Q321-	26	0.03
Maharashtra	ORF1a:F1779-	1	0.03	ORF1b:V835-	1	0.07	ORF1a:S1133-	3	0.05	S:P322-	26	0.03
Maharashtra	ORF1a:K1780-	1	0.03	ORF1b:D836-	1	0.07	ORF1a:A1134-	3	0.05	S:I326-	26	0.03
Maharashtra	ORF1a:K1781-	1	0.03	ORF1b:D837-	1	0.07	ORF1a:Y1135-	3	0.05	S:V327-	26	0.03
Maharashtra	ORF1a:G1782-	1	0.03	ORF1b:I838-	1	0.07	ORF1a:V1169-	3	0.05	S:N501-	26	0.03
Maharashtra	ORF1a:V1783-	1	0.03	ORF1b:V839-	1	0.07	ORF1a:L2271-	3	0.05	S:G502-	26	0.03
Maharashtra	ORF1a:Q1784-	1	0.03	ORF1b:K840-	1	0.07	ORF1a:N2272-	3	0.05	S:V503-	26	0.03
Maharashtra	ORF1a:I1785-	1	0.03	ORF1b:T841-	1	0.07	ORF1a:S2273-	3	0.05	S:G504-	26	0.03
Maharashtra	ORF1a:P1786-	1	0.03	ORF1b:D842-	1	0.07	ORF1a:T2274-	3	0.05	S:Y505-	26	0.03
Maharashtra	ORF1a:C1787-	1	0.03	ORF1b:G843-	1	0.07	ORF1b:R1430-	3	0.05	S:Q506-	26	0.03
Maharashtra	ORF1a:F1804-	1	0.03	ORF1b:T844-	1	0.07	ORF1b:K1431-	3	0.05	S:P507-	26	0.03
Maharashtra	ORF1a:V1805-	1	0.03	ORF1b:L845-	1	0.07	ORF1b:A1432-	3	0.05	S:I569-	26	0.03
Maharashtra	ORF1a:M1806-	1	0.03	ORF1b:M846-	1	0.07	ORF1b:V1433-	3	0.05	S:T618-	26	0.03
Maharashtra	ORF1a:N1830-	1	0.03	ORF1b:I847-	1	0.07	ORF1b:F1434-	3	0.05	S:C749-	26	0.03
Maharashtra	ORF1a:Y1831-	1	0.03	ORF1b:E848-	1	0.07	ORF1b:I1435-	3	0.05	S:S750-	26	0.03
Maharashtra	ORF1a:Q1832-	1	0.03	ORF1b:R849-	1	0.07	ORF1b:S1436-	3	0.05	S:N751-	26	0.03
Maharashtra	ORF1a:C1833-	1	0.03	ORF1b:F850-	1	0.07	ORF1b:S1446-	3	0.05	S:G769-	26	0.03
Maharashtra	ORF1a:G1834-	1	0.03	ORF1b:V851-	1	0.07	ORF1b:K1447-	3	0.05	S:I770-	26	0.03
Maharashtra	ORF1a:H1835-	1	0.03	ORF1b:S852-	1	0.07	ORF1b:F1925-	3	0.05	S:A771-	26	0.03
Maharashtra	ORF1a:Y1836-	1	0.03	ORF1b:L853-	1	0.07	ORF1b:R1928-	3	0.05	S:V772-	26	0.03
Maharashtra	ORF1a:I1848-	1	0.03	ORF1b:A854-	1	0.07	ORF1b:V1929-	3	0.05	S:E773-	26	0.03
Maharashtra	ORF1a:D1849-	1	0.03	ORF1b:I855-	1	0.07	ORF1b:L1930-	3	0.05	S:E780-	26	0.03
Maharashtra	ORF1a:G1850-	1	0.03	ORF1b:D856-	1	0.07	ORF1b:S1931-	3	0.05	S:V781-	26	0.03
Maharashtra	ORF1a:A1851-	1	0.03	ORF1b:A857-	1	0.07	ORF1b:N1932-	3	0.05	S:F782-	26	0.03
Maharashtra	ORF1a:L1852-	1	0.03	ORF1b:Y858-	1	0.07	ORF1b:L1933-	3	0.05	S:A783-	26	0.03
Maharashtra	ORF1a:S1856-	1	0.03	ORF1b:P859-	1	0.07	ORF1b:N1934-	3	0.05	S:L806-	26	0.03
Maharashtra	ORF1a:E1909-	1	0.03	ORF1b:L860-	1	0.07	ORF1b:L1935-	3	0.05	S:E309-	26	0.03
Maharashtra	ORF1a:Q1910-	1	0.03	ORF1b:T861-	1	0.07	ORF1b:P1936-	3	0.05	S:E406-	26	0.03
Maharashtra	ORF1a:P1911-	1	0.03	ORF1b:K862-	1	0.07	ORF1b:G1937-	3	0.05	S:V407-	26	0.03
Maharashtra	ORF1a:I1912-	1	0.03	ORF1b:H863-	1	0.07	ORF1b:C1938-	3	0.05	S:G446-	26	0.03
Maharashtra	ORF1a:D1913-	1	0.03	ORF1b:P864-	1	0.07	ORF1b:D1939-	3	0.05	S:G447-	26	0.03
Maharashtra	ORF1a:L1914-	1	0.03	ORF1b:N865-	1	0.07	ORF1b:G1940-	3	0.05	S:N448-	26	0.03
Maharashtra	ORF1a:V1915-	1	0.03	ORF1b:Q866-	1	0.07	ORF1b:G1941-	3	0.05	S:Y449-	26	0.03
Maharashtra	ORF1a:P1916-	1	0.03	ORF1b:E867-	1	0.07	ORF1a:F1618-	2	0.03	S:N450-	26	0.03
Maharashtra	ORF1a:P1950-	1	0.03	ORF1b:Y868-	1	0.07	ORF1a:N4256-	2	0.03	S:Y451-	26	0.03
Maharashtra	ORF1a:A1951-	1	0.03	ORF1b:A869-	1	0.07	S:K147-	2	0.03	S:T470-	26	0.03
Maharashtra	ORF1a:S1952-	1	0.03	ORF1b:D870-	1	0.07	S:N148-	2	0.03	S:E471-	26	0.03
Maharashtra	ORF1a:R1953-	1	0.03	ORF1b:V871-	1	0.07	S:N149-	2	0.03	S:I472-	26	0.03
Maharashtra	ORF1a:E1954-	1	0.03	ORF1b:F872-	1	0.07	S:K150-	2	0.03	S:Y473-	26	0.03
Maharashtra	ORF1a:L1955-	1	0.03	ORF1b:H873-	1	0.07	S:G142-	2	0.03	S:K557-	26	0.03
Maharashtra	ORF1a:K1956-	1	0.03	ORF1b:L874-	1	0.07	S:V143-	2	0.03	S:K558-	26	0.03
Maharashtra	ORF1a:V1957-	1	0.03	ORF1b:Y875-	1	0.07	S:W152-	2	0.03	S:F559-	26	0.03
Maharashtra	ORF1a:R2011-	1	0.03	ORF1b:L876-	1	0.07	S:N165-	2	0.03	S:L560-	26	0.03
Maharashtra	ORF1a:C2012-	1	0.03	ORF1b:Q877-	1	0.07	S:C166-	2	0.03	S:L611-	26	0.03
Maharashtra	ORF1a:L2013-	1	0.03	ORF1b:Y878-	1	0.07	S:T167-	2	0.03	ORF1a:N2361-	25	0.03
Maharashtra	ORF1a:E2042-	1	0.03	ORF1b:I879-	1	0.07	S:F168-	2	0.03	ORF1a:S2362-	25	0.03
Maharashtra	ORF1a:D2043-	1	0.03	ORF1b:R880-	1	0.07	S:E169-	2	0.03	ORF1a:A2379-	25	0.03
Maharashtra	ORF1a:P2046-	1	0.03	ORF1b:K881-	1	0.07	S:Y170-	2	0.03	ORF1a:M2380-	25	0.03
Maharashtra	ORF1a:M2096-	1	0.03	ORF1b:S961-	1	0.07	S:V171-	2	0.03	ORF1a:V2381-	25	0.03
Maharashtra	ORF1a:L2105-	1	0.03	ORF1b:H962-	1	0.07	S:S172-	2	0.03	ORF1a:R2382-	25	0.03
Maharashtra	ORF1a:T2106-	1	0.03	ORF1b:K963-	1	0.07	S:Q173-	2	0.03	ORF1a:N2449-	25	0.03
Maharashtra	ORF1a:I2107-	1	0.03	ORF1b:L964-	1	0.07	S:P174-	2	0.03	ORF1a:W2450-	25	0.03
Maharashtra	ORF1a:K2108-	1	0.03	ORF1b:V965-	1	0.07	S:F175-	2	0.03	ORF1a:N2451-	25	0.03
Maharashtra	ORF1a:K2109-	1	0.03	ORF1b:L966-	1	0.07	S:L176-	2	0.03	ORF1a:C2452-	25	0.03
Maharashtra	ORF1a:P2110-	1	0.03	ORF1b:S967-	1	0.07	S:E191-	2	0.03	ORF1a:V2453-	25	0.03
Maharashtra	ORF1a:Q2178-	1	0.03	ORF1b:V968-	1	0.07	S:F192-	2	0.03	ORF1a:Q2476-	25	0.03

Maharashtra	ORF1a:L2179-	1	0.03	ORF1b:N969-	1	0.07	S:P217-	2	0.03	ORF1a:F2477-	25	0.03
Maharashtra	ORF1a:C2180-	1	0.03	ORF1b:P970-	1	0.07	S:Q218-	2	0.03	ORF1a:V2604-	25	0.03
Maharashtra	ORF1a:T2181-	1	0.03	ORF1b:Y971-	1	0.07	S:G219-	2	0.03	ORF1a:P2605-	25	0.03
Maharashtra	ORF1a:F2182-	1	0.03	ORF1b:V972-	1	0.07	S:F220-	2	0.03	ORF1a:M2606-	25	0.03
Maharashtra	ORF1a:T2183-	1	0.03	ORF1b:C973-	1	0.07	S:S221-	2	0.03	ORF1a:V2613-	25	0.03
Maharashtra	ORF1a:R2184-	1	0.03	ORF1b:N974-	1	0.07	S:A222-	2	0.03	ORF1a:A2614-	25	0.03
Maharashtra	ORF1a:S2185-	1	0.03	ORF1b:A975-	1	0.07	S:L223-	2	0.03	ORF1a:A2618-	25	0.03
Maharashtra	ORF1a:T2186-	1	0.03	ORF1b:P976-	1	0.07	S:E224-	2	0.03	ORF1a:E2619-	25	0.03
Maharashtra	ORF1a:N2187-	1	0.03	ORF1b:G977-	1	0.07	S:P225-	2	0.03	ORF1a:L2620-	25	0.03
Maharashtra	ORF1a:S2188-	1	0.03	ORF1b:C978-	1	0.07	S:P230-	2	0.03	ORF1a:Q2639-	25	0.03
Maharashtra	ORF1a:R2189-	1	0.03	ORF1b:D979-	1	0.07	S:I231-	2	0.03	ORF1a:G2640-	25	0.03
Maharashtra	ORF1a:I2190-	1	0.03	ORF1b:V980-	1	0.07	S:G232-	2	0.03	ORF1a:F2641-	25	0.03
Maharashtra	ORF1a:L2250-	1	0.03	ORF1b:T981-	1	0.07	S:I233-	2	0.03	ORF1a:V2642-	25	0.03
Maharashtra	ORF1a:G2251-	1	0.03	ORF1b:D982-	1	0.07	S:N234-	2	0.03	ORF1a:D2643-	25	0.03
Maharashtra	ORF1a:V2252-	1	0.03	ORF1b:V983-	1	0.07	S:I235-	2	0.03	ORF1a:S2644-	25	0.03
Maharashtra	ORF1a:L2253-	1	0.03	ORF1b:T984-	1	0.07	S:T236-	2	0.03	ORF1a:D2645-	25	0.03
Maharashtra	ORF1a:C2282-	1	0.03	ORF1b:Q985-	1	0.07	S:R237-	2	0.03	ORF1a:E2647-	25	0.03
Maharashtra	ORF1a:T2283-	1	0.03	ORF1b:L986-	1	0.07	S:F238-	2	0.03	ORF1a:E2664-	25	0.03
Maharashtra	ORF1a:G2284-	1	0.03	ORF1b:Y987-	1	0.07	S:Q239-	2	0.03	ORF1a:M2674-	25	0.03
Maharashtra	ORF1a:S2285-	1	0.03	ORF1b:L988-	1	0.07	S:T250-	2	0.03	ORF1a:V2865-	25	0.03
Maharashtra	ORF1a:I2286-	1	0.03	ORF1b:G989-	1	0.07	S:P251-	2	0.03	ORF1a:V2866-	25	0.03
Maharashtra	ORF1a:P2287-	1	0.03	ORF1b:G990-	1	0.07	S:G252-	2	0.03	ORF1a:P2867-	25	0.03
Maharashtra	ORF1a:C2288-	1	0.03	ORF1b:M991-	1	0.07	S:D253-	2	0.03	ORF1a:G2868-	25	0.03
Maharashtra	ORF1a:S2289-	1	0.03	ORF1b:S992-	1	0.07	S:S254-	2	0.03	ORF1a:P2870-	25	0.03
Maharashtra	ORF1a:V2290-	1	0.03	ORF1b:Y993-	1	0.07	S:S255-	2	0.03	ORF1a:P2950-	25	0.03
Maharashtra	ORF1a:C2291-	1	0.03	ORF1b:Y994-	1	0.07	S:S256-	2	0.03	ORF1a:D2951-	25	0.03
Maharashtra	ORF1a:A2320-	1	0.03	ORF1b:C995-	1	0.07	S:G257-	2	0.03	ORF1a:T2952-	25	0.03
Maharashtra	ORF1a:L2323-	1	0.03	ORF1b:K996-	1	0.07	S:W258-	2	0.03	ORF1a:R2953-	25	0.03
Maharashtra	ORF1a:L2329-	1	0.03	ORF1b:S997-	1	0.07	S:T259-	2	0.03	ORF1a:P3015-	25	0.03
Maharashtra	ORF1a:A2330-	1	0.03	ORF1b:H998-	1	0.07	S:A260-	2	0.03	ORF1a:G3016-	25	0.03
Maharashtra	ORF1a:Y2331-	1	0.03	ORF1b:K999-	1	0.07	S:G261-	2	0.03	ORF1a:V3017-	25	0.03
Maharashtra	ORF1a:I2332-	1	0.03	ORF1b:P1000-	1	0.07	S:A262-	2	0.03	ORF1a:F3018-	25	0.03
Maharashtra	ORF1a:L2333-	1	0.03	ORF1b:P1001-	1	0.07	S:A263-	2	0.03	ORF1a:C3019-	25	0.03
Maharashtra	ORF1a:V2372-	1	0.03	ORF1b:I1002-	1	0.07	S:A264-	2	0.03	ORF1a:G3020-	25	0.03
Maharashtra	ORF1a:I2377-	1	0.03	ORF1b:S1003-	1	0.07	S:Y265-	2	0.03	ORF1a:V3021-	25	0.03
Maharashtra	ORF1a:S2378-	1	0.03	ORF1b:F1004-	1	0.07	S:Q271-	2	0.03	ORF1a:F3064-	25	0.03
Maharashtra	ORF1a:M85-	1	0.03	ORF1b:P1005-	1	0.07	S:P272-	2	0.03	ORF1a:M3065-	25	0.03
Maharashtra	ORF8:G66-	1	0.03	ORF1b:L1006-	1	0.07	S:R273-	2	0.03	ORF1a:L3180-	25	0.03
Maharashtra	ORF8:S67-	1	0.03	ORF1b:C1007-	1	0.07	S:T274-	2	0.03	ORF1a:C3181-	25	0.03
Maharashtra	ORF1a:Q1198-	1	0.03	ORF1b:A1008-	1	0.07	S:F275-	2	0.03	ORF1a:T3182-	25	0.03
Maharashtra	ORF1a:V1199-	1	0.03	ORF1b:N1009-	1	0.07	S:L276-	2	0.03	ORF1a:F3183-	25	0.03
Maharashtra	ORF1a:E1200-	1	0.03	ORF1b:G1010-	1	0.07	S:L277-	2	0.03	ORF1a:A3260-	25	0.03
Maharashtra	ORF1a:Q1201-	1	0.03	ORF1b:Q1011-	1	0.07	S:K278-	2	0.03	ORF1a:V3261-	25	0.03
Maharashtra	ORF1a:K1202-	1	0.03	ORF1b:V1012-	1	0.07	S:Y279-	2	0.03	S:V289-	25	0.03
Maharashtra	ORF1a:I1203-	1	0.03	ORF1b:F1013-	1	0.07	S:N280-	2	0.03	S:L752-	25	0.03
Maharashtra	ORF1a:A1204-	1	0.03	ORF1b:G1014-	1	0.07	S:E281-	2	0.03	S:Q853-	25	0.03
Maharashtra	ORF1a:E1205-	1	0.03	ORF1b:L1015-	1	0.07	S:N282-	2	0.03	S:K854-	25	0.03
Maharashtra	ORF1a:I1206-	1	0.03	ORF1b:Y1016-	1	0.07	S:G283-	2	0.03	S:F855-	25	0.03
Maharashtra	ORF1a:P1207-	1	0.03	ORF1b:K1017-	1	0.07	S:T284-	2	0.03	S:N856-	25	0.03
Maharashtra	ORF1a:K1208-	1	0.03	ORF1b:N1018-	1	0.07	S:I285-	2	0.03	S:G857-	25	0.03
Maharashtra	ORF1a:E1209-	1	0.03	ORF1b:T1019-	1	0.07	S:T286-	2	0.03	S:L858-	25	0.03
Maharashtra	ORF1a:E1210-	1	0.03	ORF1b:C1020-	1	0.07	S:D287-	2	0.03	S:T941-	25	0.03
Maharashtra	ORF1a:V1211-	1	0.03	ORF1b:V1021-	1	0.07	S:A288-	2	0.03	S:H245-	25	0.03
Maharashtra	ORF1a:K1212-	1	0.03	ORF1b:G1022-	1	0.07	S:V289-	2	0.03	S:R246-	25	0.03
Maharashtra	ORF1a:P1213-	1	0.03	ORF1b:S1023-	1	0.07	S:D290-	2	0.03	S:S247-	25	0.03
Maharashtra	ORF1a:F1214-	1	0.03	ORF1b:D1024-	1	0.07	S:K304-	2	0.03	S:Y248-	25	0.03
Maharashtra	ORF1a:I1215-	1	0.03	ORF1b:N1025-	1	0.07	S:S305-	2	0.03	S:L249-	25	0.03
Maharashtra	ORF1a:T1216-	1	0.03	ORF1b:V1026-	1	0.07	S:F306-	2	0.03	S:T385-	25	0.03
Maharashtra	ORF1a:E1217-	1	0.03	ORF1b:T1027-	1	0.07	S:T307-	2	0.03	S:K386-	25	0.03
Maharashtra	ORF1a:S1218-	1	0.03	ORF1b:D1028-	1	0.07	S:P330-	2	0.03	S:L387-	25	0.03
Maharashtra	ORF1a:K1219-	1	0.03	ORF1b:F1029-	1	0.07	S:P337-	2	0.03	S:N388-	25	0.03
Maharashtra	ORF1a:P1220-	1	0.03	ORF1b:N1030-	1	0.07	S:F338-	2	0.03	S:G416-	25	0.03
Maharashtra	ORF1a:S1221-	1	0.03	ORF1b:A1031-	1	0.07	S:G339-	2	0.03	S:K417-	25	0.03
Maharashtra	ORF1a:V1222-	1	0.03	ORF1b:I1032-	1	0.07	S:E340-	2	0.03	S:I418-	25	0.03
Maharashtra	ORF1a:E1223-	1	0.03	ORF1b:A1033-	1	0.07	S:V341-	2	0.03	S:A419-	25	0.03
Maharashtra	ORF1a:Q1224-	1	0.03	ORF1b:T1034-	1	0.07	S:F342-	2	0.03	S:D420-	25	0.03
Maharashtra	ORF1a:I1232-	1	0.03	ORF1b:C1035-	1	0.07	S:N343-	2	0.03	S:Y421-	25	0.03
Maharashtra	ORF1a:K1233-	1	0.03	ORF1b:D1036-	1	0.07	S:V350-	2	0.03	S:F515-	25	0.03
Maharashtra	ORF1a:A1234-	1	0.03	ORF1b:W1037-	1	0.07	S:Y351-	2	0.03	S:E516-	25	0.03
Maharashtra	ORF1a:C1235-	1	0.03	ORF1b:T1038-	1	0.07	S:A352-	2	0.03	S:L517-	25	0.03

Maharashtra	ORF1a:V1236-	1	0.03	ORF1b:N1039-	1	0.07	S:W353-	2	0.03	S:S730-	25	0.03
Maharashtra	ORF1a:E1237-	1	0.03	ORF1b:A1040-	1	0.07	S:N354-	2	0.03	S:M731-	25	0.03
Maharashtra	ORF1a:E1238-	1	0.03	ORF1b:G1041-	1	0.07	S:R355-	2	0.03	S:T732-	25	0.03
Maharashtra	ORF1a:V1239-	1	0.03	ORF1b:D1042-	1	0.07	S:K356-	2	0.03	ORF1a:G2403-	24	0.03
Maharashtra	ORF1a:T1240-	1	0.03	ORF1b:Y1043-	1	0.07	S:R357-	2	0.03	ORF1a:C2404-	24	0.03
Maharashtra	ORF1a:T1241-	1	0.03	ORF1b:I1044-	1	0.07	S:I358-	2	0.03	ORF1a:N2405-	24	0.03
Maharashtra	ORF1a:T1242-	1	0.03	ORF1b:L1045-	1	0.07	S:S359-	2	0.03	ORF1a:S2406-	24	0.03
Maharashtra	ORF1a:L1243-	1	0.03	ORF1b:A1046-	1	0.07	S:N360-	2	0.03	ORF1a:S2407-	24	0.03
Maharashtra	ORF1a:E1244-	1	0.03	ORF1b:N1047-	1	0.07	S:C361-	2	0.03	ORF1a:T2408-	24	0.03
Maharashtra	ORF1a:E1245-	1	0.03	ORF1b:T1048-	1	0.07	S:V362-	2	0.03	ORF1a:I2481-	24	0.03
Maharashtra	ORF1a:T1246-	1	0.03	ORF1b:C1049-	1	0.07	S:A363-	2	0.03	ORF1a:F2521-	24	0.03
Maharashtra	ORF1a:K1247-	1	0.03	ORF1b:T1050-	1	0.07	S:D364-	2	0.03	ORF1a:V2522-	24	0.03
Maharashtra	ORF1a:F1248-	1	0.03	ORF1b:E1051-	1	0.07	S:Y380-	2	0.03	ORF1a:N2523-	24	0.03
Maharashtra	ORF1a:L1249-	1	0.03	ORF1b:R1052-	1	0.07	S:G381-	2	0.03	ORF1a:F2543-	24	0.03
Maharashtra	ORF1a:T1250-	1	0.03	ORF1b:L1053-	1	0.07	S:V382-	2	0.03	ORF1a:D2544-	24	0.03
Maharashtra	ORF1a:E1251-	1	0.03	ORF1b:K1054-	1	0.07	S:S383-	2	0.03	ORF1a:G2545-	24	0.03
Maharashtra	ORF1a:N1252-	1	0.03	ORF1b:N1587-	1	0.07	S:P384-	2	0.03	ORF1a:K2757-	24	0.03
Maharashtra	ORF1a:D1258-	1	0.03	ORF1b:Y1588-	1	0.07	S:D389-	2	0.03	ORF1a:I2758-	24	0.03
Maharashtra	ORF1a:I1259-	1	0.03	ORF1b:Q1589-	1	0.07	S:F392-	2	0.03	ORF1a:A2759-	24	0.03
Maharashtra	ORF1a:N1260-	1	0.03	ORF1b:V1590-	1	0.07	S:T393-	2	0.03	ORF1a:L2760-	24	0.03
Maharashtra	ORF1a:G1261-	1	0.03	ORF1b:N1591-	1	0.07	S:N394-	2	0.03	ORF1a:K2761-	24	0.03
Maharashtra	ORF1a:N1262-	1	0.03	ORF1b:G1592-	1	0.07	S:V395-	2	0.03	ORF1a:V2783-	24	0.03
Maharashtra	ORF1a:L1263-	1	0.03	ORF1b:Y1593-	1	0.07	S:F400-	2	0.03	ORF1a:A2784-	24	0.03
Maharashtra	ORF1a:H1264-	1	0.03	ORF1b:P1594-	1	0.07	S:V401-	2	0.03	ORF1a:A2785-	24	0.03
Maharashtra	ORF1a:P1265-	1	0.03	ORF1b:N1595-	1	0.07	S:I402-	2	0.03	ORF1a:I2786-	24	0.03
Maharashtra	ORF1a:D1266-	1	0.03	ORF1b:M1596-	1	0.07	S:R403-	2	0.03	ORF1a:F2787-	24	0.03
Maharashtra	ORF1a:S1267-	1	0.03	ORF1b:F1597-	1	0.07	S:G404-	2	0.03	ORF1a:Y2788-	24	0.03
Maharashtra	ORF1a:A1268-	1	0.03	ORF1b:I1598-	1	0.07	S:P412-	2	0.03	ORF1a:K2810-	24	0.03
Maharashtra	ORF1a:T1269-	1	0.03	ORF1b:T1599-	1	0.07	S:G413-	2	0.03	ORF1a:A2811-	24	0.03
Maharashtra	ORF1a:L1270-	1	0.03	ORF1b:R1600-	1	0.07	S:Q414-	2	0.03	ORF1a:I2812-	24	0.03
Maharashtra	ORF1a:V1271-	1	0.03	ORF1b:E1601-	1	0.07	S:T415-	2	0.03	ORF1a:D2813-	24	0.03
Maharashtra	ORF1a:S1272-	1	0.03	ORF1b:E1602-	1	0.07	S:P426-	2	0.03	ORF1a:G2814-	24	0.03
Maharashtra	ORF1a:K1281-	1	0.03	ORF1b:A1603-	1	0.07	S:N437-	2	0.03	ORF1a:G2815-	24	0.03
Maharashtra	ORF1a:D1282-	1	0.03	ORF1b:I1604-	1	0.07	S:S438-	2	0.03	ORF1a:V2816-	24	0.03
Maharashtra	ORF1a:A1283-	1	0.03	ORF1b:R1605-	1	0.07	S:N439-	2	0.03	ORF1a:L2869-	24	0.03
Maharashtra	ORF1a:P1284-	1	0.03	ORF1b:H1606-	1	0.07	S:N440-	2	0.03	ORF1a:G2871-	24	0.03
Maharashtra	ORF1a:Y1285-	1	0.03	ORF1b:V1607-	1	0.07	S:L441-	2	0.03	ORF1a:T2872-	24	0.03
Maharashtra	ORF1a:I1286-	1	0.03	ORF1b:R1608-	1	0.07	S:D442-	2	0.03	ORF1a:I2903-	24	0.03
Maharashtra	ORF1a:V1287-	1	0.03	ORF1b:A1609-	1	0.07	S:S443-	2	0.03	ORF1a:A2909-	24	0.03
Maharashtra	ORF1a:G1288-	1	0.03	ORF1b:W1610-	1	0.07	S:K462-	2	0.03	ORF1a:G2927-	24	0.03
Maharashtra	ORF1a:D1289-	1	0.03	ORF1b:I1611-	1	0.07	S:P463-	2	0.03	ORF1a:K2928-	24	0.03
Maharashtra	ORF1a:V1290-	1	0.03	ORF1b:G1612-	1	0.07	S:F464-	2	0.03	ORF1a:P2929-	24	0.03
Maharashtra	ORF1a:V1291-	1	0.03	ORF1b:F1613-	1	0.07	S:E465-	2	0.03	ORF1a:Y2934-	24	0.03
Maharashtra	ORF1a:Q1292-	1	0.03	ORF1b:D1614-	1	0.07	S:R466-	2	0.03	ORF1a:D2935-	24	0.03
Maharashtra	ORF1a:G1294-	1	0.03	ORF1b:V1615-	1	0.07	S:D467-	2	0.03	ORF1a:D2958-	24	0.03
Maharashtra	ORF1a:V1295-	1	0.03	ORF1b:E1616-	1	0.07	S:I468-	2	0.03	ORF1a:G2959-	24	0.03
Maharashtra	ORF1a:L1296-	1	0.03	ORF1b:G1617-	1	0.07	S:T478-	2	0.03	ORF1a:S2960-	24	0.03
Maharashtra	ORF1a:T1297-	1	0.03	ORF1b:C1618-	1	0.07	S:P479-	2	0.03	ORF1a:I2961-	24	0.03
Maharashtra	ORF1a:A1298-	1	0.03	ORF1b:H1619-	1	0.07	S:N501-	2	0.03	ORF1a:I2962-	24	0.03
Maharashtra	ORF1a:V1299-	1	0.03	ORF1b:A1620-	1	0.07	S:G502-	2	0.03	ORF1a:Q2963-	24	0.03
Maharashtra	ORF1a:V1300-	1	0.03	ORF1b:T1621-	1	0.07	S:V503-	2	0.03	ORF1a:F2964-	24	0.03
Maharashtra	ORF1a:I1301-	1	0.03	ORF1b:R1622-	1	0.07	S:G504-	2	0.03	ORF1a:S2981-	24	0.03
Maharashtra	ORF1a:P1302-	1	0.03	ORF1b:E1623-	1	0.07	S:Y505-	2	0.03	ORF1a:K3151-	24	0.03
Maharashtra	ORF1a:T1303-	1	0.03	ORF1b:A1624-	1	0.07	S:Q506-	2	0.03	ORF1a:H3152-	24	0.03
Maharashtra	ORF1a:K1304-	1	0.03	ORF1b:V1625-	1	0.07	S:P507-	2	0.03	ORF1a:T3202-	24	0.03
Maharashtra	ORF1a:K1305-	1	0.03	ORF1b:G1626-	1	0.07	S:Y508-	2	0.03	ORF1a:Q3203-	24	0.03
Maharashtra	ORF1a:A1306-	1	0.03	ORF1b:T1627-	1	0.07	S:R509-	2	0.03	ORF1a:Y3204-	24	0.03
Maharashtra	ORF1a:G1307-	1	0.03	ORF1b:N1628-	1	0.07	S:V510-	2	0.03	ORF1a:N3205-	24	0.03
Maharashtra	ORF1a:R1318-	1	0.03	ORF1b:L1629-	1	0.07	S:H519-	2	0.03	ORF1a:R3206-	24	0.03
Maharashtra	ORF1a:K1319-	1	0.03	ORF1b:P1630-	1	0.07	S:A520-	2	0.03	ORF1a:Y3207-	24	0.03
Maharashtra	ORF1a:V1320-	1	0.03	ORF1b:L1631-	1	0.07	S:P521-	2	0.03	ORF1a:L3208-	24	0.03
Maharashtra	ORF1a:P1321-	1	0.03	ORF1b:Q1632-	1	0.07	S:A522-	2	0.03	ORF1a:A3209-	24	0.03
Maharashtra	ORF1a:T1322-	1	0.03	ORF1b:L1633-	1	0.07	S:T523-	2	0.03	ORF1a:P3302-	24	0.03
Maharashtra	ORF1a:D1323-	1	0.03	ORF1b:G1634-	1	0.07	S:V524-	2	0.03	ORF1a:R3303-	24	0.03
Maharashtra	ORF1a:N1324-	1	0.03	ORF1b:F1635-	1	0.07	S:C525-	2	0.03	ORF1a:G3334-	24	0.03
Maharashtra	ORF1a:Y1325-	1	0.03	ORF1b:D1650-	1	0.07	S:G526-	2	0.03	ORF1a:V3340-	24	0.03
Maharashtra	ORF1a:I1326-	1	0.03	ORF1b:T1651-	1	0.07	S:P527-	2	0.03	ORF1a:G3383-	24	0.03
Maharashtra	ORF1a:T1327-	1	0.03	ORF1b:P1652-	1	0.07	S:K528-	2	0.03	ORF1a:C3391-	24	0.03
Maharashtra	ORF1a:T1328-	1	0.03	ORF1b:N1653-	1	0.07	S:G545-	2	0.03	ORF1a:A3392-	24	0.03
Maharashtra	ORF1a:Y1329-	1	0.03	ORF1b:N1654-	1	0.07	S:G548-	2	0.03	ORF1a:M3393-	24	0.03

Maharashtra	ORF1a:L1334-	1	0.03	ORF1b:T1655-	1	0.07	S:T549-	2	0.03	ORF1a:R3394-	24	0.03
Maharashtra	ORF1a:N1335-	1	0.03	ORF1b:D1656-	1	0.07	S:G550-	2	0.03	ORF1a:P3395-	24	0.03
Maharashtra	ORF1a:G1336-	1	0.03	ORF1b:F1657-	1	0.07	S:V551-	2	0.03	ORF1a:K3400-	24	0.03
Maharashtra	ORF1a:Y1337-	1	0.03	ORF1b:S1658-	1	0.07	S:L552-	2	0.03	ORF1a:N3414-	24	0.03
Maharashtra	ORF1a:T1338-	1	0.03	ORF1b:R1659-	1	0.07	S:T553-	2	0.03	ORF1a:I3415-	24	0.03
Maharashtra	ORF1a:V1339-	1	0.03	ORF1b:V1660-	1	0.07	S:E554-	2	0.03	ORF1a:D3416-	24	0.03
Maharashtra	ORF1a:E1340-	1	0.03	ORF1b:S1661-	1	0.07	S:N556-	2	0.03	ORF1a:Y3417-	24	0.03
Maharashtra	ORF1a:E1341-	1	0.03	ORF1b:A1662-	1	0.07	S:P561-	2	0.03	ORF1a:D3418-	24	0.03
Maharashtra	ORF1a:A1342-	1	0.03	ORF1b:K1663-	1	0.07	S:F562-	2	0.03	ORF1a:C3419-	24	0.03
Maharashtra	ORF1a:K1343-	1	0.03	ORF1b:P1664-	1	0.07	S:Q563-	2	0.03	ORF1a:V3420-	24	0.03
Maharashtra	ORF1a:T1344-	1	0.03	ORF1b:P1665-	1	0.07	S:Q564-	2	0.03	ORF1a:S3421-	24	0.03
Maharashtra	ORF1a:V1345-	1	0.03	ORF1b:P1666-	1	0.07	S:F565-	2	0.03	ORF1a:M3425-	24	0.03
Maharashtra	ORF1a:L1346-	1	0.03	ORF1b:G1667-	1	0.07	S:G566-	2	0.03	ORF1a:H3426-	24	0.03
Maharashtra	ORF1a:K1347-	1	0.03	ORF1b:D1668-	1	0.07	S:R567-	2	0.03	ORF1a:H3427-	24	0.03
Maharashtra	ORF1a:K1348-	1	0.03	ORF1b:Y1678-	1	0.07	S:D568-	2	0.03	ORF1a:M3428-	24	0.03
Maharashtra	ORF1a:E1363-	1	0.03	ORF1b:K1679-	1	0.07	S:I569-	2	0.03	ORF1a:E3429-	24	0.03
Maharashtra	ORF1a:K1364-	1	0.03	ORF1b:G1680-	1	0.07	S:A575-	2	0.03	ORF1a:L3430-	24	0.03
Maharashtra	ORF1a:Q1365-	1	0.03	ORF1b:L1681-	1	0.07	S:V576-	2	0.03	ORF1a:N4256-	24	0.03
Maharashtra	ORF1a:E1366-	1	0.03	ORF1b:P1682-	1	0.07	S:R577-	2	0.03	ORF1a:T1567-	24	0.03
Maharashtra	ORF1a:I1367-	1	0.03	ORF1b:W1683-	1	0.07	S:D578-	2	0.03	ORF1a:I1568-	24	0.03
Maharashtra	ORF1a:L1368-	1	0.03	ORF1b:N1684-	1	0.07	S:P579-	2	0.03	ORF1a:K1569-	24	0.03
Maharashtra	ORF1a:G1369-	1	0.03	ORF1b:V1685-	1	0.07	S:D586-	2	0.03	ORF1a:M1806-	24	0.03
Maharashtra	ORF1a:T1370-	1	0.03	ORF1b:V1686-	1	0.07	S:I587-	2	0.03	ORF1a:M1807-	24	0.03
Maharashtra	ORF1a:V1371-	1	0.03	ORF1b:R1687-	1	0.07	S:T588-	2	0.03	ORF1a:S1808-	24	0.03
Maharashtra	ORF1a:L1375-	1	0.03	ORF1b:I1688-	1	0.07	S:P589-	2	0.03	S:R646-	24	0.03
Maharashtra	ORF1a:R1376-	1	0.03	ORF1b:K1689-	1	0.07	S:C590-	2	0.03	S:Q677-	24	0.03
Maharashtra	ORF1a:E1377-	1	0.03	ORF1b:I1690-	1	0.07	S:S591-	2	0.03	S:T678-	24	0.03
Maharashtra	ORF1a:M1378-	1	0.03	ORF1b:V1691-	1	0.07	S:F592-	2	0.03	S:N679-	24	0.03
Maharashtra	ORF1a:L1379-	1	0.03	ORF1b:Q1692-	1	0.07	S:G593-	2	0.03	S:D294-	24	0.03
Maharashtra	ORF1a:A1380-	1	0.03	ORF1b:M1693-	1	0.07	S:G594-	2	0.03	S:P295-	24	0.03
Maharashtra	ORF1a:H1381-	1	0.03	ORF1b:L1694-	1	0.07	S:Y612-	2	0.03	S:L452-	24	0.03
Maharashtra	ORF1a:A1382-	1	0.03	ORF1b:S1695-	1	0.07	S:Q613-	2	0.03	S:L699-	24	0.03
Maharashtra	ORF1a:E1383-	1	0.03	ORF1b:D1696-	1	0.07	S:D614-	2	0.03	S:I712-	24	0.03
Maharashtra	ORF1a:E1384-	1	0.03	ORF1b:T1697-	1	0.07	S:V615-	2	0.03	S:A713-	24	0.03
Maharashtra	ORF1a:T1385-	1	0.03	ORF1b:L1698-	1	0.07	S:N616-	2	0.03	S:I714-	24	0.03
Maharashtra	ORF1a:M1389-	1	0.03	ORF1b:K1699-	1	0.07	S:T618-	2	0.03	S:P715-	24	0.03
Maharashtra	ORF1a:P1390-	1	0.03	ORF1b:N1700-	1	0.07	S:E619-	2	0.03	S:F718-	24	0.03
Maharashtra	ORF1a:V1391-	1	0.03	ORF1b:L1701-	1	0.07	S:V620-	2	0.03	S:T719-	24	0.03
Maharashtra	ORF1a:C1392-	1	0.03	ORF1b:S1702-	1	0.07	S:P621-	2	0.03	S:I720-	24	0.03
Maharashtra	ORF1a:V1393-	1	0.03	ORF1b:D1703-	1	0.07	S:V622-	2	0.03	S:V722-	24	0.03
Maharashtra	ORF1a:E1394-	1	0.03	ORF1b:R1704-	1	0.07	S:A623-	2	0.03	S:T723-	24	0.03
Maharashtra	ORF1a:T1395-	1	0.03	ORF1b:V1705-	1	0.07	S:I624-	2	0.03	S:T724-	24	0.03
Maharashtra	ORF1a:K1396-	1	0.03	ORF1b:V1706-	1	0.07	S:H625-	2	0.03	S:E725-	24	0.03
Maharashtra	ORF1a:A1397-	1	0.03	ORF1b:F1707-	1	0.07	S:T630-	2	0.03	S:I726-	24	0.03
Maharashtra	ORF1a:I1398-	1	0.03	ORF1b:V1708-	1	0.07	S:P631-	2	0.03	S:L727-	24	0.03
Maharashtra	ORF1a:V1399-	1	0.03	ORF1b:L1709-	1	0.07	S:T632-	2	0.03	S:P728-	24	0.03
Maharashtra	ORF1a:S1400-	1	0.03	ORF1b:W1710-	1	0.07	S:W633-	2	0.03	S:V729-	24	0.03
Maharashtra	ORF1a:T1401-	1	0.03	ORF1b:A1711-	1	0.07	S:R634-	2	0.03	S:L753-	24	0.03
Maharashtra	ORF1a:I1402-	1	0.03	ORF1b:H1712-	1	0.07	S:V635-	2	0.03	S:L754-	24	0.03
Maharashtra	ORF1a:Q1403-	1	0.03	ORF1b:K1724-	1	0.07	S:Y636-	2	0.03	S:Q755-	24	0.03
Maharashtra	ORF1a:R1404-	1	0.03	ORF1b:I1725-	1	0.07	S:R646-	2	0.03	S:N164-	24	0.03
Maharashtra	ORF1a:K1405-	1	0.03	ORF1b:G1726-	1	0.07	S:A647-	2	0.03	ORF1a:N1995-	24	0.03
Maharashtra	ORF1a:Y1406-	1	0.03	ORF1b:P1727-	1	0.07	S:C671-	2	0.03	ORF1a:N1996-	24	0.03
Maharashtra	ORF1a:K1407-	1	0.03	ORF1b:E1728-	1	0.07	S:A672-	2	0.03	ORF1a:A1997-	24	0.03
Maharashtra	ORF1a:G1408-	1	0.03	ORF1b:R1729-	1	0.07	S:S673-	2	0.03	ORF1a:T1998-	24	0.03
Maharashtra	ORF1a:I1409-	1	0.03	ORF1b:T1730-	1	0.07	S:Y674-	2	0.03	ORF1a:N1999-	24	0.03
Maharashtra	ORF1a:K1410-	1	0.03	ORF1b:C1731-	1	0.07	S:Q675-	2	0.03	ORF1a:K2000-	24	0.03
Maharashtra	ORF1a:I1411-	1	0.03	ORF1b:C1732-	1	0.07	S:T676-	2	0.03	ORF1a:A2001-	24	0.03
Maharashtra	ORF1a:Q1412-	1	0.03	ORF1b:L1733-	1	0.07	S:Q677-	2	0.03	ORF1a:T2002-	24	0.03
Maharashtra	ORF1a:E1413-	1	0.03	ORF1b:C1734-	1	0.07	S:T678-	2	0.03	ORF1a:Y2003-	24	0.03
Maharashtra	ORF1a:G1414-	1	0.03	ORF1b:D1735-	1	0.07	S:N679-	2	0.03	ORF1a:K2546-	23	0.03
Maharashtra	ORF1a:V1415-	1	0.03	ORF1b:R1736-	1	0.07	S:S680-	2	0.03	ORF1a:A2621-	23	0.03
Maharashtra	ORF1a:V1416-	1	0.03	ORF1b:R1737-	1	0.07	S:P681-	2	0.03	ORF1a:V2646-	23	0.03
Maharashtra	ORF1a:L1445-	1	0.03	ORF1b:A1738-	1	0.07	ORF1b:I1355-	2	0.03	ORF1a:I2858-	23	0.03
Maharashtra	ORF1a:V1446-	1	0.03	ORF1b:T1739-	1	0.07	ORF1b:G1356-	2	0.03	ORF1a:T2859-	23	0.03
Maharashtra	ORF1a:T1447-	1	0.03	ORF1b:C1740-	1	0.07	ORF1b:P1357-	2	0.03	ORF1a:R2860-	23	0.03
Maharashtra	ORF1a:M1448-	1	0.03	ORF1b:F1741-	1	0.07	ORF1b:D1358-	2	0.03	ORF1a:I2873-	23	0.03
Maharashtra	ORF1a:P1449-	1	0.03	ORF1b:S1742-	1	0.07	ORF1b:M1359-	2	0.03	ORF1a:F2979-	23	0.03
Maharashtra	ORF1a:L1450-	1	0.03	ORF1b:T1743-	1	0.07	ORF1b:F1360-	2	0.03	ORF1a:D2980-	23	0.03
Maharashtra	ORF1a:G1451-	1	0.03	ORF1b:A1744-	1	0.07	ORF1b:L1361-	2	0.03	ORF1a:S2999-	23	0.03

Maharashtra	ORF1a:Y1452-	1	0.03	ORF1b:S1745-	1	0.07	ORF1b:G1362-	2	0.03	ORF1a:T3000-	23	0.03
Maharashtra	ORF1a:V1453-	1	0.03	ORF1b:D1746-	1	0.07	ORF1b:T1363-	2	0.03	ORF1a:V3136-	23	0.03
Maharashtra	ORF1a:T1454-	1	0.03	ORF1b:T1747-	1	0.07	ORF1b:C1364-	2	0.03	ORF1a:P3137-	23	0.03
Maharashtra	ORF1a:H1455-	1	0.03	ORF1b:Y1748-	1	0.07	ORF1b:R1365-	2	0.03	ORF1a:F3138-	23	0.03
Maharashtra	ORF1a:G1456-	1	0.03	ORF1b:A1749-	1	0.07	ORF1b:L1451-	2	0.03	ORF1a:W3139-	23	0.03
Maharashtra	ORF1a:L1457-	1	0.03	ORF1b:C1750-	1	0.07	ORF1b:V1467-	2	0.03	ORF1a:I3140-	23	0.03
Maharashtra	ORF1a:N1458-	1	0.03	ORF1b:W1751-	1	0.07	ORF1b:I1468-	2	0.03	ORF1a:D3297-	23	0.03
Maharashtra	ORF1a:L1459-	1	0.03	ORF1b:H1752-	1	0.07	ORF1b:F1469-	2	0.03	ORF1a:Q3332-	23	0.03
Maharashtra	ORF1a:E1460-	1	0.03	ORF1b:H1753-	1	0.07	ORF1b:T1470-	2	0.03	ORF1a:A3333-	23	0.03
Maharashtra	ORF1a:E1461-	1	0.03	ORF1b:S1754-	1	0.07	ORF1b:Q1471-	2	0.03	ORF1a:N3335-	23	0.03
Maharashtra	ORF1a:A1462-	1	0.03	ORF1b:I1755-	1	0.07	ORF1b:T1472-	2	0.03	ORF1a:V3336-	23	0.03
Maharashtra	ORF1a:A1463-	1	0.03	ORF1b:G1756-	1	0.07	ORF1b:T1473-	2	0.03	ORF1a:Q3337-	23	0.03
Maharashtra	ORF1a:R1464-	1	0.03	ORF1b:F1757-	1	0.07	ORF1b:I1498-	2	0.03	ORF1a:L3338-	23	0.03
Maharashtra	ORF1a:Y1465-	1	0.03	ORF1b:D1758-	1	0.07	ORF1b:M1499-	2	0.03	ORF1a:R3339-	23	0.03
Maharashtra	ORF1a:M1466-	1	0.03	ORF1b:Y1759-	1	0.07	ORF1b:T1511-	2	0.03	ORF1a:N3382-	23	0.03
Maharashtra	ORF1a:R1467-	1	0.03	ORF1b:V1760-	1	0.07	ORF1b:S1512-	2	0.03	ORF1a:G3401-	23	0.03
Maharashtra	ORF1a:S1478-	1	0.03	ORF1b:Y1761-	1	0.07	ORF1b:K1537-	2	0.03	ORF1a:P3431-	23	0.03
Maharashtra	ORF1a:S1479-	1	0.03	ORF1b:N1762-	1	0.07	ORF1b:V1538-	2	0.03	ORF1a:K1668-	23	0.03
Maharashtra	ORF1a:P1480-	1	0.03	ORF1b:P1763-	1	0.07	ORF1b:I1539-	2	0.03	ORF1a:W1669-	23	0.03
Maharashtra	ORF1a:D1481-	1	0.03	ORF1b:F1764-	1	0.07	ORF1b:T1540-	2	0.03	ORF1a:A1670-	23	0.03
Maharashtra	ORF1a:A1482-	1	0.03	ORF1b:M1765-	1	0.07	ORF1b:G1541-	2	0.03	ORF1a:D1671-	23	0.03
Maharashtra	ORF1a:V1483-	1	0.03	ORF1b:I1766-	1	0.07	ORF1b:L1542-	2	0.03	ORF1a:N1672-	23	0.03
Maharashtra	ORF1a:T1484-	1	0.03	ORF1b:D1767-	1	0.07	ORF1b:H1543-	2	0.03	ORF1a:N1673-	23	0.03
Maharashtra	ORF1a:A1485-	1	0.03	ORF1b:V1768-	1	0.07	ORF1b:T1549-	2	0.03	ORF1a:C1674-	23	0.03
Maharashtra	ORF1a:Y1486-	1	0.03	ORF1b:Q1769-	1	0.07	ORF1b:H1550-	2	0.03	ORF1a:D1697-	23	0.03
Maharashtra	ORF1a:N1487-	1	0.03	ORF1b:Q1770-	1	0.07	ORF1b:L1551-	2	0.03	ORF1a:A1698-	23	0.03
Maharashtra	ORF1a:G1488-	1	0.03	ORF1b:W1771-	1	0.07	ORF1b:S1552-	2	0.03	ORF1a:E1777-	23	0.03
Maharashtra	ORF1a:Y1489-	1	0.03	ORF1b:G1772-	1	0.07	ORF1b:V1553-	2	0.03	ORF1a:Q1778-	23	0.03
Maharashtra	ORF1a:L1490-	1	0.03	ORF1b:L1783-	1	0.07	ORF1b:M1581-	2	0.03	ORF1a:F1779-	23	0.03
Maharashtra	ORF1a:T1491-	1	0.03	ORF1b:Y1784-	1	0.07	ORF1b:M1582-	2	0.03	ORF1a:K1780-	23	0.03
Maharashtra	ORF1a:S1492-	1	0.03	ORF1b:C1785-	1	0.07	ORF1b:R1622-	2	0.03	ORF1a:A1809-	23	0.03
Maharashtra	ORF1a:S1493-	1	0.03	ORF1b:Q1786-	1	0.07	ORF1b:E1623-	2	0.03	ORF1a:P1810-	23	0.03
Maharashtra	ORF1a:S1494-	1	0.03	ORF1b:V1787-	1	0.07	ORF1b:A1624-	2	0.03	ORF1a:P1811-	23	0.03
Maharashtra	ORF1a:K1495-	1	0.03	ORF1b:H1788-	1	0.07	ORF1b:V1625-	2	0.03	ORF1a:A1812-	23	0.03
Maharashtra	ORF1a:T1496-	1	0.03	ORF1b:G1789-	1	0.07	ORF1b:N1628-	2	0.03	ORF1a:Q1813-	23	0.03
Maharashtra	ORF1a:P1497-	1	0.03	ORF1b:N1790-	1	0.07	ORF1b:L1629-	2	0.03	ORF1a:Y1814-	23	0.03
Maharashtra	ORF1a:E1498-	1	0.03	ORF1b:A1791-	1	0.07	ORF1b:P1630-	2	0.03	ORF1a:E1815-	23	0.03
Maharashtra	ORF1a:E1499-	1	0.03	ORF1b:D1797-	1	0.07	ORF1b:L1631-	2	0.03	ORF1a:L1816-	23	0.03
Maharashtra	ORF1a:H1500-	1	0.03	ORF1b:A1798-	1	0.07	ORF1b:Q1632-	2	0.03	ORF1a:K1817-	23	0.03
Maharashtra	ORF1a:F1501-	1	0.03	ORF1b:I1799-	1	0.07	ORF1b:V1649-	2	0.03	S:P230-	23	0.03
Maharashtra	ORF1a:I1502-	1	0.03	ORF1b:M1800-	1	0.07	ORF1b:D1650-	2	0.03	S:I231-	23	0.03
Maharashtra	ORF1a:E1503-	1	0.03	ORF1b:T1801-	1	0.07	ORF1b:T1651-	2	0.03	S:G232-	23	0.03
Maharashtra	ORF1a:T1504-	1	0.03	ORF1b:R1802-	1	0.07	ORF1b:P1652-	2	0.03	S:D290-	23	0.03
Maharashtra	ORF1a:I1505-	1	0.03	ORF1b:C1803-	1	0.07	ORF1b:N1653-	2	0.03	S:Y636-	23	0.03
Maharashtra	ORF1a:A1508-	1	0.03	ORF1b:L1804-	1	0.07	ORF1b:N1654-	2	0.03	S:A647-	23	0.03
Maharashtra	ORF1a:G1509-	1	0.03	ORF1b:A1805-	1	0.07	ORF1b:Q1692-	2	0.03	S:T676-	23	0.03
Maharashtra	ORF1a:S1510-	1	0.03	ORF1b:V1806-	1	0.07	ORF1b:M1693-	2	0.03	S:K814-	23	0.03
Maharashtra	ORF1a:Y1511-	1	0.03	ORF1b:H1807-	1	0.07	ORF1b:L1694-	2	0.03	S:R815-	23	0.03
Maharashtra	ORF1a:K1512-	1	0.03	ORF1b:E1808-	1	0.07	ORF1b:S1695-	2	0.03	S:S816-	23	0.03
Maharashtra	ORF1a:D1513-	1	0.03	ORF1b:C1809-	1	0.07	ORF1b:S1702-	2	0.03	S:F817-	23	0.03
Maharashtra	ORF1a:W1514-	1	0.03	ORF1b:F1810-	1	0.07	ORF1b:H1712-	2	0.03	S:L141-	23	0.03
Maharashtra	ORF1a:S1515-	1	0.03	ORF1b:V1811-	1	0.07	ORF1b:F1757-	2	0.03	S:A243-	23	0.03
Maharashtra	ORF1a:Y1516-	1	0.03	ORF1b:K1812-	1	0.07	ORF1b:D1758-	2	0.03	S:Y266-	23	0.03
Maharashtra	ORF1a:S1517-	1	0.03	ORF1b:R1813-	1	0.07	ORF1b:Y1759-	2	0.03	S:V267-	23	0.03
Maharashtra	ORF1a:G1518-	1	0.03	ORF1b:V1814-	1	0.07	ORF1b:V1760-	2	0.03	S:G268-	23	0.03
Maharashtra	ORF1a:Q1519-	1	0.03	ORF1b:D1815-	1	0.07	ORF1b:Y1761-	2	0.03	S:Y269-	23	0.03
Maharashtra	ORF1a:S1520-	1	0.03	ORF1b:W1816-	1	0.07	ORF1b:N1762-	2	0.03	S:L270-	23	0.03
Maharashtra	ORF1a:T1521-	1	0.03	ORF1b:T1817-	1	0.07	ORF1b:A1798-	2	0.03	S:S721-	23	0.03
Maharashtra	ORF1a:Q1522-	1	0.03	ORF1b:I1818-	1	0.07	ORF1b:I1799-	2	0.03	ORF1a:Y1975-	23	0.03
Maharashtra	ORF1a:L1523-	1	0.03	ORF1b:E1819-	1	0.07	ORF1b:M1800-	2	0.03	ORF1a:T1976-	23	0.03
Maharashtra	ORF1a:G1524-	1	0.03	ORF1b:Y1820-	1	0.07	ORF1b:T1801-	2	0.03	ORF1a:P1977-	23	0.03
Maharashtra	ORF1a:I1525-	1	0.03	ORF1b:P1821-	1	0.07	ORF1b:R1802-	2	0.03	ORF1a:S1978-	23	0.03
Maharashtra	ORF1a:E1526-	1	0.03	ORF1b:I1822-	1	0.07	ORF1b:C1803-	2	0.03	ORF1a:F1979-	23	0.03
Maharashtra	ORF1a:F1527-	1	0.03	ORF1b:I1823-	1	0.07	ORF1b:L1804-	2	0.03	ORF1a:K1980-	23	0.03
Maharashtra	ORF1a:L1528-	1	0.03	ORF1b:G1824-	1	0.07	ORF1b:A1805-	2	0.03	ORF1a:V2047-	23	0.03
Maharashtra	ORF1a:K1529-	1	0.03	ORF1b:D1825-	1	0.07	ORF1b:V1806-	2	0.03	ORF1a:S2048-	23	0.03
Maharashtra	ORF1a:R1530-	1	0.03	ORF1b:E1826-	1	0.07	ORF1b:D1825-	2	0.03	ORF1a:E2049-	23	0.03
Maharashtra	ORF1a:G1531-	1	0.03	ORF1b:L1827-	1	0.07	ORF1b:E1826-	2	0.03	ORF1a:E2050-	23	0.03
Maharashtra	ORF1a:D1532-	1	0.03	ORF1b:K1828-	1	0.07	ORF1b:L1827-	2	0.03	ORF1a:V2051-	23	0.03
Maharashtra	ORF1a:G1548-	1	0.03	ORF1b:I1829-	1	0.07	ORF1b:K1828-	2	0.03	ORF1a:V2052-	23	0.03

Maharashtra	ORF1a:E1549-	1	0.03	ORF1b:N1830-	1	0.07	ORF1b:I1829-	2	0.03	ORF1a:E2053-	23	0.03
Maharashtra	ORF1a:V1550-	1	0.03	ORF1b:A1831-	1	0.07	ORF1b:R1834-	2	0.03	ORF1a:N2054-	23	0.03
Maharashtra	ORF1a:I1551-	1	0.03	ORF1b:A1832-	1	0.07	ORF1b:K1835-	2	0.03	ORF1a:P2055-	23	0.03
Maharashtra	ORF1a:T1552-	1	0.03	ORF1b:C1833-	1	0.07	ORF1b:V1870-	2	0.03	ORF1a:T2056-	23	0.03
Maharashtra	ORF1a:F1553-	1	0.03	ORF1b:R1834-	1	0.07	ORF1b:E1871-	2	0.03	ORF1a:S2083-	23	0.03
Maharashtra	ORF1a:D1554-	1	0.03	ORF1b:K1835-	1	0.07	ORF1b:W1872-	2	0.03	ORF1a:I2359-	22	0.03
Maharashtra	ORF1a:N1555-	1	0.03	ORF1b:V1836-	1	0.07	ORF1b:K1873-	2	0.03	ORF1a:S2360-	22	0.03
Maharashtra	ORF1a:L1556-	1	0.03	ORF1b:Q1837-	1	0.07	ORF1b:F1874-	2	0.03	ORF1a:M2383-	22	0.03
Maharashtra	ORF1a:K1557-	1	0.03	ORF1b:H1838-	1	0.07	ORF1b:Y1875-	2	0.03	ORF1a:Y2384-	22	0.03
Maharashtra	ORF1a:T1558-	1	0.03	ORF1b:M1839-	1	0.07	ORF1b:D1876-	2	0.03	ORF1a:I2385-	22	0.03
Maharashtra	ORF1a:L1559-	1	0.03	ORF1b:V1840-	1	0.07	ORF1b:Y1892-	2	0.03	ORF1a:F2386-	22	0.03
Maharashtra	ORF1a:L1560-	1	0.03	ORF1b:V1841-	1	0.07	ORF1b:S1893-	2	0.03	ORF1a:M2411-	22	0.03
Maharashtra	ORF1a:S1561-	1	0.03	ORF1b:K1842-	1	0.07	ORF1b:Y1894-	2	0.03	ORF1a:C2412-	22	0.03
Maharashtra	ORF1a:L1562-	1	0.03	ORF1b:A1843-	1	0.07	ORF1b:A1895-	2	0.03	ORF1a:Y2413-	22	0.03
Maharashtra	ORF1a:R1563-	1	0.03	ORF1b:A1844-	1	0.07	ORF1b:T1896-	2	0.03	ORF1a:K2414-	22	0.03
Maharashtra	ORF1a:E1564-	1	0.03	ORF1b:L1845-	1	0.07	ORF1b:H1897-	2	0.03	ORF1a:R2415-	22	0.03
Maharashtra	ORF1a:V1565-	1	0.03	ORF1b:L1846-	1	0.07	ORF1b:S1898-	2	0.03	ORF1a:N2454-	22	0.03
Maharashtra	ORF1a:R1566-	1	0.03	ORF1b:A1847-	1	0.07	ORF1a:V561-	2	0.03	ORF1a:C2455-	22	0.03
Maharashtra	ORF1a:T1567-	1	0.03	ORF1b:D1848-	1	0.07	ORF1a:L562-	2	0.03	ORF1a:D2456-	22	0.03
Maharashtra	ORF1a:I1568-	1	0.03	ORF1b:K1849-	1	0.07	ORF1a:S574-	2	0.03	ORF1a:A2460-	22	0.03
Maharashtra	ORF1a:K1569-	1	0.03	ORF1b:F1850-	1	0.07	ORF1a:Q575-	2	0.03	ORF1a:G2461-	22	0.03
Maharashtra	ORF1a:V1570-	1	0.03	ORF1b:P1851-	1	0.07	ORF1a:Y576-	2	0.03	ORF1a:H2520-	22	0.03
Maharashtra	ORF1a:F1571-	1	0.03	ORF1b:V1852-	1	0.07	ORF1a:S577-	2	0.03	ORF1a:T2756-	22	0.03
Maharashtra	ORF1a:T1572-	1	0.03	ORF1b:L1853-	1	0.07	ORF1a:L578-	2	0.03	ORF1a:D3022-	22	0.03
Maharashtra	ORF1a:K1791-	1	0.03	ORF1b:H1854-	1	0.07	ORF1a:R579-	2	0.03	ORF1a:A3023-	22	0.03
Maharashtra	ORF1a:Q1792-	1	0.03	ORF1b:D1855-	1	0.07	ORF1a:L580-	2	0.03	ORF1a:V3024-	22	0.03
Maharashtra	ORF1a:A1793-	1	0.03	ORF1b:I1856-	1	0.07	ORF1a:D582-	2	0.03	ORF1a:N3025-	22	0.03
Maharashtra	ORF1a:T1794-	1	0.03	ORF1b:G1857-	1	0.07	ORF1a:A583-	2	0.03	ORF1a:V3367-	22	0.03
Maharashtra	ORF1a:K1795-	1	0.03	ORF1b:N1858-	1	0.07	ORF1a:A599-	2	0.03	ORF1a:Q3390-	22	0.03
Maharashtra	ORF1a:Y1796-	1	0.03	ORF1b:P1859-	1	0.07	ORF1a:Y600-	2	0.03	ORF1a:F3422-	22	0.03
Maharashtra	ORF1a:L1797-	1	0.03	ORF1b:K1860-	1	0.07	ORF1a:I601-	2	0.03	ORF1a:C3423-	22	0.03
Maharashtra	ORF1a:V1798-	1	0.03	ORF1b:A1861-	1	0.07	ORF1a:T602-	2	0.03	ORF1a:Y3424-	22	0.03
Maharashtra	ORF1a:Q1799-	1	0.03	ORF1b:I1862-	1	0.07	ORF1a:G603-	2	0.03	ORF1a:A3220-	22	0.03
Maharashtra	ORF1a:H1818-	1	0.03	ORF1b:K1863-	1	0.07	ORF1a:G604-	2	0.03	ORF1a:M3221-	22	0.03
Maharashtra	ORF1a:G1819-	1	0.03	ORF1b:C1864-	1	0.07	ORF1a:Q611-	2	0.03	ORF1a:D3222-	22	0.03
Maharashtra	ORF1a:T1820-	1	0.03	ORF1b:V1865-	1	0.07	ORF1a:W612-	2	0.03	ORF1a:T3223-	22	0.03
Maharashtra	ORF1a:L1845-	1	0.03	ORF1b:P1866-	1	0.07	ORF1a:L613-	2	0.03	ORF1a:V1391-	22	0.03
Maharashtra	ORF1a:Y1846-	1	0.03	ORF1b:Q1867-	1	0.07	ORF1a:T614-	2	0.03	ORF1a:V1399-	22	0.03
Maharashtra	ORF1a:V1866-	1	0.03	ORF1b:A1868-	1	0.07	ORF1a:K623-	2	0.03	ORF1a:S1400-	22	0.03
Maharashtra	ORF1a:F1867-	1	0.03	ORF1b:D1869-	1	0.07	ORF1a:W646-	2	0.03	ORF1a:G1408-	22	0.03
Maharashtra	ORF1a:Y1868-	1	0.03	ORF1b:V1870-	1	0.07	ORF1a:E647-	2	0.03	ORF1a:I1409-	22	0.03
Maharashtra	ORF1a:K1869-	1	0.03	ORF1b:E1871-	1	0.07	ORF1a:I648-	2	0.03	ORF1a:K1410-	22	0.03
Maharashtra	ORF1a:E1870-	1	0.03	ORF1b:W1872-	1	0.07	ORF1a:V649-	2	0.03	ORF1a:I1411-	22	0.03
Maharashtra	ORF1a:N1871-	1	0.03	ORF1b:K1873-	1	0.07	ORF1a:A668-	2	0.03	ORF1a:Q1412-	22	0.03
Maharashtra	ORF1a:S1872-	1	0.03	ORF1b:F1874-	1	0.07	ORF1a:K669-	2	0.03	ORF1a:V1415-	22	0.03
Maharashtra	ORF1a:D1903-	1	0.03	ORF1b:Y1875-	1	0.07	ORF1a:E670-	2	0.03	ORF1a:N1540-	22	0.03
Maharashtra	ORF1a:N1904-	1	0.03	ORF1b:D1876-	1	0.07	ORF1a:I671-	2	0.03	ORF1a:P1541-	22	0.03
Maharashtra	ORF1a:S1905-	1	0.03	ORF1b:A1877-	1	0.07	ORF1a:K672-	2	0.03	ORF1a:T1542-	22	0.03
Maharashtra	ORF1a:Y1906-	1	0.03	ORF1b:Q1878-	1	0.07	ORF1a:E673-	2	0.03	ORF1a:T1543-	22	0.03
Maharashtra	ORF1a:F1907-	1	0.03	ORF1b:P1879-	1	0.07	ORF1a:S674-	2	0.03	ORF1a:F1544-	22	0.03
Maharashtra	ORF1a:F1937-	1	0.03	ORF1b:C1880-	1	0.07	ORF1a:V675-	2	0.03	ORF1a:V1570-	22	0.03
Maharashtra	ORF1a:A1938-	1	0.03	ORF1b:S1881-	1	0.07	ORF1a:Q676-	2	0.03	ORF1a:E1630-	22	0.03
Maharashtra	ORF1a:D1939-	1	0.03	ORF1b:D1882-	1	0.07	ORF1a:T677-	2	0.03	ORF1a:A1631-	22	0.03
Maharashtra	ORF1a:D1940-	1	0.03	ORF1b:K1883-	1	0.07	ORF1a:F678-	2	0.03	ORF1a:F1632-	22	0.03
Maharashtra	ORF1a:L1941-	1	0.03	ORF1b:A1884-	1	0.07	ORF1a:F679-	2	0.03	ORF1a:E1633-	22	0.03
Maharashtra	ORF1a:L1963-	1	0.03	ORF1b:Y1885-	1	0.07	ORF1a:K680-	2	0.03	ORF1a:I1667-	22	0.03
Maharashtra	ORF1a:N1964-	1	0.03	ORF1b:K1886-	1	0.07	ORF1a:L681-	2	0.03	ORF1a:K1689-	22	0.03
Maharashtra	ORF1a:K1981-	1	0.03	ORF1b:F1891-	1	0.07	ORF1a:V682-	2	0.03	ORF1a:M1732-	22	0.03
Maharashtra	ORF1a:I1990-	1	0.03	ORF1b:Y1892-	1	0.07	ORF1a:N683-	2	0.03	ORF1a:S1733-	22	0.03
Maharashtra	ORF1a:K2004-	1	0.03	ORF1b:S1893-	1	0.07	ORF1a:K684-	2	0.03	ORF1a:Y1734-	22	0.03
Maharashtra	ORF1a:P2005-	1	0.03	ORF1b:Y1894-	1	0.07	ORF1a:C689-	2	0.03	ORF1a:L1735-	22	0.03
Maharashtra	ORF1a:N2006-	1	0.03	ORF1b:A1895-	1	0.07	ORF1a:A690-	2	0.03	ORF1a:F1736-	22	0.03
Maharashtra	ORF1a:T2007-	1	0.03	ORF1b:T1896-	1	0.07	ORF1a:D691-	2	0.03	ORF1a:Q1737-	22	0.03
Maharashtra	ORF1a:W2008-	1	0.03	ORF1b:H1897-	1	0.07	ORF1a:N704-	2	0.03	ORF1a:H1738-	22	0.03
Maharashtra	ORF1a:C2009-	1	0.03	ORF1b:S1898-	1	0.07	ORF1a:L705-	2	0.03	ORF1a:A1739-	22	0.03
Maharashtra	ORF1a:I2010-	1	0.03	ORF1b:D1899-	1	0.07	ORF1a:G706-	2	0.03	ORF1a:N1740-	22	0.03
Maharashtra	ORF1a:L2028-	1	0.03	ORF1b:K1900-	1	0.07	ORF1a:E707-	2	0.03	ORF1a:Y1776-	22	0.03
Maharashtra	ORF1a:K2029-	1	0.03	ORF1b:F1901-	1	0.07	ORF1a:T708-	2	0.03	S:F275-	22	0.03
Maharashtra	ORF1a:L2062-	1	0.03	ORF1b:T1902-	1	0.07	ORF1a:F709-	2	0.03	S:K835-	22	0.03
Maharashtra	ORF1a:E2063-	1	0.03	ORF1b:D1903-	1	0.07	ORF1a:V710-	2	0.03	S:Q836-	22	0.03

Maharashtra	ORF1a:C2064-	1	0.03	ORF1b:G1904-	1	0.07	ORF1a:T711-	2	0.03	S:G842-	22	0.03
Maharashtra	ORF1a:V2090-	1	0.03	ORF1b:V1905-	1	0.07	ORF1a:H712-	2	0.03	S:T881-	22	0.03
Maharashtra	ORF1a:G2091-	1	0.03	ORF1b:C1906-	1	0.07	ORF1a:S713-	2	0.03	S:I882-	22	0.03
Maharashtra	ORF1a:H2092-	1	0.03	ORF1b:L1907-	1	0.07	ORF1a:L729-	2	0.03	S:L244-	22	0.03
Maharashtra	ORF1a:T2093-	1	0.03	ORF1b:F1908-	1	0.07	ORF1a:L730-	2	0.03	S:P209-	22	0.03
Maharashtra	ORF1a:D2094-	1	0.03	ORF1b:W1909-	1	0.07	ORF1a:M731-	2	0.03	ORF1a:G1782-	22	0.03
Maharashtra	ORF1a:L2127-	1	0.03	ORF1b:N1910-	1	0.07	ORF1a:P732-	2	0.03	ORF1a:V1783-	22	0.03
Maharashtra	ORF1a:A2128-	1	0.03	ORF1b:C1911-	1	0.07	ORF1a:L733-	2	0.03	ORF1a:Q1784-	22	0.03
Maharashtra	ORF1a:A2129-	1	0.03	ORF1b:N1912-	1	0.07	ORF1a:P748-	2	0.03	ORF1a:I1785-	22	0.03
Maharashtra	ORF1a:V2130-	1	0.03	ORF1b:V1913-	1	0.07	ORF1a:T749-	2	0.03	ORF1a:P1786-	22	0.03
Maharashtra	ORF1a:P2144-	1	0.03	ORF1b:D1914-	1	0.07	ORF1a:E750-	2	0.03	ORF1a:C1787-	22	0.03
Maharashtra	ORF1a:T2153-	1	0.03	ORF1b:R1915-	1	0.07	ORF1a:V751-	2	0.03	ORF1a:T1788-	22	0.03
Maharashtra	ORF1a:C2160-	1	0.03	ORF1b:Y1916-	1	0.07	ORF1a:L752-	2	0.03	ORF1a:C1789-	22	0.03
Maharashtra	ORF1a:L2161-	1	0.03	ORF1b:P1917-	1	0.07	ORF1a:T753-	2	0.03	ORF1a:I2057-	22	0.03
Maharashtra	ORF1a:N2162-	1	0.03	ORF1b:A1918-	1	0.07	ORF1a:V757-	2	0.03	ORF1a:D2060-	22	0.03
Maharashtra	ORF1a:R2163-	1	0.03	ORF1b:N1919-	1	0.07	ORF1a:L758-	2	0.03	ORF1a:V2061-	22	0.03
Maharashtra	ORF1a:V2164-	1	0.03	ORF1b:S1920-	1	0.07	ORF1a:K759-	2	0.03	ORF1a:L2077-	22	0.03
Maharashtra	ORF1a:C2165-	1	0.03	ORF1b:I1921-	1	0.07	ORF1a:T760-	2	0.03	ORF1a:K2078-	22	0.03
Maharashtra	ORF1a:T2166-	1	0.03	ORF1b:F1969-	1	0.07	ORF1a:G761-	2	0.03	ORF1a:P2079-	22	0.03
Maharashtra	ORF1a:N2167-	1	0.03	ORF1b:Y1970-	1	0.07	ORF1a:D762-	2	0.03	ORF1a:A2080-	22	0.03
Maharashtra	ORF1a:Y2168-	1	0.03	ORF1b:Y1971-	1	0.07	ORF1a:L763-	2	0.03	ORF1a:N2081-	22	0.03
Maharashtra	ORF1a:M2169-	1	0.03	ORF1b:S1972-	1	0.07	ORF1a:Q764-	2	0.03	ORF1a:N2082-	22	0.03
Maharashtra	ORF1a:P2170-	1	0.03	ORF1b:D1973-	1	0.07	ORF1a:P765-	2	0.03	ORF1a:L2084-	22	0.03
Maharashtra	ORF1a:Y2171-	1	0.03	ORF1b:S1974-	1	0.07	ORF1a:L766-	2	0.03	ORF1a:E2088-	22	0.03
Maharashtra	ORF1a:M2194-	1	0.03	ORF1b:P1975-	1	0.07	ORF1a:T770-	2	0.03	ORF1a:E2089-	22	0.03
Maharashtra	ORF1a:P2195-	1	0.03	ORF1b:C1976-	1	0.07	ORF1a:S771-	2	0.03	ORF1a:A2097-	22	0.03
Maharashtra	ORF1a:T2196-	1	0.03	ORF1b:E1977-	1	0.07	ORF1a:E772-	2	0.03	ORF1a:A2098-	22	0.03
Maharashtra	ORF1a:T2197-	1	0.03	ORF1b:S1978-	1	0.07	ORF1a:A773-	2	0.03	ORF1a:Y2099-	22	0.03
Maharashtra	ORF1a:I2198-	1	0.03	ORF1b:H1979-	1	0.07	ORF1a:V774-	2	0.03	ORF1a:V2100-	22	0.03
Maharashtra	ORF1a:A2199-	1	0.03	ORF1b:G1980-	1	0.07	ORF1a:C784-	2	0.03	ORF1a:D2101-	22	0.03
Maharashtra	ORF1a:K2200-	1	0.03	ORF1b:K1981-	1	0.07	ORF1a:D795-	2	0.03	ORF1a:V2133-	22	0.03
Maharashtra	ORF1a:N2201-	1	0.03	ORF1b:Q1982-	1	0.07	ORF1a:T796-	2	0.03	ORF1a:P2134-	22	0.03
Maharashtra	ORF1a:T2202-	1	0.03	ORF1b:V1983-	1	0.07	ORF1a:E797-	2	0.03	ORF1a:W2135-	22	0.03
Maharashtra	ORF1a:V2203-	1	0.03	ORF1b:V1984-	1	0.07	ORF1a:K798-	2	0.03	ORF1a:D2136-	22	0.03
Maharashtra	ORF1a:K2204-	1	0.03	ORF1b:S1985-	1	0.07	ORF1a:Y799-	2	0.03	ORF1a:T2137-	22	0.03
Maharashtra	ORF1a:S2205-	1	0.03	ORF1b:D1986-	1	0.07	ORF1a:A801-	2	0.03	ORF1a:T2154-	22	0.03
Maharashtra	ORF1a:V2206-	1	0.03	ORF1b:I1987-	1	0.07	ORF1a:L802-	2	0.03	ORF1a:N2155-	22	0.03
Maharashtra	ORF1a:G2207-	1	0.03	ORF1b:D1988-	1	0.07	ORF1a:N811-	2	0.03	ORF1a:C2409-	21	0.03
Maharashtra	ORF1a:K2208-	1	0.03	ORF1b:Y1989-	1	0.07	ORF1a:T812-	2	0.03	ORF1a:M2410-	21	0.03
Maharashtra	ORF1a:F2209-	1	0.03	ORF1b:V1990-	1	0.07	ORF1a:F813-	2	0.03	ORF1a:T2457-	21	0.03
Maharashtra	ORF1a:C2210-	1	0.03	ORF1b:P1991-	1	0.07	ORF1a:T814-	2	0.03	ORF1a:F2458-	21	0.03
Maharashtra	ORF1a:L2211-	1	0.03	ORF1b:L1992-	1	0.07	ORF1a:L815-	2	0.03	ORF1a:S2462-	21	0.03
Maharashtra	ORF1a:E2212-	1	0.03	ORF1b:K1993-	1	0.07	ORF1a:K816-	2	0.03	ORF1a:K2478-	21	0.03
Maharashtra	ORF1a:A2213-	1	0.03	ORF1b:S1994-	1	0.07	ORF1a:G817-	2	0.03	ORF1a:A2745-	21	0.03
Maharashtra	ORF1a:S2214-	1	0.03	ORF1b:A1995-	1	0.07	ORF1a:G818-	2	0.03	ORF1a:T2746-	21	0.03
Maharashtra	ORF1a:F2215-	1	0.03	ORF1b:T1996-	1	0.07	ORF1a:A819-	2	0.03	ORF1a:T2755-	21	0.03
Maharashtra	ORF1a:N2228-	1	0.03	ORF1b:C1997-	1	0.07	ORF1a:P820-	2	0.03	ORF1a:N2768-	21	0.03
Maharashtra	ORF1a:I2229-	1	0.03	ORF1b:I1998-	1	0.07	ORF1a:T821-	2	0.03	ORF1a:W2769-	21	0.03
Maharashtra	ORF1a:I2230-	1	0.03	ORF1b:T1999-	1	0.07	ORF1a:K822-	2	0.03	ORF1a:L2770-	21	0.03
Maharashtra	ORF1a:I2231-	1	0.03	ORF1b:R2000-	1	0.07	ORF1a:V823-	2	0.03	ORF1a:V2930-	21	0.03
Maharashtra	ORF1a:W2232-	1	0.03	ORF1b:C2001-	1	0.07	ORF1a:T824-	2	0.03	ORF1a:P2931-	21	0.03
Maharashtra	ORF1a:L2236-	1	0.03	ORF1b:N2002-	1	0.07	ORF1a:F825-	2	0.03	ORF1a:Y2932-	21	0.03
Maharashtra	ORF1a:S2237-	1	0.03	ORF1b:L2003-	1	0.07	ORF1a:G826-	2	0.03	ORF1a:C2933-	21	0.03
Maharashtra	ORF1a:V2238-	1	0.03	ORF1b:G2004-	1	0.07	ORF1a:D827-	2	0.03	ORF1a:Y2968-	21	0.03
Maharashtra	ORF1a:C2239-	1	0.03	ORF1b:G2005-	1	0.07	ORF1a:D828-	2	0.03	ORF1a:Y3144-	21	0.03
Maharashtra	ORF1a:L2240-	1	0.03	ORF1b:A2006-	1	0.07	ORF1a:T829-	2	0.03	ORF1a:I3145-	21	0.03
Maharashtra	ORF1a:G2241-	1	0.03	ORF1b:V2007-	1	0.07	ORF1a:R848-	2	0.03	ORF1a:C3147-	21	0.03
Maharashtra	ORF1a:S2242-	1	0.03	ORF1b:C2008-	1	0.07	ORF1a:I849-	2	0.03	ORF1a:V3298-	21	0.03
Maharashtra	ORF1a:L2243-	1	0.03	ORF1b:R2009-	1	0.07	ORF1a:D850-	2	0.03	ORF1a:V3299-	21	0.03
Maharashtra	ORF1a:I2244-	1	0.03	ORF1b:H2010-	1	0.07	ORF1a:K851-	2	0.03	ORF1a:Y3300-	21	0.03
Maharashtra	ORF1a:Y2270-	1	0.03	ORF1b:H2011-	1	0.07	ORF1a:V852-	2	0.03	ORF1a:C3301-	21	0.03
Maharashtra	ORF1a:L2271-	1	0.03	ORF1b:A2012-	1	0.07	ORF1a:L853-	2	0.03	ORF1a:A3379-	21	0.03
Maharashtra	ORF1a:N2272-	1	0.03	ORF1b:N2013-	1	0.07	ORF1a:N854-	2	0.03	ORF1a:C3380-	21	0.03
Maharashtra	ORF1a:S2273-	1	0.03	ORF1b:E2014-	1	0.07	ORF1a:G865-	2	0.03	ORF1a:Y3381-	21	0.03
Maharashtra	ORF1a:T2274-	1	0.03	ORF1b:Y2015-	1	0.07	ORF1a:T866-	2	0.03	ORF1a:T3432-	21	0.03
Maharashtra	ORF1a:N2275-	1	0.03	ORF1b:R2016-	1	0.07	ORF1a:E867-	2	0.03	ORF1a:Q3127-	21	0.03
Maharashtra	ORF1a:D2299-	1	0.03	ORF1b:L2017-	1	0.07	ORF1a:D877-	2	0.03	ORF1a:W3128-	21	0.03
Maharashtra	ORF1a:T2300-	1	0.03	ORF1b:Y2018-	1	0.07	ORF1a:A878-	2	0.03	ORF1a:T3224-	21	0.03
Maharashtra	ORF1a:Y2301-	1	0.03	ORF1b:L2019-	1	0.07	ORF1a:V879-	2	0.03	ORF1a:S3225-	21	0.03
Maharashtra	ORF1a:P2302-	1	0.03	ORF1b:D2020-	1	0.07	ORF1a:I880-	2	0.03	ORF1a:Y3226-	21	0.03

Maharashtra	ORF1a:L2364-	1	0.03	ORF1b:A2021-	1	0.07	ORF1a:K881-	2	0.03	ORF1a:R3227-	21	0.03
Maharashtra	ORF1a:M2365-	1	0.03	ORF1b:Y2022-	1	0.07	ORF1a:D909-	2	0.03	ORF1a:E3228-	21	0.03
Maharashtra	ORF1a:W2366-	1	0.03	ORF1b:N2023-	1	0.07	ORF1a:E910-	2	0.03	ORF1a:A3229-	21	0.03
Maharashtra	ORF1a:L2367-	1	0.03	ORF1b:M2024-	1	0.07	ORF1a:S911-	2	0.03	ORF1a:A3230-	21	0.03
Maharashtra	ORF1a:V2381-	1	0.03	ORF1b:M2025-	1	0.07	ORF1a:G912-	2	0.03	ORF1a:C3231-	21	0.03
Maharashtra	ORF1a:R2382-	1	0.03	ORF1b:I2026-	1	0.07	ORF1a:E913-	2	0.03	ORF1a:I1301-	21	0.03
Maharashtra	ORF1a:M2383-	1	0.03	ORF1b:S2027-	1	0.07	ORF1a:F914-	2	0.03	ORF1a:P1302-	21	0.03
Maharashtra	ORF1a:Y2384-	1	0.03	ORF1b:A2028-	1	0.07	ORF1a:K915-	2	0.03	ORF1a:Y1337-	21	0.03
Maharashtra	ORF1a:I2385-	1	0.03	ORF1b:G2029-	1	0.07	ORF1a:Y925-	2	0.03	ORF1a:T1338-	21	0.03
Maharashtra	ORF1a:F2386-	1	0.03	ORF1b:F2030-	1	0.07	ORF1a:P926-	2	0.03	ORF1a:V1339-	21	0.03
Maharashtra	ORF1a:F2387-	1	0.03	ORF1b:S2031-	1	0.07	ORF1a:P927-	2	0.03	ORF1a:E1340-	21	0.03
Maharashtra	ORF1a:A2388-	1	0.03	ORF1b:L2032-	1	0.07	ORF1a:D928-	2	0.03	ORF1a:E1363-	21	0.03
Maharashtra	ORF1a:S2389-	1	0.03	ORF1b:W2033-	1	0.07	ORF1a:E929-	2	0.03	ORF1a:K1364-	21	0.03
Maharashtra	ORF1a:F2390-	1	0.03	ORF1b:V2034-	1	0.07	ORF1a:D930-	2	0.03	ORF1a:Q1365-	21	0.03
Maharashtra	ORF1a:Y2391-	1	0.03	ORF1b:Y2041-	1	0.07	ORF1a:E931-	2	0.03	ORF1a:E1366-	21	0.03
Maharashtra	ORF1a:Y2392-	1	0.03	ORF1b:N2042-	1	0.07	ORF1a:E932-	2	0.03	ORF1a:I1367-	21	0.03
Maharashtra	ORF1a:V2393-	1	0.03	ORF1b:L2043-	1	0.07	ORF1a:E933-	2	0.03	ORF1a:E1413-	21	0.03
Maharashtra	ORF1a:W2394-	1	0.03	ORF1b:W2044-	1	0.07	ORF1a:G934-	2	0.03	ORF1a:G1414-	21	0.03
Maharashtra	ORF1a:K2395-	1	0.03	ORF1b:N2045-	1	0.07	ORF1a:D935-	2	0.03	ORF1a:V1416-	21	0.03
Maharashtra	ORF1a:D2402-	1	0.03	ORF1b:T2046-	1	0.07	ORF1a:C936-	2	0.03	ORF1a:D1417-	21	0.03
Maharashtra	ORF1a:G2403-	1	0.03	ORF1b:F2047-	1	0.07	ORF1a:E937-	2	0.03	ORF1a:S1492-	21	0.03
Maharashtra	ORF1a:C2404-	1	0.03	ORF1b:T2048-	1	0.07	ORF1a:E938-	2	0.03	ORF1a:S1493-	21	0.03
Maharashtra	ORF1a:N2405-	1	0.03	ORF1b:R2049-	1	0.07	ORF1a:E939-	2	0.03	ORF1a:S1494-	21	0.03
Maharashtra	ORF1a:S2406-	1	0.03	ORF1b:L2050-	1	0.07	ORF1a:E940-	2	0.03	ORF1a:K1495-	21	0.03
Maharashtra	ORF1a:S2407-	1	0.03	ORF1b:Q2051-	1	0.07	ORF1a:L969-	2	0.03	ORF1a:T1496-	21	0.03
Maharashtra	ORF1a:T2408-	1	0.03	ORF1b:S2052-	1	0.07	ORF1a:L980-	2	0.03	ORF1a:P1497-	21	0.03
Maharashtra	ORF1a:C2409-	1	0.03	ORF1b:L2053-	1	0.07	ORF1a:G993-	2	0.03	ORF1a:W1514-	21	0.03
Maharashtra	ORF1a:M2410-	1	0.03	ORF1b:E2054-	1	0.07	ORF1a:S994-	2	0.03	ORF1a:S1515-	21	0.03
Maharashtra	ORF1a:M2411-	1	0.03	ORF1b:N2055-	1	0.07	ORF1a:E995-	2	0.03	ORF1a:Y1516-	21	0.03
Maharashtra	ORF1a:C2412-	1	0.03	ORF1b:V2056-	1	0.07	ORF1a:Q1003-	2	0.03	ORF1a:S1517-	21	0.03
Maharashtra	ORF1a:Y2413-	1	0.03	ORF1b:A2057-	1	0.07	ORF1a:T1004-	2	0.03	ORF1a:G1518-	21	0.03
Maharashtra	ORF1a:K2414-	1	0.03	ORF1b:F2058-	1	0.07	ORF1a:I1005-	2	0.03	ORF1a:Q1519-	21	0.03
Maharashtra	ORF1a:R2415-	1	0.03	ORF1b:N2059-	1	0.07	ORF1a:V1006-	2	0.03	ORF1a:S1520-	21	0.03
Maharashtra	ORF1a:N2416-	1	0.03	ORF1b:V2060-	1	0.07	ORF1a:E1007-	2	0.03	ORF1a:T1521-	21	0.03
Maharashtra	ORF1a:G2441-	1	0.03	ORF1b:V2061-	1	0.07	ORF1a:V1008-	2	0.03	ORF1a:Q1522-	21	0.03
Maharashtra	ORF1a:K2442-	1	0.03	ORF1b:N2062-	1	0.07	ORF1a:Q1009-	2	0.03	ORF1a:S1539-	21	0.03
Maharashtra	ORF1a:G2443-	1	0.03	ORF1b:K2063-	1	0.07	ORF1a:P1010-	2	0.03	ORF1a:E1564-	21	0.03
Maharashtra	ORF1a:F2444-	1	0.03	ORF1b:G2064-	1	0.07	ORF1a:Q1011-	2	0.03	ORF1a:V1565-	21	0.03
Maharashtra	ORF1a:C2445-	1	0.03	ORF1b:H2065-	1	0.07	ORF1a:L1012-	2	0.03	ORF1a:R1566-	21	0.03
Maharashtra	ORF1a:K2446-	1	0.03	ORF1b:F2066-	1	0.07	ORF1a:Q1021-	2	0.03	ORF1a:Y1590-	21	0.03
Maharashtra	ORF1a:L2447-	1	0.03	ORF1b:D2067-	1	0.07	ORF1a:T1022-	2	0.03	ORF1a:G1591-	21	0.03
Maharashtra	ORF1a:H2448-	1	0.03	ORF1b:G2068-	1	0.07	ORF1a:I1023-	2	0.03	ORF1a:Q1592-	21	0.03
Maharashtra	ORF1a:N2449-	1	0.03	ORF1b:Q2069-	1	0.07	ORF1a:E1024-	2	0.03	ORF1a:Q1593-	21	0.03
Maharashtra	ORF1a:W2450-	1	0.03	ORF1b:Q2070-	1	0.07	ORF1a:V1025-	2	0.03	ORF1a:F1594-	21	0.03
Maharashtra	ORF1a:N2451-	1	0.03	ORF1b:G2071-	1	0.07	ORF1a:N1026-	2	0.03	ORF1a:R1729-	21	0.03
Maharashtra	ORF1a:C2452-	1	0.03	ORF1b:E2072-	1	0.07	ORF1a:N1042-	2	0.03	ORF1a:E1730-	21	0.03
Maharashtra	ORF1a:V2453-	1	0.03	ORF1b:V2073-	1	0.07	ORF1a:K1050-	2	0.03	ORF1a:T1731-	21	0.03
Maharashtra	ORF1a:N2454-	1	0.03	ORF1b:P2074-	1	0.07	ORF1a:K1051-	2	0.03	ORF1a:Q1800-	21	0.03
Maharashtra	ORF1a:C2455-	1	0.03	ORF1b:V2075-	1	0.07	ORF1a:V1052-	2	0.03	ORF1a:E1801-	21	0.03
Maharashtra	ORF1a:D2456-	1	0.03	ORF1b:S2076-	1	0.07	ORF1a:K1053-	2	0.03	ORF1a:S1802-	21	0.03
Maharashtra	ORF1a:T2457-	1	0.03	ORF1b:I2077-	1	0.07	ORF1a:P1054-	2	0.03	ORF1a:P1803-	21	0.03
Maharashtra	ORF1a:F2458-	1	0.03	ORF1b:I2078-	1	0.07	ORF1a:T1055-	2	0.03	ORF1a:F1804-	21	0.03
Maharashtra	ORF1a:C2459-	1	0.03	ORF1b:N2079-	1	0.07	ORF1a:N1059-	2	0.03	ORF1a:V1805-	21	0.03
Maharashtra	ORF1a:A2460-	1	0.03	ORF1b:N2080-	1	0.07	ORF1a:A1060-	2	0.03	ORF1a:Y1831-	21	0.03
Maharashtra	ORF1a:G2461-	1	0.03	ORF1b:T2081-	1	0.07	ORF1a:A1061-	2	0.03	ORF1a:Q1832-	21	0.03
Maharashtra	ORF1a:S2462-	1	0.03	ORF1b:V2082-	1	0.07	ORF1a:N1062-	2	0.03	ORF1a:C1833-	21	0.03
Maharashtra	ORF1a:T2463-	1	0.03	ORF1b:Y2083-	1	0.07	ORF1a:V1063-	2	0.03	ORF1a:G1834-	21	0.03
Maharashtra	ORF1a:F2464-	1	0.03	ORF1b:T2084-	1	0.07	ORF1a:Y1064-	2	0.03	ORF1a:H1835-	21	0.03
Maharashtra	ORF1a:Q2476-	1	0.03	ORF1b:K2085-	1	0.07	ORF1a:L1075-	2	0.03	ORF1a:Y1836-	21	0.03
Maharashtra	ORF1a:F2477-	1	0.03	ORF1b:V2086-	1	0.07	ORF1a:N1076-	2	0.03	ORF1a:G1861-	21	0.03
Maharashtra	ORF1a:K2478-	1	0.03	ORF1b:D2087-	1	0.07	ORF1a:K1077-	2	0.03	ORF1a:P1862-	21	0.03
Maharashtra	ORF1a:R2479-	1	0.03	ORF1b:G2088-	1	0.07	ORF1a:Y1090-	2	0.03	ORF1a:T1875-	21	0.03
Maharashtra	ORF1a:P2480-	1	0.03	ORF1b:V2089-	1	0.07	ORF1a:I1091-	2	0.03	ORF1a:T1890-	21	0.03
Maharashtra	ORF1a:I2481-	1	0.03	ORF1b:D2090-	1	0.07	ORF1a:A1092-	2	0.03	ORF1a:E1891-	21	0.03
Maharashtra	ORF1a:N2482-	1	0.03	ORF1b:V2091-	1	0.07	ORF1a:N1094-	2	0.03	ORF1a:I1892-	21	0.03
Maharashtra	ORF1a:P2483-	1	0.03	ORF1b:E2092-	1	0.07	ORF1a:G1095-	2	0.03	ORF1a:D1893-	21	0.03
Maharashtra	ORF1a:T2484-	1	0.03	ORF1b:L2093-	1	0.07	ORF1a:P1096-	2	0.03	ORF1a:P1894-	21	0.03
Maharashtra	ORF1a:D2485-	1	0.03	ORF1b:F2094-	1	0.07	ORF1a:L1097-	2	0.03	ORF1a:K1895-	21	0.03
Maharashtra	ORF1a:D2492-	1	0.03	ORF1b:E2095-	1	0.07	ORF1a:K1098-	2	0.03	ORF1a:F1925-	21	0.03
Maharashtra	ORF1a:S2493-	1	0.03	ORF1b:N2096-	1	0.07	ORF1a:G1107-	2	0.03	S:Q493-	21	0.03

Maharashtra	ORF1a:V2494-	1	0.03	ORF1b:K2097-	1	0.07	ORF1a:H1108-	2	0.03	S:S494-	21	0.03
Maharashtra	ORF1a:T2495-	1	0.03	ORF1b:T2098-	1	0.07	ORF1a:N1109-	2	0.03	S:Y495-	21	0.03
Maharashtra	ORF1a:V2496-	1	0.03	ORF1b:T2099-	1	0.07	ORF1a:H1113-	2	0.03	S:G496-	21	0.03
Maharashtra	ORF1a:K2497-	1	0.03	ORF1b:L2100-	1	0.07	ORF1a:C1114-	2	0.03	S:F497-	21	0.03
Maharashtra	ORF1a:N2498-	1	0.03	ORF1b:P2101-	1	0.07	ORF1a:L1115-	2	0.03	S:Q498-	21	0.03
Maharashtra	ORF1a:G2499-	1	0.03	ORF1b:V2102-	1	0.07	ORF1a:H1116-	2	0.03	S:Q644-	21	0.03
Maharashtra	ORF1a:S2500-	1	0.03	ORF1b:N2103-	1	0.07	ORF1a:V1117-	2	0.03	S:Y837-	21	0.03
Maharashtra	ORF1a:I2501-	1	0.03	ORF1b:V2104-	1	0.07	ORF1a:V1118-	2	0.03	S:G838-	21	0.03
Maharashtra	ORF1a:H2502-	1	0.03	ORF1b:A2105-	1	0.07	ORF1a:D1127-	2	0.03	S:D839-	21	0.03
Maharashtra	ORF1a:L2503-	1	0.03	ORF1b:F2106-	1	0.07	ORF1a:I1128-	2	0.03	S:C840-	21	0.03
Maharashtra	ORF1a:Y2504-	1	0.03	ORF1b:E2107-	1	0.07	ORF1a:Q1129-	2	0.03	S:G142-	21	0.03
Maharashtra	ORF1a:F2505-	1	0.03	ORF1b:L2108-	1	0.07	ORF1a:E1136-	2	0.03	S:K206-	21	0.03
Maharashtra	ORF1a:D2506-	1	0.03	ORF1b:W2109-	1	0.07	ORF1a:N1137-	2	0.03	S:H207-	21	0.03
Maharashtra	ORF1a:K2507-	1	0.03	ORF1b:A2110-	1	0.07	ORF1a:F1138-	2	0.03	S:T208-	21	0.03
Maharashtra	ORF1a:A2508-	1	0.03	ORF1b:K2111-	1	0.07	ORF1a:N1139-	2	0.03	ORF1a:K1781-	21	0.03
Maharashtra	ORF1a:S2519-	1	0.03	ORF1b:R2112-	1	0.07	ORF1a:Q1140-	2	0.03	ORF1a:G1965-	21	0.03
Maharashtra	ORF1a:H2520-	1	0.03	ORF1b:N2113-	1	0.07	ORF1a:H1141-	2	0.03	ORF1a:D1966-	21	0.03
Maharashtra	ORF1a:F2521-	1	0.03	ORF1b:I2114-	1	0.07	ORF1a:E1142-	2	0.03	ORF1a:V1967-	21	0.03
Maharashtra	ORF1a:V2522-	1	0.03	ORF1b:K2115-	1	0.07	ORF1a:V1143-	2	0.03	ORF1a:V1968-	21	0.03
Maharashtra	ORF1a:N2523-	1	0.03	ORF1b:P2116-	1	0.07	ORF1a:L1144-	2	0.03	ORF1a:A1969-	21	0.03
Maharashtra	ORF1a:L2524-	1	0.03	ORF1b:V2117-	1	0.07	ORF1a:L1145-	2	0.03	ORF1a:I1970-	21	0.03
Maharashtra	ORF1a:D2525-	1	0.03	ORF1b:P2118-	1	0.07	ORF1a:A1146-	2	0.03	ORF1a:D1971-	21	0.03
Maharashtra	ORF1a:N2526-	1	0.03	ORF1b:E2119-	1	0.07	ORF1a:P1147-	2	0.03	ORF1a:Y1972-	21	0.03
Maharashtra	ORF1a:L2527-	1	0.03	ORF1b:V2120-	1	0.07	ORF1a:V1166-	2	0.03	ORF1a:H1974-	21	0.03
Maharashtra	ORF1a:R2528-	1	0.03	ORF1b:K2121-	1	0.07	ORF1a:D1167-	2	0.03	ORF1a:Q2058-	21	0.03
Maharashtra	ORF1a:A2529-	1	0.03	ORF1b:I2122-	1	0.07	ORF1a:T1168-	2	0.03	ORF1a:K2059-	21	0.03
Maharashtra	ORF1a:N2530-	1	0.03	ORF1b:L2123-	1	0.07	ORF1a:R1170-	2	0.03	ORF1a:K2085-	21	0.03
Maharashtra	ORF1a:N2531-	1	0.03	ORF1b:N2124-	1	0.07	ORF1a:T1171-	2	0.03	ORF1a:K2120-	21	0.03
Maharashtra	ORF1a:T2532-	1	0.03	ORF1b:N2125-	1	0.07	ORF1a:N1172-	2	0.03	ORF1a:T2121-	21	0.03
Maharashtra	ORF1a:K2533-	1	0.03	ORF1b:L2126-	1	0.07	ORF1a:V1173-	2	0.03	ORF1a:L2122-	21	0.03
Maharashtra	ORF1a:G2534-	1	0.03	ORF1b:G2127-	1	0.07	ORF1a:Y1174-	2	0.03	ORF1a:A2123-	21	0.03
Maharashtra	ORF1a:S2535-	1	0.03	ORF1b:V2128-	1	0.07	ORF1a:L1175-	2	0.03	ORF1a:M2194-	21	0.03
Maharashtra	ORF1a:L2536-	1	0.03	ORF1b:D2129-	1	0.07	ORF1a:A1176-	2	0.03	ORF1a:K2204-	21	0.03
Maharashtra	ORF1a:P2537-	1	0.03	ORF1b:I2130-	1	0.07	ORF1a:V1177-	2	0.03	ORF1a:S2205-	21	0.03
Maharashtra	ORF1a:I2541-	1	0.03	ORF1b:A2131-	1	0.07	ORF1a:F1178-	2	0.03	ORF1a:V2206-	21	0.03
Maharashtra	ORF1a:V2542-	1	0.03	ORF1b:A2132-	1	0.07	ORF1a:D1179-	2	0.03	ORF1a:G2207-	21	0.03
Maharashtra	ORF1a:F2543-	1	0.03	ORF1b:N2133-	1	0.07	ORF1a:K1180-	2	0.03	ORF1a:C2239-	21	0.03
Maharashtra	ORF1a:D2544-	1	0.03	ORF1b:T2134-	1	0.07	ORF1a:N1181-	2	0.03	ORF1a:L2240-	21	0.03
Maharashtra	ORF1a:G2545-	1	0.03	ORF1b:V2135-	1	0.07	ORF1a:L1182-	2	0.03	ORF1a:G2241-	21	0.03
Maharashtra	ORF1a:K2546-	1	0.03	ORF1b:I2136-	1	0.07	ORF1a:E1196-	2	0.03	ORF1a:S2242-	21	0.03
Maharashtra	ORF1a:S2547-	1	0.03	ORF1b:W2137-	1	0.07	ORF1a:K1197-	2	0.03	ORF1a:L2243-	21	0.03
Maharashtra	ORF1a:K2548-	1	0.03	ORF1b:D2138-	1	0.07	ORF1a:Q1198-	2	0.03	ORF1a:I2244-	21	0.03
Maharashtra	ORF1a:C2549-	1	0.03	ORF1b:Y2139-	1	0.07	ORF1a:V1199-	2	0.03	ORF1a:G1790-	21	0.03
Maharashtra	ORF1a:E2550-	1	0.03	ORF1b:K2140-	1	0.07	ORF1a:E1200-	2	0.03	ORF1a:K2622-	20	0.03
Maharashtra	ORF1a:E2551-	1	0.03	ORF1b:R2141-	1	0.07	ORF1a:Q1201-	2	0.03	ORF1a:N2623-	20	0.03
Maharashtra	ORF1a:S2552-	1	0.03	ORF1b:D2142-	1	0.07	ORF1a:K1202-	2	0.03	ORF1a:V2624-	20	0.03
Maharashtra	ORF1a:S2553-	1	0.03	ORF1b:A2143-	1	0.07	ORF1a:I1203-	2	0.03	ORF1a:S2625-	20	0.03
Maharashtra	ORF1a:A2554-	1	0.03	ORF1b:P2144-	1	0.07	ORF1a:A1204-	2	0.03	ORF1a:L2626-	20	0.03
Maharashtra	ORF1a:Y2560-	1	0.03	ORF1b:A2145-	1	0.07	ORF1a:E1205-	2	0.03	ORF1a:D2627-	20	0.03
Maharashtra	ORF1a:Y2561-	1	0.03	ORF1b:H2146-	1	0.07	ORF1a:I1206-	2	0.03	ORF1a:D2662-	20	0.03
Maharashtra	ORF1a:S2562-	1	0.03	ORF1b:I2147-	1	0.07	ORF1a:P1207-	2	0.03	ORF1a:I2663-	20	0.03
Maharashtra	ORF1a:S2578-	1	0.03	ORF1b:E2164-	1	0.07	ORF1a:K1208-	2	0.03	ORF1a:F2827-	20	0.03
Maharashtra	ORF1a:D2579-	1	0.03	ORF1b:T2165-	1	0.07	ORF1a:P1220-	2	0.03	ORF1a:A2828-	20	0.03
Maharashtra	ORF1a:V2580-	1	0.03	ORF1b:I2166-	1	0.07	ORF1a:S1221-	2	0.03	ORF1a:N2829-	20	0.03
Maharashtra	ORF1a:G2581-	1	0.03	ORF1b:C2167-	1	0.07	ORF1a:V1222-	2	0.03	ORF1a:D2833-	20	0.03
Maharashtra	ORF1a:D2582-	1	0.03	ORF1b:A2168-	1	0.07	ORF1a:E1223-	2	0.03	ORF1a:F2834-	20	0.03
Maharashtra	ORF1a:S2583-	1	0.03	ORF1b:P2169-	1	0.07	ORF1a:Q1224-	2	0.03	ORF1a:D2835-	20	0.03
Maharashtra	ORF1a:A2584-	1	0.03	ORF1b:L2170-	1	0.07	ORF1a:R1225-	2	0.03	ORF1a:T2836-	20	0.03
Maharashtra	ORF1a:E2585-	1	0.03	ORF1b:T2171-	1	0.07	ORF1a:K1226-	2	0.03	ORF1a:A2916-	20	0.03
Maharashtra	ORF1a:V2586-	1	0.03	ORF1b:V2172-	1	0.07	ORF1a:Q1227-	2	0.03	ORF1a:A2917-	20	0.03
Maharashtra	ORF1a:A2587-	1	0.03	ORF1b:F2173-	1	0.07	ORF1a:D1228-	2	0.03	ORF1a:E2918-	20	0.03
Maharashtra	ORF1a:V2588-	1	0.03	ORF1b:F2174-	1	0.07	ORF1a:K1231-	2	0.03	ORF1a:M2957-	20	0.03
Maharashtra	ORF1a:K2589-	1	0.03	ORF1b:D2175-	1	0.07	ORF1a:I1232-	2	0.03	ORF1a:A3070-	20	0.03
Maharashtra	ORF1a:M2590-	1	0.03	ORF1b:G2176-	1	0.07	ORF1a:K1233-	2	0.03	ORF1a:D3296-	20	0.03
Maharashtra	ORF1a:F2591-	1	0.03	ORF1b:R2177-	1	0.07	ORF1a:A1234-	2	0.03	ORF1a:E3553-	20	0.03
Maharashtra	ORF1a:D2592-	1	0.03	ORF1b:V2178-	1	0.07	ORF1a:C1235-	2	0.03	ORF1a:N3159-	20	0.03
Maharashtra	ORF1a:A2593-	1	0.03	ORF1b:D2179-	1	0.07	ORF1a:V1236-	2	0.03	ORF1a:A1306-	20	0.03
Maharashtra	ORF1a:F2598-	1	0.03	ORF1b:G2180-	1	0.07	ORF1a:E1237-	2	0.03	ORF1a:G1307-	20	0.03
Maharashtra	ORF1a:S2599-	1	0.03	ORF1b:Q2181-	1	0.07	ORF1a:E1238-	2	0.03	ORF1a:E1341-	20	0.03
Maharashtra	ORF1a:S2600-	1	0.03	ORF1b:V2182-	1	0.07	ORF1a:V1239-	2	0.03	ORF1a:A1342-	20	0.03

Maharashtra	ORF1a:T2601-	1	0.03	ORF1b:D2183-	1	0.07	ORF1a:F1248-	2	0.03	ORF1a:K1343-	20	0.03
Maharashtra	ORF1a:F2602-	1	0.03	ORF1b:L2184-	1	0.07	ORF1a:L1249-	2	0.03	ORF1a:A1420-	20	0.03
Maharashtra	ORF1a:N2603-	1	0.03	ORF1b:F2185-	1	0.07	ORF1a:T1250-	2	0.03	ORF1a:E1498-	20	0.03
Maharashtra	ORF1a:V2604-	1	0.03	ORF1b:R2186-	1	0.07	ORF1a:E1251-	2	0.03	ORF1a:E1499-	20	0.03
Maharashtra	ORF1a:P2605-	1	0.03	ORF1b:N2187-	1	0.07	ORF1a:N1252-	2	0.03	ORF1a:G1595-	20	0.03
Maharashtra	ORF1a:M2606-	1	0.03	ORF1b:A2188-	1	0.07	ORF1a:L1253-	2	0.03	ORF1a:I1863-	20	0.03
Maharashtra	ORF1a:E2607-	1	0.03	ORF1b:R2189-	1	0.07	ORF1a:L1254-	2	0.03	ORF1a:T1864-	20	0.03
Maharashtra	ORF1a:K2608-	1	0.03	ORF1b:N2190-	1	0.07	ORF1a:L1255-	2	0.03	ORF1a:D1865-	20	0.03
Maharashtra	ORF1a:L2609-	1	0.03	ORF1b:G2191-	1	0.07	ORF1a:Y1256-	2	0.03	ORF1a:L1896-	20	0.03
Maharashtra	ORF1a:K2610-	1	0.03	ORF1b:V2192-	1	0.07	ORF1a:I1257-	2	0.03	ORF1a:D1897-	20	0.03
Maharashtra	ORF1a:T2611-	1	0.03	ORF1b:L2193-	1	0.07	ORF1a:D1258-	2	0.03	ORF1a:V1915-	20	0.03
Maharashtra	ORF1a:L2612-	1	0.03	ORF1b:I2194-	1	0.07	ORF1a:I1259-	2	0.03	ORF1a:P1916-	20	0.03
Maharashtra	ORF1a:V2613-	1	0.03	ORF1b:T2195-	1	0.07	ORF1a:N1260-	2	0.03	ORF1a:W2363-	20	0.03
Maharashtra	ORF1a:A2614-	1	0.03	ORF1b:E2196-	1	0.07	ORF1a:G1261-	2	0.03	ORF1a:R2515-	20	0.03
Maharashtra	ORF1a:T2615-	1	0.03	ORF1b:G2197-	1	0.07	ORF1a:N1262-	2	0.03	S:W152-	20	0.03
Maharashtra	ORF1a:A2616-	1	0.03	ORF1b:S2198-	1	0.07	ORF1a:F2215-	2	0.03	S:S555-	20	0.03
Maharashtra	ORF1a:E2617-	1	0.03	ORF1b:V2199-	1	0.07	ORF1a:L2240-	2	0.03	S:L841-	20	0.03
Maharashtra	ORF1a:A2618-	1	0.03	ORF1b:K2200-	1	0.07	ORF1a:G2241-	2	0.03	S:R214-	20	0.03
Maharashtra	ORF1a:E2619-	1	0.03	ORF1b:G2201-	1	0.07	ORF1a:S2242-	2	0.03	S:D215-	20	0.03
Maharashtra	ORF1a:S2631-	1	0.03	ORF1b:L2202-	1	0.07	ORF1a:L2243-	2	0.03	S:L216-	20	0.03
Maharashtra	ORF1a:T2632-	1	0.03	ORF1b:Q2203-	1	0.07	ORF1a:I2244-	2	0.03	S:F201-	20	0.03
Maharashtra	ORF1a:F2633-	1	0.03	ORF1b:P2204-	1	0.07	ORF1a:Y2245-	2	0.03	S:K202-	20	0.03
Maharashtra	ORF1a:I2634-	1	0.03	ORF1b:S2205-	1	0.07	ORF1a:S2246-	2	0.03	S:S205-	20	0.03
Maharashtra	ORF1a:S2635-	1	0.03	ORF1b:V2206-	1	0.07	ORF1a:T2247-	2	0.03	S:I210-	20	0.03
Maharashtra	ORF1a:A2636-	1	0.03	ORF1b:G2207-	1	0.07	ORF1a:A2248-	2	0.03	S:N211-	20	0.03
Maharashtra	ORF1a:A2637-	1	0.03	ORF1b:P2208-	1	0.07	ORF1a:A2249-	2	0.03	S:L212-	20	0.03
Maharashtra	ORF1a:R2638-	1	0.03	ORF1b:K2209-	1	0.07	ORF1a:L2250-	2	0.03	ORF1a:V1629-	20	0.03
Maharashtra	ORF1a:Q2639-	1	0.03	ORF1b:Q2210-	1	0.07	ORF1a:G2251-	2	0.03	ORF1a:D1926-	20	0.03
Maharashtra	ORF1a:G2640-	1	0.03	ORF1b:A2211-	1	0.07	ORF1a:V2252-	2	0.03	ORF1a:N1927-	20	0.03
Maharashtra	ORF1a:F2641-	1	0.03	ORF1b:S2212-	1	0.07	ORF1a:L2253-	2	0.03	ORF1a:S2237-	20	0.03
Maharashtra	ORF1a:V2642-	1	0.03	ORF1b:L2213-	1	0.07	ORF1a:M2254-	2	0.03	ORF1a:V2238-	20	0.03
Maharashtra	ORF1a:D2643-	1	0.03	ORF1b:N2214-	1	0.07	ORF1a:S2255-	2	0.03	ORF1a:R2479-	19	0.02
Maharashtra	ORF1a:S2644-	1	0.03	ORF1b:G2215-	1	0.07	ORF1a:N2256-	2	0.03	ORF1a:P2480-	19	0.02
Maharashtra	ORF1a:D2645-	1	0.03	ORF1b:V2216-	1	0.07	ORF1a:L2257-	2	0.03	ORF1a:N2482-	19	0.02
Maharashtra	ORF1a:V2646-	1	0.03	ORF1b:T2217-	1	0.07	ORF1a:G2258-	2	0.03	ORF1a:P2483-	19	0.02
Maharashtra	ORF1a:E2647-	1	0.03	ORF1b:L2218-	1	0.07	ORF1a:M2259-	2	0.03	ORF1a:E2550-	19	0.02
Maharashtra	ORF1a:T2648-	1	0.03	ORF1b:I2219-	1	0.07	ORF1a:P2260-	2	0.03	ORF1a:E2551-	19	0.02
Maharashtra	ORF1a:K2649-	1	0.03	ORF1b:G2220-	1	0.07	ORF1a:S2261-	2	0.03	ORF1a:S2552-	19	0.02
Maharashtra	ORF1a:D2650-	1	0.03	ORF1b:E2221-	1	0.07	ORF1a:Y2262-	2	0.03	ORF1a:S2553-	19	0.02
Maharashtra	ORF1a:V2651-	1	0.03	ORF1b:A2222-	1	0.07	ORF1a:C2263-	2	0.03	ORF1a:A2554-	19	0.02
Maharashtra	ORF1a:V2652-	1	0.03	ORF1b:V2223-	1	0.07	ORF1a:T2264-	2	0.03	ORF1a:K2830-	19	0.02
Maharashtra	ORF1a:E2653-	1	0.03	ORF1b:K2224-	1	0.07	ORF1a:G2265-	2	0.03	ORF1a:H2831-	19	0.02
Maharashtra	ORF1a:C2654-	1	0.03	ORF1b:T2225-	1	0.07	ORF1b:R2684-	2	0.03	ORF1a:A2832-	19	0.02
Maharashtra	ORF1a:S2658-	1	0.03	ORF1b:Q2226-	1	0.07	ORF1b:L1966-	2	0.03	ORF1a:Q2840-	19	0.02
Maharashtra	ORF1a:H2659-	1	0.03	ORF1b:F2227-	1	0.07	ORF1b:P1967-	2	0.03	ORF1a:E2861-	19	0.02
Maharashtra	ORF1a:Q2660-	1	0.03	ORF1b:N2228-	1	0.07	ORF1b:F1968-	2	0.03	ORF1a:V2862-	19	0.02
Maharashtra	ORF1a:S2661-	1	0.03	ORF1b:Y2229-	1	0.07	ORF1b:F1969-	2	0.03	ORF1a:R2949-	19	0.02
Maharashtra	ORF1a:D2662-	1	0.03	ORF1b:Y2230-	1	0.07	ORF1b:Y1970-	2	0.03	ORF1a:A3061-	19	0.02
Maharashtra	ORF1a:I2663-	1	0.03	ORF1b:K2231-	1	0.07	ORF1b:Y1971-	2	0.03	ORF1a:Y3062-	19	0.02
Maharashtra	ORF1a:E2664-	1	0.03	ORF1b:K2232-	1	0.07	ORF1b:S1972-	2	0.03	ORF1a:Y3063-	19	0.02
Maharashtra	ORF1a:V2665-	1	0.03	ORF1b:V2233-	1	0.07	ORF1b:D1973-	2	0.03	ORF1a:I3146-	19	0.02
Maharashtra	ORF1a:T2666-	1	0.03	ORF1b:D2234-	1	0.07	ORF1b:H1979-	2	0.03	ORF1a:T3150-	19	0.02
Maharashtra	ORF1a:G2667-	1	0.03	ORF1b:G2235-	1	0.07	M:M1-	2	0.03	ORF1a:F3153-	19	0.02
Maharashtra	ORF1a:D2668-	1	0.03	ORF1b:V2236-	1	0.07	M:A2-	2	0.03	ORF1a:Y3154-	19	0.02
Maharashtra	ORF1a:S2669-	1	0.03	ORF1b:L2251-	1	0.07	M:D3-	2	0.03	ORF1a:W3155-	19	0.02
Maharashtra	ORF1a:C2670-	1	0.03	ORF1b:Q2252-	1	0.07	M:S4-	2	0.03	ORF1a:S3171-	19	0.02
Maharashtra	ORF1a:N2671-	1	0.03	ORF1b:E2253-	1	0.07	M:N5-	2	0.03	ORF1a:F3172-	19	0.02
Maharashtra	ORF1a:N2672-	1	0.03	ORF1b:F2254-	1	0.07	M:G6-	2	0.03	ORF1a:I3341-	19	0.02
Maharashtra	ORF1a:Y2673-	1	0.03	ORF1b:K2255-	1	0.07	M:T7-	2	0.03	ORF1a:S3402-	19	0.02
Maharashtra	ORF1a:M2674-	1	0.03	ORF1b:P2256-	1	0.07	M:I8-	2	0.03	ORF1a:L3534-	19	0.02
Maharashtra	ORF1a:Y2677-	1	0.03	ORF1b:R2257-	1	0.07	M:T9-	2	0.03	ORF1a:L3535-	19	0.02
Maharashtra	ORF1a:N2678-	1	0.03	ORF1b:S2258-	1	0.07	M:V10-	2	0.03	ORF1a:Q3536-	19	0.02
Maharashtra	ORF1a:K2679-	1	0.03	ORF1b:Q2259-	1	0.07	M:E11-	2	0.03	ORF1a:N3537-	19	0.02
Maharashtra	ORF1a:V2680-	1	0.03	ORF1b:M2260-	1	0.07	M:E12-	2	0.03	ORF1a:G3538-	19	0.02
Maharashtra	ORF1a:E2681-	1	0.03	ORF1b:E2261-	1	0.07	M:L13-	2	0.03	ORF1a:M3539-	19	0.02
Maharashtra	ORF1a:N2682-	1	0.03	ORF1b:I2262-	1	0.07	ORF1a:V2276-	2	0.03	ORF1a:N3540-	19	0.02
Maharashtra	ORF1a:M2683-	1	0.03	ORF1b:D2263-	1	0.07	ORF1a:T2277-	2	0.03	ORF1a:L3545-	19	0.02
Maharashtra	ORF1a:T2684-	1	0.03	ORF1b:F2264-	1	0.07	ORF1a:S546-	2	0.03	ORF1a:G3546-	19	0.02
Maharashtra	ORF1a:P2685-	1	0.03	ORF1b:L2265-	1	0.07	ORF1a:I547-	2	0.03	ORF1a:S3547-	19	0.02
Maharashtra	ORF1a:R2686-	1	0.03	ORF1b:E2266-	1	0.07	ORF1a:V596-	2	0.03	ORF1a:E3551-	19	0.02

Maharashtra	ORF1a:D2687-	1	0.03	ORF1b:L2267-	1	0.07	ORF1a:V597-	2	0.03	ORF1a:D3552-	19	0.02
Maharashtra	ORF1a:L2688-	1	0.03	ORF1b:A2268-	1	0.07	ORF1a:A803-	2	0.03	ORF1a:S3075-	19	0.02
Maharashtra	ORF1a:G2689-	1	0.03	ORF1b:M2269-	1	0.07	ORF1a:P804-	2	0.03	ORF1a:M3129-	19	0.02
Maharashtra	ORF1a:A2690-	1	0.03	ORF1b:D2270-	1	0.07	ORF1a:N805-	2	0.03	ORF1a:Y3160-	19	0.02
Maharashtra	ORF1a:C2691-	1	0.03	ORF1b:E2271-	1	0.07	ORF1a:M806-	2	0.03	ORF1a:D3196-	19	0.02
Maharashtra	ORF1a:I2692-	1	0.03	ORF1b:F2272-	1	0.07	ORF1a:M807-	2	0.03	ORF1a:V3197-	19	0.02
Maharashtra	ORF1a:D2693-	1	0.03	ORF1b:I2273-	1	0.07	ORF1a:Q834-	2	0.03	ORF1a:L3198-	19	0.02
Maharashtra	ORF1a:C2694-	1	0.03	ORF1b:E2274-	1	0.07	ORF1a:T951-	2	0.03	ORF1a:L3199-	19	0.02
Maharashtra	ORF1a:S2695-	1	0.03	ORF1b:R2275-	1	0.07	ORF1a:E952-	2	0.03	ORF1a:P3200-	19	0.02
Maharashtra	ORF1a:A2696-	1	0.03	ORF1b:Y2276-	1	0.07	ORF1a:D953-	2	0.03	ORF1a:L3201-	19	0.02
Maharashtra	ORF1a:R2697-	1	0.03	ORF1b:K2277-	1	0.07	ORF1a:D954-	2	0.03	ORF1a:E1251-	19	0.02
Maharashtra	ORF1a:H2698-	1	0.03	ORF1b:L2278-	1	0.07	ORF1a:Y955-	2	0.03	ORF1a:N1252-	19	0.02
Maharashtra	ORF1a:I2699-	1	0.03	ORF1b:E2279-	1	0.07	ORF1a:Q956-	2	0.03	ORF1a:N1260-	19	0.02
Maharashtra	ORF1a:N2700-	1	0.03	ORF1b:G2298-	1	0.07	ORF1a:G957-	2	0.03	ORF1a:T1303-	19	0.02
Maharashtra	ORF1a:A2701-	1	0.03	ORF1b:L2299-	1	0.07	ORF1a:K958-	2	0.03	ORF1a:K1304-	19	0.02
Maharashtra	ORF1a:S2706-	1	0.03	ORF1b:H2300-	1	0.07	ORF1a:P959-	2	0.03	ORF1a:K1305-	19	0.02
Maharashtra	ORF1a:H2707-	1	0.03	ORF1b:L2301-	1	0.07	ORF1a:G963-	2	0.03	ORF1a:L1334-	19	0.02
Maharashtra	ORF1a:N2708-	1	0.03	ORF1b:L2302-	1	0.07	ORF1a:A964-	2	0.03	ORF1a:N1335-	19	0.02
Maharashtra	ORF1a:I2709-	1	0.03	ORF1b:I2303-	1	0.07	ORF1a:T965-	2	0.03	ORF1a:G1336-	19	0.02
Maharashtra	ORF1a:A2710-	1	0.03	ORF1b:G2304-	1	0.07	ORF1a:S966-	2	0.03	ORF1a:T1491-	19	0.02
Maharashtra	ORF1a:L2711-	1	0.03	ORF1b:L2305-	1	0.07	ORF1a:T987-	2	0.03	ORF1a:H1500-	19	0.02
Maharashtra	ORF1a:I2712-	1	0.03	ORF1b:A2306-	1	0.07	ORF1a:T1017-	2	0.03	ORF1a:H1545-	19	0.02
Maharashtra	ORF1a:W2713-	1	0.03	ORF1b:K2307-	1	0.07	ORF1a:G1069-	2	0.03	ORF1a:G1548-	19	0.02
Maharashtra	ORF1a:N2714-	1	0.03	ORF1b:R2308-	1	0.07	ORF1a:G1070-	2	0.03	ORF1a:F1571-	19	0.02
Maharashtra	ORF1a:V2715-	1	0.03	ORF1b:F2309-	1	0.07	ORF1a:V1071-	2	0.03	ORF1a:M1588-	19	0.02
Maharashtra	ORF1a:K2716-	1	0.03	ORF1b:K2310-	1	0.07	ORF1a:A1072-	2	0.03	ORF1a:T1589-	19	0.02
Maharashtra	ORF1a:D2717-	1	0.03	ORF1b:E2311-	1	0.07	ORF1a:G1073-	2	0.03	ORF1a:S2378-	19	0.02
Maharashtra	ORF1a:F2718-	1	0.03	ORF1b:S2312-	1	0.07	ORF1a:A1074-	2	0.03	ORF1a:Y2513-	19	0.02
Maharashtra	ORF1a:M2719-	1	0.03	ORF1b:P2313-	1	0.07	ORF1a:S1102-	2	0.03	ORF1a:E2514-	19	0.02
Maharashtra	ORF1a:S2720-	1	0.03	ORF1b:F2314-	1	0.07	ORF1a:C1103-	2	0.03	ORF1a:T2532-	19	0.02
Maharashtra	ORF1a:L2721-	1	0.03	ORF1b:E2315-	1	0.07	ORF1a:V1104-	2	0.03	ORF1a:K2533-	19	0.02
Maharashtra	ORF1a:S2722-	1	0.03	ORF1b:L2316-	1	0.07	ORF1b:T1455-	2	0.03	ORF1a:P2792-	19	0.02
Maharashtra	ORF1a:E2723-	1	0.03	ORF1b:E2317-	1	0.07	ORF1b:V1456-	2	0.03	ORF1a:V2793-	19	0.02
Maharashtra	ORF1a:Q2724-	1	0.03	ORF1b:D2318-	1	0.07	ORF1b:D1457-	2	0.03	ORF1a:H2794-	19	0.02
Maharashtra	ORF1a:L2725-	1	0.03	ORF1b:F2319-	1	0.07	ORF1b:S1458-	2	0.03	ORF1a:V2795-	19	0.02
Maharashtra	ORF1a:R2726-	1	0.03	ORF1b:I2320-	1	0.07	ORF1b:S1459-	2	0.03	ORF1a:M3030-	19	0.02
Maharashtra	ORF1a:K2727-	1	0.03	ORF1b:P2321-	1	0.07	ORF1b:Q1460-	2	0.03	ORF1a:F3031-	19	0.02
Maharashtra	ORF1a:Q2728-	1	0.03	ORF1b:M2322-	1	0.07	ORF1b:G1461-	2	0.03	ORF1a:T3032-	19	0.02
Maharashtra	ORF1a:I2729-	1	0.03	ORF1b:D2323-	1	0.07	ORF1b:S1462-	2	0.03	ORF1a:P3033-	19	0.02
Maharashtra	ORF1a:T2755-	1	0.03	ORF1b:S2324-	1	0.07	ORF1b:N1482-	2	0.03	ORF1a:N3396-	19	0.02
Maharashtra	ORF1a:T2756-	1	0.03	ORF1b:T2325-	1	0.07	ORF1b:R1483-	2	0.03	S:N481-	19	0.02
Maharashtra	ORF1a:K2757-	1	0.03	ORF1b:V2326-	1	0.07	ORF1b:F1484-	2	0.03	S:G482-	19	0.02
Maharashtra	ORF1a:I2758-	1	0.03	ORF1b:K2327-	1	0.07	ORF1b:A1525-	2	0.03	S:V483-	19	0.02
Maharashtra	ORF1a:A2759-	1	0.03	ORF1b:N2328-	1	0.07	ORF1b:E1526-	2	0.03	S:C136-	19	0.02
Maharashtra	ORF1a:L2760-	1	0.03	ORF1b:Y2329-	1	0.07	ORF1b:N1527-	2	0.03	S:N137-	19	0.02
Maharashtra	ORF1a:K2761-	1	0.03	ORF1b:F2330-	1	0.07	ORF1b:V1528-	2	0.03	S:D138-	19	0.02
Maharashtra	ORF1a:G2762-	1	0.03	ORF1b:I2331-	1	0.07	ORF1b:T1529-	2	0.03	S:P139-	19	0.02
Maharashtra	ORF1a:G2763-	1	0.03	ORF1b:T2332-	1	0.07	ORF1b:G1530-	2	0.03	S:F797-	19	0.02
Maharashtra	ORF1a:K2764-	1	0.03	ORF1b:D2333-	1	0.07	ORF1b:C1563-	2	0.03	S:G798-	19	0.02
Maharashtra	ORF1a:I2765-	1	0.03	ORF1b:A2334-	1	0.07	ORF1b:V1564-	2	0.03	S:G799-	19	0.02
Maharashtra	ORF1a:V2766-	1	0.03	ORF1b:Q2335-	1	0.07	ORF1b:D1565-	2	0.03	S:F800-	19	0.02
Maharashtra	ORF1a:N2767-	1	0.03	ORF1b:T2336-	1	0.07	ORF1b:I1566-	2	0.03	S:I203-	19	0.02
Maharashtra	ORF1a:N2768-	1	0.03	ORF1b:G2337-	1	0.07	ORF1b:P1567-	2	0.03	S:Y204-	19	0.02
Maharashtra	ORF1a:W2769-	1	0.03	ORF1b:S2338-	1	0.07	ORF1b:G1568-	2	0.03	S:V213-	19	0.02
Maharashtra	ORF1a:L2770-	1	0.03	ORF1b:S2339-	1	0.07	ORF1b:I1569-	2	0.03	ORF1a:Y1329-	19	0.02
Maharashtra	ORF1a:V2783-	1	0.03	ORF1b:K2340-	1	0.07	ORF1b:P1570-	2	0.03	ORF1a:L1627-	19	0.02
Maharashtra	ORF1a:A2784-	1	0.03	ORF1b:C2341-	1	0.07	ORF1b:K1571-	2	0.03	ORF1a:R1628-	19	0.02
Maharashtra	ORF1a:A2785-	1	0.03	ORF1b:V2342-	1	0.07	ORF1b:D1572-	2	0.03	ORF1a:V1750-	19	0.02
Maharashtra	ORF1a:I2786-	1	0.03	ORF1b:C2343-	1	0.07	ORF1b:M1573-	2	0.03	ORF1a:V1751-	19	0.02
Maharashtra	ORF1a:F2787-	1	0.03	ORF1b:S2344-	1	0.07	ORF1b:E1601-	2	0.03	ORF1a:C1752-	19	0.02
Maharashtra	ORF1a:Y2788-	1	0.03	ORF1b:V2345-	1	0.07	ORF1b:E1602-	2	0.03	ORF1a:K1753-	19	0.02
Maharashtra	ORF1a:L2789-	1	0.03	ORF1b:I2346-	1	0.07	ORF1b:I1604-	2	0.03	ORF1a:T1754-	19	0.02
Maharashtra	ORF1a:I2790-	1	0.03	ORF1b:D2347-	1	0.07	ORF1b:R1605-	2	0.03	ORF1a:G1764-	19	0.02
Maharashtra	ORF1a:T2791-	1	0.03	ORF1b:L2348-	1	0.07	ORF1b:H1606-	2	0.03	ORF1a:V1765-	19	0.02
Maharashtra	ORF1a:P2792-	1	0.03	ORF1b:L2349-	1	0.07	ORF1b:V1607-	2	0.03	ORF1a:E1766-	19	0.02
Maharashtra	ORF1a:V2793-	1	0.03	ORF1b:L2350-	1	0.07	ORF1b:R1608-	2	0.03	ORF1a:D1913-	19	0.02
Maharashtra	ORF1a:H2794-	1	0.03	ORF1b:D2351-	1	0.07	ORF1b:A1643-	2	0.03	ORF1a:L1914-	19	0.02
Maharashtra	ORF1a:V2795-	1	0.03	ORF1b:D2352-	1	0.07	ORF1b:T1655-	2	0.03	ORF1a:R1953-	19	0.02
Maharashtra	ORF1a:F2802-	1	0.03	ORF1b:F2353-	1	0.07	ORF1b:D1656-	2	0.03	ORF1a:T2007-	19	0.02
Maharashtra	ORF1a:S2803-	1	0.03	ORF1b:V2354-	1	0.07	ORF1b:F1657-	2	0.03	ORF1a:W2008-	19	0.02

Maharashtra	ORF1a:S2804-	1	0.03	ORF1b:E2355-	1	0.07	ORF1b:S1658-	2	0.03	ORF1a:L2323-	19	0.02
Maharashtra	ORF1a:E2805-	1	0.03	ORF1b:I2356-	1	0.07	ORF1b:R1659-	2	0.03	ORF1a:V2324-	19	0.02
Maharashtra	ORF1a:I2806-	1	0.03	ORF1b:I2357-	1	0.07	ORF1b:V1660-	2	0.03	ORF1a:A2325-	19	0.02
Maharashtra	ORF1a:I2807-	1	0.03	ORF1b:K2358-	1	0.07	ORF1b:S1661-	2	0.03	ORF1a:E2326-	19	0.02
Maharashtra	ORF1a:G2808-	1	0.03	ORF1b:S2359-	1	0.07	ORF1b:A1662-	2	0.03	ORF1a:T2124-	19	0.02
Maharashtra	ORF1a:Y2809-	1	0.03	ORF1b:Q2360-	1	0.07	ORF1b:K1663-	2	0.03	ORF1a:H2125-	19	0.02
Maharashtra	ORF1a:K2810-	1	0.03	ORF1b:D2361-	1	0.07	ORF1b:P1664-	2	0.03	ORF1a:G2126-	19	0.02
Maharashtra	ORF1a:A2811-	1	0.03	ORF1b:L2362-	1	0.07	ORF1b:P1665-	2	0.03	ORF3a:I35-	19	0.02
Maharashtra	ORF1a:I2812-	1	0.03	ORF1b:S2363-	1	0.07	ORF1b:P1666-	2	0.03	ORF3a:P36-	19	0.02
Maharashtra	ORF1a:D2813-	1	0.03	ORF1b:V2364-	1	0.07	ORF1b:G1667-	2	0.03	ORF3a:I37-	19	0.02
Maharashtra	ORF1a:G2814-	1	0.03	ORF1b:V2365-	1	0.07	ORF1b:D1668-	2	0.03	ORF3a:Q38-	19	0.02
Maharashtra	ORF1a:G2815-	1	0.03	ORF1b:S2366-	1	0.07	ORF1b:Q1669-	2	0.03	ORF3a:A39-	19	0.02
Maharashtra	ORF1a:V2816-	1	0.03	ORF1b:K2367-	1	0.07	ORF1b:F1670-	2	0.03	ORF1a:S2389-	18	0.02
Maharashtra	ORF1a:A2821-	1	0.03	ORF1b:V2368-	1	0.07	ORF1b:K1671-	2	0.03	ORF1a:F2390-	18	0.02
Maharashtra	ORF1a:S2822-	1	0.03	ORF1b:V2369-	1	0.07	ORF1b:I1674-	2	0.03	ORF1a:Y2391-	18	0.02
Maharashtra	ORF1a:T2823-	1	0.03	ORF1b:K2370-	1	0.07	ORF1b:P1675-	2	0.03	ORF1a:Y2392-	18	0.02
Maharashtra	ORF1a:D2824-	1	0.03	ORF1b:V2371-	1	0.07	ORF1b:L1676-	2	0.03	ORF1a:C2459-	18	0.02
Maharashtra	ORF1a:T2825-	1	0.03	ORF1b:T2372-	1	0.07	ORF1b:M1677-	2	0.03	ORF1a:Y2560-	18	0.02
Maharashtra	ORF1a:C2826-	1	0.03	ORF1b:I2373-	1	0.07	ORF1b:Y1678-	2	0.03	ORF1a:Y2561-	18	0.02
Maharashtra	ORF1a:F2827-	1	0.03	ORF1b:D2374-	1	0.07	ORF1b:K1679-	2	0.03	ORF1a:S2562-	18	0.02
Maharashtra	ORF1a:A2828-	1	0.03	ORF1b:Y2375-	1	0.07	ORF1b:Y1721-	2	0.03	ORF1a:A2584-	18	0.02
Maharashtra	ORF1a:N2829-	1	0.03	ORF1b:T2376-	1	0.07	ORF1b:F1722-	2	0.03	ORF1a:E2585-	18	0.02
Maharashtra	ORF1a:K2830-	1	0.03	ORF1b:E2377-	1	0.07	ORF1b:V1723-	2	0.03	ORF1a:V2665-	18	0.02
Maharashtra	ORF1a:H2831-	1	0.03	ORF1b:I2378-	1	0.07	ORF1b:C1731-	2	0.03	ORF1a:C2670-	18	0.02
Maharashtra	ORF1a:A2832-	1	0.03	ORF1b:S2379-	1	0.07	ORF1b:C1732-	2	0.03	ORF1a:N2671-	18	0.02
Maharashtra	ORF1a:D2833-	1	0.03	ORF1b:F2380-	1	0.07	ORF1b:L1733-	2	0.03	ORF1a:N2672-	18	0.02
Maharashtra	ORF1a:F2834-	1	0.03	ORF1b:M2381-	1	0.07	ORF1b:C1740-	2	0.03	ORF1a:Y2673-	18	0.02
Maharashtra	ORF1a:D2835-	1	0.03	ORF1b:L2382-	1	0.07	ORF1b:F1741-	2	0.03	ORF1a:S3173-	18	0.02
Maharashtra	ORF1a:T2836-	1	0.03	ORF1b:W2383-	1	0.07	ORF1b:S1742-	2	0.03	ORF1a:T3174-	18	0.02
Maharashtra	ORF1a:W2837-	1	0.03	ORF1b:C2384-	1	0.07	ORF1b:T1743-	2	0.03	ORF1a:L3404-	18	0.02
Maharashtra	ORF1a:F2838-	1	0.03	ORF1b:K2385-	1	0.07	ORF1b:A1744-	2	0.03	ORF1a:F3554-	18	0.02
Maharashtra	ORF1a:S2839-	1	0.03	ORF1b:D2386-	1	0.07	ORF1b:S1745-	2	0.03	ORF1a:M3655-	18	0.02
Maharashtra	ORF1a:Q2840-	1	0.03	ORF1b:G2387-	1	0.07	ORF1b:F1764-	2	0.03	ORF1a:P3656-	18	0.02
Maharashtra	ORF1a:R2841-	1	0.03	ORF1b:H2388-	1	0.07	ORF1b:M1765-	2	0.03	ORF1a:P3096-	18	0.02
Maharashtra	ORF1a:G2842-	1	0.03	ORF1b:V2389-	1	0.07	ORF1b:G1772-	2	0.03	ORF1a:V3097-	18	0.02
Maharashtra	ORF1a:G2843-	1	0.03	ORF1b:E2390-	1	0.07	ORF1b:F1773-	2	0.03	ORF1a:Y3098-	18	0.02
Maharashtra	ORF1a:S2844-	1	0.03	ORF1b:T2391-	1	0.07	ORF1b:T1774-	2	0.03	ORF1a:S3099-	18	0.02
Maharashtra	ORF1a:I2854-	1	0.03	ORF1b:F2392-	1	0.07	ORF1b:N1780-	2	0.03	ORF1a:F3100-	18	0.02
Maharashtra	ORF1a:A2855-	1	0.03	ORF1b:Y2393-	1	0.07	ORF1b:H1781-	2	0.03	ORF1a:L3101-	18	0.02
Maharashtra	ORF1a:A2856-	1	0.03	ORF1b:P2394-	1	0.07	ORF1b:D1782-	2	0.03	ORF1a:P3102-	18	0.02
Maharashtra	ORF1a:V2857-	1	0.03	ORF1b:K2395-	1	0.07	ORF1b:L1783-	2	0.03	ORF1a:G3103-	18	0.02
Maharashtra	ORF1a:I2858-	1	0.03	ORF1b:Q2400-	1	0.07	ORF1b:Y1784-	2	0.03	ORF1a:V3104-	18	0.02
Maharashtra	ORF1a:T2859-	1	0.03	ORF1b:A2401-	1	0.07	ORF1b:C1785-	2	0.03	ORF1a:Y3105-	18	0.02
Maharashtra	ORF1a:R2860-	1	0.03	ORF1b:W2402-	1	0.07	ORF1b:Q1786-	2	0.03	ORF1a:S3106-	18	0.02
Maharashtra	ORF1a:E2861-	1	0.03	ORF1b:Q2403-	1	0.07	ORF1b:V1787-	2	0.03	ORF1a:V3107-	18	0.02
Maharashtra	ORF1a:V2862-	1	0.03	ORF1b:P2404-	1	0.07	ORF1b:H1788-	2	0.03	ORF1a:I3108-	18	0.02
Maharashtra	ORF1a:G2863-	1	0.03	ORF1b:G2405-	1	0.07	ORF1b:G1789-	2	0.03	ORF1a:V3130-	18	0.02
Maharashtra	ORF1a:F2864-	1	0.03	ORF1b:V2406-	1	0.07	ORF1b:N1790-	2	0.03	ORF1a:M3131-	18	0.02
Maharashtra	ORF1a:V2865-	1	0.03	ORF1b:A2407-	1	0.07	ORF1b:A1791-	2	0.03	ORF1a:F3132-	18	0.02
Maharashtra	ORF1a:V2866-	1	0.03	ORF1b:M2408-	1	0.07	ORF1b:H1792-	2	0.03	ORF1a:F3156-	18	0.02
Maharashtra	ORF1a:P2867-	1	0.03	ORF1b:P2409-	1	0.07	ORF1b:V1793-	2	0.03	ORF1a:F3157-	18	0.02
Maharashtra	ORF1a:G2868-	1	0.03	ORF1b:N2410-	1	0.07	ORF1b:A1794-	2	0.03	ORF1a:S3158-	18	0.02
Maharashtra	ORF1a:L2869-	1	0.03	ORF1b:L2411-	1	0.07	ORF1b:S1795-	2	0.03	S:K129-	18	0.02
Maharashtra	ORF1a:P2870-	1	0.03	ORF1b:Y2412-	1	0.07	ORF1b:C1796-	2	0.03	S:V130-	18	0.02
Maharashtra	ORF1a:G2871-	1	0.03	ORF1b:K2413-	1	0.07	ORF1b:K1812-	2	0.03	S:C131-	18	0.02
Maharashtra	ORF1a:T2872-	1	0.03	ORF1b:M2414-	1	0.07	ORF1b:R1813-	2	0.03	S:Q134-	18	0.02
Maharashtra	ORF1a:I2873-	1	0.03	ORF1b:Q2415-	1	0.07	ORF1b:V1814-	2	0.03	S:F135-	18	0.02
Maharashtra	ORF1a:L2874-	1	0.03	ORF1b:R2416-	1	0.07	ORF1b:D1815-	2	0.03	ORF1a:G1119-	18	0.02
Maharashtra	ORF1a:R2875-	1	0.03	ORF1b:M2417-	1	0.07	ORF1b:W1816-	2	0.03	ORF1a:P1120-	18	0.02
Maharashtra	ORF1a:T2876-	1	0.03	ORF1b:L2418-	1	0.07	ORF1b:T1817-	2	0.03	ORF1a:N1121-	18	0.02
Maharashtra	ORF1a:T2877-	1	0.03	ORF1b:L2419-	1	0.07	ORF1b:I1818-	2	0.03	ORF1a:V1122-	18	0.02
Maharashtra	ORF1a:N2878-	1	0.03	ORF1b:E2420-	1	0.07	ORF1b:E1819-	2	0.03	ORF1a:N1123-	18	0.02
Maharashtra	ORF1a:G2879-	1	0.03	ORF1b:K2421-	1	0.07	ORF1b:Y1820-	2	0.03	ORF1a:K1124-	18	0.02
Maharashtra	ORF1a:A2891-	1	0.03	ORF1b:C2422-	1	0.07	ORF1b:V1852-	2	0.03	ORF1a:G1125-	18	0.02
Maharashtra	ORF1a:V2892-	1	0.03	ORF1b:D2423-	1	0.07	ORF1b:D1855-	2	0.03	ORF1a:E1126-	18	0.02
Maharashtra	ORF1a:G2893-	1	0.03	ORF1b:L2433-	1	0.07	ORF1b:I1856-	2	0.03	ORF1a:G1308-	18	0.02
Maharashtra	ORF1a:N2894-	1	0.03	ORF1b:P2434-	1	0.07	ORF1b:E1889-	2	0.03	ORF1a:T1309-	18	0.02
Maharashtra	ORF1a:I2895-	1	0.03	ORF1b:K2435-	1	0.07	ORF1b:G1904-	2	0.03	ORF1a:T1429-	18	0.02
Maharashtra	ORF1a:C2896-	1	0.03	ORF1b:G2436-	1	0.07	ORF1b:V1905-	2	0.03	ORF1a:T1430-	18	0.02
Maharashtra	ORF1a:Y2897-	1	0.03	ORF1b:I2437-	1	0.07	ORF1b:C1906-	2	0.03	ORF1a:V1431-	18	0.02

Maharashtra	ORF1a:T2898-	1	0.03	ORF1b:M2438-	1	0.07	ORF1b:L1907-	2	0.03	ORF1a:A1432-	18	0.02
Maharashtra	ORF1a:P2899-	1	0.03	ORF1b:M2439-	1	0.07	ORF1b:F1908-	2	0.03	ORF1a:S1433-	18	0.02
Maharashtra	ORF1a:S2900-	1	0.03	ORF1b:N2440-	1	0.07	ORF1b:W1909-	2	0.03	ORF1a:Y1452-	18	0.02
Maharashtra	ORF1a:K2901-	1	0.03	ORF1b:V2441-	1	0.07	ORF1b:N1910-	2	0.03	ORF1a:V1453-	18	0.02
Maharashtra	ORF1a:L2902-	1	0.03	ORF1b:A2442-	1	0.07	ORF1b:V1922-	2	0.03	ORF1a:T1454-	18	0.02
Maharashtra	ORF1a:I2903-	1	0.03	ORF1b:K2443-	1	0.07	ORF1b:C1923-	2	0.03	ORF1a:H1455-	18	0.02
Maharashtra	ORF1a:E2904-	1	0.03	ORF1b:Y2444-	1	0.07	ORF1b:R1924-	2	0.03	ORF1a:D1481-	18	0.02
Maharashtra	ORF1a:Y2905-	1	0.03	ORF1b:T2445-	1	0.07	ORF1b:G989-	2	0.03	ORF1a:A1482-	18	0.02
Maharashtra	ORF1a:T2906-	1	0.03	ORF1b:Q2446-	1	0.07	ORF1b:G990-	2	0.03	ORF1a:V1483-	18	0.02
Maharashtra	ORF1a:D2907-	1	0.03	ORF1b:L2447-	1	0.07	ORF1b:M991-	2	0.03	ORF1a:T1484-	18	0.02
Maharashtra	ORF1a:F2908-	1	0.03	ORF1b:C2448-	1	0.07	ORF1b:S992-	2	0.03	ORF1a:A1485-	18	0.02
Maharashtra	ORF1a:F2922-	1	0.03	ORF1b:Q2449-	1	0.07	ORF1b:Y993-	2	0.03	ORF1a:Y1486-	18	0.02
Maharashtra	ORF1a:K2923-	1	0.03	ORF1b:Y2450-	1	0.07	ORF1b:Y994-	2	0.03	ORF1a:N1487-	18	0.02
Maharashtra	ORF1a:D2924-	1	0.03	ORF1b:L2451-	1	0.07	ORF1b:C995-	2	0.03	ORF1a:G1488-	18	0.02
Maharashtra	ORF1a:A2925-	1	0.03	ORF1b:N2452-	1	0.07	ORF1b:K996-	2	0.03	ORF1a:S1561-	18	0.02
Maharashtra	ORF1a:S2926-	1	0.03	ORF1b:T2453-	1	0.07	ORF1b:S997-	2	0.03	ORF1a:L1562-	18	0.02
Maharashtra	ORF1a:G2927-	1	0.03	ORF1b:L2454-	1	0.07	ORF1b:H998-	2	0.03	ORF1a:R1563-	18	0.02
Maharashtra	ORF1a:K2928-	1	0.03	ORF1b:T2455-	1	0.07	ORF1b:K999-	2	0.03	ORF1a:S1587-	18	0.02
Maharashtra	ORF1a:P2929-	1	0.03	ORF1b:L2456-	1	0.07	ORF1b:Y2460-	2	0.03	ORF1a:Y1634-	18	0.02
Maharashtra	ORF1a:V2930-	1	0.03	ORF1b:A2457-	1	0.07	ORF1b:N2461-	2	0.03	ORF1a:Y1635-	18	0.02
Maharashtra	ORF1a:P2931-	1	0.03	ORF1b:V2458-	1	0.07	ORF1b:M2462-	2	0.03	ORF1a:N1898-	18	0.02
Maharashtra	ORF1a:Y2932-	1	0.03	ORF1b:P2459-	1	0.07	ORF1b:R2463-	2	0.03	ORF1a:I2501-	18	0.02
Maharashtra	ORF1a:C2933-	1	0.03	ORF1b:Y2460-	1	0.07	ORF1b:V2464-	2	0.03	ORF1a:H2502-	18	0.02
Maharashtra	ORF1a:Y2934-	1	0.03	ORF1b:N2461-	1	0.07	ORF1b:I2465-	2	0.03	ORF1a:G2534-	18	0.02
Maharashtra	ORF1a:D2935-	1	0.03	ORF1b:M2462-	1	0.07	ORF1b:H2466-	2	0.03	ORF1a:S2535-	18	0.02
Maharashtra	ORF1a:T2936-	1	0.03	ORF1b:R2463-	1	0.07	ORF1b:Q2635-	2	0.03	ORF1a:L2536-	18	0.02
Maharashtra	ORF1a:N2937-	1	0.03	ORF1b:V2464-	1	0.07	ORF1b:L2636-	2	0.03	ORF1a:L2789-	18	0.02
Maharashtra	ORF1a:V2938-	1	0.03	ORF1b:I2465-	1	0.07	ORF1b:V1375-	2	0.03	ORF1a:I2790-	18	0.02
Maharashtra	ORF1a:L2939-	1	0.03	ORF1b:H2466-	1	0.07	ORF1b:S1376-	2	0.03	ORF1a:T2791-	18	0.02
Maharashtra	ORF1a:E2940-	1	0.03	ORF1b:F2467-	1	0.07	ORF1b:A1377-	2	0.03	S:M153-	18	0.02
Maharashtra	ORF1a:G2941-	1	0.03	ORF1b:G2468-	1	0.07	ORF1b:S1942-	2	0.03	S:E154-	18	0.02
Maharashtra	ORF1a:S2942-	1	0.03	ORF1b:A2469-	1	0.07	S:K835-	2	0.03	S:D843-	18	0.02
Maharashtra	ORF1a:V2943-	1	0.03	ORF1b:G2470-	1	0.07	S:Q836-	2	0.03	S:V143-	18	0.02
Maharashtra	ORF1a:A2944-	1	0.03	ORF1b:S2471-	1	0.07	S:Y837-	2	0.03	S:Y145-	18	0.02
Maharashtra	ORF1a:Y2945-	1	0.03	ORF1b:D2472-	1	0.07	S:G838-	2	0.03	S:K825-	18	0.02
Maharashtra	ORF1a:E2946-	1	0.03	ORF1b:K2473-	1	0.07	S:D839-	2	0.03	S:K921-	18	0.02
Maharashtra	ORF1a:D2958-	1	0.03	ORF1b:G2489-	1	0.07	S:C840-	2	0.03	S:A924-	18	0.02
Maharashtra	ORF1a:G2959-	1	0.03	ORF1b:T2490-	1	0.07	S:L841-	2	0.03	S:N925-	18	0.02
Maharashtra	ORF1a:S2960-	1	0.03	ORF1b:L2491-	1	0.07	S:G842-	2	0.03	ORF1a:L1523-	18	0.02
Maharashtra	ORF1a:I2961-	1	0.03	ORF1b:L2492-	1	0.07	S:T881-	2	0.03	ORF1a:L2062-	18	0.02
Maharashtra	ORF1a:I2962-	1	0.03	ORF1b:V2493-	1	0.07	S:I882-	2	0.03	ORF1a:E2063-	18	0.02
Maharashtra	ORF1a:Q2963-	1	0.03	ORF1b:D2494-	1	0.07	S:T883-	2	0.03	ORF1a:I2138-	18	0.02
Maharashtra	ORF1a:F2964-	1	0.03	ORF1b:S2495-	1	0.07	S:S884-	2	0.03	ORF1a:A2139-	18	0.02
Maharashtra	ORF1a:P2965-	1	0.03	ORF1b:D2496-	1	0.07	S:G885-	2	0.03	ORF1a:N2140-	18	0.02
Maharashtra	ORF1a:N2966-	1	0.03	ORF1b:L2497-	1	0.07	S:W886-	2	0.03	ORF1a:Y2141-	18	0.02
Maharashtra	ORF1a:T2967-	1	0.03	ORF1b:N2498-	1	0.07	S:T887-	2	0.03	ORF1a:A2142-	18	0.02
Maharashtra	ORF1a:Y2968-	1	0.03	ORF1b:D2499-	1	0.07	S:F888-	2	0.03	ORF1a:K2143-	18	0.02
Maharashtra	ORF1a:E2970-	1	0.03	ORF1b:F2500-	1	0.07	S:G889-	2	0.03	ORF1a:N2162-	18	0.02
Maharashtra	ORF1a:G2971-	1	0.03	ORF1b:V2501-	1	0.07	S:A890-	2	0.03	ORF1a:R2163-	18	0.02
Maharashtra	ORF1a:S2972-	1	0.03	ORF1b:S2502-	1	0.07	S:G891-	2	0.03	ORF1a:V2164-	18	0.02
Maharashtra	ORF1a:V2973-	1	0.03	ORF1b:D2503-	1	0.07	S:A892-	2	0.03	ORF1a:C2165-	18	0.02
Maharashtra	ORF1a:R2974-	1	0.03	ORF1b:A2504-	1	0.07	S:A893-	2	0.03	ORF1a:T2166-	18	0.02
Maharashtra	ORF1a:V2975-	1	0.03	ORF1b:D2505-	1	0.07	S:Q895-	2	0.03	ORF1a:N2167-	18	0.02
Maharashtra	ORF1a:V2976-	1	0.03	ORF1b:S2506-	1	0.07	S:I896-	2	0.03	ORF1a:Y2168-	18	0.02
Maharashtra	ORF1a:T2977-	1	0.03	ORF1b:T2507-	1	0.07	S:F898-	2	0.03	ORF1a:M2169-	18	0.02
Maharashtra	ORF1a:T2978-	1	0.03	ORF1b:L2508-	1	0.07	S:A899-	2	0.03	ORF1a:P2170-	18	0.02
Maharashtra	ORF1a:F2979-	1	0.03	ORF1b:I2509-	1	0.07	S:M900-	2	0.03	ORF1a:M2254-	18	0.02
Maharashtra	ORF1a:D2980-	1	0.03	ORF1b:G2510-	1	0.07	S:Q901-	2	0.03	ORF1a:G2258-	18	0.02
Maharashtra	ORF1a:S2981-	1	0.03	ORF1b:D2511-	1	0.07	S:M902-	2	0.03	ORF1a:M2259-	18	0.02
Maharashtra	ORF1a:E2982-	1	0.03	ORF1b:C2512-	1	0.07	S:A903-	2	0.03	ORF1a:P2260-	18	0.02
Maharashtra	ORF1a:Y2983-	1	0.03	ORF1b:A2513-	1	0.07	S:Y904-	2	0.03	ORF1a:S2261-	18	0.02
Maharashtra	ORF1a:C2984-	1	0.03	ORF1b:T2514-	1	0.07	S:R905-	2	0.03	ORF1a:P2287-	18	0.02
Maharashtra	ORF1a:R2985-	1	0.03	ORF1b:V2515-	1	0.07	S:F906-	2	0.03	ORF1a:C2288-	18	0.02
Maharashtra	ORF1a:H2986-	1	0.03	ORF1b:H2516-	1	0.07	S:N907-	2	0.03	ORF1a:Q2308-	18	0.02
Maharashtra	ORF1a:G2987-	1	0.03	ORF1b:T2517-	1	0.07	S:G908-	2	0.03	ORF1a:I2309-	18	0.02
Maharashtra	ORF1a:T2988-	1	0.03	ORF1b:A2518-	1	0.07	S:Q920-	2	0.03	ORF1a:D2317-	18	0.02
Maharashtra	ORF1a:C2989-	1	0.03	ORF1b:N2519-	1	0.07	S:K921-	2	0.03	ORF1a:L2318-	18	0.02
Maharashtra	ORF1a:E2990-	1	0.03	ORF1b:K2520-	1	0.07	S:A924-	2	0.03	ORF1a:T2319-	18	0.02
Maharashtra	ORF1a:R2991-	1	0.03	ORF1b:W2521-	1	0.07	S:N925-	2	0.03	ORF1a:A2320-	18	0.02
Maharashtra	ORF1a:S2999-	1	0.03	ORF1b:D2522-	1	0.07	S:Q926-	2	0.03	ORF1a:C2012-	18	0.02

Maharashtra	ORF1a:T3000-	1	0.03	ORF1b:L2523-	1	0.07	S:F927-	2	0.03	ORF1a:L2013-	18	0.02
Maharashtra	ORF1a:S3001-	1	0.03	ORF1b:I2524-	1	0.07	S:N928-	2	0.03	ORF1a:W2014-	18	0.02
Maharashtra	ORF1a:G3002-	1	0.03	ORF1b:I2525-	1	0.07	S:S929-	2	0.03	ORF3a:M1-	18	0.02
Maharashtra	ORF1a:R3003-	1	0.03	ORF1b:S2526-	1	0.07	S:A930-	2	0.03	ORF3a:D2-	18	0.02
Maharashtra	ORF1a:W3004-	1	0.03	ORF1b:D2527-	1	0.07	S:I931-	2	0.03	ORF3a:L3-	18	0.02
Maharashtra	ORF1a:V3005-	1	0.03	ORF1b:M2528-	1	0.07	S:G932-	2	0.03	ORF3a:F4-	18	0.02
Maharashtra	ORF1a:L3006-	1	0.03	ORF1b:Y2529-	1	0.07	S:K933-	2	0.03	ORF3a:V13-	18	0.02
Maharashtra	ORF1a:N3007-	1	0.03	ORF1b:D2530-	1	0.07	S:I934-	2	0.03	ORF3a:T14-	18	0.02
Maharashtra	ORF1a:N3008-	1	0.03	ORF1b:P2531-	1	0.07	S:Q935-	2	0.03	ORF3a:L15-	18	0.02
Maharashtra	ORF1a:D3009-	1	0.03	ORF1b:K2532-	1	0.07	S:D936-	2	0.03	ORF3a:K16-	18	0.02
Maharashtra	ORF1a:Y3010-	1	0.03	ORF1b:T2533-	1	0.07	S:E988-	2	0.03	ORF3a:Q17-	18	0.02
Maharashtra	ORF1a:Y3011-	1	0.03	ORF1b:K2534-	1	0.07	S:A989-	2	0.03	ORF3a:G18-	18	0.02
Maharashtra	ORF1a:R3012-	1	0.03	ORF1b:N2535-	1	0.07	S:E990-	2	0.03	ORF3a:E19-	18	0.02
Maharashtra	ORF1a:T3028-	1	0.03	ORF1b:V2536-	1	0.07	S:V991-	2	0.03	ORF1a:A2438-	17	0.02
Maharashtra	ORF1a:N3029-	1	0.03	ORF1b:E2539-	1	0.07	S:Q992-	2	0.03	ORF1a:N2439-	17	0.02
Maharashtra	ORF1a:M3030-	1	0.03	ORF1b:N2540-	1	0.07	S:I993-	2	0.03	ORF1a:G2440-	17	0.02
Maharashtra	ORF1a:F3031-	1	0.03	ORF1b:D2541-	1	0.07	S:D994-	2	0.03	ORF1a:D2492-	17	0.02
Maharashtra	ORF1a:T3032-	1	0.03	ORF1b:S2542-	1	0.07	S:R995-	2	0.03	ORF1a:S2493-	17	0.02
Maharashtra	ORF1a:P3033-	1	0.03	ORF1b:K2543-	1	0.07	S:T998-	2	0.03	ORF1a:V2494-	17	0.02
Maharashtra	ORF1a:L3034-	1	0.03	ORF1b:E2544-	1	0.07	S:Q1005-	2	0.03	ORF1a:S2658-	17	0.02
Maharashtra	ORF1a:I3035-	1	0.03	ORF1b:G2545-	1	0.07	S:T1006-	2	0.03	ORF1a:H2659-	17	0.02
Maharashtra	ORF1a:Q3036-	1	0.03	ORF1b:F2546-	1	0.07	S:L1012-	2	0.03	ORF1a:Q2660-	17	0.02
Maharashtra	ORF1a:P3037-	1	0.03	ORF1b:F2547-	1	0.07	S:I1013-	2	0.03	ORF1a:S2695-	17	0.02
Maharashtra	ORF1a:I3038-	1	0.03	ORF1b:T2548-	1	0.07	S:R1014-	2	0.03	ORF1a:A2696-	17	0.02
Maharashtra	ORF1a:G3039-	1	0.03	ORF1b:Y2549-	1	0.07	S:A1015-	2	0.03	ORF1a:R2697-	17	0.02
Maharashtra	ORF1a:A3040-	1	0.03	ORF1b:I2550-	1	0.07	S:A1016-	2	0.03	ORF1a:H2698-	17	0.02
Maharashtra	ORF1a:L3041-	1	0.03	ORF1b:C2551-	1	0.07	S:L1034-	2	0.03	ORF1a:I2699-	17	0.02
Maharashtra	ORF1a:D3042-	1	0.03	ORF1b:G2552-	1	0.07	S:G1035-	2	0.03	ORF1a:N2700-	17	0.02
Maharashtra	ORF1a:I3043-	1	0.03	ORF1b:F2553-	1	0.07	S:A1056-	2	0.03	ORF1a:R2730-	17	0.02
Maharashtra	ORF1a:S3044-	1	0.03	ORF1b:I2554-	1	0.07	S:P1057-	2	0.03	ORF1a:S2731-	17	0.02
Maharashtra	ORF1a:A3045-	1	0.03	ORF1b:Q2555-	1	0.07	S:H1058-	2	0.03	ORF1a:A2732-	17	0.02
Maharashtra	ORF1a:S3046-	1	0.03	ORF1b:Q2556-	1	0.07	ORF1b:N1039-	2	0.03	ORF1a:A2733-	17	0.02
Maharashtra	ORF1a:I3047-	1	0.03	ORF1b:K2557-	1	0.07	ORF1b:A1040-	2	0.03	ORF1a:K2734-	17	0.02
Maharashtra	ORF1a:V3048-	1	0.03	ORF1b:L2558-	1	0.07	ORF1b:G1041-	2	0.03	ORF1a:K2735-	17	0.02
Maharashtra	ORF1a:A3049-	1	0.03	ORF1b:A2559-	1	0.07	ORF1b:D1042-	2	0.03	ORF1a:N2736-	17	0.02
Maharashtra	ORF1a:G3050-	1	0.03	ORF1b:L2560-	1	0.07	ORF1b:Y1043-	2	0.03	ORF1a:N2737-	17	0.02
Maharashtra	ORF1a:G3051-	1	0.03	ORF1b:G2561-	1	0.07	ORF1b:I1044-	2	0.03	ORF1a:L2738-	17	0.02
Maharashtra	ORF1a:I3052-	1	0.03	ORF1b:G2562-	1	0.07	ORF1b:L1045-	2	0.03	ORF1a:P2739-	17	0.02
Maharashtra	ORF1a:R3066-	1	0.03	ORF1b:S2563-	1	0.07	ORF1b:A1046-	2	0.03	ORF1a:G2762-	17	0.02
Maharashtra	ORF1a:F3067-	1	0.03	ORF1b:V2564-	1	0.07	ORF1b:N1047-	2	0.03	ORF1a:G2763-	17	0.02
Maharashtra	ORF1a:R3068-	1	0.03	ORF1b:A2565-	1	0.07	ORF1b:E1051-	2	0.03	ORF1a:K2764-	17	0.02
Maharashtra	ORF1a:R3069-	1	0.03	ORF1b:I2566-	1	0.07	ORF1b:R1052-	2	0.03	ORF1a:I2765-	17	0.02
Maharashtra	ORF1a:A3070-	1	0.03	ORF1b:K2567-	1	0.07	ORF1b:L1053-	2	0.03	ORF1a:W2837-	17	0.02
Maharashtra	ORF1a:F3071-	1	0.03	ORF1b:I2568-	1	0.07	ORF1b:K1054-	2	0.03	ORF1a:F2838-	17	0.02
Maharashtra	ORF1a:G3072-	1	0.03	ORF1b:T2569-	1	0.07	ORF1b:L1055-	2	0.03	ORF1a:S2839-	17	0.02
Maharashtra	ORF1a:E3073-	1	0.03	S:T20-	1	0.07	ORF1b:F1056-	2	0.03	ORF1a:A3124-	17	0.02
Maharashtra	ORF1a:Y3074-	1	0.03	S:R21-	1	0.07	ORF1b:A1057-	2	0.03	ORF1a:H3125-	17	0.02
Maharashtra	ORF1a:S3075-	1	0.03	S:T22-	1	0.07	ORF1b:A1058-	2	0.03	ORF1a:G3342-	17	0.02
Maharashtra	ORF1a:H3076-	1	0.03	S:Q23-	1	0.07	ORF1b:E1079-	2	0.03	ORF1a:P3362-	17	0.02
Maharashtra	ORF1a:V3077-	1	0.03	S:L24-	1	0.07	ORF1b:V1080-	2	0.03	ORF1a:K3363-	17	0.02
Maharashtra	ORF1a:V3078-	1	0.03	S:P25-	1	0.07	ORF1b:L1081-	2	0.03	ORF1a:Y3364-	17	0.02
Maharashtra	ORF1a:A3079-	1	0.03	S:P26-	1	0.07	ORF1b:S1082-	2	0.03	ORF1a:K3365-	17	0.02
Maharashtra	ORF1a:F3080-	1	0.03	S:A27-	1	0.07	ORF1b:D1083-	2	0.03	ORF1a:S3384-	17	0.02
Maharashtra	ORF1a:N3081-	1	0.03	S:Y28-	1	0.07	ORF1b:R1084-	2	0.03	ORF1a:P3385-	17	0.02
Maharashtra	ORF1a:T3082-	1	0.03	S:R34-	1	0.07	ORF1b:E1085-	2	0.03	ORF1a:N3405-	17	0.02
Maharashtra	ORF1a:L3083-	1	0.03	S:G35-	1	0.07	ORF1b:L1086-	2	0.03	ORF1a:G3406-	17	0.02
Maharashtra	ORF1a:L3084-	1	0.03	S:V36-	1	0.07	ORF1b:H1087-	2	0.03	ORF1a:L3505-	17	0.02
Maharashtra	ORF1a:F3085-	1	0.03	S:Y37-	1	0.07	ORF1b:L1088-	2	0.03	ORF1a:T3506-	17	0.02
Maharashtra	ORF1a:L3086-	1	0.03	S:Y38-	1	0.07	ORF1b:S1089-	2	0.03	ORF1a:E3533-	17	0.02
Maharashtra	ORF1a:M3087-	1	0.03	S:P39-	1	0.07	ORF1b:W1090-	2	0.03	ORF1a:T3555-	17	0.02
Maharashtra	ORF1a:S3088-	1	0.03	S:D40-	1	0.07	ORF7a:R118-	2	0.03	ORF1a:P3556-	17	0.02
Maharashtra	ORF1a:F3089-	1	0.03	S:K41-	1	0.07	ORF7a:K119-	2	0.03	ORF1a:F3557-	17	0.02
Maharashtra	ORF1a:T3090-	1	0.03	S:V42-	1	0.07	ORF7a:T120-	2	0.03	ORF1a:D3558-	17	0.02
Maharashtra	ORF1a:Y3111-	1	0.03	S:F43-	1	0.07	ORF7a:E121-	2	0.03	ORF1a:V3559-	17	0.02
Maharashtra	ORF1a:L3112-	1	0.03	S:R44-	1	0.07	ORF7a:*122-	2	0.03	ORF1a:T3646-	17	0.02
Maharashtra	ORF1a:T3113-	1	0.03	S:S45-	1	0.07	ORF1b:V1945-	1	0.02	ORF1a:A3657-	17	0.02
Maharashtra	ORF1a:F3114-	1	0.03	S:S46-	1	0.07	ORF7b:L14-	1	0.02	ORF1a:D3728-	17	0.02
Maharashtra	ORF1a:Y3115-	1	0.03	S:V47-	1	0.07	ORF8:D119-	1	0.02	ORF1a:Q3729-	17	0.02
Maharashtra	ORF1a:L3116-	1	0.03	S:L48-	1	0.07	ORF8:F120-	1	0.02	S:E132-	17	0.02
Maharashtra	ORF1a:T3117-	1	0.03	S:H49-	1	0.07	S:F86-	1	0.02	S:F133-	17	0.02

Maharashtra	ORF1a:N3118-	1	0.03	S:N61-	1	0.07	S:N87-	1	0.02	ORF1a:S1133-	17	0.02
Maharashtra	ORF1a:D3119-	1	0.03	S:V62-	1	0.07	S:D88-	1	0.02	ORF1a:A1134-	17	0.02
Maharashtra	ORF1a:V3120-	1	0.03	S:T63-	1	0.07	S:G89-	1	0.02	ORF1a:Y1135-	17	0.02
Maharashtra	ORF1a:S3121-	1	0.03	S:W64-	1	0.07	S:V90-	1	0.02	ORF1a:D1289-	17	0.02
Maharashtra	ORF1a:F3122-	1	0.03	S:F65-	1	0.07	S:Y91-	1	0.02	ORF1a:V1290-	17	0.02
Maharashtra	ORF1a:L3123-	1	0.03	S:H66-	1	0.07	S:F92-	1	0.02	ORF1a:T1310-	17	0.02
Maharashtra	ORF1a:A3124-	1	0.03	S:A67-	1	0.07	S:A93-	1	0.02	ORF1a:E1311-	17	0.02
Maharashtra	ORF1a:H3125-	1	0.03	S:I68-	1	0.07	S:S94-	1	0.02	ORF1a:T1344-	17	0.02
Maharashtra	ORF1a:I3126-	1	0.03	S:H69-	1	0.07	S:T95-	1	0.02	ORF1a:V1345-	17	0.02
Maharashtra	ORF1a:Q3127-	1	0.03	S:V70-	1	0.07	S:E96-	1	0.02	ORF1a:L1434-	17	0.02
Maharashtra	ORF1a:W3128-	1	0.03	S:S71-	1	0.07	S:K97-	1	0.02	ORF1a:G1451-	17	0.02
Maharashtra	ORF1a:M3129-	1	0.03	S:G72-	1	0.07	S:S98-	1	0.02	ORF1a:P1480-	17	0.02
Maharashtra	ORF1a:V3136-	1	0.03	S:T73-	1	0.07	S:N99-	1	0.02	ORF1a:I1502-	17	0.02
Maharashtra	ORF1a:P3137-	1	0.03	S:N74-	1	0.07	S:I100-	1	0.02	ORF1a:E1503-	17	0.02
Maharashtra	ORF1a:F3138-	1	0.03	S:G75-	1	0.07	S:I101-	1	0.02	ORF1a:E1526-	17	0.02
Maharashtra	ORF1a:W3139-	1	0.03	S:Y91-	1	0.07	S:R102-	1	0.02	ORF1a:D1639-	17	0.02
Maharashtra	ORF1a:I3140-	1	0.03	S:F92-	1	0.07	S:G103-	1	0.02	ORF1a:Y2266-	17	0.02
Maharashtra	ORF1a:T3141-	1	0.03	S:A93-	1	0.07	S:W104-	1	0.02	ORF1a:R2267-	17	0.02
Maharashtra	ORF1a:I3142-	1	0.03	S:S94-	1	0.07	S:I119-	1	0.02	ORF1a:E2268-	17	0.02
Maharashtra	ORF1a:A3143-	1	0.03	S:T95-	1	0.07	S:V120-	1	0.02	ORF1a:N2416-	17	0.02
Maharashtra	ORF1a:Y3144-	1	0.03	S:E96-	1	0.07	S:N121-	1	0.02	ORF1a:P2537-	17	0.02
Maharashtra	ORF1a:I3145-	1	0.03	S:K97-	1	0.07	S:N122-	1	0.02	ORF1a:T2910-	17	0.02
Maharashtra	ORF1a:I3146-	1	0.03	S:S98-	1	0.07	S:A123-	1	0.02	ORF1a:S2911-	17	0.02
Maharashtra	ORF1a:C3147-	1	0.03	S:N99-	1	0.07	S:T124-	1	0.02	ORF1a:A2912-	17	0.02
Maharashtra	ORF1a:K3151-	1	0.03	S:I100-	1	0.07	S:N125-	1	0.02	ORF1a:C2913-	17	0.02
Maharashtra	ORF1a:H3152-	1	0.03	S:I101-	1	0.07	S:V126-	1	0.02	ORF1a:Y2954-	17	0.02
Maharashtra	ORF1a:F3153-	1	0.03	S:R102-	1	0.07	S:V127-	1	0.02	ORF1a:L3034-	17	0.02
Maharashtra	ORF1a:Y3154-	1	0.03	S:G103-	1	0.07	S:L141-	1	0.02	ORF1a:I3035-	17	0.02
Maharashtra	ORF1a:W3155-	1	0.03	S:W104-	1	0.07	S:V308-	1	0.02	ORF1a:Q3036-	17	0.02
Maharashtra	ORF1a:F3156-	1	0.03	S:Q115-	1	0.07	S:E309-	1	0.02	ORF1a:P3037-	17	0.02
Maharashtra	ORF1a:F3157-	1	0.03	S:S116-	1	0.07	S:K310-	1	0.02	ORF1a:I3038-	17	0.02
Maharashtra	ORF1a:S3158-	1	0.03	S:L117-	1	0.07	S:G311-	1	0.02	ORF1a:G3039-	17	0.02
Maharashtra	ORF1a:N3159-	1	0.03	S:L118-	1	0.07	S:I312-	1	0.02	ORF1a:A3040-	17	0.02
Maharashtra	ORF1a:Y3160-	1	0.03	S:I119-	1	0.07	S:Y313-	1	0.02	ORF1a:L3041-	17	0.02
Maharashtra	ORF1a:L3161-	1	0.03	S:V120-	1	0.07	S:Q314-	1	0.02	S:E191-	17	0.02
Maharashtra	ORF1a:S3171-	1	0.03	S:N121-	1	0.07	S:T315-	1	0.02	S:F192-	17	0.02
Maharashtra	ORF1a:F3172-	1	0.03	S:N122-	1	0.07	S:N331-	1	0.02	S:Y279-	17	0.02
Maharashtra	ORF1a:S3173-	1	0.03	S:A123-	1	0.07	S:I332-	1	0.02	S:N280-	17	0.02
Maharashtra	ORF1a:T3174-	1	0.03	S:T124-	1	0.07	S:T333-	1	0.02	S:S316-	17	0.02
Maharashtra	ORF1a:F3175-	1	0.03	S:N125-	1	0.07	S:N334-	1	0.02	S:N317-	17	0.02
Maharashtra	ORF1a:E3176-	1	0.03	S:V126-	1	0.07	S:L335-	1	0.02	S:F318-	17	0.02
Maharashtra	ORF1a:E3177-	1	0.03	S:V127-	1	0.07	S:C336-	1	0.02	S:R328-	17	0.02
Maharashtra	ORF1a:A3178-	1	0.03	S:F140-	1	0.07	S:A344-	1	0.02	S:F329-	17	0.02
Maharashtra	ORF1a:A3179-	1	0.03	S:Y145-	1	0.07	S:T345-	1	0.02	S:D427-	17	0.02
Maharashtra	ORF1a:L3180-	1	0.03	S:W152-	1	0.07	S:R346-	1	0.02	S:C432-	17	0.02
Maharashtra	ORF1a:C3181-	1	0.03	S:M153-	1	0.07	S:Y365-	1	0.02	S:V433-	17	0.02
Maharashtra	ORF1a:T3182-	1	0.03	S:E154-	1	0.07	S:S366-	1	0.02	S:I434-	17	0.02
Maharashtra	ORF1a:F3183-	1	0.03	S:S155-	1	0.07	S:T376-	1	0.02	S:A435-	17	0.02
Maharashtra	ORF1a:V3467-	1	0.03	S:E156-	1	0.07	S:F377-	1	0.02	S:N487-	17	0.02
Maharashtra	ORF1a:L3468-	1	0.03	S:F157-	1	0.07	S:K378-	1	0.02	S:H146-	17	0.02
Maharashtra	ORF1a:A3469-	1	0.03	S:R158-	1	0.07	S:C379-	1	0.02	S:N148-	17	0.02
Maharashtra	ORF1a:W3470-	1	0.03	S:V159-	1	0.07	S:T385-	1	0.02	S:N149-	17	0.02
Maharashtra	ORF1a:L3471-	1	0.03	S:Y160-	1	0.07	S:K386-	1	0.02	S:N331-	17	0.02
Maharashtra	ORF1a:Y3472-	1	0.03	S:S161-	1	0.07	S:L387-	1	0.02	S:K529-	17	0.02
Maharashtra	ORF1a:A3473-	1	0.03	S:S162-	1	0.07	S:N388-	1	0.02	S:G648-	17	0.02
Maharashtra	ORF1a:A3474-	1	0.03	S:A163-	1	0.07	S:Y396-	1	0.02	S:P665-	17	0.02
Maharashtra	ORF1a:D3492-	1	0.03	S:N164-	1	0.07	S:A397-	1	0.02	S:I666-	17	0.02
Maharashtra	ORF1a:F3493-	1	0.03	S:N165-	1	0.07	S:D398-	1	0.02	S:T866-	17	0.02
Maharashtra	ORF1a:N3494-	1	0.03	S:C166-	1	0.07	S:S399-	1	0.02	S:D867-	17	0.02
Maharashtra	ORF1a:L3495-	1	0.03	S:D198-	1	0.07	S:D405-	1	0.02	S:E868-	17	0.02
Maharashtra	ORF1a:V3496-	1	0.03	S:G199-	1	0.07	S:E406-	1	0.02	S:M869-	17	0.02
Maharashtra	ORF1a:A3497-	1	0.03	S:Y200-	1	0.07	S:V407-	1	0.02	S:I870-	17	0.02
Maharashtra	ORF1a:M3498-	1	0.03	S:F201-	1	0.07	S:G416-	1	0.02	S:A871-	17	0.02
Maharashtra	ORF1a:K3499-	1	0.03	S:K202-	1	0.07	S:K417-	1	0.02	S:Q872-	17	0.02
Maharashtra	ORF1a:Y3500-	1	0.03	S:I203-	1	0.07	S:I418-	1	0.02	S:Y873-	17	0.02
Maharashtra	ORF1a:N3501-	1	0.03	S:Y204-	1	0.07	S:A419-	1	0.02	S:S884-	17	0.02
Maharashtra	ORF1a:Y3502-	1	0.03	S:S205-	1	0.07	S:D420-	1	0.02	S:G885-	17	0.02
Maharashtra	ORF1a:E3503-	1	0.03	S:K206-	1	0.07	S:Y421-	1	0.02	S:W886-	17	0.02
Maharashtra	ORF1a:P3504-	1	0.03	S:H207-	1	0.07	S:L425-	1	0.02	S:T887-	17	0.02
Maharashtra	ORF1a:L3505-	1	0.03	S:T208-	1	0.07	S:K444-	1	0.02	S:I896-	17	0.02

Maharashtra	ORF1a:T3506-	1	0.03	S:P209-	1	0.07	S:V445-	1	0.02	S:F898-	17	0.02
Maharashtra	ORF1a:Q3507-	1	0.03	S:I210-	1	0.07	S:G446-	1	0.02	S:A899-	17	0.02
Maharashtra	ORF1a:D3508-	1	0.03	S:N211-	1	0.07	S:G447-	1	0.02	ORF1a:Y1418-	17	0.02
Maharashtra	ORF1a:H3509-	1	0.03	S:L212-	1	0.07	S:N448-	1	0.02	ORF1a:G1419-	17	0.02
Maharashtra	ORF1a:V3510-	1	0.03	S:L244-	1	0.07	S:Y449-	1	0.02	ORF1a:N1439-	17	0.02
Maharashtra	ORF1a:D3511-	1	0.03	S:H245-	1	0.07	S:N450-	1	0.02	ORF1a:G1524-	17	0.02
Maharashtra	ORF1a:I3512-	1	0.03	S:R246-	1	0.07	S:Y451-	1	0.02	ORF1a:P2195-	17	0.02
Maharashtra	ORF1a:L3513-	1	0.03	S:S247-	1	0.07	S:L452-	1	0.02	ORF1a:T2196-	17	0.02
Maharashtra	ORF1a:G3514-	1	0.03	S:Y248-	1	0.07	S:L461-	1	0.02	ORF1a:T2197-	17	0.02
Maharashtra	ORF1a:P3515-	1	0.03	S:L249-	1	0.07	S:S469-	1	0.02	ORF1a:I2198-	17	0.02
Maharashtra	ORF1a:G3541-	1	0.03	S:T250-	1	0.07	S:T470-	1	0.02	ORF1a:A2199-	17	0.02
Maharashtra	ORF1a:R3542-	1	0.03	S:P251-	1	0.07	S:E471-	1	0.02	ORF1a:K2200-	17	0.02
Maharashtra	ORF1a:T3543-	1	0.03	S:S256-	1	0.07	S:I472-	1	0.02	ORF1a:N2201-	17	0.02
Maharashtra	ORF1a:I3544-	1	0.03	S:G257-	1	0.07	S:Y473-	1	0.02	ORF1a:T2202-	17	0.02
Maharashtra	ORF1a:L3545-	1	0.03	S:W258-	1	0.07	S:Q474-	1	0.02	ORF1a:V2203-	17	0.02
Maharashtra	ORF1a:G3546-	1	0.03	S:T259-	1	0.07	S:A475-	1	0.02	ORF1a:T2310-	17	0.02
Maharashtra	ORF1a:S3547-	1	0.03	S:A260-	1	0.07	S:G476-	1	0.02	ORF1a:I2311-	17	0.02
Maharashtra	ORF1a:A3548-	1	0.03	S:G261-	1	0.07	S:S477-	1	0.02	ORF1a:Q2373-	17	0.02
Maharashtra	ORF1a:L3549-	1	0.03	S:A262-	1	0.07	S:C480-	1	0.02	ORF1a:M2374-	17	0.02
Maharashtra	ORF1a:L3550-	1	0.03	S:A263-	1	0.07	S:V511-	1	0.02	ORF1a:A2375-	17	0.02
Maharashtra	ORF1a:E3551-	1	0.03	S:A264-	1	0.07	S:F515-	1	0.02	ORF1a:P2376-	17	0.02
Maharashtra	ORF1a:D3552-	1	0.03	S:Y265-	1	0.07	S:E516-	1	0.02	ORF1a:I2377-	17	0.02
Maharashtra	ORF1a:E3553-	1	0.03	S:Y266-	1	0.07	S:L517-	1	0.02	ORF1a:R2011-	17	0.02
Maharashtra	ORF1a:F3554-	1	0.03	S:V267-	1	0.07	S:L518-	1	0.02	ORF1a:S2303-	17	0.02
Maharashtra	ORF1a:T3555-	1	0.03	S:G268-	1	0.07	S:K529-	1	0.02	ORF1a:D2402-	16	0.02
Maharashtra	ORF1a:P3556-	1	0.03	S:Y269-	1	0.07	S:L546-	1	0.02	ORF1a:R2528-	16	0.02
Maharashtra	ORF1a:F3557-	1	0.03	S:L270-	1	0.07	S:T547-	1	0.02	ORF1a:A2529-	16	0.02
Maharashtra	ORF1a:D3558-	1	0.03	S:Q271-	1	0.07	S:K557-	1	0.02	ORF1a:D2573-	16	0.02
Maharashtra	ORF1a:V3559-	1	0.03	S:P272-	1	0.07	S:K558-	1	0.02	ORF1a:T2666-	16	0.02
Maharashtra	ORF1a:V3560-	1	0.03	S:E281-	1	0.07	S:F559-	1	0.02	ORF1a:G2667-	16	0.02
Maharashtra	ORF1a:R3561-	1	0.03	S:N282-	1	0.07	S:L560-	1	0.02	ORF1a:D2668-	16	0.02
Maharashtra	ORF1a:Q3562-	1	0.03	S:G283-	1	0.07	S:Q580-	1	0.02	ORF1a:S2669-	16	0.02
Maharashtra	ORF1a:C3563-	1	0.03	S:T284-	1	0.07	S:T581-	1	0.02	ORF1a:V2766-	16	0.02
Maharashtra	ORF1a:S3564-	1	0.03	S:I285-	1	0.07	S:L582-	1	0.02	ORF1a:A2944-	16	0.02
Maharashtra	ORF1a:G3565-	1	0.03	S:T286-	1	0.07	S:E583-	1	0.02	ORF1a:Y2945-	16	0.02
Maharashtra	ORF1a:V3566-	1	0.03	S:D287-	1	0.07	S:I584-	1	0.02	ORF1a:E2946-	16	0.02
Maharashtra	ORF1a:T3567-	1	0.03	ORF3a:L94-	1	0.07	S:L585-	1	0.02	ORF1a:S2947-	16	0.02
Maharashtra	ORF1a:L3591-	1	0.03	ORF1b:D368-	1	0.07	S:T602-	1	0.02	ORF1a:E2982-	16	0.02
Maharashtra	ORF1a:L3592-	1	0.03	ORF1b:P369-	1	0.07	S:N603-	1	0.02	ORF1a:Y2983-	16	0.02
Maharashtra	ORF1a:V3593-	1	0.03	ORF1b:A370-	1	0.07	S:T604-	1	0.02	ORF1a:F3175-	16	0.02
Maharashtra	ORF1a:L3594-	1	0.03	ORF1b:M371-	1	0.07	S:S605-	1	0.02	ORF1a:N3314-	16	0.02
Maharashtra	ORF1a:V3595-	1	0.03	ORF1b:H372-	1	0.07	S:N606-	1	0.02	ORF1a:P3315-	16	0.02
Maharashtra	ORF1a:Q3596-	1	0.03	ORF1b:A373-	1	0.07	S:Q607-	1	0.02	ORF1a:N3316-	16	0.02
Maharashtra	ORF1a:S3597-	1	0.03	ORF1b:A374-	1	0.07	S:V608-	1	0.02	ORF1a:Y3317-	16	0.02
Maharashtra	ORF1a:T3598-	1	0.03	ORF1b:S375-	1	0.07	S:A609-	1	0.02	ORF1a:H3343-	16	0.02
Maharashtra	ORF1a:Q3599-	1	0.03	ORF1b:G376-	1	0.07	S:V610-	1	0.02	ORF1a:F3403-	16	0.02
Maharashtra	ORF1a:W3600-	1	0.03	ORF1b:N377-	1	0.07	S:L611-	1	0.02	ORF1a:G3433-	16	0.02
Maharashtra	ORF1a:S3601-	1	0.03	ORF1b:L378-	1	0.07	S:A626-	1	0.02	ORF1a:L3468-	16	0.02
Maharashtra	ORF1a:L3602-	1	0.03	ORF1b:L379-	1	0.07	S:D627-	1	0.02	ORF1a:A3469-	16	0.02
Maharashtra	ORF1a:F3603-	1	0.03	ORF1b:L380-	1	0.07	S:Q628-	1	0.02	ORF1a:K3573-	16	0.02
Maharashtra	ORF1a:F3604-	1	0.03	ORF1b:D381-	1	0.07	S:L629-	1	0.02	ORF1a:R3574-	16	0.02
Maharashtra	ORF1a:F3605-	1	0.03	ORF1b:K382-	1	0.07	S:G648-	1	0.02	ORF1a:T3575-	16	0.02
Maharashtra	ORF1a:L3606-	1	0.03	ORF1b:P2612-	1	0.07	S:C649-	1	0.02	ORF1a:I3576-	16	0.02
Maharashtra	ORF1a:Y3607-	1	0.03	ORF1a:K141-	1	0.07	S:L650-	1	0.02	ORF1a:K3577-	16	0.02
Maharashtra	ORF1a:E3608-	1	0.03	ORF1a:S142-	1	0.07	S:I651-	1	0.02	ORF1a:A3645-	16	0.02
Maharashtra	ORF1a:N3609-	1	0.03	ORF1a:F143-	1	0.07	S:G652-	1	0.02	ORF1a:V3647-	16	0.02
Maharashtra	ORF1a:A3610-	1	0.03	S:L241-	1	0.07	S:A653-	1	0.02	ORF1a:A3648-	16	0.02
Maharashtra	ORF1a:F3611-	1	0.03	S:L242-	1	0.07	S:E654-	1	0.02	ORF1a:Y3649-	16	0.02
Maharashtra	ORF1a:L3612-	1	0.03	ORF1a:S2297-	1	0.07	S:H655-	1	0.02	ORF1a:I3693-	16	0.02
Maharashtra	ORF1a:S3622-	1	0.03				S:V656-	1	0.02	ORF1a:L3694-	16	0.02
Maharashtra	ORF1a:A3623-	1	0.03				S:N657-	1	0.02	ORF1a:M3695-	16	0.02
Maharashtra	ORF1a:F3624-	1	0.03				S:N658-	1	0.02	ORF1a:T3696-	16	0.02
Maharashtra	ORF1a:A3625-	1	0.03				S:S659-	1	0.02	ORF1a:A3697-	16	0.02
Maharashtra	ORF1a:M3626-	1	0.03				S:Y660-	1	0.02	ORF1a:R3698-	16	0.02
Maharashtra	ORF1a:M3627-	1	0.03				S:E661-	1	0.02	ORF1a:A3730-	16	0.02
Maharashtra	ORF1a:F3628-	1	0.03				S:G667-	1	0.02	ORF1a:E952-	16	0.02
Maharashtra	ORF1a:V3629-	1	0.03				S:A668-	1	0.02	ORF1a:D953-	16	0.02
Maharashtra	ORF1a:K3630-	1	0.03				S:G669-	1	0.02	ORF1a:D954-	16	0.02
Maharashtra	ORF1a:L3644-	1	0.03				S:I670-	1	0.02	ORF1a:Y955-	16	0.02
Maharashtra	ORF1a:A3645-	1	0.03				M:L57-	1	0.02	ORF1a:Q956-	16	0.02

Maharashtra	ORF1a:T3646-	1	0.03				M:W58-	1	0.02	ORF1a:T965-	16	0.02
Maharashtra	ORF1a:V3647-	1	0.03				M:P59-	1	0.02	ORF1a:S966-	16	0.02
Maharashtra	ORF1a:A3648-	1	0.03				M:V60-	1	0.02	ORF1a:A967-	16	0.02
Maharashtra	ORF1a:Y3649-	1	0.03				M:T61-	1	0.02	ORF1a:A968-	16	0.02
Maharashtra	ORF1a:F3650-	1	0.03				M:L62-	1	0.02	ORF1a:L969-	16	0.02
Maharashtra	ORF1a:N3651-	1	0.03				M:A63-	1	0.02	ORF1a:Q970-	16	0.02
Maharashtra	ORF1a:M3652-	1	0.03				M:C64-	1	0.02	ORF1a:P971-	16	0.02
Maharashtra	ORF1a:V3653-	1	0.03				M:F65-	1	0.02	ORF1a:E972-	16	0.02
Maharashtra	ORF1a:Y3654-	1	0.03				M:A68-	1	0.02	ORF1a:E973-	16	0.02
Maharashtra	ORF1a:M3655-	1	0.03				M:A69-	1	0.02	ORF1a:E974-	16	0.02
Maharashtra	ORF1a:P3656-	1	0.03				M:V70-	1	0.02	ORF1a:Q975-	16	0.02
Maharashtra	ORF1a:A3657-	1	0.03				M:Y71-	1	0.02	ORF1a:E976-	16	0.02
Maharashtra	ORF1a:S3658-	1	0.03				M:R72-	1	0.02	ORF1a:E977-	16	0.02
Maharashtra	ORF1a:W3659-	1	0.03				M:I73-	1	0.02	ORF1a:D981-	16	0.02
Maharashtra	ORF1a:V3660-	1	0.03				M:N74-	1	0.02	ORF1a:D982-	16	0.02
Maharashtra	ORF1a:M3661-	1	0.03				M:W75-	1	0.02	ORF1a:D983-	16	0.02
Maharashtra	ORF1a:R3662-	1	0.03				M:I76-	1	0.02	ORF1a:L3161-	16	0.02
Maharashtra	ORF1a:G3676-	1	0.03				M:T77-	1	0.02	ORF1a:P3253-	16	0.02
Maharashtra	ORF1a:F3677-	1	0.03				M:G78-	1	0.02	ORF1a:Q3254-	16	0.02
Maharashtra	ORF1a:K3678-	1	0.03				M:G79-	1	0.02	ORF1a:Q3507-	16	0.02
Maharashtra	ORF1a:L3679-	1	0.03				M:I80-	1	0.02	ORF1a:D3508-	16	0.02
Maharashtra	ORF1a:K3680-	1	0.03				M:A81-	1	0.02	ORF1a:H3509-	16	0.02
Maharashtra	ORF1a:D3681-	1	0.03				M:I82-	1	0.02	ORF1a:V3510-	16	0.02
Maharashtra	ORF1a:C3682-	1	0.03				M:A83-	1	0.02	ORF1a:K3630-	16	0.02
Maharashtra	ORF1a:V3683-	1	0.03				M:M84-	1	0.02	ORF1a:Q1084-	16	0.02
Maharashtra	ORF1a:M3684-	1	0.03				M:A85-	1	0.02	ORF1a:V1085-	16	0.02
Maharashtra	ORF1a:Y3685-	1	0.03				M:C86-	1	0.02	ORF1a:E1086-	16	0.02
Maharashtra	ORF1a:A3686-	1	0.03				M:I97-	1	0.02	ORF1a:S1087-	16	0.02
Maharashtra	ORF1a:L3692-	1	0.03				M:A98-	1	0.02	ORF1a:L1253-	16	0.02
Maharashtra	ORF1a:I3693-	1	0.03				M:S99-	1	0.02	ORF1a:D1282-	16	0.02
Maharashtra	ORF1a:L3694-	1	0.03				ORF1b:R1366-	1	0.02	ORF1a:A1283-	16	0.02
Maharashtra	ORF1a:M3695-	1	0.03				ORF1b:F2030-	1	0.02	ORF1a:P1284-	16	0.02
Maharashtra	ORF1a:T3696-	1	0.03				ORF1b:S2031-	1	0.02	ORF1a:Y1285-	16	0.02
Maharashtra	ORF1a:A3697-	1	0.03				ORF1b:L2032-	1	0.02	ORF1a:I1286-	16	0.02
Maharashtra	ORF1a:R3698-	1	0.03				ORF1b:W2033-	1	0.02	ORF1a:V1287-	16	0.02
Maharashtra	ORF1a:T3699-	1	0.03				ORF1b:V2034-	1	0.02	ORF1a:G1288-	16	0.02
Maharashtra	ORF1a:V3700-	1	0.03				ORF1b:Y2035-	1	0.02	ORF1a:V1291-	16	0.02
Maharashtra	ORF1a:Y3701-	1	0.03				ORF1b:K2036-	1	0.02	ORF1a:Q1292-	16	0.02
Maharashtra	ORF1a:D3702-	1	0.03				ORF1b:Q2037-	1	0.02	ORF1a:T1504-	16	0.02
Maharashtra	ORF1a:D3703-	1	0.03				ORF1b:F2038-	1	0.02	ORF1a:H1636-	16	0.02
Maharashtra	ORF1a:G3704-	1	0.03				ORF1b:D2039-	1	0.02	ORF1a:P1640-	16	0.02
Maharashtra	ORF1a:A3705-	1	0.03				ORF1b:T2040-	1	0.02	ORF1a:S1641-	16	0.02
Maharashtra	ORF1a:R3706-	1	0.03				ORF1b:Y2041-	1	0.02	ORF1a:F1642-	16	0.02
Maharashtra	ORF1a:Y3719-	1	0.03				ORF1b:N2042-	1	0.02	ORF1a:Q1696-	16	0.02
Maharashtra	ORF1a:K3720-	1	0.03				ORF1b:L2043-	1	0.02	ORF1a:L1741-	16	0.02
Maharashtra	ORF1a:V3721-	1	0.03				ORF1b:D2067-	1	0.02	ORF1a:D1742-	16	0.02
Maharashtra	ORF1a:Y3722-	1	0.03				ORF1b:G2068-	1	0.02	ORF1a:L2364-	16	0.02
Maharashtra	ORF1a:Y3723-	1	0.03				ORF1b:Q2069-	1	0.02	ORF1a:M2365-	16	0.02
Maharashtra	ORF1a:G3724-	1	0.03				ORF1b:Q2070-	1	0.02	ORF1a:W2366-	16	0.02
Maharashtra	ORF1a:N3725-	1	0.03				ORF1b:G2071-	1	0.02	ORF1a:L2367-	16	0.02
Maharashtra	ORF1a:A3726-	1	0.03				ORF1b:E2072-	1	0.02	ORF1a:H2448-	16	0.02
Maharashtra	ORF1a:L3727-	1	0.03				ORF1b:V2073-	1	0.02	ORF1a:E2607-	16	0.02
Maharashtra	ORF1a:D3728-	1	0.03				ORF1b:P2074-	1	0.02	ORF1a:K2608-	16	0.02
Maharashtra	ORF1a:Q3729-	1	0.03				ORF1b:V2075-	1	0.02	ORF1a:L2612-	16	0.02
Maharashtra	ORF1a:A3730-	1	0.03				ORF1b:S2076-	1	0.02	ORF1a:G2893-	16	0.02
Maharashtra	ORF1a:I3731-	1	0.03				ORF1b:I2077-	1	0.02	ORF1a:N2894-	16	0.02
Maharashtra	ORF1a:S3732-	1	0.03				ORF1b:I2078-	1	0.02	ORF1a:L3014-	16	0.02
Maharashtra	ORF1a:M3733-	1	0.03				ORF1b:N2079-	1	0.02	ORF1a:H3304-	16	0.02
Maharashtra	ORF1a:W3734-	1	0.03				ORF1b:N2080-	1	0.02	ORF1a:V3305-	16	0.02
Maharashtra	ORF1a:A3735-	1	0.03				ORF1b:T2081-	1	0.02	ORF1a:R3368-	16	0.02
Maharashtra	ORF1a:L3736-	1	0.03				ORF1b:V2082-	1	0.02	ORF1a:I3369-	16	0.02
Maharashtra	ORF1a:I3737-	1	0.03				ORF1b:T2084-	1	0.02	ORF1a:Q3370-	16	0.02
Maharashtra	ORF1a:I3738-	1	0.03				ORF1b:K2085-	1	0.02	ORF1a:P3371-	16	0.02
Maharashtra	ORF1a:S3739-	1	0.03				ORF1b:V2086-	1	0.02	ORF1a:G3372-	16	0.02
Maharashtra	ORF1a:V3740-	1	0.03				ORF1b:D2087-	1	0.02	ORF1a:I3399-	16	0.02
Maharashtra	ORF1a:T3741-	1	0.03				ORF1b:G2088-	1	0.02	S:F175-	16	0.02
Maharashtra	ORF1a:S3742-	1	0.03				ORF1b:V2089-	1	0.02	S:L176-	16	0.02
Maharashtra	ORF1a:N3743-	1	0.03				ORF1b:D2090-	1	0.02	S:L223-	16	0.02
Maharashtra	ORF1a:Y3744-	1	0.03				ORF1b:V2091-	1	0.02	S:G252-	16	0.02
Maharashtra	ORF1a:S3745-	1	0.03				ORF1b:E2092-	1	0.02	S:D253-	16	0.02

Maharashtra	ORF1a:M3752-	1	0.03				ORF1b:L2093-	1	0.02	S:S254-	16	0.02
Maharashtra	ORF1a:F3753-	1	0.03				ORF1b:F2094-	1	0.02	S:S255-	16	0.02
Maharashtra	ORF1a:L3754-	1	0.03				ORF1b:E2095-	1	0.02	S:C391-	16	0.02
Maharashtra	ORF1a:A3755-	1	0.03				ORF1b:N2096-	1	0.02	S:W436-	16	0.02
Maharashtra	ORF1a:R3756-	1	0.03				ORF1b:K2097-	1	0.02	S:Q779-	16	0.02
Maharashtra	ORF1a:G3757-	1	0.03				ORF1b:T2098-	1	0.02	S:A942-	16	0.02
Maharashtra	ORF1a:I3758-	1	0.03				ORF1b:T2099-	1	0.02	S:T1006-	16	0.02
Maharashtra	ORF1a:V3759-	1	0.03				ORF1b:L2100-	1	0.02	S:K147-	16	0.02
Maharashtra	ORF1a:F3760-	1	0.03				ORF1b:P2101-	1	0.02	S:L425-	16	0.02
Maharashtra	ORF1a:M3761-	1	0.03				ORF1b:V2102-	1	0.02	S:C649-	16	0.02
Maharashtra	ORF1a:C3762-	1	0.03				ORF1b:N2103-	1	0.02	S:L650-	16	0.02
Maharashtra	ORF1a:V3763-	1	0.03				ORF1b:N2113-	1	0.02	S:I651-	16	0.02
Maharashtra	ORF1a:E3764-	1	0.03				ORF1b:I2114-	1	0.02	S:E654-	16	0.02
Maharashtra	ORF1a:Y3765-	1	0.03				ORF1b:K2115-	1	0.02	S:V826-	16	0.02
Maharashtra	ORF1a:F3770-	1	0.03				ORF1b:P2116-	1	0.02	ORF1a:T1572-	16	0.02
Maharashtra	ORF1a:I3771-	1	0.03				ORF1b:V2117-	1	0.02	ORF1a:P1596-	16	0.02
Maharashtra	ORF1a:T3772-	1	0.03				ORF1b:P2118-	1	0.02	ORF1a:T1597-	16	0.02
Maharashtra	ORF1a:G3773-	1	0.03				ORF1b:E2119-	1	0.02	ORF1a:Y1598-	16	0.02
Maharashtra	ORF1a:N3774-	1	0.03				ORF1b:V2120-	1	0.02	ORF1a:C1755-	16	0.02
Maharashtra	ORF1a:T3775-	1	0.03				ORF1b:K2121-	1	0.02	ORF1a:G1756-	16	0.02
Maharashtra	ORF1a:L3776-	1	0.03				ORF1b:I2122-	1	0.02	ORF1a:Q1757-	16	0.02
Maharashtra	ORF1a:Q3777-	1	0.03				ORF1b:L2123-	1	0.02	ORF1a:L1762-	16	0.02
Maharashtra	ORF1a:C3778-	1	0.03				ORF1b:N2124-	1	0.02	ORF1a:K1763-	16	0.02
Maharashtra	ORF1a:I3779-	1	0.03				ORF1b:N2125-	1	0.02	ORF1a:P2046-	16	0.02
Maharashtra	ORF1a:M3780-	1	0.03				ORF1b:L2126-	1	0.02	ORF1a:M2096-	16	0.02
Maharashtra	ORF1a:C3790-	1	0.03				ORF1b:I2130-	1	0.02	ORF1a:S2273-	16	0.02
Maharashtra	ORF1a:T3791-	1	0.03				ORF1b:A2131-	1	0.02	ORF1a:T2274-	16	0.02
Maharashtra	ORF1b:D368-	1	0.03				ORF1b:A2132-	1	0.02	ORF1a:N2275-	16	0.02
Maharashtra	ORF1b:P369-	1	0.03				ORF1b:N2133-	1	0.02	ORF1a:W2327-	16	0.02
Maharashtra	ORF1b:A370-	1	0.03				ORF1b:T2134-	1	0.02	ORF1a:F2328-	16	0.02
Maharashtra	ORF1b:M371-	1	0.03				ORF1b:V2135-	1	0.02	ORF1a:L2329-	16	0.02
Maharashtra	ORF1b:H372-	1	0.03				ORF1b:D2142-	1	0.02	ORF1a:A2330-	16	0.02
Maharashtra	ORF1b:A373-	1	0.03				ORF1b:A2143-	1	0.02	ORF1a:Y2331-	16	0.02
Maharashtra	ORF1b:A374-	1	0.03				ORF1b:P2144-	1	0.02	ORF1a:I2332-	16	0.02
Maharashtra	ORF1b:S375-	1	0.03				ORF1b:A2145-	1	0.02	ORF1a:T2495-	16	0.02
Maharashtra	ORF1b:T384-	1	0.03				ORF1b:H2146-	1	0.02	ORF1a:I2541-	16	0.02
Maharashtra	ORF1b:T385-	1	0.03				ORF1b:I2147-	1	0.02	ORF1a:V2542-	16	0.02
Maharashtra	ORF1b:C386-	1	0.03				ORF1b:S2148-	1	0.02	ORF1a:E2940-	16	0.02
Maharashtra	ORF1b:F387-	1	0.03				ORF1b:T2149-	1	0.02	ORF1a:G2941-	16	0.02
Maharashtra	ORF1b:S388-	1	0.03				ORF1b:I2150-	1	0.02	ORF1a:L3378-	16	0.02
Maharashtra	ORF1b:V389-	1	0.03				ORF1b:G2151-	1	0.02	ORF1a:K1973-	16	0.02
Maharashtra	ORF1b:A390-	1	0.03				ORF1b:V2152-	1	0.02	ORF1a:C2064-	16	0.02
Maharashtra	ORF1b:A391-	1	0.03				ORF1b:A2159-	1	0.02	ORF1a:L2127-	16	0.02
Maharashtra	ORF1b:L392-	1	0.03				ORF1b:K2160-	1	0.02	ORF1a:I2156-	16	0.02
Maharashtra	ORF1b:T393-	1	0.03				ORF1b:K2161-	1	0.02	ORF1a:V2157-	16	0.02
Maharashtra	ORF1b:N394-	1	0.03				ORF1b:P2162-	1	0.02	ORF1a:T2158-	16	0.02
Maharashtra	ORF1b:N395-	1	0.03				ORF1b:T2163-	1	0.02	ORF1a:R2159-	16	0.02
Maharashtra	ORF1b:V396-	1	0.03				ORF1b:E2164-	1	0.02	ORF1a:Y2262-	16	0.02
Maharashtra	ORF1b:V401-	1	0.03				ORF1b:T2165-	1	0.02	ORF1a:C2263-	16	0.02
Maharashtra	ORF1b:K402-	1	0.03				ORF1b:I2166-	1	0.02	ORF1a:T2264-	16	0.02
Maharashtra	ORF1b:P403-	1	0.03				ORF1b:C2167-	1	0.02	ORF1a:G2265-	16	0.02
Maharashtra	ORF1b:G404-	1	0.03				ORF1b:A2168-	1	0.02	ORF1a:G2269-	16	0.02
Maharashtra	ORF1b:N405-	1	0.03				ORF1b:P2169-	1	0.02	ORF1a:T1437-	16	0.02
Maharashtra	ORF1b:F406-	1	0.03				ORF1b:L2170-	1	0.02	ORF1a:L1438-	16	0.02
Maharashtra	ORF1b:N407-	1	0.03				ORF1b:V2178-	1	0.02	ORF1a:F2387-	15	0.02
Maharashtra	ORF1b:K408-	1	0.03				ORF1b:D2179-	1	0.02	ORF1a:A2388-	15	0.02
Maharashtra	ORF1b:D409-	1	0.03				ORF1b:G2180-	1	0.02	ORF1a:F2464-	15	0.02
Maharashtra	ORF1b:F410-	1	0.03				ORF1b:Q2181-	1	0.02	ORF1a:S2661-	15	0.02
Maharashtra	ORF1b:Y411-	1	0.03				ORF1b:V2182-	1	0.02	ORF1a:F2922-	15	0.02
Maharashtra	ORF1b:D412-	1	0.03				ORF1b:D2183-	1	0.02	ORF1a:K2923-	15	0.02
Maharashtra	ORF1b:F413-	1	0.03				ORF1b:L2184-	1	0.02	ORF1a:D2924-	15	0.02
Maharashtra	ORF1b:A414-	1	0.03				ORF1b:F2185-	1	0.02	ORF1a:A2925-	15	0.02
Maharashtra	ORF1b:V415-	1	0.03				ORF1b:R2186-	1	0.02	ORF1a:S2926-	15	0.02
Maharashtra	ORF1b:S416-	1	0.03				ORF1b:N2187-	1	0.02	ORF1a:L2948-	15	0.02
Maharashtra	ORF1b:K417-	1	0.03				ORF1b:A2188-	1	0.02	ORF1a:L3210-	15	0.02
Maharashtra	ORF1b:G418-	1	0.03				ORF1b:R2189-	1	0.02	ORF1a:K3213-	15	0.02
Maharashtra	ORF1b:F419-	1	0.03				ORF1b:N2190-	1	0.02	ORF1a:Y3214-	15	0.02
Maharashtra	ORF1b:F420-	1	0.03				ORF1b:G2191-	1	0.02	ORF1a:A3357-	15	0.02
Maharashtra	ORF1b:V426-	1	0.03				ORF1b:V2192-	1	0.02	ORF1a:N3358-	15	0.02
Maharashtra	ORF1b:E427-	1	0.03				ORF1b:V2199-	1	0.02	ORF1a:P3359-	15	0.02

Maharashtra	ORF1b:L428-	1	0.03				ORF1b:K2200-	1	0.02	ORF1a:K3360-	15	0.02
Maharashtra	ORF1b:K429-	1	0.03				ORF1b:G2201-	1	0.02	ORF1a:T3361-	15	0.02
Maharashtra	ORF1b:H430-	1	0.03				ORF1b:L2202-	1	0.02	ORF1a:F3366-	15	0.02
Maharashtra	ORF1b:F431-	1	0.03				ORF1b:Q2203-	1	0.02	ORF1a:S3386-	15	0.02
Maharashtra	ORF1b:F432-	1	0.03				ORF1b:P2204-	1	0.02	ORF1a:G3387-	15	0.02
Maharashtra	ORF1b:F433-	1	0.03				ORF1b:S2205-	1	0.02	ORF1a:V3388-	15	0.02
Maharashtra	ORF1b:A434-	1	0.03				ORF1b:V2206-	1	0.02	ORF1a:Y3389-	15	0.02
Maharashtra	ORF1b:Q435-	1	0.03				ORF1b:G2207-	1	0.02	ORF1a:Y3500-	15	0.02
Maharashtra	ORF1b:D436-	1	0.03				ORF1b:P2208-	1	0.02	ORF1a:N3501-	15	0.02
Maharashtra	ORF1b:G437-	1	0.03				ORF1b:K2209-	1	0.02	ORF1a:Y3502-	15	0.02
Maharashtra	ORF1b:N438-	1	0.03				ORF1b:L2218-	1	0.02	ORF1a:E3503-	15	0.02
Maharashtra	ORF1b:A439-	1	0.03				ORF1b:I2219-	1	0.02	ORF1a:G3541-	15	0.02
Maharashtra	ORF1b:A440-	1	0.03				ORF1b:G2220-	1	0.02	ORF1a:V3560-	15	0.02
Maharashtra	ORF1b:I441-	1	0.03				ORF1b:E2221-	1	0.02	ORF1a:L3644-	15	0.02
Maharashtra	ORF1b:S442-	1	0.03				ORF1b:A2222-	1	0.02	ORF1a:G3757-	15	0.02
Maharashtra	ORF1b:D443-	1	0.03				ORF1b:V2223-	1	0.02	ORF1a:I3758-	15	0.02
Maharashtra	ORF1b:Y444-	1	0.03				ORF1b:K2224-	1	0.02	ORF1a:V3759-	15	0.02
Maharashtra	ORF1b:D445-	1	0.03				ORF1b:T2225-	1	0.02	ORF1a:N3801-	15	0.02
Maharashtra	ORF1b:L461-	1	0.03				ORF1b:Q2226-	1	0.02	ORF1a:R3802-	15	0.02
Maharashtra	ORF1b:F462-	1	0.03				ORF1b:F2227-	1	0.02	ORF1a:C873-	15	0.02
Maharashtra	ORF1b:V463-	1	0.03				ORF1b:N2228-	1	0.02	ORF1a:V874-	15	0.02
Maharashtra	ORF1b:V464-	1	0.03				ORF1b:Y2229-	1	0.02	ORF1a:V875-	15	0.02
Maharashtra	ORF1b:E465-	1	0.03				ORF1b:Y2230-	1	0.02	ORF1a:A876-	15	0.02
Maharashtra	ORF1b:V466-	1	0.03				ORF1b:K2231-	1	0.02	ORF1a:D877-	15	0.02
Maharashtra	ORF1b:V467-	1	0.03				ORF1b:K2232-	1	0.02	ORF1a:Y921-	15	0.02
Maharashtra	ORF1b:D468-	1	0.03				ORF1b:V2233-	1	0.02	ORF1a:F924-	15	0.02
Maharashtra	ORF1b:K469-	1	0.03				ORF1b:D2234-	1	0.02	ORF1a:Y925-	15	0.02
Maharashtra	ORF1b:Y470-	1	0.03				ORF1b:G2235-	1	0.02	ORF1a:P926-	15	0.02
Maharashtra	ORF1b:F471-	1	0.03				ORF1b:V2236-	1	0.02	ORF1a:P927-	15	0.02
Maharashtra	ORF1b:D472-	1	0.03				ORF1b:D2263-	1	0.02	ORF1a:D928-	15	0.02
Maharashtra	ORF1b:C473-	1	0.03				ORF1b:F2264-	1	0.02	ORF1a:E929-	15	0.02
Maharashtra	ORF1b:Y474-	1	0.03				ORF1b:L2265-	1	0.02	ORF1a:D930-	15	0.02
Maharashtra	ORF1b:D475-	1	0.03				ORF1b:E2266-	1	0.02	ORF1a:E931-	15	0.02
Maharashtra	ORF1b:G476-	1	0.03				ORF1b:L2267-	1	0.02	ORF1a:E932-	15	0.02
Maharashtra	ORF1b:G477-	1	0.03				ORF1b:A2282-	1	0.02	ORF1a:E933-	15	0.02
Maharashtra	ORF1b:C478-	1	0.03				ORF1b:F2283-	1	0.02	ORF1a:G934-	15	0.02
Maharashtra	ORF1b:I479-	1	0.03				ORF1b:E2284-	1	0.02	ORF1a:D935-	15	0.02
Maharashtra	ORF1b:N480-	1	0.03				ORF1b:H2285-	1	0.02	ORF1a:C936-	15	0.02
Maharashtra	ORF1b:A481-	1	0.03				ORF1b:I2286-	1	0.02	ORF1a:E937-	15	0.02
Maharashtra	ORF1b:N482-	1	0.03				ORF1b:V2287-	1	0.02	ORF1a:E938-	15	0.02
Maharashtra	ORF1b:Q483-	1	0.03				ORF1b:Y2288-	1	0.02	ORF1a:E939-	15	0.02
Maharashtra	ORF1b:V484-	1	0.03				ORF1b:G2289-	1	0.02	ORF1a:E940-	15	0.02
Maharashtra	ORF1b:N487-	1	0.03				ORF1b:D2290-	1	0.02	ORF1a:F941-	15	0.02
Maharashtra	ORF1b:N488-	1	0.03				ORF1b:F2291-	1	0.02	ORF1a:D3511-	15	0.02
Maharashtra	ORF1b:L489-	1	0.03				ORF1b:S2292-	1	0.02	S:V120-	15	0.02
Maharashtra	ORF1b:D490-	1	0.03				ORF1b:H2293-	1	0.02	S:A123-	15	0.02
Maharashtra	ORF1b:K491-	1	0.03				ORF1b:S2294-	1	0.02	ORF1a:T1017-	15	0.02
Maharashtra	ORF1b:S492-	1	0.03				ORF1b:G2297-	1	0.02	ORF1a:P1018-	15	0.02
Maharashtra	ORF1b:A493-	1	0.03				ORF1b:G2298-	1	0.02	ORF1a:V1019-	15	0.02
Maharashtra	ORF1b:G494-	1	0.03				ORF1b:L2299-	1	0.02	ORF1a:V1020-	15	0.02
Maharashtra	ORF1b:F495-	1	0.03				ORF1b:H2300-	1	0.02	ORF1a:A1043-	15	0.02
Maharashtra	ORF1b:L505-	1	0.03				ORF1b:L2301-	1	0.02	ORF1a:D1044-	15	0.02
Maharashtra	ORF1b:Y506-	1	0.03				ORF1b:L2302-	1	0.02	ORF1a:I1045-	15	0.02
Maharashtra	ORF1b:Y507-	1	0.03				ORF1b:I2303-	1	0.02	ORF1a:V1046-	15	0.02
Maharashtra	ORF1b:D508-	1	0.03				ORF1b:G2304-	1	0.02	ORF1a:E1047-	15	0.02
Maharashtra	ORF1b:S509-	1	0.03				ORF1b:L2305-	1	0.02	ORF1a:E1048-	15	0.02
Maharashtra	ORF1b:M510-	1	0.03				ORF1b:A2306-	1	0.02	ORF1a:A1049-	15	0.02
Maharashtra	ORF1b:S511-	1	0.03				ORF1a:L18-	1	0.02	ORF1a:A1078-	15	0.02
Maharashtra	ORF1b:Y512-	1	0.03				ORF1a:P19-	1	0.02	ORF1a:T1079-	15	0.02
Maharashtra	ORF1b:E513-	1	0.03				ORF1a:V20-	1	0.02	ORF1a:D1088-	15	0.02
Maharashtra	ORF1b:D514-	1	0.03				ORF1a:L21-	1	0.02	ORF1a:D1089-	15	0.02
Maharashtra	ORF1b:Q515-	1	0.03				ORF1a:Q22-	1	0.02	ORF1a:D1127-	15	0.02
Maharashtra	ORF1b:D516-	1	0.03				ORF1a:V23-	1	0.02	ORF1a:I1128-	15	0.02
Maharashtra	ORF1b:A517-	1	0.03				ORF1a:R24-	1	0.02	ORF1a:A1204-	15	0.02
Maharashtra	ORF1b:L518-	1	0.03				ORF1a:D25-	1	0.02	ORF1a:E1205-	15	0.02
Maharashtra	ORF1b:F519-	1	0.03				ORF1a:V26-	1	0.02	ORF1a:I1206-	15	0.02
Maharashtra	ORF1b:A520-	1	0.03				ORF1a:L27-	1	0.02	ORF1a:P1207-	15	0.02
Maharashtra	ORF1b:V526-	1	0.03				ORF1a:V28-	1	0.02	ORF1a:K1208-	15	0.02
Maharashtra	ORF1b:I527-	1	0.03				ORF1a:R29-	1	0.02	ORF1a:E1209-	15	0.02
Maharashtra	ORF1b:P528-	1	0.03				ORF1a:G30-	1	0.02	ORF1a:E1210-	15	0.02

Maharashtra	ORF1b:T529-	1	0.03				ORF1a:F31-	1	0.02	ORF1a:V1211-	15	0.02
Maharashtra	ORF1b:I530-	1	0.03				ORF1a:G32-	1	0.02	ORF1a:K1212-	15	0.02
Maharashtra	ORF1b:T531-	1	0.03				ORF1a:D33-	1	0.02	ORF1a:Q1227-	15	0.02
Maharashtra	ORF1b:Q532-	1	0.03				ORF1a:S34-	1	0.02	ORF1a:M1586-	15	0.02
Maharashtra	ORF1b:M533-	1	0.03				ORF1a:V35-	1	0.02	ORF1a:L1643-	15	0.02
Maharashtra	ORF1b:N534-	1	0.03				ORF1a:E36-	1	0.02	ORF1a:G1644-	15	0.02
Maharashtra	ORF1b:L535-	1	0.03				ORF1a:D48-	1	0.02	ORF1a:F1690-	15	0.02
Maharashtra	ORF1b:K536-	1	0.03				ORF1a:G49-	1	0.02	ORF1a:N1691-	15	0.02
Maharashtra	ORF1b:Y537-	1	0.03				ORF1a:T50-	1	0.02	ORF1a:P1692-	15	0.02
Maharashtra	ORF1b:A538-	1	0.03				ORF1a:C51-	1	0.02	ORF1a:P1693-	15	0.02
Maharashtra	ORF1b:I539-	1	0.03				ORF1a:G52-	1	0.02	ORF1a:A1694-	15	0.02
Maharashtra	ORF1b:S540-	1	0.03				ORF1a:L53-	1	0.02	ORF1a:L1695-	15	0.02
Maharashtra	ORF1b:A541-	1	0.03				ORF1a:V54-	1	0.02	ORF1a:K1791-	15	0.02
Maharashtra	ORF1b:C554-	1	0.03				ORF1a:E55-	1	0.02	ORF1a:L2609-	15	0.02
Maharashtra	ORF1b:S555-	1	0.03				ORF1a:V56-	1	0.02	ORF1a:K2610-	15	0.02
Maharashtra	ORF1b:T556-	1	0.03				ORF1a:E57-	1	0.02	ORF1a:T2611-	15	0.02
Maharashtra	ORF1b:M557-	1	0.03				ORF1a:K58-	1	0.02	ORF1a:T2648-	15	0.02
Maharashtra	ORF1b:T558-	1	0.03				ORF1a:G59-	1	0.02	ORF1a:K2649-	15	0.02
Maharashtra	ORF1b:N559-	1	0.03				ORF1a:V60-	1	0.02	ORF1a:D2650-	15	0.02
Maharashtra	ORF1b:R560-	1	0.03				ORF1a:L61-	1	0.02	ORF1a:L2675-	15	0.02
Maharashtra	ORF1b:Q561-	1	0.03				ORF1a:P62-	1	0.02	ORF1a:T2676-	15	0.02
Maharashtra	ORF1b:F562-	1	0.03				ORF1a:Q63-	1	0.02	ORF1a:A2710-	15	0.02
Maharashtra	ORF1b:H563-	1	0.03				ORF1a:L64-	1	0.02	ORF1a:L2711-	15	0.02
Maharashtra	ORF1b:Q564-	1	0.03				ORF1a:E65-	1	0.02	ORF1a:I2712-	15	0.02
Maharashtra	ORF1b:K565-	1	0.03				ORF1a:Q66-	1	0.02	ORF1a:W2713-	15	0.02
Maharashtra	ORF1b:L566-	1	0.03				ORF1a:P67-	1	0.02	ORF1a:N2714-	15	0.02
Maharashtra	ORF1b:L567-	1	0.03				ORF1a:Y68-	1	0.02	ORF1a:V2715-	15	0.02
Maharashtra	ORF1b:K568-	1	0.03				ORF1a:V69-	1	0.02	ORF1a:K2716-	15	0.02
Maharashtra	ORF1b:S569-	1	0.03				ORF1a:F70-	1	0.02	ORF1a:V2914-	15	0.02
Maharashtra	ORF1b:I570-	1	0.03				ORF1a:I71-	1	0.02	ORF1a:R2974-	15	0.02
Maharashtra	ORF1b:A571-	1	0.03				ORF1a:T78-	1	0.02	ORF1a:L3083-	15	0.02
Maharashtra	ORF1b:A572-	1	0.03				ORF1a:A79-	1	0.02	ORF1a:Q3251-	15	0.02
Maharashtra	ORF1b:T573-	1	0.03				ORF1a:P80-	1	0.02	ORF1a:P3252-	15	0.02
Maharashtra	ORF1b:R574-	1	0.03				ORF1a:H81-	1	0.02	ORF1a:C3279-	15	0.02
Maharashtra	ORF1b:G575-	1	0.03				ORF1a:G82-	1	0.02	ORF1a:M3280-	15	0.02
Maharashtra	ORF1b:A576-	1	0.03				ORF1a:H83-	1	0.02	ORF1a:V3281-	15	0.02
Maharashtra	ORF1b:T577-	1	0.03				ORF1a:V84-	1	0.02	ORF1a:Q3282-	15	0.02
Maharashtra	ORF1b:V578-	1	0.03				ORF1a:A90-	1	0.02	ORF1a:V3283-	15	0.02
Maharashtra	ORF1b:G581-	1	0.03				ORF1a:E91-	1	0.02	ORF1a:T3284-	15	0.02
Maharashtra	ORF1b:T582-	1	0.03				ORF1a:L92-	1	0.02	ORF1a:C3285-	15	0.02
Maharashtra	ORF1b:S583-	1	0.03				ORF1a:E93-	1	0.02	ORF1a:G3286-	15	0.02
Maharashtra	ORF1b:K584-	1	0.03				ORF1a:G94-	1	0.02	ORF1a:T3287-	15	0.02
Maharashtra	ORF1b:F585-	1	0.03				ORF1a:I95-	1	0.02	ORF1a:T3288-	15	0.02
Maharashtra	ORF1b:Y586-	1	0.03				ORF1a:Q96-	1	0.02	S:G219-	15	0.02
Maharashtra	ORF1b:G587-	1	0.03				ORF1a:Y97-	1	0.02	S:F220-	15	0.02
Maharashtra	ORF1b:G588-	1	0.03				ORF1a:G98-	1	0.02	S:S221-	15	0.02
Maharashtra	ORF1b:W589-	1	0.03				ORF1a:R99-	1	0.02	S:A222-	15	0.02
Maharashtra	ORF1b:H590-	1	0.03				ORF1a:S100-	1	0.02	S:E224-	15	0.02
Maharashtra	ORF1b:N591-	1	0.03				ORF1a:G101-	1	0.02	S:P225-	15	0.02
Maharashtra	ORF1b:M592-	1	0.03				ORF1a:E102-	1	0.02	S:I233-	15	0.02
Maharashtra	ORF1b:L593-	1	0.03				ORF1a:T103-	1	0.02	S:N234-	15	0.02
Maharashtra	ORF1b:K594-	1	0.03				ORF1a:L104-	1	0.02	S:I235-	15	0.02
Maharashtra	ORF1b:T595-	1	0.03				ORF1a:G105-	1	0.02	S:T236-	15	0.02
Maharashtra	ORF1b:V596-	1	0.03				ORF1a:V111-	1	0.02	S:L277-	15	0.02
Maharashtra	ORF1b:Y597-	1	0.03				ORF1a:G112-	1	0.02	S:K278-	15	0.02
Maharashtra	ORF1b:S598-	1	0.03				ORF1a:E113-	1	0.02	S:D428-	15	0.02
Maharashtra	ORF1b:D599-	1	0.03				ORF1a:I114-	1	0.02	S:F429-	15	0.02
Maharashtra	ORF1b:P611-	1	0.03				ORF1a:P115-	1	0.02	S:T430-	15	0.02
Maharashtra	ORF1b:K612-	1	0.03				ORF1a:V116-	1	0.02	S:G431-	15	0.02
Maharashtra	ORF1b:C613-	1	0.03				ORF1a:A117-	1	0.02	S:C488-	15	0.02
Maharashtra	ORF1b:D614-	1	0.03				ORF1a:Y118-	1	0.02	S:Q774-	15	0.02
Maharashtra	ORF1b:R615-	1	0.03				ORF1a:R119-	1	0.02	S:D775-	15	0.02
Maharashtra	ORF1b:A616-	1	0.03				ORF1a:N126-	1	0.02	S:K776-	15	0.02
Maharashtra	ORF1b:M617-	1	0.03				ORF1a:G127-	1	0.02	S:N777-	15	0.02
Maharashtra	ORF1b:P618-	1	0.03				ORF1a:N128-	1	0.02	S:T778-	15	0.02
Maharashtra	ORF1b:N619-	1	0.03				ORF1a:K129-	1	0.02	S:I805-	15	0.02
Maharashtra	ORF1b:M620-	1	0.03				ORF1a:G130-	1	0.02	S:S875-	15	0.02
Maharashtra	ORF1b:L621-	1	0.03				ORF1a:A131-	1	0.02	S:A876-	15	0.02
Maharashtra	ORF1b:R622-	1	0.03				ORF1a:G132-	1	0.02	S:L877-	15	0.02
Maharashtra	ORF1b:I623-	1	0.03				ORF1a:G133-	1	0.02	S:L878-	15	0.02

Maharashtra	ORF1b:M624-	1	0.03				ORF1a:H134-	1	0.02	S:A879-	15	0.02
Maharashtra	ORF1b:A625-	1	0.03				ORF1a:S135-	1	0.02	S:G880-	15	0.02
Maharashtra	ORF1b:S626-	1	0.03				ORF1a:Y136-	1	0.02	S:S975-	15	0.02
Maharashtra	ORF1b:L627-	1	0.03				ORF1a:G137-	1	0.02	S:E988-	15	0.02
Maharashtra	ORF1b:V628-	1	0.03				ORF1a:A138-	1	0.02	S:A989-	15	0.02
Maharashtra	ORF1b:L629-	1	0.03				ORF1a:D139-	1	0.02	S:E990-	15	0.02
Maharashtra	ORF1b:A630-	1	0.03				ORF1a:L140-	1	0.02	S:V991-	15	0.02
Maharashtra	ORF1b:R631-	1	0.03				ORF1a:K141-	1	0.02	S:Q992-	15	0.02
Maharashtra	ORF1b:K632-	1	0.03				ORF1a:S142-	1	0.02	S:T998-	15	0.02
Maharashtra	ORF1b:L639-	1	0.03				ORF1a:F143-	1	0.02	S:Q1005-	15	0.02
Maharashtra	ORF1b:S640-	1	0.03				ORF1a:D144-	1	0.02	S:L1034-	15	0.02
Maharashtra	ORF1b:H641-	1	0.03				ORF1a:L145-	1	0.02	S:G1035-	15	0.02
Maharashtra	ORF1b:R642-	1	0.03				ORF1a:G146-	1	0.02	S:A1056-	15	0.02
Maharashtra	ORF1b:F643-	1	0.03				ORF1a:D147-	1	0.02	S:P1057-	15	0.02
Maharashtra	ORF1b:Y644-	1	0.03				ORF1a:D152-	1	0.02	S:H1058-	15	0.02
Maharashtra	ORF1b:R645-	1	0.03				ORF1a:P153-	1	0.02	S:F140-	15	0.02
Maharashtra	ORF1b:L646-	1	0.03				ORF1a:Y154-	1	0.02	S:L296-	15	0.02
Maharashtra	ORF1b:A647-	1	0.03				ORF1a:E155-	1	0.02	S:S297-	15	0.02
Maharashtra	ORF1b:N648-	1	0.03				ORF1a:D156-	1	0.02	S:E298-	15	0.02
Maharashtra	ORF1b:E649-	1	0.03				ORF1a:F157-	1	0.02	S:T299-	15	0.02
Maharashtra	ORF1b:C650-	1	0.03				ORF1a:Q158-	1	0.02	S:K300-	15	0.02
Maharashtra	ORF1b:A651-	1	0.03				ORF1a:E159-	1	0.02	S:I332-	15	0.02
Maharashtra	ORF1b:Q652-	1	0.03				ORF1a:N160-	1	0.02	S:T333-	15	0.02
Maharashtra	ORF1b:V653-	1	0.03				ORF1a:W161-	1	0.02	S:N334-	15	0.02
Maharashtra	ORF1b:L654-	1	0.03				ORF1a:N162-	1	0.02	S:L335-	15	0.02
Maharashtra	ORF1b:S655-	1	0.03				ORF1a:T163-	1	0.02	S:C336-	15	0.02
Maharashtra	ORF1b:E656-	1	0.03				ORF1a:K164-	1	0.02	S:Y365-	15	0.02
Maharashtra	ORF1b:M657-	1	0.03				ORF1a:H165-	1	0.02	S:S366-	15	0.02
Maharashtra	ORF1b:V658-	1	0.03				ORF1a:S166-	1	0.02	S:L461-	15	0.02
Maharashtra	ORF1b:M659-	1	0.03				ORF1a:S167-	1	0.02	S:G652-	15	0.02
Maharashtra	ORF1b:C660-	1	0.03				ORF1a:G168-	1	0.02	S:A653-	15	0.02
Maharashtra	ORF1b:G661-	1	0.03				ORF1a:V169-	1	0.02	S:H655-	15	0.02
Maharashtra	ORF1b:G662-	1	0.03				ORF1a:T170-	1	0.02	S:T323-	15	0.02
Maharashtra	ORF1b:L664-	1	0.03				ORF1a:R171-	1	0.02	S:E324-	15	0.02
Maharashtra	ORF1b:Y665-	1	0.03				ORF1a:E172-	1	0.02	S:S325-	15	0.02
Maharashtra	ORF1b:V666-	1	0.03				ORF1a:L173-	1	0.02	ORF1a:I1203-	15	0.02
Maharashtra	ORF1b:K667-	1	0.03				ORF1a:M174-	1	0.02	ORF1a:I1232-	15	0.02
Maharashtra	ORF1b:P668-	1	0.03				ORF1a:D187-	1	0.02	ORF1a:P1330-	15	0.02
Maharashtra	ORF1b:G669-	1	0.03				ORF1a:N188-	1	0.02	ORF1a:G1331-	15	0.02
Maharashtra	ORF1b:G670-	1	0.03				ORF1a:N189-	1	0.02	ORF1a:Q1332-	15	0.02
Maharashtra	ORF1b:T671-	1	0.03				ORF1a:F190-	1	0.02	ORF1a:G1333-	15	0.02
Maharashtra	ORF1b:S672-	1	0.03				ORF1a:C191-	1	0.02	ORF1a:C1392-	15	0.02
Maharashtra	ORF1b:S673-	1	0.03				ORF1a:G192-	1	0.02	ORF1a:V1393-	15	0.02
Maharashtra	ORF1b:G674-	1	0.03				ORF1a:P193-	1	0.02	ORF1a:E1394-	15	0.02
Maharashtra	ORF1b:D675-	1	0.03				ORF1a:D194-	1	0.02	ORF1a:T1395-	15	0.02
Maharashtra	ORF1b:A676-	1	0.03				ORF1a:G195-	1	0.02	ORF1a:K1396-	15	0.02
Maharashtra	ORF1b:T677-	1	0.03				ORF1a:Y196-	1	0.02	ORF1a:A1397-	15	0.02
Maharashtra	ORF1b:T678-	1	0.03				ORF1a:P197-	1	0.02	ORF1a:I1398-	15	0.02
Maharashtra	ORF1b:A679-	1	0.03				ORF1a:L198-	1	0.02	ORF1a:K1470-	15	0.02
Maharashtra	ORF1b:Y680-	1	0.03				ORF1a:E199-	1	0.02	ORF1a:V1471-	15	0.02
Maharashtra	ORF1b:A681-	1	0.03				ORF1a:C200-	1	0.02	ORF1a:P1472-	15	0.02
Maharashtra	ORF1b:N682-	1	0.03				ORF1a:I201-	1	0.02	ORF1a:T1474-	15	0.02
Maharashtra	ORF1b:S683-	1	0.03				ORF1a:K202-	1	0.02	ORF1a:F1501-	15	0.02
Maharashtra	ORF1b:V684-	1	0.03				ORF1a:D203-	1	0.02	ORF1a:L1546-	15	0.02
Maharashtra	ORF1b:F685-	1	0.03				ORF1a:L204-	1	0.02	ORF1a:D1547-	15	0.02
Maharashtra	ORF1b:C688-	1	0.03				ORF1a:L205-	1	0.02	ORF1a:T1573-	15	0.02
Maharashtra	ORF1b:Q689-	1	0.03				ORF1a:A206-	1	0.02	ORF1a:V1574-	15	0.02
Maharashtra	ORF1b:A690-	1	0.03				ORF1a:R207-	1	0.02	ORF1a:D1575-	15	0.02
Maharashtra	ORF1b:V691-	1	0.03				ORF1a:S216-	1	0.02	ORF1a:N1576-	15	0.02
Maharashtra	ORF1b:T692-	1	0.03				ORF1a:E217-	1	0.02	ORF1a:K1956-	15	0.02
Maharashtra	ORF1b:A693-	1	0.03				ORF1a:Q218-	1	0.02	ORF1a:V1957-	15	0.02
Maharashtra	ORF1b:N694-	1	0.03				ORF1a:L219-	1	0.02	ORF1a:K2108-	15	0.02
Maharashtra	ORF1b:V695-	1	0.03				ORF1a:D220-	1	0.02	ORF1a:K2109-	15	0.02
Maharashtra	ORF1b:N696-	1	0.03				ORF1a:F221-	1	0.02	ORF1a:P2110-	15	0.02
Maharashtra	ORF1b:A697-	1	0.03				ORF1a:I222-	1	0.02	ORF1a:E2681-	15	0.02
Maharashtra	ORF1b:L698-	1	0.03				ORF1a:C230-	1	0.02	ORF1a:H2707-	15	0.02
Maharashtra	ORF1b:L699-	1	0.03				ORF1a:C231-	1	0.02	ORF1a:S2942-	15	0.02
Maharashtra	ORF1b:S700-	1	0.03				ORF1a:R232-	1	0.02	ORF1a:V2943-	15	0.02
Maharashtra	ORF1b:T701-	1	0.03				ORF1a:E233-	1	0.02	ORF1a:I2086-	15	0.02
Maharashtra	ORF1b:D702-	1	0.03				ORF1a:H234-	1	0.02	S:Y91-	15	0.02

Maharashtra	ORF1b:G703-	1	0.03				ORF1a:E235-	1	0.02	S:F92-	15	0.02
Maharashtra	ORF1b:N704-	1	0.03				ORF1a:H236-	1	0.02	S:A93-	15	0.02
Maharashtra	ORF1b:K705-	1	0.03				ORF1a:E237-	1	0.02	S:S98-	15	0.02
Maharashtra	ORF1b:I706-	1	0.03				ORF1a:I238-	1	0.02	S:N99-	15	0.02
Maharashtra	ORF1b:A707-	1	0.03				ORF1a:A239-	1	0.02	ORF1a:L2349-	14	0.02
Maharashtra	ORF1b:D708-	1	0.03				ORF1a:W240-	1	0.02	ORF1a:F2350-	14	0.02
Maharashtra	ORF1b:K709-	1	0.03				ORF1a:Y241-	1	0.02	ORF1a:F2351-	14	0.02
Maharashtra	ORF1b:N725-	1	0.03				ORF1a:T242-	1	0.02	ORF1a:S2352-	14	0.02
Maharashtra	ORF1b:R726-	1	0.03				ORF1a:E243-	1	0.02	ORF1a:Y2353-	14	0.02
Maharashtra	ORF1b:D727-	1	0.03				ORF1a:R244-	1	0.02	ORF1a:F2354-	14	0.02
Maharashtra	ORF1b:V728-	1	0.03				ORF1a:S245-	1	0.02	ORF1a:V2393-	14	0.02
Maharashtra	ORF1b:D729-	1	0.03				ORF1a:E246-	1	0.02	ORF1a:W2394-	14	0.02
Maharashtra	ORF1b:T730-	1	0.03				ORF1a:K247-	1	0.02	ORF1a:T2463-	14	0.02
Maharashtra	ORF1b:D731-	1	0.03				ORF1a:S248-	1	0.02	ORF1a:I2465-	14	0.02
Maharashtra	ORF1b:F732-	1	0.03				ORF1a:Y249-	1	0.02	ORF1a:S2600-	14	0.02
Maharashtra	ORF1b:V733-	1	0.03				ORF1a:E250-	1	0.02	ORF1a:T2601-	14	0.02
Maharashtra	ORF1b:N734-	1	0.03				ORF1a:K261-	1	0.02	ORF1a:F2602-	14	0.02
Maharashtra	ORF1b:E735-	1	0.03				ORF1a:K262-	1	0.02	ORF1a:N2603-	14	0.02
Maharashtra	ORF1b:F736-	1	0.03				ORF1a:F263-	1	0.02	ORF1a:T2615-	14	0.02
Maharashtra	ORF1b:Y737-	1	0.03				ORF1a:D264-	1	0.02	ORF1a:A2616-	14	0.02
Maharashtra	ORF1b:A738-	1	0.03				ORF1a:T265-	1	0.02	ORF1a:E2617-	14	0.02
Maharashtra	ORF1b:Y739-	1	0.03				ORF1a:F266-	1	0.02	ORF1a:D2687-	14	0.02
Maharashtra	ORF1b:L740-	1	0.03				ORF1a:N267-	1	0.02	ORF1a:L2688-	14	0.02
Maharashtra	ORF1b:R741-	1	0.03				ORF1a:G268-	1	0.02	ORF1a:G2689-	14	0.02
Maharashtra	ORF1b:K742-	1	0.03				ORF1a:E269-	1	0.02	ORF1a:A2690-	14	0.02
Maharashtra	ORF1b:A753-	1	0.03				ORF1a:C270-	1	0.02	ORF1a:C2691-	14	0.02
Maharashtra	ORF1b:V754-	1	0.03				ORF1a:P271-	1	0.02	ORF1a:I2692-	14	0.02
Maharashtra	ORF1b:V755-	1	0.03				ORF1a:N272-	1	0.02	ORF1a:N2767-	14	0.02
Maharashtra	ORF1b:C756-	1	0.03				ORF1a:F273-	1	0.02	ORF1a:I3047-	14	0.02
Maharashtra	ORF1b:F757-	1	0.03				ORF1a:V274-	1	0.02	ORF1a:T3141-	14	0.02
Maharashtra	ORF1b:N758-	1	0.03				ORF1a:F275-	1	0.02	ORF1a:I3142-	14	0.02
Maharashtra	ORF1b:S759-	1	0.03				ORF1a:P276-	1	0.02	ORF1a:S3344-	14	0.02
Maharashtra	ORF1b:I770-	1	0.03				ORF1a:L277-	1	0.02	ORF1a:V3467-	14	0.02
Maharashtra	ORF1b:K771-	1	0.03				ORF1a:N278-	1	0.02	ORF1a:D3492-	14	0.02
Maharashtra	ORF1b:N772-	1	0.03				ORF1a:S279-	1	0.02	ORF1a:F3493-	14	0.02
Maharashtra	ORF1b:F773-	1	0.03				ORF1a:I280-	1	0.02	ORF1a:N3494-	14	0.02
Maharashtra	ORF1b:K774-	1	0.03				ORF1a:I281-	1	0.02	ORF1a:L3495-	14	0.02
Maharashtra	ORF1b:N781-	1	0.03				ORF1a:K282-	1	0.02	ORF1a:V3496-	14	0.02
Maharashtra	ORF1b:N782-	1	0.03				ORF1a:T283-	1	0.02	ORF1a:A3497-	14	0.02
Maharashtra	ORF1b:V783-	1	0.03				ORF1a:I284-	1	0.02	ORF1a:M3498-	14	0.02
Maharashtra	ORF1b:F784-	1	0.03				ORF1a:K290-	1	0.02	ORF1a:K3499-	14	0.02
Maharashtra	ORF1b:M785-	1	0.03				ORF1a:K291-	1	0.02	ORF1a:G3578-	14	0.02
Maharashtra	ORF1b:S786-	1	0.03				ORF1a:K292-	1	0.02	ORF1a:T3579-	14	0.02
Maharashtra	ORF1b:E787-	1	0.03				ORF1a:L293-	1	0.02	ORF1a:H3580-	14	0.02
Maharashtra	ORF1b:A788-	1	0.03				ORF1a:D294-	1	0.02	ORF1a:H3581-	14	0.02
Maharashtra	ORF1b:K789-	1	0.03				ORF1a:G295-	1	0.02	ORF1a:W3582-	14	0.02
Maharashtra	ORF1b:D795-	1	0.03				ORF1a:F296-	1	0.02	ORF1a:L3583-	14	0.02
Maharashtra	ORF1b:L796-	1	0.03				ORF1a:M297-	1	0.02	ORF1a:F3760-	14	0.02
Maharashtra	ORF1b:T797-	1	0.03				ORF1a:G298-	1	0.02	ORF1a:Y3803-	14	0.02
Maharashtra	ORF1b:K798-	1	0.03				ORF1a:R299-	1	0.02	ORF1a:H3895-	14	0.02
Maharashtra	ORF1b:G799-	1	0.03				ORF1a:I300-	1	0.02	ORF1a:N3896-	14	0.02
Maharashtra	ORF1b:P800-	1	0.03				ORF1a:R301-	1	0.02	ORF1a:E3932-	14	0.02
Maharashtra	ORF1b:H801-	1	0.03				ORF1a:S302-	1	0.02	ORF1a:E3933-	14	0.02
Maharashtra	ORF1b:E802-	1	0.03				ORF1a:V303-	1	0.02	ORF1a:M3934-	14	0.02
Maharashtra	ORF1b:F803-	1	0.03				ORF1a:Y304-	1	0.02	ORF1a:E3962-	14	0.02
Maharashtra	ORF1b:C804-	1	0.03				ORF1a:P305-	1	0.02	ORF1a:A3963-	14	0.02
Maharashtra	ORF1b:S805-	1	0.03				ORF1a:V306-	1	0.02	ORF1a:Y3964-	14	0.02
Maharashtra	ORF1b:Q806-	1	0.03				ORF1a:A307-	1	0.02	ORF1a:E3965-	14	0.02
Maharashtra	ORF1b:H807-	1	0.03				ORF1a:S308-	1	0.02	ORF1a:Q3966-	14	0.02
Maharashtra	ORF1b:T808-	1	0.03				ORF1a:P309-	1	0.02	ORF1a:A3967-	14	0.02
Maharashtra	ORF1b:M809-	1	0.03				ORF1a:N310-	1	0.02	ORF1a:V3968-	14	0.02
Maharashtra	ORF1b:L810-	1	0.03				ORF1a:E311-	1	0.02	ORF1a:A3969-	14	0.02
Maharashtra	ORF1b:V811-	1	0.03				ORF1a:C312-	1	0.02	ORF1a:K3988-	14	0.02
Maharashtra	ORF1b:K812-	1	0.03				ORF1a:N313-	1	0.02	ORF1a:S3989-	14	0.02
Maharashtra	ORF1b:Q813-	1	0.03				ORF1a:Q314-	1	0.02	ORF1a:E3990-	14	0.02
Maharashtra	ORF1b:G814-	1	0.03				ORF1a:M321-	1	0.02	ORF1a:A3995-	14	0.02
Maharashtra	ORF1b:D815-	1	0.03				ORF1a:K322-	1	0.02	ORF1a:A3996-	14	0.02
Maharashtra	ORF1b:D816-	1	0.03				ORF1a:C323-	1	0.02	ORF1a:M4004-	14	0.02
Maharashtra	ORF1b:Y817-	1	0.03				ORF1a:D324-	1	0.02	ORF1a:Q4007-	14	0.02
Maharashtra	ORF1b:V818-	1	0.03				ORF1a:H325-	1	0.02	ORF1a:A4008-	14	0.02

Maharashtra	ORF1b:Y822-	1	0.03				ORF1a:C326-	1	0.02	ORF1a:M4009-	14	0.02
Maharashtra	ORF1b:P823-	1	0.03				ORF1a:G327-	1	0.02	ORF1a:K4024-	14	0.02
Maharashtra	ORF1b:D824-	1	0.03				ORF1a:E328-	1	0.02	ORF1a:V4025-	14	0.02
Maharashtra	ORF1b:P825-	1	0.03				ORF1a:T329-	1	0.02	ORF1a:T4026-	14	0.02
Maharashtra	ORF1b:S826-	1	0.03				ORF1a:S330-	1	0.02	ORF1a:M4029-	14	0.02
Maharashtra	ORF1b:R827-	1	0.03				ORF1a:W331-	1	0.02	ORF1a:Q4030-	14	0.02
Maharashtra	ORF1b:I828-	1	0.03				ORF1a:Q332-	1	0.02	ORF1a:A4052-	14	0.02
Maharashtra	ORF1b:L829-	1	0.03				ORF1a:T333-	1	0.02	ORF1a:R4053-	14	0.02
Maharashtra	ORF1b:G830-	1	0.03				ORF1a:G334-	1	0.02	ORF1a:D4054-	14	0.02
Maharashtra	ORF1b:A831-	1	0.03				ORF1a:D335-	1	0.02	ORF1a:G4055-	14	0.02
Maharashtra	ORF1b:G832-	1	0.03				ORF1a:F336-	1	0.02	ORF1a:C4056-	14	0.02
Maharashtra	ORF1b:C833-	1	0.03				ORF1a:V337-	1	0.02	ORF1a:V4057-	14	0.02
Maharashtra	ORF1b:F834-	1	0.03				ORF1a:K338-	1	0.02	ORF1a:I785-	14	0.02
Maharashtra	ORF1b:V835-	1	0.03				ORF1a:A339-	1	0.02	ORF1a:N786-	14	0.02
Maharashtra	ORF1b:L845-	1	0.03				ORF1a:T340-	1	0.02	ORF1a:G787-	14	0.02
Maharashtra	ORF1b:M846-	1	0.03				ORF1a:C341-	1	0.02	ORF1a:N869-	14	0.02
Maharashtra	ORF1b:I847-	1	0.03				ORF1a:E342-	1	0.02	ORF1a:E870-	14	0.02
Maharashtra	ORF1b:E848-	1	0.03				ORF1a:T356-	1	0.02	ORF1a:F871-	14	0.02
Maharashtra	ORF1b:R849-	1	0.03				ORF1a:C357-	1	0.02	ORF1a:A872-	14	0.02
Maharashtra	ORF1b:F850-	1	0.03				ORF1a:G358-	1	0.02	ORF1a:C922-	14	0.02
Maharashtra	ORF1b:V851-	1	0.03				ORF1a:Y359-	1	0.02	ORF1a:S923-	14	0.02
Maharashtra	ORF1b:S852-	1	0.03				ORF1a:L360-	1	0.02	ORF1a:G957-	14	0.02
Maharashtra	ORF1b:L853-	1	0.03				ORF1a:P361-	1	0.02	ORF1a:H3076-	14	0.02
Maharashtra	ORF1b:A854-	1	0.03				ORF1a:Q362-	1	0.02	ORF1a:V3077-	14	0.02
Maharashtra	ORF1b:I855-	1	0.03				ORF1a:N363-	1	0.02	ORF1a:V3078-	14	0.02
Maharashtra	ORF1b:D856-	1	0.03				ORF1a:A364-	1	0.02	ORF1a:T3133-	14	0.02
Maharashtra	ORF1b:A857-	1	0.03				ORF1a:V365-	1	0.02	ORF1a:P3134-	14	0.02
Maharashtra	ORF1b:K862-	1	0.03				ORF1a:V366-	1	0.02	ORF1a:L3135-	14	0.02
Maharashtra	ORF1b:H863-	1	0.03				ORF1a:K367-	1	0.02	ORF1a:E3608-	14	0.02
Maharashtra	ORF1b:P864-	1	0.03				ORF1a:I368-	1	0.02	ORF1a:N3609-	14	0.02
Maharashtra	ORF1b:N865-	1	0.03				ORF1a:Y369-	1	0.02	ORF1a:A3610-	14	0.02
Maharashtra	ORF1b:Q866-	1	0.03				ORF1a:C370-	1	0.02	ORF1a:F3611-	14	0.02
Maharashtra	ORF1b:E867-	1	0.03				ORF1a:P371-	1	0.02	ORF1a:L3612-	14	0.02
Maharashtra	ORF1b:Y868-	1	0.03				ORF1a:A372-	1	0.02	ORF1a:S3658-	14	0.02
Maharashtra	ORF1b:A869-	1	0.03				ORF1a:C373-	1	0.02	ORF1a:W3659-	14	0.02
Maharashtra	ORF1b:D870-	1	0.03				ORF1a:H374-	1	0.02	ORF1a:V3660-	14	0.02
Maharashtra	ORF1b:V871-	1	0.03				ORF1a:N375-	1	0.02	ORF1a:M3661-	14	0.02
Maharashtra	ORF1b:F872-	1	0.03				ORF1a:E381-	1	0.02	ORF1a:R3662-	14	0.02
Maharashtra	ORF1b:H873-	1	0.03				ORF1a:H382-	1	0.02	S:N121-	14	0.02
Maharashtra	ORF1b:L874-	1	0.03				ORF1a:S383-	1	0.02	S:N122-	14	0.02
Maharashtra	ORF1b:Y875-	1	0.03				ORF1a:L384-	1	0.02	S:V126-	14	0.02
Maharashtra	ORF1b:L876-	1	0.03				ORF1a:A385-	1	0.02	S:I128-	14	0.02
Maharashtra	ORF1b:Q877-	1	0.03				ORF1a:E386-	1	0.02	ORF1a:S984-	14	0.02
Maharashtra	ORF1b:Y878-	1	0.03				ORF1a:Y387-	1	0.02	ORF1a:Q985-	14	0.02
Maharashtra	ORF1b:I879-	1	0.03				ORF1a:H388-	1	0.02	ORF1a:Q986-	14	0.02
Maharashtra	ORF1b:R880-	1	0.03				ORF1a:N389-	1	0.02	ORF1a:T987-	14	0.02
Maharashtra	ORF1b:K881-	1	0.03				ORF1a:E390-	1	0.02	ORF1a:V988-	14	0.02
Maharashtra	ORF1b:T899-	1	0.03				ORF1a:S391-	1	0.02	ORF1a:V1056-	14	0.02
Maharashtra	ORF1b:N900-	1	0.03				ORF1a:G392-	1	0.02	ORF1a:V1057-	14	0.02
Maharashtra	ORF1b:D901-	1	0.03				ORF1a:L393-	1	0.02	ORF1a:V1058-	14	0.02
Maharashtra	ORF1b:N902-	1	0.03				ORF1a:K394-	1	0.02	ORF1a:N1080-	14	0.02
Maharashtra	ORF1b:T903-	1	0.03				ORF1a:T395-	1	0.02	ORF1a:N1081-	14	0.02
Maharashtra	ORF1b:S904-	1	0.03				ORF1a:I396-	1	0.02	ORF1a:A1082-	14	0.02
Maharashtra	ORF1b:R905-	1	0.03				ORF1a:L397-	1	0.02	ORF1a:M1083-	14	0.02
Maharashtra	ORF1b:Y906-	1	0.03				ORF1a:R398-	1	0.02	ORF1a:Y1090-	14	0.02
Maharashtra	ORF1b:W907-	1	0.03				ORF1a:K399-	1	0.02	ORF1a:I1091-	14	0.02
Maharashtra	ORF1b:E908-	1	0.03				ORF1a:G400-	1	0.02	ORF1a:V1169-	14	0.02
Maharashtra	ORF1b:P909-	1	0.03				ORF1a:G401-	1	0.02	ORF1a:R1170-	14	0.02
Maharashtra	ORF1b:E910-	1	0.03				ORF1a:F406-	1	0.02	ORF1a:T1171-	14	0.02
Maharashtra	ORF1b:F911-	1	0.03				ORF1a:G407-	1	0.02	ORF1a:N1172-	14	0.02
Maharashtra	ORF1b:Y912-	1	0.03				ORF1a:G408-	1	0.02	ORF1a:V1173-	14	0.02
Maharashtra	ORF1b:E913-	1	0.03				ORF1a:C409-	1	0.02	ORF1a:Y1174-	14	0.02
Maharashtra	ORF1b:A914-	1	0.03				ORF1a:V410-	1	0.02	ORF1a:L1175-	14	0.02
Maharashtra	ORF1b:N932-	1	0.03				ORF1a:F411-	1	0.02	ORF1a:A1176-	14	0.02
Maharashtra	ORF1b:S933-	1	0.03				ORF1a:S412-	1	0.02	ORF1a:V1177-	14	0.02
Maharashtra	ORF1b:Q934-	1	0.03				ORF1a:Y413-	1	0.02	ORF1a:V1299-	14	0.02
Maharashtra	ORF1b:T935-	1	0.03				ORF1a:V414-	1	0.02	ORF1a:V1300-	14	0.02
Maharashtra	ORF1b:F947-	1	0.03				ORF1a:G415-	1	0.02	ORF1a:V1584-	14	0.02
Maharashtra	ORF1b:L948-	1	0.03				ORF1a:C416-	1	0.02	ORF1a:D1585-	14	0.02
Maharashtra	ORF1b:C949-	1	0.03				ORF1a:H417-	1	0.02	ORF1a:R1645-	14	0.02

Maharashtra	ORF1b:C950-	1	0.03				ORF1a:N418-	1	0.02	ORF1a:Y1646-	14	0.02
Maharashtra	ORF1b:K951-	1	0.03				ORF1a:K419-	1	0.02	ORF1a:H1818-	14	0.02
Maharashtra	ORF1b:C952-	1	0.03				ORF1a:C420-	1	0.02	ORF1a:L1845-	14	0.02
Maharashtra	ORF1b:C953-	1	0.03				ORF1a:A421-	1	0.02	ORF1a:T1874-	14	0.02
Maharashtra	ORF1b:Y954-	1	0.03				ORF1a:Y422-	1	0.02	ORF1a:T1876-	14	0.02
Maharashtra	ORF1b:G989-	1	0.03				ORF1a:W423-	1	0.02	ORF1a:I1877-	14	0.02
Maharashtra	ORF1b:G990-	1	0.03				ORF1a:A427-	1	0.02	ORF1a:K1878-	14	0.02
Maharashtra	ORF1b:M991-	1	0.03				ORF1a:S428-	1	0.02	ORF1a:P1879-	14	0.02
Maharashtra	ORF1b:S992-	1	0.03				ORF1a:A429-	1	0.02	ORF1a:V1880-	14	0.02
Maharashtra	ORF1b:Y993-	1	0.03				ORF1a:N430-	1	0.02	ORF1a:A2821-	14	0.02
Maharashtra	ORF1b:Y994-	1	0.03				ORF1a:I431-	1	0.02	ORF1a:S2822-	14	0.02
Maharashtra	ORF1b:C995-	1	0.03				ORF1a:G432-	1	0.02	ORF1a:A2994-	14	0.02
Maharashtra	ORF1b:K996-	1	0.03				ORF1a:C433-	1	0.02	ORF1a:G2995-	14	0.02
Maharashtra	ORF1b:S997-	1	0.03				ORF1a:N434-	1	0.02	ORF1a:V2996-	14	0.02
Maharashtra	ORF1b:H998-	1	0.03				ORF1a:H435-	1	0.02	ORF1a:C2997-	14	0.02
Maharashtra	ORF1b:K999-	1	0.03				ORF1a:T436-	1	0.02	ORF1a:V2998-	14	0.02
Maharashtra	ORF1b:P1000-	1	0.03				ORF1a:G437-	1	0.02	ORF1a:N3477-	14	0.02
Maharashtra	ORF1b:P1001-	1	0.03				ORF1a:V438-	1	0.02	ORF1a:G3478-	14	0.02
Maharashtra	ORF1b:I1002-	1	0.03				ORF1a:V439-	1	0.02	ORF1a:D3479-	14	0.02
Maharashtra	ORF1b:S1003-	1	0.03				ORF1a:G440-	1	0.02	S:G971-	14	0.02
Maharashtra	ORF1b:F1004-	1	0.03				ORF1a:E441-	1	0.02	S:A972-	14	0.02
Maharashtra	ORF1b:P1005-	1	0.03				ORF1a:G442-	1	0.02	S:I973-	14	0.02
Maharashtra	ORF1b:N1025-	1	0.03				ORF1a:S443-	1	0.02	S:S974-	14	0.02
Maharashtra	ORF1b:V1026-	1	0.03				ORF1a:E444-	1	0.02	S:L1012-	14	0.02
Maharashtra	ORF1b:T1027-	1	0.03				ORF1a:G445-	1	0.02	S:I1013-	14	0.02
Maharashtra	ORF1b:D1028-	1	0.03				ORF1a:I453-	1	0.02	S:R1014-	14	0.02
Maharashtra	ORF1b:F1029-	1	0.03				ORF1a:L454-	1	0.02	S:A1015-	14	0.02
Maharashtra	ORF1b:N1030-	1	0.03				ORF1a:Q455-	1	0.02	S:A1016-	14	0.02
Maharashtra	ORF1b:A1031-	1	0.03				ORF1a:K456-	1	0.02	S:C662-	14	0.02
Maharashtra	ORF1b:I1032-	1	0.03				ORF1a:E457-	1	0.02	S:D663-	14	0.02
Maharashtra	ORF1b:A1033-	1	0.03				ORF1a:K458-	1	0.02	S:I664-	14	0.02
Maharashtra	ORF1b:T1034-	1	0.03				ORF1a:V459-	1	0.02	S:T827-	14	0.02
Maharashtra	ORF1b:C1035-	1	0.03				ORF1a:N460-	1	0.02	S:F347-	14	0.02
Maharashtra	ORF1b:D1036-	1	0.03				ORF1a:I461-	1	0.02	S:A348-	14	0.02
Maharashtra	ORF1b:W1037-	1	0.03				ORF1a:N462-	1	0.02	S:S349-	14	0.02
Maharashtra	ORF1b:T1038-	1	0.03				ORF1a:I463-	1	0.02	ORF1a:Q1198-	14	0.02
Maharashtra	ORF1b:N1039-	1	0.03				ORF1a:V464-	1	0.02	ORF1a:V1199-	14	0.02
Maharashtra	ORF1b:A1040-	1	0.03				ORF1a:G465-	1	0.02	ORF1a:E1200-	14	0.02
Maharashtra	ORF1b:G1041-	1	0.03				ORF1a:D466-	1	0.02	ORF1a:Q1201-	14	0.02
Maharashtra	ORF1b:D1042-	1	0.03				ORF1a:F467-	1	0.02	ORF1a:K1202-	14	0.02
Maharashtra	ORF1b:Y1043-	1	0.03				ORF1a:K468-	1	0.02	ORF1a:I1359-	14	0.02
Maharashtra	ORF1b:I1044-	1	0.03				ORF1a:L469-	1	0.02	ORF1a:I1360-	14	0.02
Maharashtra	ORF1b:L1045-	1	0.03				ORF1a:N470-	1	0.02	ORF1a:S1361-	14	0.02
Maharashtra	ORF1b:A1046-	1	0.03				ORF1a:E471-	1	0.02	ORF1a:N1362-	14	0.02
Maharashtra	ORF1b:N1047-	1	0.03				ORF1a:E472-	1	0.02	ORF1a:A1473-	14	0.02
Maharashtra	ORF1b:T1050-	1	0.03				ORF1a:I473-	1	0.02	ORF1a:E1954-	14	0.02
Maharashtra	ORF1b:E1051-	1	0.03				ORF1a:A474-	1	0.02	ORF1a:L1955-	14	0.02
Maharashtra	ORF1b:R1052-	1	0.03				ORF1a:I475-	1	0.02	ORF1a:K1981-	14	0.02
Maharashtra	ORF1b:L1053-	1	0.03				ORF1a:S481-	1	0.02	ORF1a:G1982-	14	0.02
Maharashtra	ORF1b:K1054-	1	0.03				ORF1a:A482-	1	0.02	ORF1a:A1983-	14	0.02
Maharashtra	ORF1b:L1055-	1	0.03				ORF1a:S483-	1	0.02	ORF1a:N2131-	14	0.02
Maharashtra	ORF1b:F1056-	1	0.03				ORF1a:T484-	1	0.02	ORF1a:S2132-	14	0.02
Maharashtra	ORF1b:A1057-	1	0.03				ORF1a:S485-	1	0.02	ORF1a:Y2171-	14	0.02
Maharashtra	ORF1b:A1058-	1	0.03				ORF1a:A486-	1	0.02	ORF1a:G2284-	14	0.02
Maharashtra	ORF1b:E1059-	1	0.03				ORF1a:F487-	1	0.02	ORF1a:S2285-	14	0.02
Maharashtra	ORF1b:T1060-	1	0.03				ORF1a:V488-	1	0.02	ORF1a:I2286-	14	0.02
Maharashtra	ORF1b:L1061-	1	0.03				ORF1a:E489-	1	0.02	ORF1a:F2314-	14	0.02
Maharashtra	ORF1b:K1062-	1	0.03				ORF1a:T490-	1	0.02	ORF1a:K2315-	14	0.02
Maharashtra	ORF1b:A1063-	1	0.03				ORF1a:V491-	1	0.02	ORF1a:W2316-	14	0.02
Maharashtra	ORF1b:T1064-	1	0.03				ORF1a:K492-	1	0.02	ORF1a:V2372-	14	0.02
Maharashtra	ORF1b:E1065-	1	0.03				ORF1a:G493-	1	0.02	ORF1a:A2891-	14	0.02
Maharashtra	ORF1b:E1066-	1	0.03				ORF1a:L494-	1	0.02	ORF1a:V2892-	14	0.02
Maharashtra	ORF1b:T1067-	1	0.03				ORF1a:D495-	1	0.02	ORF1a:I1935-	14	0.02
Maharashtra	ORF1b:F1068-	1	0.03				ORF1a:A516-	1	0.02	ORF1a:K1936-	14	0.02
Maharashtra	ORF1b:K1069-	1	0.03				ORF1a:K517-	1	0.02	ORF1a:P1950-	14	0.02
Maharashtra	ORF1b:L1070-	1	0.03				ORF1a:K518-	1	0.02	ORF1a:A1951-	14	0.02
Maharashtra	ORF1b:E1079-	1	0.03				ORF1a:G519-	1	0.02	ORF1a:S1952-	14	0.02
Maharashtra	ORF1b:V1080-	1	0.03				ORF1a:A520-	1	0.02	ORF1a:Q2034-	14	0.02
Maharashtra	ORF1b:L1081-	1	0.03				ORF1a:W521-	1	0.02	ORF1a:L2039-	14	0.02
Maharashtra	ORF1b:S1082-	1	0.03				ORF1a:N522-	1	0.02	ORF1a:A2040-	14	0.02

Maharashtra	ORF1b:D1083-	1	0.03				ORF1a:I523-	1	0.02	ORF1a:C2041-	14	0.02
Maharashtra	ORF1b:R1084-	1	0.03				ORF1a:G524-	1	0.02	ORF1a:Q3373-	14	0.02
Maharashtra	ORF1b:E1085-	1	0.03				ORF1a:Y534-	1	0.02	ORF1a:I1435-	14	0.02
Maharashtra	ORF1b:L1086-	1	0.03				ORF1a:A535-	1	0.02	ORF1a:N1436-	14	0.02
Maharashtra	ORF1b:H1087-	1	0.03				ORF1a:F536-	1	0.02	ORF1a:V1475-	14	0.02
Maharashtra	ORF1b:L1088-	1	0.03				ORF1a:I581-	1	0.02	ORF1b:P1967-	14	0.02
Maharashtra	ORF1b:S1089-	1	0.03				ORF1a:V605-	1	0.02	ORF1b:F1968-	14	0.02
Maharashtra	ORF1b:W1090-	1	0.03				ORF1a:V606-	1	0.02	ORF1b:F1969-	14	0.02
Maharashtra	ORF1b:E1091-	1	0.03				ORF1a:Q607-	1	0.02	ORF1b:Y1970-	14	0.02
Maharashtra	ORF1b:V1092-	1	0.03				ORF1a:L608-	1	0.02	ORF1b:Y1971-	14	0.02
Maharashtra	ORF1b:G1093-	1	0.03				ORF1a:T609-	1	0.02	ORF1b:S1972-	14	0.02
Maharashtra	ORF1b:K1094-	1	0.03				ORF1a:S610-	1	0.02	ORF1b:D1973-	14	0.02
Maharashtra	ORF1b:P1095-	1	0.03				ORF1a:L624-	1	0.02	ORF1b:S1974-	14	0.02
Maharashtra	ORF1b:R1096-	1	0.03				ORF1a:K625-	1	0.02	ORF1b:P1975-	14	0.02
Maharashtra	ORF1b:P1097-	1	0.03				ORF1a:F685-	1	0.02	ORF1b:C1976-	14	0.02
Maharashtra	ORF1b:Y1103-	1	0.03				ORF1a:L686-	1	0.02	ORF1b:E1977-	14	0.02
Maharashtra	ORF1b:V1104-	1	0.03				ORF1a:A687-	1	0.02	ORF1b:S1978-	14	0.02
Maharashtra	ORF1b:F1105-	1	0.03				ORF1a:L688-	1	0.02	ORF1b:H1979-	14	0.02
Maharashtra	ORF1b:T1106-	1	0.03				ORF1a:C720-	1	0.02	ORF1b:G1980-	14	0.02
Maharashtra	ORF1b:G1107-	1	0.03				ORF1a:V721-	1	0.02	S:V90-	14	0.02
Maharashtra	ORF1b:Y1108-	1	0.03				ORF1a:K722-	1	0.02	S:S94-	14	0.02
Maharashtra	ORF1b:R1109-	1	0.03				ORF1a:S723-	1	0.02	S:T95-	14	0.02
[truncated: 3,222,233 more chars]
